# Overlapping cell population expression profiling and regulatory inference in *C. elegans*

**DOI:** 10.1186/s12864-016-2482-z

**Published:** 2016-02-29

**Authors:** Joshua Burdick, Travis Walton, Elicia Preston, Amanda Zacharias, Arjun Raj, John Isaac Murray

**Affiliations:** Department of Genetics, University of Pennsylvania, Philadelphia, Pennsylvania USA; Department of Bioengineering, University of Pennsylvania, Philadelphia, Pennsylvania USA; Department of Genetics, Perelman School of Medicine, University of Pennsylvania, 437A Clinical Research Building, 415 Curie Boulevard, Philadelphia, PA 19104-6145 USA

**Keywords:** *C. elegans*, Embryonic development, Tissue-specific expression

## Abstract

**Background:**

Understanding gene expression across the diverse metazoan cell types during development is critical to understanding their function and regulation. However, most cell types have not been assayed for expression genome-wide.

**Results:**

We applied a novel approach we term “Profiling of Overlapping Populations of cells (POP-Seq)” to assay differential expression across all embryonic cells in the nematode *Caenorhabditis elegans*. In this approach, we use RNA-seq to define the transcriptome of diverse partially overlapping FACS-sorted cell populations. This identified thousands of transcripts differentially expressed across embryonic cells. Hierarchical clustering analysis identified over 100 sets of coexpressed genes corresponding to distinct patterns of cell type specific expression. We identified thousands of candidate regulators of these clusters based on enrichment of transcription factor motifs and experimentally determined binding sites.

**Conclusions:**

Our analysis provides new insight into embryonic gene regulation, and provides a resource for improving our knowledge of tissue-specific expression and its regulation throughout *C. elegans* development.

**Electronic supplementary material:**

The online version of this article (doi:10.1186/s12864-016-2482-z) contains supplementary material, which is available to authorized users.

## Background

The specification and differentiation of cell types during animal development requires that genes be expressed in appropriate spatiotemporal patterns. Defining the regulatory mechanisms controlling this patterning is a central goal of developmental biology research. One powerful tool to infer regulatory networks is to identify genes preferentially expressed in a cell type and screen experimentally or computationally for transcription factors (TFs) likely to bind those genes’ regulatory sequences. This approach is especially powerful in model organisms such as worms and flies, whose smaller genomes reduce the amount of DNA to search for regulatory function.

The nematode *C. elegans* is well suited for such a comprehensive study of developmental regulation because of its stereotyped development from zygote to adult, with each adult hermaphrodite developing through an identical pattern of cell divisions [1]. Each animal has the same number and organization of cells of each type, with 558 cells present at the end of embryogenesis. In addition, the signaling pathways controlling cell type specification, including the Notch (reviewed in [2]), Ras (reviewed in [3]), and Wnt [4, 5] signaling pathways, are conserved with humans and other animals. Time-lapse imaging of fluorescent reporters has generated cellular resolution expression information for many genes [6–8], and automated image analysis methods make it possible to identify all expressing cells in embryos or larvae [9, 10]. Recent studies have defined the in vivo [11, 12] and in vitro [13] binding and binding motifs [14–16] for a substantial proportion of *C. elegans* TFs, and have experimentally measured TF binding at scale in vivo [11, 12] and in vitro [13], providing a basis for regulatory inference. Integrative analysis of coexpression, genetic and protein-protein interactions, and other data sources allow predicting the functions of many genes [17, 18].

Imaging of animals using reporter genes [19], RNA FISH probes [20], or antibodies [21] can detect developmental expression patterns across all cells of the embryo. However, logistics limit the number of genes whose expression can be measured at high resolution by these methods. Alternatively, individual cell types can be isolated by flow cytometry from dissociated embryos [22, 23] or larvae [24, 25], and assayed for mRNA levels genome-wide. Similarly, tissue-specific mRNA can be isolated based on its association with an epitope-tagged poly-A binding protein expressed under the control of a tissue-specific promoter [26, 27]. These approaches have been applied to a subset of terminally differentiated cell types [23], but a comprehensive analysis across cell types is limited by the lack of individual markers for most unique cells, and by the labor and cost associated with isolating and analyzing large numbers of cell types individually. Furthermore, even different cells of the same type (e.g. body wall muscle) can have different expression profiles depending on their lineage history and position within the animal [19, 28].

**Fig. 1:**
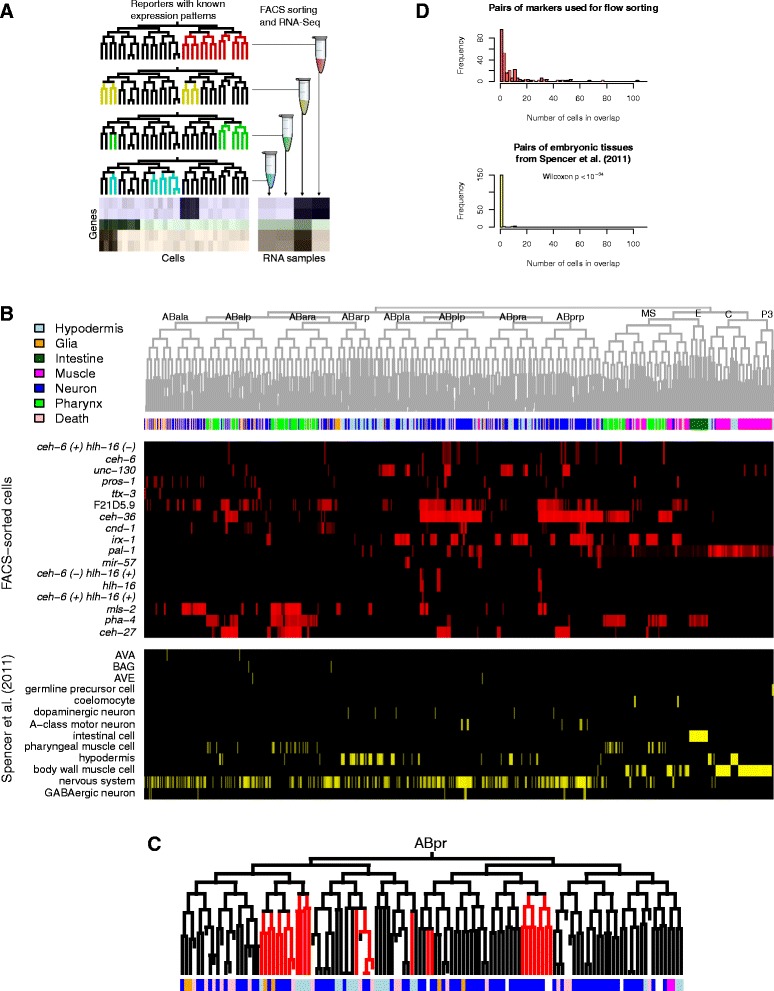
Experimental strategy. **a** Summary: we FACS sort embryonic cells, based on expression of markers with known expression patterns, and measure expression in cells expressing (or not expressing) a particular marker using RNA-seq. Genes expressed in similar sets of cells are enriched in a similar set of samples. **b** Expression patterns of cells used for sorting (*shown in red*), and in Spencer et al. [23], shown in yellow. Cell fates are shown in the colored bar at the top. **c** Expression pattern of *unc-130* (one of the markers used for sorting) in the Abpl sublineage, with cell fates colored as in (**b**). **d** Comparison of overlap of groups of cells used for sorting in this paper, with similar overlap for the groups of cells used in Spencer et al. [23]

Previous studies of differential expression in the embryo assayed expression in terminally differentiated cell types, mostly as non-overlapping populations. Here, we developed a strategy, “Profiling of Overlapping Populations of cells (POP-Seq)**”** that uses expression measurements from overlapping cell populations to identify genes differentially expressed in arbitrary patterns. We previously showed that measuring expression in multiple partially overlapping groups of cells can provide information about differential expression across the entire lineage, and is thus more comprehensive than sorting based on “cell type-specific” markers whose expression is minimally overlapping [29]. Here, we applied this concept to identify patterned gene expression across all cells of the *C. elegans* embryo by measuring expression genome-wide in multiple overlapping cell populations isolated by flow cytometry (Fig. 1a). We show that these overlapping expression measurements provide broad information about where genes are expressed in the *C. elegans* embryo and we define 300 gene expression clusters, many of which correspond to groups of genes that are coregulated in particular tissues. We identify 495 TFs whose motifs or in vivo binding are enriched near genes in 50 clusters; in many cases the putative regulators are coexpressed with their proposed targets. We validate these findings by identifying novel gene expression and regulation in the pharyngeal glands and ciliated neurons, and by comparing with existing genomic resources. These results identify general features of embryonic gene expression patterns and their regulation, and provide powerful resource for future studies of embryonic regulation.

## Results

### Selection and characterization of overlapping sort markers

We selected fifteen *C. elegans* transgenic reporter strains expressing GFP, mCherry, or both fluorescent proteins in specific embryonic cells (Additional file 1: Table S1) [19, 30]. We identified all GFP or mCherry-positive cells in each strain through the hypodermal enclosure stage by automated lineage tracing of 4D confocal movies [9, 10, 31]. This provided a cellular resolution atlas of each reporter gene’s expression, and identified new expressing cells and dynamics of expression for many reporters (Fig. 1b, Additional file 2: Figure S1, Additional file 3: Table S2).

In general, the reporters used for sorting were expressed in multiple terminal cell types. For example, PROS-1::GFP, which was previously reported to be expressed and required in the excretory canal cell [32], is also expressed in many sheath type glia cells, coelomocytes, pharyngeal glands and some neurons (Fig. 1b, Additional file 2: Figure S1). Similarly, UNC-130::GFP is expressed in progenitors of diverse cell types including a subset of muscle and hypodermal cells, the excretory system, several types of neurons and a few pharyngeal and rectal cells (Fig. 1c, Additional file 2: Figure S1) [10]. The average overlap between our cell populations is much higher than in previous genome-wide analyses of cell-specific expression, which largely focused on distinct terminal cell types (of the cell types expressing a marker, mean 10.8 overlapping cell types vs 0.4 cell types in Spencer et al. [23]; Fig. 1d).

### RNA-seq from sorted cell populations reproducibly detects differentially expressed genes

We dissociated cells from embryos and used flow cytometry to purify cells based on these strains' fluorescent marker. We analyzed both fluorescent “positive” cells and matched non-fluorescent “negative” cells from the same sort. We prepared RNA from each sample and quantified expression using strand-specific RNA-seq on the SOLiD platform [33]. This resulted in nearly a billion mapped reads (Additional file 4: Table S3).

**Fig. 2:**
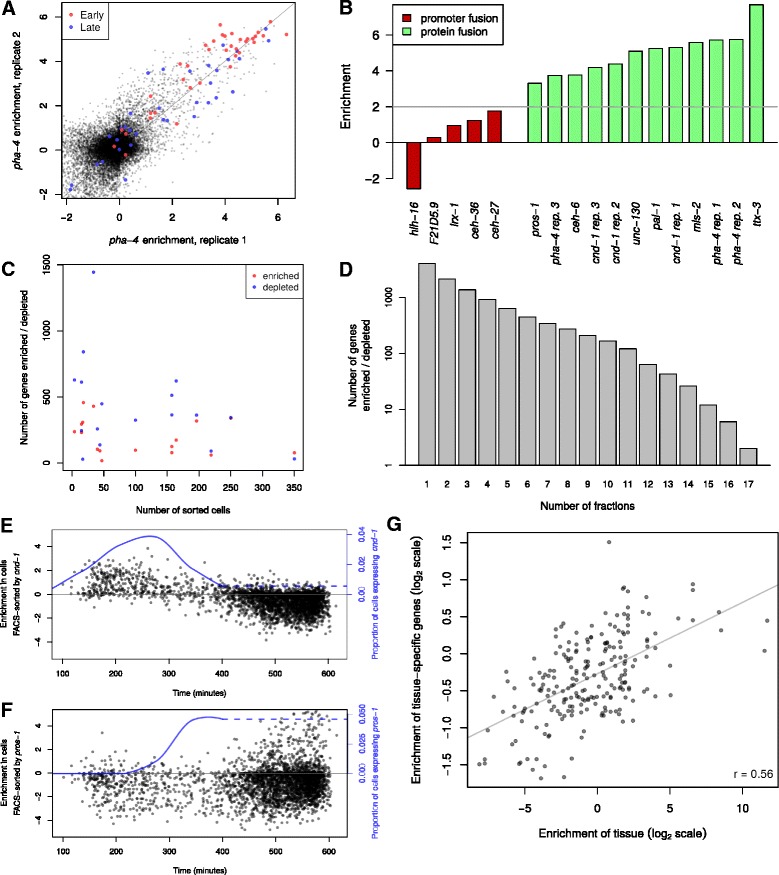
Data quality of expression measurements of FACS-sorted cells. **a** Enrichment of genes in two replicates of sorting by a *pha-4* reporter. Known pharyngeal genes defined as early or late embryonic in Gaudet et al. [37] are shown in red and blue, respectively. **b** Enrichment of mRNAs corresponding to markers used for sorting. Promoter fusions are shown in red, while protein fusions are shown in green. **c** Comparison of number of sorted cell identities (out of 1341 embryonic cell identities) in a sorted fraction with the number of genes enriched (*red*) or depleted (*blue*). **d** Number of genes enriched or depleted in different numbers of sorted fractions. **e** Enrichment of time-specific genes in cells sorted by *cnd-1*. The proportion of the total cells expressing the *cnd-1* reporter is shown in blue. (The lineage data ends at 400 min. The dotted line indicates that by eye, expression appears to continue through hatch in a similar number of cells.) **f** Same as (**e**), except for cells sorted using a *pros-1* reporter. **g** Comparison of tissues present in sorted fractions, with enrichment of known tissue-specific genes found by Spencer et al. [23]. There is one point for each pairing of a tissue *t* with a sort fraction *s*. The *x-*axis shows the enrichment of cells with known tissue *t* in sort fraction *s*. The *y-*axis shows the average enrichment in sort fraction *s* of genes annotated as expressed in tissue *t* by Spencer et al. [23]

We detected expression of 15,683 genes in at least one FACS-sorted sample, at a level of at least one RPM (reads per million mapped reads), with between 9722 and 12,455 genes detected in each individual sample (Additional file 5: Table S4). We detected more unique transcripts in cell populations with fewer embryonic cell types, and more genes with enriched or depleted expression (Fig. 2c), as compared with populations containing more embryonic cell identities. This effect was significant for depletions (Mann–Whitney *p* < 0.007). This suggests that measuring transcriptomes in smaller groups of cells increases sensitivity to detect rare, cell-type-specific transcripts.

We identified genes whose expression was enriched or depleted in each group of FACS-sorted cells by comparing each annotated gene’s expression between the positive sample and the paired negative control sample; we used mock-sorted cells as a control for two samples where the paired negative was not available. Both normalization methods gave similar results, but using the matched negative samples resulted in higher measured enrichment levels and thus an increased sensitivity to detect genes with modest expression enrichment (Additional file 6: Figure S2). These enrichments were reproducible across biological replicates for independent sort markers (mean *r* = 0.77; Fig. 2a, Additional file 6: Figure S2), indicating high overall reproducibility. Many genes were enriched or depleted in specific sort fractions; 4017 genes were enriched or depleted 4-fold in at least one sample, and 2152 were enriched or depleted in two or more samples (Fig. 2c-d). This provides a conservative list of genes likely to be differentially expressed in the embryo (Additional file 7: Table S5).

Expression of most marker genes (genes whose reporters were used for sorting) was enriched in their own positive sort fraction (Fig. 2b). This enrichment was strongest for translational reporter markers where GFP is fused to the C-terminus of the protein and the gene is surrounded by its normal genomic context (median enrichment = 36-fold). In contrast, enrichment was lower for “transcriptional” reporter markers where the marker gene’s promoter was used to drive a stable mCherry-histone fusion (median enrichment = 2-fold). This may reflect the fact that many of the marker genes are expressed transiently during embryogenesis [34], with the mCherry-histone fusion protein persisting long after the endogenous RNA. Consistent with this, protein levels of the translational reporters often show dynamic regulation mirroring that of the corresponding mRNA and are often expressed more transiently than promoter fusion reporters for the same gene [30, 35].

### FACS gating for single cells preferentially enriches for specific cell types

The combined expression of the positive and negative fractions was similar to, but not identical to, expression in bulk embryonic cells. This could be because the forward-scatter and side-scatter “gates” used during cell sorting to exclude cell clumps also preferentially exclude certain cell types. To test this, we compared expression between “singlet” cells that had been gated to exclude cell clumps with “ungated” cells that were run through the FACS machine but not gated. We identified 52 genes preferentially expressed in the ungated cells. These genes were enriched for genes expressed in the hypodermis and intestine [36], and in late embryonic cells (after 400 min; Additional file 6: Figure S3). Accounting for the effects of singlet gating improves the similarity between ungated cells and the combined positive and negative expression profiles (Additional file 6: Figure S4, one-sided Wilcoxon paired *p* < 10^−4^). Gating for single cells during flow cytometry thus provides information about an additional partially overlapping embryonic cell population that likely includes hypodermal and intestinal cells. We therefore included “singlet enrichment” in the clustering analysis described below.

### RNA-seq from FACS sorted cell populations identifies spatiotemporal gene expression signatures

Since our lineage data identifies which cells should be contained within each sort fraction (Fig. 1b, c, Additional file 2: Figure S1), we asked whether genes known to be expressed in specific cell types were enriched in the expected fraction. In some cases cell types predicted to be present or absent in a given cell population have been previously characterized for genome-wide expression. For example, the PHA-4::GFP fraction specifically labels pharynx, intestine and rectal cells, and genes identified previously as expressed in the pharynx [37] were preferentially expressed in that fraction (Fig. 2a, hypergeometric *p* < 10^−17^). We tested this more broadly by asking whether genes previously identified as tissue-specific by the modENCODE project [23] were enriched in sort fractions that preferentially contain cells from that tissue (Fig. 2g). We identified a significant relationship (Pearson r = 0.56, *p* < 10^−19^), consistent with the different fractions having the expected tissue compositions.

**Fig. 3:**
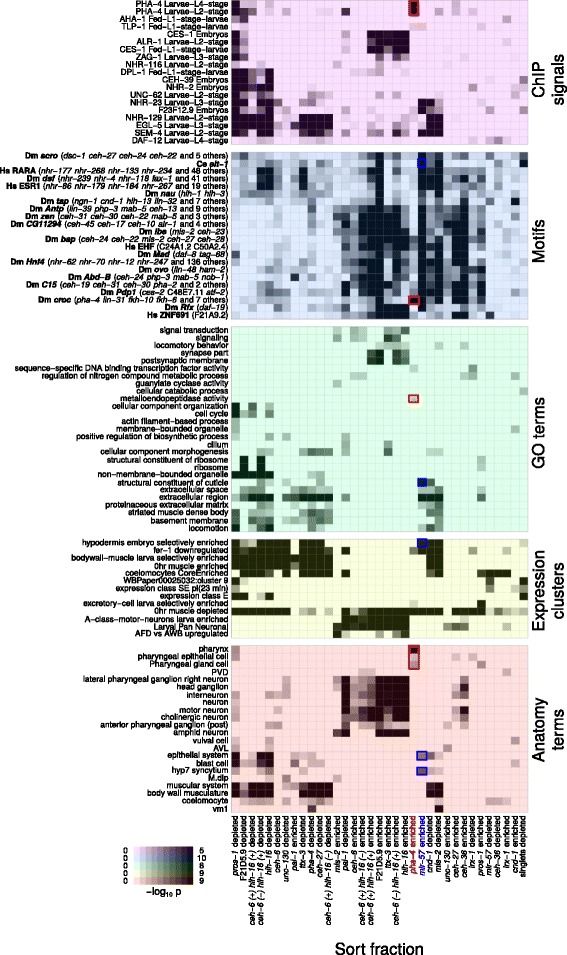
Annotation of FACS-sorted cells. Enrichment of ChIP peaks, motifs, GO terms, expression clusters, and anatomy terms associated with genes enriched in each sort fraction. Selected *pha-4 (+)* and *mir-57 (+)* enrichments mentioned in the text are boxed in red and blue, respectively

We identified many anatomy, expression, and gene ontology (GO) annotation terms significantly associated with expression in specific sorted fractions (Fig. 3; Additional file 8: Table S6, Additional file 9: Table S7 and Additional file 10: Table S8). Each sorted fraction except for the singlet cells had at least one anatomy term significantly enriched (fdr <0.05). These were generally consistent with the tissue identities of the cells present in that fraction (Additional file 8: Table S6 and Additional file 9: Table S7). Similarly, many GO terms enriched in particular fractions were consistent with the cell types present in each fraction and in some cases predicted novel gene classes (Additional file 10: Table S8). For example, the *mir-57(+)* fraction, which preferentially contains hypodermal cells, was enriched for the anatomy term “hypodermis” and the GO terms “structural constituent of cuticle” and “extracellular region,” consistent with the role of hypodermal cells in secreting the cuticular exoskeleton [38]. Similarly, the *pha-4(+)* fraction, which consisted mostly of pharyngeal cells, was enriched for genes associated with metalloendopeptidase activity. Such proteases have been implicated in remodeling of extracellular matrix during postembryonic organ growth [39], and thus may also play a role in the developing pharynx, which undergoes complex morphogenetic changes and extracellular matrix remodeling [40].

Our smallest fraction was the *ceh-6(+);hlh-16(+)* double-positive cells, which consists of only four cells: the excretory duct and pore cells (which are single-celled epithelial tubes), and DB1 and DB3 motorneurons (Additional file 2: Figure S1). Genes preferentially expressed in this fraction were enriched for annotations associated with DB neurons (such as “cholinergic neuron”, “motor neuron”, and “DB neuron”). Intriguingly, this population was also enriched for genes such as *grl-2, grl-12, ptr-5, wrt-5, grd-15,* and *ptc-1*, which are associated with “hedgehog signaling”. This pathway is not thought to be active for signaling in *C. elegans* [41], but many genes with homology to the ligands and receptors exist. Some hedgehog-related genes have been shown to be expressed in epithelial cell types and consistent with this, a *grl-2* reporter is expressed in the excretory duct and pore cells [42]. Genes homologous to the hedgehog receptor Patched have been shown to be important for lumen formation in *C. elegans* glia [43]. In total eleven genes associated with the hedgehog pathway were enriched in the *ceh-6(+);hlh-16(+)* double-positive cells. This suggests the possibility that many hedgehog-related genes may be involved in lumen formation in the tubular cells of the excretory system.

While we chose our sort markers mostly with the goal of maximizing our ability to measure spatial patterns, these reporters may also contain information about the timing of gene expression. We tested this by first identifying a group of “temporally-specific” genes expressed at different times in an RNA-seq time-course from whole embryos [34] (see methods for details). We then asked whether these temporally-specific genes were enriched or depleted in each sort fraction. Fractions predicted to contain early embryonic cells had higher expression of “early genes,” while we observed higher expression of “late genes” in cells sorted based on reporters expressed later. For example, *cnd-1* is expressed in many cells early in embryogenesis, while *pros-1* is expressed late, in a smaller fraction of cells. Genes expressed early in whole embryos tended to be enriched by *cnd-1* sorting (Fig. 2e), while sorting by *pros-1* depleted for early genes and was enriched for a subset of later genes (Fig. 2f). Each fraction was significantly enriched for specific temporal stages (Additional file 6: Figure S3). Based on this, we conclude that our expression data includes information about both spatial and temporal expression differences between embryonic cells.

### Motif enrichment predicts regulators acting in each cell population

To identify TFs that may regulate genes in each cell population, we searched for TFs that preferentially bind near genes enriched in that population as measured by ChIP data from modENCODE [11, 12], and for TF motifs overrepresented upstream of the genes enriched in each fraction. We compiled a database of 146 ChIP experiments from *C. elegans* [11, 12, 44] and 1877 TF motifs from multiple species [14, 15, 45] including 1493 motifs for 291 *C. elegans* TFs [16]. This identified motifs and ChIP signals significantly associated with each of the seventeen FACS sorting experiments (Fig. 3; Additional file 11: Table S9 and Additional file 12: Table S10). These represent candidate regulators of gene expression within the cells in each population.

For example, upstream intergenic sequences of genes in the PHA-4::GFP(+) fraction are enriched for the FOXA1 motif recognized by *pha-4* [46], and for binding of PHA-4 as measured by ChIP [44], consistent with the known role of PHA-4 in pharynx cell identity and gene expression [46, 47]. The FOXA1 motif, but not PHA-4 ChIP binding, was also significantly enriched in genes expressed in the PAL-1::GFP(+) fraction, which contains a high fraction of rectal cells. Since *pha-4* mutants have major rectal defects [48], *pha-4* may directly regulate many rectal-specific genes, similar to its role in the pharynx, but these genes may be less easily identified by ChIP on whole embryos because the rectum represents a much small fraction of all embryonic cells than the pharynx. This suggests that the limitations of whole-organism ChIP in identifying regulators important for expression in small cell populations may be partially overcome by analysis of motif enrichment.

### Clustering overlapping sort fraction expression data identifies genes coexpressed across diverse embryonic cell types

Since our experiments assayed expression in many partially overlapping populations of cells that collectively cover the full embryo (Fig. 1a), these data contain information about the expression patterns of every cell type [29]. For example, pharyngeal gland cells are enriched in *pha-4 (+)* and *pros-1 (+)* fractions (Fig. 1b, Additional file 2: Figure S1, Additional file 3: Table S2). Therefore genes preferentially expressed in pharyngeal gland cells should be enriched in these fractions and depleted in other fractions that do not contain these cells. More generally, we predict that genes with similar patterns of enrichment and depletion across sort fractions are expressed in similar tissue-specific patterns. We provide a web-based tool to allow users to find genes with an embryonic expression pattern similar to that of a query gene (Additional file 13).

**Fig. 4:**
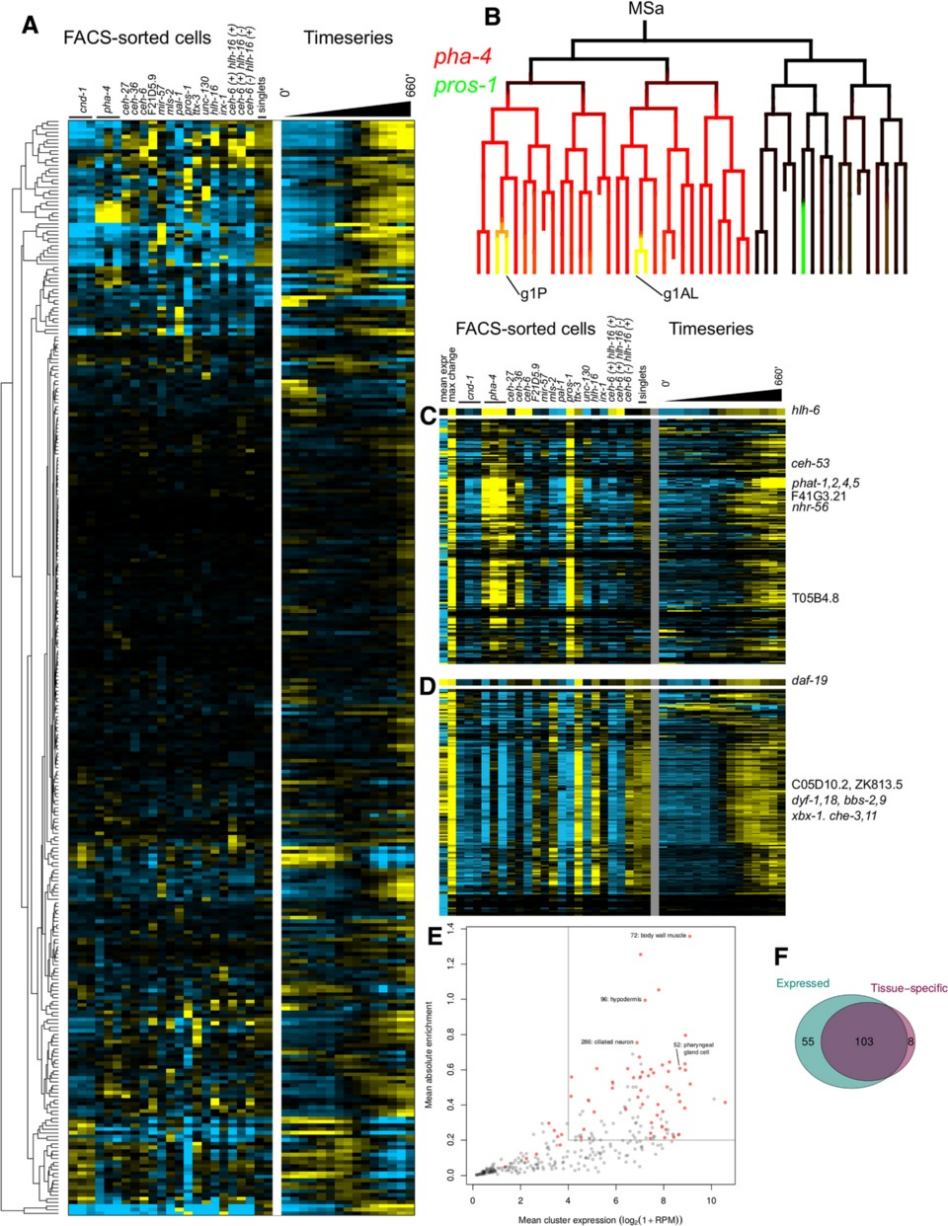
Clustering of enrichment. **a** Average enrichment for genes grouped into 300 clusters. The timeseries data is from Li et al. [34]. **b** MSa lineage, showing expression of *pha-4* (*red*) and *pros-1* (*green*); yellow indicates overlap. Pharyngeal gland cells are shown as red rectangles. **c** Cluster 52, enriched with genes known to be expressed in pharyngeal gland cells. **d** Cluster 286, enriched with genes known to be expressed in ciliated neurons. **e** Mean expression, and mean absolute enrichement, for each cluster. Clusters with known enriched anatomy annotation are shown in red; selected clusters are labeled. **f** Overlap of expressed and tissue-specific clusters

We used hierarchical clustering to identify groups of genes with similar expression patterns across all samples, suggesting they are coexpressed in the embryo (Fig. 4a). We tested different correlation cutoffs for cluster inclusion, and selected a cutoff resulting in 300 clusters that maximized our ability to detect candidate regulators of clusters by motif and ChIP analysis (see below, Additional file 14: Table S11). We did not use the temporal RNA-seq data from whole embryos [34] as part of the clustering, but examining the temporal data for genes within a clusters makes it possible to predict the temporal order of expression for genes within a cluster (Fig. 4c).

**Fig. 5:**
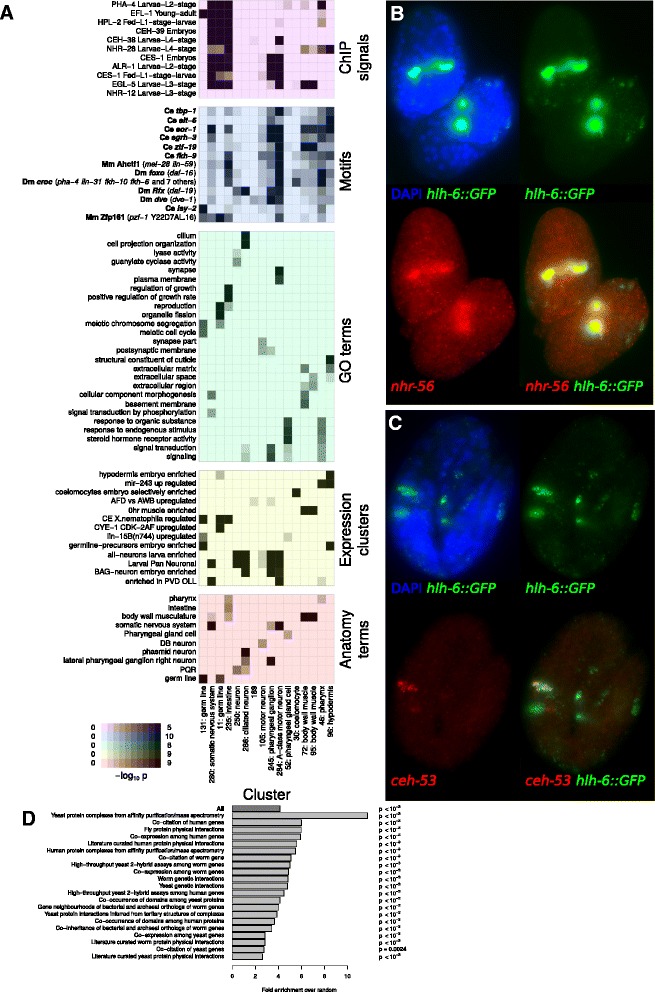
Annotation of clusters. **a** Enrichment (analogously to Fig. 1) of ChIP signals, TF motifs, GO terms, expression clusters, and anatomy terms associated with genes in clusters. **b** Expression pattern of *hlh-6* and *nhr-56* in comma-stage embryos, measured by RNA-FISH. **c** Expression pattern of *hlh-6* and *ceh-53* in a three fold embryo, measured by RNA-FISH. **d** Enrichment of co-clustered genes in WormNet [18] annotations

Many of the clusters correspond to specific tissues, based on significant enrichment of previously annotated tissue specific genes as curated by Wormbase in that cluster [36] (Fig. 5a). 18 of the 300 clusters had at least one significantly enriched Anatomy Ontology term at an FDR of 0.05 (Additional file 15: Table S12). An additional 56 clusters were significantly enriched for tissue-specific genomic expression signatures representing 11 of the 13 embryonic tissues assayed by modENCODE [23] (Additional file 16: Table S13 and Additional file 17: Table S14). Finally, 54 clusters were significantly associated with one or more genome-wide expression datasets that did not explicitly assay tissue-specific expression. These and the other clusters represent groups of genes that may be coregulated in distinct patterns not previously assayed by genome-wide methods, since such experiments can contain implicit information about cell type specificity [49]

We tested whether genes in the same cluster are coexpressed across cells by comparing to the EPIC dataset of cellular resolution expression profiles for 121 genes and to existing larval patterns for 93 genes [50]. For two genes with high-resolution expression data, genes in the same cluster had much more similar expression patterns than genes in different clusters (Additional file 6: Figure S7). This similarity was stronger for embryonic (Wilcoxon *p* < 10^−36^) than for the larval expression patterns (Wilcoxon *p* < 10^−8^). This consistency is striking given that the RNA-seq data includes information about later embryonic stages not assayed in the imaging data. Thus, known tissue-specific annotation and expression patterns support the idea that genes which occur in a given cluster are expressed in a similar set of embryonic cells.

We further validated the clusters by comparing them with WormNet [18], which combines many *C. elegans* genomic resources in a network model. Genes in the same cluster were linked by annotations in WormNet 5-12 fold more often than random, depending on the annotation (Fig. 5d). This enrichment was strongest for genes whose fly and yeast orthologs undergo protein-protein interactions, consistent with genes in a cluster acting together*.*

We also compared the resolution of our data with the Spencer et al. [23] tissue-specific dataset, by seeing if genes which clustered together in one dataset were tightly coexpressed in the other dataset (Additional file 6: Figure S8). We clustered the 13 embryonic experiments from Spencer et al. [23], and for each cluster, compared the average within-cluster correlation in that dataset, with the same correlation computed in our dataset (Additional file 6: Figure S8A). We also did the reverse comparison (Additional file 6: Figure S8B). In each case, many clusters of genes that had very similar expression patterns in one dataset often had different expression patterns in the other dataset. For instance, the cluster of genes shown in Additional file 6: Figure S8C had a mean within-cluster correlation of 0.73 in the Spencer data, and 0.04 in our dataset. These genes were enriched in the A-type motor neuron sample in the Spencer data, while in the FACS data, several of the genes (such as *unc-3*, *unc-4*, and *cutl-19*) appear somewhat different data. Possible explanations include that these genes are expressed in different subsets of A-type motor neurons, or differentially expressed in progenitor cells. Conversely, genes in a cluster which is highly correlated in our dataset (mostly enriched in the *mls-2*, *hlh-16*, and *ceh-6(-) hlh-16(+)* fractions; Additional file 6: Figure S8D) are not highly correlated in the Spencer et al. [23] dataset suggesting they correspond to a cell type not assayed in that dataset. This analysis suggests that while our data and the Spencer et al. [23] overlap in their coverage, each dataset can find similarities between genes that the other dataset cannot resolve.

In some cases, a cluster is enriched for genes known to be expressed in a particular cell type but also predicts novel additional genes to be expressed in those cells. For example, cluster 52 is defined primarily by high expression in the PHA-4::GFP(+) and *pros-1* sorted fractions, and the only cells that are included in both of these fractions are the pharyngeal gland cells (Fig. 4b-c). Furthermore this cluster contains seven genes (including *phat-1, -2, -4,* and *-5*) of the sixteen known to be expressed in the pharyngeal gland cells (hypergeometric *p* < 10^−9^) [51]. However, this cluster also contains an additional 102 genes; we predict that many of these are novel pharyngeal gland-expressed genes. These genes are enriched for transcriptional regulators, especially nuclear hormone receptors, suggesting an important role for these factors in the gland cells. We validated this by using single molecule RNA FISH [20] to examine the expression of two TFs from this cluster: *nhr-56* and *ceh-53*. Both of these genes showed expression overlapping with a reporter for the known regulator of gland cell development *hlh-6* (Fig. 5c), indicating that they are also expressed in gland cells. Taken together, our results suggest that membership of a gene in a cluster associated with known anatomy terms is predictive that the gene is expressed in that part of the anatomy.

Some clusters of the 300 contain mostly genes which are expressed at very low levels, suggesting they may represent molecular or technical noise. Other clusters have high expression but little variation between fractions suggesting they contain genes that are more ubiquitously expressed. The clusters also differ in size, from 11 to 822 genes. We predicted the tissue specificity of each cluster using the mean of the absolute value of enrichments across all the sorting experiments. We observed that 86 % of the clusters that are enriched for known tissue-specific annotations had a mean absolute enrichment >0.2 and log-expression >4. Based on this cutoff, we estimate that the genes in at least 103 of the clusters have cell type-specific expression (Fig. 4e, f, Additional file 6: Figure S9).

Only about half of these cell-type specific clusters were enriched for either anatomy ontology terms or previously described tissue specific expression (Additional file 6: Figure S9); this is not surprising, as existing annotations are limited for most cell types. Most *C. elegans* genes’ expression has not been characterized comprehensively across cells, and only a few cell types have been annotated with genome-wide approaches. This suggests that although we only sorted for fourteen markers, the dataset contains information about a much larger number of cell types.

### Enrichment of motifs and TF binding predicts novel regulators of embryonic gene expression

If genes coexpressed in a cluster have common upstream regulators, motifs or binding of these regulators should be enriched in that cluster [52]. Each cluster thus provides an opportunity to identify cell-specific regulators based on enrichment of regulatory motifs or experimentally defined TF binding. We tested each of the 1877 motifs and 146 ChIP-seq data sets described previously for enrichment within upstream intergenic sequences of genes in each cluster. We refer here to these upstream regions as “promoters”, but they likely include both promoter and enhancer elements.

We found 1406 TF binding site motifs and 110 TF ChIP signals enriched in genomic sequence upstream of genes in at least one cluster, (FDR <10^-10,^, Additional file 18: Table S15 and Additional file 19: Table S16). In many cases these motif enrichments were consistent with known regulators. For example, cluster 286 is highly enriched for genes expressed in ciliated neurons (Figs. 4d and 5a). Promoters of genes in this cluster are significantly enriched for the X-box homeodomain motif (*p* < 10^−46^) recognized by the *C. elegans* RFX homolog *daf-19*, which is known to regulate expression in ciliated neurons [53, 54]. Based on this motif enrichment, we predicted that other genes in this cluster are also regulated by *daf-19*.

**Fig. 6:**
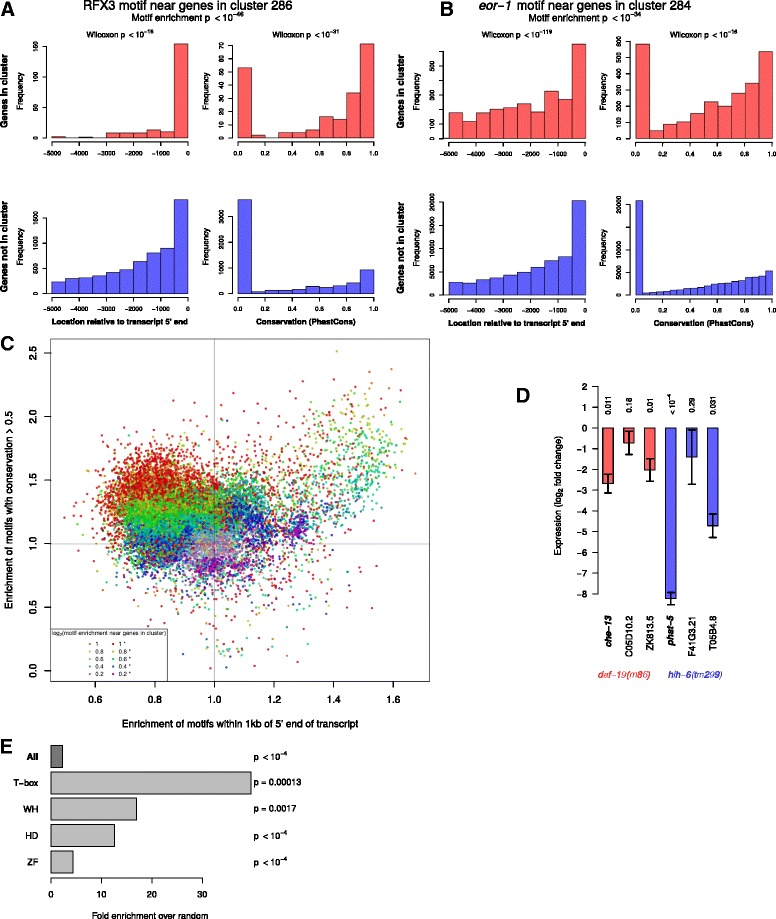
Predicted regulatory relationships. **a** Enrichment of RFX2 motif upstream of genes in cluster 286. **b** Enrichment of an *eor-1* motif upstream of genes in cluster 284. **c** Significance of motifs being more or less conserved, or nearer or further from the TSS (darker dots show cases when at least one of these was significant.) **d** Expression of known (*che-13* and *phat-5*) and predicted targets of *daf-19* and *hlh-6*, when either of those TFs is mutated. **e** Enrichment of TF-cluster pairs in Y1H data from Reece-Hoyes et al. [13]

Similarly, genes in the pharyngeal gland cluster (52) and six other clusters associated with pharyngeal annotations were highly enriched for PHA-4 ChIP binding and a Forkhead binding motif predicted to be bound by PHA-4, consistent with the broad role of *pha-4/*FOXA in regulating pharyngeal expression [37]. The pharyngeal gland cluster was also enriched for an E-box motif predicted to be bound by HLH factors, likely HLH-6, which has highly correlated expression enrichments to this cluster centroid and is known to regulate pharyngeal gland fate [55]. We tested these predictions by using qPCR to examine the expression of three genes from each cluster in mutants for the predicted regulator (either *hlh-6* or *daf-19*) (Fig. 6d). Expression of all six predicted targets was reduced, with 67 % (2/3 for each regulator) reaching statistical significance, indicating they are regulated directly or indirectly by the predicted factors.

Intriguingly, the RFX motif instances in cluster 286 were not uniformly distributed; instead they were highly biased towards positions close to the 5′ end of the annotated transcript (within 1 kb), and in conserved sequences, as compared to RFX motifs near genes outside this cluster (Fig. 6a). The enrichment in conserved sequences is consistent with the known functional importance of DAF-19 in regulating these genes. The enrichment near the transcription start site suggests that DAF-19 primarily acts by binding promoter proximal regulatory elements rather than distal enhancers. In contrast, we identified other cases where an enriched motif was preferentially located further from the 5′ end, suggesting it may act primarily in distal enhancers (Fig. 6b). Based on this, we tested different cutoffs for sequence conservation, and gene-motif distance, and found that TFs differ in those characteristics, with some enriched at specific positions or in conserved sequence, and others more uniformly distributed across upstream sequences (Fig. 6c). Motifs with the highest motif-cluster enrichments tended to be biased for locations further than 1 kb from the 5' end of the annotated transcript, and for higher conservation, although motifs for several other factors in addition to *daf-19* were enriched for proximal locations as well.

We expect that some of the regulators of clustered genes will be expressed in similar patterns to their targets. Consistent with this, many known tissue identity regulators’ expression was highly correlated (*r* > 0.7) with the centroid of a cluster containing genes expressed in that tissue, and also had its predicted binding motif significantly enriched in the same cluster. In total we identified 495 TFs coexpressed with a cluster above a correlation coefficient threshold of 0.7 and whose predicted binding motif was enriched at FDR corrected *p* < 0.001 with 50 clusters, providing many novel candidate regulators for diverse embryonic cells. For example, genes in the “coelomocyte” cluster (30) were enriched for the presence of a Forkhead binding motif in their promoters (5-fold, *p* < 10^−7^), and expression of a Forkhead TF predicted to bind that motif, *let-381*, was highly correlated with that cluster’s centroid (*r* = 0.94). *let-381* is known to be important for development of postembryonic-derived coelomocytes [56], and our work suggests that it also regulates embryonic coelomocyte development. Other prominent examples include *daf-19* in the ciliated neuron cluster, *pha-4* in the pharyngeal gland cluster (cluster 52) and *hlh-1* in a presumed body-wall muscle cluster (cluster 72).

Direct binding of most *C. elegans* TFs to the promoters of other TFs has been assessed using yeast 1-hybrid interactions [13]. We found that TFs that can bind to the promoters of one or more genes in a cluster were significantly more likely to have their motif enriched in that cluster, compared to random pairs of genes. This enrichment was higher for some classes, such as homeodomain and zinc fingers (Fig. 6e). This supports the idea that genes in our clusters often share biological functions, and that the motifs we find often correspond to actual regulatory relationships.

### Identification of cell type-specific patterns of noncoding RNA expression

**Fig. 7:**
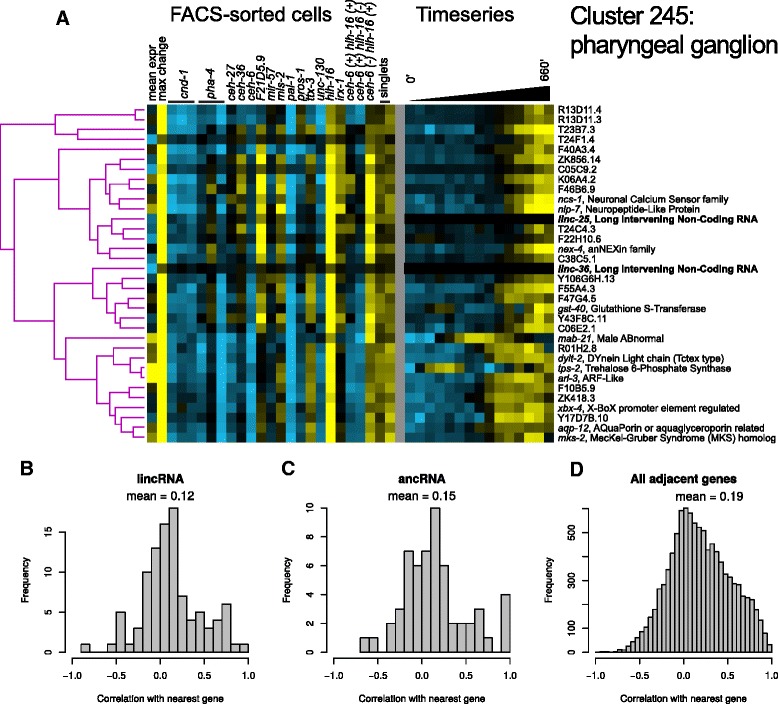
Non-coding RNAs. **a** Cluster containing *linc-25*, *linc-36* and genes with known neural expression patterns. **b**-**d** Correlation of (**b**) ancRNAs, (**c**) lincRNAs, and (**d**) all pairs of genes with their nearest neighboring gene

Improved array and sequencing technology have revealed many expressed non-coding transcripts [57], including long noncoding RNAs (lincRNA) and RNAs that are antisense to protein-coding genes (ancRNAs) [58]. This noncoding transcription is often tissue-specific [59, 60]. Our strand-specific RNA sequencing data allowed us to differentiate the expression of non-coding RNAs and nearby genes, even if they overlap on opposite strands. Consistent with previous studies, we saw higher average expression and FACS enrichment for coding transcripts compared with previously annotated lincRNA and ancRNAs. Also consistent with previous studies [58], we found a positive correlation (*r* = 0.12, *n* = 99 gene pairs, Wilcoxon *p* < 0.0005; Fig. 7b) between expression of lincRNAs and that of the nearest annotated coding gene. We found a similar positive expression correlation between antisense “ancRNAs” and the overlapping gene (*r* = 0.15, *n* = 57 gene pairs, *p* < 0.005; Fig. 7c). The mean and distribution of coexpression of lincRNAs with neighboring genes is similar to that seen between adjacent protein coding genes (*r* = 0.19, Fig. 7d) [58]. We found a similar positive expression correlation between antisense “ancRNAs” and the overlapping gene (*r* = 0.15, *n* = 57 gene pairs, *p* < 0.005; Fig. 7c).

We identified many non-coding RNAs that cluster with tissue specific genes (Additional file 20: Table S17). For instance, cluster 245 includes *linc-25* and *linc-36*, and is also significantly enriched for genes expressed in pharyngeal ganglia (Fig. 7a). Since these genes are enriched in a similar set of fractions, we expect that they are also expressed in a similar pattern of cells. In total, 29 lincRNAs and 3 ancRNAs were expressed at levels greater than 1 RPM in one or more samples within 17 clusters for which we have an annotated tissue type, and another 52 lincRNAs and 12 ancRNAs were expressed in putative cell type-specific clusters for which the tissue type is unknown (Additional file 20: Table S17). We conclude that our data identifies many noncoding RNAs likely to be differentially expressed in the embryo.

## Discussion

### A path towards profiling gene expression genome-wide at cellular resolution across the entire organism

While previous studies focused on purifying specific cell types, our “Profiling of Overlapping Populations of cells (POP-Seq)” approach to identifying tissue specific expression in principle provides information about all embryonic cell types. Using FACS sorting and RNA-seq, we found groups of genes which are expressed across a panel of partially overlapping cell populations that collectively cover the entire embryo. Each embryonic cell type has a specific pattern of enrichment and depletion across these cell populations, allowing us to identify genes expressed in tissues that have not been profiled by genome-wide approaches, as demonstrated for the pharyngeal glands. Thus we have substantially expanded our knowledge of spatially patterned gene expression across the embryo. Our dataset provides a powerful resource for *C. elegans* developmental geneticists interested in specific cell types. By identifying genes coexpressed with genes they already know are expressed in their cells of interest (as by using our web tool), they can identify potential players in the development of those cells. Similarly, the TFs for which binding or motifs are enriched near those genes provide candidate regulators of those cells’ development.

In our analysis, we identified coexpressed genes by clustering. In principle, because our data include both the expression of each gene in each cell population and the identity of the cells in each population, it should be possible to predict the expression of each gene in each cell. Previous deconvolution experiments in other systems such as the *Arabidopsis* root [61] or zebrafish embryo [62] estimated expression essentially on a grid. In contrast, our strategy uses cell populations that share partially overlapping lineage histories to increase our resolution to identify differentially expressed genes. We previously developed a computational unmixing strategy to perform this deconvolution [29]. Applying this approach to simulated data suggested that at least 30 fractions are needed for this strategy to yield accurate high-resolution expression patterns, but that smaller numbers of fractions can yield useful information about cell populations. Consistent with this, applying these methods to the data in this study gave statistically meaningful predictions. For example, the cell-specific expression predictions resulting from deconvolution could be used to predict the expression within a cell fraction not used for deconvolution (median *r* = 0.46 for leave-one-out cross validation) (Additional file 6: Figure S6). While the accuracy of these predictions is not yet high enough to be useful at resolution approaching single cells, our results suggest that with additional data, the deconvolution approach may allow high-resolution inference of expression genome-wide across all cells.

### Implications for regulatory inference

This study identified substantially more motif-cluster enrichments than were observed previously. For example, [23] identified 35 motifs enriched upstream of clusters defined by coexpression across cell types [23], while we identified 495 motif-cluster associations. This could reflect differences in the information content of the underlying data, the clustering approach, or the motif database. Applying our clustering and motif enrichment approach to the data in [23] or [63] identifies a number of motifs comparable to what we found in this study, suggesting that the increased sensitivity reflects differences in the motif association itself.

The past few years have seen dramatic growth in our knowledge of experimentally defined TF binding specificities [12–15]. Consistent with this, we identified many more enriched motif-cluster pairs from the experimentally determined binding sites than when we used FIRE [64], a *de novo* motif finding algorithm (495 vs 169). Clustering the Spencer data similarly into 300 clusters, and running FIRE on the resulting clusters yielded a similar number of motifs (177, upstream of 116 clusters.) This is more motifs than Spencer et al. [23] found, but still smaller than the number we found using known motif data. Thus, the recent influx of data on TF binding specificity provides a dramatic boost to regulatory inference. Our observation that motif enrichment was often biased towards particular levels of conservation or positions relative to the transcription start site suggests that new algorithms to integrate motif enrichment with these and other types of information (such as chromatin features) may further improve regulatory predictions.

## Conclusions

In summary, we described here a new resource of genome-wide transcript level measurements for partially overlapping cell populations from *C. elegans* embryos. We used this dataset to identify thousands of genes differentially expressed across the developmental lineage and to infer new regulators of embryonic development. This resource provides useful data for future studies of embryonic gene regulation in C. elegans and the methods may be broadly useful in other systems where partially overlapping cell populations can be isolated.

## Methods

### Reporters

We used strains containing integrated multicopy reporters, either promoter::mCherry::histone fusions [19] or C-terminal translational GFP fusions [30], along with a second-color histone-GFP or histone-mCherry reporter for cell tracking (Additional file 1: Table S1). We collected confocal 4D images of each strain by resonance scanning confocal microscopy [31] and measured expression of markers in each cell using StarryNite [6] and AceTree, as described in [19], through the hypodermal enclosure stage. After this stage, embryos are no longer dissociated by our protocol; therefore, any cells that become fluorescent after this stage will not be sorted.

### Flow sorting

For each reporter, worms were grown, and bleached to obtain embryos. The eggshells were dissolved with chitinase, and cells were isolated using standard methods [65]. Dead cells were identified and gated out using DAPI, and for singlet cells, forward/side-scatter gating, respectively. Fluorescent positive and negative cell populations were gated from the singlet population. Sort purity was measured by re-sorting each purified sample and ranged from 0.82 to 0.97, with a median of 0.88. We also used one strain (UP2216) expressing both GFP and mCherry in distinct patterns (CEH-6::GFP; *hlh-16*_*promoter*_*::*Histone*-*mCherry); we isolated four cell populations from this strain: GFP and mCherry single-positive cells, double-positive cells, and double-negative cells.

### Sequencing and transcript quantification

We extracted total RNA from each cell population using a RNAeasy kit (Qiagen), amplified the poly-A RNA using a T7 RNA polymerase aRNA protocol (Ambion MessageAMP II aRNA kit), and sequenced cDNA using SoLID sequencing, resulting in strand-specific paired-end reads with 50 bp on one end, and 35 bp on the other. We aligned reads to the WS220 (ce10) build of the *C. elegans* genome, using TopHat version 2.0.10 [66], with *de novo* junction search disabled. We aligned to 30,317 annotated genes, including 20,386 annotated as protein-coding, the “7 k ncRNA set” from [57], and the 227 non-coding RNAs from [58]. We first aligned the full-length reads; reads that didn’t match were trimmed (from 50 to 40 nt at one end, and from 35 to 29 nt at the other end), and remapped. This resulted in a median of 15 million mapped reads per sample (Additional file 4: Table S3). We measured expression as the number of reads mapping within each gene's exons, on the same strand, normalized to one million reads per sample (reads per million, or “RPM”), omitting mitochondrial and ribosomal RNA.

We computed enrichment as ${ \log}_2\frac{3+\mathrm{R}\mathrm{P}\mathrm{M}\ \mathrm{in}\ \left(+\right)\ \mathrm{fraction}}{3+\mathrm{R}\mathrm{P}\mathrm{M}\ \mathrm{in}\ \left(-\right)\ \mathrm{fraction}}$. A “pseudocount” of 3 RPM was used to conservatively estimate enrichment of genes with very low read counts. For two samples, we didn't have matched negative controls (*hlh-16, irx-1*); in these cases we computed enrichment relative to singlet cells. Since any of the gated samples (positive or negative) should be a subset of this singlet sample, this provides a conservative estimate of the actual enrichment. This was generally true, as mean enrichments were lower when calculated based on (Additional file 6: Figure S2). In the case of the cells double-sorted by *ceh-6* and *hlh-16*, we computed enrichments relative to the *ceh-6* (-) *hlh-16* (-) sample.

We called genes as “enriched” or “depleted” using an enrichment cutoff of 2 (corresponding to 4-fold changes), since enrichment or depletion at this level in one sample predicted whether the gene was enriched or depleted in a biological replicate sample with an average accuracy of 98.7 % (Fig. 2a, Additional file 6: Figure S11). To plot enrichment relative to time (Additional file 6: Figure S4), we computed the mean and standard deviation of when a gene was enriched in timeseries data [34]; genes with standard deviation below a cutoff were considered time-specific (Additional file 6: Figure S12).

Coexpression of genes in the pharyngeal gland cells was assessed by single-molecule RNA FISH, performed as previously described [20]. Briefly, we designed probes targeting GFP, *ceh-53* or *nhr-56*, and stained in strain VL7 [45], which expresses GFP in the pharyngeal glands from an *hlh-6* promoter. We used Taqman assays to measure expression of candidate targets in triplicate in TF mutants, from RNA collected using RNeasy kit (Fig. 6c).

### Clustering

We hierarchically clustered [67] the enrichments from the FACS data using correlation distance and complete linkage, using the amap package in R [68]. We displayed the clustered FACS data with an embryonic expression time series from the modENCODE project [34], which we log(2) transformed, mean-centered and standardized. We visualized the resulting clusters using TreeView [69], and provided a custom visualization webpage (Additional file 13)

### Comparison with other resources

We compared our clustering with WormNet [18], by counting how often two genes annotated as related by WormNet were in the same cluster (Fig. 5d), compared to a random shuffling. We compared this to the probability of two independently-chosen genes being in the same cluster, based on the cluster sizes. We similarly compared our clustering with a Y1H dataset [13] by measuring the proportion of TF-cluster enrichments for which there was a Y1H interaction found, with that TF as prey, and the bait in the cluster (Additional file 6: Figure S6E). In each cluster, we measured Gene Ontology enrichment using the GOstats R package, and Anatomy Ontology and WormBase Expression Cluster enrichment using a hypergeometric test.

### Motif analysis

We searched for enrichment of 1877 known TF binding motifs, including 1493 motifs either from 291 *C. elegans* TFs, or from TFs in other species orthologous to worm TFs [15]. We also searched for enrichment of 384 TF motifs from other organisms (101 fly, 88 mouse, and 195 human) which were not considered to have worm orthologs according to [15], but had worm orthologs according to at least one of Ensembl [70], Entrez Homologene [71], InParanoid [72], OrthoMCL [73], or WormBase [74].

Many TFs bind similar sequence motifs; to reduce redundancy, we compared the motifs using STAMP [75], clustering motifs with a PCC distance less than 0.01 into clusters, and only keeping one motif from each cluster.

### Motif and ChIP enrichment

We scanned for the known motifs using the *fimo* program from the MEME suite [76]. We counted motif occurrences upstream of each cluster, using different cutoffs for distance upstream of TSS (1, 2, or 3 kb), PhastCons [77] conservation score (0, 0.5, 0.7, or 0.9), and motif log p score (30, 35, or 40.) We then measured enrichment of those motifs using a hypergeometric test [78], adjusting *p*-values using the False Discovery Rate [79]. We used a similar procedure without the score component to identify enriched ChIP peaks.

### Deconvolution

To deconvolute expression of each gene in each embryonic cell, we used the pseudoinverse on fold-enrichment values as in [29]. For cross-validation, we left one sample out when performing the deconvolution, then used the deconvoluted expression values to predict the expression in the left-out sample, repeating this for each sample. We omitted the *ceh-6* and *hlh-16* “double-positive” sorts from the input data in this analysis, but included them in the testing. In the case of the double-sorted fractions, accuracies in predicting the *ceh-6(+);hlh-16(-)* and *ceh-6(-);hlh-16(+)* experiments (mean *r* = 0.84) were noticeably better than the accuracy in predicting the *ceh-6(+);hlh-16(+)* “double positive” experiment (*r* = 0.46). This suggests that the unmixing is successfully combining the *ceh-6* and *hlh-16* data to “rule out” expression in a subset of cells (although it is less successful in predicting expression in their overlap).

### Data availability

The aligned sequence data are available in the Sequence Read Archive (SRA) at accession SRP063953.

## Additional files

Additional file 1: Table S1. Strains and genotypes of embryos used. (XLSX 6 kb) Additional file 2: Figure S1. Expression of fourteen reporters used for FACS sorting, measured by lineage tracing. (PDF 21 kb) Additional file 3: Table S2. Expression intensity of reporters in each cell. (XLSX 109 kb) Additional file 4: Table S3. Total mapped reads for each experiment. (XLSX 5 kb) Additional file 5: Table S4. Reads per million for each gene. (XLSX 6277 kb) Additional file 6: Other supplemental figures. **Figure S2.** Enrichments for two replicates of *cnd-1* sorting. **Figure S3.** Comparison of enrichments using a matched control. *x*-axis: enrichment of (+) sample, compared to the corresponding (-) sample. *y*-axis: enrichment of (+) sample compared to singlet control, rather than the non-expressing (-) sample corresponding to a given expressing (+) experiment. (*hlh-16* and *irx-1* are omitted, as they lacked a matching (-) control.). **Figure S4.** Enrichments for selected pairs of samples, calculated for time-specific genes from Li et al. [34]. **Figure S5.** Accuracy predicting the expression in ungated samples using only (+) and (-) samples (*x-*axis), or using the (+), (-), and singlet control samples (*y*-axis). **Figure S6.** Unmixing cross-validation accuracy. For each sort marker *s*, the *x-*axis shows measured enrichment computed from the *s* (+) and *s* (-) samples. The *y*-axis shows the enrichment predicted for *s,* based on the measured expression of all samples except *s*. **Figure S7.** Correlation of expression patterns for genes in different clusters and the same cluster, for (A) 121 embryonic expression patterns from Murray et al. [7] and (B) 93 expression patterns from L1 stage larvae from Liu et al. [50]. **Figure S8.** Comparison of clustering of FACS-sorted samples from this study, and Spencer et al. [23]. (A) Average within-cluster correlation of genes in Spencer et al. [23] data (*x*-axis) and FACS-sorted data from this study (*y*-axis), when genes were clustered using the Spencer et al. [23] data. (B) Opposite comparison: average within-cluster correlation of genes in FACS-sorted data from this study (*x*-axis) and Spencer et al. [23] data (*y*-axis), when genes were clustered using the FACS-sorted data. (C) Part of cluster colored red in (A), clustered by Spencer et al. [23] data. (D) Part of cluster colored red in (B), clustered by FACS-sorted data from this study. **Figure S9.** Mean expression and mean absolute enrichments of clusters (as in Fig. 4e), with all clusters labeled. **Figure S10.** Annotation enrichment as in Fig. 5, but for all clusters. **Figure S11.** Reproducibility of enrichments, at different cutoffs. For each cutoff on the *x*-axis in one sample, the *y*-axis shows the fraction of genes which were enriched in a replicate experiment. **Figure S12.** Mean and standard deviation of when genes were expressed, using expression timeseries from Li et al. [34]. Genes below the horizontal line were considered “time-specific”, and used in plotting enrichments relative to time. (PDF 2495 kb) Additional file 7: Table S5. Computed enrichments for each gene. (XLSX 5515 kb) Additional file 8: Table S6. Anatomy terms enriched in FACS-sorted samples. (XLSX 188 kb) Additional file 9: Table S7. Expression annotation enriched in FACS-sorted samples. (XLSX 162 kb) Additional file 10: Table S8. Gene ontology terms enriched in FACS-sorted samples. (XLSX 93 kb) Additional file 11: Table S9. Motifs enriched upstream of genes in FACS-sorted samples. (XLSX 5512 kb) Additional file 12: Table S10. ChIP signals enriched upstream of genes in FACS-sorted samples. (XLSX 361 kb) Additional file 13: Web supplement. (DOC 21 kb) Additional file 14: Table S11. Number of motifs found significant using different numbers of clusters, at different cutoffs. (XLSX 5 kb) Additional file 15: Table S12. Anatomy terms enriched in clustered genes. (XLSX 34 kb) Additional file 16: Table S13. Expression annotation in clustered genes. (XLSX 105 kb) Additional file 17: Table S14. Gene ontology terms in clustered genes. (XLSX 102 kb) Additional file 18: Table S15. Motifs enriched upstream of clustered genes. (XLSX 3423 kb) Additional file 19: Table S16. ChIP signals enriched upstream of clustered genes. (XLSX 239 kb) Additional file 20: Table S17. Non-coding RNAs which clustered in genes with enriched anatomy annotation. (XLSX 7 kb)

## Abbreviations

FACS: Fluorescence-activated cell sorting
POP-Seq: Profiling of Overlapping Populations of cells for RNA-Seq
TF: Transcription factor
