# Supplementary figures and images for: Overlapping cell population expression profiling and regulatory inference in C. elegans
Source: BMC Genomics. 2016 Feb 29;17:159. doi: 10.1186/s12864-016-2482-z (PMC4772325; doi:10.1186/s12864-016-2482-z)

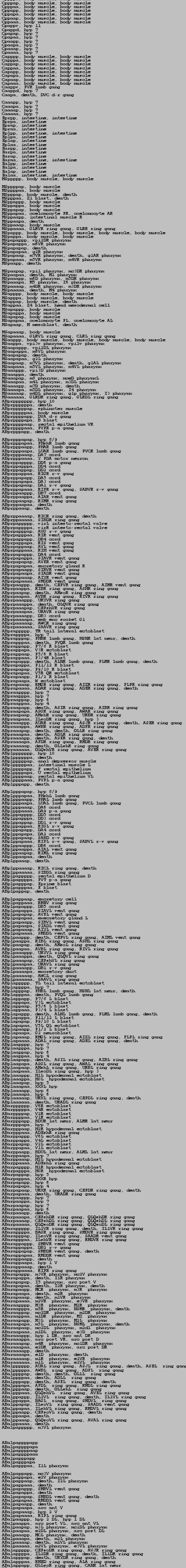







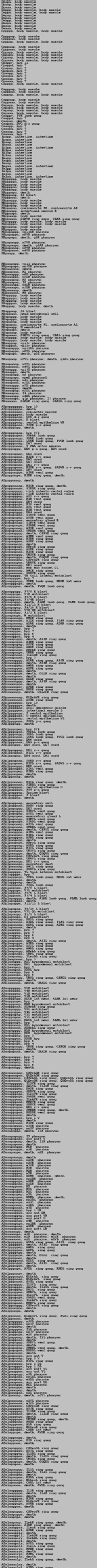











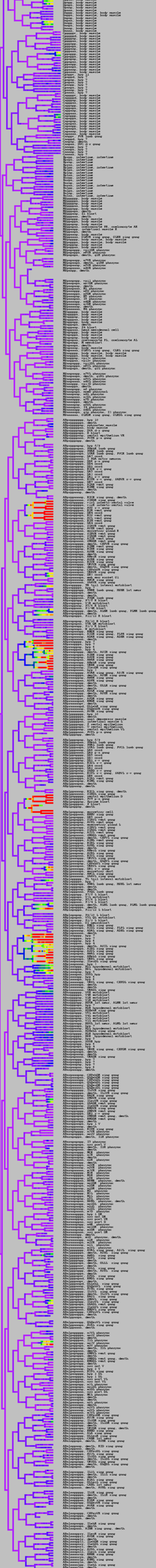

Supplement: Additional file 2: Figure S1. — Expression of fourteen reporters used for FACS sorting, measured by lineage tracing. (PDF 21 kb) [file 12864_2016_2482_MOESM2_ESM.pdf]
