# Supplementary material for: Overlapping cell population expression profiling and regulatory inference in C. elegans
Source: BMC Genomics. 2016 Feb 29;17:159. doi: 10.1186/s12864-016-2482-z (PMC4772325; doi:10.1186/s12864-016-2482-z)
Supplement: Additional file 6: — Other supplemental figures. Figure S2. Enrichments for two replicates of cnd-1 sorting. Figure S3. Comparison of enrichments using a matched control. x-axis: enrichment of (+) sample, compared to the corresponding (-) sample. y-axis: enrichment of (+) sample compared to singlet control, rather than the non-expressing (-) sample corresponding to a given expressing (+) experiment. (hlh-16 and irx-1 are omitted, as they lacked a matching (-) control.). Figure S4. Enrichments for selected pairs of samples, calculated for time-specific genes from Li et al. [34]. Figure S5. Accuracy predicting the expression in ungated samples using only (+) and (-) samples (x-axis), or using the (+), (-), and singlet control samples (y-axis). Figure S6. Unmixing cross-validation accuracy. For each sort marker s, the x-axis shows measured enrichment computed from the s (+) and s (-) samples. The y-axis shows the enrichment predicted for s, based on the measured expression of all samples except s. Figure S7. Correlation of expression patterns for genes in different clusters and the same cluster, for (A) 121 embryonic expression patterns from Murray et al. [7] and (B) 93 expression patterns from L1 stage larvae from Liu et al. [50]. Figure S8. Comparison of clustering of FACS-sorted samples from this study, and Spencer et al. [23]. (A) Average within-cluster correlation of genes in Spencer et al. [23] data (x-axis) and FACS-sorted data from this study (y-axis), when genes were clustered using the Spencer et al. [23] data. (B) Opposite comparison: average within-cluster correlation of genes in FACS-sorted data from this study (x-axis) and Spencer et al. [23] data (y-axis), when genes were clustered using the FACS-sorted data. (C) Part of cluster colored red in (A), clustered by Spencer et al. [23] data. (D) Part of cluster colored red in (B), clustered by FACS-sorted data from this study. Figure S9. Mean expression and mean absolute enrichments of clusters (as in Fig. 4e), with all clusters [file 12864_2016_2482_MOESM6_ESM.pdf]

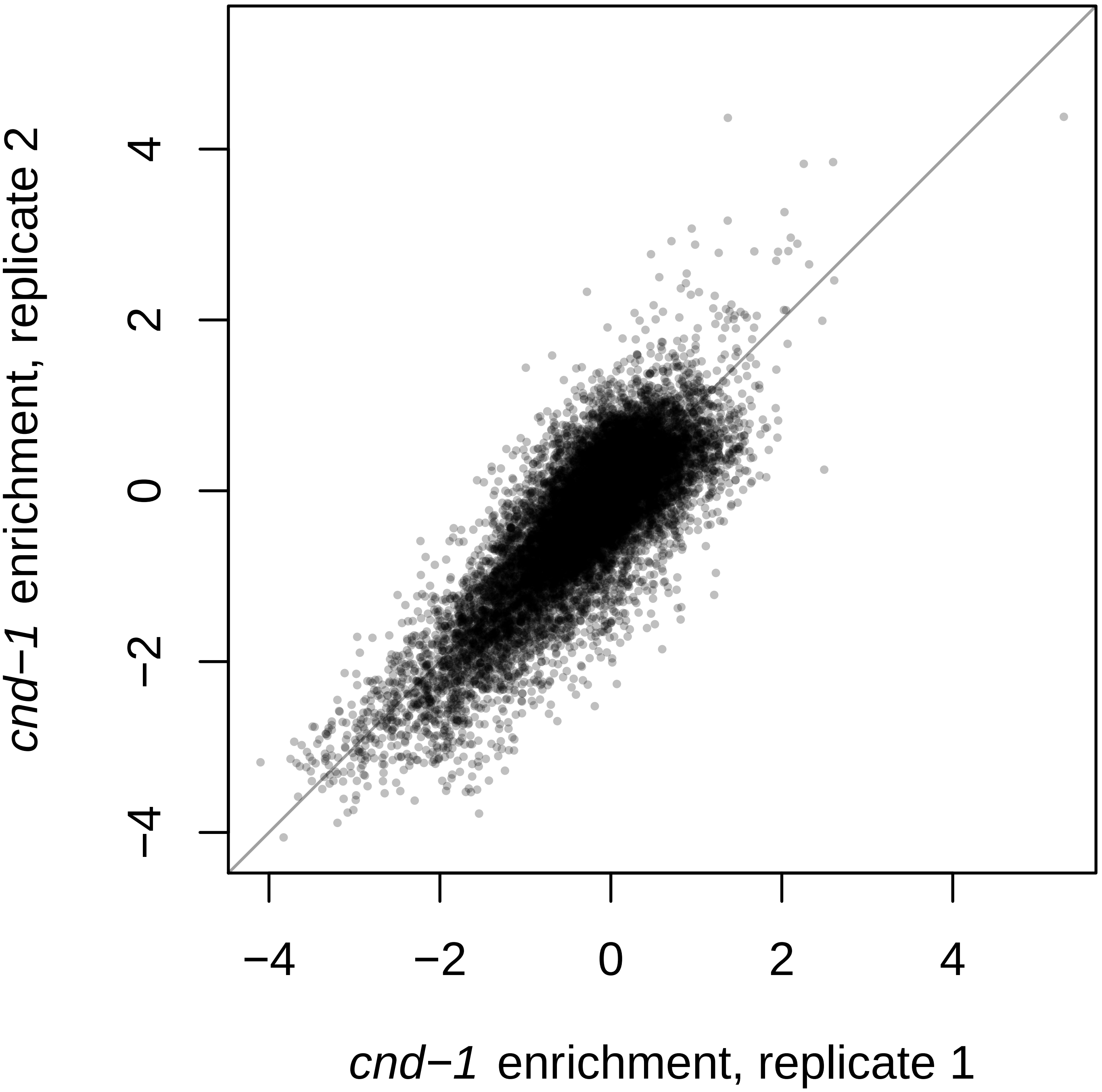

Figure S2: Enrichments for two replicates of *cnd-1* sorting.

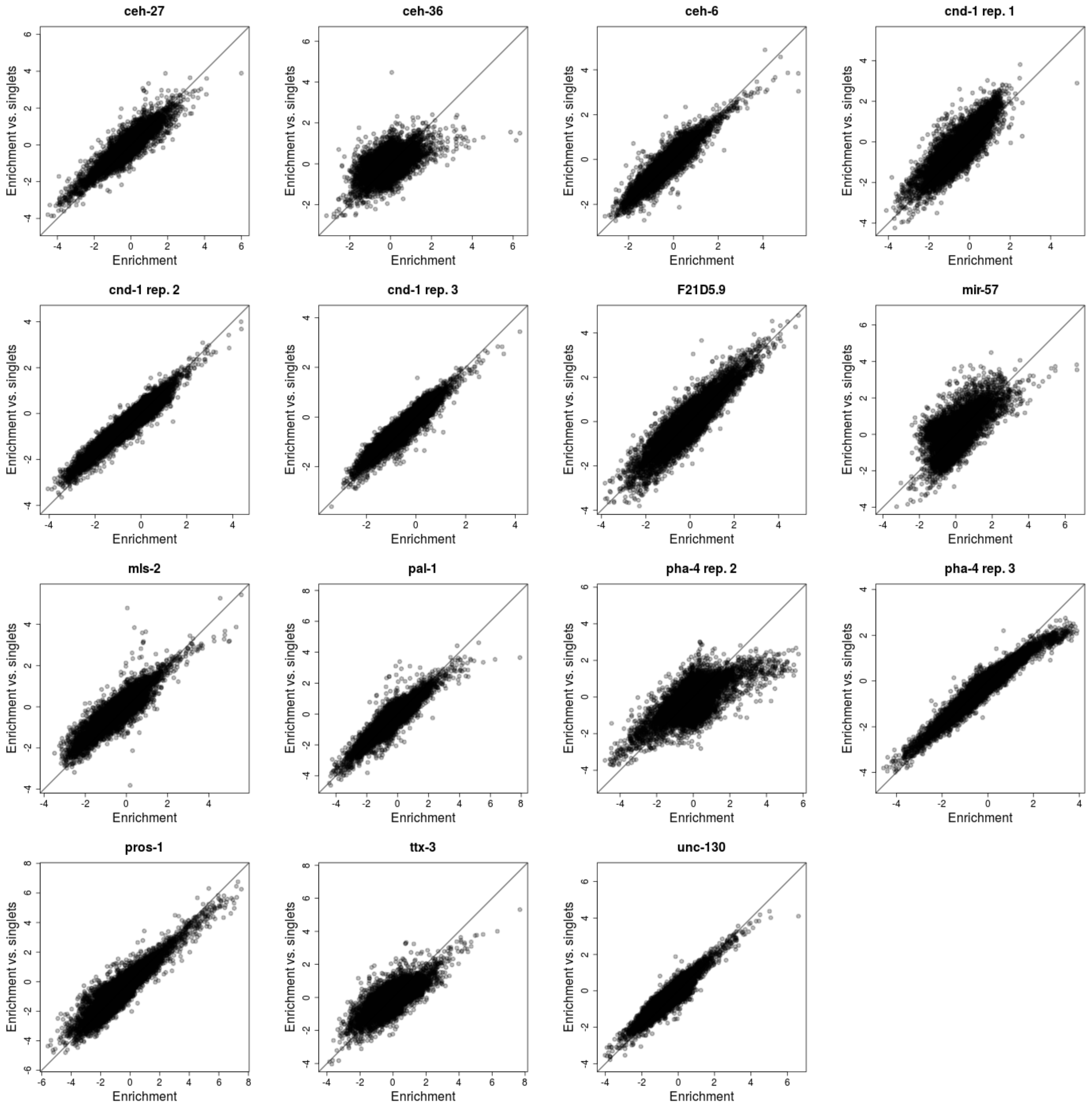

Figure S3: Comparison of enrichments using a matched control. x-axis: enrichment of (+) sample, compared to the corresponding (-) sample. y-axis: enrichment of (+) sample compared to singlet control. rather than the non-expressing (-) sample corresponding to a given expressing (+) experiment. (*hlh-16* and *irx-1* are omitted, as they lacked a matching (-) control.)

Figure S4: Enrichments for selected pairs of samples, calculated for time-specific genes from (Li et al. 2014).

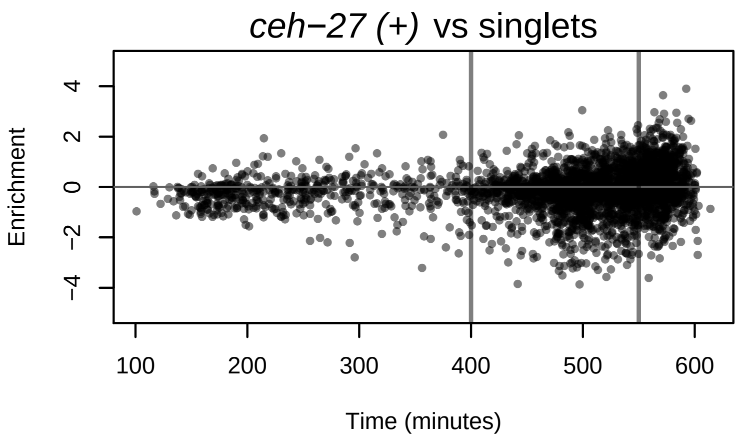
ceh−27 (+) vs singlets
Enrichment
Time (minutes)

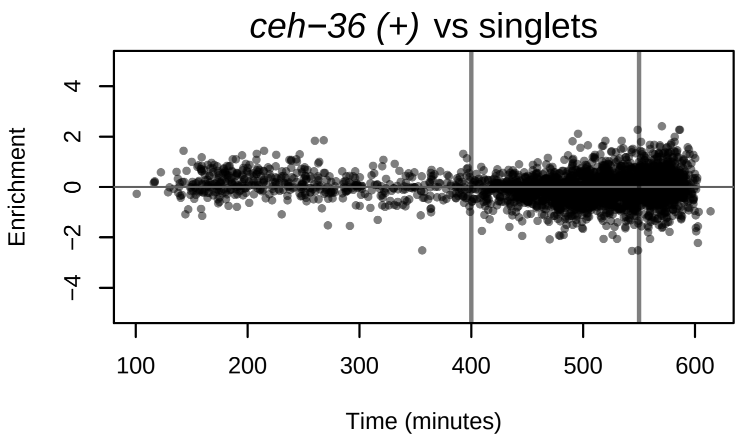
ceh−36 (+) vs singlets
Enrichment
Time (minutes)

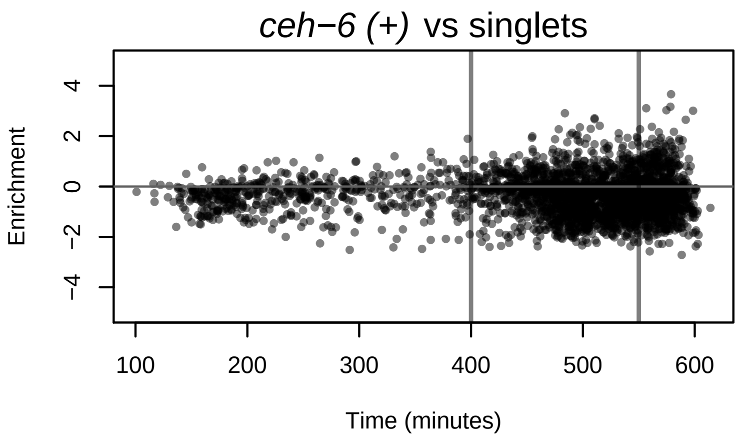
ceh−6 (+) vs singlets
Enrichment
Time (minutes)

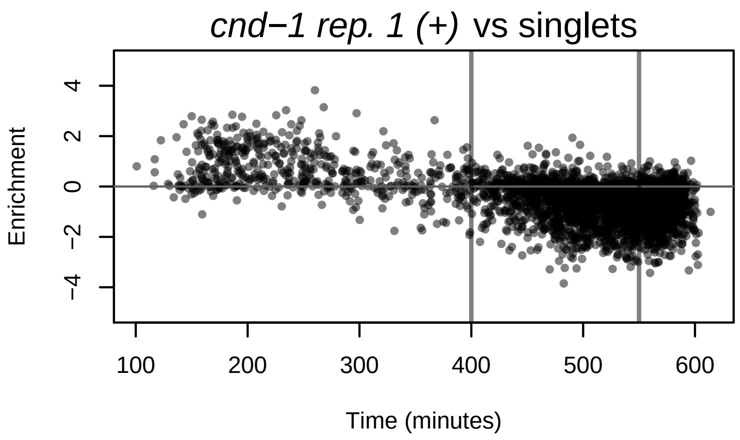
cnd−1 rep. 1 (+) vs singlets
Enrichment
Time (minutes)

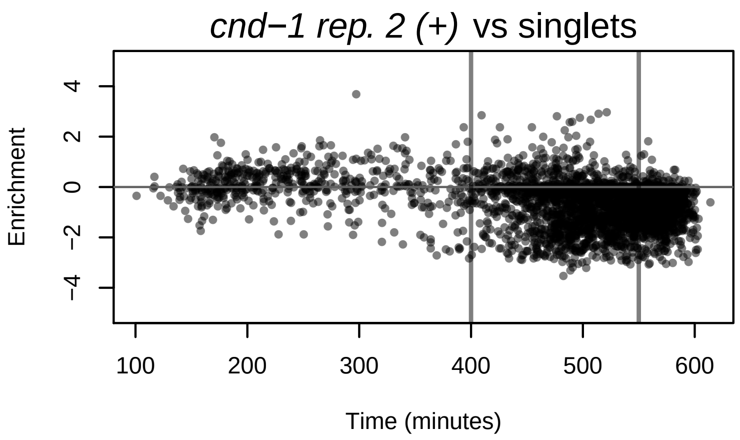
cnd−1 rep. 2 (+) vs singlets
Enrichment
Time (minutes)

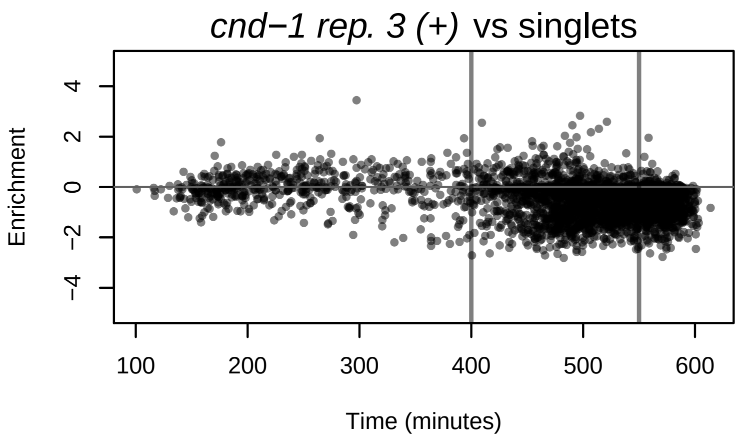
cnd−1 rep. 3 (+) vs singlets
Enrichment
Time (minutes)

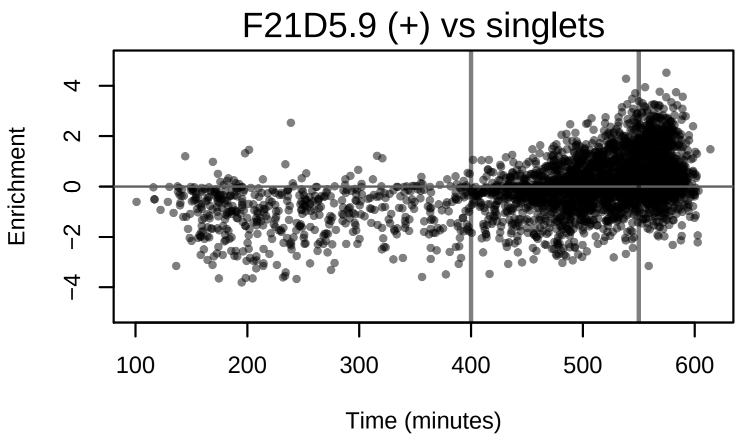
F21D5.9 (+) vs singlets
Enrichment
Time (minutes)

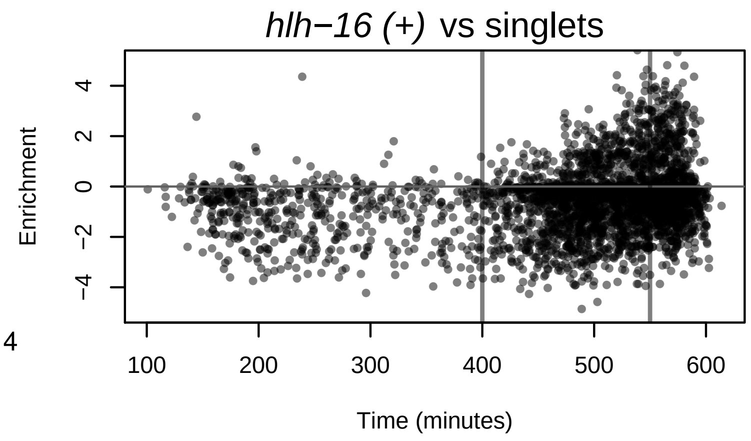
hlh−16 (+) vs singlets
Enrichment
Time (minutes)

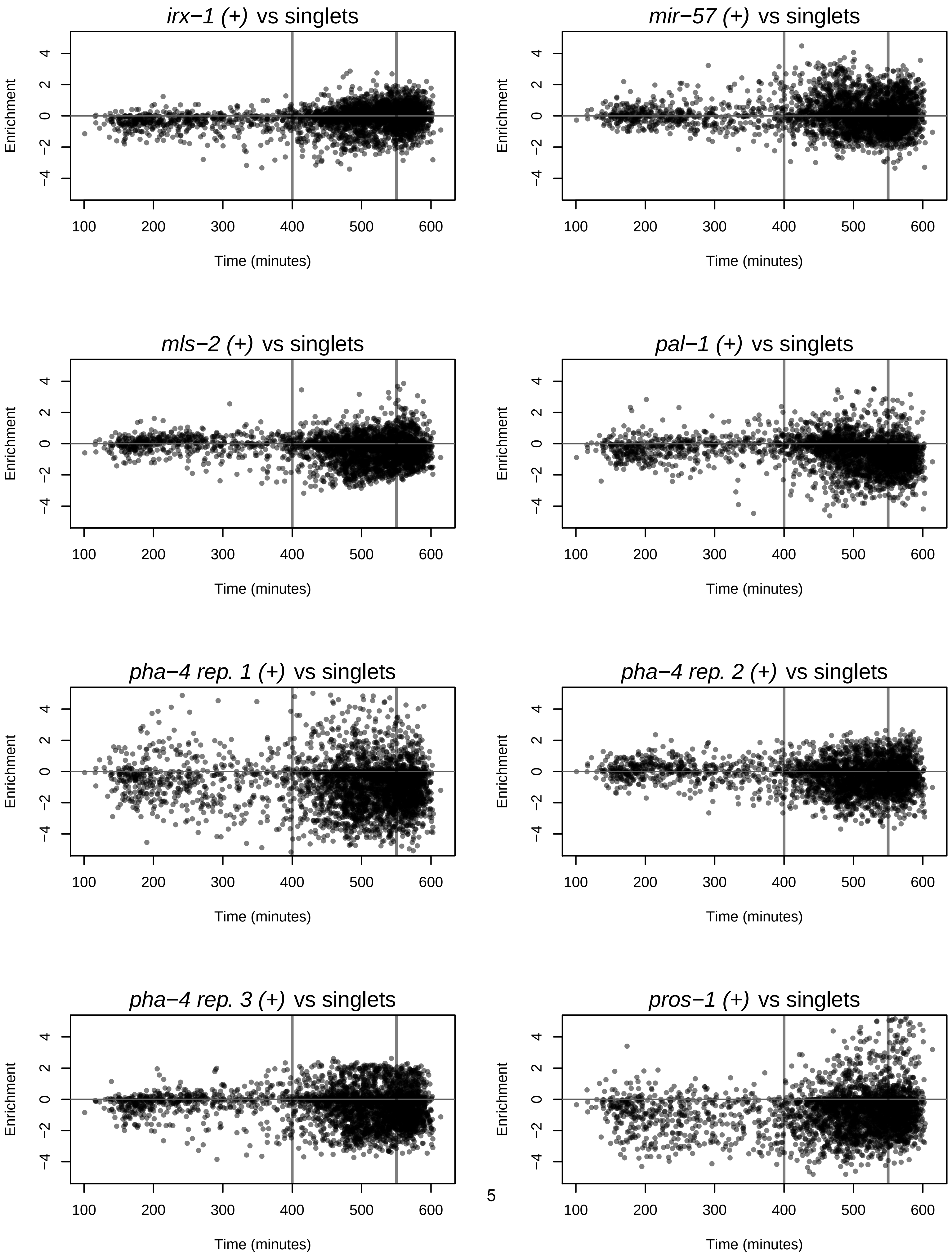
irx−1 (+) vs singlets
mir−57 (+) vs singlets
mls−2 (+) vs singlets
pal−1 (+) vs singlets
pha−4 rep. 1 (+) vs singlets
pha−4 rep. 2 (+) vs singlets
pha−4 rep. 3 (+) vs singlets
pros−1 (+) vs singlets
Enrichment
Time (minutes)
−4 −2 0 2 4
100 200 300 400 500 600

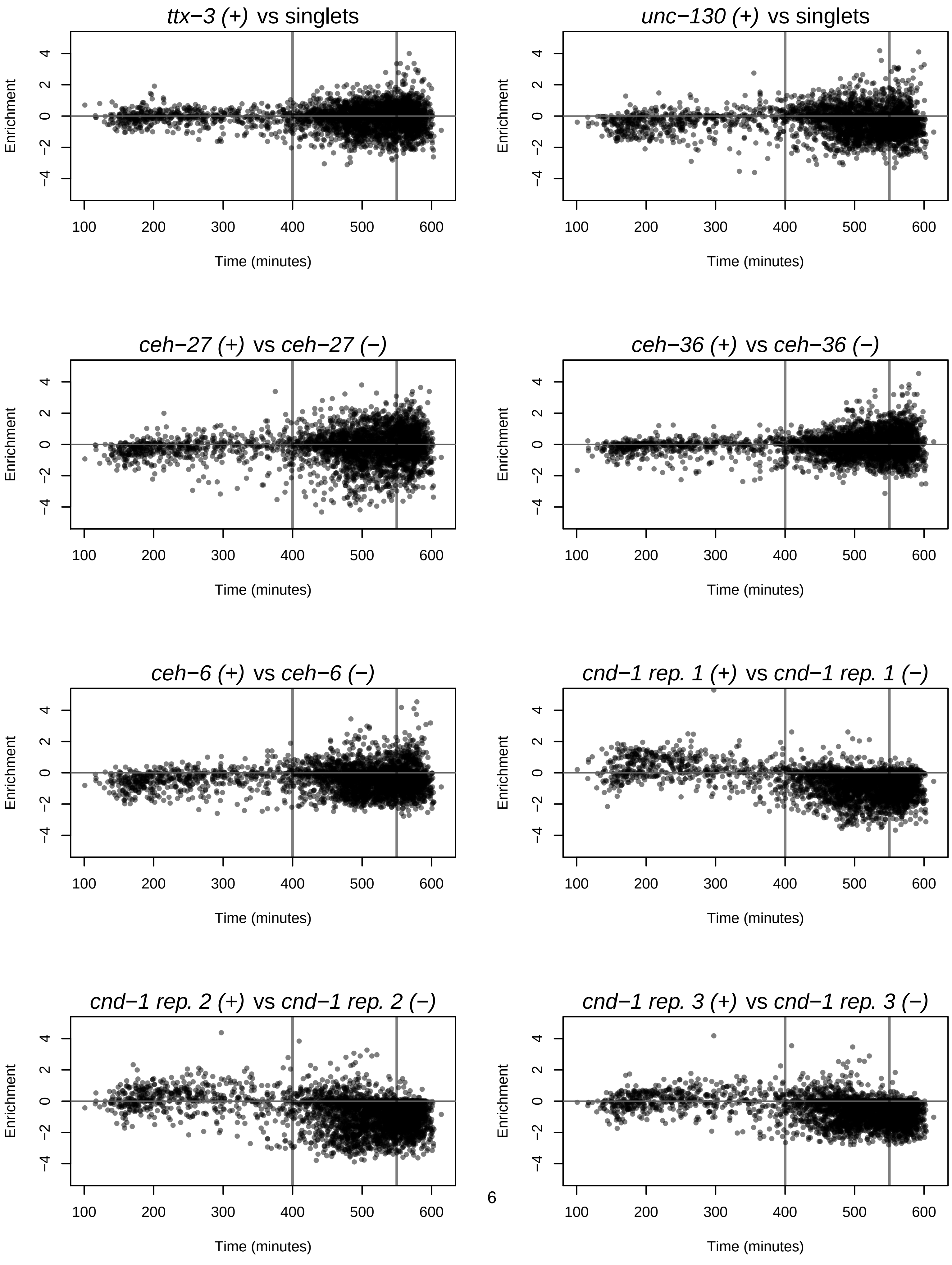
ttx−3 (+) vs singlets
unc−130 (+) vs singlets
ceh−27 (+) vs ceh−27 (−)
ceh−36 (+) vs ceh−36 (−)
ceh−6 (+) vs ceh−6 (−)
cnd−1 rep. 1 (+) vs cnd−1 rep. 1 (−)
cnd−1 rep. 2 (+) vs cnd−1 rep. 2 (−)
cnd−1 rep. 3 (+) vs cnd−1 rep. 3 (−)
Enrichment
Time (minutes)
−4
−2
0
2
4
100
200
300
400
500
600

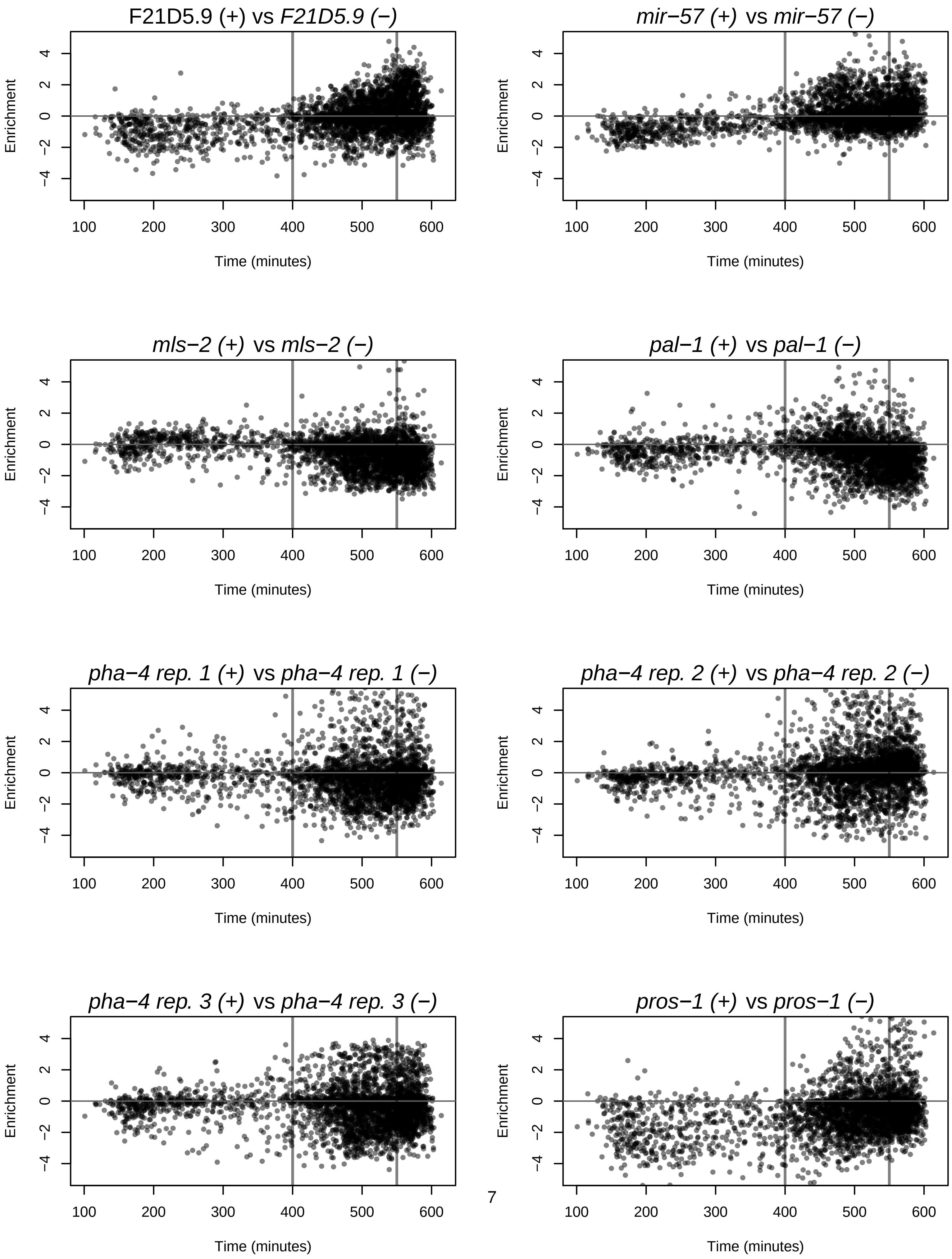
F21D5.9 (+) vs F21D5.9 (−)
mir−57 (+) vs mir−57 (−)
mls−2 (+) vs mls−2 (−)
pal−1 (+) vs pal−1 (−)
pha−4 rep. 1 (+) vs pha−4 rep. 1 (−)
pha−4 rep. 2 (+) vs pha−4 rep. 2 (−)
pha−4 rep. 3 (+) vs pha−4 rep. 3 (−)
pros−1 (+) vs pros−1 (−)
Enrichment
Time (minutes)
−4
−2
0
2
4
100
200
300
400
500
600

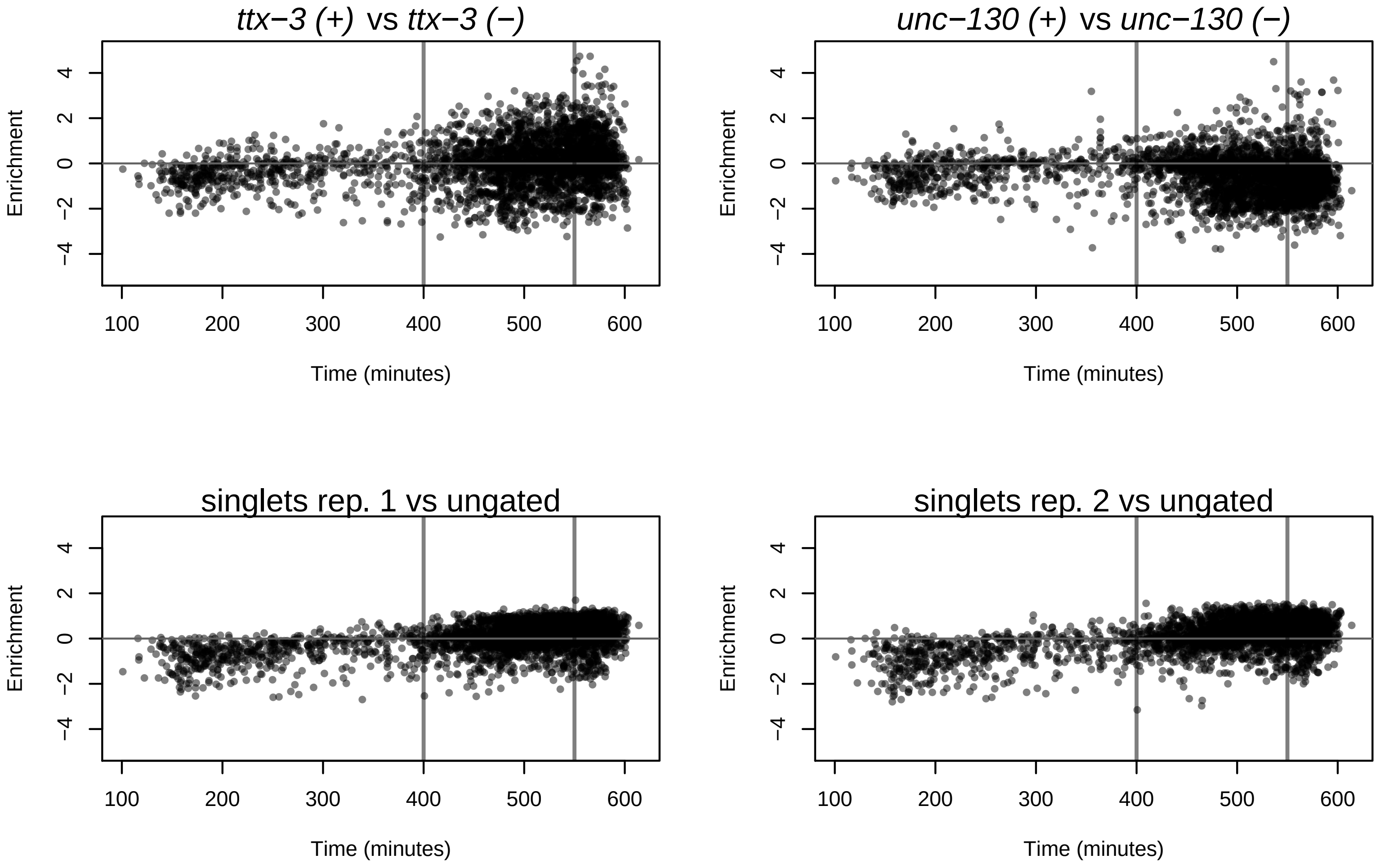
ttx−3 (+) vs ttx−3 (−)
unc−130 (+) vs unc−130 (−)
singlets rep. 1 vs ungated
singlets rep. 2 vs ungated
Enrichment
Time (minutes)
−4
−2
0
2
4
100
200
300
400
500
600

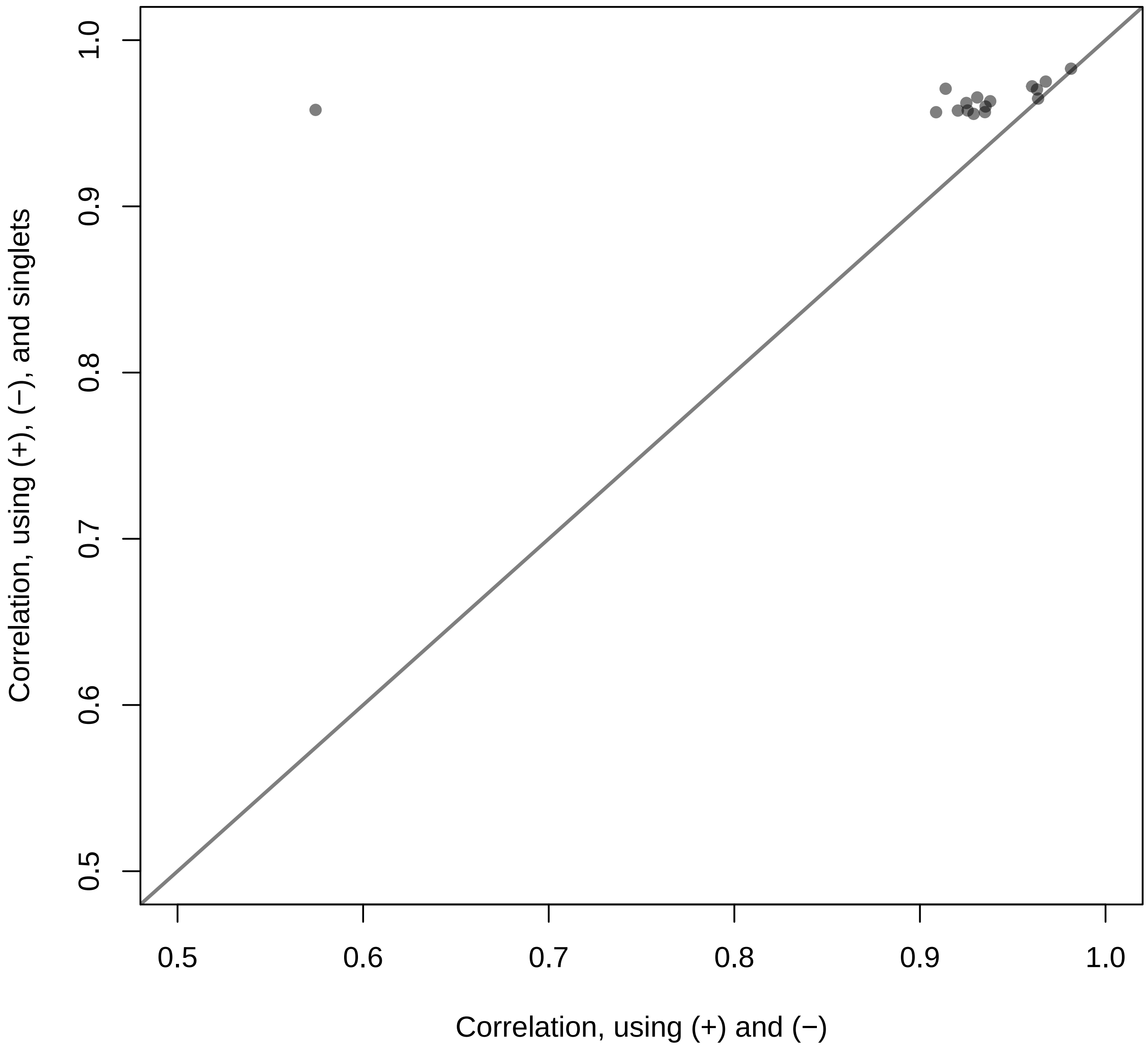

Figure S5: Accuracy predicting the expression in ungated samples using only (+) and (-) samples (x axis), or using the (+), (-), and singlet control samples (y axis).

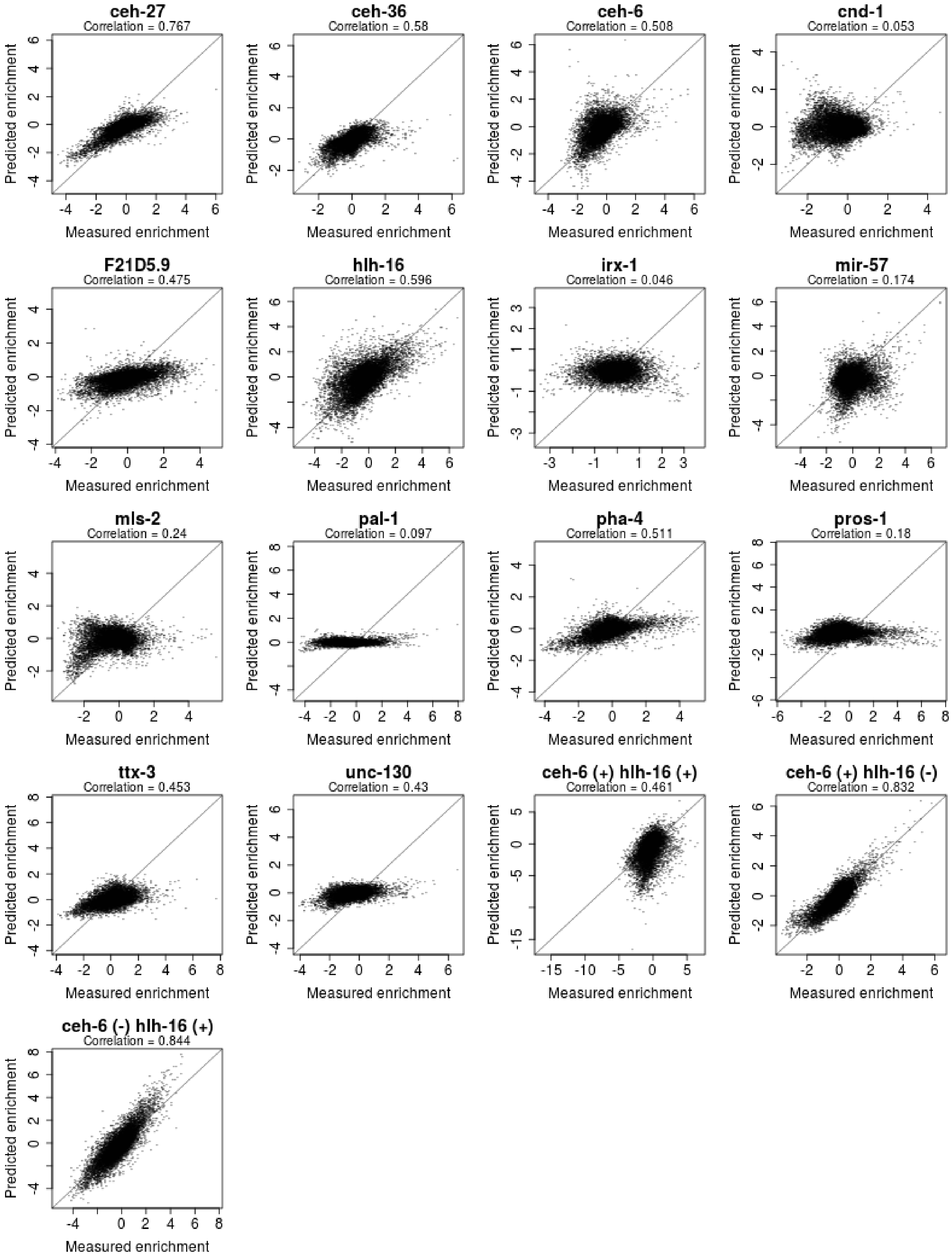

Figure S6: Unmixing cross-validation accuracy. For each sort marker s, the x axis shows measured enrichment computed from the s (+) and s (-) samples. The y axis shows the enrichment predicted for s, based on the measured expression of all samples except s.

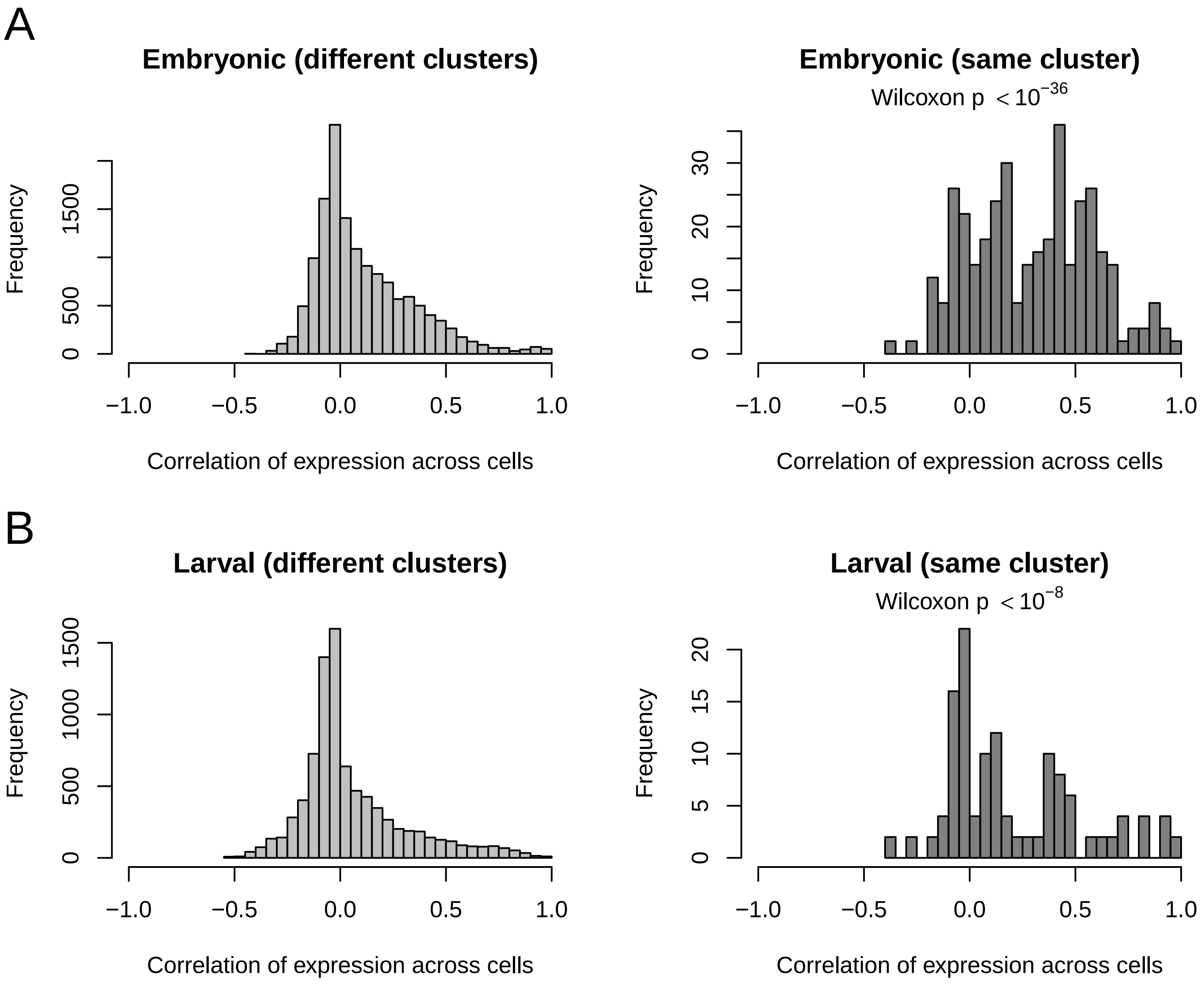

Figure S7: Correlation of expression patterns for genes in different clusters and the same cluster, for (A) 121 embryonic expression patterns from (Murray et al. 2012) and (B) 93 expression patterns from L1 stage larvae (Liu et al. 2009).

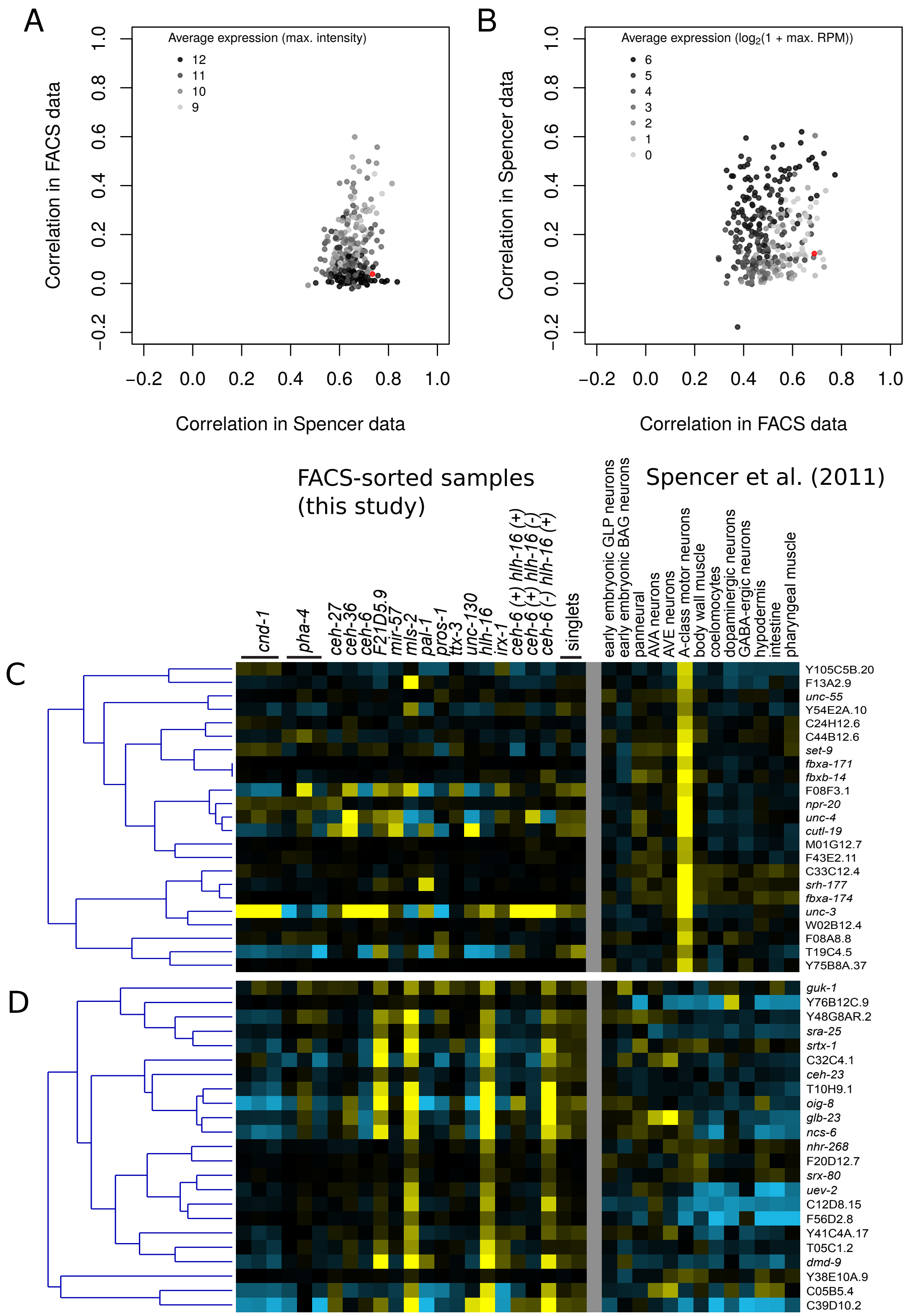

Figure S8: Comparison of clustering of FACS-sorted samples from this study, and Spencer et al. (2011). (A) Average within-cluster correlation of genes in Spencer et al. (2011) data (x axis) and FACS-sorted data from this study (y axis), when genes were clustered using the Spencer et al. (2011) data. (B) Opposite comparison: average within-cluster correlation of genes in FACS-sorted data from this study (x axis) and Spencer et al. (2011) data (y axis), when genes were clustered using the FACS-sorted data. (C) Part of cluster colored red in (A), clustered by Spencer et al. (2011) data. (D) Part of cluster colored red in (B), clustered by FACS-sorted data from this study.

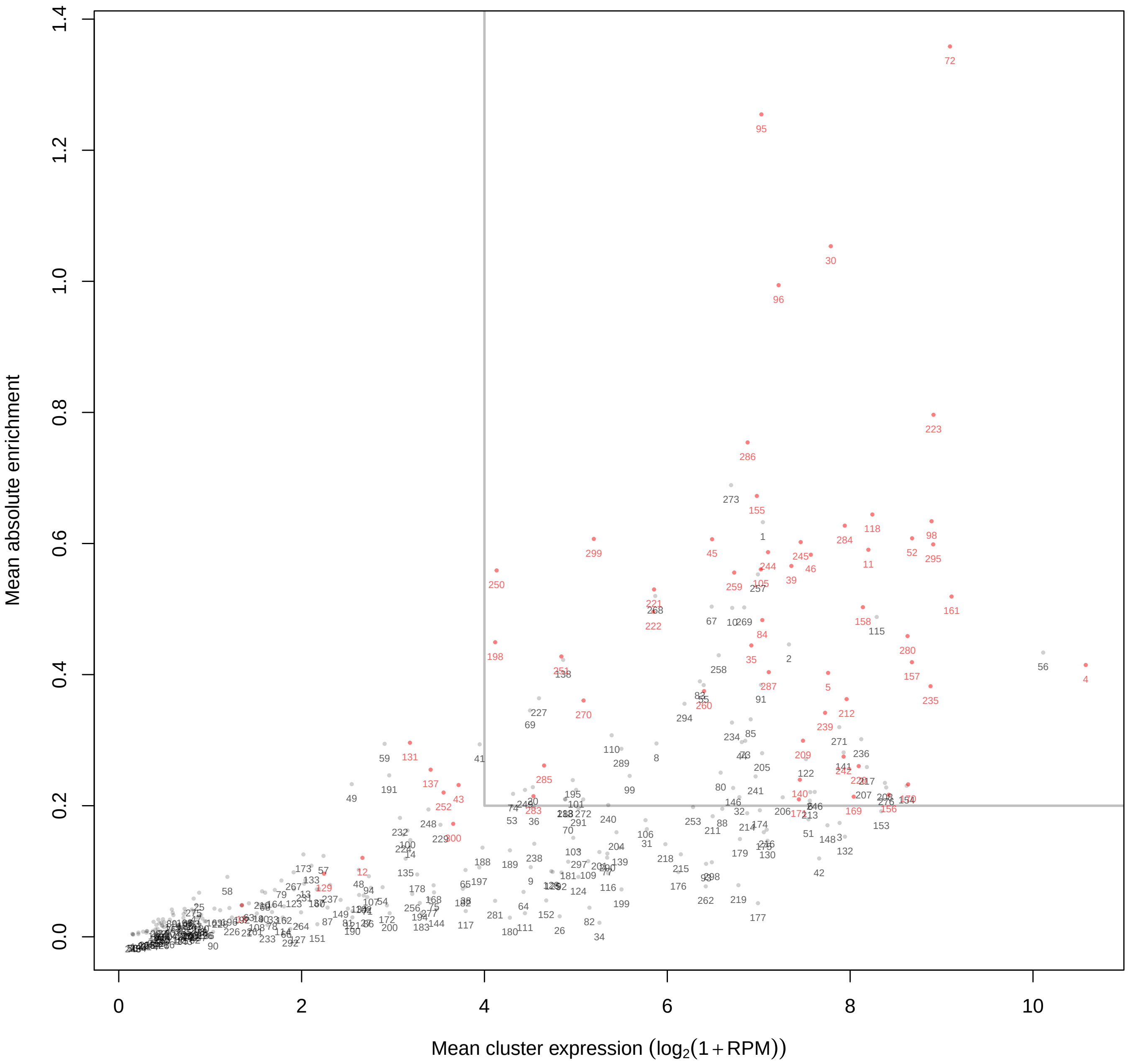

Figure S9: Mean expression and mean absolute enrichments of clusters (as in Figure 4E), with all clusters labelled.

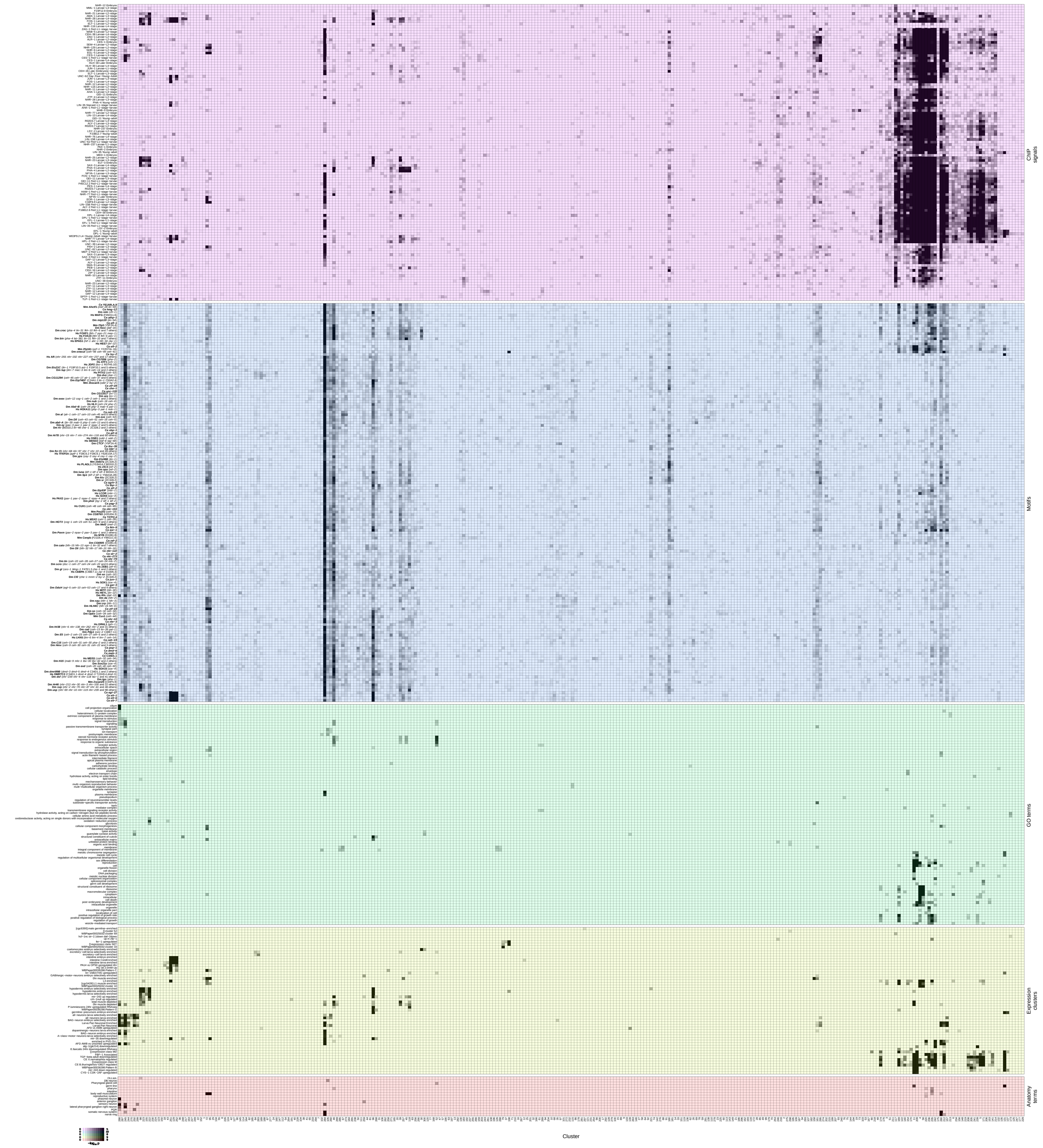

Figure S10: Annotation enrichment as in Figure 5, but for all clusters.

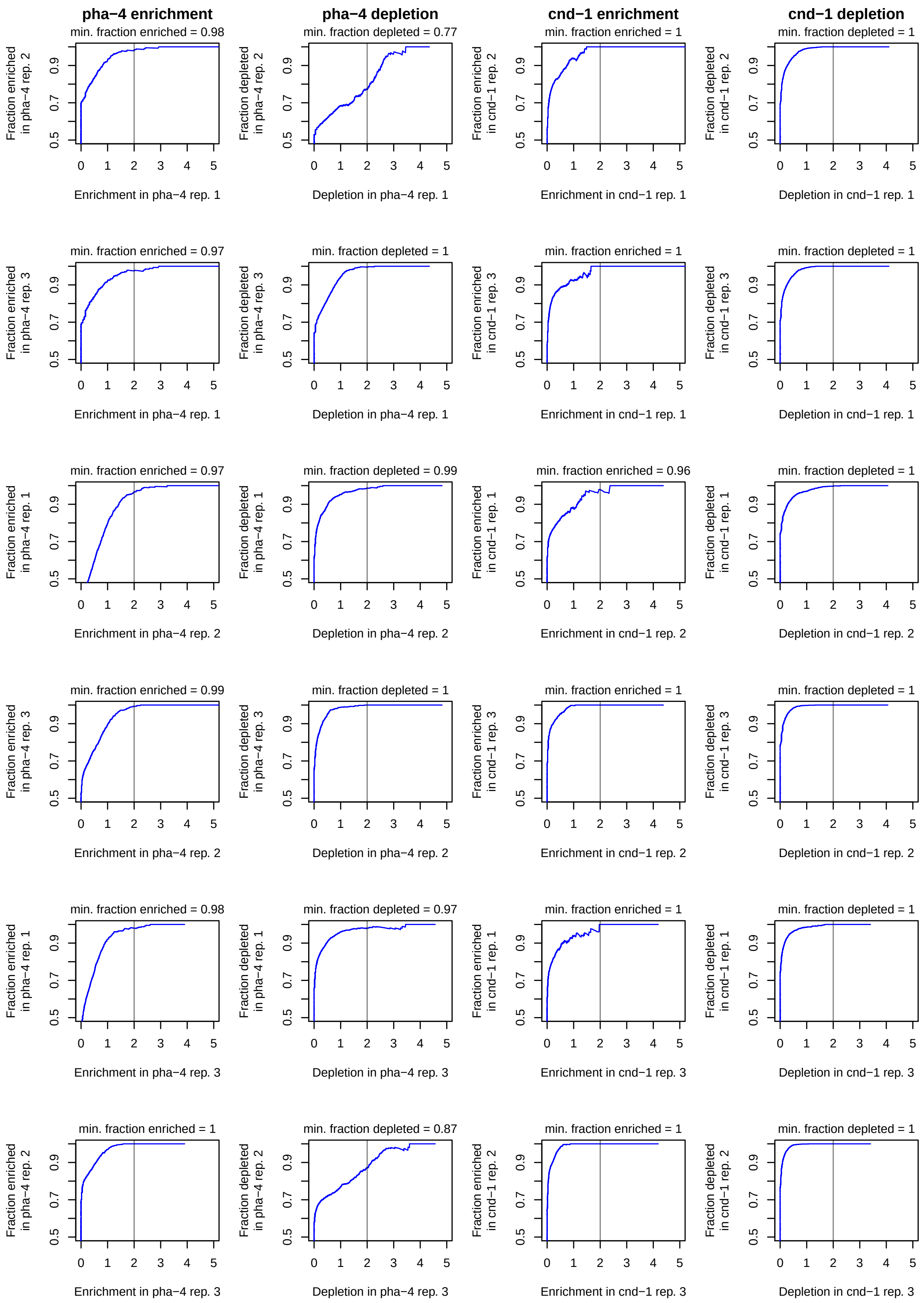

Figure S11: Reproducibility of enrichments, at different cutoffs. For each cutoff on the x axis in one sample, the y axis shows the fraction of genes which were enriched in a replicate experiment.

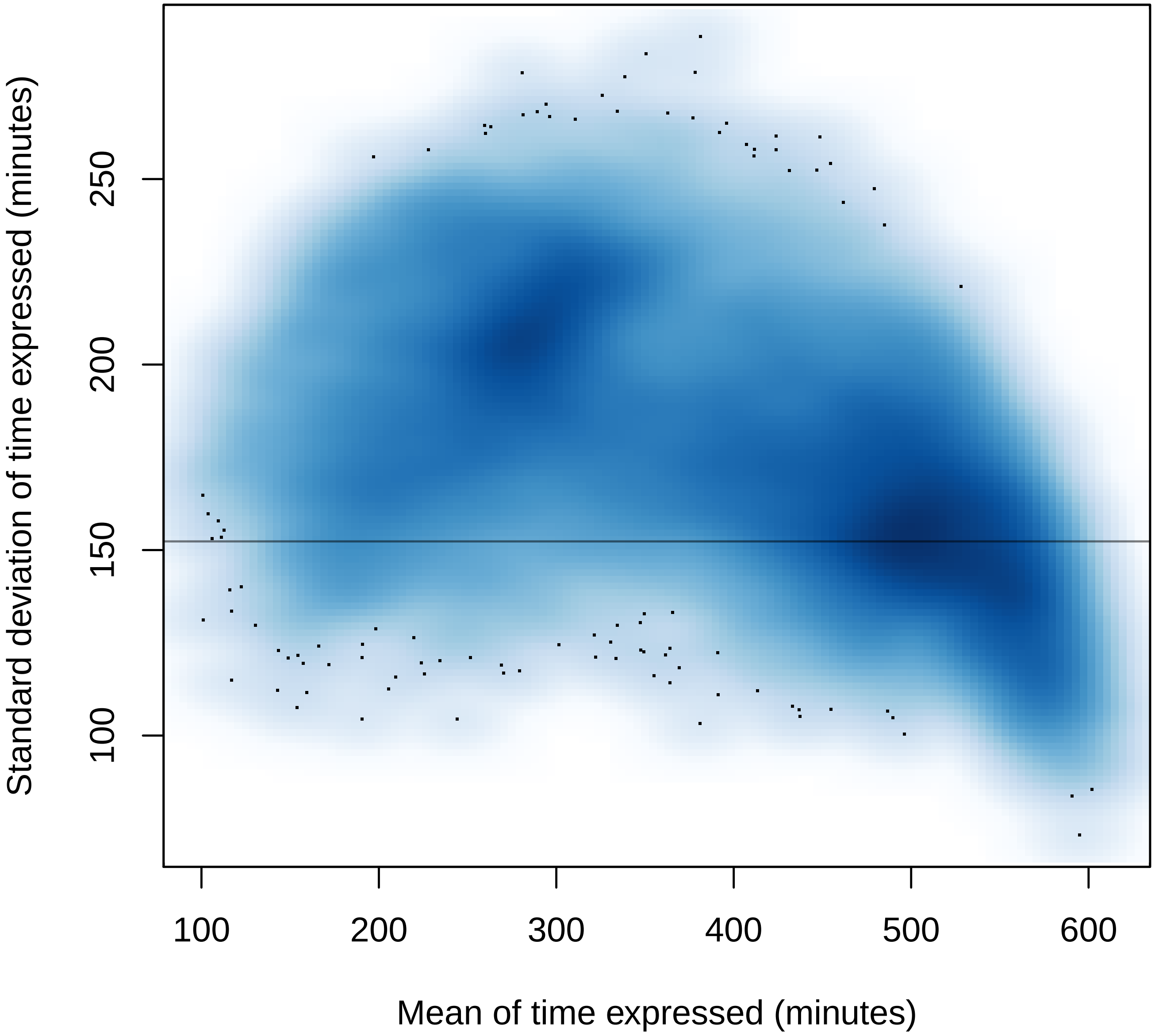

Figure S12: Mean and standard deviation of when genes were expressed, using expression timeseries from (Li et al. 2014). Genes below the horizontal line were considered "time-specific", and used in plotting enrichments relative to time.
