# Supplementary material for: Overlapping cell population expression profiling and regulatory inference in C. elegans
Source: BMC Genomics. 2016 Feb 29;17:159. doi: 10.1186/s12864-016-2482-z (PMC4772325; doi:10.1186/s12864-016-2482-z)
Supplement: Additional file 13: — Web supplement. (DOC 21 kb) [file 12864_2016_2482_MOESM13_ESM.zip › sortWeb/clusters/hier.300.clusters/10.html]

Cluster 10 

## Cluster 10

### Expression

| cnd-1 rep. 1 | cnd-1 rep. 2 | cnd-1 rep. 3 | pha-4 rep. 1 | pha-4 rep. 2 | pha-4 rep. 3 | ceh-27 | ceh-36 | ceh-6 | F21D5.9 | mir-57 | mls-2 | pal-1 | pros-1 | ttx-3 | unc-130 | hlh-16 | irx-1 | ceh-6 (+) hlh-16 (+) | ceh-6 (+) hlh-16 (-) | ceh-6 (-) hlh-16 (+) | cnd-1 singlets | pha-4 singlets | 0 | 60 | 120 | 150 | 180 | 240 | 330 | 390 | 420 | 480 | 540 | 570 | 600 | 630 | 660 | NAME | Functional description |
| --- | --- | --- | --- | --- | --- | --- | --- | --- | --- | --- | --- | --- | --- | --- | --- | --- | --- | --- | --- | --- | --- | --- | --- | --- | --- | --- | --- | --- | --- | --- | --- | --- | --- | --- | --- | --- | --- | --- | --- |
|  |  |  |  |  |  |  |  |  |  |  |  |  |  |  |  |  |  |  |  |  |  |  |  |  |  |  |  |  |  |  |  |  |  |  |  |  |  | *dbl-1* | DPP/BMP-Like |
|  |  |  |  |  |  |  |  |  |  |  |  |  |  |  |  |  |  |  |  |  |  |  |  |  |  |  |  |  |  |  |  |  |  |  |  |  |  | *clec-118* | C-type LECtin |
|  |  |  |  |  |  |  |  |  |  |  |  |  |  |  |  |  |  |  |  |  |  |  |  |  |  |  |  |  |  |  |  |  |  |  |  |  |  | F19B10.13 |  |
|  |  |  |  |  |  |  |  |  |  |  |  |  |  |  |  |  |  |  |  |  |  |  |  |  |  |  |  |  |  |  |  |  |  |  |  |  |  | M199.8 |  |
|  |  |  |  |  |  |  |  |  |  |  |  |  |  |  |  |  |  |  |  |  |  |  |  |  |  |  |  |  |  |  |  |  |  |  |  |  |  | *sri-4* | Serpentine Receptor, class I |
|  |  |  |  |  |  |  |  |  |  |  |  |  |  |  |  |  |  |  |  |  |  |  |  |  |  |  |  |  |  |  |  |  |  |  |  |  |  | R53.2 |  |
|  |  |  |  |  |  |  |  |  |  |  |  |  |  |  |  |  |  |  |  |  |  |  |  |  |  |  |  |  |  |  |  |  |  |  |  |  |  | ZC239.15 |  |
|  |  |  |  |  |  |  |  |  |  |  |  |  |  |  |  |  |  |  |  |  |  |  |  |  |  |  |  |  |  |  |  |  |  |  |  |  |  | K09C4.5 |  |
|  |  |  |  |  |  |  |  |  |  |  |  |  |  |  |  |  |  |  |  |  |  |  |  |  |  |  |  |  |  |  |  |  |  |  |  |  |  | K08F8.5 |  |
|  |  |  |  |  |  |  |  |  |  |  |  |  |  |  |  |  |  |  |  |  |  |  |  |  |  |  |  |  |  |  |  |  |  |  |  |  |  | F13D11.15 |  |
|  |  |  |  |  |  |  |  |  |  |  |  |  |  |  |  |  |  |  |  |  |  |  |  |  |  |  |  |  |  |  |  |  |  |  |  |  |  | Y58A7A.5 |  |
|  |  |  |  |  |  |  |  |  |  |  |  |  |  |  |  |  |  |  |  |  |  |  |  |  |  |  |  |  |  |  |  |  |  |  |  |  |  | C52D10.10 |  |
|  |  |  |  |  |  |  |  |  |  |  |  |  |  |  |  |  |  |  |  |  |  |  |  |  |  |  |  |  |  |  |  |  |  |  |  |  |  | R07B7.4 |  |
|  |  |  |  |  |  |  |  |  |  |  |  |  |  |  |  |  |  |  |  |  |  |  |  |  |  |  |  |  |  |  |  |  |  |  |  |  |  | *ceh-43* | C. Elegans Homeobox |
|  |  |  |  |  |  |  |  |  |  |  |  |  |  |  |  |  |  |  |  |  |  |  |  |  |  |  |  |  |  |  |  |  |  |  |  |  |  | *cutl-29* | CUTiclin-Like |
|  |  |  |  |  |  |  |  |  |  |  |  |  |  |  |  |  |  |  |  |  |  |  |  |  |  |  |  |  |  |  |  |  |  |  |  |  |  | C01B12.8 |  |
|  |  |  |  |  |  |  |  |  |  |  |  |  |  |  |  |  |  |  |  |  |  |  |  |  |  |  |  |  |  |  |  |  |  |  |  |  |  | Y71F9AL.6 |  |
|  |  |  |  |  |  |  |  |  |  |  |  |  |  |  |  |  |  |  |  |  |  |  |  |  |  |  |  |  |  |  |  |  |  |  |  |  |  | *ins-2* | INSulin related |
|  |  |  |  |  |  |  |  |  |  |  |  |  |  |  |  |  |  |  |  |  |  |  |  |  |  |  |  |  |  |  |  |  |  |  |  |  |  | *hmg-6* | HMG |
|  |  |  |  |  |  |  |  |  |  |  |  |  |  |  |  |  |  |  |  |  |  |  |  |  |  |  |  |  |  |  |  |  |  |  |  |  |  | *aqp-6* | AQuaPorin or aquaglyceroporin related |
|  |  |  |  |  |  |  |  |  |  |  |  |  |  |  |  |  |  |  |  |  |  |  |  |  |  |  |  |  |  |  |  |  |  |  |  |  |  | K02A6.1 |  |
|  |  |  |  |  |  |  |  |  |  |  |  |  |  |  |  |  |  |  |  |  |  |  |  |  |  |  |  |  |  |  |  |  |  |  |  |  |  | Y105E8A.34 |  |
|  |  |  |  |  |  |  |  |  |  |  |  |  |  |  |  |  |  |  |  |  |  |  |  |  |  |  |  |  |  |  |  |  |  |  |  |  |  | F19G12.3 |  |
|  |  |  |  |  |  |  |  |  |  |  |  |  |  |  |  |  |  |  |  |  |  |  |  |  |  |  |  |  |  |  |  |  |  |  |  |  |  | F19G12.11 |  |
|  |  |  |  |  |  |  |  |  |  |  |  |  |  |  |  |  |  |  |  |  |  |  |  |  |  |  |  |  |  |  |  |  |  |  |  |  |  | T09B4.6 |  |
|  |  |  |  |  |  |  |  |  |  |  |  |  |  |  |  |  |  |  |  |  |  |  |  |  |  |  |  |  |  |  |  |  |  |  |  |  |  | F53F4.3 |  |
|  |  |  |  |  |  |  |  |  |  |  |  |  |  |  |  |  |  |  |  |  |  |  |  |  |  |  |  |  |  |  |  |  |  |  |  |  |  | C01B10.6 |  |
|  |  |  |  |  |  |  |  |  |  |  |  |  |  |  |  |  |  |  |  |  |  |  |  |  |  |  |  |  |  |  |  |  |  |  |  |  |  | C06G3.5 |  |

### Phenotypes enriched

none found

### Anatomy terms enriched

none found

### GO terms enriched

none found

### Expression clusters enriched

none found

### Motifs enriched

|  |  |  |  |  |  |
| --- | --- | --- | --- | --- | --- |
| **Motif** | **Logo** | **Possible orthologs** | **Number of motifs in cluster** | **Enrichment** | **FDR corrected p** |
| Hlf\_1 |  | ces-2 (0.56) atf-2 | 26 | 1.71 | 0.0018 |
| pTH5074 |  | hlh-15 | 19 | 2.42 | 0.0022 |
| pTH10823 |  | B0310.2 | 14 | 3.06 | 0.0049 |
| pTH6425 |  | ceh-20 (0.54) | 24 | 1.79 | 0.0050 |
| Pbx1\_3203 |  | ceh-20 (0.54) | 22 | 1.94 | 0.0057 |
| CG9895\_SANGER\_10\_FBgn0034810 |  | klf-1 (0.51) | 13 | 3.09 | 0.0086 |
| pTH3086 |  | klf-1 (0.51) | 14 | 2.86 | 0.0091 |
| pTH10037 |  | T22C8.4 | 25 | 1.64 | 0.0092 |
| pTH5250 |  | C48E7.11 | 13 | 3.02 | 0.0100 |
| pTH6071 |  | C33G8.2 | 7 | 5.43 | 0.0190 |
| V$GATA1\_04 |  | elt-1 | 16 | 2.34 | 0.0190 |
| HXA1\_f1 |  | ceh-12 lin-39 | 15 | 2.46 | 0.0200 |
| Nkx6-1\_2825 |  | cog-1 | 12 | 2.97 | 0.0220 |
| Pou3f1\_3819 |  | ceh-6 | 7 | 5.23 | 0.0230 |
| I$KR\_01 |  | B0310.2 | 7 | 5.23 | 0.0230 |
| Irx3\_0920 |  | irx-1 | 5 | 8.28 | 0.0240 |
| pTH9342 |  | ceh-18 (-0.57) | 14 | 2.52 | 0.0270 |
| MA0500.1 |  | hlh-1 hlh-11 | 20 | 1.87 | 0.0290 |
| FOXO4\_1 |  | daf-16 | 6 | 5.96 | 0.0300 |
| pTH9306 |  | lsl-1 | 8 | 4.20 | 0.0300 |
| pTH6003 |  | nhr-134 | 21 | 1.78 | 0.0320 |
| V$YY1\_02 |  | lsy-2 | 11 | 3.01 | 0.0330 |
| V$SP1\_01 |  | klf-2 | 13 | 2.58 | 0.0350 |
| CEBPE\_f1 |  | C48E7.11 | 27 | 1.38 | 0.0370 |
| MA0546.1 |  | lin-31 | 27 | 1.38 | 0.0380 |
| pTH5778 |  | egl-5 | 10 | 3.21 | 0.0380 |
| Mv104 |  | nhr-2 | 27 | 1.38 | 0.0390 |
| pTH6482 |  | ceh-19 | 16 | 2.15 | 0.0400 |
| pTH1014 |  | atf-5 | 23 | 1.62 | 0.0400 |
| NR4A2\_1 |  | nhr-6 | 5 | 7.08 | 0.0410 |
| Sox1\_2631 |  | sox-4 | 16 | 2.14 | 0.0420 |
| V$E47\_01 |  | hlh-2 | 19 | 1.87 | 0.0450 |
| pTH9297 |  | ceh-18 (-0.57) | 7 | 4.48 | 0.0450 |
| COT1\_si |  | nhr-2 | 27 | 1.37 | 0.0450 |
| Foxj3\_1 |  | lin-31 | 23 | 1.60 | 0.0460 |
| V$SRF\_Q6 |  | unc-120 | 6 | 5.33 | 0.0470 |
| TBX2\_1 |  | tbx-39 | 9 | 3.40 | 0.0480 |

### Correlated (and anti-correlated) transcription factors

|  |  |
| --- | --- |
| **Transcription factor** | **Correlation** |
| hmg-6 | 0.88 |
| ceh-43 | 0.82 |
| sptf-1 | 0.79 |
| hlh-3 | 0.75 |
| cey-4 | 0.73 |
| hlh-16 | 0.71 |
| unc-39 | 0.70 |
| lin-32 | 0.70 |
| unc-30 | 0.69 |
| lin-11 | 0.68 |
| F26F4.8 | 0.67 |
| zim-1 | 0.66 |
| flh-3 | 0.66 |
| dhhc-6 | 0.65 |
| cebp-2 | 0.65 |
| cnd-1 | 0.65 |
| ceh-10 | 0.64 |
| hmg-5 | 0.64 |
| C16A3.4 | 0.63 |
| hmg-11 | 0.63 |
| dro-1 | 0.63 |
| sea-2 | 0.62 |
| ekl-4 | 0.61 |
| Y53F4B.3 | 0.61 |
| ztf-11 | 0.61 |
| nhr-275 | -0.47 |
| srt-58 | -0.47 |
| nhr-169 | -0.48 |
| mgl-2 | -0.48 |
| xbp-1 | -0.49 |
| nhr-286 | -0.49 |
| nhr-184 | -0.49 |
| nhr-5 | -0.50 |
| athp-3 | -0.51 |
| hlh-6 | -0.51 |
| lsy-27 | -0.51 |
| nhr-116 | -0.51 |
| nhr-135 | -0.51 |
| nhr-58 | -0.52 |
| F26A10.2 | -0.55 |
| ceh-18 | -0.57 |
| madf-4 | -0.57 |
| nhr-3 | -0.57 |
| fos-1 | -0.58 |
| nhr-71 | -0.58 |
| zfh-2 | -0.59 |
| zip-2 | -0.62 |
| mdl-1 | -0.64 |
| nhr-41 | -0.68 |
| zip-1 | -0.76 |

### ChIP peaks enriched

none found
