# Supplementary material for: Overlapping cell population expression profiling and regulatory inference in C. elegans
Source: BMC Genomics. 2016 Feb 29;17:159. doi: 10.1186/s12864-016-2482-z (PMC4772325; doi:10.1186/s12864-016-2482-z)
Supplement: Additional file 13: — Web supplement. (DOC 21 kb) [file 12864_2016_2482_MOESM13_ESM.zip › sortWeb/clusters/hier.300.clusters/101.html]

Cluster 101 

## Cluster 101

### Expression

| cnd-1 rep. 1 | cnd-1 rep. 2 | cnd-1 rep. 3 | pha-4 rep. 1 | pha-4 rep. 2 | pha-4 rep. 3 | ceh-27 | ceh-36 | ceh-6 | F21D5.9 | mir-57 | mls-2 | pal-1 | pros-1 | ttx-3 | unc-130 | hlh-16 | irx-1 | ceh-6 (+) hlh-16 (+) | ceh-6 (+) hlh-16 (-) | ceh-6 (-) hlh-16 (+) | cnd-1 singlets | pha-4 singlets | 0 | 60 | 120 | 150 | 180 | 240 | 330 | 390 | 420 | 480 | 540 | 570 | 600 | 630 | 660 | NAME | Functional description |
| --- | --- | --- | --- | --- | --- | --- | --- | --- | --- | --- | --- | --- | --- | --- | --- | --- | --- | --- | --- | --- | --- | --- | --- | --- | --- | --- | --- | --- | --- | --- | --- | --- | --- | --- | --- | --- | --- | --- | --- |
|  |  |  |  |  |  |  |  |  |  |  |  |  |  |  |  |  |  |  |  |  |  |  |  |  |  |  |  |  |  |  |  |  |  |  |  |  |  | *lron-8* | eLRR (extracellular Leucine-Rich Repeat) ONly |
|  |  |  |  |  |  |  |  |  |  |  |  |  |  |  |  |  |  |  |  |  |  |  |  |  |  |  |  |  |  |  |  |  |  |  |  |  |  | C36A4.11 |  |
|  |  |  |  |  |  |  |  |  |  |  |  |  |  |  |  |  |  |  |  |  |  |  |  |  |  |  |  |  |  |  |  |  |  |  |  |  |  | C05C10.3 |  |
|  |  |  |  |  |  |  |  |  |  |  |  |  |  |  |  |  |  |  |  |  |  |  |  |  |  |  |  |  |  |  |  |  |  |  |  |  |  | *ttr-29* | TransThyretin-Related family domain |
|  |  |  |  |  |  |  |  |  |  |  |  |  |  |  |  |  |  |  |  |  |  |  |  |  |  |  |  |  |  |  |  |  |  |  |  |  |  | *sre-44* | Serpentine Receptor, class E (epsilon) |
|  |  |  |  |  |  |  |  |  |  |  |  |  |  |  |  |  |  |  |  |  |  |  |  |  |  |  |  |  |  |  |  |  |  |  |  |  |  | *twk-5* | TWiK family of potassium channels |
|  |  |  |  |  |  |  |  |  |  |  |  |  |  |  |  |  |  |  |  |  |  |  |  |  |  |  |  |  |  |  |  |  |  |  |  |  |  | F54D5.17 |  |
|  |  |  |  |  |  |  |  |  |  |  |  |  |  |  |  |  |  |  |  |  |  |  |  |  |  |  |  |  |  |  |  |  |  |  |  |  |  | F27C1.3 |  |
|  |  |  |  |  |  |  |  |  |  |  |  |  |  |  |  |  |  |  |  |  |  |  |  |  |  |  |  |  |  |  |  |  |  |  |  |  |  | Y41G9A.14 |  |
|  |  |  |  |  |  |  |  |  |  |  |  |  |  |  |  |  |  |  |  |  |  |  |  |  |  |  |  |  |  |  |  |  |  |  |  |  |  | Y51H7C.9 |  |
|  |  |  |  |  |  |  |  |  |  |  |  |  |  |  |  |  |  |  |  |  |  |  |  |  |  |  |  |  |  |  |  |  |  |  |  |  |  | *clec-182* | C-type LECtin |
|  |  |  |  |  |  |  |  |  |  |  |  |  |  |  |  |  |  |  |  |  |  |  |  |  |  |  |  |  |  |  |  |  |  |  |  |  |  | *sre-43* | Serpentine Receptor, class E (epsilon) |
|  |  |  |  |  |  |  |  |  |  |  |  |  |  |  |  |  |  |  |  |  |  |  |  |  |  |  |  |  |  |  |  |  |  |  |  |  |  | *srbc-34* | Serpentine Receptor, class BC (class B-like) |
|  |  |  |  |  |  |  |  |  |  |  |  |  |  |  |  |  |  |  |  |  |  |  |  |  |  |  |  |  |  |  |  |  |  |  |  |  |  | *bicd-1* | BICaudal D (Drosophila) homolog |
|  |  |  |  |  |  |  |  |  |  |  |  |  |  |  |  |  |  |  |  |  |  |  |  |  |  |  |  |  |  |  |  |  |  |  |  |  |  | *mpst-2* | MercaptoPyruvate SulfurTransferase homolog |
|  |  |  |  |  |  |  |  |  |  |  |  |  |  |  |  |  |  |  |  |  |  |  |  |  |  |  |  |  |  |  |  |  |  |  |  |  |  | K11G9.1 |  |
|  |  |  |  |  |  |  |  |  |  |  |  |  |  |  |  |  |  |  |  |  |  |  |  |  |  |  |  |  |  |  |  |  |  |  |  |  |  | *nhx-4* | Na/H eXchanger |
|  |  |  |  |  |  |  |  |  |  |  |  |  |  |  |  |  |  |  |  |  |  |  |  |  |  |  |  |  |  |  |  |  |  |  |  |  |  | F52D10.2 |  |
|  |  |  |  |  |  |  |  |  |  |  |  |  |  |  |  |  |  |  |  |  |  |  |  |  |  |  |  |  |  |  |  |  |  |  |  |  |  | *gon-2* | abnormal GONad development |
|  |  |  |  |  |  |  |  |  |  |  |  |  |  |  |  |  |  |  |  |  |  |  |  |  |  |  |  |  |  |  |  |  |  |  |  |  |  | *gld-2* | defective in Germ Line Development |
|  |  |  |  |  |  |  |  |  |  |  |  |  |  |  |  |  |  |  |  |  |  |  |  |  |  |  |  |  |  |  |  |  |  |  |  |  |  | *alfa-1* | ALS/FTD Associated gene homolog |
|  |  |  |  |  |  |  |  |  |  |  |  |  |  |  |  |  |  |  |  |  |  |  |  |  |  |  |  |  |  |  |  |  |  |  |  |  |  | T27B1.7 |  |
|  |  |  |  |  |  |  |  |  |  |  |  |  |  |  |  |  |  |  |  |  |  |  |  |  |  |  |  |  |  |  |  |  |  |  |  |  |  | F40F9.11 |  |
|  |  |  |  |  |  |  |  |  |  |  |  |  |  |  |  |  |  |  |  |  |  |  |  |  |  |  |  |  |  |  |  |  |  |  |  |  |  | Y60A3A.21 |  |
|  |  |  |  |  |  |  |  |  |  |  |  |  |  |  |  |  |  |  |  |  |  |  |  |  |  |  |  |  |  |  |  |  |  |  |  |  |  | Y38C1AA.7 |  |
|  |  |  |  |  |  |  |  |  |  |  |  |  |  |  |  |  |  |  |  |  |  |  |  |  |  |  |  |  |  |  |  |  |  |  |  |  |  | F25D7.9 |  |
|  |  |  |  |  |  |  |  |  |  |  |  |  |  |  |  |  |  |  |  |  |  |  |  |  |  |  |  |  |  |  |  |  |  |  |  |  |  | T16G12.10 |  |
|  |  |  |  |  |  |  |  |  |  |  |  |  |  |  |  |  |  |  |  |  |  |  |  |  |  |  |  |  |  |  |  |  |  |  |  |  |  | *acdh-8* | Acyl CoA DeHydrogenase |
|  |  |  |  |  |  |  |  |  |  |  |  |  |  |  |  |  |  |  |  |  |  |  |  |  |  |  |  |  |  |  |  |  |  |  |  |  |  | C43C3.7 |  |
|  |  |  |  |  |  |  |  |  |  |  |  |  |  |  |  |  |  |  |  |  |  |  |  |  |  |  |  |  |  |  |  |  |  |  |  |  |  | *linc-141* | Long Intervening Non-Coding RNA |
|  |  |  |  |  |  |  |  |  |  |  |  |  |  |  |  |  |  |  |  |  |  |  |  |  |  |  |  |  |  |  |  |  |  |  |  |  |  | D1022.9 |  |
|  |  |  |  |  |  |  |  |  |  |  |  |  |  |  |  |  |  |  |  |  |  |  |  |  |  |  |  |  |  |  |  |  |  |  |  |  |  | *dmd-7* | DM (Doublesex/MAB-3) Domain family |
|  |  |  |  |  |  |  |  |  |  |  |  |  |  |  |  |  |  |  |  |  |  |  |  |  |  |  |  |  |  |  |  |  |  |  |  |  |  | H12D21.10 |  |
|  |  |  |  |  |  |  |  |  |  |  |  |  |  |  |  |  |  |  |  |  |  |  |  |  |  |  |  |  |  |  |  |  |  |  |  |  |  | F18F11.5 |  |
|  |  |  |  |  |  |  |  |  |  |  |  |  |  |  |  |  |  |  |  |  |  |  |  |  |  |  |  |  |  |  |  |  |  |  |  |  |  | K02B2.36 |  |
|  |  |  |  |  |  |  |  |  |  |  |  |  |  |  |  |  |  |  |  |  |  |  |  |  |  |  |  |  |  |  |  |  |  |  |  |  |  | *clec-114* | C-type LECtin |
|  |  |  |  |  |  |  |  |  |  |  |  |  |  |  |  |  |  |  |  |  |  |  |  |  |  |  |  |  |  |  |  |  |  |  |  |  |  | F09A5.3 |  |

### Phenotypes enriched

none found

### Anatomy terms enriched

none found

### GO terms enriched

none found

### Expression clusters enriched

none found

### Motifs enriched

|  |  |  |  |  |  |
| --- | --- | --- | --- | --- | --- |
| **Motif** | **Logo** | **Possible orthologs** | **Number of motifs in cluster** | **Enrichment** | **FDR corrected p** |
| V$E47\_01 |  | hlh-1 (0.6) hlh-2 ces-1 hlh-15 | 11 | 4.36 | 0.0034 |
| V$XFD2\_01 |  | lin-31 (0.6) | 18 | 2.58 | 0.0050 |
| MYF6\_f1 |  | hlh-1 (0.6) hlh-15 | 21 | 2.24 | 0.0058 |
| pTH9278 |  | daf-19 | 14 | 3.15 | 0.0063 |
| twi\_da\_SANGER\_5\_FBgn0003900 |  | hlh-8 (0.64) hlh-12 | 16 | 2.76 | 0.0072 |
| V$NKX25\_02 |  | ceh-24 lim-7 | 10 | 4.25 | 0.0085 |
| Ascl2\_1 |  | hlh-1 (0.6) hlh-14 lin-32 | 14 | 3.03 | 0.0088 |
| MA0040.1 |  | let-381 | 12 | 3.44 | 0.0100 |
| CART1\_1 |  | alr-1 | 27 | 1.74 | 0.0130 |
| Cdx2\_4272 |  | ceh-13 | 17 | 2.46 | 0.0130 |
| Nkx1-2\_3214 |  | ceh-30 | 22 | 2.00 | 0.0150 |
| svp\_SANGER\_5\_FBgn0003651 |  | nhr-2 | 19 | 2.17 | 0.0200 |
| Rax\_3443 |  | alr-1 | 12 | 3.11 | 0.0210 |
| HOXA1\_1 |  | ceh-12 | 19 | 2.16 | 0.0210 |
| V$MEIS1\_01 |  | ceh-32 | 14 | 2.71 | 0.0220 |
| CrebA\_SANGER\_5\_FBgn0004396 |  | C27D6.4 | 9 | 4.03 | 0.0230 |
| Mw142 |  | ceh-34 elt-6 egl-27 elt-1 | 17 | 2.31 | 0.0250 |
| TFE3\_f1 |  | hlh-30 (0.63) mdl-1 | 12 | 2.96 | 0.0290 |
| MA0476.1 |  | fos-1 | 8 | 4.25 | 0.0330 |
| Cdx1\_2245 |  | ceh-13 | 19 | 2.06 | 0.0340 |
| pTH6447 |  | ceh-19 | 29 | 1.55 | 0.0360 |
| ECC-1\_ERALPHA\_HudsonAlpha |  | nhr-71 | 17 | 2.21 | 0.0360 |
| ELF3\_2 |  | C24A1.2 | 17 | 2.21 | 0.0370 |
| Gbx2\_3110 |  | ceh-12 alr-1 | 23 | 1.80 | 0.0370 |
| Hoxd10\_2368 |  | php-3 (0.62) | 26 | 1.66 | 0.0380 |
| pTH6486 |  | nhr-145 | 6 | 5.50 | 0.0440 |
| Barhl1\_2 |  | ceh-31 | 17 | 2.16 | 0.0440 |
| pTH10028 |  | nhr-204 (0.53) | 6 | 5.38 | 0.0480 |
| pTH10040 |  | slr-2 | 28 | 1.55 | 0.0480 |
| pTH9073 |  | end-3 | 24 | 1.71 | 0.0490 |
| I$MTTFA\_01 |  | hmg-5 | 20 | 1.92 | 0.0490 |
| Nkx1-1\_3856 |  | ceh-30 | 17 | 2.13 | 0.0500 |

### Correlated (and anti-correlated) transcription factors

|  |  |
| --- | --- |
| **Transcription factor** | **Correlation** |
| ref-1 | 0.86 |
| unc-120 | 0.83 |
| nhr-34 | 0.82 |
| C30G4.7 | 0.81 |
| sbp-1 | 0.76 |
| nhr-69 | 0.76 |
| klf-2 | 0.75 |
| nhr-111 | 0.75 |
| nhr-9 | 0.72 |
| mls-1 | 0.71 |
| pat-9 | 0.70 |
| nhr-35 | 0.70 |
| odd-2 | 0.69 |
| dmd-3 | 0.69 |
| nhr-256 | 0.68 |
| mab-3 | 0.68 |
| nhr-98 | 0.68 |
| sma-3 | 0.67 |
| nhr-97 | 0.67 |
| nhr-19 | 0.67 |
| ehn-3 | 0.66 |
| atf-5 | 0.66 |
| ceh-99 | 0.65 |
| hlh-11 | 0.65 |
| elt-3 | 0.65 |
| T26A8.4 | -0.48 |
| him-8 | -0.48 |
| Y53H1A.2 | -0.48 |
| nhr-197 | -0.48 |
| madf-2 | -0.49 |
| nhr-268 | -0.49 |
| vab-3 | -0.50 |
| ceh-89 | -0.50 |
| nhr-198 | -0.50 |
| ceh-32 | -0.50 |
| fkh-2 | -0.51 |
| ceh-54 | -0.53 |
| madf-10 | -0.54 |
| ztf-4 | -0.54 |
| ccch-3 | -0.54 |
| lir-3 | -0.55 |
| sox-2 | -0.55 |
| sox-3 | -0.56 |
| npax-1 | -0.57 |
| zip-4 | -0.62 |
| ast-1 | -0.62 |
| lim-4 | -0.65 |
| unc-42 | -0.65 |
| nhr-215 | -0.65 |
| F10E7.11 | -0.66 |

### ChIP peaks enriched

none found
