# Supplementary material for: Overlapping cell population expression profiling and regulatory inference in C. elegans
Source: BMC Genomics. 2016 Feb 29;17:159. doi: 10.1186/s12864-016-2482-z (PMC4772325; doi:10.1186/s12864-016-2482-z)
Supplement: Additional file 13: — Web supplement. (DOC 21 kb) [file 12864_2016_2482_MOESM13_ESM.zip › sortWeb/clusters/hier.300.clusters/105.html]

Cluster 105 

## Cluster 105

### Expression

| cnd-1 rep. 1 | cnd-1 rep. 2 | cnd-1 rep. 3 | pha-4 rep. 1 | pha-4 rep. 2 | pha-4 rep. 3 | ceh-27 | ceh-36 | ceh-6 | F21D5.9 | mir-57 | mls-2 | pal-1 | pros-1 | ttx-3 | unc-130 | hlh-16 | irx-1 | ceh-6 (+) hlh-16 (+) | ceh-6 (+) hlh-16 (-) | ceh-6 (-) hlh-16 (+) | cnd-1 singlets | pha-4 singlets | 0 | 60 | 120 | 150 | 180 | 240 | 330 | 390 | 420 | 480 | 540 | 570 | 600 | 630 | 660 | NAME | Functional description |
| --- | --- | --- | --- | --- | --- | --- | --- | --- | --- | --- | --- | --- | --- | --- | --- | --- | --- | --- | --- | --- | --- | --- | --- | --- | --- | --- | --- | --- | --- | --- | --- | --- | --- | --- | --- | --- | --- | --- | --- |
|  |  |  |  |  |  |  |  |  |  |  |  |  |  |  |  |  |  |  |  |  |  |  |  |  |  |  |  |  |  |  |  |  |  |  |  |  |  | T25C8.1 |  |
|  |  |  |  |  |  |  |  |  |  |  |  |  |  |  |  |  |  |  |  |  |  |  |  |  |  |  |  |  |  |  |  |  |  |  |  |  |  | *cpg-8* | Chondroitin ProteoGlycan |
|  |  |  |  |  |  |  |  |  |  |  |  |  |  |  |  |  |  |  |  |  |  |  |  |  |  |  |  |  |  |  |  |  |  |  |  |  |  | *clec-219* | C-type LECtin |
|  |  |  |  |  |  |  |  |  |  |  |  |  |  |  |  |  |  |  |  |  |  |  |  |  |  |  |  |  |  |  |  |  |  |  |  |  |  | T23B12.5 |  |
|  |  |  |  |  |  |  |  |  |  |  |  |  |  |  |  |  |  |  |  |  |  |  |  |  |  |  |  |  |  |  |  |  |  |  |  |  |  | R07E3.7 |  |
|  |  |  |  |  |  |  |  |  |  |  |  |  |  |  |  |  |  |  |  |  |  |  |  |  |  |  |  |  |  |  |  |  |  |  |  |  |  | ZC84.7 |  |
|  |  |  |  |  |  |  |  |  |  |  |  |  |  |  |  |  |  |  |  |  |  |  |  |  |  |  |  |  |  |  |  |  |  |  |  |  |  | F36H1.3 |  |
|  |  |  |  |  |  |  |  |  |  |  |  |  |  |  |  |  |  |  |  |  |  |  |  |  |  |  |  |  |  |  |  |  |  |  |  |  |  | F54D12.9 |  |
|  |  |  |  |  |  |  |  |  |  |  |  |  |  |  |  |  |  |  |  |  |  |  |  |  |  |  |  |  |  |  |  |  |  |  |  |  |  | K09C8.8 |  |
|  |  |  |  |  |  |  |  |  |  |  |  |  |  |  |  |  |  |  |  |  |  |  |  |  |  |  |  |  |  |  |  |  |  |  |  |  |  | K04D7.11 |  |
|  |  |  |  |  |  |  |  |  |  |  |  |  |  |  |  |  |  |  |  |  |  |  |  |  |  |  |  |  |  |  |  |  |  |  |  |  |  | F27D9.2 |  |
|  |  |  |  |  |  |  |  |  |  |  |  |  |  |  |  |  |  |  |  |  |  |  |  |  |  |  |  |  |  |  |  |  |  |  |  |  |  | T20B12.5 |  |
|  |  |  |  |  |  |  |  |  |  |  |  |  |  |  |  |  |  |  |  |  |  |  |  |  |  |  |  |  |  |  |  |  |  |  |  |  |  | *flp-13* | FMRF-Like Peptide |
|  |  |  |  |  |  |  |  |  |  |  |  |  |  |  |  |  |  |  |  |  |  |  |  |  |  |  |  |  |  |  |  |  |  |  |  |  |  | *sra-10* | Serpentine Receptor, class A (alpha) |
|  |  |  |  |  |  |  |  |  |  |  |  |  |  |  |  |  |  |  |  |  |  |  |  |  |  |  |  |  |  |  |  |  |  |  |  |  |  | *srv-4* | Serpentine Receptor, class V |
|  |  |  |  |  |  |  |  |  |  |  |  |  |  |  |  |  |  |  |  |  |  |  |  |  |  |  |  |  |  |  |  |  |  |  |  |  |  | F15E6.5 |  |
|  |  |  |  |  |  |  |  |  |  |  |  |  |  |  |  |  |  |  |  |  |  |  |  |  |  |  |  |  |  |  |  |  |  |  |  |  |  | *tre-3* | TREhalase |
|  |  |  |  |  |  |  |  |  |  |  |  |  |  |  |  |  |  |  |  |  |  |  |  |  |  |  |  |  |  |  |  |  |  |  |  |  |  | B0416.3 |  |
|  |  |  |  |  |  |  |  |  |  |  |  |  |  |  |  |  |  |  |  |  |  |  |  |  |  |  |  |  |  |  |  |  |  |  |  |  |  | F11C1.10 |  |
|  |  |  |  |  |  |  |  |  |  |  |  |  |  |  |  |  |  |  |  |  |  |  |  |  |  |  |  |  |  |  |  |  |  |  |  |  |  | *hlh-13* | Helix Loop Helix |
|  |  |  |  |  |  |  |  |  |  |  |  |  |  |  |  |  |  |  |  |  |  |  |  |  |  |  |  |  |  |  |  |  |  |  |  |  |  | *hsd-2* | HydroxySteroid Dehydrogenase homolog |
|  |  |  |  |  |  |  |  |  |  |  |  |  |  |  |  |  |  |  |  |  |  |  |  |  |  |  |  |  |  |  |  |  |  |  |  |  |  | *clec-90* | C-type LECtin |
|  |  |  |  |  |  |  |  |  |  |  |  |  |  |  |  |  |  |  |  |  |  |  |  |  |  |  |  |  |  |  |  |  |  |  |  |  |  | K01A12.2 |  |
|  |  |  |  |  |  |  |  |  |  |  |  |  |  |  |  |  |  |  |  |  |  |  |  |  |  |  |  |  |  |  |  |  |  |  |  |  |  | *acr-21* | AcetylCholine Receptor |
|  |  |  |  |  |  |  |  |  |  |  |  |  |  |  |  |  |  |  |  |  |  |  |  |  |  |  |  |  |  |  |  |  |  |  |  |  |  | *col-149* | COLlagen |
|  |  |  |  |  |  |  |  |  |  |  |  |  |  |  |  |  |  |  |  |  |  |  |  |  |  |  |  |  |  |  |  |  |  |  |  |  |  | *rga-6* | Rho GTPase Activating protein |
|  |  |  |  |  |  |  |  |  |  |  |  |  |  |  |  |  |  |  |  |  |  |  |  |  |  |  |  |  |  |  |  |  |  |  |  |  |  | F32A5.2 |  |
|  |  |  |  |  |  |  |  |  |  |  |  |  |  |  |  |  |  |  |  |  |  |  |  |  |  |  |  |  |  |  |  |  |  |  |  |  |  | F18E9.4 |  |
|  |  |  |  |  |  |  |  |  |  |  |  |  |  |  |  |  |  |  |  |  |  |  |  |  |  |  |  |  |  |  |  |  |  |  |  |  |  | F32D8.8 |  |
|  |  |  |  |  |  |  |  |  |  |  |  |  |  |  |  |  |  |  |  |  |  |  |  |  |  |  |  |  |  |  |  |  |  |  |  |  |  | *col-131* | COLlagen |
|  |  |  |  |  |  |  |  |  |  |  |  |  |  |  |  |  |  |  |  |  |  |  |  |  |  |  |  |  |  |  |  |  |  |  |  |  |  | *col-172* | COLlagen |
|  |  |  |  |  |  |  |  |  |  |  |  |  |  |  |  |  |  |  |  |  |  |  |  |  |  |  |  |  |  |  |  |  |  |  |  |  |  | *irk-1* | Inward Rectifying K (potassium) channel family |
|  |  |  |  |  |  |  |  |  |  |  |  |  |  |  |  |  |  |  |  |  |  |  |  |  |  |  |  |  |  |  |  |  |  |  |  |  |  | *spe-47* | defective SPErmatogenesis |
|  |  |  |  |  |  |  |  |  |  |  |  |  |  |  |  |  |  |  |  |  |  |  |  |  |  |  |  |  |  |  |  |  |  |  |  |  |  | *cho-1* | CHOline transporter |
|  |  |  |  |  |  |  |  |  |  |  |  |  |  |  |  |  |  |  |  |  |  |  |  |  |  |  |  |  |  |  |  |  |  |  |  |  |  | *glr-4* | GLutamate Receptor family (AMPA) |
|  |  |  |  |  |  |  |  |  |  |  |  |  |  |  |  |  |  |  |  |  |  |  |  |  |  |  |  |  |  |  |  |  |  |  |  |  |  | C42D4.1 |  |
|  |  |  |  |  |  |  |  |  |  |  |  |  |  |  |  |  |  |  |  |  |  |  |  |  |  |  |  |  |  |  |  |  |  |  |  |  |  | *lgc-4* | Ligand-Gated ion Channel |
|  |  |  |  |  |  |  |  |  |  |  |  |  |  |  |  |  |  |  |  |  |  |  |  |  |  |  |  |  |  |  |  |  |  |  |  |  |  | T11F9.1 |  |
|  |  |  |  |  |  |  |  |  |  |  |  |  |  |  |  |  |  |  |  |  |  |  |  |  |  |  |  |  |  |  |  |  |  |  |  |  |  | F55C12.4 |  |
|  |  |  |  |  |  |  |  |  |  |  |  |  |  |  |  |  |  |  |  |  |  |  |  |  |  |  |  |  |  |  |  |  |  |  |  |  |  | *lgc-52* | Ligand-Gated ion Channel |
|  |  |  |  |  |  |  |  |  |  |  |  |  |  |  |  |  |  |  |  |  |  |  |  |  |  |  |  |  |  |  |  |  |  |  |  |  |  | T11F9.27 |  |
|  |  |  |  |  |  |  |  |  |  |  |  |  |  |  |  |  |  |  |  |  |  |  |  |  |  |  |  |  |  |  |  |  |  |  |  |  |  | *fkh-10* | ForKHead transcription factor family |
|  |  |  |  |  |  |  |  |  |  |  |  |  |  |  |  |  |  |  |  |  |  |  |  |  |  |  |  |  |  |  |  |  |  |  |  |  |  | T25F10.3 |  |
|  |  |  |  |  |  |  |  |  |  |  |  |  |  |  |  |  |  |  |  |  |  |  |  |  |  |  |  |  |  |  |  |  |  |  |  |  |  | B0563.6 |  |
|  |  |  |  |  |  |  |  |  |  |  |  |  |  |  |  |  |  |  |  |  |  |  |  |  |  |  |  |  |  |  |  |  |  |  |  |  |  | *acr-2* | AcetylCholine Receptor |
|  |  |  |  |  |  |  |  |  |  |  |  |  |  |  |  |  |  |  |  |  |  |  |  |  |  |  |  |  |  |  |  |  |  |  |  |  |  | *unc-7* | UNCoordinated |
|  |  |  |  |  |  |  |  |  |  |  |  |  |  |  |  |  |  |  |  |  |  |  |  |  |  |  |  |  |  |  |  |  |  |  |  |  |  | Y9C12A.1 |  |
|  |  |  |  |  |  |  |  |  |  |  |  |  |  |  |  |  |  |  |  |  |  |  |  |  |  |  |  |  |  |  |  |  |  |  |  |  |  | *ceh-24* | C. Elegans Homeobox |
|  |  |  |  |  |  |  |  |  |  |  |  |  |  |  |  |  |  |  |  |  |  |  |  |  |  |  |  |  |  |  |  |  |  |  |  |  |  | H04M03.12 |  |
|  |  |  |  |  |  |  |  |  |  |  |  |  |  |  |  |  |  |  |  |  |  |  |  |  |  |  |  |  |  |  |  |  |  |  |  |  |  | K01F9.2 |  |
|  |  |  |  |  |  |  |  |  |  |  |  |  |  |  |  |  |  |  |  |  |  |  |  |  |  |  |  |  |  |  |  |  |  |  |  |  |  | ZK1067.4 |  |
|  |  |  |  |  |  |  |  |  |  |  |  |  |  |  |  |  |  |  |  |  |  |  |  |  |  |  |  |  |  |  |  |  |  |  |  |  |  | *rhgf-1* | RHo Guanine nucleotide exchange Factor |
|  |  |  |  |  |  |  |  |  |  |  |  |  |  |  |  |  |  |  |  |  |  |  |  |  |  |  |  |  |  |  |  |  |  |  |  |  |  | *ceh-17* | C. Elegans Homeobox |
|  |  |  |  |  |  |  |  |  |  |  |  |  |  |  |  |  |  |  |  |  |  |  |  |  |  |  |  |  |  |  |  |  |  |  |  |  |  | C55C3.8 |  |
|  |  |  |  |  |  |  |  |  |  |  |  |  |  |  |  |  |  |  |  |  |  |  |  |  |  |  |  |  |  |  |  |  |  |  |  |  |  | R02E12.5 |  |
|  |  |  |  |  |  |  |  |  |  |  |  |  |  |  |  |  |  |  |  |  |  |  |  |  |  |  |  |  |  |  |  |  |  |  |  |  |  | *grd-7* | GRounDhog (hedgehog-like family) |

### Phenotypes enriched

none found

### Anatomy terms enriched

|  |  |  |  |
| --- | --- | --- | --- |
| **Group name** | **Number in cluster** | **Enrichment** | **FDR corrected p** |
| DB neuron | 6 | 23.67 | 0.000524 |
| cholinergic neuron | 9 | 9.40 | 0.001040 |
| motor neuron | 9 | 7.83 | 0.004280 |
| SIBVR | 4 | 40.01 | 0.006200 |
| SIBVL | 4 | 40.01 | 0.006200 |
| SIB | 4 | 38.63 | 0.006950 |
| DA neuron | 5 | 17.08 | 0.020700 |

### GO terms enriched

|  |  |  |
| --- | --- | --- |
| **GO term** | **Number of genes** | **FDR-corrected p-value** |
| synapse part | 6 | 0.00078 |
| ligand-gated channel activity | 6 | 0.00094 |
| postsynaptic membrane | 5 | 0.00140 |
| cell junction | 6 | 0.00610 |
| channel activity | 7 | 0.00690 |
| acetylcholine-activated cation-selective channel activity | 3 | 0.00970 |
| ion transport | 8 | 0.01100 |
| ion channel activity | 6 | 0.02600 |
| extracellular ligand-gated ion channel activity | 4 | 0.03400 |

### Expression clusters enriched

|  |  |  |  |
| --- | --- | --- | --- |
| **Group name** | **Number in cluster** | **Enrichment** | **FDR corrected p** |
| Genes significantly enriched (> 2x, FDR < 5%) in a particular cell-type versus a reference sample of all cells at the same stage. WBPaper00037950:all-neurons\_larva\_enriched | 13 | 3.59 | 0.0116 |
| Larval Pan-neural Enriched Genes. | 16 | 2.90 | 0.0166 |
| Genes significantly enriched (> 2x, FDR < 5%) in a particular cell-type versus a reference sample of all cells at the same stage. WBPaper00037950:glr-1(+)-neurons\_larva\_enriched | 10 | 4.24 | 0.0243 |
| Genes significantly enriched (> 2x, FDR < 5%) in a particular cell-type versus a reference sample of all cells at the same stage. WBPaper00037950:A-class-motor-neurons\_larva\_enriched | 10 | 4.06 | 0.0329 |
| Genes enriched in neuronal miRNA-induced silencing complexes (miRISC) as detected by immunoprecipitations and microarray analysis. | 10 | 4.05 | 0.0339 |

### Motifs enriched

|  |  |  |  |  |  |
| --- | --- | --- | --- | --- | --- |
| **Motif** | **Logo** | **Possible orthologs** | **Number of motifs in cluster** | **Enrichment** | **FDR corrected p** |
| pTH9957 |  | irx-1 daf-16 fkh-9 | 29 | 3.03 | 1.8e-06 |
| Uncx\_1 |  | alr-1 ceh-14 cfi-1 ZC204.2 | 31 | 2.79 | 2.4e-06 |
| MCR\_f1 |  | nhr-255 | 40 | 2.15 | 2.9e-06 |
| V$FOXO4\_01 |  | fkh-10 (0.8) fkh-7 lin-31 fkh-8 pha-4 daf-16 let-381 | 42 | 2.05 | 3.0e-06 |
| pTH9885 |  | ceh-18 (0.58) unc-86 | 34 | 2.52 | 3.2e-06 |
| FLI1\_1 |  | lin-1 C24A1.2 | 40 | 2.12 | 4.3e-06 |
| Atf6\_SANGER\_5\_FBgn0033010 |  | atf-6 fos-1 crh-1 atf-7 C27D6.4 | 33 | 2.53 | 5.3e-06 |
| Elf3\_3876 |  | C24A1.2 | 44 | 1.90 | 7.4e-06 |
| PhdP\_Cell\_FBgn0025334 |  | ceh-12 (0.75) ceh-18 (0.58) lim-4 (0.53) ceh-43 ceh-16 ceh-31 ceh-36 pha-2 lin-39 egl-5 alr-1 pal-1 lim-6 mls-2 ceh-53 ceh-30 ceh-1 lim-7 ceh-14 cog-1 and 7 others  [full list] | 39 | 2.13 | 7.4e-06 |
| PhdP\_SOLEXA\_FBgn0025334 |  | lin-39 alr-1 eyg-1 ceh-1 ceh-10 ceh-45 | 39 | 2.13 | 7.4e-06 |
| MA0041.1 |  | lin-31 let-381 | 40 | 2.07 | 9.2e-06 |
| MA0063.1 |  | ceh-24 (0.74) ceh-8 ceh-43 ceh-31 ceh-9 lin-39 ceh-19 alr-1 ceh-30 ceh-1 lim-7 cog-1 | 32 | 2.54 | 1.0e-05 |
| Elf3 |  | C24A1.2 | 42 | 1.96 | 1.0e-05 |
| pTH10638 |  | dmd-3 C34D1.1 | 37 | 2.21 | 1.1e-05 |
| MEIS2\_2 |  | ceh-32 (0.54) lin-32 lin-39 | 38 | 2.14 | 1.4e-05 |
| Lmx1b\_3433 |  | ceh-24 (0.74) ceh-16 lim-6 lim-7 | 33 | 2.37 | 2.3e-05 |
| V$OCT1\_06 |  | ceh-18 (0.58) ztf-9 | 34 | 2.31 | 2.5e-05 |
| RFX1\_4537 |  | daf-19 (0.51) | 39 | 2.04 | 2.5e-05 |
| TCF4\_2 |  | hlh-2 ztf-6 | 36 | 2.19 | 2.5e-05 |
| MEIS3\_1 |  | ceh-32 (0.54) ces-1 ceh-20 F55C5.11 | 29 | 2.64 | 2.9e-05 |
| V$POU3F2\_01 |  | ceh-18 (0.58) dmd-3 | 33 | 2.35 | 3.0e-05 |
| Sry\_2833 |  | pop-1 gei-3 sox-4 C05C9.3 | 23 | 3.27 | 3.1e-05 |
| MA0079.3 |  | klf-1 ZC328.2 klf-2 | 23 | 3.27 | 3.2e-05 |
| Elf4 |  | C24A1.2 | 39 | 2.02 | 3.4e-05 |
| V$FOXO4\_02 |  | fkh-10 (0.8) fkh-7 lin-31 fkh-8 daf-16 let-381 | 18 | 4.19 | 3.6e-05 |
| MA0475.1 |  | lin-1 C24A1.2 | 40 | 1.95 | 4.4e-05 |
| Six6\_2267 |  | ceh-32 (0.54) ceh-34 elt-1 elt-7 egl-27 elt-3 | 17 | 4.37 | 4.8e-05 |
| MA0029.1 |  | elt-1 ztf-29 | 32 | 2.36 | 4.9e-05 |
| GM12878\_SRF\_HudsonAlpha |  | hlh-10 unc-120 | 32 | 2.35 | 5.4e-05 |
| pTH6425 |  | pop-1 ceh-20 | 36 | 2.12 | 5.5e-05 |
| Elf5 |  | C24A1.2 | 29 | 2.55 | 5.7e-05 |
| Pknox2\_3077 |  | ceh-32 (0.54) | 38 | 2.02 | 5.8e-05 |
| pTH5922 |  | ceh-24 (0.74) | 18 | 4.04 | 5.9e-05 |
| pTH2936 |  | nhr-239 | 35 | 2.16 | 5.9e-05 |
| pTH10015 |  | ces-1 ztf-28 che-1 | 40 | 1.92 | 6.3e-05 |
| pTH9290 |  | ceh-6 (0.69) ceh-18 (0.58) sox-4 tbp-1 | 47 | 1.66 | 6.4e-05 |
| Spt15 |  | tbp-1 | 36 | 2.10 | 6.5e-05 |
| EHF\_si |  | lin-1 nhr-19 C24A1.2 | 33 | 2.26 | 6.5e-05 |
| V$CREB\_Q4 |  | crh-1 attf-1 W08E12.1 | 36 | 2.09 | 7.3e-05 |
| CG5669\_SANGER\_10\_FBgn0039169 |  | klf-1 klf-2 | 37 | 2.04 | 7.4e-05 |
| pTH9135 |  | pop-1 | 36 | 2.09 | 7.9e-05 |
| Elf2 |  | lin-1 C24A1.2 | 38 | 1.99 | 8.4e-05 |
| MA0537.1 |  | blmp-1 | 42 | 1.82 | 8.4e-05 |
| retn\_SANGER\_5\_FBgn0004795 |  | let-381 ceh-20 cfi-1 | 35 | 2.13 | 8.6e-05 |
| Tin\_SOLEXA\_FBgn0004110 |  | ceh-24 (0.74) ceh-22 dsc-1 | 34 | 2.18 | 8.6e-05 |
| EN2\_2 |  | ceh-43 ceh-16 ceh-31 ceh-9 lin-39 alr-1 ceh-1 lim-7 | 36 | 2.08 | 8.7e-05 |
| pTH5781 |  | ceh-32 (0.54) | 26 | 2.73 | 9.4e-05 |
| MA0173.1 |  | hlh-32 irx-1 | 48 | 1.61 | 9.5e-05 |
| Pbx1\_3203 |  | ceh-20 | 42 | 1.81 | 1.0e-04 |
| Nkx1-2\_3214 |  | ceh-30 | 26 | 2.71 | 1.0e-04 |
| HXD13\_f1 |  | mex-6 pal-1 | 29 | 2.47 | 1.1e-04 |
| HLH4C\_da\_SANGER\_5\_FBgn0011277 |  | ces-1 hlh-1 hlh-8 hlh-15 | 36 | 2.06 | 1.1e-04 |
| TBX2\_2 |  | mab-9 (0.54) tbx-38 tbx-39 tbx-43 | 28 | 2.53 | 1.1e-04 |
| Sox15\_3457 |  | sox-4 | 32 | 2.26 | 1.2e-04 |
| Etv6 |  | lin-1 C24A1.2 | 40 | 1.88 | 1.2e-04 |
| Hmx1\_3423 |  | ceh-9 | 36 | 2.04 | 1.3e-04 |
| pTH6486 |  | nhr-145 | 28 | 2.51 | 1.3e-04 |
| TBX3\_f1 |  | tbx-39 ceh-45 | 38 | 1.95 | 1.4e-04 |
| V$ARP1\_01 |  | nhr-2 nhr-19 nhr-62 | 26 | 2.66 | 1.4e-04 |
| ARI3A\_do |  | gei-3 cfi-1 | 32 | 2.24 | 1.5e-04 |
| Dlx5\_3419 |  | ceh-12 (0.75) ceh-18 (0.58) npax-3 ceh-43 lin-39 alr-1 ceh-53 ceh-1 ceh-45 | 28 | 2.50 | 1.5e-04 |
| MA0135.1 |  | lin-39 php-3 unc-86 lim-7 cfi-1 | 29 | 2.42 | 1.5e-04 |
| HXC8\_f1 |  | ceh-12 (0.75) lin-39 ceh-20 | 31 | 2.29 | 1.5e-04 |
| Sp4\_1011 |  | klf-1 klf-2 sptf-3 | 35 | 2.07 | 1.6e-04 |
| pTH2283 |  | odd-2 | 41 | 1.82 | 1.6e-04 |
| BARHL2\_6 |  | ceh-43 ceh-31 lin-39 ceh-1 | 15 | 4.50 | 1.7e-04 |
| MA0246.1 |  | ceh-32 (0.54) dmd-5 dmd-4 | 24 | 2.82 | 1.7e-04 |
| Sox17\_2837 |  | sox-4 | 43 | 1.74 | 1.7e-04 |
| pTH10645 |  | elt-1 nhr-7 nhr-100 | 17 | 3.88 | 2.0e-04 |
| Tcf7\_0950 |  | pop-1 | 37 | 1.96 | 2.1e-04 |
| pTH6108 |  | fkh-10 (0.8) lin-31 let-381 C34D1.1 | 36 | 2.00 | 2.1e-04 |
| MA0235.1 |  | ceh-48 dsc-1 | 35 | 2.04 | 2.3e-04 |
| SPDEF\_6 |  | lin-1 lin-39 nhr-100 | 42 | 1.75 | 2.4e-04 |
| pTH5119 |  | cfi-1 | 24 | 2.75 | 2.6e-04 |
| FOXK1\_1 |  | lin-31 nhr-213 | 36 | 1.98 | 2.7e-04 |
| FOXB1\_1 |  | lin-31 F55C5.11 | 38 | 1.89 | 2.8e-04 |
| V$AREB6\_02 |  | ztf-6 | 37 | 1.93 | 3.0e-04 |
| CG2052\_SANGER\_2.5\_FBgn0039905 |  | mel-28 lin-29 fkh-7 | 39 | 1.85 | 3.0e-04 |
| pTH8566 |  | lin-54 | 33 | 2.11 | 3.0e-04 |
| MA0485.1 |  | hbl-1 lin-39 php-3 | 31 | 2.21 | 3.0e-04 |
| pTH10811 |  | nhr-216 nhr-84 nhr-142 | 26 | 2.55 | 3.0e-04 |
| NR4A2\_2 |  | nhr-71 nhr-6 nhr-2 nhr-213 nhr-68 Y67D8A.3 | 31 | 2.21 | 3.1e-04 |
| V$NCX\_01 |  | ceh-19 | 25 | 2.62 | 3.2e-04 |
| NR1D1\_f1 |  | nhr-213 nhr-118 | 35 | 2.00 | 3.4e-04 |
| pTH8983 |  | tag-347 | 45 | 1.64 | 3.4e-04 |
| pTH9242 |  | mel-28 | 38 | 1.88 | 3.4e-04 |
| Pax7\_3783 |  | ceh-18 (0.58) alr-1 lim-7 | 26 | 2.52 | 3.7e-04 |
| V$MEF2\_01 |  | mel-28 mef-2 let-381 Y61A9LA.9 Y116A8C.22 | 27 | 2.44 | 3.7e-04 |
| pTH9335 |  | mel-28 | 45 | 1.63 | 3.7e-04 |
| Etv3 |  | lin-1 | 37 | 1.90 | 3.9e-04 |
| pTH3064 |  | crh-1 | 34 | 2.03 | 3.9e-04 |
| Sox1\_2631 |  | sox-4 | 32 | 2.12 | 4.3e-04 |
| pTH5118 |  | cfi-1 | 32 | 2.12 | 4.4e-04 |
| MA0124.1 |  | ceh-24 (0.74) | 31 | 2.17 | 4.5e-04 |
| SRP001585\_Tbx2 |  | slr-2 odr-7 tbx-39 nhr-28 | 38 | 1.85 | 4.6e-04 |
| CRX\_si |  | ceh-36 pha-2 dve-1 alr-1 ceh-53 ceh-45 | 33 | 2.06 | 4.6e-04 |
| pTH5462 |  | jun-1 fos-1 crh-1 sknr-1 F45H11.6 | 34 | 2.01 | 4.9e-04 |
| ZBTB49\_1 |  | C46E10.9 | 38 | 1.85 | 4.9e-04 |
| pTH9353 |  | ceh-51 | 36 | 1.92 | 5.0e-04 |
| pTH9260 |  | mel-28 | 34 | 2.00 | 5.5e-04 |
| pTH10038 |  | gei-3 sox-4 F56D1.1 | 33 | 2.04 | 5.6e-04 |
| NKX28\_f1 |  | ceh-24 (0.74) ces-1 T22H9.4 | 35 | 1.95 | 5.6e-04 |
| Irx3\_1 |  | irx-1 | 30 | 2.19 | 5.7e-04 |
| Nkx1-1\_3856 |  | ceh-30 | 24 | 2.61 | 5.8e-04 |
| FOXO6\_3 |  | ZC328.2 daf-16 | 26 | 2.45 | 5.8e-04 |
| pTH9214 |  | cfi-1 | 38 | 1.83 | 5.8e-04 |
| pTH6569 |  | ceh-43 | 25 | 2.52 | 5.9e-04 |
| pTH6106 |  | nhr-182 | 37 | 1.86 | 6.5e-04 |
| V$CDXA\_01 |  | php-3 ceh-13 | 27 | 2.36 | 6.6e-04 |
| Nkx2-3\_3435 |  | ceh-24 (0.74) dsc-1 | 32 | 2.07 | 6.9e-04 |
| Oli\_da\_SANGER\_5\_1\_FBgn0032651 |  | hlh-32 hlh-8 lin-31 hlh-15 ngn-1 | 36 | 1.89 | 7.3e-04 |
| MA0262.1 |  | mab-3 hsf-1 | 34 | 1.97 | 7.5e-04 |
| N$SKN1\_01 |  | skn-1 ceh-2 | 37 | 1.85 | 7.5e-04 |
| MA0502.1 |  | nfya-2 dro-1 lin-31 ceh-20 | 27 | 2.34 | 7.7e-04 |
| pTH10034 |  | nhr-66 | 55 | 1.29 | 7.9e-04 |
| CG4854\_SANGER\_10\_FBgn0038766 |  | K11D2.4 mxl-1 | 29 | 2.20 | 8.2e-04 |
| pTH10647 |  | nhr-232 | 25 | 2.47 | 8.2e-04 |
| V$TCF11\_01 |  | skn-1 | 25 | 2.47 | 8.2e-04 |
| pTH6497 |  | lin-31 | 35 | 1.92 | 8.4e-04 |
| pTH3998 |  | tbx-39 | 29 | 2.20 | 8.7e-04 |
| EMX1\_2 |  | ceh-16 ceh-2 | 47 | 1.53 | 8.8e-04 |
| pTH9300 |  | dmd-3 C34D1.1 | 28 | 2.25 | 8.9e-04 |
| Sox4 |  | pop-1 sox-4 nhr-100 | 32 | 2.04 | 9.1e-04 |
| MA0538.1 |  | ceh-9 daf-12 ztf-3 | 28 | 2.24 | 9.4e-04 |
| Jundm2\_0911 |  | fos-1 skn-1 | 34 | 1.94 | 1.0e-03 |
| pTH8916 |  | hmbx-1 ceh-43 lin-39 hmg-12 ceh-53 let-381 Y116A8C.22 | 29 | 2.18 | 1.0e-03 |
| HOXA13\_2 |  | ceh-24 (0.74) lin-39 pal-1 ceh-13 T27F2.4 D1005.3 | 56 | 1.24 | 1.1e-03 |
| pTH3831 |  | ces-2 C01B12.2 F23F12.9 | 35 | 1.89 | 1.1e-03 |
| tll\_FlyReg\_FBgn0003720 |  | nhr-2 nhr-239 nhr-62 | 24 | 2.48 | 1.2e-03 |
| Lbx2\_3869 |  | mls-2 | 37 | 1.81 | 1.2e-03 |
| V$CEBPA\_01 |  | C48E7.11 | 37 | 1.81 | 1.2e-03 |
| Vsx1\_1728 |  | alr-1 | 36 | 1.84 | 1.2e-03 |
| pTH5924 |  | nhr-255 | 37 | 1.81 | 1.3e-03 |
| V$TAXCREB\_02 |  | crh-1 zip-3 | 24 | 2.48 | 1.3e-03 |
| V$RFX1\_02 |  | daf-19 (0.51) F52B5.7 | 36 | 1.84 | 1.3e-03 |
| pTH10722 |  | eor-1 egrh-3 | 31 | 2.04 | 1.3e-03 |
| Hoxd10\_2368 |  | lin-39 php-3 | 25 | 2.39 | 1.3e-03 |
| Prop1\_3949 |  | ceh-16 ceh-53 | 24 | 2.45 | 1.4e-03 |
| Hlxb9\_3422 |  | ceh-12 (0.75) | 22 | 2.62 | 1.5e-03 |
| PROX1\_1 |  | crh-1 ceh-26 | 33 | 1.94 | 1.5e-03 |
| Fer2\_da\_SANGER\_5\_FBgn0038402 |  | lin-32 hlh-14 hlh-1 | 35 | 1.86 | 1.5e-03 |
| ZN384\_f1 |  | lin-29 K11D2.4 | 38 | 1.76 | 1.5e-03 |
| Hoxc13\_3127 |  | ceh-24 (0.74) pal-1 | 47 | 1.49 | 1.7e-03 |
| PLAG1\_si |  | Y53H1A.2 | 32 | 1.97 | 1.7e-03 |
| V$BRN2\_01 |  | ceh-18 (0.58) | 31 | 2.02 | 1.7e-03 |
| pTH3046 |  | Y116A8C.22 | 33 | 1.93 | 1.7e-03 |
| MA0497.1 |  | mef-2 | 19 | 2.91 | 1.7e-03 |
| Eip74EF\_FlyReg\_FBgn0000567 |  | K02D7.2 C24A1.2 | 32 | 1.97 | 1.8e-03 |
| pTH10772 |  | ceh-52 | 29 | 2.11 | 1.8e-03 |
| pTH10623 |  | scrt-1 | 15 | 3.58 | 1.8e-03 |
| pTH9245 |  | ceh-18 (0.58) | 40 | 1.68 | 1.8e-03 |
| pTH9911 |  | fos-1 atf-5 crh-1 | 51 | 1.38 | 1.9e-03 |
| MA0007.2 |  | nhr-255 npax-1 lin-14 | 38 | 1.74 | 1.9e-03 |
| pTH9974 |  | hlh-32 hlh-16 ngn-1 | 37 | 1.77 | 1.9e-03 |
| V$FREAC7\_01 |  | lin-31 | 19 | 2.88 | 2.0e-03 |
| KLF6\_si |  | klf-1 ZC328.2 klf-2 | 29 | 2.09 | 2.0e-03 |
| pTH9384 |  | cfi-1 | 21 | 2.65 | 2.0e-03 |
| pTH9137 |  | nhr-65 | 42 | 1.61 | 2.1e-03 |
| V$VMYB\_01 |  | lin-48 D1081.8 | 27 | 2.19 | 2.2e-03 |
| pTH10630 |  | lsy-27 | 36 | 1.79 | 2.2e-03 |
| PAX6\_f1 |  | pax-3 pax-2 | 33 | 1.90 | 2.2e-03 |
| Hoxc6\_3954 |  | ceh-12 (0.75) ceh-18 (0.58) ceh-43 lin-39 pal-1 | 24 | 2.38 | 2.2e-03 |
| pTH3084 |  | C01B12.2 attf-1 | 24 | 2.37 | 2.3e-03 |
| I$DFD\_01 |  | lin-39 | 21 | 2.62 | 2.3e-03 |
| Hoxb7\_3953 |  | ceh-6 (0.69) ceh-18 (0.58) lin-39 | 25 | 2.30 | 2.4e-03 |
| FLI1\_f1 |  | lin-1 | 35 | 1.82 | 2.4e-03 |
| V$CETS1P54\_02 |  | C52B9.2 | 25 | 2.29 | 2.5e-03 |
| rn\_SOLEXA\_5\_FBgn0259172 |  | lin-29 | 36 | 1.78 | 2.5e-03 |
| TBX2\_f1 |  | tbx-39 | 26 | 2.22 | 2.7e-03 |
| pTH9198 |  | dmd-3 | 40 | 1.65 | 2.8e-03 |
| pTH8991 |  | cey-3 | 34 | 1.84 | 2.9e-03 |
| Irx5\_2385 |  | irx-1 | 32 | 1.91 | 2.9e-03 |
| pTH8998 |  | mab-3 | 43 | 1.56 | 3.0e-03 |
| MAFA\_f1 |  | F45H11.6 | 37 | 1.73 | 3.0e-03 |
| pTH9165 |  | ztf-27 | 30 | 1.99 | 3.1e-03 |
| pTH10777 |  | dmd-3 | 38 | 1.70 | 3.3e-03 |
| pTH9082 |  | mab-23 | 34 | 1.83 | 3.3e-03 |
| pTH10633 |  | R07H5.10 C48E7.11 | 42 | 1.59 | 3.3e-03 |
| MYF6\_f1 |  | hlh-1 hlh-15 | 50 | 1.39 | 3.3e-03 |
| pTH9261 |  | dmd-3 lin-48 pax-3 | 35 | 1.79 | 3.3e-03 |
| V$T3R\_01 |  | nhr-239 nhr-15 | 31 | 1.94 | 3.3e-03 |
| pTH9142 |  | ztf-6 C34D1.1 gei-11 | 39 | 1.67 | 3.4e-03 |
| MA0139.1 |  | F58G1.2 Y5F2A.4 | 48 | 1.43 | 3.4e-03 |
| V$PAX2\_02 |  | pax-1 | 14 | 3.56 | 3.4e-03 |
| Zfp691\_0895 |  | F21A9.2 (0.71) CELE\_Y38H8A.5 | 31 | 1.94 | 3.5e-03 |
| pTH9884 |  | tbx-39 | 12 | 4.11 | 3.6e-03 |
| Nkx6-3\_3446 |  | cog-1 | 43 | 1.55 | 3.7e-03 |
| V$FOXJ2\_02 |  | lin-31 | 38 | 1.69 | 3.7e-03 |
| pTH9108 |  | daf-12 nhr-5 | 27 | 2.11 | 3.7e-03 |
| pTH9244 |  | tbx-39 | 27 | 2.11 | 3.8e-03 |
| K562\_GATA2\_HudsonAlpha |  | elt-1 alr-1 | 23 | 2.36 | 3.8e-03 |
| Tcf3\_3787 |  | pop-1 | 39 | 1.65 | 4.0e-03 |
| dys\_tgo\_SANGER\_5\_FBgn0015014 |  | mdl-1 (0.68) pax-1 aha-1 hlh-30 | 24 | 2.28 | 4.0e-03 |
| pTH10769 |  | Y48G1C.6 | 33 | 1.84 | 4.0e-03 |
| V$GATA6\_01 |  | elt-1 | 37 | 1.71 | 4.1e-03 |
| I$ELF1\_01 |  | sox-4 dmd-4 grh-1 | 41 | 1.60 | 4.1e-03 |
| MA0386.1 |  | K11D2.4 tbp-1 | 31 | 1.92 | 4.2e-03 |
| pTH6641 |  | lin-31 | 33 | 1.84 | 4.2e-03 |
| pTH3477 |  | daf-16 | 26 | 2.15 | 4.3e-03 |
| MA0543.1 |  | daf-8 eor-1 | 40 | 1.62 | 4.3e-03 |
| Hoxd1\_3448 |  | ceh-12 (0.75) | 23 | 2.33 | 4.5e-03 |
| pTH6562 |  | ceh-5 | 33 | 1.83 | 4.7e-03 |
| pTH1294 |  | mel-28 | 20 | 2.57 | 4.7e-03 |
| pTH3819 |  | ceh-18 (0.58) | 24 | 2.25 | 4.7e-03 |
| pTH6556 |  | lim-6 odd-1 | 24 | 2.25 | 4.7e-03 |
| HXD10\_f1 |  | php-3 nhr-2 | 41 | 1.59 | 4.8e-03 |
| So\_Cell\_FBgn0003460 |  | ceh-32 (0.54) | 34 | 1.79 | 4.8e-03 |
| pTH6071 |  | C46E10.8 C33G8.2 | 24 | 2.25 | 4.9e-03 |
| pTH5714 |  | nhr-239 | 32 | 1.86 | 4.9e-03 |
| Nkx6-1\_2825 |  | cog-1 | 24 | 2.24 | 5.1e-03 |
| pTH9026 |  | attf-1 | 24 | 2.24 | 5.1e-03 |
| Zfp652\_1 |  | ZK177.3 B0310.2 | 34 | 1.78 | 5.2e-03 |
| Irx3\_2226 |  | irx-1 | 31 | 1.89 | 5.2e-03 |
| pTH6449 |  | ceh-43 | 23 | 2.30 | 5.4e-03 |
| pTH10037 |  | T22C8.4 ref-2 | 45 | 1.48 | 5.4e-03 |
| pTH10013 |  | nhr-168 | 39 | 1.62 | 5.8e-03 |
| HepG2\_HSF1\_Stanford |  | Y53C10A.3 | 32 | 1.84 | 5.8e-03 |
| pTH10718 |  | egl-43 | 28 | 2.00 | 5.9e-03 |
| pTH2684 |  | fos-1 | 36 | 1.71 | 6.0e-03 |
| pTH9925 |  | ztf-11 nhr-100 | 30 | 1.91 | 6.0e-03 |
| V$TATA\_C |  | tbp-1 | 38 | 1.65 | 6.2e-03 |
| HXA7\_f1 |  | lin-39 elt-1 | 39 | 1.62 | 6.4e-03 |
| V$YY1\_01 |  | lsy-2 | 35 | 1.73 | 6.7e-03 |
| pTH8985 |  | athp-1 | 28 | 1.98 | 6.9e-03 |
| Smad3\_3805 |  | daf-8 | 9 | 5.09 | 7.0e-03 |
| V$HOX13\_01 |  | lin-39 | 46 | 1.44 | 7.0e-03 |
| pTH10816 |  | dmd-6 | 33 | 1.78 | 7.2e-03 |
| GRHL1\_2 |  | grh-1 | 21 | 2.38 | 7.3e-03 |
| pTH6636 |  | egl-5 | 35 | 1.72 | 7.4e-03 |
| CG8765\_SANGER\_5\_FBgn0036900 |  | H20J04.3 | 27 | 2.01 | 7.8e-03 |
| KLF8\_f1 |  | klf-1 | 16 | 2.90 | 7.9e-03 |
| Gmeb1\_1745 |  | attf-1 | 23 | 2.22 | 8.5e-03 |
| I$ABDB\_01 |  | ceh-24 (0.74) lin-39 php-3 pal-1 | 22 | 2.27 | 8.8e-03 |
| FLI1\_2 |  | lin-1 | 17 | 2.73 | 8.9e-03 |
| pTH10041 |  | ztf-29 | 31 | 1.83 | 8.9e-03 |
| pTH7875 |  | mel-28 | 26 | 2.04 | 8.9e-03 |
| Mw140 |  | efl-1 F49E12.6 | 33 | 1.76 | 8.9e-03 |
| PTF1A\_f1 |  | lin-32 | 38 | 1.62 | 9.1e-03 |
| pTH5159 |  | mxl-1 aha-1 hlh-30 mxl-2 | 29 | 1.90 | 9.2e-03 |
| pTH5887 |  | lin-39 | 33 | 1.76 | 9.3e-03 |
| pTH6003 |  | nhr-182 nhr-134 | 38 | 1.61 | 9.4e-03 |
| pTH6327 |  | dsc-1 | 30 | 1.86 | 9.5e-03 |
| pTH9237 |  | mel-28 | 44 | 1.47 | 9.5e-03 |
| pTH6436 |  | ceh-53 | 22 | 2.26 | 9.5e-03 |
| Hoxa7\_3750 |  | lin-39 | 34 | 1.72 | 9.7e-03 |
| pTH10796 |  | F10B5.3 hsf-1 Y53C10A.3 | 26 | 2.02 | 9.8e-03 |
| pTH8863 |  | hmg-12 | 37 | 1.64 | 9.8e-03 |
| TLX1\_f1 |  | ceh-19 | 18 | 2.59 | 9.9e-03 |
| pTH6268 |  | ceh-2 | 27 | 1.98 | 9.9e-03 |
| pTH9163 |  | nhr-3 (0.51) | 25 | 2.07 | 1.0e-02 |
| amos\_da\_SANGER\_10\_FBgn0003270 |  | hlh-32 hlh-15 | 51 | 1.31 | 1.1e-02 |
| Hoxb5\_3122 |  | lin-39 | 22 | 2.24 | 1.1e-02 |
| Irx3\_0920 |  | irx-1 | 27 | 1.97 | 1.1e-02 |
| Hoxa6\_1040 |  | lin-39 | 30 | 1.85 | 1.1e-02 |
| V$S8\_01 |  | ceh-45 | 37 | 1.63 | 1.1e-02 |
| Spdef\_0905 |  | lin-1 | 28 | 1.92 | 1.1e-02 |
| Mw160 |  | nhr-68 | 39 | 1.57 | 1.1e-02 |
| Hoxb8\_3780 |  | lin-39 | 33 | 1.73 | 1.1e-02 |
| Zbtb12\_2932 |  | lsy-27 ceh-90 | 37 | 1.62 | 1.2e-02 |
| Spdef |  | lin-1 | 30 | 1.83 | 1.2e-02 |
| pTH9907 |  | nhr-34 | 15 | 2.91 | 1.2e-02 |
| Plagl1\_0972 |  | Y53H1A.2 | 23 | 2.15 | 1.2e-02 |
| pTH9080 |  | mnm-2 | 30 | 1.83 | 1.2e-02 |
| pTH9215 |  | C34D1.1 | 39 | 1.57 | 1.2e-02 |
| HAND1\_si |  | sma-4 hlh-8 | 24 | 2.10 | 1.2e-02 |
| pTH6478 |  | lim-7 | 22 | 2.21 | 1.2e-02 |
| Eip93F\_SANGER\_10\_FBgn0013948 |  | mbr-1 (0.58) F26F4.8 nhr-177 bed-3 | 29 | 1.86 | 1.2e-02 |
| pTH6447 |  | ceh-19 | 34 | 1.70 | 1.2e-02 |
| V$GR\_Q6 |  | nhr-255 | 23 | 2.14 | 1.3e-02 |
| pTH9150 |  | odd-1 | 47 | 1.39 | 1.3e-02 |
| Hoxa5\_3415 |  | lin-39 | 22 | 2.20 | 1.3e-02 |
| pTH5812 |  | ceh-14 | 30 | 1.82 | 1.3e-02 |
| pTH6591 |  | lin-31 | 33 | 1.71 | 1.4e-02 |
| SMAD3\_f1 |  | daf-8 | 22 | 2.19 | 1.4e-02 |
| Nsy-7 |  | nsy-7 | 26 | 1.97 | 1.4e-02 |
| pTH2280 |  | mnm-2 | 27 | 1.93 | 1.4e-02 |
| CXXC1\_si |  | F52B11.1 | 31 | 1.78 | 1.4e-02 |
| pTH9381 |  | ceh-18 (0.58) | 37 | 1.60 | 1.4e-02 |
| Hoxa7\_2668 |  | lin-39 | 28 | 1.88 | 1.5e-02 |
| SPDEF\_2 |  | lin-1 | 27 | 1.92 | 1.5e-02 |
| Dlx1\_1741 |  | ceh-43 | 22 | 2.18 | 1.5e-02 |
| pTH10650 |  | nhr-153 | 32 | 1.74 | 1.5e-02 |
| V$CEBP\_01 |  | C48E7.11 | 56 | 1.17 | 1.5e-02 |
| MA0027.1 |  | ceh-16 | 52 | 1.28 | 1.5e-02 |
| Hmbox1\_2674 |  | hmbx-1 | 28 | 1.88 | 1.5e-02 |
| SH-SY5Y\_GATA2\_UCD |  | elt-1 | 36 | 1.62 | 1.5e-02 |
| pTH9182 |  | tbx-39 | 36 | 1.62 | 1.5e-02 |
| Barx1\_2877 |  | ceh-43 | 22 | 2.17 | 1.6e-02 |
| Hoxc8\_3429 |  | lin-39 | 23 | 2.10 | 1.6e-02 |
| pTH9709 |  | die-1 | 31 | 1.76 | 1.6e-02 |
| pTH5561 |  | nhr-239 | 34 | 1.67 | 1.6e-02 |
| pTH9254 |  | mel-28 | 44 | 1.44 | 1.6e-02 |
| MA0032.1 |  | let-381 | 44 | 1.44 | 1.6e-02 |
| MA0131.1 |  | F39B2.1 | 15 | 2.79 | 1.7e-02 |
| T-47D\_GATA3\_HudsonAlpha |  | elt-1 | 36 | 1.61 | 1.7e-02 |
| pTH4325 |  | ceh-18 (0.58) | 26 | 1.94 | 1.7e-02 |
| GATA6\_f2 |  | end-3 (0.55) elt-1 | 33 | 1.69 | 1.7e-02 |
| Mcm1 |  | unc-120 | 33 | 1.69 | 1.8e-02 |
| Dlx3\_1030 |  | ceh-43 | 22 | 2.14 | 1.8e-02 |
| Hoxa10\_2318 |  | ceh-24 (0.74) | 41 | 1.49 | 1.8e-02 |
| Hoxd13\_2356 |  | pal-1 | 27 | 1.89 | 1.8e-02 |
| pTH9125 |  | egl-13 K11D2.4 | 26 | 1.93 | 1.9e-02 |
| Dlx2\_2273 |  | ceh-43 | 22 | 2.13 | 1.9e-02 |
| Irx2\_0900 |  | irx-1 | 29 | 1.81 | 1.9e-02 |
| Hey\_SANGER\_5\_FBgn0027788 |  | lin-22 gei-11 | 28 | 1.84 | 1.9e-02 |
| pTH3751 |  | tbx-39 | 33 | 1.68 | 2.0e-02 |
| Atf1\_3026 |  | crh-1 | 34 | 1.65 | 2.0e-02 |
| pTH8745 |  | attf-1 | 21 | 2.18 | 2.0e-02 |
| pTH10805 |  | ztf-16 | 22 | 2.12 | 2.0e-02 |
| pTH9164 |  | ceh-26 | 24 | 2.01 | 2.0e-02 |
| Pou2f3\_3986 |  | ceh-18 (0.58) | 32 | 1.70 | 2.1e-02 |
| pTH9216 |  | ceh-18 (0.58) | 21 | 2.17 | 2.1e-02 |
| pTH5778 |  | egl-5 | 23 | 2.06 | 2.1e-02 |
| ECC-1\_ERALPHA\_HudsonAlpha |  | nhr-71 | 46 | 1.38 | 2.1e-02 |
| E2F4\_1 |  | nfi-1 F49E12.6 | 30 | 1.76 | 2.1e-02 |
| pTH9097 |  | Y116A8C.22 | 36 | 1.59 | 2.1e-02 |
| Pou3f4\_3773 |  | ceh-6 (0.69) | 31 | 1.73 | 2.1e-02 |
| pTH9220 |  | mbr-1 (0.58) | 28 | 1.83 | 2.2e-02 |
| V$GATA1\_02 |  | elt-1 | 19 | 2.30 | 2.2e-02 |
| pTH7032 |  | F52B11.1 | 43 | 1.44 | 2.2e-02 |
| pTH9297 |  | ceh-18 (0.58) | 38 | 1.54 | 2.4e-02 |
| Bsx\_3483 |  | ceh-31 | 21 | 2.15 | 2.4e-02 |
| pTH9924 |  | nhr-46 | 14 | 2.82 | 2.4e-02 |
| CUX1\_2 |  | ceh-48 | 39 | 1.51 | 2.4e-02 |
| pTH9380 |  | mel-28 | 36 | 1.58 | 2.4e-02 |
| pTH10717 |  | syd-9 lsl-1 | 50 | 1.29 | 2.6e-02 |
| pTH10768 |  | med-2 (0.53) | 45 | 1.39 | 2.6e-02 |
| pTH10030 |  | xbp-1 (0.54) | 29 | 1.76 | 2.7e-02 |
| pTH9043 |  | sem-2 | 25 | 1.91 | 2.7e-02 |
| Meox1\_2310 |  | ceh-31 | 31 | 1.70 | 2.8e-02 |
| MA0058.2 |  | mxl-1 | 30 | 1.73 | 2.8e-02 |
| Pou3f1\_3819 |  | ceh-6 (0.69) | 34 | 1.61 | 2.8e-02 |
| pTH5919 |  | irx-1 | 15 | 2.62 | 2.8e-02 |
| Barx2\_3447 |  | ceh-43 | 22 | 2.05 | 2.9e-02 |
| Vax1\_3499 |  | C02F12.10 | 21 | 2.10 | 3.0e-02 |
| MA0117.1 |  | F45H11.6 | 15 | 2.60 | 3.0e-02 |
| MA0163.1 |  | Y53H1A.2 | 25 | 1.89 | 3.1e-02 |
| pTH3041 |  | atf-2 | 28 | 1.78 | 3.2e-02 |
| pTH2846 |  | lin-31 | 29 | 1.74 | 3.2e-02 |
| I$UBX\_01 |  | lin-39 | 24 | 1.93 | 3.2e-02 |
| pTH6445 |  | ceh-5 | 55 | 1.18 | 3.2e-02 |
| pTH9969 |  | pag-3 | 24 | 1.93 | 3.3e-02 |
| Mafk\_3106 |  | F45H11.6 | 25 | 1.88 | 3.3e-02 |
| Lhx1\_2240 |  | lim-7 | 22 | 2.02 | 3.3e-02 |
| Vax2\_3500 |  | C02F12.10 | 21 | 2.07 | 3.4e-02 |
| MA0085.1 |  | lag-1 | 33 | 1.62 | 3.5e-02 |
| Pou3f3\_3235 |  | ceh-6 (0.69) | 33 | 1.62 | 3.5e-02 |
| pTH8318 |  | attf-1 | 21 | 2.06 | 3.7e-02 |
| Cdx2\_4272 |  | ceh-13 | 36 | 1.54 | 3.7e-02 |
| Tcf1\_2666 |  | hmbx-1 | 31 | 1.66 | 3.8e-02 |
| Evx1\_3952 |  | ceh-53 | 20 | 2.11 | 3.8e-02 |
| pTH6423 |  | pha-2 | 31 | 1.66 | 3.8e-02 |
| pTH3510 |  | nhr-177 nhr-86 | 22 | 2.00 | 3.8e-02 |
| Tcf1\_2666 |  | hmbx-1 | 24 | 1.90 | 3.9e-02 |
| MA0095.2 |  | lsy-2 | 38 | 1.49 | 3.9e-02 |
| pTH9958 |  | ztf-6 | 27 | 1.78 | 4.1e-02 |
| Barhl1\_1 |  | ceh-31 | 33 | 1.60 | 4.1e-02 |
| HLH27 |  | hlh-27 | 28 | 1.74 | 4.1e-02 |
| Hoxa3\_2783 |  | lin-39 | 20 | 2.09 | 4.1e-02 |
| pTH8216 |  | Y116A8C.22 | 31 | 1.65 | 4.2e-02 |
| pTH5516 |  | nhr-2 | 19 | 2.15 | 4.2e-02 |
| pTH9256 |  | ceh-18 (0.58) | 37 | 1.50 | 4.4e-02 |
| pTH10808 |  | ztf-19 | 31 | 1.64 | 4.5e-02 |
| V$FAC1\_01 |  | gei-8 | 30 | 1.66 | 4.7e-02 |

### Correlated (and anti-correlated) transcription factors

|  |  |
| --- | --- |
| **Transcription factor** | **Correlation** |
| ceh-17 | 0.81 |
| fkh-10 | 0.80 |
| hlh-34 | 0.79 |
| ceh-12 | 0.75 |
| zfh-2 | 0.75 |
| hlh-13 | 0.75 |
| ceh-24 | 0.74 |
| zip-1 | 0.73 |
| ztf-26 | 0.71 |
| F21A9.2 | 0.71 |
| unc-42 | 0.71 |
| fezf-1 | 0.70 |
| ceh-6 | 0.69 |
| mgl-2 | 0.69 |
| mdl-1 | 0.68 |
| ctbp-1 | 0.66 |
| ZK686.5 | 0.66 |
| daf-3 | 0.65 |
| crh-2 | 0.64 |
| nhr-1 | 0.62 |
| aptf-4 | 0.61 |
| F26A10.2 | 0.61 |
| sem-4 | 0.60 |
| madf-4 | 0.60 |
| aptf-1 | 0.60 |
| sma-4 | -0.42 |
| sup-35 | -0.43 |
| dhhc-6 | -0.43 |
| Y82E9BR.17 | -0.43 |
| icd-2 | -0.44 |
| Y48G9A.11 | -0.44 |
| nhr-267 | -0.44 |
| ces-2 | -0.45 |
| lin-32 | -0.45 |
| ceh-82 | -0.45 |
| nhr-13 | -0.45 |
| dnj-11 | -0.46 |
| duxl-1 | -0.48 |
| nhr-64 | -0.49 |
| C28G1.4 | -0.49 |
| nhr-106 | -0.49 |
| C16A3.4 | -0.49 |
| hmg-6 | -0.51 |
| C30G4.7 | -0.52 |
| F27D4.4 | -0.53 |
| zip-7 | -0.53 |
| nhr-229 | -0.55 |
| nhr-256 | -0.56 |
| Y53F4B.3 | -0.58 |
| hlh-29 | -0.61 |

### ChIP peaks enriched

none found
