# Supplementary material for: Overlapping cell population expression profiling and regulatory inference in C. elegans
Source: BMC Genomics. 2016 Feb 29;17:159. doi: 10.1186/s12864-016-2482-z (PMC4772325; doi:10.1186/s12864-016-2482-z)
Supplement: Additional file 13: — Web supplement. (DOC 21 kb) [file 12864_2016_2482_MOESM13_ESM.zip › sortWeb/clusters/hier.300.clusters/11.html]

Cluster 11 

## Cluster 11

### Expression

| cnd-1 rep. 1 | cnd-1 rep. 2 | cnd-1 rep. 3 | pha-4 rep. 1 | pha-4 rep. 2 | pha-4 rep. 3 | ceh-27 | ceh-36 | ceh-6 | F21D5.9 | mir-57 | mls-2 | pal-1 | pros-1 | ttx-3 | unc-130 | hlh-16 | irx-1 | ceh-6 (+) hlh-16 (+) | ceh-6 (+) hlh-16 (-) | ceh-6 (-) hlh-16 (+) | cnd-1 singlets | pha-4 singlets | 0 | 60 | 120 | 150 | 180 | 240 | 330 | 390 | 420 | 480 | 540 | 570 | 600 | 630 | 660 | NAME | Functional description |
| --- | --- | --- | --- | --- | --- | --- | --- | --- | --- | --- | --- | --- | --- | --- | --- | --- | --- | --- | --- | --- | --- | --- | --- | --- | --- | --- | --- | --- | --- | --- | --- | --- | --- | --- | --- | --- | --- | --- | --- |
|  |  |  |  |  |  |  |  |  |  |  |  |  |  |  |  |  |  |  |  |  |  |  |  |  |  |  |  |  |  |  |  |  |  |  |  |  |  | T05B11.1 |  |
|  |  |  |  |  |  |  |  |  |  |  |  |  |  |  |  |  |  |  |  |  |  |  |  |  |  |  |  |  |  |  |  |  |  |  |  |  |  | *dhps-1* | DeoxyHyPusine Synthase |
|  |  |  |  |  |  |  |  |  |  |  |  |  |  |  |  |  |  |  |  |  |  |  |  |  |  |  |  |  |  |  |  |  |  |  |  |  |  | T25G3.3 |  |
|  |  |  |  |  |  |  |  |  |  |  |  |  |  |  |  |  |  |  |  |  |  |  |  |  |  |  |  |  |  |  |  |  |  |  |  |  |  | E02H1.1 |  |
|  |  |  |  |  |  |  |  |  |  |  |  |  |  |  |  |  |  |  |  |  |  |  |  |  |  |  |  |  |  |  |  |  |  |  |  |  |  | C17G10.1 |  |
|  |  |  |  |  |  |  |  |  |  |  |  |  |  |  |  |  |  |  |  |  |  |  |  |  |  |  |  |  |  |  |  |  |  |  |  |  |  | K12H4.3 |  |
|  |  |  |  |  |  |  |  |  |  |  |  |  |  |  |  |  |  |  |  |  |  |  |  |  |  |  |  |  |  |  |  |  |  |  |  |  |  | Y73E7A.2 |  |
|  |  |  |  |  |  |  |  |  |  |  |  |  |  |  |  |  |  |  |  |  |  |  |  |  |  |  |  |  |  |  |  |  |  |  |  |  |  | ZK430.7 |  |
|  |  |  |  |  |  |  |  |  |  |  |  |  |  |  |  |  |  |  |  |  |  |  |  |  |  |  |  |  |  |  |  |  |  |  |  |  |  | *lpd-7* | LiPid Depleted |
|  |  |  |  |  |  |  |  |  |  |  |  |  |  |  |  |  |  |  |  |  |  |  |  |  |  |  |  |  |  |  |  |  |  |  |  |  |  | *sars-1* | Seryl Amino-acyl tRNA Synthetase |
|  |  |  |  |  |  |  |  |  |  |  |  |  |  |  |  |  |  |  |  |  |  |  |  |  |  |  |  |  |  |  |  |  |  |  |  |  |  | ZK858.7 |  |
|  |  |  |  |  |  |  |  |  |  |  |  |  |  |  |  |  |  |  |  |  |  |  |  |  |  |  |  |  |  |  |  |  |  |  |  |  |  | *dnj-11* | DNaJ domain (prokaryotic heat shock protein) |
|  |  |  |  |  |  |  |  |  |  |  |  |  |  |  |  |  |  |  |  |  |  |  |  |  |  |  |  |  |  |  |  |  |  |  |  |  |  | *hlh-2* | Helix Loop Helix |
|  |  |  |  |  |  |  |  |  |  |  |  |  |  |  |  |  |  |  |  |  |  |  |  |  |  |  |  |  |  |  |  |  |  |  |  |  |  | *pop-1* | POsterior Pharynx defect |
|  |  |  |  |  |  |  |  |  |  |  |  |  |  |  |  |  |  |  |  |  |  |  |  |  |  |  |  |  |  |  |  |  |  |  |  |  |  | *sto-1* | STOmatin |
|  |  |  |  |  |  |  |  |  |  |  |  |  |  |  |  |  |  |  |  |  |  |  |  |  |  |  |  |  |  |  |  |  |  |  |  |  |  | C27F2.8 |  |
|  |  |  |  |  |  |  |  |  |  |  |  |  |  |  |  |  |  |  |  |  |  |  |  |  |  |  |  |  |  |  |  |  |  |  |  |  |  | T19A5.1 |  |
|  |  |  |  |  |  |  |  |  |  |  |  |  |  |  |  |  |  |  |  |  |  |  |  |  |  |  |  |  |  |  |  |  |  |  |  |  |  | R119.5 |  |
|  |  |  |  |  |  |  |  |  |  |  |  |  |  |  |  |  |  |  |  |  |  |  |  |  |  |  |  |  |  |  |  |  |  |  |  |  |  | K02E7.6 |  |
|  |  |  |  |  |  |  |  |  |  |  |  |  |  |  |  |  |  |  |  |  |  |  |  |  |  |  |  |  |  |  |  |  |  |  |  |  |  | *sams-4* | S-Adenosyl Methionine Synthetase |
|  |  |  |  |  |  |  |  |  |  |  |  |  |  |  |  |  |  |  |  |  |  |  |  |  |  |  |  |  |  |  |  |  |  |  |  |  |  | *unc-132* | UNCoordinated |
|  |  |  |  |  |  |  |  |  |  |  |  |  |  |  |  |  |  |  |  |  |  |  |  |  |  |  |  |  |  |  |  |  |  |  |  |  |  | *sams-3* | S-Adenosyl Methionine Synthetase |
|  |  |  |  |  |  |  |  |  |  |  |  |  |  |  |  |  |  |  |  |  |  |  |  |  |  |  |  |  |  |  |  |  |  |  |  |  |  | *nrde-3* | Nuclear RNAi DEfective |
|  |  |  |  |  |  |  |  |  |  |  |  |  |  |  |  |  |  |  |  |  |  |  |  |  |  |  |  |  |  |  |  |  |  |  |  |  |  | C43H8.1 |  |
|  |  |  |  |  |  |  |  |  |  |  |  |  |  |  |  |  |  |  |  |  |  |  |  |  |  |  |  |  |  |  |  |  |  |  |  |  |  | B0511.6 |  |
|  |  |  |  |  |  |  |  |  |  |  |  |  |  |  |  |  |  |  |  |  |  |  |  |  |  |  |  |  |  |  |  |  |  |  |  |  |  | *unc-84* | UNCoordinated |
|  |  |  |  |  |  |  |  |  |  |  |  |  |  |  |  |  |  |  |  |  |  |  |  |  |  |  |  |  |  |  |  |  |  |  |  |  |  | *ztf-11* | Zinc finger putative Transcription Factor family |
|  |  |  |  |  |  |  |  |  |  |  |  |  |  |  |  |  |  |  |  |  |  |  |  |  |  |  |  |  |  |  |  |  |  |  |  |  |  | *mrp-5* | Multidrug Resistance Protein family |
|  |  |  |  |  |  |  |  |  |  |  |  |  |  |  |  |  |  |  |  |  |  |  |  |  |  |  |  |  |  |  |  |  |  |  |  |  |  | *clec-140* | C-type LECtin |
|  |  |  |  |  |  |  |  |  |  |  |  |  |  |  |  |  |  |  |  |  |  |  |  |  |  |  |  |  |  |  |  |  |  |  |  |  |  | T04A6.5 |  |
|  |  |  |  |  |  |  |  |  |  |  |  |  |  |  |  |  |  |  |  |  |  |  |  |  |  |  |  |  |  |  |  |  |  |  |  |  |  | *hpr-17* | Homolog of S. Pombe Rad |
|  |  |  |  |  |  |  |  |  |  |  |  |  |  |  |  |  |  |  |  |  |  |  |  |  |  |  |  |  |  |  |  |  |  |  |  |  |  | C44B7.12 |  |
|  |  |  |  |  |  |  |  |  |  |  |  |  |  |  |  |  |  |  |  |  |  |  |  |  |  |  |  |  |  |  |  |  |  |  |  |  |  | *mut-2* | MUTator |
|  |  |  |  |  |  |  |  |  |  |  |  |  |  |  |  |  |  |  |  |  |  |  |  |  |  |  |  |  |  |  |  |  |  |  |  |  |  | T07C12.12 |  |
|  |  |  |  |  |  |  |  |  |  |  |  |  |  |  |  |  |  |  |  |  |  |  |  |  |  |  |  |  |  |  |  |  |  |  |  |  |  | *taf-6.1* | TAF (TBP-associated transcription factor) family |
|  |  |  |  |  |  |  |  |  |  |  |  |  |  |  |  |  |  |  |  |  |  |  |  |  |  |  |  |  |  |  |  |  |  |  |  |  |  | W05H9.3 |  |
|  |  |  |  |  |  |  |  |  |  |  |  |  |  |  |  |  |  |  |  |  |  |  |  |  |  |  |  |  |  |  |  |  |  |  |  |  |  | C41H7.5 |  |
|  |  |  |  |  |  |  |  |  |  |  |  |  |  |  |  |  |  |  |  |  |  |  |  |  |  |  |  |  |  |  |  |  |  |  |  |  |  | *alh-7* | ALdehyde deHydrogenase |
|  |  |  |  |  |  |  |  |  |  |  |  |  |  |  |  |  |  |  |  |  |  |  |  |  |  |  |  |  |  |  |  |  |  |  |  |  |  | ZC239.1 |  |
|  |  |  |  |  |  |  |  |  |  |  |  |  |  |  |  |  |  |  |  |  |  |  |  |  |  |  |  |  |  |  |  |  |  |  |  |  |  | Y55F3AM.6 |  |
|  |  |  |  |  |  |  |  |  |  |  |  |  |  |  |  |  |  |  |  |  |  |  |  |  |  |  |  |  |  |  |  |  |  |  |  |  |  | Y48G1C.8 |  |
|  |  |  |  |  |  |  |  |  |  |  |  |  |  |  |  |  |  |  |  |  |  |  |  |  |  |  |  |  |  |  |  |  |  |  |  |  |  | T22H9.1 |  |
|  |  |  |  |  |  |  |  |  |  |  |  |  |  |  |  |  |  |  |  |  |  |  |  |  |  |  |  |  |  |  |  |  |  |  |  |  |  | *eme-1* | Essential Meiotic Endonuclease |
|  |  |  |  |  |  |  |  |  |  |  |  |  |  |  |  |  |  |  |  |  |  |  |  |  |  |  |  |  |  |  |  |  |  |  |  |  |  | C50F2.2 |  |
|  |  |  |  |  |  |  |  |  |  |  |  |  |  |  |  |  |  |  |  |  |  |  |  |  |  |  |  |  |  |  |  |  |  |  |  |  |  | *wapl-1* | WAPL (Drosophila Wings APart-Like cohesin interactor) |
|  |  |  |  |  |  |  |  |  |  |  |  |  |  |  |  |  |  |  |  |  |  |  |  |  |  |  |  |  |  |  |  |  |  |  |  |  |  | *acin-1* | ACINus (mammalian Apoptotic Chromatin condensation Inducer in the Nucleus) homolog |
|  |  |  |  |  |  |  |  |  |  |  |  |  |  |  |  |  |  |  |  |  |  |  |  |  |  |  |  |  |  |  |  |  |  |  |  |  |  | *mog-4* | Masculinisation Of Germline |
|  |  |  |  |  |  |  |  |  |  |  |  |  |  |  |  |  |  |  |  |  |  |  |  |  |  |  |  |  |  |  |  |  |  |  |  |  |  | *lin-40* | abnormal cell LINeage |
|  |  |  |  |  |  |  |  |  |  |  |  |  |  |  |  |  |  |  |  |  |  |  |  |  |  |  |  |  |  |  |  |  |  |  |  |  |  | M4.1 |  |
|  |  |  |  |  |  |  |  |  |  |  |  |  |  |  |  |  |  |  |  |  |  |  |  |  |  |  |  |  |  |  |  |  |  |  |  |  |  | *rga-4* | Rho GTPase Activating protein |
|  |  |  |  |  |  |  |  |  |  |  |  |  |  |  |  |  |  |  |  |  |  |  |  |  |  |  |  |  |  |  |  |  |  |  |  |  |  | *syp-3* | SYnaPsis in meiosis abnormal |
|  |  |  |  |  |  |  |  |  |  |  |  |  |  |  |  |  |  |  |  |  |  |  |  |  |  |  |  |  |  |  |  |  |  |  |  |  |  | *dvc-1* | DNA damage-associated VCP/p97 Cofactor homolog |
|  |  |  |  |  |  |  |  |  |  |  |  |  |  |  |  |  |  |  |  |  |  |  |  |  |  |  |  |  |  |  |  |  |  |  |  |  |  | *psf-2* | Polypyrimidine-tract-binding protein (PTB)-associated-Splicing Factor homolog |
|  |  |  |  |  |  |  |  |  |  |  |  |  |  |  |  |  |  |  |  |  |  |  |  |  |  |  |  |  |  |  |  |  |  |  |  |  |  | C08B11.9 |  |
|  |  |  |  |  |  |  |  |  |  |  |  |  |  |  |  |  |  |  |  |  |  |  |  |  |  |  |  |  |  |  |  |  |  |  |  |  |  | *psf-1* | Polypyrimidine-tract-binding protein (PTB)-associated-Splicing Factor homolog |
|  |  |  |  |  |  |  |  |  |  |  |  |  |  |  |  |  |  |  |  |  |  |  |  |  |  |  |  |  |  |  |  |  |  |  |  |  |  | F54E7.9 |  |
|  |  |  |  |  |  |  |  |  |  |  |  |  |  |  |  |  |  |  |  |  |  |  |  |  |  |  |  |  |  |  |  |  |  |  |  |  |  | *psf-3* | Polypyrimidine-tract-binding protein (PTB)-associated-Splicing Factor homolog |
|  |  |  |  |  |  |  |  |  |  |  |  |  |  |  |  |  |  |  |  |  |  |  |  |  |  |  |  |  |  |  |  |  |  |  |  |  |  | *kbp-4* | KNL (kinetochore null) Binding Protein |
|  |  |  |  |  |  |  |  |  |  |  |  |  |  |  |  |  |  |  |  |  |  |  |  |  |  |  |  |  |  |  |  |  |  |  |  |  |  | F35G12.11 |  |
|  |  |  |  |  |  |  |  |  |  |  |  |  |  |  |  |  |  |  |  |  |  |  |  |  |  |  |  |  |  |  |  |  |  |  |  |  |  | *kbp-3* | KNL (kinetochore null) Binding Protein |
|  |  |  |  |  |  |  |  |  |  |  |  |  |  |  |  |  |  |  |  |  |  |  |  |  |  |  |  |  |  |  |  |  |  |  |  |  |  | *ung-1* | Uracil DNA N-Glycosylase |
|  |  |  |  |  |  |  |  |  |  |  |  |  |  |  |  |  |  |  |  |  |  |  |  |  |  |  |  |  |  |  |  |  |  |  |  |  |  | *homt-1* | Hydroxyindole-O-MethylTransferase homolog |
|  |  |  |  |  |  |  |  |  |  |  |  |  |  |  |  |  |  |  |  |  |  |  |  |  |  |  |  |  |  |  |  |  |  |  |  |  |  | *lnp-1* | LuNaPark (membrane protein) homolog |
|  |  |  |  |  |  |  |  |  |  |  |  |  |  |  |  |  |  |  |  |  |  |  |  |  |  |  |  |  |  |  |  |  |  |  |  |  |  | *sgo-1* | ShuGOshin (yeast chromosome segregation protein) homolog |
|  |  |  |  |  |  |  |  |  |  |  |  |  |  |  |  |  |  |  |  |  |  |  |  |  |  |  |  |  |  |  |  |  |  |  |  |  |  | T04H1.5 |  |
|  |  |  |  |  |  |  |  |  |  |  |  |  |  |  |  |  |  |  |  |  |  |  |  |  |  |  |  |  |  |  |  |  |  |  |  |  |  | *mes-6* | Maternal Effect Sterile |
|  |  |  |  |  |  |  |  |  |  |  |  |  |  |  |  |  |  |  |  |  |  |  |  |  |  |  |  |  |  |  |  |  |  |  |  |  |  | *cks-1* | Cyclin-dependent protein Kinase (CDC28) regulatory Subunit |
|  |  |  |  |  |  |  |  |  |  |  |  |  |  |  |  |  |  |  |  |  |  |  |  |  |  |  |  |  |  |  |  |  |  |  |  |  |  | *kbp-2* | KNL (kinetochore null) Binding Protein |
|  |  |  |  |  |  |  |  |  |  |  |  |  |  |  |  |  |  |  |  |  |  |  |  |  |  |  |  |  |  |  |  |  |  |  |  |  |  | C35D10.13 |  |
|  |  |  |  |  |  |  |  |  |  |  |  |  |  |  |  |  |  |  |  |  |  |  |  |  |  |  |  |  |  |  |  |  |  |  |  |  |  | Y75B8A.18 |  |
|  |  |  |  |  |  |  |  |  |  |  |  |  |  |  |  |  |  |  |  |  |  |  |  |  |  |  |  |  |  |  |  |  |  |  |  |  |  | *ska-3* | Spindle and Kinetochore-Associated protein homolog |
|  |  |  |  |  |  |  |  |  |  |  |  |  |  |  |  |  |  |  |  |  |  |  |  |  |  |  |  |  |  |  |  |  |  |  |  |  |  | C09G4.4 |  |
|  |  |  |  |  |  |  |  |  |  |  |  |  |  |  |  |  |  |  |  |  |  |  |  |  |  |  |  |  |  |  |  |  |  |  |  |  |  | *zip-7* | bZIP transcription factor family |
|  |  |  |  |  |  |  |  |  |  |  |  |  |  |  |  |  |  |  |  |  |  |  |  |  |  |  |  |  |  |  |  |  |  |  |  |  |  | T19H12.2 |  |
|  |  |  |  |  |  |  |  |  |  |  |  |  |  |  |  |  |  |  |  |  |  |  |  |  |  |  |  |  |  |  |  |  |  |  |  |  |  | *kbp-1* | KNL (kinetochore null) Binding Protein |
|  |  |  |  |  |  |  |  |  |  |  |  |  |  |  |  |  |  |  |  |  |  |  |  |  |  |  |  |  |  |  |  |  |  |  |  |  |  | Y39B6A.40 |  |
|  |  |  |  |  |  |  |  |  |  |  |  |  |  |  |  |  |  |  |  |  |  |  |  |  |  |  |  |  |  |  |  |  |  |  |  |  |  | Y110A7A.4 |  |
|  |  |  |  |  |  |  |  |  |  |  |  |  |  |  |  |  |  |  |  |  |  |  |  |  |  |  |  |  |  |  |  |  |  |  |  |  |  | T22C1.4 |  |
|  |  |  |  |  |  |  |  |  |  |  |  |  |  |  |  |  |  |  |  |  |  |  |  |  |  |  |  |  |  |  |  |  |  |  |  |  |  | JC8.4 |  |
|  |  |  |  |  |  |  |  |  |  |  |  |  |  |  |  |  |  |  |  |  |  |  |  |  |  |  |  |  |  |  |  |  |  |  |  |  |  | *csc-1* | Chromosome Segregation and Cytokinesis defective |
|  |  |  |  |  |  |  |  |  |  |  |  |  |  |  |  |  |  |  |  |  |  |  |  |  |  |  |  |  |  |  |  |  |  |  |  |  |  | *mel-47* | Maternal Effect Lethal |
|  |  |  |  |  |  |  |  |  |  |  |  |  |  |  |  |  |  |  |  |  |  |  |  |  |  |  |  |  |  |  |  |  |  |  |  |  |  | *pcn-1* | PCNA (Proliferating Cell Nuclear antigen) homolog |
|  |  |  |  |  |  |  |  |  |  |  |  |  |  |  |  |  |  |  |  |  |  |  |  |  |  |  |  |  |  |  |  |  |  |  |  |  |  | *his-24* | HIStone |
|  |  |  |  |  |  |  |  |  |  |  |  |  |  |  |  |  |  |  |  |  |  |  |  |  |  |  |  |  |  |  |  |  |  |  |  |  |  | Y53F4B.3 |  |
|  |  |  |  |  |  |  |  |  |  |  |  |  |  |  |  |  |  |  |  |  |  |  |  |  |  |  |  |  |  |  |  |  |  |  |  |  |  | *knl-3* | Kinetochore NuLl |
|  |  |  |  |  |  |  |  |  |  |  |  |  |  |  |  |  |  |  |  |  |  |  |  |  |  |  |  |  |  |  |  |  |  |  |  |  |  | *unc-85* | UNCoordinated |
|  |  |  |  |  |  |  |  |  |  |  |  |  |  |  |  |  |  |  |  |  |  |  |  |  |  |  |  |  |  |  |  |  |  |  |  |  |  | *bub-3* | yeast BUB homolog |
|  |  |  |  |  |  |  |  |  |  |  |  |  |  |  |  |  |  |  |  |  |  |  |  |  |  |  |  |  |  |  |  |  |  |  |  |  |  | *sap-49* | Spliceosome-Associated Protein |
|  |  |  |  |  |  |  |  |  |  |  |  |  |  |  |  |  |  |  |  |  |  |  |  |  |  |  |  |  |  |  |  |  |  |  |  |  |  | F33H2.3 |  |
|  |  |  |  |  |  |  |  |  |  |  |  |  |  |  |  |  |  |  |  |  |  |  |  |  |  |  |  |  |  |  |  |  |  |  |  |  |  | *orc-1* | ORC (Origin Recognition Complex) subunit |
|  |  |  |  |  |  |  |  |  |  |  |  |  |  |  |  |  |  |  |  |  |  |  |  |  |  |  |  |  |  |  |  |  |  |  |  |  |  | *orc-4* | ORC (Origin Recognition Complex) subunit |
|  |  |  |  |  |  |  |  |  |  |  |  |  |  |  |  |  |  |  |  |  |  |  |  |  |  |  |  |  |  |  |  |  |  |  |  |  |  | *san-1* | Suspended ANimation (anoxia-induced) defective |
|  |  |  |  |  |  |  |  |  |  |  |  |  |  |  |  |  |  |  |  |  |  |  |  |  |  |  |  |  |  |  |  |  |  |  |  |  |  | F54D5.5 |  |
|  |  |  |  |  |  |  |  |  |  |  |  |  |  |  |  |  |  |  |  |  |  |  |  |  |  |  |  |  |  |  |  |  |  |  |  |  |  | *mcm-5* | yeast MCM (licensing factor) related |
|  |  |  |  |  |  |  |  |  |  |  |  |  |  |  |  |  |  |  |  |  |  |  |  |  |  |  |  |  |  |  |  |  |  |  |  |  |  | *brc-1* | BRCa homolog (tumor suppressor gene Brca1) |
|  |  |  |  |  |  |  |  |  |  |  |  |  |  |  |  |  |  |  |  |  |  |  |  |  |  |  |  |  |  |  |  |  |  |  |  |  |  | F12F6.7 |  |
|  |  |  |  |  |  |  |  |  |  |  |  |  |  |  |  |  |  |  |  |  |  |  |  |  |  |  |  |  |  |  |  |  |  |  |  |  |  | *sas-6* | Spindle ASsembly abnormal |
|  |  |  |  |  |  |  |  |  |  |  |  |  |  |  |  |  |  |  |  |  |  |  |  |  |  |  |  |  |  |  |  |  |  |  |  |  |  | *slx-1* | yeast SLX (Synthetic Lethal X) homolog |
|  |  |  |  |  |  |  |  |  |  |  |  |  |  |  |  |  |  |  |  |  |  |  |  |  |  |  |  |  |  |  |  |  |  |  |  |  |  | *gpr-1* | G Protein Regulator |
|  |  |  |  |  |  |  |  |  |  |  |  |  |  |  |  |  |  |  |  |  |  |  |  |  |  |  |  |  |  |  |  |  |  |  |  |  |  | *brd-1* | BaRD homolog (tumor suppressor gene Bard1) |
|  |  |  |  |  |  |  |  |  |  |  |  |  |  |  |  |  |  |  |  |  |  |  |  |  |  |  |  |  |  |  |  |  |  |  |  |  |  | *lin-54* | abnormal cell LINeage |
|  |  |  |  |  |  |  |  |  |  |  |  |  |  |  |  |  |  |  |  |  |  |  |  |  |  |  |  |  |  |  |  |  |  |  |  |  |  | T25E12.6 |  |
|  |  |  |  |  |  |  |  |  |  |  |  |  |  |  |  |  |  |  |  |  |  |  |  |  |  |  |  |  |  |  |  |  |  |  |  |  |  | *ska-1* | Spindle and Kinetochore-Associated protein homolog |
|  |  |  |  |  |  |  |  |  |  |  |  |  |  |  |  |  |  |  |  |  |  |  |  |  |  |  |  |  |  |  |  |  |  |  |  |  |  | *spn-4* | SPiNdle orientation defective |
|  |  |  |  |  |  |  |  |  |  |  |  |  |  |  |  |  |  |  |  |  |  |  |  |  |  |  |  |  |  |  |  |  |  |  |  |  |  | *kbp-5* | KNL (kinetochore null) Binding Protein |
|  |  |  |  |  |  |  |  |  |  |  |  |  |  |  |  |  |  |  |  |  |  |  |  |  |  |  |  |  |  |  |  |  |  |  |  |  |  | F10E9.7 |  |
|  |  |  |  |  |  |  |  |  |  |  |  |  |  |  |  |  |  |  |  |  |  |  |  |  |  |  |  |  |  |  |  |  |  |  |  |  |  | *ceh-16* | C. Elegans Homeobox |
|  |  |  |  |  |  |  |  |  |  |  |  |  |  |  |  |  |  |  |  |  |  |  |  |  |  |  |  |  |  |  |  |  |  |  |  |  |  | Y71H2AM.11 |  |
|  |  |  |  |  |  |  |  |  |  |  |  |  |  |  |  |  |  |  |  |  |  |  |  |  |  |  |  |  |  |  |  |  |  |  |  |  |  | Y105C5B.20 |  |
|  |  |  |  |  |  |  |  |  |  |  |  |  |  |  |  |  |  |  |  |  |  |  |  |  |  |  |  |  |  |  |  |  |  |  |  |  |  | *hil-6* | HIstone H1 Like |
|  |  |  |  |  |  |  |  |  |  |  |  |  |  |  |  |  |  |  |  |  |  |  |  |  |  |  |  |  |  |  |  |  |  |  |  |  |  | F42G9.1 |  |
|  |  |  |  |  |  |  |  |  |  |  |  |  |  |  |  |  |  |  |  |  |  |  |  |  |  |  |  |  |  |  |  |  |  |  |  |  |  | *unc-61* | UNCoordinated |
|  |  |  |  |  |  |  |  |  |  |  |  |  |  |  |  |  |  |  |  |  |  |  |  |  |  |  |  |  |  |  |  |  |  |  |  |  |  | *sqd-1* | homologous to Drosophila SQD (squid) protein |
|  |  |  |  |  |  |  |  |  |  |  |  |  |  |  |  |  |  |  |  |  |  |  |  |  |  |  |  |  |  |  |  |  |  |  |  |  |  | *npp-3* | Nuclear Pore complex Protein |
|  |  |  |  |  |  |  |  |  |  |  |  |  |  |  |  |  |  |  |  |  |  |  |  |  |  |  |  |  |  |  |  |  |  |  |  |  |  | *etf-1* | Eukaryotic Translational release Factor (RF1) |
|  |  |  |  |  |  |  |  |  |  |  |  |  |  |  |  |  |  |  |  |  |  |  |  |  |  |  |  |  |  |  |  |  |  |  |  |  |  | *tag-52* | Temporarily Assigned Gene name |
|  |  |  |  |  |  |  |  |  |  |  |  |  |  |  |  |  |  |  |  |  |  |  |  |  |  |  |  |  |  |  |  |  |  |  |  |  |  | *cyd-1* | CYclin D |
|  |  |  |  |  |  |  |  |  |  |  |  |  |  |  |  |  |  |  |  |  |  |  |  |  |  |  |  |  |  |  |  |  |  |  |  |  |  | *rnr-1* | RiboNucleotide Reductase |
|  |  |  |  |  |  |  |  |  |  |  |  |  |  |  |  |  |  |  |  |  |  |  |  |  |  |  |  |  |  |  |  |  |  |  |  |  |  | C24H12.7 |  |
|  |  |  |  |  |  |  |  |  |  |  |  |  |  |  |  |  |  |  |  |  |  |  |  |  |  |  |  |  |  |  |  |  |  |  |  |  |  | F48E3.4 |  |
|  |  |  |  |  |  |  |  |  |  |  |  |  |  |  |  |  |  |  |  |  |  |  |  |  |  |  |  |  |  |  |  |  |  |  |  |  |  | C08H9.16 |  |
|  |  |  |  |  |  |  |  |  |  |  |  |  |  |  |  |  |  |  |  |  |  |  |  |  |  |  |  |  |  |  |  |  |  |  |  |  |  | R05D3.8 |  |
|  |  |  |  |  |  |  |  |  |  |  |  |  |  |  |  |  |  |  |  |  |  |  |  |  |  |  |  |  |  |  |  |  |  |  |  |  |  | C23H4.6 |  |
|  |  |  |  |  |  |  |  |  |  |  |  |  |  |  |  |  |  |  |  |  |  |  |  |  |  |  |  |  |  |  |  |  |  |  |  |  |  | *psmd-9* | ProteaSoMe regulatory D subunit |
|  |  |  |  |  |  |  |  |  |  |  |  |  |  |  |  |  |  |  |  |  |  |  |  |  |  |  |  |  |  |  |  |  |  |  |  |  |  | T26H2.10 |  |
|  |  |  |  |  |  |  |  |  |  |  |  |  |  |  |  |  |  |  |  |  |  |  |  |  |  |  |  |  |  |  |  |  |  |  |  |  |  | Y47G6A.31 |  |
|  |  |  |  |  |  |  |  |  |  |  |  |  |  |  |  |  |  |  |  |  |  |  |  |  |  |  |  |  |  |  |  |  |  |  |  |  |  | Y54G11A.2 |  |
|  |  |  |  |  |  |  |  |  |  |  |  |  |  |  |  |  |  |  |  |  |  |  |  |  |  |  |  |  |  |  |  |  |  |  |  |  |  | F56A8.5 |  |
|  |  |  |  |  |  |  |  |  |  |  |  |  |  |  |  |  |  |  |  |  |  |  |  |  |  |  |  |  |  |  |  |  |  |  |  |  |  | *mecr-1* | Mitochondrial trans-2-Enoyl-CoA Reductase |
|  |  |  |  |  |  |  |  |  |  |  |  |  |  |  |  |  |  |  |  |  |  |  |  |  |  |  |  |  |  |  |  |  |  |  |  |  |  | *rsr-1* | SR protein related |
|  |  |  |  |  |  |  |  |  |  |  |  |  |  |  |  |  |  |  |  |  |  |  |  |  |  |  |  |  |  |  |  |  |  |  |  |  |  | Y52B11C.1 |  |
|  |  |  |  |  |  |  |  |  |  |  |  |  |  |  |  |  |  |  |  |  |  |  |  |  |  |  |  |  |  |  |  |  |  |  |  |  |  | F28D9.4 |  |
|  |  |  |  |  |  |  |  |  |  |  |  |  |  |  |  |  |  |  |  |  |  |  |  |  |  |  |  |  |  |  |  |  |  |  |  |  |  | T18D3.1 |  |
|  |  |  |  |  |  |  |  |  |  |  |  |  |  |  |  |  |  |  |  |  |  |  |  |  |  |  |  |  |  |  |  |  |  |  |  |  |  | ZK1053.4 |  |
|  |  |  |  |  |  |  |  |  |  |  |  |  |  |  |  |  |  |  |  |  |  |  |  |  |  |  |  |  |  |  |  |  |  |  |  |  |  | F09C8.2 |  |
|  |  |  |  |  |  |  |  |  |  |  |  |  |  |  |  |  |  |  |  |  |  |  |  |  |  |  |  |  |  |  |  |  |  |  |  |  |  | C07D10.5 |  |
|  |  |  |  |  |  |  |  |  |  |  |  |  |  |  |  |  |  |  |  |  |  |  |  |  |  |  |  |  |  |  |  |  |  |  |  |  |  | *pole-2* | POLE (DNA POLymerase Epsilon) homolog |
|  |  |  |  |  |  |  |  |  |  |  |  |  |  |  |  |  |  |  |  |  |  |  |  |  |  |  |  |  |  |  |  |  |  |  |  |  |  | Y48G1C.7 |  |
|  |  |  |  |  |  |  |  |  |  |  |  |  |  |  |  |  |  |  |  |  |  |  |  |  |  |  |  |  |  |  |  |  |  |  |  |  |  | *zyg-1* | ZYGote defective : embryonic lethal |
|  |  |  |  |  |  |  |  |  |  |  |  |  |  |  |  |  |  |  |  |  |  |  |  |  |  |  |  |  |  |  |  |  |  |  |  |  |  | *chaf-1* | CHromatin Assembly Factor |
|  |  |  |  |  |  |  |  |  |  |  |  |  |  |  |  |  |  |  |  |  |  |  |  |  |  |  |  |  |  |  |  |  |  |  |  |  |  | B0432.13 |  |
|  |  |  |  |  |  |  |  |  |  |  |  |  |  |  |  |  |  |  |  |  |  |  |  |  |  |  |  |  |  |  |  |  |  |  |  |  |  | *toe-2* | Target Of ERK kinase MPK-1 |
|  |  |  |  |  |  |  |  |  |  |  |  |  |  |  |  |  |  |  |  |  |  |  |  |  |  |  |  |  |  |  |  |  |  |  |  |  |  | *knl-1* | Kinetochore NuLl |
|  |  |  |  |  |  |  |  |  |  |  |  |  |  |  |  |  |  |  |  |  |  |  |  |  |  |  |  |  |  |  |  |  |  |  |  |  |  | *klp-18* | Kinesin-Like Protein |
|  |  |  |  |  |  |  |  |  |  |  |  |  |  |  |  |  |  |  |  |  |  |  |  |  |  |  |  |  |  |  |  |  |  |  |  |  |  | *lig-1* | LIGase |
|  |  |  |  |  |  |  |  |  |  |  |  |  |  |  |  |  |  |  |  |  |  |  |  |  |  |  |  |  |  |  |  |  |  |  |  |  |  | *cye-1* | CYclin E |
|  |  |  |  |  |  |  |  |  |  |  |  |  |  |  |  |  |  |  |  |  |  |  |  |  |  |  |  |  |  |  |  |  |  |  |  |  |  | F26F4.8 |  |
|  |  |  |  |  |  |  |  |  |  |  |  |  |  |  |  |  |  |  |  |  |  |  |  |  |  |  |  |  |  |  |  |  |  |  |  |  |  | *phf-5* | PHd Finger family |
|  |  |  |  |  |  |  |  |  |  |  |  |  |  |  |  |  |  |  |  |  |  |  |  |  |  |  |  |  |  |  |  |  |  |  |  |  |  | F11E6.7 |  |
|  |  |  |  |  |  |  |  |  |  |  |  |  |  |  |  |  |  |  |  |  |  |  |  |  |  |  |  |  |  |  |  |  |  |  |  |  |  | F17C11.10 |  |
|  |  |  |  |  |  |  |  |  |  |  |  |  |  |  |  |  |  |  |  |  |  |  |  |  |  |  |  |  |  |  |  |  |  |  |  |  |  | Y82E9BR.1 |  |
|  |  |  |  |  |  |  |  |  |  |  |  |  |  |  |  |  |  |  |  |  |  |  |  |  |  |  |  |  |  |  |  |  |  |  |  |  |  | *cdt-1* | CDT (S. pombe CDC10 Dependent Transcript) homolog |
|  |  |  |  |  |  |  |  |  |  |  |  |  |  |  |  |  |  |  |  |  |  |  |  |  |  |  |  |  |  |  |  |  |  |  |  |  |  | F53H1.4 |  |
|  |  |  |  |  |  |  |  |  |  |  |  |  |  |  |  |  |  |  |  |  |  |  |  |  |  |  |  |  |  |  |  |  |  |  |  |  |  | *npp-22* | Nuclear Pore complex Protein |
|  |  |  |  |  |  |  |  |  |  |  |  |  |  |  |  |  |  |  |  |  |  |  |  |  |  |  |  |  |  |  |  |  |  |  |  |  |  | *dsh-2* | DiSHevelled related |
|  |  |  |  |  |  |  |  |  |  |  |  |  |  |  |  |  |  |  |  |  |  |  |  |  |  |  |  |  |  |  |  |  |  |  |  |  |  | Y47D3A.29 |  |
|  |  |  |  |  |  |  |  |  |  |  |  |  |  |  |  |  |  |  |  |  |  |  |  |  |  |  |  |  |  |  |  |  |  |  |  |  |  | *spd-2* | SPindle Defective |
|  |  |  |  |  |  |  |  |  |  |  |  |  |  |  |  |  |  |  |  |  |  |  |  |  |  |  |  |  |  |  |  |  |  |  |  |  |  | F56D2.2 |  |
|  |  |  |  |  |  |  |  |  |  |  |  |  |  |  |  |  |  |  |  |  |  |  |  |  |  |  |  |  |  |  |  |  |  |  |  |  |  | *polk-1* | POLK (DNA polymerase kappa) homolog |
|  |  |  |  |  |  |  |  |  |  |  |  |  |  |  |  |  |  |  |  |  |  |  |  |  |  |  |  |  |  |  |  |  |  |  |  |  |  | *ect-2* | ECT2 (mammalian Rho GEF) homolog |
|  |  |  |  |  |  |  |  |  |  |  |  |  |  |  |  |  |  |  |  |  |  |  |  |  |  |  |  |  |  |  |  |  |  |  |  |  |  | B0393.3 |  |
|  |  |  |  |  |  |  |  |  |  |  |  |  |  |  |  |  |  |  |  |  |  |  |  |  |  |  |  |  |  |  |  |  |  |  |  |  |  | *dna-2* | yeast DNA helicase/endonuclease family |
|  |  |  |  |  |  |  |  |  |  |  |  |  |  |  |  |  |  |  |  |  |  |  |  |  |  |  |  |  |  |  |  |  |  |  |  |  |  | *cdt-2* | CDT (S. pombe CDC10 Dependent Transcript) homolog |
|  |  |  |  |  |  |  |  |  |  |  |  |  |  |  |  |  |  |  |  |  |  |  |  |  |  |  |  |  |  |  |  |  |  |  |  |  |  | *top-2* | TOPoisomerase |
|  |  |  |  |  |  |  |  |  |  |  |  |  |  |  |  |  |  |  |  |  |  |  |  |  |  |  |  |  |  |  |  |  |  |  |  |  |  | *plk-1* | POLO Kinase |
|  |  |  |  |  |  |  |  |  |  |  |  |  |  |  |  |  |  |  |  |  |  |  |  |  |  |  |  |  |  |  |  |  |  |  |  |  |  | W02B8.2 |  |
|  |  |  |  |  |  |  |  |  |  |  |  |  |  |  |  |  |  |  |  |  |  |  |  |  |  |  |  |  |  |  |  |  |  |  |  |  |  | *cyk-4* | CYtoKinesis defect |
|  |  |  |  |  |  |  |  |  |  |  |  |  |  |  |  |  |  |  |  |  |  |  |  |  |  |  |  |  |  |  |  |  |  |  |  |  |  | *hcp-2* | HoloCentric chromosome binding Protein |
|  |  |  |  |  |  |  |  |  |  |  |  |  |  |  |  |  |  |  |  |  |  |  |  |  |  |  |  |  |  |  |  |  |  |  |  |  |  | F59A6.5 |  |
|  |  |  |  |  |  |  |  |  |  |  |  |  |  |  |  |  |  |  |  |  |  |  |  |  |  |  |  |  |  |  |  |  |  |  |  |  |  | *smc-4* | SMC (structural maintenance of chromosomes) family |
|  |  |  |  |  |  |  |  |  |  |  |  |  |  |  |  |  |  |  |  |  |  |  |  |  |  |  |  |  |  |  |  |  |  |  |  |  |  | *knl-2* | Kinetochore NuLl |
|  |  |  |  |  |  |  |  |  |  |  |  |  |  |  |  |  |  |  |  |  |  |  |  |  |  |  |  |  |  |  |  |  |  |  |  |  |  | *ndc-80* | Yeast NDC (nuclear division cycle) homolog |
|  |  |  |  |  |  |  |  |  |  |  |  |  |  |  |  |  |  |  |  |  |  |  |  |  |  |  |  |  |  |  |  |  |  |  |  |  |  | *bub-1* | yeast BUB homolog |
|  |  |  |  |  |  |  |  |  |  |  |  |  |  |  |  |  |  |  |  |  |  |  |  |  |  |  |  |  |  |  |  |  |  |  |  |  |  | *zen-4* | Zygotic epidermal ENclosure defective |
|  |  |  |  |  |  |  |  |  |  |  |  |  |  |  |  |  |  |  |  |  |  |  |  |  |  |  |  |  |  |  |  |  |  |  |  |  |  | *hcp-4* | HoloCentric chromosome binding Protein |
|  |  |  |  |  |  |  |  |  |  |  |  |  |  |  |  |  |  |  |  |  |  |  |  |  |  |  |  |  |  |  |  |  |  |  |  |  |  | *bmk-1* | BiMC related Kinase |
|  |  |  |  |  |  |  |  |  |  |  |  |  |  |  |  |  |  |  |  |  |  |  |  |  |  |  |  |  |  |  |  |  |  |  |  |  |  | *aspm-1* | mammalian ASPM (Abnormal SPindles & primary Microcephaly) homolog |
|  |  |  |  |  |  |  |  |  |  |  |  |  |  |  |  |  |  |  |  |  |  |  |  |  |  |  |  |  |  |  |  |  |  |  |  |  |  | *hcp-1* | HoloCentric chromosome binding Protein |
|  |  |  |  |  |  |  |  |  |  |  |  |  |  |  |  |  |  |  |  |  |  |  |  |  |  |  |  |  |  |  |  |  |  |  |  |  |  | W04A8.1 |  |
|  |  |  |  |  |  |  |  |  |  |  |  |  |  |  |  |  |  |  |  |  |  |  |  |  |  |  |  |  |  |  |  |  |  |  |  |  |  | *ani-1* | ANIllin (actin binding protein) |
|  |  |  |  |  |  |  |  |  |  |  |  |  |  |  |  |  |  |  |  |  |  |  |  |  |  |  |  |  |  |  |  |  |  |  |  |  |  | *klp-19* | Kinesin-Like Protein |
|  |  |  |  |  |  |  |  |  |  |  |  |  |  |  |  |  |  |  |  |  |  |  |  |  |  |  |  |  |  |  |  |  |  |  |  |  |  | *pig-1* | Par-1 (I)-like Gene |
|  |  |  |  |  |  |  |  |  |  |  |  |  |  |  |  |  |  |  |  |  |  |  |  |  |  |  |  |  |  |  |  |  |  |  |  |  |  | F33H2.5 |  |
|  |  |  |  |  |  |  |  |  |  |  |  |  |  |  |  |  |  |  |  |  |  |  |  |  |  |  |  |  |  |  |  |  |  |  |  |  |  | *wee-1.3* | WEE homolog |
|  |  |  |  |  |  |  |  |  |  |  |  |  |  |  |  |  |  |  |  |  |  |  |  |  |  |  |  |  |  |  |  |  |  |  |  |  |  | *mcm-7* | yeast MCM (licensing factor) related |
|  |  |  |  |  |  |  |  |  |  |  |  |  |  |  |  |  |  |  |  |  |  |  |  |  |  |  |  |  |  |  |  |  |  |  |  |  |  | *mcm-4* | yeast MCM (licensing factor) related |
|  |  |  |  |  |  |  |  |  |  |  |  |  |  |  |  |  |  |  |  |  |  |  |  |  |  |  |  |  |  |  |  |  |  |  |  |  |  | *rsa-2* | Regulator of Spindle Assembly |
|  |  |  |  |  |  |  |  |  |  |  |  |  |  |  |  |  |  |  |  |  |  |  |  |  |  |  |  |  |  |  |  |  |  |  |  |  |  | *icp-1* | InCenP homolog |
|  |  |  |  |  |  |  |  |  |  |  |  |  |  |  |  |  |  |  |  |  |  |  |  |  |  |  |  |  |  |  |  |  |  |  |  |  |  | Y53F4B.9 |  |
|  |  |  |  |  |  |  |  |  |  |  |  |  |  |  |  |  |  |  |  |  |  |  |  |  |  |  |  |  |  |  |  |  |  |  |  |  |  | *prp-31* | yeast PRP (splicing factor) related |
|  |  |  |  |  |  |  |  |  |  |  |  |  |  |  |  |  |  |  |  |  |  |  |  |  |  |  |  |  |  |  |  |  |  |  |  |  |  | *ppp-1* | PyroPhosPhorylase family |
|  |  |  |  |  |  |  |  |  |  |  |  |  |  |  |  |  |  |  |  |  |  |  |  |  |  |  |  |  |  |  |  |  |  |  |  |  |  | *tbg-1* | TuBulin, Gamma |
|  |  |  |  |  |  |  |  |  |  |  |  |  |  |  |  |  |  |  |  |  |  |  |  |  |  |  |  |  |  |  |  |  |  |  |  |  |  | T13H5.8 |  |
|  |  |  |  |  |  |  |  |  |  |  |  |  |  |  |  |  |  |  |  |  |  |  |  |  |  |  |  |  |  |  |  |  |  |  |  |  |  | *eftu-2* | Elongation Factor TU family |
|  |  |  |  |  |  |  |  |  |  |  |  |  |  |  |  |  |  |  |  |  |  |  |  |  |  |  |  |  |  |  |  |  |  |  |  |  |  | *mus-101* | MUS (Drosophila mutagen sensitive) related |
|  |  |  |  |  |  |  |  |  |  |  |  |  |  |  |  |  |  |  |  |  |  |  |  |  |  |  |  |  |  |  |  |  |  |  |  |  |  | C16A3.1 |  |
|  |  |  |  |  |  |  |  |  |  |  |  |  |  |  |  |  |  |  |  |  |  |  |  |  |  |  |  |  |  |  |  |  |  |  |  |  |  | *ears-1* | glutamyl(E) Amino-acyl tRNA Synthetase |
|  |  |  |  |  |  |  |  |  |  |  |  |  |  |  |  |  |  |  |  |  |  |  |  |  |  |  |  |  |  |  |  |  |  |  |  |  |  | C35E7.5 |  |
|  |  |  |  |  |  |  |  |  |  |  |  |  |  |  |  |  |  |  |  |  |  |  |  |  |  |  |  |  |  |  |  |  |  |  |  |  |  | F44F1.6 |  |
|  |  |  |  |  |  |  |  |  |  |  |  |  |  |  |  |  |  |  |  |  |  |  |  |  |  |  |  |  |  |  |  |  |  |  |  |  |  | *asc-1* | human Activating Signal Cointegrator homolog |
|  |  |  |  |  |  |  |  |  |  |  |  |  |  |  |  |  |  |  |  |  |  |  |  |  |  |  |  |  |  |  |  |  |  |  |  |  |  | C16A3.6 |  |
|  |  |  |  |  |  |  |  |  |  |  |  |  |  |  |  |  |  |  |  |  |  |  |  |  |  |  |  |  |  |  |  |  |  |  |  |  |  | *pqn-59* | Prion-like-(Q/N-rich)-domain-bearing protein |
|  |  |  |  |  |  |  |  |  |  |  |  |  |  |  |  |  |  |  |  |  |  |  |  |  |  |  |  |  |  |  |  |  |  |  |  |  |  | *nra-3* | Nicotinic Receptor Associated |
|  |  |  |  |  |  |  |  |  |  |  |  |  |  |  |  |  |  |  |  |  |  |  |  |  |  |  |  |  |  |  |  |  |  |  |  |  |  | *ced-11* | CEll Death abnormality |
|  |  |  |  |  |  |  |  |  |  |  |  |  |  |  |  |  |  |  |  |  |  |  |  |  |  |  |  |  |  |  |  |  |  |  |  |  |  | *mes-4* | Maternal Effect Sterile |
|  |  |  |  |  |  |  |  |  |  |  |  |  |  |  |  |  |  |  |  |  |  |  |  |  |  |  |  |  |  |  |  |  |  |  |  |  |  | *hcp-6* | HoloCentric chromosome binding Protein |
|  |  |  |  |  |  |  |  |  |  |  |  |  |  |  |  |  |  |  |  |  |  |  |  |  |  |  |  |  |  |  |  |  |  |  |  |  |  | ZK328.4 |  |
|  |  |  |  |  |  |  |  |  |  |  |  |  |  |  |  |  |  |  |  |  |  |  |  |  |  |  |  |  |  |  |  |  |  |  |  |  |  | *cya-1* | CYclin A |
|  |  |  |  |  |  |  |  |  |  |  |  |  |  |  |  |  |  |  |  |  |  |  |  |  |  |  |  |  |  |  |  |  |  |  |  |  |  | *sys-1* | SYmmetrical Sister cell hermaphrodite gonad defect |
|  |  |  |  |  |  |  |  |  |  |  |  |  |  |  |  |  |  |  |  |  |  |  |  |  |  |  |  |  |  |  |  |  |  |  |  |  |  | K08F4.1 |  |
|  |  |  |  |  |  |  |  |  |  |  |  |  |  |  |  |  |  |  |  |  |  |  |  |  |  |  |  |  |  |  |  |  |  |  |  |  |  | *efl-3* | E2F-like (mammalian transcription factor) |
|  |  |  |  |  |  |  |  |  |  |  |  |  |  |  |  |  |  |  |  |  |  |  |  |  |  |  |  |  |  |  |  |  |  |  |  |  |  | Y4C6B.1 |  |
|  |  |  |  |  |  |  |  |  |  |  |  |  |  |  |  |  |  |  |  |  |  |  |  |  |  |  |  |  |  |  |  |  |  |  |  |  |  | *flh-3* | FLYWCH zinc finger transcription factor homolog |
|  |  |  |  |  |  |  |  |  |  |  |  |  |  |  |  |  |  |  |  |  |  |  |  |  |  |  |  |  |  |  |  |  |  |  |  |  |  | C03H12.1 |  |
|  |  |  |  |  |  |  |  |  |  |  |  |  |  |  |  |  |  |  |  |  |  |  |  |  |  |  |  |  |  |  |  |  |  |  |  |  |  | *gem-4* | Gon-2 Extragenic Modifier |
|  |  |  |  |  |  |  |  |  |  |  |  |  |  |  |  |  |  |  |  |  |  |  |  |  |  |  |  |  |  |  |  |  |  |  |  |  |  | R02D3.7 |  |
|  |  |  |  |  |  |  |  |  |  |  |  |  |  |  |  |  |  |  |  |  |  |  |  |  |  |  |  |  |  |  |  |  |  |  |  |  |  | *lem-3* | LEM domain protein |
|  |  |  |  |  |  |  |  |  |  |  |  |  |  |  |  |  |  |  |  |  |  |  |  |  |  |  |  |  |  |  |  |  |  |  |  |  |  | *mel-28* | Maternal Effect Lethal |
|  |  |  |  |  |  |  |  |  |  |  |  |  |  |  |  |  |  |  |  |  |  |  |  |  |  |  |  |  |  |  |  |  |  |  |  |  |  | *smc-5* | SMC (structural maintenance of chromosomes) family |
|  |  |  |  |  |  |  |  |  |  |  |  |  |  |  |  |  |  |  |  |  |  |  |  |  |  |  |  |  |  |  |  |  |  |  |  |  |  | F59E12.1 |  |
|  |  |  |  |  |  |  |  |  |  |  |  |  |  |  |  |  |  |  |  |  |  |  |  |  |  |  |  |  |  |  |  |  |  |  |  |  |  | *trr-1* | TRRAP-like (transcription/transformation domain-associated protein) |
|  |  |  |  |  |  |  |  |  |  |  |  |  |  |  |  |  |  |  |  |  |  |  |  |  |  |  |  |  |  |  |  |  |  |  |  |  |  | *ran-2* | associated with RAN (nuclear import/export) function |
|  |  |  |  |  |  |  |  |  |  |  |  |  |  |  |  |  |  |  |  |  |  |  |  |  |  |  |  |  |  |  |  |  |  |  |  |  |  | F10C2.4 |  |
|  |  |  |  |  |  |  |  |  |  |  |  |  |  |  |  |  |  |  |  |  |  |  |  |  |  |  |  |  |  |  |  |  |  |  |  |  |  | *lin-35* | abnormal cell LINeage |
|  |  |  |  |  |  |  |  |  |  |  |  |  |  |  |  |  |  |  |  |  |  |  |  |  |  |  |  |  |  |  |  |  |  |  |  |  |  | C01H6.9 |  |
|  |  |  |  |  |  |  |  |  |  |  |  |  |  |  |  |  |  |  |  |  |  |  |  |  |  |  |  |  |  |  |  |  |  |  |  |  |  | Y45G5AM.7 |  |
|  |  |  |  |  |  |  |  |  |  |  |  |  |  |  |  |  |  |  |  |  |  |  |  |  |  |  |  |  |  |  |  |  |  |  |  |  |  | *capg-2* | CAP-G condensin subunit |
|  |  |  |  |  |  |  |  |  |  |  |  |  |  |  |  |  |  |  |  |  |  |  |  |  |  |  |  |  |  |  |  |  |  |  |  |  |  | F23C8.9 |  |
|  |  |  |  |  |  |  |  |  |  |  |  |  |  |  |  |  |  |  |  |  |  |  |  |  |  |  |  |  |  |  |  |  |  |  |  |  |  | *cku-80* | Caenorhabditis KU |
|  |  |  |  |  |  |  |  |  |  |  |  |  |  |  |  |  |  |  |  |  |  |  |  |  |  |  |  |  |  |  |  |  |  |  |  |  |  | *rod-1* | ROD (Drosophila RoughDeal) homolog |
|  |  |  |  |  |  |  |  |  |  |  |  |  |  |  |  |  |  |  |  |  |  |  |  |  |  |  |  |  |  |  |  |  |  |  |  |  |  | *gos-28* | GOlgi SNAP receptor complex member |
|  |  |  |  |  |  |  |  |  |  |  |  |  |  |  |  |  |  |  |  |  |  |  |  |  |  |  |  |  |  |  |  |  |  |  |  |  |  | *cav-1* | CAVeolin |
|  |  |  |  |  |  |  |  |  |  |  |  |  |  |  |  |  |  |  |  |  |  |  |  |  |  |  |  |  |  |  |  |  |  |  |  |  |  | *fbxb-66* | F-box B protein |
|  |  |  |  |  |  |  |  |  |  |  |  |  |  |  |  |  |  |  |  |  |  |  |  |  |  |  |  |  |  |  |  |  |  |  |  |  |  | F28D1.2 |  |
|  |  |  |  |  |  |  |  |  |  |  |  |  |  |  |  |  |  |  |  |  |  |  |  |  |  |  |  |  |  |  |  |  |  |  |  |  |  | *cars-2* | Cysteinyl Amino-acyl tRNA Synthetase |
|  |  |  |  |  |  |  |  |  |  |  |  |  |  |  |  |  |  |  |  |  |  |  |  |  |  |  |  |  |  |  |  |  |  |  |  |  |  | *ced-3* | CEll Death abnormality |
|  |  |  |  |  |  |  |  |  |  |  |  |  |  |  |  |  |  |  |  |  |  |  |  |  |  |  |  |  |  |  |  |  |  |  |  |  |  | F13A7.14 |  |
|  |  |  |  |  |  |  |  |  |  |  |  |  |  |  |  |  |  |  |  |  |  |  |  |  |  |  |  |  |  |  |  |  |  |  |  |  |  | *spdl-1* | SPinDLy (Drosophila chromosome segregation) homolog |
|  |  |  |  |  |  |  |  |  |  |  |  |  |  |  |  |  |  |  |  |  |  |  |  |  |  |  |  |  |  |  |  |  |  |  |  |  |  | *him-10* | High Incidence of Males (increased X chromosome loss) |
|  |  |  |  |  |  |  |  |  |  |  |  |  |  |  |  |  |  |  |  |  |  |  |  |  |  |  |  |  |  |  |  |  |  |  |  |  |  | *air-2* | Aurora/Ipl1 Related kinase |
|  |  |  |  |  |  |  |  |  |  |  |  |  |  |  |  |  |  |  |  |  |  |  |  |  |  |  |  |  |  |  |  |  |  |  |  |  |  | *rad-51* | RADiation sensitivity abnormal/yeast RAD-related |
|  |  |  |  |  |  |  |  |  |  |  |  |  |  |  |  |  |  |  |  |  |  |  |  |  |  |  |  |  |  |  |  |  |  |  |  |  |  | C39E9.12 |  |
|  |  |  |  |  |  |  |  |  |  |  |  |  |  |  |  |  |  |  |  |  |  |  |  |  |  |  |  |  |  |  |  |  |  |  |  |  |  | *tpxl-1* | TPX2 (Targeting Protein for Xenopus Klp2)-Like |
|  |  |  |  |  |  |  |  |  |  |  |  |  |  |  |  |  |  |  |  |  |  |  |  |  |  |  |  |  |  |  |  |  |  |  |  |  |  | *rfc-3* | RFC (DNA replication factor) family |
|  |  |  |  |  |  |  |  |  |  |  |  |  |  |  |  |  |  |  |  |  |  |  |  |  |  |  |  |  |  |  |  |  |  |  |  |  |  | *mis-12* | human/fission yeast MIS (MInichromosome Stability) homolog |
|  |  |  |  |  |  |  |  |  |  |  |  |  |  |  |  |  |  |  |  |  |  |  |  |  |  |  |  |  |  |  |  |  |  |  |  |  |  | *hcp-3* | HoloCentric chromosome binding Protein |
|  |  |  |  |  |  |  |  |  |  |  |  |  |  |  |  |  |  |  |  |  |  |  |  |  |  |  |  |  |  |  |  |  |  |  |  |  |  | *ify-1* | Interactor of FizzY protein |
|  |  |  |  |  |  |  |  |  |  |  |  |  |  |  |  |  |  |  |  |  |  |  |  |  |  |  |  |  |  |  |  |  |  |  |  |  |  | Y37E11AL.3 |  |
|  |  |  |  |  |  |  |  |  |  |  |  |  |  |  |  |  |  |  |  |  |  |  |  |  |  |  |  |  |  |  |  |  |  |  |  |  |  | *cdc-7* | Cell Division Cycle related |
|  |  |  |  |  |  |  |  |  |  |  |  |  |  |  |  |  |  |  |  |  |  |  |  |  |  |  |  |  |  |  |  |  |  |  |  |  |  | *ntp-1* | Nucleoside TriPhosphatase |
|  |  |  |  |  |  |  |  |  |  |  |  |  |  |  |  |  |  |  |  |  |  |  |  |  |  |  |  |  |  |  |  |  |  |  |  |  |  | *thk-1* | THymidine Kinase |
|  |  |  |  |  |  |  |  |  |  |  |  |  |  |  |  |  |  |  |  |  |  |  |  |  |  |  |  |  |  |  |  |  |  |  |  |  |  | *sex-1* | Signal Element on X |
|  |  |  |  |  |  |  |  |  |  |  |  |  |  |  |  |  |  |  |  |  |  |  |  |  |  |  |  |  |  |  |  |  |  |  |  |  |  | *sas-5* | Spindle ASsembly abnormal |
|  |  |  |  |  |  |  |  |  |  |  |  |  |  |  |  |  |  |  |  |  |  |  |  |  |  |  |  |  |  |  |  |  |  |  |  |  |  | *lin-53* | abnormal cell LINeage |
|  |  |  |  |  |  |  |  |  |  |  |  |  |  |  |  |  |  |  |  |  |  |  |  |  |  |  |  |  |  |  |  |  |  |  |  |  |  | *cyb-1* | CYclin B |
|  |  |  |  |  |  |  |  |  |  |  |  |  |  |  |  |  |  |  |  |  |  |  |  |  |  |  |  |  |  |  |  |  |  |  |  |  |  | *cdl-1* | Cell Death Lethal |
|  |  |  |  |  |  |  |  |  |  |  |  |  |  |  |  |  |  |  |  |  |  |  |  |  |  |  |  |  |  |  |  |  |  |  |  |  |  | *fzy-1* | FiZzY (CDC20 protein family) homolog |
|  |  |  |  |  |  |  |  |  |  |  |  |  |  |  |  |  |  |  |  |  |  |  |  |  |  |  |  |  |  |  |  |  |  |  |  |  |  | *cyb-3* | CYclin B |
|  |  |  |  |  |  |  |  |  |  |  |  |  |  |  |  |  |  |  |  |  |  |  |  |  |  |  |  |  |  |  |  |  |  |  |  |  |  | *fbxc-51* | F-box C protein |
|  |  |  |  |  |  |  |  |  |  |  |  |  |  |  |  |  |  |  |  |  |  |  |  |  |  |  |  |  |  |  |  |  |  |  |  |  |  | *cdk-4* | Cyclin-Dependent Kinase family |
|  |  |  |  |  |  |  |  |  |  |  |  |  |  |  |  |  |  |  |  |  |  |  |  |  |  |  |  |  |  |  |  |  |  |  |  |  |  | *inx-2* | INneXin |
|  |  |  |  |  |  |  |  |  |  |  |  |  |  |  |  |  |  |  |  |  |  |  |  |  |  |  |  |  |  |  |  |  |  |  |  |  |  | B0432.10 |  |
|  |  |  |  |  |  |  |  |  |  |  |  |  |  |  |  |  |  |  |  |  |  |  |  |  |  |  |  |  |  |  |  |  |  |  |  |  |  | *mdf-2* | MAD (yeast Mitosis arrest DeFicient) related |
|  |  |  |  |  |  |  |  |  |  |  |  |  |  |  |  |  |  |  |  |  |  |  |  |  |  |  |  |  |  |  |  |  |  |  |  |  |  | *rfc-4* | RFC (DNA replication factor) family |
|  |  |  |  |  |  |  |  |  |  |  |  |  |  |  |  |  |  |  |  |  |  |  |  |  |  |  |  |  |  |  |  |  |  |  |  |  |  | K03H1.7 |  |
|  |  |  |  |  |  |  |  |  |  |  |  |  |  |  |  |  |  |  |  |  |  |  |  |  |  |  |  |  |  |  |  |  |  |  |  |  |  | *rba-1* | RBAp48 related |
|  |  |  |  |  |  |  |  |  |  |  |  |  |  |  |  |  |  |  |  |  |  |  |  |  |  |  |  |  |  |  |  |  |  |  |  |  |  | *cdk-1* | Cyclin-Dependent Kinase family |
|  |  |  |  |  |  |  |  |  |  |  |  |  |  |  |  |  |  |  |  |  |  |  |  |  |  |  |  |  |  |  |  |  |  |  |  |  |  | *air-1* | Aurora/Ipl1 Related kinase |
|  |  |  |  |  |  |  |  |  |  |  |  |  |  |  |  |  |  |  |  |  |  |  |  |  |  |  |  |  |  |  |  |  |  |  |  |  |  | F33H2.6 |  |
|  |  |  |  |  |  |  |  |  |  |  |  |  |  |  |  |  |  |  |  |  |  |  |  |  |  |  |  |  |  |  |  |  |  |  |  |  |  | B0432.9 |  |
|  |  |  |  |  |  |  |  |  |  |  |  |  |  |  |  |  |  |  |  |  |  |  |  |  |  |  |  |  |  |  |  |  |  |  |  |  |  | *orc-5* | ORC (Origin Recognition Complex) subunit |
|  |  |  |  |  |  |  |  |  |  |  |  |  |  |  |  |  |  |  |  |  |  |  |  |  |  |  |  |  |  |  |  |  |  |  |  |  |  | F40G9.15 |  |
|  |  |  |  |  |  |  |  |  |  |  |  |  |  |  |  |  |  |  |  |  |  |  |  |  |  |  |  |  |  |  |  |  |  |  |  |  |  | F40G9.6 |  |
|  |  |  |  |  |  |  |  |  |  |  |  |  |  |  |  |  |  |  |  |  |  |  |  |  |  |  |  |  |  |  |  |  |  |  |  |  |  | F40G9.5 |  |
|  |  |  |  |  |  |  |  |  |  |  |  |  |  |  |  |  |  |  |  |  |  |  |  |  |  |  |  |  |  |  |  |  |  |  |  |  |  | C06E1.1 |  |
|  |  |  |  |  |  |  |  |  |  |  |  |  |  |  |  |  |  |  |  |  |  |  |  |  |  |  |  |  |  |  |  |  |  |  |  |  |  | *pot-1* | Protection Of Telomeres 1 (Pot1) homolog |
|  |  |  |  |  |  |  |  |  |  |  |  |  |  |  |  |  |  |  |  |  |  |  |  |  |  |  |  |  |  |  |  |  |  |  |  |  |  | *ruvb-2* | RUVB (recombination protein) homolog |
|  |  |  |  |  |  |  |  |  |  |  |  |  |  |  |  |  |  |  |  |  |  |  |  |  |  |  |  |  |  |  |  |  |  |  |  |  |  | *fbxb-102* | F-box B protein |
|  |  |  |  |  |  |  |  |  |  |  |  |  |  |  |  |  |  |  |  |  |  |  |  |  |  |  |  |  |  |  |  |  |  |  |  |  |  | *nep-15* | NEPrilysin metallopeptidase family |
|  |  |  |  |  |  |  |  |  |  |  |  |  |  |  |  |  |  |  |  |  |  |  |  |  |  |  |  |  |  |  |  |  |  |  |  |  |  | F28H1.1 |  |
|  |  |  |  |  |  |  |  |  |  |  |  |  |  |  |  |  |  |  |  |  |  |  |  |  |  |  |  |  |  |  |  |  |  |  |  |  |  | F55C9.11 |  |
|  |  |  |  |  |  |  |  |  |  |  |  |  |  |  |  |  |  |  |  |  |  |  |  |  |  |  |  |  |  |  |  |  |  |  |  |  |  | *fbxb-54* | F-box B protein |
|  |  |  |  |  |  |  |  |  |  |  |  |  |  |  |  |  |  |  |  |  |  |  |  |  |  |  |  |  |  |  |  |  |  |  |  |  |  | *fbxb-100* | F-box B protein |
|  |  |  |  |  |  |  |  |  |  |  |  |  |  |  |  |  |  |  |  |  |  |  |  |  |  |  |  |  |  |  |  |  |  |  |  |  |  | C43D7.8 |  |
|  |  |  |  |  |  |  |  |  |  |  |  |  |  |  |  |  |  |  |  |  |  |  |  |  |  |  |  |  |  |  |  |  |  |  |  |  |  | *fbxb-8* | F-box B protein |
|  |  |  |  |  |  |  |  |  |  |  |  |  |  |  |  |  |  |  |  |  |  |  |  |  |  |  |  |  |  |  |  |  |  |  |  |  |  | *skr-19* | SKp1 Related (ubiquitin ligase complex component) |
|  |  |  |  |  |  |  |  |  |  |  |  |  |  |  |  |  |  |  |  |  |  |  |  |  |  |  |  |  |  |  |  |  |  |  |  |  |  | F45G2.9 |  |
|  |  |  |  |  |  |  |  |  |  |  |  |  |  |  |  |  |  |  |  |  |  |  |  |  |  |  |  |  |  |  |  |  |  |  |  |  |  | Y116A8A.10 |  |
|  |  |  |  |  |  |  |  |  |  |  |  |  |  |  |  |  |  |  |  |  |  |  |  |  |  |  |  |  |  |  |  |  |  |  |  |  |  | *clec-142* | C-type LECtin |
|  |  |  |  |  |  |  |  |  |  |  |  |  |  |  |  |  |  |  |  |  |  |  |  |  |  |  |  |  |  |  |  |  |  |  |  |  |  | C49F5.6 |  |
|  |  |  |  |  |  |  |  |  |  |  |  |  |  |  |  |  |  |  |  |  |  |  |  |  |  |  |  |  |  |  |  |  |  |  |  |  |  | *fbxb-15* | F-box B protein |
|  |  |  |  |  |  |  |  |  |  |  |  |  |  |  |  |  |  |  |  |  |  |  |  |  |  |  |  |  |  |  |  |  |  |  |  |  |  | T07F10.5 |  |
|  |  |  |  |  |  |  |  |  |  |  |  |  |  |  |  |  |  |  |  |  |  |  |  |  |  |  |  |  |  |  |  |  |  |  |  |  |  | *bath-21* | BTB and MATH domain containing |
|  |  |  |  |  |  |  |  |  |  |  |  |  |  |  |  |  |  |  |  |  |  |  |  |  |  |  |  |  |  |  |  |  |  |  |  |  |  | *cya-2* | CYclin A |
|  |  |  |  |  |  |  |  |  |  |  |  |  |  |  |  |  |  |  |  |  |  |  |  |  |  |  |  |  |  |  |  |  |  |  |  |  |  | W05G11.2 |  |
|  |  |  |  |  |  |  |  |  |  |  |  |  |  |  |  |  |  |  |  |  |  |  |  |  |  |  |  |  |  |  |  |  |  |  |  |  |  | T23F6.3 |  |
|  |  |  |  |  |  |  |  |  |  |  |  |  |  |  |  |  |  |  |  |  |  |  |  |  |  |  |  |  |  |  |  |  |  |  |  |  |  | Y54F10BM.9 |  |
|  |  |  |  |  |  |  |  |  |  |  |  |  |  |  |  |  |  |  |  |  |  |  |  |  |  |  |  |  |  |  |  |  |  |  |  |  |  | *lin-15B* | abnormal cell LINeage |
|  |  |  |  |  |  |  |  |  |  |  |  |  |  |  |  |  |  |  |  |  |  |  |  |  |  |  |  |  |  |  |  |  |  |  |  |  |  | R04A9.9 |  |
|  |  |  |  |  |  |  |  |  |  |  |  |  |  |  |  |  |  |  |  |  |  |  |  |  |  |  |  |  |  |  |  |  |  |  |  |  |  | F47B10.9 |  |
|  |  |  |  |  |  |  |  |  |  |  |  |  |  |  |  |  |  |  |  |  |  |  |  |  |  |  |  |  |  |  |  |  |  |  |  |  |  | *ife-1* | Initiation Factor 4E (eIF4E) family |
|  |  |  |  |  |  |  |  |  |  |  |  |  |  |  |  |  |  |  |  |  |  |  |  |  |  |  |  |  |  |  |  |  |  |  |  |  |  | *fbxb-50* | F-box B protein |
|  |  |  |  |  |  |  |  |  |  |  |  |  |  |  |  |  |  |  |  |  |  |  |  |  |  |  |  |  |  |  |  |  |  |  |  |  |  | *skr-20* | SKp1 Related (ubiquitin ligase complex component) |
|  |  |  |  |  |  |  |  |  |  |  |  |  |  |  |  |  |  |  |  |  |  |  |  |  |  |  |  |  |  |  |  |  |  |  |  |  |  | *atg-3* | AuTophaGy (yeast Atg homolog) |
|  |  |  |  |  |  |  |  |  |  |  |  |  |  |  |  |  |  |  |  |  |  |  |  |  |  |  |  |  |  |  |  |  |  |  |  |  |  | M02E1.3 |  |
|  |  |  |  |  |  |  |  |  |  |  |  |  |  |  |  |  |  |  |  |  |  |  |  |  |  |  |  |  |  |  |  |  |  |  |  |  |  | *fbxb-65* | F-box B protein |
|  |  |  |  |  |  |  |  |  |  |  |  |  |  |  |  |  |  |  |  |  |  |  |  |  |  |  |  |  |  |  |  |  |  |  |  |  |  | *arrd-1* | ARRestin Domain protein |
|  |  |  |  |  |  |  |  |  |  |  |  |  |  |  |  |  |  |  |  |  |  |  |  |  |  |  |  |  |  |  |  |  |  |  |  |  |  | *fbxb-7* | F-box B protein |
|  |  |  |  |  |  |  |  |  |  |  |  |  |  |  |  |  |  |  |  |  |  |  |  |  |  |  |  |  |  |  |  |  |  |  |  |  |  | *hda-6* | Histone DeAcetylase |
|  |  |  |  |  |  |  |  |  |  |  |  |  |  |  |  |  |  |  |  |  |  |  |  |  |  |  |  |  |  |  |  |  |  |  |  |  |  | M04B2.2 |  |
|  |  |  |  |  |  |  |  |  |  |  |  |  |  |  |  |  |  |  |  |  |  |  |  |  |  |  |  |  |  |  |  |  |  |  |  |  |  | Y34B4A.11 |  |
|  |  |  |  |  |  |  |  |  |  |  |  |  |  |  |  |  |  |  |  |  |  |  |  |  |  |  |  |  |  |  |  |  |  |  |  |  |  | C42C1.9 |  |
|  |  |  |  |  |  |  |  |  |  |  |  |  |  |  |  |  |  |  |  |  |  |  |  |  |  |  |  |  |  |  |  |  |  |  |  |  |  | ZK1248.15 |  |
|  |  |  |  |  |  |  |  |  |  |  |  |  |  |  |  |  |  |  |  |  |  |  |  |  |  |  |  |  |  |  |  |  |  |  |  |  |  | *prp-38* | yeast PRP (splicing factor) related |
|  |  |  |  |  |  |  |  |  |  |  |  |  |  |  |  |  |  |  |  |  |  |  |  |  |  |  |  |  |  |  |  |  |  |  |  |  |  | D2030.8 |  |
|  |  |  |  |  |  |  |  |  |  |  |  |  |  |  |  |  |  |  |  |  |  |  |  |  |  |  |  |  |  |  |  |  |  |  |  |  |  | R11D1.10 |  |
|  |  |  |  |  |  |  |  |  |  |  |  |  |  |  |  |  |  |  |  |  |  |  |  |  |  |  |  |  |  |  |  |  |  |  |  |  |  | T05H4.11 |  |
|  |  |  |  |  |  |  |  |  |  |  |  |  |  |  |  |  |  |  |  |  |  |  |  |  |  |  |  |  |  |  |  |  |  |  |  |  |  | F53F4.12 |  |
|  |  |  |  |  |  |  |  |  |  |  |  |  |  |  |  |  |  |  |  |  |  |  |  |  |  |  |  |  |  |  |  |  |  |  |  |  |  | *tag-261* | Temporarily Assigned Gene name |
|  |  |  |  |  |  |  |  |  |  |  |  |  |  |  |  |  |  |  |  |  |  |  |  |  |  |  |  |  |  |  |  |  |  |  |  |  |  | B0513.2 |  |
|  |  |  |  |  |  |  |  |  |  |  |  |  |  |  |  |  |  |  |  |  |  |  |  |  |  |  |  |  |  |  |  |  |  |  |  |  |  | *ooc-5* | abnormal OOCyte formation |
|  |  |  |  |  |  |  |  |  |  |  |  |  |  |  |  |  |  |  |  |  |  |  |  |  |  |  |  |  |  |  |  |  |  |  |  |  |  | Y53F4B.42 |  |
|  |  |  |  |  |  |  |  |  |  |  |  |  |  |  |  |  |  |  |  |  |  |  |  |  |  |  |  |  |  |  |  |  |  |  |  |  |  | T05F1.4 |  |
|  |  |  |  |  |  |  |  |  |  |  |  |  |  |  |  |  |  |  |  |  |  |  |  |  |  |  |  |  |  |  |  |  |  |  |  |  |  | F39H11.1 |  |
|  |  |  |  |  |  |  |  |  |  |  |  |  |  |  |  |  |  |  |  |  |  |  |  |  |  |  |  |  |  |  |  |  |  |  |  |  |  | T01C3.2 |  |
|  |  |  |  |  |  |  |  |  |  |  |  |  |  |  |  |  |  |  |  |  |  |  |  |  |  |  |  |  |  |  |  |  |  |  |  |  |  | F33H1.3 |  |
|  |  |  |  |  |  |  |  |  |  |  |  |  |  |  |  |  |  |  |  |  |  |  |  |  |  |  |  |  |  |  |  |  |  |  |  |  |  | Y66D12A.8 |  |
|  |  |  |  |  |  |  |  |  |  |  |  |  |  |  |  |  |  |  |  |  |  |  |  |  |  |  |  |  |  |  |  |  |  |  |  |  |  | *hpl-2* | HP1 Like (heterochromatin protein) |
|  |  |  |  |  |  |  |  |  |  |  |  |  |  |  |  |  |  |  |  |  |  |  |  |  |  |  |  |  |  |  |  |  |  |  |  |  |  | *cpf-2* | Cleavage and Polyadenylation Factor |
|  |  |  |  |  |  |  |  |  |  |  |  |  |  |  |  |  |  |  |  |  |  |  |  |  |  |  |  |  |  |  |  |  |  |  |  |  |  | F43C11.9 |  |
|  |  |  |  |  |  |  |  |  |  |  |  |  |  |  |  |  |  |  |  |  |  |  |  |  |  |  |  |  |  |  |  |  |  |  |  |  |  | C32E8.5 |  |
|  |  |  |  |  |  |  |  |  |  |  |  |  |  |  |  |  |  |  |  |  |  |  |  |  |  |  |  |  |  |  |  |  |  |  |  |  |  | *sel-13* | Suppressor/Enhancer of Lin-12 |
|  |  |  |  |  |  |  |  |  |  |  |  |  |  |  |  |  |  |  |  |  |  |  |  |  |  |  |  |  |  |  |  |  |  |  |  |  |  | C16A3.4 |  |
|  |  |  |  |  |  |  |  |  |  |  |  |  |  |  |  |  |  |  |  |  |  |  |  |  |  |  |  |  |  |  |  |  |  |  |  |  |  | *rpb-6* | RNA Polymerase II (B) subunit |
|  |  |  |  |  |  |  |  |  |  |  |  |  |  |  |  |  |  |  |  |  |  |  |  |  |  |  |  |  |  |  |  |  |  |  |  |  |  | *dohh-1* | DeOxyHypusine Hydroxylase |
|  |  |  |  |  |  |  |  |  |  |  |  |  |  |  |  |  |  |  |  |  |  |  |  |  |  |  |  |  |  |  |  |  |  |  |  |  |  | *wrb-1* | WRB (human W(tryptophan)-Rich Basic nuclear protein) homolog |
|  |  |  |  |  |  |  |  |  |  |  |  |  |  |  |  |  |  |  |  |  |  |  |  |  |  |  |  |  |  |  |  |  |  |  |  |  |  | *snr-2* | Small Nuclear Ribonucleoprotein |
|  |  |  |  |  |  |  |  |  |  |  |  |  |  |  |  |  |  |  |  |  |  |  |  |  |  |  |  |  |  |  |  |  |  |  |  |  |  | *hpo-8* | Hypersensitive to POre-forming toxin |
|  |  |  |  |  |  |  |  |  |  |  |  |  |  |  |  |  |  |  |  |  |  |  |  |  |  |  |  |  |  |  |  |  |  |  |  |  |  | C08F1.6 |  |
|  |  |  |  |  |  |  |  |  |  |  |  |  |  |  |  |  |  |  |  |  |  |  |  |  |  |  |  |  |  |  |  |  |  |  |  |  |  | *baf-1* | Barrier to Autointegration Factor |
|  |  |  |  |  |  |  |  |  |  |  |  |  |  |  |  |  |  |  |  |  |  |  |  |  |  |  |  |  |  |  |  |  |  |  |  |  |  | Y54G9A.9 |  |
|  |  |  |  |  |  |  |  |  |  |  |  |  |  |  |  |  |  |  |  |  |  |  |  |  |  |  |  |  |  |  |  |  |  |  |  |  |  | Y54E5A.5 |  |
|  |  |  |  |  |  |  |  |  |  |  |  |  |  |  |  |  |  |  |  |  |  |  |  |  |  |  |  |  |  |  |  |  |  |  |  |  |  | Y51H4A.15 |  |
|  |  |  |  |  |  |  |  |  |  |  |  |  |  |  |  |  |  |  |  |  |  |  |  |  |  |  |  |  |  |  |  |  |  |  |  |  |  | *ran-1* | associated with RAN (nuclear import/export) function |
|  |  |  |  |  |  |  |  |  |  |  |  |  |  |  |  |  |  |  |  |  |  |  |  |  |  |  |  |  |  |  |  |  |  |  |  |  |  | F15A4.2 |  |
|  |  |  |  |  |  |  |  |  |  |  |  |  |  |  |  |  |  |  |  |  |  |  |  |  |  |  |  |  |  |  |  |  |  |  |  |  |  | *hmg-5* | HMG |
|  |  |  |  |  |  |  |  |  |  |  |  |  |  |  |  |  |  |  |  |  |  |  |  |  |  |  |  |  |  |  |  |  |  |  |  |  |  | Y39B6A.37 |  |
|  |  |  |  |  |  |  |  |  |  |  |  |  |  |  |  |  |  |  |  |  |  |  |  |  |  |  |  |  |  |  |  |  |  |  |  |  |  | *eif-3.G* | Eukaryotic Initiation Factor |
|  |  |  |  |  |  |  |  |  |  |  |  |  |  |  |  |  |  |  |  |  |  |  |  |  |  |  |  |  |  |  |  |  |  |  |  |  |  | C41D11.5 |  |
|  |  |  |  |  |  |  |  |  |  |  |  |  |  |  |  |  |  |  |  |  |  |  |  |  |  |  |  |  |  |  |  |  |  |  |  |  |  | C41D11.9 |  |
|  |  |  |  |  |  |  |  |  |  |  |  |  |  |  |  |  |  |  |  |  |  |  |  |  |  |  |  |  |  |  |  |  |  |  |  |  |  | B0041.8 |  |
|  |  |  |  |  |  |  |  |  |  |  |  |  |  |  |  |  |  |  |  |  |  |  |  |  |  |  |  |  |  |  |  |  |  |  |  |  |  | C40A11.2 |  |
|  |  |  |  |  |  |  |  |  |  |  |  |  |  |  |  |  |  |  |  |  |  |  |  |  |  |  |  |  |  |  |  |  |  |  |  |  |  | C27H6.8 |  |
|  |  |  |  |  |  |  |  |  |  |  |  |  |  |  |  |  |  |  |  |  |  |  |  |  |  |  |  |  |  |  |  |  |  |  |  |  |  | R06C7.6 |  |
|  |  |  |  |  |  |  |  |  |  |  |  |  |  |  |  |  |  |  |  |  |  |  |  |  |  |  |  |  |  |  |  |  |  |  |  |  |  | ZK643.2 |  |
|  |  |  |  |  |  |  |  |  |  |  |  |  |  |  |  |  |  |  |  |  |  |  |  |  |  |  |  |  |  |  |  |  |  |  |  |  |  | *ssup-72* | SSU (yeast Suppressor of SUa7) Protein homolog |
|  |  |  |  |  |  |  |  |  |  |  |  |  |  |  |  |  |  |  |  |  |  |  |  |  |  |  |  |  |  |  |  |  |  |  |  |  |  | T21C9.4 |  |
|  |  |  |  |  |  |  |  |  |  |  |  |  |  |  |  |  |  |  |  |  |  |  |  |  |  |  |  |  |  |  |  |  |  |  |  |  |  | K05C4.7 |  |
|  |  |  |  |  |  |  |  |  |  |  |  |  |  |  |  |  |  |  |  |  |  |  |  |  |  |  |  |  |  |  |  |  |  |  |  |  |  | K10D2.5 |  |
|  |  |  |  |  |  |  |  |  |  |  |  |  |  |  |  |  |  |  |  |  |  |  |  |  |  |  |  |  |  |  |  |  |  |  |  |  |  | ZK418.5 |  |
|  |  |  |  |  |  |  |  |  |  |  |  |  |  |  |  |  |  |  |  |  |  |  |  |  |  |  |  |  |  |  |  |  |  |  |  |  |  | Y69A2AR.32 |  |
|  |  |  |  |  |  |  |  |  |  |  |  |  |  |  |  |  |  |  |  |  |  |  |  |  |  |  |  |  |  |  |  |  |  |  |  |  |  | F09F7.7 |  |
|  |  |  |  |  |  |  |  |  |  |  |  |  |  |  |  |  |  |  |  |  |  |  |  |  |  |  |  |  |  |  |  |  |  |  |  |  |  | F35D11.4 |  |
|  |  |  |  |  |  |  |  |  |  |  |  |  |  |  |  |  |  |  |  |  |  |  |  |  |  |  |  |  |  |  |  |  |  |  |  |  |  | C07D8.6 |  |
|  |  |  |  |  |  |  |  |  |  |  |  |  |  |  |  |  |  |  |  |  |  |  |  |  |  |  |  |  |  |  |  |  |  |  |  |  |  | *enu-3* | ENhancer of Uncoordination |
|  |  |  |  |  |  |  |  |  |  |  |  |  |  |  |  |  |  |  |  |  |  |  |  |  |  |  |  |  |  |  |  |  |  |  |  |  |  | F45D11.5 |  |
|  |  |  |  |  |  |  |  |  |  |  |  |  |  |  |  |  |  |  |  |  |  |  |  |  |  |  |  |  |  |  |  |  |  |  |  |  |  | M03B6.4 |  |
|  |  |  |  |  |  |  |  |  |  |  |  |  |  |  |  |  |  |  |  |  |  |  |  |  |  |  |  |  |  |  |  |  |  |  |  |  |  | B0205.8 |  |
|  |  |  |  |  |  |  |  |  |  |  |  |  |  |  |  |  |  |  |  |  |  |  |  |  |  |  |  |  |  |  |  |  |  |  |  |  |  | F21D9.6 |  |
|  |  |  |  |  |  |  |  |  |  |  |  |  |  |  |  |  |  |  |  |  |  |  |  |  |  |  |  |  |  |  |  |  |  |  |  |  |  | *sre-36* | Serpentine Receptor, class E (epsilon) |
|  |  |  |  |  |  |  |  |  |  |  |  |  |  |  |  |  |  |  |  |  |  |  |  |  |  |  |  |  |  |  |  |  |  |  |  |  |  | *skr-21* | SKp1 Related (ubiquitin ligase complex component) |
|  |  |  |  |  |  |  |  |  |  |  |  |  |  |  |  |  |  |  |  |  |  |  |  |  |  |  |  |  |  |  |  |  |  |  |  |  |  | *htz-1* | HisTone variant H2AZ homolog |
|  |  |  |  |  |  |  |  |  |  |  |  |  |  |  |  |  |  |  |  |  |  |  |  |  |  |  |  |  |  |  |  |  |  |  |  |  |  | Y69A2AR.28 |  |
|  |  |  |  |  |  |  |  |  |  |  |  |  |  |  |  |  |  |  |  |  |  |  |  |  |  |  |  |  |  |  |  |  |  |  |  |  |  | *hil-2* | HIstone H1 Like |
|  |  |  |  |  |  |  |  |  |  |  |  |  |  |  |  |  |  |  |  |  |  |  |  |  |  |  |  |  |  |  |  |  |  |  |  |  |  | T28B8.1 |  |
|  |  |  |  |  |  |  |  |  |  |  |  |  |  |  |  |  |  |  |  |  |  |  |  |  |  |  |  |  |  |  |  |  |  |  |  |  |  | *hil-3* | HIstone H1 Like |
|  |  |  |  |  |  |  |  |  |  |  |  |  |  |  |  |  |  |  |  |  |  |  |  |  |  |  |  |  |  |  |  |  |  |  |  |  |  | *cey-4* | C. Elegans Y-box |
|  |  |  |  |  |  |  |  |  |  |  |  |  |  |  |  |  |  |  |  |  |  |  |  |  |  |  |  |  |  |  |  |  |  |  |  |  |  | *zim-1* | Zinc finger In Meiosis |
|  |  |  |  |  |  |  |  |  |  |  |  |  |  |  |  |  |  |  |  |  |  |  |  |  |  |  |  |  |  |  |  |  |  |  |  |  |  | T07G12.8 |  |
|  |  |  |  |  |  |  |  |  |  |  |  |  |  |  |  |  |  |  |  |  |  |  |  |  |  |  |  |  |  |  |  |  |  |  |  |  |  | Y37B11A.3 |  |
|  |  |  |  |  |  |  |  |  |  |  |  |  |  |  |  |  |  |  |  |  |  |  |  |  |  |  |  |  |  |  |  |  |  |  |  |  |  | Y71F9AL.7 |  |
|  |  |  |  |  |  |  |  |  |  |  |  |  |  |  |  |  |  |  |  |  |  |  |  |  |  |  |  |  |  |  |  |  |  |  |  |  |  | F22E5.20 |  |
|  |  |  |  |  |  |  |  |  |  |  |  |  |  |  |  |  |  |  |  |  |  |  |  |  |  |  |  |  |  |  |  |  |  |  |  |  |  | Y27F2A.8 |  |
|  |  |  |  |  |  |  |  |  |  |  |  |  |  |  |  |  |  |  |  |  |  |  |  |  |  |  |  |  |  |  |  |  |  |  |  |  |  | F58D2.3 |  |
|  |  |  |  |  |  |  |  |  |  |  |  |  |  |  |  |  |  |  |  |  |  |  |  |  |  |  |  |  |  |  |  |  |  |  |  |  |  | *nape-1* | N-Acyl Phosphatidyl Ethanolamine specific phospholipase D (NAPE-PLD) homolog |
|  |  |  |  |  |  |  |  |  |  |  |  |  |  |  |  |  |  |  |  |  |  |  |  |  |  |  |  |  |  |  |  |  |  |  |  |  |  | Y80D3A.9 |  |
|  |  |  |  |  |  |  |  |  |  |  |  |  |  |  |  |  |  |  |  |  |  |  |  |  |  |  |  |  |  |  |  |  |  |  |  |  |  | *rgs-11* | Regulator of G protein Signaling |
|  |  |  |  |  |  |  |  |  |  |  |  |  |  |  |  |  |  |  |  |  |  |  |  |  |  |  |  |  |  |  |  |  |  |  |  |  |  | *try-1* | TRYpsin-like protease |
|  |  |  |  |  |  |  |  |  |  |  |  |  |  |  |  |  |  |  |  |  |  |  |  |  |  |  |  |  |  |  |  |  |  |  |  |  |  | *gyg-2* | GlYcoGenin like |
|  |  |  |  |  |  |  |  |  |  |  |  |  |  |  |  |  |  |  |  |  |  |  |  |  |  |  |  |  |  |  |  |  |  |  |  |  |  | W04A8.6 |  |
|  |  |  |  |  |  |  |  |  |  |  |  |  |  |  |  |  |  |  |  |  |  |  |  |  |  |  |  |  |  |  |  |  |  |  |  |  |  | Y47D3A.14 |  |
|  |  |  |  |  |  |  |  |  |  |  |  |  |  |  |  |  |  |  |  |  |  |  |  |  |  |  |  |  |  |  |  |  |  |  |  |  |  | F31D5.6 |  |
|  |  |  |  |  |  |  |  |  |  |  |  |  |  |  |  |  |  |  |  |  |  |  |  |  |  |  |  |  |  |  |  |  |  |  |  |  |  | *his-31* | HIStone |
|  |  |  |  |  |  |  |  |  |  |  |  |  |  |  |  |  |  |  |  |  |  |  |  |  |  |  |  |  |  |  |  |  |  |  |  |  |  | *par-2* | abnormal embryonic PARtitioning of cytoplasm |
|  |  |  |  |  |  |  |  |  |  |  |  |  |  |  |  |  |  |  |  |  |  |  |  |  |  |  |  |  |  |  |  |  |  |  |  |  |  | *skr-7* | SKp1 Related (ubiquitin ligase complex component) |
|  |  |  |  |  |  |  |  |  |  |  |  |  |  |  |  |  |  |  |  |  |  |  |  |  |  |  |  |  |  |  |  |  |  |  |  |  |  | *skr-12* | SKp1 Related (ubiquitin ligase complex component) |
|  |  |  |  |  |  |  |  |  |  |  |  |  |  |  |  |  |  |  |  |  |  |  |  |  |  |  |  |  |  |  |  |  |  |  |  |  |  | Y45G5AM.5 |  |
|  |  |  |  |  |  |  |  |  |  |  |  |  |  |  |  |  |  |  |  |  |  |  |  |  |  |  |  |  |  |  |  |  |  |  |  |  |  | C16C10.13 |  |
|  |  |  |  |  |  |  |  |  |  |  |  |  |  |  |  |  |  |  |  |  |  |  |  |  |  |  |  |  |  |  |  |  |  |  |  |  |  | *clec-104* | C-type LECtin |
|  |  |  |  |  |  |  |  |  |  |  |  |  |  |  |  |  |  |  |  |  |  |  |  |  |  |  |  |  |  |  |  |  |  |  |  |  |  | F53G2.2 |  |

### Phenotypes enriched

|  |  |  |  |
| --- | --- | --- | --- |
| **Group name** | **Number in cluster** | **Enrichment** | **FDR corrected p** |
| embryonic cell physiology variant (RNAi) | 69 | 7.03 | 1.30e-33 |
| embryonic cell organization biogenesis variant (RNAi) | 69 | 7.03 | 1.30e-33 |
| cell organization biogenesis variant (RNAi) | 72 | 6.49 | 5.20e-33 |
| cell morphology variant (RNAi) | 89 | 4.62 | 3.41e-30 |
| mitotic spindle defective early emb (RNAi) | 47 | 8.78 | 4.15e-26 |
| chromosome segregation variant (RNAi) | 57 | 6.57 | 1.27e-25 |
| spindle defective early emb (RNAi) | 47 | 8.41 | 2.27e-25 |
| nucleus defective early emb (RNAi) | 41 | 9.63 | 3.48e-24 |
| cell division variant (RNAi) | 57 | 6.04 | 7.36e-24 |
| pronuclear nuclear appearance defective early emb (RNAi) | 48 | 7.53 | 7.36e-24 |
| pronuclear nuclear appearance variant emb (RNAi) | 48 | 7.53 | 7.36e-24 |
| early embryonic lethal (RNAi) | 90 | 3.62 | 5.44e-23 |
| cell physiology variant (RNAi) | 122 | 2.80 | 6.72e-23 |
| lethal (RNAi) | 193 | 2.02 | 1.50e-22 |
| embryonic cell morphology variant (RNAi) | 36 | 9.23 | 2.52e-20 |
| morphology variant (RNAi) | 141 | 2.37 | 2.57e-20 |
| embryonic lethal (RNAi) | 175 | 2.04 | 9.19e-20 |
| embryonic development variant (RNAi) | 175 | 2.03 | 1.91e-19 |
| nuclear appearance number defective early emb (RNAi) | 34 | 9.45 | 1.91e-19 |
| nuclear number defective early emb (RNAi) | 30 | 10.60 | 2.29e-18 |
| multiple nuclei early emb (RNAi) | 30 | 10.60 | 2.29e-18 |
| cell homeostasis metabolism variant (RNAi) | 60 | 4.40 | 3.64e-18 |
| cell development variant (RNAi) | 88 | 3.12 | 4.72e-18 |
| cytokinesis defective early emb (RNAi) | 35 | 7.58 | 8.42e-17 |
| cytokinesis variant emb (RNAi) | 37 | 6.56 | 9.78e-16 |
| development variant (RNAi) | 203 | 1.71 | 4.07e-15 |
| organism development variant (RNAi) | 193 | 1.75 | 9.88e-15 |
| gametogenesis variant (RNAi) | 55 | 3.99 | 1.57e-14 |
| spindle position orientation defective early emb (RNAi) | 28 | 8.44 | 2.90e-14 |
| ectopic cleavage furrows early emb (RNAi) | 20 | 14.00 | 4.20e-14 |
| excessive blebbing early emb (RNAi) | 21 | 12.86 | 4.44e-14 |
| cytoplasmic appearance defective early emb (RNAi) | 28 | 8.19 | 6.46e-14 |
| cytoplasmic appearance variant (RNAi) | 28 | 8.13 | 7.82e-14 |
| vulva morphology variant (RNAi) | 67 | 3.22 | 1.34e-13 |
| hermaphrodite reproductive system morphology variant (RNAi) | 67 | 3.21 | 1.49e-13 |
| cytoplasmic dynamics defective early emb (RNAi) | 21 | 11.93 | 2.21e-13 |
| reproductive system morphology variant (RNAi) | 69 | 3.10 | 2.62e-13 |
| protruding vulva (RNAi) | 64 | 3.19 | 9.69e-13 |
| cleavage furrow defective early emb (RNAi) | 27 | 7.56 | 1.59e-12 |
| germ cell development variant (RNAi) | 48 | 3.94 | 3.10e-12 |
| sterile (RNAi) | 114 | 2.10 | 1.62e-11 |
| fertility reduced (RNAi) | 114 | 2.09 | 2.14e-11 |
| chromosome morphology variant (RNAi) | 14 | 18.30 | 3.26e-11 |
| reproductive system physiology variant (RNAi) | 127 | 1.95 | 5.16e-11 |
| organ system physiology variant (RNAi) | 127 | 1.95 | 6.37e-11 |
| cytokinesis fails early emb (RNAi) | 17 | 12.58 | 6.38e-11 |
| fertility variant (RNAi) | 126 | 1.95 | 7.97e-11 |
| cell cycle variant (RNAi) | 22 | 8.37 | 8.57e-11 |
| nuclear appearance variant (RNAi) | 21 | 8.95 | 8.57e-11 |
| pronucleus centrosomes defective early emb (RNAi) | 25 | 6.49 | 5.69e-10 |
| cell cycle defective early emb (RNAi) | 19 | 9.08 | 9.81e-10 |
| cell cycle timing defective early emb (RNAi) | 19 | 9.08 | 9.81e-10 |
| protein subcellular localization variant (RNAi) | 25 | 6.20 | 1.51e-09 |
| pattern protein expression variant (RNAi) | 26 | 5.86 | 1.95e-09 |
| spindle position defective early emb (RNAi) | 16 | 11.01 | 3.03e-09 |
| multiple cytoplasmic cavities early emb (RNAi) | 13 | 14.99 | 5.05e-09 |
| meiosis variant (RNAi) | 35 | 4.13 | 5.75e-09 |
| spindle orientation variant AB or P1 early emb (RNAi) | 14 | 11.93 | 2.19e-08 |
| kinetochore organization variant (RNAi) | 9 | 25.20 | 3.62e-08 |
| organ system morphology variant (RNAi) | 72 | 2.31 | 5.57e-08 |
| organelle organization biogenesis variant (RNAi) | 20 | 6.59 | 8.93e-08 |
| hermaphrodite fertility variant (RNAi) | 96 | 1.97 | 1.14e-07 |
| P0 spindle position defective early emb (RNAi) | 13 | 11.58 | 1.62e-07 |
| protein expression variant (RNAi) | 29 | 4.26 | 1.93e-07 |
| nucleus reforms cell division remnant early emb (RNAi) | 11 | 14.87 | 2.36e-07 |
| sister chromatid segregation defective early emb (RNAi) | 19 | 6.42 | 4.02e-07 |
| nuclear position defective early emb (RNAi) | 11 | 13.91 | 5.30e-07 |
| relative cell cycle timing defective early emb (RNAi) | 14 | 9.46 | 5.46e-07 |
| gene expression variant (RNAi) | 84 | 2.02 | 7.69e-07 |
| meiotic chromosome segregation variant (RNAi) | 20 | 5.81 | 8.12e-07 |
| nuclear envelope assembly variant early emb (RNAi) | 12 | 11.20 | 1.28e-06 |
| pronuclear morphology defective early emb (RNAi) | 13 | 9.80 | 1.46e-06 |
| cytoplasmic morphology defective early emb (RNAi) | 16 | 6.82 | 4.30e-06 |
| spindle orientation defective early emb (RNAi) | 14 | 7.96 | 5.51e-06 |
| polar body defective early emb (RNAi) | 15 | 7.17 | 6.34e-06 |
| germ cell mitosis variant (RNAi) | 11 | 11.06 | 7.09e-06 |
| progeny variant (RNAi) | 51 | 2.46 | 7.36e-06 |
| sterile progeny (RNAi) | 51 | 2.46 | 7.36e-06 |
| exploded through vulva (RNAi) | 30 | 3.37 | 1.90e-05 |
| spindle orientation variant (RNAi) | 14 | 7.13 | 2.21e-05 |
| pronuclei meet centrally early emb (RNAi) | 10 | 11.53 | 2.28e-05 |
| epithelial system development variant (RNAi) | 30 | 3.32 | 2.56e-05 |
| chromosome disjunction defective (RNAi) | 17 | 5.55 | 2.82e-05 |
| high incidence male progeny (RNAi) | 17 | 5.55 | 2.82e-05 |
| X chromosome nondisjunction (RNAi) | 17 | 5.55 | 2.82e-05 |
| aneuploidy (RNAi) | 17 | 5.46 | 3.53e-05 |
| pronuclear migration defective early emb (RNAi) | 14 | 6.46 | 7.32e-05 |
| chromosome segregation variant karyomeres early emb (RNAi) | 10 | 9.56 | 1.40e-04 |
| pronuclear size defective early emb (RNAi) | 10 | 9.12 | 2.18e-04 |
| receptor mediated endocytosis defective (RNAi) | 45 | 2.33 | 2.52e-04 |
| maternal sterile (RNAi) | 58 | 2.06 | 2.57e-04 |
| hermaphrodite sterile (RNAi) | 58 | 2.05 | 2.62e-04 |
| hermaphrodite fertility reduced (RNAi) | 58 | 2.05 | 2.62e-04 |
| oocyte physiology variant (RNAi) | 45 | 2.32 | 2.93e-04 |
| exaggerated asynchrony early emb (RNAi) | 9 | 10.08 | 3.52e-04 |
| cell cell contacts defective early emb (RNAi) | 10 | 8.52 | 3.97e-04 |
| cell cell contacts variant four emb (RNAi) | 10 | 8.52 | 3.97e-04 |
| sterile | 65 | 1.92 | 4.81e-04 |
| embryonic cell physiology variant | 10 | 8.17 | 5.91e-04 |
| embryonic cell organization biogenesis variant | 10 | 8.17 | 5.91e-04 |
| fertility reduced | 65 | 1.90 | 6.03e-04 |
| endocytic transport defect (RNAi) | 45 | 2.25 | 6.30e-04 |
| vulval cell induction variant (RNAi) | 19 | 3.96 | 6.96e-04 |
| endocytic transport variant (RNAi) | 45 | 2.24 | 7.12e-04 |
| spindle assembly defective early emb (RNAi) | 7 | 13.72 | 7.68e-04 |
| vulva cell fate specification variant (RNAi) | 19 | 3.84 | 1.07e-03 |
| pseudocleavage defective early emb (RNAi) | 8 | 10.46 | 1.12e-03 |
| fertility variant | 68 | 1.83 | 1.12e-03 |
| reproductive system physiology variant | 68 | 1.83 | 1.17e-03 |
| mitotic spindle defective early emb | 8 | 10.12 | 1.42e-03 |
| organ system development variant (RNAi) | 46 | 2.15 | 1.44e-03 |
| embryonic lethal | 30 | 2.70 | 1.47e-03 |
| transgene silencing variant (RNAi) | 14 | 4.95 | 1.55e-03 |
| multivulva (RNAi) | 17 | 4.06 | 1.78e-03 |
| vulva development variant (RNAi) | 19 | 3.69 | 1.83e-03 |
| vulval cell induction increased (RNAi) | 17 | 4.02 | 2.07e-03 |
| organ system physiology variant | 68 | 1.79 | 2.31e-03 |
| absolute cell cycle timing defective early emb (RNAi) | 9 | 7.67 | 3.20e-03 |
| cell cycle slow early emb (RNAi) | 9 | 7.67 | 3.20e-03 |
| germline proliferation variant (RNAi) | 16 | 4.02 | 3.87e-03 |
| cell fate specification variant (RNAi) | 19 | 3.48 | 3.97e-03 |
| pattern of transgene expression variant (RNAi) | 57 | 1.88 | 4.21e-03 |
| spindle defective early emb | 8 | 8.71 | 4.22e-03 |
| pronuclear number defective early emb (RNAi) | 9 | 7.20 | 5.30e-03 |
| larval lethal (RNAi) | 47 | 2.01 | 5.80e-03 |
| reduced brood size (RNAi) | 48 | 1.99 | 5.94e-03 |
| ectopic expression transgene (RNAi) | 10 | 6.22 | 5.95e-03 |
| physiology variant | 95 | 1.56 | 6.13e-03 |
| physiology variant (RNAi) | 192 | 1.29 | 6.14e-03 |
| brood size variant (RNAi) | 48 | 1.99 | 6.17e-03 |
| transgene expression variant (RNAi) | 66 | 1.75 | 6.59e-03 |
| RNAi resistant (RNAi) | 13 | 4.51 | 8.44e-03 |
| Variant (RNAi) | 228 | 1.24 | 8.52e-03 |
| excess maternal pronucleus early emb (RNAi) | 8 | 7.84 | 8.68e-03 |
| RNAi response variant (RNAi) | 13 | 4.47 | 9.19e-03 |
| pseudocleavage exaggerated early emb (RNAi) | 5 | 16.34 | 9.75e-03 |
| lethal | 75 | 1.65 | 1.06e-02 |
| locomotion variant (RNAi) | 67 | 1.71 | 1.07e-02 |
| movement variant (RNAi) | 67 | 1.71 | 1.13e-02 |
| mitotic chromosome segregation variant (RNAi) | 10 | 5.68 | 1.22e-02 |
| organism development variant | 81 | 1.60 | 1.26e-02 |
| cell position defective early emb (RNAi) | 6 | 11.20 | 1.34e-02 |
| somatic transgene silencing variant (RNAi) | 12 | 4.61 | 1.41e-02 |
| excess pronucleus early emb (RNAi) | 8 | 7.29 | 1.42e-02 |
| organism morphology variant (RNAi) | 56 | 1.80 | 1.43e-02 |
| cell organization biogenesis variant | 11 | 4.96 | 1.60e-02 |
| embryonic development variant | 30 | 2.35 | 1.74e-02 |
| mitosis variant (RNAi) | 8 | 6.82 | 2.18e-02 |
| cell cycle variant | 6 | 9.80 | 2.73e-02 |
| reproductive system development variant (RNAi) | 20 | 2.85 | 3.04e-02 |
| aster defective early emb (RNAi) | 6 | 9.05 | 4.17e-02 |
| cell physiology variant | 26 | 2.39 | 4.20e-02 |
| organism metabolism processing variant (RNAi) | 88 | 1.50 | 4.65e-02 |

### Anatomy terms enriched

|  |  |  |  |
| --- | --- | --- | --- |
| **Group name** | **Number in cluster** | **Enrichment** | **FDR corrected p** |
| Tissue | 37 | 3.56 | 1.56e-07 |
| germ line | 37 | 3.56 | 1.56e-07 |
| Z3 | 10 | 9.34 | 2.18e-04 |
| Z2 | 10 | 9.34 | 2.18e-04 |
| AB | 9 | 9.05 | 1.14e-03 |
| germline precursor cell | 18 | 3.81 | 2.91e-03 |
| QR | 7 | 11.44 | 3.65e-03 |
| QL | 7 | 10.56 | 6.35e-03 |
| Psub1 | 8 | 8.25 | 8.79e-03 |
| Caaaa | 5 | 15.08 | 2.15e-02 |
| Cappp | 5 | 15.08 | 2.15e-02 |
| Cpaaa | 5 | 15.08 | 2.15e-02 |
| Caaap | 5 | 15.08 | 2.15e-02 |
| Capap | 5 | 15.08 | 2.15e-02 |
| Cpaap | 5 | 15.08 | 2.15e-02 |
| Cappa | 5 | 15.08 | 2.15e-02 |
| Capaa | 5 | 15.08 | 2.15e-02 |
| Caapp | 5 | 15.08 | 2.15e-02 |
| Cpapp | 5 | 14.00 | 3.08e-02 |
| Cpapa | 5 | 14.00 | 3.08e-02 |
| Cppaa | 5 | 14.00 | 3.08e-02 |
| Cpppa | 5 | 14.00 | 3.08e-02 |
| Cpppp | 5 | 14.00 | 3.08e-02 |
| Cpaa | 5 | 14.00 | 3.08e-02 |
| Caaa | 5 | 14.00 | 3.08e-02 |
| Cppap | 5 | 14.00 | 3.08e-02 |
| ABa | 7 | 8.07 | 3.44e-02 |
| ABp | 7 | 8.07 | 3.44e-02 |
| ABarpaapa | 5 | 13.07 | 4.02e-02 |
| ABarppap | 5 | 13.07 | 4.02e-02 |
| Caap | 5 | 13.07 | 4.02e-02 |
| ABalapppp | 5 | 13.07 | 4.02e-02 |
| Caapa | 5 | 13.07 | 4.02e-02 |
| ABalapaa | 5 | 13.07 | 4.02e-02 |
| ABarapapp | 5 | 13.07 | 4.02e-02 |
| ABalappa | 5 | 13.07 | 4.02e-02 |
| Cppa | 5 | 13.07 | 4.02e-02 |
| ABalapaap | 5 | 13.07 | 4.02e-02 |
| ABalapaaa | 5 | 13.07 | 4.02e-02 |
| ABalappp | 5 | 13.07 | 4.02e-02 |
| ABarpappp | 5 | 13.07 | 4.02e-02 |
| ABarapppa | 5 | 13.07 | 4.02e-02 |
| ABarpaaa | 5 | 13.07 | 4.02e-02 |
| Capa | 5 | 13.07 | 4.02e-02 |
| ABarppaa | 5 | 13.07 | 4.02e-02 |
| ABalapppa | 5 | 13.07 | 4.02e-02 |
| ABarpapp | 5 | 13.07 | 4.02e-02 |
| ABarpaap | 5 | 13.07 | 4.02e-02 |
| ABarpppp | 5 | 13.07 | 4.02e-02 |
| ABalapap | 5 | 13.07 | 4.02e-02 |
| ABarapppp | 5 | 13.07 | 4.02e-02 |
| ABarappap | 5 | 13.07 | 4.02e-02 |
| ABarpapa | 5 | 13.07 | 4.02e-02 |
| ABarpaapp | 5 | 13.07 | 4.02e-02 |
| Cpap | 5 | 13.07 | 4.02e-02 |
| ABarapapa | 5 | 13.07 | 4.02e-02 |
| ABarpppa | 5 | 13.07 | 4.02e-02 |
| ABplapaap | 5 | 12.25 | 4.88e-02 |
| ABarapaaa | 5 | 12.25 | 4.88e-02 |
| ABarpapap | 5 | 12.25 | 4.88e-02 |
| ABprpppa | 5 | 12.25 | 4.88e-02 |
| ABalppaa | 5 | 12.25 | 4.88e-02 |
| ABprapaaa | 5 | 12.25 | 4.88e-02 |
| ABarapaap | 5 | 12.25 | 4.88e-02 |
| ABalaaap | 5 | 12.25 | 4.88e-02 |
| ABprppaa | 5 | 12.25 | 4.88e-02 |
| ABarappa | 5 | 12.25 | 4.88e-02 |
| ABplppap | 5 | 12.25 | 4.88e-02 |
| ABarpppaa | 5 | 12.25 | 4.88e-02 |
| ABarppaap | 5 | 12.25 | 4.88e-02 |
| ABalppppp | 5 | 12.25 | 4.88e-02 |
| ABplappap | 5 | 12.25 | 4.88e-02 |
| ABalpppp | 5 | 12.25 | 4.88e-02 |
| ABplappa | 5 | 12.25 | 4.88e-02 |
| Capp | 5 | 12.25 | 4.88e-02 |
| ABalppap | 5 | 12.25 | 4.88e-02 |
| ABalaapa | 5 | 12.25 | 4.88e-02 |
| ABalapapp | 5 | 12.25 | 4.88e-02 |
| ABalaaaar | 5 | 12.25 | 4.88e-02 |
| ABprapaa | 5 | 12.25 | 4.88e-02 |
| ABalappap | 5 | 12.25 | 4.88e-02 |
| ABplppaa | 5 | 12.25 | 4.88e-02 |
| ABprpppp | 5 | 12.25 | 4.88e-02 |
| ABarppppp | 5 | 12.25 | 4.88e-02 |
| ABprappap | 5 | 12.25 | 4.88e-02 |
| ABarppapp | 5 | 12.25 | 4.88e-02 |
| ABarpppap | 5 | 12.25 | 4.88e-02 |
| Cppp | 5 | 12.25 | 4.88e-02 |
| ABprappa | 5 | 12.25 | 4.88e-02 |
| ABarpappa | 5 | 12.25 | 4.88e-02 |
| ABprapapp | 5 | 12.25 | 4.88e-02 |
| ABarppppa | 5 | 12.25 | 4.88e-02 |
| ABalapapa | 5 | 12.25 | 4.88e-02 |
| ABalppppa | 5 | 12.25 | 4.88e-02 |
| ABarapap | 5 | 12.25 | 4.88e-02 |
| ABprppap | 5 | 12.25 | 4.88e-02 |
| ABprappaa | 5 | 12.25 | 4.88e-02 |
| ABplappaa | 5 | 12.25 | 4.88e-02 |
| ABalappaa | 5 | 12.25 | 4.88e-02 |
| ABalaapaa | 5 | 12.25 | 4.88e-02 |
| ABarpapaa | 5 | 12.25 | 4.88e-02 |
| ABplapapp | 5 | 12.25 | 4.88e-02 |
| ABplpppa | 5 | 12.25 | 4.88e-02 |
| ABalaaaa | 5 | 12.25 | 4.88e-02 |
| ABplappp | 5 | 12.25 | 4.88e-02 |
| ABplapaaa | 5 | 12.25 | 4.88e-02 |
| ABarpaaap | 5 | 12.25 | 4.88e-02 |
| ABprapapa | 5 | 12.25 | 4.88e-02 |
| ABarapaa | 5 | 12.25 | 4.88e-02 |
| ABarappp | 5 | 12.25 | 4.88e-02 |
| ABarppapa | 5 | 12.25 | 4.88e-02 |
| ABalaapp | 5 | 12.25 | 4.88e-02 |
| ABalaapap | 5 | 12.25 | 4.88e-02 |
| ABarpaaaa | 5 | 12.25 | 4.88e-02 |
| ABplpppp | 5 | 12.25 | 4.88e-02 |
| ABalpppa | 5 | 12.25 | 4.88e-02 |
| ABarappaa | 5 | 12.25 | 4.88e-02 |
| ABplapapa | 5 | 12.25 | 4.88e-02 |
| ABprapap | 5 | 12.25 | 4.88e-02 |
| ABalaappp | 5 | 12.25 | 4.88e-02 |
| ABalaaapa | 5 | 12.25 | 4.88e-02 |
| ABprappp | 5 | 12.25 | 4.88e-02 |
| ABalaaapp | 5 | 12.25 | 4.88e-02 |
| ABalaaaal | 5 | 12.25 | 4.88e-02 |
| ABarppaaa | 5 | 12.25 | 4.88e-02 |

### GO terms enriched

|  |  |  |
| --- | --- | --- |
| **GO term** | **Number of genes** | **FDR-corrected p-value** |
| embryo development ending in birth or egg hatching | 156 | 1.6e-23 |
| organelle fission | 53 | 9.4e-19 |
| reproduction | 113 | 7.5e-17 |
| hermaphrodite genitalia development | 66 | 3.1e-16 |
| sex differentiation | 62 | 3.2e-16 |
| organelle part | 77 | 7.6e-16 |
| reproductive structure development | 71 | 1.3e-15 |
| cytokinesis | 31 | 1.3e-14 |
| cell division | 42 | 2.4e-13 |
| cell | 137 | 3.3e-13 |
| multicellular organismal development | 89 | 1.1e-12 |
| organ development | 59 | 5.6e-12 |
| multicellular organismal process | 181 | 1.4e-11 |
| single organism reproductive process | 67 | 3.1e-11 |
| mitotic spindle organization | 20 | 5.7e-11 |
| DNA replication | 16 | 8.0e-10 |
| anatomical structure development | 70 | 7.7e-08 |
| nucleus | 56 | 9.1e-08 |
| microtubule cytoskeleton | 15 | 1.0e-07 |
| cellular component organization | 54 | 2.4e-07 |
| intracellular non-membrane-bounded organelle | 42 | 3.5e-07 |
| spindle midzone | 8 | 1.3e-06 |
| mitosis | 16 | 1.4e-06 |
| membrane-bounded organelle | 94 | 1.8e-06 |
| protein binding | 55 | 5.7e-06 |
| nucleic acid metabolic process | 68 | 9.1e-06 |
| macromolecular complex subunit organization | 15 | 1.7e-05 |
| morphogenesis of an epithelium | 32 | 2.1e-05 |
| mitotic sister chromatid segregation | 8 | 3.1e-05 |
| intracellular organelle lumen | 25 | 3.6e-05 |
| cellular aromatic compound metabolic process | 81 | 5.3e-05 |
| heterocycle metabolic process | 81 | 5.4e-05 |
| membrane-enclosed lumen | 25 | 6.7e-05 |
| organic cyclic compound metabolic process | 81 | 1.2e-04 |
| cellular nitrogen compound metabolic process | 81 | 1.2e-04 |
| meiotic chromosome segregation | 17 | 1.4e-04 |
| cellular process | 171 | 1.9e-04 |
| condensed chromosome kinetochore | 6 | 1.9e-04 |
| kinetochore | 6 | 2.6e-04 |
| condensed nuclear chromosome | 10 | 2.8e-04 |
| mitotic cell cycle phase transition | 7 | 3.1e-04 |
| centrosome cycle | 6 | 3.2e-04 |
| microtubule organizing center organization | 6 | 3.2e-04 |
| centrosome | 9 | 3.6e-04 |
| tissue development | 33 | 5.4e-04 |
| cellular macromolecule metabolic process | 91 | 7.5e-04 |
| intracellular part | 14 | 7.5e-04 |
| intracellular organelle | 49 | 7.9e-04 |
| chromosome organization | 16 | 1.5e-03 |
| DNA helicase activity | 6 | 1.9e-03 |
| receptor-mediated endocytosis | 39 | 2.5e-03 |
| chromosome segregation | 7 | 2.6e-03 |
| DNA recombination | 8 | 3.5e-03 |
| RNA interference | 14 | 3.9e-03 |
| protein complex biogenesis | 20 | 4.6e-03 |
| regulation of mitotic cell cycle | 10 | 5.3e-03 |
| posttranscriptional gene silencing | 14 | 5.6e-03 |
| negative regulation of gene expression | 18 | 6.8e-03 |
| gene silencing by RNA | 14 | 7.0e-03 |
| condensed chromosome | 5 | 7.1e-03 |
| macromolecular complex assembly | 22 | 7.5e-03 |
| nucleosome | 6 | 8.0e-03 |
| meiotic nuclear division | 15 | 8.7e-03 |
| protein-DNA complex | 6 | 8.9e-03 |
| regulation of nematode larval development | 19 | 9.5e-03 |
| DNA packaging | 6 | 1.0e-02 |
| protein kinase binding | 8 | 1.1e-02 |
| negative regulation of vulval development | 16 | 1.1e-02 |
| nucleosome assembly | 6 | 1.1e-02 |
| DNA repair | 10 | 1.2e-02 |
| negative regulation of post-embryonic development | 16 | 1.2e-02 |
| nucleotide binding | 47 | 1.2e-02 |
| post-embryonic organ development | 22 | 1.3e-02 |
| regulation of multicellular organismal development | 22 | 1.5e-02 |
| vesicle-mediated transport | 44 | 1.6e-02 |
| maintenance of protein location in cell | 5 | 1.7e-02 |
| DNA-directed DNA polymerase activity | 4 | 1.8e-02 |
| chromatin assembly or disassembly | 6 | 2.0e-02 |
| DNA duplex unwinding | 4 | 2.5e-02 |
| organelle localization | 13 | 2.6e-02 |
| DNA-dependent ATPase activity | 5 | 3.2e-02 |
| negative regulation of metabolic process | 18 | 3.4e-02 |
| polar body extrusion after meiotic divisions | 4 | 3.6e-02 |
| ATP binding | 33 | 3.7e-02 |
| hydrolase activity, acting on acid anhydrides, in phosphorus-containing anhydrides | 22 | 4.2e-02 |
| nucleotidyltransferase activity | 7 | 4.5e-02 |
| cellular response to stress | 14 | 4.8e-02 |
| gastrulation with mouth forming first | 8 | 5.0e-02 |

### Expression clusters enriched

|  |  |  |  |
| --- | --- | --- | --- |
| **Group name** | **Number in cluster** | **Enrichment** | **FDR corrected p** |
| Genes upregulated by CYE-1/CDK-2AF for more than two fold. | 107 | 19.16 | 1.96e-109 |
| Genes upregulated by CYD-1/CDK-4 for more than two fold. | 112 | 11.17 | 1.01e-83 |
| TGF- Dauer pathway adult transcriptional targets. Results obtained by comparing the microarray results of the dauer-constitutive mutants daf-7(e1372), daf-7(m62), and daf-1(m40) with dauer-defective mutants daf-3(mgDf90), daf-5(e1386), and daf-7(e1372);daf-3(mgDf90) double mutants at the permissive temperature, 20C, on the first day of adulthood. WBPaper00031040:TGF-beta\_adult\_downregulated | 272 | 2.54 | 5.79e-64 |
| Genes down regulated by mir-243(n4759). | 170 | 3.51 | 1.36e-50 |
| Embryonic class (E): genes that significantly increase in abundance at some point during embryogenesis. | 219 | 2.41 | 3.81e-41 |
| Maternal-embryonic class (ME): genes that are in the intersection of the maternal and embryonic classes. | 182 | 2.70 | 4.09e-38 |
| Caenorhabditis elegans Genes with expression levels changed significantly after treatment of Bacillus thurigiensis DB27. | 250 | 2.05 | 4.65e-37 |
| FBF-associated probe sets (FDR <2.25%) | 217 | 2.26 | 5.73e-36 |
| Candidate daf-19 up regulated genes with a statistically significant signal variation of 1.5-fold or greater. These were identified using a class comparisons tool from BRB Array Tools. | 66 | 6.88 | 4.54e-33 |
| Expression Pattern Group B, enriched for genes involved in embryonic development. These patterns have in common that they all have genes of which the expression goes up after the juvenile stage. The expression of the genes in these patterns remains high or even goes up after reproduction. | 124 | 3.43 | 5.12e-33 |
| Caenorhabditis elegans Genes with expression levels changed significantly after treatment of Xenorhabdus nematophila. | 294 | 1.65 | 2.53e-29 |
| Genes with expression level up in lin-35 mutant background. | 64 | 5.92 | 4.90e-28 |
| Caenorhabditis elegans Genes with expression levels changed significantly after treatment of Serratia marcescens. | 193 | 2.14 | 7.68e-27 |
| oogenesis-enriched | 95 | 3.53 | 5.57e-25 |
| Maternal class (M): genes that are called present in at least one of the three PC6 replicates. | 255 | 1.72 | 1.81e-24 |
| Genes that were upregulated in lin-35. | 73 | 4.32 | 1.31e-23 |
| Germline-enriched and sex-biased expression profile cluster E. | 99 | 3.26 | 1.66e-23 |
| Developmentally modulated gene cluster. cgc4386\_cluster\_6\_2 | 38 | 7.97 | 2.06e-20 |
| Genes depleted in muscle cells (24hr muscle dataset). Dissociated myo-3::GFP embryos were cultured for 24 hours before FACS sorting. | 110 | 2.71 | 4.32e-20 |
| Genes down-regulated after 300 um Tannic acid treatment. Fold change < 0.8. | 108 | 2.75 | 4.88e-20 |
| Gene significantly down-regulated by treatment with 2.0mM of HuminFeed until young adult stage (3 days), with a minimum fold change in gene expression of 0.8. | 119 | 2.53 | 1.29e-19 |
| Total muscle depleted genes (complete list of non-overlapping genes from the 0hr and 24hr muscle depleted datasets). | 142 | 2.24 | 2.96e-19 |
| Germline-intrinsic transcripts. | 92 | 2.87 | 1.03e-17 |
| Germline-enriched and sex-biased expression profile cluster F. | 65 | 3.60 | 1.51e-16 |
| 948 reproductively enriched mRNAs that co-immunoprecipitate with GLD-1. To identify GLD-1 mRNA targets, authors performed immunoprecipitation (IP) of GLD-1, followed by microarray analysis of the co-IPed mRNAs (RIP-chip). Extracts from young adult transgenic worms expressing a rescuing FLAG and GFP-tagged GLD-1, hereafter referred to as tagged GLD-1, were subjected to IP in triplicate with anti-FLAG (aFLAG IP) or anti-MYC (aMYC IP) antibodies as controls. Comparison of aFLAG IP versus aMYC IP to input revealed a large population of GLD-1-associated transcripts. Authors additionally performed complementary aFLAG IPs upon worms expressing either tagged GLD-1(GGF IP) or non-tagged GLD-1(N2 IP). Comparing transcript IP-enrichment values from both approaches revealed a correlation of 0.96, which indicated high reproducibility of GLD-1 association with specific mRNAs even on a quantitative level. | 72 | 3.19 | 1.21e-15 |
| Embryonic (E) subclasses are based on the earliest significant increase(abbreviated pi for primary increase). [cgc5767]:expression\_class\_E\_pi(23\_min) | 66 | 2.87 | 6.28e-12 |
| Genes up or down regulated by 10e-07M of progesterone. The normalized values used were G/R ratio > 2.6 for up-regulation and G/R ratio < 0.38 for down-regulation, which corresponds to 1.39 and -1.39 log(base2) G/R ratio, respectively. | 153 | 1.77 | 1.55e-11 |
| Developmentally modulated gene cluster. cgc4386\_cluster\_6\_1 | 25 | 6.95 | 1.67e-11 |
| Embryonic (E) subclasses are based on the earliest significant increase(abbreviated pi for primary increase). [cgc5767]:expression\_class\_E\_pi(53\_min) | 63 | 2.85 | 3.29e-11 |
| Early embryonic development gene expression profile. [cgc5767]:cluster\_2 | 41 | 3.79 | 1.80e-10 |
| Genome-wide analysis of developmental and sex-regulated gene expression profile. cgc4489\_group\_2 | 88 | 2.23 | 2.79e-10 |
| Significantly downregulated genes from cyc-1(RNAi) microarrays using SAM algorithm with an FDR < 0.1 from adult-only chips. | 146 | 1.72 | 9.71e-10 |
| Genes depleted in muscle cells (0hr muscle dataset). Dissociated myo-3::GFP embryos were cultured for 0 hours before FACS sorting. | 92 | 2.11 | 1.39e-09 |
| Developmentally modulated gene cluster. cgc4386\_cluster\_5\_2 | 19 | 8.01 | 1.48e-09 |
| Embryonic (E) subclasses are based on the earliest significant increase(abbreviated pi for primary increase). [cgc5767]:expression\_class\_E\_pi(41\_min) | 36 | 3.95 | 1.54e-09 |
| Genes downregulated on Comamonas DA1877 relative to E. coli OP50, Gravid adult stage | 38 | 3.63 | 4.84e-09 |
| C-lineage related expression profile. WBPaper00025032:cluster\_2 | 26 | 4.47 | 1.16e-07 |
| Maternal-embryonic transient class (MET): genes that are in the intersection of the maternal and embryonic transient classes. | 55 | 2.41 | 7.10e-07 |
| Genes with changed expression in lin-54(n2290) embryo. | 58 | 2.33 | 7.98e-07 |
| Genes showing > 1.5-fold up-regulated expression (p<0.001) both in aex-3::His-SUMO-1 and myo-4::His-SUMO-1 C. elegans. | 30 | 3.59 | 9.04e-07 |
| C-lineage related expression profile. WBPaper00025032:cluster\_7 | 18 | 5.78 | 1.13e-06 |
| Expression Pattern Group F, enriched for genes involved in embryonic development. These patterns have in common that they all have genes of which the expression goes up after the juvenile stage. The expression of the genes in these patterns remains high or even goes up after reproduction. | 102 | 1.77 | 1.31e-06 |
| Genes expressed in N2. | 282 | 1.25 | 1.92e-06 |
| C-lineage related expression profile. WBPaper00025032:cluster\_58 | 9 | 14.11 | 3.27e-06 |
| Embryonic transient class (ET): genes that are the subset of embryonic genes in which the latest significant increase is earlier than their latest significant decrease. | 69 | 2.05 | 3.34e-06 |
| Genes up or down regulated by 10e-09M of testosterone. The normalized values used were G/R ratio > 2.6 for up-regulation and G/R ratio < 0.38 for down-regulation, which corresponds to 1.39 and -1.39 log(base2) G/R ratio, respectively. | 145 | 1.53 | 4.16e-06 |
| Genes upregulated more than 2-fold by fasting for 48 hours in N2 and in kgb-1(km21). | 34 | 2.92 | 1.20e-05 |
| Genes for which heat shock F3 (fraction 3, containing heavy polysomes) versus control F3 is significantly increased. | 43 | 2.51 | 1.31e-05 |
| Candidate daf-19 up regulated genes with a statistically significant signal variation of 1.5-fold or greater. These were identified using a Significance Analysis of Microarrays (SAM). | 12 | 7.84 | 1.47e-05 |
| Genes upregulated more than 2-fold by fasting for 48 hours in N2. | 36 | 2.71 | 2.81e-05 |
| C-lineage related expression profile. WBPaper00025032:cluster\_22 | 11 | 7.99 | 4.22e-05 |
| C-lineage related expression profile. WBPaper00025032:cluster\_17 | 12 | 6.92 | 5.76e-05 |
| Genes upregulated by fasting anytime between 9 hour to 12 hour time course in N2 worms. | 41 | 2.36 | 1.33e-04 |
| Genes upregulated more than 2-fold by fasting for 48 hours in N2 and in daf-16(mu86). | 28 | 2.86 | 2.56e-04 |
| Genes that showed decreased expression after treated with 2-deoxy-D-glucose. | 17 | 4.11 | 3.34e-04 |
| Early embryonic development gene expression profile. [cgc5767]:cluster\_4 | 19 | 3.69 | 4.06e-04 |
| Genes with expression altered >= 3-fold in dpy-10(e128) mutants. | 125 | 1.47 | 5.67e-04 |
| Differentially expressed genes during worm lifespan. Medoid 1 Fig.4. | 26 | 2.82 | 7.48e-04 |
| Embryonic (E) subclasses are based on the earliest significant increase(abbreviated pi for primary increase). [cgc5767]:expression\_class\_E\_pi(66\_min) | 22 | 3.08 | 1.10e-03 |
| Gene significantly down-regulated by treatment with 0.2mM of HuminFeed until young adult stage (3 days), with a minimum fold change in gene expression of 0.8. | 25 | 2.79 | 1.35e-03 |
| Genes significantly enriched (> 2x, FDR < 5%) in a particular cell-type versus a reference sample of all cells at the same stage. WBPaper00037950:hypodermis\_embryo\_enriched | 40 | 2.14 | 1.69e-03 |
| RNP-8-associated transcripts, based on microarray experiments. | 45 | 2.02 | 1.75e-03 |
| Embryonic transient (ET) subclasses are based on time of max abundance. [cgc5767]:expression\_class\_ET\_max(122\_min) | 13 | 4.51 | 1.98e-03 |
| Genes down-regulated after 200 um Tannic acid treatment. Fold change < 0.8. | 27 | 2.50 | 3.73e-03 |
| Developmentally modulated gene cluster. cgc4386\_cluster\_5\_3 | 13 | 4.18 | 4.24e-03 |
| Genes differentially expressed under EtBr treatment without UVC exposure vs after UVC exposure but without EtBr treatment at the -3h timepoint (3 h after the third UVC dose (51h), which is also 3 h after being placed on food). | 45 | 1.91 | 6.79e-03 |
| DNA metabolism; 6.2e-04(14.60x). | 5 | 13.07 | 7.19e-03 |
| Genes that showed decreased expression after 12 hours of infection by fungi Drechmeria coniospora. | 17 | 3.17 | 7.85e-03 |
| Genes that showed expression levels higher than the corresponding reference sample (Young adult all cell reference). | 197 | 1.25 | 9.24e-03 |
| Genes shown weak response to MPK-1 signaling. | 7 | 7.42 | 9.64e-03 |
| Genes with changed expression in lin-54(n3423) germline. | 22 | 2.64 | 9.78e-03 |
| DNA metabolism; 7.6e-04(13.69x). | 5 | 12.25 | 9.88e-03 |
| Jiang M. et al. group 2; 2.0e-04(3.65x). mitotic cell cycle; 7.1e-04(11.53x). | 7 | 7.22 | 1.13e-02 |
| Genes upregulated by fasting anytime during the 48 hour time course in N2 worms. | 67 | 1.61 | 1.42e-02 |
| Jiang M. et al. group 2; 3.2e-04(3.46x). mitotic cell cycle; 8.3e-04(10.95x). | 7 | 6.86 | 1.52e-02 |
| Significantly upregulated genes from isp-1(qm150) microarrays using SAM algorithm with an FDR < 0.1 from adult-only chips. | 35 | 1.99 | 2.08e-02 |
| Jiang M. et al. group 2; 6.0e-04(3.46x). | 7 | 6.38 | 2.33e-02 |
| Embryonic (E) subclasses are based on the earliest significant increase(abbreviated pi for primary increase). [cgc5767]:expression\_class\_E\_pi(83\_min) | 24 | 2.29 | 3.42e-02 |
| Genes with no change in hcf-1(-), no change in sir-2.1(O/E) and downregulated in daf-2(-). | 35 | 1.90 | 4.77e-02 |

### Motifs enriched

|  |  |  |  |  |  |
| --- | --- | --- | --- | --- | --- |
| **Motif** | **Logo** | **Possible orthologs** | **Number of motifs in cluster** | **Enrichment** | **FDR corrected p** |
| Zfp161\_2858 |  | pzf-1 | 102 | 2.79 | 6.6e-18 |
| pTH5916 |  | efl-2 | 148 | 2.15 | 2.2e-17 |
| pTH9173 |  | efl-2 | 158 | 2.03 | 1.0e-16 |
| MA0541.1 |  | efl-1 (0.87) F49E12.6 | 127 | 2.26 | 7.0e-16 |
| pTH10696 |  | Y44A6D.3 | 220 | 1.55 | 2.5e-12 |
| pTH9393 |  | ZC416.1 | 122 | 1.89 | 7.6e-10 |
| Mw138 |  | ceh-48 dsc-1 | 147 | 1.71 | 2.5e-09 |
| pTH8982 |  | ceh-48 | 127 | 1.68 | 2.9e-07 |
| pTH8399 |  | lin-54 | 87 | 1.89 | 1.8e-06 |
| MA0536.1 |  | elt-1 | 108 | 1.63 | 2.8e-05 |
| E2F4\_1 |  | F49E12.6 | 94 | 1.68 | 5.7e-05 |
| tgo\_ss\_SANGER\_5\_FBgn0015014 |  | aha-1 (0.55) | 137 | 1.48 | 9.1e-05 |
| pTH9958 |  | ztf-6 (0.69) | 240 | 1.26 | 9.9e-05 |
| Mitf\_SANGER\_5\_FBgn0263112 |  | hlh-30 | 137 | 1.45 | 2.0e-04 |
| pTH3220 |  | Y5F2A.4 (0.58) daf-12 | 129 | 1.47 | 2.3e-04 |
| MA0535.1 |  | daf-8 | 138 | 1.44 | 3.1e-04 |
| pTH9097 |  | Y116A8C.22 | 287 | 1.19 | 3.3e-04 |
| pTH8679 |  | pax-2 | 128 | 1.45 | 4.6e-04 |
| MA0146.2 |  | F58G1.2 (0.82) | 95 | 1.56 | 8.8e-04 |
| pTH5059 |  | aha-1 (0.55) lin-22 | 120 | 1.44 | 1.6e-03 |
| pTH3046 |  | Y116A8C.22 | 162 | 1.31 | 3.4e-03 |
| pTH1294 |  | mel-28 (0.93) | 126 | 1.38 | 5.2e-03 |
| pTH2280 |  | mnm-2 | 104 | 1.44 | 5.3e-03 |
| MA0497.1 |  | mef-2 | 253 | 1.18 | 5.3e-03 |
| V$FOXD3\_01 |  | let-381 | 269 | 1.17 | 5.5e-03 |
| pTH9380 |  | mel-28 (0.93) | 243 | 1.19 | 6.8e-03 |
| CXXC1\_si |  | F52B11.1 | 151 | 1.31 | 8.2e-03 |
| pTH9096 |  | T07C12.11 | 82 | 1.51 | 8.7e-03 |
| pTH7876 |  | mel-28 (0.93) lin-29 | 258 | 1.17 | 9.6e-03 |
| V$POU3F2\_01 |  | ceh-18 (-0.73) | 242 | 1.18 | 1.0e-02 |
| Gmeb1\_1745 |  | attf-1 | 86 | 1.48 | 1.1e-02 |
| pTH9180 |  | mel-28 (0.93) | 277 | 1.15 | 1.1e-02 |
| MA0135.1 |  | lim-7 | 74 | 1.53 | 1.2e-02 |
| pTH8745 |  | attf-1 | 80 | 1.50 | 1.3e-02 |
| pTH9211 |  | C01B12.2 | 80 | 1.49 | 1.4e-02 |
| pTH9260 |  | mel-28 (0.93) | 260 | 1.16 | 1.5e-02 |
| Eip93F\_SANGER\_10\_FBgn0013948 |  | mbr-1 | 217 | 1.20 | 1.8e-02 |
| POU3F1\_2 |  | ceh-18 (-0.73) unc-86 | 223 | 1.19 | 2.2e-02 |
| pTH9026 |  | attf-1 | 78 | 1.47 | 2.3e-02 |
| MA0579.1 |  | D1081.8 (0.65) | 80 | 1.46 | 2.3e-02 |
| pTH7032 |  | F52B11.1 | 65 | 1.52 | 2.8e-02 |
| pTH8997 |  | hmg-12 | 234 | 1.17 | 3.1e-02 |
| ZN384\_f1 |  | lin-29 | 284 | 1.13 | 3.1e-02 |
| Ascl2\_2654 |  | hlh-14 | 40 | 1.74 | 3.5e-02 |
| pTH9082 |  | mab-23 | 254 | 1.15 | 3.6e-02 |
| V$TAXCREB\_01 |  | crh-1 | 222 | 1.17 | 3.8e-02 |
| EGR1\_2 |  | ZC328.2 | 59 | 1.54 | 3.9e-02 |
| FOXQ1\_f1 |  | let-381 | 229 | 1.16 | 4.2e-02 |
| pTH5250 |  | C48E7.11 | 70 | 1.46 | 4.7e-02 |
| MA0049.1 |  | hbl-1 | 265 | 1.13 | 5.0e-02 |

### Correlated (and anti-correlated) transcription factors

|  |  |
| --- | --- |
| **Transcription factor** | **Correlation** |
| F26F4.8 | 0.95 |
| Y82E9BR.17 | 0.95 |
| sex-1 | 0.95 |
| Y53F4B.3 | 0.94 |
| dhhc-6 | 0.94 |
| zim-1 | 0.93 |
| mel-28 | 0.93 |
| Y82E9BR.1 | 0.93 |
| efl-3 | 0.91 |
| hmg-5 | 0.91 |
| ekl-4 | 0.91 |
| flh-3 | 0.91 |
| zip-7 | 0.89 |
| dpl-1 | 0.89 |
| F16B12.6 | 0.87 |
| efl-1 | 0.87 |
| duxl-1 | 0.87 |
| R02D3.7 | 0.87 |
| flh-1 | 0.87 |
| pop-1 | 0.86 |
| attf-2 | 0.86 |
| ztf-11 | 0.86 |
| Y52B11A.9 | 0.85 |
| ham-1 | 0.85 |
| egl-44 | 0.84 |
| nhr-149 | -0.58 |
| nhr-135 | -0.58 |
| mxl-3 | -0.59 |
| madf-4 | -0.60 |
| madf-1 | -0.60 |
| gmeb-3 | -0.60 |
| tag-97 | -0.60 |
| ccch-1 | -0.61 |
| egl-38 | -0.62 |
| nhr-31 | -0.62 |
| C35D6.4 | -0.63 |
| jun-1 | -0.63 |
| zip-2 | -0.65 |
| mbf-1 | -0.66 |
| T18D3.7 | -0.67 |
| nhr-14 | -0.67 |
| nhr-105 | -0.69 |
| nhr-222 | -0.69 |
| nhr-41 | -0.70 |
| zfh-2 | -0.70 |
| ceh-88 | -0.72 |
| zip-1 | -0.72 |
| ceh-18 | -0.73 |
| saeg-1 | -0.74 |
| mdl-1 | -0.78 |

### ChIP peaks enriched

|  |  |  |  |  |
| --- | --- | --- | --- | --- |
| **Gene** | **Experiment** | **Number of upstream peaks** | **Enrichment** | **FDR corrected p** |
| ceh-39 | CEH-39\_Embryos | 184 | 4.55 | 6.3e-73 |
| dpl-1 | DPL-1\_Fed-L1-stage-larvae | 228 | 3.33 | 1.7e-69 |
| efl-1 | EFL-1\_Fed-L1-stage-larvae | 225 | 3.35 | 1.3e-68 |
| efl-1 | EFL-1\_Young-adult | 249 | 2.97 | 2.0e-68 |
| lin-35 | LIN-35\_Fed-L1-stage-larvae | 220 | 3.34 | 3.7e-66 |
| dpl-1 | DPL-1\_Young-adult | 218 | 3.35 | 1.2e-65 |
| efl-1 | EFL-1\_Larvae-L1-stage | 236 | 3.06 | 1.2e-65 |
| F16B12.6 | F16B12.6\_Fed-L1-stage-larvae | 147 | 4.64 | 2.0e-56 |
| lsy-2 | LSY-2\_Embryos | 184 | 3.51 | 2.9e-55 |
| lin-15 | LIN-15B\_Fed-L1-stage-larvae | 158 | 4.09 | 3.5e-54 |
| dpl-1 | DPL-1\_Larvae-L4-stage | 248 | 2.43 | 2.4e-50 |
| lsy-2 | LSY-2\_Fed-L1-stage-larvae | 203 | 2.82 | 2.4e-47 |
| pes-1 | PES-1\_Larvae-L4-stage | 207 | 2.65 | 2.8e-44 |
| nfya-1 | NFYA-1\_Late-Embryos | 191 | 2.80 | 4.9e-43 |
| gei-11 | GEI-11\_Fed-L1-stage-larvae | 195 | 2.71 | 4.1e-42 |
| C34F6.9 | C34F6.9\_Larvae-L2-stage | 202 | 2.58 | 9.0e-41 |
| C01B12.2 | C01B12.2\_Larvae-L2-stage | 236 | 2.25 | 1.1e-40 |
| C16A3.4 | C16A3.4\_Fed-L1-stage-larvae | 163 | 3.07 | 8.4e-40 |
| W03F9.2 | W03F9.2\_L4-Young-Adult-stage-larvae | 254 | 2.04 | 6.6e-38 |
| ham-1 | HAM-1\_Fed-L1-stage-larvae | 192 | 2.53 | 8.7e-37 |
| hpl-2 | HPL-2\_Fed-L1-stage-larvae | 216 | 2.30 | 8.8e-37 |
| nfya-1 | NFYA-1\_Larvae-L3-stage | 168 | 2.83 | 1.0e-36 |
| F45C12.2 | F45C12.2\_Fed-L1-stage-larvae | 174 | 2.71 | 5.5e-36 |
| lsy-2 | LSY-2\_Larvae-L1-stage | 213 | 2.28 | 1.5e-35 |
| lin-35 | LIN-35\_Young-adult | 156 | 2.83 | 1.9e-33 |
| R02D3.7 | R02D3.7\_Larvae-L3-stage | 191 | 2.38 | 8.7e-33 |
| nhr-23 | NHR-23\_Larvae-L3-stage | 171 | 2.55 | 9.1e-32 |
| eor-1 | EOR-1\_Larvae-L3-stage | 189 | 2.35 | 1.6e-31 |
| R02D3.7 | R02D3.7\_Larvae-L2-stage | 120 | 3.26 | 1.4e-29 |
| fos-1 | FOS-1\_Fed-L1-stage-larvae | 170 | 2.44 | 2.5e-29 |
| pha-4 | PHA-4\_Larvae-L2-stage | 192 | 2.20 | 1.8e-28 |
| nhr-77 | NHR-77\_Fed-L1-stage-larvae | 171 | 2.39 | 2.5e-28 |
| aly-2 | ALY-2\_Fed-L1-stage-larvae | 143 | 2.67 | 2.4e-27 |
| gei-11 | GEI-11\_Larvae-L3-stage | 172 | 2.32 | 3.7e-27 |
| nhr-77 | NHR-77\_Larvae-L4-stage | 219 | 1.93 | 7.7e-26 |
| nhr-6 | NHR-6\_Larvae-L4-stage | 120 | 2.95 | 9.1e-26 |
| nhr-2 | NHR-2\_Embryos | 92 | 3.60 | 8.1e-25 |
| nhr-237 | NHR-237\_Embryos | 98 | 3.33 | 4.9e-24 |
| lin-13 | LIN-13\_Larvae-L2-stage | 133 | 2.60 | 7.5e-24 |
| gei-11 | GEI-11\_Larvae-L2-stage | 138 | 2.52 | 1.3e-23 |
| nhr-129 | NHR-129\_Larvae-L2-stage | 204 | 1.93 | 3.3e-23 |
| lin-15 | LIN-15B\_Larvae-L4-stage | 72 | 4.24 | 7.7e-23 |
| F23B12.7 | F23B12.7\_Young-adult | 119 | 2.74 | 9.5e-23 |
| lsy-2 | LSY-2\_Larvae-L2-stage | 98 | 3.19 | 1.1e-22 |
| jun-1 | JUN-1\_Larvae-L1-stage | 133 | 2.36 | 4.9e-20 |
| nhr-25 | NHR-25\_Larvae-L2-stage | 136 | 2.21 | 4.0e-18 |
| alr-1 | ALR-1\_Larvae-L2-stage | 141 | 2.16 | 5.3e-18 |
| ces-1 | CES-1\_Embryos | 157 | 2.02 | 5.8e-18 |
| ceh-38 | CEH-38\_Larvae-L3-stage | 137 | 2.07 | 8.7e-16 |
| gei-11 | GEI-11\_Young-adult | 90 | 2.67 | 1.1e-15 |
| skn-1 | SKN-1\_Larvae-L3-stage | 82 | 2.84 | 1.2e-15 |
| lin-13 | LIN-13\_Larvae-L1-stage | 61 | 3.56 | 1.5e-15 |
| F45C12.2 | F45C12.2\_Larvae-L2-stage | 67 | 3.15 | 1.3e-14 |
| ham-1 | HAM-1\_Larvae-L4-stage | 153 | 1.88 | 2.3e-14 |
| lin-13 | LIN-13\_Larvae-L4-stage | 94 | 2.44 | 4.9e-14 |
| nhr-77 | NHR-77\_Larvae-L2-stage | 87 | 2.49 | 2.1e-13 |
| elt-3 | ELT-3\_Embryos | 100 | 2.21 | 1.7e-12 |
| dve-1 | DVE-1\_Late-Embryos | 121 | 2.00 | 1.9e-12 |
| ceh-26 | CEH-26\_Late-Embryonic-stage | 100 | 2.18 | 4.3e-12 |
| sax-3 | SAX-3\_Larvae-L4-stage | 154 | 1.76 | 4.5e-12 |
| R02D3.7 | R02D3.7\_Larvae-L4-stage | 77 | 2.51 | 7.8e-12 |
| hlh-30 | HLH-30\_Late-Embryos | 76 | 2.52 | 8.6e-12 |
| gei-11 | GEI-11\_Embryos | 60 | 2.94 | 1.0e-11 |
| nhr-77 | NHR-77\_Larvae-L3-stage | 105 | 2.06 | 3.2e-11 |
| zag-1 | ZAG-1\_Fed-L1-stage-larvae | 67 | 2.64 | 4.2e-11 |
| pax-1 | PAX-1\_Embryos | 49 | 3.21 | 9.6e-11 |
| ceh-16 | CEH-16\_Larvae-L2-stage | 63 | 2.69 | 9.8e-11 |
| egl-5 | EGL-5\_Larvae-L3-stage | 104 | 2.02 | 1.1e-10 |
| mab-5 | MAB-5\_Larvae-L2-stage | 72 | 2.47 | 1.1e-10 |
| zag-1 | ZAG-1\_Larvae-L2-stage | 96 | 2.08 | 2.0e-10 |
| nhr-237 | NHR-237\_Larvae-L1-stage | 53 | 2.90 | 4.8e-10 |
| F45C12.2 | F45C12.2\_Larvae-L3-stage | 69 | 2.42 | 8.9e-10 |
| aly-2 | ALY-2\_Larvae-L3-stage | 76 | 2.29 | 9.0e-10 |
| sem-4 | SEM-4\_Larvae-L2-stage | 132 | 1.74 | 1.4e-09 |
| zag-1 | ZAG-1\_Larvae-L3-stage | 63 | 2.49 | 2.3e-09 |
| nhr-6 | NHR-6\_Larvae-L2-stage | 122 | 1.75 | 7.8e-09 |
| zag-1 | ZAG-1\_Larvae-L4-stage | 78 | 2.07 | 3.9e-08 |
| lin-35 | LIN-35\_Starved-L1-stage-larvae | 63 | 2.27 | 8.3e-08 |
| ztf-11 | ZTF-11\_Embryos | 33 | 3.43 | 9.3e-08 |
| pha-4 | PHA-4\_Young-adult | 69 | 2.15 | 1.1e-07 |
| pha-4 | PHA-4\_Larvae-L4-stage | 89 | 1.90 | 1.5e-07 |
| unc-62 | UNC-62\_Fed-L1-stage-larvae | 42 | 2.80 | 1.9e-07 |
| nhr-76 | NHR-76\_Larvae-L4-stage | 74 | 2.04 | 2.3e-07 |
| sea-2 | SEA-2\_Larvae-L3-stage | 45 | 2.66 | 2.5e-07 |
| jun-1 | JUN-1\_Larvae-L3-stage | 84 | 1.92 | 2.7e-07 |
| sax-3 | SAX-3\_Larvae-L2-stage | 93 | 1.80 | 7.6e-07 |
| fos-1 | FOS-1\_Larvae-L2-stage | 148 | 1.51 | 9.7e-07 |
| nhr-237 | NHR-237\_Larvae-L2-stage | 23 | 4.14 | 9.7e-07 |
| jun-1 | JUN-1\_Larvae-L4-stage | 82 | 1.81 | 5.1e-06 |
| unc-39 | UNC-39\_Embryos | 38 | 2.58 | 8.1e-06 |
| ceh-38 | CEH-38\_Larvae-L4-stage | 68 | 1.92 | 8.6e-06 |
| lsy-2 | LSY-2\_Larvae-L4-stage | 56 | 2.05 | 1.6e-05 |
| F23F12.9 | F23F12.9\_Embryos | 33 | 2.67 | 2.5e-05 |
| nhr-76 | NHR-76\_Larvae-L3-stage | 59 | 1.95 | 3.8e-05 |
| hlh-30 | HLH-30\_Larvae-L4-stage | 70 | 1.82 | 4.2e-05 |
| elt-1 | ELT-1\_Larvae-L3-stage | 60 | 1.93 | 4.5e-05 |
| nfya-1 | NFYA-1\_Young-adult | 41 | 2.25 | 7.9e-05 |
| aha-1 | AHA-1\_Fed-L1-stage-larvae | 23 | 2.95 | 2.6e-04 |
| sax-3 | SAX-3\_Fed-L1-stage-larvae | 44 | 2.04 | 3.2e-04 |
| ces-1 | CES-1\_Larvae-L3-stage | 50 | 1.94 | 3.3e-04 |
| fos-1 | FOS-1\_Larvae-L3-stage | 84 | 1.60 | 3.6e-04 |
| mef-2 | MEF-2\_Fed-L1-stage-larvae | 31 | 2.41 | 3.7e-04 |
| ztf-7 | ZTF-7\_Larvae-L4-stage | 72 | 1.68 | 3.9e-04 |
| unc-62 | UNC-62\_Day-Four-Young-Adult | 74 | 1.64 | 6.6e-04 |
| unc-62 | UNC-62\_Young-adult-Day-4 | 74 | 1.64 | 6.6e-04 |
| unc-62 | UNC-62\_Larvae-L3-stage | 65 | 1.69 | 9.5e-04 |
| ces-1 | CES-1\_Fed-L1-stage-larvae | 49 | 1.85 | 1.2e-03 |
| dve-1 | DVE-1\_Larvae-L4-stage | 77 | 1.56 | 2.0e-03 |
| ces-1 | CES-1\_Larvae-L4-stage | 47 | 1.81 | 2.7e-03 |
| nhr-21 | NHR-21\_Larvae-L2-stage | 33 | 2.05 | 3.9e-03 |
| med-1 | MED-1\_Embryos | 26 | 2.27 | 4.3e-03 |
| aha-1 | AHA-1\_Larvae-L4-stage | 36 | 1.95 | 4.9e-03 |
| nhr-28 | NHR-28\_Larvae-L4-stage | 132 | 1.33 | 4.9e-03 |
| nhr-11 | NHR-11\_Larvae-L2-stage | 45 | 1.77 | 5.8e-03 |
| aly-2 | ALY-2\_Larvae-L2-stage | 38 | 1.85 | 8.3e-03 |
| daf-12 | DAF-12\_Larvae-L3-stage | 24 | 2.15 | 1.5e-02 |
| fos-1 | FOS-1\_Larvae-L4-stage | 34 | 1.81 | 2.3e-02 |
| unc-62 | UNC-62\_Larvae-L2-stage | 38 | 1.68 | 4.3e-02 |
| nhr-23 | NHR-23\_Larvae-L2-stage | 17 | 2.30 | 4.4e-02 |
