# Supplementary material for: Overlapping cell population expression profiling and regulatory inference in C. elegans
Source: BMC Genomics. 2016 Feb 29;17:159. doi: 10.1186/s12864-016-2482-z (PMC4772325; doi:10.1186/s12864-016-2482-z)
Supplement: Additional file 13: — Web supplement. (DOC 21 kb) [file 12864_2016_2482_MOESM13_ESM.zip › sortWeb/clusters/hier.300.clusters/111.html]

Cluster 111 

## Cluster 111

### Expression

| cnd-1 rep. 1 | cnd-1 rep. 2 | cnd-1 rep. 3 | pha-4 rep. 1 | pha-4 rep. 2 | pha-4 rep. 3 | ceh-27 | ceh-36 | ceh-6 | F21D5.9 | mir-57 | mls-2 | pal-1 | pros-1 | ttx-3 | unc-130 | hlh-16 | irx-1 | ceh-6 (+) hlh-16 (+) | ceh-6 (+) hlh-16 (-) | ceh-6 (-) hlh-16 (+) | cnd-1 singlets | pha-4 singlets | 0 | 60 | 120 | 150 | 180 | 240 | 330 | 390 | 420 | 480 | 540 | 570 | 600 | 630 | 660 | NAME | Functional description |
| --- | --- | --- | --- | --- | --- | --- | --- | --- | --- | --- | --- | --- | --- | --- | --- | --- | --- | --- | --- | --- | --- | --- | --- | --- | --- | --- | --- | --- | --- | --- | --- | --- | --- | --- | --- | --- | --- | --- | --- |
|  |  |  |  |  |  |  |  |  |  |  |  |  |  |  |  |  |  |  |  |  |  |  |  |  |  |  |  |  |  |  |  |  |  |  |  |  |  | W05F2.2 |  |
|  |  |  |  |  |  |  |  |  |  |  |  |  |  |  |  |  |  |  |  |  |  |  |  |  |  |  |  |  |  |  |  |  |  |  |  |  |  | Y50E8A.5 |  |
|  |  |  |  |  |  |  |  |  |  |  |  |  |  |  |  |  |  |  |  |  |  |  |  |  |  |  |  |  |  |  |  |  |  |  |  |  |  | *ndg-4* | NorDiHydroGuaiaretic acid resistant |
|  |  |  |  |  |  |  |  |  |  |  |  |  |  |  |  |  |  |  |  |  |  |  |  |  |  |  |  |  |  |  |  |  |  |  |  |  |  | *uda-1* | Uridine DiphosphAtase |
|  |  |  |  |  |  |  |  |  |  |  |  |  |  |  |  |  |  |  |  |  |  |  |  |  |  |  |  |  |  |  |  |  |  |  |  |  |  | K10D6.10 |  |
|  |  |  |  |  |  |  |  |  |  |  |  |  |  |  |  |  |  |  |  |  |  |  |  |  |  |  |  |  |  |  |  |  |  |  |  |  |  | *unc-6* | UNCoordinated |
|  |  |  |  |  |  |  |  |  |  |  |  |  |  |  |  |  |  |  |  |  |  |  |  |  |  |  |  |  |  |  |  |  |  |  |  |  |  | *srw-89* | Serpentine Receptor, class W |
|  |  |  |  |  |  |  |  |  |  |  |  |  |  |  |  |  |  |  |  |  |  |  |  |  |  |  |  |  |  |  |  |  |  |  |  |  |  | C54G4.2 |  |
|  |  |  |  |  |  |  |  |  |  |  |  |  |  |  |  |  |  |  |  |  |  |  |  |  |  |  |  |  |  |  |  |  |  |  |  |  |  | T16G1.13 |  |
|  |  |  |  |  |  |  |  |  |  |  |  |  |  |  |  |  |  |  |  |  |  |  |  |  |  |  |  |  |  |  |  |  |  |  |  |  |  | F47B7.5 |  |
|  |  |  |  |  |  |  |  |  |  |  |  |  |  |  |  |  |  |  |  |  |  |  |  |  |  |  |  |  |  |  |  |  |  |  |  |  |  | C06A1.8 |  |
|  |  |  |  |  |  |  |  |  |  |  |  |  |  |  |  |  |  |  |  |  |  |  |  |  |  |  |  |  |  |  |  |  |  |  |  |  |  | ZK867.8 |  |
|  |  |  |  |  |  |  |  |  |  |  |  |  |  |  |  |  |  |  |  |  |  |  |  |  |  |  |  |  |  |  |  |  |  |  |  |  |  | K02A11.4 |  |
|  |  |  |  |  |  |  |  |  |  |  |  |  |  |  |  |  |  |  |  |  |  |  |  |  |  |  |  |  |  |  |  |  |  |  |  |  |  | W03A3.4 |  |
|  |  |  |  |  |  |  |  |  |  |  |  |  |  |  |  |  |  |  |  |  |  |  |  |  |  |  |  |  |  |  |  |  |  |  |  |  |  | R09F10.13 |  |
|  |  |  |  |  |  |  |  |  |  |  |  |  |  |  |  |  |  |  |  |  |  |  |  |  |  |  |  |  |  |  |  |  |  |  |  |  |  | *col-52* | COLlagen |
|  |  |  |  |  |  |  |  |  |  |  |  |  |  |  |  |  |  |  |  |  |  |  |  |  |  |  |  |  |  |  |  |  |  |  |  |  |  | W03G9.11 |  |
|  |  |  |  |  |  |  |  |  |  |  |  |  |  |  |  |  |  |  |  |  |  |  |  |  |  |  |  |  |  |  |  |  |  |  |  |  |  | *nspb-3* | Nematode Specific Peptide family, group B |
|  |  |  |  |  |  |  |  |  |  |  |  |  |  |  |  |  |  |  |  |  |  |  |  |  |  |  |  |  |  |  |  |  |  |  |  |  |  | F36G3.6 |  |
|  |  |  |  |  |  |  |  |  |  |  |  |  |  |  |  |  |  |  |  |  |  |  |  |  |  |  |  |  |  |  |  |  |  |  |  |  |  | ZK381.8 |  |
|  |  |  |  |  |  |  |  |  |  |  |  |  |  |  |  |  |  |  |  |  |  |  |  |  |  |  |  |  |  |  |  |  |  |  |  |  |  | F10F2.10 |  |
|  |  |  |  |  |  |  |  |  |  |  |  |  |  |  |  |  |  |  |  |  |  |  |  |  |  |  |  |  |  |  |  |  |  |  |  |  |  | Y57G11C.1140 |  |
|  |  |  |  |  |  |  |  |  |  |  |  |  |  |  |  |  |  |  |  |  |  |  |  |  |  |  |  |  |  |  |  |  |  |  |  |  |  | B0399.t4 |  |
|  |  |  |  |  |  |  |  |  |  |  |  |  |  |  |  |  |  |  |  |  |  |  |  |  |  |  |  |  |  |  |  |  |  |  |  |  |  | F41C6.14 |  |
|  |  |  |  |  |  |  |  |  |  |  |  |  |  |  |  |  |  |  |  |  |  |  |  |  |  |  |  |  |  |  |  |  |  |  |  |  |  | *ssp-34* | Sperm Specific family, class P |
|  |  |  |  |  |  |  |  |  |  |  |  |  |  |  |  |  |  |  |  |  |  |  |  |  |  |  |  |  |  |  |  |  |  |  |  |  |  | *srw-22* | Serpentine Receptor, class W |
|  |  |  |  |  |  |  |  |  |  |  |  |  |  |  |  |  |  |  |  |  |  |  |  |  |  |  |  |  |  |  |  |  |  |  |  |  |  | *str-177* | Seven TM Receptor |
|  |  |  |  |  |  |  |  |  |  |  |  |  |  |  |  |  |  |  |  |  |  |  |  |  |  |  |  |  |  |  |  |  |  |  |  |  |  | F53B1.14 |  |
|  |  |  |  |  |  |  |  |  |  |  |  |  |  |  |  |  |  |  |  |  |  |  |  |  |  |  |  |  |  |  |  |  |  |  |  |  |  | M01G12.7 |  |
|  |  |  |  |  |  |  |  |  |  |  |  |  |  |  |  |  |  |  |  |  |  |  |  |  |  |  |  |  |  |  |  |  |  |  |  |  |  | F13D11.22 |  |
|  |  |  |  |  |  |  |  |  |  |  |  |  |  |  |  |  |  |  |  |  |  |  |  |  |  |  |  |  |  |  |  |  |  |  |  |  |  | K08D8.12 |  |
|  |  |  |  |  |  |  |  |  |  |  |  |  |  |  |  |  |  |  |  |  |  |  |  |  |  |  |  |  |  |  |  |  |  |  |  |  |  | R04F11.18 |  |
|  |  |  |  |  |  |  |  |  |  |  |  |  |  |  |  |  |  |  |  |  |  |  |  |  |  |  |  |  |  |  |  |  |  |  |  |  |  | W06D11.5 |  |
|  |  |  |  |  |  |  |  |  |  |  |  |  |  |  |  |  |  |  |  |  |  |  |  |  |  |  |  |  |  |  |  |  |  |  |  |  |  | F40H3.13 |  |
|  |  |  |  |  |  |  |  |  |  |  |  |  |  |  |  |  |  |  |  |  |  |  |  |  |  |  |  |  |  |  |  |  |  |  |  |  |  | C30E1.t1 |  |
|  |  |  |  |  |  |  |  |  |  |  |  |  |  |  |  |  |  |  |  |  |  |  |  |  |  |  |  |  |  |  |  |  |  |  |  |  |  | F41D9.t4 |  |
|  |  |  |  |  |  |  |  |  |  |  |  |  |  |  |  |  |  |  |  |  |  |  |  |  |  |  |  |  |  |  |  |  |  |  |  |  |  | F45E1.10 |  |
|  |  |  |  |  |  |  |  |  |  |  |  |  |  |  |  |  |  |  |  |  |  |  |  |  |  |  |  |  |  |  |  |  |  |  |  |  |  | C39D10.t2 |  |
|  |  |  |  |  |  |  |  |  |  |  |  |  |  |  |  |  |  |  |  |  |  |  |  |  |  |  |  |  |  |  |  |  |  |  |  |  |  | C54D2.13 |  |
|  |  |  |  |  |  |  |  |  |  |  |  |  |  |  |  |  |  |  |  |  |  |  |  |  |  |  |  |  |  |  |  |  |  |  |  |  |  | F25H10.5 |  |
|  |  |  |  |  |  |  |  |  |  |  |  |  |  |  |  |  |  |  |  |  |  |  |  |  |  |  |  |  |  |  |  |  |  |  |  |  |  | F21E9.6 |  |
|  |  |  |  |  |  |  |  |  |  |  |  |  |  |  |  |  |  |  |  |  |  |  |  |  |  |  |  |  |  |  |  |  |  |  |  |  |  | *srh-110* | Serpentine Receptor, class H |
|  |  |  |  |  |  |  |  |  |  |  |  |  |  |  |  |  |  |  |  |  |  |  |  |  |  |  |  |  |  |  |  |  |  |  |  |  |  | H12D21.17 |  |
|  |  |  |  |  |  |  |  |  |  |  |  |  |  |  |  |  |  |  |  |  |  |  |  |  |  |  |  |  |  |  |  |  |  |  |  |  |  | F07C3.11 |  |
|  |  |  |  |  |  |  |  |  |  |  |  |  |  |  |  |  |  |  |  |  |  |  |  |  |  |  |  |  |  |  |  |  |  |  |  |  |  | T09D3.11 |  |
|  |  |  |  |  |  |  |  |  |  |  |  |  |  |  |  |  |  |  |  |  |  |  |  |  |  |  |  |  |  |  |  |  |  |  |  |  |  | Y39H10A.9 |  |
|  |  |  |  |  |  |  |  |  |  |  |  |  |  |  |  |  |  |  |  |  |  |  |  |  |  |  |  |  |  |  |  |  |  |  |  |  |  | F16B4.3 |  |
|  |  |  |  |  |  |  |  |  |  |  |  |  |  |  |  |  |  |  |  |  |  |  |  |  |  |  |  |  |  |  |  |  |  |  |  |  |  | F22B3.19 |  |
|  |  |  |  |  |  |  |  |  |  |  |  |  |  |  |  |  |  |  |  |  |  |  |  |  |  |  |  |  |  |  |  |  |  |  |  |  |  | R13A1.16 |  |
|  |  |  |  |  |  |  |  |  |  |  |  |  |  |  |  |  |  |  |  |  |  |  |  |  |  |  |  |  |  |  |  |  |  |  |  |  |  | R12B2.9 |  |
|  |  |  |  |  |  |  |  |  |  |  |  |  |  |  |  |  |  |  |  |  |  |  |  |  |  |  |  |  |  |  |  |  |  |  |  |  |  | Y53G8B.t7 |  |
|  |  |  |  |  |  |  |  |  |  |  |  |  |  |  |  |  |  |  |  |  |  |  |  |  |  |  |  |  |  |  |  |  |  |  |  |  |  | K01A2.14 |  |
|  |  |  |  |  |  |  |  |  |  |  |  |  |  |  |  |  |  |  |  |  |  |  |  |  |  |  |  |  |  |  |  |  |  |  |  |  |  | *nep-13* | NEPrilysin metallopeptidase family |
|  |  |  |  |  |  |  |  |  |  |  |  |  |  |  |  |  |  |  |  |  |  |  |  |  |  |  |  |  |  |  |  |  |  |  |  |  |  | *srh-130* | Serpentine Receptor, class H |
|  |  |  |  |  |  |  |  |  |  |  |  |  |  |  |  |  |  |  |  |  |  |  |  |  |  |  |  |  |  |  |  |  |  |  |  |  |  | *gcy-1* | Guanylyl CYclase |
|  |  |  |  |  |  |  |  |  |  |  |  |  |  |  |  |  |  |  |  |  |  |  |  |  |  |  |  |  |  |  |  |  |  |  |  |  |  | C14F11.23 |  |
|  |  |  |  |  |  |  |  |  |  |  |  |  |  |  |  |  |  |  |  |  |  |  |  |  |  |  |  |  |  |  |  |  |  |  |  |  |  | *math-13* | MATH (meprin-associated Traf homology) domain containing |
|  |  |  |  |  |  |  |  |  |  |  |  |  |  |  |  |  |  |  |  |  |  |  |  |  |  |  |  |  |  |  |  |  |  |  |  |  |  | F49H12.12 |  |
|  |  |  |  |  |  |  |  |  |  |  |  |  |  |  |  |  |  |  |  |  |  |  |  |  |  |  |  |  |  |  |  |  |  |  |  |  |  | *srh-53* | Serpentine Receptor, class H |
|  |  |  |  |  |  |  |  |  |  |  |  |  |  |  |  |  |  |  |  |  |  |  |  |  |  |  |  |  |  |  |  |  |  |  |  |  |  | T19C4.19 |  |
|  |  |  |  |  |  |  |  |  |  |  |  |  |  |  |  |  |  |  |  |  |  |  |  |  |  |  |  |  |  |  |  |  |  |  |  |  |  | W02D7.8 |  |
|  |  |  |  |  |  |  |  |  |  |  |  |  |  |  |  |  |  |  |  |  |  |  |  |  |  |  |  |  |  |  |  |  |  |  |  |  |  | C30C11.5 |  |
|  |  |  |  |  |  |  |  |  |  |  |  |  |  |  |  |  |  |  |  |  |  |  |  |  |  |  |  |  |  |  |  |  |  |  |  |  |  | ZK994.t2 |  |
|  |  |  |  |  |  |  |  |  |  |  |  |  |  |  |  |  |  |  |  |  |  |  |  |  |  |  |  |  |  |  |  |  |  |  |  |  |  | *col-13* | COLlagen |
|  |  |  |  |  |  |  |  |  |  |  |  |  |  |  |  |  |  |  |  |  |  |  |  |  |  |  |  |  |  |  |  |  |  |  |  |  |  | *srt-24* | Serpentine Receptor, class T |
|  |  |  |  |  |  |  |  |  |  |  |  |  |  |  |  |  |  |  |  |  |  |  |  |  |  |  |  |  |  |  |  |  |  |  |  |  |  | C09B7.9 |  |
|  |  |  |  |  |  |  |  |  |  |  |  |  |  |  |  |  |  |  |  |  |  |  |  |  |  |  |  |  |  |  |  |  |  |  |  |  |  | C53B4.9 |  |
|  |  |  |  |  |  |  |  |  |  |  |  |  |  |  |  |  |  |  |  |  |  |  |  |  |  |  |  |  |  |  |  |  |  |  |  |  |  | K08B12.12 |  |

### Phenotypes enriched

none found

### Anatomy terms enriched

none found

### GO terms enriched

none found

### Expression clusters enriched

none found

### Motifs enriched

|  |  |  |  |  |  |
| --- | --- | --- | --- | --- | --- |
| **Motif** | **Logo** | **Possible orthologs** | **Number of motifs in cluster** | **Enrichment** | **FDR corrected p** |
| ems\_FlyReg\_FBgn0000576 |  | ceh-2 skn-1 | 19 | 3.42 | 0.00032 |
| pTH5714 |  | nhr-239 | 16 | 3.47 | 0.00160 |
| V$NKX22\_01 |  | ceh-24 ceh-22 dsc-1 | 31 | 2.04 | 0.00290 |
| Nkx2-3\_3435 |  | ceh-24 dsc-1 | 27 | 2.19 | 0.00350 |
| Tup\_Cell\_FBgn0003896 |  | ceh-31 ceh-24 lim-7 ceh-19 | 7 | 8.09 | 0.00360 |
| Al\_SOLEXA\_FBgn0000061 |  | lin-39 alr-1 ceh-14 ceh-2 | 25 | 2.29 | 0.00380 |
| RORA\_f1 |  | nhr-213 | 11 | 4.32 | 0.00530 |
| HXB1\_f1 |  | ceh-20 ceh-12 | 37 | 1.76 | 0.00590 |
| pTH5781 |  | ceh-32 | 25 | 2.19 | 0.00690 |
| FOXO6\_2 |  | daf-16 | 36 | 1.77 | 0.00730 |
| MA0485.1 |  | lin-39 | 30 | 1.95 | 0.00770 |
| V$SRF\_01 |  | unc-120 | 22 | 2.36 | 0.00780 |
| pTH9901 |  | ceh-13 D1005.3 | 30 | 1.95 | 0.00790 |
| En1\_3123 |  | ceh-16 | 27 | 2.07 | 0.00790 |
| Meis3\_1 |  | ceh-32 ces-1 | 34 | 1.81 | 0.00830 |
| pTH5539 |  | unc-120 | 10 | 4.45 | 0.00850 |
| I$SN\_02 |  | K02D7.2 | 31 | 1.88 | 0.00990 |
| MA0488.1 |  | crh-1 | 30 | 1.90 | 0.01200 |
| pTH9073 |  | elt-1 end-3 | 11 | 3.86 | 0.01200 |
| En2\_0952 |  | ceh-16 | 26 | 2.06 | 0.01200 |
| V$OCT1\_03 |  | ceh-18 | 19 | 2.48 | 0.01300 |
| EN1\_4 |  | ceh-16 | 19 | 2.47 | 0.01400 |
| BARHL2\_1 |  | ceh-31 | 25 | 2.04 | 0.01700 |
| K562b\_GATA2\_UCD |  | elt-1 | 34 | 1.73 | 0.01800 |
| Hoxa11\_2218 |  | php-3 | 26 | 1.99 | 0.01800 |
| Mv63 |  | nhr-19 C24A1.2 | 20 | 2.31 | 0.01900 |
| MEIS1\_f2 |  | ceh-32 | 11 | 3.57 | 0.01900 |
| Oli\_da\_SANGER\_5\_3\_FBgn0032651 |  | hlh-32 hlh-12 | 29 | 1.87 | 0.01900 |
| Mv90 |  | mef-2 | 32 | 1.77 | 0.02000 |
| ZN384\_f1 |  | lin-29 | 41 | 1.56 | 0.02000 |
| V$RORA1\_01 |  | nhr-213 nhr-71 | 12 | 3.28 | 0.02100 |
| V$CDPCR1\_01 |  | ceh-48 | 9 | 4.23 | 0.02200 |
| pTH9298 |  | attf-1 | 11 | 3.46 | 0.02400 |
| Hmx2\_3424 |  | ceh-9 | 26 | 1.95 | 0.02400 |
| pTH6445 |  | ceh-5 | 29 | 1.83 | 0.02500 |
| Alx4\_1 |  | alr-1 | 25 | 1.98 | 0.02500 |
| pTH6071 |  | C33G8.2 | 20 | 2.24 | 0.02600 |
| Cutl1\_3494 |  | ceh-44 | 10 | 3.71 | 0.02600 |
| Mw142 |  | egl-27 | 30 | 1.79 | 0.02700 |
| GABPA\_1 |  | lin-1 | 28 | 1.84 | 0.02900 |
| pTH9142 |  | C34D1.1 | 34 | 1.67 | 0.02900 |
| NKX28\_f1 |  | ceh-24 | 28 | 1.84 | 0.03000 |
| HXC6\_f1 |  | lin-39 | 23 | 2.03 | 0.03100 |
| pTH5561 |  | nhr-239 | 9 | 3.95 | 0.03100 |
| Msx2\_3449 |  | ceh-1 | 22 | 2.08 | 0.03100 |
| MSX2\_1 |  | ceh-14 ceh-1 | 27 | 1.86 | 0.03200 |
| GATA3\_si |  | elt-1 | 8 | 4.41 | 0.03200 |
| MYOG\_f1 |  | hlh-1 | 23 | 2.02 | 0.03400 |
| Hr46\_FlyReg\_FBgn0000448 |  | nhr-213 | 28 | 1.82 | 0.03500 |
| Osr2\_1727 |  | odd-1 | 10 | 3.53 | 0.03500 |
| pTH6019 |  | nhr-213 nhr-19 | 19 | 2.23 | 0.03600 |
| Antp\_Cell\_FBgn0000095 |  | lin-39 | 32 | 1.70 | 0.03700 |
| pTH5887 |  | lin-39 | 22 | 2.05 | 0.03700 |
| MA0261.1 |  | nhr-255 lin-14 | 23 | 2.00 | 0.03700 |
| tgo\_trh\_SANGER\_5\_FBgn0015014 |  | aha-1 | 31 | 1.72 | 0.03800 |
| pTH9969 |  | pag-3 | 30 | 1.74 | 0.04000 |
| pTH9059 |  | ztf-28 | 25 | 1.90 | 0.04000 |
| pTH9709 |  | die-1 | 28 | 1.79 | 0.04100 |
| TBX20\_4 |  | mab-9 (0.54) tbx-43 | 28 | 1.79 | 0.04100 |
| pTH6503 |  | ceh-31 | 22 | 2.02 | 0.04200 |
| Eip93F\_SANGER\_10\_FBgn0013948 |  | mbr-1 | 19 | 2.19 | 0.04200 |
| pTH8341 |  | unc-86 | 21 | 2.07 | 0.04300 |
| GM12878\_PAX5N19\_HudsonAlpha |  | pax-2 | 10 | 3.37 | 0.04500 |
| Mf28 |  | elt-1 | 35 | 1.60 | 0.04500 |
| pTH9247 |  | C34D1.1 | 19 | 2.17 | 0.04500 |
| Meox1\_2310 |  | ceh-31 | 37 | 1.56 | 0.04500 |
| pTH5118 |  | cfi-1 | 31 | 1.69 | 0.04700 |
| pTH1049 |  | elt-1 | 8 | 4.05 | 0.04800 |
| Hoxa10\_2318 |  | ceh-24 | 12 | 2.88 | 0.04900 |

### Correlated (and anti-correlated) transcription factors

|  |  |
| --- | --- |
| **Transcription factor** | **Correlation** |
| attf-4 | 0.71 |
| F26H9.2 | 0.66 |
| lim-6 | 0.65 |
| unc-130 | 0.64 |
| aptf-1 | 0.63 |
| nhr-25 | 0.61 |
| D1081.8 | 0.60 |
| lag-1 | 0.59 |
| egl-46 | 0.59 |
| ref-2 | 0.59 |
| ztf-11 | 0.59 |
| ZK337.2 | 0.58 |
| hlh-2 | 0.58 |
| mab-9 | 0.54 |
| egl-5 | 0.54 |
| hlh-17 | 0.53 |
| ceh-27 | 0.53 |
| ceh-6 | 0.53 |
| bar-1 | 0.52 |
| hlh-14 | 0.52 |
| irx-1 | 0.51 |
| pal-1 | 0.51 |
| nhr-242 | 0.51 |
| hlh-31 | 0.51 |
| ztf-29 | 0.51 |
| T26A5.8 | -0.44 |
| ceh-9 | -0.44 |
| nhr-84 | -0.46 |
| ceh-36 | -0.47 |
| nhr-286 | -0.47 |
| dmd-10 | -0.48 |
| nhr-124 | -0.48 |
| nhr-167 | -0.49 |
| nhr-101 | -0.49 |
| sox-4 | -0.50 |
| mbf-1 | -0.50 |
| C06E2.1 | -0.50 |
| gei-3 | -0.51 |
| nhr-105 | -0.51 |
| K12H6.12 | -0.51 |
| nhr-26 | -0.51 |
| ceh-23 | -0.52 |
| nhr-196 | -0.54 |
| nhr-36 | -0.58 |
| nhr-135 | -0.59 |
| nhr-197 | -0.60 |
| nhr-30 | -0.63 |
| nhr-268 | -0.63 |
| dmd-9 | -0.67 |
| fkh-2 | -0.74 |

### ChIP peaks enriched

none found
