# Supplementary material for: Overlapping cell population expression profiling and regulatory inference in C. elegans
Source: BMC Genomics. 2016 Feb 29;17:159. doi: 10.1186/s12864-016-2482-z (PMC4772325; doi:10.1186/s12864-016-2482-z)
Supplement: Additional file 13: — Web supplement. (DOC 21 kb) [file 12864_2016_2482_MOESM13_ESM.zip › sortWeb/clusters/hier.300.clusters/115.html]

Cluster 115 

## Cluster 115

### Expression

| cnd-1 rep. 1 | cnd-1 rep. 2 | cnd-1 rep. 3 | pha-4 rep. 1 | pha-4 rep. 2 | pha-4 rep. 3 | ceh-27 | ceh-36 | ceh-6 | F21D5.9 | mir-57 | mls-2 | pal-1 | pros-1 | ttx-3 | unc-130 | hlh-16 | irx-1 | ceh-6 (+) hlh-16 (+) | ceh-6 (+) hlh-16 (-) | ceh-6 (-) hlh-16 (+) | cnd-1 singlets | pha-4 singlets | 0 | 60 | 120 | 150 | 180 | 240 | 330 | 390 | 420 | 480 | 540 | 570 | 600 | 630 | 660 | NAME | Functional description |
| --- | --- | --- | --- | --- | --- | --- | --- | --- | --- | --- | --- | --- | --- | --- | --- | --- | --- | --- | --- | --- | --- | --- | --- | --- | --- | --- | --- | --- | --- | --- | --- | --- | --- | --- | --- | --- | --- | --- | --- |
|  |  |  |  |  |  |  |  |  |  |  |  |  |  |  |  |  |  |  |  |  |  |  |  |  |  |  |  |  |  |  |  |  |  |  |  |  |  | ZK973.8 |  |
|  |  |  |  |  |  |  |  |  |  |  |  |  |  |  |  |  |  |  |  |  |  |  |  |  |  |  |  |  |  |  |  |  |  |  |  |  |  | Y71A12B.28 |  |
|  |  |  |  |  |  |  |  |  |  |  |  |  |  |  |  |  |  |  |  |  |  |  |  |  |  |  |  |  |  |  |  |  |  |  |  |  |  | *mab-23* | Male ABnormal |
|  |  |  |  |  |  |  |  |  |  |  |  |  |  |  |  |  |  |  |  |  |  |  |  |  |  |  |  |  |  |  |  |  |  |  |  |  |  | Y53F4B.16 |  |
|  |  |  |  |  |  |  |  |  |  |  |  |  |  |  |  |  |  |  |  |  |  |  |  |  |  |  |  |  |  |  |  |  |  |  |  |  |  | F49F1.13 |  |
|  |  |  |  |  |  |  |  |  |  |  |  |  |  |  |  |  |  |  |  |  |  |  |  |  |  |  |  |  |  |  |  |  |  |  |  |  |  | *sri-44* | Serpentine Receptor, class I |
|  |  |  |  |  |  |  |  |  |  |  |  |  |  |  |  |  |  |  |  |  |  |  |  |  |  |  |  |  |  |  |  |  |  |  |  |  |  | F02A9.9 |  |
|  |  |  |  |  |  |  |  |  |  |  |  |  |  |  |  |  |  |  |  |  |  |  |  |  |  |  |  |  |  |  |  |  |  |  |  |  |  | M03D4.5 |  |
|  |  |  |  |  |  |  |  |  |  |  |  |  |  |  |  |  |  |  |  |  |  |  |  |  |  |  |  |  |  |  |  |  |  |  |  |  |  | B0285.16 |  |
|  |  |  |  |  |  |  |  |  |  |  |  |  |  |  |  |  |  |  |  |  |  |  |  |  |  |  |  |  |  |  |  |  |  |  |  |  |  | ZK678.3 |  |
|  |  |  |  |  |  |  |  |  |  |  |  |  |  |  |  |  |  |  |  |  |  |  |  |  |  |  |  |  |  |  |  |  |  |  |  |  |  | *srz-75* | Serpentine Receptor, class Z |
|  |  |  |  |  |  |  |  |  |  |  |  |  |  |  |  |  |  |  |  |  |  |  |  |  |  |  |  |  |  |  |  |  |  |  |  |  |  | R02D5.22 |  |
|  |  |  |  |  |  |  |  |  |  |  |  |  |  |  |  |  |  |  |  |  |  |  |  |  |  |  |  |  |  |  |  |  |  |  |  |  |  | T25D10.9 |  |
|  |  |  |  |  |  |  |  |  |  |  |  |  |  |  |  |  |  |  |  |  |  |  |  |  |  |  |  |  |  |  |  |  |  |  |  |  |  | T15H9.4 |  |
|  |  |  |  |  |  |  |  |  |  |  |  |  |  |  |  |  |  |  |  |  |  |  |  |  |  |  |  |  |  |  |  |  |  |  |  |  |  | *clec-85* | C-type LECtin |
|  |  |  |  |  |  |  |  |  |  |  |  |  |  |  |  |  |  |  |  |  |  |  |  |  |  |  |  |  |  |  |  |  |  |  |  |  |  | JC8.15 |  |
|  |  |  |  |  |  |  |  |  |  |  |  |  |  |  |  |  |  |  |  |  |  |  |  |  |  |  |  |  |  |  |  |  |  |  |  |  |  | T04B8.1 |  |
|  |  |  |  |  |  |  |  |  |  |  |  |  |  |  |  |  |  |  |  |  |  |  |  |  |  |  |  |  |  |  |  |  |  |  |  |  |  | H37A05.4 |  |
|  |  |  |  |  |  |  |  |  |  |  |  |  |  |  |  |  |  |  |  |  |  |  |  |  |  |  |  |  |  |  |  |  |  |  |  |  |  | F55B12.11 |  |
|  |  |  |  |  |  |  |  |  |  |  |  |  |  |  |  |  |  |  |  |  |  |  |  |  |  |  |  |  |  |  |  |  |  |  |  |  |  | *lbp-1* | Lipid Binding Protein |
|  |  |  |  |  |  |  |  |  |  |  |  |  |  |  |  |  |  |  |  |  |  |  |  |  |  |  |  |  |  |  |  |  |  |  |  |  |  | *lin-39* | abnormal cell LINeage |
|  |  |  |  |  |  |  |  |  |  |  |  |  |  |  |  |  |  |  |  |  |  |  |  |  |  |  |  |  |  |  |  |  |  |  |  |  |  | *lbp-3* | Lipid Binding Protein |
|  |  |  |  |  |  |  |  |  |  |  |  |  |  |  |  |  |  |  |  |  |  |  |  |  |  |  |  |  |  |  |  |  |  |  |  |  |  | K08A2.1 |  |
|  |  |  |  |  |  |  |  |  |  |  |  |  |  |  |  |  |  |  |  |  |  |  |  |  |  |  |  |  |  |  |  |  |  |  |  |  |  | *unc-62* | UNCoordinated |
|  |  |  |  |  |  |  |  |  |  |  |  |  |  |  |  |  |  |  |  |  |  |  |  |  |  |  |  |  |  |  |  |  |  |  |  |  |  | *mig-1* | abnormal cell MIGration |
|  |  |  |  |  |  |  |  |  |  |  |  |  |  |  |  |  |  |  |  |  |  |  |  |  |  |  |  |  |  |  |  |  |  |  |  |  |  | *elt-1* | Erythroid-Like Transcription factor family |
|  |  |  |  |  |  |  |  |  |  |  |  |  |  |  |  |  |  |  |  |  |  |  |  |  |  |  |  |  |  |  |  |  |  |  |  |  |  | ZK930.2 |  |
|  |  |  |  |  |  |  |  |  |  |  |  |  |  |  |  |  |  |  |  |  |  |  |  |  |  |  |  |  |  |  |  |  |  |  |  |  |  | F43G6.4 |  |
|  |  |  |  |  |  |  |  |  |  |  |  |  |  |  |  |  |  |  |  |  |  |  |  |  |  |  |  |  |  |  |  |  |  |  |  |  |  | *vab-23* | Variable ABnormal morphology |
|  |  |  |  |  |  |  |  |  |  |  |  |  |  |  |  |  |  |  |  |  |  |  |  |  |  |  |  |  |  |  |  |  |  |  |  |  |  | *asp-4* | ASpartyl Protease |
|  |  |  |  |  |  |  |  |  |  |  |  |  |  |  |  |  |  |  |  |  |  |  |  |  |  |  |  |  |  |  |  |  |  |  |  |  |  | *hum-7* | Heavy chain, Unconventional Myosin |
|  |  |  |  |  |  |  |  |  |  |  |  |  |  |  |  |  |  |  |  |  |  |  |  |  |  |  |  |  |  |  |  |  |  |  |  |  |  | *aak-1* | AMP-Activated Kinase |
|  |  |  |  |  |  |  |  |  |  |  |  |  |  |  |  |  |  |  |  |  |  |  |  |  |  |  |  |  |  |  |  |  |  |  |  |  |  | C56G2.5 |  |
|  |  |  |  |  |  |  |  |  |  |  |  |  |  |  |  |  |  |  |  |  |  |  |  |  |  |  |  |  |  |  |  |  |  |  |  |  |  | F11C3.1 |  |
|  |  |  |  |  |  |  |  |  |  |  |  |  |  |  |  |  |  |  |  |  |  |  |  |  |  |  |  |  |  |  |  |  |  |  |  |  |  | *unc-39* | UNCoordinated |
|  |  |  |  |  |  |  |  |  |  |  |  |  |  |  |  |  |  |  |  |  |  |  |  |  |  |  |  |  |  |  |  |  |  |  |  |  |  | Y116F11A.3 |  |
|  |  |  |  |  |  |  |  |  |  |  |  |  |  |  |  |  |  |  |  |  |  |  |  |  |  |  |  |  |  |  |  |  |  |  |  |  |  | C03C10.4 |  |
|  |  |  |  |  |  |  |  |  |  |  |  |  |  |  |  |  |  |  |  |  |  |  |  |  |  |  |  |  |  |  |  |  |  |  |  |  |  | *mboa-3* | Membrane Bound O-Acyl transferase, MBOAT |
|  |  |  |  |  |  |  |  |  |  |  |  |  |  |  |  |  |  |  |  |  |  |  |  |  |  |  |  |  |  |  |  |  |  |  |  |  |  | F53G12.9 |  |
|  |  |  |  |  |  |  |  |  |  |  |  |  |  |  |  |  |  |  |  |  |  |  |  |  |  |  |  |  |  |  |  |  |  |  |  |  |  | F53G12.13 |  |
|  |  |  |  |  |  |  |  |  |  |  |  |  |  |  |  |  |  |  |  |  |  |  |  |  |  |  |  |  |  |  |  |  |  |  |  |  |  | T04D3.1 |  |
|  |  |  |  |  |  |  |  |  |  |  |  |  |  |  |  |  |  |  |  |  |  |  |  |  |  |  |  |  |  |  |  |  |  |  |  |  |  | ZK930.4 |  |
|  |  |  |  |  |  |  |  |  |  |  |  |  |  |  |  |  |  |  |  |  |  |  |  |  |  |  |  |  |  |  |  |  |  |  |  |  |  | *lon-1* | LONg |
|  |  |  |  |  |  |  |  |  |  |  |  |  |  |  |  |  |  |  |  |  |  |  |  |  |  |  |  |  |  |  |  |  |  |  |  |  |  | K10G4.5 |  |
|  |  |  |  |  |  |  |  |  |  |  |  |  |  |  |  |  |  |  |  |  |  |  |  |  |  |  |  |  |  |  |  |  |  |  |  |  |  | *mab-9* | Male ABnormal |
|  |  |  |  |  |  |  |  |  |  |  |  |  |  |  |  |  |  |  |  |  |  |  |  |  |  |  |  |  |  |  |  |  |  |  |  |  |  | *hlh-32* | Helix Loop Helix |
|  |  |  |  |  |  |  |  |  |  |  |  |  |  |  |  |  |  |  |  |  |  |  |  |  |  |  |  |  |  |  |  |  |  |  |  |  |  | Y39B6A.7 |  |
|  |  |  |  |  |  |  |  |  |  |  |  |  |  |  |  |  |  |  |  |  |  |  |  |  |  |  |  |  |  |  |  |  |  |  |  |  |  | *bar-1* | Beta-catenin/Armadillo Related |
|  |  |  |  |  |  |  |  |  |  |  |  |  |  |  |  |  |  |  |  |  |  |  |  |  |  |  |  |  |  |  |  |  |  |  |  |  |  | Y49E10.16 |  |
|  |  |  |  |  |  |  |  |  |  |  |  |  |  |  |  |  |  |  |  |  |  |  |  |  |  |  |  |  |  |  |  |  |  |  |  |  |  | *hlh-17* | Helix Loop Helix |
|  |  |  |  |  |  |  |  |  |  |  |  |  |  |  |  |  |  |  |  |  |  |  |  |  |  |  |  |  |  |  |  |  |  |  |  |  |  | *srab-10* | Serpentine Receptor, class AB (class A-like) |
|  |  |  |  |  |  |  |  |  |  |  |  |  |  |  |  |  |  |  |  |  |  |  |  |  |  |  |  |  |  |  |  |  |  |  |  |  |  | *srsx-19* | Serpentine Receptor, class SX |
|  |  |  |  |  |  |  |  |  |  |  |  |  |  |  |  |  |  |  |  |  |  |  |  |  |  |  |  |  |  |  |  |  |  |  |  |  |  | F14F7.5 |  |
|  |  |  |  |  |  |  |  |  |  |  |  |  |  |  |  |  |  |  |  |  |  |  |  |  |  |  |  |  |  |  |  |  |  |  |  |  |  | Y42A5A.1 |  |
|  |  |  |  |  |  |  |  |  |  |  |  |  |  |  |  |  |  |  |  |  |  |  |  |  |  |  |  |  |  |  |  |  |  |  |  |  |  | F55G1.12 |  |
|  |  |  |  |  |  |  |  |  |  |  |  |  |  |  |  |  |  |  |  |  |  |  |  |  |  |  |  |  |  |  |  |  |  |  |  |  |  | C27C12.9 |  |

### Phenotypes enriched

|  |  |  |  |
| --- | --- | --- | --- |
| **Group name** | **Number in cluster** | **Enrichment** | **FDR corrected p** |
| egg laying variant (RNAi) | 8 | 6.04 | 0.0487 |

### Anatomy terms enriched

none found

### GO terms enriched

|  |  |  |
| --- | --- | --- |
| **GO term** | **Number of genes** | **FDR-corrected p-value** |
| cell fate commitment | 5 | 0.0032 |
| oviposition | 6 | 0.0110 |
| multi-organism reproductive behavior | 6 | 0.0150 |
| multi-multicellular organism process | 6 | 0.0150 |
| regulation of cell migration | 3 | 0.0300 |
| regulation of cellular component movement | 3 | 0.0370 |
| cell motility | 5 | 0.0400 |

### Expression clusters enriched

none found

### Motifs enriched

|  |  |  |  |  |  |
| --- | --- | --- | --- | --- | --- |
| **Motif** | **Logo** | **Possible orthologs** | **Number of motifs in cluster** | **Enrichment** | **FDR corrected p** |
| I$E74A\_01 |  | lin-1 (0.76) C24A1.2 | 32 | 2.08 | 0.00062 |
| CG33980\_SOLEXA\_2\_0\_FBgn0053980 |  | lin-39 (0.9) egl-5 (0.58) ceh-23 (-0.55) ceh-2 (-0.52) lim-7 cog-1 ceh-30 ceh-18 ceh-36 ceh-31 ceh-12 ceh-53 lim-4 ceh-45 ceh-16 ceh-43 dsc-1 lim-6 ceh-1 mls-2 and 7 others  [full list] | 16 | 3.40 | 0.00160 |
| pTH5067 |  | lin-32 hlh-1 hlh-11 | 22 | 2.55 | 0.00210 |
| pTH9182 |  | tbx-39 | 12 | 4.41 | 0.00210 |
| EN2\_2 |  | lin-39 (0.9) ceh-9 ceh-31 ceh-16 ceh-43 ceh-1 alr-1 | 15 | 3.50 | 0.00220 |
| Dr\_Cell\_FBgn0000492 |  | ceh-19 (-0.58) lim-7 ceh-30 ceh-9 ceh-31 ceh-43 ceh-1 alr-1 | 12 | 4.06 | 0.00390 |
| pTH9244 |  | tbx-39 | 12 | 4.06 | 0.00400 |
| HOXC10\_3 |  | lin-39 (0.9) php-3 | 44 | 1.52 | 0.00420 |
| Cart1\_1275 |  | lim-7 ceh-18 alr-1 | 15 | 3.26 | 0.00440 |
| Arx\_1738 |  | lin-39 (0.9) ceh-45 ceh-1 alr-1 ceh-10 | 15 | 3.25 | 0.00440 |
| ftz-f1\_FlyReg\_FBgn0001078 |  | nhr-68 | 53 | 1.30 | 0.00460 |
| PROX1\_1 |  | ceh-26 | 50 | 1.37 | 0.00480 |
| Aef1\_FlyReg\_FBgn0005694 |  | K11D2.4 | 33 | 1.79 | 0.00680 |
| pTH9342 |  | lin-39 (0.9) ceh-18 ceh-12 ceh-45 ceh-43 ceh-1 alr-1 | 13 | 3.49 | 0.00710 |
| HXA10\_f1 |  | lin-39 (0.9) | 31 | 1.85 | 0.00740 |
| PAX5\_si |  | pax-2 F45H11.6 | 28 | 1.97 | 0.00750 |
| V$EN1\_01 |  | ceh-16 | 51 | 1.32 | 0.00920 |
| pTH6445 |  | ceh-5 (0.51) | 21 | 2.33 | 0.00950 |
| V$S8\_01 |  | ceh-45 | 23 | 2.19 | 0.00960 |
| K562\_ZBTB7A\_HudsonAlpha |  | ZC328.2 | 42 | 1.51 | 0.01000 |
| V$NCX\_01 |  | ceh-19 (-0.58) | 7 | 6.48 | 0.01000 |
| Lhx4\_1719 |  | lim-7 ceh-16 lim-6 | 24 | 2.11 | 0.01100 |
| EN1\_4 |  | ceh-16 | 8 | 5.37 | 0.01100 |
| pTH2193 |  | nhr-2 | 48 | 1.37 | 0.01200 |
| NR2F1\_4 |  | nhr-2 | 49 | 1.35 | 0.01200 |
| MA0146.2 |  | F58G1.2 | 41 | 1.52 | 0.01200 |
| CG9895\_SANGER\_10\_FBgn0034810 |  | klf-1 klf-2 | 10 | 4.13 | 0.01300 |
| Gsh2\_3990 |  | ceh-31 | 21 | 2.26 | 0.01400 |
| pTH9306 |  | lsl-1 | 9 | 4.53 | 0.01400 |
| Barx2\_3447 |  | ceh-43 | 36 | 1.63 | 0.01500 |
| Vax2\_3500 |  | C02F12.10 | 22 | 2.17 | 0.01500 |
| PBX1\_do |  | lin-39 (0.9) ceh-20 (0.76) | 19 | 2.38 | 0.01600 |
| Meis1\_2335 |  | ceh-32 | 14 | 2.93 | 0.01800 |
| Dlx3\_1030 |  | ceh-43 | 22 | 2.14 | 0.01800 |
| pTH5337 |  | ZC328.2 | 40 | 1.51 | 0.01800 |
| pTH3811 |  | nhr-213 (-0.71) nhr-2 nhr-19 | 44 | 1.43 | 0.01800 |
| Dlx2\_2273 |  | ceh-43 | 22 | 2.13 | 0.01900 |
| Vsx1\_1728 |  | alr-1 | 22 | 2.13 | 0.01900 |
| NHLH1\_1 |  | hlh-15 | 32 | 1.71 | 0.01900 |
| Pou2f1\_3081 |  | ceh-18 | 16 | 2.62 | 0.02000 |
| pTH9289 |  | mab-9 (0.86) tbx-39 | 9 | 4.25 | 0.02000 |
| Hoxc11\_3718 |  | pal-1 (0.67) ceh-24 | 26 | 1.92 | 0.02000 |
| pTH2820 |  | ZC328.2 | 46 | 1.38 | 0.02000 |
| pTH6268 |  | ceh-2 (-0.52) | 15 | 2.73 | 0.02000 |
| Lhx1\_2240 |  | lim-7 | 15 | 2.70 | 0.02200 |
| tj\_SANGER\_5\_FBgn0000964 |  | F45H11.6 | 49 | 1.32 | 0.02200 |
| pTH5928 |  | ceh-34 | 45 | 1.39 | 0.02400 |
| pTH4269 |  | nhr-177 | 50 | 1.30 | 0.02400 |
| Gsc\_SOLEXA\_FBgn0010323 |  | ceh-45 | 14 | 2.80 | 0.02500 |
| Prop1\_3949 |  | ceh-53 ceh-16 | 13 | 2.96 | 0.02500 |
| MA0461.1 |  | hlh-15 | 32 | 1.68 | 0.02600 |
| Dlx1\_1741 |  | ceh-43 | 22 | 2.06 | 0.02700 |
| pTH6569 |  | ceh-43 | 21 | 2.12 | 0.02700 |
| K562b\_GATA1\_UCD |  | elt-1 (0.91) | 52 | 1.25 | 0.02800 |
| Nkx1-1\_3856 |  | ceh-30 | 20 | 2.17 | 0.02800 |
| Six1\_0935 |  | ceh-32 | 45 | 1.38 | 0.02800 |
| Hoxb4\_2627 |  | lin-39 (0.9) | 22 | 2.05 | 0.02800 |
| Pknox2\_3077 |  | ceh-32 | 12 | 3.09 | 0.02900 |
| pTH6449 |  | ceh-43 | 51 | 1.27 | 0.02900 |
| Hoxd13\_2356 |  | pal-1 (0.67) | 31 | 1.69 | 0.02900 |
| Vax1\_3499 |  | C02F12.10 | 21 | 2.10 | 0.03000 |
| Hoxa5\_3415 |  | lin-39 (0.9) | 21 | 2.10 | 0.03000 |
| MA0118.1 |  | ref-2 | 24 | 1.94 | 0.03100 |
| Hoxa6\_1040 |  | lin-39 (0.9) | 22 | 2.04 | 0.03100 |
| pTH3998 |  | tbx-39 | 14 | 2.72 | 0.03100 |
| pTH3091 |  | ZC328.2 | 45 | 1.38 | 0.03200 |
| PDX1\_do |  | lin-39 (0.9) ceh-12 alr-1 | 8 | 4.38 | 0.03200 |
| V$GATA3\_01 |  | elt-1 (0.91) | 49 | 1.30 | 0.03300 |
| pTH10707 |  | ceh-34 elt-6 elt-7 | 42 | 1.43 | 0.03300 |
| eve\_FlyReg\_FBgn0000606 |  | ceh-53 | 7 | 5.01 | 0.03400 |
| Myf6\_3824 |  | hlh-1 | 21 | 2.07 | 0.03500 |
| Hoxa11\_2218 |  | php-3 | 40 | 1.46 | 0.03500 |
| Eip75B\_SANGER\_5\_FBgn0000568 |  | nhr-118 | 50 | 1.28 | 0.03600 |
| GM12878\_GABP\_HudsonAlpha |  | lin-1 (0.76) | 19 | 2.18 | 0.03700 |
| Hoxa4\_3426 |  | lin-39 (0.9) | 21 | 2.05 | 0.03800 |
| pTH10031 |  | mbr-1 | 23 | 1.95 | 0.03800 |
| MA0599.1 |  | klf-1 klf-2 | 13 | 2.78 | 0.03900 |
| COT1\_si |  | nhr-2 | 37 | 1.52 | 0.03900 |
| Hoxa2\_3079 |  | lin-39 (0.9) | 19 | 2.16 | 0.04000 |
| V$CDC5\_01 |  | D1081.8 (0.56) | 11 | 3.13 | 0.04100 |
| Hoxa3\_2783 |  | lin-39 (0.9) | 20 | 2.09 | 0.04100 |
| pTH9072 |  | klf-1 | 12 | 2.92 | 0.04200 |
| Hoxb3\_1720 |  | lin-39 (0.9) | 19 | 2.14 | 0.04300 |
| V$GATA1\_01 |  | elt-1 (0.91) | 37 | 1.51 | 0.04300 |
| MA0095.2 |  | lsy-2 | 44 | 1.37 | 0.04400 |
| Oli\_da\_SANGER\_5\_1\_FBgn0032651 |  | hlh-32 (0.9) | 19 | 2.13 | 0.04500 |
| pTH10805 |  | ztf-16 | 32 | 1.61 | 0.04600 |
| V$GATA3\_03 |  | elt-1 (0.91) | 13 | 2.71 | 0.04700 |
| pTH6425 |  | ceh-20 (0.76) | 41 | 1.42 | 0.04700 |
| MEIS2\_2 |  | ceh-32 | 14 | 2.56 | 0.04800 |

### Correlated (and anti-correlated) transcription factors

|  |  |
| --- | --- |
| **Transcription factor** | **Correlation** |
| bar-1 | 0.96 |
| unc-62 | 0.95 |
| elt-1 | 0.91 |
| hlh-32 | 0.90 |
| lin-39 | 0.90 |
| mab-5 | 0.89 |
| mab-9 | 0.86 |
| hlh-17 | 0.81 |
| F21D5.9 | 0.79 |
| ceh-13 | 0.78 |
| ceh-20 | 0.76 |
| lin-1 | 0.76 |
| tlp-1 | 0.76 |
| cnd-1 | 0.72 |
| egl-46 | 0.70 |
| ceh-6 | 0.70 |
| unc-3 | 0.70 |
| pal-1 | 0.67 |
| unc-39 | 0.67 |
| F21A9.2 | 0.62 |
| F26H9.2 | 0.62 |
| C38D4.7 | 0.60 |
| zag-1 | 0.60 |
| attf-4 | 0.59 |
| sea-1 | 0.59 |
| nhr-56 | -0.62 |
| nhr-71 | -0.63 |
| nhr-183 | -0.65 |
| nhr-258 | -0.65 |
| nhr-61 | -0.66 |
| nhr-63 | -0.66 |
| fos-1 | -0.67 |
| nhr-62 | -0.67 |
| nhr-128 | -0.67 |
| jun-1 | -0.68 |
| nhr-213 | -0.71 |
| nhr-182 | -0.72 |
| nhr-45 | -0.73 |
| nhr-116 | -0.73 |
| nhr-58 | -0.73 |
| nhr-96 | -0.74 |
| nhr-134 | -0.74 |
| zip-2 | -0.74 |
| nhr-50 | -0.76 |
| nhr-135 | -0.78 |
| nhr-275 | -0.78 |
| nhr-21 | -0.78 |
| nhr-84 | -0.79 |
| nhr-286 | -0.81 |
| nhr-102 | -0.84 |

### ChIP peaks enriched

|  |  |  |  |  |
| --- | --- | --- | --- | --- |
| **Gene** | **Experiment** | **Number of upstream peaks** | **Enrichment** | **FDR corrected p** |
| dpl-1 | DPL-1\_Larvae-L4-stage | 5 | 7.1 | 0.024 |
