# Supplementary material for: Overlapping cell population expression profiling and regulatory inference in C. elegans
Source: BMC Genomics. 2016 Feb 29;17:159. doi: 10.1186/s12864-016-2482-z (PMC4772325; doi:10.1186/s12864-016-2482-z)
Supplement: Additional file 13: — Web supplement. (DOC 21 kb) [file 12864_2016_2482_MOESM13_ESM.zip › sortWeb/clusters/hier.300.clusters/118.html]

Cluster 118 

## Cluster 118

### Expression

| cnd-1 rep. 1 | cnd-1 rep. 2 | cnd-1 rep. 3 | pha-4 rep. 1 | pha-4 rep. 2 | pha-4 rep. 3 | ceh-27 | ceh-36 | ceh-6 | F21D5.9 | mir-57 | mls-2 | pal-1 | pros-1 | ttx-3 | unc-130 | hlh-16 | irx-1 | ceh-6 (+) hlh-16 (+) | ceh-6 (+) hlh-16 (-) | ceh-6 (-) hlh-16 (+) | cnd-1 singlets | pha-4 singlets | 0 | 60 | 120 | 150 | 180 | 240 | 330 | 390 | 420 | 480 | 540 | 570 | 600 | 630 | 660 | NAME | Functional description |
| --- | --- | --- | --- | --- | --- | --- | --- | --- | --- | --- | --- | --- | --- | --- | --- | --- | --- | --- | --- | --- | --- | --- | --- | --- | --- | --- | --- | --- | --- | --- | --- | --- | --- | --- | --- | --- | --- | --- | --- |
|  |  |  |  |  |  |  |  |  |  |  |  |  |  |  |  |  |  |  |  |  |  |  |  |  |  |  |  |  |  |  |  |  |  |  |  |  |  | *his-66* | HIStone |
|  |  |  |  |  |  |  |  |  |  |  |  |  |  |  |  |  |  |  |  |  |  |  |  |  |  |  |  |  |  |  |  |  |  |  |  |  |  | T24D8.11 |  |
|  |  |  |  |  |  |  |  |  |  |  |  |  |  |  |  |  |  |  |  |  |  |  |  |  |  |  |  |  |  |  |  |  |  |  |  |  |  | Y8A9A.2 |  |
|  |  |  |  |  |  |  |  |  |  |  |  |  |  |  |  |  |  |  |  |  |  |  |  |  |  |  |  |  |  |  |  |  |  |  |  |  |  | *fbxb-64* | F-box B protein |
|  |  |  |  |  |  |  |  |  |  |  |  |  |  |  |  |  |  |  |  |  |  |  |  |  |  |  |  |  |  |  |  |  |  |  |  |  |  | *cpg-2* | Chondroitin ProteoGlycan |
|  |  |  |  |  |  |  |  |  |  |  |  |  |  |  |  |  |  |  |  |  |  |  |  |  |  |  |  |  |  |  |  |  |  |  |  |  |  | F58H7.5 |  |
|  |  |  |  |  |  |  |  |  |  |  |  |  |  |  |  |  |  |  |  |  |  |  |  |  |  |  |  |  |  |  |  |  |  |  |  |  |  | Y105E8A.28 |  |
|  |  |  |  |  |  |  |  |  |  |  |  |  |  |  |  |  |  |  |  |  |  |  |  |  |  |  |  |  |  |  |  |  |  |  |  |  |  | *his-18* | HIStone |
|  |  |  |  |  |  |  |  |  |  |  |  |  |  |  |  |  |  |  |  |  |  |  |  |  |  |  |  |  |  |  |  |  |  |  |  |  |  | F21F3.4 |  |
|  |  |  |  |  |  |  |  |  |  |  |  |  |  |  |  |  |  |  |  |  |  |  |  |  |  |  |  |  |  |  |  |  |  |  |  |  |  | F56A8.8 |  |
|  |  |  |  |  |  |  |  |  |  |  |  |  |  |  |  |  |  |  |  |  |  |  |  |  |  |  |  |  |  |  |  |  |  |  |  |  |  | *sdz-30* | SKN-1 Dependent Zygotic transcript |
|  |  |  |  |  |  |  |  |  |  |  |  |  |  |  |  |  |  |  |  |  |  |  |  |  |  |  |  |  |  |  |  |  |  |  |  |  |  | *gadr-2* | GAstrulation Defective, Redundant |
|  |  |  |  |  |  |  |  |  |  |  |  |  |  |  |  |  |  |  |  |  |  |  |  |  |  |  |  |  |  |  |  |  |  |  |  |  |  | *best-10* | BESTrophin (chloride channel) homolog |
|  |  |  |  |  |  |  |  |  |  |  |  |  |  |  |  |  |  |  |  |  |  |  |  |  |  |  |  |  |  |  |  |  |  |  |  |  |  | *fbxb-83* | F-box B protein |
|  |  |  |  |  |  |  |  |  |  |  |  |  |  |  |  |  |  |  |  |  |  |  |  |  |  |  |  |  |  |  |  |  |  |  |  |  |  | F10D2.12 |  |
|  |  |  |  |  |  |  |  |  |  |  |  |  |  |  |  |  |  |  |  |  |  |  |  |  |  |  |  |  |  |  |  |  |  |  |  |  |  | *tkt-1* | TransKeTolase homolog |
|  |  |  |  |  |  |  |  |  |  |  |  |  |  |  |  |  |  |  |  |  |  |  |  |  |  |  |  |  |  |  |  |  |  |  |  |  |  | F13H6.3 |  |
|  |  |  |  |  |  |  |  |  |  |  |  |  |  |  |  |  |  |  |  |  |  |  |  |  |  |  |  |  |  |  |  |  |  |  |  |  |  | F01D5.8 |  |
|  |  |  |  |  |  |  |  |  |  |  |  |  |  |  |  |  |  |  |  |  |  |  |  |  |  |  |  |  |  |  |  |  |  |  |  |  |  | *wee-1.2* | WEE homolog |
|  |  |  |  |  |  |  |  |  |  |  |  |  |  |  |  |  |  |  |  |  |  |  |  |  |  |  |  |  |  |  |  |  |  |  |  |  |  | *ccch-5* | CCCH-type zinc finger putative transcription factor |
|  |  |  |  |  |  |  |  |  |  |  |  |  |  |  |  |  |  |  |  |  |  |  |  |  |  |  |  |  |  |  |  |  |  |  |  |  |  | C18G1.6 |  |
|  |  |  |  |  |  |  |  |  |  |  |  |  |  |  |  |  |  |  |  |  |  |  |  |  |  |  |  |  |  |  |  |  |  |  |  |  |  | *sams-5* | S-Adenosyl Methionine Synthetase |
|  |  |  |  |  |  |  |  |  |  |  |  |  |  |  |  |  |  |  |  |  |  |  |  |  |  |  |  |  |  |  |  |  |  |  |  |  |  | *cyp-13A1* | CYtochrome P450 family |
|  |  |  |  |  |  |  |  |  |  |  |  |  |  |  |  |  |  |  |  |  |  |  |  |  |  |  |  |  |  |  |  |  |  |  |  |  |  | *cyp-34A9* | CYtochrome P450 family |
|  |  |  |  |  |  |  |  |  |  |  |  |  |  |  |  |  |  |  |  |  |  |  |  |  |  |  |  |  |  |  |  |  |  |  |  |  |  | C06H5.6 |  |
|  |  |  |  |  |  |  |  |  |  |  |  |  |  |  |  |  |  |  |  |  |  |  |  |  |  |  |  |  |  |  |  |  |  |  |  |  |  | F55G11.4 |  |
|  |  |  |  |  |  |  |  |  |  |  |  |  |  |  |  |  |  |  |  |  |  |  |  |  |  |  |  |  |  |  |  |  |  |  |  |  |  | Y102A11A.3 |  |
|  |  |  |  |  |  |  |  |  |  |  |  |  |  |  |  |  |  |  |  |  |  |  |  |  |  |  |  |  |  |  |  |  |  |  |  |  |  | *bcmo-2* | Beta-Carotene 15,15'-MonoOxygenase |
|  |  |  |  |  |  |  |  |  |  |  |  |  |  |  |  |  |  |  |  |  |  |  |  |  |  |  |  |  |  |  |  |  |  |  |  |  |  | Y47G6A.5 |  |
|  |  |  |  |  |  |  |  |  |  |  |  |  |  |  |  |  |  |  |  |  |  |  |  |  |  |  |  |  |  |  |  |  |  |  |  |  |  | *pgp-1* | P-GlycoProtein related |
|  |  |  |  |  |  |  |  |  |  |  |  |  |  |  |  |  |  |  |  |  |  |  |  |  |  |  |  |  |  |  |  |  |  |  |  |  |  | Y39B6A.1 |  |
|  |  |  |  |  |  |  |  |  |  |  |  |  |  |  |  |  |  |  |  |  |  |  |  |  |  |  |  |  |  |  |  |  |  |  |  |  |  | R07C3.3 |  |
|  |  |  |  |  |  |  |  |  |  |  |  |  |  |  |  |  |  |  |  |  |  |  |  |  |  |  |  |  |  |  |  |  |  |  |  |  |  | *pqn-71* | Prion-like-(Q/N-rich)-domain-bearing protein |
|  |  |  |  |  |  |  |  |  |  |  |  |  |  |  |  |  |  |  |  |  |  |  |  |  |  |  |  |  |  |  |  |  |  |  |  |  |  | W04B5.3 |  |
|  |  |  |  |  |  |  |  |  |  |  |  |  |  |  |  |  |  |  |  |  |  |  |  |  |  |  |  |  |  |  |  |  |  |  |  |  |  | *ugt-37* | UDP-GlucuronosylTransferase |
|  |  |  |  |  |  |  |  |  |  |  |  |  |  |  |  |  |  |  |  |  |  |  |  |  |  |  |  |  |  |  |  |  |  |  |  |  |  | *gba-1* | beta-GlucocereBrosidAse |
|  |  |  |  |  |  |  |  |  |  |  |  |  |  |  |  |  |  |  |  |  |  |  |  |  |  |  |  |  |  |  |  |  |  |  |  |  |  | *mtl-2* | MeTaLlothionein |
|  |  |  |  |  |  |  |  |  |  |  |  |  |  |  |  |  |  |  |  |  |  |  |  |  |  |  |  |  |  |  |  |  |  |  |  |  |  | C45G7.4 |  |
|  |  |  |  |  |  |  |  |  |  |  |  |  |  |  |  |  |  |  |  |  |  |  |  |  |  |  |  |  |  |  |  |  |  |  |  |  |  | *eat-20* | EATing: abnormal pharyngeal pumping |
|  |  |  |  |  |  |  |  |  |  |  |  |  |  |  |  |  |  |  |  |  |  |  |  |  |  |  |  |  |  |  |  |  |  |  |  |  |  | *gsr-1* | Glutathione diSulfide Reductase |
|  |  |  |  |  |  |  |  |  |  |  |  |  |  |  |  |  |  |  |  |  |  |  |  |  |  |  |  |  |  |  |  |  |  |  |  |  |  | F59B1.8 |  |
|  |  |  |  |  |  |  |  |  |  |  |  |  |  |  |  |  |  |  |  |  |  |  |  |  |  |  |  |  |  |  |  |  |  |  |  |  |  | *fkh-7* | ForKHead transcription factor family |
|  |  |  |  |  |  |  |  |  |  |  |  |  |  |  |  |  |  |  |  |  |  |  |  |  |  |  |  |  |  |  |  |  |  |  |  |  |  | C06B8.7 |  |
|  |  |  |  |  |  |  |  |  |  |  |  |  |  |  |  |  |  |  |  |  |  |  |  |  |  |  |  |  |  |  |  |  |  |  |  |  |  | *pdi-6* | Protein Disulfide Isomerase |
|  |  |  |  |  |  |  |  |  |  |  |  |  |  |  |  |  |  |  |  |  |  |  |  |  |  |  |  |  |  |  |  |  |  |  |  |  |  | *spl-1* | Sphingosine Phosphate Lyase |
|  |  |  |  |  |  |  |  |  |  |  |  |  |  |  |  |  |  |  |  |  |  |  |  |  |  |  |  |  |  |  |  |  |  |  |  |  |  | *enpl-1* | ENdoPLasmin homolog |
|  |  |  |  |  |  |  |  |  |  |  |  |  |  |  |  |  |  |  |  |  |  |  |  |  |  |  |  |  |  |  |  |  |  |  |  |  |  | *fbxb-80* | F-box B protein |
|  |  |  |  |  |  |  |  |  |  |  |  |  |  |  |  |  |  |  |  |  |  |  |  |  |  |  |  |  |  |  |  |  |  |  |  |  |  | *sup-37* | SUPpressor |
|  |  |  |  |  |  |  |  |  |  |  |  |  |  |  |  |  |  |  |  |  |  |  |  |  |  |  |  |  |  |  |  |  |  |  |  |  |  | C27A12.4 |  |
|  |  |  |  |  |  |  |  |  |  |  |  |  |  |  |  |  |  |  |  |  |  |  |  |  |  |  |  |  |  |  |  |  |  |  |  |  |  | *ugt-61* | UDP-GlucuronosylTransferase |
|  |  |  |  |  |  |  |  |  |  |  |  |  |  |  |  |  |  |  |  |  |  |  |  |  |  |  |  |  |  |  |  |  |  |  |  |  |  | *ceh-60* | C. Elegans Homeobox |
|  |  |  |  |  |  |  |  |  |  |  |  |  |  |  |  |  |  |  |  |  |  |  |  |  |  |  |  |  |  |  |  |  |  |  |  |  |  | *sdz-2* | SKN-1 Dependent Zygotic transcript |
|  |  |  |  |  |  |  |  |  |  |  |  |  |  |  |  |  |  |  |  |  |  |  |  |  |  |  |  |  |  |  |  |  |  |  |  |  |  | F43C11.10 |  |
|  |  |  |  |  |  |  |  |  |  |  |  |  |  |  |  |  |  |  |  |  |  |  |  |  |  |  |  |  |  |  |  |  |  |  |  |  |  | *dnj-27* | DNaJ domain (prokaryotic heat shock protein) |
|  |  |  |  |  |  |  |  |  |  |  |  |  |  |  |  |  |  |  |  |  |  |  |  |  |  |  |  |  |  |  |  |  |  |  |  |  |  | *glit-1* | GLIoTactin (Drosophila neuroligin-like) homolog |
|  |  |  |  |  |  |  |  |  |  |  |  |  |  |  |  |  |  |  |  |  |  |  |  |  |  |  |  |  |  |  |  |  |  |  |  |  |  | T14G8.3 |  |
|  |  |  |  |  |  |  |  |  |  |  |  |  |  |  |  |  |  |  |  |  |  |  |  |  |  |  |  |  |  |  |  |  |  |  |  |  |  | C47D12.4 |  |
|  |  |  |  |  |  |  |  |  |  |  |  |  |  |  |  |  |  |  |  |  |  |  |  |  |  |  |  |  |  |  |  |  |  |  |  |  |  | *gadr-3* | GAstrulation Defective, Redundant |
|  |  |  |  |  |  |  |  |  |  |  |  |  |  |  |  |  |  |  |  |  |  |  |  |  |  |  |  |  |  |  |  |  |  |  |  |  |  | K02E10.1 |  |
|  |  |  |  |  |  |  |  |  |  |  |  |  |  |  |  |  |  |  |  |  |  |  |  |  |  |  |  |  |  |  |  |  |  |  |  |  |  | C05A9.2 |  |
|  |  |  |  |  |  |  |  |  |  |  |  |  |  |  |  |  |  |  |  |  |  |  |  |  |  |  |  |  |  |  |  |  |  |  |  |  |  | *lam-3* | LAMinin related. See also lmb- |
|  |  |  |  |  |  |  |  |  |  |  |  |  |  |  |  |  |  |  |  |  |  |  |  |  |  |  |  |  |  |  |  |  |  |  |  |  |  | *fbxc-34* | F-box C protein |
|  |  |  |  |  |  |  |  |  |  |  |  |  |  |  |  |  |  |  |  |  |  |  |  |  |  |  |  |  |  |  |  |  |  |  |  |  |  | D1022.4 |  |
|  |  |  |  |  |  |  |  |  |  |  |  |  |  |  |  |  |  |  |  |  |  |  |  |  |  |  |  |  |  |  |  |  |  |  |  |  |  | Y54G11A.7 |  |
|  |  |  |  |  |  |  |  |  |  |  |  |  |  |  |  |  |  |  |  |  |  |  |  |  |  |  |  |  |  |  |  |  |  |  |  |  |  | *sptl-1* | Serine Palmitoyl Transferase famiLy |
|  |  |  |  |  |  |  |  |  |  |  |  |  |  |  |  |  |  |  |  |  |  |  |  |  |  |  |  |  |  |  |  |  |  |  |  |  |  | F17A9.4 |  |
|  |  |  |  |  |  |  |  |  |  |  |  |  |  |  |  |  |  |  |  |  |  |  |  |  |  |  |  |  |  |  |  |  |  |  |  |  |  | C04F12.7 |  |
|  |  |  |  |  |  |  |  |  |  |  |  |  |  |  |  |  |  |  |  |  |  |  |  |  |  |  |  |  |  |  |  |  |  |  |  |  |  | *srh-171* | Serpentine Receptor, class H |
|  |  |  |  |  |  |  |  |  |  |  |  |  |  |  |  |  |  |  |  |  |  |  |  |  |  |  |  |  |  |  |  |  |  |  |  |  |  | *anr-42* | Antisense Non-coding RNA |
|  |  |  |  |  |  |  |  |  |  |  |  |  |  |  |  |  |  |  |  |  |  |  |  |  |  |  |  |  |  |  |  |  |  |  |  |  |  | *ceh-45* | C. Elegans Homeobox |
|  |  |  |  |  |  |  |  |  |  |  |  |  |  |  |  |  |  |  |  |  |  |  |  |  |  |  |  |  |  |  |  |  |  |  |  |  |  | Y51H7C.10 |  |
|  |  |  |  |  |  |  |  |  |  |  |  |  |  |  |  |  |  |  |  |  |  |  |  |  |  |  |  |  |  |  |  |  |  |  |  |  |  | *eyg-1* | EYG (Drosophila eyegone) homolog |
|  |  |  |  |  |  |  |  |  |  |  |  |  |  |  |  |  |  |  |  |  |  |  |  |  |  |  |  |  |  |  |  |  |  |  |  |  |  | F19B10.11 |  |
|  |  |  |  |  |  |  |  |  |  |  |  |  |  |  |  |  |  |  |  |  |  |  |  |  |  |  |  |  |  |  |  |  |  |  |  |  |  | F59B2.3 |  |
|  |  |  |  |  |  |  |  |  |  |  |  |  |  |  |  |  |  |  |  |  |  |  |  |  |  |  |  |  |  |  |  |  |  |  |  |  |  | *ivd-1* | IsoValeryl-CoA Dehydrogenase |
|  |  |  |  |  |  |  |  |  |  |  |  |  |  |  |  |  |  |  |  |  |  |  |  |  |  |  |  |  |  |  |  |  |  |  |  |  |  | F55F3.2 |  |
|  |  |  |  |  |  |  |  |  |  |  |  |  |  |  |  |  |  |  |  |  |  |  |  |  |  |  |  |  |  |  |  |  |  |  |  |  |  | *nhr-60* | Nuclear Hormone Receptor family |
|  |  |  |  |  |  |  |  |  |  |  |  |  |  |  |  |  |  |  |  |  |  |  |  |  |  |  |  |  |  |  |  |  |  |  |  |  |  | H08J11.2 |  |
|  |  |  |  |  |  |  |  |  |  |  |  |  |  |  |  |  |  |  |  |  |  |  |  |  |  |  |  |  |  |  |  |  |  |  |  |  |  | Y48G1BM.9 |  |
|  |  |  |  |  |  |  |  |  |  |  |  |  |  |  |  |  |  |  |  |  |  |  |  |  |  |  |  |  |  |  |  |  |  |  |  |  |  | Y48G1BR.1 |  |
|  |  |  |  |  |  |  |  |  |  |  |  |  |  |  |  |  |  |  |  |  |  |  |  |  |  |  |  |  |  |  |  |  |  |  |  |  |  | *fkh-6* | ForKHead transcription factor family |
|  |  |  |  |  |  |  |  |  |  |  |  |  |  |  |  |  |  |  |  |  |  |  |  |  |  |  |  |  |  |  |  |  |  |  |  |  |  | Y37A1A.4 |  |
|  |  |  |  |  |  |  |  |  |  |  |  |  |  |  |  |  |  |  |  |  |  |  |  |  |  |  |  |  |  |  |  |  |  |  |  |  |  | *lat-2* | LATrophilin receptor |
|  |  |  |  |  |  |  |  |  |  |  |  |  |  |  |  |  |  |  |  |  |  |  |  |  |  |  |  |  |  |  |  |  |  |  |  |  |  | Y75B7AR.1 |  |
|  |  |  |  |  |  |  |  |  |  |  |  |  |  |  |  |  |  |  |  |  |  |  |  |  |  |  |  |  |  |  |  |  |  |  |  |  |  | F07H5.6 |  |
|  |  |  |  |  |  |  |  |  |  |  |  |  |  |  |  |  |  |  |  |  |  |  |  |  |  |  |  |  |  |  |  |  |  |  |  |  |  | *pqn-10* | Prion-like-(Q/N-rich)-domain-bearing protein |
|  |  |  |  |  |  |  |  |  |  |  |  |  |  |  |  |  |  |  |  |  |  |  |  |  |  |  |  |  |  |  |  |  |  |  |  |  |  | Y57A10A.24 |  |
|  |  |  |  |  |  |  |  |  |  |  |  |  |  |  |  |  |  |  |  |  |  |  |  |  |  |  |  |  |  |  |  |  |  |  |  |  |  | *dpf-1* | Dipeptidyl Peptidase Four (IV) family |
|  |  |  |  |  |  |  |  |  |  |  |  |  |  |  |  |  |  |  |  |  |  |  |  |  |  |  |  |  |  |  |  |  |  |  |  |  |  | *col-83* | COLlagen |
|  |  |  |  |  |  |  |  |  |  |  |  |  |  |  |  |  |  |  |  |  |  |  |  |  |  |  |  |  |  |  |  |  |  |  |  |  |  | Y17G7B.23 |  |
|  |  |  |  |  |  |  |  |  |  |  |  |  |  |  |  |  |  |  |  |  |  |  |  |  |  |  |  |  |  |  |  |  |  |  |  |  |  | F42A8.1 |  |
|  |  |  |  |  |  |  |  |  |  |  |  |  |  |  |  |  |  |  |  |  |  |  |  |  |  |  |  |  |  |  |  |  |  |  |  |  |  | ZC504.1 |  |
|  |  |  |  |  |  |  |  |  |  |  |  |  |  |  |  |  |  |  |  |  |  |  |  |  |  |  |  |  |  |  |  |  |  |  |  |  |  | E03H4.8 |  |
|  |  |  |  |  |  |  |  |  |  |  |  |  |  |  |  |  |  |  |  |  |  |  |  |  |  |  |  |  |  |  |  |  |  |  |  |  |  | *chil-24* | CHItinase-Like |
|  |  |  |  |  |  |  |  |  |  |  |  |  |  |  |  |  |  |  |  |  |  |  |  |  |  |  |  |  |  |  |  |  |  |  |  |  |  | *tag-83* | Temporarily Assigned Gene name |
|  |  |  |  |  |  |  |  |  |  |  |  |  |  |  |  |  |  |  |  |  |  |  |  |  |  |  |  |  |  |  |  |  |  |  |  |  |  | B0212.6 |  |
|  |  |  |  |  |  |  |  |  |  |  |  |  |  |  |  |  |  |  |  |  |  |  |  |  |  |  |  |  |  |  |  |  |  |  |  |  |  | F46F11.11 |  |
|  |  |  |  |  |  |  |  |  |  |  |  |  |  |  |  |  |  |  |  |  |  |  |  |  |  |  |  |  |  |  |  |  |  |  |  |  |  | C53C11.5 |  |
|  |  |  |  |  |  |  |  |  |  |  |  |  |  |  |  |  |  |  |  |  |  |  |  |  |  |  |  |  |  |  |  |  |  |  |  |  |  | *ptr-19* | PaTched Related family |
|  |  |  |  |  |  |  |  |  |  |  |  |  |  |  |  |  |  |  |  |  |  |  |  |  |  |  |  |  |  |  |  |  |  |  |  |  |  | R09E10.5 |  |
|  |  |  |  |  |  |  |  |  |  |  |  |  |  |  |  |  |  |  |  |  |  |  |  |  |  |  |  |  |  |  |  |  |  |  |  |  |  | *abt-4* | ABC Transporter family |
|  |  |  |  |  |  |  |  |  |  |  |  |  |  |  |  |  |  |  |  |  |  |  |  |  |  |  |  |  |  |  |  |  |  |  |  |  |  | M162.6 |  |
|  |  |  |  |  |  |  |  |  |  |  |  |  |  |  |  |  |  |  |  |  |  |  |  |  |  |  |  |  |  |  |  |  |  |  |  |  |  | *clec-259* | C-type LECtin |
|  |  |  |  |  |  |  |  |  |  |  |  |  |  |  |  |  |  |  |  |  |  |  |  |  |  |  |  |  |  |  |  |  |  |  |  |  |  | *clec-78* | C-type LECtin |
|  |  |  |  |  |  |  |  |  |  |  |  |  |  |  |  |  |  |  |  |  |  |  |  |  |  |  |  |  |  |  |  |  |  |  |  |  |  | F47E1.5 |  |
|  |  |  |  |  |  |  |  |  |  |  |  |  |  |  |  |  |  |  |  |  |  |  |  |  |  |  |  |  |  |  |  |  |  |  |  |  |  | F47C12.1 |  |
|  |  |  |  |  |  |  |  |  |  |  |  |  |  |  |  |  |  |  |  |  |  |  |  |  |  |  |  |  |  |  |  |  |  |  |  |  |  | E03H12.9 |  |
|  |  |  |  |  |  |  |  |  |  |  |  |  |  |  |  |  |  |  |  |  |  |  |  |  |  |  |  |  |  |  |  |  |  |  |  |  |  | F35H8.10 |  |
|  |  |  |  |  |  |  |  |  |  |  |  |  |  |  |  |  |  |  |  |  |  |  |  |  |  |  |  |  |  |  |  |  |  |  |  |  |  | ZK262.3 |  |
|  |  |  |  |  |  |  |  |  |  |  |  |  |  |  |  |  |  |  |  |  |  |  |  |  |  |  |  |  |  |  |  |  |  |  |  |  |  | ZC250.6 |  |
|  |  |  |  |  |  |  |  |  |  |  |  |  |  |  |  |  |  |  |  |  |  |  |  |  |  |  |  |  |  |  |  |  |  |  |  |  |  | B0432.7 |  |
|  |  |  |  |  |  |  |  |  |  |  |  |  |  |  |  |  |  |  |  |  |  |  |  |  |  |  |  |  |  |  |  |  |  |  |  |  |  | *pha-2* | defective PHArynx development |
|  |  |  |  |  |  |  |  |  |  |  |  |  |  |  |  |  |  |  |  |  |  |  |  |  |  |  |  |  |  |  |  |  |  |  |  |  |  | R09D1.4 |  |
|  |  |  |  |  |  |  |  |  |  |  |  |  |  |  |  |  |  |  |  |  |  |  |  |  |  |  |  |  |  |  |  |  |  |  |  |  |  | *fat-5* | FATty acid desaturase |
|  |  |  |  |  |  |  |  |  |  |  |  |  |  |  |  |  |  |  |  |  |  |  |  |  |  |  |  |  |  |  |  |  |  |  |  |  |  | C44H9.8 |  |
|  |  |  |  |  |  |  |  |  |  |  |  |  |  |  |  |  |  |  |  |  |  |  |  |  |  |  |  |  |  |  |  |  |  |  |  |  |  | C44H9.5 |  |
|  |  |  |  |  |  |  |  |  |  |  |  |  |  |  |  |  |  |  |  |  |  |  |  |  |  |  |  |  |  |  |  |  |  |  |  |  |  | F49B2.6 |  |
|  |  |  |  |  |  |  |  |  |  |  |  |  |  |  |  |  |  |  |  |  |  |  |  |  |  |  |  |  |  |  |  |  |  |  |  |  |  | *nas-14* | Nematode AStacin protease |
|  |  |  |  |  |  |  |  |  |  |  |  |  |  |  |  |  |  |  |  |  |  |  |  |  |  |  |  |  |  |  |  |  |  |  |  |  |  | T10E10.4 |  |
|  |  |  |  |  |  |  |  |  |  |  |  |  |  |  |  |  |  |  |  |  |  |  |  |  |  |  |  |  |  |  |  |  |  |  |  |  |  | *myo-5* | MYOsin heavy chain structural genes |
|  |  |  |  |  |  |  |  |  |  |  |  |  |  |  |  |  |  |  |  |  |  |  |  |  |  |  |  |  |  |  |  |  |  |  |  |  |  | *myo-1* | MYOsin heavy chain structural genes |
|  |  |  |  |  |  |  |  |  |  |  |  |  |  |  |  |  |  |  |  |  |  |  |  |  |  |  |  |  |  |  |  |  |  |  |  |  |  | *myo-2* | MYOsin heavy chain structural genes |
|  |  |  |  |  |  |  |  |  |  |  |  |  |  |  |  |  |  |  |  |  |  |  |  |  |  |  |  |  |  |  |  |  |  |  |  |  |  | T20B6.3 |  |
|  |  |  |  |  |  |  |  |  |  |  |  |  |  |  |  |  |  |  |  |  |  |  |  |  |  |  |  |  |  |  |  |  |  |  |  |  |  | F41G3.3 |  |
|  |  |  |  |  |  |  |  |  |  |  |  |  |  |  |  |  |  |  |  |  |  |  |  |  |  |  |  |  |  |  |  |  |  |  |  |  |  | ZC84.6 |  |
|  |  |  |  |  |  |  |  |  |  |  |  |  |  |  |  |  |  |  |  |  |  |  |  |  |  |  |  |  |  |  |  |  |  |  |  |  |  | ZC84.1 |  |
|  |  |  |  |  |  |  |  |  |  |  |  |  |  |  |  |  |  |  |  |  |  |  |  |  |  |  |  |  |  |  |  |  |  |  |  |  |  | *mop-25.1* | MO25 (MOuse embryo scaffolding Protein) homolog |
|  |  |  |  |  |  |  |  |  |  |  |  |  |  |  |  |  |  |  |  |  |  |  |  |  |  |  |  |  |  |  |  |  |  |  |  |  |  | *itx-1* | InTestinal NeureXin-like |
|  |  |  |  |  |  |  |  |  |  |  |  |  |  |  |  |  |  |  |  |  |  |  |  |  |  |  |  |  |  |  |  |  |  |  |  |  |  | Y71H2AM.13 |  |
|  |  |  |  |  |  |  |  |  |  |  |  |  |  |  |  |  |  |  |  |  |  |  |  |  |  |  |  |  |  |  |  |  |  |  |  |  |  | *acs-22* | fatty Acid CoA Synthetase family |
|  |  |  |  |  |  |  |  |  |  |  |  |  |  |  |  |  |  |  |  |  |  |  |  |  |  |  |  |  |  |  |  |  |  |  |  |  |  | Y18H1A.9 |  |
|  |  |  |  |  |  |  |  |  |  |  |  |  |  |  |  |  |  |  |  |  |  |  |  |  |  |  |  |  |  |  |  |  |  |  |  |  |  | *ncx-2* | Na/Ca eXchangers |
|  |  |  |  |  |  |  |  |  |  |  |  |  |  |  |  |  |  |  |  |  |  |  |  |  |  |  |  |  |  |  |  |  |  |  |  |  |  | *aagr-1* | Acid Alpha Glucosidase Relate |
|  |  |  |  |  |  |  |  |  |  |  |  |  |  |  |  |  |  |  |  |  |  |  |  |  |  |  |  |  |  |  |  |  |  |  |  |  |  | K03A11.6 |  |
|  |  |  |  |  |  |  |  |  |  |  |  |  |  |  |  |  |  |  |  |  |  |  |  |  |  |  |  |  |  |  |  |  |  |  |  |  |  | R03C1.1 |  |
|  |  |  |  |  |  |  |  |  |  |  |  |  |  |  |  |  |  |  |  |  |  |  |  |  |  |  |  |  |  |  |  |  |  |  |  |  |  | C23H3.9 |  |
|  |  |  |  |  |  |  |  |  |  |  |  |  |  |  |  |  |  |  |  |  |  |  |  |  |  |  |  |  |  |  |  |  |  |  |  |  |  | T07C4.3 |  |
|  |  |  |  |  |  |  |  |  |  |  |  |  |  |  |  |  |  |  |  |  |  |  |  |  |  |  |  |  |  |  |  |  |  |  |  |  |  | *inx-6* | INneXin |
|  |  |  |  |  |  |  |  |  |  |  |  |  |  |  |  |  |  |  |  |  |  |  |  |  |  |  |  |  |  |  |  |  |  |  |  |  |  | *pitr-3* | PIT (mammalian phosphate transporter) Related |
|  |  |  |  |  |  |  |  |  |  |  |  |  |  |  |  |  |  |  |  |  |  |  |  |  |  |  |  |  |  |  |  |  |  |  |  |  |  | Y70C5C.1 |  |
|  |  |  |  |  |  |  |  |  |  |  |  |  |  |  |  |  |  |  |  |  |  |  |  |  |  |  |  |  |  |  |  |  |  |  |  |  |  | E01G6.1 |  |
|  |  |  |  |  |  |  |  |  |  |  |  |  |  |  |  |  |  |  |  |  |  |  |  |  |  |  |  |  |  |  |  |  |  |  |  |  |  | *pqn-26* | Prion-like-(Q/N-rich)-domain-bearing protein |
|  |  |  |  |  |  |  |  |  |  |  |  |  |  |  |  |  |  |  |  |  |  |  |  |  |  |  |  |  |  |  |  |  |  |  |  |  |  | *chs-2* | CHitin Synthase |
|  |  |  |  |  |  |  |  |  |  |  |  |  |  |  |  |  |  |  |  |  |  |  |  |  |  |  |  |  |  |  |  |  |  |  |  |  |  | T06A4.1 |  |
|  |  |  |  |  |  |  |  |  |  |  |  |  |  |  |  |  |  |  |  |  |  |  |  |  |  |  |  |  |  |  |  |  |  |  |  |  |  | Y43F8B.3 |  |
|  |  |  |  |  |  |  |  |  |  |  |  |  |  |  |  |  |  |  |  |  |  |  |  |  |  |  |  |  |  |  |  |  |  |  |  |  |  | H19M22.3 |  |
|  |  |  |  |  |  |  |  |  |  |  |  |  |  |  |  |  |  |  |  |  |  |  |  |  |  |  |  |  |  |  |  |  |  |  |  |  |  | *ceh-22* | C. Elegans Homeobox |
|  |  |  |  |  |  |  |  |  |  |  |  |  |  |  |  |  |  |  |  |  |  |  |  |  |  |  |  |  |  |  |  |  |  |  |  |  |  | T21D12.7 |  |
|  |  |  |  |  |  |  |  |  |  |  |  |  |  |  |  |  |  |  |  |  |  |  |  |  |  |  |  |  |  |  |  |  |  |  |  |  |  | K10D3.4 |  |
|  |  |  |  |  |  |  |  |  |  |  |  |  |  |  |  |  |  |  |  |  |  |  |  |  |  |  |  |  |  |  |  |  |  |  |  |  |  | *cdh-5* | CaDHerin family |
|  |  |  |  |  |  |  |  |  |  |  |  |  |  |  |  |  |  |  |  |  |  |  |  |  |  |  |  |  |  |  |  |  |  |  |  |  |  | *cyn-17* | CYclophyliN |
|  |  |  |  |  |  |  |  |  |  |  |  |  |  |  |  |  |  |  |  |  |  |  |  |  |  |  |  |  |  |  |  |  |  |  |  |  |  | *pqn-13* | Prion-like-(Q/N-rich)-domain-bearing protein |
|  |  |  |  |  |  |  |  |  |  |  |  |  |  |  |  |  |  |  |  |  |  |  |  |  |  |  |  |  |  |  |  |  |  |  |  |  |  | K04H4.2 |  |
|  |  |  |  |  |  |  |  |  |  |  |  |  |  |  |  |  |  |  |  |  |  |  |  |  |  |  |  |  |  |  |  |  |  |  |  |  |  | *lgx-1* | Lin-12 and Glp-1 X-hybridizing |
|  |  |  |  |  |  |  |  |  |  |  |  |  |  |  |  |  |  |  |  |  |  |  |  |  |  |  |  |  |  |  |  |  |  |  |  |  |  | *sptl-2* | Serine Palmitoyl Transferase famiLy |
|  |  |  |  |  |  |  |  |  |  |  |  |  |  |  |  |  |  |  |  |  |  |  |  |  |  |  |  |  |  |  |  |  |  |  |  |  |  | F30H5.3 |  |
|  |  |  |  |  |  |  |  |  |  |  |  |  |  |  |  |  |  |  |  |  |  |  |  |  |  |  |  |  |  |  |  |  |  |  |  |  |  | F49E10.2 |  |
|  |  |  |  |  |  |  |  |  |  |  |  |  |  |  |  |  |  |  |  |  |  |  |  |  |  |  |  |  |  |  |  |  |  |  |  |  |  | C33C12.10 |  |
|  |  |  |  |  |  |  |  |  |  |  |  |  |  |  |  |  |  |  |  |  |  |  |  |  |  |  |  |  |  |  |  |  |  |  |  |  |  | W05B2.2 |  |
|  |  |  |  |  |  |  |  |  |  |  |  |  |  |  |  |  |  |  |  |  |  |  |  |  |  |  |  |  |  |  |  |  |  |  |  |  |  | *pes-4* | Patterned Expression Site |
|  |  |  |  |  |  |  |  |  |  |  |  |  |  |  |  |  |  |  |  |  |  |  |  |  |  |  |  |  |  |  |  |  |  |  |  |  |  | *wsp-1* | WASP (actin cytoskeleton modulator) homolog |
|  |  |  |  |  |  |  |  |  |  |  |  |  |  |  |  |  |  |  |  |  |  |  |  |  |  |  |  |  |  |  |  |  |  |  |  |  |  | *best-24* | BESTrophin (chloride channel) homolog |
|  |  |  |  |  |  |  |  |  |  |  |  |  |  |  |  |  |  |  |  |  |  |  |  |  |  |  |  |  |  |  |  |  |  |  |  |  |  | F44B9.9 |  |
|  |  |  |  |  |  |  |  |  |  |  |  |  |  |  |  |  |  |  |  |  |  |  |  |  |  |  |  |  |  |  |  |  |  |  |  |  |  | C18E9.7 |  |
|  |  |  |  |  |  |  |  |  |  |  |  |  |  |  |  |  |  |  |  |  |  |  |  |  |  |  |  |  |  |  |  |  |  |  |  |  |  | R07B1.9 |  |
|  |  |  |  |  |  |  |  |  |  |  |  |  |  |  |  |  |  |  |  |  |  |  |  |  |  |  |  |  |  |  |  |  |  |  |  |  |  | R12A1.3 |  |
|  |  |  |  |  |  |  |  |  |  |  |  |  |  |  |  |  |  |  |  |  |  |  |  |  |  |  |  |  |  |  |  |  |  |  |  |  |  | F35A5.4 |  |
|  |  |  |  |  |  |  |  |  |  |  |  |  |  |  |  |  |  |  |  |  |  |  |  |  |  |  |  |  |  |  |  |  |  |  |  |  |  | *elf-1* | see eff |
|  |  |  |  |  |  |  |  |  |  |  |  |  |  |  |  |  |  |  |  |  |  |  |  |  |  |  |  |  |  |  |  |  |  |  |  |  |  | F35H10.10 |  |
|  |  |  |  |  |  |  |  |  |  |  |  |  |  |  |  |  |  |  |  |  |  |  |  |  |  |  |  |  |  |  |  |  |  |  |  |  |  | *mam-1* | MAM (Meprin, A5-protein, PTPmu) domain protein |
|  |  |  |  |  |  |  |  |  |  |  |  |  |  |  |  |  |  |  |  |  |  |  |  |  |  |  |  |  |  |  |  |  |  |  |  |  |  | Y47D3B.6 |  |
|  |  |  |  |  |  |  |  |  |  |  |  |  |  |  |  |  |  |  |  |  |  |  |  |  |  |  |  |  |  |  |  |  |  |  |  |  |  | *dpf-6* | Dipeptidyl Peptidase Four (IV) family |
|  |  |  |  |  |  |  |  |  |  |  |  |  |  |  |  |  |  |  |  |  |  |  |  |  |  |  |  |  |  |  |  |  |  |  |  |  |  | M03E7.4 |  |
|  |  |  |  |  |  |  |  |  |  |  |  |  |  |  |  |  |  |  |  |  |  |  |  |  |  |  |  |  |  |  |  |  |  |  |  |  |  | T25E4.1 |  |
|  |  |  |  |  |  |  |  |  |  |  |  |  |  |  |  |  |  |  |  |  |  |  |  |  |  |  |  |  |  |  |  |  |  |  |  |  |  | D2096.6 |  |
|  |  |  |  |  |  |  |  |  |  |  |  |  |  |  |  |  |  |  |  |  |  |  |  |  |  |  |  |  |  |  |  |  |  |  |  |  |  | R02F11.1 |  |
|  |  |  |  |  |  |  |  |  |  |  |  |  |  |  |  |  |  |  |  |  |  |  |  |  |  |  |  |  |  |  |  |  |  |  |  |  |  | F40E10.5 |  |
|  |  |  |  |  |  |  |  |  |  |  |  |  |  |  |  |  |  |  |  |  |  |  |  |  |  |  |  |  |  |  |  |  |  |  |  |  |  | *abu-14* | Activated in Blocked Unfolded protein response |
|  |  |  |  |  |  |  |  |  |  |  |  |  |  |  |  |  |  |  |  |  |  |  |  |  |  |  |  |  |  |  |  |  |  |  |  |  |  | M02G9.1 |  |
|  |  |  |  |  |  |  |  |  |  |  |  |  |  |  |  |  |  |  |  |  |  |  |  |  |  |  |  |  |  |  |  |  |  |  |  |  |  | T11F9.12 |  |
|  |  |  |  |  |  |  |  |  |  |  |  |  |  |  |  |  |  |  |  |  |  |  |  |  |  |  |  |  |  |  |  |  |  |  |  |  |  | T19H12.6 |  |
|  |  |  |  |  |  |  |  |  |  |  |  |  |  |  |  |  |  |  |  |  |  |  |  |  |  |  |  |  |  |  |  |  |  |  |  |  |  | *lron-2* | eLRR (extracellular Leucine-Rich Repeat) ONly |
|  |  |  |  |  |  |  |  |  |  |  |  |  |  |  |  |  |  |  |  |  |  |  |  |  |  |  |  |  |  |  |  |  |  |  |  |  |  | D2005.6 |  |
|  |  |  |  |  |  |  |  |  |  |  |  |  |  |  |  |  |  |  |  |  |  |  |  |  |  |  |  |  |  |  |  |  |  |  |  |  |  | *tat-2* | Transbilayer Amphipath Transporters (subfamily IV P-type ATPase) |
|  |  |  |  |  |  |  |  |  |  |  |  |  |  |  |  |  |  |  |  |  |  |  |  |  |  |  |  |  |  |  |  |  |  |  |  |  |  | *sulp-3* | SULfate Permease family |
|  |  |  |  |  |  |  |  |  |  |  |  |  |  |  |  |  |  |  |  |  |  |  |  |  |  |  |  |  |  |  |  |  |  |  |  |  |  | *acd-5* | ACid-sensitive Degenerin |
|  |  |  |  |  |  |  |  |  |  |  |  |  |  |  |  |  |  |  |  |  |  |  |  |  |  |  |  |  |  |  |  |  |  |  |  |  |  | F02C12.1 |  |
|  |  |  |  |  |  |  |  |  |  |  |  |  |  |  |  |  |  |  |  |  |  |  |  |  |  |  |  |  |  |  |  |  |  |  |  |  |  | *src-2* | SRC oncogene related |
|  |  |  |  |  |  |  |  |  |  |  |  |  |  |  |  |  |  |  |  |  |  |  |  |  |  |  |  |  |  |  |  |  |  |  |  |  |  | *spin-2* | SPINster (Drosophila lysosomal permease) homolog |
|  |  |  |  |  |  |  |  |  |  |  |  |  |  |  |  |  |  |  |  |  |  |  |  |  |  |  |  |  |  |  |  |  |  |  |  |  |  | T22B11.4 |  |
|  |  |  |  |  |  |  |  |  |  |  |  |  |  |  |  |  |  |  |  |  |  |  |  |  |  |  |  |  |  |  |  |  |  |  |  |  |  | F48E3.8 |  |
|  |  |  |  |  |  |  |  |  |  |  |  |  |  |  |  |  |  |  |  |  |  |  |  |  |  |  |  |  |  |  |  |  |  |  |  |  |  | *pqn-73* | Prion-like-(Q/N-rich)-domain-bearing protein |
|  |  |  |  |  |  |  |  |  |  |  |  |  |  |  |  |  |  |  |  |  |  |  |  |  |  |  |  |  |  |  |  |  |  |  |  |  |  | *cky-1* | CKY homolog |
|  |  |  |  |  |  |  |  |  |  |  |  |  |  |  |  |  |  |  |  |  |  |  |  |  |  |  |  |  |  |  |  |  |  |  |  |  |  | C25F6.7 |  |
|  |  |  |  |  |  |  |  |  |  |  |  |  |  |  |  |  |  |  |  |  |  |  |  |  |  |  |  |  |  |  |  |  |  |  |  |  |  | T10H10.2 |  |
|  |  |  |  |  |  |  |  |  |  |  |  |  |  |  |  |  |  |  |  |  |  |  |  |  |  |  |  |  |  |  |  |  |  |  |  |  |  | *gmeb-2* | GMEB (Glucocorticoid Modulatory Element Binding protein) transcriptional regulator homolog |
|  |  |  |  |  |  |  |  |  |  |  |  |  |  |  |  |  |  |  |  |  |  |  |  |  |  |  |  |  |  |  |  |  |  |  |  |  |  | F53B3.3 |  |
|  |  |  |  |  |  |  |  |  |  |  |  |  |  |  |  |  |  |  |  |  |  |  |  |  |  |  |  |  |  |  |  |  |  |  |  |  |  | F56C9.8 |  |
|  |  |  |  |  |  |  |  |  |  |  |  |  |  |  |  |  |  |  |  |  |  |  |  |  |  |  |  |  |  |  |  |  |  |  |  |  |  | W06F12.2 |  |
|  |  |  |  |  |  |  |  |  |  |  |  |  |  |  |  |  |  |  |  |  |  |  |  |  |  |  |  |  |  |  |  |  |  |  |  |  |  | *lron-1* | eLRR (extracellular Leucine-Rich Repeat) ONly |
|  |  |  |  |  |  |  |  |  |  |  |  |  |  |  |  |  |  |  |  |  |  |  |  |  |  |  |  |  |  |  |  |  |  |  |  |  |  | *oat-1* | Organic Anion Transporter |
|  |  |  |  |  |  |  |  |  |  |  |  |  |  |  |  |  |  |  |  |  |  |  |  |  |  |  |  |  |  |  |  |  |  |  |  |  |  | K05G3.2 |  |
|  |  |  |  |  |  |  |  |  |  |  |  |  |  |  |  |  |  |  |  |  |  |  |  |  |  |  |  |  |  |  |  |  |  |  |  |  |  | R52.5 |  |
|  |  |  |  |  |  |  |  |  |  |  |  |  |  |  |  |  |  |  |  |  |  |  |  |  |  |  |  |  |  |  |  |  |  |  |  |  |  | *str-9* | Seven TM Receptor |
|  |  |  |  |  |  |  |  |  |  |  |  |  |  |  |  |  |  |  |  |  |  |  |  |  |  |  |  |  |  |  |  |  |  |  |  |  |  | T20F5.5 |  |
|  |  |  |  |  |  |  |  |  |  |  |  |  |  |  |  |  |  |  |  |  |  |  |  |  |  |  |  |  |  |  |  |  |  |  |  |  |  | Y49G5B.1 |  |
|  |  |  |  |  |  |  |  |  |  |  |  |  |  |  |  |  |  |  |  |  |  |  |  |  |  |  |  |  |  |  |  |  |  |  |  |  |  | *sri-7* | Serpentine Receptor, class I |
|  |  |  |  |  |  |  |  |  |  |  |  |  |  |  |  |  |  |  |  |  |  |  |  |  |  |  |  |  |  |  |  |  |  |  |  |  |  | F55H12.5 |  |
|  |  |  |  |  |  |  |  |  |  |  |  |  |  |  |  |  |  |  |  |  |  |  |  |  |  |  |  |  |  |  |  |  |  |  |  |  |  | C39B5.5 |  |
|  |  |  |  |  |  |  |  |  |  |  |  |  |  |  |  |  |  |  |  |  |  |  |  |  |  |  |  |  |  |  |  |  |  |  |  |  |  | R08F11.4 |  |
|  |  |  |  |  |  |  |  |  |  |  |  |  |  |  |  |  |  |  |  |  |  |  |  |  |  |  |  |  |  |  |  |  |  |  |  |  |  | *math-23* | MATH (meprin-associated Traf homology) domain containing |
|  |  |  |  |  |  |  |  |  |  |  |  |  |  |  |  |  |  |  |  |  |  |  |  |  |  |  |  |  |  |  |  |  |  |  |  |  |  | F53C3.1 |  |
|  |  |  |  |  |  |  |  |  |  |  |  |  |  |  |  |  |  |  |  |  |  |  |  |  |  |  |  |  |  |  |  |  |  |  |  |  |  | *ubc-24* | UBiquitin Conjugating enzyme |
|  |  |  |  |  |  |  |  |  |  |  |  |  |  |  |  |  |  |  |  |  |  |  |  |  |  |  |  |  |  |  |  |  |  |  |  |  |  | C06B3.7 |  |
|  |  |  |  |  |  |  |  |  |  |  |  |  |  |  |  |  |  |  |  |  |  |  |  |  |  |  |  |  |  |  |  |  |  |  |  |  |  | B0207.5 |  |
|  |  |  |  |  |  |  |  |  |  |  |  |  |  |  |  |  |  |  |  |  |  |  |  |  |  |  |  |  |  |  |  |  |  |  |  |  |  | Y67D8C.20 |  |
|  |  |  |  |  |  |  |  |  |  |  |  |  |  |  |  |  |  |  |  |  |  |  |  |  |  |  |  |  |  |  |  |  |  |  |  |  |  | F43E2.11 |  |
|  |  |  |  |  |  |  |  |  |  |  |  |  |  |  |  |  |  |  |  |  |  |  |  |  |  |  |  |  |  |  |  |  |  |  |  |  |  | C40H1.3 |  |
|  |  |  |  |  |  |  |  |  |  |  |  |  |  |  |  |  |  |  |  |  |  |  |  |  |  |  |  |  |  |  |  |  |  |  |  |  |  | *hrde-1* |  |
|  |  |  |  |  |  |  |  |  |  |  |  |  |  |  |  |  |  |  |  |  |  |  |  |  |  |  |  |  |  |  |  |  |  |  |  |  |  | F20B6.9 |  |
|  |  |  |  |  |  |  |  |  |  |  |  |  |  |  |  |  |  |  |  |  |  |  |  |  |  |  |  |  |  |  |  |  |  |  |  |  |  | F20B6.7 |  |
|  |  |  |  |  |  |  |  |  |  |  |  |  |  |  |  |  |  |  |  |  |  |  |  |  |  |  |  |  |  |  |  |  |  |  |  |  |  | ZK546.4 |  |
|  |  |  |  |  |  |  |  |  |  |  |  |  |  |  |  |  |  |  |  |  |  |  |  |  |  |  |  |  |  |  |  |  |  |  |  |  |  | *col-96* | COLlagen |
|  |  |  |  |  |  |  |  |  |  |  |  |  |  |  |  |  |  |  |  |  |  |  |  |  |  |  |  |  |  |  |  |  |  |  |  |  |  | *ugt-40* | UDP-GlucuronosylTransferase |
|  |  |  |  |  |  |  |  |  |  |  |  |  |  |  |  |  |  |  |  |  |  |  |  |  |  |  |  |  |  |  |  |  |  |  |  |  |  | W03G1.5 |  |
|  |  |  |  |  |  |  |  |  |  |  |  |  |  |  |  |  |  |  |  |  |  |  |  |  |  |  |  |  |  |  |  |  |  |  |  |  |  | C18A3.7 |  |
|  |  |  |  |  |  |  |  |  |  |  |  |  |  |  |  |  |  |  |  |  |  |  |  |  |  |  |  |  |  |  |  |  |  |  |  |  |  | T04A8.13 |  |

### Phenotypes enriched

none found

### Anatomy terms enriched

none found

### GO terms enriched

|  |  |  |
| --- | --- | --- |
| **GO term** | **Number of genes** | **FDR-corrected p-value** |
| sphingolipid metabolic process | 4 | 0.034 |

### Expression clusters enriched

|  |  |  |  |
| --- | --- | --- | --- |
| **Group name** | **Number in cluster** | **Enrichment** | **FDR corrected p** |
| Genes with increased expression after 24 hours of infection by P.lumniescens Fold changes shown are pathogen vs OP50. WBPaper00038438:P.lumniescens\_24hr\_upregulated\_RNAseq | 87 | 1.99 | 9.65e-09 |
| Germline-enriched and sex-biased expression profile cluster C. | 39 | 3.07 | 2.16e-07 |
| Genes with increased expression after 24 hours of infection by E.faecalis Fold changes shown are pathogen vs OP50. WBPaper00038438:E.faecalis\_24hr\_upregulated\_RNAseq | 85 | 1.89 | 2.66e-07 |
| Genes depleted in muscle cells (0hr muscle dataset). Dissociated myo-3::GFP embryos were cultured for 0 hours before FACS sorting. | 54 | 2.18 | 1.03e-05 |
| Genes up regulated by mir-243(n4759). | 55 | 2.11 | 2.24e-05 |
| A large cluster of genes up-regulated during early larval development.. | 53 | 2.00 | 2.15e-04 |
| Genes with decreased expression after 24 hours of infection by S.marcescens Fold changes shown are pathogen vs OP50. WBPaper00038438:S.marcescens\_24hr\_downregulated\_RNAseq | 38 | 2.31 | 3.77e-04 |
| Genes upregulated by alg-1(-). | 44 | 2.14 | 3.98e-04 |
| Genes down regulated in crh-1(nn3315) comparing to in N2. | 24 | 3.06 | 4.35e-04 |
| Class E gene expression showed no change in lin-14(lf) in L1, up regulation in lin-4(lf) in L2. | 18 | 3.70 | 7.29e-04 |
| Total muscle depleted genes (complete list of non-overlapping genes from the 0hr and 24hr muscle depleted datasets). | 63 | 1.75 | 1.16e-03 |
| Genes downregulated on Comamonas DA1877 relative to E. coli OP50, Young adult | 17 | 3.73 | 1.22e-03 |
| Genes with differeiential expression after exposed to Au-NP. | 28 | 2.53 | 2.00e-03 |
| Genome-wide analysis of developmental and sex-regulated gene expression profile. cgc4489\_group\_8 | 22 | 2.68 | 7.41e-03 |
| Genes that were upregulated in lin-15B(n744). | 38 | 2.01 | 7.47e-03 |
| Genes that showed lower expression in N2 than in DR1350. | 35 | 2.06 | 9.11e-03 |
| Genes that showed decreased expression after exposure to 7.5uM CH3HgCl for 24 hours. | 35 | 2.04 | 1.11e-02 |
| Genes significantly enriched (> 2x, FDR < 5%) in a particular cell-type versus a reference sample of all cells at the same stage. WBPaper00037950:pharyngeal-muscle\_embryo\_enriched | 22 | 2.54 | 1.53e-02 |
| Genes that showed lower expression in RIL-17 than in RIL-14. | 9 | 4.97 | 2.08e-02 |
| Expression Pattern Group G, enriched for genes involved in locomotion. | 39 | 1.80 | 4.58e-02 |
| Genes with expression level down regulated after treatment with Methylmercury (MeHg) by RNAseq analysis. | 13 | 3.23 | 4.61e-02 |
| Genes down regulated after fed by 25 mg/ml pRJ-Fr.5 for 24 h beginning at the L4 stage. | 14 | 3.05 | 4.75e-02 |

### Motifs enriched

|  |  |  |  |  |  |
| --- | --- | --- | --- | --- | --- |
| **Motif** | **Logo** | **Possible orthologs** | **Number of motifs in cluster** | **Enrichment** | **FDR corrected p** |
| MA0016.1 |  | nhr-213 (0.8) nhr-2 nhr-10 nhr-69 nhr-19 | 180 | 1.38 | 4.2e-09 |
| HepG2\_FOXA2\_HudsonAlpha |  | lin-31 let-381 | 101 | 1.84 | 1.4e-08 |
| FOXL1\_1 |  | pha-4 (0.95) fkh-7 (0.86) fkh-8 fkh-10 daf-16 lin-31 let-381 | 109 | 1.71 | 1.1e-07 |
| V$SP1\_Q6 |  | klf-2 (0.62) ZC328.2 klf-1 | 108 | 1.71 | 1.4e-07 |
| Nkx2-9\_3082 |  | dsc-1 ceh-24 | 28 | 4.08 | 2.6e-07 |
| pTH10807 |  | F13H6.1 nhr-177 nhr-86 nhr-2 | 65 | 2.20 | 2.6e-07 |
| V$MSX1\_01 |  | ceh-31 ceh-30 ceh-1 | 118 | 1.61 | 3.9e-07 |
| Egr1\_2580 |  | ZC328.2 | 177 | 1.32 | 1.0e-06 |
| pTH10696 |  | Y44A6D.3 | 58 | 2.25 | 1.2e-06 |
| SP4\_f1 |  | klf-2 (0.62) Y53H1A.2 (-0.56) plp-2 | 101 | 1.68 | 1.8e-06 |
| pTH3091 |  | klf-2 (0.62) ZC328.2 klf-1 | 179 | 1.30 | 2.2e-06 |
| MA0264.1 |  | ceh-22 (0.98) dsc-1 ceh-24 | 34 | 3.09 | 2.7e-06 |
| F$MCM1\_01 |  | K02D7.2 unc-120 C24A1.2 | 202 | 1.21 | 3.3e-06 |
| slp2\_SANGER\_5\_FBgn0004567 |  | fkh-7 (0.86) fkh-8 fkh-10 daf-16 lin-31 let-381 | 172 | 1.32 | 3.4e-06 |
| MA0470.1 |  | efl-1 F49E12.6 | 137 | 1.45 | 3.8e-06 |
| NR2F1\_4 |  | nhr-2 nhr-19 | 116 | 1.56 | 4.5e-06 |
| pTH3998 |  | tbx-39 | 126 | 1.50 | 5.8e-06 |
| pTH9336 |  | mab-9 tbx-38 tbx-39 tbx-43 | 201 | 1.21 | 6.0e-06 |
| SP3\_1 |  | klf-2 (0.62) klf-1 | 178 | 1.29 | 6.0e-06 |
| GM12878\_ETS1\_HudsonAlpha |  | nhr-28 (0.83) lin-1 nhr-79 nhr-273 tbx-39 | 190 | 1.25 | 6.1e-06 |
| pTH2193 |  | nhr-213 (0.8) nhr-15 (0.71) nhr-239 (0.58) nhr-2 | 115 | 1.55 | 7.0e-06 |
| pTH10623 |  | scrt-1 (0.57) | 189 | 1.25 | 7.6e-06 |
| pTH5709 |  | nhr-213 (0.8) nhr-71 (0.61) nhr-68 nhr-2 nhr-10 nhr-6 Y67D8A.3 | 110 | 1.56 | 1.2e-05 |
| pTH2673 |  | fkh-10 lin-31 let-381 C34D1.1 | 90 | 1.70 | 1.3e-05 |
| pTH10647 |  | nhr-232 | 164 | 1.32 | 1.6e-05 |
| PTF1A\_f1 |  | lin-32 | 141 | 1.40 | 1.7e-05 |
| pTH10722 |  | ref-2 eor-1 egrh-3 | 177 | 1.28 | 1.7e-05 |
| MA0102.3 |  | C48E7.11 | 187 | 1.24 | 1.7e-05 |
| pTH9354 |  | ZC328.2 daf-16 | 84 | 1.73 | 1.9e-05 |
| pTH9085 |  | nhr-42 (0.77) | 110 | 1.55 | 2.1e-05 |
| Sp4\_1011 |  | klf-2 (0.62) sptf-3 klf-1 | 57 | 2.05 | 2.8e-05 |
| pTH9244 |  | tbx-39 | 129 | 1.44 | 3.3e-05 |
| pTH9387 |  | C34D1.1 | 175 | 1.27 | 3.9e-05 |
| disco-r-Cl1\_SANGER\_5\_FBgn0042650 |  | lin-31 F55C5.11 | 202 | 1.18 | 4.6e-05 |
| twi\_da\_SANGER\_5\_FBgn0003900 |  | hlh-32 hlh-8 hlh-15 ngn-1 | 182 | 1.25 | 4.7e-05 |
| pTH10714 |  | nhr-84 nhr-142 | 206 | 1.17 | 4.7e-05 |
| EPAS1\_si |  | daf-12 (0.58) Y5F2A.4 hif-1 ztf-3 | 178 | 1.26 | 5.6e-05 |
| POU3F2\_1 |  | unc-86 ceh-18 | 202 | 1.18 | 5.9e-05 |
| pTH2846 |  | lin-31 | 104 | 1.54 | 6.6e-05 |
| PAX8\_f1 |  | pax-2 | 170 | 1.27 | 7.9e-05 |
| pTH8556 |  | pax-2 | 163 | 1.29 | 8.4e-05 |
| NKX28\_f1 |  | ceh-24 C34H4.5 | 129 | 1.41 | 1.1e-04 |
| Atoh1\_1 |  | hlh-32 hlh-15 hlh-8 ngn-1 lin-31 | 186 | 1.22 | 1.2e-04 |
| pTH9043 |  | sem-2 (0.75) | 154 | 1.32 | 1.2e-04 |
| HXB1\_f1 |  | lin-39 ceh-20 ceh-12 | 196 | 1.19 | 1.5e-04 |
| I$CROC\_01 |  | mef-2 (0.86) let-381 | 54 | 1.98 | 1.6e-04 |
| GM12878\_SRF\_HudsonAlpha |  | hlh-10 unc-120 | 194 | 1.19 | 1.6e-04 |
| MA0456.1 |  | ref-2 | 95 | 1.56 | 1.6e-04 |
| MA0545.1 |  | hlh-14 hlh-11 hlh-15 hlh-1 | 190 | 1.20 | 1.6e-04 |
| CrebA\_SANGER\_5\_FBgn0004396 |  | atf-6 crh-1 atf-7 C27D6.4 | 107 | 1.49 | 1.7e-04 |
| pTH8333 |  | F39B2.1 ZC416.1 | 22 | 3.41 | 1.7e-04 |
| TFEC\_1 |  | pax-1 (0.92) hlh-30 aha-1 | 169 | 1.26 | 1.9e-04 |
| V$AREB6\_01 |  | ztf-6 | 183 | 1.22 | 1.9e-04 |
| MEIS2\_do |  | lin-39 ceh-32 | 101 | 1.52 | 2.0e-04 |
| FLI1\_f1 |  | lin-1 | 174 | 1.24 | 2.3e-04 |
| Eip75B\_SANGER\_5\_FBgn0000568 |  | nhr-213 (0.8) nhr-118 | 132 | 1.38 | 2.5e-04 |
| pTH5916 |  | efl-2 | 159 | 1.29 | 2.6e-04 |
| Irx2\_0900 |  | irx-1 | 86 | 1.60 | 2.6e-04 |
| V$AP2REP\_01 |  | ZC328.2 klf-1 | 176 | 1.24 | 2.7e-04 |
| MA0150.2 |  | fos-1 (0.61) sknr-1 jun-1 crh-1 | 185 | 1.21 | 2.9e-04 |
| EN2\_2 |  | ceh-16 ceh-31 | 184 | 1.21 | 3.2e-04 |
| pTH3751 |  | tbx-39 | 133 | 1.36 | 3.4e-04 |
| V$MYB\_Q6 |  | D1081.8 | 185 | 1.21 | 3.5e-04 |
| pTH2936 |  | nhr-239 (0.58) | 189 | 1.20 | 3.6e-04 |
| V$GATA1\_04 |  | elt-1 | 139 | 1.34 | 3.8e-04 |
| TCF4\_2 |  | hlh-2 | 182 | 1.21 | 4.1e-04 |
| V$PAX5\_02 |  | pax-2 pax-3 | 122 | 1.40 | 4.2e-04 |
| Smad3\_3805 |  | daf-8 | 189 | 1.19 | 4.5e-04 |
| pTH9173 |  | efl-2 | 155 | 1.29 | 4.9e-04 |
| V$LYF1\_01 |  | F26F4.8 mbr-1 | 90 | 1.55 | 5.2e-04 |
| V$FREAC7\_01 |  | lin-31 | 95 | 1.52 | 5.3e-04 |
| pTH6497 |  | lin-31 | 179 | 1.22 | 5.3e-04 |
| ss\_tgo\_SANGER\_10\_FBgn0015014 |  | aha-1 ahr-1 | 181 | 1.21 | 5.4e-04 |
| V$XFD3\_01 |  | let-381 | 94 | 1.52 | 5.6e-04 |
| pTH6327 |  | dsc-1 | 44 | 2.07 | 5.6e-04 |
| Zfp161\_2858 |  | pzf-1 | 90 | 1.54 | 5.6e-04 |
| Elf3 |  | C24A1.2 | 37 | 2.26 | 5.8e-04 |
| MA0579.1 |  | D1081.8 pax-2 F45H11.6 | 142 | 1.32 | 5.8e-04 |
| pTH10795 |  | ZC328.2 ref-2 lin-1 ztf-14 pax-3 | 166 | 1.25 | 6.2e-04 |
| MA0118.1 |  | ref-2 | 156 | 1.28 | 6.3e-04 |
| pTH9182 |  | tbx-39 | 124 | 1.38 | 6.9e-04 |
| pTH9260 |  | mel-28 | 157 | 1.27 | 7.0e-04 |
| pTH10769 |  | Y48G1C.6 | 123 | 1.38 | 7.0e-04 |
| pTH8649 |  | mbr-1 | 134 | 1.34 | 7.1e-04 |
| K562\_ZBTB7A\_HudsonAlpha |  | ZC328.2 | 70 | 1.68 | 7.2e-04 |
| MAX\_1 |  | mxl-1 (-0.64) K11D2.4 | 154 | 1.28 | 7.4e-04 |
| pTH5887 |  | lin-39 | 178 | 1.22 | 7.5e-04 |
| dm\_Max\_SANGER\_10\_FBgn0017578 |  | mxl-1 (-0.64) hlh-30 ref-1 aha-1 C27D6.4 | 111 | 1.43 | 7.6e-04 |
| HEN1\_si |  | hlh-15 | 162 | 1.26 | 7.6e-04 |
| MA0535.1 |  | daf-8 pax-2 F45H11.6 | 95 | 1.50 | 7.7e-04 |
| pTH10779 |  | nhr-182 (0.72) nhr-134 (0.65) | 82 | 1.58 | 7.8e-04 |
| CG31670\_SOLEXA\_5\_FBgn0031375 |  | F21A9.2 CELE\_Y38H8A.5 | 195 | 1.17 | 8.7e-04 |
| Hnf4a\_2640 |  | nhr-62 (0.84) | 91 | 1.52 | 8.8e-04 |
| ELF2\_f1 |  | lin-1 C24A1.2 | 204 | 1.15 | 8.8e-04 |
| TWST1\_f1 |  | hlh-2 ces-1 hlh-8 hlh-15 hlh-1 K02D7.2 | 179 | 1.21 | 8.8e-04 |
| ETS2\_f1 |  | lin-1 C24A1.2 | 148 | 1.29 | 9.1e-04 |
| Irx3\_0920 |  | irx-1 | 182 | 1.20 | 9.1e-04 |
| pTH5919 |  | irx-1 | 164 | 1.25 | 9.2e-04 |
| pTH10034 |  | nhr-66 (0.56) | 124 | 1.37 | 9.8e-04 |
| pTH6106 |  | nhr-182 (0.72) | 187 | 1.19 | 1.1e-03 |
| V$IK2\_01 |  | F26F4.8 | 192 | 1.17 | 1.1e-03 |
| CREM\_f1 |  | crh-1 | 64 | 1.71 | 1.2e-03 |
| pTH9135 |  | pop-1 | 159 | 1.25 | 1.4e-03 |
| Nsy-7 |  | hmg-5 nsy-7 | 93 | 1.49 | 1.4e-03 |
| pTH10823 |  | B0310.2 | 184 | 1.19 | 1.5e-03 |
| FOXK1\_1 |  | nhr-213 (0.8) lin-31 | 195 | 1.16 | 1.6e-03 |
| pTH9322 |  | nhr-10 | 130 | 1.33 | 1.7e-03 |
| MA0017.1 |  | nhr-62 (0.84) nhr-2 | 196 | 1.16 | 1.8e-03 |
| ECC-1\_ERALPHA\_HudsonAlpha |  | nhr-71 (0.61) | 166 | 1.23 | 1.8e-03 |
| pTH6591 |  | lin-31 | 178 | 1.20 | 1.9e-03 |
| pTH10717 |  | syd-9 | 60 | 1.72 | 2.0e-03 |
| EVX1\_1 |  | ceh-45 (0.81) ceh-53 (0.74) lin-39 ceh-16 ceh-43 ceh-14 lim-7 ceh-12 ceh-10 alr-1 ceh-1 ceh-18 | 91 | 1.48 | 2.0e-03 |
| pTH10721 |  | ztf-9 ceh-18 | 191 | 1.17 | 2.1e-03 |
| V$AREB6\_02 |  | ztf-6 | 185 | 1.18 | 2.1e-03 |
| ELF4\_1 |  | lin-1 nhr-19 C24A1.2 | 195 | 1.16 | 2.1e-03 |
| pTH5561 |  | nhr-239 (0.58) | 123 | 1.35 | 2.1e-03 |
| MA0007.2 |  | npax-1 nhr-255 | 70 | 1.62 | 2.2e-03 |
| pTH5083 |  | fos-1 (0.61) atf-5 | 181 | 1.19 | 2.2e-03 |
| Plagl1\_0972 |  | Y53H1A.2 (-0.56) | 134 | 1.31 | 2.2e-03 |
| pTH6641 |  | lin-31 | 175 | 1.20 | 2.3e-03 |
| V$TAXCREB\_02 |  | zip-3 crh-1 | 168 | 1.22 | 2.3e-03 |
| pTH9916 |  | crh-1 W08E12.1 | 165 | 1.23 | 2.4e-03 |
| FOXB1\_2 |  | ceh-53 (0.74) lin-31 | 70 | 1.61 | 2.5e-03 |
| pTH10026 |  | ceh-20 ces-1 ceh-32 F55C5.11 | 180 | 1.19 | 2.6e-03 |
| pTH10707 |  | ceh-34 (0.77) elt-1 elt-6 egl-27 elt-7 ceh-32 | 83 | 1.52 | 2.7e-03 |
| Dlx2\_2273 |  | ceh-43 | 201 | 1.14 | 2.8e-03 |
| V$GATA3\_01 |  | elt-1 | 181 | 1.19 | 2.8e-03 |
| pTH1001 |  | dnj-17 | 55 | 1.75 | 2.9e-03 |
| SOX10\_1 |  | sox-4 | 122 | 1.34 | 3.1e-03 |
| ELK3\_1 |  | lin-1 C24A1.2 | 195 | 1.15 | 3.2e-03 |
| V$GATA1\_01 |  | elt-1 | 158 | 1.24 | 3.2e-03 |
| pTH9149 |  | ztf-30 | 61 | 1.67 | 3.5e-03 |
| HLF\_si |  | ces-2 Y51H4A.4 | 187 | 1.17 | 3.6e-03 |
| MA0543.1 |  | eor-1 | 197 | 1.14 | 3.9e-03 |
| ELK3\_f1 |  | lin-1 | 187 | 1.17 | 3.9e-03 |
| Cutl1\_3494 |  | ceh-44 | 172 | 1.20 | 3.9e-03 |
| Mcm1 |  | unc-120 | 111 | 1.37 | 4.1e-03 |
| SRP000217\_Sox2 |  | sox-4 | 194 | 1.15 | 4.1e-03 |
| So\_Cell\_FBgn0003460 |  | ceh-32 | 101 | 1.41 | 4.2e-03 |
| MA0139.1 |  | Y5F2A.4 F58G1.2 | 165 | 1.22 | 4.2e-03 |
| CEBPE\_f1 |  | C48E7.11 | 185 | 1.17 | 4.6e-03 |
| pTH9326 |  | nhr-122 | 137 | 1.28 | 4.8e-03 |
| MA0544.1 |  | lin-22 gei-11 | 105 | 1.38 | 5.2e-03 |
| pTH6447 |  | ceh-19 (0.73) | 202 | 1.13 | 5.2e-03 |
| MA0146.2 |  | F58G1.2 aptf-1 | 75 | 1.53 | 5.2e-03 |
| pTH5922 |  | ceh-24 | 53 | 1.72 | 5.3e-03 |
| pTH9076 |  | C01G12.1 | 121 | 1.33 | 5.3e-03 |
| Msx2\_3449 |  | ceh-45 (0.81) alr-1 ceh-1 | 210 | 1.11 | 5.4e-03 |
| pTH5891 |  | nhr-49 (0.62) | 166 | 1.21 | 5.7e-03 |
| IRX2\_1 |  | irx-1 | 140 | 1.27 | 5.8e-03 |
| pTH5778 |  | egl-5 | 203 | 1.13 | 5.8e-03 |
| pTH2933 |  | F58G1.2 | 131 | 1.29 | 6.1e-03 |
| pTH9049 |  | ztf-2 | 146 | 1.25 | 6.1e-03 |
| Isx\_3445 |  | ceh-45 (0.81) ceh-43 ceh-12 alr-1 npax-3 | 200 | 1.13 | 6.5e-03 |
| V$EN1\_01 |  | ceh-16 atf-2 | 183 | 1.17 | 6.6e-03 |
| pTH5267 |  | hlh-32 ngn-1 | 169 | 1.20 | 6.7e-03 |
| ARNT2\_si |  | aha-1 | 55 | 1.68 | 7.1e-03 |
| MA0482.1 |  | elt-1 ztf-29 | 212 | 1.10 | 7.2e-03 |
| V$CDXA\_01 |  | ceh-13 | 165 | 1.20 | 7.9e-03 |
| RFX2\_f1 |  | daf-19 | 168 | 1.20 | 8.0e-03 |
| Elf2 |  | lin-1 C24A1.2 | 34 | 2.01 | 8.6e-03 |
| MA0246.1 |  | dmd-4 ceh-32 | 70 | 1.53 | 8.7e-03 |
| SMAD3\_f1 |  | daf-8 | 175 | 1.18 | 8.9e-03 |
| pTH5714 |  | nhr-239 (0.58) | 125 | 1.30 | 8.9e-03 |
| GATA5\_1 |  | elt-1 end-3 | 144 | 1.25 | 8.9e-03 |
| pTH1292 |  | pzf-1 ceh-24 | 197 | 1.13 | 9.4e-03 |
| V$TATA\_C |  | tbp-1 | 136 | 1.27 | 9.5e-03 |
| MA0027.1 |  | ceh-16 | 57 | 1.63 | 9.9e-03 |
| Irx6\_2623 |  | irx-1 | 188 | 1.15 | 9.9e-03 |
| MA0451.1 |  | nhr-2 | 132 | 1.28 | 9.9e-03 |
| pTH8996 |  | daf-8 sma-4 | 122 | 1.30 | 1.0e-02 |
| Elf5 |  | C24A1.2 | 37 | 1.92 | 1.0e-02 |
| pTH9262 |  | lin-54 | 161 | 1.21 | 1.0e-02 |
| Elf3\_3876 |  | C24A1.2 | 47 | 1.74 | 1.0e-02 |
| pTH9142 |  | C34D1.1 gei-11 | 175 | 1.18 | 1.1e-02 |
| MA0032.1 |  | let-381 | 152 | 1.22 | 1.1e-02 |
| pTH3477 |  | daf-16 | 93 | 1.40 | 1.1e-02 |
| pTH6425 |  | ceh-20 | 145 | 1.24 | 1.1e-02 |
| MA0483.1 |  | odd-1 | 85 | 1.43 | 1.1e-02 |
| HeLa-S3\_ZNF274\_UCD |  | C28G1.4 | 149 | 1.23 | 1.1e-02 |
| pTH5059 |  | lin-22 hlh-27 | 62 | 1.57 | 1.2e-02 |
| pTH9880 |  | end-1 | 105 | 1.35 | 1.2e-02 |
| pTH10030 |  | xbp-1 | 170 | 1.18 | 1.2e-02 |
| Dlx3\_1030 |  | ceh-43 | 198 | 1.13 | 1.3e-02 |
| Hoxa13\_3126 |  | pal-1 ceh-24 | 165 | 1.19 | 1.3e-02 |
| pTH10027 |  | M03D4.4 | 46 | 1.74 | 1.3e-02 |
| Hlxb9\_3422 |  | ceh-12 | 187 | 1.15 | 1.3e-02 |
| Pbx1\_3203 |  | ceh-20 | 198 | 1.13 | 1.3e-02 |
| TLX1\_f1 |  | ceh-19 (0.73) | 77 | 1.47 | 1.3e-02 |
| pTH5164 |  | hlh-32 D1081.8 | 175 | 1.17 | 1.4e-02 |
| pTH5257 |  | C48E7.11 | 128 | 1.28 | 1.4e-02 |
| Lbx2\_3869 |  | mls-2 | 176 | 1.17 | 1.4e-02 |
| pTH5914 |  | attf-1 | 58 | 1.59 | 1.5e-02 |
| LHX9\_2 |  | ceh-14 | 214 | 1.09 | 1.5e-02 |
| V$FAC1\_01 |  | gei-8 | 87 | 1.41 | 1.5e-02 |
| Hoxa9\_2622 |  | lin-39 php-3 | 204 | 1.11 | 1.6e-02 |
| pTH5976 |  | mxl-1 (-0.64) hlh-30 irx-1 | 187 | 1.14 | 1.6e-02 |
| Evx1\_3952 |  | ceh-53 (0.74) | 182 | 1.15 | 1.6e-02 |
| pTH5781 |  | ceh-32 | 19 | 2.56 | 1.6e-02 |
| pTH8411 |  | tbx-39 | 138 | 1.24 | 1.7e-02 |
| ZNF75A\_1 |  | F26F4.8 ztf-3 | 214 | 1.09 | 1.7e-02 |
| V$YY1\_02 |  | lsy-2 | 176 | 1.16 | 1.7e-02 |
| V$ROAZ\_01 |  | C09F5.3 (-0.67) Y53H1A.2 (-0.56) D1081.8 | 134 | 1.25 | 1.8e-02 |
| pTH8991 |  | cey-3 | 104 | 1.34 | 1.8e-02 |
| pTH10031 |  | mbr-1 | 131 | 1.26 | 1.8e-02 |
| En2\_0952 |  | ceh-16 | 200 | 1.12 | 1.8e-02 |
| Mw138 |  | ceh-48 dsc-1 | 178 | 1.16 | 1.8e-02 |
| MEF2A\_f1 |  | mef-2 (0.86) | 39 | 1.81 | 1.9e-02 |
| Irx5\_2385 |  | irx-1 | 90 | 1.38 | 1.9e-02 |
| TCF7L1\_1 |  | pop-1 | 103 | 1.34 | 2.0e-02 |
| HIF1A\_si |  | hif-1 | 159 | 1.20 | 2.0e-02 |
| MA0508.1 |  | blmp-1 | 203 | 1.11 | 2.0e-02 |
| Zfp652\_1 |  | ZK177.3 B0310.2 | 192 | 1.13 | 2.1e-02 |
| Isl2\_3430 |  | lim-7 | 115 | 1.30 | 2.1e-02 |
| OTX1\_f1 |  | ceh-45 (0.81) | 50 | 1.64 | 2.1e-02 |
| pTH9300 |  | dmd-3 | 209 | 1.10 | 2.1e-02 |
| pTH3712 |  | D1081.8 | 164 | 1.18 | 2.1e-02 |
| pTH9934 |  | Y53H1A.2 (-0.56) | 158 | 1.19 | 2.2e-02 |
| pTH5078 |  | ces-2 | 172 | 1.17 | 2.2e-02 |
| pTH3997 |  | C04F5.9 | 94 | 1.36 | 2.2e-02 |
| pTH5812 |  | ceh-14 | 180 | 1.15 | 2.3e-02 |
| pTH6436 |  | ceh-53 (0.74) | 96 | 1.35 | 2.3e-02 |
| CENPB\_1 |  | F21D5.4 | 46 | 1.68 | 2.3e-02 |
| Pknox2\_3077 |  | ceh-32 | 178 | 1.15 | 2.3e-02 |
| EN1\_4 |  | ceh-16 | 71 | 1.47 | 2.3e-02 |
| Sox15\_3457 |  | sox-4 | 211 | 1.09 | 2.3e-02 |
| Cdx2\_4272 |  | ceh-13 | 152 | 1.20 | 2.4e-02 |
| Sox11\_2266 |  | pop-1 sox-4 | 162 | 1.18 | 2.4e-02 |
| Hoxa7\_2668 |  | lin-39 | 114 | 1.29 | 2.4e-02 |
| pTH6071 |  | C46E10.8 C33G8.2 | 95 | 1.35 | 2.5e-02 |
| I$ABDB\_01 |  | ceh-24 | 107 | 1.31 | 2.7e-02 |
| BARHL2\_4 |  | ceh-31 | 194 | 1.12 | 2.8e-02 |
| pTH1014 |  | atf-5 | 22 | 2.24 | 2.8e-02 |
| pTH3120 |  | K11D2.4 | 180 | 1.15 | 2.8e-02 |
| pTH6486 |  | nhr-145 | 210 | 1.09 | 2.9e-02 |
| pTH3041 |  | atf-2 | 174 | 1.16 | 3.0e-02 |
| K562\_SP2\_HudsonAlpha |  | klf-2 (0.62) | 165 | 1.17 | 3.0e-02 |
| Tcf1\_2666 |  | hmbx-1 | 169 | 1.17 | 3.0e-02 |
| V$YY1\_01 |  | lsy-2 | 146 | 1.21 | 3.1e-02 |
| TBX3\_f1 |  | ceh-45 (0.81) tbx-39 | 140 | 1.22 | 3.1e-02 |
| MA0600.1 |  | daf-19 | 177 | 1.15 | 3.2e-02 |
| MA0164.1 |  | ztf-11 nhr-100 | 41 | 1.71 | 3.2e-02 |
| pTH9137 |  | nhr-65 (0.8) | 61 | 1.51 | 3.3e-02 |
| pTH9247 |  | C34D1.1 | 175 | 1.15 | 3.5e-02 |
| pTH2280 |  | mnm-2 | 74 | 1.42 | 3.5e-02 |
| pTH9261 |  | dmd-3 | 121 | 1.26 | 3.5e-02 |
| Mw160 |  | nhr-68 | 105 | 1.30 | 3.6e-02 |
| Hoxa4\_3426 |  | lin-39 | 114 | 1.28 | 3.7e-02 |
| HES1\_f1 |  | lin-22 | 32 | 1.85 | 3.8e-02 |
| I$DFD\_01 |  | lin-39 | 181 | 1.14 | 3.8e-02 |
| YBOX1\_f2 |  | cey-3 | 173 | 1.15 | 3.8e-02 |
| pTH10798 |  | Y75B8A.6 | 59 | 1.51 | 3.9e-02 |
| Hoxd1\_3448 |  | ceh-12 | 175 | 1.15 | 3.9e-02 |
| pTH10777 |  | dmd-3 | 204 | 1.10 | 3.9e-02 |
| pTH10816 |  | dmd-6 | 137 | 1.22 | 3.9e-02 |
| Hoxc4\_3491 |  | lin-39 | 92 | 1.34 | 4.0e-02 |
| Elf4 |  | C24A1.2 | 32 | 1.84 | 4.0e-02 |
| V$NCX\_01 |  | ceh-19 (0.73) | 182 | 1.14 | 4.0e-02 |
| V$CEBP\_01 |  | C48E7.11 | 156 | 1.18 | 4.0e-02 |
| pTH6569 |  | ceh-43 | 180 | 1.14 | 4.0e-02 |
| V$FOXJ2\_02 |  | lin-31 | 185 | 1.13 | 4.0e-02 |
| Dlx1\_1741 |  | ceh-43 | 197 | 1.11 | 4.1e-02 |
| Meox1\_2310 |  | ceh-31 | 191 | 1.12 | 4.1e-02 |
| MA0486.1 |  | Y53C10A.3 | 152 | 1.19 | 4.1e-02 |
| pTH10772 |  | ceh-52 | 165 | 1.17 | 4.2e-02 |
| Ara\_Cell\_FBgn0015904 |  | irx-1 | 91 | 1.34 | 4.2e-02 |
| pTH9220 |  | mbr-1 | 174 | 1.15 | 4.3e-02 |
| pTH8982 |  | ceh-48 | 137 | 1.22 | 4.3e-02 |
| pTH9216 |  | ceh-18 | 192 | 1.12 | 4.4e-02 |
| Zbtb12\_2932 |  | lsy-27 | 160 | 1.17 | 4.4e-02 |
| Bsx\_3483 |  | ceh-31 | 181 | 1.14 | 4.5e-02 |
| pTH5690 |  | ceh-32 | 166 | 1.16 | 4.8e-02 |
| V$GATA1\_03 |  | elt-1 | 22 | 2.12 | 4.8e-02 |
| Sox1\_1 |  | sox-4 | 144 | 1.20 | 4.9e-02 |
| pTH9198 |  | dmd-3 | 210 | 1.08 | 5.0e-02 |
| pTH9237 |  | mel-28 | 183 | 1.13 | 5.0e-02 |
| HepG2\_HSF1\_Stanford |  | Y53C10A.3 | 172 | 1.15 | 5.0e-02 |
| Etv3 |  | lin-1 | 60 | 1.48 | 5.0e-02 |

### Correlated (and anti-correlated) transcription factors

|  |  |
| --- | --- |
| **Transcription factor** | **Correlation** |
| ceh-22 | 0.98 |
| cky-1 | 0.98 |
| gmeb-2 | 0.98 |
| pha-4 | 0.95 |
| nhr-130 | 0.93 |
| ets-4 | 0.92 |
| pax-1 | 0.92 |
| ztf-16 | 0.90 |
| fkh-6 | 0.90 |
| nhr-54 | 0.89 |
| pha-2 | 0.89 |
| nhr-7 | 0.87 |
| nhr-117 | 0.87 |
| fkh-7 | 0.86 |
| mef-2 | 0.86 |
| peb-1 | 0.85 |
| eyg-1 | 0.85 |
| nhr-179 | 0.85 |
| nhr-203 | 0.84 |
| ceh-60 | 0.84 |
| nhr-243 | 0.84 |
| ceh-2 | 0.84 |
| nhr-62 | 0.84 |
| nhr-238 | 0.84 |
| nhr-212 | 0.84 |
| unc-3 | -0.48 |
| nhr-247 | -0.49 |
| tbx-31 | -0.49 |
| zip-4 | -0.49 |
| nhr-119 | -0.50 |
| cebp-2 | -0.50 |
| madf-5 | -0.50 |
| lin-39 | -0.50 |
| bar-1 | -0.51 |
| tra-1 | -0.51 |
| nhr-85 | -0.51 |
| nhr-157 | -0.52 |
| mxl-2 | -0.52 |
| hmg-12 | -0.52 |
| Y56A3A.18 | -0.54 |
| repo-1 | -0.55 |
| him-8 | -0.55 |
| Y53H1A.2 | -0.56 |
| Y55F3AM.14 | -0.57 |
| ceh-41 | -0.58 |
| C01F6.9 | -0.59 |
| hmg-11 | -0.61 |
| mxl-1 | -0.64 |
| C09F5.3 | -0.67 |
| F21D5.9 | -0.71 |

### ChIP peaks enriched

|  |  |  |  |  |
| --- | --- | --- | --- | --- |
| **Gene** | **Experiment** | **Number of upstream peaks** | **Enrichment** | **FDR corrected p** |
| pha-4 | PHA-4\_Larvae-L2-stage | 102 | 1.84 | 1.1e-09 |
| pha-4 | PHA-4\_Larvae-L4-stage | 61 | 1.88 | 3.0e-05 |
| peb-1 | PEB-1\_Larvae-L2-stage | 12 | 5.29 | 1.9e-04 |
