# Supplementary material for: Overlapping cell population expression profiling and regulatory inference in C. elegans
Source: BMC Genomics. 2016 Feb 29;17:159. doi: 10.1186/s12864-016-2482-z (PMC4772325; doi:10.1186/s12864-016-2482-z)
Supplement: Additional file 13: — Web supplement. (DOC 21 kb) [file 12864_2016_2482_MOESM13_ESM.zip › sortWeb/clusters/hier.300.clusters/122.html]

Cluster 122 

## Cluster 122

### Expression

| cnd-1 rep. 1 | cnd-1 rep. 2 | cnd-1 rep. 3 | pha-4 rep. 1 | pha-4 rep. 2 | pha-4 rep. 3 | ceh-27 | ceh-36 | ceh-6 | F21D5.9 | mir-57 | mls-2 | pal-1 | pros-1 | ttx-3 | unc-130 | hlh-16 | irx-1 | ceh-6 (+) hlh-16 (+) | ceh-6 (+) hlh-16 (-) | ceh-6 (-) hlh-16 (+) | cnd-1 singlets | pha-4 singlets | 0 | 60 | 120 | 150 | 180 | 240 | 330 | 390 | 420 | 480 | 540 | 570 | 600 | 630 | 660 | NAME | Functional description |
| --- | --- | --- | --- | --- | --- | --- | --- | --- | --- | --- | --- | --- | --- | --- | --- | --- | --- | --- | --- | --- | --- | --- | --- | --- | --- | --- | --- | --- | --- | --- | --- | --- | --- | --- | --- | --- | --- | --- | --- |
|  |  |  |  |  |  |  |  |  |  |  |  |  |  |  |  |  |  |  |  |  |  |  |  |  |  |  |  |  |  |  |  |  |  |  |  |  |  | *hpo-16* | Hypersensitive to POre-forming toxin |
|  |  |  |  |  |  |  |  |  |  |  |  |  |  |  |  |  |  |  |  |  |  |  |  |  |  |  |  |  |  |  |  |  |  |  |  |  |  | R05H5.3 |  |
|  |  |  |  |  |  |  |  |  |  |  |  |  |  |  |  |  |  |  |  |  |  |  |  |  |  |  |  |  |  |  |  |  |  |  |  |  |  | *srr-9* | Serpentine Receptor, class R |
|  |  |  |  |  |  |  |  |  |  |  |  |  |  |  |  |  |  |  |  |  |  |  |  |  |  |  |  |  |  |  |  |  |  |  |  |  |  | *ucr-2.1* | Ubiquinol-Cytochrome c oxidoReductase complex |
|  |  |  |  |  |  |  |  |  |  |  |  |  |  |  |  |  |  |  |  |  |  |  |  |  |  |  |  |  |  |  |  |  |  |  |  |  |  | *atp-3* | ATP synthase subunit |
|  |  |  |  |  |  |  |  |  |  |  |  |  |  |  |  |  |  |  |  |  |  |  |  |  |  |  |  |  |  |  |  |  |  |  |  |  |  | B0491.5 |  |
|  |  |  |  |  |  |  |  |  |  |  |  |  |  |  |  |  |  |  |  |  |  |  |  |  |  |  |  |  |  |  |  |  |  |  |  |  |  | F28A10.9 |  |
|  |  |  |  |  |  |  |  |  |  |  |  |  |  |  |  |  |  |  |  |  |  |  |  |  |  |  |  |  |  |  |  |  |  |  |  |  |  | *ucr-2.2* | Ubiquinol-Cytochrome c oxidoReductase complex |
|  |  |  |  |  |  |  |  |  |  |  |  |  |  |  |  |  |  |  |  |  |  |  |  |  |  |  |  |  |  |  |  |  |  |  |  |  |  | *dhs-6* | DeHydrogenases, Short chain |
|  |  |  |  |  |  |  |  |  |  |  |  |  |  |  |  |  |  |  |  |  |  |  |  |  |  |  |  |  |  |  |  |  |  |  |  |  |  | *ech-6* | Enoyl-CoA Hydratase |
|  |  |  |  |  |  |  |  |  |  |  |  |  |  |  |  |  |  |  |  |  |  |  |  |  |  |  |  |  |  |  |  |  |  |  |  |  |  | C54C8.8 |  |
|  |  |  |  |  |  |  |  |  |  |  |  |  |  |  |  |  |  |  |  |  |  |  |  |  |  |  |  |  |  |  |  |  |  |  |  |  |  | *asb-2* | ATP Synthase B homolog |
|  |  |  |  |  |  |  |  |  |  |  |  |  |  |  |  |  |  |  |  |  |  |  |  |  |  |  |  |  |  |  |  |  |  |  |  |  |  | ZK1240.6 |  |
|  |  |  |  |  |  |  |  |  |  |  |  |  |  |  |  |  |  |  |  |  |  |  |  |  |  |  |  |  |  |  |  |  |  |  |  |  |  | Y48G1BL.4 |  |
|  |  |  |  |  |  |  |  |  |  |  |  |  |  |  |  |  |  |  |  |  |  |  |  |  |  |  |  |  |  |  |  |  |  |  |  |  |  | C41H7.2 |  |
|  |  |  |  |  |  |  |  |  |  |  |  |  |  |  |  |  |  |  |  |  |  |  |  |  |  |  |  |  |  |  |  |  |  |  |  |  |  | Y17G7B.19 |  |
|  |  |  |  |  |  |  |  |  |  |  |  |  |  |  |  |  |  |  |  |  |  |  |  |  |  |  |  |  |  |  |  |  |  |  |  |  |  | *igdb-2* | IG (immunoglobulin) and DB (Cys-rich) domains |
|  |  |  |  |  |  |  |  |  |  |  |  |  |  |  |  |  |  |  |  |  |  |  |  |  |  |  |  |  |  |  |  |  |  |  |  |  |  | Y45G12B.2 |  |
|  |  |  |  |  |  |  |  |  |  |  |  |  |  |  |  |  |  |  |  |  |  |  |  |  |  |  |  |  |  |  |  |  |  |  |  |  |  | *fbxc-40* | F-box C protein |
|  |  |  |  |  |  |  |  |  |  |  |  |  |  |  |  |  |  |  |  |  |  |  |  |  |  |  |  |  |  |  |  |  |  |  |  |  |  | F36H5.4 |  |
|  |  |  |  |  |  |  |  |  |  |  |  |  |  |  |  |  |  |  |  |  |  |  |  |  |  |  |  |  |  |  |  |  |  |  |  |  |  | W01G7.4 |  |
|  |  |  |  |  |  |  |  |  |  |  |  |  |  |  |  |  |  |  |  |  |  |  |  |  |  |  |  |  |  |  |  |  |  |  |  |  |  | *prmt-1* | PRotein arginine MethylTransferase |
|  |  |  |  |  |  |  |  |  |  |  |  |  |  |  |  |  |  |  |  |  |  |  |  |  |  |  |  |  |  |  |  |  |  |  |  |  |  | *rsp-3* | SR Protein (splicing factor) |
|  |  |  |  |  |  |  |  |  |  |  |  |  |  |  |  |  |  |  |  |  |  |  |  |  |  |  |  |  |  |  |  |  |  |  |  |  |  | *cht-1* | CHiTinase |
|  |  |  |  |  |  |  |  |  |  |  |  |  |  |  |  |  |  |  |  |  |  |  |  |  |  |  |  |  |  |  |  |  |  |  |  |  |  | C56G2.7 |  |
|  |  |  |  |  |  |  |  |  |  |  |  |  |  |  |  |  |  |  |  |  |  |  |  |  |  |  |  |  |  |  |  |  |  |  |  |  |  | *let-767* | LEThal |
|  |  |  |  |  |  |  |  |  |  |  |  |  |  |  |  |  |  |  |  |  |  |  |  |  |  |  |  |  |  |  |  |  |  |  |  |  |  | *gbh-1* | Gamma Butyrobetaine Hydroxylase |
|  |  |  |  |  |  |  |  |  |  |  |  |  |  |  |  |  |  |  |  |  |  |  |  |  |  |  |  |  |  |  |  |  |  |  |  |  |  | *drr-2* | Dietary Restriction Response (WT but not eat-2 lifespan increased) |
|  |  |  |  |  |  |  |  |  |  |  |  |  |  |  |  |  |  |  |  |  |  |  |  |  |  |  |  |  |  |  |  |  |  |  |  |  |  | Y69A2AR.18 |  |
|  |  |  |  |  |  |  |  |  |  |  |  |  |  |  |  |  |  |  |  |  |  |  |  |  |  |  |  |  |  |  |  |  |  |  |  |  |  | *fbxb-10* | F-box B protein |
|  |  |  |  |  |  |  |  |  |  |  |  |  |  |  |  |  |  |  |  |  |  |  |  |  |  |  |  |  |  |  |  |  |  |  |  |  |  | *elo-1* | fatty acid ELOngation |
|  |  |  |  |  |  |  |  |  |  |  |  |  |  |  |  |  |  |  |  |  |  |  |  |  |  |  |  |  |  |  |  |  |  |  |  |  |  | Y73F8A.27 |  |
|  |  |  |  |  |  |  |  |  |  |  |  |  |  |  |  |  |  |  |  |  |  |  |  |  |  |  |  |  |  |  |  |  |  |  |  |  |  | *rmd-2* | Regulator of Microtubule Dynamics |
|  |  |  |  |  |  |  |  |  |  |  |  |  |  |  |  |  |  |  |  |  |  |  |  |  |  |  |  |  |  |  |  |  |  |  |  |  |  | *acs-2* | fatty Acid CoA Synthetase family |
|  |  |  |  |  |  |  |  |  |  |  |  |  |  |  |  |  |  |  |  |  |  |  |  |  |  |  |  |  |  |  |  |  |  |  |  |  |  | *ubc-20* | UBiquitin Conjugating enzyme |
|  |  |  |  |  |  |  |  |  |  |  |  |  |  |  |  |  |  |  |  |  |  |  |  |  |  |  |  |  |  |  |  |  |  |  |  |  |  | *coq-4* | COenzyme Q (ubiquinone) biosynthesis |
|  |  |  |  |  |  |  |  |  |  |  |  |  |  |  |  |  |  |  |  |  |  |  |  |  |  |  |  |  |  |  |  |  |  |  |  |  |  | *rpn-9* | proteasome Regulatory Particle, Non-ATPase-like |
|  |  |  |  |  |  |  |  |  |  |  |  |  |  |  |  |  |  |  |  |  |  |  |  |  |  |  |  |  |  |  |  |  |  |  |  |  |  | M03C11.6 |  |
|  |  |  |  |  |  |  |  |  |  |  |  |  |  |  |  |  |  |  |  |  |  |  |  |  |  |  |  |  |  |  |  |  |  |  |  |  |  | R119.2 |  |
|  |  |  |  |  |  |  |  |  |  |  |  |  |  |  |  |  |  |  |  |  |  |  |  |  |  |  |  |  |  |  |  |  |  |  |  |  |  | W03C9.2 |  |
|  |  |  |  |  |  |  |  |  |  |  |  |  |  |  |  |  |  |  |  |  |  |  |  |  |  |  |  |  |  |  |  |  |  |  |  |  |  | Y38F2AL.6 |  |
|  |  |  |  |  |  |  |  |  |  |  |  |  |  |  |  |  |  |  |  |  |  |  |  |  |  |  |  |  |  |  |  |  |  |  |  |  |  | F54D10.5 |  |
|  |  |  |  |  |  |  |  |  |  |  |  |  |  |  |  |  |  |  |  |  |  |  |  |  |  |  |  |  |  |  |  |  |  |  |  |  |  | Y48A5A.1 |  |
|  |  |  |  |  |  |  |  |  |  |  |  |  |  |  |  |  |  |  |  |  |  |  |  |  |  |  |  |  |  |  |  |  |  |  |  |  |  | Y41D4A.6 |  |
|  |  |  |  |  |  |  |  |  |  |  |  |  |  |  |  |  |  |  |  |  |  |  |  |  |  |  |  |  |  |  |  |  |  |  |  |  |  | *rad-23* | RADiation sensitivity abnormal/yeast RAD-related |
|  |  |  |  |  |  |  |  |  |  |  |  |  |  |  |  |  |  |  |  |  |  |  |  |  |  |  |  |  |  |  |  |  |  |  |  |  |  | T20B12.7 |  |
|  |  |  |  |  |  |  |  |  |  |  |  |  |  |  |  |  |  |  |  |  |  |  |  |  |  |  |  |  |  |  |  |  |  |  |  |  |  | *dhs-30* | DeHydrogenases, Short chain |
|  |  |  |  |  |  |  |  |  |  |  |  |  |  |  |  |  |  |  |  |  |  |  |  |  |  |  |  |  |  |  |  |  |  |  |  |  |  | *fntb-1* | FarNesylTransferase, Beta subunit |
|  |  |  |  |  |  |  |  |  |  |  |  |  |  |  |  |  |  |  |  |  |  |  |  |  |  |  |  |  |  |  |  |  |  |  |  |  |  | Y39B6A.42 |  |
|  |  |  |  |  |  |  |  |  |  |  |  |  |  |  |  |  |  |  |  |  |  |  |  |  |  |  |  |  |  |  |  |  |  |  |  |  |  | *srab-25* | Serpentine Receptor, class AB (class A-like) |
|  |  |  |  |  |  |  |  |  |  |  |  |  |  |  |  |  |  |  |  |  |  |  |  |  |  |  |  |  |  |  |  |  |  |  |  |  |  | *dnj-3* | DNaJ domain (prokaryotic heat shock protein) |
|  |  |  |  |  |  |  |  |  |  |  |  |  |  |  |  |  |  |  |  |  |  |  |  |  |  |  |  |  |  |  |  |  |  |  |  |  |  | C49A9.10 |  |
|  |  |  |  |  |  |  |  |  |  |  |  |  |  |  |  |  |  |  |  |  |  |  |  |  |  |  |  |  |  |  |  |  |  |  |  |  |  | *skr-9* | SKp1 Related (ubiquitin ligase complex component) |
|  |  |  |  |  |  |  |  |  |  |  |  |  |  |  |  |  |  |  |  |  |  |  |  |  |  |  |  |  |  |  |  |  |  |  |  |  |  | *clec-6* | C-type LECtin |
|  |  |  |  |  |  |  |  |  |  |  |  |  |  |  |  |  |  |  |  |  |  |  |  |  |  |  |  |  |  |  |  |  |  |  |  |  |  | F29B9.1 |  |
|  |  |  |  |  |  |  |  |  |  |  |  |  |  |  |  |  |  |  |  |  |  |  |  |  |  |  |  |  |  |  |  |  |  |  |  |  |  | *anr-27* | Antisense Non-coding RNA |
|  |  |  |  |  |  |  |  |  |  |  |  |  |  |  |  |  |  |  |  |  |  |  |  |  |  |  |  |  |  |  |  |  |  |  |  |  |  | T28A11.16 |  |
|  |  |  |  |  |  |  |  |  |  |  |  |  |  |  |  |  |  |  |  |  |  |  |  |  |  |  |  |  |  |  |  |  |  |  |  |  |  | K01C8.7 |  |
|  |  |  |  |  |  |  |  |  |  |  |  |  |  |  |  |  |  |  |  |  |  |  |  |  |  |  |  |  |  |  |  |  |  |  |  |  |  | C46C2.7 |  |
|  |  |  |  |  |  |  |  |  |  |  |  |  |  |  |  |  |  |  |  |  |  |  |  |  |  |  |  |  |  |  |  |  |  |  |  |  |  | *mrrf-1* | Mitochondrial Ribosome Recycling Factor |
|  |  |  |  |  |  |  |  |  |  |  |  |  |  |  |  |  |  |  |  |  |  |  |  |  |  |  |  |  |  |  |  |  |  |  |  |  |  | *cutc-1* | CUTC copper homeostasis protein homolog |
|  |  |  |  |  |  |  |  |  |  |  |  |  |  |  |  |  |  |  |  |  |  |  |  |  |  |  |  |  |  |  |  |  |  |  |  |  |  | *rad-8* | RADiation sensitivity abnormal/yeast RAD-related |
|  |  |  |  |  |  |  |  |  |  |  |  |  |  |  |  |  |  |  |  |  |  |  |  |  |  |  |  |  |  |  |  |  |  |  |  |  |  | F58H1.8 |  |
|  |  |  |  |  |  |  |  |  |  |  |  |  |  |  |  |  |  |  |  |  |  |  |  |  |  |  |  |  |  |  |  |  |  |  |  |  |  | *mrps-2* | Mitochondrial Ribosomal Protein, Small |
|  |  |  |  |  |  |  |  |  |  |  |  |  |  |  |  |  |  |  |  |  |  |  |  |  |  |  |  |  |  |  |  |  |  |  |  |  |  | *mrpl-4* | Mitochondrial Ribosomal Protein, Large |

### Phenotypes enriched

none found

### Anatomy terms enriched

none found

### GO terms enriched

|  |  |  |
| --- | --- | --- |
| **GO term** | **Number of genes** | **FDR-corrected p-value** |
| fatty acid metabolic process | 4 | 0.0073 |

### Expression clusters enriched

|  |  |  |  |
| --- | --- | --- | --- |
| **Group name** | **Number in cluster** | **Enrichment** | **FDR corrected p** |
| Maternal class (M): genes that are called present in at least one of the three PC6 replicates. | 42 | 1.74 | 0.00201 |
| Caenorhabditis elegans Genes with expression levels changed significantly after treatment of Bacillus thurigiensis DB27. | 37 | 1.86 | 0.00273 |
| Caenorhabditis elegans Genes with expression levels changed significantly after treatment of Xenorhabdus nematophila. | 44 | 1.52 | 0.03040 |
| Maternal degradation class (MD): genes that are the subset of maternal genes that decrease without first increasing in abundance. | 18 | 2.49 | 0.04080 |

### Motifs enriched

|  |  |  |  |  |  |
| --- | --- | --- | --- | --- | --- |
| **Motif** | **Logo** | **Possible orthologs** | **Number of motifs in cluster** | **Enrichment** | **FDR corrected p** |
| pTH8982 |  | ceh-48 | 14 | 3.05 | 0.014 |
| MSX2\_f1 |  | ceh-1 | 31 | 1.75 | 0.028 |
| MA0509.1 |  | daf-19 | 14 | 2.76 | 0.030 |
| ONECUT1\_1 |  | ceh-48 | 19 | 2.23 | 0.034 |
| RFX1\_f1 |  | daf-19 | 13 | 2.78 | 0.042 |

### Correlated (and anti-correlated) transcription factors

|  |  |
| --- | --- |
| **Transcription factor** | **Correlation** |
| sex-1 | 0.87 |
| Y53F4B.3 | 0.87 |
| dhhc-6 | 0.85 |
| ubxn-1 | 0.84 |
| zip-7 | 0.83 |
| Y82E9BR.17 | 0.80 |
| T11G6.8 | 0.79 |
| flh-1 | 0.79 |
| hmg-20 | 0.79 |
| efl-1 | 0.79 |
| zim-1 | 0.78 |
| dpl-1 | 0.78 |
| hmg-5 | 0.78 |
| F23A7.6 | 0.78 |
| efl-3 | 0.78 |
| ekl-4 | 0.77 |
| ces-2 | 0.76 |
| pop-1 | 0.76 |
| ceh-83 | 0.76 |
| Y82E9BR.1 | 0.76 |
| flh-3 | 0.75 |
| duxl-1 | 0.75 |
| nurf-1 | 0.74 |
| attf-2 | 0.74 |
| ztf-13 | 0.73 |
| zip-1 | -0.61 |
| ceh-62 | -0.62 |
| nhr-40 | -0.62 |
| nhr-105 | -0.62 |
| T27A8.2 | -0.63 |
| nhr-255 | -0.64 |
| nhr-41 | -0.64 |
| unc-55 | -0.64 |
| nhr-14 | -0.66 |
| nhr-275 | -0.67 |
| ccch-1 | -0.67 |
| nhr-50 | -0.68 |
| egl-38 | -0.69 |
| C35D6.4 | -0.70 |
| madf-1 | -0.70 |
| gmeb-3 | -0.70 |
| tag-97 | -0.71 |
| ceh-88 | -0.72 |
| zfh-2 | -0.72 |
| mdl-1 | -0.74 |
| nhr-222 | -0.75 |
| nhr-63 | -0.75 |
| saeg-1 | -0.77 |
| lfi-1 | -0.79 |
| ceh-18 | -0.80 |

### ChIP peaks enriched

|  |  |  |  |  |
| --- | --- | --- | --- | --- |
| **Gene** | **Experiment** | **Number of upstream peaks** | **Enrichment** | **FDR corrected p** |
| F23B12.7 | F23B12.7\_Young-adult | 25 | 3.55 | 4.9e-07 |
| efl-1 | EFL-1\_Young-adult | 33 | 2.42 | 7.3e-06 |
| R02D3.7 | R02D3.7\_Larvae-L3-stage | 32 | 2.45 | 9.7e-06 |
| R02D3.7 | R02D3.7\_Larvae-L2-stage | 21 | 3.51 | 1.3e-05 |
| dpl-1 | DPL-1\_Larvae-L4-stage | 36 | 2.17 | 2.0e-05 |
| lsy-2 | LSY-2\_Embryos | 21 | 3.39 | 2.2e-05 |
| aly-2 | ALY-2\_Fed-L1-stage-larvae | 25 | 2.88 | 2.4e-05 |
| lin-15 | LIN-15B\_Fed-L1-stage-larvae | 21 | 3.35 | 2.6e-05 |
| nhr-6 | NHR-6\_Larvae-L4-stage | 21 | 3.18 | 5.7e-05 |
| C16A3.4 | C16A3.4\_Fed-L1-stage-larvae | 24 | 2.78 | 7.9e-05 |
| eor-1 | EOR-1\_Larvae-L3-stage | 30 | 2.29 | 1.2e-04 |
| efl-1 | EFL-1\_Fed-L1-stage-larvae | 27 | 2.47 | 1.2e-04 |
| efl-1 | EFL-1\_Larvae-L1-stage | 29 | 2.31 | 1.6e-04 |
| nhr-237 | NHR-237\_Embryos | 17 | 3.55 | 1.8e-04 |
| lin-35 | LIN-35\_Fed-L1-stage-larvae | 26 | 2.43 | 2.9e-04 |
| lsy-2 | LSY-2\_Larvae-L2-stage | 17 | 3.40 | 3.1e-04 |
| C01B12.2 | C01B12.2\_Larvae-L2-stage | 34 | 2.00 | 3.5e-04 |
| lsy-2 | LSY-2\_Fed-L1-stage-larvae | 27 | 2.31 | 4.2e-04 |
| F16B12.6 | F16B12.6\_Fed-L1-stage-larvae | 17 | 3.30 | 4.5e-04 |
| skn-1 | SKN-1\_Larvae-L3-stage | 16 | 3.41 | 5.7e-04 |
| sax-3 | SAX-3\_Larvae-L4-stage | 30 | 2.11 | 6.1e-04 |
| W03F9.2 | W03F9.2\_L4-Young-Adult-stage-larvae | 37 | 1.83 | 7.1e-04 |
| gei-11 | GEI-11\_Fed-L1-stage-larvae | 26 | 2.23 | 1.2e-03 |
| gei-11 | GEI-11\_Larvae-L2-stage | 22 | 2.47 | 1.4e-03 |
| ztf-7 | ZTF-7\_Larvae-L4-stage | 19 | 2.73 | 1.6e-03 |
| nhr-237 | NHR-237\_Larvae-L1-stage | 12 | 4.04 | 1.6e-03 |
| dpl-1 | DPL-1\_Fed-L1-stage-larvae | 25 | 2.25 | 1.6e-03 |
| egl-5 | EGL-5\_Larvae-L3-stage | 21 | 2.52 | 1.8e-03 |
| nhr-77 | NHR-77\_Larvae-L4-stage | 34 | 1.84 | 1.9e-03 |
| lsy-2 | LSY-2\_Larvae-L1-stage | 30 | 1.98 | 2.1e-03 |
| fos-1 | FOS-1\_Fed-L1-stage-larvae | 25 | 2.21 | 2.1e-03 |
| dpl-1 | DPL-1\_Young-adult | 24 | 2.27 | 2.1e-03 |
| fos-1 | FOS-1\_Larvae-L3-stage | 21 | 2.47 | 2.3e-03 |
| mab-5 | MAB-5\_Larvae-L2-stage | 15 | 3.17 | 2.4e-03 |
| elt-3 | ELT-3\_Embryos | 22 | 2.38 | 2.4e-03 |
| nfya-1 | NFYA-1\_Larvae-L3-stage | 22 | 2.28 | 4.4e-03 |
| F45C12.2 | F45C12.2\_Fed-L1-stage-larvae | 23 | 2.20 | 4.8e-03 |
| hpl-2 | HPL-2\_Fed-L1-stage-larvae | 29 | 1.90 | 6.0e-03 |
| nhr-23 | NHR-23\_Larvae-L3-stage | 20 | 2.34 | 6.8e-03 |
| nhr-25 | NHR-25\_Larvae-L2-stage | 22 | 2.20 | 7.0e-03 |
| ceh-39 | CEH-39\_Embryos | 17 | 2.59 | 7.5e-03 |
| nhr-77 | NHR-77\_Fed-L1-stage-larvae | 24 | 2.06 | 8.5e-03 |
| dve-1 | DVE-1\_Larvae-L4-stage | 19 | 2.37 | 8.7e-03 |
| mab-5 | MAB-5\_Embryos | 8 | 4.87 | 8.9e-03 |
| ham-1 | HAM-1\_Larvae-L4-stage | 26 | 1.96 | 9.0e-03 |
| R02D3.7 | R02D3.7\_Larvae-L4-stage | 15 | 2.72 | 1.1e-02 |
| nhr-76 | NHR-76\_Larvae-L3-stage | 14 | 2.85 | 1.1e-02 |
| pes-1 | PES-1\_Larvae-L4-stage | 25 | 1.97 | 1.2e-02 |
| C34F6.9 | C34F6.9\_Larvae-L2-stage | 25 | 1.96 | 1.2e-02 |
| lin-13 | LIN-13\_Larvae-L4-stage | 16 | 2.55 | 1.3e-02 |
| lin-13 | LIN-13\_Larvae-L2-stage | 19 | 2.29 | 1.3e-02 |
| nhr-6 | NHR-6\_Larvae-L2-stage | 23 | 2.03 | 1.4e-02 |
| dve-1 | DVE-1\_Late-Embryos | 21 | 2.13 | 1.5e-02 |
| lin-13 | LIN-13\_Larvae-L1-stage | 10 | 3.59 | 1.5e-02 |
| ceh-38 | CEH-38\_Larvae-L3-stage | 22 | 2.04 | 1.8e-02 |
| zag-1 | ZAG-1\_Fed-L1-stage-larvae | 14 | 2.63 | 2.3e-02 |
| nfya-1 | NFYA-1\_Young-adult | 10 | 3.37 | 2.4e-02 |
| zag-1 | ZAG-1\_Larvae-L3-stage | 12 | 2.92 | 2.4e-02 |
| pha-4 | PHA-4\_Larvae-L2-stage | 26 | 1.83 | 2.4e-02 |
| pax-1 | PAX-1\_Embryos | 9 | 3.63 | 2.6e-02 |
| zag-1 | ZAG-1\_Larvae-L4-stage | 15 | 2.46 | 2.9e-02 |
| lin-35 | LIN-35\_Young-adult | 19 | 2.12 | 3.0e-02 |
| gei-11 | GEI-11\_Larvae-L3-stage | 23 | 1.91 | 3.2e-02 |
| nhr-77 | NHR-77\_Larvae-L3-stage | 18 | 2.17 | 3.3e-02 |
| hlh-30 | HLH-30\_Larvae-L4-stage | 15 | 2.39 | 3.6e-02 |
| alr-1 | ALR-1\_Larvae-L2-stage | 21 | 1.98 | 3.7e-02 |
| sax-3 | SAX-3\_Larvae-L2-stage | 18 | 2.14 | 3.8e-02 |
| sem-4 | SEM-4\_Larvae-L2-stage | 23 | 1.87 | 4.2e-02 |
