# Supplementary material for: Overlapping cell population expression profiling and regulatory inference in C. elegans
Source: BMC Genomics. 2016 Feb 29;17:159. doi: 10.1186/s12864-016-2482-z (PMC4772325; doi:10.1186/s12864-016-2482-z)
Supplement: Additional file 13: — Web supplement. (DOC 21 kb) [file 12864_2016_2482_MOESM13_ESM.zip › sortWeb/clusters/hier.300.clusters/123.html]

Cluster 123 

## Cluster 123

### Expression

| cnd-1 rep. 1 | cnd-1 rep. 2 | cnd-1 rep. 3 | pha-4 rep. 1 | pha-4 rep. 2 | pha-4 rep. 3 | ceh-27 | ceh-36 | ceh-6 | F21D5.9 | mir-57 | mls-2 | pal-1 | pros-1 | ttx-3 | unc-130 | hlh-16 | irx-1 | ceh-6 (+) hlh-16 (+) | ceh-6 (+) hlh-16 (-) | ceh-6 (-) hlh-16 (+) | cnd-1 singlets | pha-4 singlets | 0 | 60 | 120 | 150 | 180 | 240 | 330 | 390 | 420 | 480 | 540 | 570 | 600 | 630 | 660 | NAME | Functional description |
| --- | --- | --- | --- | --- | --- | --- | --- | --- | --- | --- | --- | --- | --- | --- | --- | --- | --- | --- | --- | --- | --- | --- | --- | --- | --- | --- | --- | --- | --- | --- | --- | --- | --- | --- | --- | --- | --- | --- | --- |
|  |  |  |  |  |  |  |  |  |  |  |  |  |  |  |  |  |  |  |  |  |  |  |  |  |  |  |  |  |  |  |  |  |  |  |  |  |  | *srd-18* | Serpentine Receptor, class D (delta) |
|  |  |  |  |  |  |  |  |  |  |  |  |  |  |  |  |  |  |  |  |  |  |  |  |  |  |  |  |  |  |  |  |  |  |  |  |  |  | *fbxc-28* | F-box C protein |
|  |  |  |  |  |  |  |  |  |  |  |  |  |  |  |  |  |  |  |  |  |  |  |  |  |  |  |  |  |  |  |  |  |  |  |  |  |  | *fbxc-14* | F-box C protein |
|  |  |  |  |  |  |  |  |  |  |  |  |  |  |  |  |  |  |  |  |  |  |  |  |  |  |  |  |  |  |  |  |  |  |  |  |  |  | T16A1.4 |  |
|  |  |  |  |  |  |  |  |  |  |  |  |  |  |  |  |  |  |  |  |  |  |  |  |  |  |  |  |  |  |  |  |  |  |  |  |  |  | C28A5.t2 |  |
|  |  |  |  |  |  |  |  |  |  |  |  |  |  |  |  |  |  |  |  |  |  |  |  |  |  |  |  |  |  |  |  |  |  |  |  |  |  | Y60A9.3 |  |
|  |  |  |  |  |  |  |  |  |  |  |  |  |  |  |  |  |  |  |  |  |  |  |  |  |  |  |  |  |  |  |  |  |  |  |  |  |  | *fbxa-68* | F-box A protein |
|  |  |  |  |  |  |  |  |  |  |  |  |  |  |  |  |  |  |  |  |  |  |  |  |  |  |  |  |  |  |  |  |  |  |  |  |  |  | C05B5.11 |  |
|  |  |  |  |  |  |  |  |  |  |  |  |  |  |  |  |  |  |  |  |  |  |  |  |  |  |  |  |  |  |  |  |  |  |  |  |  |  | H06O01.4 |  |
|  |  |  |  |  |  |  |  |  |  |  |  |  |  |  |  |  |  |  |  |  |  |  |  |  |  |  |  |  |  |  |  |  |  |  |  |  |  | *irld-35* | Insulin/EGF-Receptor L Domain protein |
|  |  |  |  |  |  |  |  |  |  |  |  |  |  |  |  |  |  |  |  |  |  |  |  |  |  |  |  |  |  |  |  |  |  |  |  |  |  | C15F1.5 |  |
|  |  |  |  |  |  |  |  |  |  |  |  |  |  |  |  |  |  |  |  |  |  |  |  |  |  |  |  |  |  |  |  |  |  |  |  |  |  | *sup-39* | SUPpressor |
|  |  |  |  |  |  |  |  |  |  |  |  |  |  |  |  |  |  |  |  |  |  |  |  |  |  |  |  |  |  |  |  |  |  |  |  |  |  | *linc-135* | Long Intervening Non-Coding RNA |
|  |  |  |  |  |  |  |  |  |  |  |  |  |  |  |  |  |  |  |  |  |  |  |  |  |  |  |  |  |  |  |  |  |  |  |  |  |  | *col-38* | COLlagen |
|  |  |  |  |  |  |  |  |  |  |  |  |  |  |  |  |  |  |  |  |  |  |  |  |  |  |  |  |  |  |  |  |  |  |  |  |  |  | C32H11.6 |  |
|  |  |  |  |  |  |  |  |  |  |  |  |  |  |  |  |  |  |  |  |  |  |  |  |  |  |  |  |  |  |  |  |  |  |  |  |  |  | *dct-17* | DAF-16/FOXO Controlled, germline Tumor affecting |
|  |  |  |  |  |  |  |  |  |  |  |  |  |  |  |  |  |  |  |  |  |  |  |  |  |  |  |  |  |  |  |  |  |  |  |  |  |  | F43D2.11 |  |
|  |  |  |  |  |  |  |  |  |  |  |  |  |  |  |  |  |  |  |  |  |  |  |  |  |  |  |  |  |  |  |  |  |  |  |  |  |  | *clec-25* | C-type LECtin |
|  |  |  |  |  |  |  |  |  |  |  |  |  |  |  |  |  |  |  |  |  |  |  |  |  |  |  |  |  |  |  |  |  |  |  |  |  |  | C04C11.21 |  |
|  |  |  |  |  |  |  |  |  |  |  |  |  |  |  |  |  |  |  |  |  |  |  |  |  |  |  |  |  |  |  |  |  |  |  |  |  |  | H19M22.5 |  |
|  |  |  |  |  |  |  |  |  |  |  |  |  |  |  |  |  |  |  |  |  |  |  |  |  |  |  |  |  |  |  |  |  |  |  |  |  |  | Y102A5C.4 |  |
|  |  |  |  |  |  |  |  |  |  |  |  |  |  |  |  |  |  |  |  |  |  |  |  |  |  |  |  |  |  |  |  |  |  |  |  |  |  | B0281.3 |  |
|  |  |  |  |  |  |  |  |  |  |  |  |  |  |  |  |  |  |  |  |  |  |  |  |  |  |  |  |  |  |  |  |  |  |  |  |  |  | Y53F4B.5 |  |
|  |  |  |  |  |  |  |  |  |  |  |  |  |  |  |  |  |  |  |  |  |  |  |  |  |  |  |  |  |  |  |  |  |  |  |  |  |  | H23N18.4 |  |
|  |  |  |  |  |  |  |  |  |  |  |  |  |  |  |  |  |  |  |  |  |  |  |  |  |  |  |  |  |  |  |  |  |  |  |  |  |  | *clec-31* | C-type LECtin |
|  |  |  |  |  |  |  |  |  |  |  |  |  |  |  |  |  |  |  |  |  |  |  |  |  |  |  |  |  |  |  |  |  |  |  |  |  |  | K08D8.1 |  |
|  |  |  |  |  |  |  |  |  |  |  |  |  |  |  |  |  |  |  |  |  |  |  |  |  |  |  |  |  |  |  |  |  |  |  |  |  |  | *str-171* | Seven TM Receptor |

### Phenotypes enriched

none found

### Anatomy terms enriched

none found

### GO terms enriched

none found

### Expression clusters enriched

none found

### Motifs enriched

|  |  |  |  |  |  |
| --- | --- | --- | --- | --- | --- |
| **Motif** | **Logo** | **Possible orthologs** | **Number of motifs in cluster** | **Enrichment** | **FDR corrected p** |
| FOXO3\_1 |  | daf-16 | 15 | 2.81 | 0.0052 |
| pTH6071 |  | C33G8.2 | 17 | 2.33 | 0.0110 |
| pTH10037 |  | T22C8.4 | 22 | 1.80 | 0.0130 |
| Hoxb9\_3413 |  | ceh-24 | 17 | 2.23 | 0.0170 |
| Mw142 |  | egl-27 | 16 | 2.28 | 0.0220 |
| Barx2\_3447 |  | ceh-43 | 27 | 1.35 | 0.0230 |
| pTH9159 |  | atf-6 | 25 | 1.51 | 0.0230 |
| Sox17\_2837 |  | sox-4 | 23 | 1.63 | 0.0290 |
| pTH6569 |  | ceh-43 | 26 | 1.42 | 0.0290 |
| BARX1\_1 |  | ceh-43 | 17 | 2.10 | 0.0300 |
| I$MTTFA\_01 |  | hmg-5 | 20 | 1.83 | 0.0300 |
| pTH10927 |  | tbx-43 | 27 | 1.33 | 0.0310 |
| V$GATA1\_04 |  | elt-1 | 27 | 1.33 | 0.0320 |
| OTX2\_1 |  | ceh-45 | 20 | 1.82 | 0.0330 |
| NFIA\_1 |  | nfi-1 | 25 | 1.48 | 0.0330 |
| pTH9711 |  | atf-2 | 26 | 1.40 | 0.0350 |
| pTH6404 |  | mls-2 | 19 | 1.88 | 0.0370 |
| Plagl1\_0972 |  | Y53H1A.2 | 7 | 4.64 | 0.0380 |
| BSX\_1 |  | ceh-31 | 19 | 1.85 | 0.0430 |
| Gata5\_3768 |  | elt-1 | 11 | 2.87 | 0.0430 |
| V$IK1\_01 |  | F26F4.8 | 10 | 3.12 | 0.0440 |
| ALX1\_si |  | alr-1 | 17 | 1.99 | 0.0490 |

### Correlated (and anti-correlated) transcription factors

|  |  |
| --- | --- |
| **Transcription factor** | **Correlation** |
| Y60A9.3 | 0.75 |
| pzf-1 | 0.66 |
| zip-8 | 0.60 |
| dhhc-13 | 0.57 |
| C28G1.4 | 0.57 |
| ceh-82 | 0.51 |
| nhr-246 | 0.51 |
| pqn-75 | 0.51 |
| ztf-4 | 0.48 |
| Y54G2A.20 | 0.48 |
| ztf-15 | 0.48 |
| sup-35 | 0.47 |
| nhr-220 | 0.46 |
| nhr-276 | 0.46 |
| D2030.7 | 0.46 |
| T27A8.2 | 0.45 |
| R05D3.3 | 0.45 |
| ceh-40 | 0.44 |
| cep-1 | 0.44 |
| hmg-3 | 0.43 |
| nhr-247 | 0.43 |
| nhr-261 | 0.43 |
| egrh-2 | 0.42 |
| K11H3.4 | 0.42 |
| nhr-169 | 0.41 |
| nhr-19 | -0.53 |
| lin-26 | -0.53 |
| dsc-1 | -0.53 |
| ceh-1 | -0.54 |
| nhr-245 | -0.55 |
| nhr-73 | -0.55 |
| ztf-9 | -0.55 |
| egl-5 | -0.55 |
| nhr-270 | -0.55 |
| moe-3 | -0.56 |
| nhr-43 | -0.56 |
| hlh-4 | -0.57 |
| lir-1 | -0.57 |
| nhr-218 | -0.57 |
| nhr-188 | -0.57 |
| K05F1.5 | -0.57 |
| R07E5.5 | -0.60 |
| ets-8 | -0.60 |
| elt-3 | -0.60 |
| B0310.2 | -0.61 |
| nhr-148 | -0.64 |
| npax-2 | -0.64 |
| nhr-147 | -0.64 |
| rnt-1 | -0.67 |
| ztf-30 | -0.73 |

### ChIP peaks enriched

none found
