# Supplementary material for: Overlapping cell population expression profiling and regulatory inference in C. elegans
Source: BMC Genomics. 2016 Feb 29;17:159. doi: 10.1186/s12864-016-2482-z (PMC4772325; doi:10.1186/s12864-016-2482-z)
Supplement: Additional file 13: — Web supplement. (DOC 21 kb) [file 12864_2016_2482_MOESM13_ESM.zip › sortWeb/clusters/hier.300.clusters/140.html]

Cluster 140 

## Cluster 140

### Expression

| cnd-1 rep. 1 | cnd-1 rep. 2 | cnd-1 rep. 3 | pha-4 rep. 1 | pha-4 rep. 2 | pha-4 rep. 3 | ceh-27 | ceh-36 | ceh-6 | F21D5.9 | mir-57 | mls-2 | pal-1 | pros-1 | ttx-3 | unc-130 | hlh-16 | irx-1 | ceh-6 (+) hlh-16 (+) | ceh-6 (+) hlh-16 (-) | ceh-6 (-) hlh-16 (+) | cnd-1 singlets | pha-4 singlets | 0 | 60 | 120 | 150 | 180 | 240 | 330 | 390 | 420 | 480 | 540 | 570 | 600 | 630 | 660 | NAME | Functional description |
| --- | --- | --- | --- | --- | --- | --- | --- | --- | --- | --- | --- | --- | --- | --- | --- | --- | --- | --- | --- | --- | --- | --- | --- | --- | --- | --- | --- | --- | --- | --- | --- | --- | --- | --- | --- | --- | --- | --- | --- |
|  |  |  |  |  |  |  |  |  |  |  |  |  |  |  |  |  |  |  |  |  |  |  |  |  |  |  |  |  |  |  |  |  |  |  |  |  |  | *hmg-11* | HMG |
|  |  |  |  |  |  |  |  |  |  |  |  |  |  |  |  |  |  |  |  |  |  |  |  |  |  |  |  |  |  |  |  |  |  |  |  |  |  | *linc-2* | Long Intervening Non-Coding RNA |
|  |  |  |  |  |  |  |  |  |  |  |  |  |  |  |  |  |  |  |  |  |  |  |  |  |  |  |  |  |  |  |  |  |  |  |  |  |  | *smr-1* | SMN (Survival of Motor Neuron protein) Related |
|  |  |  |  |  |  |  |  |  |  |  |  |  |  |  |  |  |  |  |  |  |  |  |  |  |  |  |  |  |  |  |  |  |  |  |  |  |  | F31E9.6 |  |
|  |  |  |  |  |  |  |  |  |  |  |  |  |  |  |  |  |  |  |  |  |  |  |  |  |  |  |  |  |  |  |  |  |  |  |  |  |  | Y57A10B.2 |  |
|  |  |  |  |  |  |  |  |  |  |  |  |  |  |  |  |  |  |  |  |  |  |  |  |  |  |  |  |  |  |  |  |  |  |  |  |  |  | F14F3.8 |  |
|  |  |  |  |  |  |  |  |  |  |  |  |  |  |  |  |  |  |  |  |  |  |  |  |  |  |  |  |  |  |  |  |  |  |  |  |  |  | F20D1.1 |  |
|  |  |  |  |  |  |  |  |  |  |  |  |  |  |  |  |  |  |  |  |  |  |  |  |  |  |  |  |  |  |  |  |  |  |  |  |  |  | ZK112.3 |  |
|  |  |  |  |  |  |  |  |  |  |  |  |  |  |  |  |  |  |  |  |  |  |  |  |  |  |  |  |  |  |  |  |  |  |  |  |  |  | T06A4.7 |  |
|  |  |  |  |  |  |  |  |  |  |  |  |  |  |  |  |  |  |  |  |  |  |  |  |  |  |  |  |  |  |  |  |  |  |  |  |  |  | M88.11 |  |
|  |  |  |  |  |  |  |  |  |  |  |  |  |  |  |  |  |  |  |  |  |  |  |  |  |  |  |  |  |  |  |  |  |  |  |  |  |  | *ubl-5* | UBiquitin-Like family |
|  |  |  |  |  |  |  |  |  |  |  |  |  |  |  |  |  |  |  |  |  |  |  |  |  |  |  |  |  |  |  |  |  |  |  |  |  |  | C30H6.9 |  |
|  |  |  |  |  |  |  |  |  |  |  |  |  |  |  |  |  |  |  |  |  |  |  |  |  |  |  |  |  |  |  |  |  |  |  |  |  |  | Y50D7A.10 |  |
|  |  |  |  |  |  |  |  |  |  |  |  |  |  |  |  |  |  |  |  |  |  |  |  |  |  |  |  |  |  |  |  |  |  |  |  |  |  | *vhl-1* | Von Hippel-Lindau tumor suppressor homolog |
|  |  |  |  |  |  |  |  |  |  |  |  |  |  |  |  |  |  |  |  |  |  |  |  |  |  |  |  |  |  |  |  |  |  |  |  |  |  | *phip-1* | Protein HIstidine Phosphatase |
|  |  |  |  |  |  |  |  |  |  |  |  |  |  |  |  |  |  |  |  |  |  |  |  |  |  |  |  |  |  |  |  |  |  |  |  |  |  | *mdt-22* | MeDiaTor |
|  |  |  |  |  |  |  |  |  |  |  |  |  |  |  |  |  |  |  |  |  |  |  |  |  |  |  |  |  |  |  |  |  |  |  |  |  |  | *lsm-1* | LSM Sm-like protein |
|  |  |  |  |  |  |  |  |  |  |  |  |  |  |  |  |  |  |  |  |  |  |  |  |  |  |  |  |  |  |  |  |  |  |  |  |  |  | C55A6.1 |  |
|  |  |  |  |  |  |  |  |  |  |  |  |  |  |  |  |  |  |  |  |  |  |  |  |  |  |  |  |  |  |  |  |  |  |  |  |  |  | *sig-7* | Silencing In Germline defective |
|  |  |  |  |  |  |  |  |  |  |  |  |  |  |  |  |  |  |  |  |  |  |  |  |  |  |  |  |  |  |  |  |  |  |  |  |  |  | C33H5.8 |  |
|  |  |  |  |  |  |  |  |  |  |  |  |  |  |  |  |  |  |  |  |  |  |  |  |  |  |  |  |  |  |  |  |  |  |  |  |  |  | *syx-17* | SYntaXin |
|  |  |  |  |  |  |  |  |  |  |  |  |  |  |  |  |  |  |  |  |  |  |  |  |  |  |  |  |  |  |  |  |  |  |  |  |  |  | *phf-30* | PHd Finger family |
|  |  |  |  |  |  |  |  |  |  |  |  |  |  |  |  |  |  |  |  |  |  |  |  |  |  |  |  |  |  |  |  |  |  |  |  |  |  | *fbxa-11* | F-box A protein |
|  |  |  |  |  |  |  |  |  |  |  |  |  |  |  |  |  |  |  |  |  |  |  |  |  |  |  |  |  |  |  |  |  |  |  |  |  |  | *pqn-68* | Prion-like-(Q/N-rich)-domain-bearing protein |
|  |  |  |  |  |  |  |  |  |  |  |  |  |  |  |  |  |  |  |  |  |  |  |  |  |  |  |  |  |  |  |  |  |  |  |  |  |  | K07A1.3 |  |
|  |  |  |  |  |  |  |  |  |  |  |  |  |  |  |  |  |  |  |  |  |  |  |  |  |  |  |  |  |  |  |  |  |  |  |  |  |  | R12C12.7 |  |
|  |  |  |  |  |  |  |  |  |  |  |  |  |  |  |  |  |  |  |  |  |  |  |  |  |  |  |  |  |  |  |  |  |  |  |  |  |  | T25G3.1 |  |
|  |  |  |  |  |  |  |  |  |  |  |  |  |  |  |  |  |  |  |  |  |  |  |  |  |  |  |  |  |  |  |  |  |  |  |  |  |  | Y52B11A.2 |  |
|  |  |  |  |  |  |  |  |  |  |  |  |  |  |  |  |  |  |  |  |  |  |  |  |  |  |  |  |  |  |  |  |  |  |  |  |  |  | Y39B6A.13 |  |
|  |  |  |  |  |  |  |  |  |  |  |  |  |  |  |  |  |  |  |  |  |  |  |  |  |  |  |  |  |  |  |  |  |  |  |  |  |  | *col-86* | COLlagen |
|  |  |  |  |  |  |  |  |  |  |  |  |  |  |  |  |  |  |  |  |  |  |  |  |  |  |  |  |  |  |  |  |  |  |  |  |  |  | T28F2.2 |  |
|  |  |  |  |  |  |  |  |  |  |  |  |  |  |  |  |  |  |  |  |  |  |  |  |  |  |  |  |  |  |  |  |  |  |  |  |  |  | Y94H6A.3 |  |
|  |  |  |  |  |  |  |  |  |  |  |  |  |  |  |  |  |  |  |  |  |  |  |  |  |  |  |  |  |  |  |  |  |  |  |  |  |  | F54E4.16 |  |
|  |  |  |  |  |  |  |  |  |  |  |  |  |  |  |  |  |  |  |  |  |  |  |  |  |  |  |  |  |  |  |  |  |  |  |  |  |  | *cebp-2* | C/EBP (CCAAT/enhancer-binding protein) homolog |
|  |  |  |  |  |  |  |  |  |  |  |  |  |  |  |  |  |  |  |  |  |  |  |  |  |  |  |  |  |  |  |  |  |  |  |  |  |  | R11G1.7 |  |
|  |  |  |  |  |  |  |  |  |  |  |  |  |  |  |  |  |  |  |  |  |  |  |  |  |  |  |  |  |  |  |  |  |  |  |  |  |  | F25H5.10 |  |
|  |  |  |  |  |  |  |  |  |  |  |  |  |  |  |  |  |  |  |  |  |  |  |  |  |  |  |  |  |  |  |  |  |  |  |  |  |  | C24H11.5 |  |
|  |  |  |  |  |  |  |  |  |  |  |  |  |  |  |  |  |  |  |  |  |  |  |  |  |  |  |  |  |  |  |  |  |  |  |  |  |  | Y17G9B.2 |  |
|  |  |  |  |  |  |  |  |  |  |  |  |  |  |  |  |  |  |  |  |  |  |  |  |  |  |  |  |  |  |  |  |  |  |  |  |  |  | F18H3.1 |  |
|  |  |  |  |  |  |  |  |  |  |  |  |  |  |  |  |  |  |  |  |  |  |  |  |  |  |  |  |  |  |  |  |  |  |  |  |  |  | *linc-4* | Long Intervening Non-Coding RNA |
|  |  |  |  |  |  |  |  |  |  |  |  |  |  |  |  |  |  |  |  |  |  |  |  |  |  |  |  |  |  |  |  |  |  |  |  |  |  | F49C12.11 |  |
|  |  |  |  |  |  |  |  |  |  |  |  |  |  |  |  |  |  |  |  |  |  |  |  |  |  |  |  |  |  |  |  |  |  |  |  |  |  | F36D1.9 |  |
|  |  |  |  |  |  |  |  |  |  |  |  |  |  |  |  |  |  |  |  |  |  |  |  |  |  |  |  |  |  |  |  |  |  |  |  |  |  | K07H8.3 |  |
|  |  |  |  |  |  |  |  |  |  |  |  |  |  |  |  |  |  |  |  |  |  |  |  |  |  |  |  |  |  |  |  |  |  |  |  |  |  | C28H8.1 |  |
|  |  |  |  |  |  |  |  |  |  |  |  |  |  |  |  |  |  |  |  |  |  |  |  |  |  |  |  |  |  |  |  |  |  |  |  |  |  | *nfyc-1* | Nuclear transcription Factor Y, C (gamma) subunit |
|  |  |  |  |  |  |  |  |  |  |  |  |  |  |  |  |  |  |  |  |  |  |  |  |  |  |  |  |  |  |  |  |  |  |  |  |  |  | *pen-2* | Presenilin ENhancer (enhancer of sel-12(null)) |
|  |  |  |  |  |  |  |  |  |  |  |  |  |  |  |  |  |  |  |  |  |  |  |  |  |  |  |  |  |  |  |  |  |  |  |  |  |  | C50F4.4 |  |
|  |  |  |  |  |  |  |  |  |  |  |  |  |  |  |  |  |  |  |  |  |  |  |  |  |  |  |  |  |  |  |  |  |  |  |  |  |  | *erg-28* | ERG (yeast ergosterol biosynthesis protein) homolog |
|  |  |  |  |  |  |  |  |  |  |  |  |  |  |  |  |  |  |  |  |  |  |  |  |  |  |  |  |  |  |  |  |  |  |  |  |  |  | R144.3 |  |
|  |  |  |  |  |  |  |  |  |  |  |  |  |  |  |  |  |  |  |  |  |  |  |  |  |  |  |  |  |  |  |  |  |  |  |  |  |  | F45G2.7 |  |
|  |  |  |  |  |  |  |  |  |  |  |  |  |  |  |  |  |  |  |  |  |  |  |  |  |  |  |  |  |  |  |  |  |  |  |  |  |  | D2030.7 |  |
|  |  |  |  |  |  |  |  |  |  |  |  |  |  |  |  |  |  |  |  |  |  |  |  |  |  |  |  |  |  |  |  |  |  |  |  |  |  | D2030.11 |  |
|  |  |  |  |  |  |  |  |  |  |  |  |  |  |  |  |  |  |  |  |  |  |  |  |  |  |  |  |  |  |  |  |  |  |  |  |  |  | C50D2.5 |  |
|  |  |  |  |  |  |  |  |  |  |  |  |  |  |  |  |  |  |  |  |  |  |  |  |  |  |  |  |  |  |  |  |  |  |  |  |  |  | *rpb-12* | RNA Polymerase II (B) subunit |
|  |  |  |  |  |  |  |  |  |  |  |  |  |  |  |  |  |  |  |  |  |  |  |  |  |  |  |  |  |  |  |  |  |  |  |  |  |  | *vps-60* | related to yeast Vacuolar Protein Sorting factor |
|  |  |  |  |  |  |  |  |  |  |  |  |  |  |  |  |  |  |  |  |  |  |  |  |  |  |  |  |  |  |  |  |  |  |  |  |  |  | Y62F5A.12 |  |
|  |  |  |  |  |  |  |  |  |  |  |  |  |  |  |  |  |  |  |  |  |  |  |  |  |  |  |  |  |  |  |  |  |  |  |  |  |  | *mdt-19* | MeDiaTor |
|  |  |  |  |  |  |  |  |  |  |  |  |  |  |  |  |  |  |  |  |  |  |  |  |  |  |  |  |  |  |  |  |  |  |  |  |  |  | *mdt-31* | MeDiaTor |
|  |  |  |  |  |  |  |  |  |  |  |  |  |  |  |  |  |  |  |  |  |  |  |  |  |  |  |  |  |  |  |  |  |  |  |  |  |  | Y37E11B.6 |  |
|  |  |  |  |  |  |  |  |  |  |  |  |  |  |  |  |  |  |  |  |  |  |  |  |  |  |  |  |  |  |  |  |  |  |  |  |  |  | *cyn-11* | CYclophyliN |
|  |  |  |  |  |  |  |  |  |  |  |  |  |  |  |  |  |  |  |  |  |  |  |  |  |  |  |  |  |  |  |  |  |  |  |  |  |  | *lsm-3* | LSM Sm-like protein |
|  |  |  |  |  |  |  |  |  |  |  |  |  |  |  |  |  |  |  |  |  |  |  |  |  |  |  |  |  |  |  |  |  |  |  |  |  |  | C08F8.2 |  |
|  |  |  |  |  |  |  |  |  |  |  |  |  |  |  |  |  |  |  |  |  |  |  |  |  |  |  |  |  |  |  |  |  |  |  |  |  |  | *pfd-1* | PreFolDin (molecular chaperone) |
|  |  |  |  |  |  |  |  |  |  |  |  |  |  |  |  |  |  |  |  |  |  |  |  |  |  |  |  |  |  |  |  |  |  |  |  |  |  | *dnc-6* | DyNactin Complex component |
|  |  |  |  |  |  |  |  |  |  |  |  |  |  |  |  |  |  |  |  |  |  |  |  |  |  |  |  |  |  |  |  |  |  |  |  |  |  | *lin-7* | abnormal cell LINeage |
|  |  |  |  |  |  |  |  |  |  |  |  |  |  |  |  |  |  |  |  |  |  |  |  |  |  |  |  |  |  |  |  |  |  |  |  |  |  | Y54G11A.17 |  |
|  |  |  |  |  |  |  |  |  |  |  |  |  |  |  |  |  |  |  |  |  |  |  |  |  |  |  |  |  |  |  |  |  |  |  |  |  |  | T10F2.5 |  |
|  |  |  |  |  |  |  |  |  |  |  |  |  |  |  |  |  |  |  |  |  |  |  |  |  |  |  |  |  |  |  |  |  |  |  |  |  |  | Y54G11A.11 |  |
|  |  |  |  |  |  |  |  |  |  |  |  |  |  |  |  |  |  |  |  |  |  |  |  |  |  |  |  |  |  |  |  |  |  |  |  |  |  | *snr-4* | Small Nuclear Ribonucleoprotein |
|  |  |  |  |  |  |  |  |  |  |  |  |  |  |  |  |  |  |  |  |  |  |  |  |  |  |  |  |  |  |  |  |  |  |  |  |  |  | *rpn-12* | proteasome Regulatory Particle, Non-ATPase-like |
|  |  |  |  |  |  |  |  |  |  |  |  |  |  |  |  |  |  |  |  |  |  |  |  |  |  |  |  |  |  |  |  |  |  |  |  |  |  | C24D10.4 |  |
|  |  |  |  |  |  |  |  |  |  |  |  |  |  |  |  |  |  |  |  |  |  |  |  |  |  |  |  |  |  |  |  |  |  |  |  |  |  | *tomm-22* | Translocase of Outer Mitochondrial Membrane |
|  |  |  |  |  |  |  |  |  |  |  |  |  |  |  |  |  |  |  |  |  |  |  |  |  |  |  |  |  |  |  |  |  |  |  |  |  |  | *fis-2* | S. cerevisiae FIS1-related |
|  |  |  |  |  |  |  |  |  |  |  |  |  |  |  |  |  |  |  |  |  |  |  |  |  |  |  |  |  |  |  |  |  |  |  |  |  |  | B0205.12 |  |
|  |  |  |  |  |  |  |  |  |  |  |  |  |  |  |  |  |  |  |  |  |  |  |  |  |  |  |  |  |  |  |  |  |  |  |  |  |  | *ubc-18* | UBiquitin Conjugating enzyme |
|  |  |  |  |  |  |  |  |  |  |  |  |  |  |  |  |  |  |  |  |  |  |  |  |  |  |  |  |  |  |  |  |  |  |  |  |  |  | C01A2.4 |  |
|  |  |  |  |  |  |  |  |  |  |  |  |  |  |  |  |  |  |  |  |  |  |  |  |  |  |  |  |  |  |  |  |  |  |  |  |  |  | *rpb-9* | RNA Polymerase II (B) subunit |

### Phenotypes enriched

none found

### Anatomy terms enriched

none found

### GO terms enriched

|  |  |  |
| --- | --- | --- |
| **GO term** | **Number of genes** | **FDR-corrected p-value** |
| RNA polymerase II transcription cofactor activity | 3 | 0.0059 |
| mediator complex | 3 | 0.0077 |
| macromolecular complex | 12 | 0.0099 |
| transcription factor binding transcription factor activity | 3 | 0.0300 |

### Expression clusters enriched

|  |  |  |  |
| --- | --- | --- | --- |
| **Group name** | **Number in cluster** | **Enrichment** | **FDR corrected p** |
| Caenorhabditis elegans Genes with expression levels changed significantly after treatment of Bacillus thurigiensis DB27. | 50 | 2.13 | 3.03e-07 |
| Caenorhabditis elegans Genes with expression levels changed significantly after treatment of Xenorhabdus nematophila. | 58 | 1.69 | 1.68e-05 |
| TGF- Dauer pathway adult transcriptional targets. Results obtained by comparing the microarray results of the dauer-constitutive mutants daf-7(e1372), daf-7(m62), and daf-1(m40) with dauer-defective mutants daf-3(mgDf90), daf-5(e1386), and daf-7(e1372);daf-3(mgDf90) double mutants at the permissive temperature, 20C, on the first day of adulthood. WBPaper00031040:TGF-beta\_adult\_downregulated | 43 | 2.08 | 3.04e-05 |
| Expression Pattern Group F, enriched for genes involved in embryonic development. These patterns have in common that they all have genes of which the expression goes up after the juvenile stage. The expression of the genes in these patterns remains high or even goes up after reproduction. | 30 | 2.71 | 4.29e-05 |
| Maternal-embryonic class (ME): genes that are in the intersection of the maternal and embryonic classes. | 29 | 2.23 | 3.06e-03 |
| Maternal degradation class (MD): genes that are the subset of maternal genes that decrease without first increasing in abundance. | 22 | 2.57 | 6.20e-03 |
| Genes enriched in intestine. | 23 | 2.34 | 1.51e-02 |
| Maternal degradation-embryonic class (MDE): genes that are the subset of maternal degradation genes that significantly increase in at least two of the eight total paired timepoint tests in the induction-following-degradation time domain. | 12 | 3.82 | 1.55e-02 |
| Genes expressed in embryonic motor neurons (identified by unc-4::GFP expressing cells). | 46 | 1.55 | 3.10e-02 |
| Genes that showed expression levels higher than the corresponding reference sample (embryonic 0hr reference). WBPaper00037950:BAG-neuron\_expressed | 38 | 1.68 | 3.69e-02 |

### Motifs enriched

|  |  |  |  |  |  |
| --- | --- | --- | --- | --- | --- |
| **Motif** | **Logo** | **Possible orthologs** | **Number of motifs in cluster** | **Enrichment** | **FDR corrected p** |
| pTH8997 |  | hmg-12 (0.65) let-381 Y116A8C.22 | 45 | 1.79 | 0.00059 |
| pTH1014 |  | atf-5 (-0.53) | 29 | 2.38 | 0.00059 |
| pTH8982 |  | ceh-48 | 22 | 2.80 | 0.00110 |
| V$CMYB\_01 |  | D1081.8 | 31 | 2.19 | 0.00120 |
| pTH5916 |  | efl-2 | 21 | 2.85 | 0.00140 |
| Zfp161\_2858 |  | pzf-1 | 28 | 2.23 | 0.00250 |
| pTH10696 |  | Y44A6D.3 | 28 | 2.19 | 0.00320 |
| pTH5257 |  | C48E7.11 | 20 | 2.73 | 0.00340 |
| pTH8863 |  | hmg-12 (0.65) | 43 | 1.70 | 0.00370 |
| pTH5166 |  | atf-2 C48E7.11 F23F12.9 | 12 | 4.12 | 0.00420 |
| V$TBP\_01 |  | tbp-1 | 52 | 1.52 | 0.00480 |
| pTH8566 |  | lin-54 | 56 | 1.46 | 0.00510 |
| MA0146.2 |  | F58G1.2 | 26 | 2.22 | 0.00530 |
| pTH9173 |  | efl-2 | 22 | 2.46 | 0.00540 |
| pTH5250 |  | C48E7.11 | 26 | 2.16 | 0.00730 |
| pTH9125 |  | egl-13 | 58 | 1.41 | 0.00790 |
| pTH9180 |  | mel-28 Y61A9LA.9 Y116A8C.22 | 54 | 1.46 | 0.00810 |
| pTH7876 |  | fkh-7 lin-29 mel-28 | 59 | 1.39 | 0.00870 |
| MA0161.1 |  | nfi-1 | 33 | 1.86 | 0.00930 |
| pTH9082 |  | mab-23 | 56 | 1.42 | 0.01100 |
| pTH9279 |  | Y116A8C.22 | 59 | 1.38 | 0.01100 |
| pTH8985 |  | athp-1 | 45 | 1.58 | 0.01100 |
| CEBPE\_f1 |  | C48E7.11 | 25 | 2.11 | 0.01300 |
| pTH9260 |  | mel-28 | 59 | 1.37 | 0.01400 |
| MA0541.1 |  | efl-1 | 32 | 1.84 | 0.01500 |
| pTH9096 |  | T07C12.11 | 23 | 2.20 | 0.01500 |
| pTH7875 |  | mel-28 | 44 | 1.57 | 0.01500 |
| PAX9\_1 |  | pax-2 | 31 | 1.84 | 0.01800 |
| NR2E3\_f1 |  | nhr-100 | 57 | 1.37 | 0.02100 |
| Bbx\_3753 |  | gei-3 | 33 | 1.76 | 0.02200 |
| pTH9958 |  | ztf-6 | 46 | 1.51 | 0.02200 |
| pTH10769 |  | Y48G1C.6 | 39 | 1.63 | 0.02300 |
| pTH8216 |  | Y116A8C.22 | 29 | 1.86 | 0.02500 |
| pTH6143 |  | pal-1 | 52 | 1.40 | 0.03300 |
| V$POU3F2\_01 |  | ceh-18 | 37 | 1.63 | 0.03400 |
| POU3F3\_2 |  | ceh-18 | 20 | 2.20 | 0.03500 |
| FOXC2\_2 |  | let-381 lin-31 | 56 | 1.35 | 0.03600 |
| NK7.1\_Cell\_FBgn0024321 |  | ceh-19 ceh-31 | 12 | 3.04 | 0.03600 |
| GM12878\_ETS1\_HudsonAlpha |  | lin-1 | 33 | 1.69 | 0.04100 |
| Mafk\_3106 |  | F45H11.6 | 13 | 2.78 | 0.04500 |
| pTH10042 |  | nhr-5 (-0.67) | 38 | 1.58 | 0.04500 |

### Correlated (and anti-correlated) transcription factors

|  |  |
| --- | --- |
| **Transcription factor** | **Correlation** |
| repo-1 | 0.93 |
| nfyc-1 | 0.86 |
| D2030.7 | 0.84 |
| C01F6.9 | 0.84 |
| cebp-2 | 0.83 |
| mxl-1 | 0.80 |
| ztf-4 | 0.80 |
| R144.3 | 0.78 |
| F37B4.10 | 0.76 |
| hmg-11 | 0.74 |
| hlh-16 | 0.68 |
| madf-10 | 0.68 |
| K11D12.12 | 0.67 |
| hmg-12 | 0.65 |
| zip-4 | 0.65 |
| ceh-41 | 0.64 |
| sptf-1 | 0.64 |
| him-8 | 0.63 |
| hmg-6 | 0.63 |
| F52B5.7 | 0.62 |
| dhhc-10 | 0.62 |
| lir-3 | 0.62 |
| dhhc-1 | 0.61 |
| hmg-5 | 0.61 |
| W02D7.6 | 0.58 |
| ets-4 | -0.62 |
| nhr-58 | -0.62 |
| nhr-66 | -0.63 |
| hlh-30 | -0.64 |
| nhr-125 | -0.64 |
| peb-1 | -0.65 |
| nhr-18 | -0.65 |
| nhr-103 | -0.65 |
| nhr-97 | -0.66 |
| nhr-3 | -0.67 |
| nhr-34 | -0.67 |
| nhr-49 | -0.67 |
| nhr-5 | -0.67 |
| let-607 | -0.67 |
| nhr-184 | -0.67 |
| ZC328.2 | -0.67 |
| nhr-128 | -0.68 |
| nhr-202 | -0.69 |
| daf-12 | -0.70 |
| mef-2 | -0.70 |
| C34B4.2 | -0.72 |
| nhr-70 | -0.74 |
| fkh-9 | -0.74 |
| ztf-27 | -0.75 |
| fos-1 | -0.79 |

### ChIP peaks enriched

|  |  |  |  |  |
| --- | --- | --- | --- | --- |
| **Gene** | **Experiment** | **Number of upstream peaks** | **Enrichment** | **FDR corrected p** |
| efl-1 | EFL-1\_Fed-L1-stage-larvae | 43 | 3.32 | 1.5e-12 |
| lsy-2 | LSY-2\_Embryos | 37 | 3.67 | 1.7e-11 |
| F45C12.2 | F45C12.2\_Fed-L1-stage-larvae | 40 | 3.23 | 5.5e-11 |
| lsy-2 | LSY-2\_Fed-L1-stage-larvae | 42 | 3.04 | 7.6e-11 |
| lin-35 | LIN-35\_Fed-L1-stage-larvae | 40 | 3.15 | 1.3e-10 |
| pes-1 | PES-1\_Larvae-L4-stage | 43 | 2.86 | 2.3e-10 |
| efl-1 | EFL-1\_Larvae-L1-stage | 42 | 2.83 | 7.8e-10 |
| C34F6.9 | C34F6.9\_Larvae-L2-stage | 42 | 2.78 | 1.3e-09 |
| dpl-1 | DPL-1\_Fed-L1-stage-larvae | 39 | 2.96 | 1.8e-09 |
| aly-2 | ALY-2\_Fed-L1-stage-larvae | 34 | 3.30 | 4.2e-09 |
| gei-11 | GEI-11\_Larvae-L3-stage | 40 | 2.81 | 4.4e-09 |
| lin-15 | LIN-15B\_Fed-L1-stage-larvae | 29 | 3.90 | 4.5e-09 |
| ces-1 | CES-1\_Embryos | 40 | 2.68 | 1.7e-08 |
| lsy-2 | LSY-2\_Larvae-L1-stage | 44 | 2.45 | 2.0e-08 |
| gei-11 | GEI-11\_Fed-L1-stage-larvae | 38 | 2.75 | 3.4e-08 |
| F16B12.6 | F16B12.6\_Fed-L1-stage-larvae | 25 | 4.10 | 5.1e-08 |
| nfya-1 | NFYA-1\_Late-Embryos | 36 | 2.74 | 1.3e-07 |
| eor-1 | EOR-1\_Larvae-L3-stage | 39 | 2.51 | 2.1e-07 |
| lsy-2 | LSY-2\_Larvae-L2-stage | 21 | 4.59 | 2.3e-07 |
| fos-1 | FOS-1\_Fed-L1-stage-larvae | 36 | 2.69 | 2.3e-07 |
| W03F9.2 | W03F9.2\_L4-Young-Adult-stage-larvae | 49 | 2.05 | 3.4e-07 |
| sem-4 | SEM-4\_Larvae-L2-stage | 37 | 2.54 | 5.5e-07 |
| ham-1 | HAM-1\_Fed-L1-stage-larvae | 37 | 2.53 | 6.0e-07 |
| efl-1 | EFL-1\_Young-adult | 39 | 2.41 | 6.6e-07 |
| C16A3.4 | C16A3.4\_Fed-L1-stage-larvae | 30 | 2.94 | 1.2e-06 |
| ceh-39 | CEH-39\_Embryos | 26 | 3.34 | 1.3e-06 |
| nhr-23 | NHR-23\_Larvae-L3-stage | 34 | 2.63 | 1.3e-06 |
| R02D3.7 | R02D3.7\_Larvae-L3-stage | 32 | 2.73 | 1.9e-06 |
| gei-11 | GEI-11\_Young-adult | 20 | 4.21 | 2.2e-06 |
| gei-11 | GEI-11\_Larvae-L2-stage | 30 | 2.84 | 2.5e-06 |
| alr-1 | ALR-1\_Larvae-L2-stage | 33 | 2.62 | 2.5e-06 |
| ztf-7 | ZTF-7\_Larvae-L4-stage | 26 | 3.15 | 3.9e-06 |
| dpl-1 | DPL-1\_Larvae-L4-stage | 42 | 2.13 | 4.2e-06 |
| hpl-2 | HPL-2\_Fed-L1-stage-larvae | 40 | 2.21 | 4.4e-06 |
| nhr-77 | NHR-77\_Fed-L1-stage-larvae | 34 | 2.47 | 6.3e-06 |
| dpl-1 | DPL-1\_Young-adult | 32 | 2.55 | 8.3e-06 |
| lin-13 | LIN-13\_Larvae-L2-stage | 28 | 2.84 | 8.3e-06 |
| ham-1 | HAM-1\_Larvae-L4-stage | 36 | 2.29 | 1.2e-05 |
| zag-1 | ZAG-1\_Fed-L1-stage-larvae | 19 | 3.89 | 1.6e-05 |
| nhr-237 | NHR-237\_Larvae-L1-stage | 16 | 4.55 | 2.1e-05 |
| nhr-77 | NHR-77\_Larvae-L4-stage | 43 | 1.96 | 2.7e-05 |
| nhr-25 | NHR-25\_Larvae-L2-stage | 30 | 2.54 | 2.8e-05 |
| lin-15 | LIN-15B\_Larvae-L4-stage | 13 | 5.55 | 3.4e-05 |
| F23B12.7 | F23B12.7\_Young-adult | 24 | 2.87 | 6.8e-05 |
| nhr-6 | NHR-6\_Larvae-L4-stage | 23 | 2.94 | 8.2e-05 |
| lsy-2 | LSY-2\_Larvae-L4-stage | 17 | 3.73 | 1.2e-04 |
| aha-1 | AHA-1\_Fed-L1-stage-larvae | 10 | 6.66 | 1.4e-04 |
| nfya-1 | NFYA-1\_Larvae-L3-stage | 28 | 2.45 | 1.5e-04 |
| R02D3.7 | R02D3.7\_Larvae-L2-stage | 17 | 3.56 | 2.1e-04 |
| C01B12.2 | C01B12.2\_Larvae-L2-stage | 39 | 1.93 | 2.2e-04 |
| zag-1 | ZAG-1\_Larvae-L4-stage | 17 | 3.51 | 2.5e-04 |
| nhr-77 | NHR-77\_Larvae-L3-stage | 21 | 2.93 | 2.6e-04 |
| egl-5 | EGL-5\_Larvae-L3-stage | 25 | 2.53 | 3.5e-04 |
| skn-1 | SKN-1\_Larvae-L3-stage | 18 | 3.24 | 3.7e-04 |
| jun-1 | JUN-1\_Larvae-L4-stage | 23 | 2.63 | 4.6e-04 |
| pha-4 | PHA-4\_Young-adult | 18 | 3.18 | 4.7e-04 |
| nhr-237 | NHR-237\_Embryos | 18 | 3.17 | 4.8e-04 |
| nhr-129 | NHR-129\_Larvae-L2-stage | 38 | 1.87 | 7.2e-04 |
| ces-1 | CES-1\_Fed-L1-stage-larvae | 15 | 3.55 | 8.1e-04 |
| nhr-2 | NHR-2\_Embryos | 16 | 3.26 | 1.1e-03 |
| zag-1 | ZAG-1\_Larvae-L2-stage | 20 | 2.74 | 1.1e-03 |
| nfya-1 | NFYA-1\_Young-adult | 12 | 4.19 | 1.2e-03 |
| skn-1 | SKN-1\_Larvae-L4-stage | 6 | 9.91 | 1.5e-03 |
| ceh-38 | CEH-38\_Larvae-L3-stage | 23 | 2.38 | 2.1e-03 |
| nhr-21 | NHR-21\_Larvae-L2-stage | 12 | 3.86 | 2.5e-03 |
| R02D3.7 | R02D3.7\_Larvae-L4-stage | 17 | 2.88 | 2.7e-03 |
| med-1 | MED-1\_Embryos | 10 | 4.54 | 3.0e-03 |
| pha-4 | PHA-4\_Larvae-L2-stage | 32 | 1.91 | 3.3e-03 |
| elt-3 | ELT-3\_Embryos | 18 | 2.70 | 3.5e-03 |
| nhr-77 | NHR-77\_Larvae-L2-stage | 18 | 2.68 | 3.8e-03 |
| sea-2 | SEA-2\_Larvae-L3-stage | 12 | 3.68 | 3.9e-03 |
| unc-62 | UNC-62\_Larvae-L3-stage | 19 | 2.56 | 4.1e-03 |
| dve-1 | DVE-1\_Late-Embryos | 21 | 2.40 | 4.1e-03 |
| hlh-30 | HLH-30\_Larvae-L4-stage | 19 | 2.56 | 4.2e-03 |
| pax-1 | PAX-1\_Embryos | 12 | 3.61 | 4.6e-03 |
| mab-5 | MAB-5\_Larvae-L2-stage | 16 | 2.85 | 4.8e-03 |
| lin-35 | LIN-35\_Starved-L1-stage-larvae | 13 | 3.34 | 5.0e-03 |
| unc-62 | UNC-62\_Fed-L1-stage-larvae | 11 | 3.82 | 5.5e-03 |
| nhr-6 | NHR-6\_Larvae-L2-stage | 27 | 2.02 | 6.0e-03 |
| hlh-30 | HLH-30\_Late-Embryos | 16 | 2.76 | 6.7e-03 |
| ceh-26 | CEH-26\_Late-Embryonic-stage | 18 | 2.53 | 7.1e-03 |
| lin-13 | LIN-13\_Larvae-L4-stage | 20 | 2.34 | 8.3e-03 |
| sax-3 | SAX-3\_Larvae-L2-stage | 22 | 2.21 | 8.5e-03 |
| jun-1 | JUN-1\_Larvae-L1-stage | 23 | 2.12 | 1.0e-02 |
| zag-1 | ZAG-1\_Larvae-L3-stage | 12 | 3.23 | 1.2e-02 |
| nhr-28 | NHR-28\_Larvae-L3-stage | 9 | 4.10 | 1.3e-02 |
| ces-1 | CES-1\_Larvae-L3-stage | 12 | 2.92 | 2.6e-02 |
