# Supplementary material for: Overlapping cell population expression profiling and regulatory inference in C. elegans
Source: BMC Genomics. 2016 Feb 29;17:159. doi: 10.1186/s12864-016-2482-z (PMC4772325; doi:10.1186/s12864-016-2482-z)
Supplement: Additional file 13: — Web supplement. (DOC 21 kb) [file 12864_2016_2482_MOESM13_ESM.zip › sortWeb/clusters/hier.300.clusters/141.html]

Cluster 141 

## Cluster 141

### Expression

| cnd-1 rep. 1 | cnd-1 rep. 2 | cnd-1 rep. 3 | pha-4 rep. 1 | pha-4 rep. 2 | pha-4 rep. 3 | ceh-27 | ceh-36 | ceh-6 | F21D5.9 | mir-57 | mls-2 | pal-1 | pros-1 | ttx-3 | unc-130 | hlh-16 | irx-1 | ceh-6 (+) hlh-16 (+) | ceh-6 (+) hlh-16 (-) | ceh-6 (-) hlh-16 (+) | cnd-1 singlets | pha-4 singlets | 0 | 60 | 120 | 150 | 180 | 240 | 330 | 390 | 420 | 480 | 540 | 570 | 600 | 630 | 660 | NAME | Functional description |
| --- | --- | --- | --- | --- | --- | --- | --- | --- | --- | --- | --- | --- | --- | --- | --- | --- | --- | --- | --- | --- | --- | --- | --- | --- | --- | --- | --- | --- | --- | --- | --- | --- | --- | --- | --- | --- | --- | --- | --- |
|  |  |  |  |  |  |  |  |  |  |  |  |  |  |  |  |  |  |  |  |  |  |  |  |  |  |  |  |  |  |  |  |  |  |  |  |  |  | F53B2.7 |  |
|  |  |  |  |  |  |  |  |  |  |  |  |  |  |  |  |  |  |  |  |  |  |  |  |  |  |  |  |  |  |  |  |  |  |  |  |  |  | *dhhc-9* | DHHC-types zinc finger protein |
|  |  |  |  |  |  |  |  |  |  |  |  |  |  |  |  |  |  |  |  |  |  |  |  |  |  |  |  |  |  |  |  |  |  |  |  |  |  | C34E10.9 |  |
|  |  |  |  |  |  |  |  |  |  |  |  |  |  |  |  |  |  |  |  |  |  |  |  |  |  |  |  |  |  |  |  |  |  |  |  |  |  | *ngn-1* | NeuroGeNin |
|  |  |  |  |  |  |  |  |  |  |  |  |  |  |  |  |  |  |  |  |  |  |  |  |  |  |  |  |  |  |  |  |  |  |  |  |  |  | ZK1320.11 |  |
|  |  |  |  |  |  |  |  |  |  |  |  |  |  |  |  |  |  |  |  |  |  |  |  |  |  |  |  |  |  |  |  |  |  |  |  |  |  | C13F10.2 |  |
|  |  |  |  |  |  |  |  |  |  |  |  |  |  |  |  |  |  |  |  |  |  |  |  |  |  |  |  |  |  |  |  |  |  |  |  |  |  | *trt-1* | Telomerase Reverse Transcriptase |
|  |  |  |  |  |  |  |  |  |  |  |  |  |  |  |  |  |  |  |  |  |  |  |  |  |  |  |  |  |  |  |  |  |  |  |  |  |  | C10A4.3 |  |
|  |  |  |  |  |  |  |  |  |  |  |  |  |  |  |  |  |  |  |  |  |  |  |  |  |  |  |  |  |  |  |  |  |  |  |  |  |  | C49C3.6 |  |
|  |  |  |  |  |  |  |  |  |  |  |  |  |  |  |  |  |  |  |  |  |  |  |  |  |  |  |  |  |  |  |  |  |  |  |  |  |  | C14A11.9 |  |
|  |  |  |  |  |  |  |  |  |  |  |  |  |  |  |  |  |  |  |  |  |  |  |  |  |  |  |  |  |  |  |  |  |  |  |  |  |  | F54D5.11 |  |
|  |  |  |  |  |  |  |  |  |  |  |  |  |  |  |  |  |  |  |  |  |  |  |  |  |  |  |  |  |  |  |  |  |  |  |  |  |  | F49E7.2 |  |
|  |  |  |  |  |  |  |  |  |  |  |  |  |  |  |  |  |  |  |  |  |  |  |  |  |  |  |  |  |  |  |  |  |  |  |  |  |  | *cic-1* | CyclIn C |
|  |  |  |  |  |  |  |  |  |  |  |  |  |  |  |  |  |  |  |  |  |  |  |  |  |  |  |  |  |  |  |  |  |  |  |  |  |  | *chn-1* | C-term of Hsp70-iNteracting protein (CHIP family) |
|  |  |  |  |  |  |  |  |  |  |  |  |  |  |  |  |  |  |  |  |  |  |  |  |  |  |  |  |  |  |  |  |  |  |  |  |  |  | W02D7.6 |  |
|  |  |  |  |  |  |  |  |  |  |  |  |  |  |  |  |  |  |  |  |  |  |  |  |  |  |  |  |  |  |  |  |  |  |  |  |  |  | *fbxc-54* | F-box C protein |
|  |  |  |  |  |  |  |  |  |  |  |  |  |  |  |  |  |  |  |  |  |  |  |  |  |  |  |  |  |  |  |  |  |  |  |  |  |  | B0025.4 |  |
|  |  |  |  |  |  |  |  |  |  |  |  |  |  |  |  |  |  |  |  |  |  |  |  |  |  |  |  |  |  |  |  |  |  |  |  |  |  | *mff-1* | Mitochondrial Fission Factor |
|  |  |  |  |  |  |  |  |  |  |  |  |  |  |  |  |  |  |  |  |  |  |  |  |  |  |  |  |  |  |  |  |  |  |  |  |  |  | F21D5.4 |  |
|  |  |  |  |  |  |  |  |  |  |  |  |  |  |  |  |  |  |  |  |  |  |  |  |  |  |  |  |  |  |  |  |  |  |  |  |  |  | *rab-10* | RAB family |
|  |  |  |  |  |  |  |  |  |  |  |  |  |  |  |  |  |  |  |  |  |  |  |  |  |  |  |  |  |  |  |  |  |  |  |  |  |  | *ceh-10* | C. Elegans Homeobox |
|  |  |  |  |  |  |  |  |  |  |  |  |  |  |  |  |  |  |  |  |  |  |  |  |  |  |  |  |  |  |  |  |  |  |  |  |  |  | F13G3.7 |  |
|  |  |  |  |  |  |  |  |  |  |  |  |  |  |  |  |  |  |  |  |  |  |  |  |  |  |  |  |  |  |  |  |  |  |  |  |  |  | *nprl-3* | Nitrogen Permease Regulator Like homolog |
|  |  |  |  |  |  |  |  |  |  |  |  |  |  |  |  |  |  |  |  |  |  |  |  |  |  |  |  |  |  |  |  |  |  |  |  |  |  | C49H3.9 |  |
|  |  |  |  |  |  |  |  |  |  |  |  |  |  |  |  |  |  |  |  |  |  |  |  |  |  |  |  |  |  |  |  |  |  |  |  |  |  | ZK673.4 |  |
|  |  |  |  |  |  |  |  |  |  |  |  |  |  |  |  |  |  |  |  |  |  |  |  |  |  |  |  |  |  |  |  |  |  |  |  |  |  | F21D5.5 |  |
|  |  |  |  |  |  |  |  |  |  |  |  |  |  |  |  |  |  |  |  |  |  |  |  |  |  |  |  |  |  |  |  |  |  |  |  |  |  | T26A5.4 |  |
|  |  |  |  |  |  |  |  |  |  |  |  |  |  |  |  |  |  |  |  |  |  |  |  |  |  |  |  |  |  |  |  |  |  |  |  |  |  | *pgs-1* | PhosphatidylGlycerophosphate Synthase |
|  |  |  |  |  |  |  |  |  |  |  |  |  |  |  |  |  |  |  |  |  |  |  |  |  |  |  |  |  |  |  |  |  |  |  |  |  |  | *hmg-1.2* | HMG |
|  |  |  |  |  |  |  |  |  |  |  |  |  |  |  |  |  |  |  |  |  |  |  |  |  |  |  |  |  |  |  |  |  |  |  |  |  |  | *sir-2.4* | yeast SIR related |
|  |  |  |  |  |  |  |  |  |  |  |  |  |  |  |  |  |  |  |  |  |  |  |  |  |  |  |  |  |  |  |  |  |  |  |  |  |  | Y97E10AR.6 |  |
|  |  |  |  |  |  |  |  |  |  |  |  |  |  |  |  |  |  |  |  |  |  |  |  |  |  |  |  |  |  |  |  |  |  |  |  |  |  | *ragc-1* | RAs-related GTP binding protein C homolog |
|  |  |  |  |  |  |  |  |  |  |  |  |  |  |  |  |  |  |  |  |  |  |  |  |  |  |  |  |  |  |  |  |  |  |  |  |  |  | C45B11.9 |  |
|  |  |  |  |  |  |  |  |  |  |  |  |  |  |  |  |  |  |  |  |  |  |  |  |  |  |  |  |  |  |  |  |  |  |  |  |  |  | Y73F8A.33 |  |
|  |  |  |  |  |  |  |  |  |  |  |  |  |  |  |  |  |  |  |  |  |  |  |  |  |  |  |  |  |  |  |  |  |  |  |  |  |  | C34F11.11 |  |
|  |  |  |  |  |  |  |  |  |  |  |  |  |  |  |  |  |  |  |  |  |  |  |  |  |  |  |  |  |  |  |  |  |  |  |  |  |  | T07F12.2 |  |
|  |  |  |  |  |  |  |  |  |  |  |  |  |  |  |  |  |  |  |  |  |  |  |  |  |  |  |  |  |  |  |  |  |  |  |  |  |  | Y49F6B.8 |  |
|  |  |  |  |  |  |  |  |  |  |  |  |  |  |  |  |  |  |  |  |  |  |  |  |  |  |  |  |  |  |  |  |  |  |  |  |  |  | Y46E12BL.8 |  |
|  |  |  |  |  |  |  |  |  |  |  |  |  |  |  |  |  |  |  |  |  |  |  |  |  |  |  |  |  |  |  |  |  |  |  |  |  |  | F01F1.14 |  |
|  |  |  |  |  |  |  |  |  |  |  |  |  |  |  |  |  |  |  |  |  |  |  |  |  |  |  |  |  |  |  |  |  |  |  |  |  |  | Y57G11C.48 |  |
|  |  |  |  |  |  |  |  |  |  |  |  |  |  |  |  |  |  |  |  |  |  |  |  |  |  |  |  |  |  |  |  |  |  |  |  |  |  | F21D5.9 |  |
|  |  |  |  |  |  |  |  |  |  |  |  |  |  |  |  |  |  |  |  |  |  |  |  |  |  |  |  |  |  |  |  |  |  |  |  |  |  | F16A11.5 |  |
|  |  |  |  |  |  |  |  |  |  |  |  |  |  |  |  |  |  |  |  |  |  |  |  |  |  |  |  |  |  |  |  |  |  |  |  |  |  | *anr-25* | Antisense Non-coding RNA |
|  |  |  |  |  |  |  |  |  |  |  |  |  |  |  |  |  |  |  |  |  |  |  |  |  |  |  |  |  |  |  |  |  |  |  |  |  |  | *mps-2* | MiRP K channel accessory Subunit |
|  |  |  |  |  |  |  |  |  |  |  |  |  |  |  |  |  |  |  |  |  |  |  |  |  |  |  |  |  |  |  |  |  |  |  |  |  |  | K01A2.4 |  |
|  |  |  |  |  |  |  |  |  |  |  |  |  |  |  |  |  |  |  |  |  |  |  |  |  |  |  |  |  |  |  |  |  |  |  |  |  |  | K01A2.3 |  |
|  |  |  |  |  |  |  |  |  |  |  |  |  |  |  |  |  |  |  |  |  |  |  |  |  |  |  |  |  |  |  |  |  |  |  |  |  |  | *tba-1* | TuBulin, Alpha |
|  |  |  |  |  |  |  |  |  |  |  |  |  |  |  |  |  |  |  |  |  |  |  |  |  |  |  |  |  |  |  |  |  |  |  |  |  |  | *oac-36* | O-ACyltransferase homolog |
|  |  |  |  |  |  |  |  |  |  |  |  |  |  |  |  |  |  |  |  |  |  |  |  |  |  |  |  |  |  |  |  |  |  |  |  |  |  | *tag-196* | Temporarily Assigned Gene name |
|  |  |  |  |  |  |  |  |  |  |  |  |  |  |  |  |  |  |  |  |  |  |  |  |  |  |  |  |  |  |  |  |  |  |  |  |  |  | *sdn-1* | SynDecaN |
|  |  |  |  |  |  |  |  |  |  |  |  |  |  |  |  |  |  |  |  |  |  |  |  |  |  |  |  |  |  |  |  |  |  |  |  |  |  | Y47H10A.4 |  |
|  |  |  |  |  |  |  |  |  |  |  |  |  |  |  |  |  |  |  |  |  |  |  |  |  |  |  |  |  |  |  |  |  |  |  |  |  |  | *linc-5* | Long Intervening Non-Coding RNA |
|  |  |  |  |  |  |  |  |  |  |  |  |  |  |  |  |  |  |  |  |  |  |  |  |  |  |  |  |  |  |  |  |  |  |  |  |  |  | K02F6.7 |  |
|  |  |  |  |  |  |  |  |  |  |  |  |  |  |  |  |  |  |  |  |  |  |  |  |  |  |  |  |  |  |  |  |  |  |  |  |  |  | F43E2.1 |  |
|  |  |  |  |  |  |  |  |  |  |  |  |  |  |  |  |  |  |  |  |  |  |  |  |  |  |  |  |  |  |  |  |  |  |  |  |  |  | K08F11.1 |  |
|  |  |  |  |  |  |  |  |  |  |  |  |  |  |  |  |  |  |  |  |  |  |  |  |  |  |  |  |  |  |  |  |  |  |  |  |  |  | F45E1.2 |  |
|  |  |  |  |  |  |  |  |  |  |  |  |  |  |  |  |  |  |  |  |  |  |  |  |  |  |  |  |  |  |  |  |  |  |  |  |  |  | C09F5.3 |  |
|  |  |  |  |  |  |  |  |  |  |  |  |  |  |  |  |  |  |  |  |  |  |  |  |  |  |  |  |  |  |  |  |  |  |  |  |  |  | *mbl-1* | MBL (muscleblind) splicing regulator homolog |
|  |  |  |  |  |  |  |  |  |  |  |  |  |  |  |  |  |  |  |  |  |  |  |  |  |  |  |  |  |  |  |  |  |  |  |  |  |  | R02D5.1 |  |
|  |  |  |  |  |  |  |  |  |  |  |  |  |  |  |  |  |  |  |  |  |  |  |  |  |  |  |  |  |  |  |  |  |  |  |  |  |  | T04F8.9 |  |
|  |  |  |  |  |  |  |  |  |  |  |  |  |  |  |  |  |  |  |  |  |  |  |  |  |  |  |  |  |  |  |  |  |  |  |  |  |  | *mdt-8* | MeDiaTor |
|  |  |  |  |  |  |  |  |  |  |  |  |  |  |  |  |  |  |  |  |  |  |  |  |  |  |  |  |  |  |  |  |  |  |  |  |  |  | K04C2.3 |  |
|  |  |  |  |  |  |  |  |  |  |  |  |  |  |  |  |  |  |  |  |  |  |  |  |  |  |  |  |  |  |  |  |  |  |  |  |  |  | C30A5.3 |  |
|  |  |  |  |  |  |  |  |  |  |  |  |  |  |  |  |  |  |  |  |  |  |  |  |  |  |  |  |  |  |  |  |  |  |  |  |  |  | *mec-15* | MEChanosensory abnormality |
|  |  |  |  |  |  |  |  |  |  |  |  |  |  |  |  |  |  |  |  |  |  |  |  |  |  |  |  |  |  |  |  |  |  |  |  |  |  | F10A3.17 |  |
|  |  |  |  |  |  |  |  |  |  |  |  |  |  |  |  |  |  |  |  |  |  |  |  |  |  |  |  |  |  |  |  |  |  |  |  |  |  | F54D5.12 |  |
|  |  |  |  |  |  |  |  |  |  |  |  |  |  |  |  |  |  |  |  |  |  |  |  |  |  |  |  |  |  |  |  |  |  |  |  |  |  | R05F9.9 |  |
|  |  |  |  |  |  |  |  |  |  |  |  |  |  |  |  |  |  |  |  |  |  |  |  |  |  |  |  |  |  |  |  |  |  |  |  |  |  | B0228.6 |  |
|  |  |  |  |  |  |  |  |  |  |  |  |  |  |  |  |  |  |  |  |  |  |  |  |  |  |  |  |  |  |  |  |  |  |  |  |  |  | C16A3.2 |  |
|  |  |  |  |  |  |  |  |  |  |  |  |  |  |  |  |  |  |  |  |  |  |  |  |  |  |  |  |  |  |  |  |  |  |  |  |  |  | Y34B4A.7 |  |
|  |  |  |  |  |  |  |  |  |  |  |  |  |  |  |  |  |  |  |  |  |  |  |  |  |  |  |  |  |  |  |  |  |  |  |  |  |  | Y71H2AM.20 |  |
|  |  |  |  |  |  |  |  |  |  |  |  |  |  |  |  |  |  |  |  |  |  |  |  |  |  |  |  |  |  |  |  |  |  |  |  |  |  | *srp-7* | SeRPin |
|  |  |  |  |  |  |  |  |  |  |  |  |  |  |  |  |  |  |  |  |  |  |  |  |  |  |  |  |  |  |  |  |  |  |  |  |  |  | C13F10.7 |  |
|  |  |  |  |  |  |  |  |  |  |  |  |  |  |  |  |  |  |  |  |  |  |  |  |  |  |  |  |  |  |  |  |  |  |  |  |  |  | *dnj-30* | DNaJ domain (prokaryotic heat shock protein) |
|  |  |  |  |  |  |  |  |  |  |  |  |  |  |  |  |  |  |  |  |  |  |  |  |  |  |  |  |  |  |  |  |  |  |  |  |  |  | *pqn-70* | Prion-like-(Q/N-rich)-domain-bearing protein |
|  |  |  |  |  |  |  |  |  |  |  |  |  |  |  |  |  |  |  |  |  |  |  |  |  |  |  |  |  |  |  |  |  |  |  |  |  |  | *bag-1* | BAG1 (human) homolog |
|  |  |  |  |  |  |  |  |  |  |  |  |  |  |  |  |  |  |  |  |  |  |  |  |  |  |  |  |  |  |  |  |  |  |  |  |  |  | *bir-1* | BIR (baculovirus inhibitory repeat) family |
|  |  |  |  |  |  |  |  |  |  |  |  |  |  |  |  |  |  |  |  |  |  |  |  |  |  |  |  |  |  |  |  |  |  |  |  |  |  | Y71H2AM.6 |  |
|  |  |  |  |  |  |  |  |  |  |  |  |  |  |  |  |  |  |  |  |  |  |  |  |  |  |  |  |  |  |  |  |  |  |  |  |  |  | *sna-1* | Small Nuclear RNA (snRNA) Associated protein |
|  |  |  |  |  |  |  |  |  |  |  |  |  |  |  |  |  |  |  |  |  |  |  |  |  |  |  |  |  |  |  |  |  |  |  |  |  |  | F49C12.9 |  |
|  |  |  |  |  |  |  |  |  |  |  |  |  |  |  |  |  |  |  |  |  |  |  |  |  |  |  |  |  |  |  |  |  |  |  |  |  |  | C25F6.1 |  |
|  |  |  |  |  |  |  |  |  |  |  |  |  |  |  |  |  |  |  |  |  |  |  |  |  |  |  |  |  |  |  |  |  |  |  |  |  |  | B0336.13 |  |
|  |  |  |  |  |  |  |  |  |  |  |  |  |  |  |  |  |  |  |  |  |  |  |  |  |  |  |  |  |  |  |  |  |  |  |  |  |  | *tag-170* | Temporarily Assigned Gene name |
|  |  |  |  |  |  |  |  |  |  |  |  |  |  |  |  |  |  |  |  |  |  |  |  |  |  |  |  |  |  |  |  |  |  |  |  |  |  | C47E8.4 |  |
|  |  |  |  |  |  |  |  |  |  |  |  |  |  |  |  |  |  |  |  |  |  |  |  |  |  |  |  |  |  |  |  |  |  |  |  |  |  | Y37E3.1 |  |
|  |  |  |  |  |  |  |  |  |  |  |  |  |  |  |  |  |  |  |  |  |  |  |  |  |  |  |  |  |  |  |  |  |  |  |  |  |  | C36B1.14 |  |
|  |  |  |  |  |  |  |  |  |  |  |  |  |  |  |  |  |  |  |  |  |  |  |  |  |  |  |  |  |  |  |  |  |  |  |  |  |  | M01E11.1 |  |
|  |  |  |  |  |  |  |  |  |  |  |  |  |  |  |  |  |  |  |  |  |  |  |  |  |  |  |  |  |  |  |  |  |  |  |  |  |  | R166.3 |  |
|  |  |  |  |  |  |  |  |  |  |  |  |  |  |  |  |  |  |  |  |  |  |  |  |  |  |  |  |  |  |  |  |  |  |  |  |  |  | Y53C10A.6 |  |
|  |  |  |  |  |  |  |  |  |  |  |  |  |  |  |  |  |  |  |  |  |  |  |  |  |  |  |  |  |  |  |  |  |  |  |  |  |  | T20D3.8 |  |
|  |  |  |  |  |  |  |  |  |  |  |  |  |  |  |  |  |  |  |  |  |  |  |  |  |  |  |  |  |  |  |  |  |  |  |  |  |  | C50E3.5 |  |
|  |  |  |  |  |  |  |  |  |  |  |  |  |  |  |  |  |  |  |  |  |  |  |  |  |  |  |  |  |  |  |  |  |  |  |  |  |  | *ceh-41* | C. Elegans Homeobox |
|  |  |  |  |  |  |  |  |  |  |  |  |  |  |  |  |  |  |  |  |  |  |  |  |  |  |  |  |  |  |  |  |  |  |  |  |  |  | *him-8* | High Incidence of Males (increased X chromosome loss) |
|  |  |  |  |  |  |  |  |  |  |  |  |  |  |  |  |  |  |  |  |  |  |  |  |  |  |  |  |  |  |  |  |  |  |  |  |  |  | *let-99* | LEThal |
|  |  |  |  |  |  |  |  |  |  |  |  |  |  |  |  |  |  |  |  |  |  |  |  |  |  |  |  |  |  |  |  |  |  |  |  |  |  | T02G5.7 |  |
|  |  |  |  |  |  |  |  |  |  |  |  |  |  |  |  |  |  |  |  |  |  |  |  |  |  |  |  |  |  |  |  |  |  |  |  |  |  | ZK1128.4 |  |
|  |  |  |  |  |  |  |  |  |  |  |  |  |  |  |  |  |  |  |  |  |  |  |  |  |  |  |  |  |  |  |  |  |  |  |  |  |  | *rab-21* | RAB family |
|  |  |  |  |  |  |  |  |  |  |  |  |  |  |  |  |  |  |  |  |  |  |  |  |  |  |  |  |  |  |  |  |  |  |  |  |  |  | *dnj-15* | DNaJ domain (prokaryotic heat shock protein) |
|  |  |  |  |  |  |  |  |  |  |  |  |  |  |  |  |  |  |  |  |  |  |  |  |  |  |  |  |  |  |  |  |  |  |  |  |  |  | *sdz-10* | SKN-1 Dependent Zygotic transcript |
|  |  |  |  |  |  |  |  |  |  |  |  |  |  |  |  |  |  |  |  |  |  |  |  |  |  |  |  |  |  |  |  |  |  |  |  |  |  | Y11D7A.19 |  |
|  |  |  |  |  |  |  |  |  |  |  |  |  |  |  |  |  |  |  |  |  |  |  |  |  |  |  |  |  |  |  |  |  |  |  |  |  |  | F37A4.2 |  |
|  |  |  |  |  |  |  |  |  |  |  |  |  |  |  |  |  |  |  |  |  |  |  |  |  |  |  |  |  |  |  |  |  |  |  |  |  |  | K11B4.2 |  |
|  |  |  |  |  |  |  |  |  |  |  |  |  |  |  |  |  |  |  |  |  |  |  |  |  |  |  |  |  |  |  |  |  |  |  |  |  |  | *rpn-7* | proteasome Regulatory Particle, Non-ATPase-like |
|  |  |  |  |  |  |  |  |  |  |  |  |  |  |  |  |  |  |  |  |  |  |  |  |  |  |  |  |  |  |  |  |  |  |  |  |  |  | B0285.4 |  |
|  |  |  |  |  |  |  |  |  |  |  |  |  |  |  |  |  |  |  |  |  |  |  |  |  |  |  |  |  |  |  |  |  |  |  |  |  |  | F37B4.10 |  |
|  |  |  |  |  |  |  |  |  |  |  |  |  |  |  |  |  |  |  |  |  |  |  |  |  |  |  |  |  |  |  |  |  |  |  |  |  |  | F32B6.3 |  |
|  |  |  |  |  |  |  |  |  |  |  |  |  |  |  |  |  |  |  |  |  |  |  |  |  |  |  |  |  |  |  |  |  |  |  |  |  |  | T04A8.8 |  |
|  |  |  |  |  |  |  |  |  |  |  |  |  |  |  |  |  |  |  |  |  |  |  |  |  |  |  |  |  |  |  |  |  |  |  |  |  |  | *cids-1* | pol II C-terminal Interaction Domain Suppressor |

### Phenotypes enriched

none found

### Anatomy terms enriched

none found

### GO terms enriched

none found

### Expression clusters enriched

|  |  |  |  |
| --- | --- | --- | --- |
| **Group name** | **Number in cluster** | **Enrichment** | **FDR corrected p** |
| FBF-associated probe sets (FDR <2.25%) | 54 | 2.08 | 2.30e-06 |
| TGF- Dauer pathway adult transcriptional targets. Results obtained by comparing the microarray results of the dauer-constitutive mutants daf-7(e1372), daf-7(m62), and daf-1(m40) with dauer-defective mutants daf-3(mgDf90), daf-5(e1386), and daf-7(e1372);daf-3(mgDf90) double mutants at the permissive temperature, 20C, on the first day of adulthood. WBPaper00031040:TGF-beta\_adult\_downregulated | 57 | 1.97 | 4.49e-06 |
| Maternal class (M): genes that are called present in at least one of the three PC6 replicates. | 69 | 1.72 | 7.44e-06 |
| Germline-enriched and sex-biased expression profile cluster E. | 27 | 3.29 | 1.19e-05 |
| Genes down-regulated after 300 um Tannic acid treatment. Fold change < 0.8. | 31 | 2.92 | 1.32e-05 |
| Genome-wide analysis of developmental and sex-regulated gene expression profile. cgc4489\_group\_2 | 30 | 2.81 | 5.01e-05 |
| oogenesis-enriched | 24 | 3.30 | 7.00e-05 |
| Caenorhabditis elegans Genes with expression levels changed significantly after treatment of Xenorhabdus nematophila. | 73 | 1.52 | 3.81e-04 |
| Expression Pattern Group F, enriched for genes involved in embryonic development. These patterns have in common that they all have genes of which the expression goes up after the juvenile stage. The expression of the genes in these patterns remains high or even goes up after reproduction. | 35 | 2.25 | 5.81e-04 |
| RNP-8-associated transcripts, based on microarray experiments. | 20 | 3.33 | 6.63e-04 |
| Caenorhabditis elegans Genes with expression levels changed significantly after treatment of Bacillus thurigiensis DB27. | 56 | 1.70 | 1.03e-03 |
| Genes for which heat shock F3 (fraction 3, containing heavy polysomes) versus control F3 is significantly increased. | 16 | 3.46 | 4.13e-03 |
| Maternal-embryonic class (ME): genes that are in the intersection of the maternal and embryonic classes. | 36 | 1.98 | 6.31e-03 |
| Genes expressed in embryonic motor neurons (identified by unc-4::GFP expressing cells). | 63 | 1.51 | 6.79e-03 |
| Embryonic class (E): genes that significantly increase in abundance at some point during embryogenesis. | 43 | 1.75 | 1.29e-02 |
| C-lineage related expression profile. WBPaper00025032:cluster\_151 | 3 | 39.60 | 1.30e-02 |
| 948 reproductively enriched mRNAs that co-immunoprecipitate with GLD-1. To identify GLD-1 mRNA targets, authors performed immunoprecipitation (IP) of GLD-1, followed by microarray analysis of the co-IPed mRNAs (RIP-chip). Extracts from young adult transgenic worms expressing a rescuing FLAG and GFP-tagged GLD-1, hereafter referred to as tagged GLD-1, were subjected to IP in triplicate with anti-FLAG (aFLAG IP) or anti-MYC (aMYC IP) antibodies as controls. Comparison of aFLAG IP versus aMYC IP to input revealed a large population of GLD-1-associated transcripts. Authors additionally performed complementary aFLAG IPs upon worms expressing either tagged GLD-1(GGF IP) or non-tagged GLD-1(N2 IP). Comparing transcript IP-enrichment values from both approaches revealed a correlation of 0.96, which indicated high reproducibility of GLD-1 association with specific mRNAs even on a quantitative level. | 17 | 2.79 | 2.64e-02 |
| Developmentally modulated gene cluster. cgc4386\_cluster\_5\_3 | 6 | 7.14 | 4.32e-02 |

### Motifs enriched

|  |  |  |  |  |  |
| --- | --- | --- | --- | --- | --- |
| **Motif** | **Logo** | **Possible orthologs** | **Number of motifs in cluster** | **Enrichment** | **FDR corrected p** |
| Mw138 |  | ceh-48 (0.87) dsc-1 | 20 | 3.42 | 0.00031 |
| MA0536.1 |  | elt-1 (0.55) | 19 | 2.85 | 0.00430 |
| pTH8982 |  | ceh-48 (0.87) | 17 | 3.03 | 0.00520 |
| pTH9054 |  | npax-1 | 17 | 2.75 | 0.01300 |
| pTH5634 |  | lin-31 | 71 | 1.36 | 0.01700 |
| CG5669\_SANGER\_10\_FBgn0039169 |  | klf-2 | 17 | 2.61 | 0.02100 |
| LHX2\_f1 |  | ceh-14 | 33 | 1.82 | 0.02300 |
| pTH2846 |  | lin-31 | 68 | 1.36 | 0.02600 |
| Zfp161\_2858 |  | pzf-1 | 17 | 2.52 | 0.02800 |
| Fkh1 |  | fkh-7 fkh-8 let-381 lin-31 | 67 | 1.36 | 0.02900 |
| pTH9393 |  | ZC416.1 | 36 | 1.72 | 0.03200 |
| pTH3477 |  | daf-16 | 66 | 1.37 | 0.03200 |
| Oli\_da\_SANGER\_5\_1\_FBgn0032651 |  | hlh-32 | 13 | 2.93 | 0.03500 |
| V$FOXJ2\_01 |  | lin-31 | 79 | 1.27 | 0.03700 |
| MA0236.1 |  | alr-1 | 55 | 1.43 | 0.04600 |
| V$GATA1\_01 |  | elt-1 (0.55) | 10 | 3.40 | 0.04700 |

### Correlated (and anti-correlated) transcription factors

|  |  |
| --- | --- |
| **Transcription factor** | **Correlation** |
| F52B5.7 | 0.88 |
| ceh-41 | 0.87 |
| ceh-48 | 0.87 |
| him-8 | 0.85 |
| T07F8.4 | 0.83 |
| dpff-1 | 0.82 |
| nfyc-1 | 0.81 |
| ceh-44 | 0.81 |
| lim-4 | 0.80 |
| dhhc-10 | 0.80 |
| W02D7.6 | 0.80 |
| syd-9 | 0.80 |
| F37B4.10 | 0.79 |
| ast-1 | 0.79 |
| Y5F2A.4 | 0.79 |
| zag-1 | 0.78 |
| unc-3 | 0.78 |
| ZK673.4 | 0.77 |
| rcor-1 | 0.77 |
| C09F5.3 | 0.77 |
| Y17G7B.22 | 0.76 |
| F54F2.9 | 0.75 |
| mbl-1 | 0.75 |
| F21D5.9 | 0.74 |
| T22C8.3 | 0.74 |
| nhr-61 | -0.66 |
| nhr-184 | -0.67 |
| nhr-103 | -0.67 |
| nhr-7 | -0.67 |
| peb-1 | -0.67 |
| nhr-58 | -0.67 |
| nhr-129 | -0.69 |
| nhr-202 | -0.69 |
| fkh-9 | -0.70 |
| nhr-133 | -0.71 |
| nhr-237 | -0.71 |
| nhr-60 | -0.72 |
| fos-1 | -0.72 |
| nhr-56 | -0.73 |
| nhr-104 | -0.74 |
| nhr-18 | -0.74 |
| nhr-5 | -0.74 |
| nhr-128 | -0.75 |
| grh-1 | -0.76 |
| nhr-141 | -0.79 |
| nhr-146 | -0.80 |
| nhr-112 | -0.80 |
| nhr-204 | -0.82 |
| ztf-27 | -0.87 |
| nhr-70 | -0.91 |

### ChIP peaks enriched

|  |  |  |  |  |
| --- | --- | --- | --- | --- |
| **Gene** | **Experiment** | **Number of upstream peaks** | **Enrichment** | **FDR corrected p** |
| efl-1 | EFL-1\_Larvae-L1-stage | 49 | 2.35 | 6.1e-08 |
| efl-1 | EFL-1\_Fed-L1-stage-larvae | 45 | 2.48 | 8.5e-08 |
| nfya-1 | NFYA-1\_Late-Embryos | 45 | 2.45 | 1.3e-07 |
| ces-1 | CES-1\_Embryos | 48 | 2.29 | 2.3e-07 |
| C34F6.9 | C34F6.9\_Larvae-L2-stage | 48 | 2.27 | 3.2e-07 |
| eor-1 | EOR-1\_Larvae-L3-stage | 48 | 2.21 | 7.6e-07 |
| F45C12.2 | F45C12.2\_Fed-L1-stage-larvae | 40 | 2.31 | 7.3e-06 |
| lsy-2 | LSY-2\_Larvae-L1-stage | 49 | 1.94 | 2.6e-05 |
| lin-35 | LIN-35\_Fed-L1-stage-larvae | 41 | 2.05 | 9.7e-05 |
| lsy-2 | LSY-2\_Embryos | 33 | 2.33 | 1.0e-04 |
| gei-11 | GEI-11\_Fed-L1-stage-larvae | 40 | 2.06 | 1.2e-04 |
| dpl-1 | DPL-1\_Larvae-L4-stage | 50 | 1.81 | 1.4e-04 |
| dpl-1 | DPL-1\_Fed-L1-stage-larvae | 38 | 2.06 | 2.5e-04 |
| lsy-2 | LSY-2\_Fed-L1-stage-larvae | 39 | 2.01 | 3.0e-04 |
| ceh-39 | CEH-39\_Embryos | 27 | 2.47 | 3.8e-04 |
| ceh-38 | CEH-38\_Larvae-L4-stage | 23 | 2.74 | 3.9e-04 |
| hpl-2 | HPL-2\_Fed-L1-stage-larvae | 46 | 1.81 | 4.7e-04 |
| gei-11 | GEI-11\_Larvae-L3-stage | 39 | 1.95 | 6.0e-04 |
| W03F9.2 | W03F9.2\_L4-Young-Adult-stage-larvae | 55 | 1.64 | 6.7e-04 |
| R02D3.7 | R02D3.7\_Larvae-L3-stage | 41 | 1.89 | 6.8e-04 |
| efl-1 | EFL-1\_Young-adult | 47 | 1.76 | 7.8e-04 |
| gei-11 | GEI-11\_Larvae-L2-stage | 34 | 2.04 | 1.1e-03 |
| ces-1 | CES-1\_Fed-L1-stage-larvae | 18 | 3.04 | 1.1e-03 |
| dpl-1 | DPL-1\_Young-adult | 35 | 1.99 | 1.3e-03 |
| nhr-2 | NHR-2\_Embryos | 18 | 2.61 | 6.5e-03 |
| nfya-1 | NFYA-1\_Larvae-L3-stage | 28 | 2.03 | 6.7e-03 |
| egl-5 | EGL-5\_Larvae-L3-stage | 28 | 2.02 | 7.2e-03 |
| R02D3.7 | R02D3.7\_Larvae-L2-stage | 26 | 2.09 | 7.6e-03 |
| C16A3.4 | C16A3.4\_Fed-L1-stage-larvae | 31 | 1.89 | 9.3e-03 |
| lin-13 | LIN-13\_Larvae-L2-stage | 23 | 2.18 | 1.0e-02 |
| ham-1 | HAM-1\_Fed-L1-stage-larvae | 36 | 1.76 | 1.1e-02 |
| nhr-23 | NHR-23\_Larvae-L3-stage | 33 | 1.82 | 1.1e-02 |
| nhr-237 | NHR-237\_Embryos | 19 | 2.39 | 1.2e-02 |
| ceh-26 | CEH-26\_Late-Embryonic-stage | 25 | 2.02 | 1.6e-02 |
| zag-1 | ZAG-1\_Fed-L1-stage-larvae | 20 | 2.26 | 1.7e-02 |
| sax-3 | SAX-3\_Larvae-L4-stage | 39 | 1.65 | 1.8e-02 |
| ham-1 | HAM-1\_Larvae-L4-stage | 37 | 1.68 | 1.9e-02 |
| dve-1 | DVE-1\_Late-Embryos | 30 | 1.83 | 1.9e-02 |
| sem-4 | SEM-4\_Larvae-L2-stage | 35 | 1.71 | 2.0e-02 |
| zag-1 | ZAG-1\_Larvae-L2-stage | 24 | 2.01 | 2.2e-02 |
| hlh-30 | HLH-30\_Late-Embryos | 18 | 2.28 | 2.8e-02 |
| jun-1 | JUN-1\_Larvae-L1-stage | 28 | 1.84 | 2.8e-02 |
| ztf-7 | ZTF-7\_Larvae-L4-stage | 23 | 1.99 | 3.2e-02 |
| pes-1 | PES-1\_Larvae-L4-stage | 35 | 1.66 | 3.3e-02 |
| sea-2 | SEA-2\_Larvae-L3-stage | 12 | 2.84 | 3.5e-02 |
| F23B12.7 | F23B12.7\_Young-adult | 25 | 1.88 | 4.1e-02 |
| nhr-6 | NHR-6\_Larvae-L4-stage | 21 | 2.02 | 4.3e-02 |
| C01B12.2 | C01B12.2\_Larvae-L2-stage | 43 | 1.52 | 4.4e-02 |
| pha-4 | PHA-4\_Larvae-L4-stage | 24 | 1.89 | 4.5e-02 |
