# Supplementary material for: Overlapping cell population expression profiling and regulatory inference in C. elegans
Source: BMC Genomics. 2016 Feb 29;17:159. doi: 10.1186/s12864-016-2482-z (PMC4772325; doi:10.1186/s12864-016-2482-z)
Supplement: Additional file 13: — Web supplement. (DOC 21 kb) [file 12864_2016_2482_MOESM13_ESM.zip › sortWeb/clusters/hier.300.clusters/147.html]

Cluster 147 

## Cluster 147

### Expression

| cnd-1 rep. 1 | cnd-1 rep. 2 | cnd-1 rep. 3 | pha-4 rep. 1 | pha-4 rep. 2 | pha-4 rep. 3 | ceh-27 | ceh-36 | ceh-6 | F21D5.9 | mir-57 | mls-2 | pal-1 | pros-1 | ttx-3 | unc-130 | hlh-16 | irx-1 | ceh-6 (+) hlh-16 (+) | ceh-6 (+) hlh-16 (-) | ceh-6 (-) hlh-16 (+) | cnd-1 singlets | pha-4 singlets | 0 | 60 | 120 | 150 | 180 | 240 | 330 | 390 | 420 | 480 | 540 | 570 | 600 | 630 | 660 | NAME | Functional description |
| --- | --- | --- | --- | --- | --- | --- | --- | --- | --- | --- | --- | --- | --- | --- | --- | --- | --- | --- | --- | --- | --- | --- | --- | --- | --- | --- | --- | --- | --- | --- | --- | --- | --- | --- | --- | --- | --- | --- | --- |
|  |  |  |  |  |  |  |  |  |  |  |  |  |  |  |  |  |  |  |  |  |  |  |  |  |  |  |  |  |  |  |  |  |  |  |  |  |  | F40E3.3 |  |
|  |  |  |  |  |  |  |  |  |  |  |  |  |  |  |  |  |  |  |  |  |  |  |  |  |  |  |  |  |  |  |  |  |  |  |  |  |  | F36A4.11 |  |
|  |  |  |  |  |  |  |  |  |  |  |  |  |  |  |  |  |  |  |  |  |  |  |  |  |  |  |  |  |  |  |  |  |  |  |  |  |  | F45E1.13 |  |
|  |  |  |  |  |  |  |  |  |  |  |  |  |  |  |  |  |  |  |  |  |  |  |  |  |  |  |  |  |  |  |  |  |  |  |  |  |  | D1081.5 |  |
|  |  |  |  |  |  |  |  |  |  |  |  |  |  |  |  |  |  |  |  |  |  |  |  |  |  |  |  |  |  |  |  |  |  |  |  |  |  | *math-29* | MATH (meprin-associated Traf homology) domain containing |
|  |  |  |  |  |  |  |  |  |  |  |  |  |  |  |  |  |  |  |  |  |  |  |  |  |  |  |  |  |  |  |  |  |  |  |  |  |  | C16E9.17 |  |
|  |  |  |  |  |  |  |  |  |  |  |  |  |  |  |  |  |  |  |  |  |  |  |  |  |  |  |  |  |  |  |  |  |  |  |  |  |  | F39B3.3 |  |
|  |  |  |  |  |  |  |  |  |  |  |  |  |  |  |  |  |  |  |  |  |  |  |  |  |  |  |  |  |  |  |  |  |  |  |  |  |  | ZK377.11 |  |
|  |  |  |  |  |  |  |  |  |  |  |  |  |  |  |  |  |  |  |  |  |  |  |  |  |  |  |  |  |  |  |  |  |  |  |  |  |  | *anr-41* | Antisense Non-coding RNA |
|  |  |  |  |  |  |  |  |  |  |  |  |  |  |  |  |  |  |  |  |  |  |  |  |  |  |  |  |  |  |  |  |  |  |  |  |  |  | *sls-2.3* | tranSpliced Leader Sequence |
|  |  |  |  |  |  |  |  |  |  |  |  |  |  |  |  |  |  |  |  |  |  |  |  |  |  |  |  |  |  |  |  |  |  |  |  |  |  | ZC64.9 |  |
|  |  |  |  |  |  |  |  |  |  |  |  |  |  |  |  |  |  |  |  |  |  |  |  |  |  |  |  |  |  |  |  |  |  |  |  |  |  | Y116A8A.7 |  |
|  |  |  |  |  |  |  |  |  |  |  |  |  |  |  |  |  |  |  |  |  |  |  |  |  |  |  |  |  |  |  |  |  |  |  |  |  |  | *grl-9* | GRound-Like (grd related) |
|  |  |  |  |  |  |  |  |  |  |  |  |  |  |  |  |  |  |  |  |  |  |  |  |  |  |  |  |  |  |  |  |  |  |  |  |  |  | K09F5.8 |  |
|  |  |  |  |  |  |  |  |  |  |  |  |  |  |  |  |  |  |  |  |  |  |  |  |  |  |  |  |  |  |  |  |  |  |  |  |  |  | C54F6.2 |  |
|  |  |  |  |  |  |  |  |  |  |  |  |  |  |  |  |  |  |  |  |  |  |  |  |  |  |  |  |  |  |  |  |  |  |  |  |  |  | F53B7.8 |  |
|  |  |  |  |  |  |  |  |  |  |  |  |  |  |  |  |  |  |  |  |  |  |  |  |  |  |  |  |  |  |  |  |  |  |  |  |  |  | F22D6.15 |  |
|  |  |  |  |  |  |  |  |  |  |  |  |  |  |  |  |  |  |  |  |  |  |  |  |  |  |  |  |  |  |  |  |  |  |  |  |  |  | F26A10.17 |  |
|  |  |  |  |  |  |  |  |  |  |  |  |  |  |  |  |  |  |  |  |  |  |  |  |  |  |  |  |  |  |  |  |  |  |  |  |  |  | R07E5.16 |  |
|  |  |  |  |  |  |  |  |  |  |  |  |  |  |  |  |  |  |  |  |  |  |  |  |  |  |  |  |  |  |  |  |  |  |  |  |  |  | C16E9.20 |  |
|  |  |  |  |  |  |  |  |  |  |  |  |  |  |  |  |  |  |  |  |  |  |  |  |  |  |  |  |  |  |  |  |  |  |  |  |  |  | ZC123.9 |  |
|  |  |  |  |  |  |  |  |  |  |  |  |  |  |  |  |  |  |  |  |  |  |  |  |  |  |  |  |  |  |  |  |  |  |  |  |  |  | F13D11.19 |  |
|  |  |  |  |  |  |  |  |  |  |  |  |  |  |  |  |  |  |  |  |  |  |  |  |  |  |  |  |  |  |  |  |  |  |  |  |  |  | R05G6.20 |  |
|  |  |  |  |  |  |  |  |  |  |  |  |  |  |  |  |  |  |  |  |  |  |  |  |  |  |  |  |  |  |  |  |  |  |  |  |  |  | B0403.12 |  |
|  |  |  |  |  |  |  |  |  |  |  |  |  |  |  |  |  |  |  |  |  |  |  |  |  |  |  |  |  |  |  |  |  |  |  |  |  |  | C06C6.6 |  |
|  |  |  |  |  |  |  |  |  |  |  |  |  |  |  |  |  |  |  |  |  |  |  |  |  |  |  |  |  |  |  |  |  |  |  |  |  |  | K02E11.9 |  |
|  |  |  |  |  |  |  |  |  |  |  |  |  |  |  |  |  |  |  |  |  |  |  |  |  |  |  |  |  |  |  |  |  |  |  |  |  |  | R144.16 |  |
|  |  |  |  |  |  |  |  |  |  |  |  |  |  |  |  |  |  |  |  |  |  |  |  |  |  |  |  |  |  |  |  |  |  |  |  |  |  | C54D10.12 |  |
|  |  |  |  |  |  |  |  |  |  |  |  |  |  |  |  |  |  |  |  |  |  |  |  |  |  |  |  |  |  |  |  |  |  |  |  |  |  | F58F9.4 |  |
|  |  |  |  |  |  |  |  |  |  |  |  |  |  |  |  |  |  |  |  |  |  |  |  |  |  |  |  |  |  |  |  |  |  |  |  |  |  | Y113G7A.12 |  |

### Phenotypes enriched

none found

### Anatomy terms enriched

none found

### GO terms enriched

none found

### Expression clusters enriched

none found

### Motifs enriched

|  |  |  |  |  |  |
| --- | --- | --- | --- | --- | --- |
| **Motif** | **Logo** | **Possible orthologs** | **Number of motifs in cluster** | **Enrichment** | **FDR corrected p** |
| Hoxd9\_2 |  | lin-39 | 8 | 4.89 | 0.015 |
| Hoxd9\_1 |  | lin-39 | 7 | 5.11 | 0.026 |
| POU3F3\_3 |  | ceh-18 | 7 | 4.99 | 0.029 |
| ovo\_FlyReg\_FBgn0003028 |  | lin-48 | 7 | 4.80 | 0.034 |
| pTH10633 |  | R07H5.10 | 15 | 2.27 | 0.044 |

### Correlated (and anti-correlated) transcription factors

|  |  |
| --- | --- |
| **Transcription factor** | **Correlation** |
| nhr-196 | 0.73 |
| npax-1 | 0.66 |
| ceh-54 | 0.62 |
| nhr-198 | 0.61 |
| nhr-197 | 0.61 |
| dmd-10 | 0.58 |
| aptf-4 | 0.58 |
| nhr-268 | 0.58 |
| nhr-101 | 0.58 |
| ceh-36 | 0.57 |
| mgl-2 | 0.55 |
| nhr-36 | 0.55 |
| mbf-1 | 0.55 |
| gei-3 | 0.54 |
| nhr-26 | 0.54 |
| dhhc-7 | 0.53 |
| lsy-27 | 0.52 |
| mbr-1 | 0.51 |
| madf-4 | 0.50 |
| ceh-9 | 0.50 |
| nfya-2 | 0.49 |
| nhr-30 | 0.49 |
| C09F5.3 | 0.49 |
| ceh-62 | 0.49 |
| xbp-1 | 0.48 |
| sem-2 | -0.42 |
| flh-3 | -0.42 |
| nhr-42 | -0.42 |
| nhr-67 | -0.42 |
| nhr-146 | -0.43 |
| alr-1 | -0.43 |
| egl-18 | -0.44 |
| dnj-11 | -0.44 |
| ztf-13 | -0.45 |
| ceh-43 | -0.46 |
| dhhc-6 | -0.46 |
| daf-8 | -0.47 |
| ces-2 | -0.47 |
| pros-1 | -0.48 |
| ztf-29 | -0.48 |
| irx-1 | -0.48 |
| pop-1 | -0.49 |
| hlh-2 | -0.49 |
| ces-1 | -0.50 |
| duxl-1 | -0.51 |
| nhr-104 | -0.51 |
| ztf-11 | -0.55 |
| nhr-13 | -0.55 |
| unc-86 | -0.55 |
| nhr-122 | -0.63 |

### ChIP peaks enriched

none found
