# Supplementary material for: Overlapping cell population expression profiling and regulatory inference in C. elegans
Source: BMC Genomics. 2016 Feb 29;17:159. doi: 10.1186/s12864-016-2482-z (PMC4772325; doi:10.1186/s12864-016-2482-z)
Supplement: Additional file 13: — Web supplement. (DOC 21 kb) [file 12864_2016_2482_MOESM13_ESM.zip › sortWeb/clusters/hier.300.clusters/153.html]

Cluster 153 

## Cluster 153

### Expression

| cnd-1 rep. 1 | cnd-1 rep. 2 | cnd-1 rep. 3 | pha-4 rep. 1 | pha-4 rep. 2 | pha-4 rep. 3 | ceh-27 | ceh-36 | ceh-6 | F21D5.9 | mir-57 | mls-2 | pal-1 | pros-1 | ttx-3 | unc-130 | hlh-16 | irx-1 | ceh-6 (+) hlh-16 (+) | ceh-6 (+) hlh-16 (-) | ceh-6 (-) hlh-16 (+) | cnd-1 singlets | pha-4 singlets | 0 | 60 | 120 | 150 | 180 | 240 | 330 | 390 | 420 | 480 | 540 | 570 | 600 | 630 | 660 | NAME | Functional description |
| --- | --- | --- | --- | --- | --- | --- | --- | --- | --- | --- | --- | --- | --- | --- | --- | --- | --- | --- | --- | --- | --- | --- | --- | --- | --- | --- | --- | --- | --- | --- | --- | --- | --- | --- | --- | --- | --- | --- | --- |
|  |  |  |  |  |  |  |  |  |  |  |  |  |  |  |  |  |  |  |  |  |  |  |  |  |  |  |  |  |  |  |  |  |  |  |  |  |  | Y65B4A.7 |  |
|  |  |  |  |  |  |  |  |  |  |  |  |  |  |  |  |  |  |  |  |  |  |  |  |  |  |  |  |  |  |  |  |  |  |  |  |  |  | C42D8.t1 |  |
|  |  |  |  |  |  |  |  |  |  |  |  |  |  |  |  |  |  |  |  |  |  |  |  |  |  |  |  |  |  |  |  |  |  |  |  |  |  | F31E8.11 |  |
|  |  |  |  |  |  |  |  |  |  |  |  |  |  |  |  |  |  |  |  |  |  |  |  |  |  |  |  |  |  |  |  |  |  |  |  |  |  | Y47H9A.1 |  |
|  |  |  |  |  |  |  |  |  |  |  |  |  |  |  |  |  |  |  |  |  |  |  |  |  |  |  |  |  |  |  |  |  |  |  |  |  |  | *srx-73* | Serpentine Receptor, class X |
|  |  |  |  |  |  |  |  |  |  |  |  |  |  |  |  |  |  |  |  |  |  |  |  |  |  |  |  |  |  |  |  |  |  |  |  |  |  | R09E12.9 |  |
|  |  |  |  |  |  |  |  |  |  |  |  |  |  |  |  |  |  |  |  |  |  |  |  |  |  |  |  |  |  |  |  |  |  |  |  |  |  | Y18H1A.4 |  |
|  |  |  |  |  |  |  |  |  |  |  |  |  |  |  |  |  |  |  |  |  |  |  |  |  |  |  |  |  |  |  |  |  |  |  |  |  |  | *fil-2* | Fasting Induced Lipase |
|  |  |  |  |  |  |  |  |  |  |  |  |  |  |  |  |  |  |  |  |  |  |  |  |  |  |  |  |  |  |  |  |  |  |  |  |  |  | F15H9.7 |  |
|  |  |  |  |  |  |  |  |  |  |  |  |  |  |  |  |  |  |  |  |  |  |  |  |  |  |  |  |  |  |  |  |  |  |  |  |  |  | C31E10.1 |  |
|  |  |  |  |  |  |  |  |  |  |  |  |  |  |  |  |  |  |  |  |  |  |  |  |  |  |  |  |  |  |  |  |  |  |  |  |  |  | *ndx-2* | NuDiX family |
|  |  |  |  |  |  |  |  |  |  |  |  |  |  |  |  |  |  |  |  |  |  |  |  |  |  |  |  |  |  |  |  |  |  |  |  |  |  | F58D5.9 |  |
|  |  |  |  |  |  |  |  |  |  |  |  |  |  |  |  |  |  |  |  |  |  |  |  |  |  |  |  |  |  |  |  |  |  |  |  |  |  | W02G9.4 |  |
|  |  |  |  |  |  |  |  |  |  |  |  |  |  |  |  |  |  |  |  |  |  |  |  |  |  |  |  |  |  |  |  |  |  |  |  |  |  | K07A1.6 |  |
|  |  |  |  |  |  |  |  |  |  |  |  |  |  |  |  |  |  |  |  |  |  |  |  |  |  |  |  |  |  |  |  |  |  |  |  |  |  | ZK1037.2 |  |
|  |  |  |  |  |  |  |  |  |  |  |  |  |  |  |  |  |  |  |  |  |  |  |  |  |  |  |  |  |  |  |  |  |  |  |  |  |  | *his-48* | HIStone |
|  |  |  |  |  |  |  |  |  |  |  |  |  |  |  |  |  |  |  |  |  |  |  |  |  |  |  |  |  |  |  |  |  |  |  |  |  |  | *his-5* | HIStone |
|  |  |  |  |  |  |  |  |  |  |  |  |  |  |  |  |  |  |  |  |  |  |  |  |  |  |  |  |  |  |  |  |  |  |  |  |  |  | *pbs-5* | Proteasome Beta Subunit |
|  |  |  |  |  |  |  |  |  |  |  |  |  |  |  |  |  |  |  |  |  |  |  |  |  |  |  |  |  |  |  |  |  |  |  |  |  |  | *glct-6* | GLuCuronosylTransferase-like |
|  |  |  |  |  |  |  |  |  |  |  |  |  |  |  |  |  |  |  |  |  |  |  |  |  |  |  |  |  |  |  |  |  |  |  |  |  |  | F56B6.6 |  |
|  |  |  |  |  |  |  |  |  |  |  |  |  |  |  |  |  |  |  |  |  |  |  |  |  |  |  |  |  |  |  |  |  |  |  |  |  |  | C06G3.3 |  |
|  |  |  |  |  |  |  |  |  |  |  |  |  |  |  |  |  |  |  |  |  |  |  |  |  |  |  |  |  |  |  |  |  |  |  |  |  |  | Y38H8A.2 |  |
|  |  |  |  |  |  |  |  |  |  |  |  |  |  |  |  |  |  |  |  |  |  |  |  |  |  |  |  |  |  |  |  |  |  |  |  |  |  | C07A4.3 |  |
|  |  |  |  |  |  |  |  |  |  |  |  |  |  |  |  |  |  |  |  |  |  |  |  |  |  |  |  |  |  |  |  |  |  |  |  |  |  | Y59E9AL.3 |  |
|  |  |  |  |  |  |  |  |  |  |  |  |  |  |  |  |  |  |  |  |  |  |  |  |  |  |  |  |  |  |  |  |  |  |  |  |  |  | *str-267* | Seven TM Receptor |
|  |  |  |  |  |  |  |  |  |  |  |  |  |  |  |  |  |  |  |  |  |  |  |  |  |  |  |  |  |  |  |  |  |  |  |  |  |  | T23G11.11 |  |
|  |  |  |  |  |  |  |  |  |  |  |  |  |  |  |  |  |  |  |  |  |  |  |  |  |  |  |  |  |  |  |  |  |  |  |  |  |  | Y69A2AR.3 |  |
|  |  |  |  |  |  |  |  |  |  |  |  |  |  |  |  |  |  |  |  |  |  |  |  |  |  |  |  |  |  |  |  |  |  |  |  |  |  | *linc-76* | Long Intervening Non-Coding RNA |
|  |  |  |  |  |  |  |  |  |  |  |  |  |  |  |  |  |  |  |  |  |  |  |  |  |  |  |  |  |  |  |  |  |  |  |  |  |  | *sdhd-1* | Succinate DeHydrogenase complex subunit D |
|  |  |  |  |  |  |  |  |  |  |  |  |  |  |  |  |  |  |  |  |  |  |  |  |  |  |  |  |  |  |  |  |  |  |  |  |  |  | *his-71* | HIStone |
|  |  |  |  |  |  |  |  |  |  |  |  |  |  |  |  |  |  |  |  |  |  |  |  |  |  |  |  |  |  |  |  |  |  |  |  |  |  | C25H3.17 |  |
|  |  |  |  |  |  |  |  |  |  |  |  |  |  |  |  |  |  |  |  |  |  |  |  |  |  |  |  |  |  |  |  |  |  |  |  |  |  | W10D9.6 |  |
|  |  |  |  |  |  |  |  |  |  |  |  |  |  |  |  |  |  |  |  |  |  |  |  |  |  |  |  |  |  |  |  |  |  |  |  |  |  | Y48G8AL.15 |  |
|  |  |  |  |  |  |  |  |  |  |  |  |  |  |  |  |  |  |  |  |  |  |  |  |  |  |  |  |  |  |  |  |  |  |  |  |  |  | K12H4.5 |  |
|  |  |  |  |  |  |  |  |  |  |  |  |  |  |  |  |  |  |  |  |  |  |  |  |  |  |  |  |  |  |  |  |  |  |  |  |  |  | F31D4.9 |  |
|  |  |  |  |  |  |  |  |  |  |  |  |  |  |  |  |  |  |  |  |  |  |  |  |  |  |  |  |  |  |  |  |  |  |  |  |  |  | *ife-4* | Initiation Factor 4E (eIF4E) family |
|  |  |  |  |  |  |  |  |  |  |  |  |  |  |  |  |  |  |  |  |  |  |  |  |  |  |  |  |  |  |  |  |  |  |  |  |  |  | C31H5.4 |  |
|  |  |  |  |  |  |  |  |  |  |  |  |  |  |  |  |  |  |  |  |  |  |  |  |  |  |  |  |  |  |  |  |  |  |  |  |  |  | *cpi-2* | Cysteine Protease Inhibitor |
|  |  |  |  |  |  |  |  |  |  |  |  |  |  |  |  |  |  |  |  |  |  |  |  |  |  |  |  |  |  |  |  |  |  |  |  |  |  | *acl-11* | ACyLtransferase-like |
|  |  |  |  |  |  |  |  |  |  |  |  |  |  |  |  |  |  |  |  |  |  |  |  |  |  |  |  |  |  |  |  |  |  |  |  |  |  | F55A4.7 |  |
|  |  |  |  |  |  |  |  |  |  |  |  |  |  |  |  |  |  |  |  |  |  |  |  |  |  |  |  |  |  |  |  |  |  |  |  |  |  | C45G9.5 |  |
|  |  |  |  |  |  |  |  |  |  |  |  |  |  |  |  |  |  |  |  |  |  |  |  |  |  |  |  |  |  |  |  |  |  |  |  |  |  | *vha-3* | Vacuolar H ATPase |
|  |  |  |  |  |  |  |  |  |  |  |  |  |  |  |  |  |  |  |  |  |  |  |  |  |  |  |  |  |  |  |  |  |  |  |  |  |  | F08B6.1 |  |
|  |  |  |  |  |  |  |  |  |  |  |  |  |  |  |  |  |  |  |  |  |  |  |  |  |  |  |  |  |  |  |  |  |  |  |  |  |  | *nduf-6* | NADH Ubiquinone oxidoreductase Fe-S protein |
|  |  |  |  |  |  |  |  |  |  |  |  |  |  |  |  |  |  |  |  |  |  |  |  |  |  |  |  |  |  |  |  |  |  |  |  |  |  | *hpo-17* | Hypersensitive to POre-forming toxin |
|  |  |  |  |  |  |  |  |  |  |  |  |  |  |  |  |  |  |  |  |  |  |  |  |  |  |  |  |  |  |  |  |  |  |  |  |  |  | K10B2.4 |  |
|  |  |  |  |  |  |  |  |  |  |  |  |  |  |  |  |  |  |  |  |  |  |  |  |  |  |  |  |  |  |  |  |  |  |  |  |  |  | *dyrb-1* | DYnein light chain (RoadBlock type) |
|  |  |  |  |  |  |  |  |  |  |  |  |  |  |  |  |  |  |  |  |  |  |  |  |  |  |  |  |  |  |  |  |  |  |  |  |  |  | *pbs-6* | Proteasome Beta Subunit |
|  |  |  |  |  |  |  |  |  |  |  |  |  |  |  |  |  |  |  |  |  |  |  |  |  |  |  |  |  |  |  |  |  |  |  |  |  |  | F48C1.5 |  |
|  |  |  |  |  |  |  |  |  |  |  |  |  |  |  |  |  |  |  |  |  |  |  |  |  |  |  |  |  |  |  |  |  |  |  |  |  |  | *vps-29* | related to yeast Vacuolar Protein Sorting factor |
|  |  |  |  |  |  |  |  |  |  |  |  |  |  |  |  |  |  |  |  |  |  |  |  |  |  |  |  |  |  |  |  |  |  |  |  |  |  | *immp-1* | Inner Mitochondrial Membrane Protease |
|  |  |  |  |  |  |  |  |  |  |  |  |  |  |  |  |  |  |  |  |  |  |  |  |  |  |  |  |  |  |  |  |  |  |  |  |  |  | F26F4.9 |  |
|  |  |  |  |  |  |  |  |  |  |  |  |  |  |  |  |  |  |  |  |  |  |  |  |  |  |  |  |  |  |  |  |  |  |  |  |  |  | Y57G11C.38 |  |
|  |  |  |  |  |  |  |  |  |  |  |  |  |  |  |  |  |  |  |  |  |  |  |  |  |  |  |  |  |  |  |  |  |  |  |  |  |  | F11E6.10 |  |
|  |  |  |  |  |  |  |  |  |  |  |  |  |  |  |  |  |  |  |  |  |  |  |  |  |  |  |  |  |  |  |  |  |  |  |  |  |  | *stdh-1* | STeroid DeHydrogenase family |
|  |  |  |  |  |  |  |  |  |  |  |  |  |  |  |  |  |  |  |  |  |  |  |  |  |  |  |  |  |  |  |  |  |  |  |  |  |  | T20G5.14 |  |
|  |  |  |  |  |  |  |  |  |  |  |  |  |  |  |  |  |  |  |  |  |  |  |  |  |  |  |  |  |  |  |  |  |  |  |  |  |  | *eif-3.K* | Eukaryotic Initiation Factor |
|  |  |  |  |  |  |  |  |  |  |  |  |  |  |  |  |  |  |  |  |  |  |  |  |  |  |  |  |  |  |  |  |  |  |  |  |  |  | Y52B11A.8 |  |
|  |  |  |  |  |  |  |  |  |  |  |  |  |  |  |  |  |  |  |  |  |  |  |  |  |  |  |  |  |  |  |  |  |  |  |  |  |  | *dct-13* | DAF-16/FOXO Controlled, germline Tumor affecting |
|  |  |  |  |  |  |  |  |  |  |  |  |  |  |  |  |  |  |  |  |  |  |  |  |  |  |  |  |  |  |  |  |  |  |  |  |  |  | B0350.79 |  |
|  |  |  |  |  |  |  |  |  |  |  |  |  |  |  |  |  |  |  |  |  |  |  |  |  |  |  |  |  |  |  |  |  |  |  |  |  |  | F10E9.16 |  |
|  |  |  |  |  |  |  |  |  |  |  |  |  |  |  |  |  |  |  |  |  |  |  |  |  |  |  |  |  |  |  |  |  |  |  |  |  |  | *ctb-1* | CyTochrome B |
|  |  |  |  |  |  |  |  |  |  |  |  |  |  |  |  |  |  |  |  |  |  |  |  |  |  |  |  |  |  |  |  |  |  |  |  |  |  | *vha-1* | Vacuolar H ATPase |
|  |  |  |  |  |  |  |  |  |  |  |  |  |  |  |  |  |  |  |  |  |  |  |  |  |  |  |  |  |  |  |  |  |  |  |  |  |  | *vha-14* | Vacuolar H ATPase |
|  |  |  |  |  |  |  |  |  |  |  |  |  |  |  |  |  |  |  |  |  |  |  |  |  |  |  |  |  |  |  |  |  |  |  |  |  |  | *vha-4* | Vacuolar H ATPase |
|  |  |  |  |  |  |  |  |  |  |  |  |  |  |  |  |  |  |  |  |  |  |  |  |  |  |  |  |  |  |  |  |  |  |  |  |  |  | *vha-17* | Vacuolar H ATPase |
|  |  |  |  |  |  |  |  |  |  |  |  |  |  |  |  |  |  |  |  |  |  |  |  |  |  |  |  |  |  |  |  |  |  |  |  |  |  | F49C12.12 |  |
|  |  |  |  |  |  |  |  |  |  |  |  |  |  |  |  |  |  |  |  |  |  |  |  |  |  |  |  |  |  |  |  |  |  |  |  |  |  | *trx-4* | ThioRedoXin [see also xtr] |
|  |  |  |  |  |  |  |  |  |  |  |  |  |  |  |  |  |  |  |  |  |  |  |  |  |  |  |  |  |  |  |  |  |  |  |  |  |  | B0546.4 |  |
|  |  |  |  |  |  |  |  |  |  |  |  |  |  |  |  |  |  |  |  |  |  |  |  |  |  |  |  |  |  |  |  |  |  |  |  |  |  | Y106G6H.8 |  |
|  |  |  |  |  |  |  |  |  |  |  |  |  |  |  |  |  |  |  |  |  |  |  |  |  |  |  |  |  |  |  |  |  |  |  |  |  |  | C41G7.9 |  |
|  |  |  |  |  |  |  |  |  |  |  |  |  |  |  |  |  |  |  |  |  |  |  |  |  |  |  |  |  |  |  |  |  |  |  |  |  |  | *vha-9* | Vacuolar H ATPase |
|  |  |  |  |  |  |  |  |  |  |  |  |  |  |  |  |  |  |  |  |  |  |  |  |  |  |  |  |  |  |  |  |  |  |  |  |  |  | F09E5.9 |  |
|  |  |  |  |  |  |  |  |  |  |  |  |  |  |  |  |  |  |  |  |  |  |  |  |  |  |  |  |  |  |  |  |  |  |  |  |  |  | F57B10.5 |  |
|  |  |  |  |  |  |  |  |  |  |  |  |  |  |  |  |  |  |  |  |  |  |  |  |  |  |  |  |  |  |  |  |  |  |  |  |  |  | F09E5.11 |  |
|  |  |  |  |  |  |  |  |  |  |  |  |  |  |  |  |  |  |  |  |  |  |  |  |  |  |  |  |  |  |  |  |  |  |  |  |  |  | *dss-1* | DSS1 (Deleted in Split hand/Split foot protein 1) homolog 26S proteasome subunit |
|  |  |  |  |  |  |  |  |  |  |  |  |  |  |  |  |  |  |  |  |  |  |  |  |  |  |  |  |  |  |  |  |  |  |  |  |  |  | F42G8.10 |  |
|  |  |  |  |  |  |  |  |  |  |  |  |  |  |  |  |  |  |  |  |  |  |  |  |  |  |  |  |  |  |  |  |  |  |  |  |  |  | *pfd-4* | PreFolDin (molecular chaperone) |
|  |  |  |  |  |  |  |  |  |  |  |  |  |  |  |  |  |  |  |  |  |  |  |  |  |  |  |  |  |  |  |  |  |  |  |  |  |  | Y74C10AL.2 |  |
|  |  |  |  |  |  |  |  |  |  |  |  |  |  |  |  |  |  |  |  |  |  |  |  |  |  |  |  |  |  |  |  |  |  |  |  |  |  | C25H3.18 |  |
|  |  |  |  |  |  |  |  |  |  |  |  |  |  |  |  |  |  |  |  |  |  |  |  |  |  |  |  |  |  |  |  |  |  |  |  |  |  | Y67D2.5 |  |
|  |  |  |  |  |  |  |  |  |  |  |  |  |  |  |  |  |  |  |  |  |  |  |  |  |  |  |  |  |  |  |  |  |  |  |  |  |  | *pas-1* | Proteasome Alpha Subunit |
|  |  |  |  |  |  |  |  |  |  |  |  |  |  |  |  |  |  |  |  |  |  |  |  |  |  |  |  |  |  |  |  |  |  |  |  |  |  | *glrx-10* | GLutaRedoXin |
|  |  |  |  |  |  |  |  |  |  |  |  |  |  |  |  |  |  |  |  |  |  |  |  |  |  |  |  |  |  |  |  |  |  |  |  |  |  | *emc-6* | EMC Endoplasmic Membrane protein Complex (yeast EMC) homolog |
|  |  |  |  |  |  |  |  |  |  |  |  |  |  |  |  |  |  |  |  |  |  |  |  |  |  |  |  |  |  |  |  |  |  |  |  |  |  | *immp-2* | Inner Mitochondrial Membrane Protease |
|  |  |  |  |  |  |  |  |  |  |  |  |  |  |  |  |  |  |  |  |  |  |  |  |  |  |  |  |  |  |  |  |  |  |  |  |  |  | C06H2.6 |  |

### Phenotypes enriched

none found

### Anatomy terms enriched

none found

### GO terms enriched

|  |  |  |
| --- | --- | --- |
| **GO term** | **Number of genes** | **FDR-corrected p-value** |
| ATP hydrolysis coupled proton transport | 4 | 0.00041 |
| hydrogen ion transmembrane transport | 4 | 0.00190 |
| nematode larval development | 18 | 0.00480 |
| post-embryonic development | 18 | 0.00530 |
| threonine-type endopeptidase activity | 3 | 0.00660 |
| hydrogen ion transmembrane transporter activity | 3 | 0.04500 |
| cell death | 8 | 0.04600 |

### Expression clusters enriched

|  |  |  |  |
| --- | --- | --- | --- |
| **Group name** | **Number in cluster** | **Enrichment** | **FDR corrected p** |
| Maternal degradation class (MD): genes that are the subset of maternal genes that decrease without first increasing in abundance. | 23 | 2.40 | 0.0119 |
| Caenorhabditis elegans Genes with expression levels changed significantly after treatment of Bacillus thurigiensis DB27. | 44 | 1.68 | 0.0129 |
| Genes enriched in intestine. | 24 | 2.18 | 0.0317 |

### Motifs enriched

|  |  |  |  |  |  |
| --- | --- | --- | --- | --- | --- |
| **Motif** | **Logo** | **Possible orthologs** | **Number of motifs in cluster** | **Enrichment** | **FDR corrected p** |
| ONECUT2\_1 |  | ceh-48 | 21 | 2.46 | 0.0087 |
| pTH5098 |  | F45H11.6 | 8 | 5.66 | 0.0094 |
| pTH9958 |  | ztf-6 (-0.74) | 25 | 2.13 | 0.0140 |
| MA0461.1 |  | hlh-15 | 16 | 2.70 | 0.0200 |
| CG32105\_Cell\_FBgn0052105 |  | lim-6 | 29 | 1.91 | 0.0200 |
| pTH2933 |  | F58G1.2 (-0.51) | 5 | 8.17 | 0.0290 |
| FOXJ3\_si |  | lin-31 | 65 | 1.30 | 0.0380 |
| pTH5169 |  | cfi-1 | 60 | 1.34 | 0.0420 |
| MA0536.1 |  | elt-1 | 14 | 2.64 | 0.0480 |
| pTH8982 |  | ceh-48 | 29 | 1.79 | 0.0480 |

### Correlated (and anti-correlated) transcription factors

|  |  |
| --- | --- |
| **Transcription factor** | **Correlation** |
| mbf-1 | 0.77 |
| Y56A3A.18 | 0.72 |
| C01F6.9 | 0.72 |
| mxl-1 | 0.71 |
| mxl-2 | 0.67 |
| madf-10 | 0.67 |
| zip-4 | 0.65 |
| ceh-88 | 0.64 |
| nhr-222 | 0.56 |
| ceh-31 | 0.56 |
| dct-13 | 0.54 |
| ccch-3 | 0.53 |
| C09F5.3 | 0.49 |
| T26A5.8 | 0.49 |
| ceh-7 | 0.48 |
| Y53H1A.2 | 0.48 |
| C35D6.4 | 0.48 |
| F21G4.5 | 0.48 |
| sdz-38 | 0.47 |
| madf-2 | 0.47 |
| W02D7.6 | 0.44 |
| repo-1 | 0.43 |
| T18D3.7 | 0.43 |
| sox-3 | 0.42 |
| nhr-78 | 0.42 |
| set-16 | -0.70 |
| slr-2 | -0.70 |
| mcd-1 | -0.71 |
| T20F7.1 | -0.71 |
| Y48C3A.12 | -0.71 |
| tbx-2 | -0.71 |
| ceh-100 | -0.71 |
| nhr-35 | -0.72 |
| gei-8 | -0.73 |
| efl-3 | -0.73 |
| nhr-22 | -0.74 |
| sma-9 | -0.74 |
| ztf-6 | -0.74 |
| F23B12.7 | -0.74 |
| attf-2 | -0.74 |
| Y48G8AL.10 | -0.75 |
| nfx-1 | -0.75 |
| B0336.3 | -0.76 |
| C52E12.1 | -0.76 |
| bed-2 | -0.81 |
| ham-1 | -0.81 |
| die-1 | -0.81 |
| ztf-7 | -0.81 |
| fkh-7 | -0.81 |
| spr-3 | -0.85 |

### ChIP peaks enriched

|  |  |  |  |  |
| --- | --- | --- | --- | --- |
| **Gene** | **Experiment** | **Number of upstream peaks** | **Enrichment** | **FDR corrected p** |
| eor-1 | EOR-1\_Larvae-L3-stage | 39 | 2.25 | 8.1e-06 |
| efl-1 | EFL-1\_Fed-L1-stage-larvae | 35 | 2.42 | 9.0e-06 |
| C34F6.9 | C34F6.9\_Larvae-L2-stage | 37 | 2.19 | 3.8e-05 |
| lin-35 | LIN-35\_Fed-L1-stage-larvae | 33 | 2.33 | 5.8e-05 |
| efl-1 | EFL-1\_Larvae-L1-stage | 36 | 2.17 | 7.6e-05 |
| F45C12.2 | F45C12.2\_Fed-L1-stage-larvae | 32 | 2.32 | 9.8e-05 |
| lsy-2 | LSY-2\_Fed-L1-stage-larvae | 34 | 2.20 | 1.3e-04 |
| nhr-6 | NHR-6\_Larvae-L4-stage | 21 | 3.02 | 2.0e-04 |
| lin-13 | LIN-13\_Larvae-L2-stage | 27 | 2.46 | 2.9e-04 |
| gei-11 | GEI-11\_Fed-L1-stage-larvae | 33 | 2.14 | 3.5e-04 |
| lsy-2 | LSY-2\_Embryos | 27 | 2.40 | 4.3e-04 |
| nhr-23 | NHR-23\_Larvae-L3-stage | 31 | 2.15 | 6.7e-04 |
| pes-1 | PES-1\_Larvae-L4-stage | 34 | 2.03 | 7.1e-04 |
| lsy-2 | LSY-2\_Larvae-L1-stage | 38 | 1.89 | 8.1e-04 |
| ham-1 | HAM-1\_Fed-L1-stage-larvae | 33 | 2.02 | 1.1e-03 |
| C16A3.4 | C16A3.4\_Fed-L1-stage-larvae | 26 | 2.28 | 1.5e-03 |
| gei-11 | GEI-11\_Larvae-L3-stage | 32 | 2.01 | 1.6e-03 |
| ceh-38 | CEH-38\_Larvae-L4-stage | 20 | 2.63 | 2.2e-03 |
| ceh-39 | CEH-39\_Embryos | 18 | 2.82 | 2.2e-03 |
| dpl-1 | DPL-1\_Fed-L1-stage-larvae | 30 | 2.04 | 2.4e-03 |
| nhr-25 | NHR-25\_Larvae-L2-stage | 28 | 2.12 | 2.4e-03 |
| sem-4 | SEM-4\_Larvae-L2-stage | 32 | 1.97 | 2.4e-03 |
| ces-1 | CES-1\_Embryos | 32 | 1.92 | 3.8e-03 |
| ham-1 | HAM-1\_Larvae-L4-stage | 33 | 1.88 | 4.0e-03 |
| dpl-1 | DPL-1\_Larvae-L4-stage | 38 | 1.73 | 5.5e-03 |
| gei-11 | GEI-11\_Larvae-L2-stage | 25 | 2.12 | 6.3e-03 |
| lin-15 | LIN-15B\_Fed-L1-stage-larvae | 20 | 2.41 | 6.5e-03 |
| ama-1 | AMA-1\_Larvae-L3-stage | 16 | 2.77 | 6.9e-03 |
| R02D3.7 | R02D3.7\_Larvae-L3-stage | 32 | 1.85 | 7.1e-03 |
| nfya-1 | NFYA-1\_Larvae-L3-stage | 26 | 2.04 | 8.4e-03 |
| hpl-2 | HPL-2\_Fed-L1-stage-larvae | 35 | 1.73 | 1.1e-02 |
| nhr-77 | NHR-77\_Fed-L1-stage-larvae | 29 | 1.88 | 1.2e-02 |
| nfya-1 | NFYA-1\_Late-Embryos | 28 | 1.91 | 1.2e-02 |
| efl-1 | EFL-1\_Young-adult | 32 | 1.77 | 1.5e-02 |
| ceh-38 | CEH-38\_Larvae-L3-stage | 27 | 1.90 | 1.8e-02 |
| dve-1 | DVE-1\_Larvae-L4-stage | 19 | 2.27 | 1.8e-02 |
| C01B12.2 | C01B12.2\_Larvae-L2-stage | 37 | 1.64 | 1.9e-02 |
| lin-13 | LIN-13\_Larvae-L4-stage | 16 | 2.44 | 2.5e-02 |
| F23B12.7 | F23B12.7\_Young-adult | 20 | 2.14 | 2.5e-02 |
| dpl-1 | DPL-1\_Young-adult | 20 | 2.14 | 2.6e-02 |
| W03F9.2 | W03F9.2\_L4-Young-Adult-stage-larvae | 41 | 1.53 | 3.1e-02 |
| lin-15 | LIN-15B\_Larvae-L4-stage | 11 | 3.01 | 3.4e-02 |
| fos-1 | FOS-1\_Fed-L1-stage-larvae | 27 | 1.81 | 3.5e-02 |
| nhr-237 | NHR-237\_Embryos | 13 | 2.62 | 4.0e-02 |
