# Supplementary material for: Overlapping cell population expression profiling and regulatory inference in C. elegans
Source: BMC Genomics. 2016 Feb 29;17:159. doi: 10.1186/s12864-016-2482-z (PMC4772325; doi:10.1186/s12864-016-2482-z)
Supplement: Additional file 13: — Web supplement. (DOC 21 kb) [file 12864_2016_2482_MOESM13_ESM.zip › sortWeb/clusters/hier.300.clusters/154.html]

Cluster 154 

## Cluster 154

### Expression

| cnd-1 rep. 1 | cnd-1 rep. 2 | cnd-1 rep. 3 | pha-4 rep. 1 | pha-4 rep. 2 | pha-4 rep. 3 | ceh-27 | ceh-36 | ceh-6 | F21D5.9 | mir-57 | mls-2 | pal-1 | pros-1 | ttx-3 | unc-130 | hlh-16 | irx-1 | ceh-6 (+) hlh-16 (+) | ceh-6 (+) hlh-16 (-) | ceh-6 (-) hlh-16 (+) | cnd-1 singlets | pha-4 singlets | 0 | 60 | 120 | 150 | 180 | 240 | 330 | 390 | 420 | 480 | 540 | 570 | 600 | 630 | 660 | NAME | Functional description |
| --- | --- | --- | --- | --- | --- | --- | --- | --- | --- | --- | --- | --- | --- | --- | --- | --- | --- | --- | --- | --- | --- | --- | --- | --- | --- | --- | --- | --- | --- | --- | --- | --- | --- | --- | --- | --- | --- | --- | --- |
|  |  |  |  |  |  |  |  |  |  |  |  |  |  |  |  |  |  |  |  |  |  |  |  |  |  |  |  |  |  |  |  |  |  |  |  |  |  | F48C1.6 |  |
|  |  |  |  |  |  |  |  |  |  |  |  |  |  |  |  |  |  |  |  |  |  |  |  |  |  |  |  |  |  |  |  |  |  |  |  |  |  | F01F1.2 |  |
|  |  |  |  |  |  |  |  |  |  |  |  |  |  |  |  |  |  |  |  |  |  |  |  |  |  |  |  |  |  |  |  |  |  |  |  |  |  | *ubc-7* | UBiquitin Conjugating enzyme |
|  |  |  |  |  |  |  |  |  |  |  |  |  |  |  |  |  |  |  |  |  |  |  |  |  |  |  |  |  |  |  |  |  |  |  |  |  |  | *mtp-18* | MiTochondrial Protein |
|  |  |  |  |  |  |  |  |  |  |  |  |  |  |  |  |  |  |  |  |  |  |  |  |  |  |  |  |  |  |  |  |  |  |  |  |  |  | *icd-1* | Inhibitor of Cell Death |
|  |  |  |  |  |  |  |  |  |  |  |  |  |  |  |  |  |  |  |  |  |  |  |  |  |  |  |  |  |  |  |  |  |  |  |  |  |  | *cyc-2.1* | CYtochrome C |
|  |  |  |  |  |  |  |  |  |  |  |  |  |  |  |  |  |  |  |  |  |  |  |  |  |  |  |  |  |  |  |  |  |  |  |  |  |  | T26A5.8 |  |
|  |  |  |  |  |  |  |  |  |  |  |  |  |  |  |  |  |  |  |  |  |  |  |  |  |  |  |  |  |  |  |  |  |  |  |  |  |  | *tin-9.1* | Transport to INner mitochondrial membrane (yeast TIM) |
|  |  |  |  |  |  |  |  |  |  |  |  |  |  |  |  |  |  |  |  |  |  |  |  |  |  |  |  |  |  |  |  |  |  |  |  |  |  | F16D3.8 |  |
|  |  |  |  |  |  |  |  |  |  |  |  |  |  |  |  |  |  |  |  |  |  |  |  |  |  |  |  |  |  |  |  |  |  |  |  |  |  | *cyn-7* | CYclophyliN |
|  |  |  |  |  |  |  |  |  |  |  |  |  |  |  |  |  |  |  |  |  |  |  |  |  |  |  |  |  |  |  |  |  |  |  |  |  |  | *ndk-1* | Nucleoside Diphosphate Kinase |
|  |  |  |  |  |  |  |  |  |  |  |  |  |  |  |  |  |  |  |  |  |  |  |  |  |  |  |  |  |  |  |  |  |  |  |  |  |  | *sre-13* | Serpentine Receptor, class E (epsilon) |
|  |  |  |  |  |  |  |  |  |  |  |  |  |  |  |  |  |  |  |  |  |  |  |  |  |  |  |  |  |  |  |  |  |  |  |  |  |  | C38C6.8 |  |
|  |  |  |  |  |  |  |  |  |  |  |  |  |  |  |  |  |  |  |  |  |  |  |  |  |  |  |  |  |  |  |  |  |  |  |  |  |  | C18E9.4 |  |
|  |  |  |  |  |  |  |  |  |  |  |  |  |  |  |  |  |  |  |  |  |  |  |  |  |  |  |  |  |  |  |  |  |  |  |  |  |  | Y45F10D.4 |  |
|  |  |  |  |  |  |  |  |  |  |  |  |  |  |  |  |  |  |  |  |  |  |  |  |  |  |  |  |  |  |  |  |  |  |  |  |  |  | *ran-4* | associated with RAN (nuclear import/export) function |
|  |  |  |  |  |  |  |  |  |  |  |  |  |  |  |  |  |  |  |  |  |  |  |  |  |  |  |  |  |  |  |  |  |  |  |  |  |  | *har-1* | HemiAsterlin Resistant |
|  |  |  |  |  |  |  |  |  |  |  |  |  |  |  |  |  |  |  |  |  |  |  |  |  |  |  |  |  |  |  |  |  |  |  |  |  |  | ZK673.2 |  |
|  |  |  |  |  |  |  |  |  |  |  |  |  |  |  |  |  |  |  |  |  |  |  |  |  |  |  |  |  |  |  |  |  |  |  |  |  |  | *mbf-1* | MBF (multiprotein bridging factor) transcriptional coactivator |
|  |  |  |  |  |  |  |  |  |  |  |  |  |  |  |  |  |  |  |  |  |  |  |  |  |  |  |  |  |  |  |  |  |  |  |  |  |  | C50B8.1 |  |
|  |  |  |  |  |  |  |  |  |  |  |  |  |  |  |  |  |  |  |  |  |  |  |  |  |  |  |  |  |  |  |  |  |  |  |  |  |  | D2005.3 |  |
|  |  |  |  |  |  |  |  |  |  |  |  |  |  |  |  |  |  |  |  |  |  |  |  |  |  |  |  |  |  |  |  |  |  |  |  |  |  | *mev-1* | abnormal MEthyl Viologen sensitivity |
|  |  |  |  |  |  |  |  |  |  |  |  |  |  |  |  |  |  |  |  |  |  |  |  |  |  |  |  |  |  |  |  |  |  |  |  |  |  | *tag-281* | Temporarily Assigned Gene name |
|  |  |  |  |  |  |  |  |  |  |  |  |  |  |  |  |  |  |  |  |  |  |  |  |  |  |  |  |  |  |  |  |  |  |  |  |  |  | Y60A3A.19 |  |
|  |  |  |  |  |  |  |  |  |  |  |  |  |  |  |  |  |  |  |  |  |  |  |  |  |  |  |  |  |  |  |  |  |  |  |  |  |  | *arf-3* | ADP-Ribosylation Factor related |
|  |  |  |  |  |  |  |  |  |  |  |  |  |  |  |  |  |  |  |  |  |  |  |  |  |  |  |  |  |  |  |  |  |  |  |  |  |  | *vem-1* | VEMA (mammalian ventral midline antigen) related |
|  |  |  |  |  |  |  |  |  |  |  |  |  |  |  |  |  |  |  |  |  |  |  |  |  |  |  |  |  |  |  |  |  |  |  |  |  |  | Y39A1A.20 |  |
|  |  |  |  |  |  |  |  |  |  |  |  |  |  |  |  |  |  |  |  |  |  |  |  |  |  |  |  |  |  |  |  |  |  |  |  |  |  | *rmo-1* |  |
|  |  |  |  |  |  |  |  |  |  |  |  |  |  |  |  |  |  |  |  |  |  |  |  |  |  |  |  |  |  |  |  |  |  |  |  |  |  | *nbet-1* | Nematode homolog of yeast BET1 (Blocked Early in Transport) |
|  |  |  |  |  |  |  |  |  |  |  |  |  |  |  |  |  |  |  |  |  |  |  |  |  |  |  |  |  |  |  |  |  |  |  |  |  |  | Y110A7A.2 |  |
|  |  |  |  |  |  |  |  |  |  |  |  |  |  |  |  |  |  |  |  |  |  |  |  |  |  |  |  |  |  |  |  |  |  |  |  |  |  | R10E11.12 |  |
|  |  |  |  |  |  |  |  |  |  |  |  |  |  |  |  |  |  |  |  |  |  |  |  |  |  |  |  |  |  |  |  |  |  |  |  |  |  | ZK858.2 |  |
|  |  |  |  |  |  |  |  |  |  |  |  |  |  |  |  |  |  |  |  |  |  |  |  |  |  |  |  |  |  |  |  |  |  |  |  |  |  | K08H10.6 |  |
|  |  |  |  |  |  |  |  |  |  |  |  |  |  |  |  |  |  |  |  |  |  |  |  |  |  |  |  |  |  |  |  |  |  |  |  |  |  | F26D11.12 |  |

### Phenotypes enriched

none found

### Anatomy terms enriched

none found

### GO terms enriched

|  |  |  |
| --- | --- | --- |
| **GO term** | **Number of genes** | **FDR-corrected p-value** |
| mitochondrion | 7 | 0.00007 |
| electron transport chain | 3 | 0.00090 |
| envelope | 5 | 0.00190 |
| respiratory chain | 3 | 0.00200 |
| mitochondrial inner membrane | 4 | 0.00200 |
| mitochondrial envelope | 4 | 0.00370 |

### Expression clusters enriched

|  |  |  |  |
| --- | --- | --- | --- |
| **Group name** | **Number in cluster** | **Enrichment** | **FDR corrected p** |
| The cluster contains genes that are significantly enriched in L1 muscle. | 15 | 4.79 | 4.26e-05 |
| Genes up or down regulated by 10e-09M of cholesterol . The normalized values used were G/R ratio > 2.6 for up-regulation and G/R ratio < 0.38 for down-regulation, which corresponds to 1.39 and -1.39 log(base2) G/R ratio, respectively. | 15 | 3.86 | 5.78e-04 |
| Genes in the top 10% of expression level across the triplicate L3 samples. To generate the top10 and bottom10 gene sets, authors ranked all genes by mean expression array signal intensity across the three replicates, then took the top and bottom deciles (1,841 genes each) to represent genes with high and low expression. | 15 | 3.76 | 7.88e-04 |
| Maternal degradation class (MD): genes that are the subset of maternal genes that decrease without first increasing in abundance. | 14 | 3.70 | 2.19e-03 |
| Caenorhabditis elegans Genes with expression levels changed significantly after treatment of Bacillus thurigiensis DB27. | 22 | 2.12 | 1.07e-02 |
| C-lineage related expression profile. WBPaper00025032:cluster\_177 | 2 | 102.50 | 3.68e-02 |
| Early embryonic development gene expression profile. [cgc5767]:cluster\_1 | 7 | 5.75 | 3.80e-02 |

### Motifs enriched

|  |  |  |  |  |  |
| --- | --- | --- | --- | --- | --- |
| **Motif** | **Logo** | **Possible orthologs** | **Number of motifs in cluster** | **Enrichment** | **FDR corrected p** |
| pTH10030 |  | xbp-1 | 13 | 3.81 | 0.0020 |
| pTH5057 |  | aha-1 (-0.79) hlh-30 | 12 | 3.62 | 0.0062 |
| MA0486.1 |  | Y53C10A.3 | 23 | 2.01 | 0.0065 |
| MA0069.1 |  | pax-3 | 22 | 2.07 | 0.0069 |
| pTH9097 |  | Y116A8C.22 | 31 | 1.51 | 0.0081 |
| Ara\_Cell\_FBgn0015904 |  | irx-1 | 13 | 3.00 | 0.0150 |
| pTH9709 |  | die-1 (-0.75) | 14 | 2.76 | 0.0170 |
| pTH10837 |  | T22H9.4 | 26 | 1.68 | 0.0200 |
| HIF1A\_si |  | hif-1 | 17 | 2.31 | 0.0200 |
| I$DRI\_01 |  | cfi-1 | 24 | 1.78 | 0.0210 |
| pTH3220 |  | Y5F2A.4 | 17 | 2.29 | 0.0230 |
| pTH1739 |  | nhr-255 | 9 | 3.97 | 0.0240 |
| pTH5065 |  | hlh-30 hlh-26 | 11 | 3.26 | 0.0240 |
| MA0042.1 |  | lin-31 | 28 | 1.54 | 0.0320 |
| ARNT2\_si |  | aha-1 (-0.79) | 5 | 7.47 | 0.0350 |
| pTH8679 |  | pax-2 | 7 | 4.76 | 0.0370 |
| SMAD3\_1 |  | daf-8 (-0.62) | 9 | 3.59 | 0.0400 |
| Eip93F\_SANGER\_10\_FBgn0013948 |  | mbr-1 | 22 | 1.77 | 0.0480 |
| V$PAX2\_02 |  | pax-1 | 7 | 4.48 | 0.0480 |
| MA0049.1 |  | hbl-1 (-0.63) | 29 | 1.46 | 0.0480 |

### Correlated (and anti-correlated) transcription factors

|  |  |
| --- | --- |
| **Transcription factor** | **Correlation** |
| mbf-1 | 0.82 |
| Y56A3A.18 | 0.81 |
| mxl-2 | 0.75 |
| T26A5.8 | 0.70 |
| C01F6.9 | 0.56 |
| nhr-222 | 0.55 |
| mxl-3 | 0.54 |
| mxl-1 | 0.53 |
| nhr-135 | 0.53 |
| sdz-38 | 0.50 |
| nhr-286 | 0.48 |
| dct-13 | 0.45 |
| nhr-205 | 0.45 |
| hlh-12 | 0.45 |
| ceh-7 | 0.43 |
| nhr-92 | 0.43 |
| madf-10 | 0.42 |
| unc-55 | 0.41 |
| ceh-31 | 0.41 |
| nhr-87 | 0.41 |
| nhr-149 | 0.41 |
| ceh-88 | 0.40 |
| nhr-41 | 0.38 |
| C35D6.4 | 0.38 |
| nhr-268 | 0.37 |
| cdc-14 | -0.76 |
| F57A8.1 | -0.76 |
| D1081.8 | -0.76 |
| daf-16 | -0.77 |
| gei-8 | -0.77 |
| C04F5.9 | -0.77 |
| B0250.4 | -0.77 |
| athp-1 | -0.79 |
| egl-27 | -0.79 |
| aha-1 | -0.79 |
| F21A10.2 | -0.79 |
| T20F7.1 | -0.79 |
| attf-2 | -0.80 |
| F39B2.1 | -0.80 |
| B0261.1 | -0.82 |
| hmp-2 | -0.82 |
| ztf-6 | -0.82 |
| ceh-100 | -0.82 |
| atg-4.1 | -0.83 |
| Y48C3A.12 | -0.83 |
| chd-7 | -0.85 |
| swsn-7 | -0.85 |
| Y48G8AL.10 | -0.87 |
| set-16 | -0.87 |
| B0336.3 | -0.89 |

### ChIP peaks enriched

|  |  |  |  |  |
| --- | --- | --- | --- | --- |
| **Gene** | **Experiment** | **Number of upstream peaks** | **Enrichment** | **FDR corrected p** |
| efl-1 | EFL-1\_Larvae-L1-stage | 22 | 4.46 | 3.6e-09 |
| efl-1 | EFL-1\_Fed-L1-stage-larvae | 21 | 4.68 | 5.9e-09 |
| dpl-1 | DPL-1\_Fed-L1-stage-larvae | 22 | 3.78 | 8.0e-08 |
| efl-1 | EFL-1\_Young-adult | 20 | 4.21 | 1.4e-07 |
| dpl-1 | DPL-1\_Young-adult | 19 | 4.44 | 1.9e-07 |
| eor-1 | EOR-1\_Larvae-L3-stage | 20 | 3.67 | 1.3e-06 |
| F45C12.2 | F45C12.2\_Fed-L1-stage-larvae | 20 | 3.66 | 1.4e-06 |
| nhr-129 | NHR-129\_Larvae-L2-stage | 22 | 3.11 | 2.8e-06 |
| F16B12.6 | F16B12.6\_Fed-L1-stage-larvae | 13 | 6.02 | 5.0e-06 |
| pes-1 | PES-1\_Larvae-L4-stage | 21 | 3.17 | 5.7e-06 |
| C34F6.9 | C34F6.9\_Larvae-L2-stage | 21 | 3.15 | 6.2e-06 |
| ceh-38 | CEH-38\_Larvae-L3-stage | 17 | 3.98 | 9.0e-06 |
| R02D3.7 | R02D3.7\_Larvae-L3-stage | 21 | 3.08 | 9.3e-06 |
| nhr-6 | NHR-6\_Larvae-L4-stage | 14 | 5.10 | 1.0e-05 |
| hpl-2 | HPL-2\_Fed-L1-stage-larvae | 20 | 3.24 | 1.0e-05 |
| nfya-1 | NFYA-1\_Larvae-L3-stage | 17 | 3.91 | 1.1e-05 |
| lin-35 | LIN-35\_Fed-L1-stage-larvae | 19 | 3.39 | 1.3e-05 |
| ham-1 | HAM-1\_Fed-L1-stage-larvae | 18 | 3.60 | 1.4e-05 |
| aly-2 | ALY-2\_Fed-L1-stage-larvae | 18 | 3.52 | 1.9e-05 |
| lsy-2 | LSY-2\_Larvae-L2-stage | 14 | 4.77 | 2.1e-05 |
| dpl-1 | DPL-1\_Larvae-L4-stage | 20 | 3.09 | 2.2e-05 |
| W03F9.2 | W03F9.2\_L4-Young-Adult-stage-larvae | 21 | 2.91 | 2.4e-05 |
| gei-11 | GEI-11\_Larvae-L3-stage | 17 | 3.68 | 2.7e-05 |
| gei-11 | GEI-11\_Young-adult | 12 | 5.72 | 2.9e-05 |
| lsy-2 | LSY-2\_Embryos | 14 | 4.32 | 6.6e-05 |
| gei-11 | GEI-11\_Fed-L1-stage-larvae | 16 | 3.68 | 6.7e-05 |
| lin-15 | LIN-15B\_Fed-L1-stage-larvae | 15 | 3.93 | 7.5e-05 |
| lin-13 | LIN-13\_Larvae-L2-stage | 14 | 4.22 | 8.6e-05 |
| C16A3.4 | C16A3.4\_Fed-L1-stage-larvae | 16 | 3.55 | 1.1e-04 |
| lsy-2 | LSY-2\_Larvae-L1-stage | 21 | 2.64 | 1.1e-04 |
| nhr-237 | NHR-237\_Larvae-L1-stage | 9 | 7.41 | 1.2e-04 |
| C01B12.2 | C01B12.2\_Larvae-L2-stage | 22 | 2.47 | 1.6e-04 |
| pha-4 | PHA-4\_Larvae-L2-stage | 20 | 2.70 | 1.8e-04 |
| jun-1 | JUN-1\_Larvae-L1-stage | 16 | 3.34 | 2.3e-04 |
| lin-13 | LIN-13\_Larvae-L4-stage | 12 | 4.63 | 2.3e-04 |
| nhr-76 | NHR-76\_Larvae-L4-stage | 12 | 4.56 | 2.7e-04 |
| jun-1 | JUN-1\_Larvae-L3-stage | 14 | 3.76 | 3.1e-04 |
| F23B12.7 | F23B12.7\_Young-adult | 12 | 4.40 | 3.8e-04 |
| F45C12.2 | F45C12.2\_Larvae-L2-stage | 9 | 6.16 | 5.0e-04 |
| gei-11 | GEI-11\_Larvae-L2-stage | 13 | 3.86 | 5.6e-04 |
| nhr-77 | NHR-77\_Larvae-L4-stage | 22 | 2.28 | 6.0e-04 |
| F45C12.2 | F45C12.2\_Larvae-L3-stage | 10 | 5.15 | 6.7e-04 |
| lin-35 | LIN-35\_Young-adult | 15 | 3.21 | 8.1e-04 |
| nhr-77 | NHR-77\_Larvae-L2-stage | 12 | 4.04 | 8.4e-04 |
| lin-13 | LIN-13\_Larvae-L1-stage | 8 | 6.69 | 9.1e-04 |
| ztf-4 | ZTF-4\_Larvae-L1-stage | 7 | 8.19 | 9.1e-04 |
| ama-1 | AMA-1\_Larvae-L3-stage | 11 | 4.40 | 9.5e-04 |
| nhr-77 | NHR-77\_Fed-L1-stage-larvae | 17 | 2.79 | 9.9e-04 |
| ztf-4 | ZTF-4\_Larvae-L2-stage | 9 | 5.60 | 1.0e-03 |
| lsy-2 | LSY-2\_Fed-L1-stage-larvae | 17 | 2.78 | 1.0e-03 |
| ham-1 | HAM-1\_Larvae-L4-stage | 18 | 2.60 | 1.3e-03 |
| R02D3.7 | R02D3.7\_Larvae-L2-stage | 10 | 4.74 | 1.3e-03 |
| unc-62 | UNC-62\_Fed-L1-stage-larvae | 8 | 6.28 | 1.4e-03 |
| lin-35 | LIN-35\_Starved-L1-stage-larvae | 9 | 5.23 | 1.7e-03 |
| sax-3 | SAX-3\_Larvae-L2-stage | 14 | 3.18 | 1.8e-03 |
| nhr-23 | NHR-23\_Larvae-L3-stage | 16 | 2.80 | 1.9e-03 |
| sem-4 | SEM-4\_Larvae-L2-stage | 17 | 2.64 | 2.0e-03 |
| fos-1 | FOS-1\_Fed-L1-stage-larvae | 14 | 3.14 | 2.1e-03 |
| jun-1 | JUN-1\_Larvae-L4-stage | 13 | 3.37 | 2.1e-03 |
| nfya-1 | NFYA-1\_Late-Embryos | 16 | 2.76 | 2.2e-03 |
| elt-1 | ELT-1\_Larvae-L3-stage | 9 | 5.01 | 2.3e-03 |
| nhr-21 | NHR-21\_Larvae-L2-stage | 8 | 5.74 | 2.4e-03 |
| pha-4 | PHA-4\_Larvae-L4-stage | 14 | 3.09 | 2.5e-03 |
| ces-1 | CES-1\_Embryos | 17 | 2.58 | 2.6e-03 |
| nhr-25 | NHR-25\_Larvae-L2-stage | 15 | 2.87 | 2.7e-03 |
| lin-15 | LIN-15B\_Larvae-L4-stage | 7 | 6.77 | 2.7e-03 |
| sax-3 | SAX-3\_Larvae-L3-stage | 9 | 4.87 | 2.7e-03 |
| nhr-237 | NHR-237\_Embryos | 9 | 4.60 | 4.1e-03 |
| nhr-76 | NHR-76\_Larvae-L3-stage | 10 | 4.04 | 4.4e-03 |
| dve-1 | DVE-1\_Larvae-L4-stage | 13 | 3.10 | 4.7e-03 |
| mab-5 | MAB-5\_Larvae-L2-stage | 9 | 4.41 | 5.4e-03 |
| hlh-30 | HLH-30\_Late-Embryos | 9 | 4.39 | 5.7e-03 |
| fos-1 | FOS-1\_Larvae-L4-stage | 8 | 5.01 | 5.8e-03 |
| nhr-77 | NHR-77\_Larvae-L3-stage | 11 | 3.48 | 6.7e-03 |
| aha-1 | AHA-1\_Fed-L1-stage-larvae | 5 | 9.28 | 7.4e-03 |
| nhr-6 | NHR-6\_Larvae-L2-stage | 13 | 2.92 | 8.2e-03 |
| unc-62 | UNC-62\_Day-Four-Young-Adult | 12 | 3.13 | 8.3e-03 |
| unc-62 | UNC-62\_Young-adult-Day-4 | 12 | 3.13 | 8.3e-03 |
| hlh-30 | HLH-30\_Larvae-L4-stage | 11 | 3.38 | 8.4e-03 |
| nfya-1 | NFYA-1\_Young-adult | 7 | 5.54 | 8.4e-03 |
| unc-62 | UNC-62\_Larvae-L3-stage | 11 | 3.36 | 8.8e-03 |
| pha-4 | PHA-4\_Young-adult | 9 | 4.00 | 1.1e-02 |
| sax-3 | SAX-3\_Larvae-L4-stage | 17 | 2.28 | 1.1e-02 |
| fos-1 | FOS-1\_Larvae-L2-stage | 18 | 2.17 | 1.2e-02 |
| nhr-10 | NHR-10\_Larvae-L4-stage | 7 | 5.12 | 1.3e-02 |
| aly-2 | ALY-2\_Larvae-L2-stage | 6 | 5.98 | 1.6e-02 |
| pax-1 | PAX-1\_Embryos | 7 | 4.77 | 1.9e-02 |
| ztf-7 | ZTF-7\_Larvae-L4-stage | 11 | 3.02 | 2.0e-02 |
| aly-2 | ALY-2\_Larvae-L3-stage | 8 | 4.04 | 2.1e-02 |
| nhr-237 | NHR-237\_Larvae-L2-stage | 4 | 10.04 | 2.3e-02 |
| egl-5 | EGL-5\_Larvae-L3-stage | 12 | 2.75 | 2.4e-02 |
| fos-1 | FOS-1\_Larvae-L3-stage | 10 | 3.16 | 2.6e-02 |
| R02D3.7 | R02D3.7\_Larvae-L4-stage | 9 | 3.45 | 2.8e-02 |
| peb-1 | PEB-1\_Larvae-L2-stage | 6 | 5.19 | 3.0e-02 |
| zag-1 | ZAG-1\_Larvae-L3-stage | 7 | 4.27 | 3.4e-02 |
| nhr-11 | NHR-11\_Larvae-L2-stage | 8 | 3.71 | 3.5e-02 |
| zag-1 | ZAG-1\_Larvae-L2-stage | 11 | 2.81 | 3.5e-02 |
| nhr-28 | NHR-28\_Larvae-L3-stage | 7 | 4.18 | 3.8e-02 |
