# Supplementary material for: Overlapping cell population expression profiling and regulatory inference in C. elegans
Source: BMC Genomics. 2016 Feb 29;17:159. doi: 10.1186/s12864-016-2482-z (PMC4772325; doi:10.1186/s12864-016-2482-z)
Supplement: Additional file 13: — Web supplement. (DOC 21 kb) [file 12864_2016_2482_MOESM13_ESM.zip › sortWeb/clusters/hier.300.clusters/155.html]

Cluster 155 

## Cluster 155

### Expression

| cnd-1 rep. 1 | cnd-1 rep. 2 | cnd-1 rep. 3 | pha-4 rep. 1 | pha-4 rep. 2 | pha-4 rep. 3 | ceh-27 | ceh-36 | ceh-6 | F21D5.9 | mir-57 | mls-2 | pal-1 | pros-1 | ttx-3 | unc-130 | hlh-16 | irx-1 | ceh-6 (+) hlh-16 (+) | ceh-6 (+) hlh-16 (-) | ceh-6 (-) hlh-16 (+) | cnd-1 singlets | pha-4 singlets | 0 | 60 | 120 | 150 | 180 | 240 | 330 | 390 | 420 | 480 | 540 | 570 | 600 | 630 | 660 | NAME | Functional description |
| --- | --- | --- | --- | --- | --- | --- | --- | --- | --- | --- | --- | --- | --- | --- | --- | --- | --- | --- | --- | --- | --- | --- | --- | --- | --- | --- | --- | --- | --- | --- | --- | --- | --- | --- | --- | --- | --- | --- | --- |
|  |  |  |  |  |  |  |  |  |  |  |  |  |  |  |  |  |  |  |  |  |  |  |  |  |  |  |  |  |  |  |  |  |  |  |  |  |  | C46H11.6 |  |
|  |  |  |  |  |  |  |  |  |  |  |  |  |  |  |  |  |  |  |  |  |  |  |  |  |  |  |  |  |  |  |  |  |  |  |  |  |  | *nsy-7* | Neuronal SYmmetry |
|  |  |  |  |  |  |  |  |  |  |  |  |  |  |  |  |  |  |  |  |  |  |  |  |  |  |  |  |  |  |  |  |  |  |  |  |  |  | C12D12.3 |  |
|  |  |  |  |  |  |  |  |  |  |  |  |  |  |  |  |  |  |  |  |  |  |  |  |  |  |  |  |  |  |  |  |  |  |  |  |  |  | *nhr-87* | Nuclear Hormone Receptor family |
|  |  |  |  |  |  |  |  |  |  |  |  |  |  |  |  |  |  |  |  |  |  |  |  |  |  |  |  |  |  |  |  |  |  |  |  |  |  | ZK856.7 |  |
|  |  |  |  |  |  |  |  |  |  |  |  |  |  |  |  |  |  |  |  |  |  |  |  |  |  |  |  |  |  |  |  |  |  |  |  |  |  | E01G4.3 |  |
|  |  |  |  |  |  |  |  |  |  |  |  |  |  |  |  |  |  |  |  |  |  |  |  |  |  |  |  |  |  |  |  |  |  |  |  |  |  | C48B6.9 |  |
|  |  |  |  |  |  |  |  |  |  |  |  |  |  |  |  |  |  |  |  |  |  |  |  |  |  |  |  |  |  |  |  |  |  |  |  |  |  | R05D7.1 |  |
|  |  |  |  |  |  |  |  |  |  |  |  |  |  |  |  |  |  |  |  |  |  |  |  |  |  |  |  |  |  |  |  |  |  |  |  |  |  | K08E4.2 |  |
|  |  |  |  |  |  |  |  |  |  |  |  |  |  |  |  |  |  |  |  |  |  |  |  |  |  |  |  |  |  |  |  |  |  |  |  |  |  | *srw-67* | Serpentine Receptor, class W |
|  |  |  |  |  |  |  |  |  |  |  |  |  |  |  |  |  |  |  |  |  |  |  |  |  |  |  |  |  |  |  |  |  |  |  |  |  |  | *asns-2* | ASparagiNe Synthetase |
|  |  |  |  |  |  |  |  |  |  |  |  |  |  |  |  |  |  |  |  |  |  |  |  |  |  |  |  |  |  |  |  |  |  |  |  |  |  | *srw-62* | Serpentine Receptor, class W |
|  |  |  |  |  |  |  |  |  |  |  |  |  |  |  |  |  |  |  |  |  |  |  |  |  |  |  |  |  |  |  |  |  |  |  |  |  |  | *srw-108* | Serpentine Receptor, class W |
|  |  |  |  |  |  |  |  |  |  |  |  |  |  |  |  |  |  |  |  |  |  |  |  |  |  |  |  |  |  |  |  |  |  |  |  |  |  | Y38C1AB.1 |  |
|  |  |  |  |  |  |  |  |  |  |  |  |  |  |  |  |  |  |  |  |  |  |  |  |  |  |  |  |  |  |  |  |  |  |  |  |  |  | T04C12.23 |  |
|  |  |  |  |  |  |  |  |  |  |  |  |  |  |  |  |  |  |  |  |  |  |  |  |  |  |  |  |  |  |  |  |  |  |  |  |  |  | *nhr-90* | Nuclear Hormone Receptor family |
|  |  |  |  |  |  |  |  |  |  |  |  |  |  |  |  |  |  |  |  |  |  |  |  |  |  |  |  |  |  |  |  |  |  |  |  |  |  | T26F2.3 |  |
|  |  |  |  |  |  |  |  |  |  |  |  |  |  |  |  |  |  |  |  |  |  |  |  |  |  |  |  |  |  |  |  |  |  |  |  |  |  | T21H3.7 |  |
|  |  |  |  |  |  |  |  |  |  |  |  |  |  |  |  |  |  |  |  |  |  |  |  |  |  |  |  |  |  |  |  |  |  |  |  |  |  | K10B3.1 |  |
|  |  |  |  |  |  |  |  |  |  |  |  |  |  |  |  |  |  |  |  |  |  |  |  |  |  |  |  |  |  |  |  |  |  |  |  |  |  | C50A2.3 |  |
|  |  |  |  |  |  |  |  |  |  |  |  |  |  |  |  |  |  |  |  |  |  |  |  |  |  |  |  |  |  |  |  |  |  |  |  |  |  | C32H11.3 |  |
|  |  |  |  |  |  |  |  |  |  |  |  |  |  |  |  |  |  |  |  |  |  |  |  |  |  |  |  |  |  |  |  |  |  |  |  |  |  | F59A3.7 |  |
|  |  |  |  |  |  |  |  |  |  |  |  |  |  |  |  |  |  |  |  |  |  |  |  |  |  |  |  |  |  |  |  |  |  |  |  |  |  | F40H3.3 |  |
|  |  |  |  |  |  |  |  |  |  |  |  |  |  |  |  |  |  |  |  |  |  |  |  |  |  |  |  |  |  |  |  |  |  |  |  |  |  | F56H11.2 |  |
|  |  |  |  |  |  |  |  |  |  |  |  |  |  |  |  |  |  |  |  |  |  |  |  |  |  |  |  |  |  |  |  |  |  |  |  |  |  | C01A2.2 |  |
|  |  |  |  |  |  |  |  |  |  |  |  |  |  |  |  |  |  |  |  |  |  |  |  |  |  |  |  |  |  |  |  |  |  |  |  |  |  | C48E7.1 |  |
|  |  |  |  |  |  |  |  |  |  |  |  |  |  |  |  |  |  |  |  |  |  |  |  |  |  |  |  |  |  |  |  |  |  |  |  |  |  | *egg-6* | EGG sterile (unfertilizable) |
|  |  |  |  |  |  |  |  |  |  |  |  |  |  |  |  |  |  |  |  |  |  |  |  |  |  |  |  |  |  |  |  |  |  |  |  |  |  | F37C4.4 |  |
|  |  |  |  |  |  |  |  |  |  |  |  |  |  |  |  |  |  |  |  |  |  |  |  |  |  |  |  |  |  |  |  |  |  |  |  |  |  | C18D11.1 |  |
|  |  |  |  |  |  |  |  |  |  |  |  |  |  |  |  |  |  |  |  |  |  |  |  |  |  |  |  |  |  |  |  |  |  |  |  |  |  | F20G2.3 |  |
|  |  |  |  |  |  |  |  |  |  |  |  |  |  |  |  |  |  |  |  |  |  |  |  |  |  |  |  |  |  |  |  |  |  |  |  |  |  | *lpr-5* | LiPocalin-Related protein |
|  |  |  |  |  |  |  |  |  |  |  |  |  |  |  |  |  |  |  |  |  |  |  |  |  |  |  |  |  |  |  |  |  |  |  |  |  |  | ZK669.5 |  |
|  |  |  |  |  |  |  |  |  |  |  |  |  |  |  |  |  |  |  |  |  |  |  |  |  |  |  |  |  |  |  |  |  |  |  |  |  |  | C07E3.10 |  |
|  |  |  |  |  |  |  |  |  |  |  |  |  |  |  |  |  |  |  |  |  |  |  |  |  |  |  |  |  |  |  |  |  |  |  |  |  |  | *paqr-3* | Progestin and AdipoQ Receptor family |
|  |  |  |  |  |  |  |  |  |  |  |  |  |  |  |  |  |  |  |  |  |  |  |  |  |  |  |  |  |  |  |  |  |  |  |  |  |  | *mdh-2* | Malate DeHydrogenase |
|  |  |  |  |  |  |  |  |  |  |  |  |  |  |  |  |  |  |  |  |  |  |  |  |  |  |  |  |  |  |  |  |  |  |  |  |  |  | F52H3.5 |  |
|  |  |  |  |  |  |  |  |  |  |  |  |  |  |  |  |  |  |  |  |  |  |  |  |  |  |  |  |  |  |  |  |  |  |  |  |  |  | *nhr-5* | Nuclear Hormone Receptor family |
|  |  |  |  |  |  |  |  |  |  |  |  |  |  |  |  |  |  |  |  |  |  |  |  |  |  |  |  |  |  |  |  |  |  |  |  |  |  | F56C11.6 |  |
|  |  |  |  |  |  |  |  |  |  |  |  |  |  |  |  |  |  |  |  |  |  |  |  |  |  |  |  |  |  |  |  |  |  |  |  |  |  | R05D7.5 |  |
|  |  |  |  |  |  |  |  |  |  |  |  |  |  |  |  |  |  |  |  |  |  |  |  |  |  |  |  |  |  |  |  |  |  |  |  |  |  | *ttr-2* | TransThyretin-Related family domain |
|  |  |  |  |  |  |  |  |  |  |  |  |  |  |  |  |  |  |  |  |  |  |  |  |  |  |  |  |  |  |  |  |  |  |  |  |  |  | *mpk-2* | MAP Kinase |
|  |  |  |  |  |  |  |  |  |  |  |  |  |  |  |  |  |  |  |  |  |  |  |  |  |  |  |  |  |  |  |  |  |  |  |  |  |  | ZK1055.4 |  |
|  |  |  |  |  |  |  |  |  |  |  |  |  |  |  |  |  |  |  |  |  |  |  |  |  |  |  |  |  |  |  |  |  |  |  |  |  |  | Y51A2D.18 |  |
|  |  |  |  |  |  |  |  |  |  |  |  |  |  |  |  |  |  |  |  |  |  |  |  |  |  |  |  |  |  |  |  |  |  |  |  |  |  | *nhr-149* | Nuclear Hormone Receptor family |
|  |  |  |  |  |  |  |  |  |  |  |  |  |  |  |  |  |  |  |  |  |  |  |  |  |  |  |  |  |  |  |  |  |  |  |  |  |  | R07B1.13 |  |
|  |  |  |  |  |  |  |  |  |  |  |  |  |  |  |  |  |  |  |  |  |  |  |  |  |  |  |  |  |  |  |  |  |  |  |  |  |  | T19B10.2 |  |
|  |  |  |  |  |  |  |  |  |  |  |  |  |  |  |  |  |  |  |  |  |  |  |  |  |  |  |  |  |  |  |  |  |  |  |  |  |  | F17H10.2 |  |
|  |  |  |  |  |  |  |  |  |  |  |  |  |  |  |  |  |  |  |  |  |  |  |  |  |  |  |  |  |  |  |  |  |  |  |  |  |  | R01H10.4 |  |
|  |  |  |  |  |  |  |  |  |  |  |  |  |  |  |  |  |  |  |  |  |  |  |  |  |  |  |  |  |  |  |  |  |  |  |  |  |  | F55A11.6 |  |
|  |  |  |  |  |  |  |  |  |  |  |  |  |  |  |  |  |  |  |  |  |  |  |  |  |  |  |  |  |  |  |  |  |  |  |  |  |  | F55A11.11 |  |
|  |  |  |  |  |  |  |  |  |  |  |  |  |  |  |  |  |  |  |  |  |  |  |  |  |  |  |  |  |  |  |  |  |  |  |  |  |  | F40A3.3 |  |
|  |  |  |  |  |  |  |  |  |  |  |  |  |  |  |  |  |  |  |  |  |  |  |  |  |  |  |  |  |  |  |  |  |  |  |  |  |  | *lon-8* | LONg |

### Phenotypes enriched

none found

### Anatomy terms enriched

none found

### GO terms enriched

none found

### Expression clusters enriched

|  |  |  |  |
| --- | --- | --- | --- |
| **Group name** | **Number in cluster** | **Enrichment** | **FDR corrected p** |
| Genes that show selective expression in a subset of cell types vs broadly expressed in many cell types. Correspond to 20% - 57% of enriched\_genes for a given cell type. WBPaper00037950:hypodermis\_larva\_SelectivelyEnriched | 13 | 6.65 | 2.10e-05 |
| Genes significantly enriched (> 2x, FDR < 5%) in a particular cell-type versus a reference sample of all cells at the same stage. WBPaper00037950:germline-precursors\_embryo\_enriched | 16 | 4.95 | 2.55e-05 |
| Genes that show selective expression in a subset of cell types vs broadly expressed in many cell types. Correspond to 20% - 57% of enriched\_genes for a given cell type. WBPaper00037950:germline-precursors\_embryo\_SelectivelyEnriched | 12 | 6.51 | 8.56e-05 |
| Genes significantly enriched (> 2x, FDR < 5%) in a particular cell-type versus a reference sample of all cells at the same stage. WBPaper00037950:hypodermis\_larva\_enriched | 16 | 3.86 | 6.01e-04 |
| Genes that show selective expression in a subset of cell types vs broadly expressed in many cell types. Correspond to 20% - 57% of enriched\_genes for a given cell type. WBPaper00037950:hypodermis\_embryo\_SelectivelyEnriched | 9 | 7.58 | 8.47e-04 |
| Genes significantly enriched (> 2x, FDR < 5%) in a particular cell-type versus a reference sample of all cells at the same stage. WBPaper00037950:hypodermis\_embryo\_enriched | 12 | 4.93 | 1.28e-03 |
| A cluster of genes that oscillates with the molting cycle during early larval development. | 7 | 10.30 | 1.60e-03 |
| Developmentally modulated gene cluster. cgc4386\_cluster\_4\_6 | 5 | 17.33 | 3.05e-03 |
| Genes with expression level up regulated after treatment with Methylmercury (MeHg) by RNAseq analysis. | 9 | 5.17 | 1.30e-02 |
| Germline-enriched and sex-biased expression profile cluster B. | 10 | 4.01 | 3.50e-02 |

### Motifs enriched

|  |  |  |  |  |  |
| --- | --- | --- | --- | --- | --- |
| **Motif** | **Logo** | **Possible orthologs** | **Number of motifs in cluster** | **Enrichment** | **FDR corrected p** |
| pTH2283 |  | odd-2 | 27 | 2.24 | 0.0011 |
| V$NKX25\_02 |  | ceh-24 | 37 | 1.72 | 0.0021 |
| GATA6\_f2 |  | elt-1 | 51 | 1.28 | 0.0023 |
| V$GATA1\_02 |  | elt-1 | 48 | 1.38 | 0.0025 |
| pTH3831 |  | ces-2 C48E7.11 | 50 | 1.31 | 0.0033 |
| Pou3f1\_3819 |  | ceh-6 | 20 | 2.59 | 0.0036 |
| Hoxd9\_2 |  | php-3 lin-39 | 30 | 1.93 | 0.0038 |
| MA0126.1 |  | lin-48 pax-3 | 34 | 1.77 | 0.0041 |
| tll\_FlyReg\_FBgn0003720 |  | nhr-239 | 43 | 1.47 | 0.0058 |
| MA0102.3 |  | C48E7.11 | 47 | 1.36 | 0.0066 |
| pTH6132 |  | ceh-14 lin-39 alr-1 ZC123.3 | 52 | 1.21 | 0.0067 |
| srp\_SANGER\_5\_FBgn0003507 |  | elt-7 elt-6 elt-1 ceh-34 | 38 | 1.59 | 0.0075 |
| POU3F4\_1 |  | ceh-18 | 25 | 2.09 | 0.0076 |
| Hoxa10\_2318 |  | ceh-24 | 34 | 1.70 | 0.0084 |
| Abd-A\_FlyReg\_FBgn0000014 |  | lin-39 | 25 | 2.04 | 0.0100 |
| SOX2\_1 |  | sox-4 | 45 | 1.39 | 0.0100 |
| V$T3R\_01 |  | nhr-2 (-0.57) nhr-15 (-0.52) | 27 | 1.94 | 0.0100 |
| V$GATA1\_06 |  | ztf-29 elt-1 | 14 | 3.10 | 0.0110 |
| pTH5250 |  | C48E7.11 | 51 | 1.23 | 0.0110 |
| Pou2f1\_3081 |  | ceh-18 | 32 | 1.73 | 0.0120 |
| FOXI1\_2 |  | let-381 (0.55) lin-31 (0.52) | 46 | 1.36 | 0.0120 |
| pTH5808 |  | pal-1 ceh-24 | 35 | 1.63 | 0.0140 |
| pTH10779 |  | nhr-182 | 50 | 1.25 | 0.0140 |
| V$GATA6\_01 |  | elt-1 | 32 | 1.69 | 0.0180 |
| Exd\_SOLEXA\_FBgn0000611 |  | ceh-32 ceh-20 | 26 | 1.91 | 0.0180 |
| Hoxa9\_2622 |  | lin-39 | 20 | 2.24 | 0.0180 |
| HXB7\_si |  | lin-39 ceh-20 | 21 | 2.17 | 0.0190 |
| Cdx2\_4272 |  | ceh-13 | 40 | 1.47 | 0.0190 |
| BARHL2\_6 |  | ceh-1 ceh-14 ceh-31 | 46 | 1.34 | 0.0190 |
| Pou2f3\_3986 |  | ceh-18 | 9 | 4.24 | 0.0200 |
| DLX2\_f1 |  | ceh-43 hmbx-1 lin-39 | 42 | 1.42 | 0.0200 |
| V$OCT1\_03 |  | ceh-18 | 11 | 3.46 | 0.0210 |
| MEIS1\_f2 |  | ceh-32 | 31 | 1.70 | 0.0210 |
| V$GATA3\_03 |  | elt-1 | 13 | 3.00 | 0.0220 |
| MEF2B\_1 |  | mef-2 | 29 | 1.77 | 0.0220 |
| V$YY1\_01 |  | lsy-2 | 51 | 1.21 | 0.0220 |
| MA0543.1 |  | eor-1 (-0.72) | 29 | 1.77 | 0.0220 |
| Alx4\_1 |  | alr-1 | 37 | 1.53 | 0.0230 |
| pTH10777 |  | dmd-3 | 25 | 1.91 | 0.0230 |
| V$CDPCR1\_01 |  | ceh-48 | 45 | 1.35 | 0.0240 |
| MA0007.2 |  | nhr-255 | 49 | 1.26 | 0.0250 |
| Gata3\_1024 |  | elt-1 | 31 | 1.68 | 0.0250 |
| IRX2\_1 |  | irx-1 | 36 | 1.54 | 0.0270 |
| Hoxc9\_2367 |  | lin-39 ceh-12 | 21 | 2.08 | 0.0290 |
| pTH10822 |  | unc-120 | 46 | 1.32 | 0.0290 |
| ems\_FlyReg\_FBgn0000576 |  | ceh-2 | 15 | 2.59 | 0.0290 |
| pTH9353 |  | ceh-51 | 12 | 3.05 | 0.0300 |
| pTH9335 |  | mel-28 (-0.58) | 37 | 1.50 | 0.0310 |
| pTH6106 |  | nhr-182 | 46 | 1.31 | 0.0320 |
| pTH5508 |  | nhr-19 (0.52) nhr-213 | 23 | 1.95 | 0.0320 |
| POU3F2\_1 |  | ceh-18 | 39 | 1.46 | 0.0330 |
| pTH5690 |  | ceh-32 | 43 | 1.37 | 0.0330 |
| pTH10717 |  | syd-9 | 16 | 2.44 | 0.0330 |
| ONEC2\_si |  | ceh-48 | 51 | 1.19 | 0.0340 |
| Six1\_0935 |  | ceh-32 | 42 | 1.39 | 0.0340 |
| GRHL1\_2 |  | grh-1 (0.61) | 9 | 3.84 | 0.0340 |
| RORA\_f1 |  | nhr-213 | 8 | 4.31 | 0.0340 |
| MA0033.1 |  | lin-31 (0.52) | 38 | 1.47 | 0.0350 |
| Mw144 |  | elt-1 | 28 | 1.74 | 0.0350 |
| pTH10808 |  | ztf-19 | 19 | 2.16 | 0.0370 |
| pTH8985 |  | athp-1 (-0.56) | 23 | 1.93 | 0.0370 |
| N$SKN1\_02 |  | skn-1 | 15 | 2.52 | 0.0370 |
| pTH4325 |  | ceh-18 | 13 | 2.78 | 0.0380 |
| V$FAC1\_01 |  | gei-8 | 19 | 2.16 | 0.0380 |
| SMAD3\_1 |  | daf-8 | 40 | 1.42 | 0.0380 |
| Lmx1a\_2238 |  | lim-6 | 31 | 1.64 | 0.0380 |
| pTH9216 |  | ceh-18 | 28 | 1.73 | 0.0390 |
| CEBPE\_f1 |  | C48E7.11 | 46 | 1.30 | 0.0390 |
| pTH6482 |  | ceh-19 | 28 | 1.72 | 0.0390 |
| pTH5919 |  | irx-1 | 26 | 1.79 | 0.0400 |
| HOXA10\_1 |  | lin-39 | 32 | 1.60 | 0.0410 |
| Pou3f4\_3773 |  | ceh-6 | 21 | 2.01 | 0.0420 |
| srp\_FlyReg\_FBgn0003507 |  | elt-1 | 43 | 1.36 | 0.0420 |
| V$NKX22\_01 |  | dsc-1 (0.67) | 20 | 2.06 | 0.0440 |
| pTH3819 |  | ceh-18 | 18 | 2.19 | 0.0440 |
| V$CREB\_01 |  | crh-1 | 29 | 1.68 | 0.0450 |
| FOXO4\_f1 |  | daf-16 (-0.52) lin-31 (0.52) | 49 | 1.23 | 0.0450 |
| CG4854\_SANGER\_10\_FBgn0038766 |  | K11D2.4 | 40 | 1.40 | 0.0490 |
| Foxg1\_2 |  | lin-31 (0.52) | 17 | 2.24 | 0.0490 |
| pTH9930 |  | lin-29 | 31 | 1.61 | 0.0490 |
| MSX2\_2 |  | ceh-1 | 9 | 3.57 | 0.0500 |

### Correlated (and anti-correlated) transcription factors

|  |  |
| --- | --- |
| **Transcription factor** | **Correlation** |
| nhr-87 | 0.83 |
| atf-8 | 0.82 |
| nhr-31 | 0.82 |
| nhr-92 | 0.82 |
| nhr-14 | 0.80 |
| nhr-23 | 0.75 |
| hlh-33 | 0.74 |
| moe-3 | 0.73 |
| nhr-43 | 0.72 |
| blmp-1 | 0.71 |
| nhr-149 | 0.70 |
| nhr-260 | 0.69 |
| dsc-1 | 0.67 |
| madf-1 | 0.67 |
| nhr-5 | 0.67 |
| mxl-3 | 0.66 |
| sdz-38 | 0.66 |
| nhr-146 | 0.66 |
| bed-3 | 0.66 |
| nhr-120 | 0.64 |
| nhr-147 | 0.63 |
| dhhc-2 | 0.63 |
| ccch-1 | 0.63 |
| hlh-8 | 0.63 |
| ztf-30 | 0.63 |
| F33H1.4 | -0.70 |
| cdc-14 | -0.70 |
| hmg-3 | -0.70 |
| nfi-1 | -0.70 |
| lin-13 | -0.70 |
| T20F7.1 | -0.71 |
| hmg-4 | -0.71 |
| isw-1 | -0.71 |
| dpl-1 | -0.71 |
| mep-1 | -0.72 |
| ztf-18 | -0.72 |
| eor-1 | -0.72 |
| nfyb-1 | -0.73 |
| B0250.4 | -0.73 |
| F21A10.2 | -0.73 |
| sdc-2 | -0.73 |
| lin-38 | -0.74 |
| mig-5 | -0.74 |
| lin-36 | -0.74 |
| aptf-2 | -0.74 |
| athp-2 | -0.74 |
| K09A11.1 | -0.76 |
| snpc-4 | -0.77 |
| T22C8.3 | -0.77 |
| wrm-1 | -0.85 |

### ChIP peaks enriched

|  |  |  |  |  |
| --- | --- | --- | --- | --- |
| **Gene** | **Experiment** | **Number of upstream peaks** | **Enrichment** | **FDR corrected p** |
| nhr-23 | NHR-23\_Larvae-L3-stage | 26 | 2.59 | 3.9e-05 |
| ama-1 | AMA-1\_Larvae-L3-stage | 11 | 5.06 | 4.3e-04 |
| nhr-25 | NHR-25\_Larvae-L2-stage | 23 | 2.37 | 1.0e-03 |
| elt-3 | ELT-3\_Embryos | 17 | 2.30 | 2.1e-02 |
| nhr-6 | NHR-6\_Larvae-L2-stage | 12 | 2.93 | 2.1e-02 |
| sax-3 | SAX-3\_Larvae-L4-stage | 25 | 1.83 | 2.1e-02 |
| nhr-28 | NHR-28\_Larvae-L4-stage | 14 | 2.56 | 2.5e-02 |
| nhr-129 | NHR-129\_Larvae-L2-stage | 26 | 1.69 | 4.7e-02 |
