# Supplementary material for: Overlapping cell population expression profiling and regulatory inference in C. elegans
Source: BMC Genomics. 2016 Feb 29;17:159. doi: 10.1186/s12864-016-2482-z (PMC4772325; doi:10.1186/s12864-016-2482-z)
Supplement: Additional file 13: — Web supplement. (DOC 21 kb) [file 12864_2016_2482_MOESM13_ESM.zip › sortWeb/clusters/hier.300.clusters/157.html]

Cluster 157 

## Cluster 157

### Expression

| cnd-1 rep. 1 | cnd-1 rep. 2 | cnd-1 rep. 3 | pha-4 rep. 1 | pha-4 rep. 2 | pha-4 rep. 3 | ceh-27 | ceh-36 | ceh-6 | F21D5.9 | mir-57 | mls-2 | pal-1 | pros-1 | ttx-3 | unc-130 | hlh-16 | irx-1 | ceh-6 (+) hlh-16 (+) | ceh-6 (+) hlh-16 (-) | ceh-6 (-) hlh-16 (+) | cnd-1 singlets | pha-4 singlets | 0 | 60 | 120 | 150 | 180 | 240 | 330 | 390 | 420 | 480 | 540 | 570 | 600 | 630 | 660 | NAME | Functional description |
| --- | --- | --- | --- | --- | --- | --- | --- | --- | --- | --- | --- | --- | --- | --- | --- | --- | --- | --- | --- | --- | --- | --- | --- | --- | --- | --- | --- | --- | --- | --- | --- | --- | --- | --- | --- | --- | --- | --- | --- |
|  |  |  |  |  |  |  |  |  |  |  |  |  |  |  |  |  |  |  |  |  |  |  |  |  |  |  |  |  |  |  |  |  |  |  |  |  |  | F25B4.7 |  |
|  |  |  |  |  |  |  |  |  |  |  |  |  |  |  |  |  |  |  |  |  |  |  |  |  |  |  |  |  |  |  |  |  |  |  |  |  |  | F39H2.3 |  |
|  |  |  |  |  |  |  |  |  |  |  |  |  |  |  |  |  |  |  |  |  |  |  |  |  |  |  |  |  |  |  |  |  |  |  |  |  |  | *nhr-78* | Nuclear Hormone Receptor family |
|  |  |  |  |  |  |  |  |  |  |  |  |  |  |  |  |  |  |  |  |  |  |  |  |  |  |  |  |  |  |  |  |  |  |  |  |  |  | B0001.4 |  |
|  |  |  |  |  |  |  |  |  |  |  |  |  |  |  |  |  |  |  |  |  |  |  |  |  |  |  |  |  |  |  |  |  |  |  |  |  |  | *rap-2* | RAP homolog (vertebrate Rap GTPase family) |
|  |  |  |  |  |  |  |  |  |  |  |  |  |  |  |  |  |  |  |  |  |  |  |  |  |  |  |  |  |  |  |  |  |  |  |  |  |  | W04A4.3 |  |
|  |  |  |  |  |  |  |  |  |  |  |  |  |  |  |  |  |  |  |  |  |  |  |  |  |  |  |  |  |  |  |  |  |  |  |  |  |  | T25B2.4 |  |
|  |  |  |  |  |  |  |  |  |  |  |  |  |  |  |  |  |  |  |  |  |  |  |  |  |  |  |  |  |  |  |  |  |  |  |  |  |  | F15D3.6 |  |
|  |  |  |  |  |  |  |  |  |  |  |  |  |  |  |  |  |  |  |  |  |  |  |  |  |  |  |  |  |  |  |  |  |  |  |  |  |  | F44E7.2 |  |
|  |  |  |  |  |  |  |  |  |  |  |  |  |  |  |  |  |  |  |  |  |  |  |  |  |  |  |  |  |  |  |  |  |  |  |  |  |  | F35C8.8 |  |
|  |  |  |  |  |  |  |  |  |  |  |  |  |  |  |  |  |  |  |  |  |  |  |  |  |  |  |  |  |  |  |  |  |  |  |  |  |  | F28B12.1 |  |
|  |  |  |  |  |  |  |  |  |  |  |  |  |  |  |  |  |  |  |  |  |  |  |  |  |  |  |  |  |  |  |  |  |  |  |  |  |  | Y34F4.6 |  |
|  |  |  |  |  |  |  |  |  |  |  |  |  |  |  |  |  |  |  |  |  |  |  |  |  |  |  |  |  |  |  |  |  |  |  |  |  |  | T02G6.4 |  |
|  |  |  |  |  |  |  |  |  |  |  |  |  |  |  |  |  |  |  |  |  |  |  |  |  |  |  |  |  |  |  |  |  |  |  |  |  |  | ZC21.14 |  |
|  |  |  |  |  |  |  |  |  |  |  |  |  |  |  |  |  |  |  |  |  |  |  |  |  |  |  |  |  |  |  |  |  |  |  |  |  |  | *dhhc-7* | DHHC-types zinc finger protein |
|  |  |  |  |  |  |  |  |  |  |  |  |  |  |  |  |  |  |  |  |  |  |  |  |  |  |  |  |  |  |  |  |  |  |  |  |  |  | T23F2.5 |  |
|  |  |  |  |  |  |  |  |  |  |  |  |  |  |  |  |  |  |  |  |  |  |  |  |  |  |  |  |  |  |  |  |  |  |  |  |  |  | F23A7.3 |  |
|  |  |  |  |  |  |  |  |  |  |  |  |  |  |  |  |  |  |  |  |  |  |  |  |  |  |  |  |  |  |  |  |  |  |  |  |  |  | C27C7.1 |  |
|  |  |  |  |  |  |  |  |  |  |  |  |  |  |  |  |  |  |  |  |  |  |  |  |  |  |  |  |  |  |  |  |  |  |  |  |  |  | C36A4.4 |  |
|  |  |  |  |  |  |  |  |  |  |  |  |  |  |  |  |  |  |  |  |  |  |  |  |  |  |  |  |  |  |  |  |  |  |  |  |  |  | D1009.8 |  |
|  |  |  |  |  |  |  |  |  |  |  |  |  |  |  |  |  |  |  |  |  |  |  |  |  |  |  |  |  |  |  |  |  |  |  |  |  |  | *mec-6* | MEChanosensory abnormality |
|  |  |  |  |  |  |  |  |  |  |  |  |  |  |  |  |  |  |  |  |  |  |  |  |  |  |  |  |  |  |  |  |  |  |  |  |  |  | ZK1073.1 |  |
|  |  |  |  |  |  |  |  |  |  |  |  |  |  |  |  |  |  |  |  |  |  |  |  |  |  |  |  |  |  |  |  |  |  |  |  |  |  | *tsp-14* | TetraSPanin family |
|  |  |  |  |  |  |  |  |  |  |  |  |  |  |  |  |  |  |  |  |  |  |  |  |  |  |  |  |  |  |  |  |  |  |  |  |  |  | K09F5.6 |  |
|  |  |  |  |  |  |  |  |  |  |  |  |  |  |  |  |  |  |  |  |  |  |  |  |  |  |  |  |  |  |  |  |  |  |  |  |  |  | F11A10.5 |  |
|  |  |  |  |  |  |  |  |  |  |  |  |  |  |  |  |  |  |  |  |  |  |  |  |  |  |  |  |  |  |  |  |  |  |  |  |  |  | Y73F8A.5 |  |
|  |  |  |  |  |  |  |  |  |  |  |  |  |  |  |  |  |  |  |  |  |  |  |  |  |  |  |  |  |  |  |  |  |  |  |  |  |  | *tag-321* | Temporarily Assigned Gene name |
|  |  |  |  |  |  |  |  |  |  |  |  |  |  |  |  |  |  |  |  |  |  |  |  |  |  |  |  |  |  |  |  |  |  |  |  |  |  | *syg-2* | SYnaptoGenesis abnormal |
|  |  |  |  |  |  |  |  |  |  |  |  |  |  |  |  |  |  |  |  |  |  |  |  |  |  |  |  |  |  |  |  |  |  |  |  |  |  | *cth-2* | CystaTHionine gamma lyase |
|  |  |  |  |  |  |  |  |  |  |  |  |  |  |  |  |  |  |  |  |  |  |  |  |  |  |  |  |  |  |  |  |  |  |  |  |  |  | ZK829.7 |  |
|  |  |  |  |  |  |  |  |  |  |  |  |  |  |  |  |  |  |  |  |  |  |  |  |  |  |  |  |  |  |  |  |  |  |  |  |  |  | *unc-24* | UNCoordinated |
|  |  |  |  |  |  |  |  |  |  |  |  |  |  |  |  |  |  |  |  |  |  |  |  |  |  |  |  |  |  |  |  |  |  |  |  |  |  | F57B7.2 |  |
|  |  |  |  |  |  |  |  |  |  |  |  |  |  |  |  |  |  |  |  |  |  |  |  |  |  |  |  |  |  |  |  |  |  |  |  |  |  | *nab-1* | NeurABin |
|  |  |  |  |  |  |  |  |  |  |  |  |  |  |  |  |  |  |  |  |  |  |  |  |  |  |  |  |  |  |  |  |  |  |  |  |  |  | *zig-10* | 2 (Zwei) IG domain protein |
|  |  |  |  |  |  |  |  |  |  |  |  |  |  |  |  |  |  |  |  |  |  |  |  |  |  |  |  |  |  |  |  |  |  |  |  |  |  | C01B7.5 |  |
|  |  |  |  |  |  |  |  |  |  |  |  |  |  |  |  |  |  |  |  |  |  |  |  |  |  |  |  |  |  |  |  |  |  |  |  |  |  | W07G4.1 |  |
|  |  |  |  |  |  |  |  |  |  |  |  |  |  |  |  |  |  |  |  |  |  |  |  |  |  |  |  |  |  |  |  |  |  |  |  |  |  | Y97E10AR.3 |  |
|  |  |  |  |  |  |  |  |  |  |  |  |  |  |  |  |  |  |  |  |  |  |  |  |  |  |  |  |  |  |  |  |  |  |  |  |  |  | F11C1.7 |  |
|  |  |  |  |  |  |  |  |  |  |  |  |  |  |  |  |  |  |  |  |  |  |  |  |  |  |  |  |  |  |  |  |  |  |  |  |  |  | *vti-1* | VTI (Vesicle Transport through t-SNARE Interaction) homolog |
|  |  |  |  |  |  |  |  |  |  |  |  |  |  |  |  |  |  |  |  |  |  |  |  |  |  |  |  |  |  |  |  |  |  |  |  |  |  | C25H3.12 |  |
|  |  |  |  |  |  |  |  |  |  |  |  |  |  |  |  |  |  |  |  |  |  |  |  |  |  |  |  |  |  |  |  |  |  |  |  |  |  | R03A10.1 |  |
|  |  |  |  |  |  |  |  |  |  |  |  |  |  |  |  |  |  |  |  |  |  |  |  |  |  |  |  |  |  |  |  |  |  |  |  |  |  | *zip-4* | bZIP transcription factor family |
|  |  |  |  |  |  |  |  |  |  |  |  |  |  |  |  |  |  |  |  |  |  |  |  |  |  |  |  |  |  |  |  |  |  |  |  |  |  | *sbds-1* | Shwachman-Bodian-Diamond Syndrome protein homolog |
|  |  |  |  |  |  |  |  |  |  |  |  |  |  |  |  |  |  |  |  |  |  |  |  |  |  |  |  |  |  |  |  |  |  |  |  |  |  | K07C11.7 |  |
|  |  |  |  |  |  |  |  |  |  |  |  |  |  |  |  |  |  |  |  |  |  |  |  |  |  |  |  |  |  |  |  |  |  |  |  |  |  | C14A11.6 |  |
|  |  |  |  |  |  |  |  |  |  |  |  |  |  |  |  |  |  |  |  |  |  |  |  |  |  |  |  |  |  |  |  |  |  |  |  |  |  | F30F8.5 |  |
|  |  |  |  |  |  |  |  |  |  |  |  |  |  |  |  |  |  |  |  |  |  |  |  |  |  |  |  |  |  |  |  |  |  |  |  |  |  | *nlp-35* | Neuropeptide-Like Protein |
|  |  |  |  |  |  |  |  |  |  |  |  |  |  |  |  |  |  |  |  |  |  |  |  |  |  |  |  |  |  |  |  |  |  |  |  |  |  | F34D10.3 |  |
|  |  |  |  |  |  |  |  |  |  |  |  |  |  |  |  |  |  |  |  |  |  |  |  |  |  |  |  |  |  |  |  |  |  |  |  |  |  | ZK470.2 |  |
|  |  |  |  |  |  |  |  |  |  |  |  |  |  |  |  |  |  |  |  |  |  |  |  |  |  |  |  |  |  |  |  |  |  |  |  |  |  | Y32H12A.6 |  |
|  |  |  |  |  |  |  |  |  |  |  |  |  |  |  |  |  |  |  |  |  |  |  |  |  |  |  |  |  |  |  |  |  |  |  |  |  |  | *unc-42* | UNCoordinated |
|  |  |  |  |  |  |  |  |  |  |  |  |  |  |  |  |  |  |  |  |  |  |  |  |  |  |  |  |  |  |  |  |  |  |  |  |  |  | F28E10.1 |  |
|  |  |  |  |  |  |  |  |  |  |  |  |  |  |  |  |  |  |  |  |  |  |  |  |  |  |  |  |  |  |  |  |  |  |  |  |  |  | F07C3.2 |  |
|  |  |  |  |  |  |  |  |  |  |  |  |  |  |  |  |  |  |  |  |  |  |  |  |  |  |  |  |  |  |  |  |  |  |  |  |  |  | *jip-1* | JNK Interacting Protein (scaffold protein) |
|  |  |  |  |  |  |  |  |  |  |  |  |  |  |  |  |  |  |  |  |  |  |  |  |  |  |  |  |  |  |  |  |  |  |  |  |  |  | *ubh-1* | UBiquitin C-terminal Hydrolase (family 1) |
|  |  |  |  |  |  |  |  |  |  |  |  |  |  |  |  |  |  |  |  |  |  |  |  |  |  |  |  |  |  |  |  |  |  |  |  |  |  | F29A7.4 |  |
|  |  |  |  |  |  |  |  |  |  |  |  |  |  |  |  |  |  |  |  |  |  |  |  |  |  |  |  |  |  |  |  |  |  |  |  |  |  | *pdl-1* | Phosphodiesterase DeLta-like |
|  |  |  |  |  |  |  |  |  |  |  |  |  |  |  |  |  |  |  |  |  |  |  |  |  |  |  |  |  |  |  |  |  |  |  |  |  |  | *calf-1* | CAlcium channel Localization Factor |
|  |  |  |  |  |  |  |  |  |  |  |  |  |  |  |  |  |  |  |  |  |  |  |  |  |  |  |  |  |  |  |  |  |  |  |  |  |  | *lron-3* | eLRR (extracellular Leucine-Rich Repeat) ONly |
|  |  |  |  |  |  |  |  |  |  |  |  |  |  |  |  |  |  |  |  |  |  |  |  |  |  |  |  |  |  |  |  |  |  |  |  |  |  | K08E7.6 |  |
|  |  |  |  |  |  |  |  |  |  |  |  |  |  |  |  |  |  |  |  |  |  |  |  |  |  |  |  |  |  |  |  |  |  |  |  |  |  | F29G6.2 |  |
|  |  |  |  |  |  |  |  |  |  |  |  |  |  |  |  |  |  |  |  |  |  |  |  |  |  |  |  |  |  |  |  |  |  |  |  |  |  | *pdhk-2* | Pyruvate DeHydrogenase Kinase |
|  |  |  |  |  |  |  |  |  |  |  |  |  |  |  |  |  |  |  |  |  |  |  |  |  |  |  |  |  |  |  |  |  |  |  |  |  |  | C49C3.15 |  |
|  |  |  |  |  |  |  |  |  |  |  |  |  |  |  |  |  |  |  |  |  |  |  |  |  |  |  |  |  |  |  |  |  |  |  |  |  |  | *lntl-1* | LIN-24 (Twenty-four) Like |
|  |  |  |  |  |  |  |  |  |  |  |  |  |  |  |  |  |  |  |  |  |  |  |  |  |  |  |  |  |  |  |  |  |  |  |  |  |  | *fbxa-137* | F-box A protein |
|  |  |  |  |  |  |  |  |  |  |  |  |  |  |  |  |  |  |  |  |  |  |  |  |  |  |  |  |  |  |  |  |  |  |  |  |  |  | T21E12.2 |  |
|  |  |  |  |  |  |  |  |  |  |  |  |  |  |  |  |  |  |  |  |  |  |  |  |  |  |  |  |  |  |  |  |  |  |  |  |  |  | *ykt-6* | YKT6 (yeast v-SNARE) homolog |
|  |  |  |  |  |  |  |  |  |  |  |  |  |  |  |  |  |  |  |  |  |  |  |  |  |  |  |  |  |  |  |  |  |  |  |  |  |  | *dop-6* | DOPamine receptor |
|  |  |  |  |  |  |  |  |  |  |  |  |  |  |  |  |  |  |  |  |  |  |  |  |  |  |  |  |  |  |  |  |  |  |  |  |  |  | F30F8.10 |  |
|  |  |  |  |  |  |  |  |  |  |  |  |  |  |  |  |  |  |  |  |  |  |  |  |  |  |  |  |  |  |  |  |  |  |  |  |  |  | *gpb-2* | G Protein, Beta subunit |
|  |  |  |  |  |  |  |  |  |  |  |  |  |  |  |  |  |  |  |  |  |  |  |  |  |  |  |  |  |  |  |  |  |  |  |  |  |  | *vps-36* | related to yeast Vacuolar Protein Sorting factor |
|  |  |  |  |  |  |  |  |  |  |  |  |  |  |  |  |  |  |  |  |  |  |  |  |  |  |  |  |  |  |  |  |  |  |  |  |  |  | E02A10.7 |  |
|  |  |  |  |  |  |  |  |  |  |  |  |  |  |  |  |  |  |  |  |  |  |  |  |  |  |  |  |  |  |  |  |  |  |  |  |  |  | F31E9.11 |  |
|  |  |  |  |  |  |  |  |  |  |  |  |  |  |  |  |  |  |  |  |  |  |  |  |  |  |  |  |  |  |  |  |  |  |  |  |  |  | *madf-5* | MADF domain transcription factor |
|  |  |  |  |  |  |  |  |  |  |  |  |  |  |  |  |  |  |  |  |  |  |  |  |  |  |  |  |  |  |  |  |  |  |  |  |  |  | Y71G12B.23 |  |
|  |  |  |  |  |  |  |  |  |  |  |  |  |  |  |  |  |  |  |  |  |  |  |  |  |  |  |  |  |  |  |  |  |  |  |  |  |  | *vha-16* | Vacuolar H ATPase |
|  |  |  |  |  |  |  |  |  |  |  |  |  |  |  |  |  |  |  |  |  |  |  |  |  |  |  |  |  |  |  |  |  |  |  |  |  |  | *rpi-2* | Retinitis PIgmentosa (RP) disease gene homolog |
|  |  |  |  |  |  |  |  |  |  |  |  |  |  |  |  |  |  |  |  |  |  |  |  |  |  |  |  |  |  |  |  |  |  |  |  |  |  | C48B4.3 |  |
|  |  |  |  |  |  |  |  |  |  |  |  |  |  |  |  |  |  |  |  |  |  |  |  |  |  |  |  |  |  |  |  |  |  |  |  |  |  | *dct-1* | DAF-16/FOXO Controlled, germline Tumor affecting |
|  |  |  |  |  |  |  |  |  |  |  |  |  |  |  |  |  |  |  |  |  |  |  |  |  |  |  |  |  |  |  |  |  |  |  |  |  |  | ZK688.9 |  |
|  |  |  |  |  |  |  |  |  |  |  |  |  |  |  |  |  |  |  |  |  |  |  |  |  |  |  |  |  |  |  |  |  |  |  |  |  |  | ZK688.11 |  |
|  |  |  |  |  |  |  |  |  |  |  |  |  |  |  |  |  |  |  |  |  |  |  |  |  |  |  |  |  |  |  |  |  |  |  |  |  |  | *lgg-2* | LC3, GABARAP and GATE-16 family |
|  |  |  |  |  |  |  |  |  |  |  |  |  |  |  |  |  |  |  |  |  |  |  |  |  |  |  |  |  |  |  |  |  |  |  |  |  |  | Y44E3A.1 |  |
|  |  |  |  |  |  |  |  |  |  |  |  |  |  |  |  |  |  |  |  |  |  |  |  |  |  |  |  |  |  |  |  |  |  |  |  |  |  | *bkip-1* | BK channel Interacting Protein |
|  |  |  |  |  |  |  |  |  |  |  |  |  |  |  |  |  |  |  |  |  |  |  |  |  |  |  |  |  |  |  |  |  |  |  |  |  |  | *linc-73* | Long Intervening Non-Coding RNA |
|  |  |  |  |  |  |  |  |  |  |  |  |  |  |  |  |  |  |  |  |  |  |  |  |  |  |  |  |  |  |  |  |  |  |  |  |  |  | *par-5* | abnormal embryonic PARtitioning of cytoplasm |
|  |  |  |  |  |  |  |  |  |  |  |  |  |  |  |  |  |  |  |  |  |  |  |  |  |  |  |  |  |  |  |  |  |  |  |  |  |  | *blos-1* | BLOC (Biogenesis of Lysosome-related Organelles Complex) Subunit homolog |
|  |  |  |  |  |  |  |  |  |  |  |  |  |  |  |  |  |  |  |  |  |  |  |  |  |  |  |  |  |  |  |  |  |  |  |  |  |  | *tag-299* | Temporarily Assigned Gene name |
|  |  |  |  |  |  |  |  |  |  |  |  |  |  |  |  |  |  |  |  |  |  |  |  |  |  |  |  |  |  |  |  |  |  |  |  |  |  | *oxy-5* | abnormal OXYgen sensitivity |
|  |  |  |  |  |  |  |  |  |  |  |  |  |  |  |  |  |  |  |  |  |  |  |  |  |  |  |  |  |  |  |  |  |  |  |  |  |  | *cuc-1* | CU (copper) Chaperonin |
|  |  |  |  |  |  |  |  |  |  |  |  |  |  |  |  |  |  |  |  |  |  |  |  |  |  |  |  |  |  |  |  |  |  |  |  |  |  | *msra-1* | Methionine Sulfoxide Reductase A |
|  |  |  |  |  |  |  |  |  |  |  |  |  |  |  |  |  |  |  |  |  |  |  |  |  |  |  |  |  |  |  |  |  |  |  |  |  |  | *pfn-1* | ProFiliN |
|  |  |  |  |  |  |  |  |  |  |  |  |  |  |  |  |  |  |  |  |  |  |  |  |  |  |  |  |  |  |  |  |  |  |  |  |  |  | C54E10.6 |  |
|  |  |  |  |  |  |  |  |  |  |  |  |  |  |  |  |  |  |  |  |  |  |  |  |  |  |  |  |  |  |  |  |  |  |  |  |  |  | F48F7.6 |  |
|  |  |  |  |  |  |  |  |  |  |  |  |  |  |  |  |  |  |  |  |  |  |  |  |  |  |  |  |  |  |  |  |  |  |  |  |  |  | *vps-25* | related to yeast Vacuolar Protein Sorting factor |
|  |  |  |  |  |  |  |  |  |  |  |  |  |  |  |  |  |  |  |  |  |  |  |  |  |  |  |  |  |  |  |  |  |  |  |  |  |  | T24H7.3 |  |
|  |  |  |  |  |  |  |  |  |  |  |  |  |  |  |  |  |  |  |  |  |  |  |  |  |  |  |  |  |  |  |  |  |  |  |  |  |  | *snpn-1* | SNaPiN (synaptic protein) homolog |
|  |  |  |  |  |  |  |  |  |  |  |  |  |  |  |  |  |  |  |  |  |  |  |  |  |  |  |  |  |  |  |  |  |  |  |  |  |  | F45E1.1 |  |
|  |  |  |  |  |  |  |  |  |  |  |  |  |  |  |  |  |  |  |  |  |  |  |  |  |  |  |  |  |  |  |  |  |  |  |  |  |  | C13F10.1 |  |
|  |  |  |  |  |  |  |  |  |  |  |  |  |  |  |  |  |  |  |  |  |  |  |  |  |  |  |  |  |  |  |  |  |  |  |  |  |  | *linc-14* | Long Intervening Non-Coding RNA |
|  |  |  |  |  |  |  |  |  |  |  |  |  |  |  |  |  |  |  |  |  |  |  |  |  |  |  |  |  |  |  |  |  |  |  |  |  |  | ZK809.10 |  |
|  |  |  |  |  |  |  |  |  |  |  |  |  |  |  |  |  |  |  |  |  |  |  |  |  |  |  |  |  |  |  |  |  |  |  |  |  |  | *rbx-2* | yeast RBX (ring finger protein) homolog |
|  |  |  |  |  |  |  |  |  |  |  |  |  |  |  |  |  |  |  |  |  |  |  |  |  |  |  |  |  |  |  |  |  |  |  |  |  |  | *hsb-1* | Heat Shock factor Binding protein |
|  |  |  |  |  |  |  |  |  |  |  |  |  |  |  |  |  |  |  |  |  |  |  |  |  |  |  |  |  |  |  |  |  |  |  |  |  |  | Y87G2A.18 |  |
|  |  |  |  |  |  |  |  |  |  |  |  |  |  |  |  |  |  |  |  |  |  |  |  |  |  |  |  |  |  |  |  |  |  |  |  |  |  | ZK632.10 |  |
|  |  |  |  |  |  |  |  |  |  |  |  |  |  |  |  |  |  |  |  |  |  |  |  |  |  |  |  |  |  |  |  |  |  |  |  |  |  | C56A3.4 |  |
|  |  |  |  |  |  |  |  |  |  |  |  |  |  |  |  |  |  |  |  |  |  |  |  |  |  |  |  |  |  |  |  |  |  |  |  |  |  | *mvb-12* | MVB (yeast MultiVesicular Body sorting factor) related |
|  |  |  |  |  |  |  |  |  |  |  |  |  |  |  |  |  |  |  |  |  |  |  |  |  |  |  |  |  |  |  |  |  |  |  |  |  |  | *aip-1* | Arsenite Inducible Protein |
|  |  |  |  |  |  |  |  |  |  |  |  |  |  |  |  |  |  |  |  |  |  |  |  |  |  |  |  |  |  |  |  |  |  |  |  |  |  | T21H3.5 |  |
|  |  |  |  |  |  |  |  |  |  |  |  |  |  |  |  |  |  |  |  |  |  |  |  |  |  |  |  |  |  |  |  |  |  |  |  |  |  | F46G11.4 |  |
|  |  |  |  |  |  |  |  |  |  |  |  |  |  |  |  |  |  |  |  |  |  |  |  |  |  |  |  |  |  |  |  |  |  |  |  |  |  | F38B2.4 |  |
|  |  |  |  |  |  |  |  |  |  |  |  |  |  |  |  |  |  |  |  |  |  |  |  |  |  |  |  |  |  |  |  |  |  |  |  |  |  | K10G9.2 |  |
|  |  |  |  |  |  |  |  |  |  |  |  |  |  |  |  |  |  |  |  |  |  |  |  |  |  |  |  |  |  |  |  |  |  |  |  |  |  | *sbt-1* | Seven B Two (mammalian 7BT prohormone convertase chaperone) homolog |
|  |  |  |  |  |  |  |  |  |  |  |  |  |  |  |  |  |  |  |  |  |  |  |  |  |  |  |  |  |  |  |  |  |  |  |  |  |  | T26E3.10 |  |
|  |  |  |  |  |  |  |  |  |  |  |  |  |  |  |  |  |  |  |  |  |  |  |  |  |  |  |  |  |  |  |  |  |  |  |  |  |  | K01A2.10 |  |
|  |  |  |  |  |  |  |  |  |  |  |  |  |  |  |  |  |  |  |  |  |  |  |  |  |  |  |  |  |  |  |  |  |  |  |  |  |  | F44A6.4 |  |
|  |  |  |  |  |  |  |  |  |  |  |  |  |  |  |  |  |  |  |  |  |  |  |  |  |  |  |  |  |  |  |  |  |  |  |  |  |  | W07G4.5 |  |
|  |  |  |  |  |  |  |  |  |  |  |  |  |  |  |  |  |  |  |  |  |  |  |  |  |  |  |  |  |  |  |  |  |  |  |  |  |  | *cysl-1* | CYsteine Synthase Like |
|  |  |  |  |  |  |  |  |  |  |  |  |  |  |  |  |  |  |  |  |  |  |  |  |  |  |  |  |  |  |  |  |  |  |  |  |  |  | *ceh-88* | C. Elegans Homeobox |
|  |  |  |  |  |  |  |  |  |  |  |  |  |  |  |  |  |  |  |  |  |  |  |  |  |  |  |  |  |  |  |  |  |  |  |  |  |  | *atat-2* | Alpha Tubulin AcetylTransferase |
|  |  |  |  |  |  |  |  |  |  |  |  |  |  |  |  |  |  |  |  |  |  |  |  |  |  |  |  |  |  |  |  |  |  |  |  |  |  | F07H5.4 |  |
|  |  |  |  |  |  |  |  |  |  |  |  |  |  |  |  |  |  |  |  |  |  |  |  |  |  |  |  |  |  |  |  |  |  |  |  |  |  | *gpc-2* | G Protein, Gamma subunit |
|  |  |  |  |  |  |  |  |  |  |  |  |  |  |  |  |  |  |  |  |  |  |  |  |  |  |  |  |  |  |  |  |  |  |  |  |  |  | *unc-69* | UNCoordinated |
|  |  |  |  |  |  |  |  |  |  |  |  |  |  |  |  |  |  |  |  |  |  |  |  |  |  |  |  |  |  |  |  |  |  |  |  |  |  | R03G5.7 |  |
|  |  |  |  |  |  |  |  |  |  |  |  |  |  |  |  |  |  |  |  |  |  |  |  |  |  |  |  |  |  |  |  |  |  |  |  |  |  | F21F3.7 |  |
|  |  |  |  |  |  |  |  |  |  |  |  |  |  |  |  |  |  |  |  |  |  |  |  |  |  |  |  |  |  |  |  |  |  |  |  |  |  | *linc-8* | Long Intervening Non-Coding RNA |
|  |  |  |  |  |  |  |  |  |  |  |  |  |  |  |  |  |  |  |  |  |  |  |  |  |  |  |  |  |  |  |  |  |  |  |  |  |  | C32E8.3 |  |
|  |  |  |  |  |  |  |  |  |  |  |  |  |  |  |  |  |  |  |  |  |  |  |  |  |  |  |  |  |  |  |  |  |  |  |  |  |  | C44B11.6 |  |
|  |  |  |  |  |  |  |  |  |  |  |  |  |  |  |  |  |  |  |  |  |  |  |  |  |  |  |  |  |  |  |  |  |  |  |  |  |  | *marc-4* | MARCH (Membrane-Associated Ring finger (C3HC4)) homolog |
|  |  |  |  |  |  |  |  |  |  |  |  |  |  |  |  |  |  |  |  |  |  |  |  |  |  |  |  |  |  |  |  |  |  |  |  |  |  | F53C3.4 |  |
|  |  |  |  |  |  |  |  |  |  |  |  |  |  |  |  |  |  |  |  |  |  |  |  |  |  |  |  |  |  |  |  |  |  |  |  |  |  | ZK512.11 |  |
|  |  |  |  |  |  |  |  |  |  |  |  |  |  |  |  |  |  |  |  |  |  |  |  |  |  |  |  |  |  |  |  |  |  |  |  |  |  | *gras-1* | GRASP (General Receptor for phosphoinositides 1-Associated Scaffold Protein) homolog |
|  |  |  |  |  |  |  |  |  |  |  |  |  |  |  |  |  |  |  |  |  |  |  |  |  |  |  |  |  |  |  |  |  |  |  |  |  |  | *cdk-5* | Cyclin-Dependent Kinase family |
|  |  |  |  |  |  |  |  |  |  |  |  |  |  |  |  |  |  |  |  |  |  |  |  |  |  |  |  |  |  |  |  |  |  |  |  |  |  | Y73B6BL.27 |  |
|  |  |  |  |  |  |  |  |  |  |  |  |  |  |  |  |  |  |  |  |  |  |  |  |  |  |  |  |  |  |  |  |  |  |  |  |  |  | F27D4.7 |  |
|  |  |  |  |  |  |  |  |  |  |  |  |  |  |  |  |  |  |  |  |  |  |  |  |  |  |  |  |  |  |  |  |  |  |  |  |  |  | *tli-1* | ToLlIp homolog |
|  |  |  |  |  |  |  |  |  |  |  |  |  |  |  |  |  |  |  |  |  |  |  |  |  |  |  |  |  |  |  |  |  |  |  |  |  |  | *aps-2* | AdaPtin, Small chain (clathrin associated complex) |
|  |  |  |  |  |  |  |  |  |  |  |  |  |  |  |  |  |  |  |  |  |  |  |  |  |  |  |  |  |  |  |  |  |  |  |  |  |  | Y62E10A.20 |  |
|  |  |  |  |  |  |  |  |  |  |  |  |  |  |  |  |  |  |  |  |  |  |  |  |  |  |  |  |  |  |  |  |  |  |  |  |  |  | *rab-19* | RAB family |
|  |  |  |  |  |  |  |  |  |  |  |  |  |  |  |  |  |  |  |  |  |  |  |  |  |  |  |  |  |  |  |  |  |  |  |  |  |  | F56F10.2 |  |
|  |  |  |  |  |  |  |  |  |  |  |  |  |  |  |  |  |  |  |  |  |  |  |  |  |  |  |  |  |  |  |  |  |  |  |  |  |  | F08G12.5 |  |
|  |  |  |  |  |  |  |  |  |  |  |  |  |  |  |  |  |  |  |  |  |  |  |  |  |  |  |  |  |  |  |  |  |  |  |  |  |  | K04F10.1 |  |
|  |  |  |  |  |  |  |  |  |  |  |  |  |  |  |  |  |  |  |  |  |  |  |  |  |  |  |  |  |  |  |  |  |  |  |  |  |  | *fbxa-141* | F-box A protein |
|  |  |  |  |  |  |  |  |  |  |  |  |  |  |  |  |  |  |  |  |  |  |  |  |  |  |  |  |  |  |  |  |  |  |  |  |  |  | F27D9.4 |  |
|  |  |  |  |  |  |  |  |  |  |  |  |  |  |  |  |  |  |  |  |  |  |  |  |  |  |  |  |  |  |  |  |  |  |  |  |  |  | VM106R.1 |  |
|  |  |  |  |  |  |  |  |  |  |  |  |  |  |  |  |  |  |  |  |  |  |  |  |  |  |  |  |  |  |  |  |  |  |  |  |  |  | R12B2.8 |  |
|  |  |  |  |  |  |  |  |  |  |  |  |  |  |  |  |  |  |  |  |  |  |  |  |  |  |  |  |  |  |  |  |  |  |  |  |  |  | *clc-3* | CLaudin-like in Caenorhabditis |

### Phenotypes enriched

none found

### Anatomy terms enriched

none found

### GO terms enriched

none found

### Expression clusters enriched

|  |  |  |  |
| --- | --- | --- | --- |
| **Group name** | **Number in cluster** | **Enrichment** | **FDR corrected p** |
| WT-Pico Pan-neural Enriched Genes, with genes found multiple times in a single dataset removed (without dups). | 53 | 3.53 | 4.49e-14 |
| Larval Pan-neural Enriched Genes. | 52 | 3.59 | 4.60e-14 |
| Genes significantly enriched (> 2x, FDR < 5%) in a particular cell-type versus a reference sample of all cells at the same stage. WBPaper00037950:A-class-motor-neurons\_larva\_enriched | 35 | 5.42 | 1.29e-13 |
| Embryonic Pan-neural Enriched Genes. | 50 | 3.29 | 6.67e-12 |
| Genes expressed in embryonic motor neurons (identified by unc-4::GFP expressing cells). | 96 | 1.69 | 3.06e-08 |
| Expressed transcripts enriched in embryonic motor neurons (identified by unc-4::GFP expressing cells). | 32 | 3.52 | 1.93e-07 |
| Embryonic A-class motor neuron enriched genes. | 32 | 3.52 | 1.93e-07 |
| Genes enriched in HLH-1 heat shock dataset. | 49 | 2.25 | 6.46e-06 |
| Genes significantly enriched (> 2x, FDR < 5%) in a particular cell-type versus a reference sample of all cells at the same stage. WBPaper00037950:all-neurons\_larva\_enriched | 30 | 3.16 | 7.57e-06 |
| Genes that showed expression levels higher than the corresponding reference sample (embryonic 0hr reference). WBPaper00037950:BAG-neuron\_expressed | 73 | 1.69 | 6.68e-05 |
| Genes significantly enriched (> 2x, FDR < 5%) in a particular cell-type versus a reference sample of all cells at the same stage. WBPaper00037950:glr-1(+)-neurons\_larva\_enriched | 22 | 3.55 | 9.67e-05 |
| Larval A-class motor neuron enriched genes. | 17 | 4.49 | 1.04e-04 |
| Genes significantly enriched (> 2x, FDR < 5%) in a particular cell-type versus a reference sample of all cells at the same stage. WBPaper00037950:dopaminergic-neurons\_larva\_enriched | 28 | 2.43 | 2.88e-03 |
| Genes with expression enriched in PVD and OLL neurons. Data sets were normalized by RMA and transcripts showing relative PVD enrichment (>= 1.5X) vs. the reference sample were identified by SAM analysis (False Discovery Rate, FDR < 1%). | 39 | 1.91 | 9.95e-03 |

### Motifs enriched

|  |  |  |  |  |  |
| --- | --- | --- | --- | --- | --- |
| **Motif** | **Logo** | **Possible orthologs** | **Number of motifs in cluster** | **Enrichment** | **FDR corrected p** |
| MA0543.1 |  | daf-8 eor-1 | 75 | 2.11 | 2.7e-09 |
| V$NGFIC\_01 |  | klf-2 (-0.51) klf-1 ZC328.2 | 80 | 1.76 | 1.7e-06 |
| MA0537.1 |  | blmp-1 | 124 | 1.35 | 1.9e-06 |
| NR2F6\_f1 |  | nhr-62 nhr-2 nhr-239 | 104 | 1.50 | 2.7e-06 |
| FOXF2\_f1 |  | let-381 lin-31 | 96 | 1.57 | 3.2e-06 |
| ONECUT2\_1 |  | ceh-48 (0.74) dsc-1 | 75 | 1.79 | 3.7e-06 |
| pTH9180 |  | mel-28 mef-2 let-381 Y61A9LA.9 Y116A8C.22 | 101 | 1.52 | 3.8e-06 |
| pTH2673 |  | fkh-10 (0.63) let-381 lin-31 C34D1.1 | 112 | 1.43 | 4.6e-06 |
| MA0497.1 |  | mef-2 | 119 | 1.37 | 4.8e-06 |
| klu\_SOLEXA\_5\_FBgn0013469 |  | ZC328.2 daf-16 | 79 | 1.73 | 4.8e-06 |
| V$FOXO1\_01 |  | fkh-7 (-0.67) fkh-8 (0.67) fkh-10 (0.63) pha-4 (-0.57) let-381 lin-31 daf-16 | 77 | 1.75 | 4.8e-06 |
| PURA\_f1 |  | Y53H1A.2 (0.74) klf-2 (-0.51) plp-2 | 88 | 1.62 | 6.0e-06 |
| Hgtx\_Cell\_FBgn0040318 |  | ceh-31 (0.75) lim-4 (0.74) pha-2 (-0.61) ceh-30 (0.56) ceh-23 ceh-53 ceh-16 ceh-18 ceh-10 pal-1 ceh-36 ceh-12 ceh-45 ceh-14 ceh-2 mls-2 lim-7 alr-1 cog-1 lin-39 and 8 others  [full list] | 65 | 1.91 | 6.2e-06 |
| CG31670\_SOLEXA\_5\_FBgn0031375 |  | F21A9.2 CELE\_Y38H8A.5 | 116 | 1.38 | 1.0e-05 |
| SP3\_f1 |  | klf-2 (-0.51) klf-1 | 81 | 1.66 | 1.5e-05 |
| pTH9254 |  | mel-28 | 122 | 1.33 | 1.6e-05 |
| V$CART1\_01 |  | ceh-18 pal-1 alr-1 lin-39 ceh-43 | 83 | 1.63 | 1.8e-05 |
| MSX2\_f1 |  | eyg-1 ceh-10 ceh-45 alr-1 lin-39 ceh-1 | 100 | 1.48 | 1.8e-05 |
| V$ZIC1\_01 |  | ref-2 pax-3 ztf-14 | 70 | 1.78 | 1.9e-05 |
| V$FOXO3\_01 |  | fkh-7 (-0.67) fkh-8 (0.67) fkh-10 (0.63) let-381 lin-31 daf-16 | 47 | 2.22 | 1.9e-05 |
| MA0579.1 |  | D1081.8 F45H11.6 | 56 | 2.01 | 1.9e-05 |
| pTH6003 |  | nhr-182 nhr-134 | 59 | 1.95 | 2.1e-05 |
| MA0007.2 |  | npax-1 (0.6) nhr-255 lin-14 | 55 | 2.02 | 2.2e-05 |
| V$XFD3\_01 |  | ceh-20 let-381 cfi-1 | 112 | 1.39 | 2.5e-05 |
| ZN384\_f1 |  | lin-29 K11D2.4 | 113 | 1.38 | 2.6e-05 |
| HOXC10\_2 |  | ceh-24 (0.66) pal-1 php-3 lin-39 | 104 | 1.44 | 3.2e-05 |
| pTH5166 |  | ces-2 (-0.56) atf-2 C48E7.11 F23F12.9 Y51H4A.4 | 70 | 1.75 | 3.3e-05 |
| HXD10\_f1 |  | php-3 nhr-2 | 78 | 1.66 | 3.5e-05 |
| MA0500.1 |  | hlh-11 lin-32 hlh-1 hlh-14 hlh-15 | 48 | 2.14 | 3.7e-05 |
| MA0039.2 |  | klf-2 (-0.51) klf-1 | 78 | 1.65 | 4.0e-05 |
| V$CEBP\_01 |  | C48E7.11 | 97 | 1.48 | 4.2e-05 |
| Mw154 |  | ceh-12 ceh-20 lin-39 | 103 | 1.44 | 4.2e-05 |
| pTH9242 |  | mel-28 | 122 | 1.31 | 4.5e-05 |
| MA0483.1 |  | odd-1 lim-6 | 97 | 1.48 | 4.9e-05 |
| MA0124.1 |  | ceh-48 (0.74) ceh-24 (0.66) | 79 | 1.63 | 5.0e-05 |
| POU3F4\_2 |  | ceh-18 unc-86 | 92 | 1.51 | 5.2e-05 |
| MA0482.1 |  | elt-1 | 93 | 1.51 | 5.5e-05 |
| pTH10623 |  | scrt-1 | 80 | 1.62 | 5.6e-05 |
| pTH5117 |  | cfi-1 | 69 | 1.74 | 5.9e-05 |
| MA0060.2 |  | nfya-2 (0.53) dro-1 ceh-20 lin-31 | 84 | 1.58 | 6.0e-05 |
| V$ZID\_01 |  | ztf-28 | 78 | 1.64 | 6.1e-05 |
| pTH6591 |  | lin-31 | 74 | 1.67 | 6.7e-05 |
| pTH1001 |  | dnj-17 | 79 | 1.62 | 7.4e-05 |
| pTH9082 |  | mab-23 | 108 | 1.39 | 8.4e-05 |
| FOXO1\_si |  | irx-1 fkh-9 daf-16 | 95 | 1.48 | 8.6e-05 |
| ARI3A\_f1 |  | alr-1 cfi-1 ZC204.2 | 67 | 1.74 | 8.8e-05 |
| pTH9384 |  | cfi-1 | 108 | 1.38 | 9.0e-05 |
| cad\_FlyReg\_FBgn0000251 |  | ceh-24 (0.66) pal-1 php-3 lin-39 ceh-13 D1005.3 | 106 | 1.40 | 9.3e-05 |
| pTH10769 |  | Y48G1C.6 | 87 | 1.53 | 1.1e-04 |
| pTH6486 |  | nhr-145 (0.61) | 97 | 1.45 | 1.2e-04 |
| pTH8982 |  | ceh-48 (0.74) | 88 | 1.52 | 1.3e-04 |
| MA0452.2 |  | B0310.2 | 85 | 1.54 | 1.4e-04 |
| sqz\_SANGER\_5\_FBgn0010768 |  | fkh-7 (-0.67) lin-29 mel-28 | 109 | 1.37 | 1.4e-04 |
| Hoxc11\_3718 |  | ceh-24 (0.66) pal-1 | 87 | 1.52 | 1.5e-04 |
| pTH9911 |  | fos-1 atf-5 crh-1 | 79 | 1.59 | 1.6e-04 |
| Bsh\_Cell\_FBgn0000529 |  | ceh-31 (0.75) ceh-24 (0.66) ceh-9 (0.62) ceh-30 (0.56) ceh-8 (0.53) lim-7 alr-1 cog-1 lin-39 ceh-19 ceh-1 ceh-43 | 80 | 1.58 | 1.6e-04 |
| pTH9279 |  | Y116A8C.22 | 108 | 1.37 | 1.7e-04 |
| MA0014.2 |  | pax-3 pax-2 | 74 | 1.63 | 1.7e-04 |
| pTH8399 |  | lin-54 | 103 | 1.40 | 1.8e-04 |
| pTH10714 |  | nhr-142 nhr-216 nhr-84 | 87 | 1.51 | 2.0e-04 |
| TBX2\_f1 |  | tbx-39 | 100 | 1.41 | 2.3e-04 |
| pTH3477 |  | daf-16 | 95 | 1.44 | 2.3e-04 |
| TBX1\_1 |  | mab-9 tbx-39 | 98 | 1.42 | 2.4e-04 |
| Nkx3-1\_2923 |  | ceh-24 (0.66) dsc-1 | 75 | 1.60 | 2.5e-04 |
| FLI1\_f1 |  | lin-1 | 79 | 1.56 | 2.7e-04 |
| Smad3\_3805 |  | daf-8 | 80 | 1.56 | 2.7e-04 |
| pTH9118 |  | eor-1 egrh-3 | 62 | 1.75 | 2.7e-04 |
| pTH5119 |  | cfi-1 | 103 | 1.39 | 2.7e-04 |
| pTH4425 |  | php-3 lim-7 lin-39 cfi-1 | 99 | 1.41 | 3.1e-04 |
| Antp\_FlyReg\_FBgn0000095 |  | ceh-53 hmg-12 let-381 lin-39 lin-31 hmbx-1 ceh-43 Y116A8C.22 | 99 | 1.41 | 3.1e-04 |
| pnr\_SANGER\_5\_FBgn0003117 |  | elt-1 | 114 | 1.32 | 3.2e-04 |
| pTH9222 |  | mel-28 | 76 | 1.58 | 3.3e-04 |
| pTH9137 |  | nhr-65 | 112 | 1.33 | 3.3e-04 |
| Hoxd13\_2356 |  | pal-1 | 87 | 1.49 | 3.4e-04 |
| Mafk\_3106 |  | F45H11.6 | 101 | 1.39 | 3.8e-04 |
| HXD9\_f1 |  | hbl-1 php-3 lin-39 | 81 | 1.53 | 3.8e-04 |
| pTH9900 |  | C46E10.8 | 54 | 1.83 | 4.3e-04 |
| SRP000712\_Sox2 |  | sox-4 (0.68) ceh-18 ceh-6 | 88 | 1.47 | 4.4e-04 |
| MA0536.1 |  | elt-1 | 57 | 1.78 | 4.6e-04 |
| V$CDC5\_01 |  | irx-1 D1081.8 | 75 | 1.58 | 4.7e-04 |
| NR2E3\_f1 |  | nhr-100 (0.53) lin-1 lin-39 | 107 | 1.35 | 5.1e-04 |
| rn\_SOLEXA\_5\_FBgn0259172 |  | lin-29 | 33 | 2.35 | 5.7e-04 |
| Mw140 |  | efl-1 F49E12.6 | 86 | 1.48 | 5.7e-04 |
| MA0538.1 |  | ceh-9 (0.62) daf-12 hif-1 ztf-3 | 85 | 1.48 | 5.8e-04 |
| pTH3046 |  | Y116A8C.22 | 84 | 1.49 | 5.9e-04 |
| pTH9142 |  | C34D1.1 gei-11 | 99 | 1.39 | 6.2e-04 |
| TBX3\_f1 |  | ceh-45 tbx-39 | 91 | 1.44 | 6.4e-04 |
| OLIG3\_1 |  | hlh-8 hlh-32 lin-31 hlh-15 | 72 | 1.59 | 6.5e-04 |
| ATF1\_si |  | crh-1 | 76 | 1.55 | 6.7e-04 |
| Sox1\_2631 |  | sox-4 (0.68) | 65 | 1.66 | 6.8e-04 |
| pTH6641 |  | lin-31 | 69 | 1.62 | 6.8e-04 |
| V$CEBPA\_01 |  | C48E7.11 | 81 | 1.51 | 6.8e-04 |
| pTH5118 |  | cfi-1 | 78 | 1.53 | 6.9e-04 |
| V$RFX1\_02 |  | daf-19 (0.76) | 67 | 1.64 | 7.0e-04 |
| V$FOXJ2\_02 |  | lin-31 | 101 | 1.37 | 7.1e-04 |
| MA0535.1 |  | daf-8 pax-2 F45H11.6 | 72 | 1.57 | 8.7e-04 |
| pTH10633 |  | R07H5.10 C48E7.11 | 72 | 1.57 | 8.8e-04 |
| MA0498.1 |  | ceh-32 lin-39 | 72 | 1.57 | 8.8e-04 |
| pTH2846 |  | lin-31 | 104 | 1.35 | 9.1e-04 |
| Pbx1\_3203 |  | ceh-20 | 88 | 1.45 | 9.3e-04 |
| EMX2\_2 |  | ceh-2 | 92 | 1.42 | 9.9e-04 |
| pTH9951 |  | pal-1 mex-6 | 100 | 1.37 | 1.0e-03 |
| pTH9925 |  | ztf-11 | 74 | 1.55 | 1.0e-03 |
| pTH5423 |  | klf-2 (-0.51) | 74 | 1.55 | 1.0e-03 |
| Ceh-22 |  | ceh-24 (0.66) ceh-22 dsc-1 | 75 | 1.54 | 1.1e-03 |
| pTH9044 |  | mbr-1 (0.64) nhr-177 F26F4.8 bed-3 | 79 | 1.51 | 1.1e-03 |
| SOX10\_1 |  | sox-4 (0.68) egl-13 (0.51) | 104 | 1.34 | 1.1e-03 |
| En2\_0952 |  | ceh-24 (0.66) ceh-16 lim-7 lim-6 | 46 | 1.91 | 1.1e-03 |
| pTH3086 |  | klf-2 (-0.51) klf-1 sptf-3 | 73 | 1.55 | 1.1e-03 |
| Sox17\_2837 |  | sox-4 (0.68) | 102 | 1.35 | 1.1e-03 |
| pTH5250 |  | C48E7.11 | 78 | 1.51 | 1.2e-03 |
| V$TCF11\_01 |  | skn-1 | 83 | 1.47 | 1.2e-03 |
| Hoxa9\_2622 |  | php-3 lin-39 | 73 | 1.55 | 1.2e-03 |
| NDF1\_f1 |  | hlh-8 ngn-1 hlh-32 hlh-12 hlh-15 | 78 | 1.51 | 1.3e-03 |
| GRHL1\_2 |  | grh-1 | 64 | 1.63 | 1.3e-03 |
| RFX4\_2 |  | daf-19 (0.76) | 56 | 1.73 | 1.3e-03 |
| pTH6497 |  | lin-31 | 39 | 2.05 | 1.4e-03 |
| pTH9177 |  | F10B5.3 | 98 | 1.37 | 1.4e-03 |
| BARHL2\_3 |  | ceh-31 (0.75) lin-39 ceh-1 ceh-43 | 83 | 1.47 | 1.4e-03 |
| Egr1\_2580 |  | ZC328.2 | 71 | 1.56 | 1.5e-03 |
| Rfxdc2\_3516 |  | daf-19 (0.76) mab-3 | 61 | 1.66 | 1.6e-03 |
| BSX\_1 |  | ceh-31 (0.75) ceh-9 (0.62) ceh-16 lim-7 alr-1 lin-39 ceh-1 ceh-43 | 43 | 1.94 | 1.6e-03 |
| MA0067.1 |  | hlh-30 pax-1 aha-1 | 76 | 1.51 | 1.6e-03 |
| pTH6106 |  | nhr-182 | 78 | 1.50 | 1.6e-03 |
| pTH9335 |  | mel-28 | 110 | 1.30 | 1.7e-03 |
| Tcf7l2\_3461 |  | pop-1 (-0.54) ceh-20 | 98 | 1.36 | 1.7e-03 |
| pTH5916 |  | efl-2 | 64 | 1.61 | 1.8e-03 |
| Dlx5\_3419 |  | ceh-18 ceh-12 ceh-45 alr-1 npax-3 ceh-1 ceh-43 | 76 | 1.51 | 1.8e-03 |
| pTH9915 |  | zip-3 | 78 | 1.49 | 1.8e-03 |
| pTH10696 |  | Y44A6D.3 | 57 | 1.69 | 1.8e-03 |
| SOX2\_4 |  | sox-4 (0.68) | 99 | 1.35 | 1.9e-03 |
| Hr46\_FlyReg\_FBgn0000448 |  | nhr-213 lin-31 | 23 | 2.72 | 1.9e-03 |
| Irx3\_2226 |  | irx-1 | 63 | 1.62 | 2.0e-03 |
| V$FAC1\_01 |  | gei-8 | 68 | 1.57 | 2.0e-03 |
| pTH9059 |  | ztf-28 | 49 | 1.80 | 2.1e-03 |
| V$BRN2\_01 |  | ceh-18 | 109 | 1.30 | 2.2e-03 |
| pTH10630 |  | lsy-27 | 78 | 1.48 | 2.2e-03 |
| pTH9135 |  | pop-1 (-0.54) | 70 | 1.55 | 2.2e-03 |
| pTH9353 |  | ceh-51 | 27 | 2.43 | 2.2e-03 |
| pTH8863 |  | hmg-12 | 85 | 1.43 | 2.3e-03 |
| Plagl1\_0972 |  | Y53H1A.2 (0.74) | 48 | 1.80 | 2.4e-03 |
| Irx5\_2385 |  | irx-1 | 72 | 1.52 | 2.4e-03 |
| MA0495.1 |  | jun-1 sknr-1 fos-1 crh-1 F45H11.6 | 93 | 1.38 | 2.6e-03 |
| Poxm\_SOLEXA\_5\_FBgn0003129 |  | pax-2 | 67 | 1.56 | 2.7e-03 |
| pTH10647 |  | nhr-232 | 88 | 1.41 | 2.7e-03 |
| pTH5257 |  | C48E7.11 | 79 | 1.46 | 2.8e-03 |
| pTH9709 |  | die-1 | 106 | 1.30 | 2.8e-03 |
| pTH3037 |  | hlh-1 hlh-15 | 66 | 1.57 | 2.9e-03 |
| pTH5914 |  | attf-1 | 56 | 1.67 | 2.9e-03 |
| MA0161.1 |  | nfi-1 F49E12.6 | 69 | 1.54 | 2.9e-03 |
| Mcm1 |  | unc-120 | 76 | 1.48 | 2.9e-03 |
| V$OCT1\_06 |  | ztf-9 (0.66) ceh-18 | 105 | 1.31 | 3.0e-03 |
| SMAD3\_f1 |  | daf-8 | 63 | 1.59 | 3.1e-03 |
| pTH6445 |  | ceh-5 | 87 | 1.41 | 3.2e-03 |
| Gmeb1\_1745 |  | attf-1 | 52 | 1.72 | 3.3e-03 |
| pTH10013 |  | nhr-168 | 88 | 1.40 | 3.4e-03 |
| pTH10837 |  | ces-1 T22H9.4 | 101 | 1.32 | 3.6e-03 |
| pTH10718 |  | egl-43 | 73 | 1.49 | 3.8e-03 |
| V$AP2REP\_01 |  | klf-1 ZC328.2 | 60 | 1.61 | 3.8e-03 |
| pTH9043 |  | sem-2 (-0.56) | 63 | 1.58 | 3.9e-03 |
| pTH9879 |  | hlh-30 C27D6.4 | 63 | 1.58 | 3.9e-03 |
| pTH6327 |  | dsc-1 | 74 | 1.48 | 4.0e-03 |
| V$GR\_Q6 |  | nhr-255 | 84 | 1.41 | 4.0e-03 |
| MA0474.1 |  | lin-1 C24A1.2 | 95 | 1.35 | 4.0e-03 |
| CrebA\_SANGER\_5\_FBgn0004396 |  | atf-6 fos-1 atf-7 C27D6.4 | 70 | 1.51 | 4.1e-03 |
| Pou2f2\_3748 |  | ceh-18 alr-1 | 80 | 1.44 | 4.2e-03 |
| Hmbox1\_2674 |  | hmbx-1 | 62 | 1.58 | 4.3e-03 |
| HLH4C\_da\_SANGER\_5\_FBgn0011277 |  | hlh-2 ces-1 hlh-1 hlh-15 | 78 | 1.45 | 4.3e-03 |
| ARI3A\_do |  | gei-3 (0.65) lim-6 cfi-1 | 94 | 1.35 | 4.4e-03 |
| pTH9189 |  | dmd-3 ceh-18 | 106 | 1.29 | 4.4e-03 |
| pTH10816 |  | dmd-6 | 90 | 1.37 | 4.4e-03 |
| HeLa-S3\_ZNF274\_UCD |  | C28G1.4 | 90 | 1.37 | 4.4e-03 |
| pTH1292 |  | ceh-24 (0.66) pzf-1 | 84 | 1.41 | 4.5e-03 |
| MA0262.1 |  | mab-3 hsf-1 | 51 | 1.71 | 4.6e-03 |
| Mw145 |  | ceh-34 (-0.56) ceh-32 elt-3 elt-6 elt-1 elt-7 egl-27 | 84 | 1.41 | 4.8e-03 |
| Sox8\_1733 |  | sox-4 (0.68) gei-3 (0.65) pop-1 (-0.54) | 86 | 1.40 | 4.8e-03 |
| Hoxd11\_3873 |  | php-3 ceh-13 | 74 | 1.47 | 4.8e-03 |
| V$S8\_01 |  | ceh-45 | 84 | 1.41 | 4.8e-03 |
| HES1\_f1 |  | lin-22 | 63 | 1.57 | 4.8e-03 |
| pTH8216 |  | Y116A8C.22 | 73 | 1.48 | 4.9e-03 |
| Jundm2\_0911 |  | fos-1 | 76 | 1.46 | 5.0e-03 |
| PTF1A\_f1 |  | lin-32 | 86 | 1.39 | 5.1e-03 |
| Zfp161\_2858 |  | pzf-1 | 60 | 1.59 | 5.1e-03 |
| Vsx1\_1728 |  | alr-1 | 75 | 1.46 | 5.2e-03 |
| MA0547.1 |  | ceh-2 skn-1 | 84 | 1.40 | 5.3e-03 |
| MA0095.2 |  | lsy-2 | 91 | 1.36 | 5.3e-03 |
| Spt15 |  | tbp-1 | 56 | 1.63 | 5.5e-03 |
| TBP\_f1 |  | tbp-1 | 86 | 1.39 | 5.5e-03 |
| Hoxb7\_3953 |  | lin-39 | 75 | 1.46 | 5.9e-03 |
| pnt\_SANGER\_5\_FBgn0003118 |  | lin-1 C24A1.2 | 96 | 1.33 | 5.9e-03 |
| pTH5922 |  | ceh-24 (0.66) | 63 | 1.55 | 5.9e-03 |
| GM12878\_ETS1\_HudsonAlpha |  | nhr-79 lin-1 tbx-39 | 72 | 1.48 | 6.1e-03 |
| OTX2\_si |  | pha-2 (-0.61) dve-1 ceh-53 ceh-45 | 79 | 1.43 | 6.2e-03 |
| MA0544.1 |  | lin-22 gei-11 | 59 | 1.59 | 6.3e-03 |
| CG8765\_SANGER\_5\_FBgn0036900 |  | H20J04.3 | 94 | 1.34 | 6.4e-03 |
| Pou3f3\_3235 |  | ceh-6 | 77 | 1.44 | 6.6e-03 |
| Nkx6-1\_2825 |  | cog-1 | 48 | 1.72 | 6.6e-03 |
| MITF\_f1 |  | irx-1 hlh-30 mxl-1 | 73 | 1.46 | 6.7e-03 |
| pTH9108 |  | nhr-5 daf-12 | 79 | 1.42 | 6.7e-03 |
| Hoxa11\_2218 |  | php-3 | 79 | 1.42 | 6.8e-03 |
| MA0163.1 |  | Y53H1A.2 (0.74) | 56 | 1.62 | 6.8e-03 |
| Mf28 |  | elt-1 | 83 | 1.40 | 6.8e-03 |
| Prop1\_3949 |  | ceh-53 ceh-16 | 70 | 1.49 | 6.8e-03 |
| pTH9237 |  | mel-28 | 102 | 1.30 | 6.9e-03 |
| CG4854\_SANGER\_10\_FBgn0038766 |  | K11D2.4 | 79 | 1.42 | 7.0e-03 |
| PBDE\_GATA1\_UCD |  | elt-1 | 80 | 1.42 | 7.0e-03 |
| pTH9173 |  | efl-2 | 60 | 1.57 | 7.1e-03 |
| pTH9250 |  | dmd-3 C34D1.1 | 73 | 1.46 | 7.6e-03 |
| Dlx2\_2273 |  | ceh-43 | 75 | 1.44 | 7.8e-03 |
| V$SRF\_C |  | unc-120 | 66 | 1.51 | 7.9e-03 |
| pTH8649 |  | mbr-1 (0.64) | 91 | 1.35 | 8.1e-03 |
| Cdx1\_2245 |  | ceh-13 | 83 | 1.39 | 8.3e-03 |
| pTH5924 |  | nhr-255 | 67 | 1.50 | 8.3e-03 |
| HepG2\_HSF1\_Stanford |  | Y53C10A.3 | 28 | 2.17 | 8.4e-03 |
| V$CREB\_Q4 |  | crh-1 W08E12.1 attf-1 | 75 | 1.44 | 8.5e-03 |
| Atf1\_3026 |  | crh-1 | 70 | 1.47 | 8.7e-03 |
| pTH9924 |  | nhr-46 | 94 | 1.33 | 8.9e-03 |
| pTH9247 |  | dmd-3 C34D1.1 | 88 | 1.36 | 9.3e-03 |
| pTH6508 |  | nhr-36 (0.6) | 41 | 1.80 | 9.6e-03 |
| pTH9297 |  | ceh-18 | 88 | 1.36 | 9.7e-03 |
| Srf\_3509 |  | unc-120 | 57 | 1.58 | 9.8e-03 |
| pTH10041 |  | ztf-29 | 86 | 1.36 | 1.0e-02 |
| MA0139.1 |  | Y5F2A.4 F58G1.2 | 68 | 1.48 | 1.1e-02 |
| pTH5812 |  | ceh-14 | 65 | 1.50 | 1.1e-02 |
| pTH3751 |  | tbx-39 | 69 | 1.47 | 1.1e-02 |
| pTH5887 |  | lin-39 | 45 | 1.71 | 1.1e-02 |
| FLI1\_4 |  | lin-1 | 68 | 1.47 | 1.2e-02 |
| pTH9958 |  | ztf-6 | 78 | 1.40 | 1.2e-02 |
| pTH9393 |  | F39B2.1 ZC416.1 | 59 | 1.54 | 1.2e-02 |
| pTH9260 |  | mel-28 | 69 | 1.46 | 1.3e-02 |
| Tcf1\_2666 |  | hmbx-1 | 57 | 1.56 | 1.3e-02 |
| pTH9381 |  | ceh-18 | 83 | 1.37 | 1.3e-02 |
| pTH9073 |  | end-3 elt-1 | 81 | 1.38 | 1.4e-02 |
| V$IK2\_01 |  | F26F4.8 | 62 | 1.51 | 1.4e-02 |
| V$FREAC7\_01 |  | lin-31 | 71 | 1.44 | 1.4e-02 |
| Irx3\_1 |  | irx-1 | 67 | 1.47 | 1.4e-02 |
| pTH9220 |  | mbr-1 (0.64) | 61 | 1.52 | 1.4e-02 |
| pTH10038 |  | sox-4 (0.68) gei-3 (0.65) F56D1.1 | 59 | 1.54 | 1.4e-02 |
| V$GATA6\_01 |  | elt-1 | 79 | 1.39 | 1.4e-02 |
| Nkx6-3\_3446 |  | cog-1 | 45 | 1.70 | 1.4e-02 |
| Cdx2\_4272 |  | ceh-13 | 72 | 1.43 | 1.4e-02 |
| MA0015.1 |  | che-1 | 58 | 1.54 | 1.5e-02 |
| MA0456.1 |  | ref-2 T22C8.4 | 50 | 1.62 | 1.5e-02 |
| MCR\_f1 |  | nhr-255 | 81 | 1.37 | 1.5e-02 |
| V$ARP1\_01 |  | nhr-62 nhr-2 | 36 | 1.84 | 1.5e-02 |
| Tcf2\_0913 |  | hmbx-1 | 57 | 1.54 | 1.6e-02 |
| Hoxa10\_2318 |  | ceh-24 (0.66) | 70 | 1.44 | 1.6e-02 |
| MA0016.1 |  | nhr-69 | 58 | 1.53 | 1.6e-02 |
| FOXB1\_1 |  | lin-31 | 68 | 1.45 | 1.7e-02 |
| pTH2283 |  | odd-2 (-0.53) | 81 | 1.37 | 1.7e-02 |
| pTH5919 |  | irx-1 | 60 | 1.51 | 1.7e-02 |
| pTH3819 |  | ceh-18 | 44 | 1.69 | 1.8e-02 |
| Sox4 |  | pop-1 (-0.54) nhr-100 (0.53) | 99 | 1.28 | 1.8e-02 |
| pTH9216 |  | ceh-18 | 59 | 1.52 | 1.8e-02 |
| pTH10654 |  | lsy-27 ceh-90 | 44 | 1.68 | 1.9e-02 |
| pTH9182 |  | tbx-39 | 64 | 1.47 | 2.0e-02 |
| MA0027.1 |  | ceh-16 | 72 | 1.41 | 2.0e-02 |
| pTH9245 |  | ceh-18 | 84 | 1.35 | 2.0e-02 |
| pTH9096 |  | T07C12.11 | 47 | 1.64 | 2.0e-02 |
| Lhx1\_2240 |  | lim-7 | 76 | 1.39 | 2.0e-02 |
| Nkx1-2\_3214 |  | ceh-30 (0.56) | 64 | 1.47 | 2.1e-02 |
| Evx1\_3952 |  | ceh-53 | 23 | 2.22 | 2.1e-02 |
| pTH9326 |  | nhr-122 (-0.67) | 76 | 1.38 | 2.2e-02 |
| Irx2\_0900 |  | irx-1 | 66 | 1.45 | 2.2e-02 |
| V$HOX13\_01 |  | lin-39 | 91 | 1.31 | 2.2e-02 |
| Hoxc8\_3429 |  | lin-39 | 73 | 1.40 | 2.2e-02 |
| pTH2280 |  | mnm-2 | 49 | 1.60 | 2.3e-02 |
| Pknox2\_3077 |  | ceh-32 | 70 | 1.42 | 2.4e-02 |
| MA0146.2 |  | F58G1.2 | 42 | 1.69 | 2.4e-02 |
| Dlx1\_1741 |  | ceh-43 | 44 | 1.66 | 2.4e-02 |
| V$YY1\_01 |  | lsy-2 | 74 | 1.39 | 2.4e-02 |
| CXXC1\_si |  | F52B11.1 | 66 | 1.44 | 2.5e-02 |
| Dlx3\_1030 |  | ceh-43 | 40 | 1.71 | 2.5e-02 |
| GATA5\_f1 |  | nhr-7 (-0.57) nhr-100 (0.53) elt-1 | 86 | 1.32 | 2.6e-02 |
| pTH8745 |  | attf-1 | 46 | 1.62 | 2.6e-02 |
| Irx3\_0920 |  | irx-1 | 55 | 1.53 | 2.6e-02 |
| pTH4325 |  | ceh-18 | 92 | 1.30 | 2.6e-02 |
| ARNT2\_si |  | aha-1 | 67 | 1.43 | 2.7e-02 |
| Mv102 |  | nhr-2 nhr-71 | 78 | 1.36 | 2.7e-02 |
| I$MTTFA\_01 |  | hmg-5 | 73 | 1.39 | 2.8e-02 |
| Hoxd1\_3448 |  | ceh-12 | 64 | 1.45 | 2.8e-02 |
| Lbx2\_3869 |  | mls-2 | 61 | 1.47 | 2.9e-02 |
| pTH10650 |  | nhr-153 | 77 | 1.36 | 3.0e-02 |
| exd\_SOLEXA\_2\_FBgn0000611 |  | ceh-20 F55C5.11 | 32 | 1.85 | 3.0e-02 |
| pTH3997 |  | C04F5.9 | 56 | 1.51 | 3.0e-02 |
| ELF3\_2 |  | C24A1.2 | 71 | 1.39 | 3.1e-02 |
| HAND1\_si |  | sma-4 hlh-8 | 75 | 1.37 | 3.2e-02 |
| V$OCT1\_03 |  | ceh-18 | 72 | 1.39 | 3.2e-02 |
| V$GATA1\_02 |  | elt-1 | 29 | 1.92 | 3.2e-02 |
| pTH6636 |  | egl-5 | 49 | 1.57 | 3.3e-02 |
| pTH3064 |  | crh-1 | 35 | 1.77 | 3.3e-02 |
| pTH6478 |  | lim-7 | 27 | 1.97 | 3.3e-02 |
| ETS2\_f1 |  | lin-1 | 46 | 1.60 | 3.4e-02 |
| Tcf7\_0950 |  | pop-1 (-0.54) | 79 | 1.35 | 3.4e-02 |
| pTH6612 |  | nhr-213 | 69 | 1.40 | 3.4e-02 |
| pTH3998 |  | tbx-39 | 66 | 1.42 | 3.5e-02 |
| pTH9387 |  | C34D1.1 | 45 | 1.61 | 3.5e-02 |
| pTH8991 |  | cey-3 | 60 | 1.46 | 3.6e-02 |
| T-47D\_GATA3\_HudsonAlpha |  | elt-1 | 82 | 1.33 | 3.6e-02 |
| pTH5778 |  | egl-5 | 71 | 1.39 | 3.6e-02 |
| Tcf3\_3787 |  | pop-1 (-0.54) | 82 | 1.32 | 3.9e-02 |
| pTH7875 |  | mel-28 | 84 | 1.31 | 3.9e-02 |
| ZNF75A\_1 |  | ztf-3 | 51 | 1.53 | 4.0e-02 |
| Hnf4a\_2640 |  | nhr-62 | 41 | 1.64 | 4.3e-02 |
| HLH25 |  | hlh-27 | 40 | 1.65 | 4.6e-02 |
| V$AHR\_01 |  | ahr-1 | 60 | 1.44 | 4.6e-02 |
| Hoxb8\_3780 |  | lin-39 | 69 | 1.38 | 4.7e-02 |
| Hoxa7\_3750 |  | lin-39 | 71 | 1.37 | 4.8e-02 |
| V$NCX\_01 |  | ceh-19 | 64 | 1.41 | 4.9e-02 |
| Vax2\_3500 |  | C02F12.10 | 21 | 2.14 | 4.9e-02 |

### Correlated (and anti-correlated) transcription factors

|  |  |
| --- | --- |
| **Transcription factor** | **Correlation** |
| unc-42 | 0.89 |
| madf-5 | 0.87 |
| zip-4 | 0.85 |
| ceh-74 | 0.83 |
| ceh-88 | 0.81 |
| Y17G7B.22 | 0.80 |
| nhr-95 | 0.79 |
| nhr-190 | 0.78 |
| W04B5.2 | 0.78 |
| ast-1 | 0.77 |
| daf-19 | 0.76 |
| ceh-31 | 0.75 |
| lim-4 | 0.74 |
| aptf-4 | 0.74 |
| Y53H1A.2 | 0.74 |
| C09F5.3 | 0.74 |
| ceh-48 | 0.74 |
| C34F6.9 | 0.72 |
| ctbp-1 | 0.71 |
| W02D7.6 | 0.71 |
| nhr-47 | 0.71 |
| mbl-1 | 0.70 |
| dmd-10 | 0.70 |
| ceh-54 | 0.70 |
| C02F5.12 | 0.70 |
| nhr-104 | -0.57 |
| pha-4 | -0.57 |
| nhr-60 | -0.57 |
| nhr-7 | -0.57 |
| snpc-1.2 | -0.57 |
| ham-2 | -0.58 |
| nhr-70 | -0.58 |
| ceh-60 | -0.58 |
| nhr-173 | -0.58 |
| attf-3 | -0.58 |
| nhr-64 | -0.59 |
| ztf-7 | -0.60 |
| ham-1 | -0.60 |
| sup-35 | -0.61 |
| pha-2 | -0.61 |
| bed-2 | -0.64 |
| Y48A6C.1 | -0.64 |
| nhr-13 | -0.65 |
| sbp-1 | -0.67 |
| fkh-7 | -0.67 |
| nhr-35 | -0.67 |
| nhr-106 | -0.67 |
| nhr-122 | -0.67 |
| nhr-88 | -0.67 |
| duxl-1 | -0.69 |

### ChIP peaks enriched

|  |  |  |  |  |
| --- | --- | --- | --- | --- |
| **Gene** | **Experiment** | **Number of upstream peaks** | **Enrichment** | **FDR corrected p** |
| ces-1 | CES-1\_Embryos | 97 | 2.97 | 5.8e-27 |
| C34F6.9 | C34F6.9\_Larvae-L2-stage | 86 | 2.65 | 4.4e-19 |
| alr-1 | ALR-1\_Larvae-L2-stage | 78 | 2.59 | 6.3e-16 |
| ham-1 | HAM-1\_Larvae-L4-stage | 84 | 2.37 | 2.5e-15 |
| F45C12.2 | F45C12.2\_Fed-L1-stage-larvae | 74 | 2.64 | 3.3e-15 |
| eor-1 | EOR-1\_Larvae-L3-stage | 80 | 2.40 | 1.4e-14 |
| nfya-1 | NFYA-1\_Late-Embryos | 70 | 2.49 | 9.1e-13 |
| lsy-2 | LSY-2\_Larvae-L1-stage | 86 | 2.11 | 1.0e-12 |
| sem-4 | SEM-4\_Larvae-L2-stage | 77 | 2.28 | 1.4e-12 |
| gei-11 | GEI-11\_Larvae-L3-stage | 62 | 2.63 | 6.2e-12 |
| zag-1 | ZAG-1\_Larvae-L2-stage | 53 | 3.00 | 6.8e-12 |
| ham-1 | HAM-1\_Fed-L1-stage-larvae | 65 | 2.49 | 1.4e-11 |
| dpl-1 | DPL-1\_Larvae-L4-stage | 74 | 2.23 | 2.4e-11 |
| ces-1 | CES-1\_Fed-L1-stage-larvae | 45 | 3.33 | 2.9e-11 |
| ceh-38 | CEH-38\_Larvae-L4-stage | 41 | 3.60 | 4.0e-11 |
| gei-11 | GEI-11\_Larvae-L2-stage | 50 | 2.89 | 1.7e-10 |
| lin-35 | LIN-35\_Fed-L1-stage-larvae | 57 | 2.58 | 2.2e-10 |
| nhr-129 | NHR-129\_Larvae-L2-stage | 75 | 2.10 | 3.3e-10 |
| nfya-1 | NFYA-1\_Larvae-L3-stage | 53 | 2.67 | 5.8e-10 |
| fos-1 | FOS-1\_Fed-L1-stage-larvae | 68 | 2.20 | 7.9e-10 |
| sea-2 | SEA-2\_Larvae-L3-stage | 32 | 3.87 | 3.5e-09 |
| R02D3.7 | R02D3.7\_Larvae-L3-stage | 70 | 2.09 | 3.7e-09 |
| ces-1 | CES-1\_Larvae-L4-stage | 40 | 3.14 | 4.4e-09 |
| W03F9.2 | W03F9.2\_L4-Young-Adult-stage-larvae | 92 | 1.73 | 7.1e-09 |
| lsy-2 | LSY-2\_Fed-L1-stage-larvae | 67 | 2.11 | 8.9e-09 |
| ceh-38 | CEH-38\_Larvae-L3-stage | 60 | 2.21 | 2.5e-08 |
| egl-5 | EGL-5\_Larvae-L3-stage | 50 | 2.50 | 2.5e-08 |
| pes-1 | PES-1\_Larvae-L4-stage | 57 | 2.27 | 3.1e-08 |
| ceh-26 | CEH-26\_Late-Embryonic-stage | 50 | 2.48 | 3.3e-08 |
| gei-11 | GEI-11\_Fed-L1-stage-larvae | 52 | 2.35 | 8.0e-08 |
| sax-3 | SAX-3\_Larvae-L4-stage | 60 | 2.09 | 2.0e-07 |
| nhr-6 | NHR-6\_Larvae-L2-stage | 53 | 2.26 | 2.1e-07 |
| ces-1 | CES-1\_Larvae-L3-stage | 37 | 2.90 | 2.1e-07 |
| dpl-1 | DPL-1\_Fed-L1-stage-larvae | 47 | 2.34 | 7.7e-07 |
| efl-1 | EFL-1\_Larvae-L1-stage | 53 | 2.16 | 8.9e-07 |
| sax-3 | SAX-3\_Larvae-L2-stage | 51 | 2.16 | 2.0e-06 |
| jun-1 | JUN-1\_Larvae-L1-stage | 41 | 2.32 | 1.1e-05 |
| mab-5 | MAB-5\_Larvae-L2-stage | 35 | 2.56 | 1.1e-05 |
| lin-13 | LIN-13\_Larvae-L2-stage | 39 | 2.39 | 1.1e-05 |
| zag-1 | ZAG-1\_Larvae-L4-stage | 32 | 2.65 | 1.9e-05 |
| efl-1 | EFL-1\_Fed-L1-stage-larvae | 49 | 1.98 | 4.6e-05 |
| aly-2 | ALY-2\_Fed-L1-stage-larvae | 39 | 2.20 | 7.5e-05 |
| ztf-7 | ZTF-7\_Larvae-L4-stage | 40 | 2.13 | 1.2e-04 |
| ceh-39 | CEH-39\_Embryos | 30 | 2.52 | 1.2e-04 |
| hpl-2 | HPL-2\_Fed-L1-stage-larvae | 50 | 1.87 | 1.7e-04 |
| lsy-2 | LSY-2\_Embryos | 44 | 1.95 | 3.0e-04 |
| zag-1 | ZAG-1\_Fed-L1-stage-larvae | 29 | 2.41 | 4.1e-04 |
| lsy-2 | LSY-2\_Larvae-L4-stage | 26 | 2.54 | 5.2e-04 |
| unc-62 | UNC-62\_Larvae-L3-stage | 35 | 2.12 | 5.9e-04 |
| nhr-77 | NHR-77\_Larvae-L4-stage | 58 | 1.68 | 6.0e-04 |
| lin-15 | LIN-15B\_Fed-L1-stage-larvae | 29 | 2.35 | 6.2e-04 |
| F45C12.2 | F45C12.2\_Larvae-L2-stage | 24 | 2.51 | 1.3e-03 |
| ama-1 | AMA-1\_Larvae-L3-stage | 25 | 2.37 | 2.2e-03 |
| zag-1 | ZAG-1\_Larvae-L3-stage | 23 | 2.47 | 2.3e-03 |
| unc-62 | UNC-62\_Day-Four-Young-Adult | 32 | 2.05 | 2.6e-03 |
| unc-62 | UNC-62\_Young-adult-Day-4 | 32 | 2.05 | 2.6e-03 |
| jun-1 | JUN-1\_Larvae-L3-stage | 31 | 2.08 | 2.8e-03 |
| ztf-4 | ZTF-4\_Larvae-L2-stage | 23 | 2.44 | 2.8e-03 |
| dpl-1 | DPL-1\_Young-adult | 48 | 1.70 | 3.1e-03 |
| nhr-25 | NHR-25\_Larvae-L2-stage | 47 | 1.71 | 3.3e-03 |
| nhr-23 | NHR-23\_Larvae-L3-stage | 50 | 1.66 | 3.6e-03 |
| nhr-77 | NHR-77\_Fed-L1-stage-larvae | 43 | 1.76 | 3.9e-03 |
| pha-4 | PHA-4\_Larvae-L2-stage | 44 | 1.74 | 4.0e-03 |
| unc-62 | UNC-62\_Larvae-L2-stage | 20 | 2.54 | 4.8e-03 |
| nhr-28 | NHR-28\_Larvae-L4-stage | 53 | 1.59 | 6.6e-03 |
| nhr-76 | NHR-76\_Larvae-L4-stage | 26 | 2.14 | 6.7e-03 |
| aly-2 | ALY-2\_Larvae-L3-stage | 30 | 1.99 | 7.5e-03 |
| C01B12.2 | C01B12.2\_Larvae-L2-stage | 64 | 1.44 | 1.6e-02 |
| R02D3.7 | R02D3.7\_Larvae-L2-stage | 22 | 2.16 | 1.9e-02 |
| F16B12.6 | F16B12.6\_Fed-L1-stage-larvae | 24 | 2.06 | 1.9e-02 |
| hlh-30 | HLH-30\_Larvae-L4-stage | 31 | 1.83 | 2.1e-02 |
| fkh-2 | FKH-2\_Larvae-L3-stage | 18 | 2.34 | 2.4e-02 |
| lin-13 | LIN-13\_Larvae-L1-stage | 15 | 2.60 | 2.4e-02 |
| C16A3.4 | C16A3.4\_Fed-L1-stage-larvae | 30 | 1.83 | 2.6e-02 |
| lsy-2 | LSY-2\_Larvae-L2-stage | 21 | 2.14 | 2.6e-02 |
| sax-3 | SAX-3\_Fed-L1-stage-larvae | 22 | 2.08 | 2.8e-02 |
| elt-1 | ELT-1\_Larvae-L3-stage | 20 | 2.17 | 3.0e-02 |
| mef-2 | MEF-2\_Fed-L1-stage-larvae | 15 | 2.51 | 3.2e-02 |
| hlh-30 | HLH-30\_Late-Embryos | 26 | 1.91 | 3.3e-02 |
| fos-1 | FOS-1\_Larvae-L4-stage | 23 | 1.96 | 4.5e-02 |
| lin-13 | LIN-13\_Larvae-L4-stage | 29 | 1.78 | 4.6e-02 |
| nhr-237 | NHR-237\_Larvae-L1-stage | 15 | 2.41 | 4.6e-02 |
