# Supplementary material for: Overlapping cell population expression profiling and regulatory inference in C. elegans
Source: BMC Genomics. 2016 Feb 29;17:159. doi: 10.1186/s12864-016-2482-z (PMC4772325; doi:10.1186/s12864-016-2482-z)
Supplement: Additional file 13: — Web supplement. (DOC 21 kb) [file 12864_2016_2482_MOESM13_ESM.zip › sortWeb/clusters/hier.300.clusters/158.html]

Cluster 158 

## Cluster 158

### Expression

| cnd-1 rep. 1 | cnd-1 rep. 2 | cnd-1 rep. 3 | pha-4 rep. 1 | pha-4 rep. 2 | pha-4 rep. 3 | ceh-27 | ceh-36 | ceh-6 | F21D5.9 | mir-57 | mls-2 | pal-1 | pros-1 | ttx-3 | unc-130 | hlh-16 | irx-1 | ceh-6 (+) hlh-16 (+) | ceh-6 (+) hlh-16 (-) | ceh-6 (-) hlh-16 (+) | cnd-1 singlets | pha-4 singlets | 0 | 60 | 120 | 150 | 180 | 240 | 330 | 390 | 420 | 480 | 540 | 570 | 600 | 630 | 660 | NAME | Functional description |
| --- | --- | --- | --- | --- | --- | --- | --- | --- | --- | --- | --- | --- | --- | --- | --- | --- | --- | --- | --- | --- | --- | --- | --- | --- | --- | --- | --- | --- | --- | --- | --- | --- | --- | --- | --- | --- | --- | --- | --- |
|  |  |  |  |  |  |  |  |  |  |  |  |  |  |  |  |  |  |  |  |  |  |  |  |  |  |  |  |  |  |  |  |  |  |  |  |  |  | *kel-10* | KELch-repeat containing protein |
|  |  |  |  |  |  |  |  |  |  |  |  |  |  |  |  |  |  |  |  |  |  |  |  |  |  |  |  |  |  |  |  |  |  |  |  |  |  | F58A3.3 |  |
|  |  |  |  |  |  |  |  |  |  |  |  |  |  |  |  |  |  |  |  |  |  |  |  |  |  |  |  |  |  |  |  |  |  |  |  |  |  | C41C4.12 |  |
|  |  |  |  |  |  |  |  |  |  |  |  |  |  |  |  |  |  |  |  |  |  |  |  |  |  |  |  |  |  |  |  |  |  |  |  |  |  | C34H3.23 |  |
|  |  |  |  |  |  |  |  |  |  |  |  |  |  |  |  |  |  |  |  |  |  |  |  |  |  |  |  |  |  |  |  |  |  |  |  |  |  | F58F12.4 |  |
|  |  |  |  |  |  |  |  |  |  |  |  |  |  |  |  |  |  |  |  |  |  |  |  |  |  |  |  |  |  |  |  |  |  |  |  |  |  | ZK669.3 |  |
|  |  |  |  |  |  |  |  |  |  |  |  |  |  |  |  |  |  |  |  |  |  |  |  |  |  |  |  |  |  |  |  |  |  |  |  |  |  | *fbxa-200* | F-box A protein |
|  |  |  |  |  |  |  |  |  |  |  |  |  |  |  |  |  |  |  |  |  |  |  |  |  |  |  |  |  |  |  |  |  |  |  |  |  |  | *ceh-75* | C. Elegans Homeobox |
|  |  |  |  |  |  |  |  |  |  |  |  |  |  |  |  |  |  |  |  |  |  |  |  |  |  |  |  |  |  |  |  |  |  |  |  |  |  | Y97E10AL.4 |  |
|  |  |  |  |  |  |  |  |  |  |  |  |  |  |  |  |  |  |  |  |  |  |  |  |  |  |  |  |  |  |  |  |  |  |  |  |  |  | *srx-134* | Serpentine Receptor, class X |
|  |  |  |  |  |  |  |  |  |  |  |  |  |  |  |  |  |  |  |  |  |  |  |  |  |  |  |  |  |  |  |  |  |  |  |  |  |  | F31F7.3 |  |
|  |  |  |  |  |  |  |  |  |  |  |  |  |  |  |  |  |  |  |  |  |  |  |  |  |  |  |  |  |  |  |  |  |  |  |  |  |  | C08F8.14 |  |
|  |  |  |  |  |  |  |  |  |  |  |  |  |  |  |  |  |  |  |  |  |  |  |  |  |  |  |  |  |  |  |  |  |  |  |  |  |  | *str-35* | Seven TM Receptor |
|  |  |  |  |  |  |  |  |  |  |  |  |  |  |  |  |  |  |  |  |  |  |  |  |  |  |  |  |  |  |  |  |  |  |  |  |  |  | Y39B6A.31 |  |
|  |  |  |  |  |  |  |  |  |  |  |  |  |  |  |  |  |  |  |  |  |  |  |  |  |  |  |  |  |  |  |  |  |  |  |  |  |  | ZC123.5 |  |
|  |  |  |  |  |  |  |  |  |  |  |  |  |  |  |  |  |  |  |  |  |  |  |  |  |  |  |  |  |  |  |  |  |  |  |  |  |  | *srbc-35* | Serpentine Receptor, class BC (class B-like) |
|  |  |  |  |  |  |  |  |  |  |  |  |  |  |  |  |  |  |  |  |  |  |  |  |  |  |  |  |  |  |  |  |  |  |  |  |  |  | *twk-46* | TWiK family of potassium channels |
|  |  |  |  |  |  |  |  |  |  |  |  |  |  |  |  |  |  |  |  |  |  |  |  |  |  |  |  |  |  |  |  |  |  |  |  |  |  | *pes-7* | Patterned Expression Site |
|  |  |  |  |  |  |  |  |  |  |  |  |  |  |  |  |  |  |  |  |  |  |  |  |  |  |  |  |  |  |  |  |  |  |  |  |  |  | *trp-1* | TRP (transient receptor potential) channel family |
|  |  |  |  |  |  |  |  |  |  |  |  |  |  |  |  |  |  |  |  |  |  |  |  |  |  |  |  |  |  |  |  |  |  |  |  |  |  | *flp-22* | FMRF-Like Peptide |
|  |  |  |  |  |  |  |  |  |  |  |  |  |  |  |  |  |  |  |  |  |  |  |  |  |  |  |  |  |  |  |  |  |  |  |  |  |  | *ccf-1* | yeast CCR4 associated Factor family |
|  |  |  |  |  |  |  |  |  |  |  |  |  |  |  |  |  |  |  |  |  |  |  |  |  |  |  |  |  |  |  |  |  |  |  |  |  |  | C29A12.21 |  |
|  |  |  |  |  |  |  |  |  |  |  |  |  |  |  |  |  |  |  |  |  |  |  |  |  |  |  |  |  |  |  |  |  |  |  |  |  |  | *irld-52* | Insulin/EGF-Receptor L Domain protein |
|  |  |  |  |  |  |  |  |  |  |  |  |  |  |  |  |  |  |  |  |  |  |  |  |  |  |  |  |  |  |  |  |  |  |  |  |  |  | B0399.3 |  |
|  |  |  |  |  |  |  |  |  |  |  |  |  |  |  |  |  |  |  |  |  |  |  |  |  |  |  |  |  |  |  |  |  |  |  |  |  |  | R02D5.16 |  |
|  |  |  |  |  |  |  |  |  |  |  |  |  |  |  |  |  |  |  |  |  |  |  |  |  |  |  |  |  |  |  |  |  |  |  |  |  |  | Y38H6A.7 |  |
|  |  |  |  |  |  |  |  |  |  |  |  |  |  |  |  |  |  |  |  |  |  |  |  |  |  |  |  |  |  |  |  |  |  |  |  |  |  | C56G2.4 |  |
|  |  |  |  |  |  |  |  |  |  |  |  |  |  |  |  |  |  |  |  |  |  |  |  |  |  |  |  |  |  |  |  |  |  |  |  |  |  | *aat-2* | Amino Acid Transporter |
|  |  |  |  |  |  |  |  |  |  |  |  |  |  |  |  |  |  |  |  |  |  |  |  |  |  |  |  |  |  |  |  |  |  |  |  |  |  | *raga-1* | RAs-related GTP-binding protein A |
|  |  |  |  |  |  |  |  |  |  |  |  |  |  |  |  |  |  |  |  |  |  |  |  |  |  |  |  |  |  |  |  |  |  |  |  |  |  | Y55F3AM.9 |  |
|  |  |  |  |  |  |  |  |  |  |  |  |  |  |  |  |  |  |  |  |  |  |  |  |  |  |  |  |  |  |  |  |  |  |  |  |  |  | *bre-4* | BT (Bacillus thuringiensis) toxin REsistant |
|  |  |  |  |  |  |  |  |  |  |  |  |  |  |  |  |  |  |  |  |  |  |  |  |  |  |  |  |  |  |  |  |  |  |  |  |  |  | C05D2.11 |  |
|  |  |  |  |  |  |  |  |  |  |  |  |  |  |  |  |  |  |  |  |  |  |  |  |  |  |  |  |  |  |  |  |  |  |  |  |  |  | *zig-1* | 2 (Zwei) IG domain protein |
|  |  |  |  |  |  |  |  |  |  |  |  |  |  |  |  |  |  |  |  |  |  |  |  |  |  |  |  |  |  |  |  |  |  |  |  |  |  | *somi-1* | Suppressor of Overexpressed MIcro-RNA |
|  |  |  |  |  |  |  |  |  |  |  |  |  |  |  |  |  |  |  |  |  |  |  |  |  |  |  |  |  |  |  |  |  |  |  |  |  |  | *ztf-9* | Zinc finger putative Transcription Factor family |
|  |  |  |  |  |  |  |  |  |  |  |  |  |  |  |  |  |  |  |  |  |  |  |  |  |  |  |  |  |  |  |  |  |  |  |  |  |  | *lev-1* | LEVamisole resistant |
|  |  |  |  |  |  |  |  |  |  |  |  |  |  |  |  |  |  |  |  |  |  |  |  |  |  |  |  |  |  |  |  |  |  |  |  |  |  | *sto-5* | STOmatin |
|  |  |  |  |  |  |  |  |  |  |  |  |  |  |  |  |  |  |  |  |  |  |  |  |  |  |  |  |  |  |  |  |  |  |  |  |  |  | *calm-1* | CALMyrin (Calcium and Integrin Binding protein) homolog |
|  |  |  |  |  |  |  |  |  |  |  |  |  |  |  |  |  |  |  |  |  |  |  |  |  |  |  |  |  |  |  |  |  |  |  |  |  |  | Y39A1C.1 |  |
|  |  |  |  |  |  |  |  |  |  |  |  |  |  |  |  |  |  |  |  |  |  |  |  |  |  |  |  |  |  |  |  |  |  |  |  |  |  | *ckk-1* | CaM Kinase Kinase |
|  |  |  |  |  |  |  |  |  |  |  |  |  |  |  |  |  |  |  |  |  |  |  |  |  |  |  |  |  |  |  |  |  |  |  |  |  |  | F42C5.9 |  |
|  |  |  |  |  |  |  |  |  |  |  |  |  |  |  |  |  |  |  |  |  |  |  |  |  |  |  |  |  |  |  |  |  |  |  |  |  |  | C07A9.9 |  |
|  |  |  |  |  |  |  |  |  |  |  |  |  |  |  |  |  |  |  |  |  |  |  |  |  |  |  |  |  |  |  |  |  |  |  |  |  |  | *unc-64* | UNCoordinated |
|  |  |  |  |  |  |  |  |  |  |  |  |  |  |  |  |  |  |  |  |  |  |  |  |  |  |  |  |  |  |  |  |  |  |  |  |  |  | *pat-2* | Paralysed Arrest at Two-fold |
|  |  |  |  |  |  |  |  |  |  |  |  |  |  |  |  |  |  |  |  |  |  |  |  |  |  |  |  |  |  |  |  |  |  |  |  |  |  | W07E11.1 |  |
|  |  |  |  |  |  |  |  |  |  |  |  |  |  |  |  |  |  |  |  |  |  |  |  |  |  |  |  |  |  |  |  |  |  |  |  |  |  | C50F7.6 |  |
|  |  |  |  |  |  |  |  |  |  |  |  |  |  |  |  |  |  |  |  |  |  |  |  |  |  |  |  |  |  |  |  |  |  |  |  |  |  | Y45G5AM.6 |  |
|  |  |  |  |  |  |  |  |  |  |  |  |  |  |  |  |  |  |  |  |  |  |  |  |  |  |  |  |  |  |  |  |  |  |  |  |  |  | *tsp-11* | TetraSPanin family |
|  |  |  |  |  |  |  |  |  |  |  |  |  |  |  |  |  |  |  |  |  |  |  |  |  |  |  |  |  |  |  |  |  |  |  |  |  |  | H06I04.5 |  |
|  |  |  |  |  |  |  |  |  |  |  |  |  |  |  |  |  |  |  |  |  |  |  |  |  |  |  |  |  |  |  |  |  |  |  |  |  |  | *cwp-3* | Coexpressed With Polycystins |
|  |  |  |  |  |  |  |  |  |  |  |  |  |  |  |  |  |  |  |  |  |  |  |  |  |  |  |  |  |  |  |  |  |  |  |  |  |  | C37H5.3 |  |
|  |  |  |  |  |  |  |  |  |  |  |  |  |  |  |  |  |  |  |  |  |  |  |  |  |  |  |  |  |  |  |  |  |  |  |  |  |  | *ndx-1* | NuDiX family |
|  |  |  |  |  |  |  |  |  |  |  |  |  |  |  |  |  |  |  |  |  |  |  |  |  |  |  |  |  |  |  |  |  |  |  |  |  |  | F48F7.5 |  |
|  |  |  |  |  |  |  |  |  |  |  |  |  |  |  |  |  |  |  |  |  |  |  |  |  |  |  |  |  |  |  |  |  |  |  |  |  |  | *vdac-1* | VDAC (Voltage Dependent Anion Channel) homolog |
|  |  |  |  |  |  |  |  |  |  |  |  |  |  |  |  |  |  |  |  |  |  |  |  |  |  |  |  |  |  |  |  |  |  |  |  |  |  | *ftt-2* | Fourteen-Three-Three family |
|  |  |  |  |  |  |  |  |  |  |  |  |  |  |  |  |  |  |  |  |  |  |  |  |  |  |  |  |  |  |  |  |  |  |  |  |  |  | R12C12.10 |  |
|  |  |  |  |  |  |  |  |  |  |  |  |  |  |  |  |  |  |  |  |  |  |  |  |  |  |  |  |  |  |  |  |  |  |  |  |  |  | *oig-4* | One IG domain |
|  |  |  |  |  |  |  |  |  |  |  |  |  |  |  |  |  |  |  |  |  |  |  |  |  |  |  |  |  |  |  |  |  |  |  |  |  |  | *dmsr-7* | DroMyoSuppressin Receptor related |
|  |  |  |  |  |  |  |  |  |  |  |  |  |  |  |  |  |  |  |  |  |  |  |  |  |  |  |  |  |  |  |  |  |  |  |  |  |  | *cal-2* | CALmodulin related genes |
|  |  |  |  |  |  |  |  |  |  |  |  |  |  |  |  |  |  |  |  |  |  |  |  |  |  |  |  |  |  |  |  |  |  |  |  |  |  | K07C11.8 |  |
|  |  |  |  |  |  |  |  |  |  |  |  |  |  |  |  |  |  |  |  |  |  |  |  |  |  |  |  |  |  |  |  |  |  |  |  |  |  | *hot-8* | Homolog of Odr-2 (Two) |
|  |  |  |  |  |  |  |  |  |  |  |  |  |  |  |  |  |  |  |  |  |  |  |  |  |  |  |  |  |  |  |  |  |  |  |  |  |  | *let-70* | LEThal |
|  |  |  |  |  |  |  |  |  |  |  |  |  |  |  |  |  |  |  |  |  |  |  |  |  |  |  |  |  |  |  |  |  |  |  |  |  |  | C24A3.2 |  |
|  |  |  |  |  |  |  |  |  |  |  |  |  |  |  |  |  |  |  |  |  |  |  |  |  |  |  |  |  |  |  |  |  |  |  |  |  |  | D2007.1 |  |
|  |  |  |  |  |  |  |  |  |  |  |  |  |  |  |  |  |  |  |  |  |  |  |  |  |  |  |  |  |  |  |  |  |  |  |  |  |  | M03B6.5 |  |
|  |  |  |  |  |  |  |  |  |  |  |  |  |  |  |  |  |  |  |  |  |  |  |  |  |  |  |  |  |  |  |  |  |  |  |  |  |  | R102.1 |  |
|  |  |  |  |  |  |  |  |  |  |  |  |  |  |  |  |  |  |  |  |  |  |  |  |  |  |  |  |  |  |  |  |  |  |  |  |  |  | Y41E3.6 |  |
|  |  |  |  |  |  |  |  |  |  |  |  |  |  |  |  |  |  |  |  |  |  |  |  |  |  |  |  |  |  |  |  |  |  |  |  |  |  | Y76A2B.5 |  |
|  |  |  |  |  |  |  |  |  |  |  |  |  |  |  |  |  |  |  |  |  |  |  |  |  |  |  |  |  |  |  |  |  |  |  |  |  |  | *mce-1* | Methylmalonyl-CoA Epimerase |
|  |  |  |  |  |  |  |  |  |  |  |  |  |  |  |  |  |  |  |  |  |  |  |  |  |  |  |  |  |  |  |  |  |  |  |  |  |  | *sir-2.2* | yeast SIR related |
|  |  |  |  |  |  |  |  |  |  |  |  |  |  |  |  |  |  |  |  |  |  |  |  |  |  |  |  |  |  |  |  |  |  |  |  |  |  | ZK1290.5 |  |
|  |  |  |  |  |  |  |  |  |  |  |  |  |  |  |  |  |  |  |  |  |  |  |  |  |  |  |  |  |  |  |  |  |  |  |  |  |  | F42H10.3 |  |
|  |  |  |  |  |  |  |  |  |  |  |  |  |  |  |  |  |  |  |  |  |  |  |  |  |  |  |  |  |  |  |  |  |  |  |  |  |  | Y102E9.5 |  |
|  |  |  |  |  |  |  |  |  |  |  |  |  |  |  |  |  |  |  |  |  |  |  |  |  |  |  |  |  |  |  |  |  |  |  |  |  |  | K08E7.8 |  |
|  |  |  |  |  |  |  |  |  |  |  |  |  |  |  |  |  |  |  |  |  |  |  |  |  |  |  |  |  |  |  |  |  |  |  |  |  |  | *lron-9* | eLRR (extracellular Leucine-Rich Repeat) ONly |
|  |  |  |  |  |  |  |  |  |  |  |  |  |  |  |  |  |  |  |  |  |  |  |  |  |  |  |  |  |  |  |  |  |  |  |  |  |  | *acs-20* | fatty Acid CoA Synthetase family |
|  |  |  |  |  |  |  |  |  |  |  |  |  |  |  |  |  |  |  |  |  |  |  |  |  |  |  |  |  |  |  |  |  |  |  |  |  |  | *agmo-1* | AlkylGlycerol MonoOxygenase |
|  |  |  |  |  |  |  |  |  |  |  |  |  |  |  |  |  |  |  |  |  |  |  |  |  |  |  |  |  |  |  |  |  |  |  |  |  |  | *tag-89* | Temporarily Assigned Gene name |
|  |  |  |  |  |  |  |  |  |  |  |  |  |  |  |  |  |  |  |  |  |  |  |  |  |  |  |  |  |  |  |  |  |  |  |  |  |  | *cpn-2* | CalPoNin |
|  |  |  |  |  |  |  |  |  |  |  |  |  |  |  |  |  |  |  |  |  |  |  |  |  |  |  |  |  |  |  |  |  |  |  |  |  |  | *spp-13* | SaPosin-like Protein family |
|  |  |  |  |  |  |  |  |  |  |  |  |  |  |  |  |  |  |  |  |  |  |  |  |  |  |  |  |  |  |  |  |  |  |  |  |  |  | *aldo-1* | ALDOlase (fructose bisphosphate aldolase) |
|  |  |  |  |  |  |  |  |  |  |  |  |  |  |  |  |  |  |  |  |  |  |  |  |  |  |  |  |  |  |  |  |  |  |  |  |  |  | F19C7.8 |  |
|  |  |  |  |  |  |  |  |  |  |  |  |  |  |  |  |  |  |  |  |  |  |  |  |  |  |  |  |  |  |  |  |  |  |  |  |  |  | *got-2.2* | Glutamate Oxaloacetate Transaminase |
|  |  |  |  |  |  |  |  |  |  |  |  |  |  |  |  |  |  |  |  |  |  |  |  |  |  |  |  |  |  |  |  |  |  |  |  |  |  | *gpd-3* | GPD (Glyceraldehyde 3-Phosphate Dehydrogenase) |
|  |  |  |  |  |  |  |  |  |  |  |  |  |  |  |  |  |  |  |  |  |  |  |  |  |  |  |  |  |  |  |  |  |  |  |  |  |  | *mai-1* | Mitochondrial ATPase Inhibitor family |
|  |  |  |  |  |  |  |  |  |  |  |  |  |  |  |  |  |  |  |  |  |  |  |  |  |  |  |  |  |  |  |  |  |  |  |  |  |  | *gpd-2* | GPD (Glyceraldehyde 3-Phosphate Dehydrogenase) |
|  |  |  |  |  |  |  |  |  |  |  |  |  |  |  |  |  |  |  |  |  |  |  |  |  |  |  |  |  |  |  |  |  |  |  |  |  |  | F49D11.3 |  |
|  |  |  |  |  |  |  |  |  |  |  |  |  |  |  |  |  |  |  |  |  |  |  |  |  |  |  |  |  |  |  |  |  |  |  |  |  |  | *lin-18* | abnormal cell LINeage |
|  |  |  |  |  |  |  |  |  |  |  |  |  |  |  |  |  |  |  |  |  |  |  |  |  |  |  |  |  |  |  |  |  |  |  |  |  |  | *srz-59* | Serpentine Receptor, class Z |
|  |  |  |  |  |  |  |  |  |  |  |  |  |  |  |  |  |  |  |  |  |  |  |  |  |  |  |  |  |  |  |  |  |  |  |  |  |  | *kvs-1* | K (potassium) Voltage-Sensitive channel subunit |
|  |  |  |  |  |  |  |  |  |  |  |  |  |  |  |  |  |  |  |  |  |  |  |  |  |  |  |  |  |  |  |  |  |  |  |  |  |  | *dhp-1* | DiHydroPyrimidinase |
|  |  |  |  |  |  |  |  |  |  |  |  |  |  |  |  |  |  |  |  |  |  |  |  |  |  |  |  |  |  |  |  |  |  |  |  |  |  | C01H6.2 |  |
|  |  |  |  |  |  |  |  |  |  |  |  |  |  |  |  |  |  |  |  |  |  |  |  |  |  |  |  |  |  |  |  |  |  |  |  |  |  | F13B12.4 |  |
|  |  |  |  |  |  |  |  |  |  |  |  |  |  |  |  |  |  |  |  |  |  |  |  |  |  |  |  |  |  |  |  |  |  |  |  |  |  | *hot-5* | Homolog of Odr-2 (Two) |
|  |  |  |  |  |  |  |  |  |  |  |  |  |  |  |  |  |  |  |  |  |  |  |  |  |  |  |  |  |  |  |  |  |  |  |  |  |  | W07A12.4 |  |
|  |  |  |  |  |  |  |  |  |  |  |  |  |  |  |  |  |  |  |  |  |  |  |  |  |  |  |  |  |  |  |  |  |  |  |  |  |  | *hlh-10* | Helix Loop Helix |

### Phenotypes enriched

none found

### Anatomy terms enriched

none found

### GO terms enriched

|  |  |  |
| --- | --- | --- |
| **GO term** | **Number of genes** | **FDR-corrected p-value** |
| glycolysis | 3 | 0.0055 |
| hexose catabolic process | 3 | 0.0096 |
| glucose metabolic process | 3 | 0.0220 |
| single-organism carbohydrate catabolic process | 3 | 0.0270 |

### Expression clusters enriched

|  |  |  |  |
| --- | --- | --- | --- |
| **Group name** | **Number in cluster** | **Enrichment** | **FDR corrected p** |
| Genes enriched in muscle cells (0hr muscle dataset). Dissociated myo-3::GFP embryos were cultured for 0 hours before FACS sorting. | 28 | 6.00 | 6.09e-12 |
| Total muscle enriched genes (complete list of non-overlapping genes from the 0hr and 24hr muscle enriched datasets). | 33 | 4.19 | 3.04e-10 |
| Genes enriched in HLH-1 heat shock dataset. | 39 | 2.75 | 4.00e-07 |
| Genes that show selective expression in a subset of cell types vs broadly expressed in many cell types. Correspond to 20% - 57% of enriched\_genes for a given cell type. WBPaper00037950:bodywall-muscle\_larva\_SelectivelyEnriched | 14 | 5.11 | 2.23e-04 |
| Genes enriched in muscle cells (24hr muscle dataset). Dissociated myo-3::GFP embryos were cultured for 24 hours before FACS sorting. | 17 | 3.03 | 1.01e-02 |
| Genes enriched in body wall muscle AIN-2 miRISCs. Pmyo-3-ain-2-gfp IP was performed in mixed stage worms. | 12 | 3.75 | 2.00e-02 |
| Genes specifically affected by organophosphorus pesticide exposure. Dic | 5 | 9.50 | 4.05e-02 |

### Motifs enriched

|  |  |  |  |  |  |
| --- | --- | --- | --- | --- | --- |
| **Motif** | **Logo** | **Possible orthologs** | **Number of motifs in cluster** | **Enrichment** | **FDR corrected p** |
| V$SP1\_Q6 |  | ZC328.2 klf-1 klf-2 | 40 | 2.33 | 1.9e-05 |
| FOXO6\_3 |  | ZC328.2 daf-16 | 58 | 1.77 | 4.3e-05 |
| MA0543.1 |  | daf-8 eor-1 | 55 | 1.81 | 5.8e-05 |
| Six6\_2267 |  | ceh-34 ceh-32 | 43 | 2.04 | 1.6e-04 |
| MA0246.1 |  | dmd-4 dmd-5 ceh-32 | 29 | 2.58 | 2.4e-04 |
| ELF3\_2 |  | nhr-19 (0.59) lin-1 C24A1.2 | 43 | 1.97 | 3.4e-04 |
| pTH10623 |  | scrt-1 | 50 | 1.80 | 3.8e-04 |
| Blimp-1\_NAR\_FBgn0035625 |  | blmp-1 | 36 | 2.18 | 3.9e-04 |
| NR2F6\_f1 |  | nhr-2 nhr-239 | 73 | 1.45 | 4.2e-04 |
| MA0482.1 |  | elt-1 | 69 | 1.49 | 5.0e-04 |
| SPDEF\_2 |  | ztf-14 lin-1 ZC328.2 | 52 | 1.74 | 5.4e-04 |
| Elf2 |  | lin-1 F19F10.1 C24A1.2 | 61 | 1.57 | 7.7e-04 |
| Six1\_0935 |  | ceh-32 | 45 | 1.85 | 7.8e-04 |
| SP3\_1 |  | klf-1 klf-2 | 37 | 2.07 | 7.9e-04 |
| VDR\_1 |  | gei-3 (0.63) cfi-1 nhr-208 | 32 | 2.26 | 7.9e-04 |
| V$FREAC2\_01 |  | fkh-8 (0.54) fkh-7 lin-31 fkh-10 let-381 daf-16 | 68 | 1.48 | 8.8e-04 |
| V$AP2REP\_01 |  | klf-1 | 50 | 1.74 | 8.9e-04 |
| bin\_SANGER\_5\_FBgn0045759 |  | fkh-8 (0.54) fkh-7 pha-4 lin-31 let-381 daf-16 | 55 | 1.65 | 9.4e-04 |
| MA0260.1 |  | che-1 | 58 | 1.61 | 9.6e-04 |
| pTH9924 |  | nhr-46 | 68 | 1.47 | 1.0e-03 |
| FOXC1\_f1 |  | lin-31 let-381 | 64 | 1.49 | 1.8e-03 |
| SH-SY5Y\_GATA2\_UCD |  | elt-1 | 53 | 1.64 | 1.8e-03 |
| V$GATA3\_01 |  | elt-1 | 32 | 2.13 | 2.1e-03 |
| pTH9711 |  | ces-2 atf-2 | 84 | 1.28 | 2.2e-03 |
| Six3\_1732 |  | ceh-34 | 44 | 1.79 | 2.2e-03 |
| MA0085.1 |  | ztf-3 lag-1 | 42 | 1.83 | 2.3e-03 |
| pTH8216 |  | Y116A8C.22 | 50 | 1.68 | 2.3e-03 |
| Mf28 |  | elt-1 | 51 | 1.66 | 2.4e-03 |
| Pax7\_3783 |  | alr-1 | 52 | 1.64 | 2.5e-03 |
| MAFA\_f1 |  | daf-8 F45H11.6 | 50 | 1.67 | 2.6e-03 |
| pTH5057 |  | mdl-1 (0.53) aha-1 hlh-30 | 27 | 2.32 | 2.7e-03 |
| V$FAC1\_01 |  | gei-8 | 67 | 1.44 | 2.8e-03 |
| HeLa-S3\_ZNF274\_UCD |  | C28G1.4 | 38 | 1.91 | 2.8e-03 |
| V$AREB6\_04 |  | ztf-6 C34D1.1 gei-11 | 64 | 1.47 | 2.9e-03 |
| pTH10808 |  | ztf-19 | 62 | 1.49 | 2.9e-03 |
| pTH9072 |  | sptf-3 klf-1 klf-2 | 39 | 1.88 | 2.9e-03 |
| pTH8411 |  | tbx-39 | 58 | 1.54 | 3.2e-03 |
| HEN1\_si |  | hlh-15 | 31 | 2.12 | 3.2e-03 |
| MA0598.1 |  | lin-1 C24A1.2 | 20 | 2.77 | 3.6e-03 |
| Elf5 |  | C24A1.2 | 40 | 1.83 | 3.7e-03 |
| Spdef |  | lin-1 | 50 | 1.64 | 3.9e-03 |
| EGR3\_f1 |  | ZC328.2 klf-2 | 48 | 1.67 | 4.0e-03 |
| MA0142.1 |  | ceh-18 (0.57) ceh-6 tbp-1 | 72 | 1.37 | 4.0e-03 |
| MA0468.1 |  | ceh-14 ZC204.2 | 67 | 1.42 | 4.1e-03 |
| Eip74EF\_FlyReg\_FBgn0000567 |  | unc-120 C24A1.2 | 79 | 1.31 | 4.2e-03 |
| V$PAX2\_02 |  | pax-1 | 19 | 2.82 | 4.5e-03 |
| Ets97D\_SANGER\_10\_FBgn0004510 |  | lin-1 C24A1.2 | 43 | 1.75 | 4.5e-03 |
| ETS2\_f1 |  | lin-1 C24A1.2 | 41 | 1.79 | 4.5e-03 |
| pTH10013 |  | nhr-168 | 59 | 1.50 | 4.9e-03 |
| pTH9247 |  | C34D1.1 | 42 | 1.76 | 5.1e-03 |
| pTH3467 |  | nhr-6 | 50 | 1.62 | 5.2e-03 |
| PTF1A\_f1 |  | lin-32 | 60 | 1.49 | 5.2e-03 |
| V$MYB\_Q6 |  | D1081.8 | 43 | 1.74 | 5.2e-03 |
| ONECUT1\_1 |  | dsc-1 (0.52) ceh-48 | 71 | 1.37 | 5.2e-03 |
| V$PAX5\_02 |  | pax-3 pax-2 | 32 | 2.01 | 5.5e-03 |
| pTH9322 |  | nhr-7 nhr-10 elt-1 | 54 | 1.56 | 5.6e-03 |
| MA0146.2 |  | F58G1.2 | 20 | 2.66 | 5.7e-03 |
| SHOX2\_1 |  | pha-2 ceh-45 ceh-16 ceh-14 lin-39 alr-1 ceh-43 | 52 | 1.58 | 6.0e-03 |
| TBX20\_5 |  | mab-9 | 57 | 1.51 | 6.1e-03 |
| HNF4A\_6 |  | nhr-19 (0.59) nhr-2 nhr-62 | 50 | 1.61 | 6.2e-03 |
| MEF2B\_1 |  | mef-2 | 22 | 2.47 | 6.4e-03 |
| MA0476.1 |  | fos-1 | 35 | 1.90 | 6.8e-03 |
| pTH10779 |  | nhr-182 nhr-134 | 58 | 1.50 | 6.9e-03 |
| pTH9708 |  | ceh-34 | 45 | 1.68 | 6.9e-03 |
| nau\_SANGER\_5\_FBgn0002922 |  | hlh-1 hlh-14 hlh-11 lin-32 hlh-15 | 32 | 1.98 | 7.1e-03 |
| Gata3\_1024 |  | elt-1 | 46 | 1.66 | 7.2e-03 |
| V$XFD3\_01 |  | cfi-1 ceh-20 let-381 | 70 | 1.37 | 7.2e-03 |
| MA0444.1 |  | ceh-31 (0.61) ceh-8 cog-1 ceh-19 | 49 | 1.61 | 7.5e-03 |
| CG14962\_SANGER\_5\_FBgn0035407 |  | C34H4.5 | 68 | 1.39 | 7.7e-03 |
| MSX2\_1 |  | ceh-1 | 86 | 1.22 | 7.8e-03 |
| SPDEF\_3 |  | lin-1 | 55 | 1.52 | 8.5e-03 |
| Elf3\_3876 |  | C24A1.2 | 40 | 1.75 | 8.5e-03 |
| pTH5690 |  | ceh-32 | 45 | 1.66 | 8.5e-03 |
| Elf3 |  | C24A1.2 | 64 | 1.42 | 8.6e-03 |
| pTH9880 |  | end-1 | 35 | 1.87 | 8.8e-03 |
| HOXC10\_1 |  | hbl-1 lin-39 php-3 | 62 | 1.44 | 9.1e-03 |
| pTH10656 |  | hlh-12 hlh-32 | 49 | 1.59 | 9.1e-03 |
| EN1\_1 |  | ceh-31 (0.61) ceh-16 ceh-1 | 49 | 1.59 | 9.3e-03 |
| Elf4 |  | C24A1.2 | 58 | 1.48 | 9.5e-03 |
| pTH10630 |  | lsy-27 (0.52) | 44 | 1.67 | 9.6e-03 |
| pTH10811 |  | nhr-216 nhr-142 | 61 | 1.44 | 9.8e-03 |
| MA0262.1 |  | mab-3 | 64 | 1.41 | 9.9e-03 |
| Gbx2\_3110 |  | ceh-45 ceh-1 ceh-12 alr-1 | 49 | 1.59 | 1.0e-02 |
| RORA\_2 |  | nhr-213 nhr-118 | 47 | 1.61 | 1.0e-02 |
| pTH10768 |  | med-2 | 33 | 1.90 | 1.1e-02 |
| pTH2283 |  | odd-2 | 45 | 1.64 | 1.1e-02 |
| Atf6\_SANGER\_5\_FBgn0033010 |  | atf-6 | 39 | 1.75 | 1.1e-02 |
| pTH10629 |  | ztf-3 daf-12 | 57 | 1.47 | 1.2e-02 |
| pTH5928 |  | ceh-34 | 42 | 1.68 | 1.2e-02 |
| HXD10\_f1 |  | php-3 | 71 | 1.34 | 1.2e-02 |
| V$YY1\_01 |  | lsy-2 | 39 | 1.74 | 1.2e-02 |
| MA0174.1 |  | ceh-24 | 63 | 1.41 | 1.2e-02 |
| PO3F2\_si |  | ceh-18 (0.57) dmd-3 | 70 | 1.35 | 1.2e-02 |
| PBDE\_GATA1\_UCD |  | elt-1 | 55 | 1.49 | 1.3e-02 |
| CG31670\_SOLEXA\_5\_FBgn0031375 |  | CELE\_Y38H8A.5 | 46 | 1.61 | 1.4e-02 |
| pTH9242 |  | mel-28 (-0.53) | 70 | 1.34 | 1.4e-02 |
| pTH5065 |  | hlh-26 aha-1 mxl-1 hlh-30 | 22 | 2.31 | 1.4e-02 |
| pTH10718 |  | egl-43 | 54 | 1.49 | 1.5e-02 |
| srp\_SANGER\_5\_FBgn0003507 |  | elt-3 ceh-34 ceh-32 elt-1 elt-6 | 52 | 1.52 | 1.5e-02 |
| MA0600.1 |  | daf-19 (0.54) F52B5.7 | 51 | 1.52 | 1.7e-02 |
| pTH10041 |  | ztf-29 | 59 | 1.43 | 1.7e-02 |
| HXB1\_f1 |  | ceh-12 | 34 | 1.82 | 1.7e-02 |
| pTH9353 |  | ceh-51 | 66 | 1.37 | 1.7e-02 |
| pTH9261 |  | dmd-3 | 51 | 1.52 | 1.7e-02 |
| MA0540.1 |  | dpy-27 | 35 | 1.79 | 1.8e-02 |
| pTH3477 |  | daf-16 | 60 | 1.42 | 1.8e-02 |
| N$SKN1\_02 |  | skn-1 ztf-28 | 63 | 1.39 | 1.8e-02 |
| pTH9913 |  | skn-1 | 57 | 1.45 | 1.8e-02 |
| Eip78C\_SANGER\_5\_FBgn0004865 |  | nhr-10 | 36 | 1.76 | 1.9e-02 |
| EMX2\_2 |  | ceh-2 | 60 | 1.42 | 1.9e-02 |
| pTH1001 |  | dnj-17 | 49 | 1.54 | 2.0e-02 |
| pTH10650 |  | nhr-153 | 52 | 1.50 | 2.0e-02 |
| TBX2\_f1 |  | tbx-39 | 40 | 1.67 | 2.0e-02 |
| V$TST1\_01 |  | ceh-18 (0.57) pal-1 | 59 | 1.42 | 2.1e-02 |
| MA0488.1 |  | crh-1 | 38 | 1.70 | 2.1e-02 |
| MA0547.1 |  | skn-1 | 71 | 1.31 | 2.1e-02 |
| pTH10823 |  | B0310.2 | 44 | 1.60 | 2.1e-02 |
| Barhl1\_1 |  | ceh-31 (0.61) | 40 | 1.67 | 2.1e-02 |
| FOXG1\_2 |  | lin-31 | 45 | 1.58 | 2.2e-02 |
| V$HOX13\_01 |  | lin-39 | 61 | 1.40 | 2.3e-02 |
| pTH9073 |  | end-3 elt-1 | 38 | 1.69 | 2.3e-02 |
| TCF7L1\_1 |  | sox-4 (0.6) pop-1 (-0.56) | 65 | 1.36 | 2.4e-02 |
| Etv3 |  | lin-1 | 56 | 1.44 | 2.4e-02 |
| CG8765\_SANGER\_5\_FBgn0036900 |  | H20J04.3 | 63 | 1.38 | 2.4e-02 |
| Rax\_3443 |  | ceh-45 alr-1 | 48 | 1.53 | 2.4e-02 |
| pTH9180 |  | mel-28 (-0.53) | 77 | 1.26 | 2.5e-02 |
| sqz\_SOLEXA\_5\_FBgn0010768 |  | lin-29 | 70 | 1.31 | 2.5e-02 |
| En2\_0952 |  | ceh-16 | 60 | 1.40 | 2.5e-02 |
| pTH5924 |  | nhr-255 | 52 | 1.48 | 2.5e-02 |
| FLI1\_f1 |  | lin-1 | 44 | 1.58 | 2.6e-02 |
| Eip93F\_SANGER\_10\_FBgn0013948 |  | mbr-1 (0.62) | 65 | 1.35 | 2.6e-02 |
| pTH9108 |  | daf-12 | 45 | 1.57 | 2.6e-02 |
| SP4\_f1 |  | klf-2 plp-2 | 29 | 1.89 | 2.7e-02 |
| pTH9150 |  | odd-1 | 48 | 1.52 | 2.7e-02 |
| IRX5\_1 |  | irx-1 (-0.55) | 58 | 1.41 | 2.8e-02 |
| Nkx3-1\_2923 |  | ceh-24 | 22 | 2.16 | 2.8e-02 |
| HLH25 |  | hlh-27 | 32 | 1.80 | 2.8e-02 |
| V$OCT1\_06 |  | ceh-18 (0.57) | 69 | 1.32 | 2.9e-02 |
| FOXO1\_si |  | daf-16 | 47 | 1.53 | 2.9e-02 |
| pTH1049 |  | elt-1 | 46 | 1.54 | 2.9e-02 |
| pTH8649 |  | mbr-1 (0.62) | 61 | 1.38 | 2.9e-02 |
| pTH10816 |  | dmd-6 | 66 | 1.34 | 2.9e-02 |
| pTH10654 |  | ceh-90 | 44 | 1.57 | 3.0e-02 |
| CEBPE\_f1 |  | C48E7.11 | 52 | 1.47 | 3.0e-02 |
| Irx4\_2242 |  | irx-1 (-0.55) | 44 | 1.57 | 3.0e-02 |
| Tcf7\_0950 |  | pop-1 (-0.56) | 34 | 1.74 | 3.1e-02 |
| Fer1\_da\_SANGER\_10\_FBgn0037475 |  | ceh-32 lin-32 | 80 | 1.23 | 3.1e-02 |
| TLX1\_f1 |  | ceh-19 | 21 | 2.20 | 3.1e-02 |
| MA0095.2 |  | lsy-2 | 54 | 1.45 | 3.1e-02 |
| pTH10040 |  | slr-2 | 58 | 1.41 | 3.1e-02 |
| FLI1\_4 |  | lin-1 | 13 | 2.95 | 3.2e-02 |
| eve\_FlyReg\_FBgn0000606 |  | ceh-53 (-0.65) hmg-12 | 44 | 1.56 | 3.2e-02 |
| pTH4269 |  | nhr-177 | 48 | 1.51 | 3.2e-02 |
| pTH6108 |  | lin-31 | 44 | 1.56 | 3.3e-02 |
| V$GATA6\_01 |  | elt-1 | 37 | 1.67 | 3.3e-02 |
| pTH9215 |  | C34D1.1 | 36 | 1.69 | 3.3e-02 |
| MA0066.1 |  | nhr-43 (0.52) nhr-6 | 36 | 1.69 | 3.3e-02 |
| pTH5976 |  | irx-1 (-0.55) | 31 | 1.80 | 3.4e-02 |
| pTH9901 |  | D1005.3 T27F2.4 | 43 | 1.57 | 3.5e-02 |
| POU2F2\_2 |  | ceh-18 (0.57) | 21 | 2.17 | 3.5e-02 |
| En1\_3123 |  | ceh-16 | 89 | 1.16 | 3.5e-02 |
| pTH9164 |  | ceh-26 | 46 | 1.53 | 3.6e-02 |
| pTH10797 |  | K11D2.4 | 68 | 1.31 | 3.6e-02 |
| V$GATA1\_01 |  | elt-1 | 41 | 1.60 | 3.7e-02 |
| MA0486.1 |  | Y53C10A.3 | 56 | 1.41 | 3.8e-02 |
| pTH9163 |  | nhr-3 | 49 | 1.49 | 3.8e-02 |
| MA0331.1 |  | unc-120 | 84 | 1.20 | 3.8e-02 |
| pTH6106 |  | nhr-182 | 45 | 1.53 | 3.8e-02 |
| Sox17\_2837 |  | sox-4 (0.6) | 15 | 2.61 | 3.8e-02 |
| Mw140 |  | efl-1 | 14 | 2.72 | 4.0e-02 |
| V$FREAC7\_01 |  | lin-31 | 48 | 1.49 | 4.1e-02 |
| pTH9222 |  | mel-28 (-0.53) | 54 | 1.42 | 4.3e-02 |
| Otx1\_2 |  | ceh-45 | 42 | 1.57 | 4.3e-02 |
| pTH9237 |  | mel-28 (-0.53) | 48 | 1.49 | 4.3e-02 |
| V$GATA1\_02 |  | elt-1 | 40 | 1.59 | 4.3e-02 |
| RFX1\_f1 |  | daf-19 (0.54) | 39 | 1.60 | 4.5e-02 |
| MA0222.1 |  | ceh-20 | 61 | 1.36 | 4.6e-02 |
| pTH5270 |  | ngn-1 | 41 | 1.57 | 4.7e-02 |
| I$ELF1\_01 |  | sox-4 (0.6) grh-1 | 58 | 1.38 | 4.7e-02 |
| pTH9279 |  | Y116A8C.22 | 29 | 1.81 | 4.8e-02 |
| YY1\_1 |  | lsy-2 | 48 | 1.48 | 4.8e-02 |
| MYB\_f1 |  | D1081.8 | 82 | 1.20 | 4.9e-02 |
| pTH10038 |  | gei-3 (0.63) | 40 | 1.58 | 5.0e-02 |
| pTH9137 |  | nhr-65 | 43 | 1.54 | 5.0e-02 |

### Correlated (and anti-correlated) transcription factors

|  |  |
| --- | --- |
| **Transcription factor** | **Correlation** |
| nhr-145 | 0.84 |
| hlh-10 | 0.78 |
| ztf-9 | 0.75 |
| nhr-101 | 0.74 |
| nhr-105 | 0.73 |
| camt-1 | 0.73 |
| nhr-138 | 0.73 |
| nhr-40 | 0.73 |
| mml-1 | 0.72 |
| nhr-100 | 0.72 |
| nhr-95 | 0.72 |
| saeg-1 | 0.68 |
| ccch-1 | 0.67 |
| tag-97 | 0.66 |
| ztf-26 | 0.65 |
| jun-1 | 0.64 |
| madf-4 | 0.63 |
| nhr-198 | 0.63 |
| gei-3 | 0.63 |
| tag-68 | 0.63 |
| nhr-36 | 0.62 |
| nhr-190 | 0.62 |
| mbr-1 | 0.62 |
| nhr-78 | 0.62 |
| ceh-31 | 0.61 |
| attf-3 | -0.52 |
| nhr-106 | -0.53 |
| mel-28 | -0.53 |
| Y53G8AR.9 | -0.54 |
| snpc-1.2 | -0.54 |
| bed-2 | -0.55 |
| sex-1 | -0.55 |
| irx-1 | -0.55 |
| Y53F4B.3 | -0.55 |
| F27D4.4 | -0.55 |
| ham-1 | -0.55 |
| hmg-3 | -0.55 |
| C52E12.1 | -0.55 |
| Y82E9BR.1 | -0.56 |
| pop-1 | -0.56 |
| hmg-5 | -0.57 |
| lin-40 | -0.59 |
| gla-3 | -0.59 |
| ztf-11 | -0.61 |
| Y82E9BR.17 | -0.62 |
| zip-7 | -0.62 |
| ztf-13 | -0.63 |
| sup-35 | -0.63 |
| ceh-53 | -0.65 |
| duxl-1 | -0.67 |

### ChIP peaks enriched

|  |  |  |  |  |
| --- | --- | --- | --- | --- |
| **Gene** | **Experiment** | **Number of upstream peaks** | **Enrichment** | **FDR corrected p** |
| ces-1 | CES-1\_Embryos | 27 | 2.76 | 4.3e-05 |
| egl-5 | EGL-5\_Larvae-L3-stage | 34 | 2.17 | 2.3e-04 |
| nhr-129 | NHR-129\_Larvae-L2-stage | 42 | 1.80 | 1.1e-03 |
| lsy-2 | LSY-2\_Larvae-L4-stage | 13 | 3.93 | 1.2e-03 |
| sem-4 | SEM-4\_Larvae-L2-stage | 35 | 1.97 | 1.3e-03 |
| efl-1 | EFL-1\_Larvae-L1-stage | 19 | 2.86 | 1.3e-03 |
| zag-1 | ZAG-1\_Larvae-L2-stage | 19 | 2.82 | 1.6e-03 |
| nhr-21 | NHR-21\_Larvae-L2-stage | 9 | 5.59 | 1.6e-03 |
| C34F6.9 | C34F6.9\_Larvae-L2-stage | 34 | 1.95 | 1.9e-03 |
| gei-11 | GEI-11\_Larvae-L2-stage | 16 | 3.15 | 2.0e-03 |
| eor-1 | EOR-1\_Larvae-L3-stage | 23 | 2.40 | 2.5e-03 |
| sax-3 | SAX-3\_Larvae-L2-stage | 19 | 2.70 | 2.7e-03 |
| nfya-1 | NFYA-1\_Late-Embryos | 20 | 2.51 | 4.4e-03 |
| nfya-1 | NFYA-1\_Larvae-L3-stage | 16 | 2.86 | 5.4e-03 |
| hlh-30 | HLH-30\_Larvae-L4-stage | 13 | 3.27 | 6.6e-03 |
| ham-1 | HAM-1\_Larvae-L4-stage | 34 | 1.82 | 7.0e-03 |
| lsy-2 | LSY-2\_Larvae-L1-stage | 36 | 1.72 | 1.2e-02 |
| elt-1 | ELT-1\_Larvae-L3-stage | 12 | 3.14 | 1.6e-02 |
| aha-1 | AHA-1\_Larvae-L4-stage | 13 | 2.96 | 1.6e-02 |
| F45C12.2 | F45C12.2\_Fed-L1-stage-larvae | 17 | 2.46 | 1.7e-02 |
| gei-11 | GEI-11\_Fed-L1-stage-larvae | 16 | 2.54 | 1.8e-02 |
| unc-62 | UNC-62\_Day-Four-Young-Adult | 15 | 2.63 | 1.9e-02 |
| unc-62 | UNC-62\_Young-adult-Day-4 | 15 | 2.63 | 1.9e-02 |
| pha-4 | PHA-4\_Young-adult | 10 | 3.50 | 2.0e-02 |
| alr-1 | ALR-1\_Larvae-L2-stage | 17 | 2.40 | 2.1e-02 |
| daf-12 | DAF-12\_Larvae-L4-stage | 3 | 18.14 | 2.1e-02 |
| ceh-16 | CEH-16\_Larvae-L2-stage | 11 | 3.12 | 2.7e-02 |
| zag-1 | ZAG-1\_Larvae-L4-stage | 15 | 2.48 | 3.1e-02 |
| ces-1 | CES-1\_Fed-L1-stage-larvae | 13 | 2.68 | 3.5e-02 |
| lin-13 | LIN-13\_Larvae-L2-stage | 11 | 2.92 | 4.3e-02 |
| ceh-38 | CEH-38\_Larvae-L4-stage | 11 | 2.91 | 4.5e-02 |
| lsy-2 | LSY-2\_Fed-L1-stage-larvae | 28 | 1.75 | 4.9e-02 |
