# Supplementary material for: Overlapping cell population expression profiling and regulatory inference in C. elegans
Source: BMC Genomics. 2016 Feb 29;17:159. doi: 10.1186/s12864-016-2482-z (PMC4772325; doi:10.1186/s12864-016-2482-z)
Supplement: Additional file 13: — Web supplement. (DOC 21 kb) [file 12864_2016_2482_MOESM13_ESM.zip › sortWeb/clusters/hier.300.clusters/161.html]

Cluster 161 

## Cluster 161

### Expression

| cnd-1 rep. 1 | cnd-1 rep. 2 | cnd-1 rep. 3 | pha-4 rep. 1 | pha-4 rep. 2 | pha-4 rep. 3 | ceh-27 | ceh-36 | ceh-6 | F21D5.9 | mir-57 | mls-2 | pal-1 | pros-1 | ttx-3 | unc-130 | hlh-16 | irx-1 | ceh-6 (+) hlh-16 (+) | ceh-6 (+) hlh-16 (-) | ceh-6 (-) hlh-16 (+) | cnd-1 singlets | pha-4 singlets | 0 | 60 | 120 | 150 | 180 | 240 | 330 | 390 | 420 | 480 | 540 | 570 | 600 | 630 | 660 | NAME | Functional description |
| --- | --- | --- | --- | --- | --- | --- | --- | --- | --- | --- | --- | --- | --- | --- | --- | --- | --- | --- | --- | --- | --- | --- | --- | --- | --- | --- | --- | --- | --- | --- | --- | --- | --- | --- | --- | --- | --- | --- | --- |
|  |  |  |  |  |  |  |  |  |  |  |  |  |  |  |  |  |  |  |  |  |  |  |  |  |  |  |  |  |  |  |  |  |  |  |  |  |  | T21C12.9 |  |
|  |  |  |  |  |  |  |  |  |  |  |  |  |  |  |  |  |  |  |  |  |  |  |  |  |  |  |  |  |  |  |  |  |  |  |  |  |  | F40F11.5 |  |
|  |  |  |  |  |  |  |  |  |  |  |  |  |  |  |  |  |  |  |  |  |  |  |  |  |  |  |  |  |  |  |  |  |  |  |  |  |  | Y43E12A.3 |  |
|  |  |  |  |  |  |  |  |  |  |  |  |  |  |  |  |  |  |  |  |  |  |  |  |  |  |  |  |  |  |  |  |  |  |  |  |  |  | C24A3.10 |  |
|  |  |  |  |  |  |  |  |  |  |  |  |  |  |  |  |  |  |  |  |  |  |  |  |  |  |  |  |  |  |  |  |  |  |  |  |  |  | *anr-2* | Antisense Non-coding RNA |
|  |  |  |  |  |  |  |  |  |  |  |  |  |  |  |  |  |  |  |  |  |  |  |  |  |  |  |  |  |  |  |  |  |  |  |  |  |  | Y61A9LA.5 |  |
|  |  |  |  |  |  |  |  |  |  |  |  |  |  |  |  |  |  |  |  |  |  |  |  |  |  |  |  |  |  |  |  |  |  |  |  |  |  | C25A8.9 |  |
|  |  |  |  |  |  |  |  |  |  |  |  |  |  |  |  |  |  |  |  |  |  |  |  |  |  |  |  |  |  |  |  |  |  |  |  |  |  | R07D5.6 |  |
|  |  |  |  |  |  |  |  |  |  |  |  |  |  |  |  |  |  |  |  |  |  |  |  |  |  |  |  |  |  |  |  |  |  |  |  |  |  | F42H11.1 |  |
|  |  |  |  |  |  |  |  |  |  |  |  |  |  |  |  |  |  |  |  |  |  |  |  |  |  |  |  |  |  |  |  |  |  |  |  |  |  | K11C4.9 |  |
|  |  |  |  |  |  |  |  |  |  |  |  |  |  |  |  |  |  |  |  |  |  |  |  |  |  |  |  |  |  |  |  |  |  |  |  |  |  | B0495.15 |  |
|  |  |  |  |  |  |  |  |  |  |  |  |  |  |  |  |  |  |  |  |  |  |  |  |  |  |  |  |  |  |  |  |  |  |  |  |  |  | *vha-13* | Vacuolar H ATPase |
|  |  |  |  |  |  |  |  |  |  |  |  |  |  |  |  |  |  |  |  |  |  |  |  |  |  |  |  |  |  |  |  |  |  |  |  |  |  | *vha-12* | Vacuolar H ATPase |
|  |  |  |  |  |  |  |  |  |  |  |  |  |  |  |  |  |  |  |  |  |  |  |  |  |  |  |  |  |  |  |  |  |  |  |  |  |  | Y47A7.2 |  |
|  |  |  |  |  |  |  |  |  |  |  |  |  |  |  |  |  |  |  |  |  |  |  |  |  |  |  |  |  |  |  |  |  |  |  |  |  |  | F47B10.8 |  |
|  |  |  |  |  |  |  |  |  |  |  |  |  |  |  |  |  |  |  |  |  |  |  |  |  |  |  |  |  |  |  |  |  |  |  |  |  |  | *spe-9* | defective SPErmatogenesis |
|  |  |  |  |  |  |  |  |  |  |  |  |  |  |  |  |  |  |  |  |  |  |  |  |  |  |  |  |  |  |  |  |  |  |  |  |  |  | F52H2.4 |  |
|  |  |  |  |  |  |  |  |  |  |  |  |  |  |  |  |  |  |  |  |  |  |  |  |  |  |  |  |  |  |  |  |  |  |  |  |  |  | *pxn-1* | PeroXidasiN (Drosophila peroxidase) homolog |
|  |  |  |  |  |  |  |  |  |  |  |  |  |  |  |  |  |  |  |  |  |  |  |  |  |  |  |  |  |  |  |  |  |  |  |  |  |  | *gbb-1* | GABA B receptor subunit |
|  |  |  |  |  |  |  |  |  |  |  |  |  |  |  |  |  |  |  |  |  |  |  |  |  |  |  |  |  |  |  |  |  |  |  |  |  |  | *twk-30* | TWiK family of potassium channels |
|  |  |  |  |  |  |  |  |  |  |  |  |  |  |  |  |  |  |  |  |  |  |  |  |  |  |  |  |  |  |  |  |  |  |  |  |  |  | *gbb-2* | GABA B receptor subunit |
|  |  |  |  |  |  |  |  |  |  |  |  |  |  |  |  |  |  |  |  |  |  |  |  |  |  |  |  |  |  |  |  |  |  |  |  |  |  | *gly-12* | GLYcosylation related |
|  |  |  |  |  |  |  |  |  |  |  |  |  |  |  |  |  |  |  |  |  |  |  |  |  |  |  |  |  |  |  |  |  |  |  |  |  |  | *ssu-2* | Suppressor of Stomatin mutant Uncoordination |
|  |  |  |  |  |  |  |  |  |  |  |  |  |  |  |  |  |  |  |  |  |  |  |  |  |  |  |  |  |  |  |  |  |  |  |  |  |  | *unc-38* | UNCoordinated |
|  |  |  |  |  |  |  |  |  |  |  |  |  |  |  |  |  |  |  |  |  |  |  |  |  |  |  |  |  |  |  |  |  |  |  |  |  |  | F21C10.3 |  |
|  |  |  |  |  |  |  |  |  |  |  |  |  |  |  |  |  |  |  |  |  |  |  |  |  |  |  |  |  |  |  |  |  |  |  |  |  |  | Y52B11A.4 |  |
|  |  |  |  |  |  |  |  |  |  |  |  |  |  |  |  |  |  |  |  |  |  |  |  |  |  |  |  |  |  |  |  |  |  |  |  |  |  | *cca-1* | Calcium Channel, Alpha subunit |
|  |  |  |  |  |  |  |  |  |  |  |  |  |  |  |  |  |  |  |  |  |  |  |  |  |  |  |  |  |  |  |  |  |  |  |  |  |  | *msi-1* | MuSashI (fly neural) family |
|  |  |  |  |  |  |  |  |  |  |  |  |  |  |  |  |  |  |  |  |  |  |  |  |  |  |  |  |  |  |  |  |  |  |  |  |  |  | *glb-2* | GLoBin related |
|  |  |  |  |  |  |  |  |  |  |  |  |  |  |  |  |  |  |  |  |  |  |  |  |  |  |  |  |  |  |  |  |  |  |  |  |  |  | *abts-3* | Anion/Bicarbonate TranSporter family |
|  |  |  |  |  |  |  |  |  |  |  |  |  |  |  |  |  |  |  |  |  |  |  |  |  |  |  |  |  |  |  |  |  |  |  |  |  |  | *glc-4* | Glutamate-gated ChLoride channel |
|  |  |  |  |  |  |  |  |  |  |  |  |  |  |  |  |  |  |  |  |  |  |  |  |  |  |  |  |  |  |  |  |  |  |  |  |  |  | ZK813.4 |  |
|  |  |  |  |  |  |  |  |  |  |  |  |  |  |  |  |  |  |  |  |  |  |  |  |  |  |  |  |  |  |  |  |  |  |  |  |  |  | *arrd-17* | ARRestin Domain protein |
|  |  |  |  |  |  |  |  |  |  |  |  |  |  |  |  |  |  |  |  |  |  |  |  |  |  |  |  |  |  |  |  |  |  |  |  |  |  | *dyc-1* | DYstrophin-like phenotype and CAPON related |
|  |  |  |  |  |  |  |  |  |  |  |  |  |  |  |  |  |  |  |  |  |  |  |  |  |  |  |  |  |  |  |  |  |  |  |  |  |  | F20D1.3 |  |
|  |  |  |  |  |  |  |  |  |  |  |  |  |  |  |  |  |  |  |  |  |  |  |  |  |  |  |  |  |  |  |  |  |  |  |  |  |  | R10E9.2 |  |
|  |  |  |  |  |  |  |  |  |  |  |  |  |  |  |  |  |  |  |  |  |  |  |  |  |  |  |  |  |  |  |  |  |  |  |  |  |  | R12H7.4 |  |
|  |  |  |  |  |  |  |  |  |  |  |  |  |  |  |  |  |  |  |  |  |  |  |  |  |  |  |  |  |  |  |  |  |  |  |  |  |  | *ipgm-1* | cofactor-Independent PhosphoGlycerate Mutase homolog |
|  |  |  |  |  |  |  |  |  |  |  |  |  |  |  |  |  |  |  |  |  |  |  |  |  |  |  |  |  |  |  |  |  |  |  |  |  |  | *npr-5* | NeuroPeptide Receptor family |
|  |  |  |  |  |  |  |  |  |  |  |  |  |  |  |  |  |  |  |  |  |  |  |  |  |  |  |  |  |  |  |  |  |  |  |  |  |  | *aat-5* | Amino Acid Transporter |
|  |  |  |  |  |  |  |  |  |  |  |  |  |  |  |  |  |  |  |  |  |  |  |  |  |  |  |  |  |  |  |  |  |  |  |  |  |  | *sms-1* | SphingoMyelin Synthase |
|  |  |  |  |  |  |  |  |  |  |  |  |  |  |  |  |  |  |  |  |  |  |  |  |  |  |  |  |  |  |  |  |  |  |  |  |  |  | *vha-11* | Vacuolar H ATPase |
|  |  |  |  |  |  |  |  |  |  |  |  |  |  |  |  |  |  |  |  |  |  |  |  |  |  |  |  |  |  |  |  |  |  |  |  |  |  | *vha-8* | Vacuolar H ATPase |
|  |  |  |  |  |  |  |  |  |  |  |  |  |  |  |  |  |  |  |  |  |  |  |  |  |  |  |  |  |  |  |  |  |  |  |  |  |  | *mak-1* | MAP kinase Activated protein Kinase |
|  |  |  |  |  |  |  |  |  |  |  |  |  |  |  |  |  |  |  |  |  |  |  |  |  |  |  |  |  |  |  |  |  |  |  |  |  |  | *acs-19* | fatty Acid CoA Synthetase family |
|  |  |  |  |  |  |  |  |  |  |  |  |  |  |  |  |  |  |  |  |  |  |  |  |  |  |  |  |  |  |  |  |  |  |  |  |  |  | *ddo-2* |  |
|  |  |  |  |  |  |  |  |  |  |  |  |  |  |  |  |  |  |  |  |  |  |  |  |  |  |  |  |  |  |  |  |  |  |  |  |  |  | Y12A6A.2 |  |
|  |  |  |  |  |  |  |  |  |  |  |  |  |  |  |  |  |  |  |  |  |  |  |  |  |  |  |  |  |  |  |  |  |  |  |  |  |  | R10F2.5 |  |
|  |  |  |  |  |  |  |  |  |  |  |  |  |  |  |  |  |  |  |  |  |  |  |  |  |  |  |  |  |  |  |  |  |  |  |  |  |  | F15G9.5 |  |
|  |  |  |  |  |  |  |  |  |  |  |  |  |  |  |  |  |  |  |  |  |  |  |  |  |  |  |  |  |  |  |  |  |  |  |  |  |  | *cpna-5* | CoPiNe domain protein, Atypical |
|  |  |  |  |  |  |  |  |  |  |  |  |  |  |  |  |  |  |  |  |  |  |  |  |  |  |  |  |  |  |  |  |  |  |  |  |  |  | *shk-1* | SHaKer family of potassium channels |
|  |  |  |  |  |  |  |  |  |  |  |  |  |  |  |  |  |  |  |  |  |  |  |  |  |  |  |  |  |  |  |  |  |  |  |  |  |  | ZC123.4 |  |
|  |  |  |  |  |  |  |  |  |  |  |  |  |  |  |  |  |  |  |  |  |  |  |  |  |  |  |  |  |  |  |  |  |  |  |  |  |  | *unc-105* | UNCoordinated |
|  |  |  |  |  |  |  |  |  |  |  |  |  |  |  |  |  |  |  |  |  |  |  |  |  |  |  |  |  |  |  |  |  |  |  |  |  |  | *unc-49* | UNCoordinated |
|  |  |  |  |  |  |  |  |  |  |  |  |  |  |  |  |  |  |  |  |  |  |  |  |  |  |  |  |  |  |  |  |  |  |  |  |  |  | *shw-1* | SHaW family of potassium channels |
|  |  |  |  |  |  |  |  |  |  |  |  |  |  |  |  |  |  |  |  |  |  |  |  |  |  |  |  |  |  |  |  |  |  |  |  |  |  | *zyx-1* | ZYXin |
|  |  |  |  |  |  |  |  |  |  |  |  |  |  |  |  |  |  |  |  |  |  |  |  |  |  |  |  |  |  |  |  |  |  |  |  |  |  | *camt-1* | CAMTA (CAlModulin-binding Transcriptional activator) |
|  |  |  |  |  |  |  |  |  |  |  |  |  |  |  |  |  |  |  |  |  |  |  |  |  |  |  |  |  |  |  |  |  |  |  |  |  |  | *lpr-2* | LiPocalin-Related protein |
|  |  |  |  |  |  |  |  |  |  |  |  |  |  |  |  |  |  |  |  |  |  |  |  |  |  |  |  |  |  |  |  |  |  |  |  |  |  | *kvs-5* | K (potassium) Voltage-Sensitive channel subunit |
|  |  |  |  |  |  |  |  |  |  |  |  |  |  |  |  |  |  |  |  |  |  |  |  |  |  |  |  |  |  |  |  |  |  |  |  |  |  | *gly-4* | GLYcosylation related |
|  |  |  |  |  |  |  |  |  |  |  |  |  |  |  |  |  |  |  |  |  |  |  |  |  |  |  |  |  |  |  |  |  |  |  |  |  |  | F02A9.1 |  |
|  |  |  |  |  |  |  |  |  |  |  |  |  |  |  |  |  |  |  |  |  |  |  |  |  |  |  |  |  |  |  |  |  |  |  |  |  |  | *far-1* | Fatty Acid/Retinol binding protein |
|  |  |  |  |  |  |  |  |  |  |  |  |  |  |  |  |  |  |  |  |  |  |  |  |  |  |  |  |  |  |  |  |  |  |  |  |  |  | *far-2* | Fatty Acid/Retinol binding protein |
|  |  |  |  |  |  |  |  |  |  |  |  |  |  |  |  |  |  |  |  |  |  |  |  |  |  |  |  |  |  |  |  |  |  |  |  |  |  | ZK418.2 |  |
|  |  |  |  |  |  |  |  |  |  |  |  |  |  |  |  |  |  |  |  |  |  |  |  |  |  |  |  |  |  |  |  |  |  |  |  |  |  | ZK994.9 |  |

### Phenotypes enriched

none found

### Anatomy terms enriched

none found

### GO terms enriched

|  |  |  |
| --- | --- | --- |
| **GO term** | **Number of genes** | **FDR-corrected p-value** |
| ion transmembrane transporter activity | 15 | 5.9e-07 |
| substrate-specific transporter activity | 15 | 5.4e-06 |
| monovalent inorganic cation transmembrane transporter activity | 9 | 1.3e-04 |
| ATP hydrolysis coupled proton transport | 4 | 5.8e-04 |
| regulation of ion transmembrane transport | 4 | 1.3e-03 |
| substrate-specific channel activity | 9 | 1.3e-03 |
| passive transmembrane transporter activity | 9 | 2.2e-03 |
| hydrogen ion transmembrane transport | 4 | 2.7e-03 |
| cation channel complex | 4 | 4.2e-03 |
| proton-transporting ATPase activity, rotational mechanism | 3 | 4.2e-03 |
| hydrogen transport | 4 | 4.7e-03 |
| monovalent inorganic cation transport | 5 | 4.8e-03 |
| voltage-gated cation channel activity | 4 | 5.6e-03 |
| voltage-gated channel activity | 4 | 1.1e-02 |
| membrane | 25 | 1.5e-02 |
| transmembrane transporter complex | 4 | 1.5e-02 |
| voltage-gated potassium channel complex | 3 | 2.2e-02 |
| protein homooligomerization | 3 | 2.3e-02 |
| potassium ion transmembrane transport | 4 | 2.5e-02 |
| transmembrane transporter activity | 6 | 3.5e-02 |
| metal ion transmembrane transporter activity | 6 | 3.7e-02 |
| regulation of transport | 4 | 4.2e-02 |

### Expression clusters enriched

|  |  |  |  |
| --- | --- | --- | --- |
| **Group name** | **Number in cluster** | **Enrichment** | **FDR corrected p** |
| Genes enriched in muscle cells (0hr muscle dataset). Dissociated myo-3::GFP embryos were cultured for 0 hours before FACS sorting. | 16 | 5.07 | 2.70e-05 |
| Total muscle enriched genes (complete list of non-overlapping genes from the 0hr and 24hr muscle enriched datasets). | 20 | 3.75 | 5.75e-05 |
| Genes enriched in muscle cells (24hr muscle dataset). Dissociated myo-3::GFP embryos were cultured for 24 hours before FACS sorting. | 15 | 3.94 | 1.31e-03 |
| Genes significantly enriched (> 2x, FDR < 5%) in a particular cell-type versus a reference sample of all cells at the same stage. WBPaper00037950:A-class-motor-neurons\_larva\_enriched | 13 | 4.55 | 1.37e-03 |
| Genes enriched in body wall muscle AIN-2 miRISCs. Pmyo-3-ain-2-gfp IP was performed in mixed stage worms. | 11 | 5.07 | 2.77e-03 |
| Genes significantly enriched (> 2x, FDR < 5%) in a particular cell-type versus a reference sample of all cells at the same stage. WBPaper00037950:GABAergic-motor-neurons\_embryo\_enriched | 9 | 6.02 | 4.95e-03 |
| Genes enriched in neuronal miRNA-induced silencing complexes (miRISC) as detected by immunoprecipitations and microarray analysis. | 12 | 4.18 | 6.58e-03 |
| Genes enriched in HLH-1 heat shock dataset. | 23 | 2.39 | 8.30e-03 |
| Larval Pan-neural Enriched Genes. | 18 | 2.81 | 1.01e-02 |
| mRNAs that were significantly enriched in the AIN-2 immunoprecipitation samples, compared to the control total mRNAs in the input extracts (p < 0.01). | 19 | 2.59 | 1.69e-02 |
| Genes that show selective expression in a subset of cell types vs broadly expressed in many cell types. Correspond to 20% - 57% of enriched\_genes for a given cell type. WBPaper00037950:GABAergic-motor-neurons\_embryo\_SelectivelyEnriched | 5 | 10.97 | 2.21e-02 |
| Genes significantly enriched (> 2x, FDR < 5%) in a particular cell-type versus a reference sample of all cells at the same stage. WBPaper00037950:dopaminergic-neurons\_larva\_enriched | 15 | 2.94 | 2.80e-02 |
| WT-Pico Pan-neural Enriched Genes, with genes found multiple times in a single dataset removed (without dups). | 17 | 2.56 | 4.59e-02 |

### Motifs enriched

|  |  |  |  |  |  |
| --- | --- | --- | --- | --- | --- |
| **Motif** | **Logo** | **Possible orthologs** | **Number of motifs in cluster** | **Enrichment** | **FDR corrected p** |
| MA0039.2 |  | klf-2 klf-1 | 25 | 3.63 | 2.0e-06 |
| Pou3f3\_3235 |  | ceh-6 | 47 | 1.99 | 2.4e-06 |
| pTH9256 |  | ceh-18 (0.61) | 50 | 1.84 | 4.5e-06 |
| MA0599.1 |  | klf-2 klf-1 | 19 | 4.65 | 5.1e-06 |
| pTH9381 |  | ceh-18 (0.61) | 49 | 1.83 | 1.0e-05 |
| LHX3\_f1 |  | lin-39 unc-86 cfi-1 lim-7 | 32 | 2.57 | 1.8e-05 |
| MA0078.1 |  | gei-3 (0.55) pop-1 (-0.52) sox-4 (0.51) C05C9.3 | 38 | 2.21 | 2.1e-05 |
| MA0180.1 |  | ceh-53 (-0.59) pha-2 (-0.59) lim-4 ceh-31 ceh-16 ceh-12 ceh-45 egl-5 ceh-14 ceh-30 ceh-23 lin-39 ceh-36 dsc-1 ceh-2 alr-1 cog-1 ceh-10 lim-7 ceh-43 and 5 others  [full list] | 46 | 1.88 | 2.5e-05 |
| pTH9297 |  | ceh-18 (0.61) | 50 | 1.74 | 2.8e-05 |
| CG31670\_SANGER\_5\_FBgn0031375 |  | F21A9.2 (0.56) CELE\_Y38H8A.5 | 48 | 1.79 | 3.4e-05 |
| Mv90 |  | mef-2 | 55 | 1.58 | 3.9e-05 |
| ZN384\_f1 |  | lin-29 K11D2.4 | 51 | 1.69 | 4.5e-05 |
| pTH3086 |  | klf-2 klf-1 sptf-3 | 23 | 3.28 | 4.7e-05 |
| MA0537.1 |  | blmp-1 | 49 | 1.74 | 4.8e-05 |
| pTH9242 |  | mel-28 | 59 | 1.46 | 5.4e-05 |
| pTH9180 |  | mel-28 let-381 mef-2 Y116A8C.22 | 55 | 1.57 | 5.7e-05 |
| Gbx2\_3110 |  | ceh-53 (-0.59) ceh-12 ceh-45 lin-39 alr-1 ceh-43 ceh-1 | 30 | 2.56 | 5.9e-05 |
| MA0499.1 |  | lin-32 hlh-1 hlh-15 hlh-11 hlh-14 | 42 | 1.95 | 6.4e-05 |
| SPDEF\_6 |  | nhr-100 lin-1 lin-39 | 41 | 1.98 | 6.9e-05 |
| HLH4C\_da\_SANGER\_5\_4\_FBgn0011277 |  | hlh-2 hlh-1 hlh-15 hlh-8 K02D7.2 | 43 | 1.87 | 1.2e-04 |
| FOXP2\_si |  | fkh-7 pha-4 fkh-8 lin-31 let-381 daf-16 | 40 | 1.97 | 1.4e-04 |
| COT2\_f1 |  | nhr-6 nhr-2 nhr-71 nhr-213 nhr-68 nhr-10 | 43 | 1.86 | 1.4e-04 |
| pTH8556 |  | pax-2 | 36 | 2.11 | 1.7e-04 |
| ARI3A\_do |  | nhr-208 cfi-1 | 54 | 1.55 | 1.8e-04 |
| pTH9237 |  | mel-28 | 39 | 1.95 | 2.5e-04 |
| rn\_SOLEXA\_5\_FBgn0259172 |  | lin-29 | 37 | 2.02 | 2.8e-04 |
| pTH3046 |  | Y116A8C.22 | 37 | 2.02 | 2.8e-04 |
| HAND1\_si |  | hlh-8 | 17 | 3.82 | 2.9e-04 |
| pTH9335 |  | mel-28 | 51 | 1.59 | 3.1e-04 |
| pTH2280 |  | mnm-2 | 31 | 2.29 | 3.1e-04 |
| V$CEBPA\_01 |  | C48E7.11 | 32 | 2.23 | 3.1e-04 |
| MSX1\_1 |  | ceh-31 lin-39 ceh-43 ceh-1 | 31 | 2.27 | 3.6e-04 |
| pTH9125 |  | egl-13 (0.64) sox-4 (0.51) K11D2.4 | 39 | 1.92 | 3.6e-04 |
| GABPA\_f1 |  | lin-1 C24A1.2 | 33 | 2.17 | 3.6e-04 |
| MA0139.1 |  | F58G1.2 | 35 | 2.08 | 3.6e-04 |
| MA0046.1 |  | ceh-53 (-0.59) let-381 hmbx-1 ceh-43 Y116A8C.22 | 47 | 1.68 | 3.7e-04 |
| pTH6569 |  | ceh-43 | 28 | 2.43 | 4.0e-04 |
| CG34031\_Cell\_FBgn0054031 |  | ceh-24 (0.64) ceh-9 ceh-31 ceh-8 ceh-30 lin-39 alr-1 cog-1 lim-7 ceh-19 ceh-43 ceh-1 | 42 | 1.82 | 4.1e-04 |
| ALX1\_si |  | ceh-14 alr-1 ZC204.2 | 51 | 1.58 | 4.2e-04 |
| Otx1\_2 |  | ceh-53 (-0.59) pha-2 (-0.59) ceh-45 dve-1 ceh-36 alr-1 | 34 | 2.10 | 4.3e-04 |
| tgo\_tai\_SANGER\_5\_FBgn0015014 |  | aha-1 hlh-30 | 38 | 1.94 | 4.3e-04 |
| Plagl1\_0972 |  | Y53H1A.2 | 31 | 2.24 | 4.4e-04 |
| POU3F3\_1 |  | ceh-18 (0.61) sox-4 (0.51) ceh-6 tbp-1 | 35 | 2.06 | 4.4e-04 |
| pTH9222 |  | mel-28 | 54 | 1.50 | 4.4e-04 |
| MA0481.1 |  | fkh-10 (0.6) fkh-7 fkh-8 lin-31 let-381 daf-16 | 43 | 1.78 | 4.7e-04 |
| GATA5\_f1 |  | elt-1 | 43 | 1.78 | 4.7e-04 |
| pTH9142 |  | C34D1.1 gei-11 | 46 | 1.69 | 4.9e-04 |
| pTH6215 |  | ceh-12 lin-39 alr-1 pal-1 ceh-43 | 36 | 2.00 | 5.1e-04 |
| SPDEF\_2 |  | lin-1 ztf-14 | 34 | 2.08 | 5.1e-04 |
| Lhx4\_1719 |  | ceh-16 lim-7 | 30 | 2.28 | 5.2e-04 |
| MA0262.1 |  | mab-3 | 44 | 1.73 | 5.9e-04 |
| MA0543.1 |  | daf-8 eor-1 | 41 | 1.81 | 6.3e-04 |
| En1\_3123 |  | ceh-53 (-0.59) ceh-16 | 38 | 1.91 | 6.5e-04 |
| pTH10623 |  | scrt-1 | 32 | 2.15 | 6.6e-04 |
| EMX2\_2 |  | ceh-16 ceh-2 | 40 | 1.84 | 6.6e-04 |
| pTH10027 |  | M03D4.4 | 36 | 1.98 | 6.7e-04 |
| exd\_FlyReg\_FBgn0000611 |  | ceh-20 let-381 cfi-1 | 42 | 1.78 | 7.1e-04 |
| Fer1\_da\_SANGER\_10\_FBgn0037475 |  | lin-39 lin-32 ceh-32 | 40 | 1.84 | 7.2e-04 |
| FOXC1\_3 |  | lin-31 let-381 | 54 | 1.48 | 7.2e-04 |
| Pou2f3\_3986 |  | ceh-18 (0.61) | 45 | 1.69 | 7.3e-04 |
| pTH3037 |  | hlh-1 hlh-15 | 38 | 1.89 | 7.9e-04 |
| SOX2\_4 |  | sox-4 (0.51) dmd-4 | 33 | 2.08 | 8.0e-04 |
| sqz\_SANGER\_5\_FBgn0010768 |  | fkh-7 mel-28 lin-29 | 54 | 1.48 | 8.6e-04 |
| pTH9177 |  | F10B5.3 (0.52) hsf-1 Y53C10A.3 | 54 | 1.48 | 8.6e-04 |
| pTH5169 |  | cfi-1 | 50 | 1.56 | 8.8e-04 |
| HXA10\_f1 |  | hbl-1 lin-39 php-3 | 32 | 2.11 | 9.2e-04 |
| MA0483.1 |  | odd-1 lim-6 | 42 | 1.76 | 9.4e-04 |
| pTH9254 |  | mel-28 | 53 | 1.49 | 9.7e-04 |
| PURA\_f1 |  | Y53H1A.2 klf-2 plp-2 | 42 | 1.75 | 1.0e-03 |
| pTH3469 |  | nhr-19 nhr-2 nhr-213 | 35 | 1.97 | 1.0e-03 |
| FOXO1\_si |  | irx-1 daf-16 fkh-9 | 51 | 1.53 | 1.1e-03 |
| MA0467.1 |  | ceh-45 tbx-39 | 34 | 2.01 | 1.1e-03 |
| KLF6\_si |  | klf-2 ZC328.2 klf-1 | 36 | 1.93 | 1.1e-03 |
| EPAS1\_si |  | hif-1 ceh-9 | 24 | 2.56 | 1.1e-03 |
| ZBT7A\_f1 |  | ZC328.2 klf-1 | 35 | 1.96 | 1.1e-03 |
| HeLa-S3\_TR4\_UCD |  | nhr-19 lin-1 C24A1.2 | 42 | 1.74 | 1.1e-03 |
| Elf3\_3876 |  | C24A1.2 | 45 | 1.66 | 1.2e-03 |
| ELF2\_f1 |  | lin-1 C24A1.2 | 27 | 2.33 | 1.2e-03 |
| pTH4325 |  | ceh-18 (0.61) | 43 | 1.70 | 1.3e-03 |
| V$POU3F2\_01 |  | ceh-18 (0.61) dmd-3 | 33 | 2.02 | 1.3e-03 |
| STF1\_f1 |  | nhr-68 | 36 | 1.91 | 1.4e-03 |
| pTH9951 |  | mex-6 pal-1 | 55 | 1.43 | 1.5e-03 |
| MA0143.3 |  | pop-1 (-0.52) sox-4 (0.51) | 43 | 1.69 | 1.5e-03 |
| Pou3f1\_3819 |  | ceh-6 | 42 | 1.72 | 1.6e-03 |
| pTH6612 |  | nhr-2 nhr-213 nhr-15 | 36 | 1.89 | 1.6e-03 |
| Barx1\_2877 |  | ceh-43 | 27 | 2.29 | 1.6e-03 |
| Pou2f1\_3081 |  | ceh-18 (0.61) | 42 | 1.71 | 1.7e-03 |
| pTH6423 |  | pha-2 (-0.59) | 35 | 1.93 | 1.7e-03 |
| MSX2\_f1 |  | ceh-45 lin-39 eyg-1 alr-1 ceh-10 ceh-1 | 30 | 2.12 | 1.8e-03 |
| V$HOX13\_01 |  | lin-39 | 47 | 1.59 | 1.8e-03 |
| ems\_FlyReg\_FBgn0000576 |  | ceh-2 | 33 | 1.99 | 1.9e-03 |
| V$CETS1P54\_02 |  | C52B9.2 | 46 | 1.61 | 1.9e-03 |
| Tcfap2a\_2337 |  | aptf-1 | 22 | 2.62 | 1.9e-03 |
| Fli1 |  | lin-1 F19F10.1 C24A1.2 | 44 | 1.65 | 2.0e-03 |
| pnr\_SANGER\_5\_FBgn0003117 |  | elt-1 | 37 | 1.84 | 2.0e-03 |
| pTH6636 |  | egl-5 | 35 | 1.91 | 2.0e-03 |
| pTH9261 |  | dmd-3 | 31 | 2.06 | 2.1e-03 |
| pTH10026 |  | ces-1 (-0.52) ceh-32 F55C5.11 | 30 | 2.09 | 2.3e-03 |
| pTH10037 |  | T22C8.4 ref-2 | 29 | 2.13 | 2.4e-03 |
| pTH10656 |  | hlh-32 hlh-12 hlh-15 hlh-8 ngn-1 | 31 | 2.03 | 2.5e-03 |
| pTH10816 |  | dmd-6 | 46 | 1.59 | 2.6e-03 |
| Hmx1\_3423 |  | ceh-9 | 42 | 1.68 | 2.6e-03 |
| pTH6497 |  | lin-31 | 46 | 1.59 | 2.6e-03 |
| V$S8\_01 |  | ceh-45 | 27 | 2.22 | 2.7e-03 |
| V$PBX1\_01 |  | ceh-12 lin-39 ceh-20 | 37 | 1.81 | 2.7e-03 |
| pTH10031 |  | mbr-1 (0.69) | 27 | 2.21 | 2.8e-03 |
| MA0069.1 |  | pax-3 pax-2 | 37 | 1.81 | 2.8e-03 |
| pTH5778 |  | egl-5 | 28 | 2.16 | 2.9e-03 |
| MA0085.1 |  | lag-1 | 45 | 1.60 | 2.9e-03 |
| pTH10769 |  | Y48G1C.6 | 42 | 1.67 | 3.0e-03 |
| Gsh2\_3990 |  | ceh-31 | 25 | 2.32 | 3.0e-03 |
| pTH9247 |  | dmd-3 C34D1.1 | 37 | 1.80 | 3.1e-03 |
| Lhx1\_2240 |  | lim-7 | 34 | 1.90 | 3.2e-03 |
| pTH6503 |  | ceh-9 ceh-31 ceh-16 lin-39 alr-1 ceh-1 | 32 | 1.97 | 3.2e-03 |
| cad\_FlyReg\_FBgn0000251 |  | lin-39 pal-1 ceh-13 T27F2.4 | 50 | 1.49 | 3.4e-03 |
| pTH9384 |  | cfi-1 | 37 | 1.79 | 3.4e-03 |
| pTH3831 |  | ces-2 C01B12.2 C48E7.11 | 26 | 2.23 | 3.4e-03 |
| MA0482.1 |  | elt-1 ztf-29 | 47 | 1.55 | 3.5e-03 |
| Etv6 |  | lin-1 C24A1.2 | 47 | 1.55 | 3.5e-03 |
| POU3F1\_2 |  | ceh-18 (0.61) unc-86 | 50 | 1.49 | 3.5e-03 |
| V$E47\_02 |  | hlh-2 lin-32 | 38 | 1.76 | 3.6e-03 |
| Isl2\_3430 |  | alr-1 lim-7 | 24 | 2.34 | 3.8e-03 |
| MA0249.1 |  | hlh-32 hlh-15 hlh-8 ngn-1 | 40 | 1.70 | 3.8e-03 |
| V$CREB\_Q2 |  | crh-1 | 34 | 1.87 | 4.0e-03 |
| FLI1\_f1 |  | lin-1 | 37 | 1.78 | 4.1e-03 |
| Hoxb4\_2627 |  | lin-39 | 33 | 1.90 | 4.1e-03 |
| pTH9108 |  | daf-12 | 41 | 1.67 | 4.1e-03 |
| pTH9097 |  | Y116A8C.22 | 54 | 1.41 | 4.1e-03 |
| V$BRN2\_01 |  | ceh-18 (0.61) | 52 | 1.44 | 4.6e-03 |
| pTH8566 |  | lin-54 | 46 | 1.55 | 4.6e-03 |
| Nkx6-3\_3446 |  | cog-1 | 27 | 2.14 | 4.7e-03 |
| K562\_ZBTB7A\_HudsonAlpha |  | ZC328.2 | 33 | 1.88 | 5.0e-03 |
| MAFA\_f1 |  | daf-8 F45H11.6 | 41 | 1.65 | 5.1e-03 |
| REF1 |  | ref-1 mxl-1 aha-1 mxl-2 | 32 | 1.91 | 5.1e-03 |
| Pou3f4\_3773 |  | ceh-6 | 41 | 1.65 | 5.2e-03 |
| MITF\_f1 |  | mxl-1 hlh-30 | 38 | 1.72 | 5.4e-03 |
| Six4\_2860 |  | ceh-32 | 31 | 1.94 | 5.5e-03 |
| V$ZID\_01 |  | ztf-28 | 23 | 2.32 | 6.3e-03 |
| Cdx2\_4272 |  | ceh-13 | 29 | 2.00 | 6.3e-03 |
| HXD10\_f1 |  | nhr-2 php-3 | 34 | 1.82 | 6.4e-03 |
| K562\_SP2\_HudsonAlpha |  | klf-2 | 15 | 3.15 | 6.7e-03 |
| pTH10650 |  | nhr-153 | 37 | 1.73 | 6.7e-03 |
| pTH5250 |  | C48E7.11 | 37 | 1.73 | 7.0e-03 |
| NR2F6\_f1 |  | nhr-2 nhr-62 | 39 | 1.68 | 7.0e-03 |
| pTH2283 |  | odd-2 | 42 | 1.60 | 7.3e-03 |
| GM12878\_ETS1\_HudsonAlpha |  | lin-1 tbx-39 | 37 | 1.72 | 7.9e-03 |
| Etv3 |  | lin-1 | 42 | 1.60 | 8.0e-03 |
| RFX3\_2 |  | daf-19 (0.67) | 11 | 4.05 | 8.2e-03 |
| MA0509.1 |  | daf-19 (0.67) | 37 | 1.71 | 8.3e-03 |
| PROX1\_1 |  | crh-1 ceh-26 | 14 | 3.25 | 8.3e-03 |
| Tbp\_pr781 |  | tbp-1 | 47 | 1.49 | 8.4e-03 |
| RORG\_f1 |  | nhr-213 | 23 | 2.25 | 8.8e-03 |
| Nkx6-1\_2825 |  | cog-1 | 26 | 2.09 | 8.8e-03 |
| Hoxc4\_3491 |  | lin-39 | 32 | 1.85 | 9.1e-03 |
| Hoxa4\_3426 |  | lin-39 | 33 | 1.81 | 9.3e-03 |
| NR2C1\_si |  | nhr-19 nhr-2 | 32 | 1.84 | 9.4e-03 |
| MA0174.1 |  | ceh-24 (0.64) lin-39 pal-1 php-3 | 33 | 1.81 | 9.4e-03 |
| Elf3 |  | C24A1.2 | 46 | 1.51 | 9.5e-03 |
| pTH9365 |  | ceh-18 (0.61) ceh-6 lin-39 | 35 | 1.75 | 9.6e-03 |
| Smad3\_3805 |  | daf-8 | 38 | 1.67 | 9.9e-03 |
| MA0235.1 |  | ceh-48 (0.56) dsc-1 | 46 | 1.50 | 1.0e-02 |
| pTH9080 |  | mnm-2 | 22 | 2.28 | 1.1e-02 |
| Sox17\_2837 |  | sox-4 (0.51) | 35 | 1.74 | 1.1e-02 |
| Tcf7\_0950 |  | pop-1 (-0.52) | 50 | 1.43 | 1.1e-02 |
| pTH10633 |  | R07H5.10 C48E7.11 | 46 | 1.50 | 1.1e-02 |
| V$GATA3\_01 |  | elt-1 | 33 | 1.79 | 1.1e-02 |
| CG7386\_F10-12\_SANGER\_5\_FBgn0035691 |  | F56D1.1 | 30 | 1.89 | 1.2e-02 |
| pTH8991 |  | cey-3 | 26 | 2.05 | 1.2e-02 |
| Nkx3-1\_2923 |  | ceh-24 (0.64) | 26 | 2.05 | 1.2e-02 |
| V$VMYB\_01 |  | D1081.8 | 16 | 2.81 | 1.2e-02 |
| V$TAXCREB\_02 |  | crh-1 zip-3 | 35 | 1.73 | 1.2e-02 |
| TCF4\_2 |  | hlh-2 | 37 | 1.67 | 1.2e-02 |
| Vsx1\_1728 |  | alr-1 | 25 | 2.08 | 1.3e-02 |
| pTH5812 |  | ceh-14 | 21 | 2.31 | 1.3e-02 |
| MA0452.2 |  | B0310.2 ZK177.3 | 38 | 1.65 | 1.3e-02 |
| pTH8983 |  | tag-347 | 16 | 2.78 | 1.3e-02 |
| pTH6447 |  | ceh-19 | 33 | 1.77 | 1.3e-02 |
| pTH10805 |  | ztf-16 | 25 | 2.07 | 1.3e-02 |
| pTH10823 |  | B0310.2 | 33 | 1.77 | 1.3e-02 |
| V$LYF1\_01 |  | mbr-1 (0.69) F26F4.8 | 40 | 1.60 | 1.4e-02 |
| pTH10722 |  | ref-2 eor-1 egrh-3 | 16 | 2.76 | 1.4e-02 |
| Six6\_2267 |  | ceh-34 (-0.51) | 12 | 3.45 | 1.4e-02 |
| Hlxb9\_3422 |  | ceh-12 | 35 | 1.70 | 1.5e-02 |
| pTH9246 |  | lin-31 C34D1.1 | 43 | 1.53 | 1.5e-02 |
| V$CMYB\_01 |  | D1081.8 | 8 | 5.07 | 1.6e-02 |
| pTH5887 |  | lin-39 | 33 | 1.75 | 1.7e-02 |
| pTH4269 |  | nhr-177 | 31 | 1.81 | 1.7e-02 |
| Dlx1\_1741 |  | ceh-43 | 31 | 1.80 | 1.7e-02 |
| I$UBX\_01 |  | lin-39 | 18 | 2.48 | 1.7e-02 |
| MA0161.1 |  | nfi-1 | 34 | 1.72 | 1.8e-02 |
| pTH6641 |  | lin-31 | 44 | 1.50 | 1.8e-02 |
| V$GATA1\_03 |  | elt-1 | 35 | 1.69 | 1.8e-02 |
| FOXI1\_f1 |  | cey-3 nfya-2 ceh-20 lin-31 | 43 | 1.52 | 1.8e-02 |
| pTH9165 |  | ztf-27 | 33 | 1.74 | 1.8e-02 |
| CG14962\_SANGER\_5\_FBgn0035407 |  | C34H4.5 | 43 | 1.51 | 1.9e-02 |
| Meox1\_2310 |  | ceh-31 | 37 | 1.64 | 1.9e-02 |
| pTH9354 |  | ZC328.2 daf-16 | 24 | 2.06 | 1.9e-02 |
| I$DFD\_01 |  | lin-39 | 29 | 1.85 | 1.9e-02 |
| pTH10714 |  | nhr-142 nhr-84 | 40 | 1.57 | 2.0e-02 |
| IRX2\_1 |  | irx-1 | 28 | 1.88 | 2.0e-02 |
| V$GR\_Q6 |  | nhr-255 | 30 | 1.81 | 2.0e-02 |
| pTH9049 |  | ztf-2 | 38 | 1.60 | 2.1e-02 |
| Hoxa13\_3126 |  | ceh-24 (0.64) pal-1 | 38 | 1.60 | 2.1e-02 |
| Hoxa7\_3750 |  | lin-39 | 32 | 1.75 | 2.1e-02 |
| MA0503.1 |  | ceh-24 (0.64) pzf-1 | 37 | 1.62 | 2.1e-02 |
| Hoxd13\_2356 |  | pal-1 | 30 | 1.81 | 2.1e-02 |
| Hoxa6\_1040 |  | lin-39 | 25 | 1.99 | 2.1e-02 |
| pTH9135 |  | pop-1 (-0.52) | 48 | 1.42 | 2.2e-02 |
| pTH10640 |  | dmd-4 | 34 | 1.69 | 2.2e-02 |
| pTH3477 |  | daf-16 | 43 | 1.50 | 2.2e-02 |
| V$YY1\_01 |  | lsy-2 | 43 | 1.50 | 2.2e-02 |
| Mf28 |  | elt-1 | 39 | 1.58 | 2.3e-02 |
| Tcf7l2\_3461 |  | pop-1 (-0.52) | 42 | 1.52 | 2.3e-02 |
| pTH6445 |  | ceh-5 | 30 | 1.80 | 2.3e-02 |
| pTH6591 |  | lin-31 | 45 | 1.46 | 2.4e-02 |
| pTH8671 |  | attf-1 | 28 | 1.86 | 2.4e-02 |
| Hoxa3\_2783 |  | lin-39 | 23 | 2.07 | 2.4e-02 |
| NKX25\_f1 |  | ceh-24 (0.64) dsc-1 | 40 | 1.55 | 2.4e-02 |
| pTH9900 |  | C46E10.8 | 27 | 1.89 | 2.4e-02 |
| Hoxa7\_2668 |  | lin-39 | 24 | 2.02 | 2.4e-02 |
| pTH9043 |  | sem-2 | 39 | 1.57 | 2.4e-02 |
| Elf4 |  | C24A1.2 | 42 | 1.51 | 2.4e-02 |
| pTH8985 |  | athp-1 | 23 | 2.06 | 2.5e-02 |
| pTH9924 |  | nhr-46 | 41 | 1.53 | 2.5e-02 |
| pTH9250 |  | dmd-3 C34D1.1 | 36 | 1.63 | 2.6e-02 |
| MA0118.1 |  | ref-2 | 27 | 1.88 | 2.7e-02 |
| CG8765\_SANGER\_5\_FBgn0036900 |  | H20J04.3 | 28 | 1.84 | 2.7e-02 |
| pTH5928 |  | ceh-34 (-0.51) | 30 | 1.78 | 2.7e-02 |
| CREB1\_f1 |  | crh-1 | 36 | 1.62 | 2.7e-02 |
| V$TATA\_01 |  | tbp-1 | 9 | 4.02 | 2.8e-02 |
| Eip74EF\_FlyReg\_FBgn0000567 |  | C24A1.2 | 44 | 1.47 | 2.8e-02 |
| T-47D\_GATA3\_HudsonAlpha |  | elt-1 | 36 | 1.62 | 2.9e-02 |
| Mafk\_3106 |  | F45H11.6 | 46 | 1.43 | 2.9e-02 |
| Hoxd11\_3873 |  | php-3 | 41 | 1.52 | 2.9e-02 |
| pTH8649 |  | mbr-1 (0.69) | 10 | 3.62 | 2.9e-02 |
| pTH6268 |  | ceh-2 | 33 | 1.68 | 3.0e-02 |
| Dlx2\_2273 |  | ceh-43 | 30 | 1.77 | 3.0e-02 |
| pTH8216 |  | Y116A8C.22 | 31 | 1.73 | 3.2e-02 |
| pTH10718 |  | egl-43 | 29 | 1.79 | 3.2e-02 |
| MAX\_1 |  | mxl-1 | 22 | 2.07 | 3.2e-02 |
| pTH10013 |  | nhr-168 | 27 | 1.85 | 3.2e-02 |
| pTH10772 |  | ceh-52 | 8 | 4.38 | 3.3e-02 |
| Hoxa2\_3079 |  | lin-39 | 35 | 1.62 | 3.3e-02 |
| pTH6003 |  | nhr-134 | 36 | 1.60 | 3.4e-02 |
| MA0579.1 |  | D1081.8 | 15 | 2.58 | 3.5e-02 |
| TBX1\_1 |  | mab-9 | 43 | 1.46 | 3.6e-02 |
| pTH2846 |  | lin-31 | 34 | 1.64 | 3.6e-02 |
| pTH9969 |  | pag-3 | 33 | 1.66 | 3.6e-02 |
| pTH10647 |  | nhr-232 | 37 | 1.57 | 3.7e-02 |
| Sox15\_3457 |  | sox-4 (0.51) | 29 | 1.76 | 3.8e-02 |
| pTH5561 |  | nhr-239 | 37 | 1.57 | 3.8e-02 |
| Bsx\_3483 |  | ceh-31 | 29 | 1.76 | 3.8e-02 |
| pTH9245 |  | ceh-18 (0.61) | 27 | 1.82 | 3.9e-02 |
| Vax2\_3500 |  | C02F12.10 | 28 | 1.79 | 3.9e-02 |
| pTH3819 |  | ceh-18 (0.61) | 37 | 1.57 | 3.9e-02 |
| Hoxa5\_3415 |  | lin-39 | 23 | 1.98 | 3.9e-02 |
| pTH9260 |  | mel-28 | 20 | 2.13 | 3.9e-02 |
| pTH5119 |  | cfi-1 | 21 | 2.08 | 4.0e-02 |
| pTH7875 |  | mel-28 | 20 | 2.13 | 4.1e-02 |
| MA0470.1 |  | F49E12.6 | 37 | 1.56 | 4.1e-02 |
| pTH9163 |  | nhr-3 | 37 | 1.56 | 4.2e-02 |
| pTH10808 |  | ztf-19 | 35 | 1.60 | 4.2e-02 |
| MA0476.1 |  | fos-1 | 35 | 1.60 | 4.2e-02 |
| pTH9709 |  | die-1 | 27 | 1.81 | 4.2e-02 |
| Hey\_SANGER\_5\_FBgn0027788 |  | lin-22 | 34 | 1.62 | 4.3e-02 |
| Tcf3\_3787 |  | pop-1 (-0.52) | 45 | 1.42 | 4.4e-02 |
| HES1\_f1 |  | lin-22 | 34 | 1.61 | 4.5e-02 |
| pTH10041 |  | ztf-29 | 37 | 1.55 | 4.5e-02 |
| Evx1\_3952 |  | ceh-53 (-0.59) | 22 | 2.00 | 4.5e-02 |
| pTH8998 |  | mab-3 | 40 | 1.50 | 4.6e-02 |
| Jundm2\_0911 |  | fos-1 | 26 | 1.82 | 4.8e-02 |
| V$FOXJ2\_02 |  | lin-31 | 12 | 2.88 | 4.9e-02 |
| SRF\_2 |  | unc-120 | 15 | 2.47 | 4.9e-02 |
| V$TCF11\_01 |  | skn-1 | 38 | 1.52 | 4.9e-02 |
| ELK3\_f1 |  | lin-1 | 36 | 1.56 | 4.9e-02 |
| pTH6508 |  | nhr-36 (0.51) | 39 | 1.51 | 4.9e-02 |
| Cdx1\_2245 |  | ceh-13 | 26 | 1.82 | 4.9e-02 |

### Correlated (and anti-correlated) transcription factors

|  |  |
| --- | --- |
| **Transcription factor** | **Correlation** |
| nhr-145 | 0.81 |
| tag-97 | 0.76 |
| ctbp-1 | 0.76 |
| ztf-26 | 0.75 |
| nhr-95 | 0.73 |
| sem-4 | 0.71 |
| ztf-9 | 0.71 |
| camt-1 | 0.70 |
| mbr-1 | 0.69 |
| zip-1 | 0.69 |
| madf-4 | 0.69 |
| nhr-40 | 0.67 |
| hlh-13 | 0.67 |
| daf-19 | 0.67 |
| saeg-1 | 0.67 |
| unc-42 | 0.66 |
| daf-3 | 0.66 |
| nhr-1 | 0.66 |
| nhr-91 | 0.66 |
| Y17G7B.22 | 0.66 |
| zfh-2 | 0.65 |
| tag-68 | 0.65 |
| aptf-4 | 0.65 |
| egl-13 | 0.64 |
| ceh-88 | 0.64 |
| icd-2 | -0.50 |
| ham-1 | -0.50 |
| ceh-34 | -0.51 |
| nhr-13 | -0.51 |
| ces-1 | -0.52 |
| pop-1 | -0.52 |
| ceh-40 | -0.53 |
| dnj-11 | -0.53 |
| C28G1.4 | -0.53 |
| nhr-122 | -0.54 |
| hmg-5 | -0.55 |
| ceh-60 | -0.56 |
| sex-1 | -0.57 |
| Y48A6C.1 | -0.57 |
| ceh-53 | -0.59 |
| pha-2 | -0.59 |
| Y82E9BR.17 | -0.59 |
| ztf-13 | -0.60 |
| F27D4.4 | -0.61 |
| nhr-106 | -0.63 |
| duxl-1 | -0.64 |
| nhr-64 | -0.64 |
| sup-35 | -0.64 |
| Y53F4B.3 | -0.65 |
| zip-7 | -0.68 |

### ChIP peaks enriched

|  |  |  |  |  |
| --- | --- | --- | --- | --- |
| **Gene** | **Experiment** | **Number of upstream peaks** | **Enrichment** | **FDR corrected p** |
| zag-1 | ZAG-1\_Larvae-L2-stage | 26 | 2.75 | 0.00003 |
| ces-1 | CES-1\_Embryos | 33 | 2.13 | 0.00013 |
| ces-1 | CES-1\_Larvae-L4-stage | 16 | 3.69 | 0.00022 |
| alr-1 | ALR-1\_Larvae-L2-stage | 18 | 3.31 | 0.00023 |
| sem-4 | SEM-4\_Larvae-L2-stage | 26 | 2.30 | 0.00070 |
| ces-1 | CES-1\_Fed-L1-stage-larvae | 16 | 3.29 | 0.00086 |
| mab-5 | MAB-5\_Larvae-L2-stage | 15 | 3.02 | 0.00380 |
| aha-1 | AHA-1\_Larvae-L4-stage | 11 | 3.70 | 0.00660 |
| mef-2 | MEF-2\_Fed-L1-stage-larvae | 9 | 3.72 | 0.02300 |
| nhr-129 | NHR-129\_Larvae-L2-stage | 28 | 1.77 | 0.02400 |
| ztf-4 | ZTF-4\_Larvae-L2-stage | 6 | 4.99 | 0.04100 |
