# Supplementary material for: Overlapping cell population expression profiling and regulatory inference in C. elegans
Source: BMC Genomics. 2016 Feb 29;17:159. doi: 10.1186/s12864-016-2482-z (PMC4772325; doi:10.1186/s12864-016-2482-z)
Supplement: Additional file 13: — Web supplement. (DOC 21 kb) [file 12864_2016_2482_MOESM13_ESM.zip › sortWeb/clusters/hier.300.clusters/169.html]

Cluster 169 

## Cluster 169

### Expression

| cnd-1 rep. 1 | cnd-1 rep. 2 | cnd-1 rep. 3 | pha-4 rep. 1 | pha-4 rep. 2 | pha-4 rep. 3 | ceh-27 | ceh-36 | ceh-6 | F21D5.9 | mir-57 | mls-2 | pal-1 | pros-1 | ttx-3 | unc-130 | hlh-16 | irx-1 | ceh-6 (+) hlh-16 (+) | ceh-6 (+) hlh-16 (-) | ceh-6 (-) hlh-16 (+) | cnd-1 singlets | pha-4 singlets | 0 | 60 | 120 | 150 | 180 | 240 | 330 | 390 | 420 | 480 | 540 | 570 | 600 | 630 | 660 | NAME | Functional description |
| --- | --- | --- | --- | --- | --- | --- | --- | --- | --- | --- | --- | --- | --- | --- | --- | --- | --- | --- | --- | --- | --- | --- | --- | --- | --- | --- | --- | --- | --- | --- | --- | --- | --- | --- | --- | --- | --- | --- | --- |
|  |  |  |  |  |  |  |  |  |  |  |  |  |  |  |  |  |  |  |  |  |  |  |  |  |  |  |  |  |  |  |  |  |  |  |  |  |  | Y49F6C.7 |  |
|  |  |  |  |  |  |  |  |  |  |  |  |  |  |  |  |  |  |  |  |  |  |  |  |  |  |  |  |  |  |  |  |  |  |  |  |  |  | Y7A5A.10 |  |
|  |  |  |  |  |  |  |  |  |  |  |  |  |  |  |  |  |  |  |  |  |  |  |  |  |  |  |  |  |  |  |  |  |  |  |  |  |  | *nuo-2* | NADH Ubiquinone Oxidoreductase |
|  |  |  |  |  |  |  |  |  |  |  |  |  |  |  |  |  |  |  |  |  |  |  |  |  |  |  |  |  |  |  |  |  |  |  |  |  |  | C25H3.9 |  |
|  |  |  |  |  |  |  |  |  |  |  |  |  |  |  |  |  |  |  |  |  |  |  |  |  |  |  |  |  |  |  |  |  |  |  |  |  |  | *ant-1.1* | Adenine Nucleotide Translocator |
|  |  |  |  |  |  |  |  |  |  |  |  |  |  |  |  |  |  |  |  |  |  |  |  |  |  |  |  |  |  |  |  |  |  |  |  |  |  | C34B2.8 |  |
|  |  |  |  |  |  |  |  |  |  |  |  |  |  |  |  |  |  |  |  |  |  |  |  |  |  |  |  |  |  |  |  |  |  |  |  |  |  | *nuo-3* | NADH Ubiquinone Oxidoreductase |
|  |  |  |  |  |  |  |  |  |  |  |  |  |  |  |  |  |  |  |  |  |  |  |  |  |  |  |  |  |  |  |  |  |  |  |  |  |  | F13G3.10 |  |
|  |  |  |  |  |  |  |  |  |  |  |  |  |  |  |  |  |  |  |  |  |  |  |  |  |  |  |  |  |  |  |  |  |  |  |  |  |  | *mif-1* | MIF (Macrophage migration Inhibitory Factor) related |
|  |  |  |  |  |  |  |  |  |  |  |  |  |  |  |  |  |  |  |  |  |  |  |  |  |  |  |  |  |  |  |  |  |  |  |  |  |  | Y51H1A.3 |  |
|  |  |  |  |  |  |  |  |  |  |  |  |  |  |  |  |  |  |  |  |  |  |  |  |  |  |  |  |  |  |  |  |  |  |  |  |  |  | Y67D2.3 |  |
|  |  |  |  |  |  |  |  |  |  |  |  |  |  |  |  |  |  |  |  |  |  |  |  |  |  |  |  |  |  |  |  |  |  |  |  |  |  | *tag-174* | Temporarily Assigned Gene name |
|  |  |  |  |  |  |  |  |  |  |  |  |  |  |  |  |  |  |  |  |  |  |  |  |  |  |  |  |  |  |  |  |  |  |  |  |  |  | Y94H6A.8 |  |
|  |  |  |  |  |  |  |  |  |  |  |  |  |  |  |  |  |  |  |  |  |  |  |  |  |  |  |  |  |  |  |  |  |  |  |  |  |  | ZK809.3 |  |
|  |  |  |  |  |  |  |  |  |  |  |  |  |  |  |  |  |  |  |  |  |  |  |  |  |  |  |  |  |  |  |  |  |  |  |  |  |  | F43C11.6 |  |
|  |  |  |  |  |  |  |  |  |  |  |  |  |  |  |  |  |  |  |  |  |  |  |  |  |  |  |  |  |  |  |  |  |  |  |  |  |  | *his-68* | HIStone |
|  |  |  |  |  |  |  |  |  |  |  |  |  |  |  |  |  |  |  |  |  |  |  |  |  |  |  |  |  |  |  |  |  |  |  |  |  |  | *dad-1* | DAD (Defender against Apoptotic Death) homolog |
|  |  |  |  |  |  |  |  |  |  |  |  |  |  |  |  |  |  |  |  |  |  |  |  |  |  |  |  |  |  |  |  |  |  |  |  |  |  | *nxt-1* | NTF2-related eXporT protein |
|  |  |  |  |  |  |  |  |  |  |  |  |  |  |  |  |  |  |  |  |  |  |  |  |  |  |  |  |  |  |  |  |  |  |  |  |  |  | *nmat-2* | Nicotinamide Mononucleotide AdenylylTransferase homolog |
|  |  |  |  |  |  |  |  |  |  |  |  |  |  |  |  |  |  |  |  |  |  |  |  |  |  |  |  |  |  |  |  |  |  |  |  |  |  | *cox-17* | Cytochrome OXidase assembly protein |
|  |  |  |  |  |  |  |  |  |  |  |  |  |  |  |  |  |  |  |  |  |  |  |  |  |  |  |  |  |  |  |  |  |  |  |  |  |  | R07E5.13 |  |
|  |  |  |  |  |  |  |  |  |  |  |  |  |  |  |  |  |  |  |  |  |  |  |  |  |  |  |  |  |  |  |  |  |  |  |  |  |  | R53.4 |  |
|  |  |  |  |  |  |  |  |  |  |  |  |  |  |  |  |  |  |  |  |  |  |  |  |  |  |  |  |  |  |  |  |  |  |  |  |  |  | C47G2.3 |  |
|  |  |  |  |  |  |  |  |  |  |  |  |  |  |  |  |  |  |  |  |  |  |  |  |  |  |  |  |  |  |  |  |  |  |  |  |  |  | W02B12.15 |  |
|  |  |  |  |  |  |  |  |  |  |  |  |  |  |  |  |  |  |  |  |  |  |  |  |  |  |  |  |  |  |  |  |  |  |  |  |  |  | *hpo-19* | Hypersensitive to POre-forming toxin |
|  |  |  |  |  |  |  |  |  |  |  |  |  |  |  |  |  |  |  |  |  |  |  |  |  |  |  |  |  |  |  |  |  |  |  |  |  |  | *mrps-16* | Mitochondrial Ribosomal Protein, Small |
|  |  |  |  |  |  |  |  |  |  |  |  |  |  |  |  |  |  |  |  |  |  |  |  |  |  |  |  |  |  |  |  |  |  |  |  |  |  | *mrpl-18* | Mitochondrial Ribosomal Protein, Large |
|  |  |  |  |  |  |  |  |  |  |  |  |  |  |  |  |  |  |  |  |  |  |  |  |  |  |  |  |  |  |  |  |  |  |  |  |  |  | *mrps-25* | Mitochondrial Ribosomal Protein, Small |
|  |  |  |  |  |  |  |  |  |  |  |  |  |  |  |  |  |  |  |  |  |  |  |  |  |  |  |  |  |  |  |  |  |  |  |  |  |  | Y57A10A.29 |  |
|  |  |  |  |  |  |  |  |  |  |  |  |  |  |  |  |  |  |  |  |  |  |  |  |  |  |  |  |  |  |  |  |  |  |  |  |  |  | *mrpl-55* | Mitochondrial Ribosomal Protein, Large |
|  |  |  |  |  |  |  |  |  |  |  |  |  |  |  |  |  |  |  |  |  |  |  |  |  |  |  |  |  |  |  |  |  |  |  |  |  |  | *mrpl-20* | Mitochondrial Ribosomal Protein, Large |
|  |  |  |  |  |  |  |  |  |  |  |  |  |  |  |  |  |  |  |  |  |  |  |  |  |  |  |  |  |  |  |  |  |  |  |  |  |  | *tin-9.2* | Transport to INner mitochondrial membrane (yeast TIM) |
|  |  |  |  |  |  |  |  |  |  |  |  |  |  |  |  |  |  |  |  |  |  |  |  |  |  |  |  |  |  |  |  |  |  |  |  |  |  | Y116A8C.30 |  |
|  |  |  |  |  |  |  |  |  |  |  |  |  |  |  |  |  |  |  |  |  |  |  |  |  |  |  |  |  |  |  |  |  |  |  |  |  |  | *lpd-5* | LiPid Depleted |
|  |  |  |  |  |  |  |  |  |  |  |  |  |  |  |  |  |  |  |  |  |  |  |  |  |  |  |  |  |  |  |  |  |  |  |  |  |  | C16A3.5 |  |
|  |  |  |  |  |  |  |  |  |  |  |  |  |  |  |  |  |  |  |  |  |  |  |  |  |  |  |  |  |  |  |  |  |  |  |  |  |  | F54A3.5 |  |
|  |  |  |  |  |  |  |  |  |  |  |  |  |  |  |  |  |  |  |  |  |  |  |  |  |  |  |  |  |  |  |  |  |  |  |  |  |  | Y82E9BR.22 |  |
|  |  |  |  |  |  |  |  |  |  |  |  |  |  |  |  |  |  |  |  |  |  |  |  |  |  |  |  |  |  |  |  |  |  |  |  |  |  | F57B10.14 |  |
|  |  |  |  |  |  |  |  |  |  |  |  |  |  |  |  |  |  |  |  |  |  |  |  |  |  |  |  |  |  |  |  |  |  |  |  |  |  | T14B4.2 |  |
|  |  |  |  |  |  |  |  |  |  |  |  |  |  |  |  |  |  |  |  |  |  |  |  |  |  |  |  |  |  |  |  |  |  |  |  |  |  | F59A2.5 |  |
|  |  |  |  |  |  |  |  |  |  |  |  |  |  |  |  |  |  |  |  |  |  |  |  |  |  |  |  |  |  |  |  |  |  |  |  |  |  | *moma-1* | Mitochondrial Outer Membrane Abnormal |
|  |  |  |  |  |  |  |  |  |  |  |  |  |  |  |  |  |  |  |  |  |  |  |  |  |  |  |  |  |  |  |  |  |  |  |  |  |  | *sec-22* | yeast SEC homolog |
|  |  |  |  |  |  |  |  |  |  |  |  |  |  |  |  |  |  |  |  |  |  |  |  |  |  |  |  |  |  |  |  |  |  |  |  |  |  | *taf-9* | TAF (TBP-associated transcription factor) family |
|  |  |  |  |  |  |  |  |  |  |  |  |  |  |  |  |  |  |  |  |  |  |  |  |  |  |  |  |  |  |  |  |  |  |  |  |  |  | *mrps-21* | Mitochondrial Ribosomal Protein, Small |
|  |  |  |  |  |  |  |  |  |  |  |  |  |  |  |  |  |  |  |  |  |  |  |  |  |  |  |  |  |  |  |  |  |  |  |  |  |  | *ufc-1* | UFM Conjugating Enzyme |
|  |  |  |  |  |  |  |  |  |  |  |  |  |  |  |  |  |  |  |  |  |  |  |  |  |  |  |  |  |  |  |  |  |  |  |  |  |  | T03F1.12 |  |
|  |  |  |  |  |  |  |  |  |  |  |  |  |  |  |  |  |  |  |  |  |  |  |  |  |  |  |  |  |  |  |  |  |  |  |  |  |  | M04F3.4 |  |
|  |  |  |  |  |  |  |  |  |  |  |  |  |  |  |  |  |  |  |  |  |  |  |  |  |  |  |  |  |  |  |  |  |  |  |  |  |  | *blos-4* | BLOC (Biogenesis of Lysosome-related Organelles Complex) Subunit homolog |
|  |  |  |  |  |  |  |  |  |  |  |  |  |  |  |  |  |  |  |  |  |  |  |  |  |  |  |  |  |  |  |  |  |  |  |  |  |  | Y56A3A.18 |  |
|  |  |  |  |  |  |  |  |  |  |  |  |  |  |  |  |  |  |  |  |  |  |  |  |  |  |  |  |  |  |  |  |  |  |  |  |  |  | K10D2.7 |  |
|  |  |  |  |  |  |  |  |  |  |  |  |  |  |  |  |  |  |  |  |  |  |  |  |  |  |  |  |  |  |  |  |  |  |  |  |  |  | *taf-10* | TAF (TBP-associated transcription factor) family |
|  |  |  |  |  |  |  |  |  |  |  |  |  |  |  |  |  |  |  |  |  |  |  |  |  |  |  |  |  |  |  |  |  |  |  |  |  |  | *prdx-3* | PeRoxireDoXin |
|  |  |  |  |  |  |  |  |  |  |  |  |  |  |  |  |  |  |  |  |  |  |  |  |  |  |  |  |  |  |  |  |  |  |  |  |  |  | *mrpl-14* | Mitochondrial Ribosomal Protein, Large |
|  |  |  |  |  |  |  |  |  |  |  |  |  |  |  |  |  |  |  |  |  |  |  |  |  |  |  |  |  |  |  |  |  |  |  |  |  |  | T09A5.15 |  |
|  |  |  |  |  |  |  |  |  |  |  |  |  |  |  |  |  |  |  |  |  |  |  |  |  |  |  |  |  |  |  |  |  |  |  |  |  |  | C18E9.10 |  |
|  |  |  |  |  |  |  |  |  |  |  |  |  |  |  |  |  |  |  |  |  |  |  |  |  |  |  |  |  |  |  |  |  |  |  |  |  |  | C14B9.10 |  |
|  |  |  |  |  |  |  |  |  |  |  |  |  |  |  |  |  |  |  |  |  |  |  |  |  |  |  |  |  |  |  |  |  |  |  |  |  |  | *mai-2* | Mitochondrial ATPase Inhibitor family |
|  |  |  |  |  |  |  |  |  |  |  |  |  |  |  |  |  |  |  |  |  |  |  |  |  |  |  |  |  |  |  |  |  |  |  |  |  |  | Y48A5A.3 |  |
|  |  |  |  |  |  |  |  |  |  |  |  |  |  |  |  |  |  |  |  |  |  |  |  |  |  |  |  |  |  |  |  |  |  |  |  |  |  | F45H10.3 |  |
|  |  |  |  |  |  |  |  |  |  |  |  |  |  |  |  |  |  |  |  |  |  |  |  |  |  |  |  |  |  |  |  |  |  |  |  |  |  | Y37D8A.25 |  |
|  |  |  |  |  |  |  |  |  |  |  |  |  |  |  |  |  |  |  |  |  |  |  |  |  |  |  |  |  |  |  |  |  |  |  |  |  |  | Y63D3A.7 |  |
|  |  |  |  |  |  |  |  |  |  |  |  |  |  |  |  |  |  |  |  |  |  |  |  |  |  |  |  |  |  |  |  |  |  |  |  |  |  | F45H10.2 |  |
|  |  |  |  |  |  |  |  |  |  |  |  |  |  |  |  |  |  |  |  |  |  |  |  |  |  |  |  |  |  |  |  |  |  |  |  |  |  | F26E4.6 |  |
|  |  |  |  |  |  |  |  |  |  |  |  |  |  |  |  |  |  |  |  |  |  |  |  |  |  |  |  |  |  |  |  |  |  |  |  |  |  | *atp-4* | ATP synthase subunit |
|  |  |  |  |  |  |  |  |  |  |  |  |  |  |  |  |  |  |  |  |  |  |  |  |  |  |  |  |  |  |  |  |  |  |  |  |  |  | *sod-1* | SOD (superoxide dismutase) |
|  |  |  |  |  |  |  |  |  |  |  |  |  |  |  |  |  |  |  |  |  |  |  |  |  |  |  |  |  |  |  |  |  |  |  |  |  |  | C48B6.10 |  |
|  |  |  |  |  |  |  |  |  |  |  |  |  |  |  |  |  |  |  |  |  |  |  |  |  |  |  |  |  |  |  |  |  |  |  |  |  |  | F29B9.11 |  |
|  |  |  |  |  |  |  |  |  |  |  |  |  |  |  |  |  |  |  |  |  |  |  |  |  |  |  |  |  |  |  |  |  |  |  |  |  |  | T07A5.5 |  |
|  |  |  |  |  |  |  |  |  |  |  |  |  |  |  |  |  |  |  |  |  |  |  |  |  |  |  |  |  |  |  |  |  |  |  |  |  |  | *ufm-1* | Ubiquitin-Fold Modifier homolog |
|  |  |  |  |  |  |  |  |  |  |  |  |  |  |  |  |  |  |  |  |  |  |  |  |  |  |  |  |  |  |  |  |  |  |  |  |  |  | ZK512.4 |  |
|  |  |  |  |  |  |  |  |  |  |  |  |  |  |  |  |  |  |  |  |  |  |  |  |  |  |  |  |  |  |  |  |  |  |  |  |  |  | *ddp-1* | human DDP related |
|  |  |  |  |  |  |  |  |  |  |  |  |  |  |  |  |  |  |  |  |  |  |  |  |  |  |  |  |  |  |  |  |  |  |  |  |  |  | *tomm-7* | Translocase of Outer Mitochondrial Membrane |
|  |  |  |  |  |  |  |  |  |  |  |  |  |  |  |  |  |  |  |  |  |  |  |  |  |  |  |  |  |  |  |  |  |  |  |  |  |  | F37F2.2 |  |
|  |  |  |  |  |  |  |  |  |  |  |  |  |  |  |  |  |  |  |  |  |  |  |  |  |  |  |  |  |  |  |  |  |  |  |  |  |  | C34B2.10 |  |
|  |  |  |  |  |  |  |  |  |  |  |  |  |  |  |  |  |  |  |  |  |  |  |  |  |  |  |  |  |  |  |  |  |  |  |  |  |  | Y97E10AR.7 |  |
|  |  |  |  |  |  |  |  |  |  |  |  |  |  |  |  |  |  |  |  |  |  |  |  |  |  |  |  |  |  |  |  |  |  |  |  |  |  | Y38E10A.24 |  |
|  |  |  |  |  |  |  |  |  |  |  |  |  |  |  |  |  |  |  |  |  |  |  |  |  |  |  |  |  |  |  |  |  |  |  |  |  |  | F29C4.2 |  |
|  |  |  |  |  |  |  |  |  |  |  |  |  |  |  |  |  |  |  |  |  |  |  |  |  |  |  |  |  |  |  |  |  |  |  |  |  |  | Y11D7A.10 |  |
|  |  |  |  |  |  |  |  |  |  |  |  |  |  |  |  |  |  |  |  |  |  |  |  |  |  |  |  |  |  |  |  |  |  |  |  |  |  | Y67H2A.5 |  |
|  |  |  |  |  |  |  |  |  |  |  |  |  |  |  |  |  |  |  |  |  |  |  |  |  |  |  |  |  |  |  |  |  |  |  |  |  |  | C53H9.3 |  |
|  |  |  |  |  |  |  |  |  |  |  |  |  |  |  |  |  |  |  |  |  |  |  |  |  |  |  |  |  |  |  |  |  |  |  |  |  |  | *zhp-3* | Zip (yeast meiotic zipper) Homologous Protein |
|  |  |  |  |  |  |  |  |  |  |  |  |  |  |  |  |  |  |  |  |  |  |  |  |  |  |  |  |  |  |  |  |  |  |  |  |  |  | T02H6.11 |  |
|  |  |  |  |  |  |  |  |  |  |  |  |  |  |  |  |  |  |  |  |  |  |  |  |  |  |  |  |  |  |  |  |  |  |  |  |  |  | Y57E12AM.1 |  |
|  |  |  |  |  |  |  |  |  |  |  |  |  |  |  |  |  |  |  |  |  |  |  |  |  |  |  |  |  |  |  |  |  |  |  |  |  |  | F23H11.5 |  |
|  |  |  |  |  |  |  |  |  |  |  |  |  |  |  |  |  |  |  |  |  |  |  |  |  |  |  |  |  |  |  |  |  |  |  |  |  |  | Y57E12AL.6 |  |
|  |  |  |  |  |  |  |  |  |  |  |  |  |  |  |  |  |  |  |  |  |  |  |  |  |  |  |  |  |  |  |  |  |  |  |  |  |  | Y71H2AM.4 |  |
|  |  |  |  |  |  |  |  |  |  |  |  |  |  |  |  |  |  |  |  |  |  |  |  |  |  |  |  |  |  |  |  |  |  |  |  |  |  | R04F11.2 |  |
|  |  |  |  |  |  |  |  |  |  |  |  |  |  |  |  |  |  |  |  |  |  |  |  |  |  |  |  |  |  |  |  |  |  |  |  |  |  | T09A5.7 |  |
|  |  |  |  |  |  |  |  |  |  |  |  |  |  |  |  |  |  |  |  |  |  |  |  |  |  |  |  |  |  |  |  |  |  |  |  |  |  | *lgg-3* | LC3, GABARAP and GATE-16 family |
|  |  |  |  |  |  |  |  |  |  |  |  |  |  |  |  |  |  |  |  |  |  |  |  |  |  |  |  |  |  |  |  |  |  |  |  |  |  | Y105E8A.11 |  |
|  |  |  |  |  |  |  |  |  |  |  |  |  |  |  |  |  |  |  |  |  |  |  |  |  |  |  |  |  |  |  |  |  |  |  |  |  |  | Y55B1AL.2 |  |
|  |  |  |  |  |  |  |  |  |  |  |  |  |  |  |  |  |  |  |  |  |  |  |  |  |  |  |  |  |  |  |  |  |  |  |  |  |  | ZK1098.11 |  |
|  |  |  |  |  |  |  |  |  |  |  |  |  |  |  |  |  |  |  |  |  |  |  |  |  |  |  |  |  |  |  |  |  |  |  |  |  |  | *mif-2* | MIF (Macrophage migration Inhibitory Factor) related |
|  |  |  |  |  |  |  |  |  |  |  |  |  |  |  |  |  |  |  |  |  |  |  |  |  |  |  |  |  |  |  |  |  |  |  |  |  |  | *tos-1* | Target Of Splicing |
|  |  |  |  |  |  |  |  |  |  |  |  |  |  |  |  |  |  |  |  |  |  |  |  |  |  |  |  |  |  |  |  |  |  |  |  |  |  | *asg-2* | ATP Synthase G homolog |
|  |  |  |  |  |  |  |  |  |  |  |  |  |  |  |  |  |  |  |  |  |  |  |  |  |  |  |  |  |  |  |  |  |  |  |  |  |  | *nduf-5* | NADH Ubiquinone oxidoreductase Fe-S protein |
|  |  |  |  |  |  |  |  |  |  |  |  |  |  |  |  |  |  |  |  |  |  |  |  |  |  |  |  |  |  |  |  |  |  |  |  |  |  | *lpd-9* | LiPid Depleted |
|  |  |  |  |  |  |  |  |  |  |  |  |  |  |  |  |  |  |  |  |  |  |  |  |  |  |  |  |  |  |  |  |  |  |  |  |  |  | C42C1.12 |  |
|  |  |  |  |  |  |  |  |  |  |  |  |  |  |  |  |  |  |  |  |  |  |  |  |  |  |  |  |  |  |  |  |  |  |  |  |  |  | Y97E10AL.3 |  |
|  |  |  |  |  |  |  |  |  |  |  |  |  |  |  |  |  |  |  |  |  |  |  |  |  |  |  |  |  |  |  |  |  |  |  |  |  |  | F25G6.8 |  |
|  |  |  |  |  |  |  |  |  |  |  |  |  |  |  |  |  |  |  |  |  |  |  |  |  |  |  |  |  |  |  |  |  |  |  |  |  |  | F54F2.7 |  |
|  |  |  |  |  |  |  |  |  |  |  |  |  |  |  |  |  |  |  |  |  |  |  |  |  |  |  |  |  |  |  |  |  |  |  |  |  |  | F44E2.9 |  |
|  |  |  |  |  |  |  |  |  |  |  |  |  |  |  |  |  |  |  |  |  |  |  |  |  |  |  |  |  |  |  |  |  |  |  |  |  |  | *cope-1* | COat Protein complex 1, Epsilon subunit |
|  |  |  |  |  |  |  |  |  |  |  |  |  |  |  |  |  |  |  |  |  |  |  |  |  |  |  |  |  |  |  |  |  |  |  |  |  |  | *lbp-4* | Lipid Binding Protein |
|  |  |  |  |  |  |  |  |  |  |  |  |  |  |  |  |  |  |  |  |  |  |  |  |  |  |  |  |  |  |  |  |  |  |  |  |  |  | Y54F10AM.5 |  |
|  |  |  |  |  |  |  |  |  |  |  |  |  |  |  |  |  |  |  |  |  |  |  |  |  |  |  |  |  |  |  |  |  |  |  |  |  |  | *mrpl-35* | Mitochondrial Ribosomal Protein, Large |
|  |  |  |  |  |  |  |  |  |  |  |  |  |  |  |  |  |  |  |  |  |  |  |  |  |  |  |  |  |  |  |  |  |  |  |  |  |  | F37C12.3 |  |
|  |  |  |  |  |  |  |  |  |  |  |  |  |  |  |  |  |  |  |  |  |  |  |  |  |  |  |  |  |  |  |  |  |  |  |  |  |  | *sod-2* | SOD (superoxide dismutase) |
|  |  |  |  |  |  |  |  |  |  |  |  |  |  |  |  |  |  |  |  |  |  |  |  |  |  |  |  |  |  |  |  |  |  |  |  |  |  | *mrps-10* | Mitochondrial Ribosomal Protein, Small |
|  |  |  |  |  |  |  |  |  |  |  |  |  |  |  |  |  |  |  |  |  |  |  |  |  |  |  |  |  |  |  |  |  |  |  |  |  |  | *mrpl-51* | Mitochondrial Ribosomal Protein, Large |
|  |  |  |  |  |  |  |  |  |  |  |  |  |  |  |  |  |  |  |  |  |  |  |  |  |  |  |  |  |  |  |  |  |  |  |  |  |  | *mrpl-34* | Mitochondrial Ribosomal Protein, Large |
|  |  |  |  |  |  |  |  |  |  |  |  |  |  |  |  |  |  |  |  |  |  |  |  |  |  |  |  |  |  |  |  |  |  |  |  |  |  | CD4.3 |  |
|  |  |  |  |  |  |  |  |  |  |  |  |  |  |  |  |  |  |  |  |  |  |  |  |  |  |  |  |  |  |  |  |  |  |  |  |  |  | K08E4.6 |  |
|  |  |  |  |  |  |  |  |  |  |  |  |  |  |  |  |  |  |  |  |  |  |  |  |  |  |  |  |  |  |  |  |  |  |  |  |  |  | *mtss-1* | MiTochondrial Single Stranded DNA binding protein |
|  |  |  |  |  |  |  |  |  |  |  |  |  |  |  |  |  |  |  |  |  |  |  |  |  |  |  |  |  |  |  |  |  |  |  |  |  |  | *mrps-23* | Mitochondrial Ribosomal Protein, Small |
|  |  |  |  |  |  |  |  |  |  |  |  |  |  |  |  |  |  |  |  |  |  |  |  |  |  |  |  |  |  |  |  |  |  |  |  |  |  | F56D12.8 |  |
|  |  |  |  |  |  |  |  |  |  |  |  |  |  |  |  |  |  |  |  |  |  |  |  |  |  |  |  |  |  |  |  |  |  |  |  |  |  | *fbxc-49* | F-box C protein |
|  |  |  |  |  |  |  |  |  |  |  |  |  |  |  |  |  |  |  |  |  |  |  |  |  |  |  |  |  |  |  |  |  |  |  |  |  |  | F45D11.14 |  |
|  |  |  |  |  |  |  |  |  |  |  |  |  |  |  |  |  |  |  |  |  |  |  |  |  |  |  |  |  |  |  |  |  |  |  |  |  |  | F48C1.9 |  |
|  |  |  |  |  |  |  |  |  |  |  |  |  |  |  |  |  |  |  |  |  |  |  |  |  |  |  |  |  |  |  |  |  |  |  |  |  |  | F54D12.16 |  |
|  |  |  |  |  |  |  |  |  |  |  |  |  |  |  |  |  |  |  |  |  |  |  |  |  |  |  |  |  |  |  |  |  |  |  |  |  |  | *fbxa-202* | F-box A protein |
|  |  |  |  |  |  |  |  |  |  |  |  |  |  |  |  |  |  |  |  |  |  |  |  |  |  |  |  |  |  |  |  |  |  |  |  |  |  | *tin-13* | Transport to INner mitochondrial membrane (yeast TIM) |
|  |  |  |  |  |  |  |  |  |  |  |  |  |  |  |  |  |  |  |  |  |  |  |  |  |  |  |  |  |  |  |  |  |  |  |  |  |  | *mrps-17* | Mitochondrial Ribosomal Protein, Small |
|  |  |  |  |  |  |  |  |  |  |  |  |  |  |  |  |  |  |  |  |  |  |  |  |  |  |  |  |  |  |  |  |  |  |  |  |  |  | *clpp-1* | CLP Protease family |
|  |  |  |  |  |  |  |  |  |  |  |  |  |  |  |  |  |  |  |  |  |  |  |  |  |  |  |  |  |  |  |  |  |  |  |  |  |  | ZK1236.5 |  |
|  |  |  |  |  |  |  |  |  |  |  |  |  |  |  |  |  |  |  |  |  |  |  |  |  |  |  |  |  |  |  |  |  |  |  |  |  |  | *tomm-20* | Translocase of Outer Mitochondrial Membrane |

### Phenotypes enriched

|  |  |  |  |
| --- | --- | --- | --- |
| **Group name** | **Number in cluster** | **Enrichment** | **FDR corrected p** |
| slow growth (RNAi) | 44 | 2.64 | 1.48e-06 |
| general pace of development variant (RNAi) | 9 | 13.50 | 5.44e-05 |
| general pace of development defective early emb (RNAi) | 7 | 13.83 | 1.27e-03 |
| embryonic developmental delay early emb (RNAi) | 7 | 12.63 | 2.19e-03 |
| embryo delayed at pronuclear contact early emb (RNAi) | 7 | 12.63 | 2.19e-03 |
| paraquat resistant (RNAi) | 7 | 10.25 | 7.61e-03 |
| extended life span (RNAi) | 13 | 4.22 | 1.60e-02 |
| oxidative stress response variant (RNAi) | 7 | 8.71 | 1.90e-02 |
| paraquat response variant (RNAi) | 7 | 8.71 | 1.90e-02 |
| pesticide response variant (RNAi) | 7 | 8.63 | 2.01e-02 |
| organism oxidative stress response variant (RNAi) | 7 | 7.51 | 4.29e-02 |
| postembryonic development variant (RNAi) | 33 | 2.03 | 4.44e-02 |

### Anatomy terms enriched

none found

### GO terms enriched

|  |  |  |
| --- | --- | --- |
| **GO term** | **Number of genes** | **FDR-corrected p-value** |
| mitochondrion | 28 | 2.5e-22 |
| positive regulation of growth rate | 38 | 3.0e-09 |
| regulation of growth | 38 | 6.3e-07 |
| positive regulation of biological process | 40 | 1.8e-06 |
| ribosome | 11 | 7.4e-06 |
| embryo development ending in birth or egg hatching | 47 | 2.6e-05 |
| mitochondrial inner membrane | 8 | 4.8e-05 |
| intracellular | 43 | 1.4e-04 |
| nematode larval development | 37 | 2.7e-04 |
| post-embryonic development | 37 | 3.2e-04 |
| organelle envelope | 9 | 3.3e-04 |
| structural constituent of ribosome | 7 | 1.0e-03 |
| macromolecular complex | 22 | 2.1e-03 |
| intracellular organelle | 37 | 3.7e-03 |
| anatomical structure development | 50 | 4.6e-03 |
| mitochondrial membrane | 4 | 5.1e-03 |
| receptor-mediated endocytosis | 17 | 1.1e-02 |
| cell | 43 | 1.2e-02 |
| protein localization to membrane | 3 | 2.2e-02 |
| organelle outer membrane | 3 | 4.2e-02 |

### Expression clusters enriched

|  |  |  |  |
| --- | --- | --- | --- |
| **Group name** | **Number in cluster** | **Enrichment** | **FDR corrected p** |
| Genes in the top 10% of expression level across the triplicate L3 samples. To generate the top10 and bottom10 gene sets, authors ranked all genes by mean expression array signal intensity across the three replicates, then took the top and bottom deciles (1,841 genes each) to represent genes with high and low expression. | 73 | 4.94 | 2.34e-32 |
| Caenorhabditis elegans Genes with expression levels changed significantly after treatment of Bacillus thurigiensis DB27. | 98 | 2.55 | 1.42e-24 |
| Caenorhabditis elegans Genes with expression levels changed significantly after treatment of Xenorhabdus nematophila. | 108 | 1.93 | 9.45e-19 |
| Maternal degradation (MD) subclasses are based on the earliest significant decrease (abbreviated pd for primary decrease). [cgc5767]:expression\_class\_MD\_pd(23\_min) | 31 | 7.09 | 4.08e-15 |
| The cluster contains genes that are significantly enriched in L1 muscle. | 46 | 3.96 | 5.75e-14 |
| Maternal degradation class (MD): genes that are the subset of maternal genes that decrease without first increasing in abundance. | 50 | 3.57 | 1.06e-13 |
| Differentially expressed genes during worm lifespan. Medoid 7 Fig.4. | 23 | 8.62 | 2.04e-12 |
| Maternal degradation-embryonic class (MDE): genes that are the subset of maternal degradation genes that significantly increase in at least two of the eight total paired timepoint tests in the induction-following-degradation time domain. | 30 | 5.84 | 2.51e-12 |
| Genes expressed in embryonic motor neurons (identified by unc-4::GFP expressing cells). | 92 | 1.89 | 3.89e-12 |
| Maternal class (M): genes that are called present in at least one of the three PC6 replicates. | 89 | 1.90 | 1.90e-11 |
| Genes with no change in hcf-1(-), no change in sir-2.1(O/E) and downregulated in daf-2(-). | 30 | 5.16 | 5.96e-11 |
| Genes up or down regulated by 10e-09M of cholesterol . The normalized values used were G/R ratio > 2.6 for up-regulation and G/R ratio < 0.38 for down-regulation, which corresponds to 1.39 and -1.39 log(base2) G/R ratio, respectively. | 46 | 3.20 | 1.41e-10 |
| Genes enriched in intestine. | 47 | 2.92 | 1.73e-09 |
| C-lineage related expression profile. WBPaper00025032:cluster\_152 | 6 | 67.89 | 7.46e-08 |
| Genes enriched in muscle. | 20 | 6.01 | 7.76e-08 |
| Genes significantly enriched (> 2x, FDR < 5%) in a particular cell-type versus a reference sample of all cells at the same stage. WBPaper00037950:hypodermis\_larva\_enriched | 34 | 3.38 | 9.80e-08 |
| Genes up or down regulated by 10e-05M of progesterone. The normalized values used were G/R ratio > 2.6 for up-regulation and G/R ratio < 0.38 for down-regulation, which corresponds to 1.39 and -1.39 log(base2) G/R ratio, respectively. | 48 | 2.49 | 2.16e-07 |
| Genes with expression level affected by biotin deficiency. Genes were ranked by log2 (rank metric score) of their mean (n = 3 for each condition) differences between biotin sufficiency and deficiency. Statistical analysis included the t test comparing condition control to condition-deficient, false discovery rate calculation to control for multiple hypothesis testing and calculation of fold change (expressed as log2) value between the two conditions. Authors used these data to produce preordered ranked gene lists, which were then tested for enrichment by Gene Set Enrichment Analysis (GSEA v. 2.0, Broad Institute, Cambridge, Mass., USA). Genes included in this list were those belonging to the leading edge subset of GSEA. Also included are genes with expression level changed 2-fold or over with p <= 0.05. | 11 | 13.69 | 2.90e-07 |
| Maternal-embryonic class (ME): genes that are in the intersection of the maternal and embryonic classes. | 49 | 2.30 | 1.70e-06 |
| Embryonic (E) subclasses are based on the earliest significant increase(abbreviated pi for primary increase). [cgc5767]:expression\_class\_E\_pi(186\_min) | 14 | 6.31 | 2.23e-05 |
| Genes expressed in N2. | 100 | 1.41 | 2.39e-05 |
| Genes with decreased expression after 24 hours of infection by E.faecalis Fold changes shown are pathogen vs OP50. WBPaper00038438:E.faecalis\_24hr\_downregulated\_RNAseq | 22 | 3.75 | 3.59e-05 |
| C-lineage related expression profile. WBPaper00025032:cluster\_103 | 5 | 41.49 | 3.99e-05 |
| Developmentally modulated gene cluster. cgc4386\_cluster\_1\_4 | 9 | 10.98 | 5.72e-05 |
| Early embryonic development gene expression profile. [cgc5767]:cluster\_6 | 10 | 8.83 | 8.59e-05 |
| Caenorhabditis elegans Genes with expression levels changed significantly after treatment of Serratia marcescens. | 54 | 1.90 | 1.27e-04 |
| Genes for which heat shock F3 (fraction 3, containing heavy polysomes) versus control F3 is significantly increased. | 19 | 3.52 | 6.17e-04 |
| Expression Pattern Group G, enriched for genes involved in locomotion. | 30 | 2.50 | 6.96e-04 |
| Genes up or down regulated by 10e-05M of estrogen. The normalized values used were G/R ratio > 2.6 for up-regulation and G/R ratio < 0.38 for down-regulation, which corresponds to 1.39 and -1.39 log(base2) G/R ratio, respectively. | 27 | 2.55 | 1.62e-03 |
| Embryonic class (E): genes that significantly increase in abundance at some point during embryogenesis. | 51 | 1.78 | 1.95e-03 |
| Developmentally modulated gene cluster. cgc4386\_cluster\_2\_6 | 7 | 9.17 | 3.43e-03 |
| Genes that showed expression levels higher than the corresponding reference sample (L3/L4 all cell reference). | 72 | 1.49 | 3.88e-03 |
| Genes that showed expression levels higher than the corresponding reference sample (L2 all cell reference). WBPaper00037950:excretory-cell\_expressed | 67 | 1.53 | 4.70e-03 |
| Developmentally modulated gene cluster. cgc4386\_cluster\_3\_6 | 6 | 8.79 | 1.56e-02 |
| Genes significantly enriched (> 2x, FDR < 5%) in a particular cell-type versus a reference sample of all cells at the same stage. WBPaper00037950:PVD-OLL-neurons\_larva\_enriched | 19 | 2.69 | 1.81e-02 |
| C-lineage related expression profile. WBPaper00025032:cluster\_130 | 3 | 31.12 | 2.52e-02 |
| Developmentally modulated gene cluster. cgc4386\_cluster\_4\_5 | 6 | 7.78 | 2.88e-02 |
| Genes that showed expression levels higher than the corresponding reference sample (embryonic 0hr reference). WBPaper00037950:BAG-neuron\_expressed | 56 | 1.52 | 4.59e-02 |

### Motifs enriched

|  |  |  |  |  |  |
| --- | --- | --- | --- | --- | --- |
| **Motif** | **Logo** | **Possible orthologs** | **Number of motifs in cluster** | **Enrichment** | **FDR corrected p** |
| pTH10624 |  | mef-2 mel-28 let-381 Y61A9LA.9 Y116A8C.22 | 109 | 1.56 | 1.3e-10 |
| CG2052\_SANGER\_2.5\_FBgn0039905 |  | fkh-7 mel-28 lin-29 | 115 | 1.46 | 3.8e-10 |
| FOXC1\_1 |  | let-381 lin-31 | 109 | 1.53 | 6.6e-10 |
| Mw138 |  | ceh-48 dsc-1 | 36 | 3.83 | 1.6e-09 |
| V$TBP\_01 |  | tbp-1 | 95 | 1.70 | 2.2e-09 |
| Hoxc10\_2 |  | pal-1 php-3 lin-39 | 100 | 1.62 | 2.2e-09 |
| pTH9951 |  | mex-6 | 104 | 1.54 | 8.4e-09 |
| pTH9260 |  | mel-28 | 106 | 1.50 | 1.9e-08 |
| exd\_FlyReg\_FBgn0000611 |  | let-381 cfi-1 ceh-20 | 109 | 1.46 | 1.9e-08 |
| POU3F3\_3 |  | ceh-18 unc-86 | 97 | 1.60 | 2.5e-08 |
| ARI3A\_do |  | cfi-1 | 93 | 1.65 | 3.0e-08 |
| pTH9125 |  | egl-13 (-0.61) | 103 | 1.53 | 3.1e-08 |
| Abd-B\_FlyReg\_FBgn0000015 |  | ceh-24 ceh-13 php-3 D1005.3 T27F2.4 | 93 | 1.63 | 7.5e-08 |
| pTH9254 |  | mel-28 | 106 | 1.47 | 7.7e-08 |
| pTH9097 |  | Y116A8C.22 | 109 | 1.43 | 1.1e-07 |
| rn\_SOLEXA\_5\_FBgn0259172 |  | lin-29 | 108 | 1.44 | 1.4e-07 |
| FOXJ3\_1 |  | daf-16 (-0.54) fkh-7 fkh-10 fkh-8 let-381 lin-31 | 105 | 1.47 | 1.7e-07 |
| pTH9380 |  | mel-28 | 107 | 1.44 | 2.1e-07 |
| MA0497.1 |  | mef-2 | 101 | 1.50 | 2.7e-07 |
| pTH8997 |  | let-381 lin-31 hmg-12 lin-39 hmbx-1 Y116A8C.22 | 97 | 1.54 | 3.7e-07 |
| pTH8982 |  | ceh-48 | 31 | 3.49 | 4.3e-07 |
| MA0135.1 |  | lim-7 cfi-1 | 65 | 1.99 | 5.1e-07 |
| Zfp161\_2858 |  | pzf-1 | 49 | 2.38 | 6.7e-07 |
| MA0594.1 |  | hbl-1 (-0.61) php-3 lin-39 | 83 | 1.68 | 7.2e-07 |
| pTH9709 |  | die-1 (-0.54) | 95 | 1.53 | 1.2e-06 |
| FOXJ3\_si |  | daf-16 (-0.54) fkh-7 fkh-8 let-381 pha-4 lin-31 | 104 | 1.42 | 2.3e-06 |
| pTH9335 |  | mel-28 | 97 | 1.49 | 2.6e-06 |
| pTH10797 |  | K11D2.4 lin-29 | 107 | 1.38 | 3.2e-06 |
| pTH9082 |  | mab-23 | 100 | 1.43 | 6.5e-06 |
| pTH9958 |  | ztf-6 | 91 | 1.52 | 7.4e-06 |
| V$PBX1\_01 |  | lin-39 ceh-20 | 91 | 1.52 | 8.0e-06 |
| pTH9242 |  | mel-28 | 86 | 1.57 | 8.4e-06 |
| SPDEF\_3 |  | nhr-100 lin-1 lin-39 | 96 | 1.46 | 8.7e-06 |
| PO3F2\_si |  | ceh-18 dmd-3 | 91 | 1.51 | 9.1e-06 |
| SRP000712\_Sox2 |  | sox-4 ceh-18 ceh-6 | 99 | 1.43 | 1.1e-05 |
| pnr\_SANGER\_5\_FBgn0003117 |  | elt-1 | 97 | 1.45 | 1.3e-05 |
| pTH9137 |  | nhr-65 | 95 | 1.46 | 1.6e-05 |
| pTH10696 |  | Y44A6D.3 | 46 | 2.20 | 2.1e-05 |
| pTH5169 |  | cfi-1 | 95 | 1.45 | 2.4e-05 |
| BARX1\_1 |  | ceh-31 ceh-43 ceh-1 | 85 | 1.54 | 2.5e-05 |
| pTH8863 |  | hmg-12 | 45 | 2.21 | 2.9e-05 |
| pTH3796 |  | fkh-10 let-381 lin-31 | 91 | 1.47 | 4.6e-05 |
| CG8765\_SANGER\_5\_FBgn0036900 |  | H20J04.3 | 80 | 1.55 | 7.9e-05 |
| FOXO1\_si |  | daf-16 (-0.54) fkh-9 irx-1 | 97 | 1.40 | 8.0e-05 |
| Tbp\_pr781 |  | tbp-1 | 81 | 1.54 | 8.3e-05 |
| pTH7875 |  | mel-28 | 74 | 1.61 | 8.4e-05 |
| pTH9173 |  | efl-2 | 49 | 2.00 | 1.0e-04 |
| pTH3046 |  | Y116A8C.22 | 66 | 1.70 | 1.1e-04 |
| pTH9177 |  | F10B5.3 (-0.69) hsf-1 (-0.64) | 65 | 1.71 | 1.2e-04 |
| MA0537.1 |  | blmp-1 | 101 | 1.35 | 1.3e-04 |
| pTH3220 |  | ceh-9 Y5F2A.4 | 59 | 1.79 | 1.3e-04 |
| pTH1294 |  | mel-28 | 54 | 1.87 | 1.4e-04 |
| pTH8985 |  | athp-1 (-0.52) | 74 | 1.58 | 1.7e-04 |
| CG4328\_Cell\_FBgn0036274 |  | alr-1 ceh-18 lim-6 pha-2 lin-39 ZC123.3 | 78 | 1.54 | 1.8e-04 |
| EN1\_2 |  | ceh-2 ceh-16 | 68 | 1.65 | 1.8e-04 |
| pTH9237 |  | mel-28 | 89 | 1.44 | 2.1e-04 |
| MA0536.1 |  | elt-1 | 20 | 3.54 | 2.2e-04 |
| V$BRN2\_01 |  | ceh-18 | 91 | 1.40 | 3.6e-04 |
| MA0095.2 |  | lsy-2 (-0.53) | 26 | 2.78 | 3.8e-04 |
| pTH8566 |  | lin-54 | 89 | 1.41 | 4.3e-04 |
| CG31670\_SANGER\_5\_FBgn0031375 |  | CELE\_Y38H8A.5 | 86 | 1.43 | 4.8e-04 |
| Eip93F\_SANGER\_10\_FBgn0013948 |  | mbr-1 bed-3 nhr-177 | 83 | 1.45 | 5.9e-04 |
| Mv109 |  | pax-2 | 34 | 2.27 | 6.1e-04 |
| PRRX1\_3 |  | alr-1 ceh-14 cfi-1 ZC204.2 | 46 | 1.92 | 6.1e-04 |
| HSFY2\_1 |  | hsf-1 (-0.64) | 63 | 1.64 | 6.8e-04 |
| pTH5916 |  | efl-2 | 43 | 1.98 | 7.2e-04 |
| pTH9220 |  | mbr-1 | 75 | 1.51 | 8.5e-04 |
| pTH9393 |  | F39B2.1 (-0.52) ZC416.1 | 46 | 1.88 | 1.0e-03 |
| pTH8649 |  | mbr-1 | 74 | 1.51 | 1.0e-03 |
| CXXC1\_si |  | F52B11.1 | 60 | 1.65 | 1.2e-03 |
| MA0543.1 |  | eor-1 | 91 | 1.37 | 1.2e-03 |
| pTH10798 |  | Y75B8A.6 | 46 | 1.85 | 1.5e-03 |
| Mafk\_3106 |  | F45H11.6 | 78 | 1.45 | 1.8e-03 |
| HMX1\_1 |  | alr-1 cog-1 ceh-24 ceh-9 ceh-31 lin-39 | 72 | 1.50 | 1.8e-03 |
| Abd-A\_FlyReg\_FBgn0000014 |  | lin-39 | 76 | 1.46 | 2.0e-03 |
| tgo\_cyc\_SANGER\_5\_FBgn0015014 |  | aha-1 (-0.54) hlh-30 | 34 | 2.12 | 2.2e-03 |
| V$FAC1\_01 |  | gei-8 (-0.55) | 88 | 1.36 | 2.5e-03 |
| MA0470.1 |  | efl-1 F49E12.6 | 59 | 1.61 | 2.8e-03 |
| pTH9934 |  | Y53H1A.2 | 50 | 1.73 | 2.8e-03 |
| V$FREAC7\_01 |  | lin-31 | 80 | 1.41 | 3.3e-03 |
| pTH5260 |  | aha-1 (-0.54) lin-22 | 48 | 1.74 | 3.5e-03 |
| pTH4381 |  | mxl-1 (0.73) aha-1 (-0.54) hlh-30 lin-22 | 47 | 1.76 | 3.5e-03 |
| V$TST1\_01 |  | alr-1 ceh-18 lin-39 | 68 | 1.50 | 3.6e-03 |
| MA0253.1 |  | dsc-1 | 73 | 1.45 | 4.5e-03 |
| MA0547.1 |  | skn-1 (-0.61) | 88 | 1.34 | 5.0e-03 |
| Atf1\_3026 |  | crh-1 | 58 | 1.58 | 5.7e-03 |
| V$CDC5\_01 |  | D1081.8 | 66 | 1.50 | 5.8e-03 |
| Hmx2\_3424 |  | ceh-9 | 58 | 1.58 | 5.8e-03 |
| MA0459.1 |  | nhr-239 | 78 | 1.40 | 6.6e-03 |
| pTH9052 |  | atf-2 ces-2 F23F12.9 C48E7.11 | 54 | 1.61 | 6.9e-03 |
| pnt\_SANGER\_5\_FBgn0003118 |  | lin-1 | 65 | 1.49 | 7.0e-03 |
| CG12029\_SANGER\_10\_FBgn0035454 |  | klf-1 | 51 | 1.63 | 8.1e-03 |
| HES1\_f1 |  | lin-22 | 51 | 1.63 | 9.1e-03 |
| Hoxd11\_3873 |  | php-3 | 64 | 1.49 | 9.2e-03 |
| HIF1A\_si |  | hif-1 (-0.73) | 46 | 1.69 | 9.8e-03 |
| pTH10633 |  | R07H5.10 | 83 | 1.35 | 9.9e-03 |
| pTH9969 |  | pag-3 | 70 | 1.43 | 1.1e-02 |
| V$CEBPA\_01 |  | C48E7.11 | 69 | 1.44 | 1.1e-02 |
| pTH5098 |  | D1081.8 F45H11.6 | 47 | 1.66 | 1.2e-02 |
| V$OCT1\_06 |  | ceh-18 | 82 | 1.34 | 1.3e-02 |
| HXD10\_f1 |  | php-3 | 83 | 1.34 | 1.3e-02 |
| pTH9911 |  | atf-5 | 35 | 1.86 | 1.4e-02 |
| FLI1\_4 |  | lin-1 | 41 | 1.74 | 1.4e-02 |
| Hr51\_SANGER\_5\_FBgn0034012 |  | nhr-100 | 76 | 1.37 | 1.4e-02 |
| MA0227.1 |  | ceh-32 | 55 | 1.55 | 1.4e-02 |
| pTH3477 |  | daf-16 (-0.54) | 77 | 1.37 | 1.5e-02 |
| tgo\_ss\_SANGER\_5\_FBgn0015014 |  | aha-1 (-0.54) | 33 | 1.90 | 1.5e-02 |
| MA0495.1 |  | F45H11.6 | 57 | 1.52 | 1.5e-02 |
| pTH3516 |  | daf-19 (-0.56) | 47 | 1.64 | 1.6e-02 |
| Sox17\_2837 |  | sox-4 | 77 | 1.36 | 1.6e-02 |
| SP4\_f1 |  | klf-2 | 28 | 2.02 | 1.8e-02 |
| Eip74EF\_SANGER\_5\_FBgn0000567 |  | C24A1.2 | 54 | 1.54 | 1.8e-02 |
| MA0146.2 |  | F58G1.2 | 35 | 1.82 | 1.9e-02 |
| pTH9384 |  | cfi-1 | 79 | 1.34 | 1.9e-02 |
| Hoxd13\_2356 |  | pal-1 | 70 | 1.40 | 2.0e-02 |
| HLH1 |  | hlh-1 | 29 | 1.98 | 2.0e-02 |
| pTH9314 |  | atf-7 | 21 | 2.32 | 2.1e-02 |
| pTH10808 |  | ztf-19 | 65 | 1.43 | 2.2e-02 |
| Rfxdc2\_3516 |  | daf-19 (-0.56) | 46 | 1.61 | 2.3e-02 |
| pTH9916 |  | crh-1 W08E12.1 | 10 | 3.86 | 2.3e-02 |
| Mw151 |  | gei-11 | 84 | 1.30 | 2.4e-02 |
| V$PAX2\_02 |  | pax-1 | 57 | 1.49 | 2.4e-02 |
| Lmx1b\_3433 |  | lim-6 | 39 | 1.71 | 2.4e-02 |
| pTH5250 |  | C48E7.11 | 35 | 1.78 | 2.7e-02 |
| MA0038.1 |  | odd-1 | 68 | 1.39 | 2.7e-02 |
| EGR1\_2 |  | ZC328.2 klf-2 | 46 | 1.59 | 2.9e-02 |
| pTH6641 |  | lin-31 | 77 | 1.33 | 3.0e-02 |
| MA0085.1 |  | lag-1 | 67 | 1.39 | 3.1e-02 |
| ATF1\_si |  | crh-1 | 10 | 3.67 | 3.1e-02 |
| Lhx1\_2240 |  | lim-7 | 19 | 2.35 | 3.1e-02 |
| pTH6591 |  | lin-31 | 80 | 1.31 | 3.2e-02 |
| HEN1\_si |  | hlh-15 | 47 | 1.57 | 3.2e-02 |
| pTH9089 |  | ref-2 | 41 | 1.65 | 3.4e-02 |
| Sox1\_2631 |  | sox-4 | 69 | 1.37 | 3.5e-02 |
| pTH9096 |  | T07C12.11 | 37 | 1.71 | 3.5e-02 |
| pTH1014 |  | atf-5 | 42 | 1.63 | 3.6e-02 |
| pTH2846 |  | lin-31 | 77 | 1.32 | 3.7e-02 |
| pTH9164 |  | ceh-26 | 11 | 3.27 | 3.9e-02 |
| NFIA\_1 |  | nfi-1 | 12 | 3.04 | 4.1e-02 |
| V$MYB\_Q6 |  | D1081.8 | 39 | 1.65 | 4.3e-02 |
| Hoxb9\_3413 |  | ceh-24 pal-1 | 69 | 1.36 | 4.4e-02 |
| MA0600.1 |  | daf-19 (-0.56) | 48 | 1.53 | 4.4e-02 |
| TLX1\_f1 |  | ceh-19 | 45 | 1.56 | 4.6e-02 |
| pTH6425 |  | ceh-20 | 66 | 1.38 | 4.6e-02 |
| pTH5118 |  | cfi-1 | 74 | 1.33 | 4.6e-02 |
| pTH8679 |  | pax-2 | 22 | 2.08 | 4.8e-02 |
| Gmeb1\_1745 |  | attf-1 | 37 | 1.67 | 4.8e-02 |

### Correlated (and anti-correlated) transcription factors

|  |  |
| --- | --- |
| **Transcription factor** | **Correlation** |
| Y56A3A.18 | 0.84 |
| mxl-1 | 0.73 |
| C01F6.9 | 0.72 |
| mxl-2 | 0.68 |
| T26A5.8 | 0.66 |
| lst-5 | 0.54 |
| madf-10 | 0.48 |
| C16A3.4 | 0.48 |
| C02F12.5 | 0.46 |
| hmg-11 | 0.43 |
| hlh-12 | 0.39 |
| nhr-205 | 0.38 |
| hlh-15 | 0.38 |
| cebp-2 | 0.37 |
| mbf-1 | 0.35 |
| dct-13 | 0.35 |
| repo-1 | 0.34 |
| nhr-269 | 0.33 |
| nhr-273 | 0.33 |
| dhhc-1 | 0.33 |
| madf-2 | 0.33 |
| lir-3 | 0.33 |
| snu-23 | 0.32 |
| ztf-4 | 0.31 |
| nhr-122 | 0.30 |
| tag-68 | -0.61 |
| hbl-1 | -0.61 |
| C04F5.9 | -0.62 |
| hsf-1 | -0.64 |
| dhhc-14 | -0.65 |
| T10D4.6 | -0.65 |
| nhr-1 | -0.65 |
| set-16 | -0.65 |
| B0261.1 | -0.66 |
| sem-4 | -0.67 |
| let-526 | -0.68 |
| rbr-2 | -0.68 |
| B0336.3 | -0.68 |
| Y48G8AL.10 | -0.68 |
| tag-146 | -0.69 |
| F10B5.3 | -0.69 |
| nhr-48 | -0.69 |
| nhr-214 | -0.70 |
| nhr-20 | -0.70 |
| F57A8.1 | -0.70 |
| miz-1 | -0.71 |
| hif-1 | -0.73 |
| med-2 | -0.73 |
| chd-7 | -0.73 |
| egrh-1 | -0.78 |

### ChIP peaks enriched

|  |  |  |  |  |
| --- | --- | --- | --- | --- |
| **Gene** | **Experiment** | **Number of upstream peaks** | **Enrichment** | **FDR corrected p** |
| efl-1 | EFL-1\_Fed-L1-stage-larvae | 75 | 3.54 | 2.1e-24 |
| lsy-2 | LSY-2\_Fed-L1-stage-larvae | 75 | 3.31 | 1.4e-22 |
| C34F6.9 | C34F6.9\_Larvae-L2-stage | 77 | 3.12 | 8.6e-22 |
| lsy-2 | LSY-2\_Embryos | 64 | 3.88 | 1.2e-21 |
| efl-1 | EFL-1\_Larvae-L1-stage | 76 | 3.13 | 1.9e-21 |
| dpl-1 | DPL-1\_Fed-L1-stage-larvae | 71 | 3.29 | 1.0e-20 |
| lin-35 | LIN-35\_Fed-L1-stage-larvae | 69 | 3.32 | 4.4e-20 |
| lsy-2 | LSY-2\_Larvae-L1-stage | 81 | 2.75 | 5.8e-20 |
| efl-1 | EFL-1\_Young-adult | 77 | 2.91 | 6.1e-20 |
| lin-13 | LIN-13\_Larvae-L2-stage | 59 | 3.66 | 3.0e-18 |
| dpl-1 | DPL-1\_Larvae-L4-stage | 81 | 2.52 | 1.9e-17 |
| nfya-1 | NFYA-1\_Larvae-L3-stage | 61 | 3.26 | 1.4e-16 |
| ham-1 | HAM-1\_Larvae-L4-stage | 71 | 2.77 | 2.0e-16 |
| F23B12.7 | F23B12.7\_Young-adult | 52 | 3.80 | 3.3e-16 |
| eor-1 | EOR-1\_Larvae-L3-stage | 70 | 2.76 | 5.1e-16 |
| ces-1 | CES-1\_Embryos | 68 | 2.78 | 1.4e-15 |
| C01B12.2 | C01B12.2\_Larvae-L2-stage | 79 | 2.39 | 2.0e-15 |
| W03F9.2 | W03F9.2\_L4-Young-Adult-stage-larvae | 86 | 2.20 | 2.4e-15 |
| gei-11 | GEI-11\_Larvae-L3-stage | 66 | 2.83 | 2.8e-15 |
| hpl-2 | HPL-2\_Fed-L1-stage-larvae | 74 | 2.50 | 6.2e-15 |
| F16B12.6 | F16B12.6\_Fed-L1-stage-larvae | 43 | 4.31 | 1.0e-14 |
| dpl-1 | DPL-1\_Young-adult | 61 | 2.98 | 1.1e-14 |
| aly-2 | ALY-2\_Fed-L1-stage-larvae | 55 | 3.27 | 1.6e-14 |
| nhr-237 | NHR-237\_Embryos | 41 | 4.42 | 2.8e-14 |
| R02D3.7 | R02D3.7\_Larvae-L3-stage | 67 | 2.65 | 3.9e-14 |
| gei-11 | GEI-11\_Fed-L1-stage-larvae | 63 | 2.78 | 5.8e-14 |
| lin-15 | LIN-15B\_Fed-L1-stage-larvae | 46 | 3.78 | 7.3e-14 |
| nfya-1 | NFYA-1\_Late-Embryos | 60 | 2.80 | 3.7e-13 |
| nhr-77 | NHR-77\_Larvae-L4-stage | 78 | 2.18 | 9.3e-13 |
| R02D3.7 | R02D3.7\_Larvae-L2-stage | 43 | 3.70 | 1.7e-12 |
| ceh-38 | CEH-38\_Larvae-L4-stage | 42 | 3.77 | 2.1e-12 |
| R02D3.7 | R02D3.7\_Larvae-L4-stage | 39 | 4.03 | 3.0e-12 |
| gei-11 | GEI-11\_Larvae-L2-stage | 52 | 3.01 | 3.9e-12 |
| ceh-38 | CEH-38\_Larvae-L3-stage | 57 | 2.73 | 7.2e-12 |
| C16A3.4 | C16A3.4\_Fed-L1-stage-larvae | 50 | 2.99 | 1.9e-11 |
| ham-1 | HAM-1\_Fed-L1-stage-larvae | 60 | 2.51 | 4.3e-11 |
| pes-1 | PES-1\_Larvae-L4-stage | 60 | 2.44 | 1.3e-10 |
| fos-1 | FOS-1\_Fed-L1-stage-larvae | 56 | 2.56 | 2.0e-10 |
| F45C12.2 | F45C12.2\_Fed-L1-stage-larvae | 53 | 2.62 | 4.3e-10 |
| sem-4 | SEM-4\_Larvae-L2-stage | 58 | 2.43 | 4.7e-10 |
| nhr-77 | NHR-77\_Fed-L1-stage-larvae | 56 | 2.48 | 6.3e-10 |
| lsy-2 | LSY-2\_Larvae-L2-stage | 35 | 3.62 | 1.5e-09 |
| ceh-39 | CEH-39\_Embryos | 40 | 3.14 | 2.6e-09 |
| nhr-23 | NHR-23\_Larvae-L3-stage | 52 | 2.46 | 7.5e-09 |
| lin-15 | LIN-15B\_Larvae-L4-stage | 25 | 4.67 | 1.1e-08 |
| ztf-7 | ZTF-7\_Larvae-L4-stage | 40 | 2.96 | 1.4e-08 |
| lin-13 | LIN-13\_Larvae-L4-stage | 37 | 3.04 | 3.9e-08 |
| nhr-25 | NHR-25\_Larvae-L2-stage | 48 | 2.48 | 4.2e-08 |
| nhr-129 | NHR-129\_Larvae-L2-stage | 66 | 1.98 | 5.0e-08 |
| pha-4 | PHA-4\_Larvae-L2-stage | 58 | 2.11 | 1.2e-07 |
| nhr-6 | NHR-6\_Larvae-L4-stage | 37 | 2.89 | 1.5e-07 |
| nhr-6 | NHR-6\_Larvae-L2-stage | 50 | 2.28 | 2.5e-07 |
| nhr-77 | NHR-77\_Larvae-L2-stage | 33 | 3.00 | 5.5e-07 |
| lin-35 | LIN-35\_Young-adult | 31 | 3.08 | 9.3e-07 |
| lin-13 | LIN-13\_Larvae-L1-stage | 20 | 4.51 | 1.4e-06 |
| gei-11 | GEI-11\_Young-adult | 31 | 2.92 | 3.1e-06 |
| zag-1 | ZAG-1\_Larvae-L3-stage | 26 | 3.26 | 5.4e-06 |
| alr-1 | ALR-1\_Larvae-L2-stage | 45 | 2.19 | 7.1e-06 |
| zag-1 | ZAG-1\_Larvae-L2-stage | 35 | 2.41 | 3.1e-05 |
| F45C12.2 | F45C12.2\_Larvae-L3-stage | 23 | 3.20 | 4.2e-05 |
| ces-1 | CES-1\_Fed-L1-stage-larvae | 25 | 2.99 | 4.3e-05 |
| nhr-76 | NHR-76\_Larvae-L4-stage | 30 | 2.62 | 4.4e-05 |
| sax-3 | SAX-3\_Larvae-L4-stage | 52 | 1.89 | 4.7e-05 |
| skn-1 | SKN-1\_Larvae-L3-stage | 26 | 2.86 | 5.7e-05 |
| hlh-30 | HLH-30\_Late-Embryos | 26 | 2.82 | 7.3e-05 |
| ztf-4 | ZTF-4\_Larvae-L2-stage | 22 | 3.17 | 8.4e-05 |
| lin-35 | LIN-35\_Starved-L1-stage-larvae | 25 | 2.86 | 9.6e-05 |
| F45C12.2 | F45C12.2\_Larvae-L2-stage | 19 | 3.51 | 1.1e-04 |
| nhr-77 | NHR-77\_Larvae-L3-stage | 36 | 2.24 | 1.1e-04 |
| dve-1 | DVE-1\_Late-Embryos | 40 | 2.10 | 1.2e-04 |
| pha-4 | PHA-4\_Young-adult | 24 | 2.88 | 1.4e-04 |
| elt-3 | ELT-3\_Embryos | 28 | 2.56 | 1.6e-04 |
| aly-2 | ALY-2\_Larvae-L3-stage | 31 | 2.40 | 1.7e-04 |
| zag-1 | ZAG-1\_Fed-L1-stage-larvae | 23 | 2.88 | 2.2e-04 |
| mab-5 | MAB-5\_Embryos | 12 | 4.74 | 4.9e-04 |
| nhr-237 | NHR-237\_Larvae-L2-stage | 10 | 5.71 | 5.7e-04 |
| nhr-28 | NHR-28\_Larvae-L3-stage | 14 | 3.89 | 7.6e-04 |
| lsy-2 | LSY-2\_Larvae-L4-stage | 21 | 2.82 | 7.9e-04 |
| nhr-237 | NHR-237\_Larvae-L1-stage | 18 | 3.13 | 8.6e-04 |
| sax-3 | SAX-3\_Larvae-L2-stage | 34 | 2.09 | 9.1e-04 |
| sea-2 | SEA-2\_Larvae-L3-stage | 17 | 3.19 | 1.1e-03 |
| ama-1 | AMA-1\_Larvae-L3-stage | 22 | 2.60 | 1.5e-03 |
| egl-5 | EGL-5\_Larvae-L3-stage | 33 | 2.04 | 1.9e-03 |
| pha-4 | PHA-4\_Larvae-L4-stage | 31 | 2.10 | 2.0e-03 |
| nhr-21 | NHR-21\_Larvae-L2-stage | 17 | 3.02 | 2.1e-03 |
| ztf-11 | ZTF-11\_Embryos | 12 | 3.96 | 2.5e-03 |
| ces-1 | CES-1\_Larvae-L3-stage | 21 | 2.58 | 2.5e-03 |
| hlh-30 | HLH-30\_Larvae-L4-stage | 24 | 2.36 | 2.8e-03 |
| med-1 | MED-1\_Embryos | 13 | 3.61 | 3.0e-03 |
| aha-1 | AHA-1\_Larvae-L4-stage | 17 | 2.92 | 3.1e-03 |
| jun-1 | JUN-1\_Larvae-L4-stage | 26 | 2.24 | 3.1e-03 |
| unc-62 | UNC-62\_Fed-L1-stage-larvae | 15 | 3.18 | 3.3e-03 |
| jun-1 | JUN-1\_Larvae-L1-stage | 34 | 1.92 | 4.4e-03 |
| nhr-76 | NHR-76\_Larvae-L3-stage | 22 | 2.31 | 7.3e-03 |
| aha-1 | AHA-1\_Fed-L1-stage-larvae | 10 | 4.07 | 7.5e-03 |
| ceh-26 | CEH-26\_Late-Embryonic-stage | 25 | 2.15 | 7.6e-03 |
| nfya-1 | NFYA-1\_Young-adult | 16 | 2.78 | 8.1e-03 |
| gei-11 | GEI-11\_Embryos | 15 | 2.90 | 8.2e-03 |
| nhr-11 | NHR-11\_Larvae-L2-stage | 17 | 2.66 | 8.5e-03 |
| zag-1 | ZAG-1\_Larvae-L4-stage | 25 | 2.11 | 9.9e-03 |
| unc-62 | UNC-62\_Larvae-L2-stage | 18 | 2.52 | 1.0e-02 |
| fos-1 | FOS-1\_Larvae-L4-stage | 16 | 2.70 | 1.1e-02 |
| mab-5 | MAB-5\_Larvae-L2-stage | 21 | 2.29 | 1.1e-02 |
| ztf-4 | ZTF-4\_Larvae-L1-stage | 12 | 3.28 | 1.2e-02 |
| pax-1 | PAX-1\_Embryos | 14 | 2.91 | 1.2e-02 |
| fos-1 | FOS-1\_Larvae-L3-stage | 31 | 1.88 | 1.2e-02 |
| fkh-2 | FKH-2\_Larvae-L3-stage | 15 | 2.75 | 1.4e-02 |
| sax-3 | SAX-3\_Fed-L1-stage-larvae | 14 | 2.76 | 2.0e-02 |
| ztf-4 | ZTF-4\_Larvae-L3-stage | 11 | 3.16 | 2.6e-02 |
| daf-12 | DAF-12\_Larvae-L3-stage | 9 | 3.61 | 3.1e-02 |
| nhr-2 | NHR-2\_Embryos | 18 | 2.24 | 3.5e-02 |
| unc-62 | UNC-62\_Larvae-L3-stage | 21 | 2.05 | 4.1e-02 |
