# Supplementary material for: Overlapping cell population expression profiling and regulatory inference in C. elegans
Source: BMC Genomics. 2016 Feb 29;17:159. doi: 10.1186/s12864-016-2482-z (PMC4772325; doi:10.1186/s12864-016-2482-z)
Supplement: Additional file 13: — Web supplement. (DOC 21 kb) [file 12864_2016_2482_MOESM13_ESM.zip › sortWeb/clusters/hier.300.clusters/170.html]

Cluster 170 

## Cluster 170

### Expression

| cnd-1 rep. 1 | cnd-1 rep. 2 | cnd-1 rep. 3 | pha-4 rep. 1 | pha-4 rep. 2 | pha-4 rep. 3 | ceh-27 | ceh-36 | ceh-6 | F21D5.9 | mir-57 | mls-2 | pal-1 | pros-1 | ttx-3 | unc-130 | hlh-16 | irx-1 | ceh-6 (+) hlh-16 (+) | ceh-6 (+) hlh-16 (-) | ceh-6 (-) hlh-16 (+) | cnd-1 singlets | pha-4 singlets | 0 | 60 | 120 | 150 | 180 | 240 | 330 | 390 | 420 | 480 | 540 | 570 | 600 | 630 | 660 | NAME | Functional description |
| --- | --- | --- | --- | --- | --- | --- | --- | --- | --- | --- | --- | --- | --- | --- | --- | --- | --- | --- | --- | --- | --- | --- | --- | --- | --- | --- | --- | --- | --- | --- | --- | --- | --- | --- | --- | --- | --- | --- | --- |
|  |  |  |  |  |  |  |  |  |  |  |  |  |  |  |  |  |  |  |  |  |  |  |  |  |  |  |  |  |  |  |  |  |  |  |  |  |  | Y47H9B.2 |  |
|  |  |  |  |  |  |  |  |  |  |  |  |  |  |  |  |  |  |  |  |  |  |  |  |  |  |  |  |  |  |  |  |  |  |  |  |  |  | *ccch-3* | CCCH-type zinc finger putative transcription factor |
|  |  |  |  |  |  |  |  |  |  |  |  |  |  |  |  |  |  |  |  |  |  |  |  |  |  |  |  |  |  |  |  |  |  |  |  |  |  | K11D2.1 |  |
|  |  |  |  |  |  |  |  |  |  |  |  |  |  |  |  |  |  |  |  |  |  |  |  |  |  |  |  |  |  |  |  |  |  |  |  |  |  | *pas-5* | Proteasome Alpha Subunit |
|  |  |  |  |  |  |  |  |  |  |  |  |  |  |  |  |  |  |  |  |  |  |  |  |  |  |  |  |  |  |  |  |  |  |  |  |  |  | *pbs-7* | Proteasome Beta Subunit |
|  |  |  |  |  |  |  |  |  |  |  |  |  |  |  |  |  |  |  |  |  |  |  |  |  |  |  |  |  |  |  |  |  |  |  |  |  |  | *rpb-11* | RNA Polymerase II (B) subunit |
|  |  |  |  |  |  |  |  |  |  |  |  |  |  |  |  |  |  |  |  |  |  |  |  |  |  |  |  |  |  |  |  |  |  |  |  |  |  | Y48G1C.9 |  |
|  |  |  |  |  |  |  |  |  |  |  |  |  |  |  |  |  |  |  |  |  |  |  |  |  |  |  |  |  |  |  |  |  |  |  |  |  |  | *copz-1* | COat Protein complex 1, Zeta subunit |
|  |  |  |  |  |  |  |  |  |  |  |  |  |  |  |  |  |  |  |  |  |  |  |  |  |  |  |  |  |  |  |  |  |  |  |  |  |  | W03C9.5 |  |
|  |  |  |  |  |  |  |  |  |  |  |  |  |  |  |  |  |  |  |  |  |  |  |  |  |  |  |  |  |  |  |  |  |  |  |  |  |  | ZK686.1 |  |
|  |  |  |  |  |  |  |  |  |  |  |  |  |  |  |  |  |  |  |  |  |  |  |  |  |  |  |  |  |  |  |  |  |  |  |  |  |  | *arx-5* | ARp2/3 compleX component |
|  |  |  |  |  |  |  |  |  |  |  |  |  |  |  |  |  |  |  |  |  |  |  |  |  |  |  |  |  |  |  |  |  |  |  |  |  |  | F40F8.3 |  |
|  |  |  |  |  |  |  |  |  |  |  |  |  |  |  |  |  |  |  |  |  |  |  |  |  |  |  |  |  |  |  |  |  |  |  |  |  |  | F40F8.12 |  |
|  |  |  |  |  |  |  |  |  |  |  |  |  |  |  |  |  |  |  |  |  |  |  |  |  |  |  |  |  |  |  |  |  |  |  |  |  |  | W04C9.2 |  |
|  |  |  |  |  |  |  |  |  |  |  |  |  |  |  |  |  |  |  |  |  |  |  |  |  |  |  |  |  |  |  |  |  |  |  |  |  |  | T09A12.5 |  |
|  |  |  |  |  |  |  |  |  |  |  |  |  |  |  |  |  |  |  |  |  |  |  |  |  |  |  |  |  |  |  |  |  |  |  |  |  |  | F02E9.1 |  |
|  |  |  |  |  |  |  |  |  |  |  |  |  |  |  |  |  |  |  |  |  |  |  |  |  |  |  |  |  |  |  |  |  |  |  |  |  |  | *urm-1* | Ubiquitin Related Modifier (yeast URM) homolog |
|  |  |  |  |  |  |  |  |  |  |  |  |  |  |  |  |  |  |  |  |  |  |  |  |  |  |  |  |  |  |  |  |  |  |  |  |  |  | F42A9.8 |  |
|  |  |  |  |  |  |  |  |  |  |  |  |  |  |  |  |  |  |  |  |  |  |  |  |  |  |  |  |  |  |  |  |  |  |  |  |  |  | *fkb-2* | FK506-Binding protein family |
|  |  |  |  |  |  |  |  |  |  |  |  |  |  |  |  |  |  |  |  |  |  |  |  |  |  |  |  |  |  |  |  |  |  |  |  |  |  | *his-72* | HIStone |
|  |  |  |  |  |  |  |  |  |  |  |  |  |  |  |  |  |  |  |  |  |  |  |  |  |  |  |  |  |  |  |  |  |  |  |  |  |  | *moa-2* | Modifier Of Apl-1 activity |
|  |  |  |  |  |  |  |  |  |  |  |  |  |  |  |  |  |  |  |  |  |  |  |  |  |  |  |  |  |  |  |  |  |  |  |  |  |  | *dml-1* | yeast DiM Like |
|  |  |  |  |  |  |  |  |  |  |  |  |  |  |  |  |  |  |  |  |  |  |  |  |  |  |  |  |  |  |  |  |  |  |  |  |  |  | *ave-1* | AVEugle (Drosophila eye differentiation) homolog |
|  |  |  |  |  |  |  |  |  |  |  |  |  |  |  |  |  |  |  |  |  |  |  |  |  |  |  |  |  |  |  |  |  |  |  |  |  |  | *elb-1* | ELongin B |
|  |  |  |  |  |  |  |  |  |  |  |  |  |  |  |  |  |  |  |  |  |  |  |  |  |  |  |  |  |  |  |  |  |  |  |  |  |  | *pbs-2* | Proteasome Beta Subunit |
|  |  |  |  |  |  |  |  |  |  |  |  |  |  |  |  |  |  |  |  |  |  |  |  |  |  |  |  |  |  |  |  |  |  |  |  |  |  | *coq-3* | COenzyme Q (ubiquinone) biosynthesis |
|  |  |  |  |  |  |  |  |  |  |  |  |  |  |  |  |  |  |  |  |  |  |  |  |  |  |  |  |  |  |  |  |  |  |  |  |  |  | Y7A9D.1 |  |
|  |  |  |  |  |  |  |  |  |  |  |  |  |  |  |  |  |  |  |  |  |  |  |  |  |  |  |  |  |  |  |  |  |  |  |  |  |  | F54D11.4 |  |
|  |  |  |  |  |  |  |  |  |  |  |  |  |  |  |  |  |  |  |  |  |  |  |  |  |  |  |  |  |  |  |  |  |  |  |  |  |  | *ubc-12* | UBiquitin Conjugating enzyme |
|  |  |  |  |  |  |  |  |  |  |  |  |  |  |  |  |  |  |  |  |  |  |  |  |  |  |  |  |  |  |  |  |  |  |  |  |  |  | *mxl-2* | MaX-Like |
|  |  |  |  |  |  |  |  |  |  |  |  |  |  |  |  |  |  |  |  |  |  |  |  |  |  |  |  |  |  |  |  |  |  |  |  |  |  | *cut-1* | CUTiclin |
|  |  |  |  |  |  |  |  |  |  |  |  |  |  |  |  |  |  |  |  |  |  |  |  |  |  |  |  |  |  |  |  |  |  |  |  |  |  | F36A2.7 |  |
|  |  |  |  |  |  |  |  |  |  |  |  |  |  |  |  |  |  |  |  |  |  |  |  |  |  |  |  |  |  |  |  |  |  |  |  |  |  | Y39A3CL.3 |  |
|  |  |  |  |  |  |  |  |  |  |  |  |  |  |  |  |  |  |  |  |  |  |  |  |  |  |  |  |  |  |  |  |  |  |  |  |  |  | Y71H2AM.5 |  |
|  |  |  |  |  |  |  |  |  |  |  |  |  |  |  |  |  |  |  |  |  |  |  |  |  |  |  |  |  |  |  |  |  |  |  |  |  |  | *cco-1* | Cytochrome C Oxidase |
|  |  |  |  |  |  |  |  |  |  |  |  |  |  |  |  |  |  |  |  |  |  |  |  |  |  |  |  |  |  |  |  |  |  |  |  |  |  | *dpy-30* | DumPY: shorter than wild-type |
|  |  |  |  |  |  |  |  |  |  |  |  |  |  |  |  |  |  |  |  |  |  |  |  |  |  |  |  |  |  |  |  |  |  |  |  |  |  | B0284.3 |  |
|  |  |  |  |  |  |  |  |  |  |  |  |  |  |  |  |  |  |  |  |  |  |  |  |  |  |  |  |  |  |  |  |  |  |  |  |  |  | *mxl-1* | MaX-Like |
|  |  |  |  |  |  |  |  |  |  |  |  |  |  |  |  |  |  |  |  |  |  |  |  |  |  |  |  |  |  |  |  |  |  |  |  |  |  | *mif-3* | MIF (Macrophage migration Inhibitory Factor) related |
|  |  |  |  |  |  |  |  |  |  |  |  |  |  |  |  |  |  |  |  |  |  |  |  |  |  |  |  |  |  |  |  |  |  |  |  |  |  | C07H6.2 |  |
|  |  |  |  |  |  |  |  |  |  |  |  |  |  |  |  |  |  |  |  |  |  |  |  |  |  |  |  |  |  |  |  |  |  |  |  |  |  | C01F6.9 |  |
|  |  |  |  |  |  |  |  |  |  |  |  |  |  |  |  |  |  |  |  |  |  |  |  |  |  |  |  |  |  |  |  |  |  |  |  |  |  | H08J11.10 |  |
|  |  |  |  |  |  |  |  |  |  |  |  |  |  |  |  |  |  |  |  |  |  |  |  |  |  |  |  |  |  |  |  |  |  |  |  |  |  | F41G4.11 |  |
|  |  |  |  |  |  |  |  |  |  |  |  |  |  |  |  |  |  |  |  |  |  |  |  |  |  |  |  |  |  |  |  |  |  |  |  |  |  | F02A9.7 |  |
|  |  |  |  |  |  |  |  |  |  |  |  |  |  |  |  |  |  |  |  |  |  |  |  |  |  |  |  |  |  |  |  |  |  |  |  |  |  | *linc-88* | Long Intervening Non-Coding RNA |
|  |  |  |  |  |  |  |  |  |  |  |  |  |  |  |  |  |  |  |  |  |  |  |  |  |  |  |  |  |  |  |  |  |  |  |  |  |  | Y54E5A.8 |  |
|  |  |  |  |  |  |  |  |  |  |  |  |  |  |  |  |  |  |  |  |  |  |  |  |  |  |  |  |  |  |  |  |  |  |  |  |  |  | *dao-3* | Dauer or Aging adult Overexpression |
|  |  |  |  |  |  |  |  |  |  |  |  |  |  |  |  |  |  |  |  |  |  |  |  |  |  |  |  |  |  |  |  |  |  |  |  |  |  | C45G9.7 |  |
|  |  |  |  |  |  |  |  |  |  |  |  |  |  |  |  |  |  |  |  |  |  |  |  |  |  |  |  |  |  |  |  |  |  |  |  |  |  | *cyn-6* | CYclophyliN |
|  |  |  |  |  |  |  |  |  |  |  |  |  |  |  |  |  |  |  |  |  |  |  |  |  |  |  |  |  |  |  |  |  |  |  |  |  |  | *let-754* | LEThal |

### Phenotypes enriched

none found

### Anatomy terms enriched

none found

### GO terms enriched

|  |  |  |
| --- | --- | --- |
| **GO term** | **Number of genes** | **FDR-corrected p-value** |
| threonine-type endopeptidase activity | 3 | 0.0028 |

### Expression clusters enriched

|  |  |  |  |
| --- | --- | --- | --- |
| **Group name** | **Number in cluster** | **Enrichment** | **FDR corrected p** |
| Genes with decreased expression after 24 hours of infection by E.faecalis Fold changes shown are pathogen vs OP50. WBPaper00038438:E.faecalis\_24hr\_downregulated\_RNAseq | 13 | 5.58 | 0.000133 |
| Caenorhabditis elegans Genes with expression levels changed significantly after treatment of Bacillus thurigiensis DB27. | 31 | 2.03 | 0.001400 |
| Maternal degradation (MD) subclasses are based on the earliest significant decrease (abbreviated pd for primary decrease). [cgc5767]:expression\_class\_MD\_pd(23\_min) | 10 | 5.77 | 0.002200 |
| Genes in the top 10% of expression level across the triplicate L3 samples. To generate the top10 and bottom10 gene sets, authors ranked all genes by mean expression array signal intensity across the three replicates, then took the top and bottom deciles (1,841 genes each) to represent genes with high and low expression. | 18 | 3.07 | 0.002310 |
| Embryonic Pan-neural Enriched Genes. | 16 | 3.10 | 0.007100 |
| Maternal degradation-embryonic class (MDE): genes that are the subset of maternal degradation genes that significantly increase in at least two of the eight total paired timepoint tests in the induction-following-degradation time domain. | 10 | 4.91 | 0.007690 |
| Maternal class (M): genes that are called present in at least one of the three PC6 replicates. | 33 | 1.78 | 0.008770 |
| Maternal degradation class (MD): genes that are the subset of maternal genes that decrease without first increasing in abundance. | 16 | 2.88 | 0.015800 |
| Genes enriched in intestine. | 17 | 2.66 | 0.022100 |
| Genes significantly enriched (> 2x, FDR < 5%) in a particular cell-type versus a reference sample of all cells at the same stage. WBPaper00037950:hypodermis\_larva\_enriched | 13 | 3.26 | 0.025700 |
| Genes that showed expression levels higher than the corresponding reference sample (embryonic 0hr reference). WBPaper00037950:BAG-neuron\_expressed | 27 | 1.84 | 0.047400 |

### Motifs enriched

|  |  |  |  |  |  |
| --- | --- | --- | --- | --- | --- |
| **Motif** | **Logo** | **Possible orthologs** | **Number of motifs in cluster** | **Enrichment** | **FDR corrected p** |
| ONECUT1\_1 |  | dsc-1 ceh-48 | 35 | 2.45 | 9.4e-07 |
| MA0536.1 |  | elt-1 | 34 | 2.12 | 6.9e-05 |
| Blimp-1\_SANGER\_5\_FBgn0035625 |  | blmp-1 | 43 | 1.57 | 6.2e-04 |
| pTH5098 |  | F45H11.6 | 30 | 2.12 | 6.2e-04 |
| pTH9173 |  | efl-2 | 16 | 3.64 | 6.9e-04 |
| Zfp161\_2858 |  | pzf-1 | 15 | 3.45 | 2.2e-03 |
| pTH2280 |  | mnm-2 | 21 | 2.53 | 2.9e-03 |
| Mw151 |  | gei-11 | 40 | 1.56 | 3.3e-03 |
| Foxc1\_2 |  | daf-16 lin-31 let-381 | 38 | 1.62 | 3.7e-03 |
| pTH5656 |  | fkh-7 (-0.69) daf-16 lin-31 let-381 fkh-8 fkh-10 | 38 | 1.62 | 3.9e-03 |
| MEIS2\_2 |  | ceh-32 | 9 | 5.46 | 4.4e-03 |
| Mrg2\_2302 |  | ceh-32 | 9 | 5.41 | 4.6e-03 |
| FOXC2\_3 |  | lin-31 let-381 | 19 | 2.57 | 5.9e-03 |
| Sox30\_2781 |  | gei-3 C05C9.3 | 35 | 1.67 | 6.7e-03 |
| pTH9097 |  | Y116A8C.22 | 43 | 1.42 | 8.7e-03 |
| pTH4425 |  | cfi-1 | 34 | 1.66 | 1.1e-02 |
| retn\_SANGER\_5\_FBgn0004795 |  | cfi-1 ceh-20 | 39 | 1.51 | 1.1e-02 |
| pTH9254 |  | mel-28 | 30 | 1.78 | 1.3e-02 |
| eve\_FlyReg\_FBgn0000606 |  | ceh-53 | 36 | 1.58 | 1.4e-02 |
| FOXO1\_si |  | daf-16 | 40 | 1.45 | 1.8e-02 |
| MA0453.1 |  | ceh-18 tbp-1 | 37 | 1.53 | 1.9e-02 |
| V$AHR\_01 |  | ahr-1 | 7 | 5.67 | 1.9e-02 |
| MA0473.1 |  | C24A1.2 | 34 | 1.60 | 1.9e-02 |
| Pknox2\_3077 |  | ceh-32 | 8 | 4.80 | 2.0e-02 |
| pTH6497 |  | lin-31 | 35 | 1.56 | 2.3e-02 |
| POU4F2\_1 |  | unc-86 | 29 | 1.74 | 2.4e-02 |
| Arid3a\_3875 |  | cfi-1 | 38 | 1.48 | 2.4e-02 |
| pTH9260 |  | mel-28 | 30 | 1.70 | 2.5e-02 |
| V$BRN2\_01 |  | ceh-18 | 38 | 1.48 | 2.6e-02 |
| pTH3796 |  | let-381 | 37 | 1.50 | 2.6e-02 |
| CG2052\_SOLEXA\_2.5\_FBgn0039905 |  | fkh-7 (-0.69) | 42 | 1.38 | 2.6e-02 |
| pTH9335 |  | mel-28 | 38 | 1.47 | 2.9e-02 |
| pTH5916 |  | efl-2 | 21 | 2.07 | 2.9e-02 |
| Irx2\_0900 |  | irx-1 | 11 | 3.27 | 3.0e-02 |
| pTH1014 |  | atf-5 | 21 | 2.05 | 3.2e-02 |
| tgo\_cyc\_SANGER\_5\_FBgn0015014 |  | aha-1 (-0.55) | 19 | 2.18 | 3.3e-02 |
| Tbp\_pr781 |  | tbp-1 | 33 | 1.58 | 3.3e-02 |
| pTH9306 |  | lsl-1 | 14 | 2.66 | 3.4e-02 |
| CXXC1\_si |  | F52B11.1 | 26 | 1.80 | 3.4e-02 |
| pTH2885 |  | hlh-30 (-0.72) | 5 | 7.57 | 3.6e-02 |
| V$NKX61\_01 |  | cog-1 | 16 | 2.38 | 4.0e-02 |
| pTH8982 |  | ceh-48 | 11 | 3.12 | 4.0e-02 |
| MA0541.1 |  | efl-1 | 22 | 1.95 | 4.2e-02 |
| ARI3A\_do |  | cfi-1 | 41 | 1.37 | 4.5e-02 |
| pTH3220 |  | Y5F2A.4 | 24 | 1.84 | 4.6e-02 |
| ESRRA\_3 |  | nhr-71 (-0.55) | 9 | 3.61 | 4.6e-02 |
| V$CDC5\_01 |  | D1081.8 | 29 | 1.66 | 4.7e-02 |

### Correlated (and anti-correlated) transcription factors

|  |  |
| --- | --- |
| **Transcription factor** | **Correlation** |
| mxl-1 | 0.86 |
| mxl-2 | 0.84 |
| Y56A3A.18 | 0.84 |
| C01F6.9 | 0.84 |
| hmg-11 | 0.69 |
| cebp-2 | 0.67 |
| madf-10 | 0.65 |
| ccch-3 | 0.62 |
| T26A5.8 | 0.61 |
| repo-1 | 0.60 |
| hmg-12 | 0.57 |
| ztf-4 | 0.54 |
| nfyc-1 | 0.52 |
| D2030.7 | 0.52 |
| lir-3 | 0.48 |
| pie-1 | 0.47 |
| zip-4 | 0.46 |
| ceh-13 | 0.46 |
| mbf-1 | 0.45 |
| R144.3 | 0.45 |
| C09F5.3 | 0.44 |
| F37B4.10 | 0.44 |
| lst-5 | 0.42 |
| nhr-205 | 0.42 |
| cey-2 | 0.41 |
| F13H6.1 | -0.65 |
| nhr-212 | -0.65 |
| nhr-1 | -0.65 |
| ZC328.2 | -0.66 |
| ets-4 | -0.66 |
| sma-9 | -0.66 |
| nhr-186 | -0.66 |
| nhr-143 | -0.67 |
| Y48G8AL.10 | -0.67 |
| fkh-7 | -0.69 |
| daf-12 | -0.69 |
| nhr-15 | -0.69 |
| ztf-7 | -0.69 |
| nhr-46 | -0.69 |
| nhr-103 | -0.69 |
| nhr-12 | -0.70 |
| die-1 | -0.70 |
| mef-2 | -0.70 |
| hlh-30 | -0.72 |
| let-607 | -0.72 |
| C34B4.2 | -0.75 |
| egrh-1 | -0.76 |
| nhr-49 | -0.77 |
| nhr-125 | -0.81 |
| nhr-20 | -0.81 |

### ChIP peaks enriched

|  |  |  |  |  |
| --- | --- | --- | --- | --- |
| **Gene** | **Experiment** | **Number of upstream peaks** | **Enrichment** | **FDR corrected p** |
| efl-1 | EFL-1\_Fed-L1-stage-larvae | 33 | 3.93 | 2.2e-12 |
| ceh-38 | CEH-38\_Larvae-L3-stage | 28 | 4.46 | 4.2e-11 |
| F16B12.6 | F16B12.6\_Fed-L1-stage-larvae | 23 | 5.81 | 8.1e-11 |
| lin-35 | LIN-35\_Fed-L1-stage-larvae | 31 | 3.76 | 8.3e-11 |
| nhr-23 | NHR-23\_Larvae-L3-stage | 33 | 3.42 | 1.0e-10 |
| lsy-2 | LSY-2\_Larvae-L1-stage | 35 | 3.00 | 4.6e-10 |
| lsy-2 | LSY-2\_Fed-L1-stage-larvae | 31 | 3.45 | 7.5e-10 |
| pes-1 | PES-1\_Larvae-L4-stage | 32 | 3.28 | 9.5e-10 |
| dpl-1 | DPL-1\_Fed-L1-stage-larvae | 30 | 3.51 | 1.4e-09 |
| F45C12.2 | F45C12.2\_Fed-L1-stage-larvae | 29 | 3.61 | 2.1e-09 |
| nfya-1 | NFYA-1\_Larvae-L3-stage | 28 | 3.77 | 2.2e-09 |
| nhr-77 | NHR-77\_Larvae-L4-stage | 37 | 2.60 | 3.6e-09 |
| efl-1 | EFL-1\_Larvae-L1-stage | 31 | 3.21 | 4.6e-09 |
| lin-15 | LIN-15B\_Larvae-L4-stage | 17 | 7.35 | 4.8e-09 |
| C34F6.9 | C34F6.9\_Larvae-L2-stage | 31 | 3.16 | 6.9e-09 |
| C16A3.4 | C16A3.4\_Fed-L1-stage-larvae | 26 | 3.92 | 7.4e-09 |
| eor-1 | EOR-1\_Larvae-L3-stage | 31 | 3.08 | 1.3e-08 |
| ham-1 | HAM-1\_Larvae-L4-stage | 31 | 3.04 | 1.8e-08 |
| ham-1 | HAM-1\_Fed-L1-stage-larvae | 30 | 3.16 | 1.8e-08 |
| hpl-2 | HPL-2\_Fed-L1-stage-larvae | 33 | 2.81 | 2.2e-08 |
| efl-1 | EFL-1\_Young-adult | 26 | 3.72 | 2.3e-08 |
| lin-15 | LIN-15B\_Fed-L1-stage-larvae | 22 | 4.56 | 2.6e-08 |
| W03F9.2 | W03F9.2\_L4-Young-Adult-stage-larvae | 37 | 2.38 | 5.2e-08 |
| C01B12.2 | C01B12.2\_Larvae-L2-stage | 34 | 2.59 | 7.1e-08 |
| fos-1 | FOS-1\_Fed-L1-stage-larvae | 28 | 3.22 | 7.7e-08 |
| gei-11 | GEI-11\_Larvae-L2-stage | 25 | 3.65 | 8.9e-08 |
| dpl-1 | DPL-1\_Young-adult | 27 | 3.32 | 1.0e-07 |
| gei-11 | GEI-11\_Fed-L1-stage-larvae | 28 | 3.12 | 1.6e-07 |
| lsy-2 | LSY-2\_Embryos | 24 | 3.67 | 2.1e-07 |
| nfya-1 | NFYA-1\_Late-Embryos | 27 | 3.17 | 2.6e-07 |
| gei-11 | GEI-11\_Larvae-L3-stage | 28 | 3.03 | 3.0e-07 |
| ceh-39 | CEH-39\_Embryos | 22 | 3.95 | 3.4e-07 |
| R02D3.7 | R02D3.7\_Larvae-L3-stage | 29 | 2.89 | 3.7e-07 |
| nhr-25 | NHR-25\_Larvae-L2-stage | 25 | 3.25 | 8.6e-07 |
| lsy-2 | LSY-2\_Larvae-L2-stage | 18 | 4.69 | 1.1e-06 |
| R02D3.7 | R02D3.7\_Larvae-L2-stage | 19 | 4.13 | 2.8e-06 |
| dve-1 | DVE-1\_Late-Embryos | 24 | 3.17 | 3.2e-06 |
| ztf-7 | ZTF-7\_Larvae-L4-stage | 18 | 4.27 | 4.2e-06 |
| dpl-1 | DPL-1\_Larvae-L4-stage | 31 | 2.43 | 4.3e-06 |
| F23B12.7 | F23B12.7\_Young-adult | 21 | 3.55 | 5.0e-06 |
| nhr-129 | NHR-129\_Larvae-L2-stage | 31 | 2.35 | 9.4e-06 |
| sax-3 | SAX-3\_Larvae-L4-stage | 28 | 2.56 | 1.1e-05 |
| jun-1 | JUN-1\_Larvae-L4-stage | 20 | 3.53 | 1.3e-05 |
| lin-13 | LIN-13\_Larvae-L2-stage | 22 | 3.15 | 1.6e-05 |
| zag-1 | ZAG-1\_Larvae-L4-stage | 18 | 3.83 | 1.9e-05 |
| jun-1 | JUN-1\_Larvae-L3-stage | 19 | 3.47 | 3.5e-05 |
| skn-1 | SKN-1\_Larvae-L3-stage | 16 | 3.98 | 6.2e-05 |
| nhr-77 | NHR-77\_Fed-L1-stage-larvae | 24 | 2.68 | 6.5e-05 |
| ces-1 | CES-1\_Embryos | 25 | 2.58 | 6.8e-05 |
| fos-1 | FOS-1\_Larvae-L2-stage | 28 | 2.29 | 1.0e-04 |
| pha-4 | PHA-4\_Larvae-L2-stage | 26 | 2.38 | 1.6e-04 |
| sem-4 | SEM-4\_Larvae-L2-stage | 24 | 2.54 | 1.7e-04 |
| alr-1 | ALR-1\_Larvae-L2-stage | 22 | 2.69 | 2.1e-04 |
| aly-2 | ALY-2\_Fed-L1-stage-larvae | 21 | 2.79 | 2.2e-04 |
| nhr-6 | NHR-6\_Larvae-L4-stage | 18 | 3.18 | 2.4e-04 |
| lin-35 | LIN-35\_Young-adult | 15 | 3.76 | 2.7e-04 |
| nhr-76 | NHR-76\_Larvae-L4-stage | 17 | 3.26 | 3.6e-04 |
| sax-3 | SAX-3\_Larvae-L2-stage | 19 | 2.94 | 3.7e-04 |
| nhr-237 | NHR-237\_Larvae-L1-stage | 10 | 5.60 | 4.7e-04 |
| zag-1 | ZAG-1\_Larvae-L3-stage | 13 | 4.11 | 5.0e-04 |
| nhr-237 | NHR-237\_Embryos | 14 | 3.80 | 5.1e-04 |
| hlh-30 | HLH-30\_Late-Embryos | 15 | 3.49 | 6.3e-04 |
| elt-3 | ELT-3\_Embryos | 15 | 3.46 | 6.9e-04 |
| zag-1 | ZAG-1\_Larvae-L2-stage | 17 | 2.95 | 1.2e-03 |
| gei-11 | GEI-11\_Embryos | 10 | 4.88 | 1.4e-03 |
| nhr-28 | NHR-28\_Larvae-L4-stage | 26 | 2.10 | 1.5e-03 |
| gei-11 | GEI-11\_Young-adult | 15 | 3.17 | 1.8e-03 |
| nhr-6 | NHR-6\_Larvae-L2-stage | 21 | 2.42 | 1.8e-03 |
| lin-13 | LIN-13\_Larvae-L1-stage | 10 | 4.67 | 2.0e-03 |
| nhr-77 | NHR-77\_Larvae-L3-stage | 19 | 2.58 | 2.1e-03 |
| lin-13 | LIN-13\_Larvae-L4-stage | 15 | 3.11 | 2.2e-03 |
| unc-62 | UNC-62\_Fed-L1-stage-larvae | 10 | 4.58 | 2.3e-03 |
| dve-1 | DVE-1\_Larvae-L4-stage | 18 | 2.66 | 2.4e-03 |
| ztf-4 | ZTF-4\_Larvae-L2-stage | 11 | 4.00 | 3.0e-03 |
| ztf-4 | ZTF-4\_Larvae-L1-stage | 8 | 5.51 | 3.8e-03 |
| jun-1 | JUN-1\_Larvae-L1-stage | 18 | 2.56 | 3.9e-03 |
| ama-1 | AMA-1\_Larvae-L3-stage | 13 | 3.28 | 4.3e-03 |
| hlh-30 | HLH-30\_Larvae-L4-stage | 15 | 2.77 | 7.1e-03 |
| R02D3.7 | R02D3.7\_Larvae-L4-stage | 13 | 3.07 | 8.0e-03 |
| nhr-2 | NHR-2\_Embryos | 11 | 3.45 | 9.9e-03 |
| nhr-77 | NHR-77\_Larvae-L2-stage | 13 | 2.98 | 1.0e-02 |
| nhr-76 | NHR-76\_Larvae-L3-stage | 13 | 2.97 | 1.1e-02 |
| ces-1 | CES-1\_Larvae-L3-stage | 11 | 3.41 | 1.1e-02 |
| lsy-2 | LSY-2\_Larvae-L4-stage | 12 | 3.16 | 1.1e-02 |
| ceh-26 | CEH-26\_Late-Embryonic-stage | 15 | 2.62 | 1.3e-02 |
| egl-5 | EGL-5\_Larvae-L3-stage | 16 | 2.49 | 1.3e-02 |
| nhr-12 | NHR-12\_Larvae-L2-stage | 4 | 11.49 | 1.5e-02 |
| mef-2 | MEF-2\_Fed-L1-stage-larvae | 8 | 4.30 | 1.7e-02 |
| lin-35 | LIN-35\_Starved-L1-stage-larvae | 11 | 3.17 | 1.9e-02 |
| aly-2 | ALY-2\_Larvae-L3-stage | 13 | 2.74 | 2.1e-02 |
| ceh-38 | CEH-38\_Larvae-L4-stage | 12 | 2.72 | 3.6e-02 |
| pha-4 | PHA-4\_Young-adult | 10 | 3.02 | 4.5e-02 |
| fos-1 | FOS-1\_Larvae-L4-stage | 10 | 3.02 | 4.5e-02 |
