# Supplementary material for: Overlapping cell population expression profiling and regulatory inference in C. elegans
Source: BMC Genomics. 2016 Feb 29;17:159. doi: 10.1186/s12864-016-2482-z (PMC4772325; doi:10.1186/s12864-016-2482-z)
Supplement: Additional file 13: — Web supplement. (DOC 21 kb) [file 12864_2016_2482_MOESM13_ESM.zip › sortWeb/clusters/hier.300.clusters/171.html]

Cluster 171 

## Cluster 171

### Expression

| cnd-1 rep. 1 | cnd-1 rep. 2 | cnd-1 rep. 3 | pha-4 rep. 1 | pha-4 rep. 2 | pha-4 rep. 3 | ceh-27 | ceh-36 | ceh-6 | F21D5.9 | mir-57 | mls-2 | pal-1 | pros-1 | ttx-3 | unc-130 | hlh-16 | irx-1 | ceh-6 (+) hlh-16 (+) | ceh-6 (+) hlh-16 (-) | ceh-6 (-) hlh-16 (+) | cnd-1 singlets | pha-4 singlets | 0 | 60 | 120 | 150 | 180 | 240 | 330 | 390 | 420 | 480 | 540 | 570 | 600 | 630 | 660 | NAME | Functional description |
| --- | --- | --- | --- | --- | --- | --- | --- | --- | --- | --- | --- | --- | --- | --- | --- | --- | --- | --- | --- | --- | --- | --- | --- | --- | --- | --- | --- | --- | --- | --- | --- | --- | --- | --- | --- | --- | --- | --- | --- |
|  |  |  |  |  |  |  |  |  |  |  |  |  |  |  |  |  |  |  |  |  |  |  |  |  |  |  |  |  |  |  |  |  |  |  |  |  |  | *perm-4* | PERMeable eggshell |
|  |  |  |  |  |  |  |  |  |  |  |  |  |  |  |  |  |  |  |  |  |  |  |  |  |  |  |  |  |  |  |  |  |  |  |  |  |  | R10E8.6 |  |
|  |  |  |  |  |  |  |  |  |  |  |  |  |  |  |  |  |  |  |  |  |  |  |  |  |  |  |  |  |  |  |  |  |  |  |  |  |  | C18H9.3 |  |
|  |  |  |  |  |  |  |  |  |  |  |  |  |  |  |  |  |  |  |  |  |  |  |  |  |  |  |  |  |  |  |  |  |  |  |  |  |  | Y57G11C.33 |  |
|  |  |  |  |  |  |  |  |  |  |  |  |  |  |  |  |  |  |  |  |  |  |  |  |  |  |  |  |  |  |  |  |  |  |  |  |  |  | *fbxa-65* | F-box A protein |
|  |  |  |  |  |  |  |  |  |  |  |  |  |  |  |  |  |  |  |  |  |  |  |  |  |  |  |  |  |  |  |  |  |  |  |  |  |  | W02D9.9 |  |
|  |  |  |  |  |  |  |  |  |  |  |  |  |  |  |  |  |  |  |  |  |  |  |  |  |  |  |  |  |  |  |  |  |  |  |  |  |  | C17H12.7 |  |
|  |  |  |  |  |  |  |  |  |  |  |  |  |  |  |  |  |  |  |  |  |  |  |  |  |  |  |  |  |  |  |  |  |  |  |  |  |  | C15C6.4 |  |
|  |  |  |  |  |  |  |  |  |  |  |  |  |  |  |  |  |  |  |  |  |  |  |  |  |  |  |  |  |  |  |  |  |  |  |  |  |  | *hpl-1* | HP1 Like (heterochromatin protein) |
|  |  |  |  |  |  |  |  |  |  |  |  |  |  |  |  |  |  |  |  |  |  |  |  |  |  |  |  |  |  |  |  |  |  |  |  |  |  | C26F1.3 |  |
|  |  |  |  |  |  |  |  |  |  |  |  |  |  |  |  |  |  |  |  |  |  |  |  |  |  |  |  |  |  |  |  |  |  |  |  |  |  | *rpb-5* | RNA Polymerase II (B) subunit |
|  |  |  |  |  |  |  |  |  |  |  |  |  |  |  |  |  |  |  |  |  |  |  |  |  |  |  |  |  |  |  |  |  |  |  |  |  |  | *rpac-19* | RNA Polymerase I/III (A/C) shared subunit |
|  |  |  |  |  |  |  |  |  |  |  |  |  |  |  |  |  |  |  |  |  |  |  |  |  |  |  |  |  |  |  |  |  |  |  |  |  |  | ZK265.6 |  |
|  |  |  |  |  |  |  |  |  |  |  |  |  |  |  |  |  |  |  |  |  |  |  |  |  |  |  |  |  |  |  |  |  |  |  |  |  |  | C49H3.3 |  |
|  |  |  |  |  |  |  |  |  |  |  |  |  |  |  |  |  |  |  |  |  |  |  |  |  |  |  |  |  |  |  |  |  |  |  |  |  |  | C48B6.2 |  |
|  |  |  |  |  |  |  |  |  |  |  |  |  |  |  |  |  |  |  |  |  |  |  |  |  |  |  |  |  |  |  |  |  |  |  |  |  |  | F10E7.5 |  |
|  |  |  |  |  |  |  |  |  |  |  |  |  |  |  |  |  |  |  |  |  |  |  |  |  |  |  |  |  |  |  |  |  |  |  |  |  |  | W02B12.10 |  |
|  |  |  |  |  |  |  |  |  |  |  |  |  |  |  |  |  |  |  |  |  |  |  |  |  |  |  |  |  |  |  |  |  |  |  |  |  |  | R08B4.3 |  |
|  |  |  |  |  |  |  |  |  |  |  |  |  |  |  |  |  |  |  |  |  |  |  |  |  |  |  |  |  |  |  |  |  |  |  |  |  |  | *nola-3* | NucleOLus Associated protein homolog |
|  |  |  |  |  |  |  |  |  |  |  |  |  |  |  |  |  |  |  |  |  |  |  |  |  |  |  |  |  |  |  |  |  |  |  |  |  |  | C25A1.16 |  |
|  |  |  |  |  |  |  |  |  |  |  |  |  |  |  |  |  |  |  |  |  |  |  |  |  |  |  |  |  |  |  |  |  |  |  |  |  |  | F47G9.1 |  |
|  |  |  |  |  |  |  |  |  |  |  |  |  |  |  |  |  |  |  |  |  |  |  |  |  |  |  |  |  |  |  |  |  |  |  |  |  |  | Y60A3A.9 |  |
|  |  |  |  |  |  |  |  |  |  |  |  |  |  |  |  |  |  |  |  |  |  |  |  |  |  |  |  |  |  |  |  |  |  |  |  |  |  | Y71H2AM.12 |  |
|  |  |  |  |  |  |  |  |  |  |  |  |  |  |  |  |  |  |  |  |  |  |  |  |  |  |  |  |  |  |  |  |  |  |  |  |  |  | *cyn-9* | CYclophyliN |
|  |  |  |  |  |  |  |  |  |  |  |  |  |  |  |  |  |  |  |  |  |  |  |  |  |  |  |  |  |  |  |  |  |  |  |  |  |  | B0395.t1 |  |
|  |  |  |  |  |  |  |  |  |  |  |  |  |  |  |  |  |  |  |  |  |  |  |  |  |  |  |  |  |  |  |  |  |  |  |  |  |  | *hint-1* | HIstidiNe Triad nucleotide-binding protein |
|  |  |  |  |  |  |  |  |  |  |  |  |  |  |  |  |  |  |  |  |  |  |  |  |  |  |  |  |  |  |  |  |  |  |  |  |  |  | C34B2.5 |  |
|  |  |  |  |  |  |  |  |  |  |  |  |  |  |  |  |  |  |  |  |  |  |  |  |  |  |  |  |  |  |  |  |  |  |  |  |  |  | ZK616.2 |  |
|  |  |  |  |  |  |  |  |  |  |  |  |  |  |  |  |  |  |  |  |  |  |  |  |  |  |  |  |  |  |  |  |  |  |  |  |  |  | T02G6.3 |  |
|  |  |  |  |  |  |  |  |  |  |  |  |  |  |  |  |  |  |  |  |  |  |  |  |  |  |  |  |  |  |  |  |  |  |  |  |  |  | F52C12.2 |  |
|  |  |  |  |  |  |  |  |  |  |  |  |  |  |  |  |  |  |  |  |  |  |  |  |  |  |  |  |  |  |  |  |  |  |  |  |  |  | C14A6.10 |  |
|  |  |  |  |  |  |  |  |  |  |  |  |  |  |  |  |  |  |  |  |  |  |  |  |  |  |  |  |  |  |  |  |  |  |  |  |  |  | F58H1.25 |  |
|  |  |  |  |  |  |  |  |  |  |  |  |  |  |  |  |  |  |  |  |  |  |  |  |  |  |  |  |  |  |  |  |  |  |  |  |  |  | R155.8 |  |
|  |  |  |  |  |  |  |  |  |  |  |  |  |  |  |  |  |  |  |  |  |  |  |  |  |  |  |  |  |  |  |  |  |  |  |  |  |  | C06A6.13 |  |
|  |  |  |  |  |  |  |  |  |  |  |  |  |  |  |  |  |  |  |  |  |  |  |  |  |  |  |  |  |  |  |  |  |  |  |  |  |  | Y54G2A.3 |  |
|  |  |  |  |  |  |  |  |  |  |  |  |  |  |  |  |  |  |  |  |  |  |  |  |  |  |  |  |  |  |  |  |  |  |  |  |  |  | *linc-67* | Long Intervening Non-Coding RNA |
|  |  |  |  |  |  |  |  |  |  |  |  |  |  |  |  |  |  |  |  |  |  |  |  |  |  |  |  |  |  |  |  |  |  |  |  |  |  | *dot-1.2* | DOT1 histone methyltransferase family |
|  |  |  |  |  |  |  |  |  |  |  |  |  |  |  |  |  |  |  |  |  |  |  |  |  |  |  |  |  |  |  |  |  |  |  |  |  |  | B0564.2 |  |
|  |  |  |  |  |  |  |  |  |  |  |  |  |  |  |  |  |  |  |  |  |  |  |  |  |  |  |  |  |  |  |  |  |  |  |  |  |  | C25G4.2 |  |
|  |  |  |  |  |  |  |  |  |  |  |  |  |  |  |  |  |  |  |  |  |  |  |  |  |  |  |  |  |  |  |  |  |  |  |  |  |  | T20G5.9 |  |
|  |  |  |  |  |  |  |  |  |  |  |  |  |  |  |  |  |  |  |  |  |  |  |  |  |  |  |  |  |  |  |  |  |  |  |  |  |  | *dph-3* | DiPHthamide biosynthesis protein |
|  |  |  |  |  |  |  |  |  |  |  |  |  |  |  |  |  |  |  |  |  |  |  |  |  |  |  |  |  |  |  |  |  |  |  |  |  |  | *mrpl-40* | Mitochondrial Ribosomal Protein, Large |
|  |  |  |  |  |  |  |  |  |  |  |  |  |  |  |  |  |  |  |  |  |  |  |  |  |  |  |  |  |  |  |  |  |  |  |  |  |  | C35D10.17 |  |
|  |  |  |  |  |  |  |  |  |  |  |  |  |  |  |  |  |  |  |  |  |  |  |  |  |  |  |  |  |  |  |  |  |  |  |  |  |  | K01G5.8 |  |
|  |  |  |  |  |  |  |  |  |  |  |  |  |  |  |  |  |  |  |  |  |  |  |  |  |  |  |  |  |  |  |  |  |  |  |  |  |  | W02D3.12 |  |
|  |  |  |  |  |  |  |  |  |  |  |  |  |  |  |  |  |  |  |  |  |  |  |  |  |  |  |  |  |  |  |  |  |  |  |  |  |  | Y39B6A.3 |  |
|  |  |  |  |  |  |  |  |  |  |  |  |  |  |  |  |  |  |  |  |  |  |  |  |  |  |  |  |  |  |  |  |  |  |  |  |  |  | *dhod-1* | DiHydroOrotate Dehydrogenas |
|  |  |  |  |  |  |  |  |  |  |  |  |  |  |  |  |  |  |  |  |  |  |  |  |  |  |  |  |  |  |  |  |  |  |  |  |  |  | *cytb-5.2* | YTochrome B |
|  |  |  |  |  |  |  |  |  |  |  |  |  |  |  |  |  |  |  |  |  |  |  |  |  |  |  |  |  |  |  |  |  |  |  |  |  |  | F44G4.2 |  |
|  |  |  |  |  |  |  |  |  |  |  |  |  |  |  |  |  |  |  |  |  |  |  |  |  |  |  |  |  |  |  |  |  |  |  |  |  |  | Y39B6A.34 |  |
|  |  |  |  |  |  |  |  |  |  |  |  |  |  |  |  |  |  |  |  |  |  |  |  |  |  |  |  |  |  |  |  |  |  |  |  |  |  | K02F2.7 |  |
|  |  |  |  |  |  |  |  |  |  |  |  |  |  |  |  |  |  |  |  |  |  |  |  |  |  |  |  |  |  |  |  |  |  |  |  |  |  | H25P19.1 |  |
|  |  |  |  |  |  |  |  |  |  |  |  |  |  |  |  |  |  |  |  |  |  |  |  |  |  |  |  |  |  |  |  |  |  |  |  |  |  | *cni-1* | CorNIchon (trafficking protein) homolog |
|  |  |  |  |  |  |  |  |  |  |  |  |  |  |  |  |  |  |  |  |  |  |  |  |  |  |  |  |  |  |  |  |  |  |  |  |  |  | Y39G10AR.20 |  |
|  |  |  |  |  |  |  |  |  |  |  |  |  |  |  |  |  |  |  |  |  |  |  |  |  |  |  |  |  |  |  |  |  |  |  |  |  |  | F32H2.7 |  |
|  |  |  |  |  |  |  |  |  |  |  |  |  |  |  |  |  |  |  |  |  |  |  |  |  |  |  |  |  |  |  |  |  |  |  |  |  |  | Y76B12C.3 |  |
|  |  |  |  |  |  |  |  |  |  |  |  |  |  |  |  |  |  |  |  |  |  |  |  |  |  |  |  |  |  |  |  |  |  |  |  |  |  | Y54E10BR.5 |  |
|  |  |  |  |  |  |  |  |  |  |  |  |  |  |  |  |  |  |  |  |  |  |  |  |  |  |  |  |  |  |  |  |  |  |  |  |  |  | K12H4.4 |  |
|  |  |  |  |  |  |  |  |  |  |  |  |  |  |  |  |  |  |  |  |  |  |  |  |  |  |  |  |  |  |  |  |  |  |  |  |  |  | *sel-9* | Suppressor/Enhancer of Lin-12 |
|  |  |  |  |  |  |  |  |  |  |  |  |  |  |  |  |  |  |  |  |  |  |  |  |  |  |  |  |  |  |  |  |  |  |  |  |  |  | *gna-1* | Glucosamine phosphate N-Acetyl transferase |
|  |  |  |  |  |  |  |  |  |  |  |  |  |  |  |  |  |  |  |  |  |  |  |  |  |  |  |  |  |  |  |  |  |  |  |  |  |  | *dnc-5* | DyNactin Complex component |
|  |  |  |  |  |  |  |  |  |  |  |  |  |  |  |  |  |  |  |  |  |  |  |  |  |  |  |  |  |  |  |  |  |  |  |  |  |  | *hpo-21* | Hypersensitive to POre-forming toxin |
|  |  |  |  |  |  |  |  |  |  |  |  |  |  |  |  |  |  |  |  |  |  |  |  |  |  |  |  |  |  |  |  |  |  |  |  |  |  | F35H10.5 |  |
|  |  |  |  |  |  |  |  |  |  |  |  |  |  |  |  |  |  |  |  |  |  |  |  |  |  |  |  |  |  |  |  |  |  |  |  |  |  | *lsm-4* | LSM Sm-like protein |
|  |  |  |  |  |  |  |  |  |  |  |  |  |  |  |  |  |  |  |  |  |  |  |  |  |  |  |  |  |  |  |  |  |  |  |  |  |  | *dnc-3* | DyNactin Complex component |
|  |  |  |  |  |  |  |  |  |  |  |  |  |  |  |  |  |  |  |  |  |  |  |  |  |  |  |  |  |  |  |  |  |  |  |  |  |  | *gut-2* | GUT differentiation defective |
|  |  |  |  |  |  |  |  |  |  |  |  |  |  |  |  |  |  |  |  |  |  |  |  |  |  |  |  |  |  |  |  |  |  |  |  |  |  | *did-2* | Doa4-Independent Degradation, homologous to yeast Did2 |
|  |  |  |  |  |  |  |  |  |  |  |  |  |  |  |  |  |  |  |  |  |  |  |  |  |  |  |  |  |  |  |  |  |  |  |  |  |  | F35G12.7 |  |
|  |  |  |  |  |  |  |  |  |  |  |  |  |  |  |  |  |  |  |  |  |  |  |  |  |  |  |  |  |  |  |  |  |  |  |  |  |  | D2030.4 |  |
|  |  |  |  |  |  |  |  |  |  |  |  |  |  |  |  |  |  |  |  |  |  |  |  |  |  |  |  |  |  |  |  |  |  |  |  |  |  | Y94H6A.12 |  |
|  |  |  |  |  |  |  |  |  |  |  |  |  |  |  |  |  |  |  |  |  |  |  |  |  |  |  |  |  |  |  |  |  |  |  |  |  |  | *lsm-7* | LSM Sm-like protein |
|  |  |  |  |  |  |  |  |  |  |  |  |  |  |  |  |  |  |  |  |  |  |  |  |  |  |  |  |  |  |  |  |  |  |  |  |  |  | *rpb-7* | RNA Polymerase II (B) subunit |
|  |  |  |  |  |  |  |  |  |  |  |  |  |  |  |  |  |  |  |  |  |  |  |  |  |  |  |  |  |  |  |  |  |  |  |  |  |  | *emb-1* | abnormal EMBroygenesis |
|  |  |  |  |  |  |  |  |  |  |  |  |  |  |  |  |  |  |  |  |  |  |  |  |  |  |  |  |  |  |  |  |  |  |  |  |  |  | *hpo-18* | Hypersensitive to POre-forming toxin |
|  |  |  |  |  |  |  |  |  |  |  |  |  |  |  |  |  |  |  |  |  |  |  |  |  |  |  |  |  |  |  |  |  |  |  |  |  |  | *snr-7* | Small Nuclear Ribonucleoprotein |
|  |  |  |  |  |  |  |  |  |  |  |  |  |  |  |  |  |  |  |  |  |  |  |  |  |  |  |  |  |  |  |  |  |  |  |  |  |  | T12D8.10 |  |
|  |  |  |  |  |  |  |  |  |  |  |  |  |  |  |  |  |  |  |  |  |  |  |  |  |  |  |  |  |  |  |  |  |  |  |  |  |  | W03G9.8 |  |
|  |  |  |  |  |  |  |  |  |  |  |  |  |  |  |  |  |  |  |  |  |  |  |  |  |  |  |  |  |  |  |  |  |  |  |  |  |  | *snr-1* | Small Nuclear Ribonucleoprotein |
|  |  |  |  |  |  |  |  |  |  |  |  |  |  |  |  |  |  |  |  |  |  |  |  |  |  |  |  |  |  |  |  |  |  |  |  |  |  | *cyn-12* | CYclophyliN |
|  |  |  |  |  |  |  |  |  |  |  |  |  |  |  |  |  |  |  |  |  |  |  |  |  |  |  |  |  |  |  |  |  |  |  |  |  |  | *rpb-8* | RNA Polymerase II (B) subunit |
|  |  |  |  |  |  |  |  |  |  |  |  |  |  |  |  |  |  |  |  |  |  |  |  |  |  |  |  |  |  |  |  |  |  |  |  |  |  | C11D2.7 |  |
|  |  |  |  |  |  |  |  |  |  |  |  |  |  |  |  |  |  |  |  |  |  |  |  |  |  |  |  |  |  |  |  |  |  |  |  |  |  | *rbx-1* | yeast RBX (ring finger protein) homolog |
|  |  |  |  |  |  |  |  |  |  |  |  |  |  |  |  |  |  |  |  |  |  |  |  |  |  |  |  |  |  |  |  |  |  |  |  |  |  | *rnp-4* | RNP (RRM RNA binding domain) containing |
|  |  |  |  |  |  |  |  |  |  |  |  |  |  |  |  |  |  |  |  |  |  |  |  |  |  |  |  |  |  |  |  |  |  |  |  |  |  | *lsm-5* | LSM Sm-like protein |
|  |  |  |  |  |  |  |  |  |  |  |  |  |  |  |  |  |  |  |  |  |  |  |  |  |  |  |  |  |  |  |  |  |  |  |  |  |  | *mag-1* | Drosophila MAGonashi homolog |
|  |  |  |  |  |  |  |  |  |  |  |  |  |  |  |  |  |  |  |  |  |  |  |  |  |  |  |  |  |  |  |  |  |  |  |  |  |  | *snr-6* | Small Nuclear Ribonucleoprotein |
|  |  |  |  |  |  |  |  |  |  |  |  |  |  |  |  |  |  |  |  |  |  |  |  |  |  |  |  |  |  |  |  |  |  |  |  |  |  | C33A12.1 |  |
|  |  |  |  |  |  |  |  |  |  |  |  |  |  |  |  |  |  |  |  |  |  |  |  |  |  |  |  |  |  |  |  |  |  |  |  |  |  | ZK856.16 |  |
|  |  |  |  |  |  |  |  |  |  |  |  |  |  |  |  |  |  |  |  |  |  |  |  |  |  |  |  |  |  |  |  |  |  |  |  |  |  | F46B6.12 |  |
|  |  |  |  |  |  |  |  |  |  |  |  |  |  |  |  |  |  |  |  |  |  |  |  |  |  |  |  |  |  |  |  |  |  |  |  |  |  | C13A10.5 |  |
|  |  |  |  |  |  |  |  |  |  |  |  |  |  |  |  |  |  |  |  |  |  |  |  |  |  |  |  |  |  |  |  |  |  |  |  |  |  | *mmab-1* | MethylMalonic Aciduria type B homolog |
|  |  |  |  |  |  |  |  |  |  |  |  |  |  |  |  |  |  |  |  |  |  |  |  |  |  |  |  |  |  |  |  |  |  |  |  |  |  | *arl-5* | ARF-Like |
|  |  |  |  |  |  |  |  |  |  |  |  |  |  |  |  |  |  |  |  |  |  |  |  |  |  |  |  |  |  |  |  |  |  |  |  |  |  | F39B2.5 |  |
|  |  |  |  |  |  |  |  |  |  |  |  |  |  |  |  |  |  |  |  |  |  |  |  |  |  |  |  |  |  |  |  |  |  |  |  |  |  | *atg-5* | AuTophaGy (yeast Atg homolog) |
|  |  |  |  |  |  |  |  |  |  |  |  |  |  |  |  |  |  |  |  |  |  |  |  |  |  |  |  |  |  |  |  |  |  |  |  |  |  | *smi-1* | SMN (survival of motor neuron) protein Interactor |
|  |  |  |  |  |  |  |  |  |  |  |  |  |  |  |  |  |  |  |  |  |  |  |  |  |  |  |  |  |  |  |  |  |  |  |  |  |  | *lsm-6* | LSM Sm-like protein |
|  |  |  |  |  |  |  |  |  |  |  |  |  |  |  |  |  |  |  |  |  |  |  |  |  |  |  |  |  |  |  |  |  |  |  |  |  |  | *pfd-2* | PreFolDin (molecular chaperone) |
|  |  |  |  |  |  |  |  |  |  |  |  |  |  |  |  |  |  |  |  |  |  |  |  |  |  |  |  |  |  |  |  |  |  |  |  |  |  | Y66D12A.21 |  |
|  |  |  |  |  |  |  |  |  |  |  |  |  |  |  |  |  |  |  |  |  |  |  |  |  |  |  |  |  |  |  |  |  |  |  |  |  |  | *uev-1* | Ubiquitin E2 (conjugating enzyme) variant |
|  |  |  |  |  |  |  |  |  |  |  |  |  |  |  |  |  |  |  |  |  |  |  |  |  |  |  |  |  |  |  |  |  |  |  |  |  |  | T06D8.7 |  |
|  |  |  |  |  |  |  |  |  |  |  |  |  |  |  |  |  |  |  |  |  |  |  |  |  |  |  |  |  |  |  |  |  |  |  |  |  |  | Y73E7A.1 |  |
|  |  |  |  |  |  |  |  |  |  |  |  |  |  |  |  |  |  |  |  |  |  |  |  |  |  |  |  |  |  |  |  |  |  |  |  |  |  | Y105E8B.6 |  |
|  |  |  |  |  |  |  |  |  |  |  |  |  |  |  |  |  |  |  |  |  |  |  |  |  |  |  |  |  |  |  |  |  |  |  |  |  |  | *art-1* | steroid Alpha ReducTase family |
|  |  |  |  |  |  |  |  |  |  |  |  |  |  |  |  |  |  |  |  |  |  |  |  |  |  |  |  |  |  |  |  |  |  |  |  |  |  | *rab-35* | RAB family |
|  |  |  |  |  |  |  |  |  |  |  |  |  |  |  |  |  |  |  |  |  |  |  |  |  |  |  |  |  |  |  |  |  |  |  |  |  |  | F45G2.10 |  |
|  |  |  |  |  |  |  |  |  |  |  |  |  |  |  |  |  |  |  |  |  |  |  |  |  |  |  |  |  |  |  |  |  |  |  |  |  |  | C08B6.8 |  |
|  |  |  |  |  |  |  |  |  |  |  |  |  |  |  |  |  |  |  |  |  |  |  |  |  |  |  |  |  |  |  |  |  |  |  |  |  |  | ZK856.11 |  |
|  |  |  |  |  |  |  |  |  |  |  |  |  |  |  |  |  |  |  |  |  |  |  |  |  |  |  |  |  |  |  |  |  |  |  |  |  |  | *snpc-3.3* | SNAPc (Small Nuclear RNA Activating Complex) homolog |
|  |  |  |  |  |  |  |  |  |  |  |  |  |  |  |  |  |  |  |  |  |  |  |  |  |  |  |  |  |  |  |  |  |  |  |  |  |  | E02H1.6 |  |
|  |  |  |  |  |  |  |  |  |  |  |  |  |  |  |  |  |  |  |  |  |  |  |  |  |  |  |  |  |  |  |  |  |  |  |  |  |  | F58B3.6 |  |
|  |  |  |  |  |  |  |  |  |  |  |  |  |  |  |  |  |  |  |  |  |  |  |  |  |  |  |  |  |  |  |  |  |  |  |  |  |  | *tut-2* | Thiolation of Uridine in TRNA |
|  |  |  |  |  |  |  |  |  |  |  |  |  |  |  |  |  |  |  |  |  |  |  |  |  |  |  |  |  |  |  |  |  |  |  |  |  |  | *suds-3* | SUDS (vertebrate SUppressor of Defective Silencing) homolog |
|  |  |  |  |  |  |  |  |  |  |  |  |  |  |  |  |  |  |  |  |  |  |  |  |  |  |  |  |  |  |  |  |  |  |  |  |  |  | *rpb-4* | RNA Polymerase II (B) subunit |
|  |  |  |  |  |  |  |  |  |  |  |  |  |  |  |  |  |  |  |  |  |  |  |  |  |  |  |  |  |  |  |  |  |  |  |  |  |  | *rnp-3* | RNP (RRM RNA binding domain) containing |
|  |  |  |  |  |  |  |  |  |  |  |  |  |  |  |  |  |  |  |  |  |  |  |  |  |  |  |  |  |  |  |  |  |  |  |  |  |  | F58A4.6 |  |
|  |  |  |  |  |  |  |  |  |  |  |  |  |  |  |  |  |  |  |  |  |  |  |  |  |  |  |  |  |  |  |  |  |  |  |  |  |  | F23F1.10 |  |
|  |  |  |  |  |  |  |  |  |  |  |  |  |  |  |  |  |  |  |  |  |  |  |  |  |  |  |  |  |  |  |  |  |  |  |  |  |  | *cyn-10* | CYclophyliN |
|  |  |  |  |  |  |  |  |  |  |  |  |  |  |  |  |  |  |  |  |  |  |  |  |  |  |  |  |  |  |  |  |  |  |  |  |  |  | C07A9.2 |  |
|  |  |  |  |  |  |  |  |  |  |  |  |  |  |  |  |  |  |  |  |  |  |  |  |  |  |  |  |  |  |  |  |  |  |  |  |  |  | C02B10.4 |  |
|  |  |  |  |  |  |  |  |  |  |  |  |  |  |  |  |  |  |  |  |  |  |  |  |  |  |  |  |  |  |  |  |  |  |  |  |  |  | *frg-1* | FRG1 (FSH muscular dystrophy Region Gene 1) homolog |
|  |  |  |  |  |  |  |  |  |  |  |  |  |  |  |  |  |  |  |  |  |  |  |  |  |  |  |  |  |  |  |  |  |  |  |  |  |  | *mdt-6* | MeDiaTor |
|  |  |  |  |  |  |  |  |  |  |  |  |  |  |  |  |  |  |  |  |  |  |  |  |  |  |  |  |  |  |  |  |  |  |  |  |  |  | C16C10.4 |  |
|  |  |  |  |  |  |  |  |  |  |  |  |  |  |  |  |  |  |  |  |  |  |  |  |  |  |  |  |  |  |  |  |  |  |  |  |  |  | F08B4.7 |  |
|  |  |  |  |  |  |  |  |  |  |  |  |  |  |  |  |  |  |  |  |  |  |  |  |  |  |  |  |  |  |  |  |  |  |  |  |  |  | *snr-3* | Small Nuclear Ribonucleoprotein |
|  |  |  |  |  |  |  |  |  |  |  |  |  |  |  |  |  |  |  |  |  |  |  |  |  |  |  |  |  |  |  |  |  |  |  |  |  |  | F28F8.5 |  |
|  |  |  |  |  |  |  |  |  |  |  |  |  |  |  |  |  |  |  |  |  |  |  |  |  |  |  |  |  |  |  |  |  |  |  |  |  |  | F33E2.5 |  |
|  |  |  |  |  |  |  |  |  |  |  |  |  |  |  |  |  |  |  |  |  |  |  |  |  |  |  |  |  |  |  |  |  |  |  |  |  |  | *lsy-13* | Laterally SYmmetric (defective in lateral asymmetry) |
|  |  |  |  |  |  |  |  |  |  |  |  |  |  |  |  |  |  |  |  |  |  |  |  |  |  |  |  |  |  |  |  |  |  |  |  |  |  | F19B10.1 |  |
|  |  |  |  |  |  |  |  |  |  |  |  |  |  |  |  |  |  |  |  |  |  |  |  |  |  |  |  |  |  |  |  |  |  |  |  |  |  | Y66A7A.2 |  |
|  |  |  |  |  |  |  |  |  |  |  |  |  |  |  |  |  |  |  |  |  |  |  |  |  |  |  |  |  |  |  |  |  |  |  |  |  |  | *rnp-2* | RNP (RRM RNA binding domain) containing |

### Phenotypes enriched

|  |  |  |  |
| --- | --- | --- | --- |
| **Group name** | **Number in cluster** | **Enrichment** | **FDR corrected p** |
| cell proliferation increased (RNAi) | 14 | 18.56 | 1.60e-10 |
| excess intestinal cells (RNAi) | 14 | 18.36 | 1.82e-10 |
| cell proliferation variant (RNAi) | 14 | 17.78 | 2.86e-10 |
| intestinal development variant (RNAi) | 14 | 11.41 | 8.92e-08 |
| alimentary system development variant (RNAi) | 14 | 7.93 | 7.90e-06 |
| organ system development variant (RNAi) | 20 | 2.88 | 2.17e-02 |
| P granule localization defective (RNAi) | 4 | 20.11 | 4.37e-02 |

### Anatomy terms enriched

none found

### GO terms enriched

|  |  |  |
| --- | --- | --- |
| **GO term** | **Number of genes** | **FDR-corrected p-value** |
| embryo development ending in birth or egg hatching | 43 | 2.5e-05 |
| spliceosomal complex | 5 | 5.6e-05 |
| RNA splicing, via transesterification reactions | 5 | 1.7e-03 |
| germ cell development | 9 | 2.0e-03 |
| RNA binding | 9 | 3.3e-03 |
| mRNA processing | 5 | 3.5e-03 |
| nucleus | 23 | 5.6e-03 |
| RNA splicing | 4 | 5.8e-03 |
| nucleolus | 5 | 9.7e-03 |
| negative regulation of vulval development | 8 | 1.5e-02 |
| negative regulation of post-embryonic development | 8 | 1.6e-02 |
| cellular process involved in reproduction | 12 | 1.9e-02 |
| cell | 36 | 2.0e-02 |
| multicellular organismal process | 40 | 2.4e-02 |
| receptor-mediated endocytosis | 15 | 2.5e-02 |
| macromolecular complex | 17 | 3.1e-02 |
| vesicle-mediated transport | 17 | 4.2e-02 |
| nucleotidyltransferase activity | 4 | 5.0e-02 |

### Expression clusters enriched

|  |  |  |  |
| --- | --- | --- | --- |
| **Group name** | **Number in cluster** | **Enrichment** | **FDR corrected p** |
| Caenorhabditis elegans Genes with expression levels changed significantly after treatment of Bacillus thurigiensis DB27. | 87 | 2.19 | 1.39e-14 |
| TGF- Dauer pathway adult transcriptional targets. Results obtained by comparing the microarray results of the dauer-constitutive mutants daf-7(e1372), daf-7(m62), and daf-1(m40) with dauer-defective mutants daf-3(mgDf90), daf-5(e1386), and daf-7(e1372);daf-3(mgDf90) double mutants at the permissive temperature, 20C, on the first day of adulthood. WBPaper00031040:TGF-beta\_adult\_downregulated | 80 | 2.30 | 9.59e-14 |
| Caenorhabditis elegans Genes with expression levels changed significantly after treatment of Xenorhabdus nematophila. | 104 | 1.80 | 1.01e-13 |
| Maternal class (M): genes that are called present in at least one of the three PC6 replicates. | 91 | 1.88 | 2.32e-11 |
| Genes expressed in embryonic motor neurons (identified by unc-4::GFP expressing cells). | 91 | 1.81 | 2.86e-10 |
| Maternal-embryonic class (ME): genes that are in the intersection of the maternal and embryonic classes. | 53 | 2.42 | 6.00e-08 |
| Maternal degradation class (MD): genes that are the subset of maternal genes that decrease without first increasing in abundance. | 38 | 2.63 | 6.92e-06 |
| Genes with no change in hcf-1(-), no change in sir-2.1(O/E) and downregulated in daf-2(-). | 23 | 3.83 | 1.29e-05 |
| Genes that showed expression levels higher than the corresponding reference sample (L2 all cell reference). WBPaper00037950:excretory-cell\_expressed | 76 | 1.68 | 1.30e-05 |
| Genes enriched in intestine. | 40 | 2.41 | 2.60e-05 |
| oogenesis-enriched | 26 | 2.97 | 1.85e-04 |
| FBF-associated probe sets (FDR <2.25%) | 57 | 1.82 | 2.06e-04 |
| Genes that showed expression levels higher than the corresponding reference sample (embryonic 0hr reference). WBPaper00037950:BAG-neuron\_expressed | 65 | 1.71 | 2.11e-04 |
| Genes in the top 10% of expression level across the triplicate L3 samples. To generate the top10 and bottom10 gene sets, authors ranked all genes by mean expression array signal intensity across the three replicates, then took the top and bottom deciles (1,841 genes each) to represent genes with high and low expression. | 36 | 2.36 | 2.15e-04 |
| Differentially expressed genes during worm lifespan. Medoid 7 Fig.4. | 14 | 5.09 | 2.62e-04 |
| Maternal degradation (MD) subclasses are based on the earliest significant decrease (abbreviated pd for primary decrease). [cgc5767]:expression\_class\_MD\_pd(23\_min) | 17 | 3.77 | 9.15e-04 |
| Embryonic class (E): genes that significantly increase in abundance at some point during embryogenesis. | 53 | 1.79 | 1.07e-03 |
| Genes significantly enriched (> 2x, FDR < 5%) in a particular cell-type versus a reference sample of all cells at the same stage. WBPaper00037950:hypodermis\_larva\_enriched | 27 | 2.60 | 1.16e-03 |
| Maternal degradation-embryonic class (MDE): genes that are the subset of maternal degradation genes that significantly increase in at least two of the eight total paired timepoint tests in the induction-following-degradation time domain. | 18 | 3.40 | 1.79e-03 |
| Genes significantly enriched (> 2x, FDR < 5%) in a particular cell-type versus a reference sample of all cells at the same stage. WBPaper00037950:bodywall-muscle\_larva\_enriched | 25 | 2.62 | 2.28e-03 |
| Expression Pattern Group F, enriched for genes involved in embryonic development. These patterns have in common that they all have genes of which the expression goes up after the juvenile stage. The expression of the genes in these patterns remains high or even goes up after reproduction. | 38 | 2.03 | 2.82e-03 |
| Genes up or down regulated by 10e-05M of progesterone. The normalized values used were G/R ratio > 2.6 for up-regulation and G/R ratio < 0.38 for down-regulation, which corresponds to 1.39 and -1.39 log(base2) G/R ratio, respectively. | 38 | 1.91 | 1.01e-02 |
| The cluster contains genes that are significantly enriched in L1 muscle. | 27 | 2.25 | 1.19e-02 |
| Genes up or down regulated by 10e-09M of cholesterol . The normalized values used were G/R ratio > 2.6 for up-regulation and G/R ratio < 0.38 for down-regulation, which corresponds to 1.39 and -1.39 log(base2) G/R ratio, respectively. | 31 | 2.09 | 1.24e-02 |
| Genes that showed expression levels higher than the corresponding reference sample (L3/L4 all cell reference). | 72 | 1.45 | 1.37e-02 |
| Genes significantly enriched (> 2x, FDR < 5%) in a particular cell-type versus a reference sample of all cells at the same stage. WBPaper00037950:PVD-OLL-neurons\_larva\_enriched | 19 | 2.61 | 2.65e-02 |
| Genome-wide analysis of developmental and sex-regulated gene expression profile. cgc4489\_group\_2 | 27 | 2.10 | 3.39e-02 |

### Motifs enriched

|  |  |  |  |  |  |
| --- | --- | --- | --- | --- | --- |
| **Motif** | **Logo** | **Possible orthologs** | **Number of motifs in cluster** | **Enrichment** | **FDR corrected p** |
| pTH7876 |  | mel-28 fkh-7 lin-29 | 98 | 1.37 | 0.00035 |
| pTH9335 |  | mel-28 | 93 | 1.38 | 0.00068 |
| pTH9180 |  | mel-28 Y116A8C.22 Y61A9LA.9 | 88 | 1.41 | 0.00092 |
| pTH5656 |  | fkh-7 fkh-10 let-381 daf-16 | 86 | 1.41 | 0.00150 |
| pTH9116 |  | fkh-7 lin-31 let-381 daf-16 | 89 | 1.37 | 0.00210 |
| FLI1\_4 |  | lin-1 | 33 | 2.15 | 0.00230 |
| HepG2\_FOXA2\_HudsonAlpha |  | lin-31 let-381 | 77 | 1.45 | 0.00240 |
| pTH9097 |  | Y116A8C.22 | 101 | 1.28 | 0.00330 |
| pTH9380 |  | mel-28 | 99 | 1.29 | 0.00410 |
| pTH9958 |  | ztf-6 | 85 | 1.37 | 0.00410 |
| pTH8916 |  | Y116A8C.22 | 87 | 1.36 | 0.00490 |
| pTH2673 |  | fkh-10 let-381 | 53 | 1.65 | 0.00520 |
| ONECUT3\_1 |  | dsc-1 ceh-48 | 93 | 1.32 | 0.00540 |
| pnt\_SANGER\_5\_FBgn0003118 |  | lin-1 | 77 | 1.42 | 0.00570 |
| MA0497.1 |  | mef-2 (-0.63) | 91 | 1.31 | 0.00920 |
| pTH9393 |  | ZC416.1 | 38 | 1.81 | 0.01300 |
| MA0537.1 |  | blmp-1 | 97 | 1.26 | 0.01600 |
| V$POU3F2\_01 |  | ceh-18 (-0.66) | 87 | 1.31 | 0.01800 |
| sv\_SOLEXA\_5\_FBgn0005561 |  | pax-2 | 37 | 1.76 | 0.02300 |
| pTH9262 |  | lin-54 | 85 | 1.30 | 0.02700 |
| pTH9951 |  | mex-6 | 89 | 1.28 | 0.02800 |
| Barhl1\_3 |  | ceh-31 | 73 | 1.37 | 0.02800 |
| pTH1294 |  | mel-28 | 47 | 1.58 | 0.03000 |
| Abd-A\_FlyReg\_FBgn0000014 |  | lin-39 | 73 | 1.36 | 0.03200 |
| pTH10797 |  | K11D2.4 | 98 | 1.23 | 0.03500 |
| pTH5260 |  | lin-22 | 11 | 3.28 | 0.03800 |
| Hbn\_Cell\_FBgn0008636 |  | alr-1 lim-6 | 34 | 1.76 | 0.03800 |
| pTH10798 |  | Y75B8A.6 | 32 | 1.80 | 0.03900 |
| V$FREAC7\_01 |  | lin-31 | 51 | 1.51 | 0.04100 |
| pTH9260 |  | mel-28 | 91 | 1.25 | 0.04800 |
| pTH9242 |  | mel-28 | 94 | 1.23 | 0.04800 |
| pTH8982 |  | ceh-48 | 20 | 2.18 | 0.04900 |
| pTH5169 |  | cfi-1 | 86 | 1.27 | 0.05000 |

### Correlated (and anti-correlated) transcription factors

|  |  |
| --- | --- |
| **Transcription factor** | **Correlation** |
| C01F6.9 | 0.81 |
| mxl-1 | 0.80 |
| C16A3.4 | 0.80 |
| repo-1 | 0.76 |
| hmg-11 | 0.73 |
| D2030.7 | 0.73 |
| nfyc-1 | 0.73 |
| R144.3 | 0.72 |
| cebp-2 | 0.71 |
| hmg-5 | 0.71 |
| dhhc-1 | 0.71 |
| ztf-4 | 0.70 |
| lst-5 | 0.70 |
| F37B4.10 | 0.69 |
| ceh-13 | 0.65 |
| Y56A3A.18 | 0.62 |
| hmg-6 | 0.62 |
| zip-8 | 0.61 |
| zip-7 | 0.59 |
| dhhc-10 | 0.57 |
| hmg-12 | 0.57 |
| K11D12.12 | 0.56 |
| sptf-1 | 0.56 |
| ces-1 | 0.56 |
| lir-3 | 0.56 |
| nhr-125 | -0.65 |
| nhr-58 | -0.66 |
| med-2 | -0.66 |
| tag-97 | -0.66 |
| lin-48 | -0.66 |
| ceh-18 | -0.66 |
| madf-4 | -0.67 |
| ZC328.2 | -0.67 |
| saeg-1 | -0.67 |
| nhr-46 | -0.68 |
| nhr-214 | -0.68 |
| nhr-40 | -0.69 |
| nhr-4 | -0.69 |
| fkh-9 | -0.71 |
| nhr-71 | -0.71 |
| F10B5.3 | -0.71 |
| zip-1 | -0.72 |
| nhr-20 | -0.73 |
| F26A10.2 | -0.73 |
| daf-12 | -0.73 |
| F13H6.1 | -0.74 |
| nhr-49 | -0.75 |
| fos-1 | -0.76 |
| nhr-3 | -0.76 |
| nhr-1 | -0.80 |

### ChIP peaks enriched

|  |  |  |  |  |
| --- | --- | --- | --- | --- |
| **Gene** | **Experiment** | **Number of upstream peaks** | **Enrichment** | **FDR corrected p** |
| efl-1 | EFL-1\_Fed-L1-stage-larvae | 65 | 2.98 | 7.2e-16 |
| efl-1 | EFL-1\_Young-adult | 72 | 2.64 | 2.1e-15 |
| dpl-1 | DPL-1\_Fed-L1-stage-larvae | 63 | 2.83 | 3.5e-14 |
| efl-1 | EFL-1\_Larvae-L1-stage | 67 | 2.67 | 3.9e-14 |
| F16B12.6 | F16B12.6\_Fed-L1-stage-larvae | 41 | 3.98 | 9.8e-13 |
| dpl-1 | DPL-1\_Larvae-L4-stage | 74 | 2.23 | 5.1e-12 |
| dpl-1 | DPL-1\_Young-adult | 57 | 2.69 | 1.7e-11 |
| lin-15 | LIN-15B\_Fed-L1-stage-larvae | 43 | 3.43 | 2.6e-11 |
| pes-1 | PES-1\_Larvae-L4-stage | 60 | 2.37 | 6.4e-10 |
| nhr-77 | NHR-77\_Fed-L1-stage-larvae | 57 | 2.45 | 8.0e-10 |
| lsy-2 | LSY-2\_Fed-L1-stage-larvae | 57 | 2.44 | 8.9e-10 |
| aly-2 | ALY-2\_Fed-L1-stage-larvae | 48 | 2.76 | 1.4e-09 |
| eor-1 | EOR-1\_Larvae-L3-stage | 60 | 2.29 | 2.4e-09 |
| lsy-2 | LSY-2\_Embryos | 47 | 2.76 | 2.4e-09 |
| lin-13 | LIN-13\_Larvae-L2-stage | 46 | 2.77 | 4.0e-09 |
| lin-35 | LIN-35\_Fed-L1-stage-larvae | 53 | 2.47 | 4.4e-09 |
| R02D3.7 | R02D3.7\_Larvae-L3-stage | 59 | 2.26 | 6.9e-09 |
| C34F6.9 | C34F6.9\_Larvae-L2-stage | 57 | 2.24 | 2.6e-08 |
| W03F9.2 | W03F9.2\_L4-Young-Adult-stage-larvae | 75 | 1.86 | 3.0e-08 |
| C16A3.4 | C16A3.4\_Fed-L1-stage-larvae | 45 | 2.61 | 4.3e-08 |
| hpl-2 | HPL-2\_Fed-L1-stage-larvae | 63 | 2.06 | 4.7e-08 |
| F45C12.2 | F45C12.2\_Fed-L1-stage-larvae | 49 | 2.35 | 1.8e-07 |
| lsy-2 | LSY-2\_Larvae-L2-stage | 32 | 3.20 | 2.3e-07 |
| lsy-2 | LSY-2\_Larvae-L1-stage | 61 | 2.01 | 2.8e-07 |
| gei-11 | GEI-11\_Larvae-L2-stage | 44 | 2.47 | 3.7e-07 |
| nfya-1 | NFYA-1\_Late-Embryos | 50 | 2.26 | 4.0e-07 |
| nfya-1 | NFYA-1\_Larvae-L3-stage | 46 | 2.38 | 4.3e-07 |
| nhr-23 | NHR-23\_Larvae-L3-stage | 49 | 2.24 | 7.4e-07 |
| gei-11 | GEI-11\_Fed-L1-stage-larvae | 50 | 2.14 | 2.2e-06 |
| C01B12.2 | C01B12.2\_Larvae-L2-stage | 63 | 1.85 | 3.3e-06 |
| ceh-39 | CEH-39\_Embryos | 35 | 2.66 | 3.5e-06 |
| ceh-38 | CEH-38\_Larvae-L3-stage | 47 | 2.18 | 3.8e-06 |
| gei-11 | GEI-11\_Larvae-L3-stage | 50 | 2.08 | 5.5e-06 |
| R02D3.7 | R02D3.7\_Larvae-L2-stage | 32 | 2.67 | 1.3e-05 |
| fos-1 | FOS-1\_Fed-L1-stage-larvae | 47 | 2.08 | 1.6e-05 |
| F23B12.7 | F23B12.7\_Young-adult | 35 | 2.48 | 1.7e-05 |
| sem-4 | SEM-4\_Larvae-L2-stage | 49 | 1.99 | 2.8e-05 |
| R02D3.7 | R02D3.7\_Larvae-L4-stage | 27 | 2.71 | 9.9e-05 |
| nhr-237 | NHR-237\_Embryos | 26 | 2.72 | 1.5e-04 |
| nhr-6 | NHR-6\_Larvae-L4-stage | 31 | 2.35 | 2.6e-04 |
| jun-1 | JUN-1\_Larvae-L1-stage | 38 | 2.08 | 3.2e-04 |
| ham-1 | HAM-1\_Fed-L1-stage-larvae | 46 | 1.86 | 4.1e-04 |
| nhr-77 | NHR-77\_Larvae-L4-stage | 60 | 1.62 | 6.5e-04 |
| dve-1 | DVE-1\_Late-Embryos | 39 | 1.98 | 6.5e-04 |
| ham-1 | HAM-1\_Larvae-L4-stage | 47 | 1.77 | 1.1e-03 |
| pha-4 | PHA-4\_Larvae-L2-stage | 49 | 1.73 | 1.3e-03 |
| nhr-129 | NHR-129\_Larvae-L2-stage | 56 | 1.63 | 1.4e-03 |
| alr-1 | ALR-1\_Larvae-L2-stage | 40 | 1.88 | 1.5e-03 |
| aly-2 | ALY-2\_Larvae-L3-stage | 25 | 2.31 | 2.7e-03 |
| lsy-2 | LSY-2\_Larvae-L4-stage | 22 | 2.48 | 2.9e-03 |
| ces-1 | CES-1\_Embryos | 44 | 1.75 | 3.1e-03 |
| lin-13 | LIN-13\_Larvae-L4-stage | 27 | 2.15 | 4.3e-03 |
| pha-4 | PHA-4\_Larvae-L4-stage | 30 | 1.97 | 7.8e-03 |
| jun-1 | JUN-1\_Larvae-L4-stage | 29 | 1.97 | 1.0e-02 |
| nhr-77 | NHR-77\_Larvae-L3-stage | 31 | 1.87 | 1.4e-02 |
| zag-1 | ZAG-1\_Fed-L1-stage-larvae | 16 | 2.63 | 1.4e-02 |
| skn-1 | SKN-1\_Larvae-L3-stage | 21 | 2.24 | 1.5e-02 |
| mab-5 | MAB-5\_Larvae-L2-stage | 21 | 2.22 | 1.7e-02 |
| nhr-25 | NHR-25\_Larvae-L2-stage | 35 | 1.75 | 1.8e-02 |
| F45C12.2 | F45C12.2\_Larvae-L2-stage | 17 | 2.46 | 1.9e-02 |
| nhr-6 | NHR-6\_Larvae-L2-stage | 38 | 1.68 | 2.1e-02 |
| lin-35 | LIN-35\_Young-adult | 22 | 2.12 | 2.1e-02 |
| ceh-38 | CEH-38\_Larvae-L4-stage | 23 | 2.00 | 3.3e-02 |
| lin-15 | LIN-15B\_Larvae-L4-stage | 14 | 2.53 | 4.1e-02 |
