# Supplementary material for: Overlapping cell population expression profiling and regulatory inference in C. elegans
Source: BMC Genomics. 2016 Feb 29;17:159. doi: 10.1186/s12864-016-2482-z (PMC4772325; doi:10.1186/s12864-016-2482-z)
Supplement: Additional file 13: — Web supplement. (DOC 21 kb) [file 12864_2016_2482_MOESM13_ESM.zip › sortWeb/clusters/hier.300.clusters/174.html]

Cluster 174 

## Cluster 174

### Expression

| cnd-1 rep. 1 | cnd-1 rep. 2 | cnd-1 rep. 3 | pha-4 rep. 1 | pha-4 rep. 2 | pha-4 rep. 3 | ceh-27 | ceh-36 | ceh-6 | F21D5.9 | mir-57 | mls-2 | pal-1 | pros-1 | ttx-3 | unc-130 | hlh-16 | irx-1 | ceh-6 (+) hlh-16 (+) | ceh-6 (+) hlh-16 (-) | ceh-6 (-) hlh-16 (+) | cnd-1 singlets | pha-4 singlets | 0 | 60 | 120 | 150 | 180 | 240 | 330 | 390 | 420 | 480 | 540 | 570 | 600 | 630 | 660 | NAME | Functional description |
| --- | --- | --- | --- | --- | --- | --- | --- | --- | --- | --- | --- | --- | --- | --- | --- | --- | --- | --- | --- | --- | --- | --- | --- | --- | --- | --- | --- | --- | --- | --- | --- | --- | --- | --- | --- | --- | --- | --- | --- |
|  |  |  |  |  |  |  |  |  |  |  |  |  |  |  |  |  |  |  |  |  |  |  |  |  |  |  |  |  |  |  |  |  |  |  |  |  |  | *mrpl-32* | Mitochondrial Ribosomal Protein, Large |
|  |  |  |  |  |  |  |  |  |  |  |  |  |  |  |  |  |  |  |  |  |  |  |  |  |  |  |  |  |  |  |  |  |  |  |  |  |  | R02D5.8 |  |
|  |  |  |  |  |  |  |  |  |  |  |  |  |  |  |  |  |  |  |  |  |  |  |  |  |  |  |  |  |  |  |  |  |  |  |  |  |  | *coq-5* | COenzyme Q (ubiquinone) biosynthesis |
|  |  |  |  |  |  |  |  |  |  |  |  |  |  |  |  |  |  |  |  |  |  |  |  |  |  |  |  |  |  |  |  |  |  |  |  |  |  | *sdhb-1* | Succinate DeHydrogenase complex subunit B |
|  |  |  |  |  |  |  |  |  |  |  |  |  |  |  |  |  |  |  |  |  |  |  |  |  |  |  |  |  |  |  |  |  |  |  |  |  |  | R144.13 |  |
|  |  |  |  |  |  |  |  |  |  |  |  |  |  |  |  |  |  |  |  |  |  |  |  |  |  |  |  |  |  |  |  |  |  |  |  |  |  | F44G4.3 |  |
|  |  |  |  |  |  |  |  |  |  |  |  |  |  |  |  |  |  |  |  |  |  |  |  |  |  |  |  |  |  |  |  |  |  |  |  |  |  | *mrps-34* | Mitochondrial Ribosomal Protein, Small |
|  |  |  |  |  |  |  |  |  |  |  |  |  |  |  |  |  |  |  |  |  |  |  |  |  |  |  |  |  |  |  |  |  |  |  |  |  |  | *mrpl-47* | Mitochondrial Ribosomal Protein, Large |
|  |  |  |  |  |  |  |  |  |  |  |  |  |  |  |  |  |  |  |  |  |  |  |  |  |  |  |  |  |  |  |  |  |  |  |  |  |  | F59C6.5 |  |
|  |  |  |  |  |  |  |  |  |  |  |  |  |  |  |  |  |  |  |  |  |  |  |  |  |  |  |  |  |  |  |  |  |  |  |  |  |  | *glrx-5* | GLutaRedoXin |
|  |  |  |  |  |  |  |  |  |  |  |  |  |  |  |  |  |  |  |  |  |  |  |  |  |  |  |  |  |  |  |  |  |  |  |  |  |  | *mrps-11* | Mitochondrial Ribosomal Protein, Small |
|  |  |  |  |  |  |  |  |  |  |  |  |  |  |  |  |  |  |  |  |  |  |  |  |  |  |  |  |  |  |  |  |  |  |  |  |  |  | Y56A3A.19 |  |
|  |  |  |  |  |  |  |  |  |  |  |  |  |  |  |  |  |  |  |  |  |  |  |  |  |  |  |  |  |  |  |  |  |  |  |  |  |  | F28B3.10 |  |
|  |  |  |  |  |  |  |  |  |  |  |  |  |  |  |  |  |  |  |  |  |  |  |  |  |  |  |  |  |  |  |  |  |  |  |  |  |  | *atp-5* | ATP synthase subunit |
|  |  |  |  |  |  |  |  |  |  |  |  |  |  |  |  |  |  |  |  |  |  |  |  |  |  |  |  |  |  |  |  |  |  |  |  |  |  | *nduf-7* | NADH Ubiquinone oxidoreductase Fe-S protein |
|  |  |  |  |  |  |  |  |  |  |  |  |  |  |  |  |  |  |  |  |  |  |  |  |  |  |  |  |  |  |  |  |  |  |  |  |  |  | *chch-3* | Coiled coil Helix Coiled coiled Helix domain |
|  |  |  |  |  |  |  |  |  |  |  |  |  |  |  |  |  |  |  |  |  |  |  |  |  |  |  |  |  |  |  |  |  |  |  |  |  |  | *mrps-33* | Mitochondrial Ribosomal Protein, Small |
|  |  |  |  |  |  |  |  |  |  |  |  |  |  |  |  |  |  |  |  |  |  |  |  |  |  |  |  |  |  |  |  |  |  |  |  |  |  | R186.8 |  |
|  |  |  |  |  |  |  |  |  |  |  |  |  |  |  |  |  |  |  |  |  |  |  |  |  |  |  |  |  |  |  |  |  |  |  |  |  |  | T19H5.4 |  |
|  |  |  |  |  |  |  |  |  |  |  |  |  |  |  |  |  |  |  |  |  |  |  |  |  |  |  |  |  |  |  |  |  |  |  |  |  |  | C43H6.10 |  |
|  |  |  |  |  |  |  |  |  |  |  |  |  |  |  |  |  |  |  |  |  |  |  |  |  |  |  |  |  |  |  |  |  |  |  |  |  |  | *mrpl-19* | Mitochondrial Ribosomal Protein, Large |
|  |  |  |  |  |  |  |  |  |  |  |  |  |  |  |  |  |  |  |  |  |  |  |  |  |  |  |  |  |  |  |  |  |  |  |  |  |  | R05D7.4 |  |
|  |  |  |  |  |  |  |  |  |  |  |  |  |  |  |  |  |  |  |  |  |  |  |  |  |  |  |  |  |  |  |  |  |  |  |  |  |  | *dpm-1* | Dolichol Phosphate Mannosyltransferase |
|  |  |  |  |  |  |  |  |  |  |  |  |  |  |  |  |  |  |  |  |  |  |  |  |  |  |  |  |  |  |  |  |  |  |  |  |  |  | Y61A9LA.11 |  |
|  |  |  |  |  |  |  |  |  |  |  |  |  |  |  |  |  |  |  |  |  |  |  |  |  |  |  |  |  |  |  |  |  |  |  |  |  |  | *dylt-1* | DYnein Light chain (Tctex type) |
|  |  |  |  |  |  |  |  |  |  |  |  |  |  |  |  |  |  |  |  |  |  |  |  |  |  |  |  |  |  |  |  |  |  |  |  |  |  | *frh-1* | FRataxin (involved in human Friedrich's ataxia) Homolog |
|  |  |  |  |  |  |  |  |  |  |  |  |  |  |  |  |  |  |  |  |  |  |  |  |  |  |  |  |  |  |  |  |  |  |  |  |  |  | C35D10.5 |  |
|  |  |  |  |  |  |  |  |  |  |  |  |  |  |  |  |  |  |  |  |  |  |  |  |  |  |  |  |  |  |  |  |  |  |  |  |  |  | F31C3.12 |  |
|  |  |  |  |  |  |  |  |  |  |  |  |  |  |  |  |  |  |  |  |  |  |  |  |  |  |  |  |  |  |  |  |  |  |  |  |  |  | F31C3.16 |  |
|  |  |  |  |  |  |  |  |  |  |  |  |  |  |  |  |  |  |  |  |  |  |  |  |  |  |  |  |  |  |  |  |  |  |  |  |  |  | *cblc-1* | CoBaLamin deficiency, C complementation group (human) homolog |
|  |  |  |  |  |  |  |  |  |  |  |  |  |  |  |  |  |  |  |  |  |  |  |  |  |  |  |  |  |  |  |  |  |  |  |  |  |  | C01G10.7 |  |
|  |  |  |  |  |  |  |  |  |  |  |  |  |  |  |  |  |  |  |  |  |  |  |  |  |  |  |  |  |  |  |  |  |  |  |  |  |  | *glod-4* | GLyOxalase Domain containing |
|  |  |  |  |  |  |  |  |  |  |  |  |  |  |  |  |  |  |  |  |  |  |  |  |  |  |  |  |  |  |  |  |  |  |  |  |  |  | *mrpl-50* | Mitochondrial Ribosomal Protein, Large |
|  |  |  |  |  |  |  |  |  |  |  |  |  |  |  |  |  |  |  |  |  |  |  |  |  |  |  |  |  |  |  |  |  |  |  |  |  |  | C01G10.8 |  |
|  |  |  |  |  |  |  |  |  |  |  |  |  |  |  |  |  |  |  |  |  |  |  |  |  |  |  |  |  |  |  |  |  |  |  |  |  |  | K07C5.2 |  |
|  |  |  |  |  |  |  |  |  |  |  |  |  |  |  |  |  |  |  |  |  |  |  |  |  |  |  |  |  |  |  |  |  |  |  |  |  |  | F40F4.7 |  |
|  |  |  |  |  |  |  |  |  |  |  |  |  |  |  |  |  |  |  |  |  |  |  |  |  |  |  |  |  |  |  |  |  |  |  |  |  |  | F35H12.5 |  |
|  |  |  |  |  |  |  |  |  |  |  |  |  |  |  |  |  |  |  |  |  |  |  |  |  |  |  |  |  |  |  |  |  |  |  |  |  |  | ZK563.7 |  |
|  |  |  |  |  |  |  |  |  |  |  |  |  |  |  |  |  |  |  |  |  |  |  |  |  |  |  |  |  |  |  |  |  |  |  |  |  |  | Y43F8B.6 |  |
|  |  |  |  |  |  |  |  |  |  |  |  |  |  |  |  |  |  |  |  |  |  |  |  |  |  |  |  |  |  |  |  |  |  |  |  |  |  | F46F6.5 |  |
|  |  |  |  |  |  |  |  |  |  |  |  |  |  |  |  |  |  |  |  |  |  |  |  |  |  |  |  |  |  |  |  |  |  |  |  |  |  | C53D6.4 |  |
|  |  |  |  |  |  |  |  |  |  |  |  |  |  |  |  |  |  |  |  |  |  |  |  |  |  |  |  |  |  |  |  |  |  |  |  |  |  | *tag-115* | Temporarily Assigned Gene name |
|  |  |  |  |  |  |  |  |  |  |  |  |  |  |  |  |  |  |  |  |  |  |  |  |  |  |  |  |  |  |  |  |  |  |  |  |  |  | F01G4.5 |  |
|  |  |  |  |  |  |  |  |  |  |  |  |  |  |  |  |  |  |  |  |  |  |  |  |  |  |  |  |  |  |  |  |  |  |  |  |  |  | *ztf-4* | Zinc finger putative Transcription Factor family |
|  |  |  |  |  |  |  |  |  |  |  |  |  |  |  |  |  |  |  |  |  |  |  |  |  |  |  |  |  |  |  |  |  |  |  |  |  |  | B0336.5 |  |
|  |  |  |  |  |  |  |  |  |  |  |  |  |  |  |  |  |  |  |  |  |  |  |  |  |  |  |  |  |  |  |  |  |  |  |  |  |  | *dhs-22* | DeHydrogenases, Short chain |
|  |  |  |  |  |  |  |  |  |  |  |  |  |  |  |  |  |  |  |  |  |  |  |  |  |  |  |  |  |  |  |  |  |  |  |  |  |  | *ned-8* | mouse NEDd8 related |
|  |  |  |  |  |  |  |  |  |  |  |  |  |  |  |  |  |  |  |  |  |  |  |  |  |  |  |  |  |  |  |  |  |  |  |  |  |  | *set-23* | SET (trithorax/polycomb) domain containing |
|  |  |  |  |  |  |  |  |  |  |  |  |  |  |  |  |  |  |  |  |  |  |  |  |  |  |  |  |  |  |  |  |  |  |  |  |  |  | *dhfr-1* | DiHydroFolate Reductase |
|  |  |  |  |  |  |  |  |  |  |  |  |  |  |  |  |  |  |  |  |  |  |  |  |  |  |  |  |  |  |  |  |  |  |  |  |  |  | E02H1.5 |  |
|  |  |  |  |  |  |  |  |  |  |  |  |  |  |  |  |  |  |  |  |  |  |  |  |  |  |  |  |  |  |  |  |  |  |  |  |  |  | *hap-1* | yeast HAP (HydroxylAminoPurine sensitivity) related |
|  |  |  |  |  |  |  |  |  |  |  |  |  |  |  |  |  |  |  |  |  |  |  |  |  |  |  |  |  |  |  |  |  |  |  |  |  |  | *mdt-18* | MeDiaTor |
|  |  |  |  |  |  |  |  |  |  |  |  |  |  |  |  |  |  |  |  |  |  |  |  |  |  |  |  |  |  |  |  |  |  |  |  |  |  | B0511.7 |  |
|  |  |  |  |  |  |  |  |  |  |  |  |  |  |  |  |  |  |  |  |  |  |  |  |  |  |  |  |  |  |  |  |  |  |  |  |  |  | F27C1.2 |  |
|  |  |  |  |  |  |  |  |  |  |  |  |  |  |  |  |  |  |  |  |  |  |  |  |  |  |  |  |  |  |  |  |  |  |  |  |  |  | *ubc-9* | UBiquitin Conjugating enzyme |
|  |  |  |  |  |  |  |  |  |  |  |  |  |  |  |  |  |  |  |  |  |  |  |  |  |  |  |  |  |  |  |  |  |  |  |  |  |  | *zhit-2* | Zinc finger, HIT-type |
|  |  |  |  |  |  |  |  |  |  |  |  |  |  |  |  |  |  |  |  |  |  |  |  |  |  |  |  |  |  |  |  |  |  |  |  |  |  | *stl-1* | STomatin-Like |
|  |  |  |  |  |  |  |  |  |  |  |  |  |  |  |  |  |  |  |  |  |  |  |  |  |  |  |  |  |  |  |  |  |  |  |  |  |  | *sds-22* | SDS22 (conserved phosphatase regulator) homolog |
|  |  |  |  |  |  |  |  |  |  |  |  |  |  |  |  |  |  |  |  |  |  |  |  |  |  |  |  |  |  |  |  |  |  |  |  |  |  | *syx-7* | SYntaXin |
|  |  |  |  |  |  |  |  |  |  |  |  |  |  |  |  |  |  |  |  |  |  |  |  |  |  |  |  |  |  |  |  |  |  |  |  |  |  | C29E4.12 |  |
|  |  |  |  |  |  |  |  |  |  |  |  |  |  |  |  |  |  |  |  |  |  |  |  |  |  |  |  |  |  |  |  |  |  |  |  |  |  | F33D4.5 |  |
|  |  |  |  |  |  |  |  |  |  |  |  |  |  |  |  |  |  |  |  |  |  |  |  |  |  |  |  |  |  |  |  |  |  |  |  |  |  | M88.7 |  |
|  |  |  |  |  |  |  |  |  |  |  |  |  |  |  |  |  |  |  |  |  |  |  |  |  |  |  |  |  |  |  |  |  |  |  |  |  |  | ZK563.5 |  |
|  |  |  |  |  |  |  |  |  |  |  |  |  |  |  |  |  |  |  |  |  |  |  |  |  |  |  |  |  |  |  |  |  |  |  |  |  |  | Y95D11A.1 |  |
|  |  |  |  |  |  |  |  |  |  |  |  |  |  |  |  |  |  |  |  |  |  |  |  |  |  |  |  |  |  |  |  |  |  |  |  |  |  | K01A11.2 |  |
|  |  |  |  |  |  |  |  |  |  |  |  |  |  |  |  |  |  |  |  |  |  |  |  |  |  |  |  |  |  |  |  |  |  |  |  |  |  | *jmjd-5* | JuMonJi (transcription factor) Domain protein |
|  |  |  |  |  |  |  |  |  |  |  |  |  |  |  |  |  |  |  |  |  |  |  |  |  |  |  |  |  |  |  |  |  |  |  |  |  |  | *mrpl-17* | Mitochondrial Ribosomal Protein, Large |
|  |  |  |  |  |  |  |  |  |  |  |  |  |  |  |  |  |  |  |  |  |  |  |  |  |  |  |  |  |  |  |  |  |  |  |  |  |  | *mrpl-53* | Mitochondrial Ribosomal Protein, Large |
|  |  |  |  |  |  |  |  |  |  |  |  |  |  |  |  |  |  |  |  |  |  |  |  |  |  |  |  |  |  |  |  |  |  |  |  |  |  | Y47G6A.22 |  |
|  |  |  |  |  |  |  |  |  |  |  |  |  |  |  |  |  |  |  |  |  |  |  |  |  |  |  |  |  |  |  |  |  |  |  |  |  |  | *trpp-5* | TRansport Protein Particle |

### Phenotypes enriched

none found

### Anatomy terms enriched

none found

### GO terms enriched

|  |  |  |
| --- | --- | --- |
| **GO term** | **Number of genes** | **FDR-corrected p-value** |
| positive regulation of growth rate | 19 | 4.3e-05 |
| mitochondrion | 8 | 1.7e-04 |
| positive regulation of biological process | 20 | 8.2e-04 |
| regulation of growth | 14 | 2.2e-03 |
| embryo development ending in birth or egg hatching | 23 | 3.8e-03 |
| multicellular organismal process | 29 | 5.8e-03 |
| ribosome | 5 | 7.9e-03 |
| structural constituent of ribosome | 5 | 9.0e-03 |
| anatomical structure development | 25 | 2.5e-02 |

### Expression clusters enriched

|  |  |  |  |
| --- | --- | --- | --- |
| **Group name** | **Number in cluster** | **Enrichment** | **FDR corrected p** |
| Caenorhabditis elegans Genes with expression levels changed significantly after treatment of Bacillus thurigiensis DB27. | 54 | 2.53 | 1.19e-12 |
| Caenorhabditis elegans Genes with expression levels changed significantly after treatment of Xenorhabdus nematophila. | 59 | 1.90 | 4.73e-09 |
| TGF- Dauer pathway adult transcriptional targets. Results obtained by comparing the microarray results of the dauer-constitutive mutants daf-7(e1372), daf-7(m62), and daf-1(m40) with dauer-defective mutants daf-3(mgDf90), daf-5(e1386), and daf-7(e1372);daf-3(mgDf90) double mutants at the permissive temperature, 20C, on the first day of adulthood. WBPaper00031040:TGF-beta\_adult\_downregulated | 44 | 2.35 | 1.74e-07 |
| Maternal degradation class (MD): genes that are the subset of maternal genes that decrease without first increasing in abundance. | 27 | 3.47 | 1.33e-06 |
| Maternal class (M): genes that are called present in at least one of the three PC6 replicates. | 50 | 1.92 | 3.08e-06 |
| Genes with no change in hcf-1(-), no change in sir-2.1(O/E) and downregulated in daf-2(-). | 15 | 4.64 | 2.17e-04 |
| Maternal-embryonic class (ME): genes that are in the intersection of the maternal and embryonic classes. | 27 | 2.29 | 3.64e-03 |
| Maternal degradation-embryonic class (MDE): genes that are the subset of maternal degradation genes that significantly increase in at least two of the eight total paired timepoint tests in the induction-following-degradation time domain. | 12 | 4.21 | 6.53e-03 |
| Genes in the top 10% of expression level across the triplicate L3 samples. To generate the top10 and bottom10 gene sets, authors ranked all genes by mean expression array signal intensity across the three replicates, then took the top and bottom deciles (1,841 genes each) to represent genes with high and low expression. | 21 | 2.56 | 8.97e-03 |
| Maternal degradation (MD) subclasses are based on the earliest significant decrease (abbreviated pd for primary decrease). [cgc5767]:expression\_class\_MD\_pd(41\_min) | 9 | 5.52 | 9.24e-03 |
| Developmentally modulated gene cluster. cgc4386\_cluster\_3\_5 | 5 | 12.59 | 1.24e-02 |
| Strictly maternal degradation class (SMD): genes are the subset of maternal degradation genes that are not also classified as embryonic. | 14 | 3.32 | 1.55e-02 |
| Maternal degradation (MD) subclasses are based on the earliest significant decrease (abbreviated pd for primary decrease). [cgc5767]:expression\_class\_MD\_pd(53\_min) | 12 | 3.68 | 2.07e-02 |
| C-lineage related expression profile. WBPaper00025032:cluster\_8 | 5 | 10.18 | 3.04e-02 |
| FBF-associated probe sets (FDR <2.25%) | 31 | 1.84 | 3.52e-02 |
| Genes up or down regulated by 10e-09M of cholesterol . The normalized values used were G/R ratio > 2.6 for up-regulation and G/R ratio < 0.38 for down-regulation, which corresponds to 1.39 and -1.39 log(base2) G/R ratio, respectively. | 19 | 2.38 | 4.93e-02 |

### Motifs enriched

|  |  |  |  |  |  |
| --- | --- | --- | --- | --- | --- |
| **Motif** | **Logo** | **Possible orthologs** | **Number of motifs in cluster** | **Enrichment** | **FDR corrected p** |
| FOXC2\_2 |  | lin-31 let-381 | 62 | 1.65 | 3.4e-07 |
| pTH8896 |  | pha-4 fkh-7 lin-31 daf-16 fkh-8 let-381 | 61 | 1.67 | 4.3e-07 |
| pTH9180 |  | mef-2 mel-28 let-381 Y61A9LA.9 Y116A8C.22 | 65 | 1.54 | 4.8e-07 |
| Mw151 |  | gei-11 C34D1.1 | 59 | 1.65 | 3.4e-06 |
| pTH9254 |  | mel-28 | 62 | 1.55 | 5.5e-06 |
| ONECUT3\_1 |  | dsc-1 ceh-48 | 60 | 1.58 | 1.1e-05 |
| pTH9056 |  | bed-3 (-0.51) nhr-177 | 41 | 2.17 | 1.1e-05 |
| pTH9380 |  | mel-28 | 58 | 1.62 | 1.3e-05 |
| pTH3043 |  | fkh-10 lin-31 let-381 C34D1.1 | 56 | 1.67 | 1.3e-05 |
| MA0094.2 |  | ceh-18 ceh-16 egl-5 ceh-2 alr-1 lim-6 lin-39 cog-1 ceh-14 ceh-45 ceh-53 ceh-43 mls-2 ceh-10 ceh-31 ceh-12 lim-7 ceh-30 ZC123.3 | 52 | 1.74 | 3.1e-05 |
| pTH8985 |  | athp-1 | 48 | 1.85 | 3.2e-05 |
| pTH6641 |  | lin-31 | 54 | 1.68 | 3.4e-05 |
| POU3F3\_3 |  | ceh-18 unc-86 | 55 | 1.64 | 5.1e-05 |
| FOXO1\_si |  | fkh-9 (-0.59) irx-1 daf-16 | 62 | 1.47 | 5.6e-05 |
| pTH9097 |  | Y116A8C.22 | 62 | 1.46 | 6.4e-05 |
| pTH6591 |  | lin-31 | 55 | 1.62 | 6.6e-05 |
| MA0468.1 |  | alr-1 cfi-1 ceh-14 ZC204.2 | 51 | 1.71 | 8.0e-05 |
| pTH9958 |  | ztf-2 ztf-6 | 36 | 2.21 | 8.5e-05 |
| MA0481.1 |  | fkh-7 fkh-10 lin-31 daf-16 fkh-8 | 61 | 1.48 | 8.6e-05 |
| exd\_FlyReg\_FBgn0000611 |  | ceh-20 cfi-1 let-381 | 61 | 1.47 | 9.1e-05 |
| pTH5169 |  | cfi-1 | 57 | 1.56 | 9.4e-05 |
| pTH9177 |  | F10B5.3 hsf-1 Y53C10A.3 | 56 | 1.58 | 1.0e-04 |
| pTH9260 |  | mel-28 | 59 | 1.50 | 1.4e-04 |
| pTH6497 |  | lin-31 | 52 | 1.66 | 1.4e-04 |
| MA0165.1 |  | ceh-24 php-3 D1005.3 | 49 | 1.71 | 2.0e-04 |
| pTH4425 |  | lin-39 cfi-1 unc-86 lim-7 | 49 | 1.71 | 2.2e-04 |
| CG2052\_SANGER\_2.5\_FBgn0039905 |  | mel-28 fkh-7 lin-29 | 62 | 1.41 | 3.0e-04 |
| Irx3\_1 |  | irx-1 | 48 | 1.71 | 3.2e-04 |
| MEF2A\_f1 |  | mef-2 | 49 | 1.68 | 3.3e-04 |
| CG34031\_Cell\_FBgn0054031 |  | ceh-19 alr-1 cog-1 lin-39 ceh-43 lim-7 ceh-30 | 46 | 1.75 | 3.7e-04 |
| Tbp\_pr781 |  | tbp-1 | 49 | 1.68 | 3.8e-04 |
| HXC6\_f1 |  | lin-39 | 56 | 1.52 | 3.8e-04 |
| PO3F2\_si |  | ceh-18 dmd-3 | 53 | 1.59 | 3.9e-04 |
| MA0174.1 |  | pal-1 lin-39 ceh-24 php-3 | 49 | 1.67 | 3.9e-04 |
| pTH8916 |  | hmg-12 hmbx-1 lin-39 lin-31 ceh-53 ceh-43 let-381 Y116A8C.22 | 54 | 1.56 | 4.0e-04 |
| rn\_SOLEXA\_5\_FBgn0259172 |  | lin-29 | 60 | 1.44 | 4.5e-04 |
| pTH10837 |  | ceh-24 T22H9.4 C34H4.5 | 51 | 1.60 | 6.6e-04 |
| pTH8982 |  | ceh-48 | 41 | 1.84 | 7.7e-04 |
| PDX1\_1 |  | ceh-1 lin-39 ceh-31 | 41 | 1.82 | 9.9e-04 |
| pTH2846 |  | lin-31 | 51 | 1.58 | 1.1e-03 |
| MA0249.1 |  | hlh-8 | 43 | 1.75 | 1.2e-03 |
| HepG2b\_TR4\_UCD |  | nhr-19 | 15 | 3.80 | 1.2e-03 |
| Dll\_Cell\_FBgn0000157 |  | lin-39 ceh-43 ceh-12 | 46 | 1.67 | 1.3e-03 |
| V$YY1\_01 |  | lsy-2 | 44 | 1.71 | 1.4e-03 |
| Cdx2\_4272 |  | ceh-13 | 42 | 1.76 | 1.5e-03 |
| ARI3A\_do |  | cfi-1 | 59 | 1.41 | 1.5e-03 |
| HXB7\_si |  | ceh-20 lin-39 | 48 | 1.61 | 1.7e-03 |
| pTH9137 |  | nhr-65 | 54 | 1.49 | 1.7e-03 |
| Abd-A\_FlyReg\_FBgn0000014 |  | alr-1 lin-39 | 47 | 1.63 | 1.8e-03 |
| pTH9242 |  | mel-28 | 58 | 1.41 | 2.0e-03 |
| V$TBP\_01 |  | tbp-1 | 49 | 1.57 | 2.2e-03 |
| pTH8399 |  | lin-54 | 54 | 1.48 | 2.4e-03 |
| pTH10633 |  | R07H5.10 C48E7.11 | 46 | 1.63 | 2.4e-03 |
| Hoxa13\_3126 |  | pal-1 | 27 | 2.25 | 2.6e-03 |
| Hoxd13\_2356 |  | pal-1 | 27 | 2.24 | 2.6e-03 |
| pTH10650 |  | nhr-153 | 34 | 1.94 | 2.6e-03 |
| MA0594.1 |  | lin-39 hbl-1 php-3 | 45 | 1.64 | 2.7e-03 |
| Hoxd11\_3873 |  | php-3 | 41 | 1.72 | 3.5e-03 |
| Cdx1\_2245 |  | ceh-13 | 40 | 1.73 | 3.8e-03 |
| ems\_FlyReg\_FBgn0000576 |  | ceh-2 | 40 | 1.73 | 4.0e-03 |
| pTH9480 |  | ces-2 | 39 | 1.75 | 4.1e-03 |
| pTH4325 |  | ceh-18 | 46 | 1.59 | 4.1e-03 |
| MA0253.1 |  | dsc-1 | 45 | 1.61 | 4.4e-03 |
| pTH5778 |  | egl-5 | 37 | 1.79 | 4.6e-03 |
| V$GATA3\_03 |  | nhr-7 elt-1 nhr-100 | 43 | 1.64 | 4.8e-03 |
| ftz\_FlyReg\_FBgn0001077 |  | ceh-1 lin-39 ceh-31 lim-7 | 37 | 1.79 | 4.9e-03 |
| YY1\_1 |  | lsy-2 | 36 | 1.81 | 5.3e-03 |
| pTH9135 |  | pop-1 | 49 | 1.52 | 5.7e-03 |
| Hoxa6\_1040 |  | lin-39 | 36 | 1.80 | 5.7e-03 |
| Lmx1b\_3433 |  | lim-6 | 34 | 1.86 | 5.9e-03 |
| Barx1\_2877 |  | ceh-43 | 32 | 1.92 | 5.9e-03 |
| pTH3477 |  | daf-16 | 48 | 1.53 | 5.9e-03 |
| Mv129 |  | ceh-18 sox-4 ceh-6 tbp-1 | 46 | 1.57 | 6.1e-03 |
| Vsx1\_1728 |  | alr-1 | 35 | 1.82 | 6.2e-03 |
| pTH9709 |  | die-1 | 51 | 1.48 | 6.4e-03 |
| HXD10\_f1 |  | nhr-2 php-3 | 51 | 1.48 | 6.4e-03 |
| TFEB\_f1 |  | hlh-30 | 32 | 1.91 | 6.6e-03 |
| V$S8\_01 |  | ceh-45 | 24 | 2.26 | 6.8e-03 |
| N$SKN1\_02 |  | skn-1 | 17 | 2.86 | 6.9e-03 |
| pTH6449 |  | ceh-43 | 32 | 1.90 | 7.4e-03 |
| pnr\_SANGER\_5\_FBgn0003117 |  | elt-1 | 9 | 5.11 | 7.4e-03 |
| pTH9335 |  | mel-28 | 55 | 1.40 | 7.7e-03 |
| Vax1\_3499 |  | C02F12.10 | 33 | 1.85 | 8.4e-03 |
| Hoxd3\_1742 |  | ceh-18 alr-1 lin-39 ceh-43 | 34 | 1.81 | 8.8e-03 |
| Otx1\_1 |  | alr-1 dve-1 ceh-45 ceh-53 | 35 | 1.78 | 9.1e-03 |
| pTH10797 |  | K11D2.4 lin-29 | 58 | 1.35 | 9.2e-03 |
| pTH6569 |  | ceh-43 | 31 | 1.90 | 9.4e-03 |
| Dlx1\_1741 |  | ceh-43 | 35 | 1.77 | 1.0e-02 |
| Bsx\_3483 |  | ceh-31 | 31 | 1.89 | 1.0e-02 |
| Hoxa3\_2783 |  | lin-39 | 32 | 1.85 | 1.0e-02 |
| Barx2\_3447 |  | ceh-43 | 17 | 2.73 | 1.1e-02 |
| pTH9951 |  | mex-6 (0.53) | 53 | 1.41 | 1.1e-02 |
| pTH9125 |  | egl-13 K11D2.4 | 53 | 1.41 | 1.1e-02 |
| Nkx6-3\_3446 |  | cog-1 | 36 | 1.72 | 1.2e-02 |
| V$GATA1\_06 |  | elt-1 | 35 | 1.74 | 1.4e-02 |
| pTH9925 |  | ztf-11 | 48 | 1.48 | 1.4e-02 |
| Hoxa5\_3415 |  | lin-39 | 32 | 1.81 | 1.5e-02 |
| pTH6516 |  | F19F10.1 | 38 | 1.66 | 1.5e-02 |
| V$FREAC7\_01 |  | lin-31 | 47 | 1.49 | 1.5e-02 |
| pTH8863 |  | hmg-12 | 31 | 1.83 | 1.6e-02 |
| pTH9915 |  | zip-3 | 18 | 2.52 | 1.6e-02 |
| CG31670\_SOLEXA\_5\_FBgn0031375 |  | CELE\_Y38H8A.5 | 45 | 1.52 | 1.6e-02 |
| V$FAC1\_01 |  | gei-8 | 51 | 1.42 | 1.6e-02 |
| pTH5005 |  | crh-1 W08E12.1 | 31 | 1.83 | 1.6e-02 |
| pTH9384 |  | cfi-1 | 48 | 1.47 | 1.7e-02 |
| MA0537.1 |  | blmp-1 (-0.6) | 56 | 1.35 | 1.7e-02 |
| HSFY2\_1 |  | hsf-1 | 36 | 1.69 | 1.7e-02 |
| Hoxb5\_3122 |  | lin-39 | 31 | 1.82 | 1.7e-02 |
| Dlx2\_2273 |  | ceh-43 | 34 | 1.74 | 1.7e-02 |
| Nkx6-1\_2825 |  | cog-1 | 36 | 1.68 | 1.8e-02 |
| Hoxa10\_2318 |  | ceh-24 | 38 | 1.64 | 1.8e-02 |
| Hoxa2\_3079 |  | lin-39 | 32 | 1.78 | 1.9e-02 |
| Cart1\_1275 |  | alr-1 | 35 | 1.70 | 1.9e-02 |
| pTH5111 |  | mxl-1 (0.55) aha-1 | 16 | 2.66 | 2.0e-02 |
| MA0536.1 |  | elt-1 | 14 | 2.90 | 2.2e-02 |
| pTH3046 |  | Y116A8C.22 | 36 | 1.67 | 2.2e-02 |
| pTH9215 |  | C34D1.1 | 41 | 1.56 | 2.4e-02 |
| PAX6\_f1 |  | pax-3 | 35 | 1.67 | 2.6e-02 |
| Hoxb7\_3953 |  | lin-39 | 33 | 1.72 | 2.6e-02 |
| pTH6482 |  | ceh-19 | 30 | 1.80 | 2.6e-02 |
| pTH9164 |  | ceh-26 | 38 | 1.60 | 2.7e-02 |
| Sox17\_2837 |  | sox-4 | 46 | 1.46 | 2.7e-02 |
| pTH9924 |  | nhr-46 | 25 | 1.97 | 2.8e-02 |
| pTH9247 |  | C34D1.1 | 46 | 1.46 | 2.9e-02 |
| Nkx2-3\_3435 |  | dsc-1 ceh-24 | 31 | 1.76 | 2.9e-02 |
| Dlx3\_1030 |  | ceh-43 | 33 | 1.70 | 3.0e-02 |
| MA0467.1 |  | ceh-45 | 33 | 1.70 | 3.0e-02 |
| SOX2\_2 |  | sox-4 | 39 | 1.57 | 3.1e-02 |
| Hoxa7\_2668 |  | lin-39 | 31 | 1.75 | 3.1e-02 |
| Gmeb1\_1745 |  | attf-1 | 8 | 4.42 | 3.2e-02 |
| pTH9913 |  | skn-1 | 11 | 3.29 | 3.3e-02 |
| pTH8745 |  | attf-1 | 8 | 4.39 | 3.3e-02 |
| V$ARP1\_01 |  | nhr-2 | 30 | 1.77 | 3.4e-02 |
| Zfp161\_2858 |  | pzf-1 | 16 | 2.50 | 3.4e-02 |
| MA0062.2 |  | lin-1 | 25 | 1.93 | 3.4e-02 |
| Mv75 |  | elt-1 | 18 | 2.32 | 3.5e-02 |
| MA0386.1 |  | tbp-1 | 22 | 2.06 | 3.5e-02 |
| pTH9907 |  | nhr-34 | 21 | 2.11 | 3.6e-02 |
| Hoxb4\_2627 |  | lin-39 | 33 | 1.68 | 3.7e-02 |
| Hoxa9\_2622 |  | lin-39 | 20 | 2.16 | 3.7e-02 |
| Gsh2\_3990 |  | ceh-31 | 30 | 1.75 | 3.7e-02 |
| Hoxc8\_3429 |  | lin-39 | 21 | 2.10 | 3.7e-02 |
| pTH9336 |  | tbx-39 | 19 | 2.21 | 4.1e-02 |
| Hoxb3\_1720 |  | lin-39 | 31 | 1.71 | 4.1e-02 |
| Hoxa4\_3426 |  | lin-39 | 32 | 1.68 | 4.3e-02 |
| FOXI1\_f1 |  | lin-31 | 34 | 1.64 | 4.3e-02 |
| Irx6\_2623 |  | irx-1 | 32 | 1.68 | 4.4e-02 |
| PAX8\_f1 |  | pax-2 | 18 | 2.25 | 4.5e-02 |
| Meox1\_2310 |  | ceh-31 | 32 | 1.67 | 4.6e-02 |
| pTH6445 |  | ceh-5 | 20 | 2.11 | 4.6e-02 |
| pTH10640 |  | dmd-4 | 35 | 1.61 | 4.7e-02 |
| pTH9245 |  | ceh-18 | 38 | 1.55 | 4.9e-02 |
| HeLa-S3\_RFX5\_Stanford |  | daf-19 | 32 | 1.67 | 5.0e-02 |
| RFX2\_1 |  | daf-19 | 13 | 2.72 | 5.0e-02 |

### Correlated (and anti-correlated) transcription factors

|  |  |
| --- | --- |
| **Transcription factor** | **Correlation** |
| F37B4.10 | 0.67 |
| dhhc-1 | 0.67 |
| dhhc-10 | 0.67 |
| ztf-4 | 0.67 |
| repo-1 | 0.63 |
| K04C1.3 | 0.62 |
| C01F6.9 | 0.62 |
| K11D12.12 | 0.62 |
| C16A3.4 | 0.62 |
| R144.3 | 0.61 |
| lir-3 | 0.61 |
| hmg-5 | 0.59 |
| nfyc-1 | 0.59 |
| mxl-1 | 0.55 |
| mex-6 | 0.53 |
| Y5F2A.4 | 0.51 |
| D2030.7 | 0.51 |
| W02D7.6 | 0.50 |
| ceh-41 | 0.50 |
| sptf-1 | 0.49 |
| T26A5.8 | 0.49 |
| dhhc-6 | 0.48 |
| madf-8 | 0.48 |
| sma-4 | 0.48 |
| Y53F4B.3 | 0.46 |
| nhr-137 | -0.48 |
| atf-8 | -0.48 |
| nhr-112 | -0.50 |
| nhr-91 | -0.50 |
| npax-3 | -0.50 |
| grh-1 | -0.51 |
| bed-3 | -0.51 |
| nhr-163 | -0.51 |
| madf-1 | -0.51 |
| nhr-66 | -0.52 |
| nhr-202 | -0.52 |
| ets-5 | -0.52 |
| fos-1 | -0.52 |
| F13H6.1 | -0.54 |
| nhr-5 | -0.54 |
| nhr-14 | -0.56 |
| zfp-3 | -0.57 |
| lin-48 | -0.57 |
| fkh-9 | -0.59 |
| med-2 | -0.59 |
| nhr-221 | -0.60 |
| blmp-1 | -0.60 |
| daf-12 | -0.62 |
| nhr-214 | -0.64 |
| nhr-255 | -0.64 |

### ChIP peaks enriched

|  |  |  |  |  |
| --- | --- | --- | --- | --- |
| **Gene** | **Experiment** | **Number of upstream peaks** | **Enrichment** | **FDR corrected p** |
| efl-1 | EFL-1\_Larvae-L1-stage | 34 | 2.52 | 2.4e-06 |
| efl-1 | EFL-1\_Fed-L1-stage-larvae | 33 | 2.43 | 9.3e-06 |
| efl-1 | EFL-1\_Young-adult | 34 | 2.31 | 1.8e-05 |
| dpl-1 | DPL-1\_Larvae-L4-stage | 37 | 2.07 | 5.9e-05 |
| dpl-1 | DPL-1\_Fed-L1-stage-larvae | 31 | 2.32 | 7.4e-05 |
| lsy-2 | LSY-2\_Fed-L1-stage-larvae | 29 | 2.31 | 2.2e-04 |
| F16B12.6 | F16B12.6\_Fed-L1-stage-larvae | 19 | 3.12 | 3.0e-04 |
| aly-2 | ALY-2\_Fed-L1-stage-larvae | 24 | 2.56 | 3.6e-04 |
| hpl-2 | HPL-2\_Fed-L1-stage-larvae | 33 | 2.00 | 6.6e-04 |
| W03F9.2 | W03F9.2\_L4-Young-Adult-stage-larvae | 39 | 1.79 | 8.2e-04 |
| lsy-2 | LSY-2\_Larvae-L1-stage | 32 | 1.96 | 1.5e-03 |
| ham-1 | HAM-1\_Larvae-L4-stage | 29 | 2.03 | 2.2e-03 |
| C01B12.2 | C01B12.2\_Larvae-L2-stage | 34 | 1.85 | 2.3e-03 |
| lsy-2 | LSY-2\_Embryos | 24 | 2.23 | 3.1e-03 |
| R02D3.7 | R02D3.7\_Larvae-L2-stage | 14 | 3.22 | 3.7e-03 |
| ceh-39 | CEH-39\_Embryos | 20 | 2.43 | 4.6e-03 |
| dpl-1 | DPL-1\_Young-adult | 24 | 2.11 | 7.1e-03 |
| C34F6.9 | C34F6.9\_Larvae-L2-stage | 27 | 1.97 | 7.4e-03 |
| lin-35 | LIN-35\_Fed-L1-stage-larvae | 24 | 2.08 | 8.6e-03 |
| lsy-2 | LSY-2\_Larvae-L2-stage | 15 | 2.79 | 9.0e-03 |
| R02D3.7 | R02D3.7\_Larvae-L3-stage | 27 | 1.92 | 1.1e-02 |
| nhr-77 | NHR-77\_Larvae-L4-stage | 36 | 1.63 | 1.6e-02 |
| ama-1 | AMA-1\_Larvae-L3-stage | 13 | 2.77 | 2.4e-02 |
| lin-13 | LIN-13\_Larvae-L2-stage | 19 | 2.12 | 3.2e-02 |
| ces-1 | CES-1\_Embryos | 25 | 1.84 | 3.3e-02 |
| pes-1 | PES-1\_Larvae-L4-stage | 25 | 1.83 | 3.6e-02 |
| gei-11 | GEI-11\_Larvae-L3-stage | 24 | 1.85 | 4.0e-02 |
| C16A3.4 | C16A3.4\_Fed-L1-stage-larvae | 21 | 1.98 | 4.0e-02 |
| fos-1 | FOS-1\_Fed-L1-stage-larvae | 23 | 1.89 | 4.0e-02 |
