# Supplementary material for: Overlapping cell population expression profiling and regulatory inference in C. elegans
Source: BMC Genomics. 2016 Feb 29;17:159. doi: 10.1186/s12864-016-2482-z (PMC4772325; doi:10.1186/s12864-016-2482-z)
Supplement: Additional file 13: — Web supplement. (DOC 21 kb) [file 12864_2016_2482_MOESM13_ESM.zip › sortWeb/clusters/hier.300.clusters/180.html]

Cluster 180 

## Cluster 180

### Expression

| cnd-1 rep. 1 | cnd-1 rep. 2 | cnd-1 rep. 3 | pha-4 rep. 1 | pha-4 rep. 2 | pha-4 rep. 3 | ceh-27 | ceh-36 | ceh-6 | F21D5.9 | mir-57 | mls-2 | pal-1 | pros-1 | ttx-3 | unc-130 | hlh-16 | irx-1 | ceh-6 (+) hlh-16 (+) | ceh-6 (+) hlh-16 (-) | ceh-6 (-) hlh-16 (+) | cnd-1 singlets | pha-4 singlets | 0 | 60 | 120 | 150 | 180 | 240 | 330 | 390 | 420 | 480 | 540 | 570 | 600 | 630 | 660 | NAME | Functional description |
| --- | --- | --- | --- | --- | --- | --- | --- | --- | --- | --- | --- | --- | --- | --- | --- | --- | --- | --- | --- | --- | --- | --- | --- | --- | --- | --- | --- | --- | --- | --- | --- | --- | --- | --- | --- | --- | --- | --- | --- |
|  |  |  |  |  |  |  |  |  |  |  |  |  |  |  |  |  |  |  |  |  |  |  |  |  |  |  |  |  |  |  |  |  |  |  |  |  |  | K12D9.1 |  |
|  |  |  |  |  |  |  |  |  |  |  |  |  |  |  |  |  |  |  |  |  |  |  |  |  |  |  |  |  |  |  |  |  |  |  |  |  |  | *srb-8* | Serpentine Receptor, class B (beta) |
|  |  |  |  |  |  |  |  |  |  |  |  |  |  |  |  |  |  |  |  |  |  |  |  |  |  |  |  |  |  |  |  |  |  |  |  |  |  | C31G12.4 |  |
|  |  |  |  |  |  |  |  |  |  |  |  |  |  |  |  |  |  |  |  |  |  |  |  |  |  |  |  |  |  |  |  |  |  |  |  |  |  | *klp-17* | Kinesin-Like Protein |
|  |  |  |  |  |  |  |  |  |  |  |  |  |  |  |  |  |  |  |  |  |  |  |  |  |  |  |  |  |  |  |  |  |  |  |  |  |  | Y15E3A.16 |  |
|  |  |  |  |  |  |  |  |  |  |  |  |  |  |  |  |  |  |  |  |  |  |  |  |  |  |  |  |  |  |  |  |  |  |  |  |  |  | R07G3.8 |  |
|  |  |  |  |  |  |  |  |  |  |  |  |  |  |  |  |  |  |  |  |  |  |  |  |  |  |  |  |  |  |  |  |  |  |  |  |  |  | K11H3.2 |  |
|  |  |  |  |  |  |  |  |  |  |  |  |  |  |  |  |  |  |  |  |  |  |  |  |  |  |  |  |  |  |  |  |  |  |  |  |  |  | M01G12.6 |  |
|  |  |  |  |  |  |  |  |  |  |  |  |  |  |  |  |  |  |  |  |  |  |  |  |  |  |  |  |  |  |  |  |  |  |  |  |  |  | *dsl-4* | Delta/Serrate/Lag-2 domain |
|  |  |  |  |  |  |  |  |  |  |  |  |  |  |  |  |  |  |  |  |  |  |  |  |  |  |  |  |  |  |  |  |  |  |  |  |  |  | F25H2.3 |  |
|  |  |  |  |  |  |  |  |  |  |  |  |  |  |  |  |  |  |  |  |  |  |  |  |  |  |  |  |  |  |  |  |  |  |  |  |  |  | F10E9.14 |  |
|  |  |  |  |  |  |  |  |  |  |  |  |  |  |  |  |  |  |  |  |  |  |  |  |  |  |  |  |  |  |  |  |  |  |  |  |  |  | *fbxa-50* | F-box A protein |
|  |  |  |  |  |  |  |  |  |  |  |  |  |  |  |  |  |  |  |  |  |  |  |  |  |  |  |  |  |  |  |  |  |  |  |  |  |  | *ceh-30* | C. Elegans Homeobox |
|  |  |  |  |  |  |  |  |  |  |  |  |  |  |  |  |  |  |  |  |  |  |  |  |  |  |  |  |  |  |  |  |  |  |  |  |  |  | *ceh-31* | C. Elegans Homeobox |
|  |  |  |  |  |  |  |  |  |  |  |  |  |  |  |  |  |  |  |  |  |  |  |  |  |  |  |  |  |  |  |  |  |  |  |  |  |  | *clec-181* | C-type LECtin |
|  |  |  |  |  |  |  |  |  |  |  |  |  |  |  |  |  |  |  |  |  |  |  |  |  |  |  |  |  |  |  |  |  |  |  |  |  |  | F38C2.7 |  |
|  |  |  |  |  |  |  |  |  |  |  |  |  |  |  |  |  |  |  |  |  |  |  |  |  |  |  |  |  |  |  |  |  |  |  |  |  |  | *fbxa-97* | F-box A protein |
|  |  |  |  |  |  |  |  |  |  |  |  |  |  |  |  |  |  |  |  |  |  |  |  |  |  |  |  |  |  |  |  |  |  |  |  |  |  | T24B8.10 |  |
|  |  |  |  |  |  |  |  |  |  |  |  |  |  |  |  |  |  |  |  |  |  |  |  |  |  |  |  |  |  |  |  |  |  |  |  |  |  | *sqv-8* | SQuashed Vulva |
|  |  |  |  |  |  |  |  |  |  |  |  |  |  |  |  |  |  |  |  |  |  |  |  |  |  |  |  |  |  |  |  |  |  |  |  |  |  | *nep-2* | NEPrilysin metallopeptidase family |
|  |  |  |  |  |  |  |  |  |  |  |  |  |  |  |  |  |  |  |  |  |  |  |  |  |  |  |  |  |  |  |  |  |  |  |  |  |  | *srz-80* | Serpentine Receptor, class Z |
|  |  |  |  |  |  |  |  |  |  |  |  |  |  |  |  |  |  |  |  |  |  |  |  |  |  |  |  |  |  |  |  |  |  |  |  |  |  | F32A6.8 |  |
|  |  |  |  |  |  |  |  |  |  |  |  |  |  |  |  |  |  |  |  |  |  |  |  |  |  |  |  |  |  |  |  |  |  |  |  |  |  | *ferl-1* | FER-1 Like |
|  |  |  |  |  |  |  |  |  |  |  |  |  |  |  |  |  |  |  |  |  |  |  |  |  |  |  |  |  |  |  |  |  |  |  |  |  |  | C08C3.8 |  |
|  |  |  |  |  |  |  |  |  |  |  |  |  |  |  |  |  |  |  |  |  |  |  |  |  |  |  |  |  |  |  |  |  |  |  |  |  |  | R07B5.10 |  |
|  |  |  |  |  |  |  |  |  |  |  |  |  |  |  |  |  |  |  |  |  |  |  |  |  |  |  |  |  |  |  |  |  |  |  |  |  |  | C47G2.7 |  |
|  |  |  |  |  |  |  |  |  |  |  |  |  |  |  |  |  |  |  |  |  |  |  |  |  |  |  |  |  |  |  |  |  |  |  |  |  |  | *col-73* | COLlagen |
|  |  |  |  |  |  |  |  |  |  |  |  |  |  |  |  |  |  |  |  |  |  |  |  |  |  |  |  |  |  |  |  |  |  |  |  |  |  | *ubc-6* | UBiquitin Conjugating enzyme |
|  |  |  |  |  |  |  |  |  |  |  |  |  |  |  |  |  |  |  |  |  |  |  |  |  |  |  |  |  |  |  |  |  |  |  |  |  |  | B0524.3 |  |
|  |  |  |  |  |  |  |  |  |  |  |  |  |  |  |  |  |  |  |  |  |  |  |  |  |  |  |  |  |  |  |  |  |  |  |  |  |  | Y65B4BL.1 |  |
|  |  |  |  |  |  |  |  |  |  |  |  |  |  |  |  |  |  |  |  |  |  |  |  |  |  |  |  |  |  |  |  |  |  |  |  |  |  | Y43F8B.17 |  |
|  |  |  |  |  |  |  |  |  |  |  |  |  |  |  |  |  |  |  |  |  |  |  |  |  |  |  |  |  |  |  |  |  |  |  |  |  |  | *ooc-3* | abnormal OOCyte formation |
|  |  |  |  |  |  |  |  |  |  |  |  |  |  |  |  |  |  |  |  |  |  |  |  |  |  |  |  |  |  |  |  |  |  |  |  |  |  | C41H7.11 |  |
|  |  |  |  |  |  |  |  |  |  |  |  |  |  |  |  |  |  |  |  |  |  |  |  |  |  |  |  |  |  |  |  |  |  |  |  |  |  | H09I01.2 |  |
|  |  |  |  |  |  |  |  |  |  |  |  |  |  |  |  |  |  |  |  |  |  |  |  |  |  |  |  |  |  |  |  |  |  |  |  |  |  | C16E9.13 |  |
|  |  |  |  |  |  |  |  |  |  |  |  |  |  |  |  |  |  |  |  |  |  |  |  |  |  |  |  |  |  |  |  |  |  |  |  |  |  | R155.6 |  |
|  |  |  |  |  |  |  |  |  |  |  |  |  |  |  |  |  |  |  |  |  |  |  |  |  |  |  |  |  |  |  |  |  |  |  |  |  |  | T05G5.14 |  |
|  |  |  |  |  |  |  |  |  |  |  |  |  |  |  |  |  |  |  |  |  |  |  |  |  |  |  |  |  |  |  |  |  |  |  |  |  |  | F37B12.6 |  |
|  |  |  |  |  |  |  |  |  |  |  |  |  |  |  |  |  |  |  |  |  |  |  |  |  |  |  |  |  |  |  |  |  |  |  |  |  |  | ZC132.2 |  |
|  |  |  |  |  |  |  |  |  |  |  |  |  |  |  |  |  |  |  |  |  |  |  |  |  |  |  |  |  |  |  |  |  |  |  |  |  |  | *nas-16* | Nematode AStacin protease |
|  |  |  |  |  |  |  |  |  |  |  |  |  |  |  |  |  |  |  |  |  |  |  |  |  |  |  |  |  |  |  |  |  |  |  |  |  |  | Y79H2A.4 |  |
|  |  |  |  |  |  |  |  |  |  |  |  |  |  |  |  |  |  |  |  |  |  |  |  |  |  |  |  |  |  |  |  |  |  |  |  |  |  | C09C7.2 |  |
|  |  |  |  |  |  |  |  |  |  |  |  |  |  |  |  |  |  |  |  |  |  |  |  |  |  |  |  |  |  |  |  |  |  |  |  |  |  | T22F3.1 |  |
|  |  |  |  |  |  |  |  |  |  |  |  |  |  |  |  |  |  |  |  |  |  |  |  |  |  |  |  |  |  |  |  |  |  |  |  |  |  | Y48G9A.12 |  |
|  |  |  |  |  |  |  |  |  |  |  |  |  |  |  |  |  |  |  |  |  |  |  |  |  |  |  |  |  |  |  |  |  |  |  |  |  |  | M03F4.4 |  |
|  |  |  |  |  |  |  |  |  |  |  |  |  |  |  |  |  |  |  |  |  |  |  |  |  |  |  |  |  |  |  |  |  |  |  |  |  |  | ZK669.7 |  |
|  |  |  |  |  |  |  |  |  |  |  |  |  |  |  |  |  |  |  |  |  |  |  |  |  |  |  |  |  |  |  |  |  |  |  |  |  |  | Y69H2.18 |  |
|  |  |  |  |  |  |  |  |  |  |  |  |  |  |  |  |  |  |  |  |  |  |  |  |  |  |  |  |  |  |  |  |  |  |  |  |  |  | C27H5.9 |  |
|  |  |  |  |  |  |  |  |  |  |  |  |  |  |  |  |  |  |  |  |  |  |  |  |  |  |  |  |  |  |  |  |  |  |  |  |  |  | T05B11.5 |  |
|  |  |  |  |  |  |  |  |  |  |  |  |  |  |  |  |  |  |  |  |  |  |  |  |  |  |  |  |  |  |  |  |  |  |  |  |  |  | *vps-32.1* | related to yeast Vacuolar Protein Sorting factor |
|  |  |  |  |  |  |  |  |  |  |  |  |  |  |  |  |  |  |  |  |  |  |  |  |  |  |  |  |  |  |  |  |  |  |  |  |  |  | T17A3.2 |  |
|  |  |  |  |  |  |  |  |  |  |  |  |  |  |  |  |  |  |  |  |  |  |  |  |  |  |  |  |  |  |  |  |  |  |  |  |  |  | ZC13.1 |  |
|  |  |  |  |  |  |  |  |  |  |  |  |  |  |  |  |  |  |  |  |  |  |  |  |  |  |  |  |  |  |  |  |  |  |  |  |  |  | *ndx-4* | NuDiX family |
|  |  |  |  |  |  |  |  |  |  |  |  |  |  |  |  |  |  |  |  |  |  |  |  |  |  |  |  |  |  |  |  |  |  |  |  |  |  | C49C3.8 |  |
|  |  |  |  |  |  |  |  |  |  |  |  |  |  |  |  |  |  |  |  |  |  |  |  |  |  |  |  |  |  |  |  |  |  |  |  |  |  | T10B11.6 |  |
|  |  |  |  |  |  |  |  |  |  |  |  |  |  |  |  |  |  |  |  |  |  |  |  |  |  |  |  |  |  |  |  |  |  |  |  |  |  | C23F12.4 |  |
|  |  |  |  |  |  |  |  |  |  |  |  |  |  |  |  |  |  |  |  |  |  |  |  |  |  |  |  |  |  |  |  |  |  |  |  |  |  | *unc-63* | UNCoordinated |
|  |  |  |  |  |  |  |  |  |  |  |  |  |  |  |  |  |  |  |  |  |  |  |  |  |  |  |  |  |  |  |  |  |  |  |  |  |  | Y41D4A.7 |  |
|  |  |  |  |  |  |  |  |  |  |  |  |  |  |  |  |  |  |  |  |  |  |  |  |  |  |  |  |  |  |  |  |  |  |  |  |  |  | Y71H2B.8 |  |
|  |  |  |  |  |  |  |  |  |  |  |  |  |  |  |  |  |  |  |  |  |  |  |  |  |  |  |  |  |  |  |  |  |  |  |  |  |  | *linc-22* | Long Intervening Non-Coding RNA |
|  |  |  |  |  |  |  |  |  |  |  |  |  |  |  |  |  |  |  |  |  |  |  |  |  |  |  |  |  |  |  |  |  |  |  |  |  |  | *nhr-157* | Nuclear Hormone Receptor family |
|  |  |  |  |  |  |  |  |  |  |  |  |  |  |  |  |  |  |  |  |  |  |  |  |  |  |  |  |  |  |  |  |  |  |  |  |  |  | T19C3.4 |  |
|  |  |  |  |  |  |  |  |  |  |  |  |  |  |  |  |  |  |  |  |  |  |  |  |  |  |  |  |  |  |  |  |  |  |  |  |  |  | *grk-2* | G-protein-coupled Receptor Kinase |
|  |  |  |  |  |  |  |  |  |  |  |  |  |  |  |  |  |  |  |  |  |  |  |  |  |  |  |  |  |  |  |  |  |  |  |  |  |  | *snb-1* | SyNaptoBrevin related |
|  |  |  |  |  |  |  |  |  |  |  |  |  |  |  |  |  |  |  |  |  |  |  |  |  |  |  |  |  |  |  |  |  |  |  |  |  |  | *tsp-6* | TetraSPanin family |
|  |  |  |  |  |  |  |  |  |  |  |  |  |  |  |  |  |  |  |  |  |  |  |  |  |  |  |  |  |  |  |  |  |  |  |  |  |  | ZC64.14 |  |
|  |  |  |  |  |  |  |  |  |  |  |  |  |  |  |  |  |  |  |  |  |  |  |  |  |  |  |  |  |  |  |  |  |  |  |  |  |  | T22B7.18 |  |
|  |  |  |  |  |  |  |  |  |  |  |  |  |  |  |  |  |  |  |  |  |  |  |  |  |  |  |  |  |  |  |  |  |  |  |  |  |  | F49E10.17 |  |
|  |  |  |  |  |  |  |  |  |  |  |  |  |  |  |  |  |  |  |  |  |  |  |  |  |  |  |  |  |  |  |  |  |  |  |  |  |  | *clec-98* | C-type LECtin |
|  |  |  |  |  |  |  |  |  |  |  |  |  |  |  |  |  |  |  |  |  |  |  |  |  |  |  |  |  |  |  |  |  |  |  |  |  |  | ZK270.3 |  |
|  |  |  |  |  |  |  |  |  |  |  |  |  |  |  |  |  |  |  |  |  |  |  |  |  |  |  |  |  |  |  |  |  |  |  |  |  |  | F43B10.1 |  |
|  |  |  |  |  |  |  |  |  |  |  |  |  |  |  |  |  |  |  |  |  |  |  |  |  |  |  |  |  |  |  |  |  |  |  |  |  |  | T24E12.10 |  |
|  |  |  |  |  |  |  |  |  |  |  |  |  |  |  |  |  |  |  |  |  |  |  |  |  |  |  |  |  |  |  |  |  |  |  |  |  |  | F49F1.8 |  |
|  |  |  |  |  |  |  |  |  |  |  |  |  |  |  |  |  |  |  |  |  |  |  |  |  |  |  |  |  |  |  |  |  |  |  |  |  |  | F47H4.12 |  |
|  |  |  |  |  |  |  |  |  |  |  |  |  |  |  |  |  |  |  |  |  |  |  |  |  |  |  |  |  |  |  |  |  |  |  |  |  |  | B0302.2 |  |
|  |  |  |  |  |  |  |  |  |  |  |  |  |  |  |  |  |  |  |  |  |  |  |  |  |  |  |  |  |  |  |  |  |  |  |  |  |  | B0412.6 |  |
|  |  |  |  |  |  |  |  |  |  |  |  |  |  |  |  |  |  |  |  |  |  |  |  |  |  |  |  |  |  |  |  |  |  |  |  |  |  | B0244.t1 |  |
|  |  |  |  |  |  |  |  |  |  |  |  |  |  |  |  |  |  |  |  |  |  |  |  |  |  |  |  |  |  |  |  |  |  |  |  |  |  | T19C3.6 |  |
|  |  |  |  |  |  |  |  |  |  |  |  |  |  |  |  |  |  |  |  |  |  |  |  |  |  |  |  |  |  |  |  |  |  |  |  |  |  | K08F4.14 |  |
|  |  |  |  |  |  |  |  |  |  |  |  |  |  |  |  |  |  |  |  |  |  |  |  |  |  |  |  |  |  |  |  |  |  |  |  |  |  | F53A3.8 |  |
|  |  |  |  |  |  |  |  |  |  |  |  |  |  |  |  |  |  |  |  |  |  |  |  |  |  |  |  |  |  |  |  |  |  |  |  |  |  | F12F3.5 |  |
|  |  |  |  |  |  |  |  |  |  |  |  |  |  |  |  |  |  |  |  |  |  |  |  |  |  |  |  |  |  |  |  |  |  |  |  |  |  | *srbc-84* | Serpentine Receptor, class BC (class B-like) |
|  |  |  |  |  |  |  |  |  |  |  |  |  |  |  |  |  |  |  |  |  |  |  |  |  |  |  |  |  |  |  |  |  |  |  |  |  |  | *irld-56* | Insulin/EGF-Receptor L Domain protein |
|  |  |  |  |  |  |  |  |  |  |  |  |  |  |  |  |  |  |  |  |  |  |  |  |  |  |  |  |  |  |  |  |  |  |  |  |  |  | K08A8.31 |  |
|  |  |  |  |  |  |  |  |  |  |  |  |  |  |  |  |  |  |  |  |  |  |  |  |  |  |  |  |  |  |  |  |  |  |  |  |  |  | R08C7.17 |  |
|  |  |  |  |  |  |  |  |  |  |  |  |  |  |  |  |  |  |  |  |  |  |  |  |  |  |  |  |  |  |  |  |  |  |  |  |  |  | F12D9.3 |  |
|  |  |  |  |  |  |  |  |  |  |  |  |  |  |  |  |  |  |  |  |  |  |  |  |  |  |  |  |  |  |  |  |  |  |  |  |  |  | K01A11.3 |  |
|  |  |  |  |  |  |  |  |  |  |  |  |  |  |  |  |  |  |  |  |  |  |  |  |  |  |  |  |  |  |  |  |  |  |  |  |  |  | K08A8.19 |  |
|  |  |  |  |  |  |  |  |  |  |  |  |  |  |  |  |  |  |  |  |  |  |  |  |  |  |  |  |  |  |  |  |  |  |  |  |  |  | ZK1053.6 |  |
|  |  |  |  |  |  |  |  |  |  |  |  |  |  |  |  |  |  |  |  |  |  |  |  |  |  |  |  |  |  |  |  |  |  |  |  |  |  | F36F12.2 |  |
|  |  |  |  |  |  |  |  |  |  |  |  |  |  |  |  |  |  |  |  |  |  |  |  |  |  |  |  |  |  |  |  |  |  |  |  |  |  | Y67D8C.4 |  |
|  |  |  |  |  |  |  |  |  |  |  |  |  |  |  |  |  |  |  |  |  |  |  |  |  |  |  |  |  |  |  |  |  |  |  |  |  |  | *wago-10* | Worm ArGOnaute protein |
|  |  |  |  |  |  |  |  |  |  |  |  |  |  |  |  |  |  |  |  |  |  |  |  |  |  |  |  |  |  |  |  |  |  |  |  |  |  | T22E5.7 |  |
|  |  |  |  |  |  |  |  |  |  |  |  |  |  |  |  |  |  |  |  |  |  |  |  |  |  |  |  |  |  |  |  |  |  |  |  |  |  | *cri-1* | Conserved Regulator of Innate immunity |
|  |  |  |  |  |  |  |  |  |  |  |  |  |  |  |  |  |  |  |  |  |  |  |  |  |  |  |  |  |  |  |  |  |  |  |  |  |  | F56E3.9 |  |
|  |  |  |  |  |  |  |  |  |  |  |  |  |  |  |  |  |  |  |  |  |  |  |  |  |  |  |  |  |  |  |  |  |  |  |  |  |  | F11A6.8 |  |
|  |  |  |  |  |  |  |  |  |  |  |  |  |  |  |  |  |  |  |  |  |  |  |  |  |  |  |  |  |  |  |  |  |  |  |  |  |  | C09G9.8 |  |
|  |  |  |  |  |  |  |  |  |  |  |  |  |  |  |  |  |  |  |  |  |  |  |  |  |  |  |  |  |  |  |  |  |  |  |  |  |  | C54H2.10 |  |
|  |  |  |  |  |  |  |  |  |  |  |  |  |  |  |  |  |  |  |  |  |  |  |  |  |  |  |  |  |  |  |  |  |  |  |  |  |  | Y37E3.22 |  |
|  |  |  |  |  |  |  |  |  |  |  |  |  |  |  |  |  |  |  |  |  |  |  |  |  |  |  |  |  |  |  |  |  |  |  |  |  |  | ZK816.6 |  |
|  |  |  |  |  |  |  |  |  |  |  |  |  |  |  |  |  |  |  |  |  |  |  |  |  |  |  |  |  |  |  |  |  |  |  |  |  |  | T01B6.6 |  |
|  |  |  |  |  |  |  |  |  |  |  |  |  |  |  |  |  |  |  |  |  |  |  |  |  |  |  |  |  |  |  |  |  |  |  |  |  |  | F11D5.23 |  |
|  |  |  |  |  |  |  |  |  |  |  |  |  |  |  |  |  |  |  |  |  |  |  |  |  |  |  |  |  |  |  |  |  |  |  |  |  |  | Y108G3AL.9 |  |
|  |  |  |  |  |  |  |  |  |  |  |  |  |  |  |  |  |  |  |  |  |  |  |  |  |  |  |  |  |  |  |  |  |  |  |  |  |  | *fbxa-172* | F-box A protein |
|  |  |  |  |  |  |  |  |  |  |  |  |  |  |  |  |  |  |  |  |  |  |  |  |  |  |  |  |  |  |  |  |  |  |  |  |  |  | W03G11.14 |  |
|  |  |  |  |  |  |  |  |  |  |  |  |  |  |  |  |  |  |  |  |  |  |  |  |  |  |  |  |  |  |  |  |  |  |  |  |  |  | T27F6.1 |  |
|  |  |  |  |  |  |  |  |  |  |  |  |  |  |  |  |  |  |  |  |  |  |  |  |  |  |  |  |  |  |  |  |  |  |  |  |  |  | T28A8.2 |  |
|  |  |  |  |  |  |  |  |  |  |  |  |  |  |  |  |  |  |  |  |  |  |  |  |  |  |  |  |  |  |  |  |  |  |  |  |  |  | *linc-131* | Long Intervening Non-Coding RNA |
|  |  |  |  |  |  |  |  |  |  |  |  |  |  |  |  |  |  |  |  |  |  |  |  |  |  |  |  |  |  |  |  |  |  |  |  |  |  | T04C9.3 |  |
|  |  |  |  |  |  |  |  |  |  |  |  |  |  |  |  |  |  |  |  |  |  |  |  |  |  |  |  |  |  |  |  |  |  |  |  |  |  | K08F11.2 |  |
|  |  |  |  |  |  |  |  |  |  |  |  |  |  |  |  |  |  |  |  |  |  |  |  |  |  |  |  |  |  |  |  |  |  |  |  |  |  | T19E7.27 |  |
|  |  |  |  |  |  |  |  |  |  |  |  |  |  |  |  |  |  |  |  |  |  |  |  |  |  |  |  |  |  |  |  |  |  |  |  |  |  | *puf-4* | PUF (Pumilio/FBF) domain-containing |
|  |  |  |  |  |  |  |  |  |  |  |  |  |  |  |  |  |  |  |  |  |  |  |  |  |  |  |  |  |  |  |  |  |  |  |  |  |  | F28F8.7 |  |
|  |  |  |  |  |  |  |  |  |  |  |  |  |  |  |  |  |  |  |  |  |  |  |  |  |  |  |  |  |  |  |  |  |  |  |  |  |  | C02B4.6 |  |
|  |  |  |  |  |  |  |  |  |  |  |  |  |  |  |  |  |  |  |  |  |  |  |  |  |  |  |  |  |  |  |  |  |  |  |  |  |  | Y51H4A.2 |  |
|  |  |  |  |  |  |  |  |  |  |  |  |  |  |  |  |  |  |  |  |  |  |  |  |  |  |  |  |  |  |  |  |  |  |  |  |  |  | Y35H6.3 |  |
|  |  |  |  |  |  |  |  |  |  |  |  |  |  |  |  |  |  |  |  |  |  |  |  |  |  |  |  |  |  |  |  |  |  |  |  |  |  | *twk-4* | TWiK family of potassium channels |
|  |  |  |  |  |  |  |  |  |  |  |  |  |  |  |  |  |  |  |  |  |  |  |  |  |  |  |  |  |  |  |  |  |  |  |  |  |  | F54F12.1 |  |
|  |  |  |  |  |  |  |  |  |  |  |  |  |  |  |  |  |  |  |  |  |  |  |  |  |  |  |  |  |  |  |  |  |  |  |  |  |  | *linc-90* | Long Intervening Non-Coding RNA |
|  |  |  |  |  |  |  |  |  |  |  |  |  |  |  |  |  |  |  |  |  |  |  |  |  |  |  |  |  |  |  |  |  |  |  |  |  |  | F48B9.1 |  |
|  |  |  |  |  |  |  |  |  |  |  |  |  |  |  |  |  |  |  |  |  |  |  |  |  |  |  |  |  |  |  |  |  |  |  |  |  |  | ZK380.3 |  |
|  |  |  |  |  |  |  |  |  |  |  |  |  |  |  |  |  |  |  |  |  |  |  |  |  |  |  |  |  |  |  |  |  |  |  |  |  |  | F31F6.3 |  |
|  |  |  |  |  |  |  |  |  |  |  |  |  |  |  |  |  |  |  |  |  |  |  |  |  |  |  |  |  |  |  |  |  |  |  |  |  |  | F52B10.17 |  |
|  |  |  |  |  |  |  |  |  |  |  |  |  |  |  |  |  |  |  |  |  |  |  |  |  |  |  |  |  |  |  |  |  |  |  |  |  |  | T23F2.9 |  |
|  |  |  |  |  |  |  |  |  |  |  |  |  |  |  |  |  |  |  |  |  |  |  |  |  |  |  |  |  |  |  |  |  |  |  |  |  |  | B0412.7 |  |
|  |  |  |  |  |  |  |  |  |  |  |  |  |  |  |  |  |  |  |  |  |  |  |  |  |  |  |  |  |  |  |  |  |  |  |  |  |  | F16B4.6 |  |
|  |  |  |  |  |  |  |  |  |  |  |  |  |  |  |  |  |  |  |  |  |  |  |  |  |  |  |  |  |  |  |  |  |  |  |  |  |  | Y54E5B.7 |  |
|  |  |  |  |  |  |  |  |  |  |  |  |  |  |  |  |  |  |  |  |  |  |  |  |  |  |  |  |  |  |  |  |  |  |  |  |  |  | *nhr-119* | Nuclear Hormone Receptor family |
|  |  |  |  |  |  |  |  |  |  |  |  |  |  |  |  |  |  |  |  |  |  |  |  |  |  |  |  |  |  |  |  |  |  |  |  |  |  | E03A3.1 |  |
|  |  |  |  |  |  |  |  |  |  |  |  |  |  |  |  |  |  |  |  |  |  |  |  |  |  |  |  |  |  |  |  |  |  |  |  |  |  | F57C7.8 |  |
|  |  |  |  |  |  |  |  |  |  |  |  |  |  |  |  |  |  |  |  |  |  |  |  |  |  |  |  |  |  |  |  |  |  |  |  |  |  | R10E8.10 |  |
|  |  |  |  |  |  |  |  |  |  |  |  |  |  |  |  |  |  |  |  |  |  |  |  |  |  |  |  |  |  |  |  |  |  |  |  |  |  | K08B12.6 |  |
|  |  |  |  |  |  |  |  |  |  |  |  |  |  |  |  |  |  |  |  |  |  |  |  |  |  |  |  |  |  |  |  |  |  |  |  |  |  | H22K11.12 |  |
|  |  |  |  |  |  |  |  |  |  |  |  |  |  |  |  |  |  |  |  |  |  |  |  |  |  |  |  |  |  |  |  |  |  |  |  |  |  | M01E10.3 |  |
|  |  |  |  |  |  |  |  |  |  |  |  |  |  |  |  |  |  |  |  |  |  |  |  |  |  |  |  |  |  |  |  |  |  |  |  |  |  | Y39B6A.80 |  |
|  |  |  |  |  |  |  |  |  |  |  |  |  |  |  |  |  |  |  |  |  |  |  |  |  |  |  |  |  |  |  |  |  |  |  |  |  |  | F12F6.18 |  |
|  |  |  |  |  |  |  |  |  |  |  |  |  |  |  |  |  |  |  |  |  |  |  |  |  |  |  |  |  |  |  |  |  |  |  |  |  |  | K08H10.3 |  |
|  |  |  |  |  |  |  |  |  |  |  |  |  |  |  |  |  |  |  |  |  |  |  |  |  |  |  |  |  |  |  |  |  |  |  |  |  |  | E01G6.11 |  |
|  |  |  |  |  |  |  |  |  |  |  |  |  |  |  |  |  |  |  |  |  |  |  |  |  |  |  |  |  |  |  |  |  |  |  |  |  |  | C06G1.6 |  |
|  |  |  |  |  |  |  |  |  |  |  |  |  |  |  |  |  |  |  |  |  |  |  |  |  |  |  |  |  |  |  |  |  |  |  |  |  |  | R07G3.10 |  |
|  |  |  |  |  |  |  |  |  |  |  |  |  |  |  |  |  |  |  |  |  |  |  |  |  |  |  |  |  |  |  |  |  |  |  |  |  |  | R02D5.14 |  |
|  |  |  |  |  |  |  |  |  |  |  |  |  |  |  |  |  |  |  |  |  |  |  |  |  |  |  |  |  |  |  |  |  |  |  |  |  |  | T06D8.11 |  |
|  |  |  |  |  |  |  |  |  |  |  |  |  |  |  |  |  |  |  |  |  |  |  |  |  |  |  |  |  |  |  |  |  |  |  |  |  |  | F57C7.13 |  |
|  |  |  |  |  |  |  |  |  |  |  |  |  |  |  |  |  |  |  |  |  |  |  |  |  |  |  |  |  |  |  |  |  |  |  |  |  |  | F26A10.8 |  |
|  |  |  |  |  |  |  |  |  |  |  |  |  |  |  |  |  |  |  |  |  |  |  |  |  |  |  |  |  |  |  |  |  |  |  |  |  |  | C01B7.8 |  |
|  |  |  |  |  |  |  |  |  |  |  |  |  |  |  |  |  |  |  |  |  |  |  |  |  |  |  |  |  |  |  |  |  |  |  |  |  |  | F56F3.9 |  |
|  |  |  |  |  |  |  |  |  |  |  |  |  |  |  |  |  |  |  |  |  |  |  |  |  |  |  |  |  |  |  |  |  |  |  |  |  |  | T28B11.12 |  |
|  |  |  |  |  |  |  |  |  |  |  |  |  |  |  |  |  |  |  |  |  |  |  |  |  |  |  |  |  |  |  |  |  |  |  |  |  |  | *ceh-14* | C. Elegans Homeobox |
|  |  |  |  |  |  |  |  |  |  |  |  |  |  |  |  |  |  |  |  |  |  |  |  |  |  |  |  |  |  |  |  |  |  |  |  |  |  | K11D9.6 |  |
|  |  |  |  |  |  |  |  |  |  |  |  |  |  |  |  |  |  |  |  |  |  |  |  |  |  |  |  |  |  |  |  |  |  |  |  |  |  | Y55F3AM.14 |  |
|  |  |  |  |  |  |  |  |  |  |  |  |  |  |  |  |  |  |  |  |  |  |  |  |  |  |  |  |  |  |  |  |  |  |  |  |  |  | Y34B4A.17 |  |
|  |  |  |  |  |  |  |  |  |  |  |  |  |  |  |  |  |  |  |  |  |  |  |  |  |  |  |  |  |  |  |  |  |  |  |  |  |  | Y67D2.t3 |  |
|  |  |  |  |  |  |  |  |  |  |  |  |  |  |  |  |  |  |  |  |  |  |  |  |  |  |  |  |  |  |  |  |  |  |  |  |  |  | Y73B6BL.276 |  |
|  |  |  |  |  |  |  |  |  |  |  |  |  |  |  |  |  |  |  |  |  |  |  |  |  |  |  |  |  |  |  |  |  |  |  |  |  |  | W02H5.4 |  |
|  |  |  |  |  |  |  |  |  |  |  |  |  |  |  |  |  |  |  |  |  |  |  |  |  |  |  |  |  |  |  |  |  |  |  |  |  |  | F15A8.11 |  |
|  |  |  |  |  |  |  |  |  |  |  |  |  |  |  |  |  |  |  |  |  |  |  |  |  |  |  |  |  |  |  |  |  |  |  |  |  |  | C10A4.2 |  |
|  |  |  |  |  |  |  |  |  |  |  |  |  |  |  |  |  |  |  |  |  |  |  |  |  |  |  |  |  |  |  |  |  |  |  |  |  |  | K09E9.4 |  |
|  |  |  |  |  |  |  |  |  |  |  |  |  |  |  |  |  |  |  |  |  |  |  |  |  |  |  |  |  |  |  |  |  |  |  |  |  |  | F19H8.6 |  |
|  |  |  |  |  |  |  |  |  |  |  |  |  |  |  |  |  |  |  |  |  |  |  |  |  |  |  |  |  |  |  |  |  |  |  |  |  |  | K08A8.18 |  |
|  |  |  |  |  |  |  |  |  |  |  |  |  |  |  |  |  |  |  |  |  |  |  |  |  |  |  |  |  |  |  |  |  |  |  |  |  |  | F56H6.3 |  |
|  |  |  |  |  |  |  |  |  |  |  |  |  |  |  |  |  |  |  |  |  |  |  |  |  |  |  |  |  |  |  |  |  |  |  |  |  |  | C08D8.3 |  |
|  |  |  |  |  |  |  |  |  |  |  |  |  |  |  |  |  |  |  |  |  |  |  |  |  |  |  |  |  |  |  |  |  |  |  |  |  |  | Y46E12BL.9 |  |
|  |  |  |  |  |  |  |  |  |  |  |  |  |  |  |  |  |  |  |  |  |  |  |  |  |  |  |  |  |  |  |  |  |  |  |  |  |  | F28E10.14 |  |
|  |  |  |  |  |  |  |  |  |  |  |  |  |  |  |  |  |  |  |  |  |  |  |  |  |  |  |  |  |  |  |  |  |  |  |  |  |  | T10B9.11 |  |
|  |  |  |  |  |  |  |  |  |  |  |  |  |  |  |  |  |  |  |  |  |  |  |  |  |  |  |  |  |  |  |  |  |  |  |  |  |  | C50C3.13 |  |
|  |  |  |  |  |  |  |  |  |  |  |  |  |  |  |  |  |  |  |  |  |  |  |  |  |  |  |  |  |  |  |  |  |  |  |  |  |  | E01G6.10 |  |
|  |  |  |  |  |  |  |  |  |  |  |  |  |  |  |  |  |  |  |  |  |  |  |  |  |  |  |  |  |  |  |  |  |  |  |  |  |  | F59H6.5 |  |
|  |  |  |  |  |  |  |  |  |  |  |  |  |  |  |  |  |  |  |  |  |  |  |  |  |  |  |  |  |  |  |  |  |  |  |  |  |  | R08E3.9 |  |
|  |  |  |  |  |  |  |  |  |  |  |  |  |  |  |  |  |  |  |  |  |  |  |  |  |  |  |  |  |  |  |  |  |  |  |  |  |  | C16E9.16 |  |
|  |  |  |  |  |  |  |  |  |  |  |  |  |  |  |  |  |  |  |  |  |  |  |  |  |  |  |  |  |  |  |  |  |  |  |  |  |  | Y5H2A.5 |  |
|  |  |  |  |  |  |  |  |  |  |  |  |  |  |  |  |  |  |  |  |  |  |  |  |  |  |  |  |  |  |  |  |  |  |  |  |  |  | B0507.4 |  |
|  |  |  |  |  |  |  |  |  |  |  |  |  |  |  |  |  |  |  |  |  |  |  |  |  |  |  |  |  |  |  |  |  |  |  |  |  |  | F46F5.12 |  |
|  |  |  |  |  |  |  |  |  |  |  |  |  |  |  |  |  |  |  |  |  |  |  |  |  |  |  |  |  |  |  |  |  |  |  |  |  |  | F56E3.7 |  |
|  |  |  |  |  |  |  |  |  |  |  |  |  |  |  |  |  |  |  |  |  |  |  |  |  |  |  |  |  |  |  |  |  |  |  |  |  |  | F09E5.23 |  |
|  |  |  |  |  |  |  |  |  |  |  |  |  |  |  |  |  |  |  |  |  |  |  |  |  |  |  |  |  |  |  |  |  |  |  |  |  |  | T12G3.10 |  |
|  |  |  |  |  |  |  |  |  |  |  |  |  |  |  |  |  |  |  |  |  |  |  |  |  |  |  |  |  |  |  |  |  |  |  |  |  |  | ZK262.18 |  |
|  |  |  |  |  |  |  |  |  |  |  |  |  |  |  |  |  |  |  |  |  |  |  |  |  |  |  |  |  |  |  |  |  |  |  |  |  |  | F52B10.16 |  |
|  |  |  |  |  |  |  |  |  |  |  |  |  |  |  |  |  |  |  |  |  |  |  |  |  |  |  |  |  |  |  |  |  |  |  |  |  |  | F07B7.14 |  |
|  |  |  |  |  |  |  |  |  |  |  |  |  |  |  |  |  |  |  |  |  |  |  |  |  |  |  |  |  |  |  |  |  |  |  |  |  |  | F07C6.5 |  |
|  |  |  |  |  |  |  |  |  |  |  |  |  |  |  |  |  |  |  |  |  |  |  |  |  |  |  |  |  |  |  |  |  |  |  |  |  |  | B0286.8 |  |
|  |  |  |  |  |  |  |  |  |  |  |  |  |  |  |  |  |  |  |  |  |  |  |  |  |  |  |  |  |  |  |  |  |  |  |  |  |  | B0496.12 |  |
|  |  |  |  |  |  |  |  |  |  |  |  |  |  |  |  |  |  |  |  |  |  |  |  |  |  |  |  |  |  |  |  |  |  |  |  |  |  | W08F4.18 |  |
|  |  |  |  |  |  |  |  |  |  |  |  |  |  |  |  |  |  |  |  |  |  |  |  |  |  |  |  |  |  |  |  |  |  |  |  |  |  | B0244.14 |  |
|  |  |  |  |  |  |  |  |  |  |  |  |  |  |  |  |  |  |  |  |  |  |  |  |  |  |  |  |  |  |  |  |  |  |  |  |  |  | M70.13 |  |
|  |  |  |  |  |  |  |  |  |  |  |  |  |  |  |  |  |  |  |  |  |  |  |  |  |  |  |  |  |  |  |  |  |  |  |  |  |  | F49E10.21 |  |
|  |  |  |  |  |  |  |  |  |  |  |  |  |  |  |  |  |  |  |  |  |  |  |  |  |  |  |  |  |  |  |  |  |  |  |  |  |  | C36F7.21 |  |
|  |  |  |  |  |  |  |  |  |  |  |  |  |  |  |  |  |  |  |  |  |  |  |  |  |  |  |  |  |  |  |  |  |  |  |  |  |  | C36F7.17 |  |
|  |  |  |  |  |  |  |  |  |  |  |  |  |  |  |  |  |  |  |  |  |  |  |  |  |  |  |  |  |  |  |  |  |  |  |  |  |  | F11A6.11 |  |
|  |  |  |  |  |  |  |  |  |  |  |  |  |  |  |  |  |  |  |  |  |  |  |  |  |  |  |  |  |  |  |  |  |  |  |  |  |  | K07C5.16 |  |
|  |  |  |  |  |  |  |  |  |  |  |  |  |  |  |  |  |  |  |  |  |  |  |  |  |  |  |  |  |  |  |  |  |  |  |  |  |  | F01G12.14 |  |
|  |  |  |  |  |  |  |  |  |  |  |  |  |  |  |  |  |  |  |  |  |  |  |  |  |  |  |  |  |  |  |  |  |  |  |  |  |  | T05A7.9 |  |
|  |  |  |  |  |  |  |  |  |  |  |  |  |  |  |  |  |  |  |  |  |  |  |  |  |  |  |  |  |  |  |  |  |  |  |  |  |  | F46G11.13 |  |
|  |  |  |  |  |  |  |  |  |  |  |  |  |  |  |  |  |  |  |  |  |  |  |  |  |  |  |  |  |  |  |  |  |  |  |  |  |  | K08A8.6 |  |
|  |  |  |  |  |  |  |  |  |  |  |  |  |  |  |  |  |  |  |  |  |  |  |  |  |  |  |  |  |  |  |  |  |  |  |  |  |  | C43F9.16 |  |
|  |  |  |  |  |  |  |  |  |  |  |  |  |  |  |  |  |  |  |  |  |  |  |  |  |  |  |  |  |  |  |  |  |  |  |  |  |  | F21A3.t2 |  |
|  |  |  |  |  |  |  |  |  |  |  |  |  |  |  |  |  |  |  |  |  |  |  |  |  |  |  |  |  |  |  |  |  |  |  |  |  |  | F46C3.5 |  |
|  |  |  |  |  |  |  |  |  |  |  |  |  |  |  |  |  |  |  |  |  |  |  |  |  |  |  |  |  |  |  |  |  |  |  |  |  |  | F38B6.24 |  |
|  |  |  |  |  |  |  |  |  |  |  |  |  |  |  |  |  |  |  |  |  |  |  |  |  |  |  |  |  |  |  |  |  |  |  |  |  |  | Y53F4A.5 |  |
|  |  |  |  |  |  |  |  |  |  |  |  |  |  |  |  |  |  |  |  |  |  |  |  |  |  |  |  |  |  |  |  |  |  |  |  |  |  | F02D10.t2 |  |
|  |  |  |  |  |  |  |  |  |  |  |  |  |  |  |  |  |  |  |  |  |  |  |  |  |  |  |  |  |  |  |  |  |  |  |  |  |  | F28F9.10 |  |
|  |  |  |  |  |  |  |  |  |  |  |  |  |  |  |  |  |  |  |  |  |  |  |  |  |  |  |  |  |  |  |  |  |  |  |  |  |  | C06A6.11 |  |
|  |  |  |  |  |  |  |  |  |  |  |  |  |  |  |  |  |  |  |  |  |  |  |  |  |  |  |  |  |  |  |  |  |  |  |  |  |  | C43D7.15 |  |
|  |  |  |  |  |  |  |  |  |  |  |  |  |  |  |  |  |  |  |  |  |  |  |  |  |  |  |  |  |  |  |  |  |  |  |  |  |  | F13D11.14 |  |
|  |  |  |  |  |  |  |  |  |  |  |  |  |  |  |  |  |  |  |  |  |  |  |  |  |  |  |  |  |  |  |  |  |  |  |  |  |  | C01F4.6 |  |
|  |  |  |  |  |  |  |  |  |  |  |  |  |  |  |  |  |  |  |  |  |  |  |  |  |  |  |  |  |  |  |  |  |  |  |  |  |  | C09D8.14 |  |
|  |  |  |  |  |  |  |  |  |  |  |  |  |  |  |  |  |  |  |  |  |  |  |  |  |  |  |  |  |  |  |  |  |  |  |  |  |  | Y40A1A.1 |  |
|  |  |  |  |  |  |  |  |  |  |  |  |  |  |  |  |  |  |  |  |  |  |  |  |  |  |  |  |  |  |  |  |  |  |  |  |  |  | Y102A11A.15 |  |
|  |  |  |  |  |  |  |  |  |  |  |  |  |  |  |  |  |  |  |  |  |  |  |  |  |  |  |  |  |  |  |  |  |  |  |  |  |  | F19H8.t1 |  |
|  |  |  |  |  |  |  |  |  |  |  |  |  |  |  |  |  |  |  |  |  |  |  |  |  |  |  |  |  |  |  |  |  |  |  |  |  |  | M79.11 |  |
|  |  |  |  |  |  |  |  |  |  |  |  |  |  |  |  |  |  |  |  |  |  |  |  |  |  |  |  |  |  |  |  |  |  |  |  |  |  | W05H12.3 |  |
|  |  |  |  |  |  |  |  |  |  |  |  |  |  |  |  |  |  |  |  |  |  |  |  |  |  |  |  |  |  |  |  |  |  |  |  |  |  | F26H11.9 |  |
|  |  |  |  |  |  |  |  |  |  |  |  |  |  |  |  |  |  |  |  |  |  |  |  |  |  |  |  |  |  |  |  |  |  |  |  |  |  | T27B1.6 |  |
|  |  |  |  |  |  |  |  |  |  |  |  |  |  |  |  |  |  |  |  |  |  |  |  |  |  |  |  |  |  |  |  |  |  |  |  |  |  | F21D9.7 |  |
|  |  |  |  |  |  |  |  |  |  |  |  |  |  |  |  |  |  |  |  |  |  |  |  |  |  |  |  |  |  |  |  |  |  |  |  |  |  | F48A11.7 |  |
|  |  |  |  |  |  |  |  |  |  |  |  |  |  |  |  |  |  |  |  |  |  |  |  |  |  |  |  |  |  |  |  |  |  |  |  |  |  | F59G1.13 |  |
|  |  |  |  |  |  |  |  |  |  |  |  |  |  |  |  |  |  |  |  |  |  |  |  |  |  |  |  |  |  |  |  |  |  |  |  |  |  | F49E10.26 |  |
|  |  |  |  |  |  |  |  |  |  |  |  |  |  |  |  |  |  |  |  |  |  |  |  |  |  |  |  |  |  |  |  |  |  |  |  |  |  | C14F11.13 |  |
|  |  |  |  |  |  |  |  |  |  |  |  |  |  |  |  |  |  |  |  |  |  |  |  |  |  |  |  |  |  |  |  |  |  |  |  |  |  | Y47D3B.15 |  |
|  |  |  |  |  |  |  |  |  |  |  |  |  |  |  |  |  |  |  |  |  |  |  |  |  |  |  |  |  |  |  |  |  |  |  |  |  |  | K04C2.9 |  |
|  |  |  |  |  |  |  |  |  |  |  |  |  |  |  |  |  |  |  |  |  |  |  |  |  |  |  |  |  |  |  |  |  |  |  |  |  |  | F46G11.8 |  |
|  |  |  |  |  |  |  |  |  |  |  |  |  |  |  |  |  |  |  |  |  |  |  |  |  |  |  |  |  |  |  |  |  |  |  |  |  |  | C32A3.6 |  |
|  |  |  |  |  |  |  |  |  |  |  |  |  |  |  |  |  |  |  |  |  |  |  |  |  |  |  |  |  |  |  |  |  |  |  |  |  |  | K02H8.6 |  |
|  |  |  |  |  |  |  |  |  |  |  |  |  |  |  |  |  |  |  |  |  |  |  |  |  |  |  |  |  |  |  |  |  |  |  |  |  |  | T27B1.5 |  |
|  |  |  |  |  |  |  |  |  |  |  |  |  |  |  |  |  |  |  |  |  |  |  |  |  |  |  |  |  |  |  |  |  |  |  |  |  |  | F47C8.4 |  |
|  |  |  |  |  |  |  |  |  |  |  |  |  |  |  |  |  |  |  |  |  |  |  |  |  |  |  |  |  |  |  |  |  |  |  |  |  |  | F09B12.8 |  |
|  |  |  |  |  |  |  |  |  |  |  |  |  |  |  |  |  |  |  |  |  |  |  |  |  |  |  |  |  |  |  |  |  |  |  |  |  |  | M163.15 |  |
|  |  |  |  |  |  |  |  |  |  |  |  |  |  |  |  |  |  |  |  |  |  |  |  |  |  |  |  |  |  |  |  |  |  |  |  |  |  | B0198.10 |  |
|  |  |  |  |  |  |  |  |  |  |  |  |  |  |  |  |  |  |  |  |  |  |  |  |  |  |  |  |  |  |  |  |  |  |  |  |  |  | Y71H9A.7 |  |
|  |  |  |  |  |  |  |  |  |  |  |  |  |  |  |  |  |  |  |  |  |  |  |  |  |  |  |  |  |  |  |  |  |  |  |  |  |  | C05C9.6 |  |
|  |  |  |  |  |  |  |  |  |  |  |  |  |  |  |  |  |  |  |  |  |  |  |  |  |  |  |  |  |  |  |  |  |  |  |  |  |  | T21B6.14 |  |
|  |  |  |  |  |  |  |  |  |  |  |  |  |  |  |  |  |  |  |  |  |  |  |  |  |  |  |  |  |  |  |  |  |  |  |  |  |  | M79.8 |  |
|  |  |  |  |  |  |  |  |  |  |  |  |  |  |  |  |  |  |  |  |  |  |  |  |  |  |  |  |  |  |  |  |  |  |  |  |  |  | F09B9.7 |  |
|  |  |  |  |  |  |  |  |  |  |  |  |  |  |  |  |  |  |  |  |  |  |  |  |  |  |  |  |  |  |  |  |  |  |  |  |  |  | F12D9.6 |  |
|  |  |  |  |  |  |  |  |  |  |  |  |  |  |  |  |  |  |  |  |  |  |  |  |  |  |  |  |  |  |  |  |  |  |  |  |  |  | F27D9.9 |  |
|  |  |  |  |  |  |  |  |  |  |  |  |  |  |  |  |  |  |  |  |  |  |  |  |  |  |  |  |  |  |  |  |  |  |  |  |  |  | F26A10.15 |  |
|  |  |  |  |  |  |  |  |  |  |  |  |  |  |  |  |  |  |  |  |  |  |  |  |  |  |  |  |  |  |  |  |  |  |  |  |  |  | T10A3.5 |  |
|  |  |  |  |  |  |  |  |  |  |  |  |  |  |  |  |  |  |  |  |  |  |  |  |  |  |  |  |  |  |  |  |  |  |  |  |  |  | F46G11.12 |  |
|  |  |  |  |  |  |  |  |  |  |  |  |  |  |  |  |  |  |  |  |  |  |  |  |  |  |  |  |  |  |  |  |  |  |  |  |  |  | C26B9.9 |  |
|  |  |  |  |  |  |  |  |  |  |  |  |  |  |  |  |  |  |  |  |  |  |  |  |  |  |  |  |  |  |  |  |  |  |  |  |  |  | C42D8.18 |  |
|  |  |  |  |  |  |  |  |  |  |  |  |  |  |  |  |  |  |  |  |  |  |  |  |  |  |  |  |  |  |  |  |  |  |  |  |  |  | C15C7.10 |  |
|  |  |  |  |  |  |  |  |  |  |  |  |  |  |  |  |  |  |  |  |  |  |  |  |  |  |  |  |  |  |  |  |  |  |  |  |  |  | F52E4.13 |  |
|  |  |  |  |  |  |  |  |  |  |  |  |  |  |  |  |  |  |  |  |  |  |  |  |  |  |  |  |  |  |  |  |  |  |  |  |  |  | M02F4.11 |  |
|  |  |  |  |  |  |  |  |  |  |  |  |  |  |  |  |  |  |  |  |  |  |  |  |  |  |  |  |  |  |  |  |  |  |  |  |  |  | F52B10.9 |  |
|  |  |  |  |  |  |  |  |  |  |  |  |  |  |  |  |  |  |  |  |  |  |  |  |  |  |  |  |  |  |  |  |  |  |  |  |  |  | Y60A3A.33 |  |
|  |  |  |  |  |  |  |  |  |  |  |  |  |  |  |  |  |  |  |  |  |  |  |  |  |  |  |  |  |  |  |  |  |  |  |  |  |  | ZK262.17 |  |
|  |  |  |  |  |  |  |  |  |  |  |  |  |  |  |  |  |  |  |  |  |  |  |  |  |  |  |  |  |  |  |  |  |  |  |  |  |  | K02E11.t1 |  |
|  |  |  |  |  |  |  |  |  |  |  |  |  |  |  |  |  |  |  |  |  |  |  |  |  |  |  |  |  |  |  |  |  |  |  |  |  |  | R04F11.27 |  |
|  |  |  |  |  |  |  |  |  |  |  |  |  |  |  |  |  |  |  |  |  |  |  |  |  |  |  |  |  |  |  |  |  |  |  |  |  |  | F35B12.16 |  |
|  |  |  |  |  |  |  |  |  |  |  |  |  |  |  |  |  |  |  |  |  |  |  |  |  |  |  |  |  |  |  |  |  |  |  |  |  |  | EGAP798.1 |  |
|  |  |  |  |  |  |  |  |  |  |  |  |  |  |  |  |  |  |  |  |  |  |  |  |  |  |  |  |  |  |  |  |  |  |  |  |  |  | F13H6.13 |  |
|  |  |  |  |  |  |  |  |  |  |  |  |  |  |  |  |  |  |  |  |  |  |  |  |  |  |  |  |  |  |  |  |  |  |  |  |  |  | C10F3.9 |  |
|  |  |  |  |  |  |  |  |  |  |  |  |  |  |  |  |  |  |  |  |  |  |  |  |  |  |  |  |  |  |  |  |  |  |  |  |  |  | T06A1.9 |  |
|  |  |  |  |  |  |  |  |  |  |  |  |  |  |  |  |  |  |  |  |  |  |  |  |  |  |  |  |  |  |  |  |  |  |  |  |  |  | F55B12.13 |  |
|  |  |  |  |  |  |  |  |  |  |  |  |  |  |  |  |  |  |  |  |  |  |  |  |  |  |  |  |  |  |  |  |  |  |  |  |  |  | Y57G11C.1141 |  |
|  |  |  |  |  |  |  |  |  |  |  |  |  |  |  |  |  |  |  |  |  |  |  |  |  |  |  |  |  |  |  |  |  |  |  |  |  |  | F35G2.20 |  |
|  |  |  |  |  |  |  |  |  |  |  |  |  |  |  |  |  |  |  |  |  |  |  |  |  |  |  |  |  |  |  |  |  |  |  |  |  |  | B0478.24 |  |
|  |  |  |  |  |  |  |  |  |  |  |  |  |  |  |  |  |  |  |  |  |  |  |  |  |  |  |  |  |  |  |  |  |  |  |  |  |  | B0350.69 |  |
|  |  |  |  |  |  |  |  |  |  |  |  |  |  |  |  |  |  |  |  |  |  |  |  |  |  |  |  |  |  |  |  |  |  |  |  |  |  | Y41D4B.29 |  |
|  |  |  |  |  |  |  |  |  |  |  |  |  |  |  |  |  |  |  |  |  |  |  |  |  |  |  |  |  |  |  |  |  |  |  |  |  |  | F29C4.10 |  |
|  |  |  |  |  |  |  |  |  |  |  |  |  |  |  |  |  |  |  |  |  |  |  |  |  |  |  |  |  |  |  |  |  |  |  |  |  |  | Y47D3B.20 |  |
|  |  |  |  |  |  |  |  |  |  |  |  |  |  |  |  |  |  |  |  |  |  |  |  |  |  |  |  |  |  |  |  |  |  |  |  |  |  | ZK757.5 |  |
|  |  |  |  |  |  |  |  |  |  |  |  |  |  |  |  |  |  |  |  |  |  |  |  |  |  |  |  |  |  |  |  |  |  |  |  |  |  | K04C2.t2 |  |
|  |  |  |  |  |  |  |  |  |  |  |  |  |  |  |  |  |  |  |  |  |  |  |  |  |  |  |  |  |  |  |  |  |  |  |  |  |  | C05D2.13 |  |
|  |  |  |  |  |  |  |  |  |  |  |  |  |  |  |  |  |  |  |  |  |  |  |  |  |  |  |  |  |  |  |  |  |  |  |  |  |  | Y39A3A.9 |  |
|  |  |  |  |  |  |  |  |  |  |  |  |  |  |  |  |  |  |  |  |  |  |  |  |  |  |  |  |  |  |  |  |  |  |  |  |  |  | Y71D11A.7 |  |
|  |  |  |  |  |  |  |  |  |  |  |  |  |  |  |  |  |  |  |  |  |  |  |  |  |  |  |  |  |  |  |  |  |  |  |  |  |  | T01B6.10 |  |
|  |  |  |  |  |  |  |  |  |  |  |  |  |  |  |  |  |  |  |  |  |  |  |  |  |  |  |  |  |  |  |  |  |  |  |  |  |  | T26G10.4 |  |
|  |  |  |  |  |  |  |  |  |  |  |  |  |  |  |  |  |  |  |  |  |  |  |  |  |  |  |  |  |  |  |  |  |  |  |  |  |  | Y46E12BL.5 |  |
|  |  |  |  |  |  |  |  |  |  |  |  |  |  |  |  |  |  |  |  |  |  |  |  |  |  |  |  |  |  |  |  |  |  |  |  |  |  | Y48B6A.21 |  |
|  |  |  |  |  |  |  |  |  |  |  |  |  |  |  |  |  |  |  |  |  |  |  |  |  |  |  |  |  |  |  |  |  |  |  |  |  |  | W03C9.9 |  |
|  |  |  |  |  |  |  |  |  |  |  |  |  |  |  |  |  |  |  |  |  |  |  |  |  |  |  |  |  |  |  |  |  |  |  |  |  |  | T24B8.12 |  |
|  |  |  |  |  |  |  |  |  |  |  |  |  |  |  |  |  |  |  |  |  |  |  |  |  |  |  |  |  |  |  |  |  |  |  |  |  |  | K02C4.9 |  |
|  |  |  |  |  |  |  |  |  |  |  |  |  |  |  |  |  |  |  |  |  |  |  |  |  |  |  |  |  |  |  |  |  |  |  |  |  |  | C33F10.19 |  |
|  |  |  |  |  |  |  |  |  |  |  |  |  |  |  |  |  |  |  |  |  |  |  |  |  |  |  |  |  |  |  |  |  |  |  |  |  |  | Y49F6B.6 |  |
|  |  |  |  |  |  |  |  |  |  |  |  |  |  |  |  |  |  |  |  |  |  |  |  |  |  |  |  |  |  |  |  |  |  |  |  |  |  | *str-21* | Seven TM Receptor |
|  |  |  |  |  |  |  |  |  |  |  |  |  |  |  |  |  |  |  |  |  |  |  |  |  |  |  |  |  |  |  |  |  |  |  |  |  |  | T07D3.11 |  |
|  |  |  |  |  |  |  |  |  |  |  |  |  |  |  |  |  |  |  |  |  |  |  |  |  |  |  |  |  |  |  |  |  |  |  |  |  |  | T01H3.8 |  |
|  |  |  |  |  |  |  |  |  |  |  |  |  |  |  |  |  |  |  |  |  |  |  |  |  |  |  |  |  |  |  |  |  |  |  |  |  |  | B0432.17 |  |
|  |  |  |  |  |  |  |  |  |  |  |  |  |  |  |  |  |  |  |  |  |  |  |  |  |  |  |  |  |  |  |  |  |  |  |  |  |  | F47G4.11 |  |
|  |  |  |  |  |  |  |  |  |  |  |  |  |  |  |  |  |  |  |  |  |  |  |  |  |  |  |  |  |  |  |  |  |  |  |  |  |  | Y71A12B.26 |  |
|  |  |  |  |  |  |  |  |  |  |  |  |  |  |  |  |  |  |  |  |  |  |  |  |  |  |  |  |  |  |  |  |  |  |  |  |  |  | F33E2.7 |  |
|  |  |  |  |  |  |  |  |  |  |  |  |  |  |  |  |  |  |  |  |  |  |  |  |  |  |  |  |  |  |  |  |  |  |  |  |  |  | Y105E8A.42 |  |
|  |  |  |  |  |  |  |  |  |  |  |  |  |  |  |  |  |  |  |  |  |  |  |  |  |  |  |  |  |  |  |  |  |  |  |  |  |  | Y95D11A.t1 |  |
|  |  |  |  |  |  |  |  |  |  |  |  |  |  |  |  |  |  |  |  |  |  |  |  |  |  |  |  |  |  |  |  |  |  |  |  |  |  | F49D11.12 |  |
|  |  |  |  |  |  |  |  |  |  |  |  |  |  |  |  |  |  |  |  |  |  |  |  |  |  |  |  |  |  |  |  |  |  |  |  |  |  | F10G8.12 |  |
|  |  |  |  |  |  |  |  |  |  |  |  |  |  |  |  |  |  |  |  |  |  |  |  |  |  |  |  |  |  |  |  |  |  |  |  |  |  | ZC123.11 |  |
|  |  |  |  |  |  |  |  |  |  |  |  |  |  |  |  |  |  |  |  |  |  |  |  |  |  |  |  |  |  |  |  |  |  |  |  |  |  | C36F7.20 |  |
|  |  |  |  |  |  |  |  |  |  |  |  |  |  |  |  |  |  |  |  |  |  |  |  |  |  |  |  |  |  |  |  |  |  |  |  |  |  | *dhc-4* | Dynein Heavy Chain |
|  |  |  |  |  |  |  |  |  |  |  |  |  |  |  |  |  |  |  |  |  |  |  |  |  |  |  |  |  |  |  |  |  |  |  |  |  |  | *nhr-85* | Nuclear Hormone Receptor family |
|  |  |  |  |  |  |  |  |  |  |  |  |  |  |  |  |  |  |  |  |  |  |  |  |  |  |  |  |  |  |  |  |  |  |  |  |  |  | F54E2.4 |  |
|  |  |  |  |  |  |  |  |  |  |  |  |  |  |  |  |  |  |  |  |  |  |  |  |  |  |  |  |  |  |  |  |  |  |  |  |  |  | F09E5.21 |  |
|  |  |  |  |  |  |  |  |  |  |  |  |  |  |  |  |  |  |  |  |  |  |  |  |  |  |  |  |  |  |  |  |  |  |  |  |  |  | F41G4.5 |  |
|  |  |  |  |  |  |  |  |  |  |  |  |  |  |  |  |  |  |  |  |  |  |  |  |  |  |  |  |  |  |  |  |  |  |  |  |  |  | Y60A9A.1 |  |
|  |  |  |  |  |  |  |  |  |  |  |  |  |  |  |  |  |  |  |  |  |  |  |  |  |  |  |  |  |  |  |  |  |  |  |  |  |  | Y53F4B.56 |  |
|  |  |  |  |  |  |  |  |  |  |  |  |  |  |  |  |  |  |  |  |  |  |  |  |  |  |  |  |  |  |  |  |  |  |  |  |  |  | ZK994.7 |  |
|  |  |  |  |  |  |  |  |  |  |  |  |  |  |  |  |  |  |  |  |  |  |  |  |  |  |  |  |  |  |  |  |  |  |  |  |  |  | C01G6.13 |  |
|  |  |  |  |  |  |  |  |  |  |  |  |  |  |  |  |  |  |  |  |  |  |  |  |  |  |  |  |  |  |  |  |  |  |  |  |  |  | C34F11.17 |  |
|  |  |  |  |  |  |  |  |  |  |  |  |  |  |  |  |  |  |  |  |  |  |  |  |  |  |  |  |  |  |  |  |  |  |  |  |  |  | C02D4.8 |  |
|  |  |  |  |  |  |  |  |  |  |  |  |  |  |  |  |  |  |  |  |  |  |  |  |  |  |  |  |  |  |  |  |  |  |  |  |  |  | C01F1.8 |  |
|  |  |  |  |  |  |  |  |  |  |  |  |  |  |  |  |  |  |  |  |  |  |  |  |  |  |  |  |  |  |  |  |  |  |  |  |  |  | Y57E12B.6 |  |
|  |  |  |  |  |  |  |  |  |  |  |  |  |  |  |  |  |  |  |  |  |  |  |  |  |  |  |  |  |  |  |  |  |  |  |  |  |  | C14B4.t1 |  |
|  |  |  |  |  |  |  |  |  |  |  |  |  |  |  |  |  |  |  |  |  |  |  |  |  |  |  |  |  |  |  |  |  |  |  |  |  |  | T19B4.8 |  |
|  |  |  |  |  |  |  |  |  |  |  |  |  |  |  |  |  |  |  |  |  |  |  |  |  |  |  |  |  |  |  |  |  |  |  |  |  |  | Y66A7AL.4 |  |
|  |  |  |  |  |  |  |  |  |  |  |  |  |  |  |  |  |  |  |  |  |  |  |  |  |  |  |  |  |  |  |  |  |  |  |  |  |  | ZK867.9 |  |
|  |  |  |  |  |  |  |  |  |  |  |  |  |  |  |  |  |  |  |  |  |  |  |  |  |  |  |  |  |  |  |  |  |  |  |  |  |  | K02A4.11 |  |
|  |  |  |  |  |  |  |  |  |  |  |  |  |  |  |  |  |  |  |  |  |  |  |  |  |  |  |  |  |  |  |  |  |  |  |  |  |  | K12D12.6 |  |
|  |  |  |  |  |  |  |  |  |  |  |  |  |  |  |  |  |  |  |  |  |  |  |  |  |  |  |  |  |  |  |  |  |  |  |  |  |  | BE10.3 |  |
|  |  |  |  |  |  |  |  |  |  |  |  |  |  |  |  |  |  |  |  |  |  |  |  |  |  |  |  |  |  |  |  |  |  |  |  |  |  | Y38E10A.20 |  |
|  |  |  |  |  |  |  |  |  |  |  |  |  |  |  |  |  |  |  |  |  |  |  |  |  |  |  |  |  |  |  |  |  |  |  |  |  |  | K08A8.20 |  |
|  |  |  |  |  |  |  |  |  |  |  |  |  |  |  |  |  |  |  |  |  |  |  |  |  |  |  |  |  |  |  |  |  |  |  |  |  |  | M163.13 |  |
|  |  |  |  |  |  |  |  |  |  |  |  |  |  |  |  |  |  |  |  |  |  |  |  |  |  |  |  |  |  |  |  |  |  |  |  |  |  | *srv-15* | Serpentine Receptor, class V |
|  |  |  |  |  |  |  |  |  |  |  |  |  |  |  |  |  |  |  |  |  |  |  |  |  |  |  |  |  |  |  |  |  |  |  |  |  |  | C31H2.6 |  |
|  |  |  |  |  |  |  |  |  |  |  |  |  |  |  |  |  |  |  |  |  |  |  |  |  |  |  |  |  |  |  |  |  |  |  |  |  |  | C05D2.17 |  |
|  |  |  |  |  |  |  |  |  |  |  |  |  |  |  |  |  |  |  |  |  |  |  |  |  |  |  |  |  |  |  |  |  |  |  |  |  |  | F07G6.9 |  |
|  |  |  |  |  |  |  |  |  |  |  |  |  |  |  |  |  |  |  |  |  |  |  |  |  |  |  |  |  |  |  |  |  |  |  |  |  |  | T23E1.5 |  |
|  |  |  |  |  |  |  |  |  |  |  |  |  |  |  |  |  |  |  |  |  |  |  |  |  |  |  |  |  |  |  |  |  |  |  |  |  |  | K08A8.30 |  |

### Phenotypes enriched

none found

### Anatomy terms enriched

none found

### GO terms enriched

none found

### Expression clusters enriched

none found

### Motifs enriched

|  |  |  |  |  |  |
| --- | --- | --- | --- | --- | --- |
| **Motif** | **Logo** | **Possible orthologs** | **Number of motifs in cluster** | **Enrichment** | **FDR corrected p** |
| Plagl1\_0972 |  | Y53H1A.2 (0.73) | 85 | 2.19 | 1.7e-09 |
| MA0118.1 |  | ref-2 | 85 | 2.16 | 3.2e-09 |
| ZBT7A\_f1 |  | ZC328.2 klf-1 | 71 | 2.40 | 3.3e-09 |
| SP4\_f1 |  | Y53H1A.2 (0.73) klf-2 plp-2 | 100 | 1.98 | 3.8e-09 |
| pTH2818 |  | ref-2 eor-1 egrh-3 | 123 | 1.78 | 4.6e-09 |
| K562\_ZBTB7A\_HudsonAlpha |  | ZC328.2 | 142 | 1.67 | 5.7e-09 |
| EGR1\_1 |  | klf-2 ZC328.2 klf-1 | 146 | 1.64 | 6.2e-09 |
| MA0472.1 |  | ZC328.2 daf-16 | 154 | 1.58 | 1.6e-08 |
| HepG2\_ELF1\_HudsonAlpha |  | lin-1 C24A1.2 | 131 | 1.70 | 1.7e-08 |
| pTH10015 |  | ces-1 che-1 ztf-28 | 153 | 1.55 | 9.8e-08 |
| pTH9249 |  | daf-19 (0.56) | 97 | 1.88 | 1.3e-07 |
| Elf4 |  | C24A1.2 | 134 | 1.63 | 1.4e-07 |
| TBX20\_4 |  | tbx-38 mab-9 tbx-39 tbx-43 tbx-42 | 124 | 1.68 | 1.7e-07 |
| ELF2\_f1 |  | lin-1 C24A1.2 | 123 | 1.68 | 1.9e-07 |
| EPAS1\_si |  | Y5F2A.4 (0.54) ceh-9 hif-1 ztf-3 daf-12 | 107 | 1.78 | 2.3e-07 |
| pTH6747 |  | nhr-2 nhr-213 nhr-19 nhr-10 nhr-69 | 96 | 1.86 | 2.7e-07 |
| V$ZIC1\_01 |  | ztf-14 pax-3 lin-1 ref-2 ZC328.2 | 81 | 2.00 | 3.2e-07 |
| HepG2b\_TR4\_UCD |  | lin-1 nhr-19 C24A1.2 | 112 | 1.73 | 3.4e-07 |
| Etv6 |  | lin-1 C24A1.2 | 138 | 1.58 | 4.2e-07 |
| Elf2 |  | lin-1 F19F10.1 C24A1.2 | 131 | 1.61 | 5.0e-07 |
| pTH10623 |  | scrt-1 | 150 | 1.53 | 5.2e-07 |
| Oli\_da\_SANGER\_5\_3\_FBgn0032651 |  | ngn-1 hlh-32 hlh-8 hlh-15 hlh-12 | 120 | 1.66 | 6.1e-07 |
| Oli\_da\_SANGER\_5\_1\_FBgn0032651 |  | ngn-1 lin-31 hlh-32 hlh-8 hlh-15 | 108 | 1.73 | 6.2e-07 |
| Foxg1\_2 |  | nhr-213 lin-31 | 112 | 1.70 | 7.4e-07 |
| pTH2820 |  | ZC328.2 | 98 | 1.79 | 9.3e-07 |
| MA0600.1 |  | daf-19 (0.56) F52B5.7 | 92 | 1.84 | 1.1e-06 |
| MA0498.1 |  | lin-39 ceh-32 lin-32 | 113 | 1.68 | 1.2e-06 |
| V$SRF\_C |  | unc-120 | 57 | 2.32 | 1.2e-06 |
| ESRRA\_3 |  | nhr-2 nhr-68 nhr-213 nhr-71 nhr-6 nhr-10 Y67D8A.3 | 104 | 1.73 | 1.5e-06 |
| MYOG\_f1 |  | hlh-15 hlh-11 lin-32 hlh-1 hlh-14 | 96 | 1.79 | 1.5e-06 |
| SP3\_f1 |  | klf-2 ZC328.2 klf-1 | 98 | 1.78 | 1.5e-06 |
| MA0066.1 |  | nhr-71 nhr-43 | 81 | 1.92 | 1.8e-06 |
| MA0456.1 |  | ref-2 T22C8.4 | 111 | 1.68 | 2.0e-06 |
| pTH10630 |  | lsy-27 | 143 | 1.52 | 2.0e-06 |
| MA0470.1 |  | efl-1 F49E12.6 | 166 | 1.44 | 2.1e-06 |
| MA0163.1 |  | Y53H1A.2 (0.73) C09F5.3 (0.57) D1081.8 | 90 | 1.82 | 2.3e-06 |
| FLI1\_f1 |  | lin-1 | 158 | 1.46 | 2.7e-06 |
| V$PAX5\_02 |  | pax-2 pax-3 | 95 | 1.77 | 2.7e-06 |
| pTH6429 |  | nhr-2 nhr-177 F13H6.1 nhr-86 | 47 | 2.51 | 3.3e-06 |
| MA0544.1 |  | lin-22 gei-11 | 106 | 1.69 | 3.4e-06 |
| MA0543.1 |  | daf-8 eor-1 | 121 | 1.60 | 3.9e-06 |
| SOX2\_2 |  | dmd-4 sox-4 grh-1 | 100 | 1.72 | 4.2e-06 |
| MA0139.1 |  | Y5F2A.4 (0.54) F58G1.2 | 94 | 1.77 | 4.2e-06 |
| pTH9393 |  | F39B2.1 ZC416.1 | 92 | 1.78 | 4.4e-06 |
| MA0204.1 |  | dmd-4 ceh-32 | 126 | 1.57 | 5.0e-06 |
| YBOX1\_f2 |  | cey-3 dro-1 ceh-20 nfya-2 lin-31 | 102 | 1.70 | 5.4e-06 |
| K562\_ETS1\_HudsonAlpha |  | nhr-273 lin-1 odr-7 tbx-39 | 77 | 1.91 | 5.8e-06 |
| V$AHR\_01 |  | aha-1 ahr-1 | 70 | 1.98 | 7.6e-06 |
| HLH4C\_da\_SANGER\_5\_FBgn0011277 |  | hlh-2 ces-1 K02D7.2 hlh-8 hlh-15 hlh-1 | 113 | 1.62 | 8.3e-06 |
| MA0331.1 |  | unc-120 | 97 | 1.71 | 9.1e-06 |
| pTH6106 |  | nhr-182 | 160 | 1.43 | 9.6e-06 |
| Pbx1\_3203 |  | ceh-20 | 134 | 1.52 | 1.0e-05 |
| HEN1\_si |  | hlh-15 hlh-1 | 75 | 1.90 | 1.1e-05 |
| CG5669\_SANGER\_10\_FBgn0039169 |  | klf-2 klf-1 | 149 | 1.46 | 1.1e-05 |
| NR2F1\_3 |  | nhr-2 nhr-213 nhr-15 nhr-239 | 97 | 1.70 | 1.1e-05 |
| Irx6\_2623 |  | irx-1 | 111 | 1.62 | 1.2e-05 |
| pTH9934 |  | Y53H1A.2 (0.73) | 127 | 1.54 | 1.4e-05 |
| Nr2f6\_1 |  | nhr-2 nhr-62 nhr-19 | 96 | 1.70 | 1.4e-05 |
| MA0451.1 |  | nhr-2 php-3 | 111 | 1.61 | 1.4e-05 |
| TLX1\_f1 |  | ceh-19 | 97 | 1.69 | 1.5e-05 |
| Zbtb12\_2932 |  | lsy-27 | 88 | 1.75 | 1.7e-05 |
| pTH9164 |  | ceh-26 | 109 | 1.62 | 1.8e-05 |
| V$GR\_Q6 |  | nhr-255 | 143 | 1.47 | 1.8e-05 |
| V$NCX\_01 |  | ceh-19 | 123 | 1.55 | 1.8e-05 |
| V$YY1\_02 |  | lsy-2 | 98 | 1.67 | 2.0e-05 |
| Spdef |  | lin-1 | 137 | 1.48 | 2.2e-05 |
| FLI1\_4 |  | lin-1 | 92 | 1.71 | 2.3e-05 |
| pTH9080 |  | mnm-2 | 107 | 1.61 | 2.5e-05 |
| V$TAXCREB\_01 |  | crh-1 attf-1 | 122 | 1.54 | 2.8e-05 |
| Etv3 |  | lin-1 | 120 | 1.54 | 2.9e-05 |
| PTF1A\_f1 |  | hlh-2 lin-32 | 111 | 1.59 | 3.0e-05 |
| pTH10696 |  | Y44A6D.3 dpy-27 | 77 | 1.82 | 3.3e-05 |
| SMAD3\_1 |  | daf-8 hlh-8 sma-4 | 93 | 1.69 | 3.5e-05 |
| MA0015.1 |  | che-1 K11D2.4 tbp-1 | 72 | 1.87 | 3.6e-05 |
| Elf3\_3876 |  | C24A1.2 | 134 | 1.48 | 3.6e-05 |
| CREM\_f1 |  | crh-1 ceh-26 | 112 | 1.57 | 3.7e-05 |
| MA0161.1 |  | nfi-1 F49E12.6 | 111 | 1.58 | 3.9e-05 |
| MA0535.1 |  | pax-2 daf-8 F45H11.6 | 97 | 1.65 | 4.1e-05 |
| pTH5561 |  | nhr-239 | 120 | 1.53 | 4.2e-05 |
| F$MCM1\_01 |  | K02D7.2 unc-120 C24A1.2 | 84 | 1.75 | 4.2e-05 |
| Hnf4a\_2640 |  | nhr-62 | 91 | 1.69 | 4.3e-05 |
| HXB1\_f1 |  | ceh-20 lin-39 ceh-12 | 121 | 1.52 | 5.0e-05 |
| pTH5423 |  | klf-2 | 72 | 1.85 | 5.1e-05 |
| Mw137 |  | blmp-1 | 121 | 1.52 | 5.1e-05 |
| HXD13\_f1 |  | pal-1 | 114 | 1.55 | 5.4e-05 |
| MA0117.1 |  | pax-2 F45H11.6 | 96 | 1.65 | 5.8e-05 |
| SMAD3\_f1 |  | daf-8 | 145 | 1.43 | 6.4e-05 |
| CENPB\_1 |  | F21D5.4 F52B11.1 | 125 | 1.50 | 6.8e-05 |
| HES1\_f1 |  | lin-22 | 98 | 1.63 | 6.8e-05 |
| pTH9026 |  | attf-1 | 78 | 1.77 | 6.9e-05 |
| NR2F6\_f1 |  | nhr-2 nhr-62 nhr-239 | 136 | 1.46 | 7.0e-05 |
| LMX1A\_1 |  | ceh-14 (0.79) pha-2 (-0.7) ceh-53 ceh-30 ceh-31 lim-4 ceh-45 ceh-16 alr-1 ceh-10 ceh-43 egl-5 ceh-2 mls-2 lin-39 cog-1 ceh-23 ceh-18 lim-7 ceh-12 and 8 others  [full list] | 74 | 1.81 | 7.6e-05 |
| Hr46\_SANGER\_5\_FBgn0000448 |  | nhr-213 nhr-118 | 98 | 1.62 | 8.3e-05 |
| MA0146.2 |  | F58G1.2 aptf-1 | 104 | 1.58 | 8.8e-05 |
| LHX2\_f1 |  | ceh-14 (0.79) alr-1 cfi-1 ZC204.2 | 104 | 1.58 | 9.4e-05 |
| pTH1001 |  | dnj-17 | 132 | 1.46 | 9.8e-05 |
| Antp\_Cell\_FBgn0000095 |  | lin-39 lim-7 unc-86 | 99 | 1.61 | 1.0e-04 |
| Lmx1a\_2238 |  | ceh-16 lim-7 lim-6 | 87 | 1.68 | 1.0e-04 |
| pTH10779 |  | nhr-182 nhr-134 | 120 | 1.50 | 1.0e-04 |
| pTH6486 |  | nhr-145 | 167 | 1.35 | 1.1e-04 |
| pTH10031 |  | mbr-1 | 70 | 1.82 | 1.1e-04 |
| pTH10638 |  | dmd-3 C34D1.1 | 63 | 1.91 | 1.1e-04 |
| Mrg1\_2246 |  | ceh-20 ces-1 ceh-32 F55C5.11 | 119 | 1.51 | 1.2e-04 |
| CG33980\_SOLEXA\_2\_10\_FBgn0053980 |  | eyg-1 ceh-45 alr-1 ceh-10 lin-39 ceh-1 | 92 | 1.64 | 1.2e-04 |
| TBX2\_f1 |  | ztf-6 tbx-39 | 97 | 1.61 | 1.2e-04 |
| MA0254.1 |  | ceh-18 unc-86 | 106 | 1.56 | 1.3e-04 |
| Smad3\_3805 |  | daf-8 | 135 | 1.44 | 1.4e-04 |
| bin\_FlyReg\_FBgn0045759 |  | fkh-8 (0.63) fkh-7 fkh-10 daf-16 lin-31 let-381 | 99 | 1.59 | 1.4e-04 |
| tin\_FlyReg\_FBgn0004110 |  | ceh-22 (-0.51) ceh-24 dsc-1 | 145 | 1.41 | 1.4e-04 |
| FOXO6\_1 |  | fkh-8 (0.63) fkh-7 daf-16 lin-31 let-381 | 112 | 1.53 | 1.5e-04 |
| pTH5887 |  | lin-39 | 92 | 1.63 | 1.5e-04 |
| Emx2\_3420 |  | ceh-2 | 39 | 2.36 | 1.8e-04 |
| Eip93F\_SANGER\_10\_FBgn0013948 |  | mbr-1 F26F4.8 | 120 | 1.49 | 1.8e-04 |
| I$KR\_01 |  | ZK177.3 B0310.2 | 136 | 1.43 | 1.8e-04 |
| pTH10810 |  | syd-9 (0.55) lsy-2 lsl-1 | 75 | 1.75 | 1.9e-04 |
| pTH10772 |  | ceh-52 | 82 | 1.69 | 1.9e-04 |
| Six4\_2860 |  | ceh-32 | 93 | 1.61 | 2.0e-04 |
| V$MEF2\_02 |  | mef-2 | 75 | 1.74 | 2.0e-04 |
| pTH9182 |  | tbx-39 | 134 | 1.43 | 2.1e-04 |
| pTH10798 |  | Y75B8A.6 | 96 | 1.59 | 2.2e-04 |
| tgo\_trh\_SANGER\_5\_FBgn0015014 |  | pax-1 mdl-1 aha-1 hlh-30 | 70 | 1.78 | 2.3e-04 |
| T-47D\_FOXA1\_HudsonAlpha |  | lin-31 let-381 | 132 | 1.44 | 2.3e-04 |
| Hmbox1\_2674 |  | hmbx-1 | 103 | 1.55 | 2.5e-04 |
| pTH9974 |  | hlh-16 ngn-1 hlh-32 | 79 | 1.70 | 2.6e-04 |
| Osr2\_1727 |  | odd-1 (-0.6) odd-2 | 87 | 1.64 | 2.6e-04 |
| pTH9096 |  | T07C12.11 | 113 | 1.50 | 2.8e-04 |
| V$ZID\_01 |  | skn-1 ztf-28 | 105 | 1.54 | 2.8e-04 |
| ZNF75A\_1 |  | ztf-3 F26F4.8 lag-1 | 123 | 1.46 | 2.8e-04 |
| Six3\_1732 |  | ceh-34 | 82 | 1.67 | 3.2e-04 |
| disco-r-Cl1\_SANGER\_5\_FBgn0042650 |  | nhr-68 lin-31 F55C5.11 | 110 | 1.51 | 3.2e-04 |
| Elf5 |  | C24A1.2 | 123 | 1.46 | 3.4e-04 |
| MA0078.1 |  | sox-4 gei-3 pop-1 | 122 | 1.46 | 3.4e-04 |
| Pou2f1\_3081 |  | ceh-18 | 93 | 1.59 | 3.5e-04 |
| pTH9381 |  | ceh-18 | 105 | 1.53 | 3.6e-04 |
| pTH9244 |  | tbx-39 | 136 | 1.41 | 3.6e-04 |
| pTH1292 |  | ceh-24 pzf-1 | 116 | 1.48 | 3.7e-04 |
| MA0547.1 |  | ceh-2 skn-1 | 146 | 1.38 | 3.8e-04 |
| V$MYB\_Q6 |  | D1081.8 | 119 | 1.46 | 4.3e-04 |
| pTH4381 |  | lin-22 ref-1 mxl-1 aha-1 hlh-26 | 89 | 1.60 | 4.4e-04 |
| pTH7032 |  | F52B11.1 | 80 | 1.66 | 4.8e-04 |
| Atf1\_3026 |  | crh-1 | 113 | 1.48 | 5.0e-04 |
| CG4854\_SANGER\_10\_FBgn0038766 |  | K11D2.4 | 90 | 1.59 | 5.0e-04 |
| Spdef\_0905 |  | lin-1 | 93 | 1.57 | 5.3e-04 |
| ARNT2\_si |  | aha-1 C46E10.9 | 85 | 1.62 | 5.4e-04 |
| pTH9155 |  | B0310.2 D1081.8 lin-48 | 76 | 1.68 | 5.5e-04 |
| Vsx1\_1728 |  | alr-1 | 92 | 1.58 | 5.5e-04 |
| pTH1014 |  | atf-5 | 69 | 1.74 | 5.8e-04 |
| Hr51\_SANGER\_5\_FBgn0034012 |  | nhr-100 sox-4 pop-1 | 120 | 1.45 | 6.1e-04 |
| MA0505.1 |  | nhr-68 | 86 | 1.61 | 6.2e-04 |
| Elf3 |  | C24A1.2 | 125 | 1.43 | 6.3e-04 |
| V$YY1\_01 |  | lsy-2 | 106 | 1.50 | 6.4e-04 |
| pTH2283 |  | odd-2 | 128 | 1.42 | 6.4e-04 |
| MA0244.1 |  | C48E7.11 | 133 | 1.40 | 6.7e-04 |
| pTH9925 |  | nhr-100 ztf-11 | 126 | 1.42 | 6.8e-04 |
| pTH9394 |  | klf-2 klf-1 sptf-3 | 138 | 1.39 | 7.0e-04 |
| Mw142 |  | elt-1 elt-7 egl-27 | 122 | 1.44 | 7.0e-04 |
| MA0027.1 |  | ceh-16 | 127 | 1.42 | 7.1e-04 |
| pTH10768 |  | med-2 | 66 | 1.75 | 7.4e-04 |
| ovo\_FlyReg\_FBgn0003028 |  | dmd-3 pax-3 lin-48 | 49 | 1.97 | 7.7e-04 |
| srp\_FlyReg\_FBgn0003507 |  | lin-39 elt-1 | 120 | 1.44 | 7.8e-04 |
| pTH5916 |  | efl-2 | 122 | 1.43 | 7.9e-04 |
| Slou\_Cell\_FBgn0002941 |  | ceh-30 ceh-31 ceh-24 alr-1 ceh-9 ceh-43 ceh-8 lin-39 cog-1 lim-7 ceh-19 ceh-1 | 93 | 1.55 | 7.9e-04 |
| Barhl2\_3868 |  | ceh-31 ceh-16 alr-1 ceh-43 ceh-9 lim-7 ceh-1 | 63 | 1.78 | 8.3e-04 |
| pTH8649 |  | mbr-1 | 110 | 1.47 | 8.5e-04 |
| pTH9108 |  | nhr-5 daf-12 | 107 | 1.48 | 8.9e-04 |
| V$DELTAEF1\_01 |  | ztf-6 hlh-2 | 74 | 1.67 | 9.4e-04 |
| MA0007.2 |  | npax-1 nhr-255 lin-14 | 102 | 1.50 | 9.4e-04 |
| Hoxd3\_1742 |  | ceh-53 ceh-45 alr-1 ceh-43 lin-39 ceh-18 ceh-12 npax-3 ceh-1 | 92 | 1.55 | 9.5e-04 |
| Nkx3-1\_2923 |  | ceh-24 dsc-1 | 119 | 1.44 | 9.6e-04 |
| HLH25 |  | lin-22 aha-1 hlh-27 hlh-28 | 91 | 1.56 | 9.8e-04 |
| pTH9290 |  | ceh-18 ceh-6 tbp-1 | 75 | 1.66 | 1.0e-03 |
| pTH6478 |  | lim-7 | 88 | 1.57 | 1.0e-03 |
| V$AREB6\_02 |  | ztf-6 | 93 | 1.54 | 1.0e-03 |
| CUX1\_2 |  | ceh-48 (0.59) | 104 | 1.49 | 1.0e-03 |
| pTH5257 |  | C48E7.11 | 100 | 1.51 | 1.1e-03 |
| V$OCT1\_03 |  | ceh-18 | 85 | 1.58 | 1.1e-03 |
| pTH8411 |  | tbx-39 | 72 | 1.68 | 1.1e-03 |
| pTH3751 |  | tbx-39 | 103 | 1.49 | 1.1e-03 |
| Hoxb7\_3953 |  | lin-39 ceh-18 ceh-6 | 94 | 1.53 | 1.1e-03 |
| pTH9049 |  | ztf-2 | 46 | 1.99 | 1.2e-03 |
| pTH5714 |  | nhr-239 | 105 | 1.48 | 1.2e-03 |
| pTH1294 |  | mel-28 | 73 | 1.66 | 1.2e-03 |
| pTH5928 |  | ceh-34 | 101 | 1.50 | 1.3e-03 |
| CEBPE\_f1 |  | C48E7.11 | 65 | 1.72 | 1.4e-03 |
| pTH9913 |  | skn-1 | 143 | 1.35 | 1.4e-03 |
| pTH10823 |  | B0310.2 | 91 | 1.54 | 1.4e-03 |
| MA0235.1 |  | ceh-48 (0.59) dsc-1 | 131 | 1.38 | 1.4e-03 |
| HeLa-S3\_ZNF274\_UCD |  | C28G1.4 | 102 | 1.49 | 1.4e-03 |
| pTH5812 |  | ceh-14 (0.79) | 73 | 1.65 | 1.4e-03 |
| V$PAX2\_02 |  | pax-1 | 97 | 1.51 | 1.5e-03 |
| pTH6445 |  | ceh-5 | 33 | 2.30 | 1.5e-03 |
| pTH10041 |  | ztf-29 | 132 | 1.38 | 1.5e-03 |
| Rfxdc2\_3516 |  | daf-19 (0.56) mab-3 | 86 | 1.56 | 1.5e-03 |
| Tcf7l2\_3461 |  | ceh-20 pop-1 | 127 | 1.39 | 1.5e-03 |
| pTH8991 |  | cey-3 | 124 | 1.40 | 1.5e-03 |
| pTH3998 |  | tbx-39 | 154 | 1.32 | 1.6e-03 |
| V$CETS1P54\_02 |  | C52B9.2 | 154 | 1.32 | 1.6e-03 |
| MA0224.1 |  | alr-1 lin-39 ceh-18 ceh-12 pal-1 | 89 | 1.54 | 1.6e-03 |
| MA0482.1 |  | ztf-29 elt-1 | 127 | 1.39 | 1.6e-03 |
| pTH5083 |  | fos-1 | 100 | 1.49 | 1.6e-03 |
| pTH5078 |  | ces-2 | 84 | 1.57 | 1.6e-03 |
| MA0486.1 |  | Y53C10A.3 | 97 | 1.50 | 1.6e-03 |
| pTH2933 |  | F58G1.2 | 75 | 1.63 | 1.7e-03 |
| V$BRN2\_01 |  | ceh-18 | 122 | 1.41 | 1.7e-03 |
| CG14962\_SANGER\_5\_FBgn0035407 |  | ceh-24 ces-1 C34H4.5 T22H9.4 | 121 | 1.41 | 1.7e-03 |
| pTH6508 |  | nhr-36 | 112 | 1.44 | 1.7e-03 |
| pTH9300 |  | dmd-3 C34D1.1 | 109 | 1.45 | 1.8e-03 |
| pTH8679 |  | pax-2 | 89 | 1.54 | 1.8e-03 |
| Lbx2\_3869 |  | mls-2 | 42 | 2.02 | 1.9e-03 |
| pTH9246 |  | lin-31 let-381 C34D1.1 | 76 | 1.61 | 2.0e-03 |
| pTH9220 |  | mbr-1 | 111 | 1.44 | 2.0e-03 |
| pTH9219 |  | xbp-1 C01B12.2 | 94 | 1.51 | 2.0e-03 |
| Vax1\_3499 |  | C02F12.10 | 23 | 2.77 | 2.1e-03 |
| V$AREB6\_04 |  | ztf-6 C34D1.1 gei-11 | 199 | 1.23 | 2.1e-03 |
| pTH10811 |  | nhr-142 nhr-216 nhr-84 | 116 | 1.42 | 2.1e-03 |
| Hoxc10\_2 |  | ceh-24 lin-39 pal-1 php-3 | 124 | 1.39 | 2.1e-03 |
| V$EN1\_01 |  | ceh-16 atf-2 | 45 | 1.95 | 2.1e-03 |
| MA0124.1 |  | ceh-24 | 77 | 1.60 | 2.2e-03 |
| pTH5877 |  | nhr-7 (-0.54) nhr-100 elt-1 | 126 | 1.39 | 2.2e-03 |
| MA0150.2 |  | sknr-1 fos-1 jun-1 crh-1 F45H11.6 | 91 | 1.52 | 2.2e-03 |
| Hmx1\_3423 |  | ceh-9 | 101 | 1.47 | 2.2e-03 |
| V$POU3F2\_01 |  | ceh-18 | 113 | 1.43 | 2.3e-03 |
| pTH9198 |  | dmd-3 | 108 | 1.44 | 2.3e-03 |
| pTH9969 |  | pag-3 | 114 | 1.42 | 2.3e-03 |
| Poxm\_SOLEXA\_5\_FBgn0003129 |  | pax-2 | 107 | 1.45 | 2.3e-03 |
| MA0165.1 |  | ceh-24 T27F2.4 | 68 | 1.66 | 2.4e-03 |
| sqz\_SANGER\_5\_FBgn0010768 |  | fkh-7 lin-29 mel-28 | 146 | 1.33 | 2.4e-03 |
| Evx1\_3952 |  | ceh-53 | 21 | 2.89 | 2.5e-03 |
| HXC6\_f1 |  | lin-39 lin-1 | 132 | 1.36 | 2.5e-03 |
| FOXO1\_si |  | fkh-9 irx-1 daf-16 | 139 | 1.35 | 2.6e-03 |
| pTH6436 |  | ceh-53 | 23 | 2.71 | 2.6e-03 |
| pTH5169 |  | cfi-1 | 69 | 1.65 | 2.6e-03 |
| V$GATA1\_03 |  | elt-1 | 70 | 1.64 | 2.6e-03 |
| Pou3f4\_3773 |  | ceh-6 | 92 | 1.50 | 2.7e-03 |
| pTH10013 |  | nhr-168 | 111 | 1.42 | 2.8e-03 |
| V$TCF11\_01 |  | skn-1 | 27 | 2.45 | 2.9e-03 |
| pTH10718 |  | egl-43 | 112 | 1.42 | 2.9e-03 |
| Atf6\_SANGER\_5\_FBgn0033010 |  | fos-1 atf-6 atf-7 C27D6.4 | 138 | 1.34 | 2.9e-03 |
| pTH5778 |  | egl-5 | 94 | 1.49 | 3.1e-03 |
| I$CROC\_01 |  | mef-2 mel-28 let-381 | 104 | 1.44 | 3.4e-03 |
| pTH2684 |  | fos-1 | 146 | 1.32 | 3.4e-03 |
| V$IK2\_01 |  | F26F4.8 | 84 | 1.53 | 3.5e-03 |
| pTH10797 |  | lin-29 K11D2.4 | 109 | 1.42 | 3.6e-03 |
| Nkx1-2\_3214 |  | ceh-30 | 83 | 1.54 | 3.6e-03 |
| pTH9137 |  | nhr-65 | 130 | 1.36 | 3.8e-03 |
| Pknox2\_3077 |  | ceh-32 | 141 | 1.33 | 3.9e-03 |
| pTH9165 |  | ztf-27 | 126 | 1.36 | 4.1e-03 |
| pTH9215 |  | C34D1.1 | 105 | 1.43 | 4.1e-03 |
| Gsh2\_3990 |  | ceh-31 | 21 | 2.76 | 4.1e-03 |
| Hoxb5\_3122 |  | lin-39 | 20 | 2.85 | 4.2e-03 |
| pTH10650 |  | nhr-153 | 108 | 1.42 | 4.3e-03 |
| Hoxa2\_3079 |  | lin-39 | 18 | 3.02 | 4.7e-03 |
| Six1\_0935 |  | ceh-32 | 95 | 1.46 | 4.8e-03 |
| pTH5781 |  | ceh-32 | 82 | 1.53 | 4.8e-03 |
| HepG2\_HSF1\_Stanford |  | Y53C10A.3 | 76 | 1.56 | 4.8e-03 |
| pTH8671 |  | attf-1 | 96 | 1.46 | 4.8e-03 |
| Cutl1\_3494 |  | ceh-44 (0.51) | 126 | 1.36 | 5.0e-03 |
| VDR\_1 |  | cfi-1 gei-3 nhr-208 | 81 | 1.53 | 5.0e-03 |
| Pou2f2\_3748 |  | alr-1 ceh-18 lim-7 | 91 | 1.48 | 5.1e-03 |
| pTH5119 |  | cfi-1 | 122 | 1.37 | 5.4e-03 |
| V$FAC1\_01 |  | gei-8 | 85 | 1.50 | 5.4e-03 |
| EN1\_2 |  | ceh-16 ceh-2 | 85 | 1.50 | 5.6e-03 |
| pTH9254 |  | mel-28 | 143 | 1.31 | 5.8e-03 |
| Hoxd9\_1 |  | lin-39 | 111 | 1.40 | 5.8e-03 |
| pTH9043 |  | sem-2 | 155 | 1.28 | 6.4e-03 |
| FOXC2\_f1 |  | ceh-20 cfi-1 let-381 | 98 | 1.44 | 6.5e-03 |
| V$GATA1\_01 |  | elt-1 | 102 | 1.42 | 6.5e-03 |
| YY1\_1 |  | lsy-2 | 95 | 1.45 | 6.9e-03 |
| pTH10788 |  | tbx-33 | 119 | 1.36 | 7.0e-03 |
| Sox15\_3457 |  | sox-4 | 111 | 1.39 | 7.0e-03 |
| T-47D\_GATA3\_HudsonAlpha |  | elt-1 | 98 | 1.43 | 7.1e-03 |
| pTH2936 |  | nhr-239 | 96 | 1.44 | 7.2e-03 |
| pTH5891 |  | nhr-49 | 88 | 1.47 | 7.3e-03 |
| MA0032.1 |  | let-381 | 79 | 1.52 | 7.3e-03 |
| Gmeb1\_1745 |  | attf-1 | 94 | 1.44 | 8.0e-03 |
| Tcf3\_3787 |  | pop-1 | 128 | 1.34 | 8.1e-03 |
| Srf\_3509 |  | unc-120 | 108 | 1.39 | 8.2e-03 |
| pTH10038 |  | sox-4 F56D1.1 gei-3 | 72 | 1.55 | 8.4e-03 |
| pTH3997 |  | C04F5.9 | 66 | 1.59 | 8.4e-03 |
| pTH9052 |  | atf-2 ces-2 Y51H4A.4 C48E7.11 | 117 | 1.36 | 8.4e-03 |
| pTH9242 |  | mel-28 | 91 | 1.45 | 8.4e-03 |
| Hoxb3\_1720 |  | lin-39 | 40 | 1.89 | 8.6e-03 |
| pTH6268 |  | ceh-2 | 60 | 1.64 | 9.2e-03 |
| pTH7875 |  | mel-28 | 64 | 1.60 | 9.2e-03 |
| pTH6569 |  | ceh-43 | 83 | 1.48 | 9.4e-03 |
| Irx3\_0920 |  | irx-1 | 73 | 1.54 | 9.6e-03 |
| pTH2846 |  | lin-31 | 133 | 1.32 | 9.7e-03 |
| Hoxa4\_3426 |  | lin-39 | 41 | 1.86 | 9.8e-03 |
| pTH10805 |  | ztf-16 | 86 | 1.47 | 1.0e-02 |
| V$FOXJ2\_02 |  | lin-31 | 104 | 1.40 | 1.0e-02 |
| pTH10777 |  | dmd-3 | 128 | 1.33 | 1.0e-02 |
| Nkx1-1\_3856 |  | ceh-30 | 70 | 1.55 | 1.0e-02 |
| Vax2\_3500 |  | C02F12.10 | 51 | 1.71 | 1.0e-02 |
| I$UBX\_01 |  | lin-39 | 99 | 1.41 | 1.0e-02 |
| Lhx1\_2240 |  | lim-7 | 89 | 1.45 | 1.1e-02 |
| pTH6423 |  | pha-2 (-0.7) | 119 | 1.35 | 1.1e-02 |
| HIF1A\_si |  | hif-1 | 80 | 1.49 | 1.1e-02 |
| Dlx3\_1030 |  | ceh-43 | 85 | 1.46 | 1.1e-02 |
| pTH8556 |  | pax-2 | 78 | 1.50 | 1.1e-02 |
| Cdx1\_2245 |  | ceh-13 | 98 | 1.41 | 1.2e-02 |
| pTH3819 |  | ceh-18 | 46 | 1.77 | 1.2e-02 |
| Sox1\_2631 |  | sox-4 | 88 | 1.45 | 1.2e-02 |
| Hoxa5\_3415 |  | lin-39 | 19 | 2.65 | 1.2e-02 |
| pTH6562 |  | ceh-5 | 83 | 1.47 | 1.3e-02 |
| V$OCT1\_06 |  | ztf-9 ceh-18 | 45 | 1.77 | 1.3e-02 |
| Irx3\_1 |  | irx-1 | 46 | 1.76 | 1.3e-02 |
| Six2\_2307 |  | ceh-34 ceh-32 | 39 | 1.86 | 1.3e-02 |
| Ptx1\_SOLEXA\_FBgn0020912 |  | ceh-53 ceh-45 alr-1 | 124 | 1.33 | 1.4e-02 |
| MA0483.1 |  | odd-1 (-0.6) | 105 | 1.38 | 1.4e-02 |
| pTH5118 |  | cfi-1 | 115 | 1.35 | 1.4e-02 |
| eve\_FlyReg\_FBgn0000606 |  | ceh-53 ceh-43 | 105 | 1.38 | 1.4e-02 |
| pTH9245 |  | ceh-18 | 57 | 1.63 | 1.4e-02 |
| pTH6497 |  | lin-31 | 134 | 1.30 | 1.5e-02 |
| Hoxb4\_2627 |  | lin-39 | 79 | 1.48 | 1.5e-02 |
| pTH9076 |  | C01G12.1 | 77 | 1.49 | 1.5e-02 |
| pTH10769 |  | Y48G1C.6 | 154 | 1.26 | 1.5e-02 |
| Zfp161\_2858 |  | pzf-1 | 198 | 1.19 | 1.5e-02 |
| pTH5690 |  | ceh-32 | 119 | 1.33 | 1.5e-02 |
| BARHL2\_4 |  | ceh-31 | 116 | 1.34 | 1.5e-02 |
| pTH6641 |  | lin-31 | 132 | 1.30 | 1.5e-02 |
| Sox17\_2837 |  | sox-4 | 85 | 1.45 | 1.6e-02 |
| pTH6591 |  | lin-31 | 133 | 1.30 | 1.6e-02 |
| pTH3064 |  | crh-1 | 139 | 1.29 | 1.7e-02 |
| CG8765\_SANGER\_5\_FBgn0036900 |  | H20J04.3 | 82 | 1.46 | 1.7e-02 |
| Spt15 |  | tbp-1 | 51 | 1.67 | 1.7e-02 |
| Pou3f3\_3235 |  | ceh-6 | 38 | 1.85 | 1.7e-02 |
| pTH9353 |  | ceh-51 (-0.59) | 81 | 1.46 | 1.8e-02 |
| Barx1\_2877 |  | ceh-43 | 83 | 1.45 | 1.8e-02 |
| pTH5924 |  | nhr-255 | 94 | 1.40 | 1.9e-02 |
| CG31670\_SANGER\_5\_FBgn0031375 |  | F21A9.2 CELE\_Y38H8A.5 | 128 | 1.30 | 1.9e-02 |
| pTH10028 |  | nhr-204 | 95 | 1.40 | 1.9e-02 |
| Dlx2\_2273 |  | ceh-43 | 84 | 1.44 | 1.9e-02 |
| pTH6449 |  | ceh-43 | 41 | 1.79 | 1.9e-02 |
| MA0058.2 |  | mxl-1 irx-1 | 100 | 1.38 | 1.9e-02 |
| pTH9125 |  | egl-13 K11D2.4 | 68 | 1.52 | 1.9e-02 |
| Dlx1\_1741 |  | ceh-43 | 86 | 1.43 | 2.0e-02 |
| pTH5914 |  | attf-1 | 52 | 1.64 | 2.0e-02 |
| Hoxa3\_2783 |  | lin-39 | 56 | 1.60 | 2.1e-02 |
| Pou2f3\_3986 |  | ceh-18 | 45 | 1.71 | 2.2e-02 |
| Prop1\_3949 |  | ceh-53 ceh-16 | 80 | 1.45 | 2.2e-02 |
| Hoxa7\_2668 |  | lin-39 | 83 | 1.44 | 2.2e-02 |
| pTH4325 |  | ceh-18 | 37 | 1.84 | 2.3e-02 |
| V$GATA1\_04 |  | elt-1 | 91 | 1.40 | 2.3e-02 |
| Hoxa7\_3750 |  | lin-39 | 85 | 1.42 | 2.6e-02 |
| pTH5808 |  | pal-1 | 100 | 1.36 | 2.6e-02 |
| pTH8745 |  | attf-1 | 86 | 1.41 | 2.7e-02 |
| Hoxa9\_2622 |  | lin-39 | 96 | 1.38 | 2.7e-02 |
| pTH9135 |  | pop-1 | 126 | 1.29 | 2.8e-02 |
| Irx5\_2385 |  | irx-1 | 32 | 1.90 | 3.0e-02 |
| pTH9216 |  | ceh-18 | 21 | 2.29 | 3.0e-02 |
| pTH8318 |  | attf-1 | 89 | 1.39 | 3.4e-02 |
| Cdx2\_4272 |  | ceh-13 | 96 | 1.36 | 3.4e-02 |
| Hoxc4\_3491 |  | lin-39 | 77 | 1.44 | 3.5e-02 |
| pTH10647 |  | nhr-232 | 81 | 1.42 | 3.5e-02 |
| MCR\_f1 |  | nhr-255 | 156 | 1.23 | 3.5e-02 |
| Hoxd1\_3448 |  | ceh-12 | 56 | 1.56 | 3.5e-02 |
| pTH9149 |  | ztf-30 | 181 | 1.19 | 3.6e-02 |
| pTH6447 |  | ceh-19 | 64 | 1.50 | 3.8e-02 |
| pTH9384 |  | cfi-1 | 116 | 1.30 | 3.8e-02 |
| MA0467.1 |  | ceh-45 tbx-39 | 117 | 1.30 | 3.8e-02 |
| rn\_SOLEXA\_5\_FBgn0259172 |  | lin-29 | 116 | 1.30 | 3.9e-02 |
| Irx2\_0900 |  | irx-1 | 81 | 1.41 | 4.0e-02 |
| Tcf1\_2666 |  | hmbx-1 | 83 | 1.40 | 4.0e-02 |
| Pou3f1\_3819 |  | ceh-6 | 86 | 1.39 | 4.1e-02 |
| MSX2\_1 |  | ceh-1 | 84 | 1.39 | 4.3e-02 |
| V$GATA3\_01 |  | elt-1 | 116 | 1.30 | 4.5e-02 |
| Bsx\_3483 |  | ceh-31 | 78 | 1.41 | 4.6e-02 |
| pTH2280 |  | mnm-2 | 90 | 1.37 | 4.7e-02 |
| MA0173.1 |  | irx-1 | 95 | 1.35 | 4.8e-02 |
| MA0262.1 |  | hsf-1 mab-3 | 44 | 1.64 | 4.8e-02 |
| pTH9387 |  | C34D1.1 | 58 | 1.52 | 4.9e-02 |

### Correlated (and anti-correlated) transcription factors

|  |  |
| --- | --- |
| **Transcription factor** | **Correlation** |
| nhr-85 | 0.93 |
| Y55F3AM.14 | 0.87 |
| nhr-157 | 0.84 |
| ceh-14 | 0.79 |
| nhr-119 | 0.76 |
| madf-5 | 0.74 |
| Y53H1A.2 | 0.73 |
| C34D10.2 | 0.72 |
| nhr-190 | 0.70 |
| nhr-241 | 0.70 |
| T07F8.4 | 0.68 |
| tra-1 | 0.67 |
| nhr-95 | 0.67 |
| irld-33 | 0.66 |
| gmeb-1 | 0.66 |
| W02D7.6 | 0.66 |
| F38C2.7 | 0.65 |
| fkh-8 | 0.63 |
| mbl-1 | 0.63 |
| Y17G7B.22 | 0.63 |
| ceh-41 | 0.62 |
| ZK673.4 | 0.61 |
| mex-1 | 0.61 |
| nhr-247 | 0.60 |
| spr-1 | 0.60 |
| nhr-122 | -0.50 |
| mef-2 | -0.50 |
| ceh-60 | -0.50 |
| gla-3 | -0.50 |
| nhr-64 | -0.51 |
| ceh-22 | -0.51 |
| Y57A10A.31 | -0.51 |
| ets-4 | -0.52 |
| nhr-60 | -0.53 |
| nhr-7 | -0.54 |
| nhr-76 | -0.55 |
| gmeb-2 | -0.56 |
| sup-35 | -0.56 |
| cky-1 | -0.56 |
| pha-4 | -0.58 |
| ceh-51 | -0.59 |
| odd-1 | -0.60 |
| nhr-88 | -0.61 |
| ccch-5 | -0.64 |
| fkh-6 | -0.65 |
| cebp-1 | -0.68 |
| pha-2 | -0.70 |
| nhr-106 | -0.72 |
| Y48A6C.1 | -0.84 |
| nhr-74 | -0.95 |

### ChIP peaks enriched

|  |  |  |  |  |
| --- | --- | --- | --- | --- |
| **Gene** | **Experiment** | **Number of upstream peaks** | **Enrichment** | **FDR corrected p** |
| pes-1 | PES-1\_Larvae-L4-stage | 57 | 2.17 | 1.7e-06 |
| ceh-16 | CEH-16\_Larvae-L2-stage | 34 | 2.93 | 1.9e-06 |
| ceh-26 | CEH-26\_Late-Embryonic-stage | 52 | 2.17 | 7.3e-06 |
| ces-1 | CES-1\_Embryos | 61 | 1.90 | 4.5e-05 |
| ham-1 | HAM-1\_Larvae-L4-stage | 61 | 1.90 | 4.6e-05 |
| lsy-2 | LSY-2\_Larvae-L1-stage | 66 | 1.82 | 6.2e-05 |
| nfya-1 | NFYA-1\_Late-Embryos | 52 | 1.98 | 9.6e-05 |
| mab-5 | MAB-5\_Larvae-L2-stage | 33 | 2.46 | 1.1e-04 |
| lsy-2 | LSY-2\_Fed-L1-stage-larvae | 48 | 1.87 | 9.7e-04 |
| ces-1 | CES-1\_Larvae-L3-stage | 9 | 5.63 | 1.6e-03 |
| hlh-30 | HLH-30\_Late-Embryos | 28 | 2.32 | 1.6e-03 |
| pha-4 | PHA-4\_Larvae-L4-stage | 36 | 2.01 | 2.5e-03 |
| dpl-1 | DPL-1\_Larvae-L4-stage | 57 | 1.68 | 2.7e-03 |
| nhr-2 | NHR-2\_Embryos | 23 | 2.41 | 4.3e-03 |
| unc-62 | UNC-62\_Larvae-L3-stage | 58 | 1.64 | 4.6e-03 |
| peb-1 | PEB-1\_Larvae-L2-stage | 35 | 1.96 | 4.7e-03 |
| nhr-129 | NHR-129\_Larvae-L2-stage | 68 | 1.55 | 5.2e-03 |
| egl-5 | EGL-5\_Larvae-L3-stage | 44 | 1.78 | 6.0e-03 |
| zag-1 | ZAG-1\_Larvae-L2-stage | 40 | 1.80 | 8.8e-03 |
| sax-3 | SAX-3\_Larvae-L4-stage | 56 | 1.60 | 9.7e-03 |
| fos-1 | FOS-1\_Fed-L1-stage-larvae | 42 | 1.75 | 1.1e-02 |
| R02D3.7 | R02D3.7\_Larvae-L3-stage | 50 | 1.64 | 1.2e-02 |
| aly-2 | ALY-2\_Larvae-L3-stage | 6 | 6.26 | 1.5e-02 |
| lsy-2 | LSY-2\_Embryos | 28 | 1.99 | 1.7e-02 |
| sem-4 | SEM-4\_Larvae-L2-stage | 50 | 1.59 | 2.5e-02 |
| nfya-1 | NFYA-1\_Larvae-L3-stage | 33 | 1.79 | 3.0e-02 |
| gei-11 | GEI-11\_Larvae-L3-stage | 38 | 1.70 | 3.1e-02 |
| ztf-11 | ZTF-11\_Embryos | 18 | 2.30 | 3.2e-02 |
| hpl-2 | HPL-2\_Fed-L1-stage-larvae | 50 | 1.54 | 4.3e-02 |
| fos-1 | FOS-1\_Larvae-L2-stage | 51 | 1.53 | 4.3e-02 |
| unc-62 | UNC-62\_Larvae-L2-stage | 40 | 1.63 | 4.8e-02 |
