# Supplementary material for: Overlapping cell population expression profiling and regulatory inference in C. elegans
Source: BMC Genomics. 2016 Feb 29;17:159. doi: 10.1186/s12864-016-2482-z (PMC4772325; doi:10.1186/s12864-016-2482-z)
Supplement: Additional file 13: — Web supplement. (DOC 21 kb) [file 12864_2016_2482_MOESM13_ESM.zip › sortWeb/clusters/hier.300.clusters/181.html]

Cluster 181 

## Cluster 181

### Expression

| cnd-1 rep. 1 | cnd-1 rep. 2 | cnd-1 rep. 3 | pha-4 rep. 1 | pha-4 rep. 2 | pha-4 rep. 3 | ceh-27 | ceh-36 | ceh-6 | F21D5.9 | mir-57 | mls-2 | pal-1 | pros-1 | ttx-3 | unc-130 | hlh-16 | irx-1 | ceh-6 (+) hlh-16 (+) | ceh-6 (+) hlh-16 (-) | ceh-6 (-) hlh-16 (+) | cnd-1 singlets | pha-4 singlets | 0 | 60 | 120 | 150 | 180 | 240 | 330 | 390 | 420 | 480 | 540 | 570 | 600 | 630 | 660 | NAME | Functional description |
| --- | --- | --- | --- | --- | --- | --- | --- | --- | --- | --- | --- | --- | --- | --- | --- | --- | --- | --- | --- | --- | --- | --- | --- | --- | --- | --- | --- | --- | --- | --- | --- | --- | --- | --- | --- | --- | --- | --- | --- |
|  |  |  |  |  |  |  |  |  |  |  |  |  |  |  |  |  |  |  |  |  |  |  |  |  |  |  |  |  |  |  |  |  |  |  |  |  |  | K09E9.5 |  |
|  |  |  |  |  |  |  |  |  |  |  |  |  |  |  |  |  |  |  |  |  |  |  |  |  |  |  |  |  |  |  |  |  |  |  |  |  |  | F58D5.5 |  |
|  |  |  |  |  |  |  |  |  |  |  |  |  |  |  |  |  |  |  |  |  |  |  |  |  |  |  |  |  |  |  |  |  |  |  |  |  |  | K07C10.6 |  |
|  |  |  |  |  |  |  |  |  |  |  |  |  |  |  |  |  |  |  |  |  |  |  |  |  |  |  |  |  |  |  |  |  |  |  |  |  |  | C53B7.8 |  |
|  |  |  |  |  |  |  |  |  |  |  |  |  |  |  |  |  |  |  |  |  |  |  |  |  |  |  |  |  |  |  |  |  |  |  |  |  |  | C06G1.11 |  |
|  |  |  |  |  |  |  |  |  |  |  |  |  |  |  |  |  |  |  |  |  |  |  |  |  |  |  |  |  |  |  |  |  |  |  |  |  |  | M01B2.10 |  |
|  |  |  |  |  |  |  |  |  |  |  |  |  |  |  |  |  |  |  |  |  |  |  |  |  |  |  |  |  |  |  |  |  |  |  |  |  |  | *gpa-5* | G Protein, Alpha subunit |
|  |  |  |  |  |  |  |  |  |  |  |  |  |  |  |  |  |  |  |  |  |  |  |  |  |  |  |  |  |  |  |  |  |  |  |  |  |  | T02G6.2 |  |
|  |  |  |  |  |  |  |  |  |  |  |  |  |  |  |  |  |  |  |  |  |  |  |  |  |  |  |  |  |  |  |  |  |  |  |  |  |  | T02G6.1 |  |
|  |  |  |  |  |  |  |  |  |  |  |  |  |  |  |  |  |  |  |  |  |  |  |  |  |  |  |  |  |  |  |  |  |  |  |  |  |  | C16D6.1 |  |
|  |  |  |  |  |  |  |  |  |  |  |  |  |  |  |  |  |  |  |  |  |  |  |  |  |  |  |  |  |  |  |  |  |  |  |  |  |  | T01G1.6 |  |
|  |  |  |  |  |  |  |  |  |  |  |  |  |  |  |  |  |  |  |  |  |  |  |  |  |  |  |  |  |  |  |  |  |  |  |  |  |  | K11E4.t5 |  |
|  |  |  |  |  |  |  |  |  |  |  |  |  |  |  |  |  |  |  |  |  |  |  |  |  |  |  |  |  |  |  |  |  |  |  |  |  |  | H14A12.9 |  |
|  |  |  |  |  |  |  |  |  |  |  |  |  |  |  |  |  |  |  |  |  |  |  |  |  |  |  |  |  |  |  |  |  |  |  |  |  |  | Y66A7A.3 |  |
|  |  |  |  |  |  |  |  |  |  |  |  |  |  |  |  |  |  |  |  |  |  |  |  |  |  |  |  |  |  |  |  |  |  |  |  |  |  | B0344.4 |  |
|  |  |  |  |  |  |  |  |  |  |  |  |  |  |  |  |  |  |  |  |  |  |  |  |  |  |  |  |  |  |  |  |  |  |  |  |  |  | Y102A11A.13 |  |
|  |  |  |  |  |  |  |  |  |  |  |  |  |  |  |  |  |  |  |  |  |  |  |  |  |  |  |  |  |  |  |  |  |  |  |  |  |  | H06I04.8 |  |
|  |  |  |  |  |  |  |  |  |  |  |  |  |  |  |  |  |  |  |  |  |  |  |  |  |  |  |  |  |  |  |  |  |  |  |  |  |  | F46H5.10 |  |
|  |  |  |  |  |  |  |  |  |  |  |  |  |  |  |  |  |  |  |  |  |  |  |  |  |  |  |  |  |  |  |  |  |  |  |  |  |  | F55A4.11 |  |
|  |  |  |  |  |  |  |  |  |  |  |  |  |  |  |  |  |  |  |  |  |  |  |  |  |  |  |  |  |  |  |  |  |  |  |  |  |  | F28H6.t1 |  |
|  |  |  |  |  |  |  |  |  |  |  |  |  |  |  |  |  |  |  |  |  |  |  |  |  |  |  |  |  |  |  |  |  |  |  |  |  |  | W01C8.15 |  |
|  |  |  |  |  |  |  |  |  |  |  |  |  |  |  |  |  |  |  |  |  |  |  |  |  |  |  |  |  |  |  |  |  |  |  |  |  |  | F59D12.10 |  |
|  |  |  |  |  |  |  |  |  |  |  |  |  |  |  |  |  |  |  |  |  |  |  |  |  |  |  |  |  |  |  |  |  |  |  |  |  |  | F56A6.7 |  |
|  |  |  |  |  |  |  |  |  |  |  |  |  |  |  |  |  |  |  |  |  |  |  |  |  |  |  |  |  |  |  |  |  |  |  |  |  |  | F46E10.13 |  |
|  |  |  |  |  |  |  |  |  |  |  |  |  |  |  |  |  |  |  |  |  |  |  |  |  |  |  |  |  |  |  |  |  |  |  |  |  |  | C50H2.4 |  |
|  |  |  |  |  |  |  |  |  |  |  |  |  |  |  |  |  |  |  |  |  |  |  |  |  |  |  |  |  |  |  |  |  |  |  |  |  |  | Y50D7A.17 |  |
|  |  |  |  |  |  |  |  |  |  |  |  |  |  |  |  |  |  |  |  |  |  |  |  |  |  |  |  |  |  |  |  |  |  |  |  |  |  | K11G9.10 |  |
|  |  |  |  |  |  |  |  |  |  |  |  |  |  |  |  |  |  |  |  |  |  |  |  |  |  |  |  |  |  |  |  |  |  |  |  |  |  | Y71F9AL.10 |  |
|  |  |  |  |  |  |  |  |  |  |  |  |  |  |  |  |  |  |  |  |  |  |  |  |  |  |  |  |  |  |  |  |  |  |  |  |  |  | C18A3.12 |  |
|  |  |  |  |  |  |  |  |  |  |  |  |  |  |  |  |  |  |  |  |  |  |  |  |  |  |  |  |  |  |  |  |  |  |  |  |  |  | C09F9.7 |  |
|  |  |  |  |  |  |  |  |  |  |  |  |  |  |  |  |  |  |  |  |  |  |  |  |  |  |  |  |  |  |  |  |  |  |  |  |  |  | C01H6.6 |  |
|  |  |  |  |  |  |  |  |  |  |  |  |  |  |  |  |  |  |  |  |  |  |  |  |  |  |  |  |  |  |  |  |  |  |  |  |  |  | Y76A2B.4 |  |
|  |  |  |  |  |  |  |  |  |  |  |  |  |  |  |  |  |  |  |  |  |  |  |  |  |  |  |  |  |  |  |  |  |  |  |  |  |  | R03H10.6 |  |
|  |  |  |  |  |  |  |  |  |  |  |  |  |  |  |  |  |  |  |  |  |  |  |  |  |  |  |  |  |  |  |  |  |  |  |  |  |  | *sesn-1* | SEStriN (peroxiredoxin reductase) homolog |
|  |  |  |  |  |  |  |  |  |  |  |  |  |  |  |  |  |  |  |  |  |  |  |  |  |  |  |  |  |  |  |  |  |  |  |  |  |  | *etr-1* | ELAV-Type RNA binding-protein family |
|  |  |  |  |  |  |  |  |  |  |  |  |  |  |  |  |  |  |  |  |  |  |  |  |  |  |  |  |  |  |  |  |  |  |  |  |  |  | F38E9.1 |  |
|  |  |  |  |  |  |  |  |  |  |  |  |  |  |  |  |  |  |  |  |  |  |  |  |  |  |  |  |  |  |  |  |  |  |  |  |  |  | C53D5.1 |  |
|  |  |  |  |  |  |  |  |  |  |  |  |  |  |  |  |  |  |  |  |  |  |  |  |  |  |  |  |  |  |  |  |  |  |  |  |  |  | F15B9.10 |  |
|  |  |  |  |  |  |  |  |  |  |  |  |  |  |  |  |  |  |  |  |  |  |  |  |  |  |  |  |  |  |  |  |  |  |  |  |  |  | R151.1 |  |
|  |  |  |  |  |  |  |  |  |  |  |  |  |  |  |  |  |  |  |  |  |  |  |  |  |  |  |  |  |  |  |  |  |  |  |  |  |  | *tag-275* | Temporarily Assigned Gene name |
|  |  |  |  |  |  |  |  |  |  |  |  |  |  |  |  |  |  |  |  |  |  |  |  |  |  |  |  |  |  |  |  |  |  |  |  |  |  | Y6B3B.1 |  |
|  |  |  |  |  |  |  |  |  |  |  |  |  |  |  |  |  |  |  |  |  |  |  |  |  |  |  |  |  |  |  |  |  |  |  |  |  |  | *tra-1* | TRAnsformer: XX animals transformed into males |
|  |  |  |  |  |  |  |  |  |  |  |  |  |  |  |  |  |  |  |  |  |  |  |  |  |  |  |  |  |  |  |  |  |  |  |  |  |  | F43E2.9 |  |
|  |  |  |  |  |  |  |  |  |  |  |  |  |  |  |  |  |  |  |  |  |  |  |  |  |  |  |  |  |  |  |  |  |  |  |  |  |  | *set-27* | SET (trithorax/polycomb) domain containing |
|  |  |  |  |  |  |  |  |  |  |  |  |  |  |  |  |  |  |  |  |  |  |  |  |  |  |  |  |  |  |  |  |  |  |  |  |  |  | F35D2.15 |  |
|  |  |  |  |  |  |  |  |  |  |  |  |  |  |  |  |  |  |  |  |  |  |  |  |  |  |  |  |  |  |  |  |  |  |  |  |  |  | *mltn-3* | MLt-TeN (mlt-10) related |
|  |  |  |  |  |  |  |  |  |  |  |  |  |  |  |  |  |  |  |  |  |  |  |  |  |  |  |  |  |  |  |  |  |  |  |  |  |  | *btb-7* | BTB (Broad/complex/Tramtrack/Bric a brac) domain protein |
|  |  |  |  |  |  |  |  |  |  |  |  |  |  |  |  |  |  |  |  |  |  |  |  |  |  |  |  |  |  |  |  |  |  |  |  |  |  | B0205.15 |  |
|  |  |  |  |  |  |  |  |  |  |  |  |  |  |  |  |  |  |  |  |  |  |  |  |  |  |  |  |  |  |  |  |  |  |  |  |  |  | F52E10.2 |  |
|  |  |  |  |  |  |  |  |  |  |  |  |  |  |  |  |  |  |  |  |  |  |  |  |  |  |  |  |  |  |  |  |  |  |  |  |  |  | C50E3.12 |  |
|  |  |  |  |  |  |  |  |  |  |  |  |  |  |  |  |  |  |  |  |  |  |  |  |  |  |  |  |  |  |  |  |  |  |  |  |  |  | *srh-269* | Serpentine Receptor, class H |
|  |  |  |  |  |  |  |  |  |  |  |  |  |  |  |  |  |  |  |  |  |  |  |  |  |  |  |  |  |  |  |  |  |  |  |  |  |  | F45E6.7 |  |
|  |  |  |  |  |  |  |  |  |  |  |  |  |  |  |  |  |  |  |  |  |  |  |  |  |  |  |  |  |  |  |  |  |  |  |  |  |  | T08G2.4 |  |
|  |  |  |  |  |  |  |  |  |  |  |  |  |  |  |  |  |  |  |  |  |  |  |  |  |  |  |  |  |  |  |  |  |  |  |  |  |  | C35A11.3 |  |
|  |  |  |  |  |  |  |  |  |  |  |  |  |  |  |  |  |  |  |  |  |  |  |  |  |  |  |  |  |  |  |  |  |  |  |  |  |  | F14H8.5 |  |
|  |  |  |  |  |  |  |  |  |  |  |  |  |  |  |  |  |  |  |  |  |  |  |  |  |  |  |  |  |  |  |  |  |  |  |  |  |  | *math-16* | MATH (meprin-associated Traf homology) domain containing |
|  |  |  |  |  |  |  |  |  |  |  |  |  |  |  |  |  |  |  |  |  |  |  |  |  |  |  |  |  |  |  |  |  |  |  |  |  |  | C54D2.14 |  |
|  |  |  |  |  |  |  |  |  |  |  |  |  |  |  |  |  |  |  |  |  |  |  |  |  |  |  |  |  |  |  |  |  |  |  |  |  |  | C29E6.13 |  |
|  |  |  |  |  |  |  |  |  |  |  |  |  |  |  |  |  |  |  |  |  |  |  |  |  |  |  |  |  |  |  |  |  |  |  |  |  |  | F20B10.9 |  |
|  |  |  |  |  |  |  |  |  |  |  |  |  |  |  |  |  |  |  |  |  |  |  |  |  |  |  |  |  |  |  |  |  |  |  |  |  |  | C06E2.11 |  |
|  |  |  |  |  |  |  |  |  |  |  |  |  |  |  |  |  |  |  |  |  |  |  |  |  |  |  |  |  |  |  |  |  |  |  |  |  |  | *ins-31* | INSulin related |
|  |  |  |  |  |  |  |  |  |  |  |  |  |  |  |  |  |  |  |  |  |  |  |  |  |  |  |  |  |  |  |  |  |  |  |  |  |  | F35B3.9 |  |

### Phenotypes enriched

none found

### Anatomy terms enriched

none found

### GO terms enriched

none found

### Expression clusters enriched

none found

### Motifs enriched

|  |  |  |  |  |  |
| --- | --- | --- | --- | --- | --- |
| **Motif** | **Logo** | **Possible orthologs** | **Number of motifs in cluster** | **Enrichment** | **FDR corrected p** |
| pTH9089 |  | ref-2 | 30 | 2.45 | 0.00011 |
| pTH10031 |  | mbr-1 | 25 | 2.74 | 0.00021 |
| SP3\_f1 |  | ZC328.2 klf-2 | 33 | 2.16 | 0.00030 |
| Plagl1\_0972 |  | Y53H1A.2 | 28 | 2.37 | 0.00054 |
| GM12878\_EGR1\_HudsonAlpha |  | ZC328.2 klf-2 klf-1 | 39 | 1.77 | 0.00170 |
| Sp4\_1011 |  | klf-2 klf-1 | 38 | 1.79 | 0.00210 |
| pTH10623 |  | scrt-1 | 25 | 2.33 | 0.00240 |
| pTH9934 |  | Y53H1A.2 | 25 | 2.33 | 0.00250 |
| MA0085.1 |  | F26F4.8 lag-1 ztf-3 | 48 | 1.49 | 0.00460 |
| ARNT2\_si |  | aha-1 | 14 | 3.46 | 0.00470 |
| Hoxb7\_3953 |  | lin-39 | 41 | 1.64 | 0.00490 |
| MA0470.1 |  | F49E12.6 | 27 | 2.06 | 0.00690 |
| K562\_ZBTB7A\_HudsonAlpha |  | ZC328.2 | 41 | 1.61 | 0.00710 |
| MA0114.2 |  | nhr-62 | 12 | 3.60 | 0.01000 |
| pTH10699 |  | pha-2 (-0.71) ceh-53 (-0.67) | 22 | 2.26 | 0.01100 |
| pTH8996 |  | daf-8 sma-4 | 11 | 3.83 | 0.01200 |
| pTH5891 |  | nhr-49 | 15 | 2.93 | 0.01200 |
| I$UBX\_01 |  | lin-39 | 9 | 4.65 | 0.01200 |
| NEUROD2\_1 |  | hlh-8 (0.53) hlh-32 hlh-15 ngn-1 | 7 | 6.23 | 0.01300 |
| MA0016.1 |  | nhr-69 nhr-2 | 13 | 3.27 | 0.01300 |
| Hoxa7\_2668 |  | lin-39 | 36 | 1.67 | 0.01400 |
| pTH5267 |  | hlh-32 ngn-1 | 8 | 5.08 | 0.01600 |
| Hoxa6\_1040 |  | lin-39 | 38 | 1.61 | 0.01600 |
| V$MYOD\_01 |  | hlh-1 | 31 | 1.79 | 0.01600 |
| CG5669\_SANGER\_10\_FBgn0039169 |  | klf-2 | 50 | 1.38 | 0.01700 |
| BARHL2\_5 |  | ceh-31 ceh-9 | 21 | 2.24 | 0.01700 |
| MA0139.1 |  | F58G1.2 | 16 | 2.67 | 0.01800 |
| tgo\_ss\_SANGER\_5\_FBgn0015014 |  | aha-1 | 52 | 1.34 | 0.01900 |
| SMAD3\_f1 |  | daf-8 | 11 | 3.57 | 0.01900 |
| klu\_SOLEXA\_5\_FBgn0013469 |  | ZC328.2 | 38 | 1.59 | 0.02000 |
| K562\_SRF\_HudsonAlpha |  | unc-120 | 10 | 3.80 | 0.02200 |
| pTH9118 |  | eor-1 | 26 | 1.93 | 0.02300 |
| pTH2820 |  | ZC328.2 | 50 | 1.36 | 0.02500 |
| Oli\_da\_SANGER\_5\_2\_FBgn0032651 |  | hlh-32 | 10 | 3.70 | 0.02600 |
| pTH5778 |  | egl-5 (0.63) | 59 | 1.21 | 0.02600 |
| ECC-1\_ERALPHA\_HudsonAlpha |  | nhr-71 | 25 | 1.94 | 0.02700 |
| MA0163.1 |  | Y53H1A.2 | 22 | 2.09 | 0.02700 |
| pTH6445 |  | ceh-5 | 45 | 1.43 | 0.02800 |
| CENPB\_1 |  | F21D5.4 | 13 | 2.93 | 0.02900 |
| pTH6449 |  | ceh-43 | 12 | 3.08 | 0.03100 |
| pTH9393 |  | ZC416.1 | 17 | 2.41 | 0.03100 |
| Nkx2-3\_3435 |  | ceh-24 | 13 | 2.87 | 0.03300 |
| V$AREB6\_01 |  | ztf-6 | 53 | 1.30 | 0.03300 |
| pTH6569 |  | ceh-43 | 34 | 1.62 | 0.03600 |
| LHX9\_2 |  | ceh-14 (0.65) | 32 | 1.67 | 0.03700 |
| Barx1\_2877 |  | ceh-43 | 24 | 1.93 | 0.03700 |
| Barx2\_3447 |  | ceh-43 | 9 | 3.81 | 0.03700 |
| Bsx\_3483 |  | ceh-31 | 33 | 1.64 | 0.03800 |
| ZBTB7A\_1 |  | ZC328.2 | 23 | 1.97 | 0.03900 |
| RFX1\_4537 |  | daf-19 | 48 | 1.36 | 0.04000 |
| MA0247.2 |  | ceh-22 (-0.58) | 37 | 1.55 | 0.04000 |
| Hoxb3\_1720 |  | lin-39 | 14 | 2.65 | 0.04000 |
| Hoxc4\_3491 |  | lin-39 | 16 | 2.41 | 0.04200 |
| TFAP4\_si |  | hlh-11 | 13 | 2.77 | 0.04200 |
| PURA\_f1 |  | plp-2 | 19 | 2.16 | 0.04300 |
| V$PAX5\_02 |  | pax-2 | 25 | 1.86 | 0.04400 |
| NR2F6\_2 |  | nhr-2 | 18 | 2.22 | 0.04600 |
| Hoxa3\_2783 |  | lin-39 | 12 | 2.90 | 0.04600 |
| SMAD1\_si |  | daf-8 | 14 | 2.59 | 0.04800 |
| pTH5976 |  | irx-1 | 9 | 3.62 | 0.04800 |
| MA0118.1 |  | ref-2 | 20 | 2.07 | 0.05000 |

### Correlated (and anti-correlated) transcription factors

|  |  |
| --- | --- |
| **Transcription factor** | **Correlation** |
| tra-1 | 0.87 |
| ccch-1 | 0.76 |
| Y55F3AM.14 | 0.74 |
| gmeb-1 | 0.72 |
| nhr-119 | 0.69 |
| nhr-87 | 0.68 |
| nhr-85 | 0.67 |
| unc-98 | 0.65 |
| ceh-14 | 0.65 |
| nhr-91 | 0.64 |
| nhr-31 | 0.64 |
| nhr-157 | 0.63 |
| pat-9 | 0.63 |
| egl-5 | 0.63 |
| camt-1 | 0.62 |
| nhr-241 | 0.60 |
| nhr-19 | 0.59 |
| K05F1.5 | 0.59 |
| mls-1 | 0.59 |
| dsc-1 | 0.58 |
| mml-1 | 0.58 |
| ceh-99 | 0.57 |
| egl-38 | 0.55 |
| hlh-8 | 0.53 |
| madf-9 | 0.53 |
| ztf-13 | -0.51 |
| nhr-38 | -0.51 |
| nhr-7 | -0.51 |
| zip-12 | -0.52 |
| nfx-1 | -0.52 |
| eyg-1 | -0.53 |
| zip-7 | -0.53 |
| gla-3 | -0.54 |
| R05D3.3 | -0.54 |
| ccch-5 | -0.55 |
| cky-1 | -0.56 |
| gmeb-2 | -0.58 |
| nhr-269 | -0.58 |
| ceh-22 | -0.58 |
| pax-1 | -0.59 |
| madf-7 | -0.59 |
| ceh-45 | -0.59 |
| pha-4 | -0.59 |
| fkh-6 | -0.60 |
| ceh-60 | -0.62 |
| nhr-106 | -0.63 |
| Y48A6C.1 | -0.64 |
| ceh-53 | -0.67 |
| nhr-74 | -0.70 |
| pha-2 | -0.71 |

### ChIP peaks enriched

|  |  |  |  |  |
| --- | --- | --- | --- | --- |
| **Gene** | **Experiment** | **Number of upstream peaks** | **Enrichment** | **FDR corrected p** |
| lin-35 | LIN-35\_Starved-L1-stage-larvae | 7 | 5.24 | 0.014 |
| nhr-2 | NHR-2\_Embryos | 8 | 4.28 | 0.019 |
| nhr-11 | NHR-11\_Larvae-L2-stage | 13 | 2.81 | 0.020 |
| pes-1 | PES-1\_Larvae-L4-stage | 21 | 1.98 | 0.033 |
