# Supplementary material for: Overlapping cell population expression profiling and regulatory inference in C. elegans
Source: BMC Genomics. 2016 Feb 29;17:159. doi: 10.1186/s12864-016-2482-z (PMC4772325; doi:10.1186/s12864-016-2482-z)
Supplement: Additional file 13: — Web supplement. (DOC 21 kb) [file 12864_2016_2482_MOESM13_ESM.zip › sortWeb/clusters/hier.300.clusters/195.html]

Cluster 195 

## Cluster 195

### Expression

| cnd-1 rep. 1 | cnd-1 rep. 2 | cnd-1 rep. 3 | pha-4 rep. 1 | pha-4 rep. 2 | pha-4 rep. 3 | ceh-27 | ceh-36 | ceh-6 | F21D5.9 | mir-57 | mls-2 | pal-1 | pros-1 | ttx-3 | unc-130 | hlh-16 | irx-1 | ceh-6 (+) hlh-16 (+) | ceh-6 (+) hlh-16 (-) | ceh-6 (-) hlh-16 (+) | cnd-1 singlets | pha-4 singlets | 0 | 60 | 120 | 150 | 180 | 240 | 330 | 390 | 420 | 480 | 540 | 570 | 600 | 630 | 660 | NAME | Functional description |
| --- | --- | --- | --- | --- | --- | --- | --- | --- | --- | --- | --- | --- | --- | --- | --- | --- | --- | --- | --- | --- | --- | --- | --- | --- | --- | --- | --- | --- | --- | --- | --- | --- | --- | --- | --- | --- | --- | --- | --- |
|  |  |  |  |  |  |  |  |  |  |  |  |  |  |  |  |  |  |  |  |  |  |  |  |  |  |  |  |  |  |  |  |  |  |  |  |  |  | *linc-91* | Long Intervening Non-Coding RNA |
|  |  |  |  |  |  |  |  |  |  |  |  |  |  |  |  |  |  |  |  |  |  |  |  |  |  |  |  |  |  |  |  |  |  |  |  |  |  | C46C2.3 |  |
|  |  |  |  |  |  |  |  |  |  |  |  |  |  |  |  |  |  |  |  |  |  |  |  |  |  |  |  |  |  |  |  |  |  |  |  |  |  | *ntr-2* | NemaTocin Receptor |
|  |  |  |  |  |  |  |  |  |  |  |  |  |  |  |  |  |  |  |  |  |  |  |  |  |  |  |  |  |  |  |  |  |  |  |  |  |  | *pag-3* | PAttern of reporter Gene expression abnormal |
|  |  |  |  |  |  |  |  |  |  |  |  |  |  |  |  |  |  |  |  |  |  |  |  |  |  |  |  |  |  |  |  |  |  |  |  |  |  | F18A1.1 |  |
|  |  |  |  |  |  |  |  |  |  |  |  |  |  |  |  |  |  |  |  |  |  |  |  |  |  |  |  |  |  |  |  |  |  |  |  |  |  | *srx-45* | Serpentine Receptor, class X |
|  |  |  |  |  |  |  |  |  |  |  |  |  |  |  |  |  |  |  |  |  |  |  |  |  |  |  |  |  |  |  |  |  |  |  |  |  |  | T01G5.8 |  |
|  |  |  |  |  |  |  |  |  |  |  |  |  |  |  |  |  |  |  |  |  |  |  |  |  |  |  |  |  |  |  |  |  |  |  |  |  |  | K02D3.1 |  |
|  |  |  |  |  |  |  |  |  |  |  |  |  |  |  |  |  |  |  |  |  |  |  |  |  |  |  |  |  |  |  |  |  |  |  |  |  |  | B0035.18 |  |
|  |  |  |  |  |  |  |  |  |  |  |  |  |  |  |  |  |  |  |  |  |  |  |  |  |  |  |  |  |  |  |  |  |  |  |  |  |  | Y73F8A.22 |  |
|  |  |  |  |  |  |  |  |  |  |  |  |  |  |  |  |  |  |  |  |  |  |  |  |  |  |  |  |  |  |  |  |  |  |  |  |  |  | C14C11.10 |  |
|  |  |  |  |  |  |  |  |  |  |  |  |  |  |  |  |  |  |  |  |  |  |  |  |  |  |  |  |  |  |  |  |  |  |  |  |  |  | *clec-23* | C-type LECtin |
|  |  |  |  |  |  |  |  |  |  |  |  |  |  |  |  |  |  |  |  |  |  |  |  |  |  |  |  |  |  |  |  |  |  |  |  |  |  | ZK1193.2 |  |
|  |  |  |  |  |  |  |  |  |  |  |  |  |  |  |  |  |  |  |  |  |  |  |  |  |  |  |  |  |  |  |  |  |  |  |  |  |  | *lin-42* | abnormal cell LINeage |
|  |  |  |  |  |  |  |  |  |  |  |  |  |  |  |  |  |  |  |  |  |  |  |  |  |  |  |  |  |  |  |  |  |  |  |  |  |  | *acd-3* | ACid-sensitive Degenerin |

### Phenotypes enriched

none found

### Anatomy terms enriched

none found

### GO terms enriched

none found

### Expression clusters enriched

none found

### Motifs enriched

|  |  |  |  |  |  |
| --- | --- | --- | --- | --- | --- |
| **Motif** | **Logo** | **Possible orthologs** | **Number of motifs in cluster** | **Enrichment** | **FDR corrected p** |
| pTH10777 |  | dmd-3 | 15 | 1.82 | 0.013 |
| HNF4A\_6 |  | nhr-62 | 14 | 2.03 | 0.015 |
| REF1 |  | lin-22 ref-1 | 7 | 4.87 | 0.020 |
| V$POU3F2\_02 |  | ceh-18 | 15 | 1.70 | 0.027 |
| Srf\_3509 |  | unc-120 | 5 | 6.66 | 0.041 |
| Bsh\_Cell\_FBgn0000529 |  | ceh-43 | 9 | 3.05 | 0.045 |
| pTH6408 |  | irx-1 | 10 | 2.66 | 0.050 |

### Correlated (and anti-correlated) transcription factors

|  |  |
| --- | --- |
| **Transcription factor** | **Correlation** |
| pag-3 | 0.85 |
| mls-2 | 0.68 |
| hlh-34 | 0.66 |
| ttx-1 | 0.64 |
| nhr-118 | 0.64 |
| nhr-242 | 0.60 |
| ref-2 | 0.60 |
| hlh-14 | 0.59 |
| grl-25 | 0.58 |
| ttx-3 | 0.57 |
| ceh-17 | 0.55 |
| ngn-1 | 0.54 |
| sma-2 | 0.54 |
| dac-1 | 0.54 |
| nhr-277 | 0.53 |
| nhr-154 | 0.53 |
| egl-43 | 0.52 |
| nhr-153 | 0.51 |
| tbx-39 | 0.51 |
| hlh-27 | 0.51 |
| nhr-195 | 0.50 |
| Y105C5A.15 | 0.50 |
| end-3 | 0.49 |
| nhr-25 | 0.49 |
| nhr-230 | 0.49 |
| mbf-1 | -0.31 |
| tbx-40 | -0.31 |
| Y60A9.3 | -0.31 |
| nhr-229 | -0.32 |
| dhhc-13 | -0.32 |
| dct-13 | -0.32 |
| nhr-247 | -0.32 |
| srab-2 | -0.33 |
| mex-6 | -0.33 |
| lin-32 | -0.33 |
| tbx-36 | -0.33 |
| lst-5 | -0.34 |
| lin-22 | -0.34 |
| ceh-7 | -0.35 |
| Y56A3A.18 | -0.36 |
| F21G4.5 | -0.37 |
| nhr-220 | -0.37 |
| Y48G9A.11 | -0.37 |
| hlh-12 | -0.38 |
| nhr-175 | -0.38 |
| sknr-1 | -0.40 |
| spe-44 | -0.44 |
| hlh-29 | -0.44 |
| ceh-82 | -0.47 |
| pos-1 | -0.48 |

### ChIP peaks enriched

|  |  |  |  |  |
| --- | --- | --- | --- | --- |
| **Gene** | **Experiment** | **Number of upstream peaks** | **Enrichment** | **FDR corrected p** |
| nhr-116 | NHR-116\_Larvae-L4-stage | 2 | 52.37 | 0.023 |
