# Supplementary material for: Overlapping cell population expression profiling and regulatory inference in C. elegans
Source: BMC Genomics. 2016 Feb 29;17:159. doi: 10.1186/s12864-016-2482-z (PMC4772325; doi:10.1186/s12864-016-2482-z)
Supplement: Additional file 13: — Web supplement. (DOC 21 kb) [file 12864_2016_2482_MOESM13_ESM.zip › sortWeb/clusters/hier.300.clusters/196.html]

Cluster 196 

## Cluster 196

### Expression

| cnd-1 rep. 1 | cnd-1 rep. 2 | cnd-1 rep. 3 | pha-4 rep. 1 | pha-4 rep. 2 | pha-4 rep. 3 | ceh-27 | ceh-36 | ceh-6 | F21D5.9 | mir-57 | mls-2 | pal-1 | pros-1 | ttx-3 | unc-130 | hlh-16 | irx-1 | ceh-6 (+) hlh-16 (+) | ceh-6 (+) hlh-16 (-) | ceh-6 (-) hlh-16 (+) | cnd-1 singlets | pha-4 singlets | 0 | 60 | 120 | 150 | 180 | 240 | 330 | 390 | 420 | 480 | 540 | 570 | 600 | 630 | 660 | NAME | Functional description |
| --- | --- | --- | --- | --- | --- | --- | --- | --- | --- | --- | --- | --- | --- | --- | --- | --- | --- | --- | --- | --- | --- | --- | --- | --- | --- | --- | --- | --- | --- | --- | --- | --- | --- | --- | --- | --- | --- | --- | --- |
|  |  |  |  |  |  |  |  |  |  |  |  |  |  |  |  |  |  |  |  |  |  |  |  |  |  |  |  |  |  |  |  |  |  |  |  |  |  | Y46E12BL.6 |  |
|  |  |  |  |  |  |  |  |  |  |  |  |  |  |  |  |  |  |  |  |  |  |  |  |  |  |  |  |  |  |  |  |  |  |  |  |  |  | Y39G8C.10 |  |
|  |  |  |  |  |  |  |  |  |  |  |  |  |  |  |  |  |  |  |  |  |  |  |  |  |  |  |  |  |  |  |  |  |  |  |  |  |  | W01A11.13 |  |
|  |  |  |  |  |  |  |  |  |  |  |  |  |  |  |  |  |  |  |  |  |  |  |  |  |  |  |  |  |  |  |  |  |  |  |  |  |  | *linc-124* | Long Intervening Non-Coding RNA |
|  |  |  |  |  |  |  |  |  |  |  |  |  |  |  |  |  |  |  |  |  |  |  |  |  |  |  |  |  |  |  |  |  |  |  |  |  |  | *fbxa-26* | F-box A protein |
|  |  |  |  |  |  |  |  |  |  |  |  |  |  |  |  |  |  |  |  |  |  |  |  |  |  |  |  |  |  |  |  |  |  |  |  |  |  | F56A6.6 |  |
|  |  |  |  |  |  |  |  |  |  |  |  |  |  |  |  |  |  |  |  |  |  |  |  |  |  |  |  |  |  |  |  |  |  |  |  |  |  | C30B5.10 |  |
|  |  |  |  |  |  |  |  |  |  |  |  |  |  |  |  |  |  |  |  |  |  |  |  |  |  |  |  |  |  |  |  |  |  |  |  |  |  | cTel3X.2 |  |
|  |  |  |  |  |  |  |  |  |  |  |  |  |  |  |  |  |  |  |  |  |  |  |  |  |  |  |  |  |  |  |  |  |  |  |  |  |  | T03D3.13 |  |
|  |  |  |  |  |  |  |  |  |  |  |  |  |  |  |  |  |  |  |  |  |  |  |  |  |  |  |  |  |  |  |  |  |  |  |  |  |  | *lov-1* | Location Of Vulva defective |
|  |  |  |  |  |  |  |  |  |  |  |  |  |  |  |  |  |  |  |  |  |  |  |  |  |  |  |  |  |  |  |  |  |  |  |  |  |  | R193.2 |  |
|  |  |  |  |  |  |  |  |  |  |  |  |  |  |  |  |  |  |  |  |  |  |  |  |  |  |  |  |  |  |  |  |  |  |  |  |  |  | *srx-123* | Serpentine Receptor, class X |
|  |  |  |  |  |  |  |  |  |  |  |  |  |  |  |  |  |  |  |  |  |  |  |  |  |  |  |  |  |  |  |  |  |  |  |  |  |  | K09H11.13 |  |
|  |  |  |  |  |  |  |  |  |  |  |  |  |  |  |  |  |  |  |  |  |  |  |  |  |  |  |  |  |  |  |  |  |  |  |  |  |  | D1046.t1 |  |
|  |  |  |  |  |  |  |  |  |  |  |  |  |  |  |  |  |  |  |  |  |  |  |  |  |  |  |  |  |  |  |  |  |  |  |  |  |  | T24D5.6 |  |
|  |  |  |  |  |  |  |  |  |  |  |  |  |  |  |  |  |  |  |  |  |  |  |  |  |  |  |  |  |  |  |  |  |  |  |  |  |  | Y51A2B.11 |  |
|  |  |  |  |  |  |  |  |  |  |  |  |  |  |  |  |  |  |  |  |  |  |  |  |  |  |  |  |  |  |  |  |  |  |  |  |  |  | *sri-33* | Serpentine Receptor, class I |
|  |  |  |  |  |  |  |  |  |  |  |  |  |  |  |  |  |  |  |  |  |  |  |  |  |  |  |  |  |  |  |  |  |  |  |  |  |  | *sre-46* | Serpentine Receptor, class E (epsilon) |
|  |  |  |  |  |  |  |  |  |  |  |  |  |  |  |  |  |  |  |  |  |  |  |  |  |  |  |  |  |  |  |  |  |  |  |  |  |  | F52F12.9 |  |
|  |  |  |  |  |  |  |  |  |  |  |  |  |  |  |  |  |  |  |  |  |  |  |  |  |  |  |  |  |  |  |  |  |  |  |  |  |  | F20H11.4 |  |

### Phenotypes enriched

none found

### Anatomy terms enriched

none found

### GO terms enriched

none found

### Expression clusters enriched

none found

### Motifs enriched

None found

### Correlated (and anti-correlated) transcription factors

|  |  |
| --- | --- |
| **Transcription factor** | **Correlation** |
| nhr-242 | 0.82 |
| nhr-199 | 0.72 |
| D1081.8 | 0.61 |
| dmd-4 | 0.61 |
| gla-3 | 0.60 |
| hlh-34 | 0.59 |
| hlh-6 | 0.57 |
| ceh-6 | 0.56 |
| madf-7 | 0.55 |
| athp-1 | 0.50 |
| eyg-1 | 0.49 |
| ceh-100 | 0.49 |
| T10D4.6 | 0.49 |
| mab-23 | 0.47 |
| Y48C3A.12 | 0.47 |
| hlh-17 | 0.46 |
| B0250.4 | 0.46 |
| ceh-89 | 0.45 |
| Y57A10A.31 | 0.44 |
| aptf-1 | 0.44 |
| odd-1 | 0.44 |
| R05D3.3 | 0.44 |
| F26H9.2 | 0.44 |
| hmbx-1 | 0.43 |
| aptf-2 | 0.43 |
| moe-3 | -0.36 |
| nhr-247 | -0.36 |
| unc-98 | -0.36 |
| nhr-9 | -0.37 |
| Y55F3AM.14 | -0.38 |
| ceh-1 | -0.38 |
| nhr-115 | -0.38 |
| hlh-8 | -0.38 |
| C01F6.9 | -0.39 |
| mls-1 | -0.39 |
| dsc-1 | -0.40 |
| nhr-14 | -0.40 |
| ceh-43 | -0.41 |
| madf-1 | -0.41 |
| atf-5 | -0.42 |
| nhr-90 | -0.44 |
| lin-31 | -0.45 |
| dct-13 | -0.46 |
| nhr-87 | -0.47 |
| dro-1 | -0.48 |
| ceh-14 | -0.48 |
| ceh-30 | -0.48 |
| nhr-11 | -0.65 |
| tbx-40 | -0.65 |
| nhr-252 | -0.66 |

### ChIP peaks enriched

none found
