# Supplementary material for: Overlapping cell population expression profiling and regulatory inference in C. elegans
Source: BMC Genomics. 2016 Feb 29;17:159. doi: 10.1186/s12864-016-2482-z (PMC4772325; doi:10.1186/s12864-016-2482-z)
Supplement: Additional file 13: — Web supplement. (DOC 21 kb) [file 12864_2016_2482_MOESM13_ESM.zip › sortWeb/clusters/hier.300.clusters/197.html]

Cluster 197 

## Cluster 197

### Expression

| cnd-1 rep. 1 | cnd-1 rep. 2 | cnd-1 rep. 3 | pha-4 rep. 1 | pha-4 rep. 2 | pha-4 rep. 3 | ceh-27 | ceh-36 | ceh-6 | F21D5.9 | mir-57 | mls-2 | pal-1 | pros-1 | ttx-3 | unc-130 | hlh-16 | irx-1 | ceh-6 (+) hlh-16 (+) | ceh-6 (+) hlh-16 (-) | ceh-6 (-) hlh-16 (+) | cnd-1 singlets | pha-4 singlets | 0 | 60 | 120 | 150 | 180 | 240 | 330 | 390 | 420 | 480 | 540 | 570 | 600 | 630 | 660 | NAME | Functional description |
| --- | --- | --- | --- | --- | --- | --- | --- | --- | --- | --- | --- | --- | --- | --- | --- | --- | --- | --- | --- | --- | --- | --- | --- | --- | --- | --- | --- | --- | --- | --- | --- | --- | --- | --- | --- | --- | --- | --- | --- |
|  |  |  |  |  |  |  |  |  |  |  |  |  |  |  |  |  |  |  |  |  |  |  |  |  |  |  |  |  |  |  |  |  |  |  |  |  |  | *oac-58* | O-ACyltransferase homolog |
|  |  |  |  |  |  |  |  |  |  |  |  |  |  |  |  |  |  |  |  |  |  |  |  |  |  |  |  |  |  |  |  |  |  |  |  |  |  | Y116A8B.4 |  |
|  |  |  |  |  |  |  |  |  |  |  |  |  |  |  |  |  |  |  |  |  |  |  |  |  |  |  |  |  |  |  |  |  |  |  |  |  |  | Y108G3AL.8 |  |
|  |  |  |  |  |  |  |  |  |  |  |  |  |  |  |  |  |  |  |  |  |  |  |  |  |  |  |  |  |  |  |  |  |  |  |  |  |  | *spr-2* | Suppressor of PResenilin defect |
|  |  |  |  |  |  |  |  |  |  |  |  |  |  |  |  |  |  |  |  |  |  |  |  |  |  |  |  |  |  |  |  |  |  |  |  |  |  | R04E5.t6 |  |
|  |  |  |  |  |  |  |  |  |  |  |  |  |  |  |  |  |  |  |  |  |  |  |  |  |  |  |  |  |  |  |  |  |  |  |  |  |  | *anr-35* | Antisense Non-coding RNA |
|  |  |  |  |  |  |  |  |  |  |  |  |  |  |  |  |  |  |  |  |  |  |  |  |  |  |  |  |  |  |  |  |  |  |  |  |  |  | B0507.8 |  |
|  |  |  |  |  |  |  |  |  |  |  |  |  |  |  |  |  |  |  |  |  |  |  |  |  |  |  |  |  |  |  |  |  |  |  |  |  |  | *skr-10* | SKp1 Related (ubiquitin ligase complex component) |
|  |  |  |  |  |  |  |  |  |  |  |  |  |  |  |  |  |  |  |  |  |  |  |  |  |  |  |  |  |  |  |  |  |  |  |  |  |  | C34C12.11 |  |
|  |  |  |  |  |  |  |  |  |  |  |  |  |  |  |  |  |  |  |  |  |  |  |  |  |  |  |  |  |  |  |  |  |  |  |  |  |  | Y105C5A.1270 |  |
|  |  |  |  |  |  |  |  |  |  |  |  |  |  |  |  |  |  |  |  |  |  |  |  |  |  |  |  |  |  |  |  |  |  |  |  |  |  | Y37E3.26 |  |
|  |  |  |  |  |  |  |  |  |  |  |  |  |  |  |  |  |  |  |  |  |  |  |  |  |  |  |  |  |  |  |  |  |  |  |  |  |  | *clec-184* | C-type LECtin |
|  |  |  |  |  |  |  |  |  |  |  |  |  |  |  |  |  |  |  |  |  |  |  |  |  |  |  |  |  |  |  |  |  |  |  |  |  |  | B0554.5 |  |
|  |  |  |  |  |  |  |  |  |  |  |  |  |  |  |  |  |  |  |  |  |  |  |  |  |  |  |  |  |  |  |  |  |  |  |  |  |  | *srt-64* | Serpentine Receptor, class T |

### Phenotypes enriched

none found

### Anatomy terms enriched

none found

### GO terms enriched

none found

### Expression clusters enriched

none found

### Motifs enriched

|  |  |  |  |  |  |
| --- | --- | --- | --- | --- | --- |
| **Motif** | **Logo** | **Possible orthologs** | **Number of motifs in cluster** | **Enrichment** | **FDR corrected p** |
| pTH3037 |  | hlh-15 | 7 | 4.06 | 0.043 |

### Correlated (and anti-correlated) transcription factors

|  |  |
| --- | --- |
| **Transcription factor** | **Correlation** |
| dhhc-7 | 0.65 |
| ceh-74 | 0.60 |
| aptf-4 | 0.58 |
| C34F6.9 | 0.58 |
| nhr-196 | 0.56 |
| unc-42 | 0.54 |
| ceh-54 | 0.54 |
| ceh-9 | 0.51 |
| dmd-10 | 0.51 |
| ceh-62 | 0.50 |
| nhr-268 | 0.49 |
| zip-4 | 0.49 |
| nfya-2 | 0.49 |
| F21G4.5 | 0.49 |
| mbr-1 | 0.48 |
| Y53H1A.2 | 0.48 |
| bed-1 | 0.47 |
| ceh-36 | 0.46 |
| ceh-24 | 0.46 |
| mbf-1 | 0.46 |
| ZK185.1 | 0.46 |
| nhr-47 | 0.46 |
| sox-3 | 0.46 |
| nhr-198 | 0.46 |
| sox-4 | 0.46 |
| nhr-13 | -0.45 |
| hlh-30 | -0.45 |
| egl-18 | -0.45 |
| fkh-9 | -0.46 |
| nhr-256 | -0.46 |
| nhr-126 | -0.46 |
| ztf-27 | -0.46 |
| egl-43 | -0.48 |
| dmd-11 | -0.48 |
| nhr-97 | -0.48 |
| nhr-156 | -0.49 |
| nhr-98 | -0.49 |
| nhr-136 | -0.49 |
| ceh-34 | -0.51 |
| dhhc-12 | -0.51 |
| nhr-201 | -0.52 |
| nhr-177 | -0.52 |
| nhr-146 | -0.53 |
| nhr-202 | -0.55 |
| nhr-42 | -0.56 |
| ahr-1 | -0.57 |
| nhr-122 | -0.57 |
| ztf-14 | -0.59 |
| nhr-34 | -0.59 |
| sem-2 | -0.62 |

### ChIP peaks enriched

none found
