# Supplementary material for: Overlapping cell population expression profiling and regulatory inference in C. elegans
Source: BMC Genomics. 2016 Feb 29;17:159. doi: 10.1186/s12864-016-2482-z (PMC4772325; doi:10.1186/s12864-016-2482-z)
Supplement: Additional file 13: — Web supplement. (DOC 21 kb) [file 12864_2016_2482_MOESM13_ESM.zip › sortWeb/clusters/hier.300.clusters/198.html]

Cluster 198 

## Cluster 198

### Expression

| cnd-1 rep. 1 | cnd-1 rep. 2 | cnd-1 rep. 3 | pha-4 rep. 1 | pha-4 rep. 2 | pha-4 rep. 3 | ceh-27 | ceh-36 | ceh-6 | F21D5.9 | mir-57 | mls-2 | pal-1 | pros-1 | ttx-3 | unc-130 | hlh-16 | irx-1 | ceh-6 (+) hlh-16 (+) | ceh-6 (+) hlh-16 (-) | ceh-6 (-) hlh-16 (+) | cnd-1 singlets | pha-4 singlets | 0 | 60 | 120 | 150 | 180 | 240 | 330 | 390 | 420 | 480 | 540 | 570 | 600 | 630 | 660 | NAME | Functional description |
| --- | --- | --- | --- | --- | --- | --- | --- | --- | --- | --- | --- | --- | --- | --- | --- | --- | --- | --- | --- | --- | --- | --- | --- | --- | --- | --- | --- | --- | --- | --- | --- | --- | --- | --- | --- | --- | --- | --- | --- |
|  |  |  |  |  |  |  |  |  |  |  |  |  |  |  |  |  |  |  |  |  |  |  |  |  |  |  |  |  |  |  |  |  |  |  |  |  |  | *dsl-2* | Delta/Serrate/Lag-2 domain |
|  |  |  |  |  |  |  |  |  |  |  |  |  |  |  |  |  |  |  |  |  |  |  |  |  |  |  |  |  |  |  |  |  |  |  |  |  |  | *skr-15* | SKp1 Related (ubiquitin ligase complex component) |
|  |  |  |  |  |  |  |  |  |  |  |  |  |  |  |  |  |  |  |  |  |  |  |  |  |  |  |  |  |  |  |  |  |  |  |  |  |  | R11.4 |  |
|  |  |  |  |  |  |  |  |  |  |  |  |  |  |  |  |  |  |  |  |  |  |  |  |  |  |  |  |  |  |  |  |  |  |  |  |  |  | *fbxb-33* | F-box B protein |
|  |  |  |  |  |  |  |  |  |  |  |  |  |  |  |  |  |  |  |  |  |  |  |  |  |  |  |  |  |  |  |  |  |  |  |  |  |  | F42A10.9 |  |
|  |  |  |  |  |  |  |  |  |  |  |  |  |  |  |  |  |  |  |  |  |  |  |  |  |  |  |  |  |  |  |  |  |  |  |  |  |  | *arrd-2* | ARRestin Domain protein |
|  |  |  |  |  |  |  |  |  |  |  |  |  |  |  |  |  |  |  |  |  |  |  |  |  |  |  |  |  |  |  |  |  |  |  |  |  |  | *fbxb-49* | F-box B protein |
|  |  |  |  |  |  |  |  |  |  |  |  |  |  |  |  |  |  |  |  |  |  |  |  |  |  |  |  |  |  |  |  |  |  |  |  |  |  | K05F6.4 |  |
|  |  |  |  |  |  |  |  |  |  |  |  |  |  |  |  |  |  |  |  |  |  |  |  |  |  |  |  |  |  |  |  |  |  |  |  |  |  | *fbxb-46* | F-box B protein |
|  |  |  |  |  |  |  |  |  |  |  |  |  |  |  |  |  |  |  |  |  |  |  |  |  |  |  |  |  |  |  |  |  |  |  |  |  |  | Y106G6D.2 |  |
|  |  |  |  |  |  |  |  |  |  |  |  |  |  |  |  |  |  |  |  |  |  |  |  |  |  |  |  |  |  |  |  |  |  |  |  |  |  | *fbxb-82* | F-box B protein |
|  |  |  |  |  |  |  |  |  |  |  |  |  |  |  |  |  |  |  |  |  |  |  |  |  |  |  |  |  |  |  |  |  |  |  |  |  |  | C40A11.7 |  |
|  |  |  |  |  |  |  |  |  |  |  |  |  |  |  |  |  |  |  |  |  |  |  |  |  |  |  |  |  |  |  |  |  |  |  |  |  |  | *fbxb-119* | F-box B protein |
|  |  |  |  |  |  |  |  |  |  |  |  |  |  |  |  |  |  |  |  |  |  |  |  |  |  |  |  |  |  |  |  |  |  |  |  |  |  | *fbxb-59* | F-box B protein |
|  |  |  |  |  |  |  |  |  |  |  |  |  |  |  |  |  |  |  |  |  |  |  |  |  |  |  |  |  |  |  |  |  |  |  |  |  |  | T26E3.11 |  |
|  |  |  |  |  |  |  |  |  |  |  |  |  |  |  |  |  |  |  |  |  |  |  |  |  |  |  |  |  |  |  |  |  |  |  |  |  |  | *dot-1.4* | DOT1 histone methyltransferase family |
|  |  |  |  |  |  |  |  |  |  |  |  |  |  |  |  |  |  |  |  |  |  |  |  |  |  |  |  |  |  |  |  |  |  |  |  |  |  | F17A2.13 |  |
|  |  |  |  |  |  |  |  |  |  |  |  |  |  |  |  |  |  |  |  |  |  |  |  |  |  |  |  |  |  |  |  |  |  |  |  |  |  | *nhr-2* | Nuclear Hormone Receptor family |
|  |  |  |  |  |  |  |  |  |  |  |  |  |  |  |  |  |  |  |  |  |  |  |  |  |  |  |  |  |  |  |  |  |  |  |  |  |  | *skr-8* | SKp1 Related (ubiquitin ligase complex component) |
|  |  |  |  |  |  |  |  |  |  |  |  |  |  |  |  |  |  |  |  |  |  |  |  |  |  |  |  |  |  |  |  |  |  |  |  |  |  | Y71A12B.11 |  |
|  |  |  |  |  |  |  |  |  |  |  |  |  |  |  |  |  |  |  |  |  |  |  |  |  |  |  |  |  |  |  |  |  |  |  |  |  |  | *btb-11* | BTB (Broad/complex/Tramtrack/Bric a brac) domain protein |
|  |  |  |  |  |  |  |  |  |  |  |  |  |  |  |  |  |  |  |  |  |  |  |  |  |  |  |  |  |  |  |  |  |  |  |  |  |  | *sdz-28* | SKN-1 Dependent Zygotic transcript |
|  |  |  |  |  |  |  |  |  |  |  |  |  |  |  |  |  |  |  |  |  |  |  |  |  |  |  |  |  |  |  |  |  |  |  |  |  |  | F46F11.10 |  |
|  |  |  |  |  |  |  |  |  |  |  |  |  |  |  |  |  |  |  |  |  |  |  |  |  |  |  |  |  |  |  |  |  |  |  |  |  |  | Y47H9C.7 |  |
|  |  |  |  |  |  |  |  |  |  |  |  |  |  |  |  |  |  |  |  |  |  |  |  |  |  |  |  |  |  |  |  |  |  |  |  |  |  | *btb-10* | BTB (Broad/complex/Tramtrack/Bric a brac) domain protein |
|  |  |  |  |  |  |  |  |  |  |  |  |  |  |  |  |  |  |  |  |  |  |  |  |  |  |  |  |  |  |  |  |  |  |  |  |  |  | Y45F10C.6 |  |
|  |  |  |  |  |  |  |  |  |  |  |  |  |  |  |  |  |  |  |  |  |  |  |  |  |  |  |  |  |  |  |  |  |  |  |  |  |  | *btb-8* | BTB (Broad/complex/Tramtrack/Bric a brac) domain protein |
|  |  |  |  |  |  |  |  |  |  |  |  |  |  |  |  |  |  |  |  |  |  |  |  |  |  |  |  |  |  |  |  |  |  |  |  |  |  | F15A4.10 |  |
|  |  |  |  |  |  |  |  |  |  |  |  |  |  |  |  |  |  |  |  |  |  |  |  |  |  |  |  |  |  |  |  |  |  |  |  |  |  | F23A7.1 |  |
|  |  |  |  |  |  |  |  |  |  |  |  |  |  |  |  |  |  |  |  |  |  |  |  |  |  |  |  |  |  |  |  |  |  |  |  |  |  | *fbxb-32* | F-box B protein |
|  |  |  |  |  |  |  |  |  |  |  |  |  |  |  |  |  |  |  |  |  |  |  |  |  |  |  |  |  |  |  |  |  |  |  |  |  |  | *sdz-26* | SKN-1 Dependent Zygotic transcript |
|  |  |  |  |  |  |  |  |  |  |  |  |  |  |  |  |  |  |  |  |  |  |  |  |  |  |  |  |  |  |  |  |  |  |  |  |  |  | *btb-12* | BTB (Broad/complex/Tramtrack/Bric a brac) domain protein |
|  |  |  |  |  |  |  |  |  |  |  |  |  |  |  |  |  |  |  |  |  |  |  |  |  |  |  |  |  |  |  |  |  |  |  |  |  |  | *clec-43* | C-type LECtin |
|  |  |  |  |  |  |  |  |  |  |  |  |  |  |  |  |  |  |  |  |  |  |  |  |  |  |  |  |  |  |  |  |  |  |  |  |  |  | *btb-13* | BTB (Broad/complex/Tramtrack/Bric a brac) domain protein |
|  |  |  |  |  |  |  |  |  |  |  |  |  |  |  |  |  |  |  |  |  |  |  |  |  |  |  |  |  |  |  |  |  |  |  |  |  |  | *tbx-43* | T BoX family |
|  |  |  |  |  |  |  |  |  |  |  |  |  |  |  |  |  |  |  |  |  |  |  |  |  |  |  |  |  |  |  |  |  |  |  |  |  |  | F49B2.7 |  |
|  |  |  |  |  |  |  |  |  |  |  |  |  |  |  |  |  |  |  |  |  |  |  |  |  |  |  |  |  |  |  |  |  |  |  |  |  |  | *ceh-51* | C. Elegans Homeobox |
|  |  |  |  |  |  |  |  |  |  |  |  |  |  |  |  |  |  |  |  |  |  |  |  |  |  |  |  |  |  |  |  |  |  |  |  |  |  | M04D5.1 |  |
|  |  |  |  |  |  |  |  |  |  |  |  |  |  |  |  |  |  |  |  |  |  |  |  |  |  |  |  |  |  |  |  |  |  |  |  |  |  | F40E3.7 |  |
|  |  |  |  |  |  |  |  |  |  |  |  |  |  |  |  |  |  |  |  |  |  |  |  |  |  |  |  |  |  |  |  |  |  |  |  |  |  | Y105C5B.9 |  |
|  |  |  |  |  |  |  |  |  |  |  |  |  |  |  |  |  |  |  |  |  |  |  |  |  |  |  |  |  |  |  |  |  |  |  |  |  |  | Y82E9BL.6 |  |
|  |  |  |  |  |  |  |  |  |  |  |  |  |  |  |  |  |  |  |  |  |  |  |  |  |  |  |  |  |  |  |  |  |  |  |  |  |  | *hmbx-1* | HMBOX (mammalian HoMeoBOX gene) homolog |
|  |  |  |  |  |  |  |  |  |  |  |  |  |  |  |  |  |  |  |  |  |  |  |  |  |  |  |  |  |  |  |  |  |  |  |  |  |  | F57A10.4 |  |
|  |  |  |  |  |  |  |  |  |  |  |  |  |  |  |  |  |  |  |  |  |  |  |  |  |  |  |  |  |  |  |  |  |  |  |  |  |  | *fbxa-168* | F-box A protein |
|  |  |  |  |  |  |  |  |  |  |  |  |  |  |  |  |  |  |  |  |  |  |  |  |  |  |  |  |  |  |  |  |  |  |  |  |  |  | *fbxb-87* | F-box B protein |
|  |  |  |  |  |  |  |  |  |  |  |  |  |  |  |  |  |  |  |  |  |  |  |  |  |  |  |  |  |  |  |  |  |  |  |  |  |  | *crn-4* | Cell-death-Related Nuclease |
|  |  |  |  |  |  |  |  |  |  |  |  |  |  |  |  |  |  |  |  |  |  |  |  |  |  |  |  |  |  |  |  |  |  |  |  |  |  | C52E2.3 |  |
|  |  |  |  |  |  |  |  |  |  |  |  |  |  |  |  |  |  |  |  |  |  |  |  |  |  |  |  |  |  |  |  |  |  |  |  |  |  | F43C9.9 |  |
|  |  |  |  |  |  |  |  |  |  |  |  |  |  |  |  |  |  |  |  |  |  |  |  |  |  |  |  |  |  |  |  |  |  |  |  |  |  | *lips-12* | LIPaSe related |
|  |  |  |  |  |  |  |  |  |  |  |  |  |  |  |  |  |  |  |  |  |  |  |  |  |  |  |  |  |  |  |  |  |  |  |  |  |  | *end-1* | ENDoderm determining |
|  |  |  |  |  |  |  |  |  |  |  |  |  |  |  |  |  |  |  |  |  |  |  |  |  |  |  |  |  |  |  |  |  |  |  |  |  |  | *cyp-29A3* | CYtochrome P450 family |
|  |  |  |  |  |  |  |  |  |  |  |  |  |  |  |  |  |  |  |  |  |  |  |  |  |  |  |  |  |  |  |  |  |  |  |  |  |  | *ugt-34* | UDP-GlucuronosylTransferase |

### Phenotypes enriched

none found

### Anatomy terms enriched

none found

### GO terms enriched

none found

### Expression clusters enriched

|  |  |  |  |
| --- | --- | --- | --- |
| **Group name** | **Number in cluster** | **Enrichment** | **FDR corrected p** |
| Strictly embryonic transient class (SET): genes that are the subset of embryonic transient genes that are not also classified as maternal. | 21 | 14.80 | 1.15e-16 |
| Embryonic transient class (ET): genes that are the subset of embryonic genes in which the latest significant increase is earlier than their latest significant decrease. | 29 | 6.64 | 3.37e-15 |
| Early embryonic development gene expression profile. [cgc5767]:cluster\_7 | 13 | 35.32 | 3.41e-14 |
| Embryonic transient (ET) subclasses are based on time of max abundance. [cgc5767]:expression\_class\_ET\_max(83\_min) | 16 | 17.74 | 2.96e-13 |
| Strictly embryonic class (SE): genes that are the subset of embryonic genes that are not also classified as maternal. | 22 | 7.07 | 4.48e-11 |
| Genes upregulated by fer-1 mutants hc1 and hc24, with > 4 fold change in expression level. | 11 | 29.89 | 5.97e-11 |
| C-lineage related expression profile. WBPaper00025032:cluster\_9 | 10 | 28.19 | 1.48e-09 |
| Genes with decreased expression after 24 hours of infection by E.faecalis Fold changes shown are pathogen vs OP50. WBPaper00038438:E.faecalis\_24hr\_downregulated\_TilingArray | 12 | 15.60 | 7.54e-09 |
| Expression Pattern Group D, enriched for genes involved in catabolic processes. | 22 | 5.34 | 9.95e-09 |
| Genes that showed decreased expression after exposure to 7.5uM CH3HgCl for 24 hours. | 21 | 5.34 | 3.39e-08 |
| Genes with expression level up regulated in mir-35 mutants comparing with N2. | 15 | 8.58 | 6.47e-08 |
| Genes with changed expression in lin-54(n2290) embryo. | 19 | 5.87 | 7.69e-08 |
| C-lineage related expression profile. WBPaper00025032:cluster\_14 | 8 | 32.17 | 8.19e-08 |
| Strictly embryonic (SE) subclasses are based on the earliest significant increase(abbreviated pi for primary increase). [cgc5767]:expression\_class\_SE\_pi(53\_min) | 12 | 10.90 | 4.08e-07 |
| Genes upregulated by fasting anytime during the 48 hour time course in N2 worms. | 21 | 3.88 | 8.34e-06 |
| Embryonic (E) subclasses are based on the earliest significant increase(abbreviated pi for primary increase). [cgc5767]:expression\_class\_E\_pi(23\_min) | 16 | 5.34 | 9.66e-06 |
| Strictly embryonic (SE) subclasses are based on the earliest significant increase(abbreviated pi for primary increase). [cgc5767]:expression\_class\_SE\_pi(41\_min) | 6 | 31.75 | 1.55e-05 |
| Strictly embryonic (SE) subclasses are based on the earliest significant increase(abbreviated pi for primary increase). [cgc5767]:expression\_class\_SE\_pi(23\_min) | 8 | 15.27 | 2.20e-05 |
| Embryonic class (E): genes that significantly increase in abundance at some point during embryogenesis. | 30 | 2.54 | 2.62e-05 |
| Genes significantly enriched (> 2x, FDR < 5%) in a particular cell-type versus a reference sample of all cells at the same stage. WBPaper00037950:hypodermis\_embryo\_enriched | 13 | 5.34 | 2.19e-04 |
| Significantly downregulated genes from cyc-1(RNAi) microarrays using SAM algorithm with an FDR < 0.1 from adult-only chips. | 27 | 2.44 | 4.14e-04 |
| Genes upregulated by fasting anytime between 9 hour to 12 hour time course in N2 worms. | 12 | 5.31 | 6.41e-04 |
| Differentially expressed genes during worm lifespan. Medoid 1 Fig.4. | 9 | 7.52 | 9.02e-04 |
| Early embryonic development gene expression profile. [cgc5767]:cluster\_10 | 5 | 20.38 | 1.51e-03 |
| Germline-enriched and sex-biased expression profile cluster E. | 15 | 3.80 | 1.54e-03 |
| Genes downregulated in rde-4(-/-) adult animals by at least 1.5 fold and P < 0.05, as determined by a multisample t-test. | 5 | 19.58 | 1.79e-03 |
| Embryonic transient (ET) subclasses are based on time of max abundance. [cgc5767]:expression\_class\_ET\_max(101\_min) | 7 | 9.73 | 2.23e-03 |
| Genes with decreased expression after 24 hours of infection by P.lumniescens Fold changes shown are pathogen vs OP50. WBPaper00038438:P.lumniescens\_24hr\_downregulated\_TilingArray | 8 | 7.71 | 2.53e-03 |
| Embryonic (E) subclasses are based on the earliest significant increase(abbreviated pi for primary increase). [cgc5767]:expression\_class\_E\_pi(53\_min) | 12 | 4.18 | 5.74e-03 |
| Genes that showed significantly changed expression during aging (ANOVA, p < 0.0001) | 14 | 3.46 | 8.07e-03 |
| Genes upregulated more than 2-fold by fasting for 48 hours in N2 and in kgb-1(km21). | 8 | 5.28 | 2.80e-02 |
| mixed oogenesis/somatic | 9 | 4.29 | 4.51e-02 |

### Motifs enriched

|  |  |  |  |  |  |
| --- | --- | --- | --- | --- | --- |
| **Motif** | **Logo** | **Possible orthologs** | **Number of motifs in cluster** | **Enrichment** | **FDR corrected p** |
| pTH8982 |  | ceh-48 | 34 | 3.47 | 4.4e-10 |
| SRF\_1 |  | unc-120 | 33 | 2.24 | 5.0e-05 |
| ONECUT1\_2 |  | dsc-1 ceh-48 | 39 | 1.87 | 9.6e-05 |
| pTH8411 |  | tbx-39 | 34 | 1.84 | 1.8e-03 |
| V$CDPCR1\_01 |  | ceh-48 | 26 | 2.18 | 2.8e-03 |
| MA0536.1 |  | elt-1 lin-39 | 43 | 1.52 | 2.8e-03 |
| V$NCX\_01 |  | ceh-19 | 44 | 1.39 | 1.5e-02 |
| MA0029.1 |  | ztf-29 | 40 | 1.47 | 1.9e-02 |
| Nkx2-3\_3435 |  | ceh-24 | 43 | 1.40 | 1.9e-02 |
| ZBTB49\_1 |  | C46E10.9 | 14 | 2.86 | 2.0e-02 |
| NDF1\_f1 |  | ngn-1 | 18 | 2.38 | 2.0e-02 |
| MA0139.1 |  | F58G1.2 | 42 | 1.42 | 2.1e-02 |
| MA0078.1 |  | sox-4 | 32 | 1.66 | 2.5e-02 |
| MA0161.1 |  | nfi-1 | 32 | 1.65 | 2.5e-02 |
| Ceh-22 |  | dsc-1 | 47 | 1.29 | 2.9e-02 |
| RFX1\_4537 |  | daf-19 | 46 | 1.31 | 3.4e-02 |
| MEIS2\_do |  | ceh-32 | 27 | 1.77 | 3.5e-02 |
| Oli\_da\_SANGER\_5\_1\_FBgn0032651 |  | hlh-32 | 47 | 1.29 | 3.5e-02 |
| HLF\_si |  | ces-2 | 24 | 1.89 | 3.6e-02 |
| CG12029\_SANGER\_10\_FBgn0035454 |  | klf-1 | 39 | 1.43 | 4.4e-02 |
| MA0505.1 |  | nhr-68 | 45 | 1.31 | 4.4e-02 |
| tj\_SANGER\_5\_FBgn0000964 |  | F45H11.6 | 27 | 1.74 | 4.6e-02 |
| pTH10796 |  | hsf-1 | 40 | 1.41 | 4.7e-02 |

### Correlated (and anti-correlated) transcription factors

|  |  |
| --- | --- |
| **Transcription factor** | **Correlation** |
| ceh-51 | 0.86 |
| nhr-2 | 0.84 |
| tbx-9 | 0.81 |
| aptf-2 | 0.77 |
| nhr-246 | 0.77 |
| nhr-171 | 0.76 |
| cep-1 | 0.75 |
| Y57A10A.31 | 0.75 |
| ceh-83 | 0.72 |
| ceh-91 | 0.71 |
| pax-3 | 0.71 |
| pqn-75 | 0.70 |
| ceh-40 | 0.70 |
| elt-7 | 0.69 |
| tbx-8 | 0.68 |
| cey-3 | 0.68 |
| nhr-232 | 0.67 |
| ubxn-1 | 0.64 |
| hmg-3 | 0.64 |
| zip-12 | 0.62 |
| ztf-15 | 0.62 |
| cey-2 | 0.62 |
| F49E8.2 | 0.61 |
| zip-11 | 0.61 |
| atf-7 | 0.60 |
| nhr-146 | -0.50 |
| C35D6.4 | -0.50 |
| alr-1 | -0.51 |
| nhr-50 | -0.51 |
| nhr-188 | -0.52 |
| nhr-31 | -0.52 |
| nhr-216 | -0.53 |
| elt-6 | -0.53 |
| nhr-14 | -0.54 |
| let-381 | -0.55 |
| Y55F3AM.14 | -0.56 |
| blmp-1 | -0.56 |
| gmeb-1 | -0.56 |
| mxl-3 | -0.56 |
| nhr-187 | -0.56 |
| nhr-87 | -0.57 |
| nhr-91 | -0.59 |
| egl-38 | -0.60 |
| pros-1 | -0.63 |
| hlh-4 | -0.63 |
| ccch-1 | -0.63 |
| nhr-63 | -0.65 |
| madf-1 | -0.66 |
| atf-8 | -0.68 |
| bed-3 | -0.74 |

### ChIP peaks enriched

none found
