# Supplementary material for: Overlapping cell population expression profiling and regulatory inference in C. elegans
Source: BMC Genomics. 2016 Feb 29;17:159. doi: 10.1186/s12864-016-2482-z (PMC4772325; doi:10.1186/s12864-016-2482-z)
Supplement: Additional file 13: — Web supplement. (DOC 21 kb) [file 12864_2016_2482_MOESM13_ESM.zip › sortWeb/clusters/hier.300.clusters/205.html]

Cluster 205 

## Cluster 205

### Expression

| cnd-1 rep. 1 | cnd-1 rep. 2 | cnd-1 rep. 3 | pha-4 rep. 1 | pha-4 rep. 2 | pha-4 rep. 3 | ceh-27 | ceh-36 | ceh-6 | F21D5.9 | mir-57 | mls-2 | pal-1 | pros-1 | ttx-3 | unc-130 | hlh-16 | irx-1 | ceh-6 (+) hlh-16 (+) | ceh-6 (+) hlh-16 (-) | ceh-6 (-) hlh-16 (+) | cnd-1 singlets | pha-4 singlets | 0 | 60 | 120 | 150 | 180 | 240 | 330 | 390 | 420 | 480 | 540 | 570 | 600 | 630 | 660 | NAME | Functional description |
| --- | --- | --- | --- | --- | --- | --- | --- | --- | --- | --- | --- | --- | --- | --- | --- | --- | --- | --- | --- | --- | --- | --- | --- | --- | --- | --- | --- | --- | --- | --- | --- | --- | --- | --- | --- | --- | --- | --- | --- |
|  |  |  |  |  |  |  |  |  |  |  |  |  |  |  |  |  |  |  |  |  |  |  |  |  |  |  |  |  |  |  |  |  |  |  |  |  |  | H21P03.2 |  |
|  |  |  |  |  |  |  |  |  |  |  |  |  |  |  |  |  |  |  |  |  |  |  |  |  |  |  |  |  |  |  |  |  |  |  |  |  |  | W03F9.2 |  |
|  |  |  |  |  |  |  |  |  |  |  |  |  |  |  |  |  |  |  |  |  |  |  |  |  |  |  |  |  |  |  |  |  |  |  |  |  |  | Y57G11C.43 |  |
|  |  |  |  |  |  |  |  |  |  |  |  |  |  |  |  |  |  |  |  |  |  |  |  |  |  |  |  |  |  |  |  |  |  |  |  |  |  | C01G5.5 |  |
|  |  |  |  |  |  |  |  |  |  |  |  |  |  |  |  |  |  |  |  |  |  |  |  |  |  |  |  |  |  |  |  |  |  |  |  |  |  | Y47H9C.8 |  |
|  |  |  |  |  |  |  |  |  |  |  |  |  |  |  |  |  |  |  |  |  |  |  |  |  |  |  |  |  |  |  |  |  |  |  |  |  |  | F01D4.5 |  |
|  |  |  |  |  |  |  |  |  |  |  |  |  |  |  |  |  |  |  |  |  |  |  |  |  |  |  |  |  |  |  |  |  |  |  |  |  |  | R04F11.3 |  |
|  |  |  |  |  |  |  |  |  |  |  |  |  |  |  |  |  |  |  |  |  |  |  |  |  |  |  |  |  |  |  |  |  |  |  |  |  |  | *prp-4* | yeast PRP (splicing factor) related |
|  |  |  |  |  |  |  |  |  |  |  |  |  |  |  |  |  |  |  |  |  |  |  |  |  |  |  |  |  |  |  |  |  |  |  |  |  |  | *dnj-22* | DNaJ domain (prokaryotic heat shock protein) |
|  |  |  |  |  |  |  |  |  |  |  |  |  |  |  |  |  |  |  |  |  |  |  |  |  |  |  |  |  |  |  |  |  |  |  |  |  |  | *emb-27* | abnormal EMBroygenesis |
|  |  |  |  |  |  |  |  |  |  |  |  |  |  |  |  |  |  |  |  |  |  |  |  |  |  |  |  |  |  |  |  |  |  |  |  |  |  | F23A7.6 |  |
|  |  |  |  |  |  |  |  |  |  |  |  |  |  |  |  |  |  |  |  |  |  |  |  |  |  |  |  |  |  |  |  |  |  |  |  |  |  | *hyls-1* | HYLS1 (human HYdroLethalus Syndrome) homolog |
|  |  |  |  |  |  |  |  |  |  |  |  |  |  |  |  |  |  |  |  |  |  |  |  |  |  |  |  |  |  |  |  |  |  |  |  |  |  | *lin-61* | abnormal cell LINeage |
|  |  |  |  |  |  |  |  |  |  |  |  |  |  |  |  |  |  |  |  |  |  |  |  |  |  |  |  |  |  |  |  |  |  |  |  |  |  | T24B8.2 |  |
|  |  |  |  |  |  |  |  |  |  |  |  |  |  |  |  |  |  |  |  |  |  |  |  |  |  |  |  |  |  |  |  |  |  |  |  |  |  | C27C12.1 |  |
|  |  |  |  |  |  |  |  |  |  |  |  |  |  |  |  |  |  |  |  |  |  |  |  |  |  |  |  |  |  |  |  |  |  |  |  |  |  | *rnh-2* | RNase H |
|  |  |  |  |  |  |  |  |  |  |  |  |  |  |  |  |  |  |  |  |  |  |  |  |  |  |  |  |  |  |  |  |  |  |  |  |  |  | F44B9.8 |  |
|  |  |  |  |  |  |  |  |  |  |  |  |  |  |  |  |  |  |  |  |  |  |  |  |  |  |  |  |  |  |  |  |  |  |  |  |  |  | *aly-2* | Ref/ALY RNA export adaptor family |
|  |  |  |  |  |  |  |  |  |  |  |  |  |  |  |  |  |  |  |  |  |  |  |  |  |  |  |  |  |  |  |  |  |  |  |  |  |  | Y39B6A.36 |  |
|  |  |  |  |  |  |  |  |  |  |  |  |  |  |  |  |  |  |  |  |  |  |  |  |  |  |  |  |  |  |  |  |  |  |  |  |  |  | Y39G8B.2 |  |
|  |  |  |  |  |  |  |  |  |  |  |  |  |  |  |  |  |  |  |  |  |  |  |  |  |  |  |  |  |  |  |  |  |  |  |  |  |  | *rnr-2* | RiboNucleotide Reductase |
|  |  |  |  |  |  |  |  |  |  |  |  |  |  |  |  |  |  |  |  |  |  |  |  |  |  |  |  |  |  |  |  |  |  |  |  |  |  | C01G10.9 |  |
|  |  |  |  |  |  |  |  |  |  |  |  |  |  |  |  |  |  |  |  |  |  |  |  |  |  |  |  |  |  |  |  |  |  |  |  |  |  | *thoc-3* | THO Complex (transcription factor/nuclear export) subunit |
|  |  |  |  |  |  |  |  |  |  |  |  |  |  |  |  |  |  |  |  |  |  |  |  |  |  |  |  |  |  |  |  |  |  |  |  |  |  | *csn-5* | COP-9 SigNalosome subunit |
|  |  |  |  |  |  |  |  |  |  |  |  |  |  |  |  |  |  |  |  |  |  |  |  |  |  |  |  |  |  |  |  |  |  |  |  |  |  | Y47G6A.9 |  |
|  |  |  |  |  |  |  |  |  |  |  |  |  |  |  |  |  |  |  |  |  |  |  |  |  |  |  |  |  |  |  |  |  |  |  |  |  |  | F30A10.9 |  |
|  |  |  |  |  |  |  |  |  |  |  |  |  |  |  |  |  |  |  |  |  |  |  |  |  |  |  |  |  |  |  |  |  |  |  |  |  |  | F52C12.6 |  |
|  |  |  |  |  |  |  |  |  |  |  |  |  |  |  |  |  |  |  |  |  |  |  |  |  |  |  |  |  |  |  |  |  |  |  |  |  |  | *kin-10* | protein KINase |
|  |  |  |  |  |  |  |  |  |  |  |  |  |  |  |  |  |  |  |  |  |  |  |  |  |  |  |  |  |  |  |  |  |  |  |  |  |  | Y75B8A.31 |  |
|  |  |  |  |  |  |  |  |  |  |  |  |  |  |  |  |  |  |  |  |  |  |  |  |  |  |  |  |  |  |  |  |  |  |  |  |  |  | Y52B11A.11 |  |
|  |  |  |  |  |  |  |  |  |  |  |  |  |  |  |  |  |  |  |  |  |  |  |  |  |  |  |  |  |  |  |  |  |  |  |  |  |  | K08D10.12 |  |
|  |  |  |  |  |  |  |  |  |  |  |  |  |  |  |  |  |  |  |  |  |  |  |  |  |  |  |  |  |  |  |  |  |  |  |  |  |  | Y38A10A.7 |  |
|  |  |  |  |  |  |  |  |  |  |  |  |  |  |  |  |  |  |  |  |  |  |  |  |  |  |  |  |  |  |  |  |  |  |  |  |  |  | ZK1067.3 |  |
|  |  |  |  |  |  |  |  |  |  |  |  |  |  |  |  |  |  |  |  |  |  |  |  |  |  |  |  |  |  |  |  |  |  |  |  |  |  | *rde-4* | RNAi DEfective |
|  |  |  |  |  |  |  |  |  |  |  |  |  |  |  |  |  |  |  |  |  |  |  |  |  |  |  |  |  |  |  |  |  |  |  |  |  |  | *fbxb-76* | F-box B protein |
|  |  |  |  |  |  |  |  |  |  |  |  |  |  |  |  |  |  |  |  |  |  |  |  |  |  |  |  |  |  |  |  |  |  |  |  |  |  | *lab-1* | Long Arms of the Bivalent protein |
|  |  |  |  |  |  |  |  |  |  |  |  |  |  |  |  |  |  |  |  |  |  |  |  |  |  |  |  |  |  |  |  |  |  |  |  |  |  | F34D6.1 |  |
|  |  |  |  |  |  |  |  |  |  |  |  |  |  |  |  |  |  |  |  |  |  |  |  |  |  |  |  |  |  |  |  |  |  |  |  |  |  | *zip-8* | bZIP transcription factor family |
|  |  |  |  |  |  |  |  |  |  |  |  |  |  |  |  |  |  |  |  |  |  |  |  |  |  |  |  |  |  |  |  |  |  |  |  |  |  | *skr-13* | SKp1 Related (ubiquitin ligase complex component) |
|  |  |  |  |  |  |  |  |  |  |  |  |  |  |  |  |  |  |  |  |  |  |  |  |  |  |  |  |  |  |  |  |  |  |  |  |  |  | F02D10.6 |  |
|  |  |  |  |  |  |  |  |  |  |  |  |  |  |  |  |  |  |  |  |  |  |  |  |  |  |  |  |  |  |  |  |  |  |  |  |  |  | F39F10.4 |  |
|  |  |  |  |  |  |  |  |  |  |  |  |  |  |  |  |  |  |  |  |  |  |  |  |  |  |  |  |  |  |  |  |  |  |  |  |  |  | W06E11.1 |  |
|  |  |  |  |  |  |  |  |  |  |  |  |  |  |  |  |  |  |  |  |  |  |  |  |  |  |  |  |  |  |  |  |  |  |  |  |  |  | F57G9.3 |  |
|  |  |  |  |  |  |  |  |  |  |  |  |  |  |  |  |  |  |  |  |  |  |  |  |  |  |  |  |  |  |  |  |  |  |  |  |  |  | T27A8.5 |  |
|  |  |  |  |  |  |  |  |  |  |  |  |  |  |  |  |  |  |  |  |  |  |  |  |  |  |  |  |  |  |  |  |  |  |  |  |  |  | F42H10.2 |  |
|  |  |  |  |  |  |  |  |  |  |  |  |  |  |  |  |  |  |  |  |  |  |  |  |  |  |  |  |  |  |  |  |  |  |  |  |  |  | W09B6.4 |  |
|  |  |  |  |  |  |  |  |  |  |  |  |  |  |  |  |  |  |  |  |  |  |  |  |  |  |  |  |  |  |  |  |  |  |  |  |  |  | M106.7 |  |
|  |  |  |  |  |  |  |  |  |  |  |  |  |  |  |  |  |  |  |  |  |  |  |  |  |  |  |  |  |  |  |  |  |  |  |  |  |  | *gst-1* | Glutathione S-Transferase |
|  |  |  |  |  |  |  |  |  |  |  |  |  |  |  |  |  |  |  |  |  |  |  |  |  |  |  |  |  |  |  |  |  |  |  |  |  |  | F10E9.11 |  |
|  |  |  |  |  |  |  |  |  |  |  |  |  |  |  |  |  |  |  |  |  |  |  |  |  |  |  |  |  |  |  |  |  |  |  |  |  |  | Y66D12A.7 |  |
|  |  |  |  |  |  |  |  |  |  |  |  |  |  |  |  |  |  |  |  |  |  |  |  |  |  |  |  |  |  |  |  |  |  |  |  |  |  | Y46B2A.3 |  |
|  |  |  |  |  |  |  |  |  |  |  |  |  |  |  |  |  |  |  |  |  |  |  |  |  |  |  |  |  |  |  |  |  |  |  |  |  |  | F11C1.9 |  |
|  |  |  |  |  |  |  |  |  |  |  |  |  |  |  |  |  |  |  |  |  |  |  |  |  |  |  |  |  |  |  |  |  |  |  |  |  |  | *hus-1* | human HUS1 related |
|  |  |  |  |  |  |  |  |  |  |  |  |  |  |  |  |  |  |  |  |  |  |  |  |  |  |  |  |  |  |  |  |  |  |  |  |  |  | C28C12.12 |  |
|  |  |  |  |  |  |  |  |  |  |  |  |  |  |  |  |  |  |  |  |  |  |  |  |  |  |  |  |  |  |  |  |  |  |  |  |  |  | *such-1* | SUppressor of spindle CHeckpoint defect |
|  |  |  |  |  |  |  |  |  |  |  |  |  |  |  |  |  |  |  |  |  |  |  |  |  |  |  |  |  |  |  |  |  |  |  |  |  |  | *hmg-12* | HMG |
|  |  |  |  |  |  |  |  |  |  |  |  |  |  |  |  |  |  |  |  |  |  |  |  |  |  |  |  |  |  |  |  |  |  |  |  |  |  | F58A4.2 |  |
|  |  |  |  |  |  |  |  |  |  |  |  |  |  |  |  |  |  |  |  |  |  |  |  |  |  |  |  |  |  |  |  |  |  |  |  |  |  | *fbxa-123* | F-box A protein |
|  |  |  |  |  |  |  |  |  |  |  |  |  |  |  |  |  |  |  |  |  |  |  |  |  |  |  |  |  |  |  |  |  |  |  |  |  |  | T01C3.9 |  |
|  |  |  |  |  |  |  |  |  |  |  |  |  |  |  |  |  |  |  |  |  |  |  |  |  |  |  |  |  |  |  |  |  |  |  |  |  |  | T09B4.2 |  |
|  |  |  |  |  |  |  |  |  |  |  |  |  |  |  |  |  |  |  |  |  |  |  |  |  |  |  |  |  |  |  |  |  |  |  |  |  |  | Y111B2A.2 |  |
|  |  |  |  |  |  |  |  |  |  |  |  |  |  |  |  |  |  |  |  |  |  |  |  |  |  |  |  |  |  |  |  |  |  |  |  |  |  | Y54E2A.5 |  |
|  |  |  |  |  |  |  |  |  |  |  |  |  |  |  |  |  |  |  |  |  |  |  |  |  |  |  |  |  |  |  |  |  |  |  |  |  |  | B0524.6 |  |
|  |  |  |  |  |  |  |  |  |  |  |  |  |  |  |  |  |  |  |  |  |  |  |  |  |  |  |  |  |  |  |  |  |  |  |  |  |  | *tag-179* | Temporarily Assigned Gene name |
|  |  |  |  |  |  |  |  |  |  |  |  |  |  |  |  |  |  |  |  |  |  |  |  |  |  |  |  |  |  |  |  |  |  |  |  |  |  | Y53C12A.10 |  |
|  |  |  |  |  |  |  |  |  |  |  |  |  |  |  |  |  |  |  |  |  |  |  |  |  |  |  |  |  |  |  |  |  |  |  |  |  |  | C16C10.8 |  |
|  |  |  |  |  |  |  |  |  |  |  |  |  |  |  |  |  |  |  |  |  |  |  |  |  |  |  |  |  |  |  |  |  |  |  |  |  |  | T01G1.4 |  |
|  |  |  |  |  |  |  |  |  |  |  |  |  |  |  |  |  |  |  |  |  |  |  |  |  |  |  |  |  |  |  |  |  |  |  |  |  |  | *exos-8* | EXOSome (multiexonuclease complex) component |
|  |  |  |  |  |  |  |  |  |  |  |  |  |  |  |  |  |  |  |  |  |  |  |  |  |  |  |  |  |  |  |  |  |  |  |  |  |  | ZC434.4 |  |
|  |  |  |  |  |  |  |  |  |  |  |  |  |  |  |  |  |  |  |  |  |  |  |  |  |  |  |  |  |  |  |  |  |  |  |  |  |  | C56A3.5 |  |
|  |  |  |  |  |  |  |  |  |  |  |  |  |  |  |  |  |  |  |  |  |  |  |  |  |  |  |  |  |  |  |  |  |  |  |  |  |  | K10C3.9 |  |
|  |  |  |  |  |  |  |  |  |  |  |  |  |  |  |  |  |  |  |  |  |  |  |  |  |  |  |  |  |  |  |  |  |  |  |  |  |  | F26A1.14 |  |
|  |  |  |  |  |  |  |  |  |  |  |  |  |  |  |  |  |  |  |  |  |  |  |  |  |  |  |  |  |  |  |  |  |  |  |  |  |  | *usp-3* | Ubiquitin Specific Protease |
|  |  |  |  |  |  |  |  |  |  |  |  |  |  |  |  |  |  |  |  |  |  |  |  |  |  |  |  |  |  |  |  |  |  |  |  |  |  | Y50D7A.2 |  |
|  |  |  |  |  |  |  |  |  |  |  |  |  |  |  |  |  |  |  |  |  |  |  |  |  |  |  |  |  |  |  |  |  |  |  |  |  |  | C44E4.2 |  |
|  |  |  |  |  |  |  |  |  |  |  |  |  |  |  |  |  |  |  |  |  |  |  |  |  |  |  |  |  |  |  |  |  |  |  |  |  |  | Y54G2A.21 |  |
|  |  |  |  |  |  |  |  |  |  |  |  |  |  |  |  |  |  |  |  |  |  |  |  |  |  |  |  |  |  |  |  |  |  |  |  |  |  | *rom-5* | RhOMboid (Drosophila) related |
|  |  |  |  |  |  |  |  |  |  |  |  |  |  |  |  |  |  |  |  |  |  |  |  |  |  |  |  |  |  |  |  |  |  |  |  |  |  | *nubp-1* | NUcleotide Binding Protein (Nubp1) homolog |
|  |  |  |  |  |  |  |  |  |  |  |  |  |  |  |  |  |  |  |  |  |  |  |  |  |  |  |  |  |  |  |  |  |  |  |  |  |  | Y81B9A.2 |  |
|  |  |  |  |  |  |  |  |  |  |  |  |  |  |  |  |  |  |  |  |  |  |  |  |  |  |  |  |  |  |  |  |  |  |  |  |  |  | C31C9.2 |  |
|  |  |  |  |  |  |  |  |  |  |  |  |  |  |  |  |  |  |  |  |  |  |  |  |  |  |  |  |  |  |  |  |  |  |  |  |  |  | *ubxn-1* | UBX-containing protein in Nematodes |
|  |  |  |  |  |  |  |  |  |  |  |  |  |  |  |  |  |  |  |  |  |  |  |  |  |  |  |  |  |  |  |  |  |  |  |  |  |  | R04D3.4 |  |
|  |  |  |  |  |  |  |  |  |  |  |  |  |  |  |  |  |  |  |  |  |  |  |  |  |  |  |  |  |  |  |  |  |  |  |  |  |  | *pqbp-1.2* | PQBP1 (polyglutamine tract-binding neurodegeneration protein) homolog |
|  |  |  |  |  |  |  |  |  |  |  |  |  |  |  |  |  |  |  |  |  |  |  |  |  |  |  |  |  |  |  |  |  |  |  |  |  |  | Y39E4B.14 |  |
|  |  |  |  |  |  |  |  |  |  |  |  |  |  |  |  |  |  |  |  |  |  |  |  |  |  |  |  |  |  |  |  |  |  |  |  |  |  | Y41D4A.5 |  |
|  |  |  |  |  |  |  |  |  |  |  |  |  |  |  |  |  |  |  |  |  |  |  |  |  |  |  |  |  |  |  |  |  |  |  |  |  |  | C50D2.8 |  |
|  |  |  |  |  |  |  |  |  |  |  |  |  |  |  |  |  |  |  |  |  |  |  |  |  |  |  |  |  |  |  |  |  |  |  |  |  |  | W08F4.13 |  |
|  |  |  |  |  |  |  |  |  |  |  |  |  |  |  |  |  |  |  |  |  |  |  |  |  |  |  |  |  |  |  |  |  |  |  |  |  |  | *npp-18* | Nuclear Pore complex Protein |
|  |  |  |  |  |  |  |  |  |  |  |  |  |  |  |  |  |  |  |  |  |  |  |  |  |  |  |  |  |  |  |  |  |  |  |  |  |  | *map-1* | Methionine AminoPeptidase |
|  |  |  |  |  |  |  |  |  |  |  |  |  |  |  |  |  |  |  |  |  |  |  |  |  |  |  |  |  |  |  |  |  |  |  |  |  |  | Y69H2.7 |  |
|  |  |  |  |  |  |  |  |  |  |  |  |  |  |  |  |  |  |  |  |  |  |  |  |  |  |  |  |  |  |  |  |  |  |  |  |  |  | C25A1.4 |  |
|  |  |  |  |  |  |  |  |  |  |  |  |  |  |  |  |  |  |  |  |  |  |  |  |  |  |  |  |  |  |  |  |  |  |  |  |  |  | *aly-3* | Ref/ALY RNA export adaptor family |
|  |  |  |  |  |  |  |  |  |  |  |  |  |  |  |  |  |  |  |  |  |  |  |  |  |  |  |  |  |  |  |  |  |  |  |  |  |  | K07H8.9 |  |
|  |  |  |  |  |  |  |  |  |  |  |  |  |  |  |  |  |  |  |  |  |  |  |  |  |  |  |  |  |  |  |  |  |  |  |  |  |  | Y116A8C.51 |  |
|  |  |  |  |  |  |  |  |  |  |  |  |  |  |  |  |  |  |  |  |  |  |  |  |  |  |  |  |  |  |  |  |  |  |  |  |  |  | T05D4.2 |  |
|  |  |  |  |  |  |  |  |  |  |  |  |  |  |  |  |  |  |  |  |  |  |  |  |  |  |  |  |  |  |  |  |  |  |  |  |  |  | K04G2.10 |  |
|  |  |  |  |  |  |  |  |  |  |  |  |  |  |  |  |  |  |  |  |  |  |  |  |  |  |  |  |  |  |  |  |  |  |  |  |  |  | *fbxb-72* | F-box B protein |
|  |  |  |  |  |  |  |  |  |  |  |  |  |  |  |  |  |  |  |  |  |  |  |  |  |  |  |  |  |  |  |  |  |  |  |  |  |  | R52.8 |  |
|  |  |  |  |  |  |  |  |  |  |  |  |  |  |  |  |  |  |  |  |  |  |  |  |  |  |  |  |  |  |  |  |  |  |  |  |  |  | K06B9.2 |  |
|  |  |  |  |  |  |  |  |  |  |  |  |  |  |  |  |  |  |  |  |  |  |  |  |  |  |  |  |  |  |  |  |  |  |  |  |  |  | F55F8.9 |  |
|  |  |  |  |  |  |  |  |  |  |  |  |  |  |  |  |  |  |  |  |  |  |  |  |  |  |  |  |  |  |  |  |  |  |  |  |  |  | *pme-2* | Poly(ADP-ribose) Metabolism Enzyme |
|  |  |  |  |  |  |  |  |  |  |  |  |  |  |  |  |  |  |  |  |  |  |  |  |  |  |  |  |  |  |  |  |  |  |  |  |  |  | C26E6.12 |  |
|  |  |  |  |  |  |  |  |  |  |  |  |  |  |  |  |  |  |  |  |  |  |  |  |  |  |  |  |  |  |  |  |  |  |  |  |  |  | *pph-6* | Protein PHosphatase |
|  |  |  |  |  |  |  |  |  |  |  |  |  |  |  |  |  |  |  |  |  |  |  |  |  |  |  |  |  |  |  |  |  |  |  |  |  |  | H04D03.4 |  |
|  |  |  |  |  |  |  |  |  |  |  |  |  |  |  |  |  |  |  |  |  |  |  |  |  |  |  |  |  |  |  |  |  |  |  |  |  |  | T27A3.7 |  |
|  |  |  |  |  |  |  |  |  |  |  |  |  |  |  |  |  |  |  |  |  |  |  |  |  |  |  |  |  |  |  |  |  |  |  |  |  |  | Y57A10A.27 |  |
|  |  |  |  |  |  |  |  |  |  |  |  |  |  |  |  |  |  |  |  |  |  |  |  |  |  |  |  |  |  |  |  |  |  |  |  |  |  | *unc-59* | UNCoordinated |
|  |  |  |  |  |  |  |  |  |  |  |  |  |  |  |  |  |  |  |  |  |  |  |  |  |  |  |  |  |  |  |  |  |  |  |  |  |  | *lmn-1* | nuclear LaMiN |
|  |  |  |  |  |  |  |  |  |  |  |  |  |  |  |  |  |  |  |  |  |  |  |  |  |  |  |  |  |  |  |  |  |  |  |  |  |  | C33F10.4 |  |
|  |  |  |  |  |  |  |  |  |  |  |  |  |  |  |  |  |  |  |  |  |  |  |  |  |  |  |  |  |  |  |  |  |  |  |  |  |  | T22C1.5 |  |
|  |  |  |  |  |  |  |  |  |  |  |  |  |  |  |  |  |  |  |  |  |  |  |  |  |  |  |  |  |  |  |  |  |  |  |  |  |  | T19F4.4 |  |
|  |  |  |  |  |  |  |  |  |  |  |  |  |  |  |  |  |  |  |  |  |  |  |  |  |  |  |  |  |  |  |  |  |  |  |  |  |  | *lact-5* | beta-LACTamase domain containing |
|  |  |  |  |  |  |  |  |  |  |  |  |  |  |  |  |  |  |  |  |  |  |  |  |  |  |  |  |  |  |  |  |  |  |  |  |  |  | *tbb-2* | TuBulin, Beta |
|  |  |  |  |  |  |  |  |  |  |  |  |  |  |  |  |  |  |  |  |  |  |  |  |  |  |  |  |  |  |  |  |  |  |  |  |  |  | T13G4.4 |  |
|  |  |  |  |  |  |  |  |  |  |  |  |  |  |  |  |  |  |  |  |  |  |  |  |  |  |  |  |  |  |  |  |  |  |  |  |  |  | F46H5.3 |  |
|  |  |  |  |  |  |  |  |  |  |  |  |  |  |  |  |  |  |  |  |  |  |  |  |  |  |  |  |  |  |  |  |  |  |  |  |  |  | D2096.1 |  |
|  |  |  |  |  |  |  |  |  |  |  |  |  |  |  |  |  |  |  |  |  |  |  |  |  |  |  |  |  |  |  |  |  |  |  |  |  |  | *cpt-6* | Carnitine Palmitoyl Transferase |

### Phenotypes enriched

none found

### Anatomy terms enriched

none found

### GO terms enriched

|  |  |  |
| --- | --- | --- |
| **GO term** | **Number of genes** | **FDR-corrected p-value** |
| cell division | 12 | 0.0055 |
| organelle fission | 10 | 0.0070 |

### Expression clusters enriched

|  |  |  |  |
| --- | --- | --- | --- |
| **Group name** | **Number in cluster** | **Enrichment** | **FDR corrected p** |
| Expression Pattern Group B, enriched for genes involved in embryonic development. These patterns have in common that they all have genes of which the expression goes up after the juvenile stage. The expression of the genes in these patterns remains high or even goes up after reproduction. | 41 | 3.87 | 6.82e-12 |
| TGF- Dauer pathway adult transcriptional targets. Results obtained by comparing the microarray results of the dauer-constitutive mutants daf-7(e1372), daf-7(m62), and daf-1(m40) with dauer-defective mutants daf-3(mgDf90), daf-5(e1386), and daf-7(e1372);daf-3(mgDf90) double mutants at the permissive temperature, 20C, on the first day of adulthood. WBPaper00031040:TGF-beta\_adult\_downregulated | 70 | 2.23 | 4.91e-11 |
| Caenorhabditis elegans Genes with expression levels changed significantly after treatment of Bacillus thurigiensis DB27. | 66 | 1.85 | 2.85e-06 |
| Germline-intrinsic transcripts. | 29 | 3.09 | 1.45e-05 |
| Genes down regulated by mir-243(n4759). | 35 | 2.47 | 8.86e-05 |
| Caenorhabditis elegans Genes with expression levels changed significantly after treatment of Xenorhabdus nematophila. | 79 | 1.52 | 1.59e-04 |
| Germline-enriched and sex-biased expression profile cluster E. | 24 | 2.70 | 2.05e-03 |
| Genes down-regulated after 300 um Tannic acid treatment. Fold change < 0.8. | 26 | 2.26 | 1.41e-02 |
| Embryonic class (E): genes that significantly increase in abundance at some point during embryogenesis. | 45 | 1.69 | 2.17e-02 |
| Maternal-embryonic class (ME): genes that are in the intersection of the maternal and embryonic classes. | 36 | 1.82 | 3.33e-02 |

### Motifs enriched

|  |  |  |  |  |  |
| --- | --- | --- | --- | --- | --- |
| **Motif** | **Logo** | **Possible orthologs** | **Number of motifs in cluster** | **Enrichment** | **FDR corrected p** |
| pTH9262 |  | lin-54 | 58 | 1.53 | 0.0098 |
| pTH9173 |  | efl-2 | 39 | 1.71 | 0.0220 |
| pTH9242 |  | mel-28 (0.68) | 69 | 1.35 | 0.0380 |
| Antp\_FlyReg\_FBgn0000095 |  | lin-39 | 66 | 1.37 | 0.0380 |
| pTH4425 |  | cfi-1 | 50 | 1.51 | 0.0410 |
| FOXC1\_1 |  | let-381 | 85 | 1.25 | 0.0450 |

### Correlated (and anti-correlated) transcription factors

|  |  |
| --- | --- |
| **Transcription factor** | **Correlation** |
| F23A7.6 | 0.89 |
| efl-1 | 0.89 |
| ubxn-1 | 0.87 |
| ekl-4 | 0.86 |
| dpl-1 | 0.85 |
| mig-5 | 0.85 |
| T22C8.3 | 0.85 |
| madf-8 | 0.84 |
| hmg-5 | 0.83 |
| ztf-18 | 0.83 |
| lir-2 | 0.82 |
| R151.8 | 0.82 |
| hmg-4 | 0.81 |
| isw-1 | 0.81 |
| dhhc-6 | 0.81 |
| Y82E9BR.1 | 0.80 |
| rcor-1 | 0.80 |
| T11G6.8 | 0.79 |
| flh-1 | 0.79 |
| hmg-20 | 0.78 |
| ztf-23 | 0.78 |
| nfi-1 | 0.78 |
| eor-1 | 0.77 |
| K09A11.1 | 0.77 |
| lin-38 | 0.76 |
| ccch-1 | -0.63 |
| bed-3 | -0.63 |
| sdz-38 | -0.63 |
| mdl-1 | -0.64 |
| nhr-5 | -0.65 |
| egl-38 | -0.65 |
| nhr-149 | -0.65 |
| ztf-27 | -0.66 |
| nhr-146 | -0.66 |
| zip-2 | -0.67 |
| nhr-96 | -0.67 |
| grh-1 | -0.69 |
| nhr-66 | -0.69 |
| gmeb-3 | -0.70 |
| mxl-3 | -0.72 |
| dhhc-2 | -0.73 |
| nhr-50 | -0.73 |
| atf-8 | -0.74 |
| nhr-63 | -0.74 |
| nhr-14 | -0.75 |
| ceh-18 | -0.75 |
| fos-1 | -0.75 |
| nhr-41 | -0.76 |
| nhr-58 | -0.77 |
| madf-1 | -0.79 |

### ChIP peaks enriched

|  |  |  |  |  |
| --- | --- | --- | --- | --- |
| **Gene** | **Experiment** | **Number of upstream peaks** | **Enrichment** | **FDR corrected p** |
| efl-1 | EFL-1\_Young-adult | 65 | 2.65 | 5.6e-14 |
| efl-1 | EFL-1\_Fed-L1-stage-larvae | 56 | 2.85 | 1.2e-12 |
| dpl-1 | DPL-1\_Young-adult | 53 | 2.78 | 2.4e-11 |
| F16B12.6 | F16B12.6\_Fed-L1-stage-larvae | 36 | 3.88 | 7.9e-11 |
| efl-1 | EFL-1\_Larvae-L1-stage | 60 | 2.41 | 1.1e-10 |
| dpl-1 | DPL-1\_Fed-L1-stage-larvae | 53 | 2.65 | 1.6e-10 |
| lin-35 | LIN-35\_Fed-L1-stage-larvae | 51 | 2.64 | 5.8e-10 |
| aly-2 | ALY-2\_Fed-L1-stage-larvae | 44 | 2.81 | 4.4e-09 |
| dpl-1 | DPL-1\_Larvae-L4-stage | 63 | 2.11 | 6.9e-09 |
| W03F9.2 | W03F9.2\_L4-Young-Adult-stage-larvae | 70 | 1.93 | 1.3e-08 |
| ceh-39 | CEH-39\_Embryos | 40 | 2.91 | 1.7e-08 |
| lin-15 | LIN-15B\_Fed-L1-stage-larvae | 38 | 2.90 | 6.4e-08 |
| lsy-2 | LSY-2\_Embryos | 41 | 2.68 | 1.1e-07 |
| lsy-2 | LSY-2\_Larvae-L1-stage | 57 | 2.09 | 1.7e-07 |
| C01B12.2 | C01B12.2\_Larvae-L2-stage | 61 | 1.99 | 1.8e-07 |
| hpl-2 | HPL-2\_Fed-L1-stage-larvae | 57 | 2.07 | 2.2e-07 |
| C16A3.4 | C16A3.4\_Fed-L1-stage-larvae | 40 | 2.58 | 5.0e-07 |
| eor-1 | EOR-1\_Larvae-L3-stage | 51 | 2.16 | 6.1e-07 |
| pes-1 | PES-1\_Larvae-L4-stage | 53 | 2.07 | 1.1e-06 |
| lsy-2 | LSY-2\_Fed-L1-stage-larvae | 47 | 2.24 | 1.2e-06 |
| C34F6.9 | C34F6.9\_Larvae-L2-stage | 49 | 2.14 | 2.1e-06 |
| gei-11 | GEI-11\_Larvae-L3-stage | 47 | 2.17 | 2.9e-06 |
| F45C12.2 | F45C12.2\_Fed-L1-stage-larvae | 43 | 2.29 | 3.3e-06 |
| nfya-1 | NFYA-1\_Late-Embryos | 44 | 2.21 | 6.0e-06 |
| gei-11 | GEI-11\_Fed-L1-stage-larvae | 44 | 2.09 | 2.6e-05 |
| R02D3.7 | R02D3.7\_Larvae-L3-stage | 47 | 2.00 | 3.2e-05 |
| ham-1 | HAM-1\_Fed-L1-stage-larvae | 45 | 2.03 | 4.4e-05 |
| nfya-1 | NFYA-1\_Larvae-L3-stage | 38 | 2.19 | 7.5e-05 |
| ham-1 | HAM-1\_Larvae-L4-stage | 46 | 1.93 | 1.2e-04 |
| egl-5 | EGL-5\_Larvae-L3-stage | 34 | 2.26 | 1.5e-04 |
| gei-11 | GEI-11\_Larvae-L2-stage | 35 | 2.18 | 2.3e-04 |
| alr-1 | ALR-1\_Larvae-L2-stage | 39 | 2.04 | 2.6e-04 |
| pha-4 | PHA-4\_Larvae-L2-stage | 47 | 1.84 | 3.0e-04 |
| lin-13 | LIN-13\_Larvae-L2-stage | 33 | 2.21 | 3.7e-04 |
| nhr-77 | NHR-77\_Fed-L1-stage-larvae | 41 | 1.96 | 3.8e-04 |
| ces-1 | CES-1\_Embryos | 43 | 1.90 | 4.5e-04 |
| fos-1 | FOS-1\_Fed-L1-stage-larvae | 40 | 1.97 | 4.6e-04 |
| F23B12.7 | F23B12.7\_Young-adult | 29 | 2.29 | 7.7e-04 |
| R02D3.7 | R02D3.7\_Larvae-L2-stage | 26 | 2.41 | 9.2e-04 |
| nhr-23 | NHR-23\_Larvae-L3-stage | 38 | 1.93 | 1.2e-03 |
| nhr-77 | NHR-77\_Larvae-L4-stage | 54 | 1.62 | 1.6e-03 |
| lin-15 | LIN-15B\_Larvae-L4-stage | 16 | 3.22 | 1.7e-03 |
| nhr-237 | NHR-237\_Embryos | 22 | 2.55 | 1.9e-03 |
| lsy-2 | LSY-2\_Larvae-L2-stage | 22 | 2.45 | 3.3e-03 |
| R02D3.7 | R02D3.7\_Larvae-L4-stage | 24 | 2.28 | 4.3e-03 |
| sem-4 | SEM-4\_Larvae-L2-stage | 39 | 1.76 | 6.1e-03 |
| ceh-38 | CEH-38\_Larvae-L3-stage | 38 | 1.76 | 7.9e-03 |
| nhr-129 | NHR-129\_Larvae-L2-stage | 49 | 1.58 | 8.1e-03 |
| ztf-7 | ZTF-7\_Larvae-L4-stage | 26 | 2.07 | 9.0e-03 |
| nhr-6 | NHR-6\_Larvae-L4-stage | 25 | 2.10 | 9.7e-03 |
| skn-1 | SKN-1\_Larvae-L3-stage | 22 | 2.23 | 1.1e-02 |
| mab-5 | MAB-5\_Larvae-L2-stage | 20 | 2.35 | 1.1e-02 |
| jun-1 | JUN-1\_Larvae-L1-stage | 31 | 1.88 | 1.1e-02 |
| nhr-76 | NHR-76\_Larvae-L4-stage | 23 | 2.17 | 1.2e-02 |
| aly-2 | ALY-2\_Larvae-L3-stage | 17 | 2.50 | 1.6e-02 |
| nhr-25 | NHR-25\_Larvae-L2-stage | 32 | 1.78 | 2.2e-02 |
| nhr-6 | NHR-6\_Larvae-L2-stage | 28 | 1.83 | 3.3e-02 |
| nhr-77 | NHR-77\_Larvae-L2-stage | 21 | 2.06 | 3.7e-02 |
