# Supplementary material for: Overlapping cell population expression profiling and regulatory inference in C. elegans
Source: BMC Genomics. 2016 Feb 29;17:159. doi: 10.1186/s12864-016-2482-z (PMC4772325; doi:10.1186/s12864-016-2482-z)
Supplement: Additional file 13: — Web supplement. (DOC 21 kb) [file 12864_2016_2482_MOESM13_ESM.zip › sortWeb/clusters/hier.300.clusters/208.html]

Cluster 208 

## Cluster 208

### Expression

| cnd-1 rep. 1 | cnd-1 rep. 2 | cnd-1 rep. 3 | pha-4 rep. 1 | pha-4 rep. 2 | pha-4 rep. 3 | ceh-27 | ceh-36 | ceh-6 | F21D5.9 | mir-57 | mls-2 | pal-1 | pros-1 | ttx-3 | unc-130 | hlh-16 | irx-1 | ceh-6 (+) hlh-16 (+) | ceh-6 (+) hlh-16 (-) | ceh-6 (-) hlh-16 (+) | cnd-1 singlets | pha-4 singlets | 0 | 60 | 120 | 150 | 180 | 240 | 330 | 390 | 420 | 480 | 540 | 570 | 600 | 630 | 660 | NAME | Functional description |
| --- | --- | --- | --- | --- | --- | --- | --- | --- | --- | --- | --- | --- | --- | --- | --- | --- | --- | --- | --- | --- | --- | --- | --- | --- | --- | --- | --- | --- | --- | --- | --- | --- | --- | --- | --- | --- | --- | --- | --- |
|  |  |  |  |  |  |  |  |  |  |  |  |  |  |  |  |  |  |  |  |  |  |  |  |  |  |  |  |  |  |  |  |  |  |  |  |  |  | T27A1.9 |  |
|  |  |  |  |  |  |  |  |  |  |  |  |  |  |  |  |  |  |  |  |  |  |  |  |  |  |  |  |  |  |  |  |  |  |  |  |  |  | Y55F3BR.10 |  |
|  |  |  |  |  |  |  |  |  |  |  |  |  |  |  |  |  |  |  |  |  |  |  |  |  |  |  |  |  |  |  |  |  |  |  |  |  |  | M163.8 |  |
|  |  |  |  |  |  |  |  |  |  |  |  |  |  |  |  |  |  |  |  |  |  |  |  |  |  |  |  |  |  |  |  |  |  |  |  |  |  | F40F11.3 |  |
|  |  |  |  |  |  |  |  |  |  |  |  |  |  |  |  |  |  |  |  |  |  |  |  |  |  |  |  |  |  |  |  |  |  |  |  |  |  | F32D8.4 |  |
|  |  |  |  |  |  |  |  |  |  |  |  |  |  |  |  |  |  |  |  |  |  |  |  |  |  |  |  |  |  |  |  |  |  |  |  |  |  | K04C2.8 |  |
|  |  |  |  |  |  |  |  |  |  |  |  |  |  |  |  |  |  |  |  |  |  |  |  |  |  |  |  |  |  |  |  |  |  |  |  |  |  | T22G5.3 |  |
|  |  |  |  |  |  |  |  |  |  |  |  |  |  |  |  |  |  |  |  |  |  |  |  |  |  |  |  |  |  |  |  |  |  |  |  |  |  | *snu-23* | SNU (yeast Small NUclear ribonucleoprotein associated) homolog |
|  |  |  |  |  |  |  |  |  |  |  |  |  |  |  |  |  |  |  |  |  |  |  |  |  |  |  |  |  |  |  |  |  |  |  |  |  |  | *fbxa-67* | F-box A protein |
|  |  |  |  |  |  |  |  |  |  |  |  |  |  |  |  |  |  |  |  |  |  |  |  |  |  |  |  |  |  |  |  |  |  |  |  |  |  | *let-49* | LEThal |
|  |  |  |  |  |  |  |  |  |  |  |  |  |  |  |  |  |  |  |  |  |  |  |  |  |  |  |  |  |  |  |  |  |  |  |  |  |  | F11C1.1 |  |
|  |  |  |  |  |  |  |  |  |  |  |  |  |  |  |  |  |  |  |  |  |  |  |  |  |  |  |  |  |  |  |  |  |  |  |  |  |  | *mdt-9* | MeDiaTor |
|  |  |  |  |  |  |  |  |  |  |  |  |  |  |  |  |  |  |  |  |  |  |  |  |  |  |  |  |  |  |  |  |  |  |  |  |  |  | *apc-11* | Anaphase Promoting Complex; see also mat |
|  |  |  |  |  |  |  |  |  |  |  |  |  |  |  |  |  |  |  |  |  |  |  |  |  |  |  |  |  |  |  |  |  |  |  |  |  |  | T12A2.7 |  |
|  |  |  |  |  |  |  |  |  |  |  |  |  |  |  |  |  |  |  |  |  |  |  |  |  |  |  |  |  |  |  |  |  |  |  |  |  |  | K01A2.12 |  |
|  |  |  |  |  |  |  |  |  |  |  |  |  |  |  |  |  |  |  |  |  |  |  |  |  |  |  |  |  |  |  |  |  |  |  |  |  |  | F23C8.3 |  |
|  |  |  |  |  |  |  |  |  |  |  |  |  |  |  |  |  |  |  |  |  |  |  |  |  |  |  |  |  |  |  |  |  |  |  |  |  |  | F39B2.3 |  |
|  |  |  |  |  |  |  |  |  |  |  |  |  |  |  |  |  |  |  |  |  |  |  |  |  |  |  |  |  |  |  |  |  |  |  |  |  |  | E01A2.8 |  |
|  |  |  |  |  |  |  |  |  |  |  |  |  |  |  |  |  |  |  |  |  |  |  |  |  |  |  |  |  |  |  |  |  |  |  |  |  |  | T25G12.6 |  |
|  |  |  |  |  |  |  |  |  |  |  |  |  |  |  |  |  |  |  |  |  |  |  |  |  |  |  |  |  |  |  |  |  |  |  |  |  |  | *srx-85* | Serpentine Receptor, class X |
|  |  |  |  |  |  |  |  |  |  |  |  |  |  |  |  |  |  |  |  |  |  |  |  |  |  |  |  |  |  |  |  |  |  |  |  |  |  | *gop-1* | Gro-1 OPeron gene |
|  |  |  |  |  |  |  |  |  |  |  |  |  |  |  |  |  |  |  |  |  |  |  |  |  |  |  |  |  |  |  |  |  |  |  |  |  |  | *haf-1* | HAlF transporter (PGP related) |
|  |  |  |  |  |  |  |  |  |  |  |  |  |  |  |  |  |  |  |  |  |  |  |  |  |  |  |  |  |  |  |  |  |  |  |  |  |  | *use-1* | Unconventional SNARE in the Endoplasmic Reticulum |
|  |  |  |  |  |  |  |  |  |  |  |  |  |  |  |  |  |  |  |  |  |  |  |  |  |  |  |  |  |  |  |  |  |  |  |  |  |  | B0207.6 |  |
|  |  |  |  |  |  |  |  |  |  |  |  |  |  |  |  |  |  |  |  |  |  |  |  |  |  |  |  |  |  |  |  |  |  |  |  |  |  | C40A11.6 |  |
|  |  |  |  |  |  |  |  |  |  |  |  |  |  |  |  |  |  |  |  |  |  |  |  |  |  |  |  |  |  |  |  |  |  |  |  |  |  | T14B4.3 |  |
|  |  |  |  |  |  |  |  |  |  |  |  |  |  |  |  |  |  |  |  |  |  |  |  |  |  |  |  |  |  |  |  |  |  |  |  |  |  | Y17G9B.11 |  |
|  |  |  |  |  |  |  |  |  |  |  |  |  |  |  |  |  |  |  |  |  |  |  |  |  |  |  |  |  |  |  |  |  |  |  |  |  |  | *aos-1* | Activator Of Sumo (yeast AOS homolog) |
|  |  |  |  |  |  |  |  |  |  |  |  |  |  |  |  |  |  |  |  |  |  |  |  |  |  |  |  |  |  |  |  |  |  |  |  |  |  | F58B3.7 |  |
|  |  |  |  |  |  |  |  |  |  |  |  |  |  |  |  |  |  |  |  |  |  |  |  |  |  |  |  |  |  |  |  |  |  |  |  |  |  | *syx-3* | SYntaXin |
|  |  |  |  |  |  |  |  |  |  |  |  |  |  |  |  |  |  |  |  |  |  |  |  |  |  |  |  |  |  |  |  |  |  |  |  |  |  | *memb-2* | MEMBrin (SNARE protein) |
|  |  |  |  |  |  |  |  |  |  |  |  |  |  |  |  |  |  |  |  |  |  |  |  |  |  |  |  |  |  |  |  |  |  |  |  |  |  | *linc-31* | Long Intervening Non-Coding RNA |
|  |  |  |  |  |  |  |  |  |  |  |  |  |  |  |  |  |  |  |  |  |  |  |  |  |  |  |  |  |  |  |  |  |  |  |  |  |  | K11D12.12 |  |
|  |  |  |  |  |  |  |  |  |  |  |  |  |  |  |  |  |  |  |  |  |  |  |  |  |  |  |  |  |  |  |  |  |  |  |  |  |  | F26A1.1 |  |
|  |  |  |  |  |  |  |  |  |  |  |  |  |  |  |  |  |  |  |  |  |  |  |  |  |  |  |  |  |  |  |  |  |  |  |  |  |  | D1054.3 |  |
|  |  |  |  |  |  |  |  |  |  |  |  |  |  |  |  |  |  |  |  |  |  |  |  |  |  |  |  |  |  |  |  |  |  |  |  |  |  | F22B5.10 |  |
|  |  |  |  |  |  |  |  |  |  |  |  |  |  |  |  |  |  |  |  |  |  |  |  |  |  |  |  |  |  |  |  |  |  |  |  |  |  | *pir-1* | Phosphatase Interacting with RNA/RNP |
|  |  |  |  |  |  |  |  |  |  |  |  |  |  |  |  |  |  |  |  |  |  |  |  |  |  |  |  |  |  |  |  |  |  |  |  |  |  | *fbxb-78* | F-box B protein |
|  |  |  |  |  |  |  |  |  |  |  |  |  |  |  |  |  |  |  |  |  |  |  |  |  |  |  |  |  |  |  |  |  |  |  |  |  |  | C08F1.10 |  |
|  |  |  |  |  |  |  |  |  |  |  |  |  |  |  |  |  |  |  |  |  |  |  |  |  |  |  |  |  |  |  |  |  |  |  |  |  |  | EEED8.15 |  |
|  |  |  |  |  |  |  |  |  |  |  |  |  |  |  |  |  |  |  |  |  |  |  |  |  |  |  |  |  |  |  |  |  |  |  |  |  |  | F56A8.4 |  |
|  |  |  |  |  |  |  |  |  |  |  |  |  |  |  |  |  |  |  |  |  |  |  |  |  |  |  |  |  |  |  |  |  |  |  |  |  |  | F26A3.1 |  |
|  |  |  |  |  |  |  |  |  |  |  |  |  |  |  |  |  |  |  |  |  |  |  |  |  |  |  |  |  |  |  |  |  |  |  |  |  |  | C40A11.3 |  |
|  |  |  |  |  |  |  |  |  |  |  |  |  |  |  |  |  |  |  |  |  |  |  |  |  |  |  |  |  |  |  |  |  |  |  |  |  |  | *pabp-2* | PolyA Binding Protein (nuclear) |
|  |  |  |  |  |  |  |  |  |  |  |  |  |  |  |  |  |  |  |  |  |  |  |  |  |  |  |  |  |  |  |  |  |  |  |  |  |  | *cec-4* | C.Elegans Chromodomain protein |
|  |  |  |  |  |  |  |  |  |  |  |  |  |  |  |  |  |  |  |  |  |  |  |  |  |  |  |  |  |  |  |  |  |  |  |  |  |  | C01A2.5 |  |
|  |  |  |  |  |  |  |  |  |  |  |  |  |  |  |  |  |  |  |  |  |  |  |  |  |  |  |  |  |  |  |  |  |  |  |  |  |  | *adr-2* | Adenosine Deaminase acting on RNA |
|  |  |  |  |  |  |  |  |  |  |  |  |  |  |  |  |  |  |  |  |  |  |  |  |  |  |  |  |  |  |  |  |  |  |  |  |  |  | *mys-1* | MYST family histone acetyltransferase-like |
|  |  |  |  |  |  |  |  |  |  |  |  |  |  |  |  |  |  |  |  |  |  |  |  |  |  |  |  |  |  |  |  |  |  |  |  |  |  | C55A6.9 |  |
|  |  |  |  |  |  |  |  |  |  |  |  |  |  |  |  |  |  |  |  |  |  |  |  |  |  |  |  |  |  |  |  |  |  |  |  |  |  | *fbxb-77* | F-box B protein |
|  |  |  |  |  |  |  |  |  |  |  |  |  |  |  |  |  |  |  |  |  |  |  |  |  |  |  |  |  |  |  |  |  |  |  |  |  |  | *dhhc-10* | DHHC-types zinc finger protein |
|  |  |  |  |  |  |  |  |  |  |  |  |  |  |  |  |  |  |  |  |  |  |  |  |  |  |  |  |  |  |  |  |  |  |  |  |  |  | F48E3.6 |  |
|  |  |  |  |  |  |  |  |  |  |  |  |  |  |  |  |  |  |  |  |  |  |  |  |  |  |  |  |  |  |  |  |  |  |  |  |  |  | W10D9.2 |  |
|  |  |  |  |  |  |  |  |  |  |  |  |  |  |  |  |  |  |  |  |  |  |  |  |  |  |  |  |  |  |  |  |  |  |  |  |  |  | W10D9.1 |  |
|  |  |  |  |  |  |  |  |  |  |  |  |  |  |  |  |  |  |  |  |  |  |  |  |  |  |  |  |  |  |  |  |  |  |  |  |  |  | *pinn-1* | ParvulIN of Nematode |
|  |  |  |  |  |  |  |  |  |  |  |  |  |  |  |  |  |  |  |  |  |  |  |  |  |  |  |  |  |  |  |  |  |  |  |  |  |  | Y66D12A.10 |  |
|  |  |  |  |  |  |  |  |  |  |  |  |  |  |  |  |  |  |  |  |  |  |  |  |  |  |  |  |  |  |  |  |  |  |  |  |  |  | F15D4.2 |  |
|  |  |  |  |  |  |  |  |  |  |  |  |  |  |  |  |  |  |  |  |  |  |  |  |  |  |  |  |  |  |  |  |  |  |  |  |  |  | *snr-5* | Small Nuclear Ribonucleoprotein |
|  |  |  |  |  |  |  |  |  |  |  |  |  |  |  |  |  |  |  |  |  |  |  |  |  |  |  |  |  |  |  |  |  |  |  |  |  |  | *tin-10* | Transport to INner mitochondrial membrane (yeast TIM) |
|  |  |  |  |  |  |  |  |  |  |  |  |  |  |  |  |  |  |  |  |  |  |  |  |  |  |  |  |  |  |  |  |  |  |  |  |  |  | *djr-1.1* | DJ-1 (mammalian transcriptional regulator) Related |
|  |  |  |  |  |  |  |  |  |  |  |  |  |  |  |  |  |  |  |  |  |  |  |  |  |  |  |  |  |  |  |  |  |  |  |  |  |  | *mdt-20* | MeDiaTor |
|  |  |  |  |  |  |  |  |  |  |  |  |  |  |  |  |  |  |  |  |  |  |  |  |  |  |  |  |  |  |  |  |  |  |  |  |  |  | F37A4.5 |  |
|  |  |  |  |  |  |  |  |  |  |  |  |  |  |  |  |  |  |  |  |  |  |  |  |  |  |  |  |  |  |  |  |  |  |  |  |  |  | *sut-1* | SUppressor of Tau pathology |
|  |  |  |  |  |  |  |  |  |  |  |  |  |  |  |  |  |  |  |  |  |  |  |  |  |  |  |  |  |  |  |  |  |  |  |  |  |  | *smo-1* | SUMO (ubiquitin-related) homolog |
|  |  |  |  |  |  |  |  |  |  |  |  |  |  |  |  |  |  |  |  |  |  |  |  |  |  |  |  |  |  |  |  |  |  |  |  |  |  | *rsp-5* | SR Protein (splicing factor) |
|  |  |  |  |  |  |  |  |  |  |  |  |  |  |  |  |  |  |  |  |  |  |  |  |  |  |  |  |  |  |  |  |  |  |  |  |  |  | F28B3.6 |  |
|  |  |  |  |  |  |  |  |  |  |  |  |  |  |  |  |  |  |  |  |  |  |  |  |  |  |  |  |  |  |  |  |  |  |  |  |  |  | F56C11.3 |  |
|  |  |  |  |  |  |  |  |  |  |  |  |  |  |  |  |  |  |  |  |  |  |  |  |  |  |  |  |  |  |  |  |  |  |  |  |  |  | DY3.8 |  |
|  |  |  |  |  |  |  |  |  |  |  |  |  |  |  |  |  |  |  |  |  |  |  |  |  |  |  |  |  |  |  |  |  |  |  |  |  |  | *mage-1* | Melanoma-Associated-antiGEn homolog |
|  |  |  |  |  |  |  |  |  |  |  |  |  |  |  |  |  |  |  |  |  |  |  |  |  |  |  |  |  |  |  |  |  |  |  |  |  |  | C35D10.6 |  |
|  |  |  |  |  |  |  |  |  |  |  |  |  |  |  |  |  |  |  |  |  |  |  |  |  |  |  |  |  |  |  |  |  |  |  |  |  |  | F44E2.8 |  |
|  |  |  |  |  |  |  |  |  |  |  |  |  |  |  |  |  |  |  |  |  |  |  |  |  |  |  |  |  |  |  |  |  |  |  |  |  |  | F23F1.5 |  |
|  |  |  |  |  |  |  |  |  |  |  |  |  |  |  |  |  |  |  |  |  |  |  |  |  |  |  |  |  |  |  |  |  |  |  |  |  |  | C02F5.3 |  |
|  |  |  |  |  |  |  |  |  |  |  |  |  |  |  |  |  |  |  |  |  |  |  |  |  |  |  |  |  |  |  |  |  |  |  |  |  |  | T24H10.1 |  |
|  |  |  |  |  |  |  |  |  |  |  |  |  |  |  |  |  |  |  |  |  |  |  |  |  |  |  |  |  |  |  |  |  |  |  |  |  |  | R02D3.8 |  |
|  |  |  |  |  |  |  |  |  |  |  |  |  |  |  |  |  |  |  |  |  |  |  |  |  |  |  |  |  |  |  |  |  |  |  |  |  |  | *linc-29* | Long Intervening Non-Coding RNA |
|  |  |  |  |  |  |  |  |  |  |  |  |  |  |  |  |  |  |  |  |  |  |  |  |  |  |  |  |  |  |  |  |  |  |  |  |  |  | *mog-2* | Masculinisation Of Germline |
|  |  |  |  |  |  |  |  |  |  |  |  |  |  |  |  |  |  |  |  |  |  |  |  |  |  |  |  |  |  |  |  |  |  |  |  |  |  | F33A8.6 |  |
|  |  |  |  |  |  |  |  |  |  |  |  |  |  |  |  |  |  |  |  |  |  |  |  |  |  |  |  |  |  |  |  |  |  |  |  |  |  | *aptf-2* | AP-2 Transcription Factor family |
|  |  |  |  |  |  |  |  |  |  |  |  |  |  |  |  |  |  |  |  |  |  |  |  |  |  |  |  |  |  |  |  |  |  |  |  |  |  | Y80D3A.11 |  |
|  |  |  |  |  |  |  |  |  |  |  |  |  |  |  |  |  |  |  |  |  |  |  |  |  |  |  |  |  |  |  |  |  |  |  |  |  |  | *par-4* | abnormal embryonic PARtitioning of cytoplasm |
|  |  |  |  |  |  |  |  |  |  |  |  |  |  |  |  |  |  |  |  |  |  |  |  |  |  |  |  |  |  |  |  |  |  |  |  |  |  | W08E3.4 |  |
|  |  |  |  |  |  |  |  |  |  |  |  |  |  |  |  |  |  |  |  |  |  |  |  |  |  |  |  |  |  |  |  |  |  |  |  |  |  | *sre-54* | Serpentine Receptor, class E (epsilon) |
|  |  |  |  |  |  |  |  |  |  |  |  |  |  |  |  |  |  |  |  |  |  |  |  |  |  |  |  |  |  |  |  |  |  |  |  |  |  | *snrp-27* | Small Nuclear RibonucleoProtein homolog |
|  |  |  |  |  |  |  |  |  |  |  |  |  |  |  |  |  |  |  |  |  |  |  |  |  |  |  |  |  |  |  |  |  |  |  |  |  |  | F25H9.6 |  |
|  |  |  |  |  |  |  |  |  |  |  |  |  |  |  |  |  |  |  |  |  |  |  |  |  |  |  |  |  |  |  |  |  |  |  |  |  |  | C02F5.13 |  |
|  |  |  |  |  |  |  |  |  |  |  |  |  |  |  |  |  |  |  |  |  |  |  |  |  |  |  |  |  |  |  |  |  |  |  |  |  |  | C50F4.6 |  |
|  |  |  |  |  |  |  |  |  |  |  |  |  |  |  |  |  |  |  |  |  |  |  |  |  |  |  |  |  |  |  |  |  |  |  |  |  |  | F42A6.6 |  |
|  |  |  |  |  |  |  |  |  |  |  |  |  |  |  |  |  |  |  |  |  |  |  |  |  |  |  |  |  |  |  |  |  |  |  |  |  |  | Y43F8C.7 |  |
|  |  |  |  |  |  |  |  |  |  |  |  |  |  |  |  |  |  |  |  |  |  |  |  |  |  |  |  |  |  |  |  |  |  |  |  |  |  | C38D4.9 |  |
|  |  |  |  |  |  |  |  |  |  |  |  |  |  |  |  |  |  |  |  |  |  |  |  |  |  |  |  |  |  |  |  |  |  |  |  |  |  | C33F10.14 |  |
|  |  |  |  |  |  |  |  |  |  |  |  |  |  |  |  |  |  |  |  |  |  |  |  |  |  |  |  |  |  |  |  |  |  |  |  |  |  | *vps-2* | related to yeast Vacuolar Protein Sorting factor |
|  |  |  |  |  |  |  |  |  |  |  |  |  |  |  |  |  |  |  |  |  |  |  |  |  |  |  |  |  |  |  |  |  |  |  |  |  |  | *npl-4.1* | NPL (yeast Nuclear Protein Localization) homolog |
|  |  |  |  |  |  |  |  |  |  |  |  |  |  |  |  |  |  |  |  |  |  |  |  |  |  |  |  |  |  |  |  |  |  |  |  |  |  | *exos-1* | EXOSome (multiexonuclease complex) component |
|  |  |  |  |  |  |  |  |  |  |  |  |  |  |  |  |  |  |  |  |  |  |  |  |  |  |  |  |  |  |  |  |  |  |  |  |  |  | T10C6.5 |  |
|  |  |  |  |  |  |  |  |  |  |  |  |  |  |  |  |  |  |  |  |  |  |  |  |  |  |  |  |  |  |  |  |  |  |  |  |  |  | *rsp-2* | SR Protein (splicing factor) |
|  |  |  |  |  |  |  |  |  |  |  |  |  |  |  |  |  |  |  |  |  |  |  |  |  |  |  |  |  |  |  |  |  |  |  |  |  |  | *hmg-1.1* | HMG |
|  |  |  |  |  |  |  |  |  |  |  |  |  |  |  |  |  |  |  |  |  |  |  |  |  |  |  |  |  |  |  |  |  |  |  |  |  |  | *lin-52* | abnormal cell LINeage |
|  |  |  |  |  |  |  |  |  |  |  |  |  |  |  |  |  |  |  |  |  |  |  |  |  |  |  |  |  |  |  |  |  |  |  |  |  |  | *mdt-10* | MeDiaTor |
|  |  |  |  |  |  |  |  |  |  |  |  |  |  |  |  |  |  |  |  |  |  |  |  |  |  |  |  |  |  |  |  |  |  |  |  |  |  | *evl-20* | abnormal Eversion of VuLva |
|  |  |  |  |  |  |  |  |  |  |  |  |  |  |  |  |  |  |  |  |  |  |  |  |  |  |  |  |  |  |  |  |  |  |  |  |  |  | F25H2.4 |  |
|  |  |  |  |  |  |  |  |  |  |  |  |  |  |  |  |  |  |  |  |  |  |  |  |  |  |  |  |  |  |  |  |  |  |  |  |  |  | *lgg-1* | LC3, GABARAP and GATE-16 family |
|  |  |  |  |  |  |  |  |  |  |  |  |  |  |  |  |  |  |  |  |  |  |  |  |  |  |  |  |  |  |  |  |  |  |  |  |  |  | *mdt-11* | MeDiaTor |
|  |  |  |  |  |  |  |  |  |  |  |  |  |  |  |  |  |  |  |  |  |  |  |  |  |  |  |  |  |  |  |  |  |  |  |  |  |  | F26F4.12 |  |
|  |  |  |  |  |  |  |  |  |  |  |  |  |  |  |  |  |  |  |  |  |  |  |  |  |  |  |  |  |  |  |  |  |  |  |  |  |  | F26A3.7 |  |
|  |  |  |  |  |  |  |  |  |  |  |  |  |  |  |  |  |  |  |  |  |  |  |  |  |  |  |  |  |  |  |  |  |  |  |  |  |  | *cfim-1* | Cleavage Factor IM (CFIm) homolog |

### Phenotypes enriched

|  |  |  |  |
| --- | --- | --- | --- |
| **Group name** | **Number in cluster** | **Enrichment** | **FDR corrected p** |
| progeny variant (RNAi) | 18 | 3.28 | 0.0106 |
| sterile progeny (RNAi) | 18 | 3.28 | 0.0106 |

### Anatomy terms enriched

none found

### GO terms enriched

|  |  |  |
| --- | --- | --- |
| **GO term** | **Number of genes** | **FDR-corrected p-value** |
| nucleoplasm | 6 | 0.0039 |
| nucleus | 19 | 0.0057 |
| embryo development ending in birth or egg hatching | 30 | 0.0068 |
| gastrulation with mouth forming first | 5 | 0.0070 |
| positive regulation of biological process | 23 | 0.0170 |
| mediator complex | 3 | 0.0190 |
| RNA polymerase II transcription cofactor activity | 3 | 0.0210 |
| membrane-bounded organelle | 22 | 0.0250 |
| RNA binding | 7 | 0.0280 |
| morphogenesis of an epithelium | 9 | 0.0430 |
| positive regulation of growth rate | 18 | 0.0450 |

### Expression clusters enriched

|  |  |  |  |
| --- | --- | --- | --- |
| **Group name** | **Number in cluster** | **Enrichment** | **FDR corrected p** |
| TGF- Dauer pathway adult transcriptional targets. Results obtained by comparing the microarray results of the dauer-constitutive mutants daf-7(e1372), daf-7(m62), and daf-1(m40) with dauer-defective mutants daf-3(mgDf90), daf-5(e1386), and daf-7(e1372);daf-3(mgDf90) double mutants at the permissive temperature, 20C, on the first day of adulthood. WBPaper00031040:TGF-beta\_adult\_downregulated | 62 | 2.18 | 4.42e-09 |
| Caenorhabditis elegans Genes with expression levels changed significantly after treatment of Bacillus thurigiensis DB27. | 64 | 1.98 | 1.25e-07 |
| Expression Pattern Group F, enriched for genes involved in embryonic development. These patterns have in common that they all have genes of which the expression goes up after the juvenile stage. The expression of the genes in these patterns remains high or even goes up after reproduction. | 39 | 2.56 | 4.30e-06 |
| Early embryonic development gene expression profile. [cgc5767]:cluster\_8 | 8 | 11.17 | 2.33e-04 |
| Caenorhabditis elegans Genes with expression levels changed significantly after treatment of Xenorhabdus nematophila. | 71 | 1.51 | 8.32e-04 |
| Maternal class (M): genes that are called present in at least one of the three PC6 replicates. | 62 | 1.58 | 2.00e-03 |
| FBF-associated probe sets (FDR <2.25%) | 46 | 1.81 | 2.69e-03 |
| Maternal-embryonic class (ME): genes that are in the intersection of the maternal and embryonic classes. | 36 | 2.01 | 4.18e-03 |
| Expression Pattern Group B, enriched for genes involved in embryonic development. These patterns have in common that they all have genes of which the expression goes up after the juvenile stage. The expression of the genes in these patterns remains high or even goes up after reproduction. | 24 | 2.50 | 5.80e-03 |
| Germline-enriched and sex-biased expression profile cluster E. | 21 | 2.61 | 1.09e-02 |
| Genes expressed in embryonic motor neurons (identified by unc-4::GFP expressing cells). | 61 | 1.49 | 1.43e-02 |
| Germline-intrinsic transcripts. | 21 | 2.47 | 2.15e-02 |
| Genes with changed expression in lin-54(n2290) embryo. | 18 | 2.73 | 2.15e-02 |
| Genes for which heat shock F3 (fraction 3, containing heavy polysomes) versus control F3 is significantly increased. | 14 | 3.09 | 3.75e-02 |
| Developmentally modulated gene cluster. cgc4386\_cluster\_5\_3 | 6 | 7.28 | 3.96e-02 |

### Motifs enriched

|  |  |  |  |  |  |
| --- | --- | --- | --- | --- | --- |
| **Motif** | **Logo** | **Possible orthologs** | **Number of motifs in cluster** | **Enrichment** | **FDR corrected p** |
| pTH3846 |  | daf-16 (0.53) let-381 lin-31 pha-4 fkh-7 | 73 | 1.43 | 0.0020 |
| pTH9380 |  | mel-28 (0.62) | 56 | 1.59 | 0.0037 |
| pTH9285 |  | daf-19 | 43 | 1.77 | 0.0048 |
| pTH10656 |  | hlh-12 | 47 | 1.68 | 0.0061 |
| pTH6591 |  | lin-31 | 71 | 1.39 | 0.0089 |
| pTH9097 |  | Y116A8C.22 | 76 | 1.34 | 0.0099 |
| pTH9260 |  | mel-28 (0.62) | 82 | 1.29 | 0.0110 |
| pTH3043 |  | lin-31 | 70 | 1.38 | 0.0120 |
| pTH3866 |  | hlh-2 | 44 | 1.66 | 0.0140 |
| pTH5634 |  | daf-16 (0.53) lin-31 fkh-10 fkh-7 | 70 | 1.37 | 0.0150 |
| V$HFH8\_01 |  | let-381 lin-31 | 67 | 1.38 | 0.0200 |
| pTH5074 |  | hlh-15 | 39 | 1.70 | 0.0220 |
| pTH9335 |  | mel-28 (0.62) | 63 | 1.40 | 0.0230 |
| pTH2846 |  | lin-31 | 67 | 1.37 | 0.0250 |
| V$POU3F2\_02 |  | ceh-18 (-0.6) | 59 | 1.43 | 0.0270 |
| MA0069.1 |  | pax-3 | 21 | 2.24 | 0.0270 |
| pTH10042 |  | nhr-5 (-0.77) | 13 | 3.01 | 0.0290 |
| pTH9177 |  | F10B5.3 | 71 | 1.32 | 0.0330 |
| pTH7876 |  | mel-28 (0.62) | 56 | 1.44 | 0.0350 |
| HMGA1\_f1 |  | Y116A8C.22 | 78 | 1.27 | 0.0380 |
| Ara\_Cell\_FBgn0015904 |  | irx-1 | 73 | 1.30 | 0.0380 |
| pTH10798 |  | Y75B8A.6 | 41 | 1.61 | 0.0390 |
| pTH9958 |  | ztf-6 | 72 | 1.31 | 0.0400 |
| pTH8679 |  | pax-2 | 11 | 3.17 | 0.0460 |

### Correlated (and anti-correlated) transcription factors

|  |  |
| --- | --- |
| **Transcription factor** | **Correlation** |
| dhhc-10 | 0.90 |
| nfyc-1 | 0.89 |
| T22C8.3 | 0.89 |
| F37B4.10 | 0.87 |
| mig-5 | 0.87 |
| dhhc-1 | 0.85 |
| hmg-5 | 0.85 |
| lir-2 | 0.84 |
| K11D12.12 | 0.83 |
| R144.3 | 0.81 |
| efl-1 | 0.79 |
| F23A7.6 | 0.79 |
| hmg-1.1 | 0.78 |
| ztf-4 | 0.78 |
| D2030.7 | 0.78 |
| dhhc-6 | 0.77 |
| dpl-1 | 0.77 |
| K09A11.1 | 0.77 |
| R151.8 | 0.76 |
| ekl-4 | 0.75 |
| flh-3 | 0.75 |
| cey-4 | 0.75 |
| ztf-18 | 0.75 |
| madf-8 | 0.74 |
| isw-1 | 0.74 |
| bed-3 | -0.61 |
| nhr-184 | -0.61 |
| nhr-149 | -0.62 |
| nhr-112 | -0.62 |
| daf-12 | -0.63 |
| ccch-1 | -0.63 |
| nhr-66 | -0.65 |
| nhr-141 | -0.65 |
| nhr-204 | -0.67 |
| gmeb-3 | -0.68 |
| nhr-31 | -0.68 |
| grh-1 | -0.70 |
| atf-8 | -0.71 |
| blmp-1 | -0.72 |
| madf-1 | -0.72 |
| fkh-9 | -0.73 |
| dhhc-2 | -0.74 |
| nhr-70 | -0.74 |
| nhr-146 | -0.75 |
| nhr-58 | -0.75 |
| ztf-27 | -0.76 |
| nhr-5 | -0.77 |
| nhr-41 | -0.77 |
| fos-1 | -0.79 |
| nhr-14 | -0.80 |

### ChIP peaks enriched

|  |  |  |  |  |
| --- | --- | --- | --- | --- |
| **Gene** | **Experiment** | **Number of upstream peaks** | **Enrichment** | **FDR corrected p** |
| ceh-39 | CEH-39\_Embryos | 36 | 3.05 | 3.8e-08 |
| efl-1 | EFL-1\_Fed-L1-stage-larvae | 46 | 2.35 | 2.5e-07 |
| dpl-1 | DPL-1\_Fed-L1-stage-larvae | 42 | 2.32 | 2.5e-06 |
| lsy-2 | LSY-2\_Embryos | 35 | 2.52 | 7.2e-06 |
| efl-1 | EFL-1\_Larvae-L1-stage | 44 | 2.15 | 8.3e-06 |
| lin-35 | LIN-35\_Fed-L1-stage-larvae | 40 | 2.29 | 8.3e-06 |
| C16A3.4 | C16A3.4\_Fed-L1-stage-larvae | 35 | 2.49 | 9.7e-06 |
| efl-1 | EFL-1\_Young-adult | 46 | 2.07 | 1.1e-05 |
| pes-1 | PES-1\_Larvae-L4-stage | 42 | 2.03 | 8.1e-05 |
| lin-15 | LIN-15B\_Fed-L1-stage-larvae | 29 | 2.44 | 2.0e-04 |
| nfya-1 | NFYA-1\_Larvae-L3-stage | 34 | 2.16 | 3.3e-04 |
| lsy-2 | LSY-2\_Fed-L1-stage-larvae | 38 | 2.00 | 4.7e-04 |
| lin-15 | LIN-15B\_Larvae-L4-stage | 17 | 3.31 | 6.8e-04 |
| F16B12.6 | F16B12.6\_Fed-L1-stage-larvae | 23 | 2.57 | 1.0e-03 |
| eor-1 | EOR-1\_Larvae-L3-stage | 40 | 1.87 | 1.0e-03 |
| lsy-2 | LSY-2\_Larvae-L1-stage | 44 | 1.78 | 1.2e-03 |
| hpl-2 | HPL-2\_Fed-L1-stage-larvae | 44 | 1.76 | 1.4e-03 |
| dpl-1 | DPL-1\_Young-adult | 34 | 1.97 | 2.0e-03 |
| nfya-1 | NFYA-1\_Late-Embryos | 35 | 1.94 | 2.1e-03 |
| dpl-1 | DPL-1\_Larvae-L4-stage | 46 | 1.70 | 2.2e-03 |
| ces-1 | CES-1\_Embryos | 38 | 1.85 | 2.4e-03 |
| gei-11 | GEI-11\_Fed-L1-stage-larvae | 36 | 1.89 | 2.6e-03 |
| C34F6.9 | C34F6.9\_Larvae-L2-stage | 38 | 1.83 | 3.0e-03 |
| R02D3.7 | R02D3.7\_Larvae-L2-stage | 23 | 2.36 | 3.5e-03 |
| F45C12.2 | F45C12.2\_Fed-L1-stage-larvae | 33 | 1.94 | 3.6e-03 |
| nhr-237 | NHR-237\_Embryos | 17 | 2.78 | 4.8e-03 |
| R02D3.7 | R02D3.7\_Larvae-L3-stage | 38 | 1.78 | 4.8e-03 |
| lin-13 | LIN-13\_Larvae-L2-stage | 28 | 2.07 | 4.9e-03 |
| W03F9.2 | W03F9.2\_L4-Young-Adult-stage-larvae | 51 | 1.55 | 7.1e-03 |
| C01B12.2 | C01B12.2\_Larvae-L2-stage | 45 | 1.62 | 8.1e-03 |
| aly-2 | ALY-2\_Fed-L1-stage-larvae | 28 | 1.98 | 9.7e-03 |
| pha-4 | PHA-4\_Larvae-L2-stage | 39 | 1.69 | 1.2e-02 |
| dve-1 | DVE-1\_Late-Embryos | 30 | 1.87 | 1.4e-02 |
| ham-1 | HAM-1\_Larvae-L4-stage | 36 | 1.67 | 2.6e-02 |
| zag-1 | ZAG-1\_Larvae-L4-stage | 21 | 2.11 | 2.7e-02 |
| lsy-2 | LSY-2\_Larvae-L4-stage | 17 | 2.35 | 2.7e-02 |
| fos-1 | FOS-1\_Fed-L1-stage-larvae | 32 | 1.74 | 2.8e-02 |
| nhr-6 | NHR-6\_Larvae-L4-stage | 22 | 2.04 | 2.9e-02 |
| ham-1 | HAM-1\_Fed-L1-stage-larvae | 34 | 1.69 | 3.0e-02 |
| nhr-23 | NHR-23\_Larvae-L3-stage | 31 | 1.74 | 3.3e-02 |
| lin-13 | LIN-13\_Larvae-L4-stage | 21 | 2.05 | 3.6e-02 |
| lsy-2 | LSY-2\_Larvae-L2-stage | 18 | 2.21 | 3.7e-02 |
| gei-11 | GEI-11\_Larvae-L3-stage | 33 | 1.68 | 3.8e-02 |
| nhr-77 | NHR-77\_Fed-L1-stage-larvae | 32 | 1.69 | 4.4e-02 |
| skn-1 | SKN-1\_Larvae-L3-stage | 17 | 2.22 | 4.7e-02 |
