# Supplementary material for: Overlapping cell population expression profiling and regulatory inference in C. elegans
Source: BMC Genomics. 2016 Feb 29;17:159. doi: 10.1186/s12864-016-2482-z (PMC4772325; doi:10.1186/s12864-016-2482-z)
Supplement: Additional file 13: — Web supplement. (DOC 21 kb) [file 12864_2016_2482_MOESM13_ESM.zip › sortWeb/clusters/hier.300.clusters/212.html]

Cluster 212 

## Cluster 212

### Expression

| cnd-1 rep. 1 | cnd-1 rep. 2 | cnd-1 rep. 3 | pha-4 rep. 1 | pha-4 rep. 2 | pha-4 rep. 3 | ceh-27 | ceh-36 | ceh-6 | F21D5.9 | mir-57 | mls-2 | pal-1 | pros-1 | ttx-3 | unc-130 | hlh-16 | irx-1 | ceh-6 (+) hlh-16 (+) | ceh-6 (+) hlh-16 (-) | ceh-6 (-) hlh-16 (+) | cnd-1 singlets | pha-4 singlets | 0 | 60 | 120 | 150 | 180 | 240 | 330 | 390 | 420 | 480 | 540 | 570 | 600 | 630 | 660 | NAME | Functional description |
| --- | --- | --- | --- | --- | --- | --- | --- | --- | --- | --- | --- | --- | --- | --- | --- | --- | --- | --- | --- | --- | --- | --- | --- | --- | --- | --- | --- | --- | --- | --- | --- | --- | --- | --- | --- | --- | --- | --- | --- |
|  |  |  |  |  |  |  |  |  |  |  |  |  |  |  |  |  |  |  |  |  |  |  |  |  |  |  |  |  |  |  |  |  |  |  |  |  |  | C10G11.6 |  |
|  |  |  |  |  |  |  |  |  |  |  |  |  |  |  |  |  |  |  |  |  |  |  |  |  |  |  |  |  |  |  |  |  |  |  |  |  |  | *srh-158* | Serpentine Receptor, class H |
|  |  |  |  |  |  |  |  |  |  |  |  |  |  |  |  |  |  |  |  |  |  |  |  |  |  |  |  |  |  |  |  |  |  |  |  |  |  | Y46G5A.20 |  |
|  |  |  |  |  |  |  |  |  |  |  |  |  |  |  |  |  |  |  |  |  |  |  |  |  |  |  |  |  |  |  |  |  |  |  |  |  |  | *srj-34* | Serpentine Receptor, class J |
|  |  |  |  |  |  |  |  |  |  |  |  |  |  |  |  |  |  |  |  |  |  |  |  |  |  |  |  |  |  |  |  |  |  |  |  |  |  | *fbxa-136* | F-box A protein |
|  |  |  |  |  |  |  |  |  |  |  |  |  |  |  |  |  |  |  |  |  |  |  |  |  |  |  |  |  |  |  |  |  |  |  |  |  |  | R04D3.3 |  |
|  |  |  |  |  |  |  |  |  |  |  |  |  |  |  |  |  |  |  |  |  |  |  |  |  |  |  |  |  |  |  |  |  |  |  |  |  |  | *akir-1* | AKIRin (conserved nuclear protein family) homolog |
|  |  |  |  |  |  |  |  |  |  |  |  |  |  |  |  |  |  |  |  |  |  |  |  |  |  |  |  |  |  |  |  |  |  |  |  |  |  | *nud-2* | Aspergillus NUclear Division related |
|  |  |  |  |  |  |  |  |  |  |  |  |  |  |  |  |  |  |  |  |  |  |  |  |  |  |  |  |  |  |  |  |  |  |  |  |  |  | *ran-5* | associated with RAN (nuclear import/export) function |
|  |  |  |  |  |  |  |  |  |  |  |  |  |  |  |  |  |  |  |  |  |  |  |  |  |  |  |  |  |  |  |  |  |  |  |  |  |  | *srh-234* | Serpentine Receptor, class H |
|  |  |  |  |  |  |  |  |  |  |  |  |  |  |  |  |  |  |  |  |  |  |  |  |  |  |  |  |  |  |  |  |  |  |  |  |  |  | *stn-1* | SynTrophiN |
|  |  |  |  |  |  |  |  |  |  |  |  |  |  |  |  |  |  |  |  |  |  |  |  |  |  |  |  |  |  |  |  |  |  |  |  |  |  | *bca-1* | Beta Carbonic Anhydrase |
|  |  |  |  |  |  |  |  |  |  |  |  |  |  |  |  |  |  |  |  |  |  |  |  |  |  |  |  |  |  |  |  |  |  |  |  |  |  | C17E4.10 |  |
|  |  |  |  |  |  |  |  |  |  |  |  |  |  |  |  |  |  |  |  |  |  |  |  |  |  |  |  |  |  |  |  |  |  |  |  |  |  | W05H12.2 |  |
|  |  |  |  |  |  |  |  |  |  |  |  |  |  |  |  |  |  |  |  |  |  |  |  |  |  |  |  |  |  |  |  |  |  |  |  |  |  | F59F4.3 |  |
|  |  |  |  |  |  |  |  |  |  |  |  |  |  |  |  |  |  |  |  |  |  |  |  |  |  |  |  |  |  |  |  |  |  |  |  |  |  | T26C11.4 |  |
|  |  |  |  |  |  |  |  |  |  |  |  |  |  |  |  |  |  |  |  |  |  |  |  |  |  |  |  |  |  |  |  |  |  |  |  |  |  | F17C8.7 |  |
|  |  |  |  |  |  |  |  |  |  |  |  |  |  |  |  |  |  |  |  |  |  |  |  |  |  |  |  |  |  |  |  |  |  |  |  |  |  | D1054.1 |  |
|  |  |  |  |  |  |  |  |  |  |  |  |  |  |  |  |  |  |  |  |  |  |  |  |  |  |  |  |  |  |  |  |  |  |  |  |  |  | *paqr-1* | Progestin and AdipoQ Receptor family |
|  |  |  |  |  |  |  |  |  |  |  |  |  |  |  |  |  |  |  |  |  |  |  |  |  |  |  |  |  |  |  |  |  |  |  |  |  |  | F20G2.7 |  |
|  |  |  |  |  |  |  |  |  |  |  |  |  |  |  |  |  |  |  |  |  |  |  |  |  |  |  |  |  |  |  |  |  |  |  |  |  |  | *mtch-1* | MiTochondrial Carrier Homolog |
|  |  |  |  |  |  |  |  |  |  |  |  |  |  |  |  |  |  |  |  |  |  |  |  |  |  |  |  |  |  |  |  |  |  |  |  |  |  | ZC443.3 |  |
|  |  |  |  |  |  |  |  |  |  |  |  |  |  |  |  |  |  |  |  |  |  |  |  |  |  |  |  |  |  |  |  |  |  |  |  |  |  | C56A3.6 |  |
|  |  |  |  |  |  |  |  |  |  |  |  |  |  |  |  |  |  |  |  |  |  |  |  |  |  |  |  |  |  |  |  |  |  |  |  |  |  | *fut-3* | FUcosyl Transferase |
|  |  |  |  |  |  |  |  |  |  |  |  |  |  |  |  |  |  |  |  |  |  |  |  |  |  |  |  |  |  |  |  |  |  |  |  |  |  | R07G3.7 |  |
|  |  |  |  |  |  |  |  |  |  |  |  |  |  |  |  |  |  |  |  |  |  |  |  |  |  |  |  |  |  |  |  |  |  |  |  |  |  | *vps-22* | related to yeast Vacuolar Protein Sorting factor |
|  |  |  |  |  |  |  |  |  |  |  |  |  |  |  |  |  |  |  |  |  |  |  |  |  |  |  |  |  |  |  |  |  |  |  |  |  |  | *fbxa-10* | F-box A protein |
|  |  |  |  |  |  |  |  |  |  |  |  |  |  |  |  |  |  |  |  |  |  |  |  |  |  |  |  |  |  |  |  |  |  |  |  |  |  | *syx-16* | SYntaXin |
|  |  |  |  |  |  |  |  |  |  |  |  |  |  |  |  |  |  |  |  |  |  |  |  |  |  |  |  |  |  |  |  |  |  |  |  |  |  | C45B2.6 |  |
|  |  |  |  |  |  |  |  |  |  |  |  |  |  |  |  |  |  |  |  |  |  |  |  |  |  |  |  |  |  |  |  |  |  |  |  |  |  | *maco-1* | MACOilin homolog |
|  |  |  |  |  |  |  |  |  |  |  |  |  |  |  |  |  |  |  |  |  |  |  |  |  |  |  |  |  |  |  |  |  |  |  |  |  |  | *eat-16* | EATing: abnormal pharyngeal pumping |
|  |  |  |  |  |  |  |  |  |  |  |  |  |  |  |  |  |  |  |  |  |  |  |  |  |  |  |  |  |  |  |  |  |  |  |  |  |  | T25G3.4 |  |
|  |  |  |  |  |  |  |  |  |  |  |  |  |  |  |  |  |  |  |  |  |  |  |  |  |  |  |  |  |  |  |  |  |  |  |  |  |  | *tbc-16* | TBC (Tre-2/Bub2/Cdc16) domain family |
|  |  |  |  |  |  |  |  |  |  |  |  |  |  |  |  |  |  |  |  |  |  |  |  |  |  |  |  |  |  |  |  |  |  |  |  |  |  | *gpa-7* | G Protein, Alpha subunit |
|  |  |  |  |  |  |  |  |  |  |  |  |  |  |  |  |  |  |  |  |  |  |  |  |  |  |  |  |  |  |  |  |  |  |  |  |  |  | F39B2.8 |  |
|  |  |  |  |  |  |  |  |  |  |  |  |  |  |  |  |  |  |  |  |  |  |  |  |  |  |  |  |  |  |  |  |  |  |  |  |  |  | C42C1.2 |  |
|  |  |  |  |  |  |  |  |  |  |  |  |  |  |  |  |  |  |  |  |  |  |  |  |  |  |  |  |  |  |  |  |  |  |  |  |  |  | *snf-1* | Sodium: Neurotransmitter symporter Family |
|  |  |  |  |  |  |  |  |  |  |  |  |  |  |  |  |  |  |  |  |  |  |  |  |  |  |  |  |  |  |  |  |  |  |  |  |  |  | C04E7.4 |  |
|  |  |  |  |  |  |  |  |  |  |  |  |  |  |  |  |  |  |  |  |  |  |  |  |  |  |  |  |  |  |  |  |  |  |  |  |  |  | *sad-1* | Synapses of Amphids Defective |
|  |  |  |  |  |  |  |  |  |  |  |  |  |  |  |  |  |  |  |  |  |  |  |  |  |  |  |  |  |  |  |  |  |  |  |  |  |  | *aex-3* | ABoc, EXpulsion (defecation) defective |
|  |  |  |  |  |  |  |  |  |  |  |  |  |  |  |  |  |  |  |  |  |  |  |  |  |  |  |  |  |  |  |  |  |  |  |  |  |  | M03C11.1 |  |
|  |  |  |  |  |  |  |  |  |  |  |  |  |  |  |  |  |  |  |  |  |  |  |  |  |  |  |  |  |  |  |  |  |  |  |  |  |  | *fmi-1* | FlaMIngo (cadherin plus 7TM domain) homolog |
|  |  |  |  |  |  |  |  |  |  |  |  |  |  |  |  |  |  |  |  |  |  |  |  |  |  |  |  |  |  |  |  |  |  |  |  |  |  | *unc-104* | UNCoordinated |
|  |  |  |  |  |  |  |  |  |  |  |  |  |  |  |  |  |  |  |  |  |  |  |  |  |  |  |  |  |  |  |  |  |  |  |  |  |  | *cccp-1* | Conserved Coiled-Coil Protein |
|  |  |  |  |  |  |  |  |  |  |  |  |  |  |  |  |  |  |  |  |  |  |  |  |  |  |  |  |  |  |  |  |  |  |  |  |  |  | *tbc-18* | TBC (Tre-2/Bub2/Cdc16) domain family |
|  |  |  |  |  |  |  |  |  |  |  |  |  |  |  |  |  |  |  |  |  |  |  |  |  |  |  |  |  |  |  |  |  |  |  |  |  |  | Y106G6G.6 |  |
|  |  |  |  |  |  |  |  |  |  |  |  |  |  |  |  |  |  |  |  |  |  |  |  |  |  |  |  |  |  |  |  |  |  |  |  |  |  | T20F10.5 |  |
|  |  |  |  |  |  |  |  |  |  |  |  |  |  |  |  |  |  |  |  |  |  |  |  |  |  |  |  |  |  |  |  |  |  |  |  |  |  | F45E4.3 |  |
|  |  |  |  |  |  |  |  |  |  |  |  |  |  |  |  |  |  |  |  |  |  |  |  |  |  |  |  |  |  |  |  |  |  |  |  |  |  | *unc-41* | UNCoordinated |
|  |  |  |  |  |  |  |  |  |  |  |  |  |  |  |  |  |  |  |  |  |  |  |  |  |  |  |  |  |  |  |  |  |  |  |  |  |  | K09C4.10 |  |
|  |  |  |  |  |  |  |  |  |  |  |  |  |  |  |  |  |  |  |  |  |  |  |  |  |  |  |  |  |  |  |  |  |  |  |  |  |  | F46H5.7 |  |
|  |  |  |  |  |  |  |  |  |  |  |  |  |  |  |  |  |  |  |  |  |  |  |  |  |  |  |  |  |  |  |  |  |  |  |  |  |  | *nhr-47* | Nuclear Hormone Receptor family |
|  |  |  |  |  |  |  |  |  |  |  |  |  |  |  |  |  |  |  |  |  |  |  |  |  |  |  |  |  |  |  |  |  |  |  |  |  |  | C34F6.9 |  |
|  |  |  |  |  |  |  |  |  |  |  |  |  |  |  |  |  |  |  |  |  |  |  |  |  |  |  |  |  |  |  |  |  |  |  |  |  |  | *zbp-1* | Zipcode Binding Protein homolog |
|  |  |  |  |  |  |  |  |  |  |  |  |  |  |  |  |  |  |  |  |  |  |  |  |  |  |  |  |  |  |  |  |  |  |  |  |  |  | T05H10.7 |  |
|  |  |  |  |  |  |  |  |  |  |  |  |  |  |  |  |  |  |  |  |  |  |  |  |  |  |  |  |  |  |  |  |  |  |  |  |  |  | *set-6* | SET (trithorax/polycomb) domain containing |
|  |  |  |  |  |  |  |  |  |  |  |  |  |  |  |  |  |  |  |  |  |  |  |  |  |  |  |  |  |  |  |  |  |  |  |  |  |  | *trcs-2* | TRansport of membrane to Cell Surface |
|  |  |  |  |  |  |  |  |  |  |  |  |  |  |  |  |  |  |  |  |  |  |  |  |  |  |  |  |  |  |  |  |  |  |  |  |  |  | C09E7.4 |  |
|  |  |  |  |  |  |  |  |  |  |  |  |  |  |  |  |  |  |  |  |  |  |  |  |  |  |  |  |  |  |  |  |  |  |  |  |  |  | *hda-11* | Histone DeAcetylase |
|  |  |  |  |  |  |  |  |  |  |  |  |  |  |  |  |  |  |  |  |  |  |  |  |  |  |  |  |  |  |  |  |  |  |  |  |  |  | R08D7.5 |  |
|  |  |  |  |  |  |  |  |  |  |  |  |  |  |  |  |  |  |  |  |  |  |  |  |  |  |  |  |  |  |  |  |  |  |  |  |  |  | F13C5.1 |  |
|  |  |  |  |  |  |  |  |  |  |  |  |  |  |  |  |  |  |  |  |  |  |  |  |  |  |  |  |  |  |  |  |  |  |  |  |  |  | B0303.14 |  |
|  |  |  |  |  |  |  |  |  |  |  |  |  |  |  |  |  |  |  |  |  |  |  |  |  |  |  |  |  |  |  |  |  |  |  |  |  |  | D1025.1 |  |
|  |  |  |  |  |  |  |  |  |  |  |  |  |  |  |  |  |  |  |  |  |  |  |  |  |  |  |  |  |  |  |  |  |  |  |  |  |  | R08D7.4 |  |
|  |  |  |  |  |  |  |  |  |  |  |  |  |  |  |  |  |  |  |  |  |  |  |  |  |  |  |  |  |  |  |  |  |  |  |  |  |  | *rabn-5* | RABaptiN (rab effector) |
|  |  |  |  |  |  |  |  |  |  |  |  |  |  |  |  |  |  |  |  |  |  |  |  |  |  |  |  |  |  |  |  |  |  |  |  |  |  | ZC487.1 |  |
|  |  |  |  |  |  |  |  |  |  |  |  |  |  |  |  |  |  |  |  |  |  |  |  |  |  |  |  |  |  |  |  |  |  |  |  |  |  | *nhr-190* | Nuclear Hormone Receptor family |
|  |  |  |  |  |  |  |  |  |  |  |  |  |  |  |  |  |  |  |  |  |  |  |  |  |  |  |  |  |  |  |  |  |  |  |  |  |  | *ceh-74* | C. Elegans Homeobox |
|  |  |  |  |  |  |  |  |  |  |  |  |  |  |  |  |  |  |  |  |  |  |  |  |  |  |  |  |  |  |  |  |  |  |  |  |  |  | C56C10.10 |  |
|  |  |  |  |  |  |  |  |  |  |  |  |  |  |  |  |  |  |  |  |  |  |  |  |  |  |  |  |  |  |  |  |  |  |  |  |  |  | C54C6.6 |  |
|  |  |  |  |  |  |  |  |  |  |  |  |  |  |  |  |  |  |  |  |  |  |  |  |  |  |  |  |  |  |  |  |  |  |  |  |  |  | *magu-3* | MAGUK family |
|  |  |  |  |  |  |  |  |  |  |  |  |  |  |  |  |  |  |  |  |  |  |  |  |  |  |  |  |  |  |  |  |  |  |  |  |  |  | F52B10.3 |  |
|  |  |  |  |  |  |  |  |  |  |  |  |  |  |  |  |  |  |  |  |  |  |  |  |  |  |  |  |  |  |  |  |  |  |  |  |  |  | *nas-36* | Nematode AStacin protease |
|  |  |  |  |  |  |  |  |  |  |  |  |  |  |  |  |  |  |  |  |  |  |  |  |  |  |  |  |  |  |  |  |  |  |  |  |  |  | *uev-3* | Ubiquitin E2 (conjugating enzyme) variant |
|  |  |  |  |  |  |  |  |  |  |  |  |  |  |  |  |  |  |  |  |  |  |  |  |  |  |  |  |  |  |  |  |  |  |  |  |  |  | F56C9.11 |  |
|  |  |  |  |  |  |  |  |  |  |  |  |  |  |  |  |  |  |  |  |  |  |  |  |  |  |  |  |  |  |  |  |  |  |  |  |  |  | *mlh-1* | MLH (MutL Homolog) family |
|  |  |  |  |  |  |  |  |  |  |  |  |  |  |  |  |  |  |  |  |  |  |  |  |  |  |  |  |  |  |  |  |  |  |  |  |  |  | *wrm-1* | Worm aRMadillo |
|  |  |  |  |  |  |  |  |  |  |  |  |  |  |  |  |  |  |  |  |  |  |  |  |  |  |  |  |  |  |  |  |  |  |  |  |  |  | *hid-1* | High temperature-Induced Dauer formation |
|  |  |  |  |  |  |  |  |  |  |  |  |  |  |  |  |  |  |  |  |  |  |  |  |  |  |  |  |  |  |  |  |  |  |  |  |  |  | *hex-4* | HEXosaminidase |
|  |  |  |  |  |  |  |  |  |  |  |  |  |  |  |  |  |  |  |  |  |  |  |  |  |  |  |  |  |  |  |  |  |  |  |  |  |  | *lat-1* | LATrophilin receptor |
|  |  |  |  |  |  |  |  |  |  |  |  |  |  |  |  |  |  |  |  |  |  |  |  |  |  |  |  |  |  |  |  |  |  |  |  |  |  | C41C4.11 |  |
|  |  |  |  |  |  |  |  |  |  |  |  |  |  |  |  |  |  |  |  |  |  |  |  |  |  |  |  |  |  |  |  |  |  |  |  |  |  | *snx-17* | Sorting NeXin |
|  |  |  |  |  |  |  |  |  |  |  |  |  |  |  |  |  |  |  |  |  |  |  |  |  |  |  |  |  |  |  |  |  |  |  |  |  |  | F54E4.2 |  |
|  |  |  |  |  |  |  |  |  |  |  |  |  |  |  |  |  |  |  |  |  |  |  |  |  |  |  |  |  |  |  |  |  |  |  |  |  |  | *ast-1* | Axon STeering defect |
|  |  |  |  |  |  |  |  |  |  |  |  |  |  |  |  |  |  |  |  |  |  |  |  |  |  |  |  |  |  |  |  |  |  |  |  |  |  | R12B2.2 |  |
|  |  |  |  |  |  |  |  |  |  |  |  |  |  |  |  |  |  |  |  |  |  |  |  |  |  |  |  |  |  |  |  |  |  |  |  |  |  | C01A2.1 |  |
|  |  |  |  |  |  |  |  |  |  |  |  |  |  |  |  |  |  |  |  |  |  |  |  |  |  |  |  |  |  |  |  |  |  |  |  |  |  | *trpp-12* | TRansport Protein Particle |
|  |  |  |  |  |  |  |  |  |  |  |  |  |  |  |  |  |  |  |  |  |  |  |  |  |  |  |  |  |  |  |  |  |  |  |  |  |  | *fbxa-13* | F-box A protein |
|  |  |  |  |  |  |  |  |  |  |  |  |  |  |  |  |  |  |  |  |  |  |  |  |  |  |  |  |  |  |  |  |  |  |  |  |  |  | T28H10.1 |  |
|  |  |  |  |  |  |  |  |  |  |  |  |  |  |  |  |  |  |  |  |  |  |  |  |  |  |  |  |  |  |  |  |  |  |  |  |  |  | C34F6.6 |  |
|  |  |  |  |  |  |  |  |  |  |  |  |  |  |  |  |  |  |  |  |  |  |  |  |  |  |  |  |  |  |  |  |  |  |  |  |  |  | *linc-11* | Long Intervening Non-Coding RNA |
|  |  |  |  |  |  |  |  |  |  |  |  |  |  |  |  |  |  |  |  |  |  |  |  |  |  |  |  |  |  |  |  |  |  |  |  |  |  | C05E11.3 |  |
|  |  |  |  |  |  |  |  |  |  |  |  |  |  |  |  |  |  |  |  |  |  |  |  |  |  |  |  |  |  |  |  |  |  |  |  |  |  | *drp-1* | Dynamin-Related Protein |
|  |  |  |  |  |  |  |  |  |  |  |  |  |  |  |  |  |  |  |  |  |  |  |  |  |  |  |  |  |  |  |  |  |  |  |  |  |  | *pmk-2* | P38 Map Kinase family |
|  |  |  |  |  |  |  |  |  |  |  |  |  |  |  |  |  |  |  |  |  |  |  |  |  |  |  |  |  |  |  |  |  |  |  |  |  |  | T05F1.13 |  |
|  |  |  |  |  |  |  |  |  |  |  |  |  |  |  |  |  |  |  |  |  |  |  |  |  |  |  |  |  |  |  |  |  |  |  |  |  |  | *snap-1* | SNAP (Soluble NSF Attachment Protein) homolog |
|  |  |  |  |  |  |  |  |  |  |  |  |  |  |  |  |  |  |  |  |  |  |  |  |  |  |  |  |  |  |  |  |  |  |  |  |  |  | *dpff-1* | DPF (transcription factor) Family |
|  |  |  |  |  |  |  |  |  |  |  |  |  |  |  |  |  |  |  |  |  |  |  |  |  |  |  |  |  |  |  |  |  |  |  |  |  |  | *apm-3* | AdaPtin, Mu/medium chain (clathrin associated complex) |
|  |  |  |  |  |  |  |  |  |  |  |  |  |  |  |  |  |  |  |  |  |  |  |  |  |  |  |  |  |  |  |  |  |  |  |  |  |  | *unc-40* | UNCoordinated |
|  |  |  |  |  |  |  |  |  |  |  |  |  |  |  |  |  |  |  |  |  |  |  |  |  |  |  |  |  |  |  |  |  |  |  |  |  |  | Y54E5A.7 |  |
|  |  |  |  |  |  |  |  |  |  |  |  |  |  |  |  |  |  |  |  |  |  |  |  |  |  |  |  |  |  |  |  |  |  |  |  |  |  | F09F9.4 |  |
|  |  |  |  |  |  |  |  |  |  |  |  |  |  |  |  |  |  |  |  |  |  |  |  |  |  |  |  |  |  |  |  |  |  |  |  |  |  | F12F6.1 |  |
|  |  |  |  |  |  |  |  |  |  |  |  |  |  |  |  |  |  |  |  |  |  |  |  |  |  |  |  |  |  |  |  |  |  |  |  |  |  | *apb-3* |  |
|  |  |  |  |  |  |  |  |  |  |  |  |  |  |  |  |  |  |  |  |  |  |  |  |  |  |  |  |  |  |  |  |  |  |  |  |  |  | D2085.4 |  |
|  |  |  |  |  |  |  |  |  |  |  |  |  |  |  |  |  |  |  |  |  |  |  |  |  |  |  |  |  |  |  |  |  |  |  |  |  |  | *lrk-1* | Leucine-rich repeats, Ras-like domain, Kinase |
|  |  |  |  |  |  |  |  |  |  |  |  |  |  |  |  |  |  |  |  |  |  |  |  |  |  |  |  |  |  |  |  |  |  |  |  |  |  | *clr-1* | CLeaR |
|  |  |  |  |  |  |  |  |  |  |  |  |  |  |  |  |  |  |  |  |  |  |  |  |  |  |  |  |  |  |  |  |  |  |  |  |  |  | *ceh-44* | C. Elegans Homeobox |
|  |  |  |  |  |  |  |  |  |  |  |  |  |  |  |  |  |  |  |  |  |  |  |  |  |  |  |  |  |  |  |  |  |  |  |  |  |  | *oga-1* | O-GlcNAc selective N-Acetyl-beta-D-glucosaminidase (O-GlcNAcase) |
|  |  |  |  |  |  |  |  |  |  |  |  |  |  |  |  |  |  |  |  |  |  |  |  |  |  |  |  |  |  |  |  |  |  |  |  |  |  | C52D10.12 |  |
|  |  |  |  |  |  |  |  |  |  |  |  |  |  |  |  |  |  |  |  |  |  |  |  |  |  |  |  |  |  |  |  |  |  |  |  |  |  | F25E5.1 |  |
|  |  |  |  |  |  |  |  |  |  |  |  |  |  |  |  |  |  |  |  |  |  |  |  |  |  |  |  |  |  |  |  |  |  |  |  |  |  | F52H2.1 |  |
|  |  |  |  |  |  |  |  |  |  |  |  |  |  |  |  |  |  |  |  |  |  |  |  |  |  |  |  |  |  |  |  |  |  |  |  |  |  | *ddl-3* | Daf-16-Dependent Longevity (WT but not daf-16 lifespan increased) |
|  |  |  |  |  |  |  |  |  |  |  |  |  |  |  |  |  |  |  |  |  |  |  |  |  |  |  |  |  |  |  |  |  |  |  |  |  |  | *ears-2* | glutamyl(E) Amino-acyl tRNA Synthetase |
|  |  |  |  |  |  |  |  |  |  |  |  |  |  |  |  |  |  |  |  |  |  |  |  |  |  |  |  |  |  |  |  |  |  |  |  |  |  | *bed-1* | BED-type zinc finger putative transcription factor |
|  |  |  |  |  |  |  |  |  |  |  |  |  |  |  |  |  |  |  |  |  |  |  |  |  |  |  |  |  |  |  |  |  |  |  |  |  |  | *grld-1* | Glutamate Receptor Level Decreased |
|  |  |  |  |  |  |  |  |  |  |  |  |  |  |  |  |  |  |  |  |  |  |  |  |  |  |  |  |  |  |  |  |  |  |  |  |  |  | *mab-20* | Male ABnormal |
|  |  |  |  |  |  |  |  |  |  |  |  |  |  |  |  |  |  |  |  |  |  |  |  |  |  |  |  |  |  |  |  |  |  |  |  |  |  | *unc-101* | UNCoordinated |
|  |  |  |  |  |  |  |  |  |  |  |  |  |  |  |  |  |  |  |  |  |  |  |  |  |  |  |  |  |  |  |  |  |  |  |  |  |  | *mdt-1.2* | MeDiaTor |
|  |  |  |  |  |  |  |  |  |  |  |  |  |  |  |  |  |  |  |  |  |  |  |  |  |  |  |  |  |  |  |  |  |  |  |  |  |  | *sdc-1* | Sex determination and Dosage Compensation defect |
|  |  |  |  |  |  |  |  |  |  |  |  |  |  |  |  |  |  |  |  |  |  |  |  |  |  |  |  |  |  |  |  |  |  |  |  |  |  | ZC334.14 |  |
|  |  |  |  |  |  |  |  |  |  |  |  |  |  |  |  |  |  |  |  |  |  |  |  |  |  |  |  |  |  |  |  |  |  |  |  |  |  | *cam-1* | CAN cell Migration defective |
|  |  |  |  |  |  |  |  |  |  |  |  |  |  |  |  |  |  |  |  |  |  |  |  |  |  |  |  |  |  |  |  |  |  |  |  |  |  | R05D3.2 |  |
|  |  |  |  |  |  |  |  |  |  |  |  |  |  |  |  |  |  |  |  |  |  |  |  |  |  |  |  |  |  |  |  |  |  |  |  |  |  | *cec-6* | C.Elegans Chromodomain protein |
|  |  |  |  |  |  |  |  |  |  |  |  |  |  |  |  |  |  |  |  |  |  |  |  |  |  |  |  |  |  |  |  |  |  |  |  |  |  | *emc-2* | EMC Endoplasmic Membrane protein Complex (yeast EMC) homolog |
|  |  |  |  |  |  |  |  |  |  |  |  |  |  |  |  |  |  |  |  |  |  |  |  |  |  |  |  |  |  |  |  |  |  |  |  |  |  | Y71H2AM.24 |  |
|  |  |  |  |  |  |  |  |  |  |  |  |  |  |  |  |  |  |  |  |  |  |  |  |  |  |  |  |  |  |  |  |  |  |  |  |  |  | Y48G1C.12 |  |
|  |  |  |  |  |  |  |  |  |  |  |  |  |  |  |  |  |  |  |  |  |  |  |  |  |  |  |  |  |  |  |  |  |  |  |  |  |  | F37A4.1 |  |
|  |  |  |  |  |  |  |  |  |  |  |  |  |  |  |  |  |  |  |  |  |  |  |  |  |  |  |  |  |  |  |  |  |  |  |  |  |  | *mett-10* | METhylTransferase homolog |
|  |  |  |  |  |  |  |  |  |  |  |  |  |  |  |  |  |  |  |  |  |  |  |  |  |  |  |  |  |  |  |  |  |  |  |  |  |  | *fdps-1* | Farnesyl DiPhosphate Synthetase |
|  |  |  |  |  |  |  |  |  |  |  |  |  |  |  |  |  |  |  |  |  |  |  |  |  |  |  |  |  |  |  |  |  |  |  |  |  |  | Y59A8B.8 |  |
|  |  |  |  |  |  |  |  |  |  |  |  |  |  |  |  |  |  |  |  |  |  |  |  |  |  |  |  |  |  |  |  |  |  |  |  |  |  | C33D9.13 |  |
|  |  |  |  |  |  |  |  |  |  |  |  |  |  |  |  |  |  |  |  |  |  |  |  |  |  |  |  |  |  |  |  |  |  |  |  |  |  | C48D1.1 |  |
|  |  |  |  |  |  |  |  |  |  |  |  |  |  |  |  |  |  |  |  |  |  |  |  |  |  |  |  |  |  |  |  |  |  |  |  |  |  | ZC477.3 |  |
|  |  |  |  |  |  |  |  |  |  |  |  |  |  |  |  |  |  |  |  |  |  |  |  |  |  |  |  |  |  |  |  |  |  |  |  |  |  | Y106G6H.6 |  |
|  |  |  |  |  |  |  |  |  |  |  |  |  |  |  |  |  |  |  |  |  |  |  |  |  |  |  |  |  |  |  |  |  |  |  |  |  |  | ZK546.5 |  |
|  |  |  |  |  |  |  |  |  |  |  |  |  |  |  |  |  |  |  |  |  |  |  |  |  |  |  |  |  |  |  |  |  |  |  |  |  |  | *fbxa-124* | F-box A protein |
|  |  |  |  |  |  |  |  |  |  |  |  |  |  |  |  |  |  |  |  |  |  |  |  |  |  |  |  |  |  |  |  |  |  |  |  |  |  | F56D5.4 |  |
|  |  |  |  |  |  |  |  |  |  |  |  |  |  |  |  |  |  |  |  |  |  |  |  |  |  |  |  |  |  |  |  |  |  |  |  |  |  | *hst-1* | Heparan SulphoTransferase |
|  |  |  |  |  |  |  |  |  |  |  |  |  |  |  |  |  |  |  |  |  |  |  |  |  |  |  |  |  |  |  |  |  |  |  |  |  |  | Y62E10A.6 |  |
|  |  |  |  |  |  |  |  |  |  |  |  |  |  |  |  |  |  |  |  |  |  |  |  |  |  |  |  |  |  |  |  |  |  |  |  |  |  | *mau-2* | MAternally affected Uncoordination |
|  |  |  |  |  |  |  |  |  |  |  |  |  |  |  |  |  |  |  |  |  |  |  |  |  |  |  |  |  |  |  |  |  |  |  |  |  |  | M01E11.2 |  |
|  |  |  |  |  |  |  |  |  |  |  |  |  |  |  |  |  |  |  |  |  |  |  |  |  |  |  |  |  |  |  |  |  |  |  |  |  |  | F56D1.1 |  |
|  |  |  |  |  |  |  |  |  |  |  |  |  |  |  |  |  |  |  |  |  |  |  |  |  |  |  |  |  |  |  |  |  |  |  |  |  |  | *orc-2* | ORC (Origin Recognition Complex) subunit |
|  |  |  |  |  |  |  |  |  |  |  |  |  |  |  |  |  |  |  |  |  |  |  |  |  |  |  |  |  |  |  |  |  |  |  |  |  |  | *csn-3* | COP-9 SigNalosome subunit |
|  |  |  |  |  |  |  |  |  |  |  |  |  |  |  |  |  |  |  |  |  |  |  |  |  |  |  |  |  |  |  |  |  |  |  |  |  |  | ZK1320.7 |  |
|  |  |  |  |  |  |  |  |  |  |  |  |  |  |  |  |  |  |  |  |  |  |  |  |  |  |  |  |  |  |  |  |  |  |  |  |  |  | F35G12.12 |  |
|  |  |  |  |  |  |  |  |  |  |  |  |  |  |  |  |  |  |  |  |  |  |  |  |  |  |  |  |  |  |  |  |  |  |  |  |  |  | *cash-1* | CKA And Striatin Homolog |
|  |  |  |  |  |  |  |  |  |  |  |  |  |  |  |  |  |  |  |  |  |  |  |  |  |  |  |  |  |  |  |  |  |  |  |  |  |  | *epg-4* | Ectopic P Granules |
|  |  |  |  |  |  |  |  |  |  |  |  |  |  |  |  |  |  |  |  |  |  |  |  |  |  |  |  |  |  |  |  |  |  |  |  |  |  | F54F2.9 |  |
|  |  |  |  |  |  |  |  |  |  |  |  |  |  |  |  |  |  |  |  |  |  |  |  |  |  |  |  |  |  |  |  |  |  |  |  |  |  | *mig-32* | abnormal cell MIGration |
|  |  |  |  |  |  |  |  |  |  |  |  |  |  |  |  |  |  |  |  |  |  |  |  |  |  |  |  |  |  |  |  |  |  |  |  |  |  | *ham-3* | HSN Abnormal Migration |
|  |  |  |  |  |  |  |  |  |  |  |  |  |  |  |  |  |  |  |  |  |  |  |  |  |  |  |  |  |  |  |  |  |  |  |  |  |  | Y24F12A.1 |  |
|  |  |  |  |  |  |  |  |  |  |  |  |  |  |  |  |  |  |  |  |  |  |  |  |  |  |  |  |  |  |  |  |  |  |  |  |  |  | T23G11.4 |  |
|  |  |  |  |  |  |  |  |  |  |  |  |  |  |  |  |  |  |  |  |  |  |  |  |  |  |  |  |  |  |  |  |  |  |  |  |  |  | C28G1.6 |  |
|  |  |  |  |  |  |  |  |  |  |  |  |  |  |  |  |  |  |  |  |  |  |  |  |  |  |  |  |  |  |  |  |  |  |  |  |  |  | *agt-2* | AlkylGuanine DNA alkylTransferase |
|  |  |  |  |  |  |  |  |  |  |  |  |  |  |  |  |  |  |  |  |  |  |  |  |  |  |  |  |  |  |  |  |  |  |  |  |  |  | C37A2.8 |  |
|  |  |  |  |  |  |  |  |  |  |  |  |  |  |  |  |  |  |  |  |  |  |  |  |  |  |  |  |  |  |  |  |  |  |  |  |  |  | ZC262.2 |  |
|  |  |  |  |  |  |  |  |  |  |  |  |  |  |  |  |  |  |  |  |  |  |  |  |  |  |  |  |  |  |  |  |  |  |  |  |  |  | F53A2.3 |  |
|  |  |  |  |  |  |  |  |  |  |  |  |  |  |  |  |  |  |  |  |  |  |  |  |  |  |  |  |  |  |  |  |  |  |  |  |  |  | C09B8.5 |  |
|  |  |  |  |  |  |  |  |  |  |  |  |  |  |  |  |  |  |  |  |  |  |  |  |  |  |  |  |  |  |  |  |  |  |  |  |  |  | *bath-39* | BTB and MATH domain containing |
|  |  |  |  |  |  |  |  |  |  |  |  |  |  |  |  |  |  |  |  |  |  |  |  |  |  |  |  |  |  |  |  |  |  |  |  |  |  | K04F10.3 |  |
|  |  |  |  |  |  |  |  |  |  |  |  |  |  |  |  |  |  |  |  |  |  |  |  |  |  |  |  |  |  |  |  |  |  |  |  |  |  | T01G9.2 |  |
|  |  |  |  |  |  |  |  |  |  |  |  |  |  |  |  |  |  |  |  |  |  |  |  |  |  |  |  |  |  |  |  |  |  |  |  |  |  | F12A10.8 |  |
|  |  |  |  |  |  |  |  |  |  |  |  |  |  |  |  |  |  |  |  |  |  |  |  |  |  |  |  |  |  |  |  |  |  |  |  |  |  | F57C9.4 |  |
|  |  |  |  |  |  |  |  |  |  |  |  |  |  |  |  |  |  |  |  |  |  |  |  |  |  |  |  |  |  |  |  |  |  |  |  |  |  | *eri-9* | Enhanced RNAI (RNA interference) |
|  |  |  |  |  |  |  |  |  |  |  |  |  |  |  |  |  |  |  |  |  |  |  |  |  |  |  |  |  |  |  |  |  |  |  |  |  |  | B0495.2 |  |
|  |  |  |  |  |  |  |  |  |  |  |  |  |  |  |  |  |  |  |  |  |  |  |  |  |  |  |  |  |  |  |  |  |  |  |  |  |  | F44E2.3 |  |
|  |  |  |  |  |  |  |  |  |  |  |  |  |  |  |  |  |  |  |  |  |  |  |  |  |  |  |  |  |  |  |  |  |  |  |  |  |  | *drh-3* | Dicer Related Helicase |
|  |  |  |  |  |  |  |  |  |  |  |  |  |  |  |  |  |  |  |  |  |  |  |  |  |  |  |  |  |  |  |  |  |  |  |  |  |  | C29E4.13 |  |
|  |  |  |  |  |  |  |  |  |  |  |  |  |  |  |  |  |  |  |  |  |  |  |  |  |  |  |  |  |  |  |  |  |  |  |  |  |  | *atl-1* | ATM (ataxia telangectasia mutated)-Like |
|  |  |  |  |  |  |  |  |  |  |  |  |  |  |  |  |  |  |  |  |  |  |  |  |  |  |  |  |  |  |  |  |  |  |  |  |  |  | *rde-1* | RNAi DEfective |
|  |  |  |  |  |  |  |  |  |  |  |  |  |  |  |  |  |  |  |  |  |  |  |  |  |  |  |  |  |  |  |  |  |  |  |  |  |  | T21B10.3 |  |
|  |  |  |  |  |  |  |  |  |  |  |  |  |  |  |  |  |  |  |  |  |  |  |  |  |  |  |  |  |  |  |  |  |  |  |  |  |  | ZK1098.1 |  |
|  |  |  |  |  |  |  |  |  |  |  |  |  |  |  |  |  |  |  |  |  |  |  |  |  |  |  |  |  |  |  |  |  |  |  |  |  |  | *evl-14* | abnormal Eversion of VuLva |
|  |  |  |  |  |  |  |  |  |  |  |  |  |  |  |  |  |  |  |  |  |  |  |  |  |  |  |  |  |  |  |  |  |  |  |  |  |  | *jhdm-1* | JHDM (histone demethylase) homolog |
|  |  |  |  |  |  |  |  |  |  |  |  |  |  |  |  |  |  |  |  |  |  |  |  |  |  |  |  |  |  |  |  |  |  |  |  |  |  | *aap-1* | phosphoinositide kinase AdAPter subunit |
|  |  |  |  |  |  |  |  |  |  |  |  |  |  |  |  |  |  |  |  |  |  |  |  |  |  |  |  |  |  |  |  |  |  |  |  |  |  | F33D11.10 |  |
|  |  |  |  |  |  |  |  |  |  |  |  |  |  |  |  |  |  |  |  |  |  |  |  |  |  |  |  |  |  |  |  |  |  |  |  |  |  | R10E4.1 |  |
|  |  |  |  |  |  |  |  |  |  |  |  |  |  |  |  |  |  |  |  |  |  |  |  |  |  |  |  |  |  |  |  |  |  |  |  |  |  | F25H2.6 |  |
|  |  |  |  |  |  |  |  |  |  |  |  |  |  |  |  |  |  |  |  |  |  |  |  |  |  |  |  |  |  |  |  |  |  |  |  |  |  | F25H2.7 |  |
|  |  |  |  |  |  |  |  |  |  |  |  |  |  |  |  |  |  |  |  |  |  |  |  |  |  |  |  |  |  |  |  |  |  |  |  |  |  | C45B11.8 |  |
|  |  |  |  |  |  |  |  |  |  |  |  |  |  |  |  |  |  |  |  |  |  |  |  |  |  |  |  |  |  |  |  |  |  |  |  |  |  | *sas-4* | Spindle ASsembly abnormal |
|  |  |  |  |  |  |  |  |  |  |  |  |  |  |  |  |  |  |  |  |  |  |  |  |  |  |  |  |  |  |  |  |  |  |  |  |  |  | T23C6.4 |  |
|  |  |  |  |  |  |  |  |  |  |  |  |  |  |  |  |  |  |  |  |  |  |  |  |  |  |  |  |  |  |  |  |  |  |  |  |  |  | ZK1127.6 |  |
|  |  |  |  |  |  |  |  |  |  |  |  |  |  |  |  |  |  |  |  |  |  |  |  |  |  |  |  |  |  |  |  |  |  |  |  |  |  | *eat-3* | EATing: abnormal pharyngeal pumping |
|  |  |  |  |  |  |  |  |  |  |  |  |  |  |  |  |  |  |  |  |  |  |  |  |  |  |  |  |  |  |  |  |  |  |  |  |  |  | *abl-1* | related to oncogene ABL |
|  |  |  |  |  |  |  |  |  |  |  |  |  |  |  |  |  |  |  |  |  |  |  |  |  |  |  |  |  |  |  |  |  |  |  |  |  |  | *rde-10* | RNAi DEfective |
|  |  |  |  |  |  |  |  |  |  |  |  |  |  |  |  |  |  |  |  |  |  |  |  |  |  |  |  |  |  |  |  |  |  |  |  |  |  | ZK809.5 |  |
|  |  |  |  |  |  |  |  |  |  |  |  |  |  |  |  |  |  |  |  |  |  |  |  |  |  |  |  |  |  |  |  |  |  |  |  |  |  | T24B1.1 |  |
|  |  |  |  |  |  |  |  |  |  |  |  |  |  |  |  |  |  |  |  |  |  |  |  |  |  |  |  |  |  |  |  |  |  |  |  |  |  | *cit-1.1* | CyclIn T |
|  |  |  |  |  |  |  |  |  |  |  |  |  |  |  |  |  |  |  |  |  |  |  |  |  |  |  |  |  |  |  |  |  |  |  |  |  |  | *lin-49* | abnormal cell LINeage |
|  |  |  |  |  |  |  |  |  |  |  |  |  |  |  |  |  |  |  |  |  |  |  |  |  |  |  |  |  |  |  |  |  |  |  |  |  |  | *cyn-4* | CYclophyliN |
|  |  |  |  |  |  |  |  |  |  |  |  |  |  |  |  |  |  |  |  |  |  |  |  |  |  |  |  |  |  |  |  |  |  |  |  |  |  | ZK1098.2 |  |
|  |  |  |  |  |  |  |  |  |  |  |  |  |  |  |  |  |  |  |  |  |  |  |  |  |  |  |  |  |  |  |  |  |  |  |  |  |  | B0361.2 |  |
|  |  |  |  |  |  |  |  |  |  |  |  |  |  |  |  |  |  |  |  |  |  |  |  |  |  |  |  |  |  |  |  |  |  |  |  |  |  | *cel-1* | mRNA Capping Enzyme Like |
|  |  |  |  |  |  |  |  |  |  |  |  |  |  |  |  |  |  |  |  |  |  |  |  |  |  |  |  |  |  |  |  |  |  |  |  |  |  | C13F10.6 |  |
|  |  |  |  |  |  |  |  |  |  |  |  |  |  |  |  |  |  |  |  |  |  |  |  |  |  |  |  |  |  |  |  |  |  |  |  |  |  | *vps-51* | related to yeast Vacuolar Protein Sorting factor |
|  |  |  |  |  |  |  |  |  |  |  |  |  |  |  |  |  |  |  |  |  |  |  |  |  |  |  |  |  |  |  |  |  |  |  |  |  |  | *taf-5* | TAF (TBP-associated transcription factor) family |
|  |  |  |  |  |  |  |  |  |  |  |  |  |  |  |  |  |  |  |  |  |  |  |  |  |  |  |  |  |  |  |  |  |  |  |  |  |  | T05A12.4 |  |
|  |  |  |  |  |  |  |  |  |  |  |  |  |  |  |  |  |  |  |  |  |  |  |  |  |  |  |  |  |  |  |  |  |  |  |  |  |  | *spr-1* | Suppressor of PResenilin defect |
|  |  |  |  |  |  |  |  |  |  |  |  |  |  |  |  |  |  |  |  |  |  |  |  |  |  |  |  |  |  |  |  |  |  |  |  |  |  | C33G3.6 |  |
|  |  |  |  |  |  |  |  |  |  |  |  |  |  |  |  |  |  |  |  |  |  |  |  |  |  |  |  |  |  |  |  |  |  |  |  |  |  | *sac-1* | SAC1 PIP phosphatase (yeast Suppressor of ACtin) homolog |
|  |  |  |  |  |  |  |  |  |  |  |  |  |  |  |  |  |  |  |  |  |  |  |  |  |  |  |  |  |  |  |  |  |  |  |  |  |  | *tre-1* | TREhalase |
|  |  |  |  |  |  |  |  |  |  |  |  |  |  |  |  |  |  |  |  |  |  |  |  |  |  |  |  |  |  |  |  |  |  |  |  |  |  | C53D6.6 |  |
|  |  |  |  |  |  |  |  |  |  |  |  |  |  |  |  |  |  |  |  |  |  |  |  |  |  |  |  |  |  |  |  |  |  |  |  |  |  | T04F8.2 |  |
|  |  |  |  |  |  |  |  |  |  |  |  |  |  |  |  |  |  |  |  |  |  |  |  |  |  |  |  |  |  |  |  |  |  |  |  |  |  | ZC434.7 |  |
|  |  |  |  |  |  |  |  |  |  |  |  |  |  |  |  |  |  |  |  |  |  |  |  |  |  |  |  |  |  |  |  |  |  |  |  |  |  | Y5F2A.4 |  |
|  |  |  |  |  |  |  |  |  |  |  |  |  |  |  |  |  |  |  |  |  |  |  |  |  |  |  |  |  |  |  |  |  |  |  |  |  |  | F20C5.5 |  |
|  |  |  |  |  |  |  |  |  |  |  |  |  |  |  |  |  |  |  |  |  |  |  |  |  |  |  |  |  |  |  |  |  |  |  |  |  |  | F08F8.10 |  |
|  |  |  |  |  |  |  |  |  |  |  |  |  |  |  |  |  |  |  |  |  |  |  |  |  |  |  |  |  |  |  |  |  |  |  |  |  |  | F20C5.6 |  |
|  |  |  |  |  |  |  |  |  |  |  |  |  |  |  |  |  |  |  |  |  |  |  |  |  |  |  |  |  |  |  |  |  |  |  |  |  |  | F59B2.9 |  |
|  |  |  |  |  |  |  |  |  |  |  |  |  |  |  |  |  |  |  |  |  |  |  |  |  |  |  |  |  |  |  |  |  |  |  |  |  |  | F59B2.8 |  |
|  |  |  |  |  |  |  |  |  |  |  |  |  |  |  |  |  |  |  |  |  |  |  |  |  |  |  |  |  |  |  |  |  |  |  |  |  |  | T09A5.4 |  |
|  |  |  |  |  |  |  |  |  |  |  |  |  |  |  |  |  |  |  |  |  |  |  |  |  |  |  |  |  |  |  |  |  |  |  |  |  |  | C44E4.5 |  |
|  |  |  |  |  |  |  |  |  |  |  |  |  |  |  |  |  |  |  |  |  |  |  |  |  |  |  |  |  |  |  |  |  |  |  |  |  |  | *rgs-5* | Regulator of G protein Signaling |
|  |  |  |  |  |  |  |  |  |  |  |  |  |  |  |  |  |  |  |  |  |  |  |  |  |  |  |  |  |  |  |  |  |  |  |  |  |  | *hmgr-1* | HMG-CoA Reductase |
|  |  |  |  |  |  |  |  |  |  |  |  |  |  |  |  |  |  |  |  |  |  |  |  |  |  |  |  |  |  |  |  |  |  |  |  |  |  | *asna-1* | ArSeNite-translocating ATPase family |
|  |  |  |  |  |  |  |  |  |  |  |  |  |  |  |  |  |  |  |  |  |  |  |  |  |  |  |  |  |  |  |  |  |  |  |  |  |  | *mms-19* | yeast MMS related |
|  |  |  |  |  |  |  |  |  |  |  |  |  |  |  |  |  |  |  |  |  |  |  |  |  |  |  |  |  |  |  |  |  |  |  |  |  |  | *mut-15* | MUTator |
|  |  |  |  |  |  |  |  |  |  |  |  |  |  |  |  |  |  |  |  |  |  |  |  |  |  |  |  |  |  |  |  |  |  |  |  |  |  | C44E4.8 |  |
|  |  |  |  |  |  |  |  |  |  |  |  |  |  |  |  |  |  |  |  |  |  |  |  |  |  |  |  |  |  |  |  |  |  |  |  |  |  | F58D12.3 |  |
|  |  |  |  |  |  |  |  |  |  |  |  |  |  |  |  |  |  |  |  |  |  |  |  |  |  |  |  |  |  |  |  |  |  |  |  |  |  | *bpl-1* | Biotin Protein Ligase |
|  |  |  |  |  |  |  |  |  |  |  |  |  |  |  |  |  |  |  |  |  |  |  |  |  |  |  |  |  |  |  |  |  |  |  |  |  |  | *sulp-8* | SULfate Permease family |
|  |  |  |  |  |  |  |  |  |  |  |  |  |  |  |  |  |  |  |  |  |  |  |  |  |  |  |  |  |  |  |  |  |  |  |  |  |  | F43E2.6 |  |
|  |  |  |  |  |  |  |  |  |  |  |  |  |  |  |  |  |  |  |  |  |  |  |  |  |  |  |  |  |  |  |  |  |  |  |  |  |  | T04A8.7 |  |
|  |  |  |  |  |  |  |  |  |  |  |  |  |  |  |  |  |  |  |  |  |  |  |  |  |  |  |  |  |  |  |  |  |  |  |  |  |  | *idhg-1* | Isocitrate DeHydrogenase Gamma |
|  |  |  |  |  |  |  |  |  |  |  |  |  |  |  |  |  |  |  |  |  |  |  |  |  |  |  |  |  |  |  |  |  |  |  |  |  |  | *rsbp-1* | R-Seven Binding Protein (R7BP) homolog |
|  |  |  |  |  |  |  |  |  |  |  |  |  |  |  |  |  |  |  |  |  |  |  |  |  |  |  |  |  |  |  |  |  |  |  |  |  |  | *jamp-1* | JAMP (JNK1-Associated Membrane Protein) homolog |
|  |  |  |  |  |  |  |  |  |  |  |  |  |  |  |  |  |  |  |  |  |  |  |  |  |  |  |  |  |  |  |  |  |  |  |  |  |  | *micu-1* | MItochondrial Calcium Uptake protein |
|  |  |  |  |  |  |  |  |  |  |  |  |  |  |  |  |  |  |  |  |  |  |  |  |  |  |  |  |  |  |  |  |  |  |  |  |  |  | *ekl-5* | Enhancer of Ksr-1 Lethality |
|  |  |  |  |  |  |  |  |  |  |  |  |  |  |  |  |  |  |  |  |  |  |  |  |  |  |  |  |  |  |  |  |  |  |  |  |  |  | *mlcd-1* | MaLonyl CoA Decarboxylase |
|  |  |  |  |  |  |  |  |  |  |  |  |  |  |  |  |  |  |  |  |  |  |  |  |  |  |  |  |  |  |  |  |  |  |  |  |  |  | *dyb-1* | DYstroBrevin homolog |
|  |  |  |  |  |  |  |  |  |  |  |  |  |  |  |  |  |  |  |  |  |  |  |  |  |  |  |  |  |  |  |  |  |  |  |  |  |  | *hrdl-1* | HRD-Like (E3 ubiquitin ligase related) |
|  |  |  |  |  |  |  |  |  |  |  |  |  |  |  |  |  |  |  |  |  |  |  |  |  |  |  |  |  |  |  |  |  |  |  |  |  |  | *cap-2* | CAP-z protein |
|  |  |  |  |  |  |  |  |  |  |  |  |  |  |  |  |  |  |  |  |  |  |  |  |  |  |  |  |  |  |  |  |  |  |  |  |  |  | *xbx-6* | X-BoX promoter element regulated |
|  |  |  |  |  |  |  |  |  |  |  |  |  |  |  |  |  |  |  |  |  |  |  |  |  |  |  |  |  |  |  |  |  |  |  |  |  |  | T28F4.1 |  |
|  |  |  |  |  |  |  |  |  |  |  |  |  |  |  |  |  |  |  |  |  |  |  |  |  |  |  |  |  |  |  |  |  |  |  |  |  |  | *rgs-1* | Regulator of G protein Signaling |
|  |  |  |  |  |  |  |  |  |  |  |  |  |  |  |  |  |  |  |  |  |  |  |  |  |  |  |  |  |  |  |  |  |  |  |  |  |  | *nck-1* | NCK (Non-Catalytic region of tyrosine Kinase) adaptor protein family |
|  |  |  |  |  |  |  |  |  |  |  |  |  |  |  |  |  |  |  |  |  |  |  |  |  |  |  |  |  |  |  |  |  |  |  |  |  |  | C39F7.5 |  |
|  |  |  |  |  |  |  |  |  |  |  |  |  |  |  |  |  |  |  |  |  |  |  |  |  |  |  |  |  |  |  |  |  |  |  |  |  |  | C25A11.2 |  |
|  |  |  |  |  |  |  |  |  |  |  |  |  |  |  |  |  |  |  |  |  |  |  |  |  |  |  |  |  |  |  |  |  |  |  |  |  |  | *usp-46* | Ubiquitin Specific Protease |
|  |  |  |  |  |  |  |  |  |  |  |  |  |  |  |  |  |  |  |  |  |  |  |  |  |  |  |  |  |  |  |  |  |  |  |  |  |  | *acl-14* | ACyLtransferase-like |
|  |  |  |  |  |  |  |  |  |  |  |  |  |  |  |  |  |  |  |  |  |  |  |  |  |  |  |  |  |  |  |  |  |  |  |  |  |  | C06A6.2 |  |
|  |  |  |  |  |  |  |  |  |  |  |  |  |  |  |  |  |  |  |  |  |  |  |  |  |  |  |  |  |  |  |  |  |  |  |  |  |  | *mel-26* | Maternal Effect Lethal |
|  |  |  |  |  |  |  |  |  |  |  |  |  |  |  |  |  |  |  |  |  |  |  |  |  |  |  |  |  |  |  |  |  |  |  |  |  |  | *ima-1* | IMportin Alpha family |
|  |  |  |  |  |  |  |  |  |  |  |  |  |  |  |  |  |  |  |  |  |  |  |  |  |  |  |  |  |  |  |  |  |  |  |  |  |  | *linc-102* | Long Intervening Non-Coding RNA |
|  |  |  |  |  |  |  |  |  |  |  |  |  |  |  |  |  |  |  |  |  |  |  |  |  |  |  |  |  |  |  |  |  |  |  |  |  |  | *cls-3* | CLASP family of microtubule-binding proteins |
|  |  |  |  |  |  |  |  |  |  |  |  |  |  |  |  |  |  |  |  |  |  |  |  |  |  |  |  |  |  |  |  |  |  |  |  |  |  | Y57G11C.21 |  |
|  |  |  |  |  |  |  |  |  |  |  |  |  |  |  |  |  |  |  |  |  |  |  |  |  |  |  |  |  |  |  |  |  |  |  |  |  |  | F23C8.1 |  |
|  |  |  |  |  |  |  |  |  |  |  |  |  |  |  |  |  |  |  |  |  |  |  |  |  |  |  |  |  |  |  |  |  |  |  |  |  |  | *unc-44* | UNCoordinated |
|  |  |  |  |  |  |  |  |  |  |  |  |  |  |  |  |  |  |  |  |  |  |  |  |  |  |  |  |  |  |  |  |  |  |  |  |  |  | *rde-11* | RNAi DEfective |
|  |  |  |  |  |  |  |  |  |  |  |  |  |  |  |  |  |  |  |  |  |  |  |  |  |  |  |  |  |  |  |  |  |  |  |  |  |  | Y37F4.6 |  |
|  |  |  |  |  |  |  |  |  |  |  |  |  |  |  |  |  |  |  |  |  |  |  |  |  |  |  |  |  |  |  |  |  |  |  |  |  |  | *flh-2* | FLYWCH zinc finger transcription factor homolog |
|  |  |  |  |  |  |  |  |  |  |  |  |  |  |  |  |  |  |  |  |  |  |  |  |  |  |  |  |  |  |  |  |  |  |  |  |  |  | Y102E9.2 |  |
|  |  |  |  |  |  |  |  |  |  |  |  |  |  |  |  |  |  |  |  |  |  |  |  |  |  |  |  |  |  |  |  |  |  |  |  |  |  | W01A11.7 |  |
|  |  |  |  |  |  |  |  |  |  |  |  |  |  |  |  |  |  |  |  |  |  |  |  |  |  |  |  |  |  |  |  |  |  |  |  |  |  | K03H1.13 |  |
|  |  |  |  |  |  |  |  |  |  |  |  |  |  |  |  |  |  |  |  |  |  |  |  |  |  |  |  |  |  |  |  |  |  |  |  |  |  | *rev-1* | REV1 (translesion DNA polymerase) homolog |
|  |  |  |  |  |  |  |  |  |  |  |  |  |  |  |  |  |  |  |  |  |  |  |  |  |  |  |  |  |  |  |  |  |  |  |  |  |  | F39H12.1 |  |
|  |  |  |  |  |  |  |  |  |  |  |  |  |  |  |  |  |  |  |  |  |  |  |  |  |  |  |  |  |  |  |  |  |  |  |  |  |  | R10D12.8 |  |
|  |  |  |  |  |  |  |  |  |  |  |  |  |  |  |  |  |  |  |  |  |  |  |  |  |  |  |  |  |  |  |  |  |  |  |  |  |  | ZK1098.3 |  |
|  |  |  |  |  |  |  |  |  |  |  |  |  |  |  |  |  |  |  |  |  |  |  |  |  |  |  |  |  |  |  |  |  |  |  |  |  |  | *chup-1* | CHolesterol UPtake associated |
|  |  |  |  |  |  |  |  |  |  |  |  |  |  |  |  |  |  |  |  |  |  |  |  |  |  |  |  |  |  |  |  |  |  |  |  |  |  | *nmtn-1* | NeMiTiN (neuronal enriched MAP interacting protein) homolog |
|  |  |  |  |  |  |  |  |  |  |  |  |  |  |  |  |  |  |  |  |  |  |  |  |  |  |  |  |  |  |  |  |  |  |  |  |  |  | C03A3.1 |  |
|  |  |  |  |  |  |  |  |  |  |  |  |  |  |  |  |  |  |  |  |  |  |  |  |  |  |  |  |  |  |  |  |  |  |  |  |  |  | *xpa-1* | human XPA (Xeroderma pigmentosum comp grp A) related |
|  |  |  |  |  |  |  |  |  |  |  |  |  |  |  |  |  |  |  |  |  |  |  |  |  |  |  |  |  |  |  |  |  |  |  |  |  |  | ZK858.5 |  |
|  |  |  |  |  |  |  |  |  |  |  |  |  |  |  |  |  |  |  |  |  |  |  |  |  |  |  |  |  |  |  |  |  |  |  |  |  |  | *syg-1* | SYnaptoGenesis abnormal |
|  |  |  |  |  |  |  |  |  |  |  |  |  |  |  |  |  |  |  |  |  |  |  |  |  |  |  |  |  |  |  |  |  |  |  |  |  |  | ZK180.3 |  |
|  |  |  |  |  |  |  |  |  |  |  |  |  |  |  |  |  |  |  |  |  |  |  |  |  |  |  |  |  |  |  |  |  |  |  |  |  |  | *mau-8* | MAternally affected Uncoordination |
|  |  |  |  |  |  |  |  |  |  |  |  |  |  |  |  |  |  |  |  |  |  |  |  |  |  |  |  |  |  |  |  |  |  |  |  |  |  | T05B11.7 |  |
|  |  |  |  |  |  |  |  |  |  |  |  |  |  |  |  |  |  |  |  |  |  |  |  |  |  |  |  |  |  |  |  |  |  |  |  |  |  | AH9.3 |  |
|  |  |  |  |  |  |  |  |  |  |  |  |  |  |  |  |  |  |  |  |  |  |  |  |  |  |  |  |  |  |  |  |  |  |  |  |  |  | *lrp-2* | Low-density lipoprotein RecePtor related |
|  |  |  |  |  |  |  |  |  |  |  |  |  |  |  |  |  |  |  |  |  |  |  |  |  |  |  |  |  |  |  |  |  |  |  |  |  |  | *hst-6* | Heparan SulphoTransferase |
|  |  |  |  |  |  |  |  |  |  |  |  |  |  |  |  |  |  |  |  |  |  |  |  |  |  |  |  |  |  |  |  |  |  |  |  |  |  | *ceh-48* | C. Elegans Homeobox |
|  |  |  |  |  |  |  |  |  |  |  |  |  |  |  |  |  |  |  |  |  |  |  |  |  |  |  |  |  |  |  |  |  |  |  |  |  |  | *anoh-2* | ANOctamin (calcium-activated chloride channel) Homolog |
|  |  |  |  |  |  |  |  |  |  |  |  |  |  |  |  |  |  |  |  |  |  |  |  |  |  |  |  |  |  |  |  |  |  |  |  |  |  | ZK287.1 |  |
|  |  |  |  |  |  |  |  |  |  |  |  |  |  |  |  |  |  |  |  |  |  |  |  |  |  |  |  |  |  |  |  |  |  |  |  |  |  | K10G6.4 |  |
|  |  |  |  |  |  |  |  |  |  |  |  |  |  |  |  |  |  |  |  |  |  |  |  |  |  |  |  |  |  |  |  |  |  |  |  |  |  | *frm-4* | FERM domain (protein4.1-ezrin-radixin-moesin) family |
|  |  |  |  |  |  |  |  |  |  |  |  |  |  |  |  |  |  |  |  |  |  |  |  |  |  |  |  |  |  |  |  |  |  |  |  |  |  | *syd-9* | SYnapse Defective |
|  |  |  |  |  |  |  |  |  |  |  |  |  |  |  |  |  |  |  |  |  |  |  |  |  |  |  |  |  |  |  |  |  |  |  |  |  |  | *sru-47* | Serpentine Receptor, class U |
|  |  |  |  |  |  |  |  |  |  |  |  |  |  |  |  |  |  |  |  |  |  |  |  |  |  |  |  |  |  |  |  |  |  |  |  |  |  | *sru-19* | Serpentine Receptor, class U |
|  |  |  |  |  |  |  |  |  |  |  |  |  |  |  |  |  |  |  |  |  |  |  |  |  |  |  |  |  |  |  |  |  |  |  |  |  |  | W09G3.7 |  |
|  |  |  |  |  |  |  |  |  |  |  |  |  |  |  |  |  |  |  |  |  |  |  |  |  |  |  |  |  |  |  |  |  |  |  |  |  |  | T16H12.3 |  |
|  |  |  |  |  |  |  |  |  |  |  |  |  |  |  |  |  |  |  |  |  |  |  |  |  |  |  |  |  |  |  |  |  |  |  |  |  |  | *fecl-1* | FErroChelatase-Like |
|  |  |  |  |  |  |  |  |  |  |  |  |  |  |  |  |  |  |  |  |  |  |  |  |  |  |  |  |  |  |  |  |  |  |  |  |  |  | T09B4.9 |  |
|  |  |  |  |  |  |  |  |  |  |  |  |  |  |  |  |  |  |  |  |  |  |  |  |  |  |  |  |  |  |  |  |  |  |  |  |  |  | *gspd-1* | Glucose Six (6) Phosphate Dehydrogenase |

### Phenotypes enriched

|  |  |  |  |
| --- | --- | --- | --- |
| **Group name** | **Number in cluster** | **Enrichment** | **FDR corrected p** |
| cell morphology variant | 18 | 3.56 | 0.00529 |
| transgene subcellular localization variant | 7 | 8.20 | 0.02390 |
| axon morphology variant | 8 | 6.38 | 0.03740 |
| neurite morphology variant | 8 | 6.29 | 0.04060 |

### Anatomy terms enriched

|  |  |  |  |
| --- | --- | --- | --- |
| **Group name** | **Number in cluster** | **Enrichment** | **FDR corrected p** |
| nerve ring | 29 | 2.76 | 0.00197 |
| tail | 48 | 1.82 | 0.04830 |

### GO terms enriched

|  |  |  |
| --- | --- | --- |
| **GO term** | **Number of genes** | **FDR-corrected p-value** |
| axonogenesis | 10 | 0.0023 |
| neuron projection guidance | 9 | 0.0027 |
| cell projection morphogenesis | 11 | 0.0059 |
| cellular localization | 20 | 0.0190 |
| taxis | 10 | 0.0200 |
| cell morphogenesis involved in differentiation | 7 | 0.0420 |
| cellular component morphogenesis | 15 | 0.0440 |
| Golgi apparatus | 9 | 0.0480 |
| positive regulation of locomotion | 10 | 0.0480 |

### Expression clusters enriched

|  |  |  |  |
| --- | --- | --- | --- |
| **Group name** | **Number in cluster** | **Enrichment** | **FDR corrected p** |
| Genes predicted to be upregulated more than 2.0 fold in (AFD+AWB) datasets as compared to unsorted whole embryonic cells dataset. | 57 | 3.88 | 6.19e-16 |
| Genes expressed in embryonic motor neurons (identified by unc-4::GFP expressing cells). | 183 | 1.66 | 1.35e-15 |
| Genes significantly enriched (> 2x, FDR < 5%) in a particular cell-type versus a reference sample of all cells at the same stage. WBPaper00037950:all-neurons\_embryo\_enriched | 38 | 4.65 | 2.46e-12 |
| Genes significantly enriched (> 2x, FDR < 5%) in a particular cell-type versus a reference sample of all cells at the same stage. WBPaper00037950:AVA-neuron\_embryo\_enriched | 40 | 4.11 | 2.31e-11 |
| TGF- Dauer pathway adult transcriptional targets. Results obtained by comparing the microarray results of the dauer-constitutive mutants daf-7(e1372), daf-7(m62), and daf-1(m40) with dauer-defective mutants daf-3(mgDf90), daf-5(e1386), and daf-7(e1372);daf-3(mgDf90) double mutants at the permissive temperature, 20C, on the first day of adulthood. WBPaper00031040:TGF-beta\_adult\_downregulated | 135 | 1.77 | 5.08e-11 |
| Maternal class (M): genes that are called present in at least one of the three PC6 replicates. | 166 | 1.57 | 2.24e-10 |
| Caenorhabditis elegans Genes with expression levels changed significantly after treatment of Xenorhabdus nematophila. | 187 | 1.48 | 3.01e-10 |
| Genes that showed expression levels higher than the corresponding reference sample (embryonic 0hr reference). WBPaper00037950:BAG-neuron\_expressed | 139 | 1.66 | 1.99e-09 |
| FBF-associated probe sets (FDR <2.25%) | 120 | 1.75 | 6.70e-09 |
| WT-Pico Pan-neural Enriched Genes, with genes found multiple times in a single dataset removed (without dups). | 68 | 2.33 | 1.19e-08 |
| Genes down-regulated after 300 um Tannic acid treatment. Fold change < 0.8. | 66 | 2.36 | 1.69e-08 |
| Maternal-embryonic class (ME): genes that are in the intersection of the maternal and embryonic classes. | 92 | 1.91 | 7.06e-08 |
| Genes that showed higher expression in N2 than in DR1350. | 61 | 2.26 | 5.35e-07 |
| Genes with expression enriched in PVD and OLL neurons. Data sets were normalized by RMA and transcripts showing relative PVD enrichment (>= 1.5X) vs. the reference sample were identified by SAM analysis (False Discovery Rate, FDR < 1%). | 78 | 1.97 | 8.22e-07 |
| Larval Pan-neural Enriched Genes. | 62 | 2.21 | 8.89e-07 |
| Genes expressed in N2. | 209 | 1.30 | 1.18e-06 |
| hermaphrodite sex-enriched | 22 | 4.45 | 2.91e-06 |
| Genes that showed expression levels higher than the corresponding reference sample (embryonic 24hr reference). WBPaper00037950:AVA-neuron\_expressed | 128 | 1.54 | 5.24e-06 |
| Embryonic Pan-neural Enriched Genes. | 61 | 2.07 | 1.17e-05 |
| Genes significantly enriched (> 2x, FDR < 5%) in a particular cell-type versus a reference sample of all cells at the same stage. WBPaper00037950:A-class-motor-neurons\_larva\_enriched | 35 | 2.80 | 1.83e-05 |
| miRNA targets that are significantly enriched at L1 larva stage. To generate a global view of the dynamics of miRNA-mediated regulation of gene expression during C. elegans development, authors analyzed the mRNAs in the AIN-2-GFP IP results from five developmental stages. The magnitude of the combined interaction of miRNAs with a given target mRNA was assessed by measuring the fold enrichment of that mRNA in AIN-2 IP samples, relative to the abundance of the mRNA in the corresponding total lysate. Because this enrichment in the IP sample versus total lysate directly reflects the miRISC-associated fraction of a given mRNA, high enrichment indicates the likelihood of strong miRNA-mediated regulation of the mRNA, whereas low or negative enrichment indicates the likelihood of weak or absent miRNA regulation of the mRNA. It is also possible that poor enrichment could reflect interactions that occur only in a rare subset of cells at any given stage of development. Transcripts that were significantly enriched (0 | 44 | 2.34 | 4.93e-05 |
| Germline-enriched and sex-biased expression profile cluster E. | 48 | 2.22 | 6.10e-05 |
| Embryonic class (E): genes that significantly increase in abundance at some point during embryogenesis. | 103 | 1.59 | 7.13e-05 |
| Expression Pattern Group F, enriched for genes involved in embryonic development. These patterns have in common that they all have genes of which the expression goes up after the juvenile stage. The expression of the genes in these patterns remains high or even goes up after reproduction. | 72 | 1.76 | 2.88e-04 |
| Larval A-class motor neuron enriched genes. | 23 | 3.13 | 5.29e-04 |
| Developmentally modulated gene cluster. cgc4386\_cluster\_6\_4 | 16 | 4.15 | 6.30e-04 |
| Caenorhabditis elegans Genes with expression levels changed significantly after treatment of Bacillus thurigiensis DB27. | 123 | 1.41 | 1.29e-03 |
| Expression Pattern Group B, enriched for genes involved in embryonic development. These patterns have in common that they all have genes of which the expression goes up after the juvenile stage. The expression of the genes in these patterns remains high or even goes up after reproduction. | 50 | 1.94 | 1.31e-03 |
| Maternal-embryonic transient class (MET): genes that are in the intersection of the maternal and embryonic transient classes. | 36 | 2.21 | 2.08e-03 |
| miRNA targets that are significantly enriched at L3 larva stage. To generate a global view of the dynamics of miRNA-mediated regulation of gene expression during C. elegans development, authors analyzed the mRNAs in the AIN-2-GFP IP results from five developmental stages. The magnitude of the combined interaction of miRNAs with a given target mRNA was assessed by measuring the fold enrichment of that mRNA in AIN-2 IP samples, relative to the abundance of the mRNA in the corresponding total lysate. Because this enrichment in the IP sample versus total lysate directly reflects the miRISC-associated fraction of a given mRNA, high enrichment indicates the likelihood of strong miRNA-mediated regulation of the mRNA, whereas low or negative enrichment indicates the likelihood of weak or absent miRNA regulation of the mRNA. It is also possible that poor enrichment could reflect interactions that occur only in a rare subset of cells at any given stage of development. Transcripts that were significantly enriched (0 | 35 | 2.24 | 2.19e-03 |
| Gene significantly down-regulated by treatment with 2.0mM of HuminFeed until young adult stage (3 days), with a minimum fold change in gene expression of 0.8. | 59 | 1.76 | 2.73e-03 |
| RNP-8-associated transcripts, based on microarray experiments. | 35 | 2.21 | 2.77e-03 |
| Genes significantly enriched (> 2x, FDR < 5%) in a particular cell-type versus a reference sample of all cells at the same stage. WBPaper00037950:GABAergic-motor-neurons\_embryo\_enriched | 20 | 3.05 | 3.18e-03 |
| Genes down regulated in alg-1(gk214) comparing to in N2. | 20 | 2.90 | 6.24e-03 |
| Maternal degradation class (MD): genes that are the subset of maternal genes that decrease without first increasing in abundance. | 55 | 1.73 | 8.40e-03 |
| Expressed transcripts enriched in embryonic motor neurons (identified by unc-4::GFP expressing cells). | 36 | 2.04 | 9.49e-03 |
| Embryonic A-class motor neuron enriched genes. | 36 | 2.04 | 9.49e-03 |
| Differentially expressed genes during worm lifespan. Medoid 3 Fig.4. | 13 | 3.85 | 9.86e-03 |
| Genes that showed expression levels higher than the corresponding reference sample (embryonic 24hr reference). WBPaper00037950:GABAergic-motor-neurons\_expressed | 174 | 1.23 | 1.51e-02 |
| Genes upregulated in hcf-1(-), upregulated in sir-2.1(O/E) and no change in daf-2(-). | 7 | 6.31 | 2.70e-02 |
| Significantly downregulated genes from cyc-1(RNAi) microarrays using SAM algorithm with an FDR < 0.1 from adult-only chips. | 87 | 1.43 | 3.27e-02 |
| Genes that are dosage compensated. | 18 | 2.65 | 3.91e-02 |
| Germline-enriched and sex-biased expression profile cluster F. | 27 | 2.10 | 4.86e-02 |
| mixed oogenesis/somatic | 25 | 2.18 | 4.90e-02 |

### Motifs enriched

|  |  |  |  |  |  |
| --- | --- | --- | --- | --- | --- |
| **Motif** | **Logo** | **Possible orthologs** | **Number of motifs in cluster** | **Enrichment** | **FDR corrected p** |
| pTH8982 |  | ceh-48 (0.96) | 77 | 2.64 | 2.5e-12 |
| ONECUT1\_2 |  | ceh-48 (0.96) dsc-1 | 71 | 2.61 | 6.5e-11 |
| Hbn\_Cell\_FBgn0008636 |  | ceh-10 (0.6) lim-7 alr-1 ceh-53 pha-2 ceh-31 ceh-14 ceh-23 ceh-30 lim-6 lin-39 egl-5 cog-1 ceh-45 ceh-18 ceh-16 ceh-2 ceh-43 C07E3.6 T13C5.4 | 177 | 1.44 | 8.6e-08 |
| pTH8896 |  | daf-16 (0.67) fkh-8 (0.57) fkh-10 (0.54) let-381 pha-4 fkh-7 lin-31 | 212 | 1.32 | 1.8e-07 |
| HepG2\_FOXA1\_HudsonAlpha |  | let-381 lin-31 | 180 | 1.41 | 2.7e-07 |
| pTH9279 |  | Y116A8C.22 | 223 | 1.28 | 2.9e-07 |
| pTH9380 |  | mel-28 | 226 | 1.27 | 4.4e-07 |
| pTH10797 |  | K11D2.4 lin-29 | 230 | 1.25 | 1.0e-06 |
| pTH3796 |  | fkh-10 (0.54) let-381 lin-31 C34D1.1 | 190 | 1.35 | 1.2e-06 |
| pTH9242 |  | mel-28 | 223 | 1.26 | 1.5e-06 |
| sqz\_SANGER\_5\_FBgn0010768 |  | mel-28 lin-29 fkh-7 | 222 | 1.27 | 1.5e-06 |
| V$TBP\_01 |  | tbp-1 | 194 | 1.33 | 2.4e-06 |
| MA0537.1 |  | blmp-1 | 223 | 1.26 | 2.5e-06 |
| pTH10633 |  | R07H5.10 C48E7.11 | 188 | 1.35 | 2.5e-06 |
| Alx1\_2 |  | alr-1 ceh-14 cfi-1 ZC204.2 | 147 | 1.47 | 4.4e-06 |
| pTH6143 |  | lin-39 php-3 pal-1 | 197 | 1.31 | 5.3e-06 |
| pTH9260 |  | mel-28 | 216 | 1.26 | 5.3e-06 |
| pTH9177 |  | F10B5.3 hsf-1 | 202 | 1.30 | 6.2e-06 |
| pTH9180 |  | let-381 mel-28 mef-2 Y61A9LA.9 Y116A8C.22 | 225 | 1.24 | 6.2e-06 |
| ARI3A\_do |  | gei-3 cfi-1 | 225 | 1.24 | 6.3e-06 |
| V$POU3F2\_01 |  | dmd-3 ceh-18 | 204 | 1.29 | 7.5e-06 |
| MA0481.1 |  | daf-16 (0.67) fkh-8 (0.57) fkh-10 (0.54) let-381 fkh-7 lin-31 | 212 | 1.26 | 1.5e-05 |
| pTH9254 |  | mel-28 | 217 | 1.25 | 1.6e-05 |
| V$NKX61\_01 |  | ceh-24 (0.58) lim-7 alr-1 ceh-9 ceh-8 ceh-31 ceh-30 ceh-1 lin-39 cog-1 ceh-43 ceh-19 | 182 | 1.33 | 1.6e-05 |
| pTH9951 |  | mex-6 | 208 | 1.27 | 1.6e-05 |
| MA0049.1 |  | hbl-1 (0.76) lin-39 php-3 | 219 | 1.24 | 1.9e-05 |
| pTH8997 |  | let-381 hmg-12 lin-39 hmbx-1 lin-31 Y116A8C.22 | 199 | 1.29 | 2.1e-05 |
| exd\_FlyReg\_FBgn0000611 |  | ceh-20 let-381 cfi-1 | 220 | 1.23 | 3.6e-05 |
| HXC6\_f1 |  | nhr-100 lin-1 lin-39 | 204 | 1.26 | 4.4e-05 |
| Abd-A\_FlyReg\_FBgn0000014 |  | ceh-10 (0.6) alr-1 ceh-1 lin-39 ceh-45 eyg-1 | 161 | 1.37 | 5.0e-05 |
| pTH9125 |  | egl-13 (0.66) sox-4 (0.52) | 206 | 1.26 | 5.7e-05 |
| V$FREAC7\_01 |  | lin-31 | 185 | 1.30 | 6.0e-05 |
| CG14962\_SANGER\_5\_FBgn0035407 |  | ces-1 C34H4.5 T22H9.4 | 188 | 1.29 | 8.2e-05 |
| pTH9214 |  | cfi-1 | 204 | 1.25 | 8.4e-05 |
| MA0135.1 |  | lim-7 cfi-1 lin-39 php-3 | 113 | 1.53 | 8.6e-05 |
| pTH7875 |  | mel-28 | 160 | 1.36 | 9.4e-05 |
| pTH6641 |  | lin-31 | 173 | 1.32 | 9.5e-05 |
| pTH9393 |  | ZC416.1 | 91 | 1.65 | 9.5e-05 |
| MA0497.1 |  | mef-2 | 194 | 1.27 | 9.8e-05 |
| FOXO1\_si |  | daf-16 (0.67) fkh-9 irx-1 | 208 | 1.24 | 1.2e-04 |
| MA0536.1 |  | elt-1 | 148 | 1.39 | 1.2e-04 |
| HXB8\_do |  | ceh-20 lin-39 | 181 | 1.30 | 1.3e-04 |
| pTH9958 |  | ztf-6 | 177 | 1.31 | 1.4e-04 |
| Sox17\_2837 |  | sox-4 (0.52) | 169 | 1.32 | 1.7e-04 |
| MA0543.1 |  | eor-1 (0.62) | 201 | 1.25 | 1.8e-04 |
| pTH9901 |  | ceh-24 (0.58) lin-39 ceh-13 php-3 D1005.3 T27F2.4 | 65 | 1.84 | 1.8e-04 |
| rn\_SOLEXA\_5\_FBgn0259172 |  | lin-29 | 218 | 1.21 | 1.9e-04 |
| pTH9335 |  | mel-28 | 199 | 1.25 | 2.6e-04 |
| POU3F1\_2 |  | unc-86 ceh-18 | 174 | 1.30 | 3.0e-04 |
| pTH5916 |  | efl-2 (0.58) | 92 | 1.59 | 3.0e-04 |
| pTH6445 |  | ceh-5 | 138 | 1.39 | 3.6e-04 |
| EMX2\_2 |  | ceh-16 ceh-2 | 77 | 1.68 | 4.1e-04 |
| POU3F3\_1 |  | sox-4 (0.52) ceh-6 ceh-18 | 190 | 1.25 | 5.4e-04 |
| pTH9262 |  | lin-54 | 193 | 1.24 | 6.7e-04 |
| V$BRN2\_01 |  | ceh-18 | 196 | 1.24 | 7.3e-04 |
| Eip74EF\_FlyReg\_FBgn0000567 |  | C24A1.2 | 187 | 1.25 | 7.6e-04 |
| HXD10\_f1 |  | php-3 | 190 | 1.24 | 8.7e-04 |
| CG8765\_SANGER\_5\_FBgn0036900 |  | H20J04.3 | 154 | 1.32 | 9.2e-04 |
| pTH9173 |  | efl-2 (0.58) | 87 | 1.57 | 9.5e-04 |
| pTH9384 |  | cfi-1 | 188 | 1.24 | 1.1e-03 |
| Mw151 |  | gei-11 C34D1.1 | 194 | 1.23 | 1.2e-03 |
| MA0474.1 |  | lin-1 C24A1.2 | 155 | 1.31 | 1.2e-03 |
| HEN1\_si |  | hlh-15 | 116 | 1.42 | 1.4e-03 |
| pTH10638 |  | dmd-3 C34D1.1 | 166 | 1.28 | 1.5e-03 |
| pTH9082 |  | mab-23 | 204 | 1.21 | 1.5e-03 |
| pTH9237 |  | mel-28 | 192 | 1.23 | 2.0e-03 |
| CG7386\_F10-12\_SANGER\_5\_FBgn0035691 |  | F56D1.1 (0.84) gei-3 | 130 | 1.36 | 2.0e-03 |
| HES1\_f1 |  | lin-22 | 103 | 1.45 | 2.3e-03 |
| Sox8\_1733 |  | sox-4 (0.52) gei-3 | 169 | 1.26 | 2.5e-03 |
| pTH2846 |  | lin-31 | 180 | 1.24 | 2.7e-03 |
| Tbp\_pr781 |  | tbp-1 | 173 | 1.25 | 2.7e-03 |
| pTH9297 |  | ceh-18 | 140 | 1.33 | 2.7e-03 |
| Hoxd13\_2356 |  | pal-1 | 148 | 1.31 | 2.8e-03 |
| pTH8985 |  | athp-1 | 140 | 1.33 | 2.9e-03 |
| pnt\_SANGER\_5\_FBgn0003118 |  | lin-1 C24A1.2 | 169 | 1.26 | 3.0e-03 |
| pTH9137 |  | nhr-65 | 193 | 1.21 | 3.7e-03 |
| CG31670\_SANGER\_5\_FBgn0031375 |  | CELE\_Y38H8A.5 | 170 | 1.25 | 3.7e-03 |
| V$OCT1\_06 |  | ceh-18 | 185 | 1.23 | 3.7e-03 |
| pTH5257 |  | C48E7.11 | 116 | 1.38 | 3.8e-03 |
| Pou3f2\_2824 |  | ceh-6 ceh-18 | 112 | 1.40 | 3.9e-03 |
| TCF7L1\_1 |  | pop-1 | 171 | 1.25 | 4.4e-03 |
| Hoxd11\_3873 |  | php-3 | 146 | 1.30 | 4.5e-03 |
| MA0541.1 |  | efl-1 (0.55) F49E12.6 | 113 | 1.39 | 4.5e-03 |
| Pou2f2\_3748 |  | alr-1 ceh-18 | 65 | 1.63 | 5.0e-03 |
| pTH10798 |  | Y75B8A.6 | 26 | 2.39 | 5.2e-03 |
| TFEC\_1 |  | hlh-30 | 113 | 1.38 | 5.2e-03 |
| MA0459.1 |  | nhr-239 | 173 | 1.24 | 5.3e-03 |
| Elf3\_3876 |  | C24A1.2 | 152 | 1.28 | 5.8e-03 |
| pTH6591 |  | lin-31 | 171 | 1.24 | 5.9e-03 |
| Mafk\_3106 |  | F45H11.6 | 169 | 1.24 | 5.9e-03 |
| DLX2\_1 |  | alr-1 ceh-9 ceh-1 ceh-16 ceh-43 | 115 | 1.37 | 6.3e-03 |
| Pou3f1\_3819 |  | ceh-6 | 117 | 1.36 | 6.5e-03 |
| RFX2\_f1 |  | daf-19 (0.86) | 96 | 1.44 | 6.6e-03 |
| Sox1\_2631 |  | sox-4 (0.52) | 38 | 1.96 | 6.6e-03 |
| BARHL2\_6 |  | ceh-31 lin-39 ceh-43 | 151 | 1.28 | 7.1e-03 |
| pTH5119 |  | cfi-1 | 158 | 1.26 | 7.1e-03 |
| pTH1294 |  | mel-28 | 109 | 1.38 | 7.1e-03 |
| Hoxc9\_2367 |  | ceh-12 lin-39 | 104 | 1.40 | 7.5e-03 |
| Hoxd10\_2368 |  | php-3 | 153 | 1.27 | 8.0e-03 |
| V$HOX13\_01 |  | lin-39 | 162 | 1.25 | 8.4e-03 |
| MA0547.1 |  | skn-1 (0.6) ceh-2 | 196 | 1.19 | 8.5e-03 |
| pTH5118 |  | cfi-1 | 171 | 1.23 | 9.5e-03 |
| MA0535.1 |  | daf-8 F45H11.6 | 119 | 1.34 | 9.6e-03 |
| MA0503.1 |  | ceh-24 (0.58) | 108 | 1.37 | 1.0e-02 |
| Msx1\_3031 |  | alr-1 ceh-1 lin-39 ceh-45 ceh-18 | 115 | 1.35 | 1.0e-02 |
| V$S8\_01 |  | ceh-45 | 125 | 1.32 | 1.1e-02 |
| MA0244.1 |  | C48E7.11 | 170 | 1.23 | 1.1e-02 |
| MA0386.1 |  | tbp-1 | 142 | 1.28 | 1.1e-02 |
| MA0262.1 |  | mab-3 | 161 | 1.24 | 1.1e-02 |
| IRX5\_1 |  | irx-1 | 147 | 1.27 | 1.2e-02 |
| amos\_da\_SANGER\_10\_FBgn0003270 |  | ngn-1 hlh-15 | 110 | 1.36 | 1.2e-02 |
| NEUROG2\_1 |  | ngn-1 hlh-8 hlh-32 hlh-15 | 93 | 1.42 | 1.2e-02 |
| pTH9216 |  | ceh-18 | 49 | 1.72 | 1.3e-02 |
| V$CDPCR1\_01 |  | ceh-48 (0.96) | 114 | 1.34 | 1.3e-02 |
| pTH3220 |  | Y5F2A.4 (0.81) | 126 | 1.31 | 1.4e-02 |
| Fer2\_da\_SANGER\_5\_FBgn0038402 |  | hlh-11 hlh-1 lin-32 | 115 | 1.34 | 1.5e-02 |
| pTH9135 |  | pop-1 | 174 | 1.21 | 1.6e-02 |
| pTH5808 |  | ceh-24 (0.58) pal-1 | 133 | 1.29 | 1.6e-02 |
| pTH6497 |  | lin-31 | 158 | 1.24 | 1.6e-02 |
| pTH10788 |  | tbx-33 | 142 | 1.27 | 1.6e-02 |
| V$FAC1\_01 |  | gei-8 (0.53) | 187 | 1.19 | 1.7e-02 |
| pnr\_SANGER\_5\_FBgn0003117 |  | elt-1 | 193 | 1.18 | 1.7e-02 |
| pTH6508 |  | nhr-36 | 128 | 1.30 | 1.8e-02 |
| pTH10696 |  | Y44A6D.3 | 71 | 1.50 | 1.8e-02 |
| Pou2f3\_3986 |  | ceh-18 | 125 | 1.30 | 1.9e-02 |
| BARHL2\_4 |  | ceh-31 | 128 | 1.29 | 1.9e-02 |
| V$PAX6\_01 |  | pax-2 pax-3 | 147 | 1.25 | 1.9e-02 |
| V$SRF\_C |  | unc-120 | 90 | 1.41 | 1.9e-02 |
| Elf5 |  | C24A1.2 | 140 | 1.27 | 2.0e-02 |
| SP4\_f1 |  | klf-2 | 104 | 1.35 | 2.1e-02 |
| pTH9709 |  | die-1 | 182 | 1.19 | 2.1e-02 |
| Cdx2\_4272 |  | ceh-13 | 126 | 1.29 | 2.2e-02 |
| REF1 |  | ref-1 mxl-1 | 105 | 1.35 | 2.3e-02 |
| Lhx1\_2240 |  | lim-7 | 113 | 1.32 | 2.4e-02 |
| pTH9925 |  | ztf-11 | 174 | 1.20 | 2.4e-02 |
| pTH9220 |  | mbr-1 (0.56) | 97 | 1.37 | 2.5e-02 |
| MAX\_1 |  | mxl-1 | 98 | 1.37 | 2.5e-02 |
| pTH3477 |  | daf-16 (0.67) | 170 | 1.21 | 2.6e-02 |
| pTH8983 |  | tag-347 | 121 | 1.30 | 2.7e-02 |
| pTH10037 |  | T22C8.4 | 10 | 3.78 | 2.7e-02 |
| pTH9326 |  | nhr-122 (-0.68) | 22 | 2.26 | 2.7e-02 |
| pTH3046 |  | Y116A8C.22 | 115 | 1.31 | 3.0e-02 |
| Eip93F\_SANGER\_10\_FBgn0013948 |  | mbr-1 (0.56) nhr-177 | 171 | 1.20 | 3.1e-02 |
| Pou3f4\_3773 |  | ceh-6 | 121 | 1.29 | 3.1e-02 |
| V$GATA3\_03 |  | elt-1 | 149 | 1.23 | 3.2e-02 |
| pTH5423 |  | klf-2 | 113 | 1.31 | 3.2e-02 |
| TBX20\_1 |  | mab-9 | 113 | 1.31 | 3.3e-02 |
| I$ELF1\_01 |  | grh-1 (-0.75) | 153 | 1.23 | 3.3e-02 |
| Lmx1a\_2238 |  | lim-7 lim-6 | 90 | 1.37 | 3.5e-02 |
| V$EN1\_01 |  | ceh-16 | 94 | 1.36 | 3.5e-02 |
| pTH9256 |  | ceh-18 | 132 | 1.26 | 3.6e-02 |
| ELF4\_1 |  | C24A1.2 | 132 | 1.26 | 3.8e-02 |
| pTH10808 |  | ztf-19 | 130 | 1.26 | 3.8e-02 |
| pTH9353 |  | ceh-51 | 99 | 1.34 | 3.9e-02 |
| Atf1\_3026 |  | crh-1 | 109 | 1.31 | 4.0e-02 |
| E2F4\_1 |  | F49E12.6 | 29 | 1.92 | 4.1e-02 |
| Hmx1\_3423 |  | ceh-9 | 114 | 1.30 | 4.1e-02 |
| VENTX\_1 |  | pha-2 | 124 | 1.27 | 4.5e-02 |
| MA0495.1 |  | F45H11.6 | 137 | 1.24 | 4.5e-02 |
| pTH10042 |  | nhr-5 (-0.6) | 115 | 1.29 | 4.6e-02 |
| pTH5005 |  | crh-1 | 93 | 1.35 | 4.7e-02 |
| V$CREB\_02 |  | crh-1 | 106 | 1.31 | 4.7e-02 |
| EGR3\_1 |  | ZC328.2 | 121 | 1.27 | 4.8e-02 |
| V$FOXJ2\_02 |  | lin-31 | 165 | 1.20 | 4.9e-02 |

### Correlated (and anti-correlated) transcription factors

|  |  |
| --- | --- |
| **Transcription factor** | **Correlation** |
| ceh-48 | 0.96 |
| nhr-47 | 0.95 |
| ceh-44 | 0.94 |
| dpff-1 | 0.92 |
| Y17G7B.22 | 0.92 |
| C34F6.9 | 0.91 |
| syd-9 | 0.90 |
| spr-4 | 0.90 |
| F54F2.9 | 0.90 |
| ZK673.4 | 0.89 |
| Y37F4.6 | 0.89 |
| F27D4.6 | 0.88 |
| ZK546.5 | 0.88 |
| C33H5.17 | 0.87 |
| ceh-93 | 0.87 |
| flh-2 | 0.87 |
| daf-19 | 0.86 |
| T07F8.4 | 0.85 |
| F56D1.1 | 0.84 |
| hmg-1.2 | 0.84 |
| dhhc-14 | 0.84 |
| spr-1 | 0.84 |
| F13C5.2 | 0.83 |
| ast-1 | 0.83 |
| ceh-58 | 0.83 |
| ceh-7 | -0.47 |
| nhr-88 | -0.47 |
| Y48A6C.1 | -0.48 |
| nhr-18 | -0.48 |
| nhr-237 | -0.50 |
| nhr-41 | -0.50 |
| nhr-156 | -0.51 |
| nhr-207 | -0.52 |
| atf-8 | -0.52 |
| nhr-60 | -0.53 |
| nhr-168 | -0.55 |
| zip-6 | -0.57 |
| nhr-141 | -0.57 |
| nhr-92 | -0.58 |
| nhr-112 | -0.59 |
| ceh-82 | -0.60 |
| nhr-5 | -0.60 |
| nhr-90 | -0.60 |
| nhr-70 | -0.62 |
| ztf-27 | -0.63 |
| nhr-146 | -0.64 |
| nhr-204 | -0.65 |
| nhr-104 | -0.65 |
| nhr-122 | -0.68 |
| grh-1 | -0.75 |

### ChIP peaks enriched

|  |  |  |  |  |
| --- | --- | --- | --- | --- |
| **Gene** | **Experiment** | **Number of upstream peaks** | **Enrichment** | **FDR corrected p** |
| C34F6.9 | C34F6.9\_Larvae-L2-stage | 161 | 2.43 | 1.0e-30 |
| ces-1 | CES-1\_Embryos | 154 | 2.43 | 8.5e-29 |
| lin-35 | LIN-35\_Fed-L1-stage-larvae | 141 | 2.52 | 5.8e-27 |
| efl-1 | EFL-1\_Larvae-L1-stage | 150 | 2.35 | 3.6e-26 |
| efl-1 | EFL-1\_Fed-L1-stage-larvae | 136 | 2.46 | 1.1e-24 |
| dpl-1 | DPL-1\_Larvae-L4-stage | 170 | 1.96 | 7.0e-22 |
| W03F9.2 | W03F9.2\_L4-Young-Adult-stage-larvae | 182 | 1.86 | 1.1e-21 |
| dpl-1 | DPL-1\_Fed-L1-stage-larvae | 127 | 2.34 | 2.1e-20 |
| eor-1 | EOR-1\_Larvae-L3-stage | 143 | 2.10 | 1.4e-19 |
| lsy-2 | LSY-2\_Fed-L1-stage-larvae | 135 | 2.19 | 1.5e-19 |
| F45C12.2 | F45C12.2\_Fed-L1-stage-larvae | 124 | 2.28 | 7.6e-19 |
| lsy-2 | LSY-2\_Embryos | 109 | 2.49 | 1.0e-18 |
| ham-1 | HAM-1\_Larvae-L4-stage | 142 | 2.07 | 1.2e-18 |
| nfya-1 | NFYA-1\_Late-Embryos | 124 | 2.13 | 2.2e-16 |
| ham-1 | HAM-1\_Fed-L1-stage-larvae | 133 | 2.03 | 2.5e-16 |
| gei-11 | GEI-11\_Larvae-L3-stage | 125 | 2.11 | 3.2e-16 |
| lsy-2 | LSY-2\_Larvae-L1-stage | 149 | 1.88 | 4.8e-16 |
| gei-11 | GEI-11\_Fed-L1-stage-larvae | 124 | 2.04 | 7.1e-15 |
| aly-2 | ALY-2\_Fed-L1-stage-larvae | 101 | 2.21 | 1.6e-13 |
| nfya-1 | NFYA-1\_Larvae-L3-stage | 105 | 2.12 | 4.7e-13 |
| pes-1 | PES-1\_Larvae-L4-stage | 124 | 1.89 | 2.5e-12 |
| ceh-39 | CEH-39\_Embryos | 81 | 2.42 | 2.6e-12 |
| ceh-38 | CEH-38\_Larvae-L4-stage | 76 | 2.49 | 5.3e-12 |
| hpl-2 | HPL-2\_Fed-L1-stage-larvae | 137 | 1.75 | 1.3e-11 |
| ceh-26 | CEH-26\_Late-Embryonic-stage | 92 | 2.15 | 2.6e-11 |
| zag-1 | ZAG-1\_Larvae-L2-stage | 90 | 2.17 | 3.0e-11 |
| R02D3.7 | R02D3.7\_Larvae-L3-stage | 125 | 1.81 | 3.1e-11 |
| nhr-129 | NHR-129\_Larvae-L2-stage | 148 | 1.67 | 3.3e-11 |
| fos-1 | FOS-1\_Fed-L1-stage-larvae | 109 | 1.94 | 3.9e-11 |
| alr-1 | ALR-1\_Larvae-L2-stage | 111 | 1.90 | 8.0e-11 |
| nhr-77 | NHR-77\_Larvae-L4-stage | 148 | 1.64 | 1.1e-10 |
| ceh-38 | CEH-38\_Larvae-L3-stage | 96 | 2.03 | 1.5e-10 |
| ces-1 | CES-1\_Larvae-L3-stage | 64 | 2.59 | 1.5e-10 |
| gei-11 | GEI-11\_Larvae-L2-stage | 95 | 2.03 | 2.0e-10 |
| efl-1 | EFL-1\_Young-adult | 123 | 1.74 | 9.2e-10 |
| sem-4 | SEM-4\_Larvae-L2-stage | 110 | 1.79 | 4.1e-09 |
| lin-13 | LIN-13\_Larvae-L2-stage | 82 | 2.06 | 5.6e-09 |
| dpl-1 | DPL-1\_Young-adult | 101 | 1.85 | 7.8e-09 |
| ces-1 | CES-1\_Fed-L1-stage-larvae | 62 | 2.37 | 1.2e-08 |
| jun-1 | JUN-1\_Larvae-L1-stage | 91 | 1.91 | 1.9e-08 |
| pha-4 | PHA-4\_Larvae-L2-stage | 121 | 1.64 | 6.1e-08 |
| F16B12.6 | F16B12.6\_Fed-L1-stage-larvae | 54 | 2.39 | 1.5e-07 |
| C16A3.4 | C16A3.4\_Fed-L1-stage-larvae | 82 | 1.90 | 2.7e-07 |
| C01B12.2 | C01B12.2\_Larvae-L2-stage | 120 | 1.61 | 3.2e-07 |
| sax-3 | SAX-3\_Larvae-L4-stage | 120 | 1.60 | 3.4e-07 |
| lin-15 | LIN-15B\_Fed-L1-stage-larvae | 52 | 2.30 | 1.1e-06 |
| nhr-6 | NHR-6\_Larvae-L2-stage | 97 | 1.71 | 1.1e-06 |
| sax-3 | SAX-3\_Larvae-L2-stage | 83 | 1.81 | 1.6e-06 |
| zag-1 | ZAG-1\_Larvae-L3-stage | 52 | 2.23 | 2.7e-06 |
| egl-5 | EGL-5\_Larvae-L3-stage | 83 | 1.79 | 2.9e-06 |
| hlh-30 | HLH-30\_Larvae-L4-stage | 65 | 1.98 | 3.5e-06 |
| nhr-6 | NHR-6\_Larvae-L4-stage | 64 | 1.99 | 4.1e-06 |
| nhr-77 | NHR-77\_Fed-L1-stage-larvae | 96 | 1.66 | 5.1e-06 |
| jun-1 | JUN-1\_Larvae-L4-stage | 72 | 1.86 | 6.6e-06 |
| F45C12.2 | F45C12.2\_Larvae-L2-stage | 43 | 2.32 | 1.5e-05 |
| sea-2 | SEA-2\_Larvae-L3-stage | 39 | 2.43 | 1.8e-05 |
| hlh-30 | HLH-30\_Late-Embryos | 54 | 2.05 | 2.0e-05 |
| nhr-237 | NHR-237\_Embryos | 49 | 2.11 | 3.2e-05 |
| med-1 | MED-1\_Embryos | 27 | 2.94 | 4.2e-05 |
| ztf-7 | ZTF-7\_Larvae-L4-stage | 63 | 1.84 | 7.0e-05 |
| skn-1 | SKN-1\_Larvae-L3-stage | 49 | 2.04 | 8.1e-05 |
| pha-4 | PHA-4\_Larvae-L4-stage | 61 | 1.82 | 1.3e-04 |
| zag-1 | ZAG-1\_Larvae-L4-stage | 60 | 1.83 | 1.5e-04 |
| R02D3.7 | R02D3.7\_Larvae-L2-stage | 51 | 1.94 | 1.8e-04 |
| zag-1 | ZAG-1\_Fed-L1-stage-larvae | 46 | 1.97 | 3.9e-04 |
| F45C12.2 | F45C12.2\_Larvae-L3-stage | 45 | 1.97 | 5.0e-04 |
| elt-1 | ELT-1\_Larvae-L3-stage | 44 | 1.98 | 5.2e-04 |
| nhr-25 | NHR-25\_Larvae-L2-stage | 83 | 1.56 | 5.9e-04 |
| gei-11 | GEI-11\_Young-adult | 50 | 1.85 | 7.4e-04 |
| dve-1 | DVE-1\_Late-Embryos | 76 | 1.57 | 1.2e-03 |
| aly-2 | ALY-2\_Larvae-L3-stage | 52 | 1.78 | 1.4e-03 |
| fos-1 | FOS-1\_Larvae-L2-stage | 100 | 1.44 | 1.6e-03 |
| F23B12.7 | F23B12.7\_Young-adult | 59 | 1.68 | 1.9e-03 |
| lsy-2 | LSY-2\_Larvae-L4-stage | 43 | 1.87 | 2.3e-03 |
| lsy-2 | LSY-2\_Larvae-L2-stage | 45 | 1.83 | 2.5e-03 |
| R02D3.7 | R02D3.7\_Larvae-L4-stage | 44 | 1.82 | 3.4e-03 |
| pax-1 | PAX-1\_Embryos | 25 | 2.30 | 4.4e-03 |
| mab-5 | MAB-5\_Larvae-L2-stage | 46 | 1.74 | 6.3e-03 |
| nhr-2 | NHR-2\_Embryos | 35 | 1.92 | 6.5e-03 |
| aly-2 | ALY-2\_Larvae-L2-stage | 30 | 2.05 | 6.7e-03 |
| nhr-23 | NHR-23\_Larvae-L3-stage | 72 | 1.50 | 6.9e-03 |
| sax-3 | SAX-3\_Fed-L1-stage-larvae | 38 | 1.85 | 7.1e-03 |
| unc-62 | UNC-62\_Day-Four-Young-Adult | 53 | 1.65 | 7.1e-03 |
| unc-62 | UNC-62\_Young-adult-Day-4 | 53 | 1.65 | 7.1e-03 |
| ces-1 | CES-1\_Larvae-L4-stage | 34 | 1.84 | 1.6e-02 |
| unc-62 | UNC-62\_Larvae-L3-stage | 58 | 1.53 | 2.1e-02 |
| sax-3 | SAX-3\_Larvae-L3-stage | 33 | 1.81 | 2.5e-02 |
| mab-5 | MAB-5\_Embryos | 15 | 2.62 | 2.5e-02 |
| nhr-237 | NHR-237\_Larvae-L1-stage | 28 | 1.91 | 2.8e-02 |
| fkh-2 | FKH-2\_Larvae-L3-stage | 40 | 1.66 | 3.6e-02 |
| lin-15 | LIN-15B\_Larvae-L4-stage | 26 | 1.88 | 4.9e-02 |
| mef-2 | MEF-2\_Fed-L1-stage-larvae | 19 | 2.15 | 4.9e-02 |
