# Supplementary material for: Overlapping cell population expression profiling and regulatory inference in C. elegans
Source: BMC Genomics. 2016 Feb 29;17:159. doi: 10.1186/s12864-016-2482-z (PMC4772325; doi:10.1186/s12864-016-2482-z)
Supplement: Additional file 13: — Web supplement. (DOC 21 kb) [file 12864_2016_2482_MOESM13_ESM.zip › sortWeb/clusters/hier.300.clusters/214.html]

Cluster 214 

## Cluster 214

### Expression

| cnd-1 rep. 1 | cnd-1 rep. 2 | cnd-1 rep. 3 | pha-4 rep. 1 | pha-4 rep. 2 | pha-4 rep. 3 | ceh-27 | ceh-36 | ceh-6 | F21D5.9 | mir-57 | mls-2 | pal-1 | pros-1 | ttx-3 | unc-130 | hlh-16 | irx-1 | ceh-6 (+) hlh-16 (+) | ceh-6 (+) hlh-16 (-) | ceh-6 (-) hlh-16 (+) | cnd-1 singlets | pha-4 singlets | 0 | 60 | 120 | 150 | 180 | 240 | 330 | 390 | 420 | 480 | 540 | 570 | 600 | 630 | 660 | NAME | Functional description |
| --- | --- | --- | --- | --- | --- | --- | --- | --- | --- | --- | --- | --- | --- | --- | --- | --- | --- | --- | --- | --- | --- | --- | --- | --- | --- | --- | --- | --- | --- | --- | --- | --- | --- | --- | --- | --- | --- | --- | --- |
|  |  |  |  |  |  |  |  |  |  |  |  |  |  |  |  |  |  |  |  |  |  |  |  |  |  |  |  |  |  |  |  |  |  |  |  |  |  | *fbxb-97* | F-box B protein |
|  |  |  |  |  |  |  |  |  |  |  |  |  |  |  |  |  |  |  |  |  |  |  |  |  |  |  |  |  |  |  |  |  |  |  |  |  |  | *tag-266* | Temporarily Assigned Gene name |
|  |  |  |  |  |  |  |  |  |  |  |  |  |  |  |  |  |  |  |  |  |  |  |  |  |  |  |  |  |  |  |  |  |  |  |  |  |  | C10A4.1 |  |
|  |  |  |  |  |  |  |  |  |  |  |  |  |  |  |  |  |  |  |  |  |  |  |  |  |  |  |  |  |  |  |  |  |  |  |  |  |  | R09A8.2 |  |
|  |  |  |  |  |  |  |  |  |  |  |  |  |  |  |  |  |  |  |  |  |  |  |  |  |  |  |  |  |  |  |  |  |  |  |  |  |  | Y110A7A.7 |  |
|  |  |  |  |  |  |  |  |  |  |  |  |  |  |  |  |  |  |  |  |  |  |  |  |  |  |  |  |  |  |  |  |  |  |  |  |  |  | *dlst-1* | DihydroLipoamide S-SuccinylTransferase |
|  |  |  |  |  |  |  |  |  |  |  |  |  |  |  |  |  |  |  |  |  |  |  |  |  |  |  |  |  |  |  |  |  |  |  |  |  |  | Y54G2A.50 |  |
|  |  |  |  |  |  |  |  |  |  |  |  |  |  |  |  |  |  |  |  |  |  |  |  |  |  |  |  |  |  |  |  |  |  |  |  |  |  | M116.2 |  |
|  |  |  |  |  |  |  |  |  |  |  |  |  |  |  |  |  |  |  |  |  |  |  |  |  |  |  |  |  |  |  |  |  |  |  |  |  |  | C36A4.12 |  |
|  |  |  |  |  |  |  |  |  |  |  |  |  |  |  |  |  |  |  |  |  |  |  |  |  |  |  |  |  |  |  |  |  |  |  |  |  |  | T26C11.9 |  |
|  |  |  |  |  |  |  |  |  |  |  |  |  |  |  |  |  |  |  |  |  |  |  |  |  |  |  |  |  |  |  |  |  |  |  |  |  |  | *fbxc-33* | F-box C protein |
|  |  |  |  |  |  |  |  |  |  |  |  |  |  |  |  |  |  |  |  |  |  |  |  |  |  |  |  |  |  |  |  |  |  |  |  |  |  | *strd-1* | STRAD (yeast STE20 Related Adaptor protein) homolog |
|  |  |  |  |  |  |  |  |  |  |  |  |  |  |  |  |  |  |  |  |  |  |  |  |  |  |  |  |  |  |  |  |  |  |  |  |  |  | B0491.1 |  |
|  |  |  |  |  |  |  |  |  |  |  |  |  |  |  |  |  |  |  |  |  |  |  |  |  |  |  |  |  |  |  |  |  |  |  |  |  |  | *rnp-1* | RNP (RRM RNA binding domain) containing |
|  |  |  |  |  |  |  |  |  |  |  |  |  |  |  |  |  |  |  |  |  |  |  |  |  |  |  |  |  |  |  |  |  |  |  |  |  |  | *calu-2* | CALUmenin (calcium-binding protein) homolog |
|  |  |  |  |  |  |  |  |  |  |  |  |  |  |  |  |  |  |  |  |  |  |  |  |  |  |  |  |  |  |  |  |  |  |  |  |  |  | *arl-8* | ARF-Like |
|  |  |  |  |  |  |  |  |  |  |  |  |  |  |  |  |  |  |  |  |  |  |  |  |  |  |  |  |  |  |  |  |  |  |  |  |  |  | *cap-1* | CAP-z protein |
|  |  |  |  |  |  |  |  |  |  |  |  |  |  |  |  |  |  |  |  |  |  |  |  |  |  |  |  |  |  |  |  |  |  |  |  |  |  | F38E11.9 |  |
|  |  |  |  |  |  |  |  |  |  |  |  |  |  |  |  |  |  |  |  |  |  |  |  |  |  |  |  |  |  |  |  |  |  |  |  |  |  | F28E10.4 |  |
|  |  |  |  |  |  |  |  |  |  |  |  |  |  |  |  |  |  |  |  |  |  |  |  |  |  |  |  |  |  |  |  |  |  |  |  |  |  | Y51F10.4 |  |
|  |  |  |  |  |  |  |  |  |  |  |  |  |  |  |  |  |  |  |  |  |  |  |  |  |  |  |  |  |  |  |  |  |  |  |  |  |  | *fem-1* | FEMinization of XX and XO animals |
|  |  |  |  |  |  |  |  |  |  |  |  |  |  |  |  |  |  |  |  |  |  |  |  |  |  |  |  |  |  |  |  |  |  |  |  |  |  | *sqv-2* | SQuashed Vulva |
|  |  |  |  |  |  |  |  |  |  |  |  |  |  |  |  |  |  |  |  |  |  |  |  |  |  |  |  |  |  |  |  |  |  |  |  |  |  | *rpt-1* | proteasome Regulatory Particle, ATPase-like |
|  |  |  |  |  |  |  |  |  |  |  |  |  |  |  |  |  |  |  |  |  |  |  |  |  |  |  |  |  |  |  |  |  |  |  |  |  |  | T01H8.2 |  |
|  |  |  |  |  |  |  |  |  |  |  |  |  |  |  |  |  |  |  |  |  |  |  |  |  |  |  |  |  |  |  |  |  |  |  |  |  |  | *oma-1* | Oocyte MAturation defective |
|  |  |  |  |  |  |  |  |  |  |  |  |  |  |  |  |  |  |  |  |  |  |  |  |  |  |  |  |  |  |  |  |  |  |  |  |  |  | *fbxb-20* | F-box B protein |
|  |  |  |  |  |  |  |  |  |  |  |  |  |  |  |  |  |  |  |  |  |  |  |  |  |  |  |  |  |  |  |  |  |  |  |  |  |  | Y105C5B.1413 |  |
|  |  |  |  |  |  |  |  |  |  |  |  |  |  |  |  |  |  |  |  |  |  |  |  |  |  |  |  |  |  |  |  |  |  |  |  |  |  | *bath-20* | BTB and MATH domain containing |
|  |  |  |  |  |  |  |  |  |  |  |  |  |  |  |  |  |  |  |  |  |  |  |  |  |  |  |  |  |  |  |  |  |  |  |  |  |  | Y45F10D.10 |  |
|  |  |  |  |  |  |  |  |  |  |  |  |  |  |  |  |  |  |  |  |  |  |  |  |  |  |  |  |  |  |  |  |  |  |  |  |  |  | *linc-146* | Long Intervening Non-Coding RNA |
|  |  |  |  |  |  |  |  |  |  |  |  |  |  |  |  |  |  |  |  |  |  |  |  |  |  |  |  |  |  |  |  |  |  |  |  |  |  | T16H12.1 |  |
|  |  |  |  |  |  |  |  |  |  |  |  |  |  |  |  |  |  |  |  |  |  |  |  |  |  |  |  |  |  |  |  |  |  |  |  |  |  | M02B7.2 |  |
|  |  |  |  |  |  |  |  |  |  |  |  |  |  |  |  |  |  |  |  |  |  |  |  |  |  |  |  |  |  |  |  |  |  |  |  |  |  | W04C9.6 |  |
|  |  |  |  |  |  |  |  |  |  |  |  |  |  |  |  |  |  |  |  |  |  |  |  |  |  |  |  |  |  |  |  |  |  |  |  |  |  | Y39F10C.2 |  |
|  |  |  |  |  |  |  |  |  |  |  |  |  |  |  |  |  |  |  |  |  |  |  |  |  |  |  |  |  |  |  |  |  |  |  |  |  |  | Y46G5A.18 |  |
|  |  |  |  |  |  |  |  |  |  |  |  |  |  |  |  |  |  |  |  |  |  |  |  |  |  |  |  |  |  |  |  |  |  |  |  |  |  | C05B5.13 |  |
|  |  |  |  |  |  |  |  |  |  |  |  |  |  |  |  |  |  |  |  |  |  |  |  |  |  |  |  |  |  |  |  |  |  |  |  |  |  | *fbxc-23* | F-box C protein |
|  |  |  |  |  |  |  |  |  |  |  |  |  |  |  |  |  |  |  |  |  |  |  |  |  |  |  |  |  |  |  |  |  |  |  |  |  |  | C14B1.8 |  |
|  |  |  |  |  |  |  |  |  |  |  |  |  |  |  |  |  |  |  |  |  |  |  |  |  |  |  |  |  |  |  |  |  |  |  |  |  |  | C25H3.4 |  |
|  |  |  |  |  |  |  |  |  |  |  |  |  |  |  |  |  |  |  |  |  |  |  |  |  |  |  |  |  |  |  |  |  |  |  |  |  |  | M57.1 |  |
|  |  |  |  |  |  |  |  |  |  |  |  |  |  |  |  |  |  |  |  |  |  |  |  |  |  |  |  |  |  |  |  |  |  |  |  |  |  | *prmt-5* | PRotein arginine MethylTransferase |
|  |  |  |  |  |  |  |  |  |  |  |  |  |  |  |  |  |  |  |  |  |  |  |  |  |  |  |  |  |  |  |  |  |  |  |  |  |  | Y116A8C.25 |  |
|  |  |  |  |  |  |  |  |  |  |  |  |  |  |  |  |  |  |  |  |  |  |  |  |  |  |  |  |  |  |  |  |  |  |  |  |  |  | Y6B3B.4 |  |
|  |  |  |  |  |  |  |  |  |  |  |  |  |  |  |  |  |  |  |  |  |  |  |  |  |  |  |  |  |  |  |  |  |  |  |  |  |  | Y48G10A.3 |  |
|  |  |  |  |  |  |  |  |  |  |  |  |  |  |  |  |  |  |  |  |  |  |  |  |  |  |  |  |  |  |  |  |  |  |  |  |  |  | Y56A3A.2 |  |
|  |  |  |  |  |  |  |  |  |  |  |  |  |  |  |  |  |  |  |  |  |  |  |  |  |  |  |  |  |  |  |  |  |  |  |  |  |  | *unc-34* | UNCoordinated |
|  |  |  |  |  |  |  |  |  |  |  |  |  |  |  |  |  |  |  |  |  |  |  |  |  |  |  |  |  |  |  |  |  |  |  |  |  |  | *efl-2* | E2F-like (mammalian transcription factor) |
|  |  |  |  |  |  |  |  |  |  |  |  |  |  |  |  |  |  |  |  |  |  |  |  |  |  |  |  |  |  |  |  |  |  |  |  |  |  | Y71G10AR.4 |  |
|  |  |  |  |  |  |  |  |  |  |  |  |  |  |  |  |  |  |  |  |  |  |  |  |  |  |  |  |  |  |  |  |  |  |  |  |  |  | Y73B3A.20 |  |
|  |  |  |  |  |  |  |  |  |  |  |  |  |  |  |  |  |  |  |  |  |  |  |  |  |  |  |  |  |  |  |  |  |  |  |  |  |  | Y92H12BM.1 |  |
|  |  |  |  |  |  |  |  |  |  |  |  |  |  |  |  |  |  |  |  |  |  |  |  |  |  |  |  |  |  |  |  |  |  |  |  |  |  | *pik-1* | Pelle/IL-1 receptor associated Kinase (IRAK) |
|  |  |  |  |  |  |  |  |  |  |  |  |  |  |  |  |  |  |  |  |  |  |  |  |  |  |  |  |  |  |  |  |  |  |  |  |  |  | Y56A3A.28 |  |
|  |  |  |  |  |  |  |  |  |  |  |  |  |  |  |  |  |  |  |  |  |  |  |  |  |  |  |  |  |  |  |  |  |  |  |  |  |  | C04E7.3 |  |
|  |  |  |  |  |  |  |  |  |  |  |  |  |  |  |  |  |  |  |  |  |  |  |  |  |  |  |  |  |  |  |  |  |  |  |  |  |  | Y55F3BL.2 |  |
|  |  |  |  |  |  |  |  |  |  |  |  |  |  |  |  |  |  |  |  |  |  |  |  |  |  |  |  |  |  |  |  |  |  |  |  |  |  | F48E8.2 |  |
|  |  |  |  |  |  |  |  |  |  |  |  |  |  |  |  |  |  |  |  |  |  |  |  |  |  |  |  |  |  |  |  |  |  |  |  |  |  | *pptr-1* |  |
|  |  |  |  |  |  |  |  |  |  |  |  |  |  |  |  |  |  |  |  |  |  |  |  |  |  |  |  |  |  |  |  |  |  |  |  |  |  | M02B7.5 |  |
|  |  |  |  |  |  |  |  |  |  |  |  |  |  |  |  |  |  |  |  |  |  |  |  |  |  |  |  |  |  |  |  |  |  |  |  |  |  | C04G6.6 |  |
|  |  |  |  |  |  |  |  |  |  |  |  |  |  |  |  |  |  |  |  |  |  |  |  |  |  |  |  |  |  |  |  |  |  |  |  |  |  | Y73B3A.4 |  |
|  |  |  |  |  |  |  |  |  |  |  |  |  |  |  |  |  |  |  |  |  |  |  |  |  |  |  |  |  |  |  |  |  |  |  |  |  |  | F28B3.4 |  |
|  |  |  |  |  |  |  |  |  |  |  |  |  |  |  |  |  |  |  |  |  |  |  |  |  |  |  |  |  |  |  |  |  |  |  |  |  |  | *arf-6* | ADP-Ribosylation Factor related |
|  |  |  |  |  |  |  |  |  |  |  |  |  |  |  |  |  |  |  |  |  |  |  |  |  |  |  |  |  |  |  |  |  |  |  |  |  |  | F46C3.4 |  |
|  |  |  |  |  |  |  |  |  |  |  |  |  |  |  |  |  |  |  |  |  |  |  |  |  |  |  |  |  |  |  |  |  |  |  |  |  |  | *hex-3* | HEXosaminidase |
|  |  |  |  |  |  |  |  |  |  |  |  |  |  |  |  |  |  |  |  |  |  |  |  |  |  |  |  |  |  |  |  |  |  |  |  |  |  | Y67D8A.2 |  |
|  |  |  |  |  |  |  |  |  |  |  |  |  |  |  |  |  |  |  |  |  |  |  |  |  |  |  |  |  |  |  |  |  |  |  |  |  |  | C16A3.11 |  |
|  |  |  |  |  |  |  |  |  |  |  |  |  |  |  |  |  |  |  |  |  |  |  |  |  |  |  |  |  |  |  |  |  |  |  |  |  |  | Y43F8C.22 |  |
|  |  |  |  |  |  |  |  |  |  |  |  |  |  |  |  |  |  |  |  |  |  |  |  |  |  |  |  |  |  |  |  |  |  |  |  |  |  | *bet-1* | BET (two bromodomains) family protein |
|  |  |  |  |  |  |  |  |  |  |  |  |  |  |  |  |  |  |  |  |  |  |  |  |  |  |  |  |  |  |  |  |  |  |  |  |  |  | *sel-8* | Suppressor/Enhancer of Lin-12 |
|  |  |  |  |  |  |  |  |  |  |  |  |  |  |  |  |  |  |  |  |  |  |  |  |  |  |  |  |  |  |  |  |  |  |  |  |  |  | R10E4.11 |  |
|  |  |  |  |  |  |  |  |  |  |  |  |  |  |  |  |  |  |  |  |  |  |  |  |  |  |  |  |  |  |  |  |  |  |  |  |  |  | Y38F2AR.6 |  |
|  |  |  |  |  |  |  |  |  |  |  |  |  |  |  |  |  |  |  |  |  |  |  |  |  |  |  |  |  |  |  |  |  |  |  |  |  |  | F39G3.3 |  |
|  |  |  |  |  |  |  |  |  |  |  |  |  |  |  |  |  |  |  |  |  |  |  |  |  |  |  |  |  |  |  |  |  |  |  |  |  |  | *dot-1.1* | DOT1 histone methyltransferase family |
|  |  |  |  |  |  |  |  |  |  |  |  |  |  |  |  |  |  |  |  |  |  |  |  |  |  |  |  |  |  |  |  |  |  |  |  |  |  | *sfa-1* | Splicing FActor |
|  |  |  |  |  |  |  |  |  |  |  |  |  |  |  |  |  |  |  |  |  |  |  |  |  |  |  |  |  |  |  |  |  |  |  |  |  |  | *uba-2* | UBA (human ubiquitin) related |
|  |  |  |  |  |  |  |  |  |  |  |  |  |  |  |  |  |  |  |  |  |  |  |  |  |  |  |  |  |  |  |  |  |  |  |  |  |  | F07F6.2 |  |

### Phenotypes enriched

none found

### Anatomy terms enriched

none found

### GO terms enriched

none found

### Expression clusters enriched

none found

### Motifs enriched

|  |  |  |  |  |  |
| --- | --- | --- | --- | --- | --- |
| **Motif** | **Logo** | **Possible orthologs** | **Number of motifs in cluster** | **Enrichment** | **FDR corrected p** |
| MA0536.1 |  | elt-1 (0.51) | 54 | 1.72 | 4.0e-05 |
| ONECUT2\_1 |  | ceh-48 (0.77) dsc-1 | 54 | 1.68 | 8.7e-05 |
| pTH8863 |  | hmg-12 | 22 | 2.74 | 1.3e-03 |
| pTH9279 |  | Y116A8C.22 | 44 | 1.61 | 8.1e-03 |
| HepG2\_GABP\_HudsonAlpha |  | lin-1 | 15 | 3.03 | 1.0e-02 |
| pTH9173 |  | efl-2 (0.76) | 47 | 1.53 | 1.3e-02 |
| pTH1294 |  | mel-28 | 59 | 1.35 | 1.6e-02 |
| Spdef\_0905 |  | lin-1 | 55 | 1.38 | 2.2e-02 |
| Elf3 |  | C24A1.2 | 47 | 1.46 | 3.1e-02 |
| pTH3046 |  | Y116A8C.22 | 41 | 1.54 | 3.7e-02 |

### Correlated (and anti-correlated) transcription factors

|  |  |
| --- | --- |
| **Transcription factor** | **Correlation** |
| rcor-1 | 0.87 |
| ZK546.5 | 0.85 |
| ceh-93 | 0.83 |
| F56D1.1 | 0.81 |
| dpff-1 | 0.81 |
| rabx-5 | 0.80 |
| K10B3.5 | 0.80 |
| athp-2 | 0.80 |
| T07F8.4 | 0.79 |
| F54F2.9 | 0.79 |
| R10E4.11 | 0.78 |
| C33H5.17 | 0.78 |
| F13C5.2 | 0.77 |
| ceh-48 | 0.77 |
| ceh-44 | 0.77 |
| tag-146 | 0.76 |
| efl-2 | 0.76 |
| ceh-89 | 0.76 |
| zag-1 | 0.75 |
| ZK673.4 | 0.75 |
| ztf-3 | 0.75 |
| flh-2 | 0.75 |
| lsy-2 | 0.75 |
| nfyb-1 | 0.74 |
| Y17G7B.22 | 0.74 |
| nhr-163 | -0.57 |
| nhr-168 | -0.57 |
| pros-1 | -0.58 |
| C02F12.5 | -0.58 |
| nhr-55 | -0.59 |
| let-381 | -0.59 |
| nhr-141 | -0.59 |
| nhr-140 | -0.60 |
| nhr-180 | -0.60 |
| zip-6 | -0.61 |
| nhr-92 | -0.61 |
| nhr-112 | -0.61 |
| madf-1 | -0.62 |
| nhr-149 | -0.62 |
| nhr-104 | -0.63 |
| nhr-70 | -0.64 |
| nhr-90 | -0.64 |
| nhr-5 | -0.65 |
| atf-8 | -0.65 |
| dhhc-2 | -0.66 |
| ztf-27 | -0.68 |
| nhr-122 | -0.70 |
| nhr-146 | -0.70 |
| nhr-204 | -0.71 |
| grh-1 | -0.74 |

### ChIP peaks enriched

|  |  |  |  |  |
| --- | --- | --- | --- | --- |
| **Gene** | **Experiment** | **Number of upstream peaks** | **Enrichment** | **FDR corrected p** |
| efl-1 | EFL-1\_Fed-L1-stage-larvae | 42 | 2.89 | 2.9e-10 |
| efl-1 | EFL-1\_Young-adult | 46 | 2.48 | 2.4e-09 |
| efl-1 | EFL-1\_Larvae-L1-stage | 43 | 2.56 | 6.7e-09 |
| ceh-38 | CEH-38\_Larvae-L4-stage | 28 | 3.48 | 1.1e-07 |
| lin-35 | LIN-35\_Fed-L1-stage-larvae | 38 | 2.58 | 1.6e-07 |
| pes-1 | PES-1\_Larvae-L4-stage | 40 | 2.31 | 1.0e-06 |
| dpl-1 | DPL-1\_Fed-L1-stage-larvae | 37 | 2.45 | 1.2e-06 |
| W03F9.2 | W03F9.2\_L4-Young-Adult-stage-larvae | 47 | 2.02 | 1.3e-06 |
| lin-13 | LIN-13\_Larvae-L2-stage | 31 | 2.82 | 1.4e-06 |
| ceh-38 | CEH-38\_Larvae-L3-stage | 36 | 2.45 | 2.2e-06 |
| nfya-1 | NFYA-1\_Late-Embryos | 35 | 2.44 | 4.3e-06 |
| eor-1 | EOR-1\_Larvae-L3-stage | 38 | 2.24 | 7.6e-06 |
| gei-11 | GEI-11\_Larvae-L3-stage | 36 | 2.31 | 9.4e-06 |
| hpl-2 | HPL-2\_Fed-L1-stage-larvae | 42 | 2.04 | 1.2e-05 |
| gei-11 | GEI-11\_Fed-L1-stage-larvae | 35 | 2.32 | 1.4e-05 |
| lsy-2 | LSY-2\_Embryos | 29 | 2.67 | 1.5e-05 |
| lsy-2 | LSY-2\_Fed-L1-stage-larvae | 35 | 2.29 | 2.0e-05 |
| dpl-1 | DPL-1\_Larvae-L4-stage | 44 | 1.93 | 2.5e-05 |
| ces-1 | CES-1\_Larvae-L3-stage | 20 | 3.44 | 4.4e-05 |
| ces-1 | CES-1\_Embryos | 36 | 2.16 | 4.8e-05 |
| C34F6.9 | C34F6.9\_Larvae-L2-stage | 35 | 2.11 | 1.2e-04 |
| C16A3.4 | C16A3.4\_Fed-L1-stage-larvae | 28 | 2.46 | 1.3e-04 |
| ceh-39 | CEH-39\_Embryos | 24 | 2.73 | 1.5e-04 |
| gei-11 | GEI-11\_Larvae-L2-stage | 29 | 2.36 | 1.7e-04 |
| ham-1 | HAM-1\_Larvae-L4-stage | 35 | 2.04 | 2.6e-04 |
| F45C12.2 | F45C12.2\_Fed-L1-stage-larvae | 30 | 2.23 | 3.4e-04 |
| F23B12.7 | F23B12.7\_Young-adult | 23 | 2.59 | 5.6e-04 |
| F16B12.6 | F16B12.6\_Fed-L1-stage-larvae | 18 | 3.03 | 8.4e-04 |
| lsy-2 | LSY-2\_Larvae-L1-stage | 38 | 1.82 | 1.1e-03 |
| C01B12.2 | C01B12.2\_Larvae-L2-stage | 40 | 1.76 | 1.2e-03 |
| lsy-2 | LSY-2\_Larvae-L2-stage | 18 | 2.91 | 1.4e-03 |
| F45C12.2 | F45C12.2\_Larvae-L2-stage | 15 | 3.31 | 1.7e-03 |
| R02D3.7 | R02D3.7\_Larvae-L3-stage | 33 | 1.93 | 1.8e-03 |
| jun-1 | JUN-1\_Larvae-L1-stage | 26 | 2.20 | 2.1e-03 |
| nhr-6 | NHR-6\_Larvae-L4-stage | 21 | 2.48 | 2.7e-03 |
| aly-2 | ALY-2\_Fed-L1-stage-larvae | 25 | 2.22 | 2.7e-03 |
| nhr-77 | NHR-77\_Fed-L1-stage-larvae | 30 | 1.98 | 3.1e-03 |
| lin-15 | LIN-15B\_Fed-L1-stage-larvae | 20 | 2.51 | 3.3e-03 |
| dpl-1 | DPL-1\_Young-adult | 26 | 2.13 | 3.4e-03 |
| zag-1 | ZAG-1\_Larvae-L2-stage | 23 | 2.29 | 3.6e-03 |
| nfya-1 | NFYA-1\_Larvae-L3-stage | 27 | 2.08 | 3.7e-03 |
| ham-1 | HAM-1\_Fed-L1-stage-larvae | 31 | 1.91 | 3.9e-03 |
| ces-1 | CES-1\_Fed-L1-stage-larvae | 17 | 2.75 | 4.4e-03 |
| fos-1 | FOS-1\_Fed-L1-stage-larvae | 29 | 1.96 | 4.7e-03 |
| nhr-77 | NHR-77\_Larvae-L2-stage | 19 | 2.51 | 5.1e-03 |
| nhr-77 | NHR-77\_Larvae-L3-stage | 24 | 2.17 | 5.1e-03 |
| R02D3.7 | R02D3.7\_Larvae-L2-stage | 18 | 2.61 | 5.1e-03 |
| ztf-11 | ZTF-11\_Larvae-L3-stage | 7 | 6.28 | 5.3e-03 |
| ztf-7 | ZTF-7\_Larvae-L4-stage | 21 | 2.32 | 6.1e-03 |
| sem-4 | SEM-4\_Larvae-L2-stage | 30 | 1.86 | 8.7e-03 |
| med-1 | MED-1\_Embryos | 9 | 4.19 | 1.1e-02 |
| sax-3 | SAX-3\_Larvae-L2-stage | 23 | 2.06 | 1.5e-02 |
| lin-13 | LIN-13\_Larvae-L4-stage | 19 | 2.28 | 1.5e-02 |
| mab-5 | MAB-5\_Larvae-L2-stage | 16 | 2.53 | 1.6e-02 |
| nhr-77 | NHR-77\_Larvae-L4-stage | 38 | 1.60 | 1.7e-02 |
| gei-11 | GEI-11\_Young-adult | 17 | 2.39 | 1.9e-02 |
| hlh-30 | HLH-30\_Late-Embryos | 16 | 2.48 | 1.9e-02 |
| nhr-129 | NHR-129\_Larvae-L2-stage | 36 | 1.63 | 2.0e-02 |
| alr-1 | ALR-1\_Larvae-L2-stage | 24 | 1.96 | 2.1e-02 |
| ces-1 | CES-1\_Larvae-L4-stage | 15 | 2.56 | 2.1e-02 |
| sax-3 | SAX-3\_Larvae-L4-stage | 33 | 1.68 | 2.3e-02 |
| skn-1 | SKN-1\_Larvae-L3-stage | 15 | 2.49 | 2.8e-02 |
| nhr-23 | NHR-23\_Larvae-L3-stage | 24 | 1.91 | 3.0e-02 |
| aly-2 | ALY-2\_Larvae-L2-stage | 13 | 2.69 | 3.2e-02 |
| gei-11 | GEI-11\_Embryos | 12 | 2.83 | 3.2e-02 |
| egl-5 | EGL-5\_Larvae-L3-stage | 22 | 1.95 | 3.9e-02 |
| dve-1 | DVE-1\_Late-Embryos | 22 | 1.94 | 4.1e-02 |
| R02D3.7 | R02D3.7\_Larvae-L4-stage | 15 | 2.36 | 4.4e-02 |
| elt-3 | ELT-3\_Embryos | 21 | 1.97 | 4.5e-02 |
| nhr-237 | NHR-237\_Larvae-L1-stage | 11 | 2.85 | 4.8e-02 |
| nhr-25 | NHR-25\_Larvae-L2-stage | 22 | 1.91 | 4.9e-02 |
