# Supplementary material for: Overlapping cell population expression profiling and regulatory inference in C. elegans
Source: BMC Genomics. 2016 Feb 29;17:159. doi: 10.1186/s12864-016-2482-z (PMC4772325; doi:10.1186/s12864-016-2482-z)
Supplement: Additional file 13: — Web supplement. (DOC 21 kb) [file 12864_2016_2482_MOESM13_ESM.zip › sortWeb/clusters/hier.300.clusters/220.html]

Cluster 220 

## Cluster 220

### Expression

| cnd-1 rep. 1 | cnd-1 rep. 2 | cnd-1 rep. 3 | pha-4 rep. 1 | pha-4 rep. 2 | pha-4 rep. 3 | ceh-27 | ceh-36 | ceh-6 | F21D5.9 | mir-57 | mls-2 | pal-1 | pros-1 | ttx-3 | unc-130 | hlh-16 | irx-1 | ceh-6 (+) hlh-16 (+) | ceh-6 (+) hlh-16 (-) | ceh-6 (-) hlh-16 (+) | cnd-1 singlets | pha-4 singlets | 0 | 60 | 120 | 150 | 180 | 240 | 330 | 390 | 420 | 480 | 540 | 570 | 600 | 630 | 660 | NAME | Functional description |
| --- | --- | --- | --- | --- | --- | --- | --- | --- | --- | --- | --- | --- | --- | --- | --- | --- | --- | --- | --- | --- | --- | --- | --- | --- | --- | --- | --- | --- | --- | --- | --- | --- | --- | --- | --- | --- | --- | --- | --- |
|  |  |  |  |  |  |  |  |  |  |  |  |  |  |  |  |  |  |  |  |  |  |  |  |  |  |  |  |  |  |  |  |  |  |  |  |  |  | *sra-23* | Serpentine Receptor, class A (alpha) |
|  |  |  |  |  |  |  |  |  |  |  |  |  |  |  |  |  |  |  |  |  |  |  |  |  |  |  |  |  |  |  |  |  |  |  |  |  |  | C14F11.33 |  |
|  |  |  |  |  |  |  |  |  |  |  |  |  |  |  |  |  |  |  |  |  |  |  |  |  |  |  |  |  |  |  |  |  |  |  |  |  |  | *fbxa-163* | F-box A protein |
|  |  |  |  |  |  |  |  |  |  |  |  |  |  |  |  |  |  |  |  |  |  |  |  |  |  |  |  |  |  |  |  |  |  |  |  |  |  | F52H2.13 |  |
|  |  |  |  |  |  |  |  |  |  |  |  |  |  |  |  |  |  |  |  |  |  |  |  |  |  |  |  |  |  |  |  |  |  |  |  |  |  | *set-33* | SET (trithorax/polycomb) domain containing |
|  |  |  |  |  |  |  |  |  |  |  |  |  |  |  |  |  |  |  |  |  |  |  |  |  |  |  |  |  |  |  |  |  |  |  |  |  |  | *vit-4* | VITellogenin structural genes (yolk protein genes) |
|  |  |  |  |  |  |  |  |  |  |  |  |  |  |  |  |  |  |  |  |  |  |  |  |  |  |  |  |  |  |  |  |  |  |  |  |  |  | F46G11.19 |  |
|  |  |  |  |  |  |  |  |  |  |  |  |  |  |  |  |  |  |  |  |  |  |  |  |  |  |  |  |  |  |  |  |  |  |  |  |  |  | R05G6.11 |  |
|  |  |  |  |  |  |  |  |  |  |  |  |  |  |  |  |  |  |  |  |  |  |  |  |  |  |  |  |  |  |  |  |  |  |  |  |  |  | *bcmo-1* | Beta-Carotene 15,15'-MonoOxygenase |
|  |  |  |  |  |  |  |  |  |  |  |  |  |  |  |  |  |  |  |  |  |  |  |  |  |  |  |  |  |  |  |  |  |  |  |  |  |  | F30F8.9 |  |
|  |  |  |  |  |  |  |  |  |  |  |  |  |  |  |  |  |  |  |  |  |  |  |  |  |  |  |  |  |  |  |  |  |  |  |  |  |  | *set-20* | SET (trithorax/polycomb) domain containing |
|  |  |  |  |  |  |  |  |  |  |  |  |  |  |  |  |  |  |  |  |  |  |  |  |  |  |  |  |  |  |  |  |  |  |  |  |  |  | *sdz-11* | SKN-1 Dependent Zygotic transcript |
|  |  |  |  |  |  |  |  |  |  |  |  |  |  |  |  |  |  |  |  |  |  |  |  |  |  |  |  |  |  |  |  |  |  |  |  |  |  | *spd-3* | SPindle Defective |
|  |  |  |  |  |  |  |  |  |  |  |  |  |  |  |  |  |  |  |  |  |  |  |  |  |  |  |  |  |  |  |  |  |  |  |  |  |  | F55A12.6 |  |
|  |  |  |  |  |  |  |  |  |  |  |  |  |  |  |  |  |  |  |  |  |  |  |  |  |  |  |  |  |  |  |  |  |  |  |  |  |  | F49H6.5 |  |
|  |  |  |  |  |  |  |  |  |  |  |  |  |  |  |  |  |  |  |  |  |  |  |  |  |  |  |  |  |  |  |  |  |  |  |  |  |  | F42C5.3 |  |
|  |  |  |  |  |  |  |  |  |  |  |  |  |  |  |  |  |  |  |  |  |  |  |  |  |  |  |  |  |  |  |  |  |  |  |  |  |  | F41H8.1 |  |
|  |  |  |  |  |  |  |  |  |  |  |  |  |  |  |  |  |  |  |  |  |  |  |  |  |  |  |  |  |  |  |  |  |  |  |  |  |  | D1022.3 |  |
|  |  |  |  |  |  |  |  |  |  |  |  |  |  |  |  |  |  |  |  |  |  |  |  |  |  |  |  |  |  |  |  |  |  |  |  |  |  | F12F6.12 |  |
|  |  |  |  |  |  |  |  |  |  |  |  |  |  |  |  |  |  |  |  |  |  |  |  |  |  |  |  |  |  |  |  |  |  |  |  |  |  | R04F11.9 |  |
|  |  |  |  |  |  |  |  |  |  |  |  |  |  |  |  |  |  |  |  |  |  |  |  |  |  |  |  |  |  |  |  |  |  |  |  |  |  | *srh-116* | Serpentine Receptor, class H |
|  |  |  |  |  |  |  |  |  |  |  |  |  |  |  |  |  |  |  |  |  |  |  |  |  |  |  |  |  |  |  |  |  |  |  |  |  |  | C27D6.14 |  |
|  |  |  |  |  |  |  |  |  |  |  |  |  |  |  |  |  |  |  |  |  |  |  |  |  |  |  |  |  |  |  |  |  |  |  |  |  |  | *srh-134* | Serpentine Receptor, class H |
|  |  |  |  |  |  |  |  |  |  |  |  |  |  |  |  |  |  |  |  |  |  |  |  |  |  |  |  |  |  |  |  |  |  |  |  |  |  | *srh-111* | Serpentine Receptor, class H |
|  |  |  |  |  |  |  |  |  |  |  |  |  |  |  |  |  |  |  |  |  |  |  |  |  |  |  |  |  |  |  |  |  |  |  |  |  |  | C52B9.14 |  |
|  |  |  |  |  |  |  |  |  |  |  |  |  |  |  |  |  |  |  |  |  |  |  |  |  |  |  |  |  |  |  |  |  |  |  |  |  |  | *ucr-1* | Ubiquinol-Cytochrome c oxidoReductase complex |
|  |  |  |  |  |  |  |  |  |  |  |  |  |  |  |  |  |  |  |  |  |  |  |  |  |  |  |  |  |  |  |  |  |  |  |  |  |  | Y2H9A.6 |  |
|  |  |  |  |  |  |  |  |  |  |  |  |  |  |  |  |  |  |  |  |  |  |  |  |  |  |  |  |  |  |  |  |  |  |  |  |  |  | C18D4.8 |  |
|  |  |  |  |  |  |  |  |  |  |  |  |  |  |  |  |  |  |  |  |  |  |  |  |  |  |  |  |  |  |  |  |  |  |  |  |  |  | C25A1.15 |  |
|  |  |  |  |  |  |  |  |  |  |  |  |  |  |  |  |  |  |  |  |  |  |  |  |  |  |  |  |  |  |  |  |  |  |  |  |  |  | F48A11.2 |  |
|  |  |  |  |  |  |  |  |  |  |  |  |  |  |  |  |  |  |  |  |  |  |  |  |  |  |  |  |  |  |  |  |  |  |  |  |  |  | Y39G10AR.11 |  |
|  |  |  |  |  |  |  |  |  |  |  |  |  |  |  |  |  |  |  |  |  |  |  |  |  |  |  |  |  |  |  |  |  |  |  |  |  |  | *ced-10* | CEll Death abnormality |
|  |  |  |  |  |  |  |  |  |  |  |  |  |  |  |  |  |  |  |  |  |  |  |  |  |  |  |  |  |  |  |  |  |  |  |  |  |  | *ncl-1* | abnormal NuCLeoli |
|  |  |  |  |  |  |  |  |  |  |  |  |  |  |  |  |  |  |  |  |  |  |  |  |  |  |  |  |  |  |  |  |  |  |  |  |  |  | *dyn-1* | DYNamin related |
|  |  |  |  |  |  |  |  |  |  |  |  |  |  |  |  |  |  |  |  |  |  |  |  |  |  |  |  |  |  |  |  |  |  |  |  |  |  | *rep-1* | Rab Escort Protein homolog |
|  |  |  |  |  |  |  |  |  |  |  |  |  |  |  |  |  |  |  |  |  |  |  |  |  |  |  |  |  |  |  |  |  |  |  |  |  |  | *rbg-1* | RaB GAP related |
|  |  |  |  |  |  |  |  |  |  |  |  |  |  |  |  |  |  |  |  |  |  |  |  |  |  |  |  |  |  |  |  |  |  |  |  |  |  | Y106G6H.5 |  |
|  |  |  |  |  |  |  |  |  |  |  |  |  |  |  |  |  |  |  |  |  |  |  |  |  |  |  |  |  |  |  |  |  |  |  |  |  |  | R10E11.6 |  |
|  |  |  |  |  |  |  |  |  |  |  |  |  |  |  |  |  |  |  |  |  |  |  |  |  |  |  |  |  |  |  |  |  |  |  |  |  |  | *let-526* | LEThal |
|  |  |  |  |  |  |  |  |  |  |  |  |  |  |  |  |  |  |  |  |  |  |  |  |  |  |  |  |  |  |  |  |  |  |  |  |  |  | *elks-1* | mammalian ELKS/CAST/ERC/Rab6 interacting protein homolog |
|  |  |  |  |  |  |  |  |  |  |  |  |  |  |  |  |  |  |  |  |  |  |  |  |  |  |  |  |  |  |  |  |  |  |  |  |  |  | Y106G6D.7 |  |
|  |  |  |  |  |  |  |  |  |  |  |  |  |  |  |  |  |  |  |  |  |  |  |  |  |  |  |  |  |  |  |  |  |  |  |  |  |  | Y37H9A.3 |  |
|  |  |  |  |  |  |  |  |  |  |  |  |  |  |  |  |  |  |  |  |  |  |  |  |  |  |  |  |  |  |  |  |  |  |  |  |  |  | F36D4.5 |  |
|  |  |  |  |  |  |  |  |  |  |  |  |  |  |  |  |  |  |  |  |  |  |  |  |  |  |  |  |  |  |  |  |  |  |  |  |  |  | F09A5.4 |  |
|  |  |  |  |  |  |  |  |  |  |  |  |  |  |  |  |  |  |  |  |  |  |  |  |  |  |  |  |  |  |  |  |  |  |  |  |  |  | *fbxc-57* | F-box C protein |
|  |  |  |  |  |  |  |  |  |  |  |  |  |  |  |  |  |  |  |  |  |  |  |  |  |  |  |  |  |  |  |  |  |  |  |  |  |  | W06H8.5 |  |
|  |  |  |  |  |  |  |  |  |  |  |  |  |  |  |  |  |  |  |  |  |  |  |  |  |  |  |  |  |  |  |  |  |  |  |  |  |  | *csk-1* | C-terminal Src Kinase |
|  |  |  |  |  |  |  |  |  |  |  |  |  |  |  |  |  |  |  |  |  |  |  |  |  |  |  |  |  |  |  |  |  |  |  |  |  |  | *lin-23* | abnormal cell LINeage |
|  |  |  |  |  |  |  |  |  |  |  |  |  |  |  |  |  |  |  |  |  |  |  |  |  |  |  |  |  |  |  |  |  |  |  |  |  |  | *efn-4* | Eph(F)riN |
|  |  |  |  |  |  |  |  |  |  |  |  |  |  |  |  |  |  |  |  |  |  |  |  |  |  |  |  |  |  |  |  |  |  |  |  |  |  | *sqst-1* | SeQueSTosome related |
|  |  |  |  |  |  |  |  |  |  |  |  |  |  |  |  |  |  |  |  |  |  |  |  |  |  |  |  |  |  |  |  |  |  |  |  |  |  | B0350.t1 |  |
|  |  |  |  |  |  |  |  |  |  |  |  |  |  |  |  |  |  |  |  |  |  |  |  |  |  |  |  |  |  |  |  |  |  |  |  |  |  | Y50D7A.8 |  |
|  |  |  |  |  |  |  |  |  |  |  |  |  |  |  |  |  |  |  |  |  |  |  |  |  |  |  |  |  |  |  |  |  |  |  |  |  |  | C37C3.2 |  |
|  |  |  |  |  |  |  |  |  |  |  |  |  |  |  |  |  |  |  |  |  |  |  |  |  |  |  |  |  |  |  |  |  |  |  |  |  |  | *ppfr-1* | Protein Phosphatase Four Regulatory subunit |
|  |  |  |  |  |  |  |  |  |  |  |  |  |  |  |  |  |  |  |  |  |  |  |  |  |  |  |  |  |  |  |  |  |  |  |  |  |  | *jmjd-3.1* | JuMonJi (transcription factor) Domain protein |
|  |  |  |  |  |  |  |  |  |  |  |  |  |  |  |  |  |  |  |  |  |  |  |  |  |  |  |  |  |  |  |  |  |  |  |  |  |  | K04C1.2 |  |
|  |  |  |  |  |  |  |  |  |  |  |  |  |  |  |  |  |  |  |  |  |  |  |  |  |  |  |  |  |  |  |  |  |  |  |  |  |  | *gei-17* | GEX Interacting protein |
|  |  |  |  |  |  |  |  |  |  |  |  |  |  |  |  |  |  |  |  |  |  |  |  |  |  |  |  |  |  |  |  |  |  |  |  |  |  | *mig-38* | abnormal cell MIGration |
|  |  |  |  |  |  |  |  |  |  |  |  |  |  |  |  |  |  |  |  |  |  |  |  |  |  |  |  |  |  |  |  |  |  |  |  |  |  | *rbr-2* | RB (Retinoblastoma Binding protein) Related |
|  |  |  |  |  |  |  |  |  |  |  |  |  |  |  |  |  |  |  |  |  |  |  |  |  |  |  |  |  |  |  |  |  |  |  |  |  |  | *pmk-3* | P38 Map Kinase family |
|  |  |  |  |  |  |  |  |  |  |  |  |  |  |  |  |  |  |  |  |  |  |  |  |  |  |  |  |  |  |  |  |  |  |  |  |  |  | *syd-2* | SYnapse Defective |
|  |  |  |  |  |  |  |  |  |  |  |  |  |  |  |  |  |  |  |  |  |  |  |  |  |  |  |  |  |  |  |  |  |  |  |  |  |  | *miz-1* | MIZ-type zinc finger putative transcription factor |
|  |  |  |  |  |  |  |  |  |  |  |  |  |  |  |  |  |  |  |  |  |  |  |  |  |  |  |  |  |  |  |  |  |  |  |  |  |  | *mrck-1* | Myotonic dystrophy-Related, Cdc42-binding Kinase homolog |
|  |  |  |  |  |  |  |  |  |  |  |  |  |  |  |  |  |  |  |  |  |  |  |  |  |  |  |  |  |  |  |  |  |  |  |  |  |  | *mca-3* | Membrane Calcium ATPase |
|  |  |  |  |  |  |  |  |  |  |  |  |  |  |  |  |  |  |  |  |  |  |  |  |  |  |  |  |  |  |  |  |  |  |  |  |  |  | *men-1* | Malic ENzyme |
|  |  |  |  |  |  |  |  |  |  |  |  |  |  |  |  |  |  |  |  |  |  |  |  |  |  |  |  |  |  |  |  |  |  |  |  |  |  | *dpy-23* | DumPY: shorter than wild-type |
|  |  |  |  |  |  |  |  |  |  |  |  |  |  |  |  |  |  |  |  |  |  |  |  |  |  |  |  |  |  |  |  |  |  |  |  |  |  | *egl-30* | EGg Laying defective |
|  |  |  |  |  |  |  |  |  |  |  |  |  |  |  |  |  |  |  |  |  |  |  |  |  |  |  |  |  |  |  |  |  |  |  |  |  |  | *tag-146* | Temporarily Assigned Gene name |
|  |  |  |  |  |  |  |  |  |  |  |  |  |  |  |  |  |  |  |  |  |  |  |  |  |  |  |  |  |  |  |  |  |  |  |  |  |  | *arx-3* | ARp2/3 compleX component |
|  |  |  |  |  |  |  |  |  |  |  |  |  |  |  |  |  |  |  |  |  |  |  |  |  |  |  |  |  |  |  |  |  |  |  |  |  |  | *hmr-1* | HaMmeRhead embryonic lethal |
|  |  |  |  |  |  |  |  |  |  |  |  |  |  |  |  |  |  |  |  |  |  |  |  |  |  |  |  |  |  |  |  |  |  |  |  |  |  | *epg-9* | Ectopic P Granules |
|  |  |  |  |  |  |  |  |  |  |  |  |  |  |  |  |  |  |  |  |  |  |  |  |  |  |  |  |  |  |  |  |  |  |  |  |  |  | R05H5.4 |  |
|  |  |  |  |  |  |  |  |  |  |  |  |  |  |  |  |  |  |  |  |  |  |  |  |  |  |  |  |  |  |  |  |  |  |  |  |  |  | W09D10.1 |  |
|  |  |  |  |  |  |  |  |  |  |  |  |  |  |  |  |  |  |  |  |  |  |  |  |  |  |  |  |  |  |  |  |  |  |  |  |  |  | C07A12.7 |  |
|  |  |  |  |  |  |  |  |  |  |  |  |  |  |  |  |  |  |  |  |  |  |  |  |  |  |  |  |  |  |  |  |  |  |  |  |  |  | *agr-1* | AGRin (synaptic protein) homolog |
|  |  |  |  |  |  |  |  |  |  |  |  |  |  |  |  |  |  |  |  |  |  |  |  |  |  |  |  |  |  |  |  |  |  |  |  |  |  | *cdh-9* | CaDHerin family |
|  |  |  |  |  |  |  |  |  |  |  |  |  |  |  |  |  |  |  |  |  |  |  |  |  |  |  |  |  |  |  |  |  |  |  |  |  |  | C29E4.9 |  |
|  |  |  |  |  |  |  |  |  |  |  |  |  |  |  |  |  |  |  |  |  |  |  |  |  |  |  |  |  |  |  |  |  |  |  |  |  |  | Y38C1AA.1 |  |
|  |  |  |  |  |  |  |  |  |  |  |  |  |  |  |  |  |  |  |  |  |  |  |  |  |  |  |  |  |  |  |  |  |  |  |  |  |  | *twk-26* | TWiK family of potassium channels |
|  |  |  |  |  |  |  |  |  |  |  |  |  |  |  |  |  |  |  |  |  |  |  |  |  |  |  |  |  |  |  |  |  |  |  |  |  |  | *stg-1* | STarGazin (mammalian calcium channel) homolog |
|  |  |  |  |  |  |  |  |  |  |  |  |  |  |  |  |  |  |  |  |  |  |  |  |  |  |  |  |  |  |  |  |  |  |  |  |  |  | ZC443.4 |  |
|  |  |  |  |  |  |  |  |  |  |  |  |  |  |  |  |  |  |  |  |  |  |  |  |  |  |  |  |  |  |  |  |  |  |  |  |  |  | *frm-10* | FERM domain (protein4.1-ezrin-radixin-moesin) family |
|  |  |  |  |  |  |  |  |  |  |  |  |  |  |  |  |  |  |  |  |  |  |  |  |  |  |  |  |  |  |  |  |  |  |  |  |  |  | *pamn-1* | Peptidylglycine alpha-Amidating MonooxygeNase |
|  |  |  |  |  |  |  |  |  |  |  |  |  |  |  |  |  |  |  |  |  |  |  |  |  |  |  |  |  |  |  |  |  |  |  |  |  |  | *set-19* | SET (trithorax/polycomb) domain containing |
|  |  |  |  |  |  |  |  |  |  |  |  |  |  |  |  |  |  |  |  |  |  |  |  |  |  |  |  |  |  |  |  |  |  |  |  |  |  | *vps-45* | related to yeast Vacuolar Protein Sorting factor |
|  |  |  |  |  |  |  |  |  |  |  |  |  |  |  |  |  |  |  |  |  |  |  |  |  |  |  |  |  |  |  |  |  |  |  |  |  |  | *stam-1* | STAM (Signal Transducing Adapter Molecule) homolog |
|  |  |  |  |  |  |  |  |  |  |  |  |  |  |  |  |  |  |  |  |  |  |  |  |  |  |  |  |  |  |  |  |  |  |  |  |  |  | *bath-44* | BTB and MATH domain containing |
|  |  |  |  |  |  |  |  |  |  |  |  |  |  |  |  |  |  |  |  |  |  |  |  |  |  |  |  |  |  |  |  |  |  |  |  |  |  | F07A5.2 |  |
|  |  |  |  |  |  |  |  |  |  |  |  |  |  |  |  |  |  |  |  |  |  |  |  |  |  |  |  |  |  |  |  |  |  |  |  |  |  | B0361.8 |  |
|  |  |  |  |  |  |  |  |  |  |  |  |  |  |  |  |  |  |  |  |  |  |  |  |  |  |  |  |  |  |  |  |  |  |  |  |  |  | *kpc-1* | Kex-2 Proprotein Convertase family |
|  |  |  |  |  |  |  |  |  |  |  |  |  |  |  |  |  |  |  |  |  |  |  |  |  |  |  |  |  |  |  |  |  |  |  |  |  |  | R03D7.4 |  |
|  |  |  |  |  |  |  |  |  |  |  |  |  |  |  |  |  |  |  |  |  |  |  |  |  |  |  |  |  |  |  |  |  |  |  |  |  |  | *qns-1* | glutamine(Q)-dependent NAD(+) Synthase |
|  |  |  |  |  |  |  |  |  |  |  |  |  |  |  |  |  |  |  |  |  |  |  |  |  |  |  |  |  |  |  |  |  |  |  |  |  |  | *larp-1* | LARP (RNA binding La related protein) homolog |
|  |  |  |  |  |  |  |  |  |  |  |  |  |  |  |  |  |  |  |  |  |  |  |  |  |  |  |  |  |  |  |  |  |  |  |  |  |  | *paqr-2* | Progestin and AdipoQ Receptor family |
|  |  |  |  |  |  |  |  |  |  |  |  |  |  |  |  |  |  |  |  |  |  |  |  |  |  |  |  |  |  |  |  |  |  |  |  |  |  | T28B11.1 |  |
|  |  |  |  |  |  |  |  |  |  |  |  |  |  |  |  |  |  |  |  |  |  |  |  |  |  |  |  |  |  |  |  |  |  |  |  |  |  | *zfp-1* | Zinc Finger Protein |
|  |  |  |  |  |  |  |  |  |  |  |  |  |  |  |  |  |  |  |  |  |  |  |  |  |  |  |  |  |  |  |  |  |  |  |  |  |  | *lin-66* | abnormal cell LINeage |
|  |  |  |  |  |  |  |  |  |  |  |  |  |  |  |  |  |  |  |  |  |  |  |  |  |  |  |  |  |  |  |  |  |  |  |  |  |  | *lsy-12* | Laterally SYmmetric (defective in lateral asymmetry) |
|  |  |  |  |  |  |  |  |  |  |  |  |  |  |  |  |  |  |  |  |  |  |  |  |  |  |  |  |  |  |  |  |  |  |  |  |  |  | *sma-9* | SMAll |
|  |  |  |  |  |  |  |  |  |  |  |  |  |  |  |  |  |  |  |  |  |  |  |  |  |  |  |  |  |  |  |  |  |  |  |  |  |  | *lin-45* | abnormal cell LINeage |
|  |  |  |  |  |  |  |  |  |  |  |  |  |  |  |  |  |  |  |  |  |  |  |  |  |  |  |  |  |  |  |  |  |  |  |  |  |  | *egrh-1* | EGR (Early Growth factor Response factor) Homolog |
|  |  |  |  |  |  |  |  |  |  |  |  |  |  |  |  |  |  |  |  |  |  |  |  |  |  |  |  |  |  |  |  |  |  |  |  |  |  | *tbc-11* | TBC (Tre-2/Bub2/Cdc16) domain family |
|  |  |  |  |  |  |  |  |  |  |  |  |  |  |  |  |  |  |  |  |  |  |  |  |  |  |  |  |  |  |  |  |  |  |  |  |  |  | *abt-2* | ABC Transporter family |
|  |  |  |  |  |  |  |  |  |  |  |  |  |  |  |  |  |  |  |  |  |  |  |  |  |  |  |  |  |  |  |  |  |  |  |  |  |  | *tbc-1* | TBC (Tre-2/Bub2/Cdc16) domain family |
|  |  |  |  |  |  |  |  |  |  |  |  |  |  |  |  |  |  |  |  |  |  |  |  |  |  |  |  |  |  |  |  |  |  |  |  |  |  | *let-858* | LEThal |
|  |  |  |  |  |  |  |  |  |  |  |  |  |  |  |  |  |  |  |  |  |  |  |  |  |  |  |  |  |  |  |  |  |  |  |  |  |  | R01H10.7 |  |
|  |  |  |  |  |  |  |  |  |  |  |  |  |  |  |  |  |  |  |  |  |  |  |  |  |  |  |  |  |  |  |  |  |  |  |  |  |  | *haf-6* | HAlF transporter (PGP related) |
|  |  |  |  |  |  |  |  |  |  |  |  |  |  |  |  |  |  |  |  |  |  |  |  |  |  |  |  |  |  |  |  |  |  |  |  |  |  | C39B5.6 |  |
|  |  |  |  |  |  |  |  |  |  |  |  |  |  |  |  |  |  |  |  |  |  |  |  |  |  |  |  |  |  |  |  |  |  |  |  |  |  | *tag-147* | Temporarily Assigned Gene name |
|  |  |  |  |  |  |  |  |  |  |  |  |  |  |  |  |  |  |  |  |  |  |  |  |  |  |  |  |  |  |  |  |  |  |  |  |  |  | *abcf-2* | ABC transporter, class F |
|  |  |  |  |  |  |  |  |  |  |  |  |  |  |  |  |  |  |  |  |  |  |  |  |  |  |  |  |  |  |  |  |  |  |  |  |  |  | *immt-1* | Inner Membrane of MiTochondria protein homolog |
|  |  |  |  |  |  |  |  |  |  |  |  |  |  |  |  |  |  |  |  |  |  |  |  |  |  |  |  |  |  |  |  |  |  |  |  |  |  | *mtm-9* | MTM (myotubularin) family |
|  |  |  |  |  |  |  |  |  |  |  |  |  |  |  |  |  |  |  |  |  |  |  |  |  |  |  |  |  |  |  |  |  |  |  |  |  |  | *tam-1* | Tandem Array expression Modifier |
|  |  |  |  |  |  |  |  |  |  |  |  |  |  |  |  |  |  |  |  |  |  |  |  |  |  |  |  |  |  |  |  |  |  |  |  |  |  | *nono-1* | NONO (conserved nuclear protein, aka PSF) homolog |
|  |  |  |  |  |  |  |  |  |  |  |  |  |  |  |  |  |  |  |  |  |  |  |  |  |  |  |  |  |  |  |  |  |  |  |  |  |  | *vesa-1* | VESicle-Associated |
|  |  |  |  |  |  |  |  |  |  |  |  |  |  |  |  |  |  |  |  |  |  |  |  |  |  |  |  |  |  |  |  |  |  |  |  |  |  | C12D8.1 |  |
|  |  |  |  |  |  |  |  |  |  |  |  |  |  |  |  |  |  |  |  |  |  |  |  |  |  |  |  |  |  |  |  |  |  |  |  |  |  | *daf-1* | abnormal DAuer Formation |
|  |  |  |  |  |  |  |  |  |  |  |  |  |  |  |  |  |  |  |  |  |  |  |  |  |  |  |  |  |  |  |  |  |  |  |  |  |  | Y61A9LA.3 |  |
|  |  |  |  |  |  |  |  |  |  |  |  |  |  |  |  |  |  |  |  |  |  |  |  |  |  |  |  |  |  |  |  |  |  |  |  |  |  | *exoc-8* | EXOCyst component |
|  |  |  |  |  |  |  |  |  |  |  |  |  |  |  |  |  |  |  |  |  |  |  |  |  |  |  |  |  |  |  |  |  |  |  |  |  |  | *cit-1.2* | CyclIn T |
|  |  |  |  |  |  |  |  |  |  |  |  |  |  |  |  |  |  |  |  |  |  |  |  |  |  |  |  |  |  |  |  |  |  |  |  |  |  | *ppm-1* | Protein Phosphatase, Mg2+/Mn2+ dependent |
|  |  |  |  |  |  |  |  |  |  |  |  |  |  |  |  |  |  |  |  |  |  |  |  |  |  |  |  |  |  |  |  |  |  |  |  |  |  | *ing-3* | ING (mammalian INhibitor of Growth) homolog |
|  |  |  |  |  |  |  |  |  |  |  |  |  |  |  |  |  |  |  |  |  |  |  |  |  |  |  |  |  |  |  |  |  |  |  |  |  |  | C47G2.4 |  |
|  |  |  |  |  |  |  |  |  |  |  |  |  |  |  |  |  |  |  |  |  |  |  |  |  |  |  |  |  |  |  |  |  |  |  |  |  |  | *rsy-1* | Regulator of SYnapse formation |
|  |  |  |  |  |  |  |  |  |  |  |  |  |  |  |  |  |  |  |  |  |  |  |  |  |  |  |  |  |  |  |  |  |  |  |  |  |  | *mig-15* | abnormal cell MIGration |
|  |  |  |  |  |  |  |  |  |  |  |  |  |  |  |  |  |  |  |  |  |  |  |  |  |  |  |  |  |  |  |  |  |  |  |  |  |  | *sydn-1* | SSynaptic Defective eNhancer |
|  |  |  |  |  |  |  |  |  |  |  |  |  |  |  |  |  |  |  |  |  |  |  |  |  |  |  |  |  |  |  |  |  |  |  |  |  |  | Y71A12B.18 |  |
|  |  |  |  |  |  |  |  |  |  |  |  |  |  |  |  |  |  |  |  |  |  |  |  |  |  |  |  |  |  |  |  |  |  |  |  |  |  | Y116A8C.10 |  |
|  |  |  |  |  |  |  |  |  |  |  |  |  |  |  |  |  |  |  |  |  |  |  |  |  |  |  |  |  |  |  |  |  |  |  |  |  |  | *unc-51* | UNCoordinated |
|  |  |  |  |  |  |  |  |  |  |  |  |  |  |  |  |  |  |  |  |  |  |  |  |  |  |  |  |  |  |  |  |  |  |  |  |  |  | *morc-1* | MORC (mouse microrchidia) family CW-type zinc finger protein |
|  |  |  |  |  |  |  |  |  |  |  |  |  |  |  |  |  |  |  |  |  |  |  |  |  |  |  |  |  |  |  |  |  |  |  |  |  |  | Y67D8A.1 |  |
|  |  |  |  |  |  |  |  |  |  |  |  |  |  |  |  |  |  |  |  |  |  |  |  |  |  |  |  |  |  |  |  |  |  |  |  |  |  | *hpo-20* | Hypersensitive to POre-forming toxin |
|  |  |  |  |  |  |  |  |  |  |  |  |  |  |  |  |  |  |  |  |  |  |  |  |  |  |  |  |  |  |  |  |  |  |  |  |  |  | Y53G8AR.8 |  |
|  |  |  |  |  |  |  |  |  |  |  |  |  |  |  |  |  |  |  |  |  |  |  |  |  |  |  |  |  |  |  |  |  |  |  |  |  |  | Y73B6BL.14 |  |
|  |  |  |  |  |  |  |  |  |  |  |  |  |  |  |  |  |  |  |  |  |  |  |  |  |  |  |  |  |  |  |  |  |  |  |  |  |  | *skn-1* | SKiNhead |
|  |  |  |  |  |  |  |  |  |  |  |  |  |  |  |  |  |  |  |  |  |  |  |  |  |  |  |  |  |  |  |  |  |  |  |  |  |  | *cpt-2* | Carnitine Palmitoyl Transferase |
|  |  |  |  |  |  |  |  |  |  |  |  |  |  |  |  |  |  |  |  |  |  |  |  |  |  |  |  |  |  |  |  |  |  |  |  |  |  | *scrm-4* | SCRaMblase (phospholipid scramblase) |
|  |  |  |  |  |  |  |  |  |  |  |  |  |  |  |  |  |  |  |  |  |  |  |  |  |  |  |  |  |  |  |  |  |  |  |  |  |  | *alh-11* | ALdehyde deHydrogenase |
|  |  |  |  |  |  |  |  |  |  |  |  |  |  |  |  |  |  |  |  |  |  |  |  |  |  |  |  |  |  |  |  |  |  |  |  |  |  | F42G9.6 |  |
|  |  |  |  |  |  |  |  |  |  |  |  |  |  |  |  |  |  |  |  |  |  |  |  |  |  |  |  |  |  |  |  |  |  |  |  |  |  | F32A7.5 |  |
|  |  |  |  |  |  |  |  |  |  |  |  |  |  |  |  |  |  |  |  |  |  |  |  |  |  |  |  |  |  |  |  |  |  |  |  |  |  | Y57G11C.36 |  |
|  |  |  |  |  |  |  |  |  |  |  |  |  |  |  |  |  |  |  |  |  |  |  |  |  |  |  |  |  |  |  |  |  |  |  |  |  |  | Y39A3CR.9 |  |
|  |  |  |  |  |  |  |  |  |  |  |  |  |  |  |  |  |  |  |  |  |  |  |  |  |  |  |  |  |  |  |  |  |  |  |  |  |  | *mog-3* | Masculinisation Of Germline |
|  |  |  |  |  |  |  |  |  |  |  |  |  |  |  |  |  |  |  |  |  |  |  |  |  |  |  |  |  |  |  |  |  |  |  |  |  |  | *ceh-39* | C. Elegans Homeobox |
|  |  |  |  |  |  |  |  |  |  |  |  |  |  |  |  |  |  |  |  |  |  |  |  |  |  |  |  |  |  |  |  |  |  |  |  |  |  | *nxf-1* | Nuclear eXport Factor |
|  |  |  |  |  |  |  |  |  |  |  |  |  |  |  |  |  |  |  |  |  |  |  |  |  |  |  |  |  |  |  |  |  |  |  |  |  |  | *mys-2* | MYST family histone acetyltransferase-like |
|  |  |  |  |  |  |  |  |  |  |  |  |  |  |  |  |  |  |  |  |  |  |  |  |  |  |  |  |  |  |  |  |  |  |  |  |  |  | *mes-3* | Maternal Effect Sterile |
|  |  |  |  |  |  |  |  |  |  |  |  |  |  |  |  |  |  |  |  |  |  |  |  |  |  |  |  |  |  |  |  |  |  |  |  |  |  | *vps-18* | related to yeast Vacuolar Protein Sorting factor |
|  |  |  |  |  |  |  |  |  |  |  |  |  |  |  |  |  |  |  |  |  |  |  |  |  |  |  |  |  |  |  |  |  |  |  |  |  |  | *klp-16* | Kinesin-Like Protein |
|  |  |  |  |  |  |  |  |  |  |  |  |  |  |  |  |  |  |  |  |  |  |  |  |  |  |  |  |  |  |  |  |  |  |  |  |  |  | C37C3.1 |  |
|  |  |  |  |  |  |  |  |  |  |  |  |  |  |  |  |  |  |  |  |  |  |  |  |  |  |  |  |  |  |  |  |  |  |  |  |  |  | *dpf-5* | Dipeptidyl Peptidase Four (IV) family |
|  |  |  |  |  |  |  |  |  |  |  |  |  |  |  |  |  |  |  |  |  |  |  |  |  |  |  |  |  |  |  |  |  |  |  |  |  |  | F56H6.2 |  |
|  |  |  |  |  |  |  |  |  |  |  |  |  |  |  |  |  |  |  |  |  |  |  |  |  |  |  |  |  |  |  |  |  |  |  |  |  |  | Y73B3A.3 |  |
|  |  |  |  |  |  |  |  |  |  |  |  |  |  |  |  |  |  |  |  |  |  |  |  |  |  |  |  |  |  |  |  |  |  |  |  |  |  | F46H5.2 |  |
|  |  |  |  |  |  |  |  |  |  |  |  |  |  |  |  |  |  |  |  |  |  |  |  |  |  |  |  |  |  |  |  |  |  |  |  |  |  | *ldb-1* | LIM Domain Binding protein |
|  |  |  |  |  |  |  |  |  |  |  |  |  |  |  |  |  |  |  |  |  |  |  |  |  |  |  |  |  |  |  |  |  |  |  |  |  |  | *itsn-1* | ITSN (intersectin) family |
|  |  |  |  |  |  |  |  |  |  |  |  |  |  |  |  |  |  |  |  |  |  |  |  |  |  |  |  |  |  |  |  |  |  |  |  |  |  | *apd-3* | AdaPtin, Delta chain (clathrin associated complex) |
|  |  |  |  |  |  |  |  |  |  |  |  |  |  |  |  |  |  |  |  |  |  |  |  |  |  |  |  |  |  |  |  |  |  |  |  |  |  | *cnk-1* | Connector/eNhancer of KSR |
|  |  |  |  |  |  |  |  |  |  |  |  |  |  |  |  |  |  |  |  |  |  |  |  |  |  |  |  |  |  |  |  |  |  |  |  |  |  | *spk-1* | SR Protein Kinase |
|  |  |  |  |  |  |  |  |  |  |  |  |  |  |  |  |  |  |  |  |  |  |  |  |  |  |  |  |  |  |  |  |  |  |  |  |  |  | F25B3.6 |  |
|  |  |  |  |  |  |  |  |  |  |  |  |  |  |  |  |  |  |  |  |  |  |  |  |  |  |  |  |  |  |  |  |  |  |  |  |  |  | *num-1* | NUMb related |
|  |  |  |  |  |  |  |  |  |  |  |  |  |  |  |  |  |  |  |  |  |  |  |  |  |  |  |  |  |  |  |  |  |  |  |  |  |  | K05C4.9 |  |
|  |  |  |  |  |  |  |  |  |  |  |  |  |  |  |  |  |  |  |  |  |  |  |  |  |  |  |  |  |  |  |  |  |  |  |  |  |  | *ufd-2* | Ubiquitin Fusion Degradation (yeast UFD homolog) |
|  |  |  |  |  |  |  |  |  |  |  |  |  |  |  |  |  |  |  |  |  |  |  |  |  |  |  |  |  |  |  |  |  |  |  |  |  |  | F19C6.2 |  |
|  |  |  |  |  |  |  |  |  |  |  |  |  |  |  |  |  |  |  |  |  |  |  |  |  |  |  |  |  |  |  |  |  |  |  |  |  |  | W05H9.2 |  |
|  |  |  |  |  |  |  |  |  |  |  |  |  |  |  |  |  |  |  |  |  |  |  |  |  |  |  |  |  |  |  |  |  |  |  |  |  |  | *math-33* | MATH (meprin-associated Traf homology) domain containing |
|  |  |  |  |  |  |  |  |  |  |  |  |  |  |  |  |  |  |  |  |  |  |  |  |  |  |  |  |  |  |  |  |  |  |  |  |  |  | *utx-1* | human UTX (Ubiquitously transcribed TPR on X) homolog |
|  |  |  |  |  |  |  |  |  |  |  |  |  |  |  |  |  |  |  |  |  |  |  |  |  |  |  |  |  |  |  |  |  |  |  |  |  |  | *hpo-10* | Hypersensitive to POre-forming toxin |
|  |  |  |  |  |  |  |  |  |  |  |  |  |  |  |  |  |  |  |  |  |  |  |  |  |  |  |  |  |  |  |  |  |  |  |  |  |  | *pqe-1* | PolyQ (poly glutamine tract) toxicity Enhancer |
|  |  |  |  |  |  |  |  |  |  |  |  |  |  |  |  |  |  |  |  |  |  |  |  |  |  |  |  |  |  |  |  |  |  |  |  |  |  | *ric-8* | Resistance to Inhibitors of Cholinesterase |
|  |  |  |  |  |  |  |  |  |  |  |  |  |  |  |  |  |  |  |  |  |  |  |  |  |  |  |  |  |  |  |  |  |  |  |  |  |  | *clh-5* | CLC-type chloride cHannel |
|  |  |  |  |  |  |  |  |  |  |  |  |  |  |  |  |  |  |  |  |  |  |  |  |  |  |  |  |  |  |  |  |  |  |  |  |  |  | *madf-11* | MADF domain transcription factor |
|  |  |  |  |  |  |  |  |  |  |  |  |  |  |  |  |  |  |  |  |  |  |  |  |  |  |  |  |  |  |  |  |  |  |  |  |  |  | *cfp-1* | CFP1 (CpG-binding protein, CXXC Finger Protein 1) homolog |
|  |  |  |  |  |  |  |  |  |  |  |  |  |  |  |  |  |  |  |  |  |  |  |  |  |  |  |  |  |  |  |  |  |  |  |  |  |  | *kel-3* | KELch-repeat containing protein |
|  |  |  |  |  |  |  |  |  |  |  |  |  |  |  |  |  |  |  |  |  |  |  |  |  |  |  |  |  |  |  |  |  |  |  |  |  |  | *cids-2* | pol II C-terminal Interaction Domain Suppressor |
|  |  |  |  |  |  |  |  |  |  |  |  |  |  |  |  |  |  |  |  |  |  |  |  |  |  |  |  |  |  |  |  |  |  |  |  |  |  | *vps-52* | related to yeast Vacuolar Protein Sorting factor |
|  |  |  |  |  |  |  |  |  |  |  |  |  |  |  |  |  |  |  |  |  |  |  |  |  |  |  |  |  |  |  |  |  |  |  |  |  |  | ZK524.4 |  |
|  |  |  |  |  |  |  |  |  |  |  |  |  |  |  |  |  |  |  |  |  |  |  |  |  |  |  |  |  |  |  |  |  |  |  |  |  |  | *edc-3* | yeast Enhancer of DeCapping homolog |
|  |  |  |  |  |  |  |  |  |  |  |  |  |  |  |  |  |  |  |  |  |  |  |  |  |  |  |  |  |  |  |  |  |  |  |  |  |  | *ceh-38* | C. Elegans Homeobox |
|  |  |  |  |  |  |  |  |  |  |  |  |  |  |  |  |  |  |  |  |  |  |  |  |  |  |  |  |  |  |  |  |  |  |  |  |  |  | *age-1* | AGEing alteration |
|  |  |  |  |  |  |  |  |  |  |  |  |  |  |  |  |  |  |  |  |  |  |  |  |  |  |  |  |  |  |  |  |  |  |  |  |  |  | *pqn-65* | Prion-like-(Q/N-rich)-domain-bearing protein |
|  |  |  |  |  |  |  |  |  |  |  |  |  |  |  |  |  |  |  |  |  |  |  |  |  |  |  |  |  |  |  |  |  |  |  |  |  |  | *wip-1* | Wiskott-Aldrich syndrome protein (WASP)-Interacting Protein and gene assignment |
|  |  |  |  |  |  |  |  |  |  |  |  |  |  |  |  |  |  |  |  |  |  |  |  |  |  |  |  |  |  |  |  |  |  |  |  |  |  | M57.2 |  |
|  |  |  |  |  |  |  |  |  |  |  |  |  |  |  |  |  |  |  |  |  |  |  |  |  |  |  |  |  |  |  |  |  |  |  |  |  |  | Y71F9AL.21 |  |
|  |  |  |  |  |  |  |  |  |  |  |  |  |  |  |  |  |  |  |  |  |  |  |  |  |  |  |  |  |  |  |  |  |  |  |  |  |  | *lst-4* | Lateral Signaling Target |
|  |  |  |  |  |  |  |  |  |  |  |  |  |  |  |  |  |  |  |  |  |  |  |  |  |  |  |  |  |  |  |  |  |  |  |  |  |  | ZC395.4 |  |
|  |  |  |  |  |  |  |  |  |  |  |  |  |  |  |  |  |  |  |  |  |  |  |  |  |  |  |  |  |  |  |  |  |  |  |  |  |  | ZC477.2 |  |
|  |  |  |  |  |  |  |  |  |  |  |  |  |  |  |  |  |  |  |  |  |  |  |  |  |  |  |  |  |  |  |  |  |  |  |  |  |  | F10E9.12 |  |
|  |  |  |  |  |  |  |  |  |  |  |  |  |  |  |  |  |  |  |  |  |  |  |  |  |  |  |  |  |  |  |  |  |  |  |  |  |  | Y20C6A.4 |  |
|  |  |  |  |  |  |  |  |  |  |  |  |  |  |  |  |  |  |  |  |  |  |  |  |  |  |  |  |  |  |  |  |  |  |  |  |  |  | *jac-1* | Juxtamembrane domain-Associated Catenin |
|  |  |  |  |  |  |  |  |  |  |  |  |  |  |  |  |  |  |  |  |  |  |  |  |  |  |  |  |  |  |  |  |  |  |  |  |  |  | *apb-1* |  |
|  |  |  |  |  |  |  |  |  |  |  |  |  |  |  |  |  |  |  |  |  |  |  |  |  |  |  |  |  |  |  |  |  |  |  |  |  |  | Y75B8A.24 |  |
|  |  |  |  |  |  |  |  |  |  |  |  |  |  |  |  |  |  |  |  |  |  |  |  |  |  |  |  |  |  |  |  |  |  |  |  |  |  | *pmr-1* | PMR-type Golgi ATPase |
|  |  |  |  |  |  |  |  |  |  |  |  |  |  |  |  |  |  |  |  |  |  |  |  |  |  |  |  |  |  |  |  |  |  |  |  |  |  | *cdh-1* | CaDHerin family |
|  |  |  |  |  |  |  |  |  |  |  |  |  |  |  |  |  |  |  |  |  |  |  |  |  |  |  |  |  |  |  |  |  |  |  |  |  |  | Y6B3B.9 |  |
|  |  |  |  |  |  |  |  |  |  |  |  |  |  |  |  |  |  |  |  |  |  |  |  |  |  |  |  |  |  |  |  |  |  |  |  |  |  | *mthf-1* | MethyleneTetraHydroFolate reductase |
|  |  |  |  |  |  |  |  |  |  |  |  |  |  |  |  |  |  |  |  |  |  |  |  |  |  |  |  |  |  |  |  |  |  |  |  |  |  | Y17G7B.13 |  |
|  |  |  |  |  |  |  |  |  |  |  |  |  |  |  |  |  |  |  |  |  |  |  |  |  |  |  |  |  |  |  |  |  |  |  |  |  |  | F52D2.6 |  |
|  |  |  |  |  |  |  |  |  |  |  |  |  |  |  |  |  |  |  |  |  |  |  |  |  |  |  |  |  |  |  |  |  |  |  |  |  |  | Y54F10AR.1 |  |
|  |  |  |  |  |  |  |  |  |  |  |  |  |  |  |  |  |  |  |  |  |  |  |  |  |  |  |  |  |  |  |  |  |  |  |  |  |  | C17E4.6 |  |
|  |  |  |  |  |  |  |  |  |  |  |  |  |  |  |  |  |  |  |  |  |  |  |  |  |  |  |  |  |  |  |  |  |  |  |  |  |  | *ife-3* | Initiation Factor 4E (eIF4E) family |
|  |  |  |  |  |  |  |  |  |  |  |  |  |  |  |  |  |  |  |  |  |  |  |  |  |  |  |  |  |  |  |  |  |  |  |  |  |  | *dcn-1* | Defective in Cullin Neddylation |
|  |  |  |  |  |  |  |  |  |  |  |  |  |  |  |  |  |  |  |  |  |  |  |  |  |  |  |  |  |  |  |  |  |  |  |  |  |  | *ceh-93* | C. Elegans Homeobox |
|  |  |  |  |  |  |  |  |  |  |  |  |  |  |  |  |  |  |  |  |  |  |  |  |  |  |  |  |  |  |  |  |  |  |  |  |  |  | *aho-3* | Abnormal Hunger Orientation |
|  |  |  |  |  |  |  |  |  |  |  |  |  |  |  |  |  |  |  |  |  |  |  |  |  |  |  |  |  |  |  |  |  |  |  |  |  |  | *cyy-1* | CYclin Y homolog |
|  |  |  |  |  |  |  |  |  |  |  |  |  |  |  |  |  |  |  |  |  |  |  |  |  |  |  |  |  |  |  |  |  |  |  |  |  |  | *sur-6* | SUppressor of activated let-60 Ras |
|  |  |  |  |  |  |  |  |  |  |  |  |  |  |  |  |  |  |  |  |  |  |  |  |  |  |  |  |  |  |  |  |  |  |  |  |  |  | *scp-1* | SREBP Cleavage activating Protein (SCAP) homolog |
|  |  |  |  |  |  |  |  |  |  |  |  |  |  |  |  |  |  |  |  |  |  |  |  |  |  |  |  |  |  |  |  |  |  |  |  |  |  | *scpl-3* | SCP (Small C-terminal domain Phosphatase)-Like phosphatase |
|  |  |  |  |  |  |  |  |  |  |  |  |  |  |  |  |  |  |  |  |  |  |  |  |  |  |  |  |  |  |  |  |  |  |  |  |  |  | F31D4.2 |  |
|  |  |  |  |  |  |  |  |  |  |  |  |  |  |  |  |  |  |  |  |  |  |  |  |  |  |  |  |  |  |  |  |  |  |  |  |  |  | *ced-12* | CEll Death abnormality |
|  |  |  |  |  |  |  |  |  |  |  |  |  |  |  |  |  |  |  |  |  |  |  |  |  |  |  |  |  |  |  |  |  |  |  |  |  |  | *let-60* | LEThal |
|  |  |  |  |  |  |  |  |  |  |  |  |  |  |  |  |  |  |  |  |  |  |  |  |  |  |  |  |  |  |  |  |  |  |  |  |  |  | *csnk-1* | CaSeiN Kinase |
|  |  |  |  |  |  |  |  |  |  |  |  |  |  |  |  |  |  |  |  |  |  |  |  |  |  |  |  |  |  |  |  |  |  |  |  |  |  | *ubc-25* | UBiquitin Conjugating enzyme |
|  |  |  |  |  |  |  |  |  |  |  |  |  |  |  |  |  |  |  |  |  |  |  |  |  |  |  |  |  |  |  |  |  |  |  |  |  |  | *gpx-2* | Glutathione PeroXidase |
|  |  |  |  |  |  |  |  |  |  |  |  |  |  |  |  |  |  |  |  |  |  |  |  |  |  |  |  |  |  |  |  |  |  |  |  |  |  | Y17G7B.17 |  |
|  |  |  |  |  |  |  |  |  |  |  |  |  |  |  |  |  |  |  |  |  |  |  |  |  |  |  |  |  |  |  |  |  |  |  |  |  |  | *paa-1* | Phosphatase 2A regulatory A subunit |
|  |  |  |  |  |  |  |  |  |  |  |  |  |  |  |  |  |  |  |  |  |  |  |  |  |  |  |  |  |  |  |  |  |  |  |  |  |  | *let-92* | LEThal |
|  |  |  |  |  |  |  |  |  |  |  |  |  |  |  |  |  |  |  |  |  |  |  |  |  |  |  |  |  |  |  |  |  |  |  |  |  |  | Y48E1B.3 |  |
|  |  |  |  |  |  |  |  |  |  |  |  |  |  |  |  |  |  |  |  |  |  |  |  |  |  |  |  |  |  |  |  |  |  |  |  |  |  | *src-1* | SRC oncogene related |
|  |  |  |  |  |  |  |  |  |  |  |  |  |  |  |  |  |  |  |  |  |  |  |  |  |  |  |  |  |  |  |  |  |  |  |  |  |  | *rho-1* | RHO (small G protein) family |
|  |  |  |  |  |  |  |  |  |  |  |  |  |  |  |  |  |  |  |  |  |  |  |  |  |  |  |  |  |  |  |  |  |  |  |  |  |  | *rpn-10* | proteasome Regulatory Particle, Non-ATPase-like |
|  |  |  |  |  |  |  |  |  |  |  |  |  |  |  |  |  |  |  |  |  |  |  |  |  |  |  |  |  |  |  |  |  |  |  |  |  |  | F52C12.1 |  |
|  |  |  |  |  |  |  |  |  |  |  |  |  |  |  |  |  |  |  |  |  |  |  |  |  |  |  |  |  |  |  |  |  |  |  |  |  |  | C38C10.2 |  |
|  |  |  |  |  |  |  |  |  |  |  |  |  |  |  |  |  |  |  |  |  |  |  |  |  |  |  |  |  |  |  |  |  |  |  |  |  |  | *lap-2* | Leucine AminoPeptidase |

### Phenotypes enriched

|  |  |  |  |
| --- | --- | --- | --- |
| **Group name** | **Number in cluster** | **Enrichment** | **FDR corrected p** |
| physiology variant | 64 | 1.87 | 0.000469 |
| cell development variant | 24 | 2.96 | 0.003260 |
| fertility variant | 43 | 2.07 | 0.004810 |
| reproductive system physiology variant | 43 | 2.07 | 0.004960 |
| Variant | 74 | 1.63 | 0.006100 |
| coelomocyte physiology variant | 5 | 19.45 | 0.006150 |
| coelomocyte uptake defective | 5 | 19.45 | 0.006150 |
| organ system physiology variant | 43 | 2.02 | 0.007940 |
| vulva development variant (RNAi) | 13 | 4.51 | 0.009330 |
| dead eggs laid | 4 | 28.01 | 0.010700 |
| cell death variant | 9 | 6.56 | 0.012200 |
| reproductive system development variant (RNAi) | 15 | 3.82 | 0.013000 |
| endocytic transport variant | 6 | 11.67 | 0.013900 |
| cell fate specification variant (RNAi) | 13 | 4.25 | 0.016000 |
| vulval cell induction variant (RNAi) | 12 | 4.47 | 0.020100 |
| development variant | 57 | 1.73 | 0.020400 |
| vulva cell fate specification variant (RNAi) | 12 | 4.33 | 0.026200 |
| sterile | 38 | 2.00 | 0.030700 |
| gene expression variant | 18 | 3.03 | 0.033700 |
| fertility reduced | 38 | 1.99 | 0.035100 |
| vulvaless (RNAi) | 5 | 12.97 | 0.038200 |
| egg laying defective | 11 | 4.48 | 0.039700 |
| apoptosis variant | 7 | 7.54 | 0.040500 |
| vulval cell induction reduced (RNAi) | 5 | 12.50 | 0.044300 |
| cell physiology variant | 18 | 2.95 | 0.044500 |
| engulfment variant | 4 | 18.67 | 0.049500 |

### Anatomy terms enriched

|  |  |  |  |
| --- | --- | --- | --- |
| **Group name** | **Number in cluster** | **Enrichment** | **FDR corrected p** |
| reproductive system | 46 | 1.98 | 0.00971 |
| pharynx | 43 | 1.92 | 0.03380 |
| alimentary system | 64 | 1.64 | 0.03900 |
| hermaphrodite gonad | 26 | 2.39 | 0.04880 |
| body wall musculature | 33 | 2.11 | 0.04900 |
| muscular system | 43 | 1.87 | 0.04950 |
| muscle cell | 35 | 2.05 | 0.04970 |

### GO terms enriched

|  |  |  |
| --- | --- | --- |
| **GO term** | **Number of genes** | **FDR-corrected p-value** |
| regulation of nematode larval development | 15 | 0.0016 |
| localization of cell | 15 | 0.0039 |
| regulation of multicellular organismal development | 16 | 0.0071 |
| positive regulation of vulval development | 6 | 0.0120 |
| protein modification process | 23 | 0.0160 |
| small GTPase regulator activity | 6 | 0.0190 |
| nucleoside-triphosphatase regulator activity | 7 | 0.0340 |
| cell migration | 12 | 0.0380 |
| post-embryonic development | 44 | 0.0380 |
| single-organism membrane organization | 5 | 0.0430 |
| purine nucleoside triphosphate catabolic process | 12 | 0.0450 |
| ribonucleoside triphosphate catabolic process | 12 | 0.0450 |
| ribonucleotide catabolic process | 12 | 0.0500 |
| purine ribonucleoside catabolic process | 12 | 0.0500 |

### Expression clusters enriched

|  |  |  |  |
| --- | --- | --- | --- |
| **Group name** | **Number in cluster** | **Enrichment** | **FDR corrected p** |
| FBF-associated probe sets (FDR <2.25%) | 109 | 2.03 | 7.71e-13 |
| Genes with expression enriched in PVD and OLL neurons. Data sets were normalized by RMA and transcripts showing relative PVD enrichment (>= 1.5X) vs. the reference sample were identified by SAM analysis (False Discovery Rate, FDR < 1%). | 77 | 2.47 | 5.48e-12 |
| A complete list of the genes that showed differential expression in a slr-2 mutant strain. | 62 | 2.53 | 2.09e-09 |
| Genes expressed in embryonic motor neurons (identified by unc-4::GFP expressing cells). | 130 | 1.50 | 1.39e-06 |
| Potental DAF-12 target genes identified by ChIP-chip analysis performed on strain ALF4 [daf-12 | 80 | 1.79 | 1.17e-05 |
| C-lineage related expression profile. WBPaper00025032:cluster\_54 | 7 | 18.85 | 2.71e-05 |
| Caenorhabditis elegans Genes with expression levels changed significantly after treatment of Xenorhabdus nematophila. | 139 | 1.40 | 3.50e-05 |
| Genes down-regulated after 300 um Tannic acid treatment. Fold change < 0.8. | 48 | 2.18 | 6.96e-05 |
| Genes predicted to be upregulated more than 2.0 fold in (AFD+AWB) datasets as compared to unsorted whole embryonic cells dataset. | 31 | 2.69 | 2.03e-04 |
| Genes with differeiential expression after exposed to Au-NP. | 28 | 2.56 | 1.59e-03 |
| mixed oogenesis/somatic | 24 | 2.66 | 3.88e-03 |
| RNP-8-associated transcripts, based on microarray experiments. | 29 | 2.33 | 5.58e-03 |
| Gene significantly down-regulated by treatment with 0.2mM of HuminFeed Hydroquinone until young adult stage (3 days), with a minimum fold change in gene expression of 0.8. | 8 | 6.59 | 7.93e-03 |
| C-lineage related expression profile. WBPaper00025032:cluster\_28 | 6 | 8.94 | 1.35e-02 |
| Embryonic transient (ET) subclasses are based on time of max abundance. [cgc5767]:expression\_class\_ET\_max(143\_min) | 9 | 5.00 | 2.01e-02 |
| Significantly downregulated genes from cyc-1(RNAi) microarrays using SAM algorithm with an FDR < 0.1 from adult-only chips. | 72 | 1.51 | 2.19e-02 |
| Genes downregulated more than 2 fold after 24 hours of AgNPs exposure. | 31 | 2.05 | 2.58e-02 |
| Genes differentially expressed under EtBr treatment without UVC exposure vs after UVC exposure but without EtBr treatment at the -1h timepoint (just prior to the third UVC dose (48h)). | 68 | 1.51 | 3.41e-02 |
| Genes downregulated (fold changes < 2-fold) in the worms following exposure of a-SWCNTs (500 mg\\/mL) for 48 hr. | 45 | 1.73 | 3.62e-02 |
| Embryonic (E) subclasses are based on the earliest significant increase(abbreviated pi for primary increase). [cgc5767]:expression\_class\_E\_pi(23\_min) | 27 | 2.09 | 4.80e-02 |

### Motifs enriched

|  |  |  |  |  |  |
| --- | --- | --- | --- | --- | --- |
| **Motif** | **Logo** | **Possible orthologs** | **Number of motifs in cluster** | **Enrichment** | **FDR corrected p** |
| ONECUT1\_2 |  | ceh-48 (0.67) dsc-1 | 76 | 2.46 | 3.7e-11 |
| MA0543.1 |  | eor-1 (0.82) daf-8 | 118 | 1.79 | 5.0e-10 |
| pTH8982 |  | ceh-48 (0.67) | 82 | 2.17 | 1.4e-09 |
| pTH5260 |  | aha-1 (0.84) hlh-27 lin-22 hlh-28 | 95 | 1.94 | 4.7e-09 |
| MA0536.1 |  | elt-1 | 41 | 3.27 | 1.9e-08 |
| Zfp161\_2858 |  | pzf-1 | 75 | 2.05 | 1.7e-07 |
| pTH5102 |  | aha-1 (0.84) mxl-2 (-0.68) mxl-1 (-0.55) hlh-30 hlh-26 lin-22 ref-1 | 86 | 1.84 | 8.2e-07 |
| Mv90 |  | mef-2 | 137 | 1.48 | 1.2e-06 |
| pTH10696 |  | Y44A6D.3 | 90 | 1.75 | 2.8e-06 |
| MA0537.1 |  | blmp-1 | 156 | 1.36 | 7.8e-06 |
| FOXJ3\_3 |  | daf-16 (0.83) fkh-7 let-381 pha-4 fkh-8 lin-31 | 164 | 1.32 | 1.0e-05 |
| FOXL1\_2 |  | let-381 lin-31 | 165 | 1.32 | 1.1e-05 |
| pTH9254 |  | mel-28 (0.53) | 148 | 1.37 | 2.4e-05 |
| POU3F1\_2 |  | unc-86 ceh-18 | 155 | 1.34 | 2.5e-05 |
| MA0117.1 |  | D1081.8 (0.68) F45H11.6 | 22 | 3.88 | 2.7e-05 |
| pTH9214 |  | cfi-1 | 166 | 1.30 | 3.1e-05 |
| HIF1A\_si |  | hif-1 (0.68) | 41 | 2.45 | 3.1e-05 |
| ARI3A\_do |  | cfi-1 | 150 | 1.35 | 4.5e-05 |
| YNL068C\_830 |  | daf-16 (0.83) fkh-7 fkh-10 fkh-8 lin-31 | 157 | 1.31 | 7.6e-05 |
| MA0538.1 |  | ztf-3 (0.71) hif-1 (0.68) Y5F2A.4 (0.59) daf-12 | 106 | 1.52 | 7.8e-05 |
| CG2052\_SANGER\_2.5\_FBgn0039905 |  | mel-28 (0.53) fkh-7 lin-29 | 94 | 1.59 | 9.5e-05 |
| MA0037.2 |  | elt-1 ztf-29 | 141 | 1.36 | 1.1e-04 |
| pTH5916 |  | efl-2 (0.65) | 69 | 1.79 | 1.3e-04 |
| Atf1\_3026 |  | crh-1 (0.67) | 108 | 1.49 | 1.4e-04 |
| Antp\_FlyReg\_FBgn0000095 |  | let-381 hmbx-1 hmg-12 lin-31 lin-39 Y116A8C.22 | 128 | 1.39 | 2.1e-04 |
| pTH9173 |  | efl-2 (0.65) | 74 | 1.70 | 2.6e-04 |
| K562\_ZBTB7A\_HudsonAlpha |  | ZC328.2 | 28 | 2.77 | 2.8e-04 |
| pTH9180 |  | mel-28 (0.53) let-381 Y61A9LA.9 Y116A8C.22 | 153 | 1.30 | 3.2e-04 |
| pnr\_SANGER\_5\_FBgn0003117 |  | elt-1 | 154 | 1.29 | 3.6e-04 |
| HXC6\_f1 |  | lin-1 nhr-100 lin-39 | 161 | 1.27 | 4.0e-04 |
| exd\_FlyReg\_FBgn0000611 |  | let-381 ceh-20 | 166 | 1.25 | 4.0e-04 |
| pTH8863 |  | hmg-12 | 120 | 1.40 | 4.4e-04 |
| V$POU3F2\_01 |  | dmd-3 ceh-18 | 200 | 1.16 | 4.6e-04 |
| pTH9097 |  | Y116A8C.22 | 154 | 1.29 | 4.8e-04 |
| CXXC1\_si |  | F52B11.1 | 103 | 1.48 | 5.0e-04 |
| ZNF75A\_1 |  | ztf-3 (0.71) lag-1 (0.62) F26F4.8 | 172 | 1.23 | 5.5e-04 |
| Ara\_Cell\_FBgn0015904 |  | daf-16 (0.83) irx-1 fkh-9 | 82 | 1.58 | 7.0e-04 |
| pTH5465 |  | aha-1 (0.84) hlh-30 | 84 | 1.57 | 7.1e-04 |
| V$GATA1\_05 |  | elt-1 elt-3 | 124 | 1.36 | 1.3e-03 |
| KLF6\_si |  | klf-1 ZC328.2 | 95 | 1.48 | 1.3e-03 |
| E2F4\_1 |  | nfi-1 (0.79) F49E12.6 | 96 | 1.47 | 1.4e-03 |
| HXD4\_f1 |  | cog-1 lin-39 | 147 | 1.28 | 1.5e-03 |
| HeLa-S3\_RFX5\_Stanford |  | daf-19 (0.65) | 62 | 1.71 | 1.5e-03 |
| rn\_SOLEXA\_5\_FBgn0259172 |  | lin-29 | 107 | 1.42 | 1.5e-03 |
| MA0049.1 |  | hbl-1 (0.77) php-3 | 172 | 1.21 | 1.5e-03 |
| pTH9044 |  | F26F4.8 mbr-1 bed-3 nhr-177 | 93 | 1.48 | 1.6e-03 |
| ELF3\_2 |  | C24A1.2 | 126 | 1.34 | 1.6e-03 |
| MA0076.2 |  | lin-1 C24A1.2 | 132 | 1.32 | 1.8e-03 |
| pTH9380 |  | mel-28 (0.53) | 123 | 1.35 | 1.8e-03 |
| SP1\_f2 |  | klf-2 | 110 | 1.39 | 2.1e-03 |
| pTH9237 |  | mel-28 (0.53) | 88 | 1.49 | 2.3e-03 |
| ZBTB49\_1 |  | C46E10.9 | 48 | 1.85 | 2.7e-03 |
| STF1\_f1 |  | nhr-68 | 61 | 1.68 | 2.8e-03 |
| pTH9335 |  | mel-28 (0.53) | 137 | 1.29 | 3.0e-03 |
| Tbp\_pr781 |  | tbp-1 | 135 | 1.30 | 3.0e-03 |
| Hoxc10\_2 |  | pal-1 php-3 lin-39 | 150 | 1.26 | 3.1e-03 |
| pTH10798 |  | Y75B8A.6 | 82 | 1.51 | 3.3e-03 |
| pTH10797 |  | K11D2.4 lin-29 | 96 | 1.43 | 3.8e-03 |
| NHLH1\_1 |  | hlh-15 | 98 | 1.42 | 3.8e-03 |
| V$FAC1\_01 |  | gei-8 (0.8) | 145 | 1.26 | 3.9e-03 |
| MA0600.1 |  | daf-19 (0.65) | 83 | 1.49 | 4.4e-03 |
| MA0102.3 |  | C48E7.11 | 76 | 1.53 | 4.5e-03 |
| MA0547.1 |  | skn-1 (0.76) | 116 | 1.34 | 4.9e-03 |
| MA0544.1 |  | gei-11 | 101 | 1.40 | 5.0e-03 |
| FLI1\_f1 |  | lin-1 | 106 | 1.38 | 5.0e-03 |
| pTH9260 |  | mel-28 (0.53) | 148 | 1.25 | 5.0e-03 |
| MA0135.1 |  | lim-7 | 133 | 1.29 | 5.0e-03 |
| pTH9298 |  | crh-1 (0.67) attf-1 | 63 | 1.62 | 5.1e-03 |
| pTH5166 |  | C48E7.11 | 58 | 1.67 | 5.1e-03 |
| ARI3A\_f1 |  | alr-1 cfi-1 ZC204.2 | 121 | 1.32 | 5.2e-03 |
| V$CDPCR1\_01 |  | ceh-48 (0.67) | 82 | 1.49 | 5.4e-03 |
| CG31670\_SOLEXA\_5\_FBgn0031375 |  | CELE\_Y38H8A.5 | 157 | 1.23 | 5.4e-03 |
| pTH9242 |  | mel-28 (0.53) | 151 | 1.24 | 5.7e-03 |
| V$AHR\_01 |  | ahr-1 | 27 | 2.31 | 5.7e-03 |
| MA0457.1 |  | alr-1 lim-6 egl-5 pha-2 ceh-18 | 123 | 1.32 | 5.8e-03 |
| cad\_FlyReg\_FBgn0000251 |  | ceh-13 T27F2.4 | 145 | 1.25 | 5.8e-03 |
| V$ZID\_01 |  | ztf-28 | 57 | 1.67 | 6.2e-03 |
| pTH6497 |  | lin-31 | 113 | 1.34 | 6.5e-03 |
| MA0473.1 |  | lin-1 C24A1.2 | 92 | 1.42 | 6.6e-03 |
| SP4\_f1 |  | Y53H1A.2 klf-2 | 70 | 1.55 | 6.7e-03 |
| Rfxdc2\_3516 |  | daf-19 (0.65) | 65 | 1.58 | 6.9e-03 |
| Sox17\_2837 |  | sox-4 | 140 | 1.26 | 7.0e-03 |
| pTH8745 |  | attf-1 | 19 | 2.79 | 7.1e-03 |
| POU3F3\_1 |  | sox-4 ceh-18 tbp-1 | 148 | 1.24 | 7.1e-03 |
| HXC8\_f1 |  | lin-39 | 133 | 1.28 | 7.4e-03 |
| pTH8333 |  | ZC416.1 | 83 | 1.46 | 8.0e-03 |
| pTH9177 |  | F10B5.3 | 107 | 1.36 | 8.1e-03 |
| ETS2\_f1 |  | lin-1 C24A1.2 | 79 | 1.48 | 8.2e-03 |
| pTH7875 |  | mel-28 (0.53) | 212 | 1.09 | 8.6e-03 |
| pTH3796 |  | let-381 lin-31 | 139 | 1.26 | 8.7e-03 |
| BARX1\_1 |  | ceh-43 ceh-31 | 137 | 1.26 | 8.7e-03 |
| Tcf7\_0950 |  | pop-1 | 149 | 1.23 | 9.0e-03 |
| V$PAX5\_01 |  | daf-8 pax-2 | 19 | 2.73 | 9.0e-03 |
| PITX1\_3 |  | ceh-36 alr-1 ceh-45 ceh-53 | 18 | 2.82 | 9.1e-03 |
| SPDEF\_2 |  | lin-1 | 51 | 1.70 | 9.5e-03 |
| pTH5337 |  | ZC328.2 | 91 | 1.41 | 1.1e-02 |
| K562\_SP2\_HudsonAlpha |  | klf-2 | 80 | 1.46 | 1.1e-02 |
| pTH8983 |  | tag-347 | 138 | 1.25 | 1.1e-02 |
| Elf3\_3876 |  | C24A1.2 | 136 | 1.26 | 1.2e-02 |
| pTH9082 |  | mab-23 | 118 | 1.31 | 1.2e-02 |
| MA0541.1 |  | efl-1 (0.66) F49E12.6 | 103 | 1.36 | 1.2e-02 |
| pTH9096 |  | T07C12.11 | 78 | 1.46 | 1.3e-02 |
| pTH9951 |  | mex-6 | 135 | 1.26 | 1.3e-02 |
| Elf5 |  | C24A1.2 | 130 | 1.27 | 1.3e-02 |
| Foxk1\_1 |  | lin-31 | 99 | 1.37 | 1.3e-02 |
| ZBT7A\_f1 |  | ZC328.2 | 19 | 2.62 | 1.3e-02 |
| Mv109 |  | pax-2 | 86 | 1.42 | 1.3e-02 |
| NR2F6\_f1 |  | nhr-2 | 133 | 1.26 | 1.3e-02 |
| MA0452.2 |  | B0310.2 | 130 | 1.27 | 1.4e-02 |
| V$FOXJ2\_02 |  | lin-31 | 86 | 1.42 | 1.4e-02 |
| pTH9384 |  | cfi-1 | 146 | 1.23 | 1.4e-02 |
| pTH10650 |  | nhr-153 | 61 | 1.57 | 1.4e-02 |
| pTH2280 |  | mnm-2 | 70 | 1.50 | 1.5e-02 |
| SMAD3\_f1 |  | daf-8 | 27 | 2.12 | 1.7e-02 |
| pTH2933 |  | F58G1.2 (0.55) | 36 | 1.87 | 1.7e-02 |
| pTH9245 |  | ceh-18 | 117 | 1.30 | 1.7e-02 |
| pTH8985 |  | athp-1 (0.71) | 126 | 1.27 | 1.7e-02 |
| V$TATA\_C |  | tbp-1 | 137 | 1.24 | 1.8e-02 |
| MA0146.2 |  | F58G1.2 (0.55) | 64 | 1.53 | 1.9e-02 |
| pTH9149 |  | ztf-30 | 11 | 3.69 | 1.9e-02 |
| V$E47\_01 |  | hlh-2 | 60 | 1.55 | 1.9e-02 |
| pTH3046 |  | Y116A8C.22 | 112 | 1.31 | 2.0e-02 |
| pTH1294 |  | mel-28 (0.53) | 92 | 1.37 | 2.0e-02 |
| CEBPE\_f1 |  | C48E7.11 | 71 | 1.47 | 2.0e-02 |
| pTH10031 |  | mbr-1 | 22 | 2.31 | 2.1e-02 |
| MSX2\_f1 |  | alr-1 ceh-1 lin-39 | 113 | 1.30 | 2.1e-02 |
| Jundm2\_0911 |  | fos-1 | 56 | 1.58 | 2.1e-02 |
| OLIG3\_1 |  | hlh-32 | 94 | 1.36 | 2.2e-02 |
| ATF1\_si |  | crh-1 (0.67) ceh-26 | 100 | 1.34 | 2.2e-02 |
| Klf12\_1 |  | klf-1 klf-2 | 92 | 1.37 | 2.2e-02 |
| pTH3064 |  | crh-1 (0.67) | 32 | 1.92 | 2.3e-02 |
| pTH5119 |  | cfi-1 | 135 | 1.24 | 2.4e-02 |
| pTH8649 |  | mbr-1 | 129 | 1.25 | 2.4e-02 |
| pTH9928 |  | sknr-1 jun-1 fos-1 | 47 | 1.66 | 2.5e-02 |
| pTH8679 |  | pax-2 | 84 | 1.39 | 2.6e-02 |
| Barhl1\_1 |  | ceh-31 | 78 | 1.42 | 2.7e-02 |
| I$ELF1\_01 |  | grh-1 (-0.6) | 112 | 1.29 | 2.8e-02 |
| pTH9911 |  | crh-1 (0.67) atf-5 | 82 | 1.40 | 2.8e-02 |
| pTH9026 |  | attf-1 | 28 | 2.00 | 2.8e-02 |
| YER148W\_798 |  | tbp-1 | 32 | 1.89 | 3.0e-02 |
| V$CDPCR3\_01 |  | ceh-48 (0.67) | 87 | 1.37 | 3.1e-02 |
| Gmeb1\_1745 |  | attf-1 | 17 | 2.55 | 3.1e-02 |
| ESRRA\_3 |  | nhr-71 | 85 | 1.38 | 3.2e-02 |
| pTH8318 |  | attf-1 | 14 | 2.85 | 3.3e-02 |
| pTH9220 |  | mbr-1 | 129 | 1.24 | 3.4e-02 |
| pTH9137 |  | nhr-65 | 153 | 1.19 | 3.4e-02 |
| MA0163.1 |  | Y53H1A.2 C09F5.3 | 46 | 1.64 | 3.4e-02 |
| TBX1\_1 |  | mab-9 | 47 | 1.63 | 3.4e-02 |
| pTH10030 |  | xbp-1 | 48 | 1.61 | 3.5e-02 |
| MAX\_1 |  | mxl-1 (-0.55) | 73 | 1.43 | 3.5e-02 |
| MA0467.1 |  | ceh-45 | 85 | 1.37 | 3.7e-02 |
| pTH10837 |  | T22H9.4 | 141 | 1.21 | 3.7e-02 |
| V$CREB\_02 |  | crh-1 (0.67) | 45 | 1.64 | 3.8e-02 |
| HES1\_f1 |  | lin-22 | 46 | 1.63 | 3.8e-02 |
| Fli1 |  | lin-1 | 112 | 1.28 | 3.8e-02 |
| Elf3 |  | C24A1.2 | 130 | 1.23 | 3.9e-02 |
| HXD10\_f1 |  | nhr-2 php-3 | 148 | 1.20 | 4.1e-02 |
| V$OCT1\_06 |  | ceh-18 | 143 | 1.21 | 4.1e-02 |
| pTH8556 |  | pax-2 | 72 | 1.42 | 4.3e-02 |
| V$FREAC7\_01 |  | lin-31 | 136 | 1.22 | 4.3e-02 |
| V$TAXCREB\_02 |  | crh-1 (0.67) | 93 | 1.33 | 4.4e-02 |
| TBX2\_f1 |  | tbx-39 | 128 | 1.23 | 4.5e-02 |
| pTH6003 |  | nhr-134 | 114 | 1.27 | 4.6e-02 |
| HSFY2\_1 |  | hsf-1 (0.67) | 109 | 1.28 | 4.6e-02 |
| pTH9135 |  | pop-1 | 137 | 1.21 | 4.6e-02 |
| pTH9125 |  | egl-13 (0.68) | 144 | 1.20 | 4.7e-02 |
| V$PAX2\_02 |  | pax-1 | 23 | 2.08 | 4.7e-02 |
| V$AREB6\_04 |  | ztf-6 (0.67) | 68 | 1.43 | 4.7e-02 |
| EMX1\_2 |  | ceh-2 | 207 | 1.08 | 4.8e-02 |
| pTH5914 |  | attf-1 | 72 | 1.41 | 4.8e-02 |
| pTH9164 |  | ceh-26 | 111 | 1.27 | 4.8e-02 |
| pTH5257 |  | C48E7.11 | 91 | 1.33 | 4.9e-02 |
| V$AP4\_01 |  | hlh-11 | 81 | 1.37 | 4.9e-02 |
| pTH9974 |  | hlh-16 | 107 | 1.28 | 5.0e-02 |

### Correlated (and anti-correlated) transcription factors

|  |  |
| --- | --- |
| **Transcription factor** | **Correlation** |
| chd-7 | 0.94 |
| set-16 | 0.92 |
| B0261.1 | 0.90 |
| ceh-38 | 0.90 |
| let-526 | 0.90 |
| athp-2 | 0.89 |
| miz-1 | 0.89 |
| tag-146 | 0.89 |
| gei-17 | 0.89 |
| rbr-2 | 0.87 |
| F57A8.1 | 0.87 |
| T10D4.6 | 0.87 |
| ZK546.5 | 0.86 |
| nfyb-1 | 0.86 |
| mep-1 | 0.86 |
| sta-1 | 0.85 |
| snpc-4 | 0.85 |
| K10B3.5 | 0.84 |
| aha-1 | 0.84 |
| F39B2.1 | 0.83 |
| ztf-8 | 0.83 |
| daf-16 | 0.83 |
| Y48G8AL.10 | 0.83 |
| F21A10.2 | 0.83 |
| lin-13 | 0.82 |
| C02F12.5 | -0.41 |
| nhr-207 | -0.43 |
| nhr-168 | -0.44 |
| mbf-1 | -0.44 |
| nhr-90 | -0.45 |
| C35D6.4 | -0.47 |
| gmeb-3 | -0.48 |
| nhr-122 | -0.48 |
| madf-1 | -0.48 |
| C01F6.9 | -0.52 |
| mxl-3 | -0.53 |
| ceh-82 | -0.53 |
| nhr-222 | -0.54 |
| sdz-38 | -0.54 |
| mxl-1 | -0.55 |
| nhr-87 | -0.58 |
| atf-8 | -0.58 |
| nhr-92 | -0.58 |
| T26A5.8 | -0.59 |
| zip-6 | -0.60 |
| grh-1 | -0.60 |
| hlh-12 | -0.62 |
| ceh-7 | -0.68 |
| mxl-2 | -0.68 |
| Y56A3A.18 | -0.77 |

### ChIP peaks enriched

|  |  |  |  |  |
| --- | --- | --- | --- | --- |
| **Gene** | **Experiment** | **Number of upstream peaks** | **Enrichment** | **FDR corrected p** |
| ces-1 | CES-1\_Embryos | 124 | 2.49 | 2.5e-24 |
| nfya-1 | NFYA-1\_Larvae-L3-stage | 107 | 2.75 | 3.9e-23 |
| lsy-2 | LSY-2\_Embryos | 100 | 2.91 | 6.6e-23 |
| gei-11 | GEI-11\_Larvae-L3-stage | 121 | 2.45 | 7.4e-23 |
| C34F6.9 | C34F6.9\_Larvae-L2-stage | 121 | 2.45 | 8.0e-23 |
| ceh-38 | CEH-38\_Larvae-L4-stage | 83 | 3.46 | 9.3e-23 |
| lin-35 | LIN-35\_Fed-L1-stage-larvae | 112 | 2.55 | 8.5e-22 |
| gei-11 | GEI-11\_Fed-L1-stage-larvae | 117 | 2.45 | 1.1e-21 |
| ham-1 | HAM-1\_Larvae-L4-stage | 121 | 2.36 | 1.9e-21 |
| sea-2 | SEA-2\_Larvae-L3-stage | 59 | 4.68 | 2.7e-21 |
| dpl-1 | DPL-1\_Larvae-L4-stage | 141 | 2.07 | 3.9e-21 |
| pes-1 | PES-1\_Larvae-L4-stage | 121 | 2.34 | 4.0e-21 |
| nfya-1 | NFYA-1\_Late-Embryos | 113 | 2.47 | 5.8e-21 |
| gei-11 | GEI-11\_Larvae-L2-stage | 84 | 3.18 | 9.4e-21 |
| F45C12.2 | F45C12.2\_Fed-L1-stage-larvae | 108 | 2.53 | 1.9e-20 |
| eor-1 | EOR-1\_Larvae-L3-stage | 121 | 2.27 | 7.7e-20 |
| dpl-1 | DPL-1\_Fed-L1-stage-larvae | 110 | 2.44 | 8.6e-20 |
| W03F9.2 | W03F9.2\_L4-Young-Adult-stage-larvae | 140 | 2.01 | 1.1e-19 |
| lsy-2 | LSY-2\_Fed-L1-stage-larvae | 114 | 2.35 | 1.7e-19 |
| zag-1 | ZAG-1\_Larvae-L4-stage | 68 | 3.70 | 1.9e-19 |
| efl-1 | EFL-1\_Larvae-L1-stage | 116 | 2.32 | 2.0e-19 |
| ceh-39 | CEH-39\_Embryos | 78 | 3.13 | 1.7e-18 |
| zag-1 | ZAG-1\_Larvae-L2-stage | 90 | 2.76 | 1.8e-18 |
| efl-1 | EFL-1\_Fed-L1-stage-larvae | 105 | 2.42 | 2.9e-18 |
| lsy-2 | LSY-2\_Larvae-L1-stage | 128 | 2.06 | 7.9e-18 |
| ceh-38 | CEH-38\_Larvae-L3-stage | 105 | 2.39 | 8.1e-18 |
| lin-15 | LIN-15B\_Fed-L1-stage-larvae | 65 | 3.46 | 5.6e-17 |
| hpl-2 | HPL-2\_Fed-L1-stage-larvae | 125 | 2.03 | 9.5e-17 |
| dve-1 | DVE-1\_Late-Embryos | 98 | 2.42 | 1.3e-16 |
| fos-1 | FOS-1\_Fed-L1-stage-larvae | 106 | 2.25 | 3.3e-16 |
| aly-2 | ALY-2\_Fed-L1-stage-larvae | 77 | 2.85 | 5.7e-16 |
| pha-4 | PHA-4\_Larvae-L4-stage | 84 | 2.63 | 1.2e-15 |
| hlh-30 | HLH-30\_Late-Embryos | 66 | 3.18 | 1.7e-15 |
| C01B12.2 | C01B12.2\_Larvae-L2-stage | 126 | 1.95 | 2.2e-15 |
| ceh-26 | CEH-26\_Late-Embryonic-stage | 86 | 2.55 | 2.3e-15 |
| R02D3.7 | R02D3.7\_Larvae-L3-stage | 93 | 2.41 | 2.5e-15 |
| jun-1 | JUN-1\_Larvae-L4-stage | 81 | 2.66 | 3.1e-15 |
| sem-4 | SEM-4\_Larvae-L2-stage | 93 | 2.39 | 3.7e-15 |
| ces-1 | CES-1\_Larvae-L3-stage | 63 | 3.24 | 4.7e-15 |
| zag-1 | ZAG-1\_Larvae-L3-stage | 40 | 5.06 | 5.5e-15 |
| F45C12.2 | F45C12.2\_Larvae-L3-stage | 62 | 3.23 | 1.1e-14 |
| nhr-23 | NHR-23\_Larvae-L3-stage | 101 | 2.21 | 1.6e-14 |
| skn-1 | SKN-1\_Larvae-L3-stage | 61 | 3.23 | 2.0e-14 |
| efl-1 | EFL-1\_Young-adult | 113 | 2.04 | 2.2e-14 |
| dpl-1 | DPL-1\_Young-adult | 76 | 2.70 | 2.2e-14 |
| nhr-6 | NHR-6\_Larvae-L4-stage | 74 | 2.74 | 2.8e-14 |
| lin-13 | LIN-13\_Larvae-L4-stage | 70 | 2.82 | 6.0e-14 |
| nhr-77 | NHR-77\_Larvae-L4-stage | 130 | 1.83 | 6.2e-14 |
| nhr-25 | NHR-25\_Larvae-L2-stage | 94 | 2.25 | 1.2e-13 |
| ham-1 | HAM-1\_Fed-L1-stage-larvae | 91 | 2.29 | 1.4e-13 |
| lin-13 | LIN-13\_Larvae-L2-stage | 69 | 2.77 | 2.2e-13 |
| mab-5 | MAB-5\_Larvae-L2-stage | 62 | 2.98 | 3.7e-13 |
| hlh-30 | HLH-30\_Larvae-L4-stage | 70 | 2.71 | 3.9e-13 |
| ces-1 | CES-1\_Fed-L1-stage-larvae | 55 | 3.28 | 4.1e-13 |
| unc-62 | UNC-62\_Larvae-L3-stage | 71 | 2.66 | 5.5e-13 |
| nhr-6 | NHR-6\_Larvae-L2-stage | 100 | 2.09 | 8.6e-13 |
| zag-1 | ZAG-1\_Fed-L1-stage-larvae | 57 | 3.10 | 1.2e-12 |
| lsy-2 | LSY-2\_Larvae-L4-stage | 52 | 3.33 | 1.5e-12 |
| nhr-77 | NHR-77\_Fed-L1-stage-larvae | 100 | 2.07 | 1.8e-12 |
| sax-3 | SAX-3\_Larvae-L2-stage | 73 | 2.55 | 1.8e-12 |
| jun-1 | JUN-1\_Larvae-L1-stage | 73 | 2.54 | 2.0e-12 |
| lsy-2 | LSY-2\_Larvae-L2-stage | 58 | 3.00 | 2.7e-12 |
| sax-3 | SAX-3\_Larvae-L4-stage | 93 | 2.13 | 5.0e-12 |
| pha-4 | PHA-4\_Larvae-L2-stage | 94 | 2.07 | 1.7e-11 |
| F45C12.2 | F45C12.2\_Larvae-L2-stage | 43 | 3.67 | 1.7e-11 |
| nhr-237 | NHR-237\_Embryos | 49 | 3.23 | 2.7e-11 |
| F23B12.7 | F23B12.7\_Young-adult | 69 | 2.50 | 2.9e-11 |
| unc-62 | UNC-62\_Day-Four-Young-Adult | 75 | 2.33 | 6.5e-11 |
| unc-62 | UNC-62\_Young-adult-Day-4 | 75 | 2.33 | 6.5e-11 |
| nfya-1 | NFYA-1\_Young-adult | 43 | 3.52 | 6.7e-11 |
| ztf-4 | ZTF-4\_Larvae-L2-stage | 43 | 3.49 | 8.7e-11 |
| C16A3.4 | C16A3.4\_Fed-L1-stage-larvae | 77 | 2.27 | 1.0e-10 |
| jun-1 | JUN-1\_Larvae-L3-stage | 70 | 2.39 | 1.5e-10 |
| nhr-237 | NHR-237\_Larvae-L1-stage | 42 | 3.46 | 2.0e-10 |
| nhr-129 | NHR-129\_Larvae-L2-stage | 116 | 1.75 | 2.9e-10 |
| ama-1 | AMA-1\_Larvae-L3-stage | 48 | 2.98 | 7.6e-10 |
| alr-1 | ALR-1\_Larvae-L2-stage | 86 | 2.03 | 8.0e-10 |
| nhr-76 | NHR-76\_Larvae-L4-stage | 62 | 2.47 | 1.0e-09 |
| egl-5 | EGL-5\_Larvae-L3-stage | 77 | 2.11 | 3.1e-09 |
| mef-2 | MEF-2\_Fed-L1-stage-larvae | 31 | 4.11 | 3.3e-09 |
| R02D3.7 | R02D3.7\_Larvae-L2-stage | 62 | 2.40 | 3.3e-09 |
| ztf-7 | ZTF-7\_Larvae-L4-stage | 57 | 2.53 | 3.4e-09 |
| aha-1 | AHA-1\_Larvae-L4-stage | 26 | 4.82 | 4.7e-09 |
| unc-39 | UNC-39\_Embryos | 33 | 3.79 | 5.7e-09 |
| fkh-2 | FKH-2\_Larvae-L3-stage | 51 | 2.69 | 5.7e-09 |
| elt-3 | ELT-3\_Embryos | 70 | 2.20 | 5.9e-09 |
| lin-13 | LIN-13\_Larvae-L1-stage | 37 | 3.42 | 6.2e-09 |
| ces-1 | CES-1\_Larvae-L4-stage | 31 | 3.98 | 6.7e-09 |
| ztf-11 | ZTF-11\_Embryos | 28 | 4.35 | 8.4e-09 |
| elt-1 | ELT-1\_Larvae-L3-stage | 45 | 2.89 | 9.7e-09 |
| fos-1 | FOS-1\_Larvae-L2-stage | 88 | 1.91 | 1.0e-08 |
| gei-11 | GEI-11\_Young-adult | 46 | 2.83 | 1.1e-08 |
| nhr-77 | NHR-77\_Larvae-L3-stage | 60 | 2.37 | 1.1e-08 |
| nhr-11 | NHR-11\_Larvae-L2-stage | 49 | 2.68 | 1.7e-08 |
| dve-1 | DVE-1\_Larvae-L4-stage | 59 | 2.34 | 2.7e-08 |
| pha-4 | PHA-4\_Young-adult | 42 | 2.83 | 7.5e-08 |
| lin-35 | LIN-35\_Starved-L1-stage-larvae | 39 | 2.98 | 8.4e-08 |
| R02D3.7 | R02D3.7\_Larvae-L4-stage | 41 | 2.78 | 2.1e-07 |
| nhr-28 | NHR-28\_Larvae-L4-stage | 107 | 1.63 | 3.0e-07 |
| ztf-4 | ZTF-4\_Larvae-L3-stage | 30 | 3.46 | 3.1e-07 |
| nhr-77 | NHR-77\_Larvae-L2-stage | 44 | 2.59 | 4.0e-07 |
| aly-2 | ALY-2\_Larvae-L3-stage | 53 | 2.30 | 4.3e-07 |
| unc-62 | UNC-62\_Larvae-L2-stage | 44 | 2.53 | 7.4e-07 |
| gei-11 | GEI-11\_Embryos | 37 | 2.75 | 1.6e-06 |
| nhr-2 | NHR-2\_Embryos | 45 | 2.42 | 1.8e-06 |
| nhr-76 | NHR-76\_Larvae-L3-stage | 41 | 2.52 | 2.8e-06 |
| sax-3 | SAX-3\_Larvae-L3-stage | 40 | 2.56 | 2.8e-06 |
| fos-1 | FOS-1\_Larvae-L3-stage | 55 | 2.12 | 3.3e-06 |
| sax-3 | SAX-3\_Fed-L1-stage-larvae | 33 | 2.85 | 4.3e-06 |
| aly-2 | ALY-2\_Larvae-L2-stage | 19 | 4.35 | 7.1e-06 |
| fos-1 | FOS-1\_Larvae-L4-stage | 45 | 2.28 | 9.3e-06 |
| pax-1 | PAX-1\_Embryos | 30 | 2.93 | 9.4e-06 |
| peb-1 | PEB-1\_Larvae-L2-stage | 31 | 2.86 | 9.9e-06 |
| daf-12 | DAF-12\_Larvae-L3-stage | 21 | 3.58 | 3.4e-05 |
| lin-15 | LIN-15B\_Larvae-L4-stage | 15 | 4.81 | 4.2e-05 |
| skn-1 | SKN-1\_Larvae-L4-stage | 13 | 5.48 | 5.5e-05 |
| ceh-16 | CEH-16\_Larvae-L2-stage | 38 | 2.30 | 7.3e-05 |
| ztf-4 | ZTF-4\_Larvae-L1-stage | 23 | 3.08 | 1.2e-04 |
| F16B12.6 | F16B12.6\_Fed-L1-stage-larvae | 34 | 2.39 | 1.2e-04 |
| nhr-23 | NHR-23\_Larvae-L2-stage | 19 | 3.38 | 2.4e-04 |
| nhr-21 | NHR-21\_Larvae-L2-stage | 25 | 2.72 | 3.2e-04 |
| nhr-10 | NHR-10\_Larvae-L4-stage | 26 | 2.56 | 6.0e-04 |
| unc-39 | UNC-39\_Larvae-L2-stage | 15 | 3.76 | 6.8e-04 |
| zip-2 | ZIP-2\_Larvae-L4-stage | 18 | 3.24 | 7.0e-04 |
| aha-1 | AHA-1\_Fed-L1-stage-larvae | 17 | 3.34 | 8.1e-04 |
| nhr-237 | NHR-237\_Larvae-L2-stage | 12 | 4.12 | 1.9e-03 |
| unc-62 | UNC-62\_Fed-L1-stage-larvae | 21 | 2.61 | 2.8e-03 |
| mab-5 | MAB-5\_Embryos | 19 | 2.75 | 3.2e-03 |
| nhr-12 | NHR-12\_Larvae-L2-stage | 9 | 4.31 | 1.0e-02 |
| ztf-11 | ZTF-11\_Larvae-L3-stage | 11 | 2.92 | 4.8e-02 |
| nhr-116 | NHR-116\_Larvae-L2-stage | 18 | 2.20 | 4.8e-02 |
