# Supplementary material for: Overlapping cell population expression profiling and regulatory inference in C. elegans
Source: BMC Genomics. 2016 Feb 29;17:159. doi: 10.1186/s12864-016-2482-z (PMC4772325; doi:10.1186/s12864-016-2482-z)
Supplement: Additional file 13: — Web supplement. (DOC 21 kb) [file 12864_2016_2482_MOESM13_ESM.zip › sortWeb/clusters/hier.300.clusters/221.html]

Cluster 221 

## Cluster 221

### Expression

| cnd-1 rep. 1 | cnd-1 rep. 2 | cnd-1 rep. 3 | pha-4 rep. 1 | pha-4 rep. 2 | pha-4 rep. 3 | ceh-27 | ceh-36 | ceh-6 | F21D5.9 | mir-57 | mls-2 | pal-1 | pros-1 | ttx-3 | unc-130 | hlh-16 | irx-1 | ceh-6 (+) hlh-16 (+) | ceh-6 (+) hlh-16 (-) | ceh-6 (-) hlh-16 (+) | cnd-1 singlets | pha-4 singlets | 0 | 60 | 120 | 150 | 180 | 240 | 330 | 390 | 420 | 480 | 540 | 570 | 600 | 630 | 660 | NAME | Functional description |
| --- | --- | --- | --- | --- | --- | --- | --- | --- | --- | --- | --- | --- | --- | --- | --- | --- | --- | --- | --- | --- | --- | --- | --- | --- | --- | --- | --- | --- | --- | --- | --- | --- | --- | --- | --- | --- | --- | --- | --- |
|  |  |  |  |  |  |  |  |  |  |  |  |  |  |  |  |  |  |  |  |  |  |  |  |  |  |  |  |  |  |  |  |  |  |  |  |  |  | M110.3 |  |
|  |  |  |  |  |  |  |  |  |  |  |  |  |  |  |  |  |  |  |  |  |  |  |  |  |  |  |  |  |  |  |  |  |  |  |  |  |  | *mml-1* | Myc and Mondo-Like |
|  |  |  |  |  |  |  |  |  |  |  |  |  |  |  |  |  |  |  |  |  |  |  |  |  |  |  |  |  |  |  |  |  |  |  |  |  |  | K02D10.4 |  |
|  |  |  |  |  |  |  |  |  |  |  |  |  |  |  |  |  |  |  |  |  |  |  |  |  |  |  |  |  |  |  |  |  |  |  |  |  |  | *col-60* | COLlagen |
|  |  |  |  |  |  |  |  |  |  |  |  |  |  |  |  |  |  |  |  |  |  |  |  |  |  |  |  |  |  |  |  |  |  |  |  |  |  | D1053.3 |  |
|  |  |  |  |  |  |  |  |  |  |  |  |  |  |  |  |  |  |  |  |  |  |  |  |  |  |  |  |  |  |  |  |  |  |  |  |  |  | F41D3.9 |  |
|  |  |  |  |  |  |  |  |  |  |  |  |  |  |  |  |  |  |  |  |  |  |  |  |  |  |  |  |  |  |  |  |  |  |  |  |  |  | *rmd-1* | Regulator of Microtubule Dynamics |
|  |  |  |  |  |  |  |  |  |  |  |  |  |  |  |  |  |  |  |  |  |  |  |  |  |  |  |  |  |  |  |  |  |  |  |  |  |  | B0273.112 |  |
|  |  |  |  |  |  |  |  |  |  |  |  |  |  |  |  |  |  |  |  |  |  |  |  |  |  |  |  |  |  |  |  |  |  |  |  |  |  | T28D9.12 |  |
|  |  |  |  |  |  |  |  |  |  |  |  |  |  |  |  |  |  |  |  |  |  |  |  |  |  |  |  |  |  |  |  |  |  |  |  |  |  | *srab-26* | Serpentine Receptor, class AB (class A-like) |
|  |  |  |  |  |  |  |  |  |  |  |  |  |  |  |  |  |  |  |  |  |  |  |  |  |  |  |  |  |  |  |  |  |  |  |  |  |  | C25A1.17 |  |
|  |  |  |  |  |  |  |  |  |  |  |  |  |  |  |  |  |  |  |  |  |  |  |  |  |  |  |  |  |  |  |  |  |  |  |  |  |  | T16G1.14 |  |
|  |  |  |  |  |  |  |  |  |  |  |  |  |  |  |  |  |  |  |  |  |  |  |  |  |  |  |  |  |  |  |  |  |  |  |  |  |  | B0024.3 |  |
|  |  |  |  |  |  |  |  |  |  |  |  |  |  |  |  |  |  |  |  |  |  |  |  |  |  |  |  |  |  |  |  |  |  |  |  |  |  | *arrd-26* | ARRestin Domain protein |
|  |  |  |  |  |  |  |  |  |  |  |  |  |  |  |  |  |  |  |  |  |  |  |  |  |  |  |  |  |  |  |  |  |  |  |  |  |  | Y61A9LA.1 |  |
|  |  |  |  |  |  |  |  |  |  |  |  |  |  |  |  |  |  |  |  |  |  |  |  |  |  |  |  |  |  |  |  |  |  |  |  |  |  | *fum-1* | FUMarase |
|  |  |  |  |  |  |  |  |  |  |  |  |  |  |  |  |  |  |  |  |  |  |  |  |  |  |  |  |  |  |  |  |  |  |  |  |  |  | F42A9.12 |  |
|  |  |  |  |  |  |  |  |  |  |  |  |  |  |  |  |  |  |  |  |  |  |  |  |  |  |  |  |  |  |  |  |  |  |  |  |  |  | H14E04.3 |  |
|  |  |  |  |  |  |  |  |  |  |  |  |  |  |  |  |  |  |  |  |  |  |  |  |  |  |  |  |  |  |  |  |  |  |  |  |  |  | C01C4.3 |  |
|  |  |  |  |  |  |  |  |  |  |  |  |  |  |  |  |  |  |  |  |  |  |  |  |  |  |  |  |  |  |  |  |  |  |  |  |  |  | *linc-126* | Long Intervening Non-Coding RNA |
|  |  |  |  |  |  |  |  |  |  |  |  |  |  |  |  |  |  |  |  |  |  |  |  |  |  |  |  |  |  |  |  |  |  |  |  |  |  | ZK418.11 |  |
|  |  |  |  |  |  |  |  |  |  |  |  |  |  |  |  |  |  |  |  |  |  |  |  |  |  |  |  |  |  |  |  |  |  |  |  |  |  | *sru-46* | Serpentine Receptor, class U |
|  |  |  |  |  |  |  |  |  |  |  |  |  |  |  |  |  |  |  |  |  |  |  |  |  |  |  |  |  |  |  |  |  |  |  |  |  |  | Y46E12BR.1 |  |
|  |  |  |  |  |  |  |  |  |  |  |  |  |  |  |  |  |  |  |  |  |  |  |  |  |  |  |  |  |  |  |  |  |  |  |  |  |  | F15G9.3 |  |
|  |  |  |  |  |  |  |  |  |  |  |  |  |  |  |  |  |  |  |  |  |  |  |  |  |  |  |  |  |  |  |  |  |  |  |  |  |  | F20C5.3 |  |
|  |  |  |  |  |  |  |  |  |  |  |  |  |  |  |  |  |  |  |  |  |  |  |  |  |  |  |  |  |  |  |  |  |  |  |  |  |  | *aakg-4* | AMP-Activated protein Kinase Gamma subunit |
|  |  |  |  |  |  |  |  |  |  |  |  |  |  |  |  |  |  |  |  |  |  |  |  |  |  |  |  |  |  |  |  |  |  |  |  |  |  | *frpr-7* | FMRFamide Peptide Receptor family |
|  |  |  |  |  |  |  |  |  |  |  |  |  |  |  |  |  |  |  |  |  |  |  |  |  |  |  |  |  |  |  |  |  |  |  |  |  |  | *hlh-11* | Helix Loop Helix |
|  |  |  |  |  |  |  |  |  |  |  |  |  |  |  |  |  |  |  |  |  |  |  |  |  |  |  |  |  |  |  |  |  |  |  |  |  |  | *bcat-1* | Branched Chain AminoTransferase |
|  |  |  |  |  |  |  |  |  |  |  |  |  |  |  |  |  |  |  |  |  |  |  |  |  |  |  |  |  |  |  |  |  |  |  |  |  |  | Y105E8B.14 |  |
|  |  |  |  |  |  |  |  |  |  |  |  |  |  |  |  |  |  |  |  |  |  |  |  |  |  |  |  |  |  |  |  |  |  |  |  |  |  | *tsp-5* | TetraSPanin family |
|  |  |  |  |  |  |  |  |  |  |  |  |  |  |  |  |  |  |  |  |  |  |  |  |  |  |  |  |  |  |  |  |  |  |  |  |  |  | *mec-1* | MEChanosensory abnormality |
|  |  |  |  |  |  |  |  |  |  |  |  |  |  |  |  |  |  |  |  |  |  |  |  |  |  |  |  |  |  |  |  |  |  |  |  |  |  | *sulp-2* | SULfate Permease family |
|  |  |  |  |  |  |  |  |  |  |  |  |  |  |  |  |  |  |  |  |  |  |  |  |  |  |  |  |  |  |  |  |  |  |  |  |  |  | *exp-1* | EXPulsion defective (defecation) |
|  |  |  |  |  |  |  |  |  |  |  |  |  |  |  |  |  |  |  |  |  |  |  |  |  |  |  |  |  |  |  |  |  |  |  |  |  |  | H10D18.5 |  |
|  |  |  |  |  |  |  |  |  |  |  |  |  |  |  |  |  |  |  |  |  |  |  |  |  |  |  |  |  |  |  |  |  |  |  |  |  |  | K03H9.3 |  |
|  |  |  |  |  |  |  |  |  |  |  |  |  |  |  |  |  |  |  |  |  |  |  |  |  |  |  |  |  |  |  |  |  |  |  |  |  |  | K01A11.1 |  |
|  |  |  |  |  |  |  |  |  |  |  |  |  |  |  |  |  |  |  |  |  |  |  |  |  |  |  |  |  |  |  |  |  |  |  |  |  |  | *mam-2* | MAM (Meprin, A5-protein, PTPmu) domain protein |
|  |  |  |  |  |  |  |  |  |  |  |  |  |  |  |  |  |  |  |  |  |  |  |  |  |  |  |  |  |  |  |  |  |  |  |  |  |  | W02F12.8 |  |
|  |  |  |  |  |  |  |  |  |  |  |  |  |  |  |  |  |  |  |  |  |  |  |  |  |  |  |  |  |  |  |  |  |  |  |  |  |  | F41E6.7 |  |
|  |  |  |  |  |  |  |  |  |  |  |  |  |  |  |  |  |  |  |  |  |  |  |  |  |  |  |  |  |  |  |  |  |  |  |  |  |  | *lin-31* | abnormal cell LINeage |
|  |  |  |  |  |  |  |  |  |  |  |  |  |  |  |  |  |  |  |  |  |  |  |  |  |  |  |  |  |  |  |  |  |  |  |  |  |  | F35B3.7 |  |
|  |  |  |  |  |  |  |  |  |  |  |  |  |  |  |  |  |  |  |  |  |  |  |  |  |  |  |  |  |  |  |  |  |  |  |  |  |  | *hlh-8* | Helix Loop Helix |
|  |  |  |  |  |  |  |  |  |  |  |  |  |  |  |  |  |  |  |  |  |  |  |  |  |  |  |  |  |  |  |  |  |  |  |  |  |  | *mls-1* | Mesodermal Lineage Specification |
|  |  |  |  |  |  |  |  |  |  |  |  |  |  |  |  |  |  |  |  |  |  |  |  |  |  |  |  |  |  |  |  |  |  |  |  |  |  | F49F1.14 |  |
|  |  |  |  |  |  |  |  |  |  |  |  |  |  |  |  |  |  |  |  |  |  |  |  |  |  |  |  |  |  |  |  |  |  |  |  |  |  | T04C12.3 |  |
|  |  |  |  |  |  |  |  |  |  |  |  |  |  |  |  |  |  |  |  |  |  |  |  |  |  |  |  |  |  |  |  |  |  |  |  |  |  | T19D2.3 |  |
|  |  |  |  |  |  |  |  |  |  |  |  |  |  |  |  |  |  |  |  |  |  |  |  |  |  |  |  |  |  |  |  |  |  |  |  |  |  | ZK1073.2 |  |
|  |  |  |  |  |  |  |  |  |  |  |  |  |  |  |  |  |  |  |  |  |  |  |  |  |  |  |  |  |  |  |  |  |  |  |  |  |  | Y66C5A.1 |  |
|  |  |  |  |  |  |  |  |  |  |  |  |  |  |  |  |  |  |  |  |  |  |  |  |  |  |  |  |  |  |  |  |  |  |  |  |  |  | R02E4.2 |  |
|  |  |  |  |  |  |  |  |  |  |  |  |  |  |  |  |  |  |  |  |  |  |  |  |  |  |  |  |  |  |  |  |  |  |  |  |  |  | F36F2.2 |  |
|  |  |  |  |  |  |  |  |  |  |  |  |  |  |  |  |  |  |  |  |  |  |  |  |  |  |  |  |  |  |  |  |  |  |  |  |  |  | *tni-3* | TropoNin I |
|  |  |  |  |  |  |  |  |  |  |  |  |  |  |  |  |  |  |  |  |  |  |  |  |  |  |  |  |  |  |  |  |  |  |  |  |  |  | F19F10.3 |  |
|  |  |  |  |  |  |  |  |  |  |  |  |  |  |  |  |  |  |  |  |  |  |  |  |  |  |  |  |  |  |  |  |  |  |  |  |  |  | *lgc-35* | Ligand-Gated ion Channel |
|  |  |  |  |  |  |  |  |  |  |  |  |  |  |  |  |  |  |  |  |  |  |  |  |  |  |  |  |  |  |  |  |  |  |  |  |  |  | F35D11.1 |  |
|  |  |  |  |  |  |  |  |  |  |  |  |  |  |  |  |  |  |  |  |  |  |  |  |  |  |  |  |  |  |  |  |  |  |  |  |  |  | *glb-26* | GLoBin related |
|  |  |  |  |  |  |  |  |  |  |  |  |  |  |  |  |  |  |  |  |  |  |  |  |  |  |  |  |  |  |  |  |  |  |  |  |  |  | T23B12.8 |  |
|  |  |  |  |  |  |  |  |  |  |  |  |  |  |  |  |  |  |  |  |  |  |  |  |  |  |  |  |  |  |  |  |  |  |  |  |  |  | K11D12.11 |  |
|  |  |  |  |  |  |  |  |  |  |  |  |  |  |  |  |  |  |  |  |  |  |  |  |  |  |  |  |  |  |  |  |  |  |  |  |  |  | *ttr-10* | TransThyretin-Related family domain |
|  |  |  |  |  |  |  |  |  |  |  |  |  |  |  |  |  |  |  |  |  |  |  |  |  |  |  |  |  |  |  |  |  |  |  |  |  |  | *dsc-1* | Defecation Suppressor of Clk-1 |
|  |  |  |  |  |  |  |  |  |  |  |  |  |  |  |  |  |  |  |  |  |  |  |  |  |  |  |  |  |  |  |  |  |  |  |  |  |  | *srx-34* | Serpentine Receptor, class X |
|  |  |  |  |  |  |  |  |  |  |  |  |  |  |  |  |  |  |  |  |  |  |  |  |  |  |  |  |  |  |  |  |  |  |  |  |  |  | F22E12.3 |  |
|  |  |  |  |  |  |  |  |  |  |  |  |  |  |  |  |  |  |  |  |  |  |  |  |  |  |  |  |  |  |  |  |  |  |  |  |  |  | F01F1.3 |  |
|  |  |  |  |  |  |  |  |  |  |  |  |  |  |  |  |  |  |  |  |  |  |  |  |  |  |  |  |  |  |  |  |  |  |  |  |  |  | *cyp-33C12* | CYtochrome P450 family |
|  |  |  |  |  |  |  |  |  |  |  |  |  |  |  |  |  |  |  |  |  |  |  |  |  |  |  |  |  |  |  |  |  |  |  |  |  |  | B0403.3 |  |
|  |  |  |  |  |  |  |  |  |  |  |  |  |  |  |  |  |  |  |  |  |  |  |  |  |  |  |  |  |  |  |  |  |  |  |  |  |  | Y47D9A.1 |  |
|  |  |  |  |  |  |  |  |  |  |  |  |  |  |  |  |  |  |  |  |  |  |  |  |  |  |  |  |  |  |  |  |  |  |  |  |  |  | *pfk-1* | PhosphoFructoKinase |
|  |  |  |  |  |  |  |  |  |  |  |  |  |  |  |  |  |  |  |  |  |  |  |  |  |  |  |  |  |  |  |  |  |  |  |  |  |  | ZK662.6 |  |
|  |  |  |  |  |  |  |  |  |  |  |  |  |  |  |  |  |  |  |  |  |  |  |  |  |  |  |  |  |  |  |  |  |  |  |  |  |  | *ugt-58* | UDP-GlucuronosylTransferase |
|  |  |  |  |  |  |  |  |  |  |  |  |  |  |  |  |  |  |  |  |  |  |  |  |  |  |  |  |  |  |  |  |  |  |  |  |  |  | *srd-32* | Serpentine Receptor, class D (delta) |
|  |  |  |  |  |  |  |  |  |  |  |  |  |  |  |  |  |  |  |  |  |  |  |  |  |  |  |  |  |  |  |  |  |  |  |  |  |  | *bli-3* | DUal OXidase |
|  |  |  |  |  |  |  |  |  |  |  |  |  |  |  |  |  |  |  |  |  |  |  |  |  |  |  |  |  |  |  |  |  |  |  |  |  |  | *rga-2* | Rho GTPase Activating protein |
|  |  |  |  |  |  |  |  |  |  |  |  |  |  |  |  |  |  |  |  |  |  |  |  |  |  |  |  |  |  |  |  |  |  |  |  |  |  | W09D10.5 |  |
|  |  |  |  |  |  |  |  |  |  |  |  |  |  |  |  |  |  |  |  |  |  |  |  |  |  |  |  |  |  |  |  |  |  |  |  |  |  | Y53F4B.27 |  |
|  |  |  |  |  |  |  |  |  |  |  |  |  |  |  |  |  |  |  |  |  |  |  |  |  |  |  |  |  |  |  |  |  |  |  |  |  |  | F32D8.7 |  |
|  |  |  |  |  |  |  |  |  |  |  |  |  |  |  |  |  |  |  |  |  |  |  |  |  |  |  |  |  |  |  |  |  |  |  |  |  |  | Y39A1A.9 |  |
|  |  |  |  |  |  |  |  |  |  |  |  |  |  |  |  |  |  |  |  |  |  |  |  |  |  |  |  |  |  |  |  |  |  |  |  |  |  | Y47D3B.4 |  |
|  |  |  |  |  |  |  |  |  |  |  |  |  |  |  |  |  |  |  |  |  |  |  |  |  |  |  |  |  |  |  |  |  |  |  |  |  |  | *lin-48* | abnormal cell LINeage |
|  |  |  |  |  |  |  |  |  |  |  |  |  |  |  |  |  |  |  |  |  |  |  |  |  |  |  |  |  |  |  |  |  |  |  |  |  |  | Y57G11C.42 |  |
|  |  |  |  |  |  |  |  |  |  |  |  |  |  |  |  |  |  |  |  |  |  |  |  |  |  |  |  |  |  |  |  |  |  |  |  |  |  | Y95B8A.2 |  |
|  |  |  |  |  |  |  |  |  |  |  |  |  |  |  |  |  |  |  |  |  |  |  |  |  |  |  |  |  |  |  |  |  |  |  |  |  |  | *dhs-5* | DeHydrogenases, Short chain |
|  |  |  |  |  |  |  |  |  |  |  |  |  |  |  |  |  |  |  |  |  |  |  |  |  |  |  |  |  |  |  |  |  |  |  |  |  |  | F33A8.7 |  |
|  |  |  |  |  |  |  |  |  |  |  |  |  |  |  |  |  |  |  |  |  |  |  |  |  |  |  |  |  |  |  |  |  |  |  |  |  |  | C25E10.12 |  |
|  |  |  |  |  |  |  |  |  |  |  |  |  |  |  |  |  |  |  |  |  |  |  |  |  |  |  |  |  |  |  |  |  |  |  |  |  |  | *pqn-32* | Prion-like-(Q/N-rich)-domain-bearing protein |
|  |  |  |  |  |  |  |  |  |  |  |  |  |  |  |  |  |  |  |  |  |  |  |  |  |  |  |  |  |  |  |  |  |  |  |  |  |  | F01D5.6 |  |
|  |  |  |  |  |  |  |  |  |  |  |  |  |  |  |  |  |  |  |  |  |  |  |  |  |  |  |  |  |  |  |  |  |  |  |  |  |  | H14E04.1 |  |
|  |  |  |  |  |  |  |  |  |  |  |  |  |  |  |  |  |  |  |  |  |  |  |  |  |  |  |  |  |  |  |  |  |  |  |  |  |  | F11F1.4 |  |
|  |  |  |  |  |  |  |  |  |  |  |  |  |  |  |  |  |  |  |  |  |  |  |  |  |  |  |  |  |  |  |  |  |  |  |  |  |  | Y48G8AL.12 |  |
|  |  |  |  |  |  |  |  |  |  |  |  |  |  |  |  |  |  |  |  |  |  |  |  |  |  |  |  |  |  |  |  |  |  |  |  |  |  | C55B7.10 |  |
|  |  |  |  |  |  |  |  |  |  |  |  |  |  |  |  |  |  |  |  |  |  |  |  |  |  |  |  |  |  |  |  |  |  |  |  |  |  | B0403.5 |  |
|  |  |  |  |  |  |  |  |  |  |  |  |  |  |  |  |  |  |  |  |  |  |  |  |  |  |  |  |  |  |  |  |  |  |  |  |  |  | *fmo-4* | Flavin-containing MonoOxygenase family |
|  |  |  |  |  |  |  |  |  |  |  |  |  |  |  |  |  |  |  |  |  |  |  |  |  |  |  |  |  |  |  |  |  |  |  |  |  |  | C06G1.1 |  |
|  |  |  |  |  |  |  |  |  |  |  |  |  |  |  |  |  |  |  |  |  |  |  |  |  |  |  |  |  |  |  |  |  |  |  |  |  |  | F13B9.2 |  |
|  |  |  |  |  |  |  |  |  |  |  |  |  |  |  |  |  |  |  |  |  |  |  |  |  |  |  |  |  |  |  |  |  |  |  |  |  |  | *ptr-2* | PaTched Related family |
|  |  |  |  |  |  |  |  |  |  |  |  |  |  |  |  |  |  |  |  |  |  |  |  |  |  |  |  |  |  |  |  |  |  |  |  |  |  | *hog-1* | HOG only (Hedgehog Hog domain alone) |
|  |  |  |  |  |  |  |  |  |  |  |  |  |  |  |  |  |  |  |  |  |  |  |  |  |  |  |  |  |  |  |  |  |  |  |  |  |  | *gly-8* | GLYcosylation related |
|  |  |  |  |  |  |  |  |  |  |  |  |  |  |  |  |  |  |  |  |  |  |  |  |  |  |  |  |  |  |  |  |  |  |  |  |  |  | F39D8.3 |  |
|  |  |  |  |  |  |  |  |  |  |  |  |  |  |  |  |  |  |  |  |  |  |  |  |  |  |  |  |  |  |  |  |  |  |  |  |  |  | T06D8.10 |  |
|  |  |  |  |  |  |  |  |  |  |  |  |  |  |  |  |  |  |  |  |  |  |  |  |  |  |  |  |  |  |  |  |  |  |  |  |  |  | *abu-12* | Activated in Blocked Unfolded protein response |
|  |  |  |  |  |  |  |  |  |  |  |  |  |  |  |  |  |  |  |  |  |  |  |  |  |  |  |  |  |  |  |  |  |  |  |  |  |  | T24D5.2 |  |
|  |  |  |  |  |  |  |  |  |  |  |  |  |  |  |  |  |  |  |  |  |  |  |  |  |  |  |  |  |  |  |  |  |  |  |  |  |  | *oac-46* | O-ACyltransferase homolog |
|  |  |  |  |  |  |  |  |  |  |  |  |  |  |  |  |  |  |  |  |  |  |  |  |  |  |  |  |  |  |  |  |  |  |  |  |  |  | *tep-1* | TEP (ThiolEster contaiTEP (ThiolEster containing Protein) |
|  |  |  |  |  |  |  |  |  |  |  |  |  |  |  |  |  |  |  |  |  |  |  |  |  |  |  |  |  |  |  |  |  |  |  |  |  |  | Y110A2AL.9 |  |
|  |  |  |  |  |  |  |  |  |  |  |  |  |  |  |  |  |  |  |  |  |  |  |  |  |  |  |  |  |  |  |  |  |  |  |  |  |  | *ptc-3* | PaTChed family |
|  |  |  |  |  |  |  |  |  |  |  |  |  |  |  |  |  |  |  |  |  |  |  |  |  |  |  |  |  |  |  |  |  |  |  |  |  |  | R08C7.8 |  |
|  |  |  |  |  |  |  |  |  |  |  |  |  |  |  |  |  |  |  |  |  |  |  |  |  |  |  |  |  |  |  |  |  |  |  |  |  |  | K07H8.8 |  |
|  |  |  |  |  |  |  |  |  |  |  |  |  |  |  |  |  |  |  |  |  |  |  |  |  |  |  |  |  |  |  |  |  |  |  |  |  |  | *oac-17* | O-ACyltransferase homolog |
|  |  |  |  |  |  |  |  |  |  |  |  |  |  |  |  |  |  |  |  |  |  |  |  |  |  |  |  |  |  |  |  |  |  |  |  |  |  | F32H2.8 |  |
|  |  |  |  |  |  |  |  |  |  |  |  |  |  |  |  |  |  |  |  |  |  |  |  |  |  |  |  |  |  |  |  |  |  |  |  |  |  | T28A11.20 |  |
|  |  |  |  |  |  |  |  |  |  |  |  |  |  |  |  |  |  |  |  |  |  |  |  |  |  |  |  |  |  |  |  |  |  |  |  |  |  | *str-121* | Seven TM Receptor |
|  |  |  |  |  |  |  |  |  |  |  |  |  |  |  |  |  |  |  |  |  |  |  |  |  |  |  |  |  |  |  |  |  |  |  |  |  |  | *lgc-45* | Ligand-Gated ion Channel |
|  |  |  |  |  |  |  |  |  |  |  |  |  |  |  |  |  |  |  |  |  |  |  |  |  |  |  |  |  |  |  |  |  |  |  |  |  |  | C26B2.2 |  |
|  |  |  |  |  |  |  |  |  |  |  |  |  |  |  |  |  |  |  |  |  |  |  |  |  |  |  |  |  |  |  |  |  |  |  |  |  |  | *srw-85* | Serpentine Receptor, class W |
|  |  |  |  |  |  |  |  |  |  |  |  |  |  |  |  |  |  |  |  |  |  |  |  |  |  |  |  |  |  |  |  |  |  |  |  |  |  | F18E9.8 |  |
|  |  |  |  |  |  |  |  |  |  |  |  |  |  |  |  |  |  |  |  |  |  |  |  |  |  |  |  |  |  |  |  |  |  |  |  |  |  | F56D12.t4 |  |
|  |  |  |  |  |  |  |  |  |  |  |  |  |  |  |  |  |  |  |  |  |  |  |  |  |  |  |  |  |  |  |  |  |  |  |  |  |  | *fipr-6* | FIP (Fungus-Induced Protein) Related |
|  |  |  |  |  |  |  |  |  |  |  |  |  |  |  |  |  |  |  |  |  |  |  |  |  |  |  |  |  |  |  |  |  |  |  |  |  |  | Y39A3CL.12 |  |
|  |  |  |  |  |  |  |  |  |  |  |  |  |  |  |  |  |  |  |  |  |  |  |  |  |  |  |  |  |  |  |  |  |  |  |  |  |  | *tag-38* | Temporarily Assigned Gene name |
|  |  |  |  |  |  |  |  |  |  |  |  |  |  |  |  |  |  |  |  |  |  |  |  |  |  |  |  |  |  |  |  |  |  |  |  |  |  | Y65B4A.8 |  |
|  |  |  |  |  |  |  |  |  |  |  |  |  |  |  |  |  |  |  |  |  |  |  |  |  |  |  |  |  |  |  |  |  |  |  |  |  |  | *vnut-1* | Vesicular NUcleotide Transporte |
|  |  |  |  |  |  |  |  |  |  |  |  |  |  |  |  |  |  |  |  |  |  |  |  |  |  |  |  |  |  |  |  |  |  |  |  |  |  | Y48G1A.1 |  |
|  |  |  |  |  |  |  |  |  |  |  |  |  |  |  |  |  |  |  |  |  |  |  |  |  |  |  |  |  |  |  |  |  |  |  |  |  |  | Y39H10A.5 |  |
|  |  |  |  |  |  |  |  |  |  |  |  |  |  |  |  |  |  |  |  |  |  |  |  |  |  |  |  |  |  |  |  |  |  |  |  |  |  | *ugt-42* | UDP-GlucuronosylTransferase |
|  |  |  |  |  |  |  |  |  |  |  |  |  |  |  |  |  |  |  |  |  |  |  |  |  |  |  |  |  |  |  |  |  |  |  |  |  |  | Y54G11A.1 |  |
|  |  |  |  |  |  |  |  |  |  |  |  |  |  |  |  |  |  |  |  |  |  |  |  |  |  |  |  |  |  |  |  |  |  |  |  |  |  | C27D8.1 |  |
|  |  |  |  |  |  |  |  |  |  |  |  |  |  |  |  |  |  |  |  |  |  |  |  |  |  |  |  |  |  |  |  |  |  |  |  |  |  | ZK993.2 |  |
|  |  |  |  |  |  |  |  |  |  |  |  |  |  |  |  |  |  |  |  |  |  |  |  |  |  |  |  |  |  |  |  |  |  |  |  |  |  | *fbxa-87* | F-box A protein |
|  |  |  |  |  |  |  |  |  |  |  |  |  |  |  |  |  |  |  |  |  |  |  |  |  |  |  |  |  |  |  |  |  |  |  |  |  |  | *nas-30* | Nematode AStacin protease |
|  |  |  |  |  |  |  |  |  |  |  |  |  |  |  |  |  |  |  |  |  |  |  |  |  |  |  |  |  |  |  |  |  |  |  |  |  |  | *pho-7* | intestinal acid PHOsphatase |
|  |  |  |  |  |  |  |  |  |  |  |  |  |  |  |  |  |  |  |  |  |  |  |  |  |  |  |  |  |  |  |  |  |  |  |  |  |  | F11F1.1 |  |
|  |  |  |  |  |  |  |  |  |  |  |  |  |  |  |  |  |  |  |  |  |  |  |  |  |  |  |  |  |  |  |  |  |  |  |  |  |  | F11F1.2 |  |
|  |  |  |  |  |  |  |  |  |  |  |  |  |  |  |  |  |  |  |  |  |  |  |  |  |  |  |  |  |  |  |  |  |  |  |  |  |  | T16G12.7 |  |
|  |  |  |  |  |  |  |  |  |  |  |  |  |  |  |  |  |  |  |  |  |  |  |  |  |  |  |  |  |  |  |  |  |  |  |  |  |  | *sru-1* | Serpentine Receptor, class U |
|  |  |  |  |  |  |  |  |  |  |  |  |  |  |  |  |  |  |  |  |  |  |  |  |  |  |  |  |  |  |  |  |  |  |  |  |  |  | *ifc-1* | Intermediate Filament, C |
|  |  |  |  |  |  |  |  |  |  |  |  |  |  |  |  |  |  |  |  |  |  |  |  |  |  |  |  |  |  |  |  |  |  |  |  |  |  | F58D12.1 |  |
|  |  |  |  |  |  |  |  |  |  |  |  |  |  |  |  |  |  |  |  |  |  |  |  |  |  |  |  |  |  |  |  |  |  |  |  |  |  | F42D1.12 |  |
|  |  |  |  |  |  |  |  |  |  |  |  |  |  |  |  |  |  |  |  |  |  |  |  |  |  |  |  |  |  |  |  |  |  |  |  |  |  | C18B12.14 |  |
|  |  |  |  |  |  |  |  |  |  |  |  |  |  |  |  |  |  |  |  |  |  |  |  |  |  |  |  |  |  |  |  |  |  |  |  |  |  | F15D4.4 |  |
|  |  |  |  |  |  |  |  |  |  |  |  |  |  |  |  |  |  |  |  |  |  |  |  |  |  |  |  |  |  |  |  |  |  |  |  |  |  | *frpr-11* | FMRFamide Peptide Receptor family |
|  |  |  |  |  |  |  |  |  |  |  |  |  |  |  |  |  |  |  |  |  |  |  |  |  |  |  |  |  |  |  |  |  |  |  |  |  |  | Y62F5A.14 |  |
|  |  |  |  |  |  |  |  |  |  |  |  |  |  |  |  |  |  |  |  |  |  |  |  |  |  |  |  |  |  |  |  |  |  |  |  |  |  | *srt-40* | Serpentine Receptor, class T |

### Phenotypes enriched

none found

### Anatomy terms enriched

none found

### GO terms enriched

none found

### Expression clusters enriched

|  |  |  |  |
| --- | --- | --- | --- |
| **Group name** | **Number in cluster** | **Enrichment** | **FDR corrected p** |
| Genes that show selective expression in a subset of cell types vs broadly expressed in many cell types. Correspond to 20% - 57% of enriched\_genes for a given cell type. WBPaper00037950:hypodermis\_embryo\_SelectivelyEnriched | 14 | 4.35 | 0.00145 |
| Genes down regulated in crh-1(nn3315) comparing to in N2. | 17 | 3.50 | 0.00232 |
| Genes with increased expression after 24 hours of infection by P.lumniescens Fold changes shown are pathogen vs OP50. WBPaper00038438:P.lumniescens\_24hr\_upregulated\_TilingArray | 56 | 1.65 | 0.00741 |
| A cluster of genes that oscillates with the molting cycle during early larval development. | 9 | 4.88 | 0.02370 |
| Genes down regulated after fed by 25 mg/ml pRJ-Fr.5 for 24 h beginning at the L4 stage. | 11 | 3.86 | 0.03190 |
| Genes regulated by octr-1(ok371) after infected with P. aeruginosa PA14 for 4 hours at 25 centigrade. | 21 | 2.39 | 0.03780 |

### Motifs enriched

|  |  |  |  |  |  |
| --- | --- | --- | --- | --- | --- |
| **Motif** | **Logo** | **Possible orthologs** | **Number of motifs in cluster** | **Enrichment** | **FDR corrected p** |
| klu\_SOLEXA\_5\_FBgn0013469 |  | ZC328.2 daf-16 | 64 | 1.96 | 2.9e-06 |
| EGR1\_f2 |  | klf-2 (0.54) ZC328.2 klf-1 | 115 | 1.35 | 2.2e-05 |
| CG5669\_SOLEXA\_5\_FBgn0039169 |  | klf-2 (0.54) klf-1 | 116 | 1.33 | 3.1e-05 |
| pTH10031 |  | mbr-1 | 94 | 1.45 | 1.3e-04 |
| MA0079.3 |  | klf-2 (0.54) ZC328.2 klf-1 | 96 | 1.44 | 1.4e-04 |
| pTH9198 |  | dmd-3 | 32 | 2.54 | 1.9e-04 |
| pTH9884 |  | tbx-39 | 128 | 1.22 | 2.8e-04 |
| HepG2\_RFX5\_Stanford |  | daf-19 | 78 | 1.56 | 3.0e-04 |
| Mw137 |  | blmp-1 (0.67) | 18 | 3.73 | 3.8e-04 |
| HLF\_si |  | ces-2 | 63 | 1.69 | 4.9e-04 |
| pTH9211 |  | C01B12.2 attf-1 | 53 | 1.82 | 6.2e-04 |
| pTH10722 |  | eor-1 ref-2 egrh-3 | 68 | 1.62 | 6.3e-04 |
| V$E47\_01 |  | ces-1 hlh-15 hlh-2 | 117 | 1.26 | 7.3e-04 |
| pTH10042 |  | nhr-5 | 95 | 1.39 | 7.4e-04 |
| MA0085.1 |  | ztf-3 lag-1 | 104 | 1.33 | 8.7e-04 |
| V$OCT1\_06 |  | ztf-9 ceh-18 | 77 | 1.52 | 9.0e-04 |
| MCR\_f1 |  | nhr-255 | 91 | 1.41 | 1.0e-03 |
| PAX8\_f1 |  | pax-2 | 59 | 1.69 | 1.1e-03 |
| pTH10037 |  | T22C8.4 ref-2 | 106 | 1.31 | 1.3e-03 |
| pTH5005 |  | crh-1 | 116 | 1.25 | 1.5e-03 |
| Sp4\_1011 |  | klf-2 (0.54) klf-1 sptf-3 | 113 | 1.27 | 1.6e-03 |
| pTH9215 |  | C34D1.1 | 23 | 2.75 | 1.6e-03 |
| V$ARP1\_01 |  | nhr-2 nhr-62 | 38 | 2.03 | 1.9e-03 |
| PBDE\_GATA1\_UCD |  | elt-1 | 38 | 2.03 | 1.9e-03 |
| pTH9247 |  | dmd-3 C34D1.1 | 77 | 1.48 | 2.0e-03 |
| POU3F3\_1 |  | ceh-18 tbp-1 | 97 | 1.35 | 2.2e-03 |
| pTH6019 |  | nhr-19 (0.69) nhr-2 nhr-213 | 109 | 1.28 | 2.2e-03 |
| pTH10798 |  | Y75B8A.6 | 65 | 1.58 | 2.2e-03 |
| pTH8598 |  | slr-2 nhr-28 odr-7 nhr-273 | 66 | 1.57 | 2.4e-03 |
| pTH9246 |  | C34D1.1 | 84 | 1.42 | 2.5e-03 |
| N$SKN1\_02 |  | skn-1 | 110 | 1.27 | 2.7e-03 |
| CREM\_f1 |  | crh-1 | 77 | 1.47 | 2.7e-03 |
| pTH9244 |  | tbx-39 | 121 | 1.22 | 2.7e-03 |
| V$BRACH\_01 |  | mab-9 tbx-39 | 123 | 1.20 | 2.9e-03 |
| MA0254.1 |  | ceh-18 | 76 | 1.47 | 3.4e-03 |
| Hoxc8\_3429 |  | lin-39 | 74 | 1.48 | 3.5e-03 |
| FLI1\_f1 |  | lin-1 | 60 | 1.60 | 3.7e-03 |
| pTH9261 |  | lin-48 (0.82) dmd-3 | 54 | 1.67 | 3.8e-03 |
| V$MSX1\_01 |  | ceh-1 (0.63) | 69 | 1.52 | 3.8e-03 |
| V$CREB\_02 |  | fos-1 atf-7 crh-1 C27D6.4 | 110 | 1.26 | 3.9e-03 |
| GRHL1\_2 |  | grh-1 | 17 | 3.16 | 3.9e-03 |
| MEIS3\_2 |  | ceh-32 lin-39 | 39 | 1.93 | 3.9e-03 |
| pTH5083 |  | atf-5 (0.67) fos-1 crh-1 | 78 | 1.45 | 3.9e-03 |
| SP4\_f1 |  | klf-2 (0.54) Y53H1A.2 plp-2 | 45 | 1.79 | 4.6e-03 |
| pTH9924 |  | nhr-46 | 106 | 1.28 | 4.6e-03 |
| Tcf2\_0913 |  | hmbx-1 | 66 | 1.53 | 4.9e-03 |
| MA0545.1 |  | hlh-11 (0.72) hlh-1 (0.59) lin-32 | 86 | 1.38 | 5.1e-03 |
| RORA\_2 |  | nhr-213 | 115 | 1.23 | 5.1e-03 |
| K562\_ZBTB7A\_HudsonAlpha |  | ZC328.2 | 61 | 1.57 | 5.3e-03 |
| GM12878\_MEF2C\_HudsonAlpha |  | mef-2 | 97 | 1.32 | 5.4e-03 |
| MA0246.1 |  | dmd-4 ceh-32 | 115 | 1.23 | 5.6e-03 |
| pTH9915 |  | zip-3 crh-1 | 116 | 1.22 | 5.8e-03 |
| pTH9387 |  | C34D1.1 | 108 | 1.26 | 5.8e-03 |
| pTH9142 |  | C34D1.1 | 114 | 1.23 | 5.9e-03 |
| V$ZIC1\_01 |  | ztf-14 pax-3 ref-2 | 100 | 1.30 | 5.9e-03 |
| MA0452.2 |  | B0310.2 (0.69) ZK177.3 | 88 | 1.36 | 6.1e-03 |
| GM12878\_PAX5C20\_HudsonAlpha |  | pax-3 pax-2 | 106 | 1.27 | 6.2e-03 |
| pTH10623 |  | scrt-1 | 78 | 1.42 | 6.5e-03 |
| HeLa-S3\_TR4\_UCD |  | nhr-19 (0.69) lin-1 C24A1.2 | 72 | 1.46 | 6.7e-03 |
| pTH10015 |  | ztf-28 che-1 | 72 | 1.46 | 6.7e-03 |
| THRB\_3 |  | nhr-15 nhr-239 | 43 | 1.79 | 6.8e-03 |
| pTH10034 |  | nhr-66 | 38 | 1.89 | 6.9e-03 |
| Egr1\_2580 |  | ZC328.2 | 70 | 1.48 | 7.0e-03 |
| pTH10777 |  | dmd-3 | 29 | 2.15 | 7.1e-03 |
| pTH10718 |  | egl-43 | 15 | 3.25 | 7.2e-03 |
| pTH9163 |  | nhr-3 | 71 | 1.47 | 7.5e-03 |
| Six6\_2267 |  | ceh-34 egl-27 | 123 | 1.18 | 7.6e-03 |
| MA0067.1 |  | hlh-30 (0.58) aha-1 pax-1 | 116 | 1.22 | 7.8e-03 |
| pTH6508 |  | nhr-36 | 48 | 1.70 | 7.8e-03 |
| MA0467.1 |  | ceh-45 tbx-39 | 19 | 2.72 | 7.9e-03 |
| K562\_SP2\_HudsonAlpha |  | klf-2 (0.54) | 109 | 1.25 | 8.0e-03 |
| MA0098.2 |  | lin-1 C24A1.2 | 53 | 1.63 | 8.4e-03 |
| Hoxa7\_3750 |  | lin-39 | 72 | 1.45 | 9.2e-03 |
| disco-r-Cl1\_SANGER\_5\_FBgn0042650 |  | lin-31 (0.77) F55C5.11 | 68 | 1.47 | 9.8e-03 |
| pTH6634 |  | nhr-10 nhr-2 nhr-71 | 111 | 1.23 | 1.0e-02 |
| Six2\_2307 |  | ceh-32 ceh-34 | 101 | 1.28 | 1.0e-02 |
| V$GATA3\_03 |  | elt-1 | 50 | 1.65 | 1.0e-02 |
| Hoxb6\_3428 |  | lin-39 | 64 | 1.50 | 1.0e-02 |
| pTH10807 |  | F13H6.1 (0.74) nhr-2 | 113 | 1.22 | 1.0e-02 |
| N$SKN1\_01 |  | skn-1 ceh-2 | 93 | 1.31 | 1.1e-02 |
| V$AREB6\_02 |  | ztf-6 | 117 | 1.20 | 1.1e-02 |
| tgo\_ss\_SANGER\_5\_FBgn0015014 |  | aha-1 | 33 | 1.95 | 1.1e-02 |
| V$IK2\_01 |  | F26F4.8 | 84 | 1.36 | 1.2e-02 |
| Mv75 |  | elt-1 | 118 | 1.20 | 1.2e-02 |
| Isl2\_3430 |  | lim-7 | 77 | 1.40 | 1.3e-02 |
| MA0386.1 |  | tbp-1 | 134 | 1.12 | 1.3e-02 |
| pTH9189 |  | dmd-3 | 79 | 1.38 | 1.3e-02 |
| DLX3\_do |  | ceh-43 lin-39 alr-1 mls-2 ceh-16 | 17 | 2.78 | 1.3e-02 |
| PITX3\_1 |  | ceh-53 ceh-45 | 113 | 1.22 | 1.3e-02 |
| MA0482.1 |  | elt-1 | 58 | 1.54 | 1.4e-02 |
| MA0114.2 |  | nhr-2 nhr-62 | 84 | 1.35 | 1.4e-02 |
| NDF1\_f1 |  | hlh-8 (0.8) hlh-32 ngn-1 hlh-15 | 75 | 1.41 | 1.4e-02 |
| MA0118.1 |  | ref-2 | 60 | 1.52 | 1.4e-02 |
| V$FAC1\_01 |  | gei-8 | 87 | 1.33 | 1.4e-02 |
| srp\_FlyReg\_FBgn0003507 |  | elt-1 | 103 | 1.26 | 1.5e-02 |
| PO4F2\_si |  | unc-86 | 83 | 1.36 | 1.5e-02 |
| TCF4\_2 |  | hlh-2 | 113 | 1.21 | 1.5e-02 |
| MA0139.1 |  | F58G1.2 | 105 | 1.25 | 1.6e-02 |
| Barhl1\_2 |  | ceh-31 | 61 | 1.50 | 1.6e-02 |
| pTH8566 |  | lin-54 | 56 | 1.54 | 1.6e-02 |
| IRX2\_1 |  | irx-1 | 90 | 1.31 | 1.7e-02 |
| BARHL2\_1 |  | ceh-31 | 27 | 2.09 | 1.7e-02 |
| ARNT2\_si |  | C46E10.9 aha-1 | 70 | 1.43 | 1.7e-02 |
| HeLa-S3\_ZNF274\_UCD |  | C28G1.4 | 132 | 1.13 | 1.7e-02 |
| Hoxb8\_3780 |  | lin-39 | 124 | 1.16 | 1.8e-02 |
| Six1\_0935 |  | ceh-32 | 46 | 1.65 | 1.8e-02 |
| SPDEF\_3 |  | lin-1 | 80 | 1.36 | 1.8e-02 |
| pTH10823 |  | B0310.2 (0.69) | 72 | 1.41 | 1.9e-02 |
| MYB\_f1 |  | D1081.8 | 38 | 1.78 | 1.9e-02 |
| pTH3998 |  | tbx-39 | 42 | 1.70 | 2.0e-02 |
| pTH9326 |  | nhr-122 | 67 | 1.44 | 2.0e-02 |
| Irx5\_2385 |  | irx-1 | 102 | 1.25 | 2.2e-02 |
| Gmeb1\_1745 |  | attf-1 | 96 | 1.28 | 2.2e-02 |
| pTH3997 |  | C04F5.9 | 103 | 1.24 | 2.2e-02 |
| pTH9900 |  | C46E10.8 | 132 | 1.12 | 2.3e-02 |
| Poxm\_SOLEXA\_5\_FBgn0003129 |  | pax-2 | 103 | 1.24 | 2.3e-02 |
| V$CDXA\_01 |  | ceh-13 | 30 | 1.94 | 2.3e-02 |
| Emx2\_3420 |  | ceh-2 | 61 | 1.48 | 2.3e-02 |
| Hmbox1\_2674 |  | hmbx-1 | 111 | 1.21 | 2.4e-02 |
| pTH2283 |  | odd-2 | 33 | 1.85 | 2.5e-02 |
| pTH6516 |  | F19F10.1 | 88 | 1.31 | 2.5e-02 |
| V$TCF11\_01 |  | skn-1 | 121 | 1.17 | 2.5e-02 |
| pTH2684 |  | fos-1 | 112 | 1.20 | 2.5e-02 |
| NR5A2\_f1 |  | nhr-68 | 117 | 1.18 | 2.5e-02 |
| Cutl1\_3494 |  | ceh-44 | 54 | 1.53 | 2.5e-02 |
| GABPA\_f1 |  | lin-1 C24A1.2 | 30 | 1.92 | 2.7e-02 |
| Tcf7\_0950 |  | pop-1 | 58 | 1.49 | 2.7e-02 |
| pTH1001 |  | dnj-17 | 65 | 1.44 | 2.7e-02 |
| pTH9907 |  | nhr-34 (0.66) | 26 | 2.04 | 2.7e-02 |
| ELF3\_f1 |  | C24A1.2 | 79 | 1.35 | 2.7e-02 |
| pTH3751 |  | tbx-39 | 81 | 1.34 | 2.8e-02 |
| pTH5887 |  | lin-39 | 110 | 1.21 | 2.8e-02 |
| Hoxa7\_2668 |  | lin-39 | 75 | 1.37 | 2.8e-02 |
| SOX10\_1 |  | K11D2.4 sox-4 | 44 | 1.64 | 2.8e-02 |
| CG4854\_SANGER\_10\_FBgn0038766 |  | K11D2.4 | 70 | 1.40 | 2.9e-02 |
| pTH6478 |  | lim-7 | 64 | 1.44 | 2.9e-02 |
| pTH10768 |  | med-2 | 105 | 1.23 | 3.0e-02 |
| V$BRN2\_01 |  | ceh-18 | 54 | 1.52 | 3.0e-02 |
| pTH10028 |  | nhr-204 | 48 | 1.58 | 3.0e-02 |
| pTH9044 |  | nhr-177 | 68 | 1.41 | 3.1e-02 |
| pTH9135 |  | pop-1 | 52 | 1.54 | 3.1e-02 |
| pTH3064 |  | crh-1 | 34 | 1.79 | 3.2e-02 |
| pTH5928 |  | ceh-34 | 101 | 1.24 | 3.2e-02 |
| Irx3\_0920 |  | irx-1 | 40 | 1.68 | 3.2e-02 |
| CG14962\_SANGER\_5\_FBgn0035407 |  | C34H4.5 | 25 | 2.05 | 3.2e-02 |
| Meis2\_1 |  | ceh-32 ces-1 | 76 | 1.36 | 3.3e-02 |
| pTH1292 |  | pzf-1 (-0.56) | 42 | 1.65 | 3.3e-02 |
| Hr51\_SANGER\_5\_FBgn0034012 |  | nhr-100 | 30 | 1.88 | 3.3e-02 |
| Foxj1\_3125 |  | lin-31 (0.77) | 141 | 1.06 | 3.4e-02 |
| Tcf1\_2666 |  | hmbx-1 | 30 | 1.88 | 3.4e-02 |
| MA0164.1 |  | nhr-100 | 55 | 1.50 | 3.4e-02 |
| SOX2\_4 |  | grh-1 sox-4 | 112 | 1.20 | 3.4e-02 |
| pTH9300 |  | dmd-3 | 69 | 1.39 | 3.5e-02 |
| I$UBX\_01 |  | lin-39 | 59 | 1.46 | 3.6e-02 |
| pTH5714 |  | nhr-239 | 70 | 1.39 | 3.6e-02 |
| pTH5078 |  | ces-2 | 64 | 1.42 | 3.6e-02 |
| Hoxa2\_3079 |  | lin-39 | 66 | 1.41 | 3.6e-02 |
| Irx3\_2226 |  | irx-1 | 112 | 1.19 | 3.7e-02 |
| pTH5922 |  | ceh-24 | 19 | 2.32 | 3.7e-02 |
| MAFA\_f1 |  | F45H11.6 | 118 | 1.17 | 3.7e-02 |
| V$EN1\_01 |  | ceh-16 | 115 | 1.18 | 3.7e-02 |
| Hoxc5\_2630 |  | npax-3 lin-39 | 125 | 1.14 | 3.8e-02 |
| pTH9164 |  | ceh-26 | 91 | 1.28 | 3.8e-02 |
| Tcf3\_3787 |  | pop-1 | 18 | 2.38 | 3.8e-02 |
| V$SRF\_Q6 |  | unc-120 (0.74) | 55 | 1.49 | 3.9e-02 |
| pTH6449 |  | ceh-43 | 71 | 1.38 | 3.9e-02 |
| pTH10788 |  | tbx-33 | 134 | 1.10 | 3.9e-02 |
| FOXJ3\_si |  | lin-31 (0.77) | 95 | 1.26 | 4.0e-02 |
| pTH9149 |  | ztf-30 (0.75) | 42 | 1.63 | 4.0e-02 |
| pTH9182 |  | tbx-39 | 114 | 1.18 | 4.0e-02 |
| pTH9076 |  | C01G12.1 | 19 | 2.30 | 4.0e-02 |
| MA0594.1 |  | lin-39 | 102 | 1.23 | 4.1e-02 |
| Hoxa3\_2783 |  | lin-39 | 64 | 1.42 | 4.1e-02 |
| GATA3\_si |  | elt-1 | 120 | 1.16 | 4.2e-02 |
| MA0543.1 |  | eor-1 | 68 | 1.39 | 4.3e-02 |
| RFX5\_2 |  | daf-19 | 45 | 1.58 | 4.4e-02 |
| pTH7875 |  | mel-28 | 112 | 1.19 | 4.4e-02 |
| pTH10630 |  | lsy-27 | 44 | 1.59 | 4.4e-02 |
| V$CETS1P54\_02 |  | C52B9.2 | 87 | 1.29 | 4.4e-02 |
| pTH9934 |  | Y53H1A.2 | 101 | 1.23 | 4.4e-02 |
| Jundm2\_0911 |  | fos-1 | 38 | 1.67 | 4.5e-02 |
| I$ABDB\_01 |  | ceh-24 | 116 | 1.17 | 4.6e-02 |
| Hoxb5\_3122 |  | lin-39 | 118 | 1.16 | 4.6e-02 |
| Elf5 |  | C24A1.2 | 52 | 1.50 | 4.6e-02 |
| pTH8996 |  | sma-4 | 83 | 1.30 | 4.6e-02 |
| Titf1\_1722 |  | dsc-1 (0.91) | 110 | 1.19 | 4.7e-02 |
| CG31670\_SOLEXA\_5\_FBgn0031375 |  | CELE\_Y38H8A.5 | 82 | 1.31 | 4.7e-02 |
| Max\_Mnt\_SANGER\_5\_FBgn0017578 |  | hlh-30 (0.58) mxl-1 | 33 | 1.76 | 4.8e-02 |
| MA0124.1 |  | ceh-24 | 64 | 1.40 | 4.9e-02 |
| V$HEN1\_01 |  | hlh-15 | 10 | 3.38 | 5.0e-02 |

### Correlated (and anti-correlated) transcription factors

|  |  |
| --- | --- |
| **Transcription factor** | **Correlation** |
| dsc-1 | 0.91 |
| moe-3 | 0.84 |
| mls-1 | 0.84 |
| lin-48 | 0.82 |
| hlh-8 | 0.80 |
| lin-31 | 0.77 |
| fkh-9 | 0.76 |
| pat-9 | 0.75 |
| ztf-30 | 0.75 |
| elt-3 | 0.75 |
| npax-2 | 0.75 |
| nhr-43 | 0.75 |
| unc-120 | 0.74 |
| F13H6.1 | 0.74 |
| hlh-11 | 0.72 |
| nhr-69 | 0.71 |
| nhr-97 | 0.71 |
| nhr-260 | 0.70 |
| cog-1 | 0.70 |
| nhr-19 | 0.69 |
| B0310.2 | 0.69 |
| nhr-181 | 0.69 |
| nhr-120 | 0.68 |
| unc-98 | 0.68 |
| blmp-1 | 0.67 |
| nfya-2 | -0.46 |
| madf-8 | -0.46 |
| hmg-5 | -0.47 |
| nhr-269 | -0.48 |
| wrm-1 | -0.48 |
| Y54G2A.20 | -0.48 |
| ztf-15 | -0.50 |
| zim-3 | -0.51 |
| nhr-220 | -0.52 |
| madf-7 | -0.52 |
| K11D12.12 | -0.52 |
| nhr-276 | -0.52 |
| R144.3 | -0.52 |
| hmg-3 | -0.54 |
| repo-1 | -0.54 |
| K11H3.4 | -0.54 |
| nfyc-1 | -0.54 |
| ccch-3 | -0.54 |
| pzf-1 | -0.56 |
| F37B4.10 | -0.59 |
| zip-8 | -0.60 |
| cep-1 | -0.60 |
| D2030.7 | -0.65 |
| ztf-4 | -0.73 |
| snu-23 | -0.79 |

### ChIP peaks enriched

none found
