# Supplementary material for: Overlapping cell population expression profiling and regulatory inference in C. elegans
Source: BMC Genomics. 2016 Feb 29;17:159. doi: 10.1186/s12864-016-2482-z (PMC4772325; doi:10.1186/s12864-016-2482-z)
Supplement: Additional file 13: — Web supplement. (DOC 21 kb) [file 12864_2016_2482_MOESM13_ESM.zip › sortWeb/clusters/hier.300.clusters/223.html]

Cluster 223 

## Cluster 223

### Expression

| cnd-1 rep. 1 | cnd-1 rep. 2 | cnd-1 rep. 3 | pha-4 rep. 1 | pha-4 rep. 2 | pha-4 rep. 3 | ceh-27 | ceh-36 | ceh-6 | F21D5.9 | mir-57 | mls-2 | pal-1 | pros-1 | ttx-3 | unc-130 | hlh-16 | irx-1 | ceh-6 (+) hlh-16 (+) | ceh-6 (+) hlh-16 (-) | ceh-6 (-) hlh-16 (+) | cnd-1 singlets | pha-4 singlets | 0 | 60 | 120 | 150 | 180 | 240 | 330 | 390 | 420 | 480 | 540 | 570 | 600 | 630 | 660 | NAME | Functional description |
| --- | --- | --- | --- | --- | --- | --- | --- | --- | --- | --- | --- | --- | --- | --- | --- | --- | --- | --- | --- | --- | --- | --- | --- | --- | --- | --- | --- | --- | --- | --- | --- | --- | --- | --- | --- | --- | --- | --- | --- |
|  |  |  |  |  |  |  |  |  |  |  |  |  |  |  |  |  |  |  |  |  |  |  |  |  |  |  |  |  |  |  |  |  |  |  |  |  |  | Y53G8AM.8 |  |
|  |  |  |  |  |  |  |  |  |  |  |  |  |  |  |  |  |  |  |  |  |  |  |  |  |  |  |  |  |  |  |  |  |  |  |  |  |  | R03H10.2 |  |
|  |  |  |  |  |  |  |  |  |  |  |  |  |  |  |  |  |  |  |  |  |  |  |  |  |  |  |  |  |  |  |  |  |  |  |  |  |  | *ceh-37* | C. Elegans Homeobox |
|  |  |  |  |  |  |  |  |  |  |  |  |  |  |  |  |  |  |  |  |  |  |  |  |  |  |  |  |  |  |  |  |  |  |  |  |  |  | F28F5.t1 |  |
|  |  |  |  |  |  |  |  |  |  |  |  |  |  |  |  |  |  |  |  |  |  |  |  |  |  |  |  |  |  |  |  |  |  |  |  |  |  | *elk-2* | ELK transcription factor homolog |
|  |  |  |  |  |  |  |  |  |  |  |  |  |  |  |  |  |  |  |  |  |  |  |  |  |  |  |  |  |  |  |  |  |  |  |  |  |  | *prdx-2* | PeRoxireDoXin |
|  |  |  |  |  |  |  |  |  |  |  |  |  |  |  |  |  |  |  |  |  |  |  |  |  |  |  |  |  |  |  |  |  |  |  |  |  |  | C31B8.8 |  |
|  |  |  |  |  |  |  |  |  |  |  |  |  |  |  |  |  |  |  |  |  |  |  |  |  |  |  |  |  |  |  |  |  |  |  |  |  |  | *abts-4* | Anion/Bicarbonate TranSporter family |
|  |  |  |  |  |  |  |  |  |  |  |  |  |  |  |  |  |  |  |  |  |  |  |  |  |  |  |  |  |  |  |  |  |  |  |  |  |  | *fasn-1* | Fatty Acid SyNthase |
|  |  |  |  |  |  |  |  |  |  |  |  |  |  |  |  |  |  |  |  |  |  |  |  |  |  |  |  |  |  |  |  |  |  |  |  |  |  | *fbn-1* | FiBrilliN homolog |
|  |  |  |  |  |  |  |  |  |  |  |  |  |  |  |  |  |  |  |  |  |  |  |  |  |  |  |  |  |  |  |  |  |  |  |  |  |  | T08B1.4 |  |
|  |  |  |  |  |  |  |  |  |  |  |  |  |  |  |  |  |  |  |  |  |  |  |  |  |  |  |  |  |  |  |  |  |  |  |  |  |  | F10D7.2 |  |
|  |  |  |  |  |  |  |  |  |  |  |  |  |  |  |  |  |  |  |  |  |  |  |  |  |  |  |  |  |  |  |  |  |  |  |  |  |  | *vab-2* | Variable ABnormal morphology |
|  |  |  |  |  |  |  |  |  |  |  |  |  |  |  |  |  |  |  |  |  |  |  |  |  |  |  |  |  |  |  |  |  |  |  |  |  |  | Y41G9A.5 |  |
|  |  |  |  |  |  |  |  |  |  |  |  |  |  |  |  |  |  |  |  |  |  |  |  |  |  |  |  |  |  |  |  |  |  |  |  |  |  | *vab-7* | Variable ABnormal morphology |
|  |  |  |  |  |  |  |  |  |  |  |  |  |  |  |  |  |  |  |  |  |  |  |  |  |  |  |  |  |  |  |  |  |  |  |  |  |  | *sago-1* | Synthetic secondary siRNA-deficient ArGOnaute mutant |
|  |  |  |  |  |  |  |  |  |  |  |  |  |  |  |  |  |  |  |  |  |  |  |  |  |  |  |  |  |  |  |  |  |  |  |  |  |  | *lin-1* | abnormal cell LINeage |
|  |  |  |  |  |  |  |  |  |  |  |  |  |  |  |  |  |  |  |  |  |  |  |  |  |  |  |  |  |  |  |  |  |  |  |  |  |  | *tag-260* | Temporarily Assigned Gene name |
|  |  |  |  |  |  |  |  |  |  |  |  |  |  |  |  |  |  |  |  |  |  |  |  |  |  |  |  |  |  |  |  |  |  |  |  |  |  | Y39G10AR.16 |  |
|  |  |  |  |  |  |  |  |  |  |  |  |  |  |  |  |  |  |  |  |  |  |  |  |  |  |  |  |  |  |  |  |  |  |  |  |  |  | *ptr-23* | PaTched Related family |
|  |  |  |  |  |  |  |  |  |  |  |  |  |  |  |  |  |  |  |  |  |  |  |  |  |  |  |  |  |  |  |  |  |  |  |  |  |  | D1005.1 |  |
|  |  |  |  |  |  |  |  |  |  |  |  |  |  |  |  |  |  |  |  |  |  |  |  |  |  |  |  |  |  |  |  |  |  |  |  |  |  | Y51A2D.21 |  |
|  |  |  |  |  |  |  |  |  |  |  |  |  |  |  |  |  |  |  |  |  |  |  |  |  |  |  |  |  |  |  |  |  |  |  |  |  |  | T14B4.5 |  |
|  |  |  |  |  |  |  |  |  |  |  |  |  |  |  |  |  |  |  |  |  |  |  |  |  |  |  |  |  |  |  |  |  |  |  |  |  |  | C29E4.15 |  |
|  |  |  |  |  |  |  |  |  |  |  |  |  |  |  |  |  |  |  |  |  |  |  |  |  |  |  |  |  |  |  |  |  |  |  |  |  |  | Y57A10A.23 |  |
|  |  |  |  |  |  |  |  |  |  |  |  |  |  |  |  |  |  |  |  |  |  |  |  |  |  |  |  |  |  |  |  |  |  |  |  |  |  | *aagr-4* | Acid Alpha Glucosidase Relate |
|  |  |  |  |  |  |  |  |  |  |  |  |  |  |  |  |  |  |  |  |  |  |  |  |  |  |  |  |  |  |  |  |  |  |  |  |  |  | C02D5.4 |  |
|  |  |  |  |  |  |  |  |  |  |  |  |  |  |  |  |  |  |  |  |  |  |  |  |  |  |  |  |  |  |  |  |  |  |  |  |  |  | *ztf-30* | Zinc finger putative Transcription Factor family |
|  |  |  |  |  |  |  |  |  |  |  |  |  |  |  |  |  |  |  |  |  |  |  |  |  |  |  |  |  |  |  |  |  |  |  |  |  |  | T04G9.4 |  |
|  |  |  |  |  |  |  |  |  |  |  |  |  |  |  |  |  |  |  |  |  |  |  |  |  |  |  |  |  |  |  |  |  |  |  |  |  |  | *lin-26* | abnormal cell LINeage |
|  |  |  |  |  |  |  |  |  |  |  |  |  |  |  |  |  |  |  |  |  |  |  |  |  |  |  |  |  |  |  |  |  |  |  |  |  |  | *lir-1* | LIn-26 Related |
|  |  |  |  |  |  |  |  |  |  |  |  |  |  |  |  |  |  |  |  |  |  |  |  |  |  |  |  |  |  |  |  |  |  |  |  |  |  | *cki-2* | CKI family (Cyclin-dependent Kinase Inhibitor) |
|  |  |  |  |  |  |  |  |  |  |  |  |  |  |  |  |  |  |  |  |  |  |  |  |  |  |  |  |  |  |  |  |  |  |  |  |  |  | R05D3.9 |  |
|  |  |  |  |  |  |  |  |  |  |  |  |  |  |  |  |  |  |  |  |  |  |  |  |  |  |  |  |  |  |  |  |  |  |  |  |  |  | *ctg-1* | CRAL/TRIO and GOLD domain containing |
|  |  |  |  |  |  |  |  |  |  |  |  |  |  |  |  |  |  |  |  |  |  |  |  |  |  |  |  |  |  |  |  |  |  |  |  |  |  | F56B3.2 |  |
|  |  |  |  |  |  |  |  |  |  |  |  |  |  |  |  |  |  |  |  |  |  |  |  |  |  |  |  |  |  |  |  |  |  |  |  |  |  | *cutl-25* | CUTiclin-Like |
|  |  |  |  |  |  |  |  |  |  |  |  |  |  |  |  |  |  |  |  |  |  |  |  |  |  |  |  |  |  |  |  |  |  |  |  |  |  | *noah-2* | NOmpA Homolog (Drosophila nompA: no mechanoreceptor potential A) |
|  |  |  |  |  |  |  |  |  |  |  |  |  |  |  |  |  |  |  |  |  |  |  |  |  |  |  |  |  |  |  |  |  |  |  |  |  |  | *dpy-11* | DumPY: shorter than wild-type |
|  |  |  |  |  |  |  |  |  |  |  |  |  |  |  |  |  |  |  |  |  |  |  |  |  |  |  |  |  |  |  |  |  |  |  |  |  |  | Y71G12B.6 |  |
|  |  |  |  |  |  |  |  |  |  |  |  |  |  |  |  |  |  |  |  |  |  |  |  |  |  |  |  |  |  |  |  |  |  |  |  |  |  | D1005.2 |  |
|  |  |  |  |  |  |  |  |  |  |  |  |  |  |  |  |  |  |  |  |  |  |  |  |  |  |  |  |  |  |  |  |  |  |  |  |  |  | *dpy-18* | DumPY: shorter than wild-type |
|  |  |  |  |  |  |  |  |  |  |  |  |  |  |  |  |  |  |  |  |  |  |  |  |  |  |  |  |  |  |  |  |  |  |  |  |  |  | *wrt-10* | WaRThog (hedgehog-like family) |
|  |  |  |  |  |  |  |  |  |  |  |  |  |  |  |  |  |  |  |  |  |  |  |  |  |  |  |  |  |  |  |  |  |  |  |  |  |  | *sym-1* | SYnthetic lethal with Mec |
|  |  |  |  |  |  |  |  |  |  |  |  |  |  |  |  |  |  |  |  |  |  |  |  |  |  |  |  |  |  |  |  |  |  |  |  |  |  | *noah-1* | NOmpA Homolog (Drosophila nompA: no mechanoreceptor potential A) |
|  |  |  |  |  |  |  |  |  |  |  |  |  |  |  |  |  |  |  |  |  |  |  |  |  |  |  |  |  |  |  |  |  |  |  |  |  |  | *cutl-2* | CUTiclin-Like |
|  |  |  |  |  |  |  |  |  |  |  |  |  |  |  |  |  |  |  |  |  |  |  |  |  |  |  |  |  |  |  |  |  |  |  |  |  |  | F46E10.2 |  |
|  |  |  |  |  |  |  |  |  |  |  |  |  |  |  |  |  |  |  |  |  |  |  |  |  |  |  |  |  |  |  |  |  |  |  |  |  |  | C01F1.3 |  |
|  |  |  |  |  |  |  |  |  |  |  |  |  |  |  |  |  |  |  |  |  |  |  |  |  |  |  |  |  |  |  |  |  |  |  |  |  |  | *hch-1* | defective HatCHing |
|  |  |  |  |  |  |  |  |  |  |  |  |  |  |  |  |  |  |  |  |  |  |  |  |  |  |  |  |  |  |  |  |  |  |  |  |  |  | C34E7.4 |  |
|  |  |  |  |  |  |  |  |  |  |  |  |  |  |  |  |  |  |  |  |  |  |  |  |  |  |  |  |  |  |  |  |  |  |  |  |  |  | *dsl-3* | Delta/Serrate/Lag-2 domain |
|  |  |  |  |  |  |  |  |  |  |  |  |  |  |  |  |  |  |  |  |  |  |  |  |  |  |  |  |  |  |  |  |  |  |  |  |  |  | Y41D4B.26 |  |
|  |  |  |  |  |  |  |  |  |  |  |  |  |  |  |  |  |  |  |  |  |  |  |  |  |  |  |  |  |  |  |  |  |  |  |  |  |  | C09F9.2 |  |
|  |  |  |  |  |  |  |  |  |  |  |  |  |  |  |  |  |  |  |  |  |  |  |  |  |  |  |  |  |  |  |  |  |  |  |  |  |  | K11H3.3 |  |
|  |  |  |  |  |  |  |  |  |  |  |  |  |  |  |  |  |  |  |  |  |  |  |  |  |  |  |  |  |  |  |  |  |  |  |  |  |  | *prx-14* | PeRoXisome assembly factor |
|  |  |  |  |  |  |  |  |  |  |  |  |  |  |  |  |  |  |  |  |  |  |  |  |  |  |  |  |  |  |  |  |  |  |  |  |  |  | *snf-6* | Sodium: Neurotransmitter symporter Family |
|  |  |  |  |  |  |  |  |  |  |  |  |  |  |  |  |  |  |  |  |  |  |  |  |  |  |  |  |  |  |  |  |  |  |  |  |  |  | F25E5.5 |  |

### Phenotypes enriched

none found

### Anatomy terms enriched

none found

### GO terms enriched

none found

### Expression clusters enriched

|  |  |  |  |
| --- | --- | --- | --- |
| **Group name** | **Number in cluster** | **Enrichment** | **FDR corrected p** |
| Genes significantly enriched (> 2x, FDR < 5%) in a particular cell-type versus a reference sample of all cells at the same stage. WBPaper00037950:hypodermis\_embryo\_enriched | 22 | 8.39 | 2.11e-12 |
| Genes that show selective expression in a subset of cell types vs broadly expressed in many cell types. Correspond to 20% - 57% of enriched\_genes for a given cell type. WBPaper00037950:hypodermis\_embryo\_SelectivelyEnriched | 17 | 13.30 | 3.94e-12 |
| C-lineage related expression profile. WBPaper00025032:cluster\_24 | 9 | 53.63 | 5.57e-11 |
| Genes upregulated in dcr-1(-/-) adult animals by at least 1.5 fold and P < 0.01, as determined by a multisample t-test and the Benjamini and Hochberg false discovery rate correction. | 23 | 5.99 | 4.25e-10 |
| Candidate daf-19 up regulated genes with a statistically significant signal variation of 1.5-fold or greater. These were identified using a class comparisons tool from BRB Array Tools. | 15 | 11.17 | 2.02e-09 |
| Genes that showed lower expression in N2 than in DR1350. | 21 | 5.01 | 1.36e-07 |
| Early embryonic development gene expression profile. [cgc5767]:cluster\_12 | 7 | 41.71 | 1.89e-07 |
| Total muscle depleted genes (complete list of non-overlapping genes from the 0hr and 24hr muscle depleted datasets). | 29 | 3.26 | 3.17e-07 |
| Genes depleted in muscle cells (24hr muscle dataset). Dissociated myo-3::GFP embryos were cultured for 24 hours before FACS sorting. | 23 | 4.05 | 8.22e-07 |
| Genes down-regulated after 200 um Quercetin treatment. Fold change < 0.8. | 21 | 4.51 | 8.52e-07 |
| Candidate daf-19 up regulated genes with a statistically significant signal variation of 1.5-fold or greater. These were identified using a Significance Analysis of Microarrays (SAM). | 7 | 32.67 | 1.01e-06 |
| WT-Pico Pan-neural Depleted Genes, with genes found multiple times in a single dataset removed (without dups). | 20 | 4.46 | 2.76e-06 |
| Potential PAL-1 target genes. | 9 | 15.66 | 2.83e-06 |
| Genes down-regulated after 100 um Quercetin treatment. Fold change < 0.8. | 19 | 4.38 | 9.28e-06 |
| Gene significantly up-regulated by treatment with 0.2mM of HuminFeed until older adult stage (11 days), with a minimum fold change in gene expression of 1.25. | 12 | 7.98 | 1.17e-05 |
| Genes with expression level up regulated after treatment with Methylmercury (MeHg) by RNAseq analysis. | 12 | 6.40 | 1.08e-04 |
| Germline-enriched and sex-biased expression profile cluster B. | 14 | 5.21 | 1.13e-04 |
| Genes down regulated in the absence of TDP-1, when the threshold was set at a fold change (FC) of 1.5. | 11 | 7.20 | 1.15e-04 |
| Genes that showed decreased expression in nhr-23 RNAi experiment. | 9 | 9.62 | 1.41e-04 |
| Genes dowm regulated in the dauer versus dauer-exit worms. | 11 | 7.03 | 1.43e-04 |
| Genes up regulated in crh-1(nn3315) comparing to in N2. | 12 | 6.04 | 1.92e-04 |
| Developmentally modulated gene cluster. cgc4386\_cluster\_4\_6 | 6 | 19.31 | 2.54e-04 |
| Developmentally modulated gene cluster. cgc4386\_cluster\_6\_1 | 7 | 13.90 | 2.61e-04 |
| Genes downregulated by fer-1 mutants hc1 and hc24, with > 4 fold change in expression level. | 8 | 10.23 | 3.93e-04 |
| Genes up regulated by mir-243(n4759). | 20 | 3.11 | 6.92e-04 |
| Embryonic class (E): genes that significantly increase in abundance at some point during embryogenesis. | 29 | 2.28 | 7.29e-04 |
| C-lineage related expression profile. WBPaper00025032:cluster\_50 | 4 | 41.49 | 8.64e-04 |
| Genes down regulated in the absence of TDP-1, when the threshold was set at a fold change (FC) of 1.2. | 23 | 2.70 | 9.48e-04 |
| Genes significantly enriched (> 2x, FDR < 5%) in a particular cell-type versus a reference sample of all cells at both embryonic and larval stages. WBPaper00037950:hypodermis\_CoreEnriched | 7 | 11.08 | 1.06e-03 |
| Genes depleted in muscle cells (0hr muscle dataset). Dissociated myo-3::GFP embryos were cultured for 0 hours before FACS sorting. | 19 | 3.12 | 1.27e-03 |
| Strictly embryonic class (SE): genes that are the subset of embryonic genes that are not also classified as maternal. | 14 | 4.18 | 1.28e-03 |
| Genes with expression enriched in PVD and OLL neurons. Data sets were normalized by RMA and transcripts showing relative PVD enrichment (>= 1.5X) vs. the reference sample were identified by SAM analysis (False Discovery Rate, FDR < 1%). | 21 | 2.70 | 2.85e-03 |
| Genes in the top 10% of expression level across the triplicate L3 samples. To generate the top10 and bottom10 gene sets, authors ranked all genes by mean expression array signal intensity across the three replicates, then took the top and bottom deciles (1,841 genes each) to represent genes with high and low expression. | 19 | 2.89 | 3.36e-03 |
| Early embryonic development gene expression profile. [cgc5767]:cluster\_5 | 6 | 11.28 | 4.36e-03 |
| Genes downregulated on Comamonas DA1877 relative to E. coli OP50, Young adult | 8 | 7.11 | 4.44e-03 |
| Strictly embryonic (SE) subclasses are based on the earliest significant increase(abbreviated pi for primary increase). [cgc5767]:expression\_class\_SE\_pi(186\_min) | 4 | 21.14 | 1.01e-02 |
| Genes that show selective expression in a subset of cell types vs broadly expressed in many cell types. Correspond to 20% - 57% of enriched\_genes for a given cell type. WBPaper00037950:hypodermis\_larva\_SelectivelyEnriched | 10 | 4.75 | 1.05e-02 |
| A large cluster of genes up-regulated during early larval development.. | 18 | 2.75 | 1.09e-02 |
| Embryonic (E) subclasses are based on the earliest significant increase(abbreviated pi for primary increase). [cgc5767]:expression\_class\_E\_pi(122\_min) | 8 | 6.10 | 1.17e-02 |
| Genes upregulated in sma-2 L4 (3 arrays) or sma-4 L4 (1 array) vs. N2 L4. | 9 | 4.87 | 2.03e-02 |
| Genes that change expression level at day 8 adult stage after ash-2 RNAi. | 9 | 4.58 | 3.04e-02 |
| Genome-wide analysis of developmental and sex-regulated gene expression profile. cgc4489\_group\_8 | 9 | 4.45 | 3.71e-02 |
| Genes that showed decreased expression after 24 hours of infection by fungi Drechmeria coniospora. | 5 | 9.40 | 4.14e-02 |

### Motifs enriched

|  |  |  |  |  |  |
| --- | --- | --- | --- | --- | --- |
| **Motif** | **Logo** | **Possible orthologs** | **Number of motifs in cluster** | **Enrichment** | **FDR corrected p** |
| NR2F1\_3 |  | nhr-213 nhr-239 nhr-2 | 26 | 3.67 | 4.3e-07 |
| pTH6142 |  | nhr-69 (0.61) nhr-213 nhr-19 nhr-2 nhr-10 | 25 | 3.62 | 1.3e-06 |
| pTH4269 |  | nhr-177 F13H6.1 nhr-2 nhr-86 | 25 | 3.54 | 1.9e-06 |
| STF1\_f1 |  | nhr-68 | 23 | 3.64 | 5.7e-06 |
| pTH5667 |  | nhr-213 nhr-68 nhr-71 nhr-2 nhr-6 nhr-10 | 24 | 3.41 | 7.9e-06 |
| NR2F1\_4 |  | nhr-2 | 22 | 3.21 | 8.2e-05 |
| Eip75B\_SANGER\_5\_FBgn0000568 |  | nhr-213 nhr-118 | 18 | 3.65 | 2.1e-04 |
| pTH7032 |  | F52B11.1 | 47 | 1.57 | 3.7e-04 |
| pTH2936 |  | nhr-239 | 52 | 1.37 | 1.4e-03 |
| HLF\_si |  | ces-2 | 16 | 3.42 | 1.5e-03 |
| MA0146.2 |  | F58G1.2 | 43 | 1.59 | 1.9e-03 |
| Osr2\_1727 |  | odd-1 | 35 | 1.84 | 2.0e-03 |
| V$IK2\_01 |  | F26F4.8 | 40 | 1.63 | 3.6e-03 |
| pTH10654 |  | ceh-90 | 44 | 1.53 | 3.8e-03 |
| Abd-B\_FlyReg\_FBgn0000015 |  | ceh-24 | 53 | 1.30 | 3.8e-03 |
| pTH5714 |  | nhr-239 | 18 | 2.83 | 3.9e-03 |
| V$LYF1\_01 |  | F26F4.8 | 44 | 1.52 | 4.4e-03 |
| MA0162.2 |  | ZC328.2 | 27 | 2.07 | 5.2e-03 |
| pTH2933 |  | F58G1.2 | 40 | 1.60 | 5.7e-03 |
| ftz-f1\_FlyReg\_FBgn0001078 |  | nhr-68 | 32 | 1.84 | 6.0e-03 |
| MA0594.1 |  | lin-39 (0.54) | 47 | 1.42 | 6.4e-03 |
| pTH8996 |  | hlh-8 sma-4 | 54 | 1.25 | 7.5e-03 |
| V$TAXCREB\_02 |  | crh-1 | 22 | 2.27 | 9.0e-03 |
| pTH5033 |  | hlh-8 | 32 | 1.79 | 9.7e-03 |
| pTH5561 |  | nhr-239 | 32 | 1.79 | 9.7e-03 |
| SOX10\_1 |  | sox-4 | 39 | 1.58 | 9.8e-03 |
| SOX2\_1 |  | sox-4 | 48 | 1.38 | 1.0e-02 |
| Hr46\_FlyReg\_FBgn0000448 |  | nhr-213 | 22 | 2.24 | 1.1e-02 |
| MA0488.1 |  | crh-1 | 13 | 3.30 | 1.1e-02 |
| BMAL1\_f1 |  | aha-1 | 49 | 1.35 | 1.1e-02 |
| Mv73 |  | elt-1 (0.54) | 32 | 1.77 | 1.1e-02 |
| pTH9708 |  | ceh-34 | 26 | 2.00 | 1.2e-02 |
| YLR176C\_1478 |  | daf-19 | 45 | 1.43 | 1.2e-02 |
| pTH5778 |  | egl-5 (0.68) | 10 | 4.14 | 1.2e-02 |
| Mw140 |  | efl-1 | 30 | 1.82 | 1.3e-02 |
| scrt\_SANGER\_2.5\_FBgn0004880 |  | ces-1 | 51 | 1.30 | 1.5e-02 |
| V$AREB6\_04 |  | ztf-6 | 44 | 1.44 | 1.5e-02 |
| FOXO1\_3 |  | daf-16 ZC328.2 | 12 | 3.35 | 1.7e-02 |
| NR4A2\_1 |  | nhr-71 nhr-6 | 7 | 5.76 | 1.8e-02 |
| EGR4\_f1 |  | klf-2 ZC328.2 | 45 | 1.40 | 2.0e-02 |
| ETV5\_f1 |  | lin-1 (0.79) | 36 | 1.60 | 2.0e-02 |
| pTH9049 |  | ztf-2 | 42 | 1.46 | 2.1e-02 |
| SP4\_f1 |  | klf-2 | 40 | 1.50 | 2.1e-02 |
| pTH9249 |  | daf-19 | 22 | 2.09 | 2.3e-02 |
| V$GATA1\_06 |  | elt-1 (0.54) | 27 | 1.86 | 2.3e-02 |
| pTH9073 |  | elt-1 (0.54) end-3 | 40 | 1.49 | 2.3e-02 |
| pTH10722 |  | ref-2 egrh-3 | 31 | 1.71 | 2.4e-02 |
| CUX1\_2 |  | ceh-48 | 29 | 1.77 | 2.6e-02 |
| Mv88 |  | mef-2 | 36 | 1.57 | 2.7e-02 |
| pTH10811 |  | nhr-216 | 7 | 5.24 | 2.7e-02 |
| K562\_ETS1\_HudsonAlpha |  | lin-1 (0.79) | 33 | 1.63 | 3.0e-02 |
| pTH5437 |  | ceh-34 | 45 | 1.38 | 3.1e-02 |
| Hnf4a\_2640 |  | nhr-62 | 47 | 1.34 | 3.2e-02 |
| T-47D\_GATA3\_HudsonAlpha |  | elt-1 (0.54) | 20 | 2.14 | 3.3e-02 |
| CG4854\_SANGER\_10\_FBgn0038766 |  | K11D2.4 | 56 | 1.15 | 3.3e-02 |
| pTH9880 |  | end-1 | 9 | 3.88 | 3.3e-02 |
| pTH10624 |  | Y61A9LA.9 | 47 | 1.34 | 3.4e-02 |
| Hgtx\_Cell\_FBgn0040318 |  | cog-1 (0.6) | 55 | 1.18 | 3.4e-02 |
| Barhl1\_2590 |  | ceh-31 | 35 | 1.57 | 3.5e-02 |
| pTH5928 |  | ceh-34 | 43 | 1.40 | 3.6e-02 |
| Mw142 |  | egl-27 | 27 | 1.79 | 3.6e-02 |
| pTH9326 |  | nhr-122 | 39 | 1.48 | 3.6e-02 |
| MA0579.1 |  | D1081.8 | 41 | 1.44 | 3.8e-02 |
| Hoxc11\_3718 |  | pal-1 (0.53) ceh-24 | 43 | 1.40 | 3.9e-02 |
| Mw154 |  | lin-39 (0.54) | 18 | 2.24 | 3.9e-02 |
| YMR043W\_831 |  | unc-120 | 9 | 3.75 | 3.9e-02 |
| FOXO1\_2 |  | daf-16 | 22 | 1.99 | 4.0e-02 |
| pTH9340 |  | tbx-39 | 29 | 1.71 | 4.2e-02 |
| sv\_SOLEXA\_5\_FBgn0005561 |  | pax-2 | 26 | 1.81 | 4.2e-02 |
| pTH10816 |  | dmd-6 | 40 | 1.44 | 4.4e-02 |
| pTH9907 |  | nhr-34 | 31 | 1.64 | 4.4e-02 |
| POU3F4\_2 |  | ceh-18 | 36 | 1.52 | 4.6e-02 |
| Plagl1\_0972 |  | Y53H1A.2 | 22 | 1.96 | 4.7e-02 |
| V$GATA3\_03 |  | elt-1 (0.54) | 43 | 1.39 | 4.7e-02 |
| MA0173.1 |  | irx-1 | 10 | 3.29 | 4.9e-02 |
| MA0262.1 |  | mab-3 | 47 | 1.32 | 4.9e-02 |
| Irx5\_2385 |  | irx-1 | 49 | 1.28 | 4.9e-02 |
| Gata5\_3768 |  | elt-1 (0.54) | 12 | 2.85 | 4.9e-02 |
| HLH4C\_SANGER\_5\_FBgn0011277 |  | hlh-15 | 44 | 1.36 | 5.0e-02 |

### Correlated (and anti-correlated) transcription factors

|  |  |
| --- | --- |
| **Transcription factor** | **Correlation** |
| ztf-30 | 0.90 |
| lin-26 | 0.82 |
| nhr-25 | 0.82 |
| lin-1 | 0.79 |
| vab-7 | 0.79 |
| Y41D4B.26 | 0.77 |
| lir-1 | 0.75 |
| elt-3 | 0.74 |
| nhr-152 | 0.70 |
| B0310.2 | 0.69 |
| nhr-23 | 0.69 |
| egl-5 | 0.68 |
| elk-2 | 0.67 |
| unc-130 | 0.66 |
| nhr-127 | 0.66 |
| ceh-99 | 0.64 |
| nhr-94 | 0.64 |
| nhr-270 | 0.63 |
| tlp-1 | 0.63 |
| nhr-73 | 0.62 |
| nhr-109 | 0.61 |
| pat-9 | 0.61 |
| nhr-69 | 0.61 |
| F21A9.2 | 0.61 |
| cog-1 | 0.60 |
| ceh-28 | -0.43 |
| nhr-164 | -0.43 |
| nhr-167 | -0.43 |
| nhr-286 | -0.44 |
| nhr-21 | -0.46 |
| C08G9.2 | -0.47 |
| fkh-3 | -0.47 |
| nhr-219 | -0.47 |
| nhr-162 | -0.48 |
| nhr-213 | -0.49 |
| nhr-102 | -0.50 |
| nhr-141 | -0.50 |
| srt-58 | -0.51 |
| nhr-50 | -0.52 |
| nhr-275 | -0.52 |
| nhr-222 | -0.52 |
| nhr-269 | -0.52 |
| F21G4.5 | -0.53 |
| ceh-53 | -0.54 |
| T27A8.2 | -0.55 |
| nhr-207 | -0.55 |
| nhr-215 | -0.58 |
| fkh-2 | -0.59 |
| snu-23 | -0.63 |
| unc-55 | -0.65 |

### ChIP peaks enriched

|  |  |  |  |  |
| --- | --- | --- | --- | --- |
| **Gene** | **Experiment** | **Number of upstream peaks** | **Enrichment** | **FDR corrected p** |
| elt-1 | ELT-1\_Larvae-L2-stage | 10 | 12.01 | 7.5e-07 |
| nhr-23 | NHR-23\_Larvae-L3-stage | 30 | 2.62 | 3.3e-06 |
| ceh-16 | CEH-16\_Larvae-L2-stage | 16 | 3.87 | 1.0e-04 |
| nhr-28 | NHR-28\_Larvae-L4-stage | 33 | 2.01 | 2.0e-04 |
| nhr-25 | NHR-25\_Larvae-L2-stage | 25 | 2.39 | 4.0e-04 |
| elt-1 | ELT-1\_Larvae-L3-stage | 16 | 3.32 | 6.5e-04 |
| sax-3 | SAX-3\_Larvae-L4-stage | 29 | 1.97 | 1.9e-03 |
| fos-1 | FOS-1\_Larvae-L2-stage | 30 | 1.86 | 3.6e-03 |
| ceh-38 | CEH-38\_Larvae-L3-stage | 22 | 2.00 | 1.9e-02 |
| nhr-21 | NHR-21\_Larvae-L2-stage | 6 | 5.57 | 2.5e-02 |
| unc-62 | UNC-62\_Larvae-L3-stage | 17 | 2.28 | 2.5e-02 |
| ham-1 | HAM-1\_Fed-L1-stage-larvae | 24 | 1.87 | 2.5e-02 |
| sem-4 | SEM-4\_Larvae-L2-stage | 24 | 1.87 | 2.5e-02 |
