# Supplementary material for: Overlapping cell population expression profiling and regulatory inference in C. elegans
Source: BMC Genomics. 2016 Feb 29;17:159. doi: 10.1186/s12864-016-2482-z (PMC4772325; doi:10.1186/s12864-016-2482-z)
Supplement: Additional file 13: — Web supplement. (DOC 21 kb) [file 12864_2016_2482_MOESM13_ESM.zip › sortWeb/clusters/hier.300.clusters/235.html]

Cluster 235 

## Cluster 235

### Expression

| cnd-1 rep. 1 | cnd-1 rep. 2 | cnd-1 rep. 3 | pha-4 rep. 1 | pha-4 rep. 2 | pha-4 rep. 3 | ceh-27 | ceh-36 | ceh-6 | F21D5.9 | mir-57 | mls-2 | pal-1 | pros-1 | ttx-3 | unc-130 | hlh-16 | irx-1 | ceh-6 (+) hlh-16 (+) | ceh-6 (+) hlh-16 (-) | ceh-6 (-) hlh-16 (+) | cnd-1 singlets | pha-4 singlets | 0 | 60 | 120 | 150 | 180 | 240 | 330 | 390 | 420 | 480 | 540 | 570 | 600 | 630 | 660 | NAME | Functional description |
| --- | --- | --- | --- | --- | --- | --- | --- | --- | --- | --- | --- | --- | --- | --- | --- | --- | --- | --- | --- | --- | --- | --- | --- | --- | --- | --- | --- | --- | --- | --- | --- | --- | --- | --- | --- | --- | --- | --- | --- |
|  |  |  |  |  |  |  |  |  |  |  |  |  |  |  |  |  |  |  |  |  |  |  |  |  |  |  |  |  |  |  |  |  |  |  |  |  |  | *smf-1* | yeast SMF (divalent cation transporter) homolog |
|  |  |  |  |  |  |  |  |  |  |  |  |  |  |  |  |  |  |  |  |  |  |  |  |  |  |  |  |  |  |  |  |  |  |  |  |  |  | ZK792.1 |  |
|  |  |  |  |  |  |  |  |  |  |  |  |  |  |  |  |  |  |  |  |  |  |  |  |  |  |  |  |  |  |  |  |  |  |  |  |  |  | K11G12.6 |  |
|  |  |  |  |  |  |  |  |  |  |  |  |  |  |  |  |  |  |  |  |  |  |  |  |  |  |  |  |  |  |  |  |  |  |  |  |  |  | *nft-1* | NitFhit family |
|  |  |  |  |  |  |  |  |  |  |  |  |  |  |  |  |  |  |  |  |  |  |  |  |  |  |  |  |  |  |  |  |  |  |  |  |  |  | *gas-1* | General Anaesthetic Sensitivity abnormal |
|  |  |  |  |  |  |  |  |  |  |  |  |  |  |  |  |  |  |  |  |  |  |  |  |  |  |  |  |  |  |  |  |  |  |  |  |  |  | *spr-3* | Suppressor of PResenilin defect |
|  |  |  |  |  |  |  |  |  |  |  |  |  |  |  |  |  |  |  |  |  |  |  |  |  |  |  |  |  |  |  |  |  |  |  |  |  |  | Y105E8A.24 |  |
|  |  |  |  |  |  |  |  |  |  |  |  |  |  |  |  |  |  |  |  |  |  |  |  |  |  |  |  |  |  |  |  |  |  |  |  |  |  | Y105E8A.29 |  |
|  |  |  |  |  |  |  |  |  |  |  |  |  |  |  |  |  |  |  |  |  |  |  |  |  |  |  |  |  |  |  |  |  |  |  |  |  |  | Y105E8A.25 |  |
|  |  |  |  |  |  |  |  |  |  |  |  |  |  |  |  |  |  |  |  |  |  |  |  |  |  |  |  |  |  |  |  |  |  |  |  |  |  | C34C6.4 |  |
|  |  |  |  |  |  |  |  |  |  |  |  |  |  |  |  |  |  |  |  |  |  |  |  |  |  |  |  |  |  |  |  |  |  |  |  |  |  | *nfx-1* | NF-X1 transcription factor homolog |
|  |  |  |  |  |  |  |  |  |  |  |  |  |  |  |  |  |  |  |  |  |  |  |  |  |  |  |  |  |  |  |  |  |  |  |  |  |  | *let-721* | LEThal |
|  |  |  |  |  |  |  |  |  |  |  |  |  |  |  |  |  |  |  |  |  |  |  |  |  |  |  |  |  |  |  |  |  |  |  |  |  |  | F53F1.3 |  |
|  |  |  |  |  |  |  |  |  |  |  |  |  |  |  |  |  |  |  |  |  |  |  |  |  |  |  |  |  |  |  |  |  |  |  |  |  |  | *cgt-3* | Ceramide Glucosyl Transferase |
|  |  |  |  |  |  |  |  |  |  |  |  |  |  |  |  |  |  |  |  |  |  |  |  |  |  |  |  |  |  |  |  |  |  |  |  |  |  | *scav-3* | SCAVenger receptor (CD36 family) related |
|  |  |  |  |  |  |  |  |  |  |  |  |  |  |  |  |  |  |  |  |  |  |  |  |  |  |  |  |  |  |  |  |  |  |  |  |  |  | *pars-1* | Prolyl Amino-acyl tRNA Synthetase |
|  |  |  |  |  |  |  |  |  |  |  |  |  |  |  |  |  |  |  |  |  |  |  |  |  |  |  |  |  |  |  |  |  |  |  |  |  |  | W03F8.10 |  |
|  |  |  |  |  |  |  |  |  |  |  |  |  |  |  |  |  |  |  |  |  |  |  |  |  |  |  |  |  |  |  |  |  |  |  |  |  |  | *lam-2* | LAMinin related. See also lmb- |
|  |  |  |  |  |  |  |  |  |  |  |  |  |  |  |  |  |  |  |  |  |  |  |  |  |  |  |  |  |  |  |  |  |  |  |  |  |  | *mrps-30* | Mitochondrial Ribosomal Protein, Small |
|  |  |  |  |  |  |  |  |  |  |  |  |  |  |  |  |  |  |  |  |  |  |  |  |  |  |  |  |  |  |  |  |  |  |  |  |  |  | F25B5.5 |  |
|  |  |  |  |  |  |  |  |  |  |  |  |  |  |  |  |  |  |  |  |  |  |  |  |  |  |  |  |  |  |  |  |  |  |  |  |  |  | *dyci-1* | DYnein Chain, light Intermediate |
|  |  |  |  |  |  |  |  |  |  |  |  |  |  |  |  |  |  |  |  |  |  |  |  |  |  |  |  |  |  |  |  |  |  |  |  |  |  | *rpom-1* | RNA POlymerase, Mitochondrial |
|  |  |  |  |  |  |  |  |  |  |  |  |  |  |  |  |  |  |  |  |  |  |  |  |  |  |  |  |  |  |  |  |  |  |  |  |  |  | *iars-2* | Isoleucyl Amino-acyl tRNA Synthetase |
|  |  |  |  |  |  |  |  |  |  |  |  |  |  |  |  |  |  |  |  |  |  |  |  |  |  |  |  |  |  |  |  |  |  |  |  |  |  | *abcf-3* | ABC transporter, class F |
|  |  |  |  |  |  |  |  |  |  |  |  |  |  |  |  |  |  |  |  |  |  |  |  |  |  |  |  |  |  |  |  |  |  |  |  |  |  | *ril-2* | RNAi-Induced Longevity |
|  |  |  |  |  |  |  |  |  |  |  |  |  |  |  |  |  |  |  |  |  |  |  |  |  |  |  |  |  |  |  |  |  |  |  |  |  |  | F46B6.6 |  |
|  |  |  |  |  |  |  |  |  |  |  |  |  |  |  |  |  |  |  |  |  |  |  |  |  |  |  |  |  |  |  |  |  |  |  |  |  |  | C14C10.4 |  |
|  |  |  |  |  |  |  |  |  |  |  |  |  |  |  |  |  |  |  |  |  |  |  |  |  |  |  |  |  |  |  |  |  |  |  |  |  |  | *ztf-7* | Zinc finger putative Transcription Factor family |
|  |  |  |  |  |  |  |  |  |  |  |  |  |  |  |  |  |  |  |  |  |  |  |  |  |  |  |  |  |  |  |  |  |  |  |  |  |  | *gfm-1* | GFM (mitochondrial elongation factor G) homolog |
|  |  |  |  |  |  |  |  |  |  |  |  |  |  |  |  |  |  |  |  |  |  |  |  |  |  |  |  |  |  |  |  |  |  |  |  |  |  | ZK550.3 |  |
|  |  |  |  |  |  |  |  |  |  |  |  |  |  |  |  |  |  |  |  |  |  |  |  |  |  |  |  |  |  |  |  |  |  |  |  |  |  | *vps-33.1* | related to yeast Vacuolar Protein Sorting factor |
|  |  |  |  |  |  |  |  |  |  |  |  |  |  |  |  |  |  |  |  |  |  |  |  |  |  |  |  |  |  |  |  |  |  |  |  |  |  | Y110A7A.19 |  |
|  |  |  |  |  |  |  |  |  |  |  |  |  |  |  |  |  |  |  |  |  |  |  |  |  |  |  |  |  |  |  |  |  |  |  |  |  |  | *haf-3* | HAlF transporter (PGP related) |
|  |  |  |  |  |  |  |  |  |  |  |  |  |  |  |  |  |  |  |  |  |  |  |  |  |  |  |  |  |  |  |  |  |  |  |  |  |  | *acdh-13* | Acyl CoA DeHydrogenase |
|  |  |  |  |  |  |  |  |  |  |  |  |  |  |  |  |  |  |  |  |  |  |  |  |  |  |  |  |  |  |  |  |  |  |  |  |  |  | *scpl-4* | SCP (Small C-terminal domain Phosphatase)-Like phosphatase |
|  |  |  |  |  |  |  |  |  |  |  |  |  |  |  |  |  |  |  |  |  |  |  |  |  |  |  |  |  |  |  |  |  |  |  |  |  |  | D2030.2 |  |
|  |  |  |  |  |  |  |  |  |  |  |  |  |  |  |  |  |  |  |  |  |  |  |  |  |  |  |  |  |  |  |  |  |  |  |  |  |  | *dnj-9* | DNaJ domain (prokaryotic heat shock protein) |
|  |  |  |  |  |  |  |  |  |  |  |  |  |  |  |  |  |  |  |  |  |  |  |  |  |  |  |  |  |  |  |  |  |  |  |  |  |  | R74.6 |  |
|  |  |  |  |  |  |  |  |  |  |  |  |  |  |  |  |  |  |  |  |  |  |  |  |  |  |  |  |  |  |  |  |  |  |  |  |  |  | *acdh-9* | Acyl CoA DeHydrogenase |
|  |  |  |  |  |  |  |  |  |  |  |  |  |  |  |  |  |  |  |  |  |  |  |  |  |  |  |  |  |  |  |  |  |  |  |  |  |  | *acdh-12* | Acyl CoA DeHydrogenase |
|  |  |  |  |  |  |  |  |  |  |  |  |  |  |  |  |  |  |  |  |  |  |  |  |  |  |  |  |  |  |  |  |  |  |  |  |  |  | B0303.3 |  |
|  |  |  |  |  |  |  |  |  |  |  |  |  |  |  |  |  |  |  |  |  |  |  |  |  |  |  |  |  |  |  |  |  |  |  |  |  |  | *nhr-256* | Nuclear Hormone Receptor family |
|  |  |  |  |  |  |  |  |  |  |  |  |  |  |  |  |  |  |  |  |  |  |  |  |  |  |  |  |  |  |  |  |  |  |  |  |  |  | *acl-5* | ACyLtransferase-like |
|  |  |  |  |  |  |  |  |  |  |  |  |  |  |  |  |  |  |  |  |  |  |  |  |  |  |  |  |  |  |  |  |  |  |  |  |  |  | *tufm-1* | TU elongation Factor (EF-Tu), Mitochondrial |
|  |  |  |  |  |  |  |  |  |  |  |  |  |  |  |  |  |  |  |  |  |  |  |  |  |  |  |  |  |  |  |  |  |  |  |  |  |  | *wah-1* | Worm AIF (apoptosis inducing factor) Homolog |
|  |  |  |  |  |  |  |  |  |  |  |  |  |  |  |  |  |  |  |  |  |  |  |  |  |  |  |  |  |  |  |  |  |  |  |  |  |  | H28O16.1 |  |
|  |  |  |  |  |  |  |  |  |  |  |  |  |  |  |  |  |  |  |  |  |  |  |  |  |  |  |  |  |  |  |  |  |  |  |  |  |  | *dpyd-1* | DihydroPYrimidine Dehydrogenase |
|  |  |  |  |  |  |  |  |  |  |  |  |  |  |  |  |  |  |  |  |  |  |  |  |  |  |  |  |  |  |  |  |  |  |  |  |  |  | *wts-1* | WarTS (Drosophila) protein kinase homolog |
|  |  |  |  |  |  |  |  |  |  |  |  |  |  |  |  |  |  |  |  |  |  |  |  |  |  |  |  |  |  |  |  |  |  |  |  |  |  | *sbp-1* | Sterol regulatory element Binding Protein |
|  |  |  |  |  |  |  |  |  |  |  |  |  |  |  |  |  |  |  |  |  |  |  |  |  |  |  |  |  |  |  |  |  |  |  |  |  |  | *sma-1* | SMAll |
|  |  |  |  |  |  |  |  |  |  |  |  |  |  |  |  |  |  |  |  |  |  |  |  |  |  |  |  |  |  |  |  |  |  |  |  |  |  | *frm-7* | FERM domain (protein4.1-ezrin-radixin-moesin) family |
|  |  |  |  |  |  |  |  |  |  |  |  |  |  |  |  |  |  |  |  |  |  |  |  |  |  |  |  |  |  |  |  |  |  |  |  |  |  | *mig-22* | abnormal cell MIGration |
|  |  |  |  |  |  |  |  |  |  |  |  |  |  |  |  |  |  |  |  |  |  |  |  |  |  |  |  |  |  |  |  |  |  |  |  |  |  | C46H3.2 |  |
|  |  |  |  |  |  |  |  |  |  |  |  |  |  |  |  |  |  |  |  |  |  |  |  |  |  |  |  |  |  |  |  |  |  |  |  |  |  | *vab-1* | Variable ABnormal morphology |
|  |  |  |  |  |  |  |  |  |  |  |  |  |  |  |  |  |  |  |  |  |  |  |  |  |  |  |  |  |  |  |  |  |  |  |  |  |  | *tra-3* | TRAnsformer: XX animals transformed into males |
|  |  |  |  |  |  |  |  |  |  |  |  |  |  |  |  |  |  |  |  |  |  |  |  |  |  |  |  |  |  |  |  |  |  |  |  |  |  | *atp-2* | ATP synthase subunit |
|  |  |  |  |  |  |  |  |  |  |  |  |  |  |  |  |  |  |  |  |  |  |  |  |  |  |  |  |  |  |  |  |  |  |  |  |  |  | T08B2.7 |  |
|  |  |  |  |  |  |  |  |  |  |  |  |  |  |  |  |  |  |  |  |  |  |  |  |  |  |  |  |  |  |  |  |  |  |  |  |  |  | ZK836.2 |  |
|  |  |  |  |  |  |  |  |  |  |  |  |  |  |  |  |  |  |  |  |  |  |  |  |  |  |  |  |  |  |  |  |  |  |  |  |  |  | *fat-3* | FATty acid desaturase |
|  |  |  |  |  |  |  |  |  |  |  |  |  |  |  |  |  |  |  |  |  |  |  |  |  |  |  |  |  |  |  |  |  |  |  |  |  |  | C46C11.4 |  |
|  |  |  |  |  |  |  |  |  |  |  |  |  |  |  |  |  |  |  |  |  |  |  |  |  |  |  |  |  |  |  |  |  |  |  |  |  |  | Y54G9A.7 |  |
|  |  |  |  |  |  |  |  |  |  |  |  |  |  |  |  |  |  |  |  |  |  |  |  |  |  |  |  |  |  |  |  |  |  |  |  |  |  | ZK180.8 |  |
|  |  |  |  |  |  |  |  |  |  |  |  |  |  |  |  |  |  |  |  |  |  |  |  |  |  |  |  |  |  |  |  |  |  |  |  |  |  | *seld-1* | SELD (SelD homolog) involved in selenophosphate synthesis |
|  |  |  |  |  |  |  |  |  |  |  |  |  |  |  |  |  |  |  |  |  |  |  |  |  |  |  |  |  |  |  |  |  |  |  |  |  |  | *fncm-1* | FANCM (Fanconi anemia complex component M) homolog |
|  |  |  |  |  |  |  |  |  |  |  |  |  |  |  |  |  |  |  |  |  |  |  |  |  |  |  |  |  |  |  |  |  |  |  |  |  |  | F22F7.1 |  |
|  |  |  |  |  |  |  |  |  |  |  |  |  |  |  |  |  |  |  |  |  |  |  |  |  |  |  |  |  |  |  |  |  |  |  |  |  |  | *copb-1* | COP (COat Protomer) Beta subunit |
|  |  |  |  |  |  |  |  |  |  |  |  |  |  |  |  |  |  |  |  |  |  |  |  |  |  |  |  |  |  |  |  |  |  |  |  |  |  | *vig-1* | VIG (Drosophila Vasa Intronic Gene) ortholog |
|  |  |  |  |  |  |  |  |  |  |  |  |  |  |  |  |  |  |  |  |  |  |  |  |  |  |  |  |  |  |  |  |  |  |  |  |  |  | *cyk-1* | CYtoKinesis defect |
|  |  |  |  |  |  |  |  |  |  |  |  |  |  |  |  |  |  |  |  |  |  |  |  |  |  |  |  |  |  |  |  |  |  |  |  |  |  | *inft-2* | INverted Formin/formin Three-related |
|  |  |  |  |  |  |  |  |  |  |  |  |  |  |  |  |  |  |  |  |  |  |  |  |  |  |  |  |  |  |  |  |  |  |  |  |  |  | F16B4.7 |  |
|  |  |  |  |  |  |  |  |  |  |  |  |  |  |  |  |  |  |  |  |  |  |  |  |  |  |  |  |  |  |  |  |  |  |  |  |  |  | *kup-1* | Kinase UPstream gene (in operon) |
|  |  |  |  |  |  |  |  |  |  |  |  |  |  |  |  |  |  |  |  |  |  |  |  |  |  |  |  |  |  |  |  |  |  |  |  |  |  | *vang-1* | VANG (Van Gogh/Strabismus planar polarity protein) homolog |
|  |  |  |  |  |  |  |  |  |  |  |  |  |  |  |  |  |  |  |  |  |  |  |  |  |  |  |  |  |  |  |  |  |  |  |  |  |  | *frm-2* | FERM domain (protein4.1-ezrin-radixin-moesin) family |
|  |  |  |  |  |  |  |  |  |  |  |  |  |  |  |  |  |  |  |  |  |  |  |  |  |  |  |  |  |  |  |  |  |  |  |  |  |  | *vav-1* | mammalian VAV (oncogene) related |
|  |  |  |  |  |  |  |  |  |  |  |  |  |  |  |  |  |  |  |  |  |  |  |  |  |  |  |  |  |  |  |  |  |  |  |  |  |  | *egl-44* | EGg Laying defective |
|  |  |  |  |  |  |  |  |  |  |  |  |  |  |  |  |  |  |  |  |  |  |  |  |  |  |  |  |  |  |  |  |  |  |  |  |  |  | *igcm-3* | ImmunoGlobulin-like Cell adhesion Molecule family |
|  |  |  |  |  |  |  |  |  |  |  |  |  |  |  |  |  |  |  |  |  |  |  |  |  |  |  |  |  |  |  |  |  |  |  |  |  |  | *piki-1* | phosphoInositide-3-KInase |
|  |  |  |  |  |  |  |  |  |  |  |  |  |  |  |  |  |  |  |  |  |  |  |  |  |  |  |  |  |  |  |  |  |  |  |  |  |  | *kin-32* | protein KINase |
|  |  |  |  |  |  |  |  |  |  |  |  |  |  |  |  |  |  |  |  |  |  |  |  |  |  |  |  |  |  |  |  |  |  |  |  |  |  | *seu-1* | Suppressor of Ectopic Unc-5 |
|  |  |  |  |  |  |  |  |  |  |  |  |  |  |  |  |  |  |  |  |  |  |  |  |  |  |  |  |  |  |  |  |  |  |  |  |  |  | *ham-1* | HSN Abnormal Migration |
|  |  |  |  |  |  |  |  |  |  |  |  |  |  |  |  |  |  |  |  |  |  |  |  |  |  |  |  |  |  |  |  |  |  |  |  |  |  | *chaf-2* | CHromatin Assembly Factor |
|  |  |  |  |  |  |  |  |  |  |  |  |  |  |  |  |  |  |  |  |  |  |  |  |  |  |  |  |  |  |  |  |  |  |  |  |  |  | *cdc-25.2* | Cell Division Cycle related |
|  |  |  |  |  |  |  |  |  |  |  |  |  |  |  |  |  |  |  |  |  |  |  |  |  |  |  |  |  |  |  |  |  |  |  |  |  |  | Y48G8AL.13 |  |
|  |  |  |  |  |  |  |  |  |  |  |  |  |  |  |  |  |  |  |  |  |  |  |  |  |  |  |  |  |  |  |  |  |  |  |  |  |  | *duxl-1* | DUX (vertebrate dual homeobox) Like |
|  |  |  |  |  |  |  |  |  |  |  |  |  |  |  |  |  |  |  |  |  |  |  |  |  |  |  |  |  |  |  |  |  |  |  |  |  |  | ZK355.2 |  |
|  |  |  |  |  |  |  |  |  |  |  |  |  |  |  |  |  |  |  |  |  |  |  |  |  |  |  |  |  |  |  |  |  |  |  |  |  |  | *hke-4.2* | Histidine-rich membrane protein KE4 (mouse) homolog |
|  |  |  |  |  |  |  |  |  |  |  |  |  |  |  |  |  |  |  |  |  |  |  |  |  |  |  |  |  |  |  |  |  |  |  |  |  |  | T10C6.6 |  |
|  |  |  |  |  |  |  |  |  |  |  |  |  |  |  |  |  |  |  |  |  |  |  |  |  |  |  |  |  |  |  |  |  |  |  |  |  |  | C44E4.4 |  |
|  |  |  |  |  |  |  |  |  |  |  |  |  |  |  |  |  |  |  |  |  |  |  |  |  |  |  |  |  |  |  |  |  |  |  |  |  |  | C36B7.6 |  |
|  |  |  |  |  |  |  |  |  |  |  |  |  |  |  |  |  |  |  |  |  |  |  |  |  |  |  |  |  |  |  |  |  |  |  |  |  |  | *par-6* | abnormal embryonic PARtitioning of cytoplasm |
|  |  |  |  |  |  |  |  |  |  |  |  |  |  |  |  |  |  |  |  |  |  |  |  |  |  |  |  |  |  |  |  |  |  |  |  |  |  | *rsp-1* | SR Protein (splicing factor) |
|  |  |  |  |  |  |  |  |  |  |  |  |  |  |  |  |  |  |  |  |  |  |  |  |  |  |  |  |  |  |  |  |  |  |  |  |  |  | Y53F4B.38 |  |
|  |  |  |  |  |  |  |  |  |  |  |  |  |  |  |  |  |  |  |  |  |  |  |  |  |  |  |  |  |  |  |  |  |  |  |  |  |  | *pdi-3* | Protein Disulfide Isomerase |
|  |  |  |  |  |  |  |  |  |  |  |  |  |  |  |  |  |  |  |  |  |  |  |  |  |  |  |  |  |  |  |  |  |  |  |  |  |  | *crt-1* | CalReTiculin |
|  |  |  |  |  |  |  |  |  |  |  |  |  |  |  |  |  |  |  |  |  |  |  |  |  |  |  |  |  |  |  |  |  |  |  |  |  |  | *imb-3* | IMportin Beta family |
|  |  |  |  |  |  |  |  |  |  |  |  |  |  |  |  |  |  |  |  |  |  |  |  |  |  |  |  |  |  |  |  |  |  |  |  |  |  | *copa-1* | COat Protein complex 1, Alpha subunit |
|  |  |  |  |  |  |  |  |  |  |  |  |  |  |  |  |  |  |  |  |  |  |  |  |  |  |  |  |  |  |  |  |  |  |  |  |  |  | T13C2.6 |  |
|  |  |  |  |  |  |  |  |  |  |  |  |  |  |  |  |  |  |  |  |  |  |  |  |  |  |  |  |  |  |  |  |  |  |  |  |  |  | *hsp-4* | Heat Shock Protein |
|  |  |  |  |  |  |  |  |  |  |  |  |  |  |  |  |  |  |  |  |  |  |  |  |  |  |  |  |  |  |  |  |  |  |  |  |  |  | C08H9.3 |  |
|  |  |  |  |  |  |  |  |  |  |  |  |  |  |  |  |  |  |  |  |  |  |  |  |  |  |  |  |  |  |  |  |  |  |  |  |  |  | *ribo-1* | RIBOphorin (oligosaccharyltransferase complex) homolog |
|  |  |  |  |  |  |  |  |  |  |  |  |  |  |  |  |  |  |  |  |  |  |  |  |  |  |  |  |  |  |  |  |  |  |  |  |  |  | *vars-2* | Valyl Amino-acyl tRNA Synthetase |
|  |  |  |  |  |  |  |  |  |  |  |  |  |  |  |  |  |  |  |  |  |  |  |  |  |  |  |  |  |  |  |  |  |  |  |  |  |  | Y23H5B.6 |  |
|  |  |  |  |  |  |  |  |  |  |  |  |  |  |  |  |  |  |  |  |  |  |  |  |  |  |  |  |  |  |  |  |  |  |  |  |  |  | *qars-1* | glutaminyl(Q) Amino-acyl tRNA Synthetase |
|  |  |  |  |  |  |  |  |  |  |  |  |  |  |  |  |  |  |  |  |  |  |  |  |  |  |  |  |  |  |  |  |  |  |  |  |  |  | *imp-2* | IntraMembrane Protease (IMPAS) family |
|  |  |  |  |  |  |  |  |  |  |  |  |  |  |  |  |  |  |  |  |  |  |  |  |  |  |  |  |  |  |  |  |  |  |  |  |  |  | *mspn-1* | Mitochondrial Sorting of Proteins (yeast MSP) in Nematode |
|  |  |  |  |  |  |  |  |  |  |  |  |  |  |  |  |  |  |  |  |  |  |  |  |  |  |  |  |  |  |  |  |  |  |  |  |  |  | *eef-1A.2* | Eukaryotic translation Elongation Factor |
|  |  |  |  |  |  |  |  |  |  |  |  |  |  |  |  |  |  |  |  |  |  |  |  |  |  |  |  |  |  |  |  |  |  |  |  |  |  | *tag-151* | Temporarily Assigned Gene name |
|  |  |  |  |  |  |  |  |  |  |  |  |  |  |  |  |  |  |  |  |  |  |  |  |  |  |  |  |  |  |  |  |  |  |  |  |  |  | Y53C12B.1 |  |
|  |  |  |  |  |  |  |  |  |  |  |  |  |  |  |  |  |  |  |  |  |  |  |  |  |  |  |  |  |  |  |  |  |  |  |  |  |  | C53H9.2 |  |
|  |  |  |  |  |  |  |  |  |  |  |  |  |  |  |  |  |  |  |  |  |  |  |  |  |  |  |  |  |  |  |  |  |  |  |  |  |  | *eif-3.D* | Eukaryotic Initiation Factor |
|  |  |  |  |  |  |  |  |  |  |  |  |  |  |  |  |  |  |  |  |  |  |  |  |  |  |  |  |  |  |  |  |  |  |  |  |  |  | *eef-1A.1* | Eukaryotic translation Elongation Factor |
|  |  |  |  |  |  |  |  |  |  |  |  |  |  |  |  |  |  |  |  |  |  |  |  |  |  |  |  |  |  |  |  |  |  |  |  |  |  | *eef-2* | Eukaryotic translation Elongation Factor |
|  |  |  |  |  |  |  |  |  |  |  |  |  |  |  |  |  |  |  |  |  |  |  |  |  |  |  |  |  |  |  |  |  |  |  |  |  |  | C08H9.2 |  |
|  |  |  |  |  |  |  |  |  |  |  |  |  |  |  |  |  |  |  |  |  |  |  |  |  |  |  |  |  |  |  |  |  |  |  |  |  |  | *ahcy-1* | S-AdenosylhomoCysteine HYdrolase homolog |
|  |  |  |  |  |  |  |  |  |  |  |  |  |  |  |  |  |  |  |  |  |  |  |  |  |  |  |  |  |  |  |  |  |  |  |  |  |  | *idh-1* | Isocitrate DeHydrogenase |
|  |  |  |  |  |  |  |  |  |  |  |  |  |  |  |  |  |  |  |  |  |  |  |  |  |  |  |  |  |  |  |  |  |  |  |  |  |  | *pdi-2* | Protein Disulfide Isomerase |
|  |  |  |  |  |  |  |  |  |  |  |  |  |  |  |  |  |  |  |  |  |  |  |  |  |  |  |  |  |  |  |  |  |  |  |  |  |  | *sav-1* | SAlVador (cell proliferation regulator) homolog |
|  |  |  |  |  |  |  |  |  |  |  |  |  |  |  |  |  |  |  |  |  |  |  |  |  |  |  |  |  |  |  |  |  |  |  |  |  |  | *lst-1* | Lateral Signaling Target |
|  |  |  |  |  |  |  |  |  |  |  |  |  |  |  |  |  |  |  |  |  |  |  |  |  |  |  |  |  |  |  |  |  |  |  |  |  |  | *ugt-45* | UDP-GlucuronosylTransferase |
|  |  |  |  |  |  |  |  |  |  |  |  |  |  |  |  |  |  |  |  |  |  |  |  |  |  |  |  |  |  |  |  |  |  |  |  |  |  | *acdh-10* | Acyl CoA DeHydrogenase |
|  |  |  |  |  |  |  |  |  |  |  |  |  |  |  |  |  |  |  |  |  |  |  |  |  |  |  |  |  |  |  |  |  |  |  |  |  |  | *acdh-7* | Acyl CoA DeHydrogenase |
|  |  |  |  |  |  |  |  |  |  |  |  |  |  |  |  |  |  |  |  |  |  |  |  |  |  |  |  |  |  |  |  |  |  |  |  |  |  | K07C5.4 |  |
|  |  |  |  |  |  |  |  |  |  |  |  |  |  |  |  |  |  |  |  |  |  |  |  |  |  |  |  |  |  |  |  |  |  |  |  |  |  | *eef-1G* | Eukaryotic translation Elongation Factor |
|  |  |  |  |  |  |  |  |  |  |  |  |  |  |  |  |  |  |  |  |  |  |  |  |  |  |  |  |  |  |  |  |  |  |  |  |  |  | *kars-1* | lysyl(K) Amino-acyl tRNA Synthetase |
|  |  |  |  |  |  |  |  |  |  |  |  |  |  |  |  |  |  |  |  |  |  |  |  |  |  |  |  |  |  |  |  |  |  |  |  |  |  | K01G5.5 |  |
|  |  |  |  |  |  |  |  |  |  |  |  |  |  |  |  |  |  |  |  |  |  |  |  |  |  |  |  |  |  |  |  |  |  |  |  |  |  | *nol-5* | NucleOLar protein |
|  |  |  |  |  |  |  |  |  |  |  |  |  |  |  |  |  |  |  |  |  |  |  |  |  |  |  |  |  |  |  |  |  |  |  |  |  |  | *fib-1* | FIBrillarin family |
|  |  |  |  |  |  |  |  |  |  |  |  |  |  |  |  |  |  |  |  |  |  |  |  |  |  |  |  |  |  |  |  |  |  |  |  |  |  | F10G7.5 |  |
|  |  |  |  |  |  |  |  |  |  |  |  |  |  |  |  |  |  |  |  |  |  |  |  |  |  |  |  |  |  |  |  |  |  |  |  |  |  | *mes-1* | Maternal Effect Sterile |
|  |  |  |  |  |  |  |  |  |  |  |  |  |  |  |  |  |  |  |  |  |  |  |  |  |  |  |  |  |  |  |  |  |  |  |  |  |  | ZK909.6 |  |
|  |  |  |  |  |  |  |  |  |  |  |  |  |  |  |  |  |  |  |  |  |  |  |  |  |  |  |  |  |  |  |  |  |  |  |  |  |  | *ces-2* | CEll death Specification |
|  |  |  |  |  |  |  |  |  |  |  |  |  |  |  |  |  |  |  |  |  |  |  |  |  |  |  |  |  |  |  |  |  |  |  |  |  |  | K08A2.4 |  |
|  |  |  |  |  |  |  |  |  |  |  |  |  |  |  |  |  |  |  |  |  |  |  |  |  |  |  |  |  |  |  |  |  |  |  |  |  |  | *hil-5* | HIstone H1 Like |
|  |  |  |  |  |  |  |  |  |  |  |  |  |  |  |  |  |  |  |  |  |  |  |  |  |  |  |  |  |  |  |  |  |  |  |  |  |  | *bre-5* | BT (Bacillus thuringiensis) toxin REsistant |
|  |  |  |  |  |  |  |  |  |  |  |  |  |  |  |  |  |  |  |  |  |  |  |  |  |  |  |  |  |  |  |  |  |  |  |  |  |  | W02G9.3 |  |
|  |  |  |  |  |  |  |  |  |  |  |  |  |  |  |  |  |  |  |  |  |  |  |  |  |  |  |  |  |  |  |  |  |  |  |  |  |  | ZK1067.2 |  |
|  |  |  |  |  |  |  |  |  |  |  |  |  |  |  |  |  |  |  |  |  |  |  |  |  |  |  |  |  |  |  |  |  |  |  |  |  |  | F25H8.2 |  |
|  |  |  |  |  |  |  |  |  |  |  |  |  |  |  |  |  |  |  |  |  |  |  |  |  |  |  |  |  |  |  |  |  |  |  |  |  |  | F25H8.1 |  |
|  |  |  |  |  |  |  |  |  |  |  |  |  |  |  |  |  |  |  |  |  |  |  |  |  |  |  |  |  |  |  |  |  |  |  |  |  |  | F11A10.7 |  |
|  |  |  |  |  |  |  |  |  |  |  |  |  |  |  |  |  |  |  |  |  |  |  |  |  |  |  |  |  |  |  |  |  |  |  |  |  |  | W01A11.1 |  |
|  |  |  |  |  |  |  |  |  |  |  |  |  |  |  |  |  |  |  |  |  |  |  |  |  |  |  |  |  |  |  |  |  |  |  |  |  |  | *gars-1* | Glycyl Amino-acyl tRNA Synthetase |
|  |  |  |  |  |  |  |  |  |  |  |  |  |  |  |  |  |  |  |  |  |  |  |  |  |  |  |  |  |  |  |  |  |  |  |  |  |  | *henn-1* | HEN1 (RNA 3'end methyltransferase) of Nematode |
|  |  |  |  |  |  |  |  |  |  |  |  |  |  |  |  |  |  |  |  |  |  |  |  |  |  |  |  |  |  |  |  |  |  |  |  |  |  | C38D4.7 |  |
|  |  |  |  |  |  |  |  |  |  |  |  |  |  |  |  |  |  |  |  |  |  |  |  |  |  |  |  |  |  |  |  |  |  |  |  |  |  | *hpo-3* | Hypersensitive to POre-forming toxin |
|  |  |  |  |  |  |  |  |  |  |  |  |  |  |  |  |  |  |  |  |  |  |  |  |  |  |  |  |  |  |  |  |  |  |  |  |  |  | *amph-1* | AMPHiphysin homolog |
|  |  |  |  |  |  |  |  |  |  |  |  |  |  |  |  |  |  |  |  |  |  |  |  |  |  |  |  |  |  |  |  |  |  |  |  |  |  | Y55F3BL.4 |  |
|  |  |  |  |  |  |  |  |  |  |  |  |  |  |  |  |  |  |  |  |  |  |  |  |  |  |  |  |  |  |  |  |  |  |  |  |  |  | JC8.11 |  |
|  |  |  |  |  |  |  |  |  |  |  |  |  |  |  |  |  |  |  |  |  |  |  |  |  |  |  |  |  |  |  |  |  |  |  |  |  |  | *gln-5* | GLutamiNe synthetase (glutamate-ammonia ligase) |
|  |  |  |  |  |  |  |  |  |  |  |  |  |  |  |  |  |  |  |  |  |  |  |  |  |  |  |  |  |  |  |  |  |  |  |  |  |  | C16C10.2 |  |
|  |  |  |  |  |  |  |  |  |  |  |  |  |  |  |  |  |  |  |  |  |  |  |  |  |  |  |  |  |  |  |  |  |  |  |  |  |  | E01A2.5 |  |
|  |  |  |  |  |  |  |  |  |  |  |  |  |  |  |  |  |  |  |  |  |  |  |  |  |  |  |  |  |  |  |  |  |  |  |  |  |  | Y40B1A.2 |  |
|  |  |  |  |  |  |  |  |  |  |  |  |  |  |  |  |  |  |  |  |  |  |  |  |  |  |  |  |  |  |  |  |  |  |  |  |  |  | K07E8.7 |  |
|  |  |  |  |  |  |  |  |  |  |  |  |  |  |  |  |  |  |  |  |  |  |  |  |  |  |  |  |  |  |  |  |  |  |  |  |  |  | Y43F4B.5 |  |
|  |  |  |  |  |  |  |  |  |  |  |  |  |  |  |  |  |  |  |  |  |  |  |  |  |  |  |  |  |  |  |  |  |  |  |  |  |  | *selb-1* | SELB (SelB homolog) translation factor for selenocysteine incorporation |
|  |  |  |  |  |  |  |  |  |  |  |  |  |  |  |  |  |  |  |  |  |  |  |  |  |  |  |  |  |  |  |  |  |  |  |  |  |  | T25D3.4 |  |
|  |  |  |  |  |  |  |  |  |  |  |  |  |  |  |  |  |  |  |  |  |  |  |  |  |  |  |  |  |  |  |  |  |  |  |  |  |  | *pak-1* | P21-Activated Kinase family |
|  |  |  |  |  |  |  |  |  |  |  |  |  |  |  |  |  |  |  |  |  |  |  |  |  |  |  |  |  |  |  |  |  |  |  |  |  |  | Y39E4A.3 |  |
|  |  |  |  |  |  |  |  |  |  |  |  |  |  |  |  |  |  |  |  |  |  |  |  |  |  |  |  |  |  |  |  |  |  |  |  |  |  | *mel-32* | Maternal Effect Lethal |
|  |  |  |  |  |  |  |  |  |  |  |  |  |  |  |  |  |  |  |  |  |  |  |  |  |  |  |  |  |  |  |  |  |  |  |  |  |  | F08G2.7 |  |
|  |  |  |  |  |  |  |  |  |  |  |  |  |  |  |  |  |  |  |  |  |  |  |  |  |  |  |  |  |  |  |  |  |  |  |  |  |  | *pkn-1* | Protein Kinase N (PKN) homolog |
|  |  |  |  |  |  |  |  |  |  |  |  |  |  |  |  |  |  |  |  |  |  |  |  |  |  |  |  |  |  |  |  |  |  |  |  |  |  | C37H5.6 |  |
|  |  |  |  |  |  |  |  |  |  |  |  |  |  |  |  |  |  |  |  |  |  |  |  |  |  |  |  |  |  |  |  |  |  |  |  |  |  | *idhb-1* | Isocitrate DeHydrogenase Beta |
|  |  |  |  |  |  |  |  |  |  |  |  |  |  |  |  |  |  |  |  |  |  |  |  |  |  |  |  |  |  |  |  |  |  |  |  |  |  | *erm-1* | Ezrin/Radixin/Moesin |
|  |  |  |  |  |  |  |  |  |  |  |  |  |  |  |  |  |  |  |  |  |  |  |  |  |  |  |  |  |  |  |  |  |  |  |  |  |  | F48E8.3 |  |
|  |  |  |  |  |  |  |  |  |  |  |  |  |  |  |  |  |  |  |  |  |  |  |  |  |  |  |  |  |  |  |  |  |  |  |  |  |  | F49D11.10 |  |
|  |  |  |  |  |  |  |  |  |  |  |  |  |  |  |  |  |  |  |  |  |  |  |  |  |  |  |  |  |  |  |  |  |  |  |  |  |  | *rpoa-1* | RNA POlymerase I (A) subunit |
|  |  |  |  |  |  |  |  |  |  |  |  |  |  |  |  |  |  |  |  |  |  |  |  |  |  |  |  |  |  |  |  |  |  |  |  |  |  | T10B10.3 |  |
|  |  |  |  |  |  |  |  |  |  |  |  |  |  |  |  |  |  |  |  |  |  |  |  |  |  |  |  |  |  |  |  |  |  |  |  |  |  | Y23H5B.5 |  |
|  |  |  |  |  |  |  |  |  |  |  |  |  |  |  |  |  |  |  |  |  |  |  |  |  |  |  |  |  |  |  |  |  |  |  |  |  |  | W07E6.2 |  |
|  |  |  |  |  |  |  |  |  |  |  |  |  |  |  |  |  |  |  |  |  |  |  |  |  |  |  |  |  |  |  |  |  |  |  |  |  |  | T10B5.3 |  |
|  |  |  |  |  |  |  |  |  |  |  |  |  |  |  |  |  |  |  |  |  |  |  |  |  |  |  |  |  |  |  |  |  |  |  |  |  |  | *xrn-2* | XRN (mouse/S. cerevisiae) ribonuclease related |
|  |  |  |  |  |  |  |  |  |  |  |  |  |  |  |  |  |  |  |  |  |  |  |  |  |  |  |  |  |  |  |  |  |  |  |  |  |  | Y66H1A.4 |  |
|  |  |  |  |  |  |  |  |  |  |  |  |  |  |  |  |  |  |  |  |  |  |  |  |  |  |  |  |  |  |  |  |  |  |  |  |  |  | *mac-1* | Member of AAA family binding CED-4 |
|  |  |  |  |  |  |  |  |  |  |  |  |  |  |  |  |  |  |  |  |  |  |  |  |  |  |  |  |  |  |  |  |  |  |  |  |  |  | Y46E12BL.2 |  |
|  |  |  |  |  |  |  |  |  |  |  |  |  |  |  |  |  |  |  |  |  |  |  |  |  |  |  |  |  |  |  |  |  |  |  |  |  |  | *abce-1* | ABC transporter, class E |
|  |  |  |  |  |  |  |  |  |  |  |  |  |  |  |  |  |  |  |  |  |  |  |  |  |  |  |  |  |  |  |  |  |  |  |  |  |  | *eif-3.B* | Eukaryotic Initiation Factor |
|  |  |  |  |  |  |  |  |  |  |  |  |  |  |  |  |  |  |  |  |  |  |  |  |  |  |  |  |  |  |  |  |  |  |  |  |  |  | F58G11.2 |  |
|  |  |  |  |  |  |  |  |  |  |  |  |  |  |  |  |  |  |  |  |  |  |  |  |  |  |  |  |  |  |  |  |  |  |  |  |  |  | *sec-61* | yeast SEC homolog |
|  |  |  |  |  |  |  |  |  |  |  |  |  |  |  |  |  |  |  |  |  |  |  |  |  |  |  |  |  |  |  |  |  |  |  |  |  |  | *aco-1* | ACOnitase |
|  |  |  |  |  |  |  |  |  |  |  |  |  |  |  |  |  |  |  |  |  |  |  |  |  |  |  |  |  |  |  |  |  |  |  |  |  |  | F07F6.4 |  |
|  |  |  |  |  |  |  |  |  |  |  |  |  |  |  |  |  |  |  |  |  |  |  |  |  |  |  |  |  |  |  |  |  |  |  |  |  |  | *cars-1* | Cysteinyl Amino-acyl tRNA Synthetase |
|  |  |  |  |  |  |  |  |  |  |  |  |  |  |  |  |  |  |  |  |  |  |  |  |  |  |  |  |  |  |  |  |  |  |  |  |  |  | F09F7.4 |  |
|  |  |  |  |  |  |  |  |  |  |  |  |  |  |  |  |  |  |  |  |  |  |  |  |  |  |  |  |  |  |  |  |  |  |  |  |  |  | Y39G10AR.21 |  |
|  |  |  |  |  |  |  |  |  |  |  |  |  |  |  |  |  |  |  |  |  |  |  |  |  |  |  |  |  |  |  |  |  |  |  |  |  |  | *pab-1* | PolyA Binding protein |
|  |  |  |  |  |  |  |  |  |  |  |  |  |  |  |  |  |  |  |  |  |  |  |  |  |  |  |  |  |  |  |  |  |  |  |  |  |  | *lpd-6* | LiPid Depleted |
|  |  |  |  |  |  |  |  |  |  |  |  |  |  |  |  |  |  |  |  |  |  |  |  |  |  |  |  |  |  |  |  |  |  |  |  |  |  | Y48B6A.1 |  |
|  |  |  |  |  |  |  |  |  |  |  |  |  |  |  |  |  |  |  |  |  |  |  |  |  |  |  |  |  |  |  |  |  |  |  |  |  |  | Y53F4B.4 |  |
|  |  |  |  |  |  |  |  |  |  |  |  |  |  |  |  |  |  |  |  |  |  |  |  |  |  |  |  |  |  |  |  |  |  |  |  |  |  | *rrbs-1* | Regulator of RiBosome bioSynthesis |
|  |  |  |  |  |  |  |  |  |  |  |  |  |  |  |  |  |  |  |  |  |  |  |  |  |  |  |  |  |  |  |  |  |  |  |  |  |  | T06E6.1 |  |
|  |  |  |  |  |  |  |  |  |  |  |  |  |  |  |  |  |  |  |  |  |  |  |  |  |  |  |  |  |  |  |  |  |  |  |  |  |  | *mrps-31* | Mitochondrial Ribosomal Protein, Small |
|  |  |  |  |  |  |  |  |  |  |  |  |  |  |  |  |  |  |  |  |  |  |  |  |  |  |  |  |  |  |  |  |  |  |  |  |  |  | *rpac-40* | RNA Polymerase I/III (A/C) shared subunit |
|  |  |  |  |  |  |  |  |  |  |  |  |  |  |  |  |  |  |  |  |  |  |  |  |  |  |  |  |  |  |  |  |  |  |  |  |  |  | C09H10.5 |  |
|  |  |  |  |  |  |  |  |  |  |  |  |  |  |  |  |  |  |  |  |  |  |  |  |  |  |  |  |  |  |  |  |  |  |  |  |  |  | *ech-5* | Enoyl-CoA Hydratase |
|  |  |  |  |  |  |  |  |  |  |  |  |  |  |  |  |  |  |  |  |  |  |  |  |  |  |  |  |  |  |  |  |  |  |  |  |  |  | R09F10.8 |  |
|  |  |  |  |  |  |  |  |  |  |  |  |  |  |  |  |  |  |  |  |  |  |  |  |  |  |  |  |  |  |  |  |  |  |  |  |  |  | *ceh-83* | C. Elegans Homeobox |
|  |  |  |  |  |  |  |  |  |  |  |  |  |  |  |  |  |  |  |  |  |  |  |  |  |  |  |  |  |  |  |  |  |  |  |  |  |  | *kin-34* | protein KINase |
|  |  |  |  |  |  |  |  |  |  |  |  |  |  |  |  |  |  |  |  |  |  |  |  |  |  |  |  |  |  |  |  |  |  |  |  |  |  | Y105E8A.14 |  |
|  |  |  |  |  |  |  |  |  |  |  |  |  |  |  |  |  |  |  |  |  |  |  |  |  |  |  |  |  |  |  |  |  |  |  |  |  |  | *fbxa-216* | F-box A protein |
|  |  |  |  |  |  |  |  |  |  |  |  |  |  |  |  |  |  |  |  |  |  |  |  |  |  |  |  |  |  |  |  |  |  |  |  |  |  | *snpc-1.2* | SNAPc (Small Nuclear RNA Activating Complex) homolog |
|  |  |  |  |  |  |  |  |  |  |  |  |  |  |  |  |  |  |  |  |  |  |  |  |  |  |  |  |  |  |  |  |  |  |  |  |  |  | Y41D4B.4 |  |
|  |  |  |  |  |  |  |  |  |  |  |  |  |  |  |  |  |  |  |  |  |  |  |  |  |  |  |  |  |  |  |  |  |  |  |  |  |  | *vab-15* | Variable ABnormal morphology |
|  |  |  |  |  |  |  |  |  |  |  |  |  |  |  |  |  |  |  |  |  |  |  |  |  |  |  |  |  |  |  |  |  |  |  |  |  |  | *gpn-1* | GlyPicaN |
|  |  |  |  |  |  |  |  |  |  |  |  |  |  |  |  |  |  |  |  |  |  |  |  |  |  |  |  |  |  |  |  |  |  |  |  |  |  | B0361.6 |  |
|  |  |  |  |  |  |  |  |  |  |  |  |  |  |  |  |  |  |  |  |  |  |  |  |  |  |  |  |  |  |  |  |  |  |  |  |  |  | D2030.3 |  |
|  |  |  |  |  |  |  |  |  |  |  |  |  |  |  |  |  |  |  |  |  |  |  |  |  |  |  |  |  |  |  |  |  |  |  |  |  |  | F36G3.2 |  |
|  |  |  |  |  |  |  |  |  |  |  |  |  |  |  |  |  |  |  |  |  |  |  |  |  |  |  |  |  |  |  |  |  |  |  |  |  |  | Y49F6B.2 |  |
|  |  |  |  |  |  |  |  |  |  |  |  |  |  |  |  |  |  |  |  |  |  |  |  |  |  |  |  |  |  |  |  |  |  |  |  |  |  | ZK1098.4 |  |
|  |  |  |  |  |  |  |  |  |  |  |  |  |  |  |  |  |  |  |  |  |  |  |  |  |  |  |  |  |  |  |  |  |  |  |  |  |  | *dnj-14* | DNaJ domain (prokaryotic heat shock protein) |
|  |  |  |  |  |  |  |  |  |  |  |  |  |  |  |  |  |  |  |  |  |  |  |  |  |  |  |  |  |  |  |  |  |  |  |  |  |  | F42A10.5 |  |
|  |  |  |  |  |  |  |  |  |  |  |  |  |  |  |  |  |  |  |  |  |  |  |  |  |  |  |  |  |  |  |  |  |  |  |  |  |  | B0280.9 |  |
|  |  |  |  |  |  |  |  |  |  |  |  |  |  |  |  |  |  |  |  |  |  |  |  |  |  |  |  |  |  |  |  |  |  |  |  |  |  | T20B12.3 |  |
|  |  |  |  |  |  |  |  |  |  |  |  |  |  |  |  |  |  |  |  |  |  |  |  |  |  |  |  |  |  |  |  |  |  |  |  |  |  | R05H10.3 |  |
|  |  |  |  |  |  |  |  |  |  |  |  |  |  |  |  |  |  |  |  |  |  |  |  |  |  |  |  |  |  |  |  |  |  |  |  |  |  | *nhr-13* | Nuclear Hormone Receptor family |
|  |  |  |  |  |  |  |  |  |  |  |  |  |  |  |  |  |  |  |  |  |  |  |  |  |  |  |  |  |  |  |  |  |  |  |  |  |  | *fah-1* | FumarylAcetoacetate Hydrolase |
|  |  |  |  |  |  |  |  |  |  |  |  |  |  |  |  |  |  |  |  |  |  |  |  |  |  |  |  |  |  |  |  |  |  |  |  |  |  | *mct-6* | MonoCarboxylate Transporter family |
|  |  |  |  |  |  |  |  |  |  |  |  |  |  |  |  |  |  |  |  |  |  |  |  |  |  |  |  |  |  |  |  |  |  |  |  |  |  | M01F1.8 |  |
|  |  |  |  |  |  |  |  |  |  |  |  |  |  |  |  |  |  |  |  |  |  |  |  |  |  |  |  |  |  |  |  |  |  |  |  |  |  | *ile-2* | Intracellular LEctin |

### Phenotypes enriched

|  |  |  |  |
| --- | --- | --- | --- |
| **Group name** | **Number in cluster** | **Enrichment** | **FDR corrected p** |
| slow growth (RNAi) | 81 | 2.82 | 3.37e-15 |
| growth variant (RNAi) | 93 | 2.13 | 1.03e-10 |
| pattern of transgene expression variant (RNAi) | 51 | 3.10 | 1.14e-09 |
| organ system physiology variant (RNAi) | 79 | 2.23 | 2.54e-09 |
| transgene expression variant (RNAi) | 57 | 2.79 | 2.57e-09 |
| receptor mediated endocytosis defective (RNAi) | 39 | 3.72 | 4.82e-09 |
| oocyte physiology variant (RNAi) | 39 | 3.70 | 5.75e-09 |
| reproductive system physiology variant (RNAi) | 78 | 2.21 | 6.01e-09 |
| fertility variant (RNAi) | 77 | 2.19 | 1.25e-08 |
| endocytic transport defect (RNAi) | 39 | 3.59 | 1.33e-08 |
| endocytic transport variant (RNAi) | 39 | 3.57 | 1.55e-08 |
| gene expression variant (RNAi) | 58 | 2.57 | 3.70e-08 |
| sterile (RNAi) | 68 | 2.31 | 4.34e-08 |
| fertility reduced (RNAi) | 68 | 2.30 | 5.11e-08 |
| hermaphrodite fertility variant (RNAi) | 63 | 2.38 | 8.17e-08 |
| larval arrest (RNAi) | 60 | 2.44 | 1.06e-07 |
| developmental growth variant (RNAi) | 60 | 2.44 | 1.07e-07 |
| larval growth variant (RNAi) | 60 | 2.44 | 1.09e-07 |
| maternal sterile (RNAi) | 43 | 2.81 | 1.73e-06 |
| hermaphrodite sterile (RNAi) | 43 | 2.81 | 1.77e-06 |
| hermaphrodite fertility reduced (RNAi) | 43 | 2.81 | 1.77e-06 |
| larval development variant (RNAi) | 60 | 2.26 | 1.92e-06 |
| embryonic development variant (RNAi) | 86 | 1.84 | 3.45e-06 |
| postembryonic development variant (RNAi) | 61 | 2.18 | 5.40e-06 |
| embryonic lethal (RNAi) | 85 | 1.83 | 5.73e-06 |
| organism homeostasis metabolism variant (RNAi) | 106 | 1.63 | 9.78e-06 |
| organism development variant (RNAi) | 99 | 1.65 | 2.75e-05 |
| organism metabolism processing variant (RNAi) | 64 | 2.01 | 3.79e-05 |
| organism physiology variant (RNAi) | 108 | 1.57 | 4.77e-05 |
| lethal (RNAi) | 88 | 1.70 | 7.46e-05 |
| development variant (RNAi) | 101 | 1.57 | 2.06e-04 |
| physiology variant (RNAi) | 115 | 1.43 | 2.00e-03 |
| progeny variant (RNAi) | 29 | 2.58 | 4.21e-03 |
| sterile progeny (RNAi) | 29 | 2.58 | 4.21e-03 |
| morphology variant (RNAi) | 58 | 1.80 | 5.56e-03 |
| larval lethal (RNAi) | 30 | 2.36 | 1.29e-02 |
| cell physiology variant (RNAi) | 45 | 1.91 | 1.81e-02 |
| early larval arrest (RNAi) | 12 | 4.49 | 1.92e-02 |
| organism morphology variant (RNAi) | 35 | 2.08 | 3.03e-02 |

### Anatomy terms enriched

|  |  |  |  |
| --- | --- | --- | --- |
| **Group name** | **Number in cluster** | **Enrichment** | **FDR corrected p** |
| intestine | 59 | 2.06 | 8.16e-05 |
| digestive tract | 68 | 1.84 | 3.76e-04 |
| pharynx | 47 | 2.17 | 6.33e-04 |
| epithelial system | 43 | 2.24 | 9.26e-04 |
| alimentary system | 68 | 1.80 | 9.26e-04 |
| hypodermis | 40 | 2.29 | 1.40e-03 |
| body wall musculature | 36 | 2.37 | 2.47e-03 |
| muscular system | 46 | 2.07 | 2.97e-03 |
| spermatheca | 20 | 3.16 | 1.21e-02 |
| hermaphrodite-specific | 45 | 1.96 | 1.47e-02 |
| epithelial cell | 28 | 2.47 | 1.78e-02 |
| organ | 72 | 1.61 | 1.79e-02 |
| Sex specific entity | 46 | 1.92 | 1.87e-02 |
| hermaphrodite gonad | 26 | 2.46 | 3.54e-02 |

### GO terms enriched

|  |  |  |
| --- | --- | --- |
| **GO term** | **Number of genes** | **FDR-corrected p-value** |
| positive regulation of growth rate | 61 | 1.6e-10 |
| regulation of growth | 64 | 1.4e-08 |
| positive regulation of biological process | 66 | 3.4e-07 |
| receptor-mediated endocytosis | 36 | 7.3e-07 |
| vesicle-mediated transport | 39 | 1.8e-05 |
| heterocyclic compound binding | 61 | 4.3e-05 |
| organic cyclic compound binding | 61 | 4.8e-05 |
| nematode larval development | 62 | 1.5e-04 |
| post-embryonic development | 62 | 2.0e-04 |
| embryo development ending in birth or egg hatching | 76 | 3.6e-04 |
| nucleotide binding | 33 | 6.9e-04 |
| ligase activity, forming aminoacyl-tRNA and related compounds | 6 | 7.3e-04 |
| cytoplasm | 49 | 8.2e-04 |
| tRNA aminoacylation | 6 | 1.4e-03 |
| oxoacid metabolic process | 15 | 1.6e-03 |
| metal cluster binding | 6 | 1.7e-03 |
| ncRNA metabolic process | 10 | 1.8e-03 |
| reproduction | 35 | 2.1e-03 |
| 4 iron, 4 sulfur cluster binding | 5 | 2.8e-03 |
| anion binding | 32 | 3.1e-03 |
| cofactor binding | 11 | 3.3e-03 |
| cellular amino acid metabolic process | 11 | 3.9e-03 |
| small molecule metabolic process | 28 | 4.4e-03 |
| anatomical structure development | 88 | 6.1e-03 |
| flavin adenine dinucleotide binding | 6 | 6.7e-03 |
| mitochondrion | 14 | 7.0e-03 |
| carbohydrate derivative binding | 29 | 1.3e-02 |
| purine nucleoside binding | 28 | 1.6e-02 |
| ribonucleoside binding | 28 | 1.6e-02 |
| purine ribonucleotide binding | 28 | 1.7e-02 |
| cellular respiration | 5 | 2.4e-02 |
| oxidation-reduction process | 16 | 3.5e-02 |
| biological regulation | 91 | 3.8e-02 |

### Expression clusters enriched

|  |  |  |  |
| --- | --- | --- | --- |
| **Group name** | **Number in cluster** | **Enrichment** | **FDR corrected p** |
| Caenorhabditis elegans Genes with expression levels changed significantly after treatment of Xenorhabdus nematophila. | 181 | 1.88 | 2.82e-29 |
| Maternal class (M): genes that are called present in at least one of the three PC6 replicates. | 149 | 1.85 | 4.15e-18 |
| Genes in the top 10% of expression level across the triplicate L3 samples. To generate the top10 and bottom10 gene sets, authors ranked all genes by mean expression array signal intensity across the three replicates, then took the top and bottom deciles (1,841 genes each) to represent genes with high and low expression. | 76 | 2.99 | 2.26e-16 |
| Caenorhabditis elegans Genes with expression levels changed significantly after treatment of Bacillus thurigiensis DB27. | 124 | 1.87 | 3.17e-13 |
| Maternal-embryonic class (ME): genes that are in the intersection of the maternal and embryonic classes. | 84 | 2.29 | 1.01e-11 |
| Genes up or down regulated by 10e-09M of testosterone. The normalized values used were G/R ratio > 2.6 for up-regulation and G/R ratio < 0.38 for down-regulation, which corresponds to 1.39 and -1.39 log(base2) G/R ratio, respectively. | 102 | 1.98 | 3.67e-11 |
| Genes up or down regulated by 10e-07M of progesterone. The normalized values used were G/R ratio > 2.6 for up-regulation and G/R ratio < 0.38 for down-regulation, which corresponds to 1.39 and -1.39 log(base2) G/R ratio, respectively. | 93 | 1.98 | 1.21e-09 |
| Genome-wide analysis of developmental and sex-regulated gene expression profile. cgc4489\_group\_3 | 39 | 3.63 | 1.60e-09 |
| Embryonic class (E): genes that significantly increase in abundance at some point during embryogenesis. | 95 | 1.93 | 3.06e-09 |
| FBF-associated probe sets (FDR <2.25%) | 90 | 1.73 | 4.59e-06 |
| Class A gene expression showed down regulation in lin-14(lf) in L1, no change in lin-4(lf) in L2. | 14 | 7.23 | 4.67e-06 |
| Genes with expression level up in zfp-1 mutant background. | 20 | 4.37 | 1.75e-05 |
| Genes that are up or down regulated by more than 2.1 fold with the t-test p-value less than 0.01 are included in this cluster. | 42 | 2.47 | 1.81e-05 |
| Genes expressed in N2. | 160 | 1.31 | 3.83e-05 |
| TGF- Dauer pathway adult transcriptional targets. Results obtained by comparing the microarray results of the dauer-constitutive mutants daf-7(e1372), daf-7(m62), and daf-1(m40) with dauer-defective mutants daf-3(mgDf90), daf-5(e1386), and daf-7(e1372);daf-3(mgDf90) double mutants at the permissive temperature, 20C, on the first day of adulthood. WBPaper00031040:TGF-beta\_adult\_downregulated | 94 | 1.62 | 4.22e-05 |
| Caenorhabditis elegans Genes with expression levels changed significantly after treatment of Serratia marcescens. | 83 | 1.69 | 5.45e-05 |
| A complete list of the genes that showed differential expression in a slr-2 mutant strain. | 50 | 2.10 | 9.89e-05 |
| A large cluster of genes up-regulated during early larval development.. | 51 | 2.01 | 2.76e-04 |
| Proteins that showed decreased expression 24h after infection with S. aureus. | 7 | 12.05 | 5.90e-04 |
| C-lineage related expression profile. WBPaper00025032:cluster\_2 | 14 | 4.44 | 1.25e-03 |
| Embryonic (E) subclasses are based on the earliest significant increase(abbreviated pi for primary increase). [cgc5767]:expression\_class\_E\_pi(23\_min) | 30 | 2.40 | 2.33e-03 |
| Genes down regulated in the absence of TDP-1, when the threshold was set at a fold change (FC) of 1.2. | 58 | 1.76 | 2.40e-03 |
| Genes up or down regulated by 10e-05M of estrogen. The normalized values used were G/R ratio > 2.6 for up-regulation and G/R ratio < 0.38 for down-regulation, which corresponds to 1.39 and -1.39 log(base2) G/R ratio, respectively. | 38 | 2.08 | 3.45e-03 |
| Maternal degradation class (MD): genes that are the subset of maternal genes that decrease without first increasing in abundance. | 46 | 1.91 | 3.54e-03 |
| C-lineage related expression profile. WBPaper00025032:cluster\_36 | 6 | 10.84 | 4.98e-03 |
| The cluster contains genes that are significantly enriched in L1 muscle. | 39 | 1.95 | 9.97e-03 |
| Genes significantly enriched (> 2x, FDR < 5%) in a particular cell-type versus a reference sample of all cells at the same stage. WBPaper00037950:bodywall-muscle\_larva\_enriched | 33 | 2.08 | 1.27e-02 |
| C-lineage related expression profile. WBPaper00025032:cluster\_7 | 9 | 5.33 | 1.29e-02 |
| Early embryonic development gene expression profile. [cgc5767]:cluster\_2 | 17 | 2.90 | 2.14e-02 |
| Genes that showed higher expression in N2 than in DR1350. | 38 | 1.85 | 3.37e-02 |
| Gene significantly up-regulated by treatment with 2.0mM of HuminFeed until older adult stage (11 days), with a minimum fold change in gene expression of 1.25. | 22 | 2.35 | 4.05e-02 |
| Genes that showed expression levels higher than the corresponding reference sample (L3/L4 all cell reference). | 109 | 1.31 | 4.54e-02 |
| coenzymes and prosthetic group metabolism; 7.5e-05(9.95x). cofactor metabolism; 7.5e-05(9.95x). coenzyme metabolism; 7.5e-05(9.95x). monovalent inorganic cation transporter activity; 1.3e-04(13.27x). hydrogen ion transporter activity; 1.3e-04(13.27x). primary active transporter activity; 4.5e-04(9.95x). energy pathways; 7.5e-04(4.98x). | 4 | 13.14 | 4.64e-02 |
| Maternal degradation-embryonic class (MDE): genes that are the subset of maternal degradation genes that significantly increase in at least two of the eight total paired timepoint tests in the induction-following-degradation time domain. | 21 | 2.38 | 4.74e-02 |

### Motifs enriched

|  |  |  |  |  |  |
| --- | --- | --- | --- | --- | --- |
| **Motif** | **Logo** | **Possible orthologs** | **Number of motifs in cluster** | **Enrichment** | **FDR corrected p** |
| CG2052\_SANGER\_2.5\_FBgn0039905 |  | fkh-7 (0.8) mel-28 (0.75) lin-29 | 189 | 1.39 | 2.5e-12 |
| Fkh1 |  | fkh-7 (0.8) daf-16 (0.64) pha-4 (0.6) fkh-8 lin-31 let-381 | 156 | 1.58 | 7.0e-12 |
| FOXD3\_f1 |  | lin-31 let-381 | 178 | 1.43 | 1.4e-11 |
| ZN384\_f1 |  | lin-29 K11D2.4 | 188 | 1.37 | 1.5e-11 |
| FOXO1\_si |  | irx-1 (0.75) daf-16 (0.64) fkh-9 | 173 | 1.45 | 4.6e-11 |
| pnr\_SANGER\_5\_FBgn0003117 |  | elt-1 | 156 | 1.55 | 4.8e-11 |
| Mv90 |  | mef-2 | 178 | 1.42 | 5.6e-11 |
| ONEC2\_si |  | ceh-48 dsc-1 | 172 | 1.45 | 6.7e-11 |
| pTH9242 |  | mel-28 (0.75) | 179 | 1.41 | 6.8e-11 |
| pTH2846 |  | lin-31 | 155 | 1.54 | 8.5e-11 |
| pTH6108 |  | fkh-10 lin-31 let-381 C34D1.1 | 155 | 1.54 | 1.3e-10 |
| pTH9180 |  | mel-28 (0.75) mef-2 let-381 Y61A9LA.9 Y116A8C.22 | 181 | 1.38 | 2.2e-10 |
| MA0541.1 |  | efl-1 (0.64) F49E12.6 | 112 | 1.81 | 1.0e-09 |
| pTH9222 |  | mel-28 (0.75) | 174 | 1.40 | 1.1e-09 |
| FOXJ3\_1 |  | fkh-7 (0.8) daf-16 (0.64) fkh-8 fkh-10 lin-31 let-381 | 173 | 1.40 | 1.4e-09 |
| pTH6641 |  | lin-31 | 151 | 1.52 | 1.8e-09 |
| pTH9260 |  | mel-28 (0.75) | 171 | 1.41 | 2.3e-09 |
| MA0049.1 |  | php-3 hbl-1 lin-39 | 175 | 1.38 | 3.7e-09 |
| pTH9097 |  | Y116A8C.22 | 178 | 1.36 | 6.8e-09 |
| pTH9951 |  | pal-1 (0.64) mex-6 | 164 | 1.41 | 1.6e-08 |
| pTH9254 |  | mel-28 (0.75) | 171 | 1.38 | 1.7e-08 |
| eve\_FlyReg\_FBgn0000606 |  | hmbx-1 ceh-53 lin-31 lin-39 let-381 hmg-12 Y116A8C.22 | 148 | 1.49 | 1.7e-08 |
| HXD10\_f1 |  | nhr-2 php-3 | 155 | 1.45 | 2.8e-08 |
| MA0543.1 |  | eor-1 (0.61) daf-8 (0.54) | 162 | 1.41 | 3.2e-08 |
| HES1\_f1 |  | lin-22 | 98 | 1.81 | 5.7e-08 |
| pTH9082 |  | mab-23 | 166 | 1.38 | 6.1e-08 |
| pTH9958 |  | ztf-6 (0.82) ztf-2 | 150 | 1.45 | 8.4e-08 |
| Blimp-1\_SANGER\_5\_FBgn0035625 |  | blmp-1 | 172 | 1.35 | 9.4e-08 |
| pTH9177 |  | hsf-1 (0.59) F10B5.3 Y53C10A.3 | 156 | 1.42 | 1.0e-07 |
| exd\_FlyReg\_FBgn0000611 |  | ceh-20 cfi-1 let-381 | 179 | 1.32 | 1.1e-07 |
| Eip93F\_SANGER\_10\_FBgn0013948 |  | nhr-177 mbr-1 bed-3 | 145 | 1.47 | 1.2e-07 |
| V$FAC1\_01 |  | gei-8 (0.65) | 157 | 1.41 | 1.3e-07 |
| rn\_SOLEXA\_5\_FBgn0259172 |  | lin-29 | 173 | 1.34 | 1.7e-07 |
| pTH3477 |  | daf-16 (0.64) | 143 | 1.47 | 1.9e-07 |
| MA0547.1 |  | skn-1 | 158 | 1.40 | 2.6e-07 |
| pTH9335 |  | mel-28 (0.75) | 157 | 1.40 | 3.2e-07 |
| MA0244.1 |  | C48E7.11 | 140 | 1.47 | 3.7e-07 |
| pTH9125 |  | sox-4 K11D2.4 egl-13 | 160 | 1.38 | 5.2e-07 |
| Zfp161\_2858 |  | pzf-1 | 80 | 1.91 | 6.4e-07 |
| Arid3a\_3875 |  | cfi-1 | 166 | 1.34 | 9.8e-07 |
| V$ARNT\_01 |  | mdl-1 (-0.59) aha-1 (0.58) hlh-30 | 95 | 1.74 | 1.1e-06 |
| CXXC1\_si |  | F52B11.1 F21D5.4 | 104 | 1.66 | 1.3e-06 |
| Mafk\_3106 |  | F45H11.6 | 136 | 1.47 | 1.3e-06 |
| pTH9709 |  | die-1 (0.74) | 150 | 1.40 | 1.5e-06 |
| pTH8566 |  | lin-54 | 151 | 1.39 | 1.9e-06 |
| CG31670\_SANGER\_5\_FBgn0031375 |  | CELE\_Y38H8A.5 | 146 | 1.41 | 1.9e-06 |
| pTH6497 |  | lin-31 | 140 | 1.44 | 2.0e-06 |
| pTH1294 |  | mel-28 (0.75) | 88 | 1.77 | 2.3e-06 |
| MA0198.1 |  | ceh-16 (0.69) ceh-18 (-0.56) pha-2 (0.51) ceh-45 lim-4 egl-5 ceh-36 ceh-14 lin-39 alr-1 lim-7 ceh-43 lim-6 ZC123.3 | 138 | 1.45 | 2.3e-06 |
| POU4F1\_1 |  | ceh-18 (-0.56) unc-86 | 155 | 1.37 | 2.4e-06 |
| Dll\_Cell\_FBgn0000157 |  | ceh-18 (-0.56) ceh-43 | 142 | 1.42 | 3.9e-06 |
| HLH26 |  | ref-1 (0.58) aha-1 (0.58) mxl-2 (-0.53) hlh-30 mxl-1 hlh-26 lin-22 C27D6.4 | 107 | 1.60 | 4.0e-06 |
| ARI3A\_do |  | gei-3 (-0.58) cfi-1 lim-6 | 179 | 1.27 | 4.5e-06 |
| pTH9173 |  | efl-2 | 78 | 1.85 | 4.7e-06 |
| pTH3046 |  | Y116A8C.22 | 107 | 1.60 | 4.8e-06 |
| pTH9189 |  | ceh-18 (-0.56) dmd-3 | 149 | 1.38 | 6.0e-06 |
| pTH5250 |  | C48E7.11 | 116 | 1.54 | 6.1e-06 |
| pTH4425 |  | unc-86 cfi-1 lim-7 | 130 | 1.46 | 6.3e-06 |
| pTH6591 |  | lin-31 | 158 | 1.34 | 7.6e-06 |
| SRP000217\_Sox2 |  | ceh-18 (-0.56) sox-4 ceh-6 | 164 | 1.31 | 8.8e-06 |
| cad\_FlyReg\_FBgn0000251 |  | pal-1 (0.64) ceh-24 ceh-13 lin-39 | 139 | 1.41 | 9.4e-06 |
| pTH10638 |  | dmd-3 C34D1.1 | 139 | 1.41 | 1.0e-05 |
| V$BRN2\_01 |  | ceh-18 (-0.56) | 163 | 1.31 | 1.1e-05 |
| pTH8863 |  | hmg-12 | 110 | 1.55 | 1.5e-05 |
| Eip74EF\_FlyReg\_FBgn0000567 |  | C24A1.2 | 143 | 1.38 | 1.9e-05 |
| pTH5916 |  | efl-2 | 78 | 1.77 | 2.4e-05 |
| MSX1\_1 |  | ceh-31 (-0.71) ceh-14 ceh-1 ceh-43 | 132 | 1.42 | 2.6e-05 |
| pTH9296 |  | ztf-6 (0.82) C34D1.1 gei-11 | 133 | 1.41 | 2.7e-05 |
| Abd-A\_FlyReg\_FBgn0000014 |  | ceh-45 lin-39 ceh-1 alr-1 | 139 | 1.39 | 2.7e-05 |
| pTH7875 |  | mel-28 (0.75) | 117 | 1.48 | 3.5e-05 |
| Atf1\_3026 |  | crh-1 | 100 | 1.58 | 3.5e-05 |
| Tbp\_pr781 |  | tbp-1 | 129 | 1.42 | 3.7e-05 |
| pnt\_SANGER\_5\_FBgn0003118 |  | lin-1 C24A1.2 | 129 | 1.42 | 4.1e-05 |
| Mv109 |  | pax-3 pax-2 | 77 | 1.75 | 4.1e-05 |
| Eip74EF\_SANGER\_5\_FBgn0000567 |  | lin-1 C24A1.2 | 112 | 1.50 | 4.7e-05 |
| V$NKX61\_01 |  | ceh-9 (-0.55) cog-1 lin-39 alr-1 ceh-19 ceh-43 | 142 | 1.37 | 4.7e-05 |
| pTH9711 |  | ces-2 (0.82) atf-2 Y51H4A.4 C48E7.11 C01B12.2 | 90 | 1.63 | 5.1e-05 |
| pTH10633 |  | R07H5.10 C48E7.11 | 144 | 1.35 | 5.4e-05 |
| E2F4\_1 |  | nfi-1 (0.58) F49E12.6 | 99 | 1.57 | 6.0e-05 |
| ALX1\_si |  | cfi-1 ceh-14 alr-1 | 131 | 1.40 | 7.3e-05 |
| EMX2\_2 |  | ceh-16 (0.69) ceh-2 | 117 | 1.46 | 7.4e-05 |
| NR2E3\_f1 |  | nhr-100 lin-1 lin-39 | 154 | 1.31 | 7.4e-05 |
| CUX1\_2 |  | ceh-48 | 121 | 1.44 | 7.8e-05 |
| Irx3\_1 |  | irx-1 (0.75) | 124 | 1.43 | 8.2e-05 |
| HXB8\_do |  | ceh-20 lin-39 | 133 | 1.39 | 8.5e-05 |
| pTH3220 |  | ceh-9 (-0.55) Y5F2A.4 daf-12 | 104 | 1.52 | 8.6e-05 |
| pTH5257 |  | C48E7.11 | 99 | 1.55 | 8.9e-05 |
| pTH2933 |  | F58G1.2 (0.62) | 54 | 1.99 | 1.4e-04 |
| pTH9137 |  | nhr-65 | 148 | 1.32 | 1.5e-04 |
| V$RORA1\_01 |  | nhr-68 nhr-2 nhr-71 nhr-213 nhr-6 | 103 | 1.50 | 1.9e-04 |
| pTH0978 |  | klf-2 (0.51) ZC328.2 | 82 | 1.63 | 2.3e-04 |
| pTH9237 |  | mel-28 (0.75) | 142 | 1.33 | 2.3e-04 |
| pTH10837 |  | ces-1 (0.63) T22H9.4 | 144 | 1.32 | 2.5e-04 |
| SP4\_f1 |  | klf-2 (0.51) plp-2 | 64 | 1.80 | 2.6e-04 |
| ZNF75A\_1 |  | lag-1 (0.58) ztf-3 | 125 | 1.39 | 3.0e-04 |
| pTH9969 |  | pag-3 | 119 | 1.41 | 3.1e-04 |
| RORA\_f1 |  | nhr-118 nhr-213 | 100 | 1.50 | 3.1e-04 |
| pTH9135 |  | pop-1 (0.85) | 135 | 1.35 | 3.1e-04 |
| NR2F6\_f1 |  | nhr-2 nhr-239 nhr-62 | 137 | 1.34 | 3.2e-04 |
| pTH9900 |  | C46E10.8 C33G8.2 | 109 | 1.45 | 3.4e-04 |
| Sox11\_2266 |  | pop-1 (0.85) gei-3 (-0.58) sox-4 C05C9.3 | 141 | 1.33 | 3.4e-04 |
| TBP\_f1 |  | tbp-1 | 126 | 1.38 | 3.6e-04 |
| I$ABDB\_01 |  | pal-1 (0.64) php-3 ceh-24 lin-39 | 85 | 1.59 | 3.6e-04 |
| MA0531.1 |  | F58G1.2 (0.62) Y5F2A.4 | 88 | 1.56 | 3.9e-04 |
| MA0536.1 |  | elt-1 lin-39 | 103 | 1.48 | 3.9e-04 |
| pTH10816 |  | dmd-6 | 149 | 1.30 | 4.1e-04 |
| ss\_tgo\_SANGER\_10\_FBgn0015014 |  | aha-1 (0.58) ahr-1 | 73 | 1.68 | 4.1e-04 |
| MA0545.1 |  | lin-32 hlh-1 | 93 | 1.52 | 4.9e-04 |
| MA0037.2 |  | ztf-29 elt-1 | 107 | 1.45 | 5.0e-04 |
| Gmeb1\_1745 |  | attf-1 | 66 | 1.73 | 5.4e-04 |
| ARNT2\_si |  | aha-1 (0.58) C46E10.9 | 83 | 1.58 | 5.5e-04 |
| V$TAXCREB\_01 |  | crh-1 attf-1 W08E12.1 | 78 | 1.62 | 5.7e-04 |
| MA0535.1 |  | daf-8 (0.54) pax-2 | 72 | 1.67 | 6.1e-04 |
| pTH10645 |  | nhr-7 elt-1 | 133 | 1.34 | 6.2e-04 |
| pTH3084 |  | C01B12.2 attf-1 | 64 | 1.74 | 6.3e-04 |
| pTH2280 |  | mnm-2 | 69 | 1.69 | 6.4e-04 |
| Lmx1b\_3433 |  | lim-7 lim-6 | 105 | 1.45 | 6.6e-04 |
| pTH1739 |  | nhr-255 npax-1 lin-14 | 121 | 1.38 | 6.9e-04 |
| RAX\_1 |  | ceh-31 (-0.71) ceh-16 (0.69) ceh-1 alr-1 | 116 | 1.40 | 6.9e-04 |
| V$CDXA\_01 |  | php-3 ceh-13 | 107 | 1.44 | 7.0e-04 |
| pTH9163 |  | nhr-3 | 120 | 1.38 | 7.4e-04 |
| pTH9384 |  | cfi-1 | 148 | 1.29 | 7.6e-04 |
| pTH10772 |  | ceh-52 | 81 | 1.58 | 7.8e-04 |
| Hoxd13\_2356 |  | pal-1 (0.64) | 119 | 1.38 | 8.7e-04 |
| pTH5808 |  | pal-1 (0.64) ceh-24 | 111 | 1.41 | 9.0e-04 |
| pTH8333 |  | ZC416.1 | 85 | 1.54 | 1.0e-03 |
| I$DFD\_01 |  | lin-39 | 94 | 1.49 | 1.1e-03 |
| pTH5082 |  | atf-2 | 93 | 1.49 | 1.1e-03 |
| MA0146.2 |  | F58G1.2 (0.62) | 29 | 2.48 | 1.2e-03 |
| ARNT\_f1 |  | aha-1 (0.58) hlh-27 lin-22 hlh-28 | 73 | 1.62 | 1.2e-03 |
| pTH5337 |  | daf-16 (0.64) ZC328.2 | 74 | 1.61 | 1.3e-03 |
| pTH8998 |  | mab-3 | 105 | 1.43 | 1.3e-03 |
| HIF1A\_si |  | hif-1 | 75 | 1.60 | 1.3e-03 |
| V$DELTAEF1\_01 |  | ztf-6 (0.82) | 83 | 1.54 | 1.4e-03 |
| pTH2820 |  | ZC328.2 | 81 | 1.55 | 1.4e-03 |
| Sox4 |  | pop-1 (0.85) sox-4 nhr-100 | 133 | 1.32 | 1.4e-03 |
| pTH8985 |  | athp-1 (0.71) | 128 | 1.33 | 1.5e-03 |
| pTH9925 |  | ztf-11 (0.75) | 142 | 1.29 | 1.5e-03 |
| MA0461.1 |  | ngn-1 hlh-8 hlh-32 hlh-15 | 81 | 1.55 | 1.6e-03 |
| pTH8982 |  | ceh-48 | 34 | 2.22 | 1.7e-03 |
| Sox17\_2837 |  | sox-4 | 139 | 1.29 | 1.8e-03 |
| pTH10769 |  | Y48G1C.6 | 110 | 1.39 | 1.8e-03 |
| MA0095.2 |  | lsy-2 | 126 | 1.33 | 1.9e-03 |
| pTH4325 |  | ceh-18 (-0.56) | 132 | 1.31 | 1.9e-03 |
| pTH5080 |  | crh-1 fos-1 | 77 | 1.56 | 2.0e-03 |
| CG5669\_SANGER\_10\_FBgn0039169 |  | klf-2 (0.51) klf-1 | 82 | 1.53 | 2.1e-03 |
| pTH10696 |  | Y44A6D.3 | 61 | 1.69 | 2.2e-03 |
| pTH8679 |  | pax-2 | 75 | 1.57 | 2.2e-03 |
| pTH7032 |  | F52B11.1 | 64 | 1.66 | 2.3e-03 |
| V$GATA1\_05 |  | elt-1 | 108 | 1.39 | 2.3e-03 |
| NR2F1\_4 |  | nhr-2 | 94 | 1.45 | 2.4e-03 |
| MA0249.1 |  | ngn-1 hlh-8 hlh-32 | 118 | 1.35 | 2.4e-03 |
| V$FREAC7\_01 |  | lin-31 | 139 | 1.29 | 2.5e-03 |
| pTH8318 |  | attf-1 | 54 | 1.75 | 3.0e-03 |
| Zic1\_0991 |  | ref-2 | 79 | 1.53 | 3.2e-03 |
| V$AP2REP\_01 |  | klf-1 | 88 | 1.47 | 3.3e-03 |
| pTH8556 |  | pax-2 | 60 | 1.68 | 3.3e-03 |
| SOX2\_4 |  | sox-4 grh-1 | 124 | 1.32 | 3.3e-03 |
| pTH9108 |  | daf-12 nhr-5 | 107 | 1.38 | 3.4e-03 |
| CDC5L\_si |  | D1081.8 (0.6) | 120 | 1.34 | 3.4e-03 |
| ZBTB7A\_1 |  | ref-2 ztf-14 ZC328.2 | 68 | 1.60 | 3.7e-03 |
| pTH9924 |  | nhr-46 | 120 | 1.33 | 3.7e-03 |
| pTH10788 |  | tbx-33 | 115 | 1.35 | 3.8e-03 |
| pTH1014 |  | atf-5 | 88 | 1.46 | 3.9e-03 |
| V$FOXJ2\_02 |  | lin-31 | 135 | 1.29 | 3.9e-03 |
| K562\_SP2\_HudsonAlpha |  | klf-2 (0.51) | 94 | 1.43 | 4.1e-03 |
| pTH2193 |  | nhr-2 nhr-15 nhr-239 | 75 | 1.54 | 4.1e-03 |
| HeLa-S3\_ZNF274\_UCD |  | C28G1.4 | 90 | 1.45 | 4.2e-03 |
| pTH8649 |  | mbr-1 | 114 | 1.35 | 4.4e-03 |
| V$NKX25\_01 |  | ceh-22 ceh-24 | 85 | 1.47 | 4.6e-03 |
| I$KR\_01 |  | B0310.2 | 91 | 1.44 | 5.0e-03 |
| MA0262.1 |  | mab-3 | 53 | 1.72 | 5.1e-03 |
| ETV5\_f1 |  | lin-1 C24A1.2 | 90 | 1.44 | 5.2e-03 |
| CrebA\_SANGER\_5\_FBgn0004396 |  | crh-1 atf-6 fos-1 C27D6.4 | 52 | 1.73 | 5.3e-03 |
| GRHL1\_2 |  | grh-1 | 67 | 1.58 | 5.6e-03 |
| pTH9884 |  | tbx-39 | 84 | 1.47 | 5.6e-03 |
| pTH8745 |  | attf-1 | 58 | 1.66 | 5.8e-03 |
| V$CEBP\_01 |  | C48E7.11 | 110 | 1.35 | 5.9e-03 |
| RFX5\_2 |  | daf-19 | 77 | 1.51 | 6.0e-03 |
| Mw160 |  | nhr-68 | 125 | 1.30 | 6.1e-03 |
| pTH5078 |  | ces-2 (0.82) | 86 | 1.45 | 6.4e-03 |
| pTH10650 |  | nhr-153 | 94 | 1.41 | 6.5e-03 |
| H1-hESC\_RFX5\_Stanford |  | daf-19 | 83 | 1.46 | 6.7e-03 |
| pTH10798 |  | Y75B8A.6 | 78 | 1.49 | 6.8e-03 |
| ATF1\_si |  | crh-1 | 82 | 1.47 | 7.1e-03 |
| pTH6486 |  | nhr-145 | 112 | 1.34 | 7.4e-03 |
| V$PAX2\_02 |  | pax-1 | 93 | 1.41 | 7.5e-03 |
| Sox1\_2631 |  | sox-4 | 126 | 1.29 | 7.7e-03 |
| CG8765\_SANGER\_5\_FBgn0036900 |  | H20J04.3 | 117 | 1.32 | 7.9e-03 |
| scrt\_SOLEXA\_2.5\_1\_FBgn0004880 |  | hlh-2 (0.84) ces-1 (0.63) hlh-1 hlh-8 hlh-15 | 103 | 1.36 | 9.0e-03 |
| V$CMYB\_01 |  | D1081.8 (0.6) | 75 | 1.49 | 9.1e-03 |
| pTH5714 |  | nhr-239 | 87 | 1.43 | 9.7e-03 |
| pTH10030 |  | xbp-1 (-0.54) C01B12.2 | 34 | 1.99 | 9.7e-03 |
| Plagl1\_0972 |  | Y53H1A.2 | 54 | 1.66 | 9.9e-03 |
| MGA\_2 |  | mab-9 tbx-42 | 71 | 1.51 | 9.9e-03 |
| Alx3\_3418 |  | npax-3 alr-1 | 109 | 1.34 | 1.0e-02 |
| Gsc\_Cell\_FBgn0010323 |  | ceh-45 ceh-53 | 78 | 1.47 | 1.0e-02 |
| V$AREB6\_02 |  | ztf-6 (0.82) | 84 | 1.44 | 1.1e-02 |
| pTH2283 |  | odd-2 (0.52) | 125 | 1.28 | 1.1e-02 |
| pTH9928 |  | jun-1 | 98 | 1.37 | 1.2e-02 |
| N$SKN1\_02 |  | skn-1 | 115 | 1.31 | 1.2e-02 |
| YY1\_1 |  | lsy-2 | 100 | 1.36 | 1.2e-02 |
| MA0118.1 |  | ref-2 | 63 | 1.56 | 1.2e-02 |
| pTH9907 |  | nhr-34 | 108 | 1.33 | 1.3e-02 |
| pTH10808 |  | ztf-19 | 105 | 1.34 | 1.3e-02 |
| disco-r-Cl1\_SANGER\_5\_FBgn0042650 |  | F55C5.11 | 93 | 1.39 | 1.3e-02 |
| pTH10717 |  | lsy-2 syd-9 | 50 | 1.68 | 1.3e-02 |
| pTH9880 |  | end-1 | 78 | 1.46 | 1.3e-02 |
| pTH10040 |  | slr-2 (0.81) | 104 | 1.34 | 1.5e-02 |
| HAND1\_si |  | hlh-8 | 43 | 1.76 | 1.5e-02 |
| pTH9353 |  | ceh-51 | 93 | 1.38 | 1.5e-02 |
| Hr78\_SANGER\_5\_FBgn0015239 |  | nhr-2 nhr-19 | 70 | 1.50 | 1.5e-02 |
| V$OCT1\_06 |  | ceh-18 (-0.56) | 132 | 1.25 | 1.6e-02 |
| Hoxc8\_3429 |  | lin-39 | 85 | 1.41 | 1.6e-02 |
| pTH9220 |  | mbr-1 | 112 | 1.31 | 1.7e-02 |
| pTH3041 |  | atf-2 | 55 | 1.60 | 1.7e-02 |
| pTH10722 |  | eor-1 (0.61) egrh-3 | 77 | 1.45 | 1.7e-02 |
| V$CDPCR3\_01 |  | ceh-48 | 99 | 1.35 | 1.7e-02 |
| Hoxd10\_2368 |  | php-3 | 107 | 1.32 | 1.8e-02 |
| pTH5118 |  | cfi-1 | 136 | 1.24 | 1.9e-02 |
| pTH9073 |  | end-3 | 96 | 1.35 | 2.0e-02 |
| Hoxa10\_2318 |  | ceh-24 | 97 | 1.35 | 2.0e-02 |
| Zbtb12\_2932 |  | lsy-27 ceh-90 | 89 | 1.38 | 2.0e-02 |
| EGR4\_f1 |  | klf-2 (0.51) ZC328.2 | 72 | 1.46 | 2.0e-02 |
| Mf28 |  | elt-1 | 96 | 1.35 | 2.1e-02 |
| Hoxa11\_2218 |  | php-3 | 90 | 1.37 | 2.2e-02 |
| Elf3\_3876 |  | C24A1.2 | 127 | 1.26 | 2.3e-02 |
| MYF6\_f1 |  | hlh-1 hlh-15 | 75 | 1.44 | 2.3e-02 |
| pTH9198 |  | dmd-3 | 104 | 1.32 | 2.3e-02 |
| Nkx2-6\_3437 |  | dsc-1 | 81 | 1.41 | 2.4e-02 |
| Elf3 |  | C24A1.2 | 124 | 1.26 | 2.4e-02 |
| pTH5928 |  | ceh-34 | 74 | 1.44 | 2.4e-02 |
| pTH6447 |  | ceh-19 | 85 | 1.39 | 2.5e-02 |
| pTH5119 |  | cfi-1 | 135 | 1.23 | 2.6e-02 |
| pTH9326 |  | nhr-122 | 94 | 1.35 | 2.6e-02 |
| pTH10811 |  | nhr-216 | 105 | 1.31 | 2.6e-02 |
| Cdx1\_2245 |  | ceh-13 | 96 | 1.34 | 2.6e-02 |
| pTH9245 |  | ceh-18 (-0.56) | 82 | 1.40 | 2.7e-02 |
| Gata5\_3768 |  | elt-1 | 91 | 1.36 | 2.8e-02 |
| pTH8216 |  | Y116A8C.22 | 97 | 1.33 | 2.9e-02 |
| V$S8\_01 |  | ceh-45 | 108 | 1.30 | 3.0e-02 |
| pTH8983 |  | tag-347 | 95 | 1.34 | 3.1e-02 |
| Hmx1\_3423 |  | ceh-9 (-0.55) | 103 | 1.31 | 3.1e-02 |
| pTH9026 |  | attf-1 | 47 | 1.63 | 3.1e-02 |
| pTH10807 |  | F13H6.1 | 77 | 1.41 | 3.2e-02 |
| FLI1\_4 |  | lin-1 | 86 | 1.37 | 3.4e-02 |
| MA0260.1 |  | che-1 | 101 | 1.31 | 3.5e-02 |
| pTH6327 |  | dsc-1 | 80 | 1.39 | 3.5e-02 |
| pTH10013 |  | nhr-168 | 113 | 1.27 | 3.7e-02 |
| PTF1A\_f1 |  | lin-32 | 99 | 1.31 | 3.8e-02 |
| Smad3\_3805 |  | daf-8 (0.54) | 81 | 1.38 | 3.8e-02 |
| Nsy-7 |  | nsy-7 | 71 | 1.43 | 4.0e-02 |
| MITF\_f1 |  | hlh-30 | 97 | 1.32 | 4.0e-02 |
| pTH9934 |  | Y53H1A.2 | 71 | 1.43 | 4.0e-02 |
| V$GR\_Q6 |  | nhr-255 | 107 | 1.29 | 4.0e-02 |
| Cart1\_1275 |  | alr-1 | 98 | 1.31 | 4.1e-02 |
| pTH9043 |  | sem-2 (0.52) | 117 | 1.26 | 4.2e-02 |
| K562\_ZBTB7A\_HudsonAlpha |  | ZC328.2 | 33 | 1.80 | 4.5e-02 |
| ETS2\_f1 |  | lin-1 | 116 | 1.26 | 4.5e-02 |
| MA0032.1 |  | let-381 | 41 | 1.67 | 4.5e-02 |
| Poxm\_SOLEXA\_5\_FBgn0003129 |  | pax-2 | 68 | 1.43 | 4.8e-02 |
| FLI1\_f1 |  | lin-1 | 50 | 1.56 | 4.9e-02 |
| Mcm1 |  | unc-120 | 81 | 1.37 | 5.0e-02 |

### Correlated (and anti-correlated) transcription factors

|  |  |
| --- | --- |
| **Transcription factor** | **Correlation** |
| efl-3 | 0.93 |
| egl-44 | 0.88 |
| ham-1 | 0.87 |
| duxl-1 | 0.87 |
| sex-1 | 0.86 |
| pop-1 | 0.85 |
| hlh-2 | 0.84 |
| ztf-7 | 0.84 |
| Y82E9BR.17 | 0.84 |
| dnj-11 | 0.83 |
| snpc-1.2 | 0.82 |
| ces-2 | 0.82 |
| ztf-6 | 0.82 |
| attf-2 | 0.82 |
| uaf-2 | 0.81 |
| spr-3 | 0.81 |
| slr-2 | 0.81 |
| R02D3.7 | 0.80 |
| fkh-7 | 0.80 |
| C52E12.1 | 0.78 |
| F23B12.7 | 0.78 |
| bed-2 | 0.78 |
| Y82E9BR.1 | 0.78 |
| swsn-7 | 0.78 |
| ceh-83 | 0.77 |
| xbp-1 | -0.54 |
| nhr-30 | -0.55 |
| ceh-54 | -0.55 |
| ceh-9 | -0.55 |
| nhr-196 | -0.55 |
| unc-42 | -0.55 |
| sdz-38 | -0.56 |
| ceh-18 | -0.56 |
| gei-3 | -0.58 |
| nhr-105 | -0.58 |
| dmd-10 | -0.58 |
| madf-10 | -0.59 |
| mdl-1 | -0.59 |
| zfh-2 | -0.59 |
| zip-4 | -0.61 |
| lfi-1 | -0.61 |
| sox-3 | -0.61 |
| nhr-198 | -0.62 |
| nhr-197 | -0.62 |
| saeg-1 | -0.63 |
| T18D3.7 | -0.65 |
| ceh-31 | -0.71 |
| nhr-222 | -0.71 |
| ceh-88 | -0.79 |
| mbf-1 | -0.82 |

### ChIP peaks enriched

|  |  |  |  |  |
| --- | --- | --- | --- | --- |
| **Gene** | **Experiment** | **Number of upstream peaks** | **Enrichment** | **FDR corrected p** |
| efl-1 | EFL-1\_Young-adult | 136 | 2.68 | 5.0e-32 |
| dpl-1 | DPL-1\_Larvae-L4-stage | 140 | 2.52 | 1.2e-30 |
| dpl-1 | DPL-1\_Fed-L1-stage-larvae | 120 | 2.90 | 1.0e-29 |
| efl-1 | EFL-1\_Fed-L1-stage-larvae | 119 | 2.83 | 2.3e-28 |
| efl-1 | EFL-1\_Larvae-L1-stage | 127 | 2.62 | 8.7e-28 |
| gei-11 | GEI-11\_Fed-L1-stage-larvae | 118 | 2.70 | 3.5e-26 |
| W03F9.2 | W03F9.2\_L4-Young-Adult-stage-larvae | 147 | 2.18 | 6.6e-26 |
| hpl-2 | HPL-2\_Fed-L1-stage-larvae | 127 | 2.49 | 1.2e-25 |
| lin-35 | LIN-35\_Fed-L1-stage-larvae | 114 | 2.68 | 1.7e-24 |
| nhr-23 | NHR-23\_Larvae-L3-stage | 116 | 2.62 | 2.9e-24 |
| ham-1 | HAM-1\_Larvae-L4-stage | 115 | 2.60 | 9.5e-24 |
| C01B12.2 | C01B12.2\_Larvae-L2-stage | 131 | 2.30 | 1.5e-23 |
| R02D3.7 | R02D3.7\_Larvae-L3-stage | 124 | 2.36 | 1.2e-22 |
| ham-1 | HAM-1\_Fed-L1-stage-larvae | 108 | 2.62 | 5.7e-22 |
| nhr-77 | NHR-77\_Larvae-L4-stage | 134 | 2.17 | 6.3e-22 |
| ceh-38 | CEH-38\_Larvae-L3-stage | 100 | 2.78 | 1.1e-21 |
| lsy-2 | LSY-2\_Fed-L1-stage-larvae | 115 | 2.45 | 1.6e-21 |
| gei-11 | GEI-11\_Larvae-L3-stage | 112 | 2.48 | 3.7e-21 |
| pha-4 | PHA-4\_Larvae-L2-stage | 122 | 2.31 | 3.9e-21 |
| eor-1 | EOR-1\_Larvae-L3-stage | 110 | 2.52 | 4.2e-21 |
| nfya-1 | NFYA-1\_Late-Embryos | 109 | 2.46 | 5.1e-20 |
| C34F6.9 | C34F6.9\_Larvae-L2-stage | 117 | 2.32 | 5.8e-20 |
| fos-1 | FOS-1\_Fed-L1-stage-larvae | 106 | 2.48 | 1.5e-19 |
| ceh-39 | CEH-39\_Embryos | 78 | 3.23 | 2.0e-19 |
| nhr-129 | NHR-129\_Larvae-L2-stage | 124 | 2.16 | 3.9e-19 |
| nhr-25 | NHR-25\_Larvae-L2-stage | 102 | 2.52 | 5.6e-19 |
| dpl-1 | DPL-1\_Young-adult | 102 | 2.45 | 4.0e-18 |
| lsy-2 | LSY-2\_Embryos | 87 | 2.77 | 6.8e-18 |
| pes-1 | PES-1\_Larvae-L4-stage | 109 | 2.30 | 9.4e-18 |
| nhr-237 | NHR-237\_Embryos | 64 | 3.62 | 1.0e-17 |
| lsy-2 | LSY-2\_Larvae-L1-stage | 113 | 2.23 | 1.2e-17 |
| F16B12.6 | F16B12.6\_Fed-L1-stage-larvae | 64 | 3.50 | 5.2e-17 |
| dve-1 | DVE-1\_Late-Embryos | 93 | 2.52 | 8.1e-17 |
| sax-3 | SAX-3\_Larvae-L4-stage | 107 | 2.25 | 1.2e-16 |
| nhr-77 | NHR-77\_Fed-L1-stage-larvae | 94 | 2.42 | 7.7e-16 |
| dve-1 | DVE-1\_Larvae-L4-stage | 76 | 2.84 | 1.0e-15 |
| C16A3.4 | C16A3.4\_Fed-L1-stage-larvae | 83 | 2.64 | 1.3e-15 |
| aly-2 | ALY-2\_Fed-L1-stage-larvae | 79 | 2.72 | 1.9e-15 |
| lin-15 | LIN-15B\_Fed-L1-stage-larvae | 69 | 3.00 | 3.9e-15 |
| nfya-1 | NFYA-1\_Larvae-L3-stage | 87 | 2.45 | 1.3e-14 |
| lin-13 | LIN-13\_Larvae-L2-stage | 79 | 2.61 | 2.1e-14 |
| pha-4 | PHA-4\_Larvae-L4-stage | 79 | 2.55 | 6.9e-14 |
| unc-62 | UNC-62\_Day-Four-Young-Adult | 69 | 2.82 | 8.7e-14 |
| unc-62 | UNC-62\_Young-adult-Day-4 | 69 | 2.82 | 8.7e-14 |
| gei-11 | GEI-11\_Larvae-L2-stage | 77 | 2.59 | 8.8e-14 |
| lsy-2 | LSY-2\_Larvae-L2-stage | 58 | 3.24 | 9.5e-14 |
| F45C12.2 | F45C12.2\_Fed-L1-stage-larvae | 84 | 2.41 | 1.4e-13 |
| lin-15 | LIN-15B\_Larvae-L4-stage | 41 | 4.45 | 1.5e-13 |
| nhr-6 | NHR-6\_Larvae-L4-stage | 71 | 2.71 | 1.8e-13 |
| F23B12.7 | F23B12.7\_Young-adult | 70 | 2.73 | 2.4e-13 |
| lin-13 | LIN-13\_Larvae-L4-stage | 64 | 2.79 | 2.1e-12 |
| elt-3 | ELT-3\_Embryos | 66 | 2.69 | 3.4e-12 |
| nhr-2 | NHR-2\_Embryos | 48 | 3.47 | 4.0e-12 |
| aly-2 | ALY-2\_Larvae-L3-stage | 55 | 3.05 | 6.7e-12 |
| fos-1 | FOS-1\_Larvae-L2-stage | 104 | 1.96 | 7.5e-12 |
| R02D3.7 | R02D3.7\_Larvae-L2-stage | 58 | 2.90 | 9.3e-12 |
| fos-1 | FOS-1\_Larvae-L4-stage | 48 | 3.34 | 1.5e-11 |
| ztf-7 | ZTF-7\_Larvae-L4-stage | 67 | 2.56 | 2.0e-11 |
| gei-11 | GEI-11\_Young-adult | 58 | 2.82 | 2.9e-11 |
| nhr-77 | NHR-77\_Larvae-L2-stage | 60 | 2.74 | 3.2e-11 |
| nhr-76 | NHR-76\_Larvae-L4-stage | 61 | 2.69 | 4.3e-11 |
| skn-1 | SKN-1\_Larvae-L3-stage | 52 | 2.98 | 8.5e-11 |
| ceh-38 | CEH-38\_Larvae-L4-stage | 59 | 2.71 | 9.1e-11 |
| hlh-30 | HLH-30\_Late-Embryos | 55 | 2.74 | 4.3e-10 |
| nhr-28 | NHR-28\_Larvae-L4-stage | 108 | 1.79 | 6.4e-10 |
| lin-13 | LIN-13\_Larvae-L1-stage | 37 | 3.71 | 6.7e-10 |
| nhr-77 | NHR-77\_Larvae-L3-stage | 66 | 2.38 | 7.6e-10 |
| nhr-237 | NHR-237\_Larvae-L1-stage | 36 | 3.63 | 2.2e-09 |
| sem-4 | SEM-4\_Larvae-L2-stage | 83 | 2.02 | 2.6e-09 |
| pha-4 | PHA-4\_Young-adult | 49 | 2.82 | 2.9e-09 |
| zag-1 | ZAG-1\_Fed-L1-stage-larvae | 47 | 2.87 | 4.4e-09 |
| ceh-26 | CEH-26\_Late-Embryonic-stage | 71 | 2.18 | 5.4e-09 |
| lin-35 | LIN-35\_Young-adult | 48 | 2.77 | 7.9e-09 |
| pax-1 | PAX-1\_Embryos | 35 | 3.53 | 8.5e-09 |
| jun-1 | JUN-1\_Larvae-L4-stage | 66 | 2.24 | 1.0e-08 |
| jun-1 | JUN-1\_Larvae-L3-stage | 57 | 2.40 | 2.2e-08 |
| nhr-6 | NHR-6\_Larvae-L2-stage | 83 | 1.92 | 3.0e-08 |
| aha-1 | AHA-1\_Larvae-L4-stage | 34 | 3.39 | 4.4e-08 |
| hlh-30 | HLH-30\_Larvae-L4-stage | 55 | 2.34 | 1.1e-07 |
| fos-1 | FOS-1\_Larvae-L3-stage | 62 | 2.18 | 1.2e-07 |
| zag-1 | ZAG-1\_Larvae-L4-stage | 57 | 2.28 | 1.4e-07 |
| mab-5 | MAB-5\_Larvae-L2-stage | 43 | 2.72 | 1.4e-07 |
| nhr-11 | NHR-11\_Larvae-L2-stage | 46 | 2.59 | 1.5e-07 |
| sax-3 | SAX-3\_Larvae-L2-stage | 67 | 2.07 | 1.7e-07 |
| lin-35 | LIN-35\_Starved-L1-stage-larvae | 44 | 2.65 | 1.8e-07 |
| ceh-16 | CEH-16\_Larvae-L2-stage | 41 | 2.78 | 2.0e-07 |
| R02D3.7 | R02D3.7\_Larvae-L4-stage | 44 | 2.64 | 2.1e-07 |
| nfya-1 | NFYA-1\_Young-adult | 32 | 3.23 | 4.2e-07 |
| ztf-4 | ZTF-4\_Larvae-L3-stage | 26 | 3.71 | 8.2e-07 |
| lsy-2 | LSY-2\_Larvae-L4-stage | 42 | 2.55 | 1.3e-06 |
| zag-1 | ZAG-1\_Larvae-L3-stage | 41 | 2.53 | 2.3e-06 |
| ztf-4 | ZTF-4\_Larvae-L2-stage | 36 | 2.73 | 2.9e-06 |
| zag-1 | ZAG-1\_Larvae-L2-stage | 59 | 2.03 | 4.0e-06 |
| elt-1 | ELT-1\_Larvae-L3-stage | 41 | 2.43 | 6.9e-06 |
| nhr-76 | NHR-76\_Larvae-L3-stage | 44 | 2.32 | 7.9e-06 |
| alr-1 | ALR-1\_Larvae-L2-stage | 73 | 1.78 | 1.1e-05 |
| egl-5 | EGL-5\_Larvae-L3-stage | 56 | 2.01 | 1.2e-05 |
| jun-1 | JUN-1\_Larvae-L1-stage | 67 | 1.85 | 1.2e-05 |
| ces-1 | CES-1\_Embryos | 74 | 1.76 | 1.4e-05 |
| gei-11 | GEI-11\_Embryos | 33 | 2.69 | 1.5e-05 |
| nhr-237 | NHR-237\_Larvae-L2-stage | 14 | 5.50 | 2.1e-05 |
| ama-1 | AMA-1\_Larvae-L3-stage | 26 | 3.07 | 2.7e-05 |
| nhr-10 | NHR-10\_Larvae-L4-stage | 24 | 2.99 | 1.1e-04 |
| unc-62 | UNC-62\_Fed-L1-stage-larvae | 26 | 2.74 | 1.9e-04 |
| F45C12.2 | F45C12.2\_Larvae-L2-stage | 29 | 2.52 | 2.6e-04 |
| ztf-4 | ZTF-4\_Larvae-L1-stage | 20 | 3.18 | 3.2e-04 |
| sax-3 | SAX-3\_Larvae-L3-stage | 37 | 2.13 | 4.8e-04 |
| nhr-28 | NHR-28\_Larvae-L3-stage | 27 | 2.53 | 5.0e-04 |
| F45C12.2 | F45C12.2\_Larvae-L3-stage | 37 | 2.12 | 5.2e-04 |
| zip-2 | ZIP-2\_Larvae-L4-stage | 20 | 3.02 | 6.2e-04 |
| sax-3 | SAX-3\_Fed-L1-stage-larvae | 28 | 2.40 | 8.4e-04 |
| aha-1 | AHA-1\_Fed-L1-stage-larvae | 16 | 3.46 | 9.2e-04 |
| fkh-2 | FKH-2\_Larvae-L3-stage | 27 | 2.38 | 1.3e-03 |
| unc-62 | UNC-62\_Larvae-L3-stage | 36 | 2.04 | 1.6e-03 |
| nhr-21 | NHR-21\_Larvae-L2-stage | 24 | 2.48 | 2.0e-03 |
| unc-39 | UNC-39\_Embryos | 23 | 2.52 | 2.1e-03 |
| sea-2 | SEA-2\_Larvae-L3-stage | 23 | 2.51 | 2.4e-03 |
| mef-2 | MEF-2\_Fed-L1-stage-larvae | 21 | 2.60 | 2.9e-03 |
| daf-12 | DAF-12\_Larvae-L3-stage | 16 | 3.11 | 3.0e-03 |
| ztf-11 | ZTF-11\_Embryos | 17 | 2.94 | 3.4e-03 |
| unc-62 | UNC-62\_Larvae-L2-stage | 26 | 2.24 | 4.5e-03 |
| ces-1 | CES-1\_Larvae-L3-stage | 29 | 2.07 | 6.6e-03 |
| ces-1 | CES-1\_Fed-L1-stage-larvae | 29 | 2.02 | 9.7e-03 |
| elt-1 | ELT-1\_Larvae-L2-stage | 10 | 3.93 | 1.0e-02 |
| aly-2 | ALY-2\_Larvae-L2-stage | 24 | 2.15 | 1.3e-02 |
| peb-1 | PEB-1\_Larvae-L2-stage | 20 | 2.36 | 1.3e-02 |
| med-1 | MED-1\_Embryos | 17 | 2.55 | 1.5e-02 |
| nhr-116 | NHR-116\_Larvae-L2-stage | 7 | 4.40 | 3.7e-02 |
| mab-5 | MAB-5\_Embryos | 16 | 2.39 | 3.9e-02 |
| ztf-11 | ZTF-11\_Larvae-L3-stage | 10 | 3.10 | 5.0e-02 |
