# Supplementary material for: Overlapping cell population expression profiling and regulatory inference in C. elegans
Source: BMC Genomics. 2016 Feb 29;17:159. doi: 10.1186/s12864-016-2482-z (PMC4772325; doi:10.1186/s12864-016-2482-z)
Supplement: Additional file 13: — Web supplement. (DOC 21 kb) [file 12864_2016_2482_MOESM13_ESM.zip › sortWeb/clusters/hier.300.clusters/236.html]

Cluster 236 

## Cluster 236

### Expression

| cnd-1 rep. 1 | cnd-1 rep. 2 | cnd-1 rep. 3 | pha-4 rep. 1 | pha-4 rep. 2 | pha-4 rep. 3 | ceh-27 | ceh-36 | ceh-6 | F21D5.9 | mir-57 | mls-2 | pal-1 | pros-1 | ttx-3 | unc-130 | hlh-16 | irx-1 | ceh-6 (+) hlh-16 (+) | ceh-6 (+) hlh-16 (-) | ceh-6 (-) hlh-16 (+) | cnd-1 singlets | pha-4 singlets | 0 | 60 | 120 | 150 | 180 | 240 | 330 | 390 | 420 | 480 | 540 | 570 | 600 | 630 | 660 | NAME | Functional description |
| --- | --- | --- | --- | --- | --- | --- | --- | --- | --- | --- | --- | --- | --- | --- | --- | --- | --- | --- | --- | --- | --- | --- | --- | --- | --- | --- | --- | --- | --- | --- | --- | --- | --- | --- | --- | --- | --- | --- | --- |
|  |  |  |  |  |  |  |  |  |  |  |  |  |  |  |  |  |  |  |  |  |  |  |  |  |  |  |  |  |  |  |  |  |  |  |  |  |  | *ceh-27* | C. Elegans Homeobox |
|  |  |  |  |  |  |  |  |  |  |  |  |  |  |  |  |  |  |  |  |  |  |  |  |  |  |  |  |  |  |  |  |  |  |  |  |  |  | ZC8.6 |  |
|  |  |  |  |  |  |  |  |  |  |  |  |  |  |  |  |  |  |  |  |  |  |  |  |  |  |  |  |  |  |  |  |  |  |  |  |  |  | *irx-1* | IRoquois subclass of homeoboX |
|  |  |  |  |  |  |  |  |  |  |  |  |  |  |  |  |  |  |  |  |  |  |  |  |  |  |  |  |  |  |  |  |  |  |  |  |  |  | *ncam-1* | NCAM (neural cell adhesion molecule) homolog |
|  |  |  |  |  |  |  |  |  |  |  |  |  |  |  |  |  |  |  |  |  |  |  |  |  |  |  |  |  |  |  |  |  |  |  |  |  |  | *nra-2* | Nicotinic Receptor Associated |
|  |  |  |  |  |  |  |  |  |  |  |  |  |  |  |  |  |  |  |  |  |  |  |  |  |  |  |  |  |  |  |  |  |  |  |  |  |  | *gei-12* | GEX Interacting protein |
|  |  |  |  |  |  |  |  |  |  |  |  |  |  |  |  |  |  |  |  |  |  |  |  |  |  |  |  |  |  |  |  |  |  |  |  |  |  | Y34D9A.7 |  |
|  |  |  |  |  |  |  |  |  |  |  |  |  |  |  |  |  |  |  |  |  |  |  |  |  |  |  |  |  |  |  |  |  |  |  |  |  |  | *hmt-1* | Heavy Metal Tolerance factor |
|  |  |  |  |  |  |  |  |  |  |  |  |  |  |  |  |  |  |  |  |  |  |  |  |  |  |  |  |  |  |  |  |  |  |  |  |  |  | *btf-1* | BTAF (TBP-associated factor) homolog |
|  |  |  |  |  |  |  |  |  |  |  |  |  |  |  |  |  |  |  |  |  |  |  |  |  |  |  |  |  |  |  |  |  |  |  |  |  |  | *snrp-200* | Small Nuclear RibonucleoProtein homolog |
|  |  |  |  |  |  |  |  |  |  |  |  |  |  |  |  |  |  |  |  |  |  |  |  |  |  |  |  |  |  |  |  |  |  |  |  |  |  | Y67D2.6 |  |
|  |  |  |  |  |  |  |  |  |  |  |  |  |  |  |  |  |  |  |  |  |  |  |  |  |  |  |  |  |  |  |  |  |  |  |  |  |  | *nol-6* | NucleOLar protein |
|  |  |  |  |  |  |  |  |  |  |  |  |  |  |  |  |  |  |  |  |  |  |  |  |  |  |  |  |  |  |  |  |  |  |  |  |  |  | Y54E2A.4 |  |
|  |  |  |  |  |  |  |  |  |  |  |  |  |  |  |  |  |  |  |  |  |  |  |  |  |  |  |  |  |  |  |  |  |  |  |  |  |  | Y54E5A.6 |  |
|  |  |  |  |  |  |  |  |  |  |  |  |  |  |  |  |  |  |  |  |  |  |  |  |  |  |  |  |  |  |  |  |  |  |  |  |  |  | *mel-46* | Maternal Effect Lethal |
|  |  |  |  |  |  |  |  |  |  |  |  |  |  |  |  |  |  |  |  |  |  |  |  |  |  |  |  |  |  |  |  |  |  |  |  |  |  | *xpo-2* | eXPOrtin (nuclear export receptor) |
|  |  |  |  |  |  |  |  |  |  |  |  |  |  |  |  |  |  |  |  |  |  |  |  |  |  |  |  |  |  |  |  |  |  |  |  |  |  | *cdc-48.2* | Cell Division Cycle related |
|  |  |  |  |  |  |  |  |  |  |  |  |  |  |  |  |  |  |  |  |  |  |  |  |  |  |  |  |  |  |  |  |  |  |  |  |  |  | *mcd-1* | Modifier of Cell Death phenotype |
|  |  |  |  |  |  |  |  |  |  |  |  |  |  |  |  |  |  |  |  |  |  |  |  |  |  |  |  |  |  |  |  |  |  |  |  |  |  | *ama-1* | AMAnitin resistant |
|  |  |  |  |  |  |  |  |  |  |  |  |  |  |  |  |  |  |  |  |  |  |  |  |  |  |  |  |  |  |  |  |  |  |  |  |  |  | Y116F11B.10 |  |
|  |  |  |  |  |  |  |  |  |  |  |  |  |  |  |  |  |  |  |  |  |  |  |  |  |  |  |  |  |  |  |  |  |  |  |  |  |  | *sop-2* | Suppressor of Pal-1 |
|  |  |  |  |  |  |  |  |  |  |  |  |  |  |  |  |  |  |  |  |  |  |  |  |  |  |  |  |  |  |  |  |  |  |  |  |  |  | Y48E1B.2 |  |
|  |  |  |  |  |  |  |  |  |  |  |  |  |  |  |  |  |  |  |  |  |  |  |  |  |  |  |  |  |  |  |  |  |  |  |  |  |  | *rpn-2* | proteasome Regulatory Particle, Non-ATPase-like |
|  |  |  |  |  |  |  |  |  |  |  |  |  |  |  |  |  |  |  |  |  |  |  |  |  |  |  |  |  |  |  |  |  |  |  |  |  |  | *sop-3* | Suppressor of Pal-1 |
|  |  |  |  |  |  |  |  |  |  |  |  |  |  |  |  |  |  |  |  |  |  |  |  |  |  |  |  |  |  |  |  |  |  |  |  |  |  | *prp-21* | yeast PRP (splicing factor) related |
|  |  |  |  |  |  |  |  |  |  |  |  |  |  |  |  |  |  |  |  |  |  |  |  |  |  |  |  |  |  |  |  |  |  |  |  |  |  | *patr-1* | yeast PAT1(protein associated with topo II) Related |
|  |  |  |  |  |  |  |  |  |  |  |  |  |  |  |  |  |  |  |  |  |  |  |  |  |  |  |  |  |  |  |  |  |  |  |  |  |  | *pro-1* | PROximal proliferation in germline |
|  |  |  |  |  |  |  |  |  |  |  |  |  |  |  |  |  |  |  |  |  |  |  |  |  |  |  |  |  |  |  |  |  |  |  |  |  |  | *riok-2* | RIO Kinase homolog |
|  |  |  |  |  |  |  |  |  |  |  |  |  |  |  |  |  |  |  |  |  |  |  |  |  |  |  |  |  |  |  |  |  |  |  |  |  |  | *rbm-28* | RNA Binding Motif protein homolog |
|  |  |  |  |  |  |  |  |  |  |  |  |  |  |  |  |  |  |  |  |  |  |  |  |  |  |  |  |  |  |  |  |  |  |  |  |  |  | Y48C3A.14 |  |
|  |  |  |  |  |  |  |  |  |  |  |  |  |  |  |  |  |  |  |  |  |  |  |  |  |  |  |  |  |  |  |  |  |  |  |  |  |  | *gex-2* | Gut on EXterior |
|  |  |  |  |  |  |  |  |  |  |  |  |  |  |  |  |  |  |  |  |  |  |  |  |  |  |  |  |  |  |  |  |  |  |  |  |  |  | ZK550.4 |  |
|  |  |  |  |  |  |  |  |  |  |  |  |  |  |  |  |  |  |  |  |  |  |  |  |  |  |  |  |  |  |  |  |  |  |  |  |  |  | *ddx-17* | DEAD boX helicase homolog |
|  |  |  |  |  |  |  |  |  |  |  |  |  |  |  |  |  |  |  |  |  |  |  |  |  |  |  |  |  |  |  |  |  |  |  |  |  |  | C36E6.1 |  |
|  |  |  |  |  |  |  |  |  |  |  |  |  |  |  |  |  |  |  |  |  |  |  |  |  |  |  |  |  |  |  |  |  |  |  |  |  |  | *dnj-29* | DNaJ domain (prokaryotic heat shock protein) |
|  |  |  |  |  |  |  |  |  |  |  |  |  |  |  |  |  |  |  |  |  |  |  |  |  |  |  |  |  |  |  |  |  |  |  |  |  |  | *tag-53* | Temporarily Assigned Gene name |
|  |  |  |  |  |  |  |  |  |  |  |  |  |  |  |  |  |  |  |  |  |  |  |  |  |  |  |  |  |  |  |  |  |  |  |  |  |  | *herc-1* | HECT and RCC domain E3 ubiquitin ligase |
|  |  |  |  |  |  |  |  |  |  |  |  |  |  |  |  |  |  |  |  |  |  |  |  |  |  |  |  |  |  |  |  |  |  |  |  |  |  | *acl-6* | ACyLtransferase-like |
|  |  |  |  |  |  |  |  |  |  |  |  |  |  |  |  |  |  |  |  |  |  |  |  |  |  |  |  |  |  |  |  |  |  |  |  |  |  | *gcn-2* | GCN (yeast General Control Nondrepressible) homolog |
|  |  |  |  |  |  |  |  |  |  |  |  |  |  |  |  |  |  |  |  |  |  |  |  |  |  |  |  |  |  |  |  |  |  |  |  |  |  | *pix-1* |  |
|  |  |  |  |  |  |  |  |  |  |  |  |  |  |  |  |  |  |  |  |  |  |  |  |  |  |  |  |  |  |  |  |  |  |  |  |  |  | *anr-40* | Antisense Non-coding RNA |
|  |  |  |  |  |  |  |  |  |  |  |  |  |  |  |  |  |  |  |  |  |  |  |  |  |  |  |  |  |  |  |  |  |  |  |  |  |  | *mdt-17* | MeDiaTor |
|  |  |  |  |  |  |  |  |  |  |  |  |  |  |  |  |  |  |  |  |  |  |  |  |  |  |  |  |  |  |  |  |  |  |  |  |  |  | *magu-1* | MAGUK family |
|  |  |  |  |  |  |  |  |  |  |  |  |  |  |  |  |  |  |  |  |  |  |  |  |  |  |  |  |  |  |  |  |  |  |  |  |  |  | *eif-3.E* | Eukaryotic Initiation Factor |
|  |  |  |  |  |  |  |  |  |  |  |  |  |  |  |  |  |  |  |  |  |  |  |  |  |  |  |  |  |  |  |  |  |  |  |  |  |  | *npp-13* | Nuclear Pore complex Protein |
|  |  |  |  |  |  |  |  |  |  |  |  |  |  |  |  |  |  |  |  |  |  |  |  |  |  |  |  |  |  |  |  |  |  |  |  |  |  | C17H12.2 |  |
|  |  |  |  |  |  |  |  |  |  |  |  |  |  |  |  |  |  |  |  |  |  |  |  |  |  |  |  |  |  |  |  |  |  |  |  |  |  | *fcho-1* | FCH domain Only (FCH stands for Fes/CIP4 homology domain) |
|  |  |  |  |  |  |  |  |  |  |  |  |  |  |  |  |  |  |  |  |  |  |  |  |  |  |  |  |  |  |  |  |  |  |  |  |  |  | *pam-1* | Puromycin-sensitive AMinopeptidase |
|  |  |  |  |  |  |  |  |  |  |  |  |  |  |  |  |  |  |  |  |  |  |  |  |  |  |  |  |  |  |  |  |  |  |  |  |  |  | *set-29* | SET (trithorax/polycomb) domain containing |
|  |  |  |  |  |  |  |  |  |  |  |  |  |  |  |  |  |  |  |  |  |  |  |  |  |  |  |  |  |  |  |  |  |  |  |  |  |  | Y48A6C.4 |  |
|  |  |  |  |  |  |  |  |  |  |  |  |  |  |  |  |  |  |  |  |  |  |  |  |  |  |  |  |  |  |  |  |  |  |  |  |  |  | Y39B6A.43 |  |
|  |  |  |  |  |  |  |  |  |  |  |  |  |  |  |  |  |  |  |  |  |  |  |  |  |  |  |  |  |  |  |  |  |  |  |  |  |  | Y87G2A.1 |  |
|  |  |  |  |  |  |  |  |  |  |  |  |  |  |  |  |  |  |  |  |  |  |  |  |  |  |  |  |  |  |  |  |  |  |  |  |  |  | F31C3.2 |  |
|  |  |  |  |  |  |  |  |  |  |  |  |  |  |  |  |  |  |  |  |  |  |  |  |  |  |  |  |  |  |  |  |  |  |  |  |  |  | C05G5.2 |  |
|  |  |  |  |  |  |  |  |  |  |  |  |  |  |  |  |  |  |  |  |  |  |  |  |  |  |  |  |  |  |  |  |  |  |  |  |  |  | Y92H12A.5 |  |
|  |  |  |  |  |  |  |  |  |  |  |  |  |  |  |  |  |  |  |  |  |  |  |  |  |  |  |  |  |  |  |  |  |  |  |  |  |  | Y75B8A.25 |  |
|  |  |  |  |  |  |  |  |  |  |  |  |  |  |  |  |  |  |  |  |  |  |  |  |  |  |  |  |  |  |  |  |  |  |  |  |  |  | Y50D4A.4 |  |
|  |  |  |  |  |  |  |  |  |  |  |  |  |  |  |  |  |  |  |  |  |  |  |  |  |  |  |  |  |  |  |  |  |  |  |  |  |  | *emc-1* | EMC Endoplasmic Membrane protein Complex (yeast EMC) homolog |
|  |  |  |  |  |  |  |  |  |  |  |  |  |  |  |  |  |  |  |  |  |  |  |  |  |  |  |  |  |  |  |  |  |  |  |  |  |  | *cls-2* | CLASP family of microtubule-binding proteins |
|  |  |  |  |  |  |  |  |  |  |  |  |  |  |  |  |  |  |  |  |  |  |  |  |  |  |  |  |  |  |  |  |  |  |  |  |  |  | *hpo-13* | Hypersensitive to POre-forming toxin |
|  |  |  |  |  |  |  |  |  |  |  |  |  |  |  |  |  |  |  |  |  |  |  |  |  |  |  |  |  |  |  |  |  |  |  |  |  |  | *dpy-21* | DumPY: shorter than wild-type |
|  |  |  |  |  |  |  |  |  |  |  |  |  |  |  |  |  |  |  |  |  |  |  |  |  |  |  |  |  |  |  |  |  |  |  |  |  |  | Y48G8AL.10 |  |
|  |  |  |  |  |  |  |  |  |  |  |  |  |  |  |  |  |  |  |  |  |  |  |  |  |  |  |  |  |  |  |  |  |  |  |  |  |  | *tbc-15* | TBC (Tre-2/Bub2/Cdc16) domain family |
|  |  |  |  |  |  |  |  |  |  |  |  |  |  |  |  |  |  |  |  |  |  |  |  |  |  |  |  |  |  |  |  |  |  |  |  |  |  | BE0003N10.1 |  |
|  |  |  |  |  |  |  |  |  |  |  |  |  |  |  |  |  |  |  |  |  |  |  |  |  |  |  |  |  |  |  |  |  |  |  |  |  |  | *inso-1* | INSOmniac (Drosophila sleep affecting) homolog |
|  |  |  |  |  |  |  |  |  |  |  |  |  |  |  |  |  |  |  |  |  |  |  |  |  |  |  |  |  |  |  |  |  |  |  |  |  |  | C02B8.6 |  |
|  |  |  |  |  |  |  |  |  |  |  |  |  |  |  |  |  |  |  |  |  |  |  |  |  |  |  |  |  |  |  |  |  |  |  |  |  |  | F53F8.5 |  |
|  |  |  |  |  |  |  |  |  |  |  |  |  |  |  |  |  |  |  |  |  |  |  |  |  |  |  |  |  |  |  |  |  |  |  |  |  |  | *pab-2* | PolyA Binding protein |
|  |  |  |  |  |  |  |  |  |  |  |  |  |  |  |  |  |  |  |  |  |  |  |  |  |  |  |  |  |  |  |  |  |  |  |  |  |  | *ztf-6* | Zinc finger putative Transcription Factor family |
|  |  |  |  |  |  |  |  |  |  |  |  |  |  |  |  |  |  |  |  |  |  |  |  |  |  |  |  |  |  |  |  |  |  |  |  |  |  | Y17G7B.21 |  |
|  |  |  |  |  |  |  |  |  |  |  |  |  |  |  |  |  |  |  |  |  |  |  |  |  |  |  |  |  |  |  |  |  |  |  |  |  |  | Y53F4B.21 |  |
|  |  |  |  |  |  |  |  |  |  |  |  |  |  |  |  |  |  |  |  |  |  |  |  |  |  |  |  |  |  |  |  |  |  |  |  |  |  | Y48G8AL.5 |  |
|  |  |  |  |  |  |  |  |  |  |  |  |  |  |  |  |  |  |  |  |  |  |  |  |  |  |  |  |  |  |  |  |  |  |  |  |  |  | M01F1.9 |  |
|  |  |  |  |  |  |  |  |  |  |  |  |  |  |  |  |  |  |  |  |  |  |  |  |  |  |  |  |  |  |  |  |  |  |  |  |  |  | F53C3.13 |  |
|  |  |  |  |  |  |  |  |  |  |  |  |  |  |  |  |  |  |  |  |  |  |  |  |  |  |  |  |  |  |  |  |  |  |  |  |  |  | Y53G8AR.6 |  |
|  |  |  |  |  |  |  |  |  |  |  |  |  |  |  |  |  |  |  |  |  |  |  |  |  |  |  |  |  |  |  |  |  |  |  |  |  |  | *slr-2* | Synthetic with Lin-35/Rb |
|  |  |  |  |  |  |  |  |  |  |  |  |  |  |  |  |  |  |  |  |  |  |  |  |  |  |  |  |  |  |  |  |  |  |  |  |  |  | *eri-5* | Enhanced RNAI (RNA interference) |
|  |  |  |  |  |  |  |  |  |  |  |  |  |  |  |  |  |  |  |  |  |  |  |  |  |  |  |  |  |  |  |  |  |  |  |  |  |  | C50D2.10 |  |
|  |  |  |  |  |  |  |  |  |  |  |  |  |  |  |  |  |  |  |  |  |  |  |  |  |  |  |  |  |  |  |  |  |  |  |  |  |  | *hrp-1* | human HnRNP A1 homolog |
|  |  |  |  |  |  |  |  |  |  |  |  |  |  |  |  |  |  |  |  |  |  |  |  |  |  |  |  |  |  |  |  |  |  |  |  |  |  | Y92H12BR.3 |  |
|  |  |  |  |  |  |  |  |  |  |  |  |  |  |  |  |  |  |  |  |  |  |  |  |  |  |  |  |  |  |  |  |  |  |  |  |  |  | *uaf-2* | U2AF splicing factor |
|  |  |  |  |  |  |  |  |  |  |  |  |  |  |  |  |  |  |  |  |  |  |  |  |  |  |  |  |  |  |  |  |  |  |  |  |  |  | Y40B1A.3 |  |
|  |  |  |  |  |  |  |  |  |  |  |  |  |  |  |  |  |  |  |  |  |  |  |  |  |  |  |  |  |  |  |  |  |  |  |  |  |  | *taf-6.2* | TAF (TBP-associated transcription factor) family |
|  |  |  |  |  |  |  |  |  |  |  |  |  |  |  |  |  |  |  |  |  |  |  |  |  |  |  |  |  |  |  |  |  |  |  |  |  |  | Y76B12C.6 |  |
|  |  |  |  |  |  |  |  |  |  |  |  |  |  |  |  |  |  |  |  |  |  |  |  |  |  |  |  |  |  |  |  |  |  |  |  |  |  | Y53F4B.13 |  |
|  |  |  |  |  |  |  |  |  |  |  |  |  |  |  |  |  |  |  |  |  |  |  |  |  |  |  |  |  |  |  |  |  |  |  |  |  |  | *rbc-2* | RaBConnectin related |
|  |  |  |  |  |  |  |  |  |  |  |  |  |  |  |  |  |  |  |  |  |  |  |  |  |  |  |  |  |  |  |  |  |  |  |  |  |  | Y71H2AM.2 |  |
|  |  |  |  |  |  |  |  |  |  |  |  |  |  |  |  |  |  |  |  |  |  |  |  |  |  |  |  |  |  |  |  |  |  |  |  |  |  | Y49E10.21 |  |
|  |  |  |  |  |  |  |  |  |  |  |  |  |  |  |  |  |  |  |  |  |  |  |  |  |  |  |  |  |  |  |  |  |  |  |  |  |  | *fbxc-19* | F-box C protein |
|  |  |  |  |  |  |  |  |  |  |  |  |  |  |  |  |  |  |  |  |  |  |  |  |  |  |  |  |  |  |  |  |  |  |  |  |  |  | Y75B8A.19 |  |
|  |  |  |  |  |  |  |  |  |  |  |  |  |  |  |  |  |  |  |  |  |  |  |  |  |  |  |  |  |  |  |  |  |  |  |  |  |  | ZK632.11 |  |
|  |  |  |  |  |  |  |  |  |  |  |  |  |  |  |  |  |  |  |  |  |  |  |  |  |  |  |  |  |  |  |  |  |  |  |  |  |  | Y71H2AM.1 |  |
|  |  |  |  |  |  |  |  |  |  |  |  |  |  |  |  |  |  |  |  |  |  |  |  |  |  |  |  |  |  |  |  |  |  |  |  |  |  | H34C03.2 |  |
|  |  |  |  |  |  |  |  |  |  |  |  |  |  |  |  |  |  |  |  |  |  |  |  |  |  |  |  |  |  |  |  |  |  |  |  |  |  | Y48G1C.11 |  |
|  |  |  |  |  |  |  |  |  |  |  |  |  |  |  |  |  |  |  |  |  |  |  |  |  |  |  |  |  |  |  |  |  |  |  |  |  |  | *epc-1* | Enhancer of PolyComb-like |
|  |  |  |  |  |  |  |  |  |  |  |  |  |  |  |  |  |  |  |  |  |  |  |  |  |  |  |  |  |  |  |  |  |  |  |  |  |  | *trxr-1* | ThioRedoXin Reductase |
|  |  |  |  |  |  |  |  |  |  |  |  |  |  |  |  |  |  |  |  |  |  |  |  |  |  |  |  |  |  |  |  |  |  |  |  |  |  | F54D11.2 |  |
|  |  |  |  |  |  |  |  |  |  |  |  |  |  |  |  |  |  |  |  |  |  |  |  |  |  |  |  |  |  |  |  |  |  |  |  |  |  | C15C6.3 |  |
|  |  |  |  |  |  |  |  |  |  |  |  |  |  |  |  |  |  |  |  |  |  |  |  |  |  |  |  |  |  |  |  |  |  |  |  |  |  | Y41E3.11 |  |
|  |  |  |  |  |  |  |  |  |  |  |  |  |  |  |  |  |  |  |  |  |  |  |  |  |  |  |  |  |  |  |  |  |  |  |  |  |  | Y48C3A.12 |  |
|  |  |  |  |  |  |  |  |  |  |  |  |  |  |  |  |  |  |  |  |  |  |  |  |  |  |  |  |  |  |  |  |  |  |  |  |  |  | *athp-1* | AT Hook plus PHD finger transcription factor |
|  |  |  |  |  |  |  |  |  |  |  |  |  |  |  |  |  |  |  |  |  |  |  |  |  |  |  |  |  |  |  |  |  |  |  |  |  |  | *cnt-2* | CeNTaurin |
|  |  |  |  |  |  |  |  |  |  |  |  |  |  |  |  |  |  |  |  |  |  |  |  |  |  |  |  |  |  |  |  |  |  |  |  |  |  | *rpn-1* | proteasome Regulatory Particle, Non-ATPase-like |
|  |  |  |  |  |  |  |  |  |  |  |  |  |  |  |  |  |  |  |  |  |  |  |  |  |  |  |  |  |  |  |  |  |  |  |  |  |  | *prp-6* | yeast PRP (splicing factor) related |
|  |  |  |  |  |  |  |  |  |  |  |  |  |  |  |  |  |  |  |  |  |  |  |  |  |  |  |  |  |  |  |  |  |  |  |  |  |  | Y75B8A.8 |  |
|  |  |  |  |  |  |  |  |  |  |  |  |  |  |  |  |  |  |  |  |  |  |  |  |  |  |  |  |  |  |  |  |  |  |  |  |  |  | *rsp-8* | SR Protein (splicing factor) |
|  |  |  |  |  |  |  |  |  |  |  |  |  |  |  |  |  |  |  |  |  |  |  |  |  |  |  |  |  |  |  |  |  |  |  |  |  |  | *taf-2* | TAF (TBP-associated transcription factor) family |
|  |  |  |  |  |  |  |  |  |  |  |  |  |  |  |  |  |  |  |  |  |  |  |  |  |  |  |  |  |  |  |  |  |  |  |  |  |  | *vps-4* | related to yeast Vacuolar Protein Sorting factor |
|  |  |  |  |  |  |  |  |  |  |  |  |  |  |  |  |  |  |  |  |  |  |  |  |  |  |  |  |  |  |  |  |  |  |  |  |  |  | Y104H12D.3 |  |
|  |  |  |  |  |  |  |  |  |  |  |  |  |  |  |  |  |  |  |  |  |  |  |  |  |  |  |  |  |  |  |  |  |  |  |  |  |  | *fem-2* | FEMinization of XX and XO animals |
|  |  |  |  |  |  |  |  |  |  |  |  |  |  |  |  |  |  |  |  |  |  |  |  |  |  |  |  |  |  |  |  |  |  |  |  |  |  | *nuo-1* | NADH Ubiquinone Oxidoreductase |
|  |  |  |  |  |  |  |  |  |  |  |  |  |  |  |  |  |  |  |  |  |  |  |  |  |  |  |  |  |  |  |  |  |  |  |  |  |  | Y37E11B.5 |  |
|  |  |  |  |  |  |  |  |  |  |  |  |  |  |  |  |  |  |  |  |  |  |  |  |  |  |  |  |  |  |  |  |  |  |  |  |  |  | *jmjd-2* | JuMonJi (transcription factor) Domain protein |
|  |  |  |  |  |  |  |  |  |  |  |  |  |  |  |  |  |  |  |  |  |  |  |  |  |  |  |  |  |  |  |  |  |  |  |  |  |  | D2045.2 |  |
|  |  |  |  |  |  |  |  |  |  |  |  |  |  |  |  |  |  |  |  |  |  |  |  |  |  |  |  |  |  |  |  |  |  |  |  |  |  | F33E11.3 |  |
|  |  |  |  |  |  |  |  |  |  |  |  |  |  |  |  |  |  |  |  |  |  |  |  |  |  |  |  |  |  |  |  |  |  |  |  |  |  | *larp-5* | LARP (RNA binding La related protein) homolog |
|  |  |  |  |  |  |  |  |  |  |  |  |  |  |  |  |  |  |  |  |  |  |  |  |  |  |  |  |  |  |  |  |  |  |  |  |  |  | *png-1* | PNG (Peptide:N-Glycanase) homolog |
|  |  |  |  |  |  |  |  |  |  |  |  |  |  |  |  |  |  |  |  |  |  |  |  |  |  |  |  |  |  |  |  |  |  |  |  |  |  | *chin-1* | CHImaeriN (Rac-GTPase-activating protein) homolo |
|  |  |  |  |  |  |  |  |  |  |  |  |  |  |  |  |  |  |  |  |  |  |  |  |  |  |  |  |  |  |  |  |  |  |  |  |  |  | *arp-1* | Actin-Related Proteins |
|  |  |  |  |  |  |  |  |  |  |  |  |  |  |  |  |  |  |  |  |  |  |  |  |  |  |  |  |  |  |  |  |  |  |  |  |  |  | F32A11.1 |  |
|  |  |  |  |  |  |  |  |  |  |  |  |  |  |  |  |  |  |  |  |  |  |  |  |  |  |  |  |  |  |  |  |  |  |  |  |  |  | F26F4.5 |  |
|  |  |  |  |  |  |  |  |  |  |  |  |  |  |  |  |  |  |  |  |  |  |  |  |  |  |  |  |  |  |  |  |  |  |  |  |  |  | *pph-4.2* | Protein PHosphatase |
|  |  |  |  |  |  |  |  |  |  |  |  |  |  |  |  |  |  |  |  |  |  |  |  |  |  |  |  |  |  |  |  |  |  |  |  |  |  | Y39A3CL.1 |  |
|  |  |  |  |  |  |  |  |  |  |  |  |  |  |  |  |  |  |  |  |  |  |  |  |  |  |  |  |  |  |  |  |  |  |  |  |  |  | Y71H2B.5 |  |
|  |  |  |  |  |  |  |  |  |  |  |  |  |  |  |  |  |  |  |  |  |  |  |  |  |  |  |  |  |  |  |  |  |  |  |  |  |  | *pars-2* | Prolyl Amino-acyl tRNA Synthetase |
|  |  |  |  |  |  |  |  |  |  |  |  |  |  |  |  |  |  |  |  |  |  |  |  |  |  |  |  |  |  |  |  |  |  |  |  |  |  | T09F3.2 |  |
|  |  |  |  |  |  |  |  |  |  |  |  |  |  |  |  |  |  |  |  |  |  |  |  |  |  |  |  |  |  |  |  |  |  |  |  |  |  | *alg-2* | Argonaute (plant)-Like Gene |
|  |  |  |  |  |  |  |  |  |  |  |  |  |  |  |  |  |  |  |  |  |  |  |  |  |  |  |  |  |  |  |  |  |  |  |  |  |  | *spr-5* | Suppressor of PResenilin defect |
|  |  |  |  |  |  |  |  |  |  |  |  |  |  |  |  |  |  |  |  |  |  |  |  |  |  |  |  |  |  |  |  |  |  |  |  |  |  | *cul-3* | CULlin |
|  |  |  |  |  |  |  |  |  |  |  |  |  |  |  |  |  |  |  |  |  |  |  |  |  |  |  |  |  |  |  |  |  |  |  |  |  |  | F56B3.4 |  |
|  |  |  |  |  |  |  |  |  |  |  |  |  |  |  |  |  |  |  |  |  |  |  |  |  |  |  |  |  |  |  |  |  |  |  |  |  |  | *tag-51* | Temporarily Assigned Gene name |
|  |  |  |  |  |  |  |  |  |  |  |  |  |  |  |  |  |  |  |  |  |  |  |  |  |  |  |  |  |  |  |  |  |  |  |  |  |  | *attf-2* | AT hook Transcription Factor family |
|  |  |  |  |  |  |  |  |  |  |  |  |  |  |  |  |  |  |  |  |  |  |  |  |  |  |  |  |  |  |  |  |  |  |  |  |  |  | *tbp-1* | TATA-Binding Protein |
|  |  |  |  |  |  |  |  |  |  |  |  |  |  |  |  |  |  |  |  |  |  |  |  |  |  |  |  |  |  |  |  |  |  |  |  |  |  | *tdp-1* | TAR DNA-binding Protein homolog |
|  |  |  |  |  |  |  |  |  |  |  |  |  |  |  |  |  |  |  |  |  |  |  |  |  |  |  |  |  |  |  |  |  |  |  |  |  |  | *gsp-2* | GLC7 (yeast Glc Seven) like Phosphatase |
|  |  |  |  |  |  |  |  |  |  |  |  |  |  |  |  |  |  |  |  |  |  |  |  |  |  |  |  |  |  |  |  |  |  |  |  |  |  | F56D2.6 |  |
|  |  |  |  |  |  |  |  |  |  |  |  |  |  |  |  |  |  |  |  |  |  |  |  |  |  |  |  |  |  |  |  |  |  |  |  |  |  | *skp-1* | mammalian SKIP (Ski interacting protein) homolog |
|  |  |  |  |  |  |  |  |  |  |  |  |  |  |  |  |  |  |  |  |  |  |  |  |  |  |  |  |  |  |  |  |  |  |  |  |  |  | *hda-1* | Histone DeAcetylase |
|  |  |  |  |  |  |  |  |  |  |  |  |  |  |  |  |  |  |  |  |  |  |  |  |  |  |  |  |  |  |  |  |  |  |  |  |  |  | E02D9.1 |  |
|  |  |  |  |  |  |  |  |  |  |  |  |  |  |  |  |  |  |  |  |  |  |  |  |  |  |  |  |  |  |  |  |  |  |  |  |  |  | *cdk-9* | Cyclin-Dependent Kinase family |
|  |  |  |  |  |  |  |  |  |  |  |  |  |  |  |  |  |  |  |  |  |  |  |  |  |  |  |  |  |  |  |  |  |  |  |  |  |  | *atg-4.1* | AuTophaGy (yeast Atg homolog) |
|  |  |  |  |  |  |  |  |  |  |  |  |  |  |  |  |  |  |  |  |  |  |  |  |  |  |  |  |  |  |  |  |  |  |  |  |  |  | Y63D3A.8 |  |
|  |  |  |  |  |  |  |  |  |  |  |  |  |  |  |  |  |  |  |  |  |  |  |  |  |  |  |  |  |  |  |  |  |  |  |  |  |  | *hrp-2* | human HnRNP A1 homolog |
|  |  |  |  |  |  |  |  |  |  |  |  |  |  |  |  |  |  |  |  |  |  |  |  |  |  |  |  |  |  |  |  |  |  |  |  |  |  | *cul-2* | CULlin |
|  |  |  |  |  |  |  |  |  |  |  |  |  |  |  |  |  |  |  |  |  |  |  |  |  |  |  |  |  |  |  |  |  |  |  |  |  |  | *lin-65* | abnormal cell LINeage |
|  |  |  |  |  |  |  |  |  |  |  |  |  |  |  |  |  |  |  |  |  |  |  |  |  |  |  |  |  |  |  |  |  |  |  |  |  |  | *hmp-2* | HuMPback (dorsal lumps) |
|  |  |  |  |  |  |  |  |  |  |  |  |  |  |  |  |  |  |  |  |  |  |  |  |  |  |  |  |  |  |  |  |  |  |  |  |  |  | *madf-3* | MADF domain transcription factor |
|  |  |  |  |  |  |  |  |  |  |  |  |  |  |  |  |  |  |  |  |  |  |  |  |  |  |  |  |  |  |  |  |  |  |  |  |  |  | H14E04.2 |  |
|  |  |  |  |  |  |  |  |  |  |  |  |  |  |  |  |  |  |  |  |  |  |  |  |  |  |  |  |  |  |  |  |  |  |  |  |  |  | *tbc-20* | TBC (Tre-2/Bub2/Cdc16) domain family |
|  |  |  |  |  |  |  |  |  |  |  |  |  |  |  |  |  |  |  |  |  |  |  |  |  |  |  |  |  |  |  |  |  |  |  |  |  |  | *unc-50* | UNCoordinated |
|  |  |  |  |  |  |  |  |  |  |  |  |  |  |  |  |  |  |  |  |  |  |  |  |  |  |  |  |  |  |  |  |  |  |  |  |  |  | *dcap-1* | mRNA DeCAPping enzyme |
|  |  |  |  |  |  |  |  |  |  |  |  |  |  |  |  |  |  |  |  |  |  |  |  |  |  |  |  |  |  |  |  |  |  |  |  |  |  | *taf-4* | TAF (TBP-associated transcription factor) family |
|  |  |  |  |  |  |  |  |  |  |  |  |  |  |  |  |  |  |  |  |  |  |  |  |  |  |  |  |  |  |  |  |  |  |  |  |  |  | *fust-1* | FUS/TLS RNA binding protein homolog |
|  |  |  |  |  |  |  |  |  |  |  |  |  |  |  |  |  |  |  |  |  |  |  |  |  |  |  |  |  |  |  |  |  |  |  |  |  |  | C53D5.5 |  |
|  |  |  |  |  |  |  |  |  |  |  |  |  |  |  |  |  |  |  |  |  |  |  |  |  |  |  |  |  |  |  |  |  |  |  |  |  |  | B0285.3 |  |
|  |  |  |  |  |  |  |  |  |  |  |  |  |  |  |  |  |  |  |  |  |  |  |  |  |  |  |  |  |  |  |  |  |  |  |  |  |  | *cogc-8* | Conserved Oligomeric Golgi (COG) Component |
|  |  |  |  |  |  |  |  |  |  |  |  |  |  |  |  |  |  |  |  |  |  |  |  |  |  |  |  |  |  |  |  |  |  |  |  |  |  | K10C3.4 |  |
|  |  |  |  |  |  |  |  |  |  |  |  |  |  |  |  |  |  |  |  |  |  |  |  |  |  |  |  |  |  |  |  |  |  |  |  |  |  | *thoc-1* | THO Complex (transcription factor/nuclear export) subunit |
|  |  |  |  |  |  |  |  |  |  |  |  |  |  |  |  |  |  |  |  |  |  |  |  |  |  |  |  |  |  |  |  |  |  |  |  |  |  | *rsr-2* | SR protein related |
|  |  |  |  |  |  |  |  |  |  |  |  |  |  |  |  |  |  |  |  |  |  |  |  |  |  |  |  |  |  |  |  |  |  |  |  |  |  | *rme-6* | Receptor Mediated Endocytosis |
|  |  |  |  |  |  |  |  |  |  |  |  |  |  |  |  |  |  |  |  |  |  |  |  |  |  |  |  |  |  |  |  |  |  |  |  |  |  | *vps-16* | related to yeast Vacuolar Protein Sorting factor |
|  |  |  |  |  |  |  |  |  |  |  |  |  |  |  |  |  |  |  |  |  |  |  |  |  |  |  |  |  |  |  |  |  |  |  |  |  |  | *vps-41* | related to yeast Vacuolar Protein Sorting factor |
|  |  |  |  |  |  |  |  |  |  |  |  |  |  |  |  |  |  |  |  |  |  |  |  |  |  |  |  |  |  |  |  |  |  |  |  |  |  | *cacn-1* | CACtiN (Drosophila cactus interacting protein) homolog |
|  |  |  |  |  |  |  |  |  |  |  |  |  |  |  |  |  |  |  |  |  |  |  |  |  |  |  |  |  |  |  |  |  |  |  |  |  |  | K09E4.2 |  |
|  |  |  |  |  |  |  |  |  |  |  |  |  |  |  |  |  |  |  |  |  |  |  |  |  |  |  |  |  |  |  |  |  |  |  |  |  |  | *smg-2* | Suppressor with Morphological effect on Genitalia |
|  |  |  |  |  |  |  |  |  |  |  |  |  |  |  |  |  |  |  |  |  |  |  |  |  |  |  |  |  |  |  |  |  |  |  |  |  |  | C44B7.2 |  |
|  |  |  |  |  |  |  |  |  |  |  |  |  |  |  |  |  |  |  |  |  |  |  |  |  |  |  |  |  |  |  |  |  |  |  |  |  |  | *vps-15* | related to yeast Vacuolar Protein Sorting factor |
|  |  |  |  |  |  |  |  |  |  |  |  |  |  |  |  |  |  |  |  |  |  |  |  |  |  |  |  |  |  |  |  |  |  |  |  |  |  | *smgl-2* | SMG-associated and Lethal |
|  |  |  |  |  |  |  |  |  |  |  |  |  |  |  |  |  |  |  |  |  |  |  |  |  |  |  |  |  |  |  |  |  |  |  |  |  |  | *pqn-41* | Prion-like-(Q/N-rich)-domain-bearing protein |
|  |  |  |  |  |  |  |  |  |  |  |  |  |  |  |  |  |  |  |  |  |  |  |  |  |  |  |  |  |  |  |  |  |  |  |  |  |  | *ntl-2* | NOT-Like (yeast CCR4/NOT complex component) |
|  |  |  |  |  |  |  |  |  |  |  |  |  |  |  |  |  |  |  |  |  |  |  |  |  |  |  |  |  |  |  |  |  |  |  |  |  |  | Y17G7B.20 |  |
|  |  |  |  |  |  |  |  |  |  |  |  |  |  |  |  |  |  |  |  |  |  |  |  |  |  |  |  |  |  |  |  |  |  |  |  |  |  | C10G11.7 |  |
|  |  |  |  |  |  |  |  |  |  |  |  |  |  |  |  |  |  |  |  |  |  |  |  |  |  |  |  |  |  |  |  |  |  |  |  |  |  | *epn-1* | EPsiN (endocytic protein) homolog |
|  |  |  |  |  |  |  |  |  |  |  |  |  |  |  |  |  |  |  |  |  |  |  |  |  |  |  |  |  |  |  |  |  |  |  |  |  |  | *aak-2* | AMP-Activated Kinase |
|  |  |  |  |  |  |  |  |  |  |  |  |  |  |  |  |  |  |  |  |  |  |  |  |  |  |  |  |  |  |  |  |  |  |  |  |  |  | *atln-1* | ATLastiN (endoplasmic reticulum GTPase) related |
|  |  |  |  |  |  |  |  |  |  |  |  |  |  |  |  |  |  |  |  |  |  |  |  |  |  |  |  |  |  |  |  |  |  |  |  |  |  | *aakg-2* | AMP-Activated protein Kinase Gamma subunit |
|  |  |  |  |  |  |  |  |  |  |  |  |  |  |  |  |  |  |  |  |  |  |  |  |  |  |  |  |  |  |  |  |  |  |  |  |  |  | ZK484.3 |  |
|  |  |  |  |  |  |  |  |  |  |  |  |  |  |  |  |  |  |  |  |  |  |  |  |  |  |  |  |  |  |  |  |  |  |  |  |  |  | C46F11.5 |  |
|  |  |  |  |  |  |  |  |  |  |  |  |  |  |  |  |  |  |  |  |  |  |  |  |  |  |  |  |  |  |  |  |  |  |  |  |  |  | *aha-1* | Aryl Hydrocarbon receptor Associated protein |
|  |  |  |  |  |  |  |  |  |  |  |  |  |  |  |  |  |  |  |  |  |  |  |  |  |  |  |  |  |  |  |  |  |  |  |  |  |  | *pmk-1* | P38 Map Kinase family |
|  |  |  |  |  |  |  |  |  |  |  |  |  |  |  |  |  |  |  |  |  |  |  |  |  |  |  |  |  |  |  |  |  |  |  |  |  |  | *sek-1* | SAPK/ERK kinase |
|  |  |  |  |  |  |  |  |  |  |  |  |  |  |  |  |  |  |  |  |  |  |  |  |  |  |  |  |  |  |  |  |  |  |  |  |  |  | *ain-2* | ALG-1 INteracting protein |
|  |  |  |  |  |  |  |  |  |  |  |  |  |  |  |  |  |  |  |  |  |  |  |  |  |  |  |  |  |  |  |  |  |  |  |  |  |  | M01E5.3 |  |
|  |  |  |  |  |  |  |  |  |  |  |  |  |  |  |  |  |  |  |  |  |  |  |  |  |  |  |  |  |  |  |  |  |  |  |  |  |  | *mak-2* | MAP kinase Activated protein Kinase |
|  |  |  |  |  |  |  |  |  |  |  |  |  |  |  |  |  |  |  |  |  |  |  |  |  |  |  |  |  |  |  |  |  |  |  |  |  |  | *abtm-1* | ABC Transporter, Mitochondrial |
|  |  |  |  |  |  |  |  |  |  |  |  |  |  |  |  |  |  |  |  |  |  |  |  |  |  |  |  |  |  |  |  |  |  |  |  |  |  | Y105E8A.19 |  |
|  |  |  |  |  |  |  |  |  |  |  |  |  |  |  |  |  |  |  |  |  |  |  |  |  |  |  |  |  |  |  |  |  |  |  |  |  |  | *set-25* | SET (trithorax/polycomb) domain containing |
|  |  |  |  |  |  |  |  |  |  |  |  |  |  |  |  |  |  |  |  |  |  |  |  |  |  |  |  |  |  |  |  |  |  |  |  |  |  | *lit-1* | Loss of InTestine |
|  |  |  |  |  |  |  |  |  |  |  |  |  |  |  |  |  |  |  |  |  |  |  |  |  |  |  |  |  |  |  |  |  |  |  |  |  |  | *gei-4* | GEX Interacting protein |
|  |  |  |  |  |  |  |  |  |  |  |  |  |  |  |  |  |  |  |  |  |  |  |  |  |  |  |  |  |  |  |  |  |  |  |  |  |  | *rom-4* | RhOMboid (Drosophila) related |
|  |  |  |  |  |  |  |  |  |  |  |  |  |  |  |  |  |  |  |  |  |  |  |  |  |  |  |  |  |  |  |  |  |  |  |  |  |  | *swan-2* | Seven WD repeats, AN11 family |
|  |  |  |  |  |  |  |  |  |  |  |  |  |  |  |  |  |  |  |  |  |  |  |  |  |  |  |  |  |  |  |  |  |  |  |  |  |  | *otub-2* | OTUBain deubiquitylating protease homolog |
|  |  |  |  |  |  |  |  |  |  |  |  |  |  |  |  |  |  |  |  |  |  |  |  |  |  |  |  |  |  |  |  |  |  |  |  |  |  | *fzr-1* | FiZzy Related family |
|  |  |  |  |  |  |  |  |  |  |  |  |  |  |  |  |  |  |  |  |  |  |  |  |  |  |  |  |  |  |  |  |  |  |  |  |  |  | *ppgn-1* | ParaPleGiN AAA protease family |
|  |  |  |  |  |  |  |  |  |  |  |  |  |  |  |  |  |  |  |  |  |  |  |  |  |  |  |  |  |  |  |  |  |  |  |  |  |  | *fre-1* | Flavin REductase |
|  |  |  |  |  |  |  |  |  |  |  |  |  |  |  |  |  |  |  |  |  |  |  |  |  |  |  |  |  |  |  |  |  |  |  |  |  |  | *erfa-3* | Eukaryotic Release FActor homolog |
|  |  |  |  |  |  |  |  |  |  |  |  |  |  |  |  |  |  |  |  |  |  |  |  |  |  |  |  |  |  |  |  |  |  |  |  |  |  | *sel-10* | Suppressor/Enhancer of Lin-12 |
|  |  |  |  |  |  |  |  |  |  |  |  |  |  |  |  |  |  |  |  |  |  |  |  |  |  |  |  |  |  |  |  |  |  |  |  |  |  | *dpt-1* | Dipeptidyl Peptidase Three |
|  |  |  |  |  |  |  |  |  |  |  |  |  |  |  |  |  |  |  |  |  |  |  |  |  |  |  |  |  |  |  |  |  |  |  |  |  |  | Y59A8B.10 |  |
|  |  |  |  |  |  |  |  |  |  |  |  |  |  |  |  |  |  |  |  |  |  |  |  |  |  |  |  |  |  |  |  |  |  |  |  |  |  | F26B1.2 |  |
|  |  |  |  |  |  |  |  |  |  |  |  |  |  |  |  |  |  |  |  |  |  |  |  |  |  |  |  |  |  |  |  |  |  |  |  |  |  | *ntl-11* | NOT-Like (yeast CCR4/NOT complex component) |
|  |  |  |  |  |  |  |  |  |  |  |  |  |  |  |  |  |  |  |  |  |  |  |  |  |  |  |  |  |  |  |  |  |  |  |  |  |  | *mdt-29* | MeDiaTor |
|  |  |  |  |  |  |  |  |  |  |  |  |  |  |  |  |  |  |  |  |  |  |  |  |  |  |  |  |  |  |  |  |  |  |  |  |  |  | *lst-3* | Lateral Signaling Target |
|  |  |  |  |  |  |  |  |  |  |  |  |  |  |  |  |  |  |  |  |  |  |  |  |  |  |  |  |  |  |  |  |  |  |  |  |  |  | *pot-3* | Protection Of Telomeres 1 (Pot1) homolog |
|  |  |  |  |  |  |  |  |  |  |  |  |  |  |  |  |  |  |  |  |  |  |  |  |  |  |  |  |  |  |  |  |  |  |  |  |  |  | *mat-1* | Metaphase-to-Anaphase Transition defect |
|  |  |  |  |  |  |  |  |  |  |  |  |  |  |  |  |  |  |  |  |  |  |  |  |  |  |  |  |  |  |  |  |  |  |  |  |  |  | *nra-4* | Nicotinic Receptor Associated |
|  |  |  |  |  |  |  |  |  |  |  |  |  |  |  |  |  |  |  |  |  |  |  |  |  |  |  |  |  |  |  |  |  |  |  |  |  |  | *trpp-11* | TRansport Protein Particle |
|  |  |  |  |  |  |  |  |  |  |  |  |  |  |  |  |  |  |  |  |  |  |  |  |  |  |  |  |  |  |  |  |  |  |  |  |  |  | *vps-11* | related to yeast Vacuolar Protein Sorting factor |
|  |  |  |  |  |  |  |  |  |  |  |  |  |  |  |  |  |  |  |  |  |  |  |  |  |  |  |  |  |  |  |  |  |  |  |  |  |  | Y69A2AR.16 |  |
|  |  |  |  |  |  |  |  |  |  |  |  |  |  |  |  |  |  |  |  |  |  |  |  |  |  |  |  |  |  |  |  |  |  |  |  |  |  | C41D11.3 |  |
|  |  |  |  |  |  |  |  |  |  |  |  |  |  |  |  |  |  |  |  |  |  |  |  |  |  |  |  |  |  |  |  |  |  |  |  |  |  | B0336.3 |  |
|  |  |  |  |  |  |  |  |  |  |  |  |  |  |  |  |  |  |  |  |  |  |  |  |  |  |  |  |  |  |  |  |  |  |  |  |  |  | *xpo-1* | eXPOrtin (nuclear export receptor) |
|  |  |  |  |  |  |  |  |  |  |  |  |  |  |  |  |  |  |  |  |  |  |  |  |  |  |  |  |  |  |  |  |  |  |  |  |  |  | *vbh-1* | Vasa- and Belle-like Helicase |
|  |  |  |  |  |  |  |  |  |  |  |  |  |  |  |  |  |  |  |  |  |  |  |  |  |  |  |  |  |  |  |  |  |  |  |  |  |  | *aat-9* | Amino Acid Transporter |
|  |  |  |  |  |  |  |  |  |  |  |  |  |  |  |  |  |  |  |  |  |  |  |  |  |  |  |  |  |  |  |  |  |  |  |  |  |  | *tsr-1* | Transporter of SR proteins |
|  |  |  |  |  |  |  |  |  |  |  |  |  |  |  |  |  |  |  |  |  |  |  |  |  |  |  |  |  |  |  |  |  |  |  |  |  |  | *laf-1* | Lethal And Feminizing |
|  |  |  |  |  |  |  |  |  |  |  |  |  |  |  |  |  |  |  |  |  |  |  |  |  |  |  |  |  |  |  |  |  |  |  |  |  |  | *ash-2* | ASH histone methyltransferase complex subunit (Drosophila absent, small or homeotic discs) |
|  |  |  |  |  |  |  |  |  |  |  |  |  |  |  |  |  |  |  |  |  |  |  |  |  |  |  |  |  |  |  |  |  |  |  |  |  |  | C15H9.4 |  |
|  |  |  |  |  |  |  |  |  |  |  |  |  |  |  |  |  |  |  |  |  |  |  |  |  |  |  |  |  |  |  |  |  |  |  |  |  |  | *ccr-4* | CCR (yeast CCR4/NOT complex component) homolog |
|  |  |  |  |  |  |  |  |  |  |  |  |  |  |  |  |  |  |  |  |  |  |  |  |  |  |  |  |  |  |  |  |  |  |  |  |  |  | *set-16* | SET (trithorax/polycomb) domain containing |
|  |  |  |  |  |  |  |  |  |  |  |  |  |  |  |  |  |  |  |  |  |  |  |  |  |  |  |  |  |  |  |  |  |  |  |  |  |  | *ain-1* | ALG-1 INteracting protein |
|  |  |  |  |  |  |  |  |  |  |  |  |  |  |  |  |  |  |  |  |  |  |  |  |  |  |  |  |  |  |  |  |  |  |  |  |  |  | *ifet-1* | eIF4E Transporter |
|  |  |  |  |  |  |  |  |  |  |  |  |  |  |  |  |  |  |  |  |  |  |  |  |  |  |  |  |  |  |  |  |  |  |  |  |  |  | *let-711* | LEThal |
|  |  |  |  |  |  |  |  |  |  |  |  |  |  |  |  |  |  |  |  |  |  |  |  |  |  |  |  |  |  |  |  |  |  |  |  |  |  | *chd-7* | Chromodomain and Helicase Domain protein |
|  |  |  |  |  |  |  |  |  |  |  |  |  |  |  |  |  |  |  |  |  |  |  |  |  |  |  |  |  |  |  |  |  |  |  |  |  |  | *hpk-1* | Homeodomain interacting Protein Kinase |
|  |  |  |  |  |  |  |  |  |  |  |  |  |  |  |  |  |  |  |  |  |  |  |  |  |  |  |  |  |  |  |  |  |  |  |  |  |  | *wwp-1* | WW domain Protein (E3 ubiquitin ligase) |
|  |  |  |  |  |  |  |  |  |  |  |  |  |  |  |  |  |  |  |  |  |  |  |  |  |  |  |  |  |  |  |  |  |  |  |  |  |  | *acs-13* | fatty Acid CoA Synthetase family |
|  |  |  |  |  |  |  |  |  |  |  |  |  |  |  |  |  |  |  |  |  |  |  |  |  |  |  |  |  |  |  |  |  |  |  |  |  |  | *apg-1* | AdaPtin, Gamma chain (clathrin associated complex) |
|  |  |  |  |  |  |  |  |  |  |  |  |  |  |  |  |  |  |  |  |  |  |  |  |  |  |  |  |  |  |  |  |  |  |  |  |  |  | *sec-24.2* | yeast SEC homolog |
|  |  |  |  |  |  |  |  |  |  |  |  |  |  |  |  |  |  |  |  |  |  |  |  |  |  |  |  |  |  |  |  |  |  |  |  |  |  | *tag-123* | Temporarily Assigned Gene name |
|  |  |  |  |  |  |  |  |  |  |  |  |  |  |  |  |  |  |  |  |  |  |  |  |  |  |  |  |  |  |  |  |  |  |  |  |  |  | Y37E3.10 |  |
|  |  |  |  |  |  |  |  |  |  |  |  |  |  |  |  |  |  |  |  |  |  |  |  |  |  |  |  |  |  |  |  |  |  |  |  |  |  | F43D9.3 |  |
|  |  |  |  |  |  |  |  |  |  |  |  |  |  |  |  |  |  |  |  |  |  |  |  |  |  |  |  |  |  |  |  |  |  |  |  |  |  | Y37E3.17 |  |
|  |  |  |  |  |  |  |  |  |  |  |  |  |  |  |  |  |  |  |  |  |  |  |  |  |  |  |  |  |  |  |  |  |  |  |  |  |  | *gly-9* | GLYcosylation related |
|  |  |  |  |  |  |  |  |  |  |  |  |  |  |  |  |  |  |  |  |  |  |  |  |  |  |  |  |  |  |  |  |  |  |  |  |  |  | Y75B8A.16 |  |
|  |  |  |  |  |  |  |  |  |  |  |  |  |  |  |  |  |  |  |  |  |  |  |  |  |  |  |  |  |  |  |  |  |  |  |  |  |  | Y41D4A.4 |  |
|  |  |  |  |  |  |  |  |  |  |  |  |  |  |  |  |  |  |  |  |  |  |  |  |  |  |  |  |  |  |  |  |  |  |  |  |  |  | *afd-1* | AFaDin (actin filament binding protein) homolog |
|  |  |  |  |  |  |  |  |  |  |  |  |  |  |  |  |  |  |  |  |  |  |  |  |  |  |  |  |  |  |  |  |  |  |  |  |  |  | Y65B4A.6 |  |
|  |  |  |  |  |  |  |  |  |  |  |  |  |  |  |  |  |  |  |  |  |  |  |  |  |  |  |  |  |  |  |  |  |  |  |  |  |  | *tub-2* | TUBby-related |
|  |  |  |  |  |  |  |  |  |  |  |  |  |  |  |  |  |  |  |  |  |  |  |  |  |  |  |  |  |  |  |  |  |  |  |  |  |  | *attf-3* | AT hook Transcription Factor family |
|  |  |  |  |  |  |  |  |  |  |  |  |  |  |  |  |  |  |  |  |  |  |  |  |  |  |  |  |  |  |  |  |  |  |  |  |  |  | Y110A2AM.1 |  |
|  |  |  |  |  |  |  |  |  |  |  |  |  |  |  |  |  |  |  |  |  |  |  |  |  |  |  |  |  |  |  |  |  |  |  |  |  |  | *ego-2* | Enhancer of Glp-One (glp-1) |
|  |  |  |  |  |  |  |  |  |  |  |  |  |  |  |  |  |  |  |  |  |  |  |  |  |  |  |  |  |  |  |  |  |  |  |  |  |  | W03F9.1 |  |
|  |  |  |  |  |  |  |  |  |  |  |  |  |  |  |  |  |  |  |  |  |  |  |  |  |  |  |  |  |  |  |  |  |  |  |  |  |  | Y37H9A.1 |  |
|  |  |  |  |  |  |  |  |  |  |  |  |  |  |  |  |  |  |  |  |  |  |  |  |  |  |  |  |  |  |  |  |  |  |  |  |  |  | W02H5.9 |  |
|  |  |  |  |  |  |  |  |  |  |  |  |  |  |  |  |  |  |  |  |  |  |  |  |  |  |  |  |  |  |  |  |  |  |  |  |  |  | B0350.71 |  |
|  |  |  |  |  |  |  |  |  |  |  |  |  |  |  |  |  |  |  |  |  |  |  |  |  |  |  |  |  |  |  |  |  |  |  |  |  |  | T19B10.8 |  |
|  |  |  |  |  |  |  |  |  |  |  |  |  |  |  |  |  |  |  |  |  |  |  |  |  |  |  |  |  |  |  |  |  |  |  |  |  |  | Y38C1AA.12 |  |
|  |  |  |  |  |  |  |  |  |  |  |  |  |  |  |  |  |  |  |  |  |  |  |  |  |  |  |  |  |  |  |  |  |  |  |  |  |  | *nhr-274* | Nuclear Hormone Receptor family |
|  |  |  |  |  |  |  |  |  |  |  |  |  |  |  |  |  |  |  |  |  |  |  |  |  |  |  |  |  |  |  |  |  |  |  |  |  |  | Y39H10A.6 |  |
|  |  |  |  |  |  |  |  |  |  |  |  |  |  |  |  |  |  |  |  |  |  |  |  |  |  |  |  |  |  |  |  |  |  |  |  |  |  | *sec-24.1* | yeast SEC homolog |
|  |  |  |  |  |  |  |  |  |  |  |  |  |  |  |  |  |  |  |  |  |  |  |  |  |  |  |  |  |  |  |  |  |  |  |  |  |  | *hlb-1* | Homolog of Liprin Beta |
|  |  |  |  |  |  |  |  |  |  |  |  |  |  |  |  |  |  |  |  |  |  |  |  |  |  |  |  |  |  |  |  |  |  |  |  |  |  | *mrps-22* | Mitochondrial Ribosomal Protein, Small |
|  |  |  |  |  |  |  |  |  |  |  |  |  |  |  |  |  |  |  |  |  |  |  |  |  |  |  |  |  |  |  |  |  |  |  |  |  |  | *otub-4* | OTUBain deubiquitylating protease homolog |
|  |  |  |  |  |  |  |  |  |  |  |  |  |  |  |  |  |  |  |  |  |  |  |  |  |  |  |  |  |  |  |  |  |  |  |  |  |  | K07B1.8 |  |
|  |  |  |  |  |  |  |  |  |  |  |  |  |  |  |  |  |  |  |  |  |  |  |  |  |  |  |  |  |  |  |  |  |  |  |  |  |  | *dhs-13* | DeHydrogenases, Short chain |
|  |  |  |  |  |  |  |  |  |  |  |  |  |  |  |  |  |  |  |  |  |  |  |  |  |  |  |  |  |  |  |  |  |  |  |  |  |  | T04B8.5 |  |
|  |  |  |  |  |  |  |  |  |  |  |  |  |  |  |  |  |  |  |  |  |  |  |  |  |  |  |  |  |  |  |  |  |  |  |  |  |  | *mnk-1* | MAP kinase iNtegrating Kinase (MNK) homolog |
|  |  |  |  |  |  |  |  |  |  |  |  |  |  |  |  |  |  |  |  |  |  |  |  |  |  |  |  |  |  |  |  |  |  |  |  |  |  | R166.6 |  |
|  |  |  |  |  |  |  |  |  |  |  |  |  |  |  |  |  |  |  |  |  |  |  |  |  |  |  |  |  |  |  |  |  |  |  |  |  |  | *ogdh-1* | OxoGlutarate DeHydrogenase |
|  |  |  |  |  |  |  |  |  |  |  |  |  |  |  |  |  |  |  |  |  |  |  |  |  |  |  |  |  |  |  |  |  |  |  |  |  |  | *copb-2* | COP (COat Protomer) Beta subunit |
|  |  |  |  |  |  |  |  |  |  |  |  |  |  |  |  |  |  |  |  |  |  |  |  |  |  |  |  |  |  |  |  |  |  |  |  |  |  | ZK858.6 |  |
|  |  |  |  |  |  |  |  |  |  |  |  |  |  |  |  |  |  |  |  |  |  |  |  |  |  |  |  |  |  |  |  |  |  |  |  |  |  | *let-502* | LEThal |
|  |  |  |  |  |  |  |  |  |  |  |  |  |  |  |  |  |  |  |  |  |  |  |  |  |  |  |  |  |  |  |  |  |  |  |  |  |  | *tag-153* | Temporarily Assigned Gene name |
|  |  |  |  |  |  |  |  |  |  |  |  |  |  |  |  |  |  |  |  |  |  |  |  |  |  |  |  |  |  |  |  |  |  |  |  |  |  | *gly-7* | GLYcosylation related |
|  |  |  |  |  |  |  |  |  |  |  |  |  |  |  |  |  |  |  |  |  |  |  |  |  |  |  |  |  |  |  |  |  |  |  |  |  |  | *hsp-3* | Heat Shock Protein |
|  |  |  |  |  |  |  |  |  |  |  |  |  |  |  |  |  |  |  |  |  |  |  |  |  |  |  |  |  |  |  |  |  |  |  |  |  |  | Y37E11AM.3 |  |
|  |  |  |  |  |  |  |  |  |  |  |  |  |  |  |  |  |  |  |  |  |  |  |  |  |  |  |  |  |  |  |  |  |  |  |  |  |  | *gck-1* | Germinal Center Kinase family |
|  |  |  |  |  |  |  |  |  |  |  |  |  |  |  |  |  |  |  |  |  |  |  |  |  |  |  |  |  |  |  |  |  |  |  |  |  |  | *ina-1* | INtegrin Alpha |
|  |  |  |  |  |  |  |  |  |  |  |  |  |  |  |  |  |  |  |  |  |  |  |  |  |  |  |  |  |  |  |  |  |  |  |  |  |  | F31A3.5 |  |
|  |  |  |  |  |  |  |  |  |  |  |  |  |  |  |  |  |  |  |  |  |  |  |  |  |  |  |  |  |  |  |  |  |  |  |  |  |  | *pkc-3* | Protein Kinase C |
|  |  |  |  |  |  |  |  |  |  |  |  |  |  |  |  |  |  |  |  |  |  |  |  |  |  |  |  |  |  |  |  |  |  |  |  |  |  | *cogc-4* | Conserved Oligomeric Golgi (COG) Component |
|  |  |  |  |  |  |  |  |  |  |  |  |  |  |  |  |  |  |  |  |  |  |  |  |  |  |  |  |  |  |  |  |  |  |  |  |  |  | *bed-2* | BED-type zinc finger putative transcription factor |
|  |  |  |  |  |  |  |  |  |  |  |  |  |  |  |  |  |  |  |  |  |  |  |  |  |  |  |  |  |  |  |  |  |  |  |  |  |  | *hda-10* | Histone DeAcetylase |
|  |  |  |  |  |  |  |  |  |  |  |  |  |  |  |  |  |  |  |  |  |  |  |  |  |  |  |  |  |  |  |  |  |  |  |  |  |  | Y9D1A.1 |  |
|  |  |  |  |  |  |  |  |  |  |  |  |  |  |  |  |  |  |  |  |  |  |  |  |  |  |  |  |  |  |  |  |  |  |  |  |  |  | T04C4.1 |  |
|  |  |  |  |  |  |  |  |  |  |  |  |  |  |  |  |  |  |  |  |  |  |  |  |  |  |  |  |  |  |  |  |  |  |  |  |  |  | *tut-1* | Thiolation of Uridine in TRNA |
|  |  |  |  |  |  |  |  |  |  |  |  |  |  |  |  |  |  |  |  |  |  |  |  |  |  |  |  |  |  |  |  |  |  |  |  |  |  | *cdkr-3* | CDK5 Regulation associated protein |
|  |  |  |  |  |  |  |  |  |  |  |  |  |  |  |  |  |  |  |  |  |  |  |  |  |  |  |  |  |  |  |  |  |  |  |  |  |  | *nuo-5* | NADH Ubiquinone Oxidoreductase |
|  |  |  |  |  |  |  |  |  |  |  |  |  |  |  |  |  |  |  |  |  |  |  |  |  |  |  |  |  |  |  |  |  |  |  |  |  |  | *sec-23* | yeast SEC homolog |
|  |  |  |  |  |  |  |  |  |  |  |  |  |  |  |  |  |  |  |  |  |  |  |  |  |  |  |  |  |  |  |  |  |  |  |  |  |  | *aagr-3* | Acid Alpha Glucosidase Relate |
|  |  |  |  |  |  |  |  |  |  |  |  |  |  |  |  |  |  |  |  |  |  |  |  |  |  |  |  |  |  |  |  |  |  |  |  |  |  | Y105E8A.3 |  |
|  |  |  |  |  |  |  |  |  |  |  |  |  |  |  |  |  |  |  |  |  |  |  |  |  |  |  |  |  |  |  |  |  |  |  |  |  |  | *stt-3* | STT (yeast oligosaccharyltransferase subunit) homolog |
|  |  |  |  |  |  |  |  |  |  |  |  |  |  |  |  |  |  |  |  |  |  |  |  |  |  |  |  |  |  |  |  |  |  |  |  |  |  | K11H12.13 |  |
|  |  |  |  |  |  |  |  |  |  |  |  |  |  |  |  |  |  |  |  |  |  |  |  |  |  |  |  |  |  |  |  |  |  |  |  |  |  | C35E7.6 |  |
|  |  |  |  |  |  |  |  |  |  |  |  |  |  |  |  |  |  |  |  |  |  |  |  |  |  |  |  |  |  |  |  |  |  |  |  |  |  | Y71G12B.13 |  |
|  |  |  |  |  |  |  |  |  |  |  |  |  |  |  |  |  |  |  |  |  |  |  |  |  |  |  |  |  |  |  |  |  |  |  |  |  |  | *linc-71* | Long Intervening Non-Coding RNA |
|  |  |  |  |  |  |  |  |  |  |  |  |  |  |  |  |  |  |  |  |  |  |  |  |  |  |  |  |  |  |  |  |  |  |  |  |  |  | Y54E10A.6 |  |
|  |  |  |  |  |  |  |  |  |  |  |  |  |  |  |  |  |  |  |  |  |  |  |  |  |  |  |  |  |  |  |  |  |  |  |  |  |  | F53A2.9 |  |
|  |  |  |  |  |  |  |  |  |  |  |  |  |  |  |  |  |  |  |  |  |  |  |  |  |  |  |  |  |  |  |  |  |  |  |  |  |  | *hpo-11* | Hypersensitive to POre-forming toxin |
|  |  |  |  |  |  |  |  |  |  |  |  |  |  |  |  |  |  |  |  |  |  |  |  |  |  |  |  |  |  |  |  |  |  |  |  |  |  | C05D11.8 |  |
|  |  |  |  |  |  |  |  |  |  |  |  |  |  |  |  |  |  |  |  |  |  |  |  |  |  |  |  |  |  |  |  |  |  |  |  |  |  | F02E11.4 |  |
|  |  |  |  |  |  |  |  |  |  |  |  |  |  |  |  |  |  |  |  |  |  |  |  |  |  |  |  |  |  |  |  |  |  |  |  |  |  | *fbxa-80* | F-box A protein |
|  |  |  |  |  |  |  |  |  |  |  |  |  |  |  |  |  |  |  |  |  |  |  |  |  |  |  |  |  |  |  |  |  |  |  |  |  |  | F25H2.14 |  |
|  |  |  |  |  |  |  |  |  |  |  |  |  |  |  |  |  |  |  |  |  |  |  |  |  |  |  |  |  |  |  |  |  |  |  |  |  |  | *ppfr-4* | Protein Phosphatase Four Regulatory subunit |
|  |  |  |  |  |  |  |  |  |  |  |  |  |  |  |  |  |  |  |  |  |  |  |  |  |  |  |  |  |  |  |  |  |  |  |  |  |  | T06E6.14 |  |
|  |  |  |  |  |  |  |  |  |  |  |  |  |  |  |  |  |  |  |  |  |  |  |  |  |  |  |  |  |  |  |  |  |  |  |  |  |  | T21E8.6 |  |
|  |  |  |  |  |  |  |  |  |  |  |  |  |  |  |  |  |  |  |  |  |  |  |  |  |  |  |  |  |  |  |  |  |  |  |  |  |  | *nol-1* | NucleOLar protein |
|  |  |  |  |  |  |  |  |  |  |  |  |  |  |  |  |  |  |  |  |  |  |  |  |  |  |  |  |  |  |  |  |  |  |  |  |  |  | Y41C4A.9 |  |
|  |  |  |  |  |  |  |  |  |  |  |  |  |  |  |  |  |  |  |  |  |  |  |  |  |  |  |  |  |  |  |  |  |  |  |  |  |  | *gcn-1* | GCN (yeast General Control Nondrepressible) homolog |
|  |  |  |  |  |  |  |  |  |  |  |  |  |  |  |  |  |  |  |  |  |  |  |  |  |  |  |  |  |  |  |  |  |  |  |  |  |  | Y45F10D.7 |  |
|  |  |  |  |  |  |  |  |  |  |  |  |  |  |  |  |  |  |  |  |  |  |  |  |  |  |  |  |  |  |  |  |  |  |  |  |  |  | F13H8.2 |  |
|  |  |  |  |  |  |  |  |  |  |  |  |  |  |  |  |  |  |  |  |  |  |  |  |  |  |  |  |  |  |  |  |  |  |  |  |  |  | R02F2.7 |  |
|  |  |  |  |  |  |  |  |  |  |  |  |  |  |  |  |  |  |  |  |  |  |  |  |  |  |  |  |  |  |  |  |  |  |  |  |  |  | F55F10.1 |  |
|  |  |  |  |  |  |  |  |  |  |  |  |  |  |  |  |  |  |  |  |  |  |  |  |  |  |  |  |  |  |  |  |  |  |  |  |  |  | Y61A9LA.10 |  |
|  |  |  |  |  |  |  |  |  |  |  |  |  |  |  |  |  |  |  |  |  |  |  |  |  |  |  |  |  |  |  |  |  |  |  |  |  |  | T02H6.1 |  |
|  |  |  |  |  |  |  |  |  |  |  |  |  |  |  |  |  |  |  |  |  |  |  |  |  |  |  |  |  |  |  |  |  |  |  |  |  |  | Y48G1A.4 |  |
|  |  |  |  |  |  |  |  |  |  |  |  |  |  |  |  |  |  |  |  |  |  |  |  |  |  |  |  |  |  |  |  |  |  |  |  |  |  | *elpc-2* | ELongator complex Protein Component |
|  |  |  |  |  |  |  |  |  |  |  |  |  |  |  |  |  |  |  |  |  |  |  |  |  |  |  |  |  |  |  |  |  |  |  |  |  |  | Y51H1A.1 |  |
|  |  |  |  |  |  |  |  |  |  |  |  |  |  |  |  |  |  |  |  |  |  |  |  |  |  |  |  |  |  |  |  |  |  |  |  |  |  | *aat-8* | Amino Acid Transporter |
|  |  |  |  |  |  |  |  |  |  |  |  |  |  |  |  |  |  |  |  |  |  |  |  |  |  |  |  |  |  |  |  |  |  |  |  |  |  | *fbxa-140* | F-box A protein |
|  |  |  |  |  |  |  |  |  |  |  |  |  |  |  |  |  |  |  |  |  |  |  |  |  |  |  |  |  |  |  |  |  |  |  |  |  |  | Y105E8B.5 |  |
|  |  |  |  |  |  |  |  |  |  |  |  |  |  |  |  |  |  |  |  |  |  |  |  |  |  |  |  |  |  |  |  |  |  |  |  |  |  | Y39B6A.49 |  |
|  |  |  |  |  |  |  |  |  |  |  |  |  |  |  |  |  |  |  |  |  |  |  |  |  |  |  |  |  |  |  |  |  |  |  |  |  |  | *retr-1* | RETRotransposon-like |
|  |  |  |  |  |  |  |  |  |  |  |  |  |  |  |  |  |  |  |  |  |  |  |  |  |  |  |  |  |  |  |  |  |  |  |  |  |  | *syp-1* | SYnaPsis in meiosis abnormal |
|  |  |  |  |  |  |  |  |  |  |  |  |  |  |  |  |  |  |  |  |  |  |  |  |  |  |  |  |  |  |  |  |  |  |  |  |  |  | ZC308.4 |  |
|  |  |  |  |  |  |  |  |  |  |  |  |  |  |  |  |  |  |  |  |  |  |  |  |  |  |  |  |  |  |  |  |  |  |  |  |  |  | *vha-7* | Vacuolar H ATPase |
|  |  |  |  |  |  |  |  |  |  |  |  |  |  |  |  |  |  |  |  |  |  |  |  |  |  |  |  |  |  |  |  |  |  |  |  |  |  | C33F10.8 |  |
|  |  |  |  |  |  |  |  |  |  |  |  |  |  |  |  |  |  |  |  |  |  |  |  |  |  |  |  |  |  |  |  |  |  |  |  |  |  | D2013.3 |  |
|  |  |  |  |  |  |  |  |  |  |  |  |  |  |  |  |  |  |  |  |  |  |  |  |  |  |  |  |  |  |  |  |  |  |  |  |  |  | Y111B2A.25 |  |
|  |  |  |  |  |  |  |  |  |  |  |  |  |  |  |  |  |  |  |  |  |  |  |  |  |  |  |  |  |  |  |  |  |  |  |  |  |  | *mre-11* | yeast MRE recombination/repair homolog |
|  |  |  |  |  |  |  |  |  |  |  |  |  |  |  |  |  |  |  |  |  |  |  |  |  |  |  |  |  |  |  |  |  |  |  |  |  |  | T28A8.5 |  |
|  |  |  |  |  |  |  |  |  |  |  |  |  |  |  |  |  |  |  |  |  |  |  |  |  |  |  |  |  |  |  |  |  |  |  |  |  |  | *mrpl-46* | Mitochondrial Ribosomal Protein, Large |
|  |  |  |  |  |  |  |  |  |  |  |  |  |  |  |  |  |  |  |  |  |  |  |  |  |  |  |  |  |  |  |  |  |  |  |  |  |  | Y25C1A.8 |  |
|  |  |  |  |  |  |  |  |  |  |  |  |  |  |  |  |  |  |  |  |  |  |  |  |  |  |  |  |  |  |  |  |  |  |  |  |  |  | F10C2.5 |  |
|  |  |  |  |  |  |  |  |  |  |  |  |  |  |  |  |  |  |  |  |  |  |  |  |  |  |  |  |  |  |  |  |  |  |  |  |  |  | Y48G10A.4 |  |
|  |  |  |  |  |  |  |  |  |  |  |  |  |  |  |  |  |  |  |  |  |  |  |  |  |  |  |  |  |  |  |  |  |  |  |  |  |  | H35B03.2 |  |
|  |  |  |  |  |  |  |  |  |  |  |  |  |  |  |  |  |  |  |  |  |  |  |  |  |  |  |  |  |  |  |  |  |  |  |  |  |  | T10B11.7 |  |
|  |  |  |  |  |  |  |  |  |  |  |  |  |  |  |  |  |  |  |  |  |  |  |  |  |  |  |  |  |  |  |  |  |  |  |  |  |  | *dnj-17* | DNaJ domain (prokaryotic heat shock protein) |
|  |  |  |  |  |  |  |  |  |  |  |  |  |  |  |  |  |  |  |  |  |  |  |  |  |  |  |  |  |  |  |  |  |  |  |  |  |  | C34B2.6 |  |
|  |  |  |  |  |  |  |  |  |  |  |  |  |  |  |  |  |  |  |  |  |  |  |  |  |  |  |  |  |  |  |  |  |  |  |  |  |  | R05D3.1 |  |
|  |  |  |  |  |  |  |  |  |  |  |  |  |  |  |  |  |  |  |  |  |  |  |  |  |  |  |  |  |  |  |  |  |  |  |  |  |  | T01B7.5 |  |
|  |  |  |  |  |  |  |  |  |  |  |  |  |  |  |  |  |  |  |  |  |  |  |  |  |  |  |  |  |  |  |  |  |  |  |  |  |  | ZK512.2 |  |
|  |  |  |  |  |  |  |  |  |  |  |  |  |  |  |  |  |  |  |  |  |  |  |  |  |  |  |  |  |  |  |  |  |  |  |  |  |  | *mans-1* | MAnnoSidase (family 47 glycohydrolase) |
|  |  |  |  |  |  |  |  |  |  |  |  |  |  |  |  |  |  |  |  |  |  |  |  |  |  |  |  |  |  |  |  |  |  |  |  |  |  | *smgl-1* | SMG-associated and Lethal |
|  |  |  |  |  |  |  |  |  |  |  |  |  |  |  |  |  |  |  |  |  |  |  |  |  |  |  |  |  |  |  |  |  |  |  |  |  |  | *cdc-48.3* | Cell Division Cycle related |
|  |  |  |  |  |  |  |  |  |  |  |  |  |  |  |  |  |  |  |  |  |  |  |  |  |  |  |  |  |  |  |  |  |  |  |  |  |  | *pash-1* | PArtner of DroSHa (DRSH-1 interactor) |
|  |  |  |  |  |  |  |  |  |  |  |  |  |  |  |  |  |  |  |  |  |  |  |  |  |  |  |  |  |  |  |  |  |  |  |  |  |  | ZK686.2 |  |
|  |  |  |  |  |  |  |  |  |  |  |  |  |  |  |  |  |  |  |  |  |  |  |  |  |  |  |  |  |  |  |  |  |  |  |  |  |  | F25B5.6 |  |
|  |  |  |  |  |  |  |  |  |  |  |  |  |  |  |  |  |  |  |  |  |  |  |  |  |  |  |  |  |  |  |  |  |  |  |  |  |  | F36A2.9 |  |
|  |  |  |  |  |  |  |  |  |  |  |  |  |  |  |  |  |  |  |  |  |  |  |  |  |  |  |  |  |  |  |  |  |  |  |  |  |  | *prx-10* | PeRoXisome assembly factor |
|  |  |  |  |  |  |  |  |  |  |  |  |  |  |  |  |  |  |  |  |  |  |  |  |  |  |  |  |  |  |  |  |  |  |  |  |  |  | *tag-345* | Temporarily Assigned Gene name |
|  |  |  |  |  |  |  |  |  |  |  |  |  |  |  |  |  |  |  |  |  |  |  |  |  |  |  |  |  |  |  |  |  |  |  |  |  |  | C05C8.2 |  |
|  |  |  |  |  |  |  |  |  |  |  |  |  |  |  |  |  |  |  |  |  |  |  |  |  |  |  |  |  |  |  |  |  |  |  |  |  |  | *tag-124* | Temporarily Assigned Gene name |
|  |  |  |  |  |  |  |  |  |  |  |  |  |  |  |  |  |  |  |  |  |  |  |  |  |  |  |  |  |  |  |  |  |  |  |  |  |  | Y44F5A.1 |  |
|  |  |  |  |  |  |  |  |  |  |  |  |  |  |  |  |  |  |  |  |  |  |  |  |  |  |  |  |  |  |  |  |  |  |  |  |  |  | *rpoa-2* | RNA POlymerase I (A) subunit |
|  |  |  |  |  |  |  |  |  |  |  |  |  |  |  |  |  |  |  |  |  |  |  |  |  |  |  |  |  |  |  |  |  |  |  |  |  |  | *cra-1* | Central Region Assembly in meiosis abnormal |
|  |  |  |  |  |  |  |  |  |  |  |  |  |  |  |  |  |  |  |  |  |  |  |  |  |  |  |  |  |  |  |  |  |  |  |  |  |  | T11B7.1 |  |
|  |  |  |  |  |  |  |  |  |  |  |  |  |  |  |  |  |  |  |  |  |  |  |  |  |  |  |  |  |  |  |  |  |  |  |  |  |  | T09B4.8 |  |
|  |  |  |  |  |  |  |  |  |  |  |  |  |  |  |  |  |  |  |  |  |  |  |  |  |  |  |  |  |  |  |  |  |  |  |  |  |  | F36H5.8 |  |
|  |  |  |  |  |  |  |  |  |  |  |  |  |  |  |  |  |  |  |  |  |  |  |  |  |  |  |  |  |  |  |  |  |  |  |  |  |  | *ent-2* | Equilibrative Nucleoside Transporter |
|  |  |  |  |  |  |  |  |  |  |  |  |  |  |  |  |  |  |  |  |  |  |  |  |  |  |  |  |  |  |  |  |  |  |  |  |  |  | Y6D1A.1 |  |
|  |  |  |  |  |  |  |  |  |  |  |  |  |  |  |  |  |  |  |  |  |  |  |  |  |  |  |  |  |  |  |  |  |  |  |  |  |  | T12G3.2 |  |
|  |  |  |  |  |  |  |  |  |  |  |  |  |  |  |  |  |  |  |  |  |  |  |  |  |  |  |  |  |  |  |  |  |  |  |  |  |  | F33A8.4 |  |
|  |  |  |  |  |  |  |  |  |  |  |  |  |  |  |  |  |  |  |  |  |  |  |  |  |  |  |  |  |  |  |  |  |  |  |  |  |  | JC8.2 |  |
|  |  |  |  |  |  |  |  |  |  |  |  |  |  |  |  |  |  |  |  |  |  |  |  |  |  |  |  |  |  |  |  |  |  |  |  |  |  | C18A11.4 |  |
|  |  |  |  |  |  |  |  |  |  |  |  |  |  |  |  |  |  |  |  |  |  |  |  |  |  |  |  |  |  |  |  |  |  |  |  |  |  | F27D4.4 |  |
|  |  |  |  |  |  |  |  |  |  |  |  |  |  |  |  |  |  |  |  |  |  |  |  |  |  |  |  |  |  |  |  |  |  |  |  |  |  | Y54H5A.1 |  |
|  |  |  |  |  |  |  |  |  |  |  |  |  |  |  |  |  |  |  |  |  |  |  |  |  |  |  |  |  |  |  |  |  |  |  |  |  |  | R07B7.2 |  |
|  |  |  |  |  |  |  |  |  |  |  |  |  |  |  |  |  |  |  |  |  |  |  |  |  |  |  |  |  |  |  |  |  |  |  |  |  |  | Y54E5B.2 |  |
|  |  |  |  |  |  |  |  |  |  |  |  |  |  |  |  |  |  |  |  |  |  |  |  |  |  |  |  |  |  |  |  |  |  |  |  |  |  | *rha-1* | RNA HelicAse |
|  |  |  |  |  |  |  |  |  |  |  |  |  |  |  |  |  |  |  |  |  |  |  |  |  |  |  |  |  |  |  |  |  |  |  |  |  |  | Y22D7AL.7 |  |
|  |  |  |  |  |  |  |  |  |  |  |  |  |  |  |  |  |  |  |  |  |  |  |  |  |  |  |  |  |  |  |  |  |  |  |  |  |  | *faah-2* | Fatty Acid Amide Hydrolase homolog |
|  |  |  |  |  |  |  |  |  |  |  |  |  |  |  |  |  |  |  |  |  |  |  |  |  |  |  |  |  |  |  |  |  |  |  |  |  |  | F33H2.2 |  |
|  |  |  |  |  |  |  |  |  |  |  |  |  |  |  |  |  |  |  |  |  |  |  |  |  |  |  |  |  |  |  |  |  |  |  |  |  |  | *smg-8* | Suppressor with Morphological effect on Genitalia |
|  |  |  |  |  |  |  |  |  |  |  |  |  |  |  |  |  |  |  |  |  |  |  |  |  |  |  |  |  |  |  |  |  |  |  |  |  |  | *zeel-1* | Zygotic Epistatic Embryonic Lethal |
|  |  |  |  |  |  |  |  |  |  |  |  |  |  |  |  |  |  |  |  |  |  |  |  |  |  |  |  |  |  |  |  |  |  |  |  |  |  | *gly-10* | GLYcosylation related |
|  |  |  |  |  |  |  |  |  |  |  |  |  |  |  |  |  |  |  |  |  |  |  |  |  |  |  |  |  |  |  |  |  |  |  |  |  |  | C44B7.11 |  |
|  |  |  |  |  |  |  |  |  |  |  |  |  |  |  |  |  |  |  |  |  |  |  |  |  |  |  |  |  |  |  |  |  |  |  |  |  |  | ZC373.5 |  |
|  |  |  |  |  |  |  |  |  |  |  |  |  |  |  |  |  |  |  |  |  |  |  |  |  |  |  |  |  |  |  |  |  |  |  |  |  |  | C25G4.3 |  |
|  |  |  |  |  |  |  |  |  |  |  |  |  |  |  |  |  |  |  |  |  |  |  |  |  |  |  |  |  |  |  |  |  |  |  |  |  |  | *eya-1* | EYA (Drosophila eyes absent) homolog |
|  |  |  |  |  |  |  |  |  |  |  |  |  |  |  |  |  |  |  |  |  |  |  |  |  |  |  |  |  |  |  |  |  |  |  |  |  |  | *best-11* | BESTrophin (chloride channel) homolog |
|  |  |  |  |  |  |  |  |  |  |  |  |  |  |  |  |  |  |  |  |  |  |  |  |  |  |  |  |  |  |  |  |  |  |  |  |  |  | *btb-1* | BTB (Broad/complex/Tramtrack/Bric a brac) domain protein |
|  |  |  |  |  |  |  |  |  |  |  |  |  |  |  |  |  |  |  |  |  |  |  |  |  |  |  |  |  |  |  |  |  |  |  |  |  |  | *hosl-1* | HOrmone-Sensitive Lipase homolog |
|  |  |  |  |  |  |  |  |  |  |  |  |  |  |  |  |  |  |  |  |  |  |  |  |  |  |  |  |  |  |  |  |  |  |  |  |  |  | M04F3.3 |  |
|  |  |  |  |  |  |  |  |  |  |  |  |  |  |  |  |  |  |  |  |  |  |  |  |  |  |  |  |  |  |  |  |  |  |  |  |  |  | F26E4.5 |  |
|  |  |  |  |  |  |  |  |  |  |  |  |  |  |  |  |  |  |  |  |  |  |  |  |  |  |  |  |  |  |  |  |  |  |  |  |  |  | C49A9.2 |  |
|  |  |  |  |  |  |  |  |  |  |  |  |  |  |  |  |  |  |  |  |  |  |  |  |  |  |  |  |  |  |  |  |  |  |  |  |  |  | C28G1.4 |  |
|  |  |  |  |  |  |  |  |  |  |  |  |  |  |  |  |  |  |  |  |  |  |  |  |  |  |  |  |  |  |  |  |  |  |  |  |  |  | *lips-17* | LIPaSe related |
|  |  |  |  |  |  |  |  |  |  |  |  |  |  |  |  |  |  |  |  |  |  |  |  |  |  |  |  |  |  |  |  |  |  |  |  |  |  | F41C6.6 |  |
|  |  |  |  |  |  |  |  |  |  |  |  |  |  |  |  |  |  |  |  |  |  |  |  |  |  |  |  |  |  |  |  |  |  |  |  |  |  | Y49G5B.6 |  |
|  |  |  |  |  |  |  |  |  |  |  |  |  |  |  |  |  |  |  |  |  |  |  |  |  |  |  |  |  |  |  |  |  |  |  |  |  |  | *zyg-9* | ZYGote defective : embryonic lethal |
|  |  |  |  |  |  |  |  |  |  |  |  |  |  |  |  |  |  |  |  |  |  |  |  |  |  |  |  |  |  |  |  |  |  |  |  |  |  | T08G5.2 |  |
|  |  |  |  |  |  |  |  |  |  |  |  |  |  |  |  |  |  |  |  |  |  |  |  |  |  |  |  |  |  |  |  |  |  |  |  |  |  | Y71A12B.19 |  |
|  |  |  |  |  |  |  |  |  |  |  |  |  |  |  |  |  |  |  |  |  |  |  |  |  |  |  |  |  |  |  |  |  |  |  |  |  |  | R03D7.3 |  |
|  |  |  |  |  |  |  |  |  |  |  |  |  |  |  |  |  |  |  |  |  |  |  |  |  |  |  |  |  |  |  |  |  |  |  |  |  |  | T28A8.8 |  |
|  |  |  |  |  |  |  |  |  |  |  |  |  |  |  |  |  |  |  |  |  |  |  |  |  |  |  |  |  |  |  |  |  |  |  |  |  |  | R03D7.2 |  |
|  |  |  |  |  |  |  |  |  |  |  |  |  |  |  |  |  |  |  |  |  |  |  |  |  |  |  |  |  |  |  |  |  |  |  |  |  |  | *coh-4* | COHesin family |
|  |  |  |  |  |  |  |  |  |  |  |  |  |  |  |  |  |  |  |  |  |  |  |  |  |  |  |  |  |  |  |  |  |  |  |  |  |  | *gei-14* | GEX Interacting protein |
|  |  |  |  |  |  |  |  |  |  |  |  |  |  |  |  |  |  |  |  |  |  |  |  |  |  |  |  |  |  |  |  |  |  |  |  |  |  | C06A1.4 |  |
|  |  |  |  |  |  |  |  |  |  |  |  |  |  |  |  |  |  |  |  |  |  |  |  |  |  |  |  |  |  |  |  |  |  |  |  |  |  | *fbxc-42* | F-box C protein |
|  |  |  |  |  |  |  |  |  |  |  |  |  |  |  |  |  |  |  |  |  |  |  |  |  |  |  |  |  |  |  |  |  |  |  |  |  |  | Y54F10AM.11 |  |
|  |  |  |  |  |  |  |  |  |  |  |  |  |  |  |  |  |  |  |  |  |  |  |  |  |  |  |  |  |  |  |  |  |  |  |  |  |  | *npl-4.2* | NPL (yeast Nuclear Protein Localization) homolog |
|  |  |  |  |  |  |  |  |  |  |  |  |  |  |  |  |  |  |  |  |  |  |  |  |  |  |  |  |  |  |  |  |  |  |  |  |  |  | *sars-2* | Seryl Amino-acyl tRNA Synthetase |
|  |  |  |  |  |  |  |  |  |  |  |  |  |  |  |  |  |  |  |  |  |  |  |  |  |  |  |  |  |  |  |  |  |  |  |  |  |  | *ife-5* | Initiation Factor 4E (eIF4E) family |
|  |  |  |  |  |  |  |  |  |  |  |  |  |  |  |  |  |  |  |  |  |  |  |  |  |  |  |  |  |  |  |  |  |  |  |  |  |  | Y56A3A.16 |  |
|  |  |  |  |  |  |  |  |  |  |  |  |  |  |  |  |  |  |  |  |  |  |  |  |  |  |  |  |  |  |  |  |  |  |  |  |  |  | *gla-3* | Germ Line Apoptosis abnormal |
|  |  |  |  |  |  |  |  |  |  |  |  |  |  |  |  |  |  |  |  |  |  |  |  |  |  |  |  |  |  |  |  |  |  |  |  |  |  | *hpr-9* | Homolog of S. Pombe Rad |
|  |  |  |  |  |  |  |  |  |  |  |  |  |  |  |  |  |  |  |  |  |  |  |  |  |  |  |  |  |  |  |  |  |  |  |  |  |  | *sdha-2* | Succinate DeHydrogenase complex subunit A |
|  |  |  |  |  |  |  |  |  |  |  |  |  |  |  |  |  |  |  |  |  |  |  |  |  |  |  |  |  |  |  |  |  |  |  |  |  |  | F54E12.2 |  |
|  |  |  |  |  |  |  |  |  |  |  |  |  |  |  |  |  |  |  |  |  |  |  |  |  |  |  |  |  |  |  |  |  |  |  |  |  |  | *tag-63* | Temporarily Assigned Gene name |
|  |  |  |  |  |  |  |  |  |  |  |  |  |  |  |  |  |  |  |  |  |  |  |  |  |  |  |  |  |  |  |  |  |  |  |  |  |  | *msh-6* | MSH (MutS Homolog) family |
|  |  |  |  |  |  |  |  |  |  |  |  |  |  |  |  |  |  |  |  |  |  |  |  |  |  |  |  |  |  |  |  |  |  |  |  |  |  | D1043.1 |  |
|  |  |  |  |  |  |  |  |  |  |  |  |  |  |  |  |  |  |  |  |  |  |  |  |  |  |  |  |  |  |  |  |  |  |  |  |  |  | *czw-1* | Caenorhabditis Zeste White 10 (Drosophila) homolog |
|  |  |  |  |  |  |  |  |  |  |  |  |  |  |  |  |  |  |  |  |  |  |  |  |  |  |  |  |  |  |  |  |  |  |  |  |  |  | *ztf-20* | Zinc finger putative Transcription Factor family |
|  |  |  |  |  |  |  |  |  |  |  |  |  |  |  |  |  |  |  |  |  |  |  |  |  |  |  |  |  |  |  |  |  |  |  |  |  |  | F22G12.3 |  |
|  |  |  |  |  |  |  |  |  |  |  |  |  |  |  |  |  |  |  |  |  |  |  |  |  |  |  |  |  |  |  |  |  |  |  |  |  |  | C33E10.1 |  |
|  |  |  |  |  |  |  |  |  |  |  |  |  |  |  |  |  |  |  |  |  |  |  |  |  |  |  |  |  |  |  |  |  |  |  |  |  |  | F08D12.1 |  |
|  |  |  |  |  |  |  |  |  |  |  |  |  |  |  |  |  |  |  |  |  |  |  |  |  |  |  |  |  |  |  |  |  |  |  |  |  |  | Y57A10A.31 |  |
|  |  |  |  |  |  |  |  |  |  |  |  |  |  |  |  |  |  |  |  |  |  |  |  |  |  |  |  |  |  |  |  |  |  |  |  |  |  | Y50D4C.3 |  |
|  |  |  |  |  |  |  |  |  |  |  |  |  |  |  |  |  |  |  |  |  |  |  |  |  |  |  |  |  |  |  |  |  |  |  |  |  |  | *pfs-2* | Polyadenylation Factor Subunit homolog |
|  |  |  |  |  |  |  |  |  |  |  |  |  |  |  |  |  |  |  |  |  |  |  |  |  |  |  |  |  |  |  |  |  |  |  |  |  |  | Y66D12A.15 |  |
|  |  |  |  |  |  |  |  |  |  |  |  |  |  |  |  |  |  |  |  |  |  |  |  |  |  |  |  |  |  |  |  |  |  |  |  |  |  | H24K24.4 |  |
|  |  |  |  |  |  |  |  |  |  |  |  |  |  |  |  |  |  |  |  |  |  |  |  |  |  |  |  |  |  |  |  |  |  |  |  |  |  | E01B7.1 |  |
|  |  |  |  |  |  |  |  |  |  |  |  |  |  |  |  |  |  |  |  |  |  |  |  |  |  |  |  |  |  |  |  |  |  |  |  |  |  | C25G6.3 |  |
|  |  |  |  |  |  |  |  |  |  |  |  |  |  |  |  |  |  |  |  |  |  |  |  |  |  |  |  |  |  |  |  |  |  |  |  |  |  | C24D10.2 |  |
|  |  |  |  |  |  |  |  |  |  |  |  |  |  |  |  |  |  |  |  |  |  |  |  |  |  |  |  |  |  |  |  |  |  |  |  |  |  | T08D2.8 |  |
|  |  |  |  |  |  |  |  |  |  |  |  |  |  |  |  |  |  |  |  |  |  |  |  |  |  |  |  |  |  |  |  |  |  |  |  |  |  | C34C6.2 |  |
|  |  |  |  |  |  |  |  |  |  |  |  |  |  |  |  |  |  |  |  |  |  |  |  |  |  |  |  |  |  |  |  |  |  |  |  |  |  | T21B10.4 |  |
|  |  |  |  |  |  |  |  |  |  |  |  |  |  |  |  |  |  |  |  |  |  |  |  |  |  |  |  |  |  |  |  |  |  |  |  |  |  | R144.5 |  |
|  |  |  |  |  |  |  |  |  |  |  |  |  |  |  |  |  |  |  |  |  |  |  |  |  |  |  |  |  |  |  |  |  |  |  |  |  |  | T06D4.1 |  |
|  |  |  |  |  |  |  |  |  |  |  |  |  |  |  |  |  |  |  |  |  |  |  |  |  |  |  |  |  |  |  |  |  |  |  |  |  |  | C38D4.1 |  |
|  |  |  |  |  |  |  |  |  |  |  |  |  |  |  |  |  |  |  |  |  |  |  |  |  |  |  |  |  |  |  |  |  |  |  |  |  |  | Y20F4.5 |  |
|  |  |  |  |  |  |  |  |  |  |  |  |  |  |  |  |  |  |  |  |  |  |  |  |  |  |  |  |  |  |  |  |  |  |  |  |  |  | Y49F6C.6 |  |
|  |  |  |  |  |  |  |  |  |  |  |  |  |  |  |  |  |  |  |  |  |  |  |  |  |  |  |  |  |  |  |  |  |  |  |  |  |  | *epg-5* | Ectopic P Granules |
|  |  |  |  |  |  |  |  |  |  |  |  |  |  |  |  |  |  |  |  |  |  |  |  |  |  |  |  |  |  |  |  |  |  |  |  |  |  | W09D6.1 |  |
|  |  |  |  |  |  |  |  |  |  |  |  |  |  |  |  |  |  |  |  |  |  |  |  |  |  |  |  |  |  |  |  |  |  |  |  |  |  | R12E2.1 |  |
|  |  |  |  |  |  |  |  |  |  |  |  |  |  |  |  |  |  |  |  |  |  |  |  |  |  |  |  |  |  |  |  |  |  |  |  |  |  | *tbc-14* | TBC (Tre-2/Bub2/Cdc16) domain family |
|  |  |  |  |  |  |  |  |  |  |  |  |  |  |  |  |  |  |  |  |  |  |  |  |  |  |  |  |  |  |  |  |  |  |  |  |  |  | *ctps-1* | CTP Synthase |
|  |  |  |  |  |  |  |  |  |  |  |  |  |  |  |  |  |  |  |  |  |  |  |  |  |  |  |  |  |  |  |  |  |  |  |  |  |  | *cogc-1* | Conserved Oligomeric Golgi (COG) Component |
|  |  |  |  |  |  |  |  |  |  |  |  |  |  |  |  |  |  |  |  |  |  |  |  |  |  |  |  |  |  |  |  |  |  |  |  |  |  | *rsks-1* | RSK-pSeventy (RSK-p70 kinase) homolog |
|  |  |  |  |  |  |  |  |  |  |  |  |  |  |  |  |  |  |  |  |  |  |  |  |  |  |  |  |  |  |  |  |  |  |  |  |  |  | *rle-1* | Regulation of Longevity by E3 ubiquitin ligase |
|  |  |  |  |  |  |  |  |  |  |  |  |  |  |  |  |  |  |  |  |  |  |  |  |  |  |  |  |  |  |  |  |  |  |  |  |  |  | M142.5 |  |
|  |  |  |  |  |  |  |  |  |  |  |  |  |  |  |  |  |  |  |  |  |  |  |  |  |  |  |  |  |  |  |  |  |  |  |  |  |  | *cox-10* | Cytochrome OXidase assembly protein |
|  |  |  |  |  |  |  |  |  |  |  |  |  |  |  |  |  |  |  |  |  |  |  |  |  |  |  |  |  |  |  |  |  |  |  |  |  |  | H05C05.1 |  |
|  |  |  |  |  |  |  |  |  |  |  |  |  |  |  |  |  |  |  |  |  |  |  |  |  |  |  |  |  |  |  |  |  |  |  |  |  |  | Y116A8C.13 |  |
|  |  |  |  |  |  |  |  |  |  |  |  |  |  |  |  |  |  |  |  |  |  |  |  |  |  |  |  |  |  |  |  |  |  |  |  |  |  | F54A3.6 |  |
|  |  |  |  |  |  |  |  |  |  |  |  |  |  |  |  |  |  |  |  |  |  |  |  |  |  |  |  |  |  |  |  |  |  |  |  |  |  | *arid-1* | ARID (AT-rich Interactive Domain-containing protein) homolog |
|  |  |  |  |  |  |  |  |  |  |  |  |  |  |  |  |  |  |  |  |  |  |  |  |  |  |  |  |  |  |  |  |  |  |  |  |  |  | Y95B8A.8 |  |
|  |  |  |  |  |  |  |  |  |  |  |  |  |  |  |  |  |  |  |  |  |  |  |  |  |  |  |  |  |  |  |  |  |  |  |  |  |  | *gck-3* | Germinal Center Kinase family |
|  |  |  |  |  |  |  |  |  |  |  |  |  |  |  |  |  |  |  |  |  |  |  |  |  |  |  |  |  |  |  |  |  |  |  |  |  |  | *gpa-16* | G Protein, Alpha subunit |
|  |  |  |  |  |  |  |  |  |  |  |  |  |  |  |  |  |  |  |  |  |  |  |  |  |  |  |  |  |  |  |  |  |  |  |  |  |  | Y95B8A.7 |  |
|  |  |  |  |  |  |  |  |  |  |  |  |  |  |  |  |  |  |  |  |  |  |  |  |  |  |  |  |  |  |  |  |  |  |  |  |  |  | *lin-38* | abnormal cell LINeage |
|  |  |  |  |  |  |  |  |  |  |  |  |  |  |  |  |  |  |  |  |  |  |  |  |  |  |  |  |  |  |  |  |  |  |  |  |  |  | *ssl-1* | yeast Swi2/Snf2-Like |
|  |  |  |  |  |  |  |  |  |  |  |  |  |  |  |  |  |  |  |  |  |  |  |  |  |  |  |  |  |  |  |  |  |  |  |  |  |  | Y71G10AL.1 |  |
|  |  |  |  |  |  |  |  |  |  |  |  |  |  |  |  |  |  |  |  |  |  |  |  |  |  |  |  |  |  |  |  |  |  |  |  |  |  | B0511.14 |  |
|  |  |  |  |  |  |  |  |  |  |  |  |  |  |  |  |  |  |  |  |  |  |  |  |  |  |  |  |  |  |  |  |  |  |  |  |  |  | F32B4.4 |  |
|  |  |  |  |  |  |  |  |  |  |  |  |  |  |  |  |  |  |  |  |  |  |  |  |  |  |  |  |  |  |  |  |  |  |  |  |  |  | Y75B8A.7 |  |
|  |  |  |  |  |  |  |  |  |  |  |  |  |  |  |  |  |  |  |  |  |  |  |  |  |  |  |  |  |  |  |  |  |  |  |  |  |  | *aakb-2* | AMP-Activated Kinase Beta subunit |
|  |  |  |  |  |  |  |  |  |  |  |  |  |  |  |  |  |  |  |  |  |  |  |  |  |  |  |  |  |  |  |  |  |  |  |  |  |  | *egrh-3* | EGR (Early Growth factor Response factor) Homolog |
|  |  |  |  |  |  |  |  |  |  |  |  |  |  |  |  |  |  |  |  |  |  |  |  |  |  |  |  |  |  |  |  |  |  |  |  |  |  | *ncbp-2* | Nuclear Cap Binding Protein |
|  |  |  |  |  |  |  |  |  |  |  |  |  |  |  |  |  |  |  |  |  |  |  |  |  |  |  |  |  |  |  |  |  |  |  |  |  |  | *cyh-1* | CYclin H |
|  |  |  |  |  |  |  |  |  |  |  |  |  |  |  |  |  |  |  |  |  |  |  |  |  |  |  |  |  |  |  |  |  |  |  |  |  |  | *xrn-1* | XRN (mouse/S. cerevisiae) ribonuclease related |
|  |  |  |  |  |  |  |  |  |  |  |  |  |  |  |  |  |  |  |  |  |  |  |  |  |  |  |  |  |  |  |  |  |  |  |  |  |  | *xpc-1* | XPC (Xeroderma Pigmentosum group C) DNA repair gene homolog |
|  |  |  |  |  |  |  |  |  |  |  |  |  |  |  |  |  |  |  |  |  |  |  |  |  |  |  |  |  |  |  |  |  |  |  |  |  |  | *pqn-20* | Prion-like-(Q/N-rich)-domain-bearing protein |
|  |  |  |  |  |  |  |  |  |  |  |  |  |  |  |  |  |  |  |  |  |  |  |  |  |  |  |  |  |  |  |  |  |  |  |  |  |  | M03C11.3 |  |
|  |  |  |  |  |  |  |  |  |  |  |  |  |  |  |  |  |  |  |  |  |  |  |  |  |  |  |  |  |  |  |  |  |  |  |  |  |  | Y49E10.26 |  |
|  |  |  |  |  |  |  |  |  |  |  |  |  |  |  |  |  |  |  |  |  |  |  |  |  |  |  |  |  |  |  |  |  |  |  |  |  |  | *atg-9* | AuTophaGy (yeast Atg homolog) |
|  |  |  |  |  |  |  |  |  |  |  |  |  |  |  |  |  |  |  |  |  |  |  |  |  |  |  |  |  |  |  |  |  |  |  |  |  |  | *aakg-1* | AMP-Activated protein Kinase Gamma subunit |
|  |  |  |  |  |  |  |  |  |  |  |  |  |  |  |  |  |  |  |  |  |  |  |  |  |  |  |  |  |  |  |  |  |  |  |  |  |  | *tsg-101* | Tumor Susceptibility Gene homolog |
|  |  |  |  |  |  |  |  |  |  |  |  |  |  |  |  |  |  |  |  |  |  |  |  |  |  |  |  |  |  |  |  |  |  |  |  |  |  | *adpr-1* | ADiPose (Drosophila obesity gene) Related |
|  |  |  |  |  |  |  |  |  |  |  |  |  |  |  |  |  |  |  |  |  |  |  |  |  |  |  |  |  |  |  |  |  |  |  |  |  |  | *denn-4* | DENN domain type RAB GEF |
|  |  |  |  |  |  |  |  |  |  |  |  |  |  |  |  |  |  |  |  |  |  |  |  |  |  |  |  |  |  |  |  |  |  |  |  |  |  | *grdn-1* | GiRDiN (mammalian actin-binding protein) homolog |
|  |  |  |  |  |  |  |  |  |  |  |  |  |  |  |  |  |  |  |  |  |  |  |  |  |  |  |  |  |  |  |  |  |  |  |  |  |  | Y24F12A.3 |  |
|  |  |  |  |  |  |  |  |  |  |  |  |  |  |  |  |  |  |  |  |  |  |  |  |  |  |  |  |  |  |  |  |  |  |  |  |  |  | Y9D1A.2 |  |
|  |  |  |  |  |  |  |  |  |  |  |  |  |  |  |  |  |  |  |  |  |  |  |  |  |  |  |  |  |  |  |  |  |  |  |  |  |  | *col-99* | COLlagen |
|  |  |  |  |  |  |  |  |  |  |  |  |  |  |  |  |  |  |  |  |  |  |  |  |  |  |  |  |  |  |  |  |  |  |  |  |  |  | H20J04.4 |  |
|  |  |  |  |  |  |  |  |  |  |  |  |  |  |  |  |  |  |  |  |  |  |  |  |  |  |  |  |  |  |  |  |  |  |  |  |  |  | *ell-1* | ELL transcription elongation factor homolog |
|  |  |  |  |  |  |  |  |  |  |  |  |  |  |  |  |  |  |  |  |  |  |  |  |  |  |  |  |  |  |  |  |  |  |  |  |  |  | *pro-3* | PROximal proliferation in germline |
|  |  |  |  |  |  |  |  |  |  |  |  |  |  |  |  |  |  |  |  |  |  |  |  |  |  |  |  |  |  |  |  |  |  |  |  |  |  | *taf-3* | TAF (TBP-associated transcription factor) family |
|  |  |  |  |  |  |  |  |  |  |  |  |  |  |  |  |  |  |  |  |  |  |  |  |  |  |  |  |  |  |  |  |  |  |  |  |  |  | *set-26* | SET (trithorax/polycomb) domain containing |
|  |  |  |  |  |  |  |  |  |  |  |  |  |  |  |  |  |  |  |  |  |  |  |  |  |  |  |  |  |  |  |  |  |  |  |  |  |  | Y50D4C.5 |  |

### Phenotypes enriched

|  |  |  |  |
| --- | --- | --- | --- |
| **Group name** | **Number in cluster** | **Enrichment** | **FDR corrected p** |
| gene expression variant (RNAi) | 95 | 1.93 | 5.79e-07 |
| organ system development variant (RNAi) | 60 | 2.38 | 1.26e-06 |
| cell development variant (RNAi) | 71 | 2.13 | 2.91e-06 |
| transgene expression variant (RNAi) | 84 | 1.89 | 2.10e-05 |
| fertility variant | 81 | 1.85 | 8.43e-05 |
| reproductive system physiology variant | 81 | 1.85 | 8.92e-05 |
| organ system physiology variant | 82 | 1.83 | 1.08e-04 |
| sterile | 75 | 1.88 | 1.68e-04 |
| fertility reduced | 75 | 1.86 | 2.15e-04 |
| cell physiology variant (RNAi) | 89 | 1.73 | 2.91e-04 |
| development variant (RNAi) | 190 | 1.36 | 6.96e-04 |
| epithelial system development variant (RNAi) | 30 | 2.82 | 6.96e-04 |
| pattern of transgene expression variant (RNAi) | 67 | 1.87 | 8.12e-04 |
| oocyte physiology variant (RNAi) | 49 | 2.14 | 8.47e-04 |
| endocytic transport variant (RNAi) | 50 | 2.11 | 9.72e-04 |
| mRNA surveillance defective (RNAi) | 4 | 33.23 | 1.45e-03 |
| exploded through vulva (RNAi) | 29 | 2.76 | 1.47e-03 |
| receptor mediated endocytosis defective (RNAi) | 48 | 2.11 | 1.53e-03 |
| endocytic transport defect (RNAi) | 49 | 2.07 | 1.81e-03 |
| larval lethal (RNAi) | 53 | 1.92 | 5.57e-03 |
| organism development variant (RNAi) | 175 | 1.34 | 5.75e-03 |
| level of transgene expression variant (RNAi) | 32 | 2.42 | 5.75e-03 |
| organ system morphology variant (RNAi) | 65 | 1.77 | 6.22e-03 |
| RNA expression variant (RNAi) | 10 | 6.04 | 6.89e-03 |
| hermaphrodite fertility variant (RNAi) | 91 | 1.58 | 6.89e-03 |
| organism metabolism processing variant (RNAi) | 105 | 1.52 | 7.37e-03 |
| RNA processing variant (RNAi) | 5 | 16.61 | 7.61e-03 |
| protruding vulva (RNAi) | 47 | 1.99 | 7.87e-03 |
| vulva morphology variant (RNAi) | 48 | 1.95 | 9.36e-03 |
| hermaphrodite reproductive system morphology variant (RNAi) | 48 | 1.95 | 9.88e-03 |
| slow growth (RNAi) | 96 | 1.54 | 1.11e-02 |
| reproductive system morphology variant (RNAi) | 50 | 1.91 | 1.16e-02 |
| fertility variant (RNAi) | 112 | 1.47 | 1.35e-02 |
| growth variant (RNAi) | 133 | 1.40 | 1.49e-02 |
| reproductive system physiology variant (RNAi) | 112 | 1.46 | 1.68e-02 |
| organ system physiology variant (RNAi) | 112 | 1.45 | 1.88e-02 |
| morphology variant (RNAi) | 104 | 1.48 | 1.89e-02 |
| larval arrest (RNAi) | 84 | 1.57 | 1.90e-02 |
| developmental growth variant (RNAi) | 84 | 1.57 | 1.92e-02 |
| larval growth variant (RNAi) | 84 | 1.57 | 1.96e-02 |
| late larval arrest (RNAi) | 12 | 4.38 | 2.07e-02 |
| cell morphology variant (RNAi) | 44 | 1.93 | 2.52e-02 |
| postembryonic development variant (RNAi) | 92 | 1.51 | 3.04e-02 |
| transgene expression increased (RNAi) | 23 | 2.60 | 3.28e-02 |
| lethal (RNAi) | 150 | 1.33 | 4.27e-02 |
| gametogenesis variant (RNAi) | 34 | 2.09 | 4.47e-02 |
| physiology variant | 104 | 1.45 | 4.65e-02 |

### Anatomy terms enriched

none found

### GO terms enriched

|  |  |  |
| --- | --- | --- |
| **GO term** | **Number of genes** | **FDR-corrected p-value** |
| cell | 140 | 1.3e-10 |
| organelle | 113 | 2.0e-07 |
| nucleotide binding | 64 | 1.8e-06 |
| positive regulation of growth rate | 80 | 3.2e-06 |
| receptor-mediated endocytosis | 50 | 5.4e-06 |
| nucleus | 74 | 9.4e-06 |
| embryo development ending in birth or egg hatching | 128 | 2.7e-05 |
| positive regulation of biological process | 96 | 4.4e-05 |
| nematode larval development | 89 | 5.7e-05 |
| vesicle-mediated transport | 56 | 9.9e-05 |
| post-embryonic development | 91 | 1.0e-04 |
| adenyl nucleotide binding | 45 | 1.4e-04 |
| helicase activity | 13 | 1.4e-04 |
| multicellular organismal process | 163 | 1.5e-04 |
| tissue development | 37 | 1.7e-04 |
| regulation of growth | 85 | 1.7e-04 |
| intracellular organelle part | 53 | 2.0e-04 |
| morphogenesis of an epithelium | 32 | 2.2e-04 |
| intracellular part | 32 | 2.3e-04 |
| organelle lumen | 24 | 3.0e-04 |
| RNA binding | 24 | 3.2e-04 |
| nuclear lumen | 21 | 3.5e-04 |
| ATP binding | 43 | 3.6e-04 |
| reproductive structure development | 51 | 4.6e-04 |
| reproduction | 47 | 5.7e-04 |
| heterocyclic compound binding | 95 | 8.3e-04 |
| anatomical structure development | 76 | 8.6e-04 |
| organic cyclic compound binding | 95 | 9.6e-04 |
| cytoplasm | 76 | 1.1e-03 |
| carbohydrate derivative binding | 48 | 2.3e-03 |
| hermaphrodite genitalia development | 43 | 2.3e-03 |
| purine nucleoside binding | 46 | 3.7e-03 |
| ribonucleoside binding | 46 | 3.9e-03 |
| locomotion | 78 | 3.9e-03 |
| purine ribonucleotide binding | 46 | 4.2e-03 |
| macromolecule modification | 41 | 4.5e-03 |
| ncRNA metabolic process | 12 | 8.5e-03 |
| macromolecular complex | 48 | 1.1e-02 |
| sex differentiation | 37 | 1.3e-02 |
| RNA processing | 16 | 1.4e-02 |
| rRNA processing | 7 | 1.5e-02 |
| negative regulation of macromolecule metabolic process | 20 | 1.5e-02 |
| single organism reproductive process | 49 | 1.7e-02 |
| multicellular organismal development | 68 | 1.7e-02 |
| mRNA metabolic process | 12 | 1.8e-02 |
| protein localization | 27 | 2.3e-02 |
| RNA catabolic process | 6 | 2.3e-02 |
| biological regulation | 155 | 2.8e-02 |
| anion binding | 49 | 3.4e-02 |
| ATPase activity | 15 | 3.7e-02 |
| localization of cell | 20 | 3.7e-02 |
| pyrophosphatase activity | 24 | 4.2e-02 |
| hydrolase activity, acting on acid anhydrides | 24 | 4.5e-02 |
| cellular macromolecule metabolic process | 91 | 4.8e-02 |

### Expression clusters enriched

|  |  |  |  |
| --- | --- | --- | --- |
| **Group name** | **Number in cluster** | **Enrichment** | **FDR corrected p** |
| FBF-associated probe sets (FDR <2.25%) | 196 | 1.73 | 1.57e-14 |
| A complete list of the genes that showed differential expression in a slr-2 mutant strain. | 114 | 2.20 | 1.37e-13 |
| Caenorhabditis elegans Genes with expression levels changed significantly after treatment of Xenorhabdus nematophila. | 292 | 1.39 | 1.01e-11 |
| TGF- Dauer pathway adult transcriptional targets. Results obtained by comparing the microarray results of the dauer-constitutive mutants daf-7(e1372), daf-7(m62), and daf-1(m40) with dauer-defective mutants daf-3(mgDf90), daf-5(e1386), and daf-7(e1372);daf-3(mgDf90) double mutants at the permissive temperature, 20C, on the first day of adulthood. WBPaper00031040:TGF-beta\_adult\_downregulated | 196 | 1.55 | 1.20e-09 |
| Embryonic (E) subclasses are based on the earliest significant increase(abbreviated pi for primary increase). [cgc5767]:expression\_class\_E\_pi(23\_min) | 67 | 2.47 | 4.24e-09 |
| Germline-enriched and sex-biased expression profile cluster F. | 56 | 2.63 | 2.21e-08 |
| Maternal-embryonic class (ME): genes that are in the intersection of the maternal and embryonic classes. | 135 | 1.70 | 5.76e-08 |
| Embryonic transient (ET) subclasses are based on time of max abundance. [cgc5767]:expression\_class\_ET\_max(23\_min) | 16 | 7.93 | 6.77e-08 |
| Maternal class (M): genes that are called present in at least one of the three PC6 replicates. | 240 | 1.37 | 3.22e-07 |
| Embryonic class (E): genes that significantly increase in abundance at some point during embryogenesis. | 165 | 1.54 | 3.87e-07 |
| Gene significantly up-regulated by treatment with 2.0mM of HuminFeed until older adult stage (11 days), with a minimum fold change in gene expression of 1.25. | 51 | 2.50 | 8.73e-07 |
| Genes with differeiential expression after exposed to Au-NP. | 54 | 2.34 | 2.69e-06 |
| Significantly downregulated genes from cyc-1(RNAi) microarrays using SAM algorithm with an FDR < 0.1 from adult-only chips. | 147 | 1.46 | 1.01e-04 |
| Maternal-embryonic transient class (MET): genes that are in the intersection of the maternal and embryonic transient classes. | 54 | 2.00 | 3.27e-04 |
| C-lineage related expression profile. WBPaper00025032:cluster\_31 | 9 | 6.80 | 1.73e-03 |
| Genes down-regulated after 300 um Tannic acid treatment. Fold change < 0.8. | 77 | 1.66 | 1.85e-03 |
| Expression Pattern Group B, enriched for genes involved in embryonic development. These patterns have in common that they all have genes of which the expression goes up after the juvenile stage. The expression of the genes in these patterns remains high or even goes up after reproduction. | 72 | 1.69 | 2.20e-03 |
| C-lineage related expression profile. WBPaper00025032:cluster\_30 | 9 | 6.50 | 2.42e-03 |
| Embryonic transient class (ET): genes that are the subset of embryonic genes in which the latest significant increase is earlier than their latest significant decrease. | 67 | 1.69 | 4.23e-03 |
| Genes with significantly increased expression levels only in the lin-35 mutant relative to controls. | 12 | 4.43 | 4.63e-03 |
| Genes differentially expressed under UVC exposure and EtBr treatment vs under EtBr treatment but without UVC exposure at the -45h timepoint (3 hours after the first UVC dose). | 37 | 2.08 | 6.20e-03 |
| Genes predicted to be upregulated more than 2.0 fold in rrf-2(ok210) mutant worms as compared to wild-type animals (t-test P-value < 0.05). | 23 | 2.64 | 6.44e-03 |
| Genes with expression level up in rde-4 mutant background. | 20 | 2.86 | 7.00e-03 |
| Germline-intrinsic transcripts. | 63 | 1.66 | 1.09e-02 |
| C-lineage related expression profile. WBPaper00025032:cluster\_64 | 6 | 8.67 | 1.23e-02 |
| Differentially expressed genes during worm lifespan. Medoid 3 Fig.4. | 17 | 3.04 | 1.25e-02 |
| Genes downregulated more than 2 fold after 24 hours of AgNPs exposure. | 55 | 1.73 | 1.31e-02 |
| Genes that showed increased expression after 24 hours of infection by fungi Drechmeria coniospora. WBPaper00032031:DConiospora\_upregulated\_cDNA\_24h | 28 | 2.24 | 1.55e-02 |
| C-lineage related expression profile. WBPaper00025032:cluster\_7 | 13 | 3.54 | 1.93e-02 |
| Expression Pattern Group H, enriched for genes involved in embryonic development. These patterns have in common that they all have genes of which the expression goes up after the juvenile stage. The expression of the genes in these patterns remains high or even goes up after reproduction. | 51 | 1.73 | 2.21e-02 |
| C-lineage related expression profile. WBPaper00025032:cluster\_53 | 6 | 7.67 | 2.38e-02 |
| Genes that showed decreased expression after treated with 2-deoxy-D-glucose. | 15 | 3.08 | 2.69e-02 |
| C-lineage related expression profile. WBPaper00025032:cluster\_144 | 4 | 13.29 | 3.37e-02 |
| Genes with no change in hcf-1(-), no change in sir-2.1(O/E) and downregulated in daf-2(-). | 40 | 1.84 | 3.62e-02 |
| C-lineage related expression profile. WBPaper00025032:cluster\_36 | 7 | 5.81 | 3.79e-02 |
| Gene significantly down-regulated by treatment with 0.2mM of HuminFeed Hydroquinone until young adult stage (3 days), with a minimum fold change in gene expression of 0.8. | 10 | 3.91 | 4.92e-02 |
| Embryonic transient (ET) subclasses are based on time of max abundance. [cgc5767]:expression\_class\_ET\_max(53\_min) | 19 | 2.51 | 4.96e-02 |

### Motifs enriched

|  |  |  |  |  |  |
| --- | --- | --- | --- | --- | --- |
| **Motif** | **Logo** | **Possible orthologs** | **Number of motifs in cluster** | **Enrichment** | **FDR corrected p** |
| ONECUT1\_1 |  | ceh-48 dsc-1 | 278 | 1.58 | 8.9e-18 |
| CG2052\_SOLEXA\_2.5\_FBgn0039905 |  | mel-28 (0.81) fkh-7 (0.73) lin-29 | 321 | 1.43 | 7.0e-16 |
| pTH9380 |  | mel-28 (0.81) | 365 | 1.31 | 9.5e-14 |
| pTH10797 |  | K11D2.4 lin-29 | 374 | 1.29 | 1.6e-13 |
| MA0049.1 |  | hbl-1 php-3 lin-39 | 298 | 1.43 | 2.0e-13 |
| Zfp161\_2858 |  | pzf-1 | 102 | 2.36 | 5.4e-13 |
| pTH9180 |  | mel-28 (0.81) let-381 mef-2 Y61A9LA.9 Y116A8C.22 | 368 | 1.29 | 5.5e-13 |
| MA0536.1 |  | elt-1 | 150 | 1.92 | 5.9e-13 |
| rn\_SOLEXA\_5\_FBgn0259172 |  | lin-29 | 302 | 1.40 | 1.8e-12 |
| pTH9097 |  | Y116A8C.22 | 366 | 1.28 | 4.6e-12 |
| pTH9335 |  | mel-28 (0.81) | 328 | 1.34 | 6.7e-12 |
| pTH9958 |  | ztf-6 (0.91) | 308 | 1.37 | 1.2e-11 |
| pTH9260 |  | mel-28 (0.81) | 346 | 1.31 | 1.2e-11 |
| FOXJ3\_1 |  | daf-16 (0.8) fkh-7 (0.73) lin-31 | 346 | 1.29 | 1.2e-10 |
| pTH9242 |  | mel-28 (0.81) | 351 | 1.27 | 5.8e-10 |
| pTH1294 |  | mel-28 (0.81) | 350 | 1.27 | 8.0e-10 |
| FOXD3\_f1 |  | let-381 lin-31 | 360 | 1.25 | 9.6e-10 |
| FOXJ3\_si |  | daf-16 (0.8) fkh-7 (0.73) let-381 lin-31 | 361 | 1.24 | 4.3e-09 |
| pTH9125 |  | sox-4 egl-13 | 326 | 1.29 | 4.6e-09 |
| pTH5169 |  | cfi-1 | 319 | 1.30 | 5.5e-09 |
| ARI3A\_do |  | cfi-1 | 284 | 1.35 | 9.0e-09 |
| pTH8982 |  | ceh-48 | 150 | 1.68 | 1.1e-08 |
| MA0541.1 |  | efl-1 (0.8) F49E12.6 | 203 | 1.51 | 1.2e-08 |
| Mv90 |  | mef-2 | 342 | 1.25 | 2.7e-08 |
| BARHL2\_3 |  | ceh-31 (-0.57) ceh-1 ceh-43 | 297 | 1.31 | 4.0e-08 |
| pTH9173 |  | efl-2 (0.54) | 151 | 1.64 | 4.9e-08 |
| pTH8863 |  | hmg-12 | 222 | 1.43 | 6.5e-08 |
| Hoxc10\_2 |  | pal-1 php-3 lin-39 | 299 | 1.30 | 1.0e-07 |
| exd\_FlyReg\_FBgn0000611 |  | ceh-20 let-381 cfi-1 | 366 | 1.21 | 1.2e-07 |
| Mafk\_3106 |  | F45H11.6 | 269 | 1.33 | 1.7e-07 |
| pTH9177 |  | hsf-1 (0.59) F10B5.3 | 305 | 1.28 | 3.0e-07 |
| pTH9254 |  | mel-28 (0.81) | 334 | 1.24 | 4.3e-07 |
| pTH3220 |  | Y5F2A.4 daf-12 | 183 | 1.48 | 6.2e-07 |
| MA0537.1 |  | blmp-1 | 342 | 1.22 | 6.8e-07 |
| pTH5916 |  | efl-2 (0.54) | 151 | 1.58 | 7.6e-07 |
| pTH9951 |  | mex-6 | 316 | 1.25 | 1.0e-06 |
| pTH9393 |  | ZC416.1 | 145 | 1.58 | 1.3e-06 |
| pTH9082 |  | mab-23 | 323 | 1.24 | 1.8e-06 |
| pTH8997 |  | hmbx-1 hmg-12 ceh-53 lin-39 Y116A8C.22 | 298 | 1.26 | 3.3e-06 |
| pnr\_SANGER\_5\_FBgn0003117 |  | elt-1 | 311 | 1.24 | 6.4e-06 |
| pTH9189 |  | ceh-18 dmd-3 | 320 | 1.21 | 2.1e-05 |
| pTH8566 |  | lin-54 | 312 | 1.22 | 3.1e-05 |
| pTH10696 |  | Y44A6D.3 | 123 | 1.57 | 3.6e-05 |
| pTH10798 |  | Y75B8A.6 | 164 | 1.44 | 3.6e-05 |
| pnt\_SANGER\_5\_FBgn0003118 |  | lin-1 | 252 | 1.28 | 6.8e-05 |
| FOXO1\_si |  | daf-16 (0.8) irx-1 (0.68) fkh-9 | 330 | 1.19 | 9.7e-05 |
| HXD10\_f1 |  | php-3 | 286 | 1.23 | 1.4e-04 |
| CG31670\_SANGER\_5\_FBgn0031375 |  | CELE\_Y38H8A.5 | 277 | 1.23 | 1.9e-04 |
| pTH9709 |  | die-1 (0.85) | 285 | 1.22 | 2.0e-04 |
| SPDEF\_3 |  | nhr-100 lin-1 lin-39 | 297 | 1.21 | 2.4e-04 |
| HOXD8\_1 |  | alr-1 egl-5 lin-39 | 242 | 1.27 | 2.4e-04 |
| MA0535.1 |  | daf-8 (0.57) | 159 | 1.40 | 2.7e-04 |
| V$BRN2\_01 |  | ceh-18 | 294 | 1.21 | 2.8e-04 |
| pTH3046 |  | Y116A8C.22 | 194 | 1.33 | 3.1e-04 |
| pTH5260 |  | aha-1 (0.77) lin-22 | 62 | 1.87 | 3.3e-04 |
| PHOX2B\_1 |  | alr-1 cfi-1 | 250 | 1.25 | 3.4e-04 |
| pTH3796 |  | let-381 | 283 | 1.22 | 4.1e-04 |
| MSX2\_f1 |  | ceh-1 alr-1 lin-39 | 175 | 1.36 | 4.5e-04 |
| POU3F3\_3 |  | ceh-18 unc-86 | 297 | 1.20 | 6.4e-04 |
| Eip93F\_SANGER\_10\_FBgn0013948 |  | bed-3 nhr-177 mbr-1 | 263 | 1.23 | 7.2e-04 |
| pTH7032 |  | F52B11.1 | 123 | 1.47 | 7.3e-04 |
| V$PBX1\_01 |  | ceh-20 lin-39 | 273 | 1.21 | 8.9e-04 |
| Abd-B\_FlyReg\_FBgn0000015 |  | ceh-24 D1005.3 | 262 | 1.22 | 1.1e-03 |
| pTH9384 |  | cfi-1 | 268 | 1.21 | 1.2e-03 |
| pTH8216 |  | Y116A8C.22 | 164 | 1.35 | 1.3e-03 |
| pTH9220 |  | mbr-1 | 233 | 1.25 | 1.3e-03 |
| pTH8649 |  | mbr-1 | 230 | 1.25 | 1.5e-03 |
| POU3F3\_1 |  | ceh-18 sox-4 | 277 | 1.20 | 1.6e-03 |
| Dll\_Cell\_FBgn0000157 |  | ceh-43 | 231 | 1.25 | 1.9e-03 |
| pTH8985 |  | athp-1 (0.88) | 220 | 1.26 | 1.9e-03 |
| E2F4\_1 |  | F49E12.6 | 180 | 1.31 | 2.2e-03 |
| CEBPE\_f1 |  | C48E7.11 R07H5.10 | 140 | 1.38 | 2.7e-03 |
| Mw151 |  | gei-11 | 286 | 1.19 | 2.9e-03 |
| pTH10645 |  | nhr-7 | 237 | 1.23 | 2.9e-03 |
| HES1\_f1 |  | lin-22 | 75 | 1.62 | 3.1e-03 |
| V$FAC1\_01 |  | gei-8 (0.75) | 304 | 1.17 | 3.3e-03 |
| ZNF75A\_1 |  | ztf-3 (0.67) | 240 | 1.22 | 3.4e-03 |
| CXXC1\_si |  | F52B11.1 F21D5.4 | 177 | 1.30 | 3.9e-03 |
| TBP\_f1 |  | tbp-1 | 242 | 1.22 | 4.4e-03 |
| pTH9089 |  | ref-2 | 57 | 1.72 | 5.9e-03 |
| HXD4\_f1 |  | cog-1 lin-39 | 133 | 1.37 | 6.1e-03 |
| pTH8679 |  | pax-2 | 65 | 1.64 | 6.2e-03 |
| LHX3\_f1 |  | lim-7 cfi-1 | 69 | 1.60 | 7.1e-03 |
| EGR4\_2 |  | ZC328.2 | 85 | 1.51 | 8.0e-03 |
| pTH9969 |  | pag-3 | 224 | 1.22 | 8.7e-03 |
| pTH9934 |  | Y53H1A.2 | 144 | 1.33 | 8.8e-03 |
| pTH5102 |  | mxl-1 hlh-30 lin-22 | 62 | 1.64 | 8.9e-03 |
| MA0547.1 |  | skn-1 (0.53) | 286 | 1.16 | 1.2e-02 |
| MA0543.1 |  | eor-1 (0.85) | 288 | 1.15 | 1.8e-02 |
| V$AHR\_01 |  | ahr-1 | 132 | 1.33 | 1.9e-02 |
| V$OCT1\_06 |  | ceh-18 | 288 | 1.15 | 1.9e-02 |
| pTH7875 |  | mel-28 (0.81) | 233 | 1.19 | 2.0e-02 |
| Tbp\_pr781 |  | tbp-1 | 234 | 1.19 | 2.5e-02 |
| MA0038.1 |  | odd-1 | 219 | 1.20 | 2.7e-02 |
| HSFY2\_1 |  | hsf-1 (0.59) | 107 | 1.37 | 2.8e-02 |
| SP4\_f1 |  | klf-2 | 150 | 1.28 | 2.8e-02 |
| pTH5250 |  | C48E7.11 | 101 | 1.37 | 3.4e-02 |
| EN1\_2 |  | ceh-16 (0.56) | 188 | 1.22 | 4.1e-02 |
| pTH8556 |  | pax-2 | 22 | 2.16 | 4.3e-02 |

### Correlated (and anti-correlated) transcription factors

|  |  |
| --- | --- |
| **Transcription factor** | **Correlation** |
| attf-2 | 0.95 |
| mcd-1 | 0.93 |
| F21A10.2 | 0.93 |
| swsn-7 | 0.92 |
| atg-4.1 | 0.92 |
| ztf-6 | 0.91 |
| uaf-2 | 0.91 |
| hmp-2 | 0.90 |
| Y82E9BR.1 | 0.90 |
| T20F7.1 | 0.90 |
| lin-38 | 0.90 |
| Y48C3A.12 | 0.90 |
| slr-2 | 0.89 |
| B0336.3 | 0.89 |
| Y48G8AL.10 | 0.89 |
| dpl-1 | 0.89 |
| ceh-100 | 0.88 |
| madf-3 | 0.88 |
| athp-1 | 0.88 |
| F33H1.4 | 0.87 |
| set-16 | 0.87 |
| Y82E9BR.17 | 0.86 |
| swsn-1 | 0.86 |
| F16B12.6 | 0.86 |
| snpc-4 | 0.86 |
| nhr-41 | -0.50 |
| madf-10 | -0.50 |
| mdl-1 | -0.50 |
| T18D3.7 | -0.52 |
| C35D6.4 | -0.52 |
| nhr-31 | -0.53 |
| T26A5.8 | -0.53 |
| nhr-14 | -0.54 |
| nhr-149 | -0.54 |
| gmeb-3 | -0.56 |
| egl-38 | -0.56 |
| ceh-7 | -0.56 |
| nhr-92 | -0.57 |
| nhr-87 | -0.57 |
| ccch-1 | -0.57 |
| ceh-31 | -0.57 |
| Y56A3A.18 | -0.61 |
| atf-8 | -0.61 |
| madf-1 | -0.62 |
| sdz-38 | -0.66 |
| ceh-88 | -0.67 |
| mxl-2 | -0.68 |
| nhr-222 | -0.68 |
| mxl-3 | -0.69 |
| mbf-1 | -0.79 |

### ChIP peaks enriched

|  |  |  |  |  |
| --- | --- | --- | --- | --- |
| **Gene** | **Experiment** | **Number of upstream peaks** | **Enrichment** | **FDR corrected p** |
| efl-1 | EFL-1\_Young-adult | 264 | 2.67 | 3.7e-59 |
| efl-1 | EFL-1\_Fed-L1-stage-larvae | 233 | 2.94 | 3.8e-57 |
| efl-1 | EFL-1\_Larvae-L1-stage | 255 | 2.54 | 3.9e-52 |
| lin-35 | LIN-35\_Fed-L1-stage-larvae | 237 | 2.71 | 5.4e-52 |
| lsy-2 | LSY-2\_Embryos | 207 | 3.03 | 7.9e-51 |
| dpl-1 | DPL-1\_Fed-L1-stage-larvae | 235 | 2.61 | 1.9e-48 |
| dpl-1 | DPL-1\_Young-adult | 225 | 2.63 | 4.3e-46 |
| lsy-2 | LSY-2\_Fed-L1-stage-larvae | 239 | 2.48 | 1.6e-45 |
| W03F9.2 | W03F9.2\_L4-Young-Adult-stage-larvae | 300 | 2.05 | 4.3e-45 |
| C34F6.9 | C34F6.9\_Larvae-L2-stage | 231 | 2.50 | 7.1e-44 |
| lsy-2 | LSY-2\_Larvae-L1-stage | 252 | 2.29 | 1.5e-42 |
| ceh-39 | CEH-39\_Embryos | 170 | 3.07 | 2.3e-40 |
| dpl-1 | DPL-1\_Larvae-L4-stage | 261 | 2.16 | 2.7e-40 |
| R02D3.7 | R02D3.7\_Larvae-L3-stage | 223 | 2.35 | 1.5e-37 |
| pes-1 | PES-1\_Larvae-L4-stage | 219 | 2.38 | 2.2e-37 |
| gei-11 | GEI-11\_Fed-L1-stage-larvae | 209 | 2.46 | 2.5e-37 |
| F16B12.6 | F16B12.6\_Fed-L1-stage-larvae | 137 | 3.44 | 2.5e-36 |
| fos-1 | FOS-1\_Fed-L1-stage-larvae | 202 | 2.46 | 1.5e-35 |
| eor-1 | EOR-1\_Larvae-L3-stage | 235 | 2.20 | 1.8e-35 |
| lin-15 | LIN-15B\_Fed-L1-stage-larvae | 158 | 2.98 | 2.0e-35 |
| C16A3.4 | C16A3.4\_Fed-L1-stage-larvae | 181 | 2.65 | 7.8e-35 |
| nfya-1 | NFYA-1\_Late-Embryos | 197 | 2.45 | 3.9e-34 |
| hpl-2 | HPL-2\_Fed-L1-stage-larvae | 236 | 2.13 | 2.5e-33 |
| lin-13 | LIN-13\_Larvae-L2-stage | 164 | 2.72 | 4.1e-32 |
| ceh-38 | CEH-38\_Larvae-L3-stage | 189 | 2.42 | 1.6e-31 |
| C01B12.2 | C01B12.2\_Larvae-L2-stage | 248 | 2.00 | 1.6e-31 |
| F45C12.2 | F45C12.2\_Fed-L1-stage-larvae | 205 | 2.28 | 2.2e-31 |
| aly-2 | ALY-2\_Fed-L1-stage-larvae | 178 | 2.51 | 4.4e-31 |
| ham-1 | HAM-1\_Larvae-L4-stage | 212 | 2.20 | 8.2e-31 |
| nfya-1 | NFYA-1\_Larvae-L3-stage | 175 | 2.50 | 3.3e-30 |
| nhr-25 | NHR-25\_Larvae-L2-stage | 178 | 2.45 | 6.1e-30 |
| nhr-77 | NHR-77\_Fed-L1-stage-larvae | 209 | 2.19 | 1.1e-29 |
| nhr-23 | NHR-23\_Larvae-L3-stage | 187 | 2.36 | 1.1e-29 |
| gei-11 | GEI-11\_Larvae-L2-stage | 166 | 2.57 | 1.2e-29 |
| R02D3.7 | R02D3.7\_Larvae-L2-stage | 131 | 3.01 | 1.1e-28 |
| gei-11 | GEI-11\_Larvae-L3-stage | 196 | 2.24 | 1.1e-28 |
| F23B12.7 | F23B12.7\_Young-adult | 143 | 2.79 | 2.3e-28 |
| ham-1 | HAM-1\_Fed-L1-stage-larvae | 198 | 2.21 | 3.6e-28 |
| ces-1 | CES-1\_Embryos | 200 | 2.19 | 6.1e-28 |
| lsy-2 | LSY-2\_Larvae-L2-stage | 125 | 3.07 | 6.9e-28 |
| pha-4 | PHA-4\_Larvae-L2-stage | 210 | 2.04 | 1.0e-25 |
| ceh-38 | CEH-38\_Larvae-L4-stage | 135 | 2.67 | 1.3e-24 |
| nhr-77 | NHR-77\_Larvae-L4-stage | 245 | 1.83 | 1.6e-24 |
| nhr-237 | NHR-237\_Embryos | 118 | 2.90 | 5.6e-24 |
| dve-1 | DVE-1\_Late-Embryos | 175 | 2.18 | 2.5e-23 |
| nhr-6 | NHR-6\_Larvae-L4-stage | 132 | 2.47 | 5.8e-21 |
| sem-4 | SEM-4\_Larvae-L2-stage | 179 | 2.00 | 8.4e-20 |
| nhr-76 | NHR-76\_Larvae-L4-stage | 113 | 2.64 | 1.4e-19 |
| lin-13 | LIN-13\_Larvae-L4-stage | 127 | 2.43 | 2.5e-19 |
| lin-35 | LIN-35\_Starved-L1-stage-larvae | 96 | 2.93 | 3.0e-19 |
| lin-13 | LIN-13\_Larvae-L1-stage | 76 | 3.50 | 4.0e-19 |
| ztf-7 | ZTF-7\_Larvae-L4-stage | 133 | 2.34 | 4.6e-19 |
| nhr-77 | NHR-77\_Larvae-L2-stage | 118 | 2.48 | 1.7e-18 |
| skn-1 | SKN-1\_Larvae-L3-stage | 104 | 2.61 | 1.7e-17 |
| zag-1 | ZAG-1\_Fed-L1-stage-larvae | 86 | 2.87 | 1.6e-16 |
| jun-1 | JUN-1\_Larvae-L1-stage | 151 | 2.03 | 2.2e-16 |
| egl-5 | EGL-5\_Larvae-L3-stage | 132 | 2.18 | 2.7e-16 |
| lin-15 | LIN-15B\_Larvae-L4-stage | 73 | 3.19 | 3.8e-16 |
| aly-2 | ALY-2\_Larvae-L3-stage | 100 | 2.55 | 4.8e-16 |
| F45C12.2 | F45C12.2\_Larvae-L3-stage | 91 | 2.70 | 6.3e-16 |
| elt-3 | ELT-3\_Embryos | 140 | 2.09 | 6.6e-16 |
| alr-1 | ALR-1\_Larvae-L2-stage | 153 | 1.98 | 8.6e-16 |
| nhr-77 | NHR-77\_Larvae-L3-stage | 130 | 2.16 | 1.1e-15 |
| lin-35 | LIN-35\_Young-adult | 135 | 2.08 | 4.3e-15 |
| nhr-129 | NHR-129\_Larvae-L2-stage | 209 | 1.67 | 6.8e-15 |
| sax-3 | SAX-3\_Larvae-L4-stage | 183 | 1.77 | 1.0e-14 |
| nhr-237 | NHR-237\_Larvae-L1-stage | 72 | 2.97 | 2.6e-14 |
| hlh-30 | HLH-30\_Larvae-L4-stage | 118 | 2.17 | 3.8e-14 |
| mab-5 | MAB-5\_Larvae-L2-stage | 88 | 2.56 | 5.4e-14 |
| R02D3.7 | R02D3.7\_Larvae-L4-stage | 96 | 2.40 | 1.1e-13 |
| nfya-1 | NFYA-1\_Young-adult | 73 | 2.83 | 1.6e-13 |
| F45C12.2 | F45C12.2\_Larvae-L2-stage | 71 | 2.83 | 4.1e-13 |
| jun-1 | JUN-1\_Larvae-L4-stage | 123 | 2.04 | 5.5e-13 |
| gei-11 | GEI-11\_Young-adult | 100 | 2.24 | 1.8e-12 |
| pax-1 | PAX-1\_Embryos | 57 | 3.17 | 2.8e-12 |
| sax-3 | SAX-3\_Larvae-L2-stage | 134 | 1.90 | 4.8e-12 |
| zag-1 | ZAG-1\_Larvae-L3-stage | 76 | 2.55 | 8.3e-12 |
| nhr-6 | NHR-6\_Larvae-L2-stage | 148 | 1.80 | 9.8e-12 |
| gei-11 | GEI-11\_Embryos | 58 | 3.00 | 1.6e-11 |
| hlh-30 | HLH-30\_Late-Embryos | 96 | 2.20 | 1.9e-11 |
| lsy-2 | LSY-2\_Larvae-L4-stage | 84 | 2.34 | 2.9e-11 |
| jun-1 | JUN-1\_Larvae-L3-stage | 106 | 2.05 | 5.3e-11 |
| pha-4 | PHA-4\_Young-adult | 94 | 2.11 | 3.3e-10 |
| unc-62 | UNC-62\_Fed-L1-stage-larvae | 52 | 2.94 | 5.3e-10 |
| unc-62 | UNC-62\_Day-Four-Young-Adult | 85 | 2.13 | 2.1e-09 |
| unc-62 | UNC-62\_Young-adult-Day-4 | 85 | 2.13 | 2.1e-09 |
| dve-1 | DVE-1\_Larvae-L4-stage | 109 | 1.87 | 5.1e-09 |
| fkh-2 | FKH-2\_Larvae-L3-stage | 54 | 2.64 | 9.6e-09 |
| aha-1 | AHA-1\_Fed-L1-stage-larvae | 30 | 4.01 | 1.5e-08 |
| mab-5 | MAB-5\_Embryos | 34 | 3.58 | 1.8e-08 |
| pha-4 | PHA-4\_Larvae-L4-stage | 113 | 1.79 | 2.5e-08 |
| zag-1 | ZAG-1\_Larvae-L2-stage | 101 | 1.86 | 4.6e-08 |
| ces-1 | CES-1\_Fed-L1-stage-larvae | 69 | 2.21 | 5.0e-08 |
| sax-3 | SAX-3\_Larvae-L3-stage | 83 | 2.01 | 5.7e-08 |
| nhr-11 | NHR-11\_Larvae-L2-stage | 74 | 2.10 | 9.3e-08 |
| ztf-4 | ZTF-4\_Larvae-L1-stage | 44 | 2.79 | 9.8e-08 |
| fos-1 | FOS-1\_Larvae-L4-stage | 68 | 2.18 | 1.1e-07 |
| nhr-2 | NHR-2\_Embryos | 79 | 2.01 | 1.5e-07 |
| zag-1 | ZAG-1\_Larvae-L4-stage | 86 | 1.94 | 1.5e-07 |
| ztf-4 | ZTF-4\_Larvae-L2-stage | 66 | 2.18 | 1.9e-07 |
| aha-1 | AHA-1\_Larvae-L4-stage | 60 | 2.26 | 2.6e-07 |
| ztf-4 | ZTF-4\_Larvae-L3-stage | 47 | 2.57 | 3.4e-07 |
| sax-3 | SAX-3\_Fed-L1-stage-larvae | 48 | 2.53 | 3.8e-07 |
| mef-2 | MEF-2\_Fed-L1-stage-larvae | 48 | 2.50 | 5.2e-07 |
| ceh-26 | CEH-26\_Late-Embryonic-stage | 97 | 1.79 | 5.9e-07 |
| sea-2 | SEA-2\_Larvae-L3-stage | 55 | 2.28 | 8.8e-07 |
| nhr-237 | NHR-237\_Larvae-L2-stage | 24 | 3.91 | 1.3e-06 |
| fos-1 | FOS-1\_Larvae-L3-stage | 105 | 1.70 | 1.9e-06 |
| aly-2 | ALY-2\_Larvae-L2-stage | 38 | 2.73 | 2.2e-06 |
| nhr-28 | NHR-28\_Larvae-L3-stage | 37 | 2.75 | 2.8e-06 |
| skn-1 | SKN-1\_Larvae-L4-stage | 21 | 4.20 | 3.0e-06 |
| ztf-11 | ZTF-11\_Embryos | 37 | 2.73 | 3.3e-06 |
| ama-1 | AMA-1\_Larvae-L3-stage | 64 | 2.02 | 4.7e-06 |
| unc-62 | UNC-62\_Larvae-L3-stage | 73 | 1.90 | 6.0e-06 |
| unc-62 | UNC-62\_Larvae-L2-stage | 64 | 1.98 | 9.5e-06 |
| fos-1 | FOS-1\_Larvae-L2-stage | 163 | 1.41 | 2.9e-05 |
| ceh-16 | CEH-16\_Larvae-L2-stage | 55 | 1.99 | 5.7e-05 |
| daf-12 | DAF-12\_Larvae-L3-stage | 27 | 2.89 | 6.1e-05 |
| nhr-76 | NHR-76\_Larvae-L3-stage | 65 | 1.82 | 1.1e-04 |
| nhr-28 | NHR-28\_Larvae-L4-stage | 129 | 1.42 | 5.8e-04 |
| unc-39 | UNC-39\_Embryos | 43 | 1.99 | 8.1e-04 |
| ces-1 | CES-1\_Larvae-L4-stage | 55 | 1.80 | 9.3e-04 |
| elt-1 | ELT-1\_Larvae-L3-stage | 54 | 1.75 | 2.1e-03 |
| med-1 | MED-1\_Embryos | 29 | 2.15 | 4.6e-03 |
| ces-1 | CES-1\_Larvae-L3-stage | 45 | 1.79 | 5.3e-03 |
| nhr-10 | NHR-10\_Larvae-L4-stage | 38 | 1.90 | 5.4e-03 |
| nhr-12 | NHR-12\_Larvae-L2-stage | 13 | 3.41 | 5.5e-03 |
| unc-39 | UNC-39\_Larvae-L2-stage | 27 | 2.16 | 6.9e-03 |
| nhr-21 | NHR-21\_Larvae-L2-stage | 36 | 1.89 | 8.4e-03 |
| nhr-23 | NHR-23\_Larvae-L2-stage | 24 | 2.03 | 3.2e-02 |
| peb-1 | PEB-1\_Larvae-L2-stage | 35 | 1.73 | 4.0e-02 |
| nhr-116 | NHR-116\_Larvae-L2-stage | 23 | 2.02 | 4.1e-02 |
| sma-9 | SMA-9\_Larvae-L2-stage | 21 | 2.07 | 4.8e-02 |
