# Supplementary material for: Overlapping cell population expression profiling and regulatory inference in C. elegans
Source: BMC Genomics. 2016 Feb 29;17:159. doi: 10.1186/s12864-016-2482-z (PMC4772325; doi:10.1186/s12864-016-2482-z)
Supplement: Additional file 13: — Web supplement. (DOC 21 kb) [file 12864_2016_2482_MOESM13_ESM.zip › sortWeb/clusters/hier.300.clusters/24.html]

Cluster 24 

## Cluster 24

### Expression

| cnd-1 rep. 1 | cnd-1 rep. 2 | cnd-1 rep. 3 | pha-4 rep. 1 | pha-4 rep. 2 | pha-4 rep. 3 | ceh-27 | ceh-36 | ceh-6 | F21D5.9 | mir-57 | mls-2 | pal-1 | pros-1 | ttx-3 | unc-130 | hlh-16 | irx-1 | ceh-6 (+) hlh-16 (+) | ceh-6 (+) hlh-16 (-) | ceh-6 (-) hlh-16 (+) | cnd-1 singlets | pha-4 singlets | 0 | 60 | 120 | 150 | 180 | 240 | 330 | 390 | 420 | 480 | 540 | 570 | 600 | 630 | 660 | NAME | Functional description |
| --- | --- | --- | --- | --- | --- | --- | --- | --- | --- | --- | --- | --- | --- | --- | --- | --- | --- | --- | --- | --- | --- | --- | --- | --- | --- | --- | --- | --- | --- | --- | --- | --- | --- | --- | --- | --- | --- | --- | --- |
|  |  |  |  |  |  |  |  |  |  |  |  |  |  |  |  |  |  |  |  |  |  |  |  |  |  |  |  |  |  |  |  |  |  |  |  |  |  | T23F6.2 |  |
|  |  |  |  |  |  |  |  |  |  |  |  |  |  |  |  |  |  |  |  |  |  |  |  |  |  |  |  |  |  |  |  |  |  |  |  |  |  | H12D21.11 |  |
|  |  |  |  |  |  |  |  |  |  |  |  |  |  |  |  |  |  |  |  |  |  |  |  |  |  |  |  |  |  |  |  |  |  |  |  |  |  | W04G5.3 |  |
|  |  |  |  |  |  |  |  |  |  |  |  |  |  |  |  |  |  |  |  |  |  |  |  |  |  |  |  |  |  |  |  |  |  |  |  |  |  | *chil-18* | CHItinase-Like |
|  |  |  |  |  |  |  |  |  |  |  |  |  |  |  |  |  |  |  |  |  |  |  |  |  |  |  |  |  |  |  |  |  |  |  |  |  |  | *str-22* | Seven TM Receptor |
|  |  |  |  |  |  |  |  |  |  |  |  |  |  |  |  |  |  |  |  |  |  |  |  |  |  |  |  |  |  |  |  |  |  |  |  |  |  | C33D12.17 |  |
|  |  |  |  |  |  |  |  |  |  |  |  |  |  |  |  |  |  |  |  |  |  |  |  |  |  |  |  |  |  |  |  |  |  |  |  |  |  | K09F6.11 |  |
|  |  |  |  |  |  |  |  |  |  |  |  |  |  |  |  |  |  |  |  |  |  |  |  |  |  |  |  |  |  |  |  |  |  |  |  |  |  | F19F10.1 |  |
|  |  |  |  |  |  |  |  |  |  |  |  |  |  |  |  |  |  |  |  |  |  |  |  |  |  |  |  |  |  |  |  |  |  |  |  |  |  | W03G11.6 |  |
|  |  |  |  |  |  |  |  |  |  |  |  |  |  |  |  |  |  |  |  |  |  |  |  |  |  |  |  |  |  |  |  |  |  |  |  |  |  | Y14H12A.3 |  |
|  |  |  |  |  |  |  |  |  |  |  |  |  |  |  |  |  |  |  |  |  |  |  |  |  |  |  |  |  |  |  |  |  |  |  |  |  |  | Y41D4B.25 |  |

### Phenotypes enriched

none found

### Anatomy terms enriched

none found

### GO terms enriched

none found

### Expression clusters enriched

none found

### Motifs enriched

None found

### Correlated (and anti-correlated) transcription factors

|  |  |
| --- | --- |
| **Transcription factor** | **Correlation** |
| F19F10.1 | 0.79 |
| C46E10.9 | 0.62 |
| nhr-271 | 0.60 |
| hlh-11 | 0.57 |
| mab-3 | 0.52 |
| nhr-111 | 0.52 |
| nhr-9 | 0.51 |
| hlh-15 | 0.50 |
| lin-22 | 0.50 |
| php-3 | 0.50 |
| ztf-2 | 0.46 |
| tlp-1 | 0.46 |
| nob-1 | 0.46 |
| hlh-8 | 0.45 |
| mls-1 | 0.45 |
| mab-5 | 0.45 |
| pes-1 | 0.44 |
| ehn-3 | 0.43 |
| ceh-49 | 0.43 |
| ceh-1 | 0.41 |
| dsc-1 | 0.40 |
| C38D4.7 | 0.40 |
| egl-5 | 0.39 |
| madf-9 | 0.38 |
| tbx-41 | 0.38 |
| C34F6.9 | -0.36 |
| T26A8.4 | -0.37 |
| tbx-34 | -0.37 |
| nfya-2 | -0.37 |
| aptf-1 | -0.38 |
| Y47G6A.7 | -0.38 |
| fezf-1 | -0.38 |
| egl-13 | -0.38 |
| ceh-90 | -0.38 |
| Y48G8AL.9 | -0.39 |
| nhr-159 | -0.39 |
| lin-13 | -0.40 |
| F10E7.11 | -0.40 |
| ztf-26 | -0.41 |
| ceh-9 | -0.41 |
| fkh-5 | -0.43 |
| nhr-254 | -0.44 |
| sox-3 | -0.46 |
| ceh-37 | -0.47 |
| nhr-38 | -0.47 |
| nhr-215 | -0.51 |
| sox-2 | -0.51 |
| nhr-220 | -0.52 |
| odr-7 | -0.53 |
| nhr-223 | -0.58 |

### ChIP peaks enriched

none found
