# Supplementary material for: Overlapping cell population expression profiling and regulatory inference in C. elegans
Source: BMC Genomics. 2016 Feb 29;17:159. doi: 10.1186/s12864-016-2482-z (PMC4772325; doi:10.1186/s12864-016-2482-z)
Supplement: Additional file 13: — Web supplement. (DOC 21 kb) [file 12864_2016_2482_MOESM13_ESM.zip › sortWeb/clusters/hier.300.clusters/242.html]

Cluster 242 

## Cluster 242

### Expression

| cnd-1 rep. 1 | cnd-1 rep. 2 | cnd-1 rep. 3 | pha-4 rep. 1 | pha-4 rep. 2 | pha-4 rep. 3 | ceh-27 | ceh-36 | ceh-6 | F21D5.9 | mir-57 | mls-2 | pal-1 | pros-1 | ttx-3 | unc-130 | hlh-16 | irx-1 | ceh-6 (+) hlh-16 (+) | ceh-6 (+) hlh-16 (-) | ceh-6 (-) hlh-16 (+) | cnd-1 singlets | pha-4 singlets | 0 | 60 | 120 | 150 | 180 | 240 | 330 | 390 | 420 | 480 | 540 | 570 | 600 | 630 | 660 | NAME | Functional description |
| --- | --- | --- | --- | --- | --- | --- | --- | --- | --- | --- | --- | --- | --- | --- | --- | --- | --- | --- | --- | --- | --- | --- | --- | --- | --- | --- | --- | --- | --- | --- | --- | --- | --- | --- | --- | --- | --- | --- | --- |
|  |  |  |  |  |  |  |  |  |  |  |  |  |  |  |  |  |  |  |  |  |  |  |  |  |  |  |  |  |  |  |  |  |  |  |  |  |  | R07B7.12 |  |
|  |  |  |  |  |  |  |  |  |  |  |  |  |  |  |  |  |  |  |  |  |  |  |  |  |  |  |  |  |  |  |  |  |  |  |  |  |  | F46B6.18 |  |
|  |  |  |  |  |  |  |  |  |  |  |  |  |  |  |  |  |  |  |  |  |  |  |  |  |  |  |  |  |  |  |  |  |  |  |  |  |  | *scrm-1* | SCRaMblase (phospholipid scramblase) |
|  |  |  |  |  |  |  |  |  |  |  |  |  |  |  |  |  |  |  |  |  |  |  |  |  |  |  |  |  |  |  |  |  |  |  |  |  |  | F25B4.2 |  |
|  |  |  |  |  |  |  |  |  |  |  |  |  |  |  |  |  |  |  |  |  |  |  |  |  |  |  |  |  |  |  |  |  |  |  |  |  |  | T22C1.6 |  |
|  |  |  |  |  |  |  |  |  |  |  |  |  |  |  |  |  |  |  |  |  |  |  |  |  |  |  |  |  |  |  |  |  |  |  |  |  |  | R13A5.7 |  |
|  |  |  |  |  |  |  |  |  |  |  |  |  |  |  |  |  |  |  |  |  |  |  |  |  |  |  |  |  |  |  |  |  |  |  |  |  |  | *dyf-7* | abnormal DYe Filling |
|  |  |  |  |  |  |  |  |  |  |  |  |  |  |  |  |  |  |  |  |  |  |  |  |  |  |  |  |  |  |  |  |  |  |  |  |  |  | *cst-2* | Caenorhabditis STE20-like kinase |
|  |  |  |  |  |  |  |  |  |  |  |  |  |  |  |  |  |  |  |  |  |  |  |  |  |  |  |  |  |  |  |  |  |  |  |  |  |  | *sli-1* | Suppressor of LIneage defect |
|  |  |  |  |  |  |  |  |  |  |  |  |  |  |  |  |  |  |  |  |  |  |  |  |  |  |  |  |  |  |  |  |  |  |  |  |  |  | T09B9.1 |  |
|  |  |  |  |  |  |  |  |  |  |  |  |  |  |  |  |  |  |  |  |  |  |  |  |  |  |  |  |  |  |  |  |  |  |  |  |  |  | *chs-1* | CHitin Synthase |
|  |  |  |  |  |  |  |  |  |  |  |  |  |  |  |  |  |  |  |  |  |  |  |  |  |  |  |  |  |  |  |  |  |  |  |  |  |  | F53H2.4 |  |
|  |  |  |  |  |  |  |  |  |  |  |  |  |  |  |  |  |  |  |  |  |  |  |  |  |  |  |  |  |  |  |  |  |  |  |  |  |  | F53E4.1 |  |
|  |  |  |  |  |  |  |  |  |  |  |  |  |  |  |  |  |  |  |  |  |  |  |  |  |  |  |  |  |  |  |  |  |  |  |  |  |  | F56A11.7 |  |
|  |  |  |  |  |  |  |  |  |  |  |  |  |  |  |  |  |  |  |  |  |  |  |  |  |  |  |  |  |  |  |  |  |  |  |  |  |  | Y42H9B.8 |  |
|  |  |  |  |  |  |  |  |  |  |  |  |  |  |  |  |  |  |  |  |  |  |  |  |  |  |  |  |  |  |  |  |  |  |  |  |  |  | *hlh-3* | Helix Loop Helix |
|  |  |  |  |  |  |  |  |  |  |  |  |  |  |  |  |  |  |  |  |  |  |  |  |  |  |  |  |  |  |  |  |  |  |  |  |  |  | *hil-1* | HIstone H1 Like |
|  |  |  |  |  |  |  |  |  |  |  |  |  |  |  |  |  |  |  |  |  |  |  |  |  |  |  |  |  |  |  |  |  |  |  |  |  |  | Y92H12BR.4 |  |
|  |  |  |  |  |  |  |  |  |  |  |  |  |  |  |  |  |  |  |  |  |  |  |  |  |  |  |  |  |  |  |  |  |  |  |  |  |  | C28G1.2 |  |
|  |  |  |  |  |  |  |  |  |  |  |  |  |  |  |  |  |  |  |  |  |  |  |  |  |  |  |  |  |  |  |  |  |  |  |  |  |  | *fbxb-111* | F-box B protein |
|  |  |  |  |  |  |  |  |  |  |  |  |  |  |  |  |  |  |  |  |  |  |  |  |  |  |  |  |  |  |  |  |  |  |  |  |  |  | W04A8.3 |  |
|  |  |  |  |  |  |  |  |  |  |  |  |  |  |  |  |  |  |  |  |  |  |  |  |  |  |  |  |  |  |  |  |  |  |  |  |  |  | *fbxb-45* | F-box B protein |
|  |  |  |  |  |  |  |  |  |  |  |  |  |  |  |  |  |  |  |  |  |  |  |  |  |  |  |  |  |  |  |  |  |  |  |  |  |  | *ccb-2* | Calcium Channel, Beta subunit |
|  |  |  |  |  |  |  |  |  |  |  |  |  |  |  |  |  |  |  |  |  |  |  |  |  |  |  |  |  |  |  |  |  |  |  |  |  |  | F38B7.3 |  |
|  |  |  |  |  |  |  |  |  |  |  |  |  |  |  |  |  |  |  |  |  |  |  |  |  |  |  |  |  |  |  |  |  |  |  |  |  |  | C01B12.9 |  |
|  |  |  |  |  |  |  |  |  |  |  |  |  |  |  |  |  |  |  |  |  |  |  |  |  |  |  |  |  |  |  |  |  |  |  |  |  |  | F40F9.5 |  |
|  |  |  |  |  |  |  |  |  |  |  |  |  |  |  |  |  |  |  |  |  |  |  |  |  |  |  |  |  |  |  |  |  |  |  |  |  |  | F39E9.8 |  |
|  |  |  |  |  |  |  |  |  |  |  |  |  |  |  |  |  |  |  |  |  |  |  |  |  |  |  |  |  |  |  |  |  |  |  |  |  |  | F52C9.1 |  |
|  |  |  |  |  |  |  |  |  |  |  |  |  |  |  |  |  |  |  |  |  |  |  |  |  |  |  |  |  |  |  |  |  |  |  |  |  |  | C11H1.5 |  |
|  |  |  |  |  |  |  |  |  |  |  |  |  |  |  |  |  |  |  |  |  |  |  |  |  |  |  |  |  |  |  |  |  |  |  |  |  |  | B0041.1 |  |
|  |  |  |  |  |  |  |  |  |  |  |  |  |  |  |  |  |  |  |  |  |  |  |  |  |  |  |  |  |  |  |  |  |  |  |  |  |  | *btb-16* | BTB (Broad/complex/Tramtrack/Bric a brac) domain protein |
|  |  |  |  |  |  |  |  |  |  |  |  |  |  |  |  |  |  |  |  |  |  |  |  |  |  |  |  |  |  |  |  |  |  |  |  |  |  | R13G10.4 |  |
|  |  |  |  |  |  |  |  |  |  |  |  |  |  |  |  |  |  |  |  |  |  |  |  |  |  |  |  |  |  |  |  |  |  |  |  |  |  | T08D2.3 |  |
|  |  |  |  |  |  |  |  |  |  |  |  |  |  |  |  |  |  |  |  |  |  |  |  |  |  |  |  |  |  |  |  |  |  |  |  |  |  | *try-6* | TRYpsin-like protease |
|  |  |  |  |  |  |  |  |  |  |  |  |  |  |  |  |  |  |  |  |  |  |  |  |  |  |  |  |  |  |  |  |  |  |  |  |  |  | *str-118* | Seven TM Receptor |
|  |  |  |  |  |  |  |  |  |  |  |  |  |  |  |  |  |  |  |  |  |  |  |  |  |  |  |  |  |  |  |  |  |  |  |  |  |  | F55C12.6 |  |
|  |  |  |  |  |  |  |  |  |  |  |  |  |  |  |  |  |  |  |  |  |  |  |  |  |  |  |  |  |  |  |  |  |  |  |  |  |  | *fbxb-67* | F-box B protein |
|  |  |  |  |  |  |  |  |  |  |  |  |  |  |  |  |  |  |  |  |  |  |  |  |  |  |  |  |  |  |  |  |  |  |  |  |  |  | F09B12.7 |  |
|  |  |  |  |  |  |  |  |  |  |  |  |  |  |  |  |  |  |  |  |  |  |  |  |  |  |  |  |  |  |  |  |  |  |  |  |  |  | R13A1.10 |  |
|  |  |  |  |  |  |  |  |  |  |  |  |  |  |  |  |  |  |  |  |  |  |  |  |  |  |  |  |  |  |  |  |  |  |  |  |  |  | F17C11.7 |  |
|  |  |  |  |  |  |  |  |  |  |  |  |  |  |  |  |  |  |  |  |  |  |  |  |  |  |  |  |  |  |  |  |  |  |  |  |  |  | *inx-9* | INneXin |
|  |  |  |  |  |  |  |  |  |  |  |  |  |  |  |  |  |  |  |  |  |  |  |  |  |  |  |  |  |  |  |  |  |  |  |  |  |  | F54B11.7 |  |
|  |  |  |  |  |  |  |  |  |  |  |  |  |  |  |  |  |  |  |  |  |  |  |  |  |  |  |  |  |  |  |  |  |  |  |  |  |  | Y49F6C.2 |  |
|  |  |  |  |  |  |  |  |  |  |  |  |  |  |  |  |  |  |  |  |  |  |  |  |  |  |  |  |  |  |  |  |  |  |  |  |  |  | Y75B7B.1 |  |
|  |  |  |  |  |  |  |  |  |  |  |  |  |  |  |  |  |  |  |  |  |  |  |  |  |  |  |  |  |  |  |  |  |  |  |  |  |  | *sym-4* | SYnthetic lethal with Mec |
|  |  |  |  |  |  |  |  |  |  |  |  |  |  |  |  |  |  |  |  |  |  |  |  |  |  |  |  |  |  |  |  |  |  |  |  |  |  | *hum-1* | Heavy chain, Unconventional Myosin |
|  |  |  |  |  |  |  |  |  |  |  |  |  |  |  |  |  |  |  |  |  |  |  |  |  |  |  |  |  |  |  |  |  |  |  |  |  |  | *nhr-228* | Nuclear Hormone Receptor family |
|  |  |  |  |  |  |  |  |  |  |  |  |  |  |  |  |  |  |  |  |  |  |  |  |  |  |  |  |  |  |  |  |  |  |  |  |  |  | *rig-4* | neuRonal IGCAM |
|  |  |  |  |  |  |  |  |  |  |  |  |  |  |  |  |  |  |  |  |  |  |  |  |  |  |  |  |  |  |  |  |  |  |  |  |  |  | T28C6.5 |  |
|  |  |  |  |  |  |  |  |  |  |  |  |  |  |  |  |  |  |  |  |  |  |  |  |  |  |  |  |  |  |  |  |  |  |  |  |  |  | *ifg-1* | Initiation Factor 4G (eIF4G) family |
|  |  |  |  |  |  |  |  |  |  |  |  |  |  |  |  |  |  |  |  |  |  |  |  |  |  |  |  |  |  |  |  |  |  |  |  |  |  | *ver-3* | VEGF (vascular endothelial growth factor) Receptor family |
|  |  |  |  |  |  |  |  |  |  |  |  |  |  |  |  |  |  |  |  |  |  |  |  |  |  |  |  |  |  |  |  |  |  |  |  |  |  | T04F8.6 |  |
|  |  |  |  |  |  |  |  |  |  |  |  |  |  |  |  |  |  |  |  |  |  |  |  |  |  |  |  |  |  |  |  |  |  |  |  |  |  | C32D5.10 |  |
|  |  |  |  |  |  |  |  |  |  |  |  |  |  |  |  |  |  |  |  |  |  |  |  |  |  |  |  |  |  |  |  |  |  |  |  |  |  | *tag-30* | Temporarily Assigned Gene name |
|  |  |  |  |  |  |  |  |  |  |  |  |  |  |  |  |  |  |  |  |  |  |  |  |  |  |  |  |  |  |  |  |  |  |  |  |  |  | K07H8.1 |  |
|  |  |  |  |  |  |  |  |  |  |  |  |  |  |  |  |  |  |  |  |  |  |  |  |  |  |  |  |  |  |  |  |  |  |  |  |  |  | C33D9.9 |  |
|  |  |  |  |  |  |  |  |  |  |  |  |  |  |  |  |  |  |  |  |  |  |  |  |  |  |  |  |  |  |  |  |  |  |  |  |  |  | C33D9.6 |  |
|  |  |  |  |  |  |  |  |  |  |  |  |  |  |  |  |  |  |  |  |  |  |  |  |  |  |  |  |  |  |  |  |  |  |  |  |  |  | *imp-1* | IntraMembrane Protease (IMPAS) family |
|  |  |  |  |  |  |  |  |  |  |  |  |  |  |  |  |  |  |  |  |  |  |  |  |  |  |  |  |  |  |  |  |  |  |  |  |  |  | F27D4.6 |  |
|  |  |  |  |  |  |  |  |  |  |  |  |  |  |  |  |  |  |  |  |  |  |  |  |  |  |  |  |  |  |  |  |  |  |  |  |  |  | T05F1.11 |  |
|  |  |  |  |  |  |  |  |  |  |  |  |  |  |  |  |  |  |  |  |  |  |  |  |  |  |  |  |  |  |  |  |  |  |  |  |  |  | *lig-4* | LIGase |
|  |  |  |  |  |  |  |  |  |  |  |  |  |  |  |  |  |  |  |  |  |  |  |  |  |  |  |  |  |  |  |  |  |  |  |  |  |  | B0001.6 |  |
|  |  |  |  |  |  |  |  |  |  |  |  |  |  |  |  |  |  |  |  |  |  |  |  |  |  |  |  |  |  |  |  |  |  |  |  |  |  | Y53G8AR.1 |  |
|  |  |  |  |  |  |  |  |  |  |  |  |  |  |  |  |  |  |  |  |  |  |  |  |  |  |  |  |  |  |  |  |  |  |  |  |  |  | T15B7.15 |  |
|  |  |  |  |  |  |  |  |  |  |  |  |  |  |  |  |  |  |  |  |  |  |  |  |  |  |  |  |  |  |  |  |  |  |  |  |  |  | F08G2.9 |  |
|  |  |  |  |  |  |  |  |  |  |  |  |  |  |  |  |  |  |  |  |  |  |  |  |  |  |  |  |  |  |  |  |  |  |  |  |  |  | C07C7.1 |  |
|  |  |  |  |  |  |  |  |  |  |  |  |  |  |  |  |  |  |  |  |  |  |  |  |  |  |  |  |  |  |  |  |  |  |  |  |  |  | F28C10.3 |  |
|  |  |  |  |  |  |  |  |  |  |  |  |  |  |  |  |  |  |  |  |  |  |  |  |  |  |  |  |  |  |  |  |  |  |  |  |  |  | Y55B1BR.2 |  |
|  |  |  |  |  |  |  |  |  |  |  |  |  |  |  |  |  |  |  |  |  |  |  |  |  |  |  |  |  |  |  |  |  |  |  |  |  |  | *gei-8* | GEX Interacting protein |
|  |  |  |  |  |  |  |  |  |  |  |  |  |  |  |  |  |  |  |  |  |  |  |  |  |  |  |  |  |  |  |  |  |  |  |  |  |  | Y105E8A.20 |  |
|  |  |  |  |  |  |  |  |  |  |  |  |  |  |  |  |  |  |  |  |  |  |  |  |  |  |  |  |  |  |  |  |  |  |  |  |  |  | *stau-1* | STAUfen (dsRNA binding protein) homolog |
|  |  |  |  |  |  |  |  |  |  |  |  |  |  |  |  |  |  |  |  |  |  |  |  |  |  |  |  |  |  |  |  |  |  |  |  |  |  | *csb-1* | human CSB (Cockayne Syndrome B) homolog |
|  |  |  |  |  |  |  |  |  |  |  |  |  |  |  |  |  |  |  |  |  |  |  |  |  |  |  |  |  |  |  |  |  |  |  |  |  |  | *epg-2* | Ectopic P Granules |
|  |  |  |  |  |  |  |  |  |  |  |  |  |  |  |  |  |  |  |  |  |  |  |  |  |  |  |  |  |  |  |  |  |  |  |  |  |  | *ubq-1* | UBiQuitin |
|  |  |  |  |  |  |  |  |  |  |  |  |  |  |  |  |  |  |  |  |  |  |  |  |  |  |  |  |  |  |  |  |  |  |  |  |  |  | *mbk-2* | MiniBrain Kinase (Drosophila) homolog |
|  |  |  |  |  |  |  |  |  |  |  |  |  |  |  |  |  |  |  |  |  |  |  |  |  |  |  |  |  |  |  |  |  |  |  |  |  |  | *siah-1* | SInA (Drosophila Seven In Absentia) Homolog |
|  |  |  |  |  |  |  |  |  |  |  |  |  |  |  |  |  |  |  |  |  |  |  |  |  |  |  |  |  |  |  |  |  |  |  |  |  |  | *hpo-29* | Hypersensitive to POre-forming toxin |
|  |  |  |  |  |  |  |  |  |  |  |  |  |  |  |  |  |  |  |  |  |  |  |  |  |  |  |  |  |  |  |  |  |  |  |  |  |  | *pqn-80* | Prion-like-(Q/N-rich)-domain-bearing protein |
|  |  |  |  |  |  |  |  |  |  |  |  |  |  |  |  |  |  |  |  |  |  |  |  |  |  |  |  |  |  |  |  |  |  |  |  |  |  | F26G1.1 |  |
|  |  |  |  |  |  |  |  |  |  |  |  |  |  |  |  |  |  |  |  |  |  |  |  |  |  |  |  |  |  |  |  |  |  |  |  |  |  | F44E2.7 |  |
|  |  |  |  |  |  |  |  |  |  |  |  |  |  |  |  |  |  |  |  |  |  |  |  |  |  |  |  |  |  |  |  |  |  |  |  |  |  | *flt-1* | FLecTin |
|  |  |  |  |  |  |  |  |  |  |  |  |  |  |  |  |  |  |  |  |  |  |  |  |  |  |  |  |  |  |  |  |  |  |  |  |  |  | *chd-3* | Chromodomain and Helicase Domain protein |
|  |  |  |  |  |  |  |  |  |  |  |  |  |  |  |  |  |  |  |  |  |  |  |  |  |  |  |  |  |  |  |  |  |  |  |  |  |  | *sur-2* | SUppressor of activated let-60 Ras |
|  |  |  |  |  |  |  |  |  |  |  |  |  |  |  |  |  |  |  |  |  |  |  |  |  |  |  |  |  |  |  |  |  |  |  |  |  |  | *farl-11* | FAR (Factor ARrest) Like |
|  |  |  |  |  |  |  |  |  |  |  |  |  |  |  |  |  |  |  |  |  |  |  |  |  |  |  |  |  |  |  |  |  |  |  |  |  |  | *dpy-26* | DumPY: shorter than wild-type |
|  |  |  |  |  |  |  |  |  |  |  |  |  |  |  |  |  |  |  |  |  |  |  |  |  |  |  |  |  |  |  |  |  |  |  |  |  |  | *apr-1* | APC Related |
|  |  |  |  |  |  |  |  |  |  |  |  |  |  |  |  |  |  |  |  |  |  |  |  |  |  |  |  |  |  |  |  |  |  |  |  |  |  | D1037.1 |  |
|  |  |  |  |  |  |  |  |  |  |  |  |  |  |  |  |  |  |  |  |  |  |  |  |  |  |  |  |  |  |  |  |  |  |  |  |  |  | F59E12.9 |  |
|  |  |  |  |  |  |  |  |  |  |  |  |  |  |  |  |  |  |  |  |  |  |  |  |  |  |  |  |  |  |  |  |  |  |  |  |  |  | *fzo-1* | FZO (Fzo mitochondrial fusion protein) related |
|  |  |  |  |  |  |  |  |  |  |  |  |  |  |  |  |  |  |  |  |  |  |  |  |  |  |  |  |  |  |  |  |  |  |  |  |  |  | F52C9.3 |  |
|  |  |  |  |  |  |  |  |  |  |  |  |  |  |  |  |  |  |  |  |  |  |  |  |  |  |  |  |  |  |  |  |  |  |  |  |  |  | *dnj-5* | DNaJ domain (prokaryotic heat shock protein) |
|  |  |  |  |  |  |  |  |  |  |  |  |  |  |  |  |  |  |  |  |  |  |  |  |  |  |  |  |  |  |  |  |  |  |  |  |  |  | *vps-54* | related to yeast Vacuolar Protein Sorting factor |
|  |  |  |  |  |  |  |  |  |  |  |  |  |  |  |  |  |  |  |  |  |  |  |  |  |  |  |  |  |  |  |  |  |  |  |  |  |  | *rad-26* | RADiation sensitivity abnormal/yeast RAD-related |
|  |  |  |  |  |  |  |  |  |  |  |  |  |  |  |  |  |  |  |  |  |  |  |  |  |  |  |  |  |  |  |  |  |  |  |  |  |  | *spe-39* | defective SPErmatogenesis |
|  |  |  |  |  |  |  |  |  |  |  |  |  |  |  |  |  |  |  |  |  |  |  |  |  |  |  |  |  |  |  |  |  |  |  |  |  |  | F55A3.2 |  |
|  |  |  |  |  |  |  |  |  |  |  |  |  |  |  |  |  |  |  |  |  |  |  |  |  |  |  |  |  |  |  |  |  |  |  |  |  |  | *snx-6* | Sorting NeXin |
|  |  |  |  |  |  |  |  |  |  |  |  |  |  |  |  |  |  |  |  |  |  |  |  |  |  |  |  |  |  |  |  |  |  |  |  |  |  | *mtm-3* | MTM (myotubularin) family |
|  |  |  |  |  |  |  |  |  |  |  |  |  |  |  |  |  |  |  |  |  |  |  |  |  |  |  |  |  |  |  |  |  |  |  |  |  |  | F56C9.10 |  |
|  |  |  |  |  |  |  |  |  |  |  |  |  |  |  |  |  |  |  |  |  |  |  |  |  |  |  |  |  |  |  |  |  |  |  |  |  |  | *atg-11* | AuTophaGy (yeast Atg homolog) |
|  |  |  |  |  |  |  |  |  |  |  |  |  |  |  |  |  |  |  |  |  |  |  |  |  |  |  |  |  |  |  |  |  |  |  |  |  |  | B0511.12 |  |
|  |  |  |  |  |  |  |  |  |  |  |  |  |  |  |  |  |  |  |  |  |  |  |  |  |  |  |  |  |  |  |  |  |  |  |  |  |  | *dpy-27* | DumPY: shorter than wild-type |
|  |  |  |  |  |  |  |  |  |  |  |  |  |  |  |  |  |  |  |  |  |  |  |  |  |  |  |  |  |  |  |  |  |  |  |  |  |  | *kin-18* | protein KINase |
|  |  |  |  |  |  |  |  |  |  |  |  |  |  |  |  |  |  |  |  |  |  |  |  |  |  |  |  |  |  |  |  |  |  |  |  |  |  | W09C5.7 |  |
|  |  |  |  |  |  |  |  |  |  |  |  |  |  |  |  |  |  |  |  |  |  |  |  |  |  |  |  |  |  |  |  |  |  |  |  |  |  | R06F6.8 |  |
|  |  |  |  |  |  |  |  |  |  |  |  |  |  |  |  |  |  |  |  |  |  |  |  |  |  |  |  |  |  |  |  |  |  |  |  |  |  | C28H8.3 |  |
|  |  |  |  |  |  |  |  |  |  |  |  |  |  |  |  |  |  |  |  |  |  |  |  |  |  |  |  |  |  |  |  |  |  |  |  |  |  | *cbp-1* | CBP/p300 homolog |
|  |  |  |  |  |  |  |  |  |  |  |  |  |  |  |  |  |  |  |  |  |  |  |  |  |  |  |  |  |  |  |  |  |  |  |  |  |  | B0564.7 |  |
|  |  |  |  |  |  |  |  |  |  |  |  |  |  |  |  |  |  |  |  |  |  |  |  |  |  |  |  |  |  |  |  |  |  |  |  |  |  | *rme-8* | Receptor Mediated Endocytosis |
|  |  |  |  |  |  |  |  |  |  |  |  |  |  |  |  |  |  |  |  |  |  |  |  |  |  |  |  |  |  |  |  |  |  |  |  |  |  | *lars-2* | Leucyl Amino-acyl tRNA Synthetase |
|  |  |  |  |  |  |  |  |  |  |  |  |  |  |  |  |  |  |  |  |  |  |  |  |  |  |  |  |  |  |  |  |  |  |  |  |  |  | *clu-1* | yeast CLU (mitochondrial clustering) related |
|  |  |  |  |  |  |  |  |  |  |  |  |  |  |  |  |  |  |  |  |  |  |  |  |  |  |  |  |  |  |  |  |  |  |  |  |  |  | *pek-1* | human PERK kinase homolog |
|  |  |  |  |  |  |  |  |  |  |  |  |  |  |  |  |  |  |  |  |  |  |  |  |  |  |  |  |  |  |  |  |  |  |  |  |  |  | *aars-1* | Alanyl Amino-acyl tRNA Synthetase |
|  |  |  |  |  |  |  |  |  |  |  |  |  |  |  |  |  |  |  |  |  |  |  |  |  |  |  |  |  |  |  |  |  |  |  |  |  |  | *let-716* | LEThal |
|  |  |  |  |  |  |  |  |  |  |  |  |  |  |  |  |  |  |  |  |  |  |  |  |  |  |  |  |  |  |  |  |  |  |  |  |  |  | R151.7 |  |
|  |  |  |  |  |  |  |  |  |  |  |  |  |  |  |  |  |  |  |  |  |  |  |  |  |  |  |  |  |  |  |  |  |  |  |  |  |  | *abcf-1* | ABC transporter, class F |
|  |  |  |  |  |  |  |  |  |  |  |  |  |  |  |  |  |  |  |  |  |  |  |  |  |  |  |  |  |  |  |  |  |  |  |  |  |  | *xpo-3* | eXPOrtin (nuclear export receptor) |
|  |  |  |  |  |  |  |  |  |  |  |  |  |  |  |  |  |  |  |  |  |  |  |  |  |  |  |  |  |  |  |  |  |  |  |  |  |  | *ptrn-1* | PaTRoNin (microtubule-binding protein) homolog |
|  |  |  |  |  |  |  |  |  |  |  |  |  |  |  |  |  |  |  |  |  |  |  |  |  |  |  |  |  |  |  |  |  |  |  |  |  |  | *alg-1* | Argonaute (plant)-Like Gene |
|  |  |  |  |  |  |  |  |  |  |  |  |  |  |  |  |  |  |  |  |  |  |  |  |  |  |  |  |  |  |  |  |  |  |  |  |  |  | *rlbp-1* | RaL Binding Protein |
|  |  |  |  |  |  |  |  |  |  |  |  |  |  |  |  |  |  |  |  |  |  |  |  |  |  |  |  |  |  |  |  |  |  |  |  |  |  | H05C05.2 |  |
|  |  |  |  |  |  |  |  |  |  |  |  |  |  |  |  |  |  |  |  |  |  |  |  |  |  |  |  |  |  |  |  |  |  |  |  |  |  | F21D5.7 |  |
|  |  |  |  |  |  |  |  |  |  |  |  |  |  |  |  |  |  |  |  |  |  |  |  |  |  |  |  |  |  |  |  |  |  |  |  |  |  | *flcn-1* | FoLliCuliN |
|  |  |  |  |  |  |  |  |  |  |  |  |  |  |  |  |  |  |  |  |  |  |  |  |  |  |  |  |  |  |  |  |  |  |  |  |  |  | F28H6.6 |  |
|  |  |  |  |  |  |  |  |  |  |  |  |  |  |  |  |  |  |  |  |  |  |  |  |  |  |  |  |  |  |  |  |  |  |  |  |  |  | C01G6.5 |  |
|  |  |  |  |  |  |  |  |  |  |  |  |  |  |  |  |  |  |  |  |  |  |  |  |  |  |  |  |  |  |  |  |  |  |  |  |  |  | F28C1.3 |  |
|  |  |  |  |  |  |  |  |  |  |  |  |  |  |  |  |  |  |  |  |  |  |  |  |  |  |  |  |  |  |  |  |  |  |  |  |  |  | *atg-2* | AuTophaGy (yeast Atg homolog) |
|  |  |  |  |  |  |  |  |  |  |  |  |  |  |  |  |  |  |  |  |  |  |  |  |  |  |  |  |  |  |  |  |  |  |  |  |  |  | ZK688.5 |  |
|  |  |  |  |  |  |  |  |  |  |  |  |  |  |  |  |  |  |  |  |  |  |  |  |  |  |  |  |  |  |  |  |  |  |  |  |  |  | C53B4.4 |  |
|  |  |  |  |  |  |  |  |  |  |  |  |  |  |  |  |  |  |  |  |  |  |  |  |  |  |  |  |  |  |  |  |  |  |  |  |  |  | *agef-1* | Arf-1 Guanine nucleotide Exchange Factor homolog |
|  |  |  |  |  |  |  |  |  |  |  |  |  |  |  |  |  |  |  |  |  |  |  |  |  |  |  |  |  |  |  |  |  |  |  |  |  |  | *sel-2* | Suppressor/Enhancer of Lin-12 |
|  |  |  |  |  |  |  |  |  |  |  |  |  |  |  |  |  |  |  |  |  |  |  |  |  |  |  |  |  |  |  |  |  |  |  |  |  |  | F14E5.2 |  |
|  |  |  |  |  |  |  |  |  |  |  |  |  |  |  |  |  |  |  |  |  |  |  |  |  |  |  |  |  |  |  |  |  |  |  |  |  |  | F13B9.1 |  |
|  |  |  |  |  |  |  |  |  |  |  |  |  |  |  |  |  |  |  |  |  |  |  |  |  |  |  |  |  |  |  |  |  |  |  |  |  |  | C11H1.3 |  |
|  |  |  |  |  |  |  |  |  |  |  |  |  |  |  |  |  |  |  |  |  |  |  |  |  |  |  |  |  |  |  |  |  |  |  |  |  |  | T26A8.1 |  |
|  |  |  |  |  |  |  |  |  |  |  |  |  |  |  |  |  |  |  |  |  |  |  |  |  |  |  |  |  |  |  |  |  |  |  |  |  |  | *syx-5* | SYntaXin |
|  |  |  |  |  |  |  |  |  |  |  |  |  |  |  |  |  |  |  |  |  |  |  |  |  |  |  |  |  |  |  |  |  |  |  |  |  |  | Y59C2A.2 |  |
|  |  |  |  |  |  |  |  |  |  |  |  |  |  |  |  |  |  |  |  |  |  |  |  |  |  |  |  |  |  |  |  |  |  |  |  |  |  | *set-31* | SET (trithorax/polycomb) domain containing |
|  |  |  |  |  |  |  |  |  |  |  |  |  |  |  |  |  |  |  |  |  |  |  |  |  |  |  |  |  |  |  |  |  |  |  |  |  |  | *linc-167* | Long Intervening Non-Coding RNA |
|  |  |  |  |  |  |  |  |  |  |  |  |  |  |  |  |  |  |  |  |  |  |  |  |  |  |  |  |  |  |  |  |  |  |  |  |  |  | F53F4.8 |  |
|  |  |  |  |  |  |  |  |  |  |  |  |  |  |  |  |  |  |  |  |  |  |  |  |  |  |  |  |  |  |  |  |  |  |  |  |  |  | F59A2.6 |  |
|  |  |  |  |  |  |  |  |  |  |  |  |  |  |  |  |  |  |  |  |  |  |  |  |  |  |  |  |  |  |  |  |  |  |  |  |  |  | F54D8.6 |  |
|  |  |  |  |  |  |  |  |  |  |  |  |  |  |  |  |  |  |  |  |  |  |  |  |  |  |  |  |  |  |  |  |  |  |  |  |  |  | D1069.3 |  |
|  |  |  |  |  |  |  |  |  |  |  |  |  |  |  |  |  |  |  |  |  |  |  |  |  |  |  |  |  |  |  |  |  |  |  |  |  |  | *nos-3* | NanOS related |
|  |  |  |  |  |  |  |  |  |  |  |  |  |  |  |  |  |  |  |  |  |  |  |  |  |  |  |  |  |  |  |  |  |  |  |  |  |  | *dcap-2* | mRNA DeCAPping enzyme |
|  |  |  |  |  |  |  |  |  |  |  |  |  |  |  |  |  |  |  |  |  |  |  |  |  |  |  |  |  |  |  |  |  |  |  |  |  |  | *gei-6* | GEX Interacting protein |
|  |  |  |  |  |  |  |  |  |  |  |  |  |  |  |  |  |  |  |  |  |  |  |  |  |  |  |  |  |  |  |  |  |  |  |  |  |  | *adr-1* | Adenosine Deaminase acting on RNA |
|  |  |  |  |  |  |  |  |  |  |  |  |  |  |  |  |  |  |  |  |  |  |  |  |  |  |  |  |  |  |  |  |  |  |  |  |  |  | *lin-13* | abnormal cell LINeage |
|  |  |  |  |  |  |  |  |  |  |  |  |  |  |  |  |  |  |  |  |  |  |  |  |  |  |  |  |  |  |  |  |  |  |  |  |  |  | R02D3.4 |  |
|  |  |  |  |  |  |  |  |  |  |  |  |  |  |  |  |  |  |  |  |  |  |  |  |  |  |  |  |  |  |  |  |  |  |  |  |  |  | M03C11.8 |  |
|  |  |  |  |  |  |  |  |  |  |  |  |  |  |  |  |  |  |  |  |  |  |  |  |  |  |  |  |  |  |  |  |  |  |  |  |  |  | *cul-1* | CULlin |
|  |  |  |  |  |  |  |  |  |  |  |  |  |  |  |  |  |  |  |  |  |  |  |  |  |  |  |  |  |  |  |  |  |  |  |  |  |  | F10G7.9 |  |
|  |  |  |  |  |  |  |  |  |  |  |  |  |  |  |  |  |  |  |  |  |  |  |  |  |  |  |  |  |  |  |  |  |  |  |  |  |  | F59E12.6 |  |
|  |  |  |  |  |  |  |  |  |  |  |  |  |  |  |  |  |  |  |  |  |  |  |  |  |  |  |  |  |  |  |  |  |  |  |  |  |  | T08B2.5 |  |
|  |  |  |  |  |  |  |  |  |  |  |  |  |  |  |  |  |  |  |  |  |  |  |  |  |  |  |  |  |  |  |  |  |  |  |  |  |  | T10D4.6 |  |
|  |  |  |  |  |  |  |  |  |  |  |  |  |  |  |  |  |  |  |  |  |  |  |  |  |  |  |  |  |  |  |  |  |  |  |  |  |  | F23H11.2 |  |
|  |  |  |  |  |  |  |  |  |  |  |  |  |  |  |  |  |  |  |  |  |  |  |  |  |  |  |  |  |  |  |  |  |  |  |  |  |  | *cux-7* | Clk-2 Upstream, human gene XE7 related |
|  |  |  |  |  |  |  |  |  |  |  |  |  |  |  |  |  |  |  |  |  |  |  |  |  |  |  |  |  |  |  |  |  |  |  |  |  |  | C33D9.5 |  |
|  |  |  |  |  |  |  |  |  |  |  |  |  |  |  |  |  |  |  |  |  |  |  |  |  |  |  |  |  |  |  |  |  |  |  |  |  |  | C32D5.11 |  |
|  |  |  |  |  |  |  |  |  |  |  |  |  |  |  |  |  |  |  |  |  |  |  |  |  |  |  |  |  |  |  |  |  |  |  |  |  |  | *usp-33* | Ubiquitin Specific Protease |
|  |  |  |  |  |  |  |  |  |  |  |  |  |  |  |  |  |  |  |  |  |  |  |  |  |  |  |  |  |  |  |  |  |  |  |  |  |  | *sax-2* | Sensory AXon guidance |
|  |  |  |  |  |  |  |  |  |  |  |  |  |  |  |  |  |  |  |  |  |  |  |  |  |  |  |  |  |  |  |  |  |  |  |  |  |  | W04D2.6 |  |
|  |  |  |  |  |  |  |  |  |  |  |  |  |  |  |  |  |  |  |  |  |  |  |  |  |  |  |  |  |  |  |  |  |  |  |  |  |  | Y32H12A.8 |  |
|  |  |  |  |  |  |  |  |  |  |  |  |  |  |  |  |  |  |  |  |  |  |  |  |  |  |  |  |  |  |  |  |  |  |  |  |  |  | ZK742.2 |  |
|  |  |  |  |  |  |  |  |  |  |  |  |  |  |  |  |  |  |  |  |  |  |  |  |  |  |  |  |  |  |  |  |  |  |  |  |  |  | *pqn-47* | Prion-like-(Q/N-rich)-domain-bearing protein |
|  |  |  |  |  |  |  |  |  |  |  |  |  |  |  |  |  |  |  |  |  |  |  |  |  |  |  |  |  |  |  |  |  |  |  |  |  |  | *pan-1* | P-granule Associated Novel protein |
|  |  |  |  |  |  |  |  |  |  |  |  |  |  |  |  |  |  |  |  |  |  |  |  |  |  |  |  |  |  |  |  |  |  |  |  |  |  | F41H10.4 |  |
|  |  |  |  |  |  |  |  |  |  |  |  |  |  |  |  |  |  |  |  |  |  |  |  |  |  |  |  |  |  |  |  |  |  |  |  |  |  | *let-363* | LEThal |
|  |  |  |  |  |  |  |  |  |  |  |  |  |  |  |  |  |  |  |  |  |  |  |  |  |  |  |  |  |  |  |  |  |  |  |  |  |  | *lpd-3* | LiPid Depleted |
|  |  |  |  |  |  |  |  |  |  |  |  |  |  |  |  |  |  |  |  |  |  |  |  |  |  |  |  |  |  |  |  |  |  |  |  |  |  | *ced-5* | CEll Death abnormality |
|  |  |  |  |  |  |  |  |  |  |  |  |  |  |  |  |  |  |  |  |  |  |  |  |  |  |  |  |  |  |  |  |  |  |  |  |  |  | ZC506.1 |  |
|  |  |  |  |  |  |  |  |  |  |  |  |  |  |  |  |  |  |  |  |  |  |  |  |  |  |  |  |  |  |  |  |  |  |  |  |  |  | F32A7.4 |  |
|  |  |  |  |  |  |  |  |  |  |  |  |  |  |  |  |  |  |  |  |  |  |  |  |  |  |  |  |  |  |  |  |  |  |  |  |  |  | *catp-8* | Cation transporting ATPase |
|  |  |  |  |  |  |  |  |  |  |  |  |  |  |  |  |  |  |  |  |  |  |  |  |  |  |  |  |  |  |  |  |  |  |  |  |  |  | *ppk-1* | PIP Kinase |
|  |  |  |  |  |  |  |  |  |  |  |  |  |  |  |  |  |  |  |  |  |  |  |  |  |  |  |  |  |  |  |  |  |  |  |  |  |  | *hpo-35* | Hypersensitive to POre-forming toxin |
|  |  |  |  |  |  |  |  |  |  |  |  |  |  |  |  |  |  |  |  |  |  |  |  |  |  |  |  |  |  |  |  |  |  |  |  |  |  | F21G4.6 |  |
|  |  |  |  |  |  |  |  |  |  |  |  |  |  |  |  |  |  |  |  |  |  |  |  |  |  |  |  |  |  |  |  |  |  |  |  |  |  | EEED8.16 |  |
|  |  |  |  |  |  |  |  |  |  |  |  |  |  |  |  |  |  |  |  |  |  |  |  |  |  |  |  |  |  |  |  |  |  |  |  |  |  | R11A8.7 |  |
|  |  |  |  |  |  |  |  |  |  |  |  |  |  |  |  |  |  |  |  |  |  |  |  |  |  |  |  |  |  |  |  |  |  |  |  |  |  | R12E2.2 |  |
|  |  |  |  |  |  |  |  |  |  |  |  |  |  |  |  |  |  |  |  |  |  |  |  |  |  |  |  |  |  |  |  |  |  |  |  |  |  | *samp-1* | SAMP1 (Senescence Accelerated Mouse Protein) homolog |
|  |  |  |  |  |  |  |  |  |  |  |  |  |  |  |  |  |  |  |  |  |  |  |  |  |  |  |  |  |  |  |  |  |  |  |  |  |  | *nrde-2* | Nuclear RNAi DEfective |
|  |  |  |  |  |  |  |  |  |  |  |  |  |  |  |  |  |  |  |  |  |  |  |  |  |  |  |  |  |  |  |  |  |  |  |  |  |  | *clk-2* | CLocK (biological timing) abnormality |
|  |  |  |  |  |  |  |  |  |  |  |  |  |  |  |  |  |  |  |  |  |  |  |  |  |  |  |  |  |  |  |  |  |  |  |  |  |  | *sin-3* | SIN3 (yeast Switch INdependent) histone deacetylase component homolog |
|  |  |  |  |  |  |  |  |  |  |  |  |  |  |  |  |  |  |  |  |  |  |  |  |  |  |  |  |  |  |  |  |  |  |  |  |  |  | *lex-1* | Lin-48 EXpression abnormal |
|  |  |  |  |  |  |  |  |  |  |  |  |  |  |  |  |  |  |  |  |  |  |  |  |  |  |  |  |  |  |  |  |  |  |  |  |  |  | *ced-7* | CEll Death abnormality |
|  |  |  |  |  |  |  |  |  |  |  |  |  |  |  |  |  |  |  |  |  |  |  |  |  |  |  |  |  |  |  |  |  |  |  |  |  |  | C44E4.1 |  |
|  |  |  |  |  |  |  |  |  |  |  |  |  |  |  |  |  |  |  |  |  |  |  |  |  |  |  |  |  |  |  |  |  |  |  |  |  |  | *rbg-2* | RaB GAP related |
|  |  |  |  |  |  |  |  |  |  |  |  |  |  |  |  |  |  |  |  |  |  |  |  |  |  |  |  |  |  |  |  |  |  |  |  |  |  | *alx-1* | ALIX (Apoptosis-linked gene 2 interacting protein X) homolog |
|  |  |  |  |  |  |  |  |  |  |  |  |  |  |  |  |  |  |  |  |  |  |  |  |  |  |  |  |  |  |  |  |  |  |  |  |  |  | B0261.1 |  |
|  |  |  |  |  |  |  |  |  |  |  |  |  |  |  |  |  |  |  |  |  |  |  |  |  |  |  |  |  |  |  |  |  |  |  |  |  |  | C36E8.1 |  |
|  |  |  |  |  |  |  |  |  |  |  |  |  |  |  |  |  |  |  |  |  |  |  |  |  |  |  |  |  |  |  |  |  |  |  |  |  |  | *smg-1* | Suppressor with Morphological effect on Genitalia |
|  |  |  |  |  |  |  |  |  |  |  |  |  |  |  |  |  |  |  |  |  |  |  |  |  |  |  |  |  |  |  |  |  |  |  |  |  |  | F08F8.9 |  |
|  |  |  |  |  |  |  |  |  |  |  |  |  |  |  |  |  |  |  |  |  |  |  |  |  |  |  |  |  |  |  |  |  |  |  |  |  |  | T05H10.1 |  |
|  |  |  |  |  |  |  |  |  |  |  |  |  |  |  |  |  |  |  |  |  |  |  |  |  |  |  |  |  |  |  |  |  |  |  |  |  |  | *cul-5* | CULlin |
|  |  |  |  |  |  |  |  |  |  |  |  |  |  |  |  |  |  |  |  |  |  |  |  |  |  |  |  |  |  |  |  |  |  |  |  |  |  | F39C12.1 |  |
|  |  |  |  |  |  |  |  |  |  |  |  |  |  |  |  |  |  |  |  |  |  |  |  |  |  |  |  |  |  |  |  |  |  |  |  |  |  | *tra-4* | TRAnsformer: XX animals transformed into males |
|  |  |  |  |  |  |  |  |  |  |  |  |  |  |  |  |  |  |  |  |  |  |  |  |  |  |  |  |  |  |  |  |  |  |  |  |  |  | *brf-1* | BRF (transcription factor) homolog |
|  |  |  |  |  |  |  |  |  |  |  |  |  |  |  |  |  |  |  |  |  |  |  |  |  |  |  |  |  |  |  |  |  |  |  |  |  |  | *daf-15* | abnormal DAuer Formation |
|  |  |  |  |  |  |  |  |  |  |  |  |  |  |  |  |  |  |  |  |  |  |  |  |  |  |  |  |  |  |  |  |  |  |  |  |  |  | *mtk-1* | MTK1/MEKK4 homolog |
|  |  |  |  |  |  |  |  |  |  |  |  |  |  |  |  |  |  |  |  |  |  |  |  |  |  |  |  |  |  |  |  |  |  |  |  |  |  | *emb-5* | abnormal EMBroygenesis |
|  |  |  |  |  |  |  |  |  |  |  |  |  |  |  |  |  |  |  |  |  |  |  |  |  |  |  |  |  |  |  |  |  |  |  |  |  |  | T24H7.2 |  |
|  |  |  |  |  |  |  |  |  |  |  |  |  |  |  |  |  |  |  |  |  |  |  |  |  |  |  |  |  |  |  |  |  |  |  |  |  |  | *cul-4* | CULlin |
|  |  |  |  |  |  |  |  |  |  |  |  |  |  |  |  |  |  |  |  |  |  |  |  |  |  |  |  |  |  |  |  |  |  |  |  |  |  | *spat-3* | Suppressor of PAr-Two defect |
|  |  |  |  |  |  |  |  |  |  |  |  |  |  |  |  |  |  |  |  |  |  |  |  |  |  |  |  |  |  |  |  |  |  |  |  |  |  | *vps-53* | related to yeast Vacuolar Protein Sorting factor |
|  |  |  |  |  |  |  |  |  |  |  |  |  |  |  |  |  |  |  |  |  |  |  |  |  |  |  |  |  |  |  |  |  |  |  |  |  |  | *lin-25* | abnormal cell LINeage |
|  |  |  |  |  |  |  |  |  |  |  |  |  |  |  |  |  |  |  |  |  |  |  |  |  |  |  |  |  |  |  |  |  |  |  |  |  |  | C46F11.4 |  |
|  |  |  |  |  |  |  |  |  |  |  |  |  |  |  |  |  |  |  |  |  |  |  |  |  |  |  |  |  |  |  |  |  |  |  |  |  |  | C44H4.4 |  |
|  |  |  |  |  |  |  |  |  |  |  |  |  |  |  |  |  |  |  |  |  |  |  |  |  |  |  |  |  |  |  |  |  |  |  |  |  |  | F13H8.8 |  |
|  |  |  |  |  |  |  |  |  |  |  |  |  |  |  |  |  |  |  |  |  |  |  |  |  |  |  |  |  |  |  |  |  |  |  |  |  |  | Y55D9A.2 |  |
|  |  |  |  |  |  |  |  |  |  |  |  |  |  |  |  |  |  |  |  |  |  |  |  |  |  |  |  |  |  |  |  |  |  |  |  |  |  | R07E4.5 |  |
|  |  |  |  |  |  |  |  |  |  |  |  |  |  |  |  |  |  |  |  |  |  |  |  |  |  |  |  |  |  |  |  |  |  |  |  |  |  | T23G7.3 |  |
|  |  |  |  |  |  |  |  |  |  |  |  |  |  |  |  |  |  |  |  |  |  |  |  |  |  |  |  |  |  |  |  |  |  |  |  |  |  | *plc-4* | PhosphoLipase C |
|  |  |  |  |  |  |  |  |  |  |  |  |  |  |  |  |  |  |  |  |  |  |  |  |  |  |  |  |  |  |  |  |  |  |  |  |  |  | *uri-1* |  |
|  |  |  |  |  |  |  |  |  |  |  |  |  |  |  |  |  |  |  |  |  |  |  |  |  |  |  |  |  |  |  |  |  |  |  |  |  |  | *prpf-4* | vertebrate Pre-mRNA Processing Factor |
|  |  |  |  |  |  |  |  |  |  |  |  |  |  |  |  |  |  |  |  |  |  |  |  |  |  |  |  |  |  |  |  |  |  |  |  |  |  | F43G9.12 |  |
|  |  |  |  |  |  |  |  |  |  |  |  |  |  |  |  |  |  |  |  |  |  |  |  |  |  |  |  |  |  |  |  |  |  |  |  |  |  | *mfap-1* | MicroFibrillar-Associated Protein homolog |
|  |  |  |  |  |  |  |  |  |  |  |  |  |  |  |  |  |  |  |  |  |  |  |  |  |  |  |  |  |  |  |  |  |  |  |  |  |  | C09E7.9 |  |
|  |  |  |  |  |  |  |  |  |  |  |  |  |  |  |  |  |  |  |  |  |  |  |  |  |  |  |  |  |  |  |  |  |  |  |  |  |  | T14B4.1 |  |
|  |  |  |  |  |  |  |  |  |  |  |  |  |  |  |  |  |  |  |  |  |  |  |  |  |  |  |  |  |  |  |  |  |  |  |  |  |  | R11A8.2 |  |
|  |  |  |  |  |  |  |  |  |  |  |  |  |  |  |  |  |  |  |  |  |  |  |  |  |  |  |  |  |  |  |  |  |  |  |  |  |  | *sec-5* | yeast SEC homolog |
|  |  |  |  |  |  |  |  |  |  |  |  |  |  |  |  |  |  |  |  |  |  |  |  |  |  |  |  |  |  |  |  |  |  |  |  |  |  | F20D12.2 |  |
|  |  |  |  |  |  |  |  |  |  |  |  |  |  |  |  |  |  |  |  |  |  |  |  |  |  |  |  |  |  |  |  |  |  |  |  |  |  | R07E5.1 |  |
|  |  |  |  |  |  |  |  |  |  |  |  |  |  |  |  |  |  |  |  |  |  |  |  |  |  |  |  |  |  |  |  |  |  |  |  |  |  | C07H6.4 |  |
|  |  |  |  |  |  |  |  |  |  |  |  |  |  |  |  |  |  |  |  |  |  |  |  |  |  |  |  |  |  |  |  |  |  |  |  |  |  | *pqn-21* | Prion-like-(Q/N-rich)-domain-bearing protein |
|  |  |  |  |  |  |  |  |  |  |  |  |  |  |  |  |  |  |  |  |  |  |  |  |  |  |  |  |  |  |  |  |  |  |  |  |  |  | *let-19* | LEThal |
|  |  |  |  |  |  |  |  |  |  |  |  |  |  |  |  |  |  |  |  |  |  |  |  |  |  |  |  |  |  |  |  |  |  |  |  |  |  | *ebax-1* | Elongin-B/C E3 ligase in AXon guidance |
|  |  |  |  |  |  |  |  |  |  |  |  |  |  |  |  |  |  |  |  |  |  |  |  |  |  |  |  |  |  |  |  |  |  |  |  |  |  | *chd-1* | Chromodomain and Helicase Domain protein |
|  |  |  |  |  |  |  |  |  |  |  |  |  |  |  |  |  |  |  |  |  |  |  |  |  |  |  |  |  |  |  |  |  |  |  |  |  |  | F36A2.13 |  |
|  |  |  |  |  |  |  |  |  |  |  |  |  |  |  |  |  |  |  |  |  |  |  |  |  |  |  |  |  |  |  |  |  |  |  |  |  |  | *vms-1* | VCP/Cdc48-associated Mitochondrial Stress-responsive |
|  |  |  |  |  |  |  |  |  |  |  |  |  |  |  |  |  |  |  |  |  |  |  |  |  |  |  |  |  |  |  |  |  |  |  |  |  |  | C52B9.8 |  |
|  |  |  |  |  |  |  |  |  |  |  |  |  |  |  |  |  |  |  |  |  |  |  |  |  |  |  |  |  |  |  |  |  |  |  |  |  |  | F54C9.9 |  |
|  |  |  |  |  |  |  |  |  |  |  |  |  |  |  |  |  |  |  |  |  |  |  |  |  |  |  |  |  |  |  |  |  |  |  |  |  |  | F28C1.1 |  |
|  |  |  |  |  |  |  |  |  |  |  |  |  |  |  |  |  |  |  |  |  |  |  |  |  |  |  |  |  |  |  |  |  |  |  |  |  |  | *cdh-4* | CaDHerin family |
|  |  |  |  |  |  |  |  |  |  |  |  |  |  |  |  |  |  |  |  |  |  |  |  |  |  |  |  |  |  |  |  |  |  |  |  |  |  | *aman-2* | Alpha MANnosidase |
|  |  |  |  |  |  |  |  |  |  |  |  |  |  |  |  |  |  |  |  |  |  |  |  |  |  |  |  |  |  |  |  |  |  |  |  |  |  | *dcr-1* | DiCer Related |
|  |  |  |  |  |  |  |  |  |  |  |  |  |  |  |  |  |  |  |  |  |  |  |  |  |  |  |  |  |  |  |  |  |  |  |  |  |  | *ddx-23* | DEAD boX helicase homolog |
|  |  |  |  |  |  |  |  |  |  |  |  |  |  |  |  |  |  |  |  |  |  |  |  |  |  |  |  |  |  |  |  |  |  |  |  |  |  | *npp-21* | Nuclear Pore complex Protein |
|  |  |  |  |  |  |  |  |  |  |  |  |  |  |  |  |  |  |  |  |  |  |  |  |  |  |  |  |  |  |  |  |  |  |  |  |  |  | *mog-1* | Masculinisation Of Germline |
|  |  |  |  |  |  |  |  |  |  |  |  |  |  |  |  |  |  |  |  |  |  |  |  |  |  |  |  |  |  |  |  |  |  |  |  |  |  | F18C12.3 |  |
|  |  |  |  |  |  |  |  |  |  |  |  |  |  |  |  |  |  |  |  |  |  |  |  |  |  |  |  |  |  |  |  |  |  |  |  |  |  | *tag-184* | Temporarily Assigned Gene name |
|  |  |  |  |  |  |  |  |  |  |  |  |  |  |  |  |  |  |  |  |  |  |  |  |  |  |  |  |  |  |  |  |  |  |  |  |  |  | *cid-1* | Caffeine Induced Death (S. pombe Cid) homolog |
|  |  |  |  |  |  |  |  |  |  |  |  |  |  |  |  |  |  |  |  |  |  |  |  |  |  |  |  |  |  |  |  |  |  |  |  |  |  | *him-18* | High Incidence of Males (increased X chromosome loss) |
|  |  |  |  |  |  |  |  |  |  |  |  |  |  |  |  |  |  |  |  |  |  |  |  |  |  |  |  |  |  |  |  |  |  |  |  |  |  | *rcq-5* | ReCQ DNA helicase family |
|  |  |  |  |  |  |  |  |  |  |  |  |  |  |  |  |  |  |  |  |  |  |  |  |  |  |  |  |  |  |  |  |  |  |  |  |  |  | *mbk-1* | MiniBrain Kinase (Drosophila) homolog |
|  |  |  |  |  |  |  |  |  |  |  |  |  |  |  |  |  |  |  |  |  |  |  |  |  |  |  |  |  |  |  |  |  |  |  |  |  |  | *mog-5* | Masculinisation Of Germline |
|  |  |  |  |  |  |  |  |  |  |  |  |  |  |  |  |  |  |  |  |  |  |  |  |  |  |  |  |  |  |  |  |  |  |  |  |  |  | *emb-30* | abnormal EMBroygenesis |
|  |  |  |  |  |  |  |  |  |  |  |  |  |  |  |  |  |  |  |  |  |  |  |  |  |  |  |  |  |  |  |  |  |  |  |  |  |  | *swsn-4* | SWI/SNF nucleosome remodeling complex component |
|  |  |  |  |  |  |  |  |  |  |  |  |  |  |  |  |  |  |  |  |  |  |  |  |  |  |  |  |  |  |  |  |  |  |  |  |  |  | *rbpl-1* | Retinoblastoma Binding Protein Like |
|  |  |  |  |  |  |  |  |  |  |  |  |  |  |  |  |  |  |  |  |  |  |  |  |  |  |  |  |  |  |  |  |  |  |  |  |  |  | *taf-1* | TAF (TBP-associated transcription factor) family |
|  |  |  |  |  |  |  |  |  |  |  |  |  |  |  |  |  |  |  |  |  |  |  |  |  |  |  |  |  |  |  |  |  |  |  |  |  |  | *iars-1* | Isoleucyl Amino-acyl tRNA Synthetase |
|  |  |  |  |  |  |  |  |  |  |  |  |  |  |  |  |  |  |  |  |  |  |  |  |  |  |  |  |  |  |  |  |  |  |  |  |  |  | F19F10.11 |  |
|  |  |  |  |  |  |  |  |  |  |  |  |  |  |  |  |  |  |  |  |  |  |  |  |  |  |  |  |  |  |  |  |  |  |  |  |  |  | Y54E10A.11 |  |
|  |  |  |  |  |  |  |  |  |  |  |  |  |  |  |  |  |  |  |  |  |  |  |  |  |  |  |  |  |  |  |  |  |  |  |  |  |  | C35A5.8 |  |
|  |  |  |  |  |  |  |  |  |  |  |  |  |  |  |  |  |  |  |  |  |  |  |  |  |  |  |  |  |  |  |  |  |  |  |  |  |  | T05E8.3 |  |
|  |  |  |  |  |  |  |  |  |  |  |  |  |  |  |  |  |  |  |  |  |  |  |  |  |  |  |  |  |  |  |  |  |  |  |  |  |  | *lars-1* | Leucyl Amino-acyl tRNA Synthetase |
|  |  |  |  |  |  |  |  |  |  |  |  |  |  |  |  |  |  |  |  |  |  |  |  |  |  |  |  |  |  |  |  |  |  |  |  |  |  | C01B10.9 |  |
|  |  |  |  |  |  |  |  |  |  |  |  |  |  |  |  |  |  |  |  |  |  |  |  |  |  |  |  |  |  |  |  |  |  |  |  |  |  | *dnj-10* | DNaJ domain (prokaryotic heat shock protein) |
|  |  |  |  |  |  |  |  |  |  |  |  |  |  |  |  |  |  |  |  |  |  |  |  |  |  |  |  |  |  |  |  |  |  |  |  |  |  | *rabs-5* | RABenoSyn (trafficking protein) homolog |
|  |  |  |  |  |  |  |  |  |  |  |  |  |  |  |  |  |  |  |  |  |  |  |  |  |  |  |  |  |  |  |  |  |  |  |  |  |  | *coq-8* | COenzyme Q (ubiquinone) biosynthesis |
|  |  |  |  |  |  |  |  |  |  |  |  |  |  |  |  |  |  |  |  |  |  |  |  |  |  |  |  |  |  |  |  |  |  |  |  |  |  | C52B9.4 |  |
|  |  |  |  |  |  |  |  |  |  |  |  |  |  |  |  |  |  |  |  |  |  |  |  |  |  |  |  |  |  |  |  |  |  |  |  |  |  | *klp-8* | Kinesin-Like Protein |
|  |  |  |  |  |  |  |  |  |  |  |  |  |  |  |  |  |  |  |  |  |  |  |  |  |  |  |  |  |  |  |  |  |  |  |  |  |  | F11C1.5 |  |
|  |  |  |  |  |  |  |  |  |  |  |  |  |  |  |  |  |  |  |  |  |  |  |  |  |  |  |  |  |  |  |  |  |  |  |  |  |  | K07B1.7 |  |
|  |  |  |  |  |  |  |  |  |  |  |  |  |  |  |  |  |  |  |  |  |  |  |  |  |  |  |  |  |  |  |  |  |  |  |  |  |  | *ceh-86* | C. Elegans Homeobox |
|  |  |  |  |  |  |  |  |  |  |  |  |  |  |  |  |  |  |  |  |  |  |  |  |  |  |  |  |  |  |  |  |  |  |  |  |  |  | K07E3.2 |  |
|  |  |  |  |  |  |  |  |  |  |  |  |  |  |  |  |  |  |  |  |  |  |  |  |  |  |  |  |  |  |  |  |  |  |  |  |  |  | *dpf-3* | Dipeptidyl Peptidase Four (IV) family |
|  |  |  |  |  |  |  |  |  |  |  |  |  |  |  |  |  |  |  |  |  |  |  |  |  |  |  |  |  |  |  |  |  |  |  |  |  |  | Y106G6A.1 |  |
|  |  |  |  |  |  |  |  |  |  |  |  |  |  |  |  |  |  |  |  |  |  |  |  |  |  |  |  |  |  |  |  |  |  |  |  |  |  | *sec-6* | yeast SEC homolog |
|  |  |  |  |  |  |  |  |  |  |  |  |  |  |  |  |  |  |  |  |  |  |  |  |  |  |  |  |  |  |  |  |  |  |  |  |  |  | ZK652.6 |  |
|  |  |  |  |  |  |  |  |  |  |  |  |  |  |  |  |  |  |  |  |  |  |  |  |  |  |  |  |  |  |  |  |  |  |  |  |  |  | C06G3.6 |  |
|  |  |  |  |  |  |  |  |  |  |  |  |  |  |  |  |  |  |  |  |  |  |  |  |  |  |  |  |  |  |  |  |  |  |  |  |  |  | T02E1.2 |  |
|  |  |  |  |  |  |  |  |  |  |  |  |  |  |  |  |  |  |  |  |  |  |  |  |  |  |  |  |  |  |  |  |  |  |  |  |  |  | *egl-27* | EGg Laying defective |
|  |  |  |  |  |  |  |  |  |  |  |  |  |  |  |  |  |  |  |  |  |  |  |  |  |  |  |  |  |  |  |  |  |  |  |  |  |  | *aka-1* | A Kinase Anchor protein |
|  |  |  |  |  |  |  |  |  |  |  |  |  |  |  |  |  |  |  |  |  |  |  |  |  |  |  |  |  |  |  |  |  |  |  |  |  |  | *sec-8* | yeast SEC homolog |
|  |  |  |  |  |  |  |  |  |  |  |  |  |  |  |  |  |  |  |  |  |  |  |  |  |  |  |  |  |  |  |  |  |  |  |  |  |  | *pap-1* | Poly-A Polymerase |
|  |  |  |  |  |  |  |  |  |  |  |  |  |  |  |  |  |  |  |  |  |  |  |  |  |  |  |  |  |  |  |  |  |  |  |  |  |  | *ada-2* | ADA (histone acetyltransferase complex (subuunit) |
|  |  |  |  |  |  |  |  |  |  |  |  |  |  |  |  |  |  |  |  |  |  |  |  |  |  |  |  |  |  |  |  |  |  |  |  |  |  | W05H9.4 |  |
|  |  |  |  |  |  |  |  |  |  |  |  |  |  |  |  |  |  |  |  |  |  |  |  |  |  |  |  |  |  |  |  |  |  |  |  |  |  | *mom-4* | More Of MS |
|  |  |  |  |  |  |  |  |  |  |  |  |  |  |  |  |  |  |  |  |  |  |  |  |  |  |  |  |  |  |  |  |  |  |  |  |  |  | C27A12.2 |  |
|  |  |  |  |  |  |  |  |  |  |  |  |  |  |  |  |  |  |  |  |  |  |  |  |  |  |  |  |  |  |  |  |  |  |  |  |  |  | *pbrm-1* | PolyBRoMo domain containing |
|  |  |  |  |  |  |  |  |  |  |  |  |  |  |  |  |  |  |  |  |  |  |  |  |  |  |  |  |  |  |  |  |  |  |  |  |  |  | *hda-2* | Histone DeAcetylase |
|  |  |  |  |  |  |  |  |  |  |  |  |  |  |  |  |  |  |  |  |  |  |  |  |  |  |  |  |  |  |  |  |  |  |  |  |  |  | *pak-2* | P21-Activated Kinase family |
|  |  |  |  |  |  |  |  |  |  |  |  |  |  |  |  |  |  |  |  |  |  |  |  |  |  |  |  |  |  |  |  |  |  |  |  |  |  | *gls-1* | Germ Line Survival |
|  |  |  |  |  |  |  |  |  |  |  |  |  |  |  |  |  |  |  |  |  |  |  |  |  |  |  |  |  |  |  |  |  |  |  |  |  |  | K07A12.4 |  |
|  |  |  |  |  |  |  |  |  |  |  |  |  |  |  |  |  |  |  |  |  |  |  |  |  |  |  |  |  |  |  |  |  |  |  |  |  |  | C27A12.9 |  |
|  |  |  |  |  |  |  |  |  |  |  |  |  |  |  |  |  |  |  |  |  |  |  |  |  |  |  |  |  |  |  |  |  |  |  |  |  |  | *ufl-1* | UFM-protein Ligase |
|  |  |  |  |  |  |  |  |  |  |  |  |  |  |  |  |  |  |  |  |  |  |  |  |  |  |  |  |  |  |  |  |  |  |  |  |  |  | T13H5.4 |  |
|  |  |  |  |  |  |  |  |  |  |  |  |  |  |  |  |  |  |  |  |  |  |  |  |  |  |  |  |  |  |  |  |  |  |  |  |  |  | *wdr-20* | WD Repeat protein |
|  |  |  |  |  |  |  |  |  |  |  |  |  |  |  |  |  |  |  |  |  |  |  |  |  |  |  |  |  |  |  |  |  |  |  |  |  |  | *vps-33.2* | related to yeast Vacuolar Protein Sorting factor |
|  |  |  |  |  |  |  |  |  |  |  |  |  |  |  |  |  |  |  |  |  |  |  |  |  |  |  |  |  |  |  |  |  |  |  |  |  |  | *iglr-2* | IG(immunoglobulin) and LRR(Leucine Rich Repeat) domains |
|  |  |  |  |  |  |  |  |  |  |  |  |  |  |  |  |  |  |  |  |  |  |  |  |  |  |  |  |  |  |  |  |  |  |  |  |  |  | *mrps-18A* | Mitochondrial Ribosomal Protein, Small |
|  |  |  |  |  |  |  |  |  |  |  |  |  |  |  |  |  |  |  |  |  |  |  |  |  |  |  |  |  |  |  |  |  |  |  |  |  |  | *pnk-1* | PaNtothenate Kinase |
|  |  |  |  |  |  |  |  |  |  |  |  |  |  |  |  |  |  |  |  |  |  |  |  |  |  |  |  |  |  |  |  |  |  |  |  |  |  | *dpf-7* | Dipeptidyl Peptidase Four (IV) family |
|  |  |  |  |  |  |  |  |  |  |  |  |  |  |  |  |  |  |  |  |  |  |  |  |  |  |  |  |  |  |  |  |  |  |  |  |  |  | *athp-2* | AT Hook plus PHD finger transcription factor |
|  |  |  |  |  |  |  |  |  |  |  |  |  |  |  |  |  |  |  |  |  |  |  |  |  |  |  |  |  |  |  |  |  |  |  |  |  |  | B0035.12 |  |
|  |  |  |  |  |  |  |  |  |  |  |  |  |  |  |  |  |  |  |  |  |  |  |  |  |  |  |  |  |  |  |  |  |  |  |  |  |  | R11D1.1 |  |
|  |  |  |  |  |  |  |  |  |  |  |  |  |  |  |  |  |  |  |  |  |  |  |  |  |  |  |  |  |  |  |  |  |  |  |  |  |  | C05C10.2 |  |
|  |  |  |  |  |  |  |  |  |  |  |  |  |  |  |  |  |  |  |  |  |  |  |  |  |  |  |  |  |  |  |  |  |  |  |  |  |  | F55A3.3 |  |
|  |  |  |  |  |  |  |  |  |  |  |  |  |  |  |  |  |  |  |  |  |  |  |  |  |  |  |  |  |  |  |  |  |  |  |  |  |  | *rpb-2* | RNA Polymerase II (B) subunit |
|  |  |  |  |  |  |  |  |  |  |  |  |  |  |  |  |  |  |  |  |  |  |  |  |  |  |  |  |  |  |  |  |  |  |  |  |  |  | *dic-1* | human DICE1 (Deleted In Cancer) homolog |
|  |  |  |  |  |  |  |  |  |  |  |  |  |  |  |  |  |  |  |  |  |  |  |  |  |  |  |  |  |  |  |  |  |  |  |  |  |  | *sec-3* | yeast SEC homolog |
|  |  |  |  |  |  |  |  |  |  |  |  |  |  |  |  |  |  |  |  |  |  |  |  |  |  |  |  |  |  |  |  |  |  |  |  |  |  | T05E7.3 |  |
|  |  |  |  |  |  |  |  |  |  |  |  |  |  |  |  |  |  |  |  |  |  |  |  |  |  |  |  |  |  |  |  |  |  |  |  |  |  | *xpg-1* | XPG (Xeroderma Pigmentosum group G) DNA repair gene homolog |
|  |  |  |  |  |  |  |  |  |  |  |  |  |  |  |  |  |  |  |  |  |  |  |  |  |  |  |  |  |  |  |  |  |  |  |  |  |  | *mcrs-1* | MCRS1 (microtubule-binding MiCRoSpherule Protein 1) homolog |
|  |  |  |  |  |  |  |  |  |  |  |  |  |  |  |  |  |  |  |  |  |  |  |  |  |  |  |  |  |  |  |  |  |  |  |  |  |  | *cfim-2* | Cleavage Factor IM (CFIm) homolog |
|  |  |  |  |  |  |  |  |  |  |  |  |  |  |  |  |  |  |  |  |  |  |  |  |  |  |  |  |  |  |  |  |  |  |  |  |  |  | Y32H12A.2 |  |
|  |  |  |  |  |  |  |  |  |  |  |  |  |  |  |  |  |  |  |  |  |  |  |  |  |  |  |  |  |  |  |  |  |  |  |  |  |  | *tlk-1* | Tousled-Like Kinase |
|  |  |  |  |  |  |  |  |  |  |  |  |  |  |  |  |  |  |  |  |  |  |  |  |  |  |  |  |  |  |  |  |  |  |  |  |  |  | *prp-17* | yeast PRP (splicing factor) related |
|  |  |  |  |  |  |  |  |  |  |  |  |  |  |  |  |  |  |  |  |  |  |  |  |  |  |  |  |  |  |  |  |  |  |  |  |  |  | *ntl-9* | NOT-Like (yeast CCR4/NOT complex component) |
|  |  |  |  |  |  |  |  |  |  |  |  |  |  |  |  |  |  |  |  |  |  |  |  |  |  |  |  |  |  |  |  |  |  |  |  |  |  | T23B12.4 |  |
|  |  |  |  |  |  |  |  |  |  |  |  |  |  |  |  |  |  |  |  |  |  |  |  |  |  |  |  |  |  |  |  |  |  |  |  |  |  | ZC504.3 |  |
|  |  |  |  |  |  |  |  |  |  |  |  |  |  |  |  |  |  |  |  |  |  |  |  |  |  |  |  |  |  |  |  |  |  |  |  |  |  | *him-17* | High Incidence of Males (increased X chromosome loss) |
|  |  |  |  |  |  |  |  |  |  |  |  |  |  |  |  |  |  |  |  |  |  |  |  |  |  |  |  |  |  |  |  |  |  |  |  |  |  | *mans-3* | MAnnoSidase (family 47 glycohydrolase) |
|  |  |  |  |  |  |  |  |  |  |  |  |  |  |  |  |  |  |  |  |  |  |  |  |  |  |  |  |  |  |  |  |  |  |  |  |  |  | C09D4.4 |  |
|  |  |  |  |  |  |  |  |  |  |  |  |  |  |  |  |  |  |  |  |  |  |  |  |  |  |  |  |  |  |  |  |  |  |  |  |  |  | *ddb-1* | DDB1 (UV-Damaged DNA Binding protein) homolog |
|  |  |  |  |  |  |  |  |  |  |  |  |  |  |  |  |  |  |  |  |  |  |  |  |  |  |  |  |  |  |  |  |  |  |  |  |  |  | *daf-16* | abnormal DAuer Formation |
|  |  |  |  |  |  |  |  |  |  |  |  |  |  |  |  |  |  |  |  |  |  |  |  |  |  |  |  |  |  |  |  |  |  |  |  |  |  | K02C4.3 |  |
|  |  |  |  |  |  |  |  |  |  |  |  |  |  |  |  |  |  |  |  |  |  |  |  |  |  |  |  |  |  |  |  |  |  |  |  |  |  | *snx-13* | Sorting NeXin |
|  |  |  |  |  |  |  |  |  |  |  |  |  |  |  |  |  |  |  |  |  |  |  |  |  |  |  |  |  |  |  |  |  |  |  |  |  |  | *hmp-1* | HuMPback (dorsal lumps) |
|  |  |  |  |  |  |  |  |  |  |  |  |  |  |  |  |  |  |  |  |  |  |  |  |  |  |  |  |  |  |  |  |  |  |  |  |  |  | *hse-5* | Heparan Sulfate-glucuronic acid-5-Epimerase |
|  |  |  |  |  |  |  |  |  |  |  |  |  |  |  |  |  |  |  |  |  |  |  |  |  |  |  |  |  |  |  |  |  |  |  |  |  |  | C34E10.8 |  |
|  |  |  |  |  |  |  |  |  |  |  |  |  |  |  |  |  |  |  |  |  |  |  |  |  |  |  |  |  |  |  |  |  |  |  |  |  |  | F11A3.2 |  |
|  |  |  |  |  |  |  |  |  |  |  |  |  |  |  |  |  |  |  |  |  |  |  |  |  |  |  |  |  |  |  |  |  |  |  |  |  |  | C31E10.5 |  |
|  |  |  |  |  |  |  |  |  |  |  |  |  |  |  |  |  |  |  |  |  |  |  |  |  |  |  |  |  |  |  |  |  |  |  |  |  |  | *wdr-48* | WD Repeat protein |
|  |  |  |  |  |  |  |  |  |  |  |  |  |  |  |  |  |  |  |  |  |  |  |  |  |  |  |  |  |  |  |  |  |  |  |  |  |  | *prmt-3* | PRotein arginine MethylTransferase |
|  |  |  |  |  |  |  |  |  |  |  |  |  |  |  |  |  |  |  |  |  |  |  |  |  |  |  |  |  |  |  |  |  |  |  |  |  |  | C47D12.8 |  |
|  |  |  |  |  |  |  |  |  |  |  |  |  |  |  |  |  |  |  |  |  |  |  |  |  |  |  |  |  |  |  |  |  |  |  |  |  |  | F16D3.4 |  |
|  |  |  |  |  |  |  |  |  |  |  |  |  |  |  |  |  |  |  |  |  |  |  |  |  |  |  |  |  |  |  |  |  |  |  |  |  |  | *gex-3* | Gut on EXterior |
|  |  |  |  |  |  |  |  |  |  |  |  |  |  |  |  |  |  |  |  |  |  |  |  |  |  |  |  |  |  |  |  |  |  |  |  |  |  | R151.8 |  |
|  |  |  |  |  |  |  |  |  |  |  |  |  |  |  |  |  |  |  |  |  |  |  |  |  |  |  |  |  |  |  |  |  |  |  |  |  |  | *riok-3* | RIO Kinase homolog |
|  |  |  |  |  |  |  |  |  |  |  |  |  |  |  |  |  |  |  |  |  |  |  |  |  |  |  |  |  |  |  |  |  |  |  |  |  |  | M05D6.2 |  |
|  |  |  |  |  |  |  |  |  |  |  |  |  |  |  |  |  |  |  |  |  |  |  |  |  |  |  |  |  |  |  |  |  |  |  |  |  |  | T22B7.4 |  |
|  |  |  |  |  |  |  |  |  |  |  |  |  |  |  |  |  |  |  |  |  |  |  |  |  |  |  |  |  |  |  |  |  |  |  |  |  |  | F37D6.2 |  |
|  |  |  |  |  |  |  |  |  |  |  |  |  |  |  |  |  |  |  |  |  |  |  |  |  |  |  |  |  |  |  |  |  |  |  |  |  |  | C39E9.11 |  |
|  |  |  |  |  |  |  |  |  |  |  |  |  |  |  |  |  |  |  |  |  |  |  |  |  |  |  |  |  |  |  |  |  |  |  |  |  |  | *smg-5* | Suppressor with Morphological effect on Genitalia |
|  |  |  |  |  |  |  |  |  |  |  |  |  |  |  |  |  |  |  |  |  |  |  |  |  |  |  |  |  |  |  |  |  |  |  |  |  |  | B0205.1 |  |
|  |  |  |  |  |  |  |  |  |  |  |  |  |  |  |  |  |  |  |  |  |  |  |  |  |  |  |  |  |  |  |  |  |  |  |  |  |  | K08F9.4 |  |
|  |  |  |  |  |  |  |  |  |  |  |  |  |  |  |  |  |  |  |  |  |  |  |  |  |  |  |  |  |  |  |  |  |  |  |  |  |  | B0495.5 |  |
|  |  |  |  |  |  |  |  |  |  |  |  |  |  |  |  |  |  |  |  |  |  |  |  |  |  |  |  |  |  |  |  |  |  |  |  |  |  | *stip-1* | STIP (Septin and Tuftelin Interacting Protein) homolog |
|  |  |  |  |  |  |  |  |  |  |  |  |  |  |  |  |  |  |  |  |  |  |  |  |  |  |  |  |  |  |  |  |  |  |  |  |  |  | *rfp-1* | Ring Finger Protein, UBC associated |
|  |  |  |  |  |  |  |  |  |  |  |  |  |  |  |  |  |  |  |  |  |  |  |  |  |  |  |  |  |  |  |  |  |  |  |  |  |  | W04D2.4 |  |
|  |  |  |  |  |  |  |  |  |  |  |  |  |  |  |  |  |  |  |  |  |  |  |  |  |  |  |  |  |  |  |  |  |  |  |  |  |  | *sacy-1* | Suppressor of ACY-4 sterility |
|  |  |  |  |  |  |  |  |  |  |  |  |  |  |  |  |  |  |  |  |  |  |  |  |  |  |  |  |  |  |  |  |  |  |  |  |  |  | T08A11.1 |  |
|  |  |  |  |  |  |  |  |  |  |  |  |  |  |  |  |  |  |  |  |  |  |  |  |  |  |  |  |  |  |  |  |  |  |  |  |  |  | *him-1* | High Incidence of Males (increased X chromosome loss) |
|  |  |  |  |  |  |  |  |  |  |  |  |  |  |  |  |  |  |  |  |  |  |  |  |  |  |  |  |  |  |  |  |  |  |  |  |  |  | *rrf-3* | RNA-dependent RNA polymerase Family |
|  |  |  |  |  |  |  |  |  |  |  |  |  |  |  |  |  |  |  |  |  |  |  |  |  |  |  |  |  |  |  |  |  |  |  |  |  |  | *suf-1* | SUppressor-of-Forked (Drosophila) homolog |
|  |  |  |  |  |  |  |  |  |  |  |  |  |  |  |  |  |  |  |  |  |  |  |  |  |  |  |  |  |  |  |  |  |  |  |  |  |  | *sna-2* | Small Nuclear RNA (snRNA) Associated protein |
|  |  |  |  |  |  |  |  |  |  |  |  |  |  |  |  |  |  |  |  |  |  |  |  |  |  |  |  |  |  |  |  |  |  |  |  |  |  | *ulp-1* | Ubiquitin-Like Protease |
|  |  |  |  |  |  |  |  |  |  |  |  |  |  |  |  |  |  |  |  |  |  |  |  |  |  |  |  |  |  |  |  |  |  |  |  |  |  | *ztf-18* | Zinc finger putative Transcription Factor family |
|  |  |  |  |  |  |  |  |  |  |  |  |  |  |  |  |  |  |  |  |  |  |  |  |  |  |  |  |  |  |  |  |  |  |  |  |  |  | *secs-1* | SEC(selenocysteine)-tRNA Synthase |
|  |  |  |  |  |  |  |  |  |  |  |  |  |  |  |  |  |  |  |  |  |  |  |  |  |  |  |  |  |  |  |  |  |  |  |  |  |  | B0205.9 |  |
|  |  |  |  |  |  |  |  |  |  |  |  |  |  |  |  |  |  |  |  |  |  |  |  |  |  |  |  |  |  |  |  |  |  |  |  |  |  | *sec-10* | yeast SEC homolog |
|  |  |  |  |  |  |  |  |  |  |  |  |  |  |  |  |  |  |  |  |  |  |  |  |  |  |  |  |  |  |  |  |  |  |  |  |  |  | *grp-1* | GTP exchange factor for ARFs |
|  |  |  |  |  |  |  |  |  |  |  |  |  |  |  |  |  |  |  |  |  |  |  |  |  |  |  |  |  |  |  |  |  |  |  |  |  |  | Y42H9AR.4 |  |
|  |  |  |  |  |  |  |  |  |  |  |  |  |  |  |  |  |  |  |  |  |  |  |  |  |  |  |  |  |  |  |  |  |  |  |  |  |  | *rsd-6* | RNAi Spreading Defective (see also sid) |
|  |  |  |  |  |  |  |  |  |  |  |  |  |  |  |  |  |  |  |  |  |  |  |  |  |  |  |  |  |  |  |  |  |  |  |  |  |  | *fcp-1* | FCP1 (yeast TFIIF-interacting CTD phosphatase subunit) homolog |
|  |  |  |  |  |  |  |  |  |  |  |  |  |  |  |  |  |  |  |  |  |  |  |  |  |  |  |  |  |  |  |  |  |  |  |  |  |  | *nfi-1* | NFI (Nuclear Factor I) family |
|  |  |  |  |  |  |  |  |  |  |  |  |  |  |  |  |  |  |  |  |  |  |  |  |  |  |  |  |  |  |  |  |  |  |  |  |  |  | *capg-1* | CAP-G condensin subunit |
|  |  |  |  |  |  |  |  |  |  |  |  |  |  |  |  |  |  |  |  |  |  |  |  |  |  |  |  |  |  |  |  |  |  |  |  |  |  | *aph-2* | Anterior PHarynx defective |
|  |  |  |  |  |  |  |  |  |  |  |  |  |  |  |  |  |  |  |  |  |  |  |  |  |  |  |  |  |  |  |  |  |  |  |  |  |  | *tag-77* | Temporarily Assigned Gene name |
|  |  |  |  |  |  |  |  |  |  |  |  |  |  |  |  |  |  |  |  |  |  |  |  |  |  |  |  |  |  |  |  |  |  |  |  |  |  | *met-1* | histone METhyltransferase-like |
|  |  |  |  |  |  |  |  |  |  |  |  |  |  |  |  |  |  |  |  |  |  |  |  |  |  |  |  |  |  |  |  |  |  |  |  |  |  | F13C5.2 |  |
|  |  |  |  |  |  |  |  |  |  |  |  |  |  |  |  |  |  |  |  |  |  |  |  |  |  |  |  |  |  |  |  |  |  |  |  |  |  | C31E10.6 |  |
|  |  |  |  |  |  |  |  |  |  |  |  |  |  |  |  |  |  |  |  |  |  |  |  |  |  |  |  |  |  |  |  |  |  |  |  |  |  | ZK228.12 |  |
|  |  |  |  |  |  |  |  |  |  |  |  |  |  |  |  |  |  |  |  |  |  |  |  |  |  |  |  |  |  |  |  |  |  |  |  |  |  | C30F12.2 |  |
|  |  |  |  |  |  |  |  |  |  |  |  |  |  |  |  |  |  |  |  |  |  |  |  |  |  |  |  |  |  |  |  |  |  |  |  |  |  | *inx-7* | INneXin |
|  |  |  |  |  |  |  |  |  |  |  |  |  |  |  |  |  |  |  |  |  |  |  |  |  |  |  |  |  |  |  |  |  |  |  |  |  |  | *linc-139* | Long Intervening Non-Coding RNA |
|  |  |  |  |  |  |  |  |  |  |  |  |  |  |  |  |  |  |  |  |  |  |  |  |  |  |  |  |  |  |  |  |  |  |  |  |  |  | T01A4.5 |  |
|  |  |  |  |  |  |  |  |  |  |  |  |  |  |  |  |  |  |  |  |  |  |  |  |  |  |  |  |  |  |  |  |  |  |  |  |  |  | *str-141* | Seven TM Receptor |
|  |  |  |  |  |  |  |  |  |  |  |  |  |  |  |  |  |  |  |  |  |  |  |  |  |  |  |  |  |  |  |  |  |  |  |  |  |  | *gld-4* | defective in Germ Line Development |
|  |  |  |  |  |  |  |  |  |  |  |  |  |  |  |  |  |  |  |  |  |  |  |  |  |  |  |  |  |  |  |  |  |  |  |  |  |  | F25G6.1 |  |
|  |  |  |  |  |  |  |  |  |  |  |  |  |  |  |  |  |  |  |  |  |  |  |  |  |  |  |  |  |  |  |  |  |  |  |  |  |  | *nhl-2* | NHL (ring finger b-box coiled coil) domain containing |
|  |  |  |  |  |  |  |  |  |  |  |  |  |  |  |  |  |  |  |  |  |  |  |  |  |  |  |  |  |  |  |  |  |  |  |  |  |  | *symk-1* | SYMpleKin cleavage and polyadenylation factor |
|  |  |  |  |  |  |  |  |  |  |  |  |  |  |  |  |  |  |  |  |  |  |  |  |  |  |  |  |  |  |  |  |  |  |  |  |  |  | *obr-4* | Oxysterol Binding protein (OSBP) Related |
|  |  |  |  |  |  |  |  |  |  |  |  |  |  |  |  |  |  |  |  |  |  |  |  |  |  |  |  |  |  |  |  |  |  |  |  |  |  | *smk-1* | SMEK (Dictyostelium Suppressor of MEK null) homolog |
|  |  |  |  |  |  |  |  |  |  |  |  |  |  |  |  |  |  |  |  |  |  |  |  |  |  |  |  |  |  |  |  |  |  |  |  |  |  | *letm-1* | LETM1 (Leucine zipper, EF-hand, TransMembrane mitochondrial protein) homolog |
|  |  |  |  |  |  |  |  |  |  |  |  |  |  |  |  |  |  |  |  |  |  |  |  |  |  |  |  |  |  |  |  |  |  |  |  |  |  | *klp-12* | Kinesin-Like Protein |
|  |  |  |  |  |  |  |  |  |  |  |  |  |  |  |  |  |  |  |  |  |  |  |  |  |  |  |  |  |  |  |  |  |  |  |  |  |  | T05A12.3 |  |
|  |  |  |  |  |  |  |  |  |  |  |  |  |  |  |  |  |  |  |  |  |  |  |  |  |  |  |  |  |  |  |  |  |  |  |  |  |  | F59A2.2 |  |
|  |  |  |  |  |  |  |  |  |  |  |  |  |  |  |  |  |  |  |  |  |  |  |  |  |  |  |  |  |  |  |  |  |  |  |  |  |  | C06A5.3 |  |
|  |  |  |  |  |  |  |  |  |  |  |  |  |  |  |  |  |  |  |  |  |  |  |  |  |  |  |  |  |  |  |  |  |  |  |  |  |  | F25G6.9 |  |
|  |  |  |  |  |  |  |  |  |  |  |  |  |  |  |  |  |  |  |  |  |  |  |  |  |  |  |  |  |  |  |  |  |  |  |  |  |  | *zyg-12* | ZYGote defective : embryonic lethal |
|  |  |  |  |  |  |  |  |  |  |  |  |  |  |  |  |  |  |  |  |  |  |  |  |  |  |  |  |  |  |  |  |  |  |  |  |  |  | C14A4.3 |  |
|  |  |  |  |  |  |  |  |  |  |  |  |  |  |  |  |  |  |  |  |  |  |  |  |  |  |  |  |  |  |  |  |  |  |  |  |  |  | *hpo-27* | Hypersensitive to POre-forming toxin |
|  |  |  |  |  |  |  |  |  |  |  |  |  |  |  |  |  |  |  |  |  |  |  |  |  |  |  |  |  |  |  |  |  |  |  |  |  |  | *ocrl-1* | OCRL (Lowe's oculocerebrorenal syndrome protein) homolog |
|  |  |  |  |  |  |  |  |  |  |  |  |  |  |  |  |  |  |  |  |  |  |  |  |  |  |  |  |  |  |  |  |  |  |  |  |  |  | F55A12.5 |  |
|  |  |  |  |  |  |  |  |  |  |  |  |  |  |  |  |  |  |  |  |  |  |  |  |  |  |  |  |  |  |  |  |  |  |  |  |  |  | *wnk-1* | mammalian WNK-type protein kinase homolog |
|  |  |  |  |  |  |  |  |  |  |  |  |  |  |  |  |  |  |  |  |  |  |  |  |  |  |  |  |  |  |  |  |  |  |  |  |  |  | ZK792.5 |  |
|  |  |  |  |  |  |  |  |  |  |  |  |  |  |  |  |  |  |  |  |  |  |  |  |  |  |  |  |  |  |  |  |  |  |  |  |  |  | T28F4.4 |  |
|  |  |  |  |  |  |  |  |  |  |  |  |  |  |  |  |  |  |  |  |  |  |  |  |  |  |  |  |  |  |  |  |  |  |  |  |  |  | *crn-3* | Cell-death-Related Nuclease |
|  |  |  |  |  |  |  |  |  |  |  |  |  |  |  |  |  |  |  |  |  |  |  |  |  |  |  |  |  |  |  |  |  |  |  |  |  |  | T28C6.7 |  |
|  |  |  |  |  |  |  |  |  |  |  |  |  |  |  |  |  |  |  |  |  |  |  |  |  |  |  |  |  |  |  |  |  |  |  |  |  |  | *dcaf-1* | DDB1 and CUL4-Associated Factor (DCAF1/VprBP) homolog |
|  |  |  |  |  |  |  |  |  |  |  |  |  |  |  |  |  |  |  |  |  |  |  |  |  |  |  |  |  |  |  |  |  |  |  |  |  |  | C16H3.3 |  |
|  |  |  |  |  |  |  |  |  |  |  |  |  |  |  |  |  |  |  |  |  |  |  |  |  |  |  |  |  |  |  |  |  |  |  |  |  |  | *nipi-3* | No Induction of Peptide after Drechmeria Infection |
|  |  |  |  |  |  |  |  |  |  |  |  |  |  |  |  |  |  |  |  |  |  |  |  |  |  |  |  |  |  |  |  |  |  |  |  |  |  | T11G6.5 |  |
|  |  |  |  |  |  |  |  |  |  |  |  |  |  |  |  |  |  |  |  |  |  |  |  |  |  |  |  |  |  |  |  |  |  |  |  |  |  | F44E7.4 |  |

### Phenotypes enriched

|  |  |  |  |
| --- | --- | --- | --- |
| **Group name** | **Number in cluster** | **Enrichment** | **FDR corrected p** |
| organ system development variant (RNAi) | 52 | 2.46 | 5.24e-06 |
| fertility variant | 73 | 1.99 | 2.02e-05 |
| reproductive system physiology variant | 73 | 1.99 | 2.12e-05 |
| sterile | 68 | 2.03 | 3.31e-05 |
| physiology variant | 103 | 1.71 | 3.36e-05 |
| fertility reduced | 68 | 2.02 | 4.18e-05 |
| organ system physiology variant | 73 | 1.95 | 4.77e-05 |
| gene expression variant (RNAi) | 77 | 1.87 | 9.09e-05 |
| fertility reduced (RNAi) | 93 | 1.73 | 1.28e-04 |
| protruding vulva | 12 | 7.33 | 1.61e-04 |
| sterile (RNAi) | 92 | 1.71 | 2.04e-04 |
| slow growth (RNAi) | 90 | 1.72 | 2.38e-04 |
| development variant (RNAi) | 164 | 1.40 | 4.68e-04 |
| organism development variant | 86 | 1.72 | 4.68e-04 |
| development variant | 96 | 1.65 | 5.75e-04 |
| organism development variant (RNAi) | 155 | 1.42 | 6.03e-04 |
| morphology variant (RNAi) | 96 | 1.64 | 7.87e-04 |
| growth variant (RNAi) | 120 | 1.51 | 1.07e-03 |
| cell development variant (RNAi) | 55 | 1.97 | 1.60e-03 |
| lethal | 77 | 1.71 | 2.20e-03 |
| progeny variant (RNAi) | 44 | 2.15 | 2.35e-03 |
| sterile progeny (RNAi) | 44 | 2.15 | 2.35e-03 |
| transgene expression variant (RNAi) | 67 | 1.80 | 2.39e-03 |
| organ system morphology variant (RNAi) | 58 | 1.89 | 3.04e-03 |
| organ system physiology variant (RNAi) | 99 | 1.54 | 6.27e-03 |
| reproductive system morphology variant | 17 | 3.65 | 6.53e-03 |
| intestinal development variant (RNAi) | 15 | 4.02 | 7.11e-03 |
| fertility variant (RNAi) | 98 | 1.53 | 7.44e-03 |
| reproductive system physiology variant (RNAi) | 98 | 1.53 | 9.16e-03 |
| vulva morphology variant | 13 | 4.27 | 1.44e-02 |
| hermaphrodite reproductive system morphology variant | 13 | 4.23 | 1.57e-02 |
| lethal (RNAi) | 131 | 1.39 | 1.66e-02 |
| protein expression variant (RNAi) | 20 | 2.97 | 1.81e-02 |
| pattern of transgene expression variant (RNAi) | 54 | 1.80 | 1.97e-02 |
| excess intestinal cells (RNAi) | 11 | 4.75 | 2.27e-02 |
| Variant | 114 | 1.43 | 2.46e-02 |
| embryonic lethal (RNAi) | 119 | 1.41 | 2.88e-02 |
| organism homeostasis metabolism variant (RNAi) | 156 | 1.32 | 2.99e-02 |
| embryonic development variant (RNAi) | 119 | 1.40 | 3.75e-02 |
| behavior variant (RNAi) | 71 | 1.60 | 4.06e-02 |
| organism behavior variant (RNAi) | 71 | 1.60 | 4.06e-02 |
| level of protein expression variant (RNAi) | 9 | 5.41 | 4.10e-02 |
| endocytic transport defect (RNAi) | 39 | 1.97 | 4.27e-02 |
| endocytic transport variant (RNAi) | 39 | 1.96 | 4.65e-02 |
| cell physiology variant (RNAi) | 69 | 1.60 | 4.73e-02 |

### Anatomy terms enriched

none found

### GO terms enriched

|  |  |  |
| --- | --- | --- |
| **GO term** | **Number of genes** | **FDR-corrected p-value** |
| reproduction | 96 | 5.5e-09 |
| helicase activity | 16 | 1.1e-07 |
| embryo development ending in birth or egg hatching | 120 | 2.5e-06 |
| cellular component organization | 73 | 3.3e-06 |
| ATP binding | 44 | 9.7e-06 |
| adenyl nucleotide binding | 44 | 2.0e-05 |
| multicellular organismal process | 147 | 5.4e-05 |
| nucleus | 68 | 5.8e-05 |
| multicellular organismal development | 78 | 9.7e-05 |
| ATP-dependent helicase activity | 10 | 3.0e-04 |
| anion binding | 53 | 3.1e-04 |
| ribonucleoside binding | 46 | 3.1e-04 |
| protein ubiquitination | 11 | 5.8e-04 |
| sex differentiation | 47 | 6.0e-04 |
| purine nucleoside binding | 45 | 6.5e-04 |
| carbohydrate derivative binding | 46 | 7.5e-04 |
| purine ribonucleotide binding | 45 | 7.5e-04 |
| membrane-bounded organelle | 84 | 1.5e-03 |
| nucleic acid binding | 26 | 1.6e-03 |
| regulation of growth | 74 | 1.8e-03 |
| anatomical structure development | 84 | 1.9e-03 |
| multicellular organismal reproductive process | 44 | 2.8e-03 |
| chromatin binding | 6 | 2.8e-03 |
| organ development | 34 | 3.3e-03 |
| positive regulation of biological process | 69 | 3.6e-03 |
| protein modification by small protein conjugation or removal | 11 | 3.7e-03 |
| reproductive structure development | 36 | 4.4e-03 |
| intracellular organelle | 93 | 5.4e-03 |
| positive regulation of growth rate | 62 | 7.3e-03 |
| hermaphrodite genitalia development | 38 | 8.8e-03 |
| body morphogenesis | 34 | 9.9e-03 |
| nucleotide binding | 48 | 1.2e-02 |
| protein serine/threonine kinase activity | 14 | 1.9e-02 |
| organelle fission | 25 | 1.9e-02 |
| locomotion | 68 | 1.9e-02 |
| chromosome organization | 14 | 2.2e-02 |
| intracellular part | 19 | 2.3e-02 |
| response to UV | 4 | 3.6e-02 |
| cell death | 29 | 4.2e-02 |
| germ cell development | 14 | 4.2e-02 |

### Expression clusters enriched

|  |  |  |  |
| --- | --- | --- | --- |
| **Group name** | **Number in cluster** | **Enrichment** | **FDR corrected p** |
| Genes down regulated in alg-1(gk214) comparing to in N2. | 103 | 10.76 | 1.58e-74 |
| TGF- Dauer pathway adult transcriptional targets. Results obtained by comparing the microarray results of the dauer-constitutive mutants daf-7(e1372), daf-7(m62), and daf-1(m40) with dauer-defective mutants daf-3(mgDf90), daf-5(e1386), and daf-7(e1372);daf-3(mgDf90) double mutants at the permissive temperature, 20C, on the first day of adulthood. WBPaper00031040:TGF-beta\_adult\_downregulated | 235 | 2.22 | 7.88e-40 |
| Caenorhabditis elegans Genes with expression levels changed significantly after treatment of Xenorhabdus nematophila. | 307 | 1.75 | 9.07e-39 |
| Gene significantly down-regulated by treatment with 2.0mM of HuminFeed until young adult stage (3 days), with a minimum fold change in gene expression of 0.8. | 145 | 3.13 | 1.89e-35 |
| Genes significantly enriched (> 2x, FDR < 5%) in a particular cell-type versus a reference sample of all cells at the same stage. WBPaper00037950:AVA-neuron\_embryo\_enriched | 77 | 5.71 | 2.41e-33 |
| Maternal class (M): genes that are called present in at least one of the three PC6 replicates. | 269 | 1.83 | 4.64e-33 |
| Genes expressed in embryonic motor neurons (identified by unc-4::GFP expressing cells). | 267 | 1.75 | 9.36e-29 |
| Germline-enriched and sex-biased expression profile cluster F. | 81 | 4.55 | 3.94e-28 |
| FBF-associated probe sets (FDR <2.25%) | 193 | 2.03 | 3.82e-24 |
| Genes down-regulated after 300 um Tannic acid treatment. Fold change < 0.8. | 114 | 2.94 | 6.40e-24 |
| Maternal-embryonic class (ME): genes that are in the intersection of the maternal and embryonic classes. | 148 | 2.22 | 4.24e-20 |
| Genes significantly enriched (> 2x, FDR < 5%) in a particular cell-type versus a reference sample of all cells at the same stage. WBPaper00037950:all-neurons\_embryo\_enriched | 55 | 4.85 | 1.60e-19 |
| hermaphrodite sex-enriched | 42 | 6.13 | 3.31e-18 |
| Caenorhabditis elegans Genes with expression levels changed significantly after treatment of Bacillus thurigiensis DB27. | 204 | 1.69 | 9.72e-16 |
| Genes expressed in N2. | 304 | 1.36 | 7.16e-15 |
| Embryonic class (E): genes that significantly increase in abundance at some point during embryogenesis. | 165 | 1.84 | 1.43e-14 |
| Genes that showed increased expression after 24 hours of infection by fungi Drechmeria coniospora. WBPaper00032031:DConiospora\_upregulated\_cDNA\_24h | 46 | 4.40 | 2.45e-14 |
| Genes that showed expression levels higher than the corresponding reference sample (embryonic 24hr reference). WBPaper00037950:AVA-neuron\_expressed | 191 | 1.66 | 3.89e-13 |
| mixed oogenesis/somatic | 51 | 3.20 | 1.39e-10 |
| Caenorhabditis elegans Genes with expression levels changed significantly after treatment of Serratia marcescens. | 153 | 1.72 | 1.78e-10 |
| Genes with expression enriched in PVD and OLL neurons. Data sets were normalized by RMA and transcripts showing relative PVD enrichment (>= 1.5X) vs. the reference sample were identified by SAM analysis (False Discovery Rate, FDR < 1%). | 109 | 1.99 | 3.44e-10 |
| Gene significantly up-regulated by treatment with 2.0mM of HuminFeed until older adult stage (11 days), with a minimum fold change in gene expression of 1.25. | 51 | 2.99 | 1.74e-09 |
| Genes predicted to be upregulated more than 2.0 fold in (AFD+AWB) datasets as compared to unsorted whole embryonic cells dataset. | 56 | 2.75 | 3.61e-09 |
| Maternal-embryonic transient class (MET): genes that are in the intersection of the maternal and embryonic transient classes. | 59 | 2.61 | 7.05e-09 |
| Genes that showed expression levels higher than the corresponding reference sample (embryonic 0hr reference). WBPaper00037950:BAG-neuron\_expressed | 177 | 1.53 | 2.08e-08 |
| Genes up or down regulated by 10e-07M of progesterone. The normalized values used were G/R ratio > 2.6 for up-regulation and G/R ratio < 0.38 for down-regulation, which corresponds to 1.39 and -1.39 log(base2) G/R ratio, respectively. | 141 | 1.65 | 4.56e-08 |
| Developmentally modulated gene cluster. cgc4386\_cluster\_5\_1 | 23 | 5.25 | 5.62e-08 |
| Genes significantly enriched (> 2x, FDR < 5%) in a particular cell-type versus a reference sample of all cells at the same stage. WBPaper00037950:GABAergic-motor-neurons\_embryo\_enriched | 32 | 3.52 | 4.24e-07 |
| Genes up or down regulated by 10e-09M of testosterone. The normalized values used were G/R ratio > 2.6 for up-regulation and G/R ratio < 0.38 for down-regulation, which corresponds to 1.39 and -1.39 log(base2) G/R ratio, respectively. | 147 | 1.57 | 4.88e-07 |
| Expression Pattern Group B, enriched for genes involved in embryonic development. These patterns have in common that they all have genes of which the expression goes up after the juvenile stage. The expression of the genes in these patterns remains high or even goes up after reproduction. | 74 | 2.07 | 6.26e-07 |
| Genes that showed expression levels higher than the corresponding reference sample (embryonic 24hr reference). WBPaper00037950:GABAergic-motor-neurons\_expressed | 254 | 1.30 | 1.11e-06 |
| Potental DAF-12 target genes identified by ChIP-chip analysis performed on strain ALF4 [daf-12 | 128 | 1.62 | 1.36e-06 |
| 948 reproductively enriched mRNAs that co-immunoprecipitate with GLD-1. To identify GLD-1 mRNA targets, authors performed immunoprecipitation (IP) of GLD-1, followed by microarray analysis of the co-IPed mRNAs (RIP-chip). Extracts from young adult transgenic worms expressing a rescuing FLAG and GFP-tagged GLD-1, hereafter referred to as tagged GLD-1, were subjected to IP in triplicate with anti-FLAG (aFLAG IP) or anti-MYC (aMYC IP) antibodies as controls. Comparison of aFLAG IP versus aMYC IP to input revealed a large population of GLD-1-associated transcripts. Authors additionally performed complementary aFLAG IPs upon worms expressing either tagged GLD-1(GGF IP) or non-tagged GLD-1(N2 IP). Comparing transcript IP-enrichment values from both approaches revealed a correlation of 0.96, which indicated high reproducibility of GLD-1 association with specific mRNAs even on a quantitative level. | 53 | 2.38 | 2.15e-06 |
| Germline-enriched and sex-biased expression profile cluster E. | 64 | 2.13 | 3.06e-06 |
| oogenesis-enriched | 59 | 2.22 | 3.13e-06 |
| Genes differentially expressed under EtBr treatment and UVC exposure vs under UVC exposure but without EtBr treatment at the -3h timepoint (3 h after the third UVC dose (51h), which is also 3 h after being placed on food). | 103 | 1.73 | 3.47e-06 |
| Developmentally modulated gene cluster. cgc4386\_cluster\_6\_3 | 20 | 4.81 | 3.50e-06 |
| Embryonic (E) subclasses are based on the earliest significant increase(abbreviated pi for primary increase). [cgc5767]:expression\_class\_E\_pi(23\_min) | 53 | 2.33 | 3.91e-06 |
| Gene significantly down-regulated by treatment with 0.2mM of HuminFeed until young adult stage (3 days), with a minimum fold change in gene expression of 0.8. | 29 | 3.28 | 1.08e-05 |
| Genes differentially expressed in control vs after UVC exposure and EtBr treatment at the 3h timepoint (3 h after the third UVC dose (51h), which is also 3 h after being placed on food). | 144 | 1.49 | 2.45e-05 |
| Strictly maternal class (SM): genes that are the subset of maternal genes that are not also classified as embryonic. | 126 | 1.55 | 2.65e-05 |
| Genes that showed higher expression in N2 than in DR1350. | 71 | 1.90 | 3.94e-05 |
| Embryonic transient class (ET): genes that are the subset of embryonic genes in which the latest significant increase is earlier than their latest significant decrease. | 65 | 1.96 | 4.78e-05 |
| C-lineage related expression profile. WBPaper00025032:cluster\_87 | 7 | 15.44 | 6.73e-05 |
| Germline-intrinsic transcripts. | 62 | 1.96 | 9.67e-05 |
| C-lineage related expression profile. WBPaper00025032:cluster\_76 | 7 | 14.63 | 1.01e-04 |
| Expression Pattern Group H, enriched for genes involved in embryonic development. These patterns have in common that they all have genes of which the expression goes up after the juvenile stage. The expression of the genes in these patterns remains high or even goes up after reproduction. | 52 | 2.11 | 1.12e-04 |
| Gene significantly down-regulated by treatment with 2.0mM of HuminFeed Hydroquinone until young adult stage (3 days), with a minimum fold change in gene expression of 0.8. | 39 | 2.36 | 2.32e-04 |
| Genome-wide analysis of developmental and sex-regulated gene expression profile. cgc4489\_group\_1 | 29 | 2.71 | 4.65e-04 |
| Genes downregulated on Comamonas DA1877 relative to E. coli OP50, Gravid adult stage | 28 | 2.70 | 7.07e-04 |
| Developmentally modulated gene cluster. cgc4386\_cluster\_5\_2 | 12 | 5.12 | 1.27e-03 |
| Differentially expressed genes during worm lifespan. Medoid 1 Fig.4. | 25 | 2.75 | 1.72e-03 |
| Genes up or down regulated by 10e-09M of progesterone. The normalized values used were G/R ratio > 2.6 for up-regulation and G/R ratio < 0.38 for down-regulation, which corresponds to 1.39 and -1.39 log(base2) G/R ratio, respectively. | 323 | 1.13 | 3.05e-03 |
| Embryonic (E) subclasses are based on the earliest significant increase(abbreviated pi for primary increase). [cgc5767]:expression\_class\_E\_pi(41\_min) | 24 | 2.67 | 3.98e-03 |
| Genes upregulated in sma-2;fem-1 oocytes vs. fem-1 oocytes. | 25 | 2.58 | 4.47e-03 |
| Significantly downregulated genes from cyc-1(RNAi) microarrays using SAM algorithm with an FDR < 0.1 from adult-only chips. | 119 | 1.42 | 5.37e-03 |
| Embryonic transient (ET) subclasses are based on time of max abundance. [cgc5767]:expression\_class\_ET\_max(53\_min) | 19 | 2.99 | 6.30e-03 |
| Genome-wide analysis of developmental and sex-regulated gene expression profile. cgc4489\_group\_4 | 26 | 2.36 | 1.24e-02 |
| A complete list of the genes that showed differential expression in a slr-2 mutant strain. | 69 | 1.59 | 1.45e-02 |
| Differentially expressed genes during worm lifespan. Medoid 3 Fig.4. | 15 | 3.20 | 1.86e-02 |
| C-lineage related expression profile. WBPaper00025032:cluster\_39 | 6 | 7.01 | 4.05e-02 |
| C-lineage related expression profile. WBPaper00025032:cluster\_65 | 5 | 9.02 | 4.06e-02 |
| Genes significantly enriched (> 2x, FDR < 5%) in a particular cell-type versus a reference sample of all cells at the same stage. WBPaper00037950:bodywall-muscle\_larva\_enriched | 49 | 1.69 | 4.32e-02 |
| Maternal degradation (MD) subclasses are based on the earliest significant decrease (abbreviated pd for primary decrease). [cgc5767]:expression\_class\_MD\_pd(53\_min) | 35 | 1.90 | 4.56e-02 |

### Motifs enriched

|  |  |  |  |  |  |
| --- | --- | --- | --- | --- | --- |
| **Motif** | **Logo** | **Possible orthologs** | **Number of motifs in cluster** | **Enrichment** | **FDR corrected p** |
| pTH3796 |  | let-381 fkh-10 lin-31 | 266 | 1.37 | 4.0e-10 |
| sqz\_SANGER\_5\_FBgn0010768 |  | mel-28 (0.8) fkh-7 lin-29 | 325 | 1.25 | 6.6e-10 |
| Foxl1\_2809 |  | daf-16 (0.89) fkh-7 pha-4 let-381 fkh-8 fkh-10 lin-31 | 261 | 1.37 | 8.2e-10 |
| FOXC1\_3 |  | let-381 lin-31 | 288 | 1.30 | 2.8e-09 |
| FOXJ3\_1 |  | daf-16 (0.89) fkh-7 let-381 fkh-8 fkh-10 lin-31 | 291 | 1.30 | 3.1e-09 |
| MA0543.1 |  | eor-1 (0.83) daf-8 (0.58) | 275 | 1.32 | 8.2e-09 |
| pTH9958 |  | ztf-6 (0.64) ztf-2 | 254 | 1.35 | 1.3e-08 |
| pTH9097 |  | Y116A8C.22 | 301 | 1.26 | 2.2e-08 |
| pTH9335 |  | mel-28 (0.8) | 269 | 1.32 | 2.6e-08 |
| pTH9180 |  | mel-28 (0.8) let-381 mef-2 Y116A8C.22 Y61A9LA.9 | 316 | 1.23 | 3.1e-08 |
| pTH10797 |  | lin-29 K11D2.4 | 317 | 1.22 | 1.2e-07 |
| pTH10696 |  | Y44A6D.3 | 103 | 1.86 | 1.3e-07 |
| FOXD3\_1 |  | let-381 hmg-12 hmbx-1 lin-39 lin-31 ceh-53 Y116A8C.22 | 237 | 1.36 | 1.3e-07 |
| ONECUT3\_1 |  | ceh-48 (0.63) dsc-1 | 276 | 1.29 | 1.3e-07 |
| pTH9254 |  | mel-28 (0.8) | 286 | 1.27 | 1.7e-07 |
| V$POU3F2\_01 |  | ceh-18 dmd-3 | 263 | 1.30 | 2.5e-07 |
| pTH9380 |  | mel-28 (0.8) | 292 | 1.25 | 2.5e-07 |
| ARI3A\_do |  | cfi-1 | 308 | 1.23 | 3.0e-07 |
| POU3F3\_2 |  | ceh-18 unc-86 | 250 | 1.32 | 4.3e-07 |
| MA0536.1 |  | elt-1 | 185 | 1.46 | 4.5e-07 |
| exd\_FlyReg\_FBgn0000611 |  | ceh-20 cfi-1 let-381 | 309 | 1.22 | 4.5e-07 |
| POU3F3\_1 |  | ceh-18 ceh-6 sox-4 tbp-1 | 276 | 1.27 | 5.7e-07 |
| pTH9260 |  | mel-28 (0.8) | 299 | 1.23 | 7.4e-07 |
| pTH5169 |  | cfi-1 | 264 | 1.28 | 1.0e-06 |
| V$MEF2\_03 |  | mef-2 | 237 | 1.33 | 1.3e-06 |
| pTH9957 |  | daf-16 (0.89) irx-1 fkh-9 | 275 | 1.26 | 1.3e-06 |
| pTH9242 |  | mel-28 (0.8) | 287 | 1.24 | 1.8e-06 |
| Dll\_Cell\_FBgn0000157 |  | pal-1 ceh-12 ceh-43 lin-39 | 240 | 1.32 | 1.9e-06 |
| HXD4\_f1 |  | alr-1 cog-1 ceh-31 ceh-9 ceh-8 ceh-43 lin-39 | 242 | 1.31 | 2.1e-06 |
| pnr\_SANGER\_5\_FBgn0003117 |  | elt-1 | 267 | 1.27 | 2.1e-06 |
| V$OCT1\_06 |  | ceh-18 | 248 | 1.29 | 3.4e-06 |
| Pax6\_3838 |  | ceh-16 alr-1 lim-4 lim-7 ceh-18 cog-1 pal-1 ceh-45 lim-6 egl-5 pha-2 ceh-43 lin-39 ceh-23 ZC123.3 T13C5.4 | 191 | 1.40 | 3.5e-06 |
| MA0135.1 |  | lim-7 cfi-1 unc-86 php-3 | 238 | 1.31 | 4.0e-06 |
| Abd-A\_FlyReg\_FBgn0000014 |  | alr-1 ceh-45 ceh-1 lin-39 | 219 | 1.34 | 4.1e-06 |
| BARHL2\_3 |  | ceh-31 ceh-1 ceh-14 ceh-43 | 227 | 1.33 | 4.3e-06 |
| pTH9125 |  | egl-13 sox-4 | 267 | 1.26 | 4.3e-06 |
| Tbp\_pr781 |  | tbp-1 | 239 | 1.30 | 5.2e-06 |
| pTH9082 |  | mab-23 (0.55) | 273 | 1.25 | 5.6e-06 |
| V$FREAC7\_01 |  | lin-31 | 233 | 1.31 | 7.2e-06 |
| Hoxd9\_2 |  | pal-1 ceh-24 php-3 lin-39 | 239 | 1.30 | 7.7e-06 |
| pTH9237 |  | mel-28 (0.8) | 249 | 1.28 | 7.7e-06 |
| Zfp161\_2858 |  | pzf-1 | 108 | 1.68 | 8.0e-06 |
| MA0049.1 |  | hbl-1 (0.75) php-3 lin-39 | 284 | 1.23 | 8.1e-06 |
| pTH9177 |  | hsf-1 (0.63) F10B5.3 | 254 | 1.27 | 9.7e-06 |
| Blimp-1\_SANGER\_5\_FBgn0035625 |  | blmp-1 | 270 | 1.25 | 1.1e-05 |
| SPDEF\_3 |  | lin-1 lin-39 nhr-100 | 259 | 1.26 | 1.2e-05 |
| pTH6641 |  | lin-31 | 235 | 1.30 | 1.3e-05 |
| V$BRN2\_01 |  | ceh-18 | 256 | 1.26 | 1.9e-05 |
| pTH9901 |  | pal-1 ceh-24 php-3 lin-39 D1005.3 T27F2.4 | 220 | 1.32 | 2.1e-05 |
| pTH1014 |  | atf-5 | 126 | 1.56 | 2.2e-05 |
| pTH2846 |  | lin-31 | 235 | 1.29 | 2.6e-05 |
| V$TBP\_01 |  | tbp-1 | 228 | 1.30 | 2.7e-05 |
| HXD10\_f1 |  | php-3 | 263 | 1.24 | 3.2e-05 |
| pTH9709 |  | die-1 (0.76) | 269 | 1.23 | 3.8e-05 |
| pTH6591 |  | lin-31 | 242 | 1.27 | 4.9e-05 |
| pTH9353 |  | ceh-51 | 149 | 1.45 | 4.9e-05 |
| MA0541.1 |  | efl-1 (0.78) | 160 | 1.42 | 6.3e-05 |
| pTH5250 |  | C48E7.11 | 186 | 1.35 | 8.5e-05 |
| Hoxd11\_3873 |  | php-3 | 183 | 1.36 | 9.5e-05 |
| pTH3477 |  | daf-16 (0.89) | 225 | 1.27 | 1.7e-04 |
| pTH9256 |  | ceh-18 | 192 | 1.32 | 1.9e-04 |
| V$PBX1\_01 |  | ceh-20 lin-39 | 236 | 1.25 | 2.0e-04 |
| Sox17\_2837 |  | sox-4 | 225 | 1.27 | 2.1e-04 |
| CART1\_1 |  | alr-1 cfi-1 ceh-14 ZC204.2 | 213 | 1.28 | 2.5e-04 |
| pTH5423 |  | klf-2 | 142 | 1.43 | 3.0e-04 |
| pTH9297 |  | ceh-18 | 221 | 1.27 | 3.4e-04 |
| pTH8982 |  | ceh-48 (0.63) | 71 | 1.76 | 4.1e-04 |
| rn\_SOLEXA\_5\_FBgn0259172 |  | lin-29 | 280 | 1.19 | 4.4e-04 |
| pTH5916 |  | efl-2 (0.62) | 119 | 1.48 | 4.6e-04 |
| pTH3831 |  | ces-2 atf-2 Y51H4A.4 C48E7.11 F23F12.9 C01B12.2 | 143 | 1.41 | 4.6e-04 |
| MA0547.1 |  | skn-1 (0.66) | 267 | 1.20 | 5.1e-04 |
| CG31670\_SOLEXA\_5\_FBgn0031375 |  | CELE\_Y38H8A.5 | 249 | 1.22 | 6.6e-04 |
| CXXC1\_si |  | F52B11.1 | 156 | 1.37 | 6.9e-04 |
| pTH9951 |  | mex-6 | 271 | 1.19 | 6.9e-04 |
| Lmx1b\_3433 |  | ceh-16 lim-6 | 175 | 1.33 | 7.2e-04 |
| pTH9173 |  | efl-2 (0.62) | 114 | 1.48 | 7.9e-04 |
| MA0482.1 |  | elt-1 | 210 | 1.27 | 8.1e-04 |
| pTH8985 |  | athp-1 (0.69) | 190 | 1.30 | 8.2e-04 |
| MA0538.1 |  | Y5F2A.4 (0.79) hif-1 daf-12 | 47 | 2.01 | 9.0e-04 |
| MA0535.1 |  | daf-8 (0.58) | 134 | 1.41 | 9.4e-04 |
| Sox1\_2631 |  | sox-4 | 201 | 1.28 | 9.7e-04 |
| CEBPE\_f1 |  | C48E7.11 R07H5.10 | 240 | 1.22 | 1.0e-03 |
| pTH9164 |  | ceh-26 | 176 | 1.32 | 1.1e-03 |
| ARNT\_f1 |  | aha-1 (0.81) hlh-27 lin-22 hlh-28 | 119 | 1.45 | 1.2e-03 |
| pTH9137 |  | nhr-65 | 269 | 1.19 | 1.2e-03 |
| pTH7875 |  | mel-28 (0.8) | 186 | 1.29 | 1.3e-03 |
| pTH6497 |  | lin-31 | 220 | 1.24 | 1.3e-03 |
| Hoxd10\_2368 |  | php-3 lin-39 | 190 | 1.29 | 1.4e-03 |
| MA0124.1 |  | ceh-48 (0.63) ceh-24 | 140 | 1.38 | 1.5e-03 |
| Pou2f1\_3081 |  | ceh-18 | 159 | 1.33 | 1.9e-03 |
| Sox4 |  | pop-1 (0.57) nhr-100 | 226 | 1.23 | 1.9e-03 |
| V$FOXJ2\_02 |  | lin-31 | 215 | 1.24 | 1.9e-03 |
| HES1\_f1 |  | lin-22 | 66 | 1.70 | 2.1e-03 |
| pTH8981 |  | pax-3 | 121 | 1.42 | 2.1e-03 |
| Pou3f4\_3773 |  | ceh-6 | 170 | 1.31 | 2.2e-03 |
| Sox11\_2266 |  | pop-1 (0.57) sox-4 gei-3 | 217 | 1.24 | 2.5e-03 |
| pTH5257 |  | C48E7.11 | 154 | 1.33 | 3.5e-03 |
| pTH5119 |  | cfi-1 | 214 | 1.23 | 3.6e-03 |
| pTH9384 |  | cfi-1 | 225 | 1.22 | 3.7e-03 |
| Pou2f3\_3986 |  | ceh-18 | 172 | 1.29 | 3.7e-03 |
| Cdx2\_4272 |  | ceh-13 | 174 | 1.29 | 3.7e-03 |
| MA0102.3 |  | C48E7.11 | 199 | 1.25 | 4.1e-03 |
| PAX5\_1 |  | pax-2 pax-3 | 116 | 1.41 | 4.3e-03 |
| pTH5118 |  | cfi-1 | 232 | 1.20 | 5.3e-03 |
| Mw151 |  | gei-11 C34D1.1 | 241 | 1.19 | 5.5e-03 |
| pTH9381 |  | ceh-18 | 171 | 1.28 | 6.5e-03 |
| pTH9262 |  | lin-54 | 186 | 1.26 | 6.8e-03 |
| Hoxb9\_3413 |  | ceh-24 | 197 | 1.24 | 7.1e-03 |
| Pou3f1\_3819 |  | ceh-6 | 30 | 2.18 | 7.2e-03 |
| pTH5924 |  | nhr-255 | 173 | 1.27 | 7.5e-03 |
| tgo\_sima\_SANGER\_5\_FBgn0015014 |  | aha-1 (0.81) mdl-1 hlh-30 | 116 | 1.39 | 8.0e-03 |
| gl\_FlyReg\_FBgn0004618 |  | ces-1 T22H9.4 C34H4.5 | 219 | 1.21 | 8.1e-03 |
| pTH2885 |  | aha-1 (0.81) mxl-1 hlh-30 lin-22 | 93 | 1.46 | 8.8e-03 |
| V$TAXCREB\_01 |  | crh-1 | 120 | 1.37 | 9.6e-03 |
| Smad3\_3805 |  | daf-8 (0.58) | 119 | 1.37 | 1.1e-02 |
| SOX2\_5 |  | sox-4 | 194 | 1.23 | 1.1e-02 |
| pTH8983 |  | tag-347 | 143 | 1.31 | 1.1e-02 |
| pTH8556 |  | pax-2 | 19 | 2.68 | 1.1e-02 |
| EMX2\_2 |  | ceh-2 | 211 | 1.21 | 1.2e-02 |
| Otp\_3496 |  | alr-1 | 141 | 1.31 | 1.3e-02 |
| pTH4325 |  | ceh-18 | 199 | 1.22 | 1.3e-02 |
| IRX5\_1 |  | irx-1 | 175 | 1.25 | 1.3e-02 |
| pTH9089 |  | ref-2 | 108 | 1.38 | 1.5e-02 |
| V$RFX1\_01 |  | daf-19 (0.52) | 109 | 1.37 | 1.7e-02 |
| MA0028.1 |  | lin-1 | 181 | 1.24 | 1.7e-02 |
| pnt\_SANGER\_5\_FBgn0003118 |  | lin-1 | 200 | 1.21 | 1.9e-02 |
| pTH9215 |  | C34D1.1 | 183 | 1.23 | 2.0e-02 |
| CG8765\_SANGER\_5\_FBgn0036900 |  | H20J04.3 | 196 | 1.21 | 2.2e-02 |
| pTH10038 |  | sox-4 gei-3 | 188 | 1.22 | 2.3e-02 |
| CrebA\_SANGER\_5\_FBgn0004396 |  | C27D6.4 | 125 | 1.32 | 2.3e-02 |
| pTH9096 |  | T07C12.11 | 95 | 1.40 | 2.5e-02 |
| pTH10816 |  | dmd-6 | 221 | 1.18 | 2.5e-02 |
| pTH1739 |  | nhr-255 lin-14 | 172 | 1.24 | 2.7e-02 |
| Eip74EF\_FlyReg\_FBgn0000567 |  | C24A1.2 | 247 | 1.16 | 2.7e-02 |
| CG4854\_SANGER\_10\_FBgn0038766 |  | K11D2.4 | 186 | 1.22 | 3.0e-02 |
| OTX2\_si |  | ceh-45 | 150 | 1.26 | 3.2e-02 |
| RFX3\_1 |  | daf-19 (0.52) | 95 | 1.38 | 3.3e-02 |
| Rfxdc2\_3516 |  | daf-19 (0.52) | 118 | 1.32 | 3.3e-02 |
| Optix\_SOLEXA\_FBgn0025360 |  | ceh-34 | 72 | 1.47 | 3.4e-02 |
| SMAD3\_f1 |  | daf-8 (0.58) | 125 | 1.30 | 3.5e-02 |
| pTH8863 |  | hmg-12 | 161 | 1.24 | 3.6e-02 |
| Hmx2\_3424 |  | ceh-9 | 146 | 1.26 | 3.6e-02 |
| ISL2\_1 |  | lim-7 | 145 | 1.27 | 3.6e-02 |
| pTH3866 |  | hlh-2 (0.58) | 128 | 1.29 | 3.8e-02 |
| CDC5L\_si |  | D1081.8 (0.75) | 187 | 1.21 | 3.8e-02 |
| BARHL2\_4 |  | ceh-31 | 137 | 1.28 | 3.9e-02 |
| MA0386.1 |  | tbp-1 | 345 | 1.08 | 3.9e-02 |
| MEIS1\_f2 |  | ceh-32 | 182 | 1.21 | 4.0e-02 |
| pTH2353 |  | B0310.2 | 82 | 1.42 | 4.1e-02 |
| ss\_tgo\_SANGER\_10\_FBgn0015014 |  | aha-1 (0.81) | 106 | 1.34 | 4.2e-02 |
| pTH9247 |  | C34D1.1 | 119 | 1.31 | 4.2e-02 |
| KLF3\_f1 |  | klf-1 | 22 | 2.17 | 4.2e-02 |
| Gmeb1\_1745 |  | attf-1 | 81 | 1.41 | 4.7e-02 |
| pTH10013 |  | nhr-168 | 87 | 1.39 | 5.0e-02 |
| NHLH1\_1 |  | hlh-15 | 102 | 1.34 | 5.0e-02 |

### Correlated (and anti-correlated) transcription factors

|  |  |
| --- | --- |
| **Transcription factor** | **Correlation** |
| tra-4 | 0.97 |
| C27A12.2 | 0.96 |
| egl-27 | 0.96 |
| ceh-86 | 0.96 |
| cdc-14 | 0.95 |
| T20F7.1 | 0.95 |
| B0261.1 | 0.94 |
| rad-26 | 0.93 |
| sdc-2 | 0.93 |
| swsn-7 | 0.92 |
| nfi-1 | 0.92 |
| gei-17 | 0.92 |
| snpc-4 | 0.92 |
| ada-2 | 0.92 |
| rbr-2 | 0.92 |
| W04D2.4 | 0.91 |
| pqn-21 | 0.91 |
| ztf-8 | 0.91 |
| F33H1.4 | 0.91 |
| ceh-38 | 0.90 |
| chd-7 | 0.90 |
| daf-16 | 0.89 |
| B0019.2 | 0.89 |
| F21A10.2 | 0.89 |
| mep-1 | 0.89 |
| nhr-261 | -0.44 |
| nhr-58 | -0.44 |
| gmeb-3 | -0.45 |
| nhr-264 | -0.46 |
| nhr-260 | -0.46 |
| ccch-2 | -0.47 |
| zip-6 | -0.47 |
| C35D6.4 | -0.48 |
| nhr-87 | -0.51 |
| nhr-14 | -0.52 |
| T26A5.8 | -0.53 |
| nhr-149 | -0.53 |
| nhr-222 | -0.53 |
| nhr-5 | -0.54 |
| grh-1 | -0.54 |
| ceh-7 | -0.55 |
| mxl-2 | -0.55 |
| mbf-1 | -0.57 |
| madf-1 | -0.57 |
| atf-8 | -0.58 |
| mxl-3 | -0.59 |
| Y56A3A.18 | -0.61 |
| nhr-41 | -0.62 |
| sdz-38 | -0.63 |
| nhr-92 | -0.75 |

### ChIP peaks enriched

|  |  |  |  |  |
| --- | --- | --- | --- | --- |
| **Gene** | **Experiment** | **Number of upstream peaks** | **Enrichment** | **FDR corrected p** |
| efl-1 | EFL-1\_Fed-L1-stage-larvae | 216 | 2.96 | 1.7e-55 |
| lsy-2 | LSY-2\_Embryos | 186 | 3.25 | 4.2e-51 |
| efl-1 | EFL-1\_Larvae-L1-stage | 222 | 2.64 | 4.8e-49 |
| lin-35 | LIN-35\_Fed-L1-stage-larvae | 204 | 2.79 | 5.2e-47 |
| dpl-1 | DPL-1\_Fed-L1-stage-larvae | 211 | 2.66 | 7.7e-46 |
| C34F6.9 | C34F6.9\_Larvae-L2-stage | 219 | 2.51 | 2.7e-44 |
| eor-1 | EOR-1\_Larvae-L3-stage | 219 | 2.45 | 2.3e-42 |
| dpl-1 | DPL-1\_Larvae-L4-stage | 232 | 2.30 | 1.9e-41 |
| lsy-2 | LSY-2\_Fed-L1-stage-larvae | 192 | 2.71 | 3.0e-41 |
| W03F9.2 | W03F9.2\_L4-Young-Adult-stage-larvae | 256 | 2.09 | 1.7e-40 |
| hpl-2 | HPL-2\_Fed-L1-stage-larvae | 220 | 2.37 | 2.8e-40 |
| gei-11 | GEI-11\_Fed-L1-stage-larvae | 202 | 2.54 | 3.9e-40 |
| lsy-2 | LSY-2\_Larvae-L1-stage | 217 | 2.35 | 5.6e-39 |
| nfya-1 | NFYA-1\_Larvae-L3-stage | 178 | 2.75 | 3.8e-38 |
| gei-11 | GEI-11\_Larvae-L3-stage | 201 | 2.45 | 2.5e-37 |
| nfya-1 | NFYA-1\_Late-Embryos | 195 | 2.42 | 6.0e-35 |
| ham-1 | HAM-1\_Fed-L1-stage-larvae | 201 | 2.36 | 7.1e-35 |
| efl-1 | EFL-1\_Young-adult | 209 | 2.26 | 3.3e-34 |
| F45C12.2 | F45C12.2\_Fed-L1-stage-larvae | 180 | 2.54 | 5.9e-34 |
| aly-2 | ALY-2\_Fed-L1-stage-larvae | 169 | 2.67 | 6.0e-34 |
| R02D3.7 | R02D3.7\_Larvae-L3-stage | 190 | 2.39 | 3.8e-33 |
| lin-15 | LIN-15B\_Fed-L1-stage-larvae | 138 | 3.11 | 5.3e-33 |
| C01B12.2 | C01B12.2\_Larvae-L2-stage | 219 | 2.11 | 3.9e-32 |
| ceh-39 | CEH-39\_Embryos | 139 | 3.00 | 1.5e-31 |
| ces-1 | CES-1\_Embryos | 198 | 2.25 | 2.3e-31 |
| pes-1 | PES-1\_Larvae-L4-stage | 195 | 2.26 | 7.9e-31 |
| fos-1 | FOS-1\_Fed-L1-stage-larvae | 170 | 2.47 | 4.2e-30 |
| nhr-77 | NHR-77\_Larvae-L4-stage | 224 | 2.00 | 2.5e-29 |
| lin-13 | LIN-13\_Larvae-L2-stage | 151 | 2.61 | 3.0e-28 |
| gei-11 | GEI-11\_Larvae-L2-stage | 155 | 2.54 | 6.4e-28 |
| ceh-38 | CEH-38\_Larvae-L3-stage | 161 | 2.46 | 9.3e-28 |
| C16A3.4 | C16A3.4\_Fed-L1-stage-larvae | 152 | 2.54 | 4.0e-27 |
| ham-1 | HAM-1\_Larvae-L4-stage | 192 | 2.13 | 1.2e-26 |
| nhr-237 | NHR-237\_Embryos | 110 | 3.23 | 1.9e-26 |
| nhr-77 | NHR-77\_Fed-L1-stage-larvae | 177 | 2.21 | 8.8e-26 |
| nhr-6 | NHR-6\_Larvae-L4-stage | 126 | 2.82 | 1.6e-25 |
| nhr-23 | NHR-23\_Larvae-L3-stage | 171 | 2.25 | 2.5e-25 |
| sem-4 | SEM-4\_Larvae-L2-stage | 169 | 2.26 | 3.0e-25 |
| dpl-1 | DPL-1\_Young-adult | 170 | 2.24 | 4.7e-25 |
| pha-4 | PHA-4\_Larvae-L2-stage | 195 | 2.03 | 1.6e-24 |
| nhr-25 | NHR-25\_Larvae-L2-stage | 159 | 2.30 | 5.7e-24 |
| pha-4 | PHA-4\_Larvae-L4-stage | 130 | 2.47 | 3.5e-21 |
| jun-1 | JUN-1\_Larvae-L1-stage | 143 | 2.30 | 7.0e-21 |
| dve-1 | DVE-1\_Late-Embryos | 155 | 2.17 | 1.1e-20 |
| F23B12.7 | F23B12.7\_Young-adult | 123 | 2.53 | 1.2e-20 |
| lsy-2 | LSY-2\_Larvae-L2-stage | 99 | 2.90 | 4.5e-20 |
| F16B12.6 | F16B12.6\_Fed-L1-stage-larvae | 99 | 2.88 | 8.3e-20 |
| nhr-129 | NHR-129\_Larvae-L2-stage | 193 | 1.85 | 3.1e-19 |
| hlh-30 | HLH-30\_Late-Embryos | 94 | 2.77 | 1.6e-17 |
| hlh-30 | HLH-30\_Larvae-L4-stage | 107 | 2.50 | 3.3e-17 |
| zag-1 | ZAG-1\_Fed-L1-stage-larvae | 88 | 2.72 | 8.7e-16 |
| ceh-26 | CEH-26\_Late-Embryonic-stage | 120 | 2.22 | 1.4e-15 |
| alr-1 | ALR-1\_Larvae-L2-stage | 134 | 2.08 | 1.5e-15 |
| R02D3.7 | R02D3.7\_Larvae-L2-stage | 106 | 2.33 | 7.8e-15 |
| nhr-2 | NHR-2\_Embryos | 86 | 2.62 | 1.8e-14 |
| gei-11 | GEI-11\_Young-adult | 86 | 2.58 | 4.1e-14 |
| sax-3 | SAX-3\_Larvae-L4-stage | 158 | 1.83 | 5.2e-14 |
| jun-1 | JUN-1\_Larvae-L4-stage | 115 | 2.14 | 8.9e-14 |
| jun-1 | JUN-1\_Larvae-L3-stage | 106 | 2.18 | 5.1e-13 |
| ceh-38 | CEH-38\_Larvae-L4-stage | 92 | 2.32 | 1.7e-12 |
| nhr-77 | NHR-77\_Larvae-L2-stage | 97 | 2.25 | 1.9e-12 |
| zag-1 | ZAG-1\_Larvae-L2-stage | 115 | 2.00 | 9.0e-12 |
| zag-1 | ZAG-1\_Larvae-L3-stage | 79 | 2.44 | 1.3e-11 |
| F45C12.2 | F45C12.2\_Larvae-L3-stage | 81 | 2.39 | 1.8e-11 |
| sax-3 | SAX-3\_Larvae-L2-stage | 116 | 1.97 | 2.0e-11 |
| fos-1 | FOS-1\_Larvae-L2-stage | 175 | 1.62 | 3.1e-11 |
| nhr-6 | NHR-6\_Larvae-L2-stage | 128 | 1.86 | 3.3e-11 |
| ztf-7 | ZTF-7\_Larvae-L4-stage | 100 | 2.10 | 3.6e-11 |
| pax-1 | PAX-1\_Embryos | 53 | 3.11 | 3.8e-11 |
| nhr-28 | NHR-28\_Larvae-L4-stage | 163 | 1.67 | 4.6e-11 |
| lin-13 | LIN-13\_Larvae-L4-stage | 94 | 2.15 | 6.9e-11 |
| lsy-2 | LSY-2\_Larvae-L4-stage | 76 | 2.39 | 1.2e-10 |
| sea-2 | SEA-2\_Larvae-L3-stage | 60 | 2.70 | 3.1e-10 |
| nhr-76 | NHR-76\_Larvae-L4-stage | 88 | 2.13 | 5.7e-10 |
| nhr-77 | NHR-77\_Larvae-L3-stage | 117 | 1.86 | 6.4e-10 |
| lin-35 | LIN-35\_Starved-L1-stage-larvae | 74 | 2.32 | 8.1e-10 |
| nfya-1 | NFYA-1\_Young-adult | 58 | 2.69 | 8.3e-10 |
| mab-5 | MAB-5\_Larvae-L2-stage | 80 | 2.18 | 2.2e-09 |
| R02D3.7 | R02D3.7\_Larvae-L4-stage | 77 | 2.17 | 7.1e-09 |
| lin-35 | LIN-35\_Young-adult | 71 | 2.25 | 8.6e-09 |
| egl-5 | EGL-5\_Larvae-L3-stage | 109 | 1.83 | 9.8e-09 |
| elt-3 | ELT-3\_Embryos | 99 | 1.90 | 1.1e-08 |
| dve-1 | DVE-1\_Larvae-L4-stage | 100 | 1.87 | 2.3e-08 |
| lin-13 | LIN-13\_Larvae-L1-stage | 50 | 2.62 | 5.3e-08 |
| skn-1 | SKN-1\_Larvae-L3-stage | 69 | 2.17 | 6.9e-08 |
| F45C12.2 | F45C12.2\_Larvae-L2-stage | 60 | 2.33 | 7.4e-08 |
| aly-2 | ALY-2\_Larvae-L3-stage | 81 | 2.00 | 9.3e-08 |
| nhr-11 | NHR-11\_Larvae-L2-stage | 65 | 2.20 | 1.3e-07 |
| sax-3 | SAX-3\_Larvae-L3-stage | 72 | 2.09 | 1.5e-07 |
| fos-1 | FOS-1\_Larvae-L3-stage | 106 | 1.74 | 2.7e-07 |
| nhr-237 | NHR-237\_Larvae-L1-stage | 52 | 2.43 | 2.7e-07 |
| ztf-4 | ZTF-4\_Larvae-L2-stage | 51 | 2.35 | 1.2e-06 |
| lin-15 | LIN-15B\_Larvae-L4-stage | 46 | 2.40 | 3.0e-06 |
| ces-1 | CES-1\_Fed-L1-stage-larvae | 65 | 1.99 | 4.9e-06 |
| unc-62 | UNC-62\_Day-Four-Young-Adult | 91 | 1.74 | 4.9e-06 |
| unc-62 | UNC-62\_Young-adult-Day-4 | 91 | 1.74 | 4.9e-06 |
| zag-1 | ZAG-1\_Larvae-L4-stage | 82 | 1.80 | 5.7e-06 |
| ces-1 | CES-1\_Larvae-L4-stage | 66 | 1.93 | 1.2e-05 |
| fos-1 | FOS-1\_Larvae-L4-stage | 51 | 2.16 | 1.3e-05 |
| unc-62 | UNC-62\_Larvae-L3-stage | 88 | 1.67 | 4.1e-05 |
| ces-1 | CES-1\_Larvae-L3-stage | 52 | 2.04 | 5.3e-05 |
| aha-1 | AHA-1\_Larvae-L4-stage | 47 | 2.12 | 6.3e-05 |
| nhr-76 | NHR-76\_Larvae-L3-stage | 56 | 1.95 | 7.5e-05 |
| unc-62 | UNC-62\_Fed-L1-stage-larvae | 39 | 2.26 | 1.2e-04 |
| pha-4 | PHA-4\_Young-adult | 58 | 1.89 | 1.2e-04 |
| gei-11 | GEI-11\_Embryos | 48 | 2.02 | 1.6e-04 |
| elt-1 | ELT-1\_Larvae-L3-stage | 61 | 1.80 | 3.0e-04 |
| ceh-16 | CEH-16\_Larvae-L2-stage | 51 | 1.90 | 4.4e-04 |
| nhr-28 | NHR-28\_Larvae-L3-stage | 43 | 2.00 | 7.0e-04 |
| nhr-10 | NHR-10\_Larvae-L4-stage | 37 | 2.06 | 1.3e-03 |
| ztf-4 | ZTF-4\_Larvae-L1-stage | 30 | 2.28 | 1.4e-03 |
| sax-3 | SAX-3\_Fed-L1-stage-larvae | 51 | 1.80 | 1.7e-03 |
| ztf-4 | ZTF-4\_Larvae-L3-stage | 32 | 2.09 | 3.4e-03 |
| aly-2 | ALY-2\_Larvae-L2-stage | 32 | 2.07 | 3.9e-03 |
| mab-5 | MAB-5\_Embryos | 25 | 2.24 | 7.0e-03 |
| fkh-2 | FKH-2\_Larvae-L3-stage | 33 | 1.93 | 1.0e-02 |
| aha-1 | AHA-1\_Fed-L1-stage-larvae | 21 | 2.34 | 1.2e-02 |
| unc-62 | UNC-62\_Larvae-L2-stage | 50 | 1.63 | 1.6e-02 |
| ztf-11 | ZTF-11\_Embryos | 23 | 2.02 | 3.9e-02 |
