# Supplementary material for: Overlapping cell population expression profiling and regulatory inference in C. elegans
Source: BMC Genomics. 2016 Feb 29;17:159. doi: 10.1186/s12864-016-2482-z (PMC4772325; doi:10.1186/s12864-016-2482-z)
Supplement: Additional file 13: — Web supplement. (DOC 21 kb) [file 12864_2016_2482_MOESM13_ESM.zip › sortWeb/clusters/hier.300.clusters/244.html]

Cluster 244 

## Cluster 244

### Expression

| cnd-1 rep. 1 | cnd-1 rep. 2 | cnd-1 rep. 3 | pha-4 rep. 1 | pha-4 rep. 2 | pha-4 rep. 3 | ceh-27 | ceh-36 | ceh-6 | F21D5.9 | mir-57 | mls-2 | pal-1 | pros-1 | ttx-3 | unc-130 | hlh-16 | irx-1 | ceh-6 (+) hlh-16 (+) | ceh-6 (+) hlh-16 (-) | ceh-6 (-) hlh-16 (+) | cnd-1 singlets | pha-4 singlets | 0 | 60 | 120 | 150 | 180 | 240 | 330 | 390 | 420 | 480 | 540 | 570 | 600 | 630 | 660 | NAME | Functional description |
| --- | --- | --- | --- | --- | --- | --- | --- | --- | --- | --- | --- | --- | --- | --- | --- | --- | --- | --- | --- | --- | --- | --- | --- | --- | --- | --- | --- | --- | --- | --- | --- | --- | --- | --- | --- | --- | --- | --- | --- |
|  |  |  |  |  |  |  |  |  |  |  |  |  |  |  |  |  |  |  |  |  |  |  |  |  |  |  |  |  |  |  |  |  |  |  |  |  |  | *cutl-26* | CUTiclin-Like |
|  |  |  |  |  |  |  |  |  |  |  |  |  |  |  |  |  |  |  |  |  |  |  |  |  |  |  |  |  |  |  |  |  |  |  |  |  |  | *cal-4* | CALmodulin related genes |
|  |  |  |  |  |  |  |  |  |  |  |  |  |  |  |  |  |  |  |  |  |  |  |  |  |  |  |  |  |  |  |  |  |  |  |  |  |  | *glb-25* | GLoBin related |
|  |  |  |  |  |  |  |  |  |  |  |  |  |  |  |  |  |  |  |  |  |  |  |  |  |  |  |  |  |  |  |  |  |  |  |  |  |  | F25F8.1 |  |
|  |  |  |  |  |  |  |  |  |  |  |  |  |  |  |  |  |  |  |  |  |  |  |  |  |  |  |  |  |  |  |  |  |  |  |  |  |  | *che-7* | abnormal CHEmotaxis |
|  |  |  |  |  |  |  |  |  |  |  |  |  |  |  |  |  |  |  |  |  |  |  |  |  |  |  |  |  |  |  |  |  |  |  |  |  |  | *glb-6* | GLoBin related |
|  |  |  |  |  |  |  |  |  |  |  |  |  |  |  |  |  |  |  |  |  |  |  |  |  |  |  |  |  |  |  |  |  |  |  |  |  |  | *unc-13* | UNCoordinated |
|  |  |  |  |  |  |  |  |  |  |  |  |  |  |  |  |  |  |  |  |  |  |  |  |  |  |  |  |  |  |  |  |  |  |  |  |  |  | *pkc-2* | Protein Kinase C |
|  |  |  |  |  |  |  |  |  |  |  |  |  |  |  |  |  |  |  |  |  |  |  |  |  |  |  |  |  |  |  |  |  |  |  |  |  |  | *unc-77* | UNCoordinated |
|  |  |  |  |  |  |  |  |  |  |  |  |  |  |  |  |  |  |  |  |  |  |  |  |  |  |  |  |  |  |  |  |  |  |  |  |  |  | M153.4 |  |
|  |  |  |  |  |  |  |  |  |  |  |  |  |  |  |  |  |  |  |  |  |  |  |  |  |  |  |  |  |  |  |  |  |  |  |  |  |  | K02F2.5 |  |
|  |  |  |  |  |  |  |  |  |  |  |  |  |  |  |  |  |  |  |  |  |  |  |  |  |  |  |  |  |  |  |  |  |  |  |  |  |  | *nlp-8* | Neuropeptide-Like Protein |
|  |  |  |  |  |  |  |  |  |  |  |  |  |  |  |  |  |  |  |  |  |  |  |  |  |  |  |  |  |  |  |  |  |  |  |  |  |  | F45D3.2 |  |
|  |  |  |  |  |  |  |  |  |  |  |  |  |  |  |  |  |  |  |  |  |  |  |  |  |  |  |  |  |  |  |  |  |  |  |  |  |  | K01A6.6 |  |
|  |  |  |  |  |  |  |  |  |  |  |  |  |  |  |  |  |  |  |  |  |  |  |  |  |  |  |  |  |  |  |  |  |  |  |  |  |  | *sto-6* | STOmatin |
|  |  |  |  |  |  |  |  |  |  |  |  |  |  |  |  |  |  |  |  |  |  |  |  |  |  |  |  |  |  |  |  |  |  |  |  |  |  | W05E10.2 |  |
|  |  |  |  |  |  |  |  |  |  |  |  |  |  |  |  |  |  |  |  |  |  |  |  |  |  |  |  |  |  |  |  |  |  |  |  |  |  | *ssr-2* | Sperm-Specific family, class R |
|  |  |  |  |  |  |  |  |  |  |  |  |  |  |  |  |  |  |  |  |  |  |  |  |  |  |  |  |  |  |  |  |  |  |  |  |  |  | *pde-4* | PhosphoDiEsterase |
|  |  |  |  |  |  |  |  |  |  |  |  |  |  |  |  |  |  |  |  |  |  |  |  |  |  |  |  |  |  |  |  |  |  |  |  |  |  | *catp-7* | Cation transporting ATPase |
|  |  |  |  |  |  |  |  |  |  |  |  |  |  |  |  |  |  |  |  |  |  |  |  |  |  |  |  |  |  |  |  |  |  |  |  |  |  | *rnf-5* | RiNg Finger protein |
|  |  |  |  |  |  |  |  |  |  |  |  |  |  |  |  |  |  |  |  |  |  |  |  |  |  |  |  |  |  |  |  |  |  |  |  |  |  | *ddr-2* | Discoidin Domain Receptor |
|  |  |  |  |  |  |  |  |  |  |  |  |  |  |  |  |  |  |  |  |  |  |  |  |  |  |  |  |  |  |  |  |  |  |  |  |  |  | *ggr-1* | GABA/Glycine Receptor family (see gbr) |
|  |  |  |  |  |  |  |  |  |  |  |  |  |  |  |  |  |  |  |  |  |  |  |  |  |  |  |  |  |  |  |  |  |  |  |  |  |  | F46C5.2 |  |
|  |  |  |  |  |  |  |  |  |  |  |  |  |  |  |  |  |  |  |  |  |  |  |  |  |  |  |  |  |  |  |  |  |  |  |  |  |  | *glb-24* | GLoBin related |
|  |  |  |  |  |  |  |  |  |  |  |  |  |  |  |  |  |  |  |  |  |  |  |  |  |  |  |  |  |  |  |  |  |  |  |  |  |  | F34H10.4 |  |
|  |  |  |  |  |  |  |  |  |  |  |  |  |  |  |  |  |  |  |  |  |  |  |  |  |  |  |  |  |  |  |  |  |  |  |  |  |  | *npr-18* | NeuroPeptide Receptor family |
|  |  |  |  |  |  |  |  |  |  |  |  |  |  |  |  |  |  |  |  |  |  |  |  |  |  |  |  |  |  |  |  |  |  |  |  |  |  | Y37F4.7 |  |
|  |  |  |  |  |  |  |  |  |  |  |  |  |  |  |  |  |  |  |  |  |  |  |  |  |  |  |  |  |  |  |  |  |  |  |  |  |  | F33H1.6 |  |
|  |  |  |  |  |  |  |  |  |  |  |  |  |  |  |  |  |  |  |  |  |  |  |  |  |  |  |  |  |  |  |  |  |  |  |  |  |  | *col-68* | COLlagen |
|  |  |  |  |  |  |  |  |  |  |  |  |  |  |  |  |  |  |  |  |  |  |  |  |  |  |  |  |  |  |  |  |  |  |  |  |  |  | C30G12.4 |  |
|  |  |  |  |  |  |  |  |  |  |  |  |  |  |  |  |  |  |  |  |  |  |  |  |  |  |  |  |  |  |  |  |  |  |  |  |  |  | F18A11.5 |  |
|  |  |  |  |  |  |  |  |  |  |  |  |  |  |  |  |  |  |  |  |  |  |  |  |  |  |  |  |  |  |  |  |  |  |  |  |  |  | *sprr-2* | Sex Peptide Receptor (Drosophila) Related |
|  |  |  |  |  |  |  |  |  |  |  |  |  |  |  |  |  |  |  |  |  |  |  |  |  |  |  |  |  |  |  |  |  |  |  |  |  |  | F20D6.5 |  |
|  |  |  |  |  |  |  |  |  |  |  |  |  |  |  |  |  |  |  |  |  |  |  |  |  |  |  |  |  |  |  |  |  |  |  |  |  |  | *cdr-6* | CaDmium Responsive |
|  |  |  |  |  |  |  |  |  |  |  |  |  |  |  |  |  |  |  |  |  |  |  |  |  |  |  |  |  |  |  |  |  |  |  |  |  |  | C04F12.8 |  |
|  |  |  |  |  |  |  |  |  |  |  |  |  |  |  |  |  |  |  |  |  |  |  |  |  |  |  |  |  |  |  |  |  |  |  |  |  |  | *nhr-178* | Nuclear Hormone Receptor family |
|  |  |  |  |  |  |  |  |  |  |  |  |  |  |  |  |  |  |  |  |  |  |  |  |  |  |  |  |  |  |  |  |  |  |  |  |  |  | W02A11.t1 |  |
|  |  |  |  |  |  |  |  |  |  |  |  |  |  |  |  |  |  |  |  |  |  |  |  |  |  |  |  |  |  |  |  |  |  |  |  |  |  | Y110A2AL.19 |  |
|  |  |  |  |  |  |  |  |  |  |  |  |  |  |  |  |  |  |  |  |  |  |  |  |  |  |  |  |  |  |  |  |  |  |  |  |  |  | M04D8.5 |  |
|  |  |  |  |  |  |  |  |  |  |  |  |  |  |  |  |  |  |  |  |  |  |  |  |  |  |  |  |  |  |  |  |  |  |  |  |  |  | *str-187* | Seven TM Receptor |

### Phenotypes enriched

none found

### Anatomy terms enriched

none found

### GO terms enriched

none found

### Expression clusters enriched

|  |  |  |  |
| --- | --- | --- | --- |
| **Group name** | **Number in cluster** | **Enrichment** | **FDR corrected p** |
| WT-Pico Pan-neural Enriched Genes, with genes found multiple times in a single dataset removed (without dups). | 19 | 4.64 | 1.31e-06 |
| Genes significantly enriched (> 2x, FDR < 5%) in a particular cell-type versus a reference sample of all cells at the same stage. WBPaper00037950:all-neurons\_larva\_enriched | 15 | 5.81 | 5.94e-06 |
| Larval Pan-neural Enriched Genes. | 17 | 4.31 | 3.23e-05 |
| Genes predicted to be upregulated more than 2.0 fold in (AFD+AWB) datasets as compared to unsorted whole embryonic cells dataset. | 11 | 5.34 | 1.28e-03 |
| Genes significantly enriched (> 2x, FDR < 5%) in a particular cell-type versus a reference sample of all cells at the same stage. WBPaper00037950:glr-1(+)-neurons\_larva\_enriched | 10 | 5.93 | 1.50e-03 |
| Genes enriched in neuronal miRNA-induced silencing complexes (miRISC) as detected by immunoprecipitations and microarray analysis. | 10 | 5.67 | 2.15e-03 |
| Genes that show selective expression in a subset of cell types vs broadly expressed in many cell types. Correspond to 20% - 57% of enriched\_genes for a given cell type. WBPaper00037950:all-neurons\_larva\_SelectivelyEnriched | 9 | 6.46 | 2.33e-03 |
| Genes significantly enriched (> 2x, FDR < 5%) in a particular cell-type versus a reference sample of all cells at the same stage. WBPaper00037950:AVE-neuron\_embryo\_enriched | 8 | 7.24 | 3.43e-03 |
| Embryonic Pan-neural Enriched Genes. | 14 | 3.39 | 7.25e-03 |
| Genes significantly enriched (> 2x, FDR < 5%) in a particular cell-type versus a reference sample of all cells at both embryonic and larval stages. WBPaper00037950:dopaminergic-neurons\_CoreEnriched | 5 | 13.07 | 1.01e-02 |
| Genes significantly enriched (> 2x, FDR < 5%) in a particular cell-type versus a reference sample of all cells at the same stage. WBPaper00037950:dopaminergic-neurons\_larva\_enriched | 11 | 3.50 | 4.08e-02 |

### Motifs enriched

|  |  |  |  |  |  |
| --- | --- | --- | --- | --- | --- |
| **Motif** | **Logo** | **Possible orthologs** | **Number of motifs in cluster** | **Enrichment** | **FDR corrected p** |
| MA0467.1 |  | tbx-39 ceh-45 | 17 | 5.50 | 1.2e-06 |
| MA0598.1 |  | lin-1 C24A1.2 | 21 | 3.59 | 1.0e-05 |
| Etv6 |  | lin-1 C24A1.2 | 25 | 2.87 | 1.4e-05 |
| I$E74A\_01 |  | nhr-19 lin-1 C24A1.2 | 23 | 3.13 | 1.6e-05 |
| MA0076.2 |  | lin-1 C24A1.2 | 20 | 3.62 | 2.1e-05 |
| SOX2\_4 |  | sox-4 (0.82) dmd-4 | 26 | 2.66 | 2.5e-05 |
| Foxk1\_1 |  | nhr-213 lin-31 | 21 | 3.37 | 2.6e-05 |
| sr\_SANGER\_5\_FBgn0003499 |  | klf-1 ZC328.2 klf-2 | 32 | 2.10 | 2.9e-05 |
| Blimp-1\_SANGER\_5\_FBgn0035625 |  | blmp-1 | 23 | 3.01 | 3.1e-05 |
| GM12892\_PAX5C20\_HudsonAlpha |  | pax-3 pax-2 | 27 | 2.50 | 3.7e-05 |
| SPDEF\_6 |  | nhr-100 (0.71) lin-1 | 16 | 4.52 | 3.9e-05 |
| Scr\_Cell\_FBgn0003339 |  | lim-7 php-3 lin-39 unc-86 cfi-1 | 36 | 1.79 | 4.0e-05 |
| pTH6636 |  | egl-5 | 31 | 2.13 | 4.7e-05 |
| Pou3f2\_2824 |  | ceh-18 (0.6) lin-39 ceh-6 | 34 | 1.91 | 5.1e-05 |
| pTH9059 |  | ces-1 ztf-28 che-1 | 20 | 3.39 | 5.4e-05 |
| SP1\_f2 |  | klf-1 ZC328.2 klf-2 | 20 | 3.38 | 5.5e-05 |
| pTH9085 |  | nhr-42 | 28 | 2.35 | 6.0e-05 |
| BMAL1\_f1 |  | mdl-1 (0.55) aha-1 hlh-30 | 27 | 2.43 | 6.4e-05 |
| ZN384\_f1 |  | K11D2.4 lin-29 | 35 | 1.81 | 7.4e-05 |
| HES1\_f1 |  | lin-22 | 29 | 2.24 | 7.6e-05 |
| V$GATA1\_05 |  | elt-1 elt-6 ceh-34 ceh-32 egl-27 elt-7 | 33 | 1.92 | 1.0e-04 |
| TBX20\_3 |  | mab-9 tbx-38 tbx-39 tbx-43 | 15 | 4.50 | 1.0e-04 |
| pTH9394 |  | klf-1 klf-2 sptf-3 | 20 | 3.21 | 1.1e-04 |
| Hoxc8\_3429 |  | lin-39 | 27 | 2.36 | 1.2e-04 |
| pTH10630 |  | lsy-27 | 33 | 1.91 | 1.2e-04 |
| pTH6201 |  | ceh-36 (0.57) alr-1 dve-1 ceh-53 ceh-45 | 30 | 2.11 | 1.3e-04 |
| ARI3A\_f1 |  | alr-1 ceh-14 cfi-1 ZC204.2 | 21 | 3.02 | 1.3e-04 |
| pTH10722 |  | egrh-3 | 30 | 2.10 | 1.3e-04 |
| Spdef |  | lin-1 | 11 | 6.62 | 1.3e-04 |
| MCR\_f1 |  | nhr-255 | 23 | 2.74 | 1.4e-04 |
| MA0486.1 |  | F10B5.3 (0.74) hsf-1 Y53C10A.3 | 29 | 2.17 | 1.4e-04 |
| Elf5 |  | C24A1.2 | 25 | 2.50 | 1.6e-04 |
| CG5669\_SOLEXA\_5\_FBgn0039169 |  | klf-1 klf-2 | 22 | 2.83 | 1.7e-04 |
| V$GATA1\_04 |  | elt-1 | 31 | 2.00 | 1.8e-04 |
| FOXO6\_3 |  | ZC328.2 daf-16 | 26 | 2.39 | 1.9e-04 |
| GATA3\_2 |  | end-3 elt-1 | 24 | 2.58 | 1.9e-04 |
| MA0452.2 |  | ZK177.3 B0310.2 | 9 | 8.60 | 2.0e-04 |
| V$GATA1\_06 |  | elt-1 ztf-29 | 24 | 2.56 | 2.2e-04 |
| V$NCX\_01 |  | ceh-19 | 40 | 1.42 | 2.2e-04 |
| pTH9142 |  | ztf-6 C34D1.1 gei-11 | 32 | 1.91 | 2.4e-04 |
| HMGA1\_f1 |  | mel-28 mef-2 let-381 Y116A8C.22 Y61A9LA.9 | 28 | 2.18 | 2.4e-04 |
| Elf3 |  | C24A1.2 | 24 | 2.54 | 2.5e-04 |
| Pou3f3\_3235 |  | ceh-6 | 19 | 3.19 | 2.6e-04 |
| pTH9256 |  | ceh-18 (0.6) | 23 | 2.63 | 2.6e-04 |
| pTH6486 |  | nhr-145 (0.51) | 26 | 2.32 | 2.9e-04 |
| Exex\_Cell\_FBgn0041156 |  | ceh-18 (0.6) pal-1 lin-39 alr-1 ceh-43 ceh-12 | 19 | 3.13 | 3.3e-04 |
| pTH9297 |  | ceh-18 (0.6) | 23 | 2.59 | 3.4e-04 |
| MA0535.1 |  | daf-8 F45H11.6 | 20 | 2.97 | 3.4e-04 |
| pTH9326 |  | nhr-122 (-0.52) | 19 | 3.12 | 3.4e-04 |
| Pou2f2\_3748 |  | ceh-18 (0.6) lim-7 | 35 | 1.70 | 3.7e-04 |
| pTH5539 |  | unc-120 | 28 | 2.13 | 3.9e-04 |
| pTH2283 |  | odd-2 | 35 | 1.70 | 4.0e-04 |
| HXD13\_f1 |  | pal-1 | 20 | 2.93 | 4.0e-04 |
| Hmbox1\_2674 |  | hmbx-1 | 13 | 4.64 | 4.3e-04 |
| HXB6\_f1 |  | lin-39 ceh-20 ceh-12 | 25 | 2.36 | 4.3e-04 |
| pTH10802 |  | hlh-2 hlh-15 ces-1 K02D7.2 hlh-8 hlh-1 | 14 | 4.23 | 4.5e-04 |
| Mv104 |  | nhr-2 nhr-62 nhr-19 | 16 | 3.65 | 4.5e-04 |
| MA0264.1 |  | ceh-24 (0.64) ceh-22 dsc-1 | 20 | 2.90 | 4.6e-04 |
| pTH9135 |  | pop-1 | 37 | 1.58 | 4.8e-04 |
| PO3F2\_si |  | ceh-18 (0.6) dmd-3 | 25 | 2.34 | 4.9e-04 |
| pTH1049 |  | elt-1 | 27 | 2.18 | 5.0e-04 |
| MA0163.1 |  | Y53H1A.2 | 18 | 3.19 | 5.1e-04 |
| MA0219.1 |  | ceh-31 (0.67) ceh-18 (0.6) lim-7 ceh-16 lin-39 alr-1 lim-4 ceh-10 ceh-2 ceh-14 ceh-43 ceh-53 cog-1 egl-5 pha-2 mls-2 lim-6 ceh-45 ceh-12 ZC123.3 | 29 | 2.02 | 5.6e-04 |
| pTH9220 |  | mbr-1 (0.73) | 33 | 1.78 | 6.0e-04 |
| pTH9244 |  | tbx-39 | 28 | 2.08 | 6.0e-04 |
| MA0531.1 |  | F58G1.2 Y5F2A.4 | 28 | 2.08 | 6.3e-04 |
| V$DELTAEF1\_01 |  | hlh-2 ztf-6 | 25 | 2.30 | 6.6e-04 |
| HXD9\_f1 |  | php-3 lin-39 hbl-1 | 33 | 1.77 | 6.6e-04 |
| V$RFX1\_01 |  | daf-19 (0.7) | 27 | 2.13 | 7.1e-04 |
| Pou3f4\_3773 |  | ceh-6 | 35 | 1.66 | 7.4e-04 |
| CG8765\_SANGER\_5\_FBgn0036900 |  | H20J04.3 | 23 | 2.46 | 7.4e-04 |
| SOX10\_1 |  | sox-4 (0.82) egl-13 (0.7) K11D2.4 | 18 | 3.09 | 7.6e-04 |
| pTH6729 |  | fkh-8 (0.53) fkh-10 let-381 daf-16 fkh-7 lin-31 pha-4 | 25 | 2.27 | 8.3e-04 |
| HLH29 |  | lin-22 hlh-27 hlh-28 | 25 | 2.27 | 8.3e-04 |
| Tcf1\_2666 |  | hmbx-1 | 38 | 1.49 | 8.6e-04 |
| TCF7L1\_1 |  | sox-4 (0.82) nhr-100 (0.71) pop-1 | 22 | 2.54 | 8.6e-04 |
| gl\_FlyReg\_FBgn0004618 |  | ceh-24 (0.64) ces-1 C34H4.5 T22H9.4 | 25 | 2.25 | 9.0e-04 |
| V$CEBP\_01 |  | C48E7.11 | 29 | 1.97 | 9.1e-04 |
| HeLa-S3\_ZNF274\_UCD |  | C28G1.4 | 35 | 1.64 | 9.4e-04 |
| PhdP\_SOLEXA\_FBgn0025334 |  | lin-39 alr-1 ceh-10 ceh-1 eyg-1 | 19 | 2.88 | 9.4e-04 |
| V$IK1\_01 |  | F26F4.8 lag-1 ztf-3 | 19 | 2.88 | 9.7e-04 |
| Hoxc5\_2630 |  | ceh-18 (0.6) npax-3 lin-39 alr-1 ceh-43 ceh-53 ceh-1 ceh-45 ceh-12 | 19 | 2.87 | 9.9e-04 |
| pTH10037 |  | T22C8.4 (-0.54) ref-2 | 24 | 2.31 | 1.1e-03 |
| pTH10645 |  | nhr-100 (0.71) elt-1 nhr-10 nhr-7 | 24 | 2.31 | 1.1e-03 |
| pTH9173 |  | efl-2 | 24 | 2.31 | 1.1e-03 |
| pTH9262 |  | lin-54 | 25 | 2.23 | 1.1e-03 |
| pTH1292 |  | ceh-24 (0.64) pzf-1 | 27 | 2.08 | 1.1e-03 |
| Tcf1\_2666 |  | hmbx-1 | 38 | 1.47 | 1.1e-03 |
| SOX2\_f1 |  | sox-4 (0.82) ceh-18 (0.6) ceh-6 tbp-1 | 25 | 2.22 | 1.2e-03 |
| pTH9974 |  | hlh-32 hlh-16 ngn-1 | 19 | 2.84 | 1.2e-03 |
| Elf3\_3876 |  | C24A1.2 | 24 | 2.29 | 1.2e-03 |
| SMAD3\_1 |  | sma-4 (-0.62) hlh-8 daf-8 | 35 | 1.62 | 1.3e-03 |
| V$MEF2\_02 |  | mef-2 | 17 | 3.12 | 1.3e-03 |
| MA0547.1 |  | skn-1 | 27 | 2.06 | 1.3e-03 |
| PURA\_f1 |  | Y53H1A.2 plp-2 | 20 | 2.68 | 1.3e-03 |
| pTH8649 |  | mbr-1 (0.73) | 25 | 2.20 | 1.3e-03 |
| ELF3\_f1 |  | K02D7.2 unc-120 C24A1.2 | 32 | 1.77 | 1.3e-03 |
| FLI1\_f1 |  | lin-1 | 26 | 2.13 | 1.3e-03 |
| pTH10647 |  | nhr-232 | 31 | 1.82 | 1.3e-03 |
| Oli\_da\_SANGER\_5\_3\_FBgn0032651 |  | hlh-32 hlh-15 hlh-12 | 26 | 2.12 | 1.4e-03 |
| Gata3\_1024 |  | elt-1 | 29 | 1.93 | 1.4e-03 |
| CG34031\_SOLEXA\_FBgn0054031 |  | ceh-9 (0.78) ceh-31 (0.67) ceh-8 (0.66) ceh-30 (0.64) ceh-24 (0.64) lim-7 lin-39 alr-1 ceh-19 ceh-43 cog-1 ceh-1 | 17 | 3.10 | 1.4e-03 |
| Mv73 |  | elt-1 | 27 | 2.05 | 1.4e-03 |
| Hoxb5\_3122 |  | lin-39 | 18 | 2.92 | 1.5e-03 |
| V$HOX13\_01 |  | lin-39 | 20 | 2.65 | 1.5e-03 |
| pTH10040 |  | odr-7 (0.57) nhr-79 nhr-28 slr-2 nhr-273 lin-1 tbx-39 | 23 | 2.35 | 1.5e-03 |
| Elf4 |  | C24A1.2 | 21 | 2.54 | 1.5e-03 |
| pTH9149 |  | ztf-30 | 15 | 3.47 | 1.6e-03 |
| Zbtb12\_2932 |  | ceh-90 lsy-27 | 14 | 3.72 | 1.6e-03 |
| V$HMX1\_01 |  | ceh-9 (0.78) daf-12 ztf-3 Y5F2A.4 | 29 | 1.91 | 1.6e-03 |
| MA0066.1 |  | nhr-43 | 25 | 2.17 | 1.6e-03 |
| MA0543.1 |  | daf-8 eor-1 | 35 | 1.60 | 1.6e-03 |
| Sox8\_1733 |  | sox-4 (0.82) gei-3 (0.75) pop-1 C05C9.3 | 25 | 2.16 | 1.8e-03 |
| pTH9237 |  | mel-28 | 24 | 2.24 | 1.8e-03 |
| Elk4 |  | F19F10.1 lin-1 C24A1.2 | 18 | 2.88 | 1.8e-03 |
| CG31670\_SANGER\_5\_FBgn0031375 |  | F21A9.2 CELE\_Y38H8A.5 | 27 | 2.02 | 1.8e-03 |
| pTH6447 |  | ceh-19 | 28 | 1.96 | 1.9e-03 |
| pTH9043 |  | sem-2 | 23 | 2.31 | 1.9e-03 |
| pTH9709 |  | die-1 | 22 | 2.40 | 1.9e-03 |
| Gata5\_3768 |  | elt-1 | 29 | 1.89 | 1.9e-03 |
| pTH5924 |  | nhr-255 | 7 | 8.90 | 1.9e-03 |
| pTH5117 |  | cfi-1 | 24 | 2.22 | 1.9e-03 |
| LHX6\_3 |  | nhr-208 lim-6 cfi-1 | 29 | 1.89 | 2.1e-03 |
| V$PAX2\_02 |  | pax-1 | 11 | 4.69 | 2.1e-03 |
| pTH2842 |  | ceh-24 (0.64) dsc-1 | 15 | 3.36 | 2.1e-03 |
| cad\_FlyReg\_FBgn0000251 |  | ceh-24 (0.64) ceh-13 (-0.61) D1005.3 | 25 | 2.13 | 2.2e-03 |
| Irx3\_1 |  | irx-1 | 29 | 1.88 | 2.2e-03 |
| FOXO1\_si |  | irx-1 daf-16 | 31 | 1.77 | 2.3e-03 |
| pTH3064 |  | crh-1 | 39 | 1.38 | 2.3e-03 |
| crp\_SANGER\_10\_FBgn0001994 |  | lin-32 (-0.51) hlh-15 hlh-1 hlh-11 | 28 | 1.93 | 2.4e-03 |
| NR2F6\_f1 |  | nhr-2 nhr-62 nhr-239 | 24 | 2.19 | 2.4e-03 |
| POU3F1\_2 |  | ceh-18 (0.6) unc-86 | 23 | 2.27 | 2.4e-03 |
| V$FOXJ2\_02 |  | elt-1 lin-31 | 15 | 3.31 | 2.4e-03 |
| pTH5887 |  | lin-39 | 37 | 1.48 | 2.4e-03 |
| pTH9384 |  | cfi-1 | 26 | 2.05 | 2.5e-03 |
| pTH5778 |  | egl-5 | 19 | 2.67 | 2.5e-03 |
| pTH9880 |  | end-1 | 25 | 2.11 | 2.5e-03 |
| FOXD3\_f1 |  | let-381 lin-31 | 20 | 2.54 | 2.6e-03 |
| V$YY1\_01 |  | lsy-2 | 31 | 1.76 | 2.6e-03 |
| pTH9242 |  | mel-28 | 27 | 1.98 | 2.6e-03 |
| Hoxa3\_2783 |  | lin-39 | 22 | 2.34 | 2.7e-03 |
| pTH9247 |  | dmd-3 C34D1.1 | 31 | 1.76 | 2.7e-03 |
| Max\_3864 |  | mxl-1 ref-1 mxl-2 lin-22 aha-1 hlh-26 C27D6.4 | 20 | 2.53 | 2.7e-03 |
| MA0386.1 |  | K11D2.4 tbp-1 | 36 | 1.52 | 2.9e-03 |
| Sox15\_3457 |  | sox-4 (0.82) | 31 | 1.75 | 2.9e-03 |
| pTH9381 |  | ceh-18 (0.6) | 21 | 2.41 | 2.9e-03 |
| V$LYF1\_01 |  | mbr-1 (0.73) F26F4.8 bed-3 nhr-177 | 20 | 2.51 | 2.9e-03 |
| MEIS2\_do |  | lin-32 (-0.51) lin-39 ceh-32 | 35 | 1.56 | 3.0e-03 |
| pTH9215 |  | C34D1.1 | 31 | 1.74 | 3.1e-03 |
| V$CDC5\_01 |  | irx-1 D1081.8 | 25 | 2.08 | 3.1e-03 |
| pTH9246 |  | fkh-10 let-381 C34D1.1 | 39 | 1.37 | 3.2e-03 |
| V$IK2\_01 |  | F26F4.8 | 24 | 2.15 | 3.2e-03 |
| pTH6143 |  | ceh-24 (0.64) pal-1 php-3 lin-39 | 23 | 2.22 | 3.2e-03 |
| Etv3 |  | lin-1 | 12 | 4.01 | 3.3e-03 |
| pTH10772 |  | ceh-52 | 14 | 3.43 | 3.3e-03 |
| pTH2933 |  | F58G1.2 | 31 | 1.74 | 3.4e-03 |
| CG2052\_SOLEXA\_2.5\_FBgn0039905 |  | mel-28 fkh-7 lin-29 | 13 | 3.68 | 3.4e-03 |
| Hoxb8\_3780 |  | lin-39 | 19 | 2.59 | 3.4e-03 |
| Antp\_FlyReg\_FBgn0000095 |  | lin-39 let-381 ceh-53 lin-31 Y116A8C.22 | 20 | 2.48 | 3.5e-03 |
| Osr1\_3033 |  | odd-1 odd-2 | 20 | 2.48 | 3.5e-03 |
| Hoxa7\_3750 |  | lin-39 | 24 | 2.13 | 3.5e-03 |
| pTH5334 |  | fkh-10 let-381 daf-16 fkh-7 lin-31 | 29 | 1.83 | 3.6e-03 |
| pTH3998 |  | tbx-39 | 17 | 2.84 | 3.6e-03 |
| HNF6\_f1 |  | ceh-48 dsc-1 | 32 | 1.68 | 3.7e-03 |
| Hth\_SOLEXA\_FBgn0001235 |  | ces-1 ceh-20 ceh-32 F55C5.11 | 35 | 1.55 | 3.7e-03 |
| pTH5922 |  | ceh-24 (0.64) | 37 | 1.46 | 3.7e-03 |
| Barhl1\_2590 |  | ceh-31 (0.67) lim-7 ceh-16 lin-39 alr-1 ceh-43 ceh-1 | 26 | 1.99 | 3.7e-03 |
| LHX9\_2 |  | ceh-31 (0.67) lin-39 ceh-14 ceh-43 ceh-1 | 36 | 1.50 | 3.8e-03 |
| HepG2\_HSF1\_Stanford |  | Y53C10A.3 | 21 | 2.36 | 3.9e-03 |
| Egr1\_2580 |  | ZC328.2 | 16 | 2.96 | 4.2e-03 |
| Tcf7\_0950 |  | pop-1 | 19 | 2.55 | 4.2e-03 |
| MA0146.2 |  | aptf-1 F58G1.2 | 17 | 2.80 | 4.2e-03 |
| pTH5002 |  | crh-1 W08E12.1 | 15 | 3.12 | 4.4e-03 |
| ARNT2\_si |  | C46E10.9 aha-1 | 22 | 2.25 | 4.5e-03 |
| pTH9314 |  | fos-1 atf-6 crh-1 atf-7 C27D6.4 | 37 | 1.44 | 4.8e-03 |
| pTH3751 |  | tbx-39 | 16 | 2.91 | 4.8e-03 |
| pTH5437 |  | ceh-34 | 24 | 2.09 | 4.8e-03 |
| pTH10013 |  | nhr-168 | 18 | 2.63 | 4.8e-03 |
| pTH6327 |  | dsc-1 | 28 | 1.85 | 4.8e-03 |
| pTH5828 |  | nhr-84 (0.66) nhr-216 (0.52) | 29 | 1.80 | 5.0e-03 |
| pTH10779 |  | nhr-134 nhr-182 | 29 | 1.79 | 5.0e-03 |
| V$T3R\_01 |  | nhr-213 nhr-15 | 25 | 2.02 | 5.0e-03 |
| Caup\_SOLEXA\_FBgn0015919 |  | irx-1 | 27 | 1.90 | 5.1e-03 |
| pTH10768 |  | med-2 | 36 | 1.48 | 5.1e-03 |
| pTH3468 |  | nhr-71 (0.62) nhr-2 nhr-213 nhr-6 nhr-68 Y67D8A.3 | 37 | 1.44 | 5.2e-03 |
| pTH10623 |  | scrt-1 | 25 | 2.01 | 5.3e-03 |
| GM12878\_SRF\_HudsonAlpha |  | unc-120 | 23 | 2.14 | 5.4e-03 |
| MA0095.2 |  | lsy-2 | 25 | 2.01 | 5.4e-03 |
| SPDEF\_2 |  | lin-1 | 38 | 1.39 | 5.5e-03 |
| pTH9049 |  | ztf-2 | 21 | 2.30 | 5.5e-03 |
| pTH10808 |  | ztf-19 | 30 | 1.74 | 5.6e-03 |
| V$FAC1\_01 |  | gei-8 | 19 | 2.48 | 5.6e-03 |
| pTH9054 |  | npax-1 (0.51) nhr-255 lin-14 | 30 | 1.73 | 5.7e-03 |
| V$CDXA\_01 |  | ceh-13 (-0.61) php-3 | 24 | 2.06 | 5.8e-03 |
| V$AREB6\_02 |  | ztf-6 | 17 | 2.71 | 5.8e-03 |
| MA0027.1 |  | ceh-16 | 39 | 1.34 | 5.8e-03 |
| Rfxdc2\_3516 |  | daf-19 (0.7) mab-3 | 37 | 1.43 | 5.8e-03 |
| Mf28 |  | elt-1 | 19 | 2.47 | 5.8e-03 |
| pTH10041 |  | ztf-29 | 11 | 4.07 | 6.0e-03 |
| Nkx1-1\_3856 |  | ceh-30 (0.64) | 24 | 2.06 | 6.0e-03 |
| TBP\_f1 |  | tbp-1 | 20 | 2.36 | 6.2e-03 |
| Atf1\_3026 |  | crh-1 | 26 | 1.93 | 6.4e-03 |
| Sox1\_4 |  | sox-4 (0.82) F56D1.1 | 24 | 2.05 | 6.4e-03 |
| pTH10805 |  | ztf-16 | 22 | 2.19 | 6.4e-03 |
| Hoxb3\_1720 |  | lin-39 | 17 | 2.69 | 6.4e-03 |
| pTH2936 |  | nhr-239 | 28 | 1.82 | 6.5e-03 |
| YY1\_1 |  | lsy-2 | 27 | 1.87 | 6.5e-03 |
| HSFY2\_1 |  | mab-3 hsf-1 | 19 | 2.44 | 6.9e-03 |
| pTH5166 |  | ces-2 (-0.54) atf-2 C48E7.11 F23F12.9 | 26 | 1.91 | 7.0e-03 |
| pTH5119 |  | cfi-1 | 22 | 2.17 | 7.0e-03 |
| V$BRN2\_01 |  | ceh-18 (0.6) | 23 | 2.09 | 7.3e-03 |
| pTH6449 |  | ceh-43 | 25 | 1.96 | 7.4e-03 |
| pTH10769 |  | Y48G1C.6 | 20 | 2.33 | 7.4e-03 |
| Nkx1-2\_3214 |  | ceh-30 (0.64) | 24 | 2.02 | 7.5e-03 |
| pTH6497 |  | lin-31 | 11 | 3.93 | 7.6e-03 |
| exd\_FlyReg\_FBgn0000611 |  | ceh-20 let-381 cfi-1 | 19 | 2.41 | 7.7e-03 |
| HXA7\_f1 |  | lin-39 | 24 | 2.01 | 8.3e-03 |
| NEUROD2\_1 |  | hlh-32 hlh-15 lin-31 ngn-1 | 23 | 2.07 | 8.5e-03 |
| pTH6508 |  | nhr-36 (0.75) | 31 | 1.65 | 8.6e-03 |
| pTH10823 |  | B0310.2 | 26 | 1.89 | 8.8e-03 |
| MA0505.1 |  | nhr-68 | 26 | 1.89 | 8.8e-03 |
| T-47D\_GATA3\_HudsonAlpha |  | elt-1 | 33 | 1.57 | 8.8e-03 |
| Spdef\_0905 |  | lin-1 | 21 | 2.21 | 8.9e-03 |
| pTH9182 |  | tbx-39 | 26 | 1.88 | 9.0e-03 |
| pTH9279 |  | Y116A8C.22 | 34 | 1.53 | 9.0e-03 |
| Lmx1a\_2238 |  | lim-6 | 17 | 2.60 | 9.0e-03 |
| pTH9708 |  | ceh-34 | 23 | 2.06 | 9.1e-03 |
| V$VMYB\_01 |  | D1081.8 | 16 | 2.73 | 9.1e-03 |
| Hoxc13\_3127 |  | ceh-24 (0.64) pal-1 | 24 | 1.99 | 9.2e-03 |
| Rfx3\_1 |  | daf-19 (0.7) F52B5.7 | 22 | 2.12 | 9.3e-03 |
| Emx2\_3420 |  | ceh-2 | 21 | 2.20 | 9.3e-03 |
| pTH9925 |  | nhr-100 (0.71) ztf-11 | 23 | 2.05 | 9.4e-03 |
| pTH9254 |  | mel-28 | 25 | 1.93 | 9.6e-03 |
| BARHL2\_4 |  | ceh-31 (0.67) | 26 | 1.87 | 9.7e-03 |
| pTH10777 |  | dmd-3 | 25 | 1.93 | 9.8e-03 |
| Hoxa7\_2668 |  | lin-39 | 18 | 2.46 | 9.9e-03 |
| Hoxa2\_3079 |  | lin-39 | 39 | 1.32 | 1.0e-02 |
| MA0244.1 |  | C48E7.11 | 39 | 1.31 | 1.0e-02 |
| pTH10034 |  | nhr-66 | 13 | 3.21 | 1.0e-02 |
| disco-r-Cl1\_SANGER\_5\_FBgn0042650 |  | nhr-68 lin-31 F55C5.11 | 24 | 1.98 | 1.0e-02 |
| Hoxa5\_3415 |  | lin-39 | 21 | 2.17 | 1.1e-02 |
| NFIA\_1 |  | nfi-1 F49E12.6 | 36 | 1.44 | 1.1e-02 |
| pTH1001 |  | dnj-17 | 22 | 2.10 | 1.1e-02 |
| pTH8982 |  | ceh-48 | 25 | 1.91 | 1.1e-02 |
| Spt15 |  | tbp-1 | 24 | 1.96 | 1.1e-02 |
| Plagl1\_0972 |  | Y53H1A.2 | 30 | 1.67 | 1.1e-02 |
| ERG\_2 |  | lin-1 | 24 | 1.96 | 1.2e-02 |
| pTH6562 |  | ceh-5 | 22 | 2.08 | 1.2e-02 |
| pTH8991 |  | cey-3 | 25 | 1.89 | 1.2e-02 |
| Hoxb4\_2627 |  | lin-39 | 17 | 2.51 | 1.2e-02 |
| CREB1\_f1 |  | fos-1 crh-1 atf-5 | 19 | 2.31 | 1.3e-02 |
| HEN1\_si |  | hlh-15 hlh-1 | 35 | 1.47 | 1.3e-02 |
| MA0058.2 |  | mxl-1 | 38 | 1.35 | 1.3e-02 |
| Hoxa11\_2218 |  | php-3 | 27 | 1.79 | 1.3e-02 |
| MA0488.1 |  | crh-1 ceh-26 | 25 | 1.89 | 1.3e-02 |
| Hoxc4\_3491 |  | lin-39 | 17 | 2.50 | 1.3e-02 |
| EN1\_4 |  | ceh-16 | 17 | 2.50 | 1.3e-02 |
| Vax1\_3499 |  | C02F12.10 | 22 | 2.06 | 1.4e-02 |
| pTH10030 |  | xbp-1 (0.55) | 36 | 1.42 | 1.4e-02 |
| V$CETS1P54\_02 |  | C52B9.2 | 16 | 2.61 | 1.4e-02 |
| pTH8216 |  | Y116A8C.22 | 24 | 1.93 | 1.4e-02 |
| pTH10633 |  | R07H5.10 | 24 | 1.93 | 1.4e-02 |
| pTH2280 |  | mnm-2 | 19 | 2.28 | 1.4e-02 |
| Lbx2\_3869 |  | mls-2 | 20 | 2.19 | 1.5e-02 |
| pTH9198 |  | dmd-3 | 35 | 1.45 | 1.5e-02 |
| V$CDPCR3\_01 |  | ceh-24 (0.64) ceh-48 | 22 | 2.04 | 1.5e-02 |
| Hmx1\_3423 |  | ceh-9 (0.78) | 40 | 1.24 | 1.5e-02 |
| Pou2f3\_3986 |  | ceh-18 (0.6) | 28 | 1.72 | 1.5e-02 |
| pTH5118 |  | cfi-1 | 22 | 2.04 | 1.5e-02 |
| Nkx6-3\_3446 |  | cog-1 | 17 | 2.46 | 1.6e-02 |
| Cutl1\_3494 |  | ceh-44 | 12 | 3.27 | 1.6e-02 |
| pTH10031 |  | mbr-1 (0.73) | 30 | 1.64 | 1.6e-02 |
| Sox17\_2837 |  | sox-4 (0.82) | 14 | 2.85 | 1.6e-02 |
| V$OCT1\_06 |  | ceh-18 (0.6) ztf-9 (0.51) | 38 | 1.34 | 1.6e-02 |
| pTH9164 |  | ceh-26 | 40 | 1.24 | 1.7e-02 |
| CENPB\_1 |  | F21D5.4 | 37 | 1.37 | 1.7e-02 |
| Prop1\_3949 |  | ceh-16 ceh-53 | 17 | 2.43 | 1.7e-02 |
| pTH3041 |  | atf-2 | 16 | 2.53 | 1.8e-02 |
| pTH9137 |  | nhr-65 | 32 | 1.55 | 1.8e-02 |
| pTH3997 |  | C04F5.9 | 21 | 2.07 | 1.8e-02 |
| I$DFD\_01 |  | lin-39 | 14 | 2.80 | 1.9e-02 |
| pTH10650 |  | nhr-153 | 29 | 1.65 | 2.0e-02 |
| pTH10028 |  | nhr-204 | 19 | 2.21 | 2.0e-02 |
| Hey\_SANGER\_5\_FBgn0027788 |  | lin-22 | 15 | 2.62 | 2.0e-02 |
| V$GATA1\_02 |  | elt-1 | 24 | 1.88 | 2.0e-02 |
| Nsy-7 |  | nsy-7 | 20 | 2.12 | 2.1e-02 |
| pTH10816 |  | dmd-6 | 31 | 1.57 | 2.2e-02 |
| pTH9335 |  | mel-28 | 35 | 1.43 | 2.2e-02 |
| RORG\_f1 |  | nhr-213 nhr-118 | 23 | 1.92 | 2.2e-02 |
| PAX5\_si |  | pax-2 D1081.8 | 38 | 1.32 | 2.2e-02 |
| Hoxa10\_2318 |  | ceh-24 (0.64) | 18 | 2.27 | 2.3e-02 |
| ZBT7A\_f1 |  | klf-1 ZC328.2 | 33 | 1.49 | 2.3e-02 |
| Cdx2\_4272 |  | ceh-13 (-0.61) | 18 | 2.26 | 2.3e-02 |
| pTH6071 |  | C46E10.8 C33G8.2 | 11 | 3.33 | 2.3e-02 |
| Hoxa4\_3426 |  | lin-39 | 27 | 1.72 | 2.3e-02 |
| MA0541.1 |  | efl-1 F49E12.6 | 18 | 2.26 | 2.4e-02 |
| Vsx1\_1728 |  | alr-1 | 22 | 1.96 | 2.4e-02 |
| K562\_ZBTB7A\_HudsonAlpha |  | ZC328.2 | 32 | 1.52 | 2.4e-02 |
| pTH6591 |  | lin-31 | 15 | 2.57 | 2.4e-02 |
| pTH8985 |  | athp-1 | 37 | 1.35 | 2.5e-02 |
| MA0131.1 |  | F39B2.1 | 36 | 1.38 | 2.6e-02 |
| Hoxd13\_2356 |  | pal-1 | 17 | 2.33 | 2.6e-02 |
| pTH6436 |  | ceh-53 | 23 | 1.89 | 2.6e-02 |
| pTH9082 |  | mab-23 | 17 | 2.32 | 2.6e-02 |
| TLX1\_f1 |  | ceh-19 | 23 | 1.89 | 2.6e-02 |
| pTH9261 |  | dmd-3 lin-48 | 25 | 1.79 | 2.6e-02 |
| pTH8679 |  | pax-2 | 17 | 2.32 | 2.6e-02 |
| Jundm2\_0911 |  | fos-1 | 20 | 2.07 | 2.7e-02 |
| pTH9300 |  | dmd-3 | 23 | 1.88 | 2.8e-02 |
| pTH3819 |  | ceh-18 (0.6) | 17 | 2.31 | 2.8e-02 |
| pTH6425 |  | pop-1 ceh-20 | 11 | 3.23 | 2.8e-02 |
| pTH9380 |  | mel-28 | 34 | 1.44 | 2.9e-02 |
| Pou2f1\_3081 |  | ceh-18 (0.6) | 17 | 2.30 | 2.9e-02 |
| pTH3046 |  | Y116A8C.22 | 17 | 2.29 | 2.9e-02 |
| pTH10807 |  | F13H6.1 (0.53) | 36 | 1.37 | 2.9e-02 |
| pTH9907 |  | nhr-34 | 18 | 2.20 | 3.0e-02 |
| pTH9216 |  | ceh-18 (0.6) | 21 | 1.98 | 3.0e-02 |
| K562\_SP2\_HudsonAlpha |  | klf-2 | 15 | 2.50 | 3.0e-02 |
| Vax2\_3500 |  | C02F12.10 | 21 | 1.98 | 3.0e-02 |
| CG4854\_SANGER\_10\_FBgn0038766 |  | K11D2.4 | 20 | 2.04 | 3.1e-02 |
| pTH5078 |  | ces-2 (-0.54) | 20 | 2.04 | 3.1e-02 |
| pTH9108 |  | daf-12 | 26 | 1.72 | 3.2e-02 |
| pTH7032 |  | F52B11.1 | 32 | 1.50 | 3.3e-02 |
| pTH6106 |  | nhr-182 | 25 | 1.76 | 3.3e-02 |
| pTH9924 |  | nhr-46 (0.54) | 22 | 1.91 | 3.3e-02 |
| V$AREB6\_01 |  | ztf-6 tbx-39 | 36 | 1.37 | 3.3e-02 |
| Evx1\_3952 |  | ceh-53 | 37 | 1.33 | 3.4e-02 |
| rn\_SOLEXA\_5\_FBgn0259172 |  | lin-29 | 15 | 2.46 | 3.5e-02 |
| V$GR\_Q6 |  | nhr-255 | 17 | 2.25 | 3.5e-02 |
| pTH9188 |  | dmd-5 | 22 | 1.90 | 3.5e-02 |
| V$OCT1\_03 |  | ceh-18 (0.6) | 38 | 1.29 | 3.5e-02 |
| pTH5916 |  | efl-2 | 21 | 1.95 | 3.7e-02 |
| Sox1\_2631 |  | sox-4 (0.82) | 21 | 1.95 | 3.7e-02 |
| V$ZID\_01 |  | ztf-28 | 37 | 1.32 | 3.8e-02 |
| V$TAXCREB\_02 |  | crh-1 | 16 | 2.31 | 4.0e-02 |
| Hoxd1\_3448 |  | ceh-12 | 20 | 1.99 | 4.1e-02 |
| Meox1\_2310 |  | ceh-31 (0.67) | 38 | 1.29 | 4.1e-02 |
| Irx2\_0900 |  | irx-1 | 10 | 3.30 | 4.1e-02 |
| Hoxa6\_1040 |  | lin-39 | 17 | 2.20 | 4.2e-02 |
| Pbx1\_3203 |  | ceh-20 | 25 | 1.72 | 4.3e-02 |
| HXD10\_f1 |  | php-3 | 29 | 1.57 | 4.3e-02 |
| pTH9260 |  | mel-28 | 32 | 1.47 | 4.3e-02 |
| MA0540.1 |  | dpy-27 | 35 | 1.38 | 4.4e-02 |
| pTH8411 |  | tbx-39 | 30 | 1.54 | 4.4e-02 |
| pTH6478 |  | lim-7 | 15 | 2.39 | 4.4e-02 |
| pTH7875 |  | mel-28 | 36 | 1.35 | 4.5e-02 |
| Hoxa9\_2622 |  | lin-39 | 26 | 1.68 | 4.5e-02 |
| Mafk\_3106 |  | F45H11.6 | 20 | 1.96 | 4.6e-02 |
| Barx2\_3447 |  | ceh-43 | 24 | 1.76 | 4.6e-02 |
| pTH9096 |  | T07C12.11 | 32 | 1.46 | 4.7e-02 |
| MA0032.1 |  | let-381 | 32 | 1.46 | 4.8e-02 |
| Pou3f1\_3819 |  | ceh-6 | 17 | 2.17 | 4.8e-02 |
| pTH9163 |  | nhr-3 (0.58) | 24 | 1.75 | 4.9e-02 |
| V$FREAC7\_01 |  | lin-31 | 38 | 1.28 | 5.0e-02 |

### Correlated (and anti-correlated) transcription factors

|  |  |
| --- | --- |
| **Transcription factor** | **Correlation** |
| ctbp-1 | 0.84 |
| nhr-4 | 0.84 |
| tag-97 | 0.82 |
| sox-4 | 0.82 |
| zfh-2 | 0.81 |
| saeg-1 | 0.81 |
| ceh-54 | 0.80 |
| saeg-2 | 0.79 |
| nhr-32 | 0.78 |
| ceh-9 | 0.78 |
| nhr-95 | 0.78 |
| nhr-124 | 0.78 |
| daf-3 | 0.78 |
| jun-1 | 0.77 |
| nhr-40 | 0.77 |
| ceh-62 | 0.77 |
| nhr-17 | 0.77 |
| nhr-1 | 0.77 |
| C06E2.1 | 0.77 |
| F26A10.2 | 0.76 |
| nhr-158 | 0.76 |
| nhr-105 | 0.76 |
| K12H6.12 | 0.76 |
| nhr-198 | 0.75 |
| nhr-258 | 0.75 |
| nob-1 | -0.48 |
| nhr-248 | -0.48 |
| Y48G9A.11 | -0.49 |
| pal-1 | -0.50 |
| pop-1 | -0.50 |
| zip-8 | -0.50 |
| nhr-13 | -0.51 |
| lin-32 | -0.51 |
| C16A3.4 | -0.52 |
| nhr-122 | -0.52 |
| sex-1 | -0.53 |
| F27D4.4 | -0.54 |
| T22C8.4 | -0.54 |
| ces-2 | -0.54 |
| tlp-1 | -0.54 |
| hmg-5 | -0.54 |
| cnd-1 | -0.55 |
| Y53F4B.3 | -0.56 |
| zip-7 | -0.56 |
| duxl-1 | -0.56 |
| mab-5 | -0.58 |
| nhr-210 | -0.58 |
| sup-35 | -0.58 |
| ceh-13 | -0.61 |
| sma-4 | -0.62 |

### ChIP peaks enriched

|  |  |  |  |  |
| --- | --- | --- | --- | --- |
| **Gene** | **Experiment** | **Number of upstream peaks** | **Enrichment** | **FDR corrected p** |
| ces-1 | CES-1\_Larvae-L3-stage | 13 | 4.19 | 0.00031 |
| ces-1 | CES-1\_Embryos | 20 | 2.25 | 0.00450 |
| ces-1 | CES-1\_Fed-L1-stage-larvae | 10 | 3.03 | 0.03900 |
| alr-1 | ALR-1\_Larvae-L2-stage | 17 | 2.08 | 0.04200 |
