# Supplementary material for: Overlapping cell population expression profiling and regulatory inference in C. elegans
Source: BMC Genomics. 2016 Feb 29;17:159. doi: 10.1186/s12864-016-2482-z (PMC4772325; doi:10.1186/s12864-016-2482-z)
Supplement: Additional file 13: — Web supplement. (DOC 21 kb) [file 12864_2016_2482_MOESM13_ESM.zip › sortWeb/clusters/hier.300.clusters/245.html]

Cluster 245 

## Cluster 245

### Expression

| cnd-1 rep. 1 | cnd-1 rep. 2 | cnd-1 rep. 3 | pha-4 rep. 1 | pha-4 rep. 2 | pha-4 rep. 3 | ceh-27 | ceh-36 | ceh-6 | F21D5.9 | mir-57 | mls-2 | pal-1 | pros-1 | ttx-3 | unc-130 | hlh-16 | irx-1 | ceh-6 (+) hlh-16 (+) | ceh-6 (+) hlh-16 (-) | ceh-6 (-) hlh-16 (+) | cnd-1 singlets | pha-4 singlets | 0 | 60 | 120 | 150 | 180 | 240 | 330 | 390 | 420 | 480 | 540 | 570 | 600 | 630 | 660 | NAME | Functional description |
| --- | --- | --- | --- | --- | --- | --- | --- | --- | --- | --- | --- | --- | --- | --- | --- | --- | --- | --- | --- | --- | --- | --- | --- | --- | --- | --- | --- | --- | --- | --- | --- | --- | --- | --- | --- | --- | --- | --- | --- |
|  |  |  |  |  |  |  |  |  |  |  |  |  |  |  |  |  |  |  |  |  |  |  |  |  |  |  |  |  |  |  |  |  |  |  |  |  |  | R13A1.3 |  |
|  |  |  |  |  |  |  |  |  |  |  |  |  |  |  |  |  |  |  |  |  |  |  |  |  |  |  |  |  |  |  |  |  |  |  |  |  |  | C23H4.10 |  |
|  |  |  |  |  |  |  |  |  |  |  |  |  |  |  |  |  |  |  |  |  |  |  |  |  |  |  |  |  |  |  |  |  |  |  |  |  |  | F09E5.14 |  |
|  |  |  |  |  |  |  |  |  |  |  |  |  |  |  |  |  |  |  |  |  |  |  |  |  |  |  |  |  |  |  |  |  |  |  |  |  |  | E04F6.12 |  |
|  |  |  |  |  |  |  |  |  |  |  |  |  |  |  |  |  |  |  |  |  |  |  |  |  |  |  |  |  |  |  |  |  |  |  |  |  |  | C06E1.14 |  |
|  |  |  |  |  |  |  |  |  |  |  |  |  |  |  |  |  |  |  |  |  |  |  |  |  |  |  |  |  |  |  |  |  |  |  |  |  |  | *mps-1* | MiRP K channel accessory Subunit |
|  |  |  |  |  |  |  |  |  |  |  |  |  |  |  |  |  |  |  |  |  |  |  |  |  |  |  |  |  |  |  |  |  |  |  |  |  |  | F17C8.6 |  |
|  |  |  |  |  |  |  |  |  |  |  |  |  |  |  |  |  |  |  |  |  |  |  |  |  |  |  |  |  |  |  |  |  |  |  |  |  |  | *glb-15* | GLoBin related |
|  |  |  |  |  |  |  |  |  |  |  |  |  |  |  |  |  |  |  |  |  |  |  |  |  |  |  |  |  |  |  |  |  |  |  |  |  |  | *sre-4* | Serpentine Receptor, class E (epsilon) |
|  |  |  |  |  |  |  |  |  |  |  |  |  |  |  |  |  |  |  |  |  |  |  |  |  |  |  |  |  |  |  |  |  |  |  |  |  |  | B0563.7 |  |
|  |  |  |  |  |  |  |  |  |  |  |  |  |  |  |  |  |  |  |  |  |  |  |  |  |  |  |  |  |  |  |  |  |  |  |  |  |  | K07A12.5 |  |
|  |  |  |  |  |  |  |  |  |  |  |  |  |  |  |  |  |  |  |  |  |  |  |  |  |  |  |  |  |  |  |  |  |  |  |  |  |  | F59C6.17 |  |
|  |  |  |  |  |  |  |  |  |  |  |  |  |  |  |  |  |  |  |  |  |  |  |  |  |  |  |  |  |  |  |  |  |  |  |  |  |  | *glb-28* | GLoBin related |
|  |  |  |  |  |  |  |  |  |  |  |  |  |  |  |  |  |  |  |  |  |  |  |  |  |  |  |  |  |  |  |  |  |  |  |  |  |  | *dmsr-4* | DroMyoSuppressin Receptor related |
|  |  |  |  |  |  |  |  |  |  |  |  |  |  |  |  |  |  |  |  |  |  |  |  |  |  |  |  |  |  |  |  |  |  |  |  |  |  | *hot-1* | Homolog of Odr-2 (Two) |
|  |  |  |  |  |  |  |  |  |  |  |  |  |  |  |  |  |  |  |  |  |  |  |  |  |  |  |  |  |  |  |  |  |  |  |  |  |  | F17C8.3 |  |
|  |  |  |  |  |  |  |  |  |  |  |  |  |  |  |  |  |  |  |  |  |  |  |  |  |  |  |  |  |  |  |  |  |  |  |  |  |  | *acr-8* | AcetylCholine Receptor |
|  |  |  |  |  |  |  |  |  |  |  |  |  |  |  |  |  |  |  |  |  |  |  |  |  |  |  |  |  |  |  |  |  |  |  |  |  |  | *glb-4* | GLoBin related |
|  |  |  |  |  |  |  |  |  |  |  |  |  |  |  |  |  |  |  |  |  |  |  |  |  |  |  |  |  |  |  |  |  |  |  |  |  |  | W10C4.1 |  |
|  |  |  |  |  |  |  |  |  |  |  |  |  |  |  |  |  |  |  |  |  |  |  |  |  |  |  |  |  |  |  |  |  |  |  |  |  |  | B0034.5 |  |
|  |  |  |  |  |  |  |  |  |  |  |  |  |  |  |  |  |  |  |  |  |  |  |  |  |  |  |  |  |  |  |  |  |  |  |  |  |  | *bli-5* | BLIstered cuticle |
|  |  |  |  |  |  |  |  |  |  |  |  |  |  |  |  |  |  |  |  |  |  |  |  |  |  |  |  |  |  |  |  |  |  |  |  |  |  | T06F4.3 |  |
|  |  |  |  |  |  |  |  |  |  |  |  |  |  |  |  |  |  |  |  |  |  |  |  |  |  |  |  |  |  |  |  |  |  |  |  |  |  | Y70D2A.1 |  |
|  |  |  |  |  |  |  |  |  |  |  |  |  |  |  |  |  |  |  |  |  |  |  |  |  |  |  |  |  |  |  |  |  |  |  |  |  |  | C01H6.3 |  |
|  |  |  |  |  |  |  |  |  |  |  |  |  |  |  |  |  |  |  |  |  |  |  |  |  |  |  |  |  |  |  |  |  |  |  |  |  |  | F16B3.3 |  |
|  |  |  |  |  |  |  |  |  |  |  |  |  |  |  |  |  |  |  |  |  |  |  |  |  |  |  |  |  |  |  |  |  |  |  |  |  |  | *glb-27* | GLoBin related |
|  |  |  |  |  |  |  |  |  |  |  |  |  |  |  |  |  |  |  |  |  |  |  |  |  |  |  |  |  |  |  |  |  |  |  |  |  |  | C15C8.5 |  |
|  |  |  |  |  |  |  |  |  |  |  |  |  |  |  |  |  |  |  |  |  |  |  |  |  |  |  |  |  |  |  |  |  |  |  |  |  |  | *npr-13* | NeuroPeptide Receptor family |
|  |  |  |  |  |  |  |  |  |  |  |  |  |  |  |  |  |  |  |  |  |  |  |  |  |  |  |  |  |  |  |  |  |  |  |  |  |  | *nlp-9* | Neuropeptide-Like Protein |
|  |  |  |  |  |  |  |  |  |  |  |  |  |  |  |  |  |  |  |  |  |  |  |  |  |  |  |  |  |  |  |  |  |  |  |  |  |  | F59F5.5 |  |
|  |  |  |  |  |  |  |  |  |  |  |  |  |  |  |  |  |  |  |  |  |  |  |  |  |  |  |  |  |  |  |  |  |  |  |  |  |  | T19C3.3 |  |
|  |  |  |  |  |  |  |  |  |  |  |  |  |  |  |  |  |  |  |  |  |  |  |  |  |  |  |  |  |  |  |  |  |  |  |  |  |  | *gar-1* | G-protein-linked Acetylcholine Receptor |
|  |  |  |  |  |  |  |  |  |  |  |  |  |  |  |  |  |  |  |  |  |  |  |  |  |  |  |  |  |  |  |  |  |  |  |  |  |  | B0457.6 |  |
|  |  |  |  |  |  |  |  |  |  |  |  |  |  |  |  |  |  |  |  |  |  |  |  |  |  |  |  |  |  |  |  |  |  |  |  |  |  | C54G4.5 |  |
|  |  |  |  |  |  |  |  |  |  |  |  |  |  |  |  |  |  |  |  |  |  |  |  |  |  |  |  |  |  |  |  |  |  |  |  |  |  | F25H5.2 |  |
|  |  |  |  |  |  |  |  |  |  |  |  |  |  |  |  |  |  |  |  |  |  |  |  |  |  |  |  |  |  |  |  |  |  |  |  |  |  | T21C12.3 |  |
|  |  |  |  |  |  |  |  |  |  |  |  |  |  |  |  |  |  |  |  |  |  |  |  |  |  |  |  |  |  |  |  |  |  |  |  |  |  | C17H12.12 |  |
|  |  |  |  |  |  |  |  |  |  |  |  |  |  |  |  |  |  |  |  |  |  |  |  |  |  |  |  |  |  |  |  |  |  |  |  |  |  | F54G2.2 |  |
|  |  |  |  |  |  |  |  |  |  |  |  |  |  |  |  |  |  |  |  |  |  |  |  |  |  |  |  |  |  |  |  |  |  |  |  |  |  | W07A12.8 |  |
|  |  |  |  |  |  |  |  |  |  |  |  |  |  |  |  |  |  |  |  |  |  |  |  |  |  |  |  |  |  |  |  |  |  |  |  |  |  | C37C3.11 |  |
|  |  |  |  |  |  |  |  |  |  |  |  |  |  |  |  |  |  |  |  |  |  |  |  |  |  |  |  |  |  |  |  |  |  |  |  |  |  | R08A2.7 |  |
|  |  |  |  |  |  |  |  |  |  |  |  |  |  |  |  |  |  |  |  |  |  |  |  |  |  |  |  |  |  |  |  |  |  |  |  |  |  | R11D1.12 |  |
|  |  |  |  |  |  |  |  |  |  |  |  |  |  |  |  |  |  |  |  |  |  |  |  |  |  |  |  |  |  |  |  |  |  |  |  |  |  | C31H2.3 |  |
|  |  |  |  |  |  |  |  |  |  |  |  |  |  |  |  |  |  |  |  |  |  |  |  |  |  |  |  |  |  |  |  |  |  |  |  |  |  | T10B5.2 |  |
|  |  |  |  |  |  |  |  |  |  |  |  |  |  |  |  |  |  |  |  |  |  |  |  |  |  |  |  |  |  |  |  |  |  |  |  |  |  | *nhr-198* | Nuclear Hormone Receptor family |
|  |  |  |  |  |  |  |  |  |  |  |  |  |  |  |  |  |  |  |  |  |  |  |  |  |  |  |  |  |  |  |  |  |  |  |  |  |  | C16D9.6 |  |
|  |  |  |  |  |  |  |  |  |  |  |  |  |  |  |  |  |  |  |  |  |  |  |  |  |  |  |  |  |  |  |  |  |  |  |  |  |  | *odr-2* | ODoRant response abnormal |
|  |  |  |  |  |  |  |  |  |  |  |  |  |  |  |  |  |  |  |  |  |  |  |  |  |  |  |  |  |  |  |  |  |  |  |  |  |  | F27C1.10 |  |
|  |  |  |  |  |  |  |  |  |  |  |  |  |  |  |  |  |  |  |  |  |  |  |  |  |  |  |  |  |  |  |  |  |  |  |  |  |  | Y53G8AM.4 |  |
|  |  |  |  |  |  |  |  |  |  |  |  |  |  |  |  |  |  |  |  |  |  |  |  |  |  |  |  |  |  |  |  |  |  |  |  |  |  | *sox-4* | SOX (mammalian SRY box) family |
|  |  |  |  |  |  |  |  |  |  |  |  |  |  |  |  |  |  |  |  |  |  |  |  |  |  |  |  |  |  |  |  |  |  |  |  |  |  | *ensa-1* | ENdoSulfine Alpha |
|  |  |  |  |  |  |  |  |  |  |  |  |  |  |  |  |  |  |  |  |  |  |  |  |  |  |  |  |  |  |  |  |  |  |  |  |  |  | *odr-3* | ODoRant response abnormal |
|  |  |  |  |  |  |  |  |  |  |  |  |  |  |  |  |  |  |  |  |  |  |  |  |  |  |  |  |  |  |  |  |  |  |  |  |  |  | *dmd-10* | DM (Doublesex/MAB-3) Domain family |
|  |  |  |  |  |  |  |  |  |  |  |  |  |  |  |  |  |  |  |  |  |  |  |  |  |  |  |  |  |  |  |  |  |  |  |  |  |  | F27C1.11 |  |
|  |  |  |  |  |  |  |  |  |  |  |  |  |  |  |  |  |  |  |  |  |  |  |  |  |  |  |  |  |  |  |  |  |  |  |  |  |  | *daf-11* | abnormal DAuer Formation |
|  |  |  |  |  |  |  |  |  |  |  |  |  |  |  |  |  |  |  |  |  |  |  |  |  |  |  |  |  |  |  |  |  |  |  |  |  |  | ZK971.1 |  |
|  |  |  |  |  |  |  |  |  |  |  |  |  |  |  |  |  |  |  |  |  |  |  |  |  |  |  |  |  |  |  |  |  |  |  |  |  |  | *twk-48* | TWiK family of potassium channels |
|  |  |  |  |  |  |  |  |  |  |  |  |  |  |  |  |  |  |  |  |  |  |  |  |  |  |  |  |  |  |  |  |  |  |  |  |  |  | *flp-9* | FMRF-Like Peptide |
|  |  |  |  |  |  |  |  |  |  |  |  |  |  |  |  |  |  |  |  |  |  |  |  |  |  |  |  |  |  |  |  |  |  |  |  |  |  | Y71G12B.18 |  |
|  |  |  |  |  |  |  |  |  |  |  |  |  |  |  |  |  |  |  |  |  |  |  |  |  |  |  |  |  |  |  |  |  |  |  |  |  |  | F58H10.1 |  |
|  |  |  |  |  |  |  |  |  |  |  |  |  |  |  |  |  |  |  |  |  |  |  |  |  |  |  |  |  |  |  |  |  |  |  |  |  |  | *npr-35* | NeuroPeptide Receptor family |
|  |  |  |  |  |  |  |  |  |  |  |  |  |  |  |  |  |  |  |  |  |  |  |  |  |  |  |  |  |  |  |  |  |  |  |  |  |  | *glb-3* | GLoBin related |
|  |  |  |  |  |  |  |  |  |  |  |  |  |  |  |  |  |  |  |  |  |  |  |  |  |  |  |  |  |  |  |  |  |  |  |  |  |  | *dop-3* | DOPamine receptor |
|  |  |  |  |  |  |  |  |  |  |  |  |  |  |  |  |  |  |  |  |  |  |  |  |  |  |  |  |  |  |  |  |  |  |  |  |  |  | *rab-39* | RAB family |
|  |  |  |  |  |  |  |  |  |  |  |  |  |  |  |  |  |  |  |  |  |  |  |  |  |  |  |  |  |  |  |  |  |  |  |  |  |  | F58E10.7 |  |
|  |  |  |  |  |  |  |  |  |  |  |  |  |  |  |  |  |  |  |  |  |  |  |  |  |  |  |  |  |  |  |  |  |  |  |  |  |  | F20B6.4 |  |
|  |  |  |  |  |  |  |  |  |  |  |  |  |  |  |  |  |  |  |  |  |  |  |  |  |  |  |  |  |  |  |  |  |  |  |  |  |  | M02E1.2 |  |
|  |  |  |  |  |  |  |  |  |  |  |  |  |  |  |  |  |  |  |  |  |  |  |  |  |  |  |  |  |  |  |  |  |  |  |  |  |  | ZK1320.13 |  |
|  |  |  |  |  |  |  |  |  |  |  |  |  |  |  |  |  |  |  |  |  |  |  |  |  |  |  |  |  |  |  |  |  |  |  |  |  |  | F38E9.6 |  |
|  |  |  |  |  |  |  |  |  |  |  |  |  |  |  |  |  |  |  |  |  |  |  |  |  |  |  |  |  |  |  |  |  |  |  |  |  |  | F59B8.1 |  |
|  |  |  |  |  |  |  |  |  |  |  |  |  |  |  |  |  |  |  |  |  |  |  |  |  |  |  |  |  |  |  |  |  |  |  |  |  |  | *srg-30* | Serpentine Receptor, class G (gamma) |
|  |  |  |  |  |  |  |  |  |  |  |  |  |  |  |  |  |  |  |  |  |  |  |  |  |  |  |  |  |  |  |  |  |  |  |  |  |  | K03A1.4 |  |
|  |  |  |  |  |  |  |  |  |  |  |  |  |  |  |  |  |  |  |  |  |  |  |  |  |  |  |  |  |  |  |  |  |  |  |  |  |  | M03B6.1 |  |
|  |  |  |  |  |  |  |  |  |  |  |  |  |  |  |  |  |  |  |  |  |  |  |  |  |  |  |  |  |  |  |  |  |  |  |  |  |  | Y105C5B.25 |  |
|  |  |  |  |  |  |  |  |  |  |  |  |  |  |  |  |  |  |  |  |  |  |  |  |  |  |  |  |  |  |  |  |  |  |  |  |  |  | F59A1.11 |  |
|  |  |  |  |  |  |  |  |  |  |  |  |  |  |  |  |  |  |  |  |  |  |  |  |  |  |  |  |  |  |  |  |  |  |  |  |  |  | C27B7.9 |  |
|  |  |  |  |  |  |  |  |  |  |  |  |  |  |  |  |  |  |  |  |  |  |  |  |  |  |  |  |  |  |  |  |  |  |  |  |  |  | *ubc-22* | UBiquitin Conjugating enzyme |
|  |  |  |  |  |  |  |  |  |  |  |  |  |  |  |  |  |  |  |  |  |  |  |  |  |  |  |  |  |  |  |  |  |  |  |  |  |  | F25E5.9 |  |
|  |  |  |  |  |  |  |  |  |  |  |  |  |  |  |  |  |  |  |  |  |  |  |  |  |  |  |  |  |  |  |  |  |  |  |  |  |  | *ceh-54* | C. Elegans Homeobox |
|  |  |  |  |  |  |  |  |  |  |  |  |  |  |  |  |  |  |  |  |  |  |  |  |  |  |  |  |  |  |  |  |  |  |  |  |  |  | F09C3.2 |  |
|  |  |  |  |  |  |  |  |  |  |  |  |  |  |  |  |  |  |  |  |  |  |  |  |  |  |  |  |  |  |  |  |  |  |  |  |  |  | F55D10.4 |  |
|  |  |  |  |  |  |  |  |  |  |  |  |  |  |  |  |  |  |  |  |  |  |  |  |  |  |  |  |  |  |  |  |  |  |  |  |  |  | F53B1.3 |  |
|  |  |  |  |  |  |  |  |  |  |  |  |  |  |  |  |  |  |  |  |  |  |  |  |  |  |  |  |  |  |  |  |  |  |  |  |  |  | *cec-9* | C.Elegans Chromodomain protein |
|  |  |  |  |  |  |  |  |  |  |  |  |  |  |  |  |  |  |  |  |  |  |  |  |  |  |  |  |  |  |  |  |  |  |  |  |  |  | *mgl-2* | Metabotropic GLutamate receptor family |
|  |  |  |  |  |  |  |  |  |  |  |  |  |  |  |  |  |  |  |  |  |  |  |  |  |  |  |  |  |  |  |  |  |  |  |  |  |  | *maf-1* | MAF (MusculoAponeurotic Fibrosarcoma) transcription factor homolog |
|  |  |  |  |  |  |  |  |  |  |  |  |  |  |  |  |  |  |  |  |  |  |  |  |  |  |  |  |  |  |  |  |  |  |  |  |  |  | Y73B6BL.47 |  |
|  |  |  |  |  |  |  |  |  |  |  |  |  |  |  |  |  |  |  |  |  |  |  |  |  |  |  |  |  |  |  |  |  |  |  |  |  |  | Y73B6BL.270 |  |
|  |  |  |  |  |  |  |  |  |  |  |  |  |  |  |  |  |  |  |  |  |  |  |  |  |  |  |  |  |  |  |  |  |  |  |  |  |  | *neto-1* | NETO (NEuropilin and TOlloid-like) homolog |
|  |  |  |  |  |  |  |  |  |  |  |  |  |  |  |  |  |  |  |  |  |  |  |  |  |  |  |  |  |  |  |  |  |  |  |  |  |  | *glb-18* | GLoBin related |
|  |  |  |  |  |  |  |  |  |  |  |  |  |  |  |  |  |  |  |  |  |  |  |  |  |  |  |  |  |  |  |  |  |  |  |  |  |  | *stg-2* | STarGazin (mammalian calcium channel) homolog |
|  |  |  |  |  |  |  |  |  |  |  |  |  |  |  |  |  |  |  |  |  |  |  |  |  |  |  |  |  |  |  |  |  |  |  |  |  |  | T27A10.2 |  |
|  |  |  |  |  |  |  |  |  |  |  |  |  |  |  |  |  |  |  |  |  |  |  |  |  |  |  |  |  |  |  |  |  |  |  |  |  |  | F32B4.5 |  |
|  |  |  |  |  |  |  |  |  |  |  |  |  |  |  |  |  |  |  |  |  |  |  |  |  |  |  |  |  |  |  |  |  |  |  |  |  |  | ZK370.6 |  |
|  |  |  |  |  |  |  |  |  |  |  |  |  |  |  |  |  |  |  |  |  |  |  |  |  |  |  |  |  |  |  |  |  |  |  |  |  |  | Y38H6C.8 |  |
|  |  |  |  |  |  |  |  |  |  |  |  |  |  |  |  |  |  |  |  |  |  |  |  |  |  |  |  |  |  |  |  |  |  |  |  |  |  | *egl-2* | EGg Laying defective |
|  |  |  |  |  |  |  |  |  |  |  |  |  |  |  |  |  |  |  |  |  |  |  |  |  |  |  |  |  |  |  |  |  |  |  |  |  |  | C05G5.3 |  |
|  |  |  |  |  |  |  |  |  |  |  |  |  |  |  |  |  |  |  |  |  |  |  |  |  |  |  |  |  |  |  |  |  |  |  |  |  |  | F53C3.5 |  |
|  |  |  |  |  |  |  |  |  |  |  |  |  |  |  |  |  |  |  |  |  |  |  |  |  |  |  |  |  |  |  |  |  |  |  |  |  |  | T22E5.6 |  |
|  |  |  |  |  |  |  |  |  |  |  |  |  |  |  |  |  |  |  |  |  |  |  |  |  |  |  |  |  |  |  |  |  |  |  |  |  |  | *nhr-95* | Nuclear Hormone Receptor family |
|  |  |  |  |  |  |  |  |  |  |  |  |  |  |  |  |  |  |  |  |  |  |  |  |  |  |  |  |  |  |  |  |  |  |  |  |  |  | C40H5.2 |  |
|  |  |  |  |  |  |  |  |  |  |  |  |  |  |  |  |  |  |  |  |  |  |  |  |  |  |  |  |  |  |  |  |  |  |  |  |  |  | *ppk-2* | PIP Kinase |
|  |  |  |  |  |  |  |  |  |  |  |  |  |  |  |  |  |  |  |  |  |  |  |  |  |  |  |  |  |  |  |  |  |  |  |  |  |  | *ret-1* | RETiculon protein |
|  |  |  |  |  |  |  |  |  |  |  |  |  |  |  |  |  |  |  |  |  |  |  |  |  |  |  |  |  |  |  |  |  |  |  |  |  |  | R74.8 |  |
|  |  |  |  |  |  |  |  |  |  |  |  |  |  |  |  |  |  |  |  |  |  |  |  |  |  |  |  |  |  |  |  |  |  |  |  |  |  | B0491.6 |  |
|  |  |  |  |  |  |  |  |  |  |  |  |  |  |  |  |  |  |  |  |  |  |  |  |  |  |  |  |  |  |  |  |  |  |  |  |  |  | *tag-80* | Temporarily Assigned Gene name |
|  |  |  |  |  |  |  |  |  |  |  |  |  |  |  |  |  |  |  |  |  |  |  |  |  |  |  |  |  |  |  |  |  |  |  |  |  |  | *tag-199* | Temporarily Assigned Gene name |
|  |  |  |  |  |  |  |  |  |  |  |  |  |  |  |  |  |  |  |  |  |  |  |  |  |  |  |  |  |  |  |  |  |  |  |  |  |  | ZC190.10 |  |
|  |  |  |  |  |  |  |  |  |  |  |  |  |  |  |  |  |  |  |  |  |  |  |  |  |  |  |  |  |  |  |  |  |  |  |  |  |  | Y37D8A.4 |  |
|  |  |  |  |  |  |  |  |  |  |  |  |  |  |  |  |  |  |  |  |  |  |  |  |  |  |  |  |  |  |  |  |  |  |  |  |  |  | *acly-2* | ATP Citrate Lyas |
|  |  |  |  |  |  |  |  |  |  |  |  |  |  |  |  |  |  |  |  |  |  |  |  |  |  |  |  |  |  |  |  |  |  |  |  |  |  | Y57G11C.6 |  |
|  |  |  |  |  |  |  |  |  |  |  |  |  |  |  |  |  |  |  |  |  |  |  |  |  |  |  |  |  |  |  |  |  |  |  |  |  |  | ZK742.6 |  |
|  |  |  |  |  |  |  |  |  |  |  |  |  |  |  |  |  |  |  |  |  |  |  |  |  |  |  |  |  |  |  |  |  |  |  |  |  |  | *nlp-1* | Neuropeptide-Like Protein |
|  |  |  |  |  |  |  |  |  |  |  |  |  |  |  |  |  |  |  |  |  |  |  |  |  |  |  |  |  |  |  |  |  |  |  |  |  |  | Y57G11C.5 |  |
|  |  |  |  |  |  |  |  |  |  |  |  |  |  |  |  |  |  |  |  |  |  |  |  |  |  |  |  |  |  |  |  |  |  |  |  |  |  | *tcc-1* | Transmembrane and Coiled-Coil protein |
|  |  |  |  |  |  |  |  |  |  |  |  |  |  |  |  |  |  |  |  |  |  |  |  |  |  |  |  |  |  |  |  |  |  |  |  |  |  | *cyp-25A4* | CYtochrome P450 family |
|  |  |  |  |  |  |  |  |  |  |  |  |  |  |  |  |  |  |  |  |  |  |  |  |  |  |  |  |  |  |  |  |  |  |  |  |  |  | *nac-3* | NADC (Na+-coupled dicarboxylate transporter) family |
|  |  |  |  |  |  |  |  |  |  |  |  |  |  |  |  |  |  |  |  |  |  |  |  |  |  |  |  |  |  |  |  |  |  |  |  |  |  | *pqn-44* | Prion-like-(Q/N-rich)-domain-bearing protein |
|  |  |  |  |  |  |  |  |  |  |  |  |  |  |  |  |  |  |  |  |  |  |  |  |  |  |  |  |  |  |  |  |  |  |  |  |  |  | *ser-3* | SERotonin/octopamine receptor family |
|  |  |  |  |  |  |  |  |  |  |  |  |  |  |  |  |  |  |  |  |  |  |  |  |  |  |  |  |  |  |  |  |  |  |  |  |  |  | T20F10.4 |  |
|  |  |  |  |  |  |  |  |  |  |  |  |  |  |  |  |  |  |  |  |  |  |  |  |  |  |  |  |  |  |  |  |  |  |  |  |  |  | F25B5.3 |  |
|  |  |  |  |  |  |  |  |  |  |  |  |  |  |  |  |  |  |  |  |  |  |  |  |  |  |  |  |  |  |  |  |  |  |  |  |  |  | *upp-1* | Uridine PhosPhorylase |
|  |  |  |  |  |  |  |  |  |  |  |  |  |  |  |  |  |  |  |  |  |  |  |  |  |  |  |  |  |  |  |  |  |  |  |  |  |  | *xbp-1* | X-box Binding Protein homolog |
|  |  |  |  |  |  |  |  |  |  |  |  |  |  |  |  |  |  |  |  |  |  |  |  |  |  |  |  |  |  |  |  |  |  |  |  |  |  | *madf-4* | MADF domain transcription factor |
|  |  |  |  |  |  |  |  |  |  |  |  |  |  |  |  |  |  |  |  |  |  |  |  |  |  |  |  |  |  |  |  |  |  |  |  |  |  | *scrm-3* | SCRaMblase (phospholipid scramblase) |
|  |  |  |  |  |  |  |  |  |  |  |  |  |  |  |  |  |  |  |  |  |  |  |  |  |  |  |  |  |  |  |  |  |  |  |  |  |  | *nhr-145* | Nuclear Hormone Receptor family |
|  |  |  |  |  |  |  |  |  |  |  |  |  |  |  |  |  |  |  |  |  |  |  |  |  |  |  |  |  |  |  |  |  |  |  |  |  |  | Y95B8A.6 |  |
|  |  |  |  |  |  |  |  |  |  |  |  |  |  |  |  |  |  |  |  |  |  |  |  |  |  |  |  |  |  |  |  |  |  |  |  |  |  | W05B5.1 |  |
|  |  |  |  |  |  |  |  |  |  |  |  |  |  |  |  |  |  |  |  |  |  |  |  |  |  |  |  |  |  |  |  |  |  |  |  |  |  | *cnb-1* | CalciNeurin B |
|  |  |  |  |  |  |  |  |  |  |  |  |  |  |  |  |  |  |  |  |  |  |  |  |  |  |  |  |  |  |  |  |  |  |  |  |  |  | Y37F4.3 |  |
|  |  |  |  |  |  |  |  |  |  |  |  |  |  |  |  |  |  |  |  |  |  |  |  |  |  |  |  |  |  |  |  |  |  |  |  |  |  | *npr-22* | NeuroPeptide Receptor family |
|  |  |  |  |  |  |  |  |  |  |  |  |  |  |  |  |  |  |  |  |  |  |  |  |  |  |  |  |  |  |  |  |  |  |  |  |  |  | T27E4.7 |  |
|  |  |  |  |  |  |  |  |  |  |  |  |  |  |  |  |  |  |  |  |  |  |  |  |  |  |  |  |  |  |  |  |  |  |  |  |  |  | T22D1.5 |  |
|  |  |  |  |  |  |  |  |  |  |  |  |  |  |  |  |  |  |  |  |  |  |  |  |  |  |  |  |  |  |  |  |  |  |  |  |  |  | *ceh-9* | C. Elegans Homeobox |
|  |  |  |  |  |  |  |  |  |  |  |  |  |  |  |  |  |  |  |  |  |  |  |  |  |  |  |  |  |  |  |  |  |  |  |  |  |  | *cyp-14A2* | CYtochrome P450 family |
|  |  |  |  |  |  |  |  |  |  |  |  |  |  |  |  |  |  |  |  |  |  |  |  |  |  |  |  |  |  |  |  |  |  |  |  |  |  | *sox-3* | SOX (mammalian SRY box) family |
|  |  |  |  |  |  |  |  |  |  |  |  |  |  |  |  |  |  |  |  |  |  |  |  |  |  |  |  |  |  |  |  |  |  |  |  |  |  | C38H2.3 |  |
|  |  |  |  |  |  |  |  |  |  |  |  |  |  |  |  |  |  |  |  |  |  |  |  |  |  |  |  |  |  |  |  |  |  |  |  |  |  | K05B2.2 |  |
|  |  |  |  |  |  |  |  |  |  |  |  |  |  |  |  |  |  |  |  |  |  |  |  |  |  |  |  |  |  |  |  |  |  |  |  |  |  | *ced-1* | CEll Death abnormality |
|  |  |  |  |  |  |  |  |  |  |  |  |  |  |  |  |  |  |  |  |  |  |  |  |  |  |  |  |  |  |  |  |  |  |  |  |  |  | Y23H5A.10 |  |
|  |  |  |  |  |  |  |  |  |  |  |  |  |  |  |  |  |  |  |  |  |  |  |  |  |  |  |  |  |  |  |  |  |  |  |  |  |  | T08A9.4 |  |
|  |  |  |  |  |  |  |  |  |  |  |  |  |  |  |  |  |  |  |  |  |  |  |  |  |  |  |  |  |  |  |  |  |  |  |  |  |  | C30G4.2 |  |
|  |  |  |  |  |  |  |  |  |  |  |  |  |  |  |  |  |  |  |  |  |  |  |  |  |  |  |  |  |  |  |  |  |  |  |  |  |  | C16C8.19 |  |
|  |  |  |  |  |  |  |  |  |  |  |  |  |  |  |  |  |  |  |  |  |  |  |  |  |  |  |  |  |  |  |  |  |  |  |  |  |  | D2023.15 |  |
|  |  |  |  |  |  |  |  |  |  |  |  |  |  |  |  |  |  |  |  |  |  |  |  |  |  |  |  |  |  |  |  |  |  |  |  |  |  | *ser-6* | SERotonin/octopamine receptor family |
|  |  |  |  |  |  |  |  |  |  |  |  |  |  |  |  |  |  |  |  |  |  |  |  |  |  |  |  |  |  |  |  |  |  |  |  |  |  | *srd-12* | Serpentine Receptor, class D (delta) |
|  |  |  |  |  |  |  |  |  |  |  |  |  |  |  |  |  |  |  |  |  |  |  |  |  |  |  |  |  |  |  |  |  |  |  |  |  |  | F10F2.2 |  |
|  |  |  |  |  |  |  |  |  |  |  |  |  |  |  |  |  |  |  |  |  |  |  |  |  |  |  |  |  |  |  |  |  |  |  |  |  |  | *pbo-4* | PBOc defective (defecation) |
|  |  |  |  |  |  |  |  |  |  |  |  |  |  |  |  |  |  |  |  |  |  |  |  |  |  |  |  |  |  |  |  |  |  |  |  |  |  | H23N18.6 |  |
|  |  |  |  |  |  |  |  |  |  |  |  |  |  |  |  |  |  |  |  |  |  |  |  |  |  |  |  |  |  |  |  |  |  |  |  |  |  | *gpx-7* | Glutathione PeroXidase |
|  |  |  |  |  |  |  |  |  |  |  |  |  |  |  |  |  |  |  |  |  |  |  |  |  |  |  |  |  |  |  |  |  |  |  |  |  |  | *sprr-3* | Sex Peptide Receptor (Drosophila) Related |
|  |  |  |  |  |  |  |  |  |  |  |  |  |  |  |  |  |  |  |  |  |  |  |  |  |  |  |  |  |  |  |  |  |  |  |  |  |  | *nhr-27* | Nuclear Hormone Receptor family |
|  |  |  |  |  |  |  |  |  |  |  |  |  |  |  |  |  |  |  |  |  |  |  |  |  |  |  |  |  |  |  |  |  |  |  |  |  |  | F27B3.7 |  |
|  |  |  |  |  |  |  |  |  |  |  |  |  |  |  |  |  |  |  |  |  |  |  |  |  |  |  |  |  |  |  |  |  |  |  |  |  |  | F41D9.15 |  |
|  |  |  |  |  |  |  |  |  |  |  |  |  |  |  |  |  |  |  |  |  |  |  |  |  |  |  |  |  |  |  |  |  |  |  |  |  |  | *svh-1* | Suppressor of VHp-1 deletion lethality |
|  |  |  |  |  |  |  |  |  |  |  |  |  |  |  |  |  |  |  |  |  |  |  |  |  |  |  |  |  |  |  |  |  |  |  |  |  |  | *srv-6* | Serpentine Receptor, class V |
|  |  |  |  |  |  |  |  |  |  |  |  |  |  |  |  |  |  |  |  |  |  |  |  |  |  |  |  |  |  |  |  |  |  |  |  |  |  | Y37F4.1 |  |
|  |  |  |  |  |  |  |  |  |  |  |  |  |  |  |  |  |  |  |  |  |  |  |  |  |  |  |  |  |  |  |  |  |  |  |  |  |  | *acr-18* | AcetylCholine Receptor |
|  |  |  |  |  |  |  |  |  |  |  |  |  |  |  |  |  |  |  |  |  |  |  |  |  |  |  |  |  |  |  |  |  |  |  |  |  |  | *acr-14* | AcetylCholine Receptor |
|  |  |  |  |  |  |  |  |  |  |  |  |  |  |  |  |  |  |  |  |  |  |  |  |  |  |  |  |  |  |  |  |  |  |  |  |  |  | *nhr-167* | Nuclear Hormone Receptor family |
|  |  |  |  |  |  |  |  |  |  |  |  |  |  |  |  |  |  |  |  |  |  |  |  |  |  |  |  |  |  |  |  |  |  |  |  |  |  | R01E6.7 |  |
|  |  |  |  |  |  |  |  |  |  |  |  |  |  |  |  |  |  |  |  |  |  |  |  |  |  |  |  |  |  |  |  |  |  |  |  |  |  | C07A4.2 |  |
|  |  |  |  |  |  |  |  |  |  |  |  |  |  |  |  |  |  |  |  |  |  |  |  |  |  |  |  |  |  |  |  |  |  |  |  |  |  | *pde-1* | PhosphoDiEsterase |
|  |  |  |  |  |  |  |  |  |  |  |  |  |  |  |  |  |  |  |  |  |  |  |  |  |  |  |  |  |  |  |  |  |  |  |  |  |  | *flp-18* | FMRF-Like Peptide |
|  |  |  |  |  |  |  |  |  |  |  |  |  |  |  |  |  |  |  |  |  |  |  |  |  |  |  |  |  |  |  |  |  |  |  |  |  |  | ZK721.3 |  |
|  |  |  |  |  |  |  |  |  |  |  |  |  |  |  |  |  |  |  |  |  |  |  |  |  |  |  |  |  |  |  |  |  |  |  |  |  |  | *gpa-13* | G Protein, Alpha subunit |
|  |  |  |  |  |  |  |  |  |  |  |  |  |  |  |  |  |  |  |  |  |  |  |  |  |  |  |  |  |  |  |  |  |  |  |  |  |  | *nlp-3* | Neuropeptide-Like Protein |
|  |  |  |  |  |  |  |  |  |  |  |  |  |  |  |  |  |  |  |  |  |  |  |  |  |  |  |  |  |  |  |  |  |  |  |  |  |  | *npr-4* | NeuroPeptide Receptor family |
|  |  |  |  |  |  |  |  |  |  |  |  |  |  |  |  |  |  |  |  |  |  |  |  |  |  |  |  |  |  |  |  |  |  |  |  |  |  | *npr-7* | NeuroPeptide Receptor family |
|  |  |  |  |  |  |  |  |  |  |  |  |  |  |  |  |  |  |  |  |  |  |  |  |  |  |  |  |  |  |  |  |  |  |  |  |  |  | *twk-2* | TWiK family of potassium channels |
|  |  |  |  |  |  |  |  |  |  |  |  |  |  |  |  |  |  |  |  |  |  |  |  |  |  |  |  |  |  |  |  |  |  |  |  |  |  | *npr-9* | NeuroPeptide Receptor family |
|  |  |  |  |  |  |  |  |  |  |  |  |  |  |  |  |  |  |  |  |  |  |  |  |  |  |  |  |  |  |  |  |  |  |  |  |  |  | *slo-2* | SLOwpoke potassium channel family |
|  |  |  |  |  |  |  |  |  |  |  |  |  |  |  |  |  |  |  |  |  |  |  |  |  |  |  |  |  |  |  |  |  |  |  |  |  |  | C05D12.7 |  |
|  |  |  |  |  |  |  |  |  |  |  |  |  |  |  |  |  |  |  |  |  |  |  |  |  |  |  |  |  |  |  |  |  |  |  |  |  |  | *dgk-3* | DiacylGlycerol Kinase |
|  |  |  |  |  |  |  |  |  |  |  |  |  |  |  |  |  |  |  |  |  |  |  |  |  |  |  |  |  |  |  |  |  |  |  |  |  |  | *twk-32* | TWiK family of potassium channels |
|  |  |  |  |  |  |  |  |  |  |  |  |  |  |  |  |  |  |  |  |  |  |  |  |  |  |  |  |  |  |  |  |  |  |  |  |  |  | T02E1.7 |  |
|  |  |  |  |  |  |  |  |  |  |  |  |  |  |  |  |  |  |  |  |  |  |  |  |  |  |  |  |  |  |  |  |  |  |  |  |  |  | *paf-2* | PAF-acetylhydrolase |
|  |  |  |  |  |  |  |  |  |  |  |  |  |  |  |  |  |  |  |  |  |  |  |  |  |  |  |  |  |  |  |  |  |  |  |  |  |  | *tiam-1* | TIAM (mammalian Tumor Invasion And Metastasis factor) homolog |
|  |  |  |  |  |  |  |  |  |  |  |  |  |  |  |  |  |  |  |  |  |  |  |  |  |  |  |  |  |  |  |  |  |  |  |  |  |  | F58B4.2 |  |
|  |  |  |  |  |  |  |  |  |  |  |  |  |  |  |  |  |  |  |  |  |  |  |  |  |  |  |  |  |  |  |  |  |  |  |  |  |  | K01B6.4 |  |
|  |  |  |  |  |  |  |  |  |  |  |  |  |  |  |  |  |  |  |  |  |  |  |  |  |  |  |  |  |  |  |  |  |  |  |  |  |  | Y43F4A.4 |  |
|  |  |  |  |  |  |  |  |  |  |  |  |  |  |  |  |  |  |  |  |  |  |  |  |  |  |  |  |  |  |  |  |  |  |  |  |  |  | *nas-4* | Nematode AStacin protease |
|  |  |  |  |  |  |  |  |  |  |  |  |  |  |  |  |  |  |  |  |  |  |  |  |  |  |  |  |  |  |  |  |  |  |  |  |  |  | F46G11.1 |  |
|  |  |  |  |  |  |  |  |  |  |  |  |  |  |  |  |  |  |  |  |  |  |  |  |  |  |  |  |  |  |  |  |  |  |  |  |  |  | T02E1.6 |  |
|  |  |  |  |  |  |  |  |  |  |  |  |  |  |  |  |  |  |  |  |  |  |  |  |  |  |  |  |  |  |  |  |  |  |  |  |  |  | C11G6.3 |  |
|  |  |  |  |  |  |  |  |  |  |  |  |  |  |  |  |  |  |  |  |  |  |  |  |  |  |  |  |  |  |  |  |  |  |  |  |  |  | *glb-31* | GLoBin related |
|  |  |  |  |  |  |  |  |  |  |  |  |  |  |  |  |  |  |  |  |  |  |  |  |  |  |  |  |  |  |  |  |  |  |  |  |  |  | F49E12.7 |  |
|  |  |  |  |  |  |  |  |  |  |  |  |  |  |  |  |  |  |  |  |  |  |  |  |  |  |  |  |  |  |  |  |  |  |  |  |  |  | *glr-3* | GLutamate Receptor family (AMPA) |
|  |  |  |  |  |  |  |  |  |  |  |  |  |  |  |  |  |  |  |  |  |  |  |  |  |  |  |  |  |  |  |  |  |  |  |  |  |  | *amt-3* | AMmonium Transporter homolog |
|  |  |  |  |  |  |  |  |  |  |  |  |  |  |  |  |  |  |  |  |  |  |  |  |  |  |  |  |  |  |  |  |  |  |  |  |  |  | C13G3.1 |  |
|  |  |  |  |  |  |  |  |  |  |  |  |  |  |  |  |  |  |  |  |  |  |  |  |  |  |  |  |  |  |  |  |  |  |  |  |  |  | F14F11.2 |  |
|  |  |  |  |  |  |  |  |  |  |  |  |  |  |  |  |  |  |  |  |  |  |  |  |  |  |  |  |  |  |  |  |  |  |  |  |  |  | R01H2.7 |  |
|  |  |  |  |  |  |  |  |  |  |  |  |  |  |  |  |  |  |  |  |  |  |  |  |  |  |  |  |  |  |  |  |  |  |  |  |  |  | B0496.6 |  |
|  |  |  |  |  |  |  |  |  |  |  |  |  |  |  |  |  |  |  |  |  |  |  |  |  |  |  |  |  |  |  |  |  |  |  |  |  |  | C10E2.2 |  |
|  |  |  |  |  |  |  |  |  |  |  |  |  |  |  |  |  |  |  |  |  |  |  |  |  |  |  |  |  |  |  |  |  |  |  |  |  |  | K07C10.2 |  |
|  |  |  |  |  |  |  |  |  |  |  |  |  |  |  |  |  |  |  |  |  |  |  |  |  |  |  |  |  |  |  |  |  |  |  |  |  |  | C13G3.9 |  |
|  |  |  |  |  |  |  |  |  |  |  |  |  |  |  |  |  |  |  |  |  |  |  |  |  |  |  |  |  |  |  |  |  |  |  |  |  |  | *ceh-62* | C. Elegans Homeobox |
|  |  |  |  |  |  |  |  |  |  |  |  |  |  |  |  |  |  |  |  |  |  |  |  |  |  |  |  |  |  |  |  |  |  |  |  |  |  | *cpx-1* | ComPleXin (synaptic protein) homolog |
|  |  |  |  |  |  |  |  |  |  |  |  |  |  |  |  |  |  |  |  |  |  |  |  |  |  |  |  |  |  |  |  |  |  |  |  |  |  | *pghm-1* | PeptidylGlycine-alpha-Hydroxylating Monooxygenase |
|  |  |  |  |  |  |  |  |  |  |  |  |  |  |  |  |  |  |  |  |  |  |  |  |  |  |  |  |  |  |  |  |  |  |  |  |  |  | T02C12.5 |  |
|  |  |  |  |  |  |  |  |  |  |  |  |  |  |  |  |  |  |  |  |  |  |  |  |  |  |  |  |  |  |  |  |  |  |  |  |  |  | E04D5.5 |  |
|  |  |  |  |  |  |  |  |  |  |  |  |  |  |  |  |  |  |  |  |  |  |  |  |  |  |  |  |  |  |  |  |  |  |  |  |  |  | *che-12* | abnormal CHEmotaxis |
|  |  |  |  |  |  |  |  |  |  |  |  |  |  |  |  |  |  |  |  |  |  |  |  |  |  |  |  |  |  |  |  |  |  |  |  |  |  | C53C7.5 |  |
|  |  |  |  |  |  |  |  |  |  |  |  |  |  |  |  |  |  |  |  |  |  |  |  |  |  |  |  |  |  |  |  |  |  |  |  |  |  | C56G7.3 |  |
|  |  |  |  |  |  |  |  |  |  |  |  |  |  |  |  |  |  |  |  |  |  |  |  |  |  |  |  |  |  |  |  |  |  |  |  |  |  | ZK1320.5 |  |
|  |  |  |  |  |  |  |  |  |  |  |  |  |  |  |  |  |  |  |  |  |  |  |  |  |  |  |  |  |  |  |  |  |  |  |  |  |  | C25B8.8 |  |
|  |  |  |  |  |  |  |  |  |  |  |  |  |  |  |  |  |  |  |  |  |  |  |  |  |  |  |  |  |  |  |  |  |  |  |  |  |  | C35E7.11 |  |
|  |  |  |  |  |  |  |  |  |  |  |  |  |  |  |  |  |  |  |  |  |  |  |  |  |  |  |  |  |  |  |  |  |  |  |  |  |  | C35E7.2 |  |
|  |  |  |  |  |  |  |  |  |  |  |  |  |  |  |  |  |  |  |  |  |  |  |  |  |  |  |  |  |  |  |  |  |  |  |  |  |  | C35D10.12 |  |
|  |  |  |  |  |  |  |  |  |  |  |  |  |  |  |  |  |  |  |  |  |  |  |  |  |  |  |  |  |  |  |  |  |  |  |  |  |  | F39H12.3 |  |
|  |  |  |  |  |  |  |  |  |  |  |  |  |  |  |  |  |  |  |  |  |  |  |  |  |  |  |  |  |  |  |  |  |  |  |  |  |  | F41E7.9 |  |
|  |  |  |  |  |  |  |  |  |  |  |  |  |  |  |  |  |  |  |  |  |  |  |  |  |  |  |  |  |  |  |  |  |  |  |  |  |  | *dyf-6* | abnormal DYe Filling |
|  |  |  |  |  |  |  |  |  |  |  |  |  |  |  |  |  |  |  |  |  |  |  |  |  |  |  |  |  |  |  |  |  |  |  |  |  |  | *lgc-38* | Ligand-Gated ion Channel |
|  |  |  |  |  |  |  |  |  |  |  |  |  |  |  |  |  |  |  |  |  |  |  |  |  |  |  |  |  |  |  |  |  |  |  |  |  |  | W01A8.7 |  |
|  |  |  |  |  |  |  |  |  |  |  |  |  |  |  |  |  |  |  |  |  |  |  |  |  |  |  |  |  |  |  |  |  |  |  |  |  |  | T12B3.1 |  |
|  |  |  |  |  |  |  |  |  |  |  |  |  |  |  |  |  |  |  |  |  |  |  |  |  |  |  |  |  |  |  |  |  |  |  |  |  |  | *rgs-3* | Regulator of G protein Signaling |
|  |  |  |  |  |  |  |  |  |  |  |  |  |  |  |  |  |  |  |  |  |  |  |  |  |  |  |  |  |  |  |  |  |  |  |  |  |  | F10E9.1 |  |
|  |  |  |  |  |  |  |  |  |  |  |  |  |  |  |  |  |  |  |  |  |  |  |  |  |  |  |  |  |  |  |  |  |  |  |  |  |  | T21B4.15 |  |
|  |  |  |  |  |  |  |  |  |  |  |  |  |  |  |  |  |  |  |  |  |  |  |  |  |  |  |  |  |  |  |  |  |  |  |  |  |  | F18E9.1 |  |
|  |  |  |  |  |  |  |  |  |  |  |  |  |  |  |  |  |  |  |  |  |  |  |  |  |  |  |  |  |  |  |  |  |  |  |  |  |  | F35H12.6 |  |
|  |  |  |  |  |  |  |  |  |  |  |  |  |  |  |  |  |  |  |  |  |  |  |  |  |  |  |  |  |  |  |  |  |  |  |  |  |  | *mbr-1* | honeybee MBlk-1 Related factor |
|  |  |  |  |  |  |  |  |  |  |  |  |  |  |  |  |  |  |  |  |  |  |  |  |  |  |  |  |  |  |  |  |  |  |  |  |  |  | *nhr-4* | Nuclear Hormone Receptor family |
|  |  |  |  |  |  |  |  |  |  |  |  |  |  |  |  |  |  |  |  |  |  |  |  |  |  |  |  |  |  |  |  |  |  |  |  |  |  | *trk-1* | TRK (vertebrate neurotrophin receptor tyrosine kinase) homolog |
|  |  |  |  |  |  |  |  |  |  |  |  |  |  |  |  |  |  |  |  |  |  |  |  |  |  |  |  |  |  |  |  |  |  |  |  |  |  | W07G1.5 |  |
|  |  |  |  |  |  |  |  |  |  |  |  |  |  |  |  |  |  |  |  |  |  |  |  |  |  |  |  |  |  |  |  |  |  |  |  |  |  | F54C1.6 |  |
|  |  |  |  |  |  |  |  |  |  |  |  |  |  |  |  |  |  |  |  |  |  |  |  |  |  |  |  |  |  |  |  |  |  |  |  |  |  | *inx-19* | INneXin |
|  |  |  |  |  |  |  |  |  |  |  |  |  |  |  |  |  |  |  |  |  |  |  |  |  |  |  |  |  |  |  |  |  |  |  |  |  |  | *arrd-6* | ARRestin Domain protein |
|  |  |  |  |  |  |  |  |  |  |  |  |  |  |  |  |  |  |  |  |  |  |  |  |  |  |  |  |  |  |  |  |  |  |  |  |  |  | F13E6.2 |  |
|  |  |  |  |  |  |  |  |  |  |  |  |  |  |  |  |  |  |  |  |  |  |  |  |  |  |  |  |  |  |  |  |  |  |  |  |  |  | *daf-38* |  |
|  |  |  |  |  |  |  |  |  |  |  |  |  |  |  |  |  |  |  |  |  |  |  |  |  |  |  |  |  |  |  |  |  |  |  |  |  |  | *rab-37* | RAB family |
|  |  |  |  |  |  |  |  |  |  |  |  |  |  |  |  |  |  |  |  |  |  |  |  |  |  |  |  |  |  |  |  |  |  |  |  |  |  | Y54E5A.2 |  |
|  |  |  |  |  |  |  |  |  |  |  |  |  |  |  |  |  |  |  |  |  |  |  |  |  |  |  |  |  |  |  |  |  |  |  |  |  |  | *mek-1* | MAP kinase kinase or Erk Kinase |
|  |  |  |  |  |  |  |  |  |  |  |  |  |  |  |  |  |  |  |  |  |  |  |  |  |  |  |  |  |  |  |  |  |  |  |  |  |  | *tax-2* | abnormal CHEmotaxis |
|  |  |  |  |  |  |  |  |  |  |  |  |  |  |  |  |  |  |  |  |  |  |  |  |  |  |  |  |  |  |  |  |  |  |  |  |  |  | T06G6.3 |  |
|  |  |  |  |  |  |  |  |  |  |  |  |  |  |  |  |  |  |  |  |  |  |  |  |  |  |  |  |  |  |  |  |  |  |  |  |  |  | F38E11.6 |  |
|  |  |  |  |  |  |  |  |  |  |  |  |  |  |  |  |  |  |  |  |  |  |  |  |  |  |  |  |  |  |  |  |  |  |  |  |  |  | *mod-1* | Modulation Of locomotion Defective |
|  |  |  |  |  |  |  |  |  |  |  |  |  |  |  |  |  |  |  |  |  |  |  |  |  |  |  |  |  |  |  |  |  |  |  |  |  |  | *dgk-5* | DiacylGlycerol Kinase |
|  |  |  |  |  |  |  |  |  |  |  |  |  |  |  |  |  |  |  |  |  |  |  |  |  |  |  |  |  |  |  |  |  |  |  |  |  |  | *tax-4* | abnormal CHEmotaxis |
|  |  |  |  |  |  |  |  |  |  |  |  |  |  |  |  |  |  |  |  |  |  |  |  |  |  |  |  |  |  |  |  |  |  |  |  |  |  | *nhr-158* | Nuclear Hormone Receptor family |
|  |  |  |  |  |  |  |  |  |  |  |  |  |  |  |  |  |  |  |  |  |  |  |  |  |  |  |  |  |  |  |  |  |  |  |  |  |  | *gem-1* | Gon-2 Extragenic Modifier |
|  |  |  |  |  |  |  |  |  |  |  |  |  |  |  |  |  |  |  |  |  |  |  |  |  |  |  |  |  |  |  |  |  |  |  |  |  |  | F34D10.6 |  |
|  |  |  |  |  |  |  |  |  |  |  |  |  |  |  |  |  |  |  |  |  |  |  |  |  |  |  |  |  |  |  |  |  |  |  |  |  |  | *nphp-2* | NePHronoPhthisis (human kidney disease) homolog |
|  |  |  |  |  |  |  |  |  |  |  |  |  |  |  |  |  |  |  |  |  |  |  |  |  |  |  |  |  |  |  |  |  |  |  |  |  |  | Y108G3AL.3 |  |
|  |  |  |  |  |  |  |  |  |  |  |  |  |  |  |  |  |  |  |  |  |  |  |  |  |  |  |  |  |  |  |  |  |  |  |  |  |  | Y17D7B.2 |  |
|  |  |  |  |  |  |  |  |  |  |  |  |  |  |  |  |  |  |  |  |  |  |  |  |  |  |  |  |  |  |  |  |  |  |  |  |  |  | Y55D5A.1 |  |
|  |  |  |  |  |  |  |  |  |  |  |  |  |  |  |  |  |  |  |  |  |  |  |  |  |  |  |  |  |  |  |  |  |  |  |  |  |  | *nhr-138* | Nuclear Hormone Receptor family |
|  |  |  |  |  |  |  |  |  |  |  |  |  |  |  |  |  |  |  |  |  |  |  |  |  |  |  |  |  |  |  |  |  |  |  |  |  |  | *nhr-100* | Nuclear Hormone Receptor family |
|  |  |  |  |  |  |  |  |  |  |  |  |  |  |  |  |  |  |  |  |  |  |  |  |  |  |  |  |  |  |  |  |  |  |  |  |  |  | F07A11.4 |  |
|  |  |  |  |  |  |  |  |  |  |  |  |  |  |  |  |  |  |  |  |  |  |  |  |  |  |  |  |  |  |  |  |  |  |  |  |  |  | C46C11.3 |  |
|  |  |  |  |  |  |  |  |  |  |  |  |  |  |  |  |  |  |  |  |  |  |  |  |  |  |  |  |  |  |  |  |  |  |  |  |  |  | *wrk-1* | Wrapper/Rega-1/Klingon homolog |
|  |  |  |  |  |  |  |  |  |  |  |  |  |  |  |  |  |  |  |  |  |  |  |  |  |  |  |  |  |  |  |  |  |  |  |  |  |  | Y53G8AM.7 |  |
|  |  |  |  |  |  |  |  |  |  |  |  |  |  |  |  |  |  |  |  |  |  |  |  |  |  |  |  |  |  |  |  |  |  |  |  |  |  | Y67D8B.2 |  |
|  |  |  |  |  |  |  |  |  |  |  |  |  |  |  |  |  |  |  |  |  |  |  |  |  |  |  |  |  |  |  |  |  |  |  |  |  |  | B0222.1 |  |
|  |  |  |  |  |  |  |  |  |  |  |  |  |  |  |  |  |  |  |  |  |  |  |  |  |  |  |  |  |  |  |  |  |  |  |  |  |  | T05G5.5 |  |
|  |  |  |  |  |  |  |  |  |  |  |  |  |  |  |  |  |  |  |  |  |  |  |  |  |  |  |  |  |  |  |  |  |  |  |  |  |  | *gpc-1* | G Protein, Gamma subunit |
|  |  |  |  |  |  |  |  |  |  |  |  |  |  |  |  |  |  |  |  |  |  |  |  |  |  |  |  |  |  |  |  |  |  |  |  |  |  | *hsp-16.1* | Heat Shock Protein |
|  |  |  |  |  |  |  |  |  |  |  |  |  |  |  |  |  |  |  |  |  |  |  |  |  |  |  |  |  |  |  |  |  |  |  |  |  |  | *hsp-16.49* | Heat Shock Protein |
|  |  |  |  |  |  |  |  |  |  |  |  |  |  |  |  |  |  |  |  |  |  |  |  |  |  |  |  |  |  |  |  |  |  |  |  |  |  | *nhr-159* | Nuclear Hormone Receptor family |
|  |  |  |  |  |  |  |  |  |  |  |  |  |  |  |  |  |  |  |  |  |  |  |  |  |  |  |  |  |  |  |  |  |  |  |  |  |  | *cwp-2* | Coexpressed With Polycystins |
|  |  |  |  |  |  |  |  |  |  |  |  |  |  |  |  |  |  |  |  |  |  |  |  |  |  |  |  |  |  |  |  |  |  |  |  |  |  | F49C5.7 |  |
|  |  |  |  |  |  |  |  |  |  |  |  |  |  |  |  |  |  |  |  |  |  |  |  |  |  |  |  |  |  |  |  |  |  |  |  |  |  | F44E5.4 |  |
|  |  |  |  |  |  |  |  |  |  |  |  |  |  |  |  |  |  |  |  |  |  |  |  |  |  |  |  |  |  |  |  |  |  |  |  |  |  | F44E5.5 |  |
|  |  |  |  |  |  |  |  |  |  |  |  |  |  |  |  |  |  |  |  |  |  |  |  |  |  |  |  |  |  |  |  |  |  |  |  |  |  | F32E10.9 |  |
|  |  |  |  |  |  |  |  |  |  |  |  |  |  |  |  |  |  |  |  |  |  |  |  |  |  |  |  |  |  |  |  |  |  |  |  |  |  | *swt-1* | SWEET sugar transporter family |
|  |  |  |  |  |  |  |  |  |  |  |  |  |  |  |  |  |  |  |  |  |  |  |  |  |  |  |  |  |  |  |  |  |  |  |  |  |  | C16D2.2 |  |
|  |  |  |  |  |  |  |  |  |  |  |  |  |  |  |  |  |  |  |  |  |  |  |  |  |  |  |  |  |  |  |  |  |  |  |  |  |  | W05B10.6 |  |
|  |  |  |  |  |  |  |  |  |  |  |  |  |  |  |  |  |  |  |  |  |  |  |  |  |  |  |  |  |  |  |  |  |  |  |  |  |  | Y73B6BL.31 |  |
|  |  |  |  |  |  |  |  |  |  |  |  |  |  |  |  |  |  |  |  |  |  |  |  |  |  |  |  |  |  |  |  |  |  |  |  |  |  | C06E2.2 |  |
|  |  |  |  |  |  |  |  |  |  |  |  |  |  |  |  |  |  |  |  |  |  |  |  |  |  |  |  |  |  |  |  |  |  |  |  |  |  | Y41D4B.1 |  |
|  |  |  |  |  |  |  |  |  |  |  |  |  |  |  |  |  |  |  |  |  |  |  |  |  |  |  |  |  |  |  |  |  |  |  |  |  |  | F52D10.4 |  |
|  |  |  |  |  |  |  |  |  |  |  |  |  |  |  |  |  |  |  |  |  |  |  |  |  |  |  |  |  |  |  |  |  |  |  |  |  |  | F36F2.8 |  |
|  |  |  |  |  |  |  |  |  |  |  |  |  |  |  |  |  |  |  |  |  |  |  |  |  |  |  |  |  |  |  |  |  |  |  |  |  |  | *nhr-33* | Nuclear Hormone Receptor family |
|  |  |  |  |  |  |  |  |  |  |  |  |  |  |  |  |  |  |  |  |  |  |  |  |  |  |  |  |  |  |  |  |  |  |  |  |  |  | *egl-38* | EGg Laying defective |
|  |  |  |  |  |  |  |  |  |  |  |  |  |  |  |  |  |  |  |  |  |  |  |  |  |  |  |  |  |  |  |  |  |  |  |  |  |  | F46F11.6 |  |
|  |  |  |  |  |  |  |  |  |  |  |  |  |  |  |  |  |  |  |  |  |  |  |  |  |  |  |  |  |  |  |  |  |  |  |  |  |  | *lin-14* | abnormal cell LINeage |
|  |  |  |  |  |  |  |  |  |  |  |  |  |  |  |  |  |  |  |  |  |  |  |  |  |  |  |  |  |  |  |  |  |  |  |  |  |  | *coel-1* | tubulin folding COfactor E-Like protein |
|  |  |  |  |  |  |  |  |  |  |  |  |  |  |  |  |  |  |  |  |  |  |  |  |  |  |  |  |  |  |  |  |  |  |  |  |  |  | F37A8.5 |  |
|  |  |  |  |  |  |  |  |  |  |  |  |  |  |  |  |  |  |  |  |  |  |  |  |  |  |  |  |  |  |  |  |  |  |  |  |  |  | C04G2.8 |  |
|  |  |  |  |  |  |  |  |  |  |  |  |  |  |  |  |  |  |  |  |  |  |  |  |  |  |  |  |  |  |  |  |  |  |  |  |  |  | *fbxa-150* | F-box A protein |
|  |  |  |  |  |  |  |  |  |  |  |  |  |  |  |  |  |  |  |  |  |  |  |  |  |  |  |  |  |  |  |  |  |  |  |  |  |  | Y37D8A.6 |  |
|  |  |  |  |  |  |  |  |  |  |  |  |  |  |  |  |  |  |  |  |  |  |  |  |  |  |  |  |  |  |  |  |  |  |  |  |  |  | K06G5.3 |  |
|  |  |  |  |  |  |  |  |  |  |  |  |  |  |  |  |  |  |  |  |  |  |  |  |  |  |  |  |  |  |  |  |  |  |  |  |  |  | T18D3.6 |  |
|  |  |  |  |  |  |  |  |  |  |  |  |  |  |  |  |  |  |  |  |  |  |  |  |  |  |  |  |  |  |  |  |  |  |  |  |  |  | *saeg-1* | Suppressor of Activated EGL-4 |
|  |  |  |  |  |  |  |  |  |  |  |  |  |  |  |  |  |  |  |  |  |  |  |  |  |  |  |  |  |  |  |  |  |  |  |  |  |  | *mec-2* | MEChanosensory abnormality |
|  |  |  |  |  |  |  |  |  |  |  |  |  |  |  |  |  |  |  |  |  |  |  |  |  |  |  |  |  |  |  |  |  |  |  |  |  |  | *tiar-3* | TIA-1/TIAL RNA binding protein homolog |
|  |  |  |  |  |  |  |  |  |  |  |  |  |  |  |  |  |  |  |  |  |  |  |  |  |  |  |  |  |  |  |  |  |  |  |  |  |  | F54D10.3 |  |
|  |  |  |  |  |  |  |  |  |  |  |  |  |  |  |  |  |  |  |  |  |  |  |  |  |  |  |  |  |  |  |  |  |  |  |  |  |  | W04A4.6 |  |
|  |  |  |  |  |  |  |  |  |  |  |  |  |  |  |  |  |  |  |  |  |  |  |  |  |  |  |  |  |  |  |  |  |  |  |  |  |  | Y38F1A.2 |  |
|  |  |  |  |  |  |  |  |  |  |  |  |  |  |  |  |  |  |  |  |  |  |  |  |  |  |  |  |  |  |  |  |  |  |  |  |  |  | R05C11.4 |  |
|  |  |  |  |  |  |  |  |  |  |  |  |  |  |  |  |  |  |  |  |  |  |  |  |  |  |  |  |  |  |  |  |  |  |  |  |  |  | *vab-8* | Variable ABnormal morphology |
|  |  |  |  |  |  |  |  |  |  |  |  |  |  |  |  |  |  |  |  |  |  |  |  |  |  |  |  |  |  |  |  |  |  |  |  |  |  | *pef-1* | Phosphatase with EF hands |
|  |  |  |  |  |  |  |  |  |  |  |  |  |  |  |  |  |  |  |  |  |  |  |  |  |  |  |  |  |  |  |  |  |  |  |  |  |  | *nhr-105* | Nuclear Hormone Receptor family |
|  |  |  |  |  |  |  |  |  |  |  |  |  |  |  |  |  |  |  |  |  |  |  |  |  |  |  |  |  |  |  |  |  |  |  |  |  |  | *acr-15* | AcetylCholine Receptor |
|  |  |  |  |  |  |  |  |  |  |  |  |  |  |  |  |  |  |  |  |  |  |  |  |  |  |  |  |  |  |  |  |  |  |  |  |  |  | *acr-16* | AcetylCholine Receptor |
|  |  |  |  |  |  |  |  |  |  |  |  |  |  |  |  |  |  |  |  |  |  |  |  |  |  |  |  |  |  |  |  |  |  |  |  |  |  | *seb-3* | SEcretin/class B GPCR |
|  |  |  |  |  |  |  |  |  |  |  |  |  |  |  |  |  |  |  |  |  |  |  |  |  |  |  |  |  |  |  |  |  |  |  |  |  |  | *cpd-2* | CarboxyPeptidase D family |
|  |  |  |  |  |  |  |  |  |  |  |  |  |  |  |  |  |  |  |  |  |  |  |  |  |  |  |  |  |  |  |  |  |  |  |  |  |  | E04F6.10 |  |
|  |  |  |  |  |  |  |  |  |  |  |  |  |  |  |  |  |  |  |  |  |  |  |  |  |  |  |  |  |  |  |  |  |  |  |  |  |  | C08G5.5 |  |
|  |  |  |  |  |  |  |  |  |  |  |  |  |  |  |  |  |  |  |  |  |  |  |  |  |  |  |  |  |  |  |  |  |  |  |  |  |  | C08G5.7 |  |
|  |  |  |  |  |  |  |  |  |  |  |  |  |  |  |  |  |  |  |  |  |  |  |  |  |  |  |  |  |  |  |  |  |  |  |  |  |  | *clh-1* | CLC-type chloride cHannel |
|  |  |  |  |  |  |  |  |  |  |  |  |  |  |  |  |  |  |  |  |  |  |  |  |  |  |  |  |  |  |  |  |  |  |  |  |  |  | *aqp-7* | AQuaPorin or aquaglyceroporin related |
|  |  |  |  |  |  |  |  |  |  |  |  |  |  |  |  |  |  |  |  |  |  |  |  |  |  |  |  |  |  |  |  |  |  |  |  |  |  | T13C2.7 |  |
|  |  |  |  |  |  |  |  |  |  |  |  |  |  |  |  |  |  |  |  |  |  |  |  |  |  |  |  |  |  |  |  |  |  |  |  |  |  | *nlp-47* | Neuropeptide-Like Protein |
|  |  |  |  |  |  |  |  |  |  |  |  |  |  |  |  |  |  |  |  |  |  |  |  |  |  |  |  |  |  |  |  |  |  |  |  |  |  | F10E9.10 |  |
|  |  |  |  |  |  |  |  |  |  |  |  |  |  |  |  |  |  |  |  |  |  |  |  |  |  |  |  |  |  |  |  |  |  |  |  |  |  | T01G1.2 |  |
|  |  |  |  |  |  |  |  |  |  |  |  |  |  |  |  |  |  |  |  |  |  |  |  |  |  |  |  |  |  |  |  |  |  |  |  |  |  | *npr-31* | NeuroPeptide Receptor family |
|  |  |  |  |  |  |  |  |  |  |  |  |  |  |  |  |  |  |  |  |  |  |  |  |  |  |  |  |  |  |  |  |  |  |  |  |  |  | C39B10.1 |  |
|  |  |  |  |  |  |  |  |  |  |  |  |  |  |  |  |  |  |  |  |  |  |  |  |  |  |  |  |  |  |  |  |  |  |  |  |  |  | CD4.1 |  |
|  |  |  |  |  |  |  |  |  |  |  |  |  |  |  |  |  |  |  |  |  |  |  |  |  |  |  |  |  |  |  |  |  |  |  |  |  |  | *lpin-1* | LiPIN (mammalian lipodystrophy associated) homolog |
|  |  |  |  |  |  |  |  |  |  |  |  |  |  |  |  |  |  |  |  |  |  |  |  |  |  |  |  |  |  |  |  |  |  |  |  |  |  | F49E2.1 |  |
|  |  |  |  |  |  |  |  |  |  |  |  |  |  |  |  |  |  |  |  |  |  |  |  |  |  |  |  |  |  |  |  |  |  |  |  |  |  | T10B10.4 |  |
|  |  |  |  |  |  |  |  |  |  |  |  |  |  |  |  |  |  |  |  |  |  |  |  |  |  |  |  |  |  |  |  |  |  |  |  |  |  | *fbxa-24* | F-box A protein |
|  |  |  |  |  |  |  |  |  |  |  |  |  |  |  |  |  |  |  |  |  |  |  |  |  |  |  |  |  |  |  |  |  |  |  |  |  |  | *twk-40* | TWiK family of potassium channels |
|  |  |  |  |  |  |  |  |  |  |  |  |  |  |  |  |  |  |  |  |  |  |  |  |  |  |  |  |  |  |  |  |  |  |  |  |  |  | R13D11.4 |  |
|  |  |  |  |  |  |  |  |  |  |  |  |  |  |  |  |  |  |  |  |  |  |  |  |  |  |  |  |  |  |  |  |  |  |  |  |  |  | R13D11.3 |  |
|  |  |  |  |  |  |  |  |  |  |  |  |  |  |  |  |  |  |  |  |  |  |  |  |  |  |  |  |  |  |  |  |  |  |  |  |  |  | T23B7.3 |  |
|  |  |  |  |  |  |  |  |  |  |  |  |  |  |  |  |  |  |  |  |  |  |  |  |  |  |  |  |  |  |  |  |  |  |  |  |  |  | T24F1.4 |  |
|  |  |  |  |  |  |  |  |  |  |  |  |  |  |  |  |  |  |  |  |  |  |  |  |  |  |  |  |  |  |  |  |  |  |  |  |  |  | F40A3.4 |  |
|  |  |  |  |  |  |  |  |  |  |  |  |  |  |  |  |  |  |  |  |  |  |  |  |  |  |  |  |  |  |  |  |  |  |  |  |  |  | ZK856.14 |  |
|  |  |  |  |  |  |  |  |  |  |  |  |  |  |  |  |  |  |  |  |  |  |  |  |  |  |  |  |  |  |  |  |  |  |  |  |  |  | C05C9.2 |  |
|  |  |  |  |  |  |  |  |  |  |  |  |  |  |  |  |  |  |  |  |  |  |  |  |  |  |  |  |  |  |  |  |  |  |  |  |  |  | K06A4.2 |  |
|  |  |  |  |  |  |  |  |  |  |  |  |  |  |  |  |  |  |  |  |  |  |  |  |  |  |  |  |  |  |  |  |  |  |  |  |  |  | F46B6.9 |  |
|  |  |  |  |  |  |  |  |  |  |  |  |  |  |  |  |  |  |  |  |  |  |  |  |  |  |  |  |  |  |  |  |  |  |  |  |  |  | *ncs-1* | Neuronal Calcium Sensor family |
|  |  |  |  |  |  |  |  |  |  |  |  |  |  |  |  |  |  |  |  |  |  |  |  |  |  |  |  |  |  |  |  |  |  |  |  |  |  | *nlp-7* | Neuropeptide-Like Protein |
|  |  |  |  |  |  |  |  |  |  |  |  |  |  |  |  |  |  |  |  |  |  |  |  |  |  |  |  |  |  |  |  |  |  |  |  |  |  | *linc-25* | Long Intervening Non-Coding RNA |
|  |  |  |  |  |  |  |  |  |  |  |  |  |  |  |  |  |  |  |  |  |  |  |  |  |  |  |  |  |  |  |  |  |  |  |  |  |  | T24C4.3 |  |
|  |  |  |  |  |  |  |  |  |  |  |  |  |  |  |  |  |  |  |  |  |  |  |  |  |  |  |  |  |  |  |  |  |  |  |  |  |  | F22H10.6 |  |
|  |  |  |  |  |  |  |  |  |  |  |  |  |  |  |  |  |  |  |  |  |  |  |  |  |  |  |  |  |  |  |  |  |  |  |  |  |  | *nex-4* | anNEXin family |
|  |  |  |  |  |  |  |  |  |  |  |  |  |  |  |  |  |  |  |  |  |  |  |  |  |  |  |  |  |  |  |  |  |  |  |  |  |  | C38C5.1 |  |
|  |  |  |  |  |  |  |  |  |  |  |  |  |  |  |  |  |  |  |  |  |  |  |  |  |  |  |  |  |  |  |  |  |  |  |  |  |  | *linc-36* | Long Intervening Non-Coding RNA |
|  |  |  |  |  |  |  |  |  |  |  |  |  |  |  |  |  |  |  |  |  |  |  |  |  |  |  |  |  |  |  |  |  |  |  |  |  |  | Y106G6H.13 |  |
|  |  |  |  |  |  |  |  |  |  |  |  |  |  |  |  |  |  |  |  |  |  |  |  |  |  |  |  |  |  |  |  |  |  |  |  |  |  | F55A4.3 |  |
|  |  |  |  |  |  |  |  |  |  |  |  |  |  |  |  |  |  |  |  |  |  |  |  |  |  |  |  |  |  |  |  |  |  |  |  |  |  | F47G4.5 |  |
|  |  |  |  |  |  |  |  |  |  |  |  |  |  |  |  |  |  |  |  |  |  |  |  |  |  |  |  |  |  |  |  |  |  |  |  |  |  | *gst-40* | Glutathione S-Transferase |
|  |  |  |  |  |  |  |  |  |  |  |  |  |  |  |  |  |  |  |  |  |  |  |  |  |  |  |  |  |  |  |  |  |  |  |  |  |  | Y43F8C.11 |  |
|  |  |  |  |  |  |  |  |  |  |  |  |  |  |  |  |  |  |  |  |  |  |  |  |  |  |  |  |  |  |  |  |  |  |  |  |  |  | C06E2.1 |  |
|  |  |  |  |  |  |  |  |  |  |  |  |  |  |  |  |  |  |  |  |  |  |  |  |  |  |  |  |  |  |  |  |  |  |  |  |  |  | *mab-21* | Male ABnormal |
|  |  |  |  |  |  |  |  |  |  |  |  |  |  |  |  |  |  |  |  |  |  |  |  |  |  |  |  |  |  |  |  |  |  |  |  |  |  | R01H2.8 |  |
|  |  |  |  |  |  |  |  |  |  |  |  |  |  |  |  |  |  |  |  |  |  |  |  |  |  |  |  |  |  |  |  |  |  |  |  |  |  | *dylt-2* | DYnein Light chain (Tctex type) |
|  |  |  |  |  |  |  |  |  |  |  |  |  |  |  |  |  |  |  |  |  |  |  |  |  |  |  |  |  |  |  |  |  |  |  |  |  |  | *tps-2* | Trehalose 6-Phosphate Synthase |
|  |  |  |  |  |  |  |  |  |  |  |  |  |  |  |  |  |  |  |  |  |  |  |  |  |  |  |  |  |  |  |  |  |  |  |  |  |  | *arl-3* | ARF-Like |
|  |  |  |  |  |  |  |  |  |  |  |  |  |  |  |  |  |  |  |  |  |  |  |  |  |  |  |  |  |  |  |  |  |  |  |  |  |  | F10B5.9 |  |
|  |  |  |  |  |  |  |  |  |  |  |  |  |  |  |  |  |  |  |  |  |  |  |  |  |  |  |  |  |  |  |  |  |  |  |  |  |  | ZK418.3 |  |
|  |  |  |  |  |  |  |  |  |  |  |  |  |  |  |  |  |  |  |  |  |  |  |  |  |  |  |  |  |  |  |  |  |  |  |  |  |  | *xbx-4* | X-BoX promoter element regulated |
|  |  |  |  |  |  |  |  |  |  |  |  |  |  |  |  |  |  |  |  |  |  |  |  |  |  |  |  |  |  |  |  |  |  |  |  |  |  | Y17D7B.10 |  |
|  |  |  |  |  |  |  |  |  |  |  |  |  |  |  |  |  |  |  |  |  |  |  |  |  |  |  |  |  |  |  |  |  |  |  |  |  |  | *aqp-12* | AQuaPorin or aquaglyceroporin related |
|  |  |  |  |  |  |  |  |  |  |  |  |  |  |  |  |  |  |  |  |  |  |  |  |  |  |  |  |  |  |  |  |  |  |  |  |  |  | *mks-2* | MecKel-Gruber Syndrome (MKS) homolog |
|  |  |  |  |  |  |  |  |  |  |  |  |  |  |  |  |  |  |  |  |  |  |  |  |  |  |  |  |  |  |  |  |  |  |  |  |  |  | ZK185.2 |  |
|  |  |  |  |  |  |  |  |  |  |  |  |  |  |  |  |  |  |  |  |  |  |  |  |  |  |  |  |  |  |  |  |  |  |  |  |  |  | T24H10.4 |  |
|  |  |  |  |  |  |  |  |  |  |  |  |  |  |  |  |  |  |  |  |  |  |  |  |  |  |  |  |  |  |  |  |  |  |  |  |  |  | *jun-1* | JUN transcription factor homolog |
|  |  |  |  |  |  |  |  |  |  |  |  |  |  |  |  |  |  |  |  |  |  |  |  |  |  |  |  |  |  |  |  |  |  |  |  |  |  | C16D2.1 |  |
|  |  |  |  |  |  |  |  |  |  |  |  |  |  |  |  |  |  |  |  |  |  |  |  |  |  |  |  |  |  |  |  |  |  |  |  |  |  | *ncx-4* | Na/Ca eXchangers |
|  |  |  |  |  |  |  |  |  |  |  |  |  |  |  |  |  |  |  |  |  |  |  |  |  |  |  |  |  |  |  |  |  |  |  |  |  |  | ZK652.8 |  |
|  |  |  |  |  |  |  |  |  |  |  |  |  |  |  |  |  |  |  |  |  |  |  |  |  |  |  |  |  |  |  |  |  |  |  |  |  |  | *npr-32* | NeuroPeptide Receptor family |
|  |  |  |  |  |  |  |  |  |  |  |  |  |  |  |  |  |  |  |  |  |  |  |  |  |  |  |  |  |  |  |  |  |  |  |  |  |  | *lact-1* | beta-LACTamase domain containing |
|  |  |  |  |  |  |  |  |  |  |  |  |  |  |  |  |  |  |  |  |  |  |  |  |  |  |  |  |  |  |  |  |  |  |  |  |  |  | *srr-4* | Serpentine Receptor, class R |
|  |  |  |  |  |  |  |  |  |  |  |  |  |  |  |  |  |  |  |  |  |  |  |  |  |  |  |  |  |  |  |  |  |  |  |  |  |  | *ist-1* | Insulin receptor SubsTrate homolog |
|  |  |  |  |  |  |  |  |  |  |  |  |  |  |  |  |  |  |  |  |  |  |  |  |  |  |  |  |  |  |  |  |  |  |  |  |  |  | *lron-5* | eLRR (extracellular Leucine-Rich Repeat) ONly |
|  |  |  |  |  |  |  |  |  |  |  |  |  |  |  |  |  |  |  |  |  |  |  |  |  |  |  |  |  |  |  |  |  |  |  |  |  |  | *unc-58* | UNCoordinated |
|  |  |  |  |  |  |  |  |  |  |  |  |  |  |  |  |  |  |  |  |  |  |  |  |  |  |  |  |  |  |  |  |  |  |  |  |  |  | *dop-2* | DOPamine receptor |
|  |  |  |  |  |  |  |  |  |  |  |  |  |  |  |  |  |  |  |  |  |  |  |  |  |  |  |  |  |  |  |  |  |  |  |  |  |  | *twk-49* | TWiK family of potassium channels |
|  |  |  |  |  |  |  |  |  |  |  |  |  |  |  |  |  |  |  |  |  |  |  |  |  |  |  |  |  |  |  |  |  |  |  |  |  |  | *lgc-31* | Ligand-Gated ion Channel |
|  |  |  |  |  |  |  |  |  |  |  |  |  |  |  |  |  |  |  |  |  |  |  |  |  |  |  |  |  |  |  |  |  |  |  |  |  |  | F09G8.5 |  |
|  |  |  |  |  |  |  |  |  |  |  |  |  |  |  |  |  |  |  |  |  |  |  |  |  |  |  |  |  |  |  |  |  |  |  |  |  |  | M04B2.6 |  |
|  |  |  |  |  |  |  |  |  |  |  |  |  |  |  |  |  |  |  |  |  |  |  |  |  |  |  |  |  |  |  |  |  |  |  |  |  |  | R07B1.11 |  |
|  |  |  |  |  |  |  |  |  |  |  |  |  |  |  |  |  |  |  |  |  |  |  |  |  |  |  |  |  |  |  |  |  |  |  |  |  |  | *glb-22* | GLoBin related |
|  |  |  |  |  |  |  |  |  |  |  |  |  |  |  |  |  |  |  |  |  |  |  |  |  |  |  |  |  |  |  |  |  |  |  |  |  |  | *nlp-21* | Neuropeptide-Like Protein |
|  |  |  |  |  |  |  |  |  |  |  |  |  |  |  |  |  |  |  |  |  |  |  |  |  |  |  |  |  |  |  |  |  |  |  |  |  |  | *nlp-13* | Neuropeptide-Like Protein |
|  |  |  |  |  |  |  |  |  |  |  |  |  |  |  |  |  |  |  |  |  |  |  |  |  |  |  |  |  |  |  |  |  |  |  |  |  |  | *ins-17* | INSulin related |
|  |  |  |  |  |  |  |  |  |  |  |  |  |  |  |  |  |  |  |  |  |  |  |  |  |  |  |  |  |  |  |  |  |  |  |  |  |  | F17C11.2 |  |
|  |  |  |  |  |  |  |  |  |  |  |  |  |  |  |  |  |  |  |  |  |  |  |  |  |  |  |  |  |  |  |  |  |  |  |  |  |  | R02F2.8 |  |
|  |  |  |  |  |  |  |  |  |  |  |  |  |  |  |  |  |  |  |  |  |  |  |  |  |  |  |  |  |  |  |  |  |  |  |  |  |  | *octr-1* | OCTopamine Receptor (GPCR) |
|  |  |  |  |  |  |  |  |  |  |  |  |  |  |  |  |  |  |  |  |  |  |  |  |  |  |  |  |  |  |  |  |  |  |  |  |  |  | C46F11.6 |  |
|  |  |  |  |  |  |  |  |  |  |  |  |  |  |  |  |  |  |  |  |  |  |  |  |  |  |  |  |  |  |  |  |  |  |  |  |  |  | F20D6.11 |  |
|  |  |  |  |  |  |  |  |  |  |  |  |  |  |  |  |  |  |  |  |  |  |  |  |  |  |  |  |  |  |  |  |  |  |  |  |  |  | *phf-34* | PHd Finger family |
|  |  |  |  |  |  |  |  |  |  |  |  |  |  |  |  |  |  |  |  |  |  |  |  |  |  |  |  |  |  |  |  |  |  |  |  |  |  | C08G9.1 |  |
|  |  |  |  |  |  |  |  |  |  |  |  |  |  |  |  |  |  |  |  |  |  |  |  |  |  |  |  |  |  |  |  |  |  |  |  |  |  | R13H4.5 |  |

### Phenotypes enriched

|  |  |  |  |
| --- | --- | --- | --- |
| **Group name** | **Number in cluster** | **Enrichment** | **FDR corrected p** |
| chemosensory response variant | 15 | 5.01 | 0.000630 |
| chemosensory behavior variant | 15 | 4.97 | 0.000696 |
| aqueous chemosensory response variant | 12 | 6.03 | 0.001170 |
| odorant positive chemotaxis defective | 8 | 7.94 | 0.008240 |
| organism environmental stimulus response variant | 32 | 2.32 | 0.012200 |
| positive chemotaxis variant | 11 | 5.10 | 0.012900 |
| chemotaxis variant | 12 | 4.55 | 0.016200 |
| odorant positive chemotaxis variant | 8 | 7.10 | 0.017400 |
| chemical response variant | 24 | 2.66 | 0.017700 |
| positive chemotaxis defective | 10 | 5.28 | 0.021600 |
| AWC odorant chemotaxis variant | 6 | 9.27 | 0.038100 |

### Anatomy terms enriched

|  |  |  |  |
| --- | --- | --- | --- |
| **Group name** | **Number in cluster** | **Enrichment** | **FDR corrected p** |
| lateral pharyngeal ganglion right neuron | 35 | 4.45 | 1.61e-09 |
| lateral pharyngeal ganglion right | 35 | 4.42 | 1.84e-09 |
| lateral pharyngeal ganglion left neuron | 35 | 4.42 | 1.84e-09 |
| lateral pharyngeal ganglion left | 35 | 4.40 | 2.10e-09 |
| lateral ganglion | 35 | 4.16 | 9.45e-09 |
| head ganglion | 43 | 3.32 | 4.07e-08 |
| AWBR | 14 | 10.24 | 2.66e-07 |
| AWBL | 14 | 10.24 | 2.66e-07 |
| AWB | 14 | 10.24 | 2.66e-07 |
| sensory neuron | 38 | 3.32 | 4.18e-07 |
| ganglion | 74 | 2.16 | 6.59e-07 |
| AWCR | 14 | 8.11 | 5.22e-06 |
| AWCL | 14 | 8.11 | 5.22e-06 |
| AWC | 14 | 8.11 | 5.22e-06 |
| odorsensory neuron | 18 | 5.69 | 9.30e-06 |
| head neuron | 84 | 1.88 | 1.70e-05 |
| chemosensory neuron | 25 | 3.86 | 2.80e-05 |
| embryonic cell | 38 | 2.81 | 3.27e-05 |
| interneuron | 29 | 3.36 | 4.15e-05 |
| ciliated neuron | 38 | 2.73 | 6.37e-05 |
| amphid neuron | 33 | 2.95 | 8.99e-05 |
| neuron | 96 | 1.70 | 1.79e-04 |
| amphid right sensillum | 25 | 3.49 | 1.89e-04 |
| amphid left sensillum | 25 | 3.46 | 2.14e-04 |
| ventral ganglion | 19 | 4.33 | 2.57e-04 |
| AFDR | 10 | 9.07 | 3.07e-04 |
| AFDL | 10 | 8.87 | 3.76e-04 |
| AFD | 10 | 8.87 | 3.76e-04 |
| thermosensory neuron | 10 | 8.87 | 3.76e-04 |
| BAGR | 9 | 10.15 | 4.64e-04 |
| BAGL | 9 | 10.15 | 4.64e-04 |
| BAG | 9 | 10.15 | 4.64e-04 |
| tail neuron | 56 | 2.06 | 4.97e-04 |
| amphid sensillum | 33 | 2.71 | 5.95e-04 |
| ASER | 14 | 5.51 | 6.36e-04 |
| sensillum | 39 | 2.44 | 7.20e-04 |
| tail ganglion | 56 | 2.03 | 7.69e-04 |
| lumbar lateral left ganglion neuron | 20 | 3.81 | 8.60e-04 |
| ASEL | 14 | 5.31 | 9.53e-04 |
| somatic nervous system | 57 | 1.98 | 1.21e-03 |
| lumbar lateral right ganglion neuron | 20 | 3.71 | 1.29e-03 |
| nerve ring neuron | 23 | 3.32 | 1.30e-03 |
| head | 89 | 1.66 | 1.46e-03 |
| lumbar neuron | 20 | 3.67 | 1.46e-03 |
| ASE | 14 | 5.08 | 1.59e-03 |
| lumbar lateral left ganglion | 20 | 3.60 | 2.01e-03 |
| nervous system | 85 | 1.67 | 2.39e-03 |
| lumbar lateral ganglion | 20 | 3.52 | 2.76e-03 |
| lumbar lateral right ganglion | 20 | 3.52 | 2.76e-03 |
| somatic neuron | 42 | 2.12 | 7.08e-03 |
| lumbar ganglion | 20 | 3.28 | 7.21e-03 |
| ASIR | 12 | 5.01 | 9.86e-03 |
| ASIL | 12 | 5.01 | 9.86e-03 |
| ASI | 12 | 5.01 | 9.86e-03 |
| ASJR | 9 | 6.47 | 1.78e-02 |
| ASJL | 9 | 6.36 | 2.01e-02 |
| ASJ | 9 | 6.36 | 2.01e-02 |

### GO terms enriched

|  |  |  |
| --- | --- | --- |
| **GO term** | **Number of genes** | **FDR-corrected p-value** |
| signaling | 49 | 2.3e-07 |
| signal transduction | 44 | 8.6e-07 |
| G-protein coupled receptor signaling pathway | 21 | 6.9e-06 |
| molecular transducer activity | 33 | 1.2e-04 |
| passive transmembrane transporter activity | 19 | 3.0e-04 |
| oxygen transport | 7 | 3.5e-04 |
| oxygen binding | 7 | 1.2e-03 |
| potassium ion transmembrane transport | 8 | 4.2e-03 |
| olfactory behavior | 5 | 7.7e-03 |
| postsynaptic membrane | 8 | 1.0e-02 |
| ligand-gated channel activity | 10 | 1.2e-02 |
| iron ion binding | 8 | 1.4e-02 |
| substrate-specific transmembrane transporter activity | 21 | 1.4e-02 |
| single-organism behavior | 8 | 1.5e-02 |
| regulation of membrane potential | 4 | 1.5e-02 |
| response to external stimulus | 13 | 1.5e-02 |
| potassium ion transport | 8 | 1.6e-02 |
| neurotransmitter receptor activity | 5 | 1.8e-02 |
| voltage-gated ion channel activity | 6 | 1.9e-02 |
| cation transmembrane transporter activity | 15 | 3.5e-02 |
| transporter activity | 24 | 3.8e-02 |
| response to stimulus | 29 | 4.3e-02 |

### Expression clusters enriched

|  |  |  |  |
| --- | --- | --- | --- |
| **Group name** | **Number in cluster** | **Enrichment** | **FDR corrected p** |
| Larval Pan-neural Enriched Genes. | 151 | 4.08 | 2.20e-52 |
| Genes significantly enriched (> 2x, FDR < 5%) in a particular cell-type versus a reference sample of all cells at the same stage. WBPaper00037950:all-neurons\_larva\_enriched | 123 | 5.06 | 5.40e-51 |
| Genes significantly enriched (> 2x, FDR < 5%) in a particular cell-type versus a reference sample of all cells at the same stage. WBPaper00037950:BAG-neuron\_embryo\_enriched | 69 | 6.34 | 2.02e-32 |
| WT-Pico Pan-neural Enriched Genes, with genes found multiple times in a single dataset removed (without dups). | 125 | 3.25 | 2.36e-31 |
| Genes significantly enriched (> 2x, FDR < 5%) in a particular cell-type versus a reference sample of all cells at the same stage. WBPaper00037950:glr-1(+)-neurons\_larva\_enriched | 80 | 5.05 | 5.67e-31 |
| Genes that show selective expression in a subset of cell types vs broadly expressed in many cell types. Correspond to 20% - 57% of enriched\_genes for a given cell type. WBPaper00037950:all-neurons\_larva\_SelectivelyEnriched | 70 | 5.35 | 3.46e-28 |
| Genes enriched in neuronal miRNA-induced silencing complexes (miRISC) as detected by immunoprecipitations and microarray analysis. | 77 | 4.64 | 3.35e-27 |
| Embryonic Pan-neural Enriched Genes. | 110 | 2.83 | 8.80e-22 |
| Genes that show selective expression in a subset of cell types vs broadly expressed in many cell types. Correspond to 20% - 57% of enriched\_genes for a given cell type. WBPaper00037950:BAG-neuron\_embryo\_SelectivelyEnriched | 36 | 7.18 | 1.21e-17 |
| Genes that show selective expression in a subset of cell types vs broadly expressed in many cell types. Correspond to 20% - 57% of enriched\_genes for a given cell type. WBPaper00037950:glr-1(+)-neurons\_larva\_SelectivelyEnriched | 37 | 6.00 | 1.59e-15 |
| Genes significantly enriched (> 2x, FDR < 5%) in a particular cell-type versus a reference sample of all cells at the same stage. WBPaper00037950:AVE-neuron\_embryo\_enriched | 44 | 4.24 | 4.74e-13 |
| Genes significantly enriched (> 2x, FDR < 5%) in a particular cell-type versus a reference sample of all cells at the same stage. WBPaper00037950:A-class-motor-neurons\_larva\_enriched | 53 | 3.21 | 4.16e-11 |
| Genes predicted to be upregulated more than 2.0 fold in (AFD+AWB) datasets as compared to unsorted whole embryonic cells dataset. | 56 | 2.89 | 5.13e-10 |
| Genes significantly enriched (> 2x, FDR < 5%) in a particular cell-type versus a reference sample of all cells at the same stage. WBPaper00037950:dopaminergic-neurons\_embryo\_enriched | 40 | 3.58 | 2.01e-09 |
| Genes significantly enriched (> 2x, FDR < 5%) in a particular cell-type versus a reference sample of all cells at the same stage. WBPaper00037950:dopaminergic-neurons\_larva\_enriched | 67 | 2.27 | 1.02e-07 |
| Genes that show selective expression in a subset of cell types vs broadly expressed in many cell types. Correspond to 20% - 57% of enriched\_genes for a given cell type. WBPaper00037950:all-neurons\_embryo\_SelectivelyEnriched | 16 | 6.18 | 3.26e-06 |
| Genes that show selective expression in a subset of cell types vs broadly expressed in many cell types. Correspond to 20% - 57% of enriched\_genes for a given cell type. WBPaper00037950:AVE-neuron\_embryo\_SelectivelyEnriched | 18 | 5.29 | 4.58e-06 |
| Genes depleted in muscle cells (0hr muscle dataset). Dissociated myo-3::GFP embryos were cultured for 0 hours before FACS sorting. | 75 | 1.83 | 5.90e-05 |
| Genes significantly enriched (> 2x, FDR < 5%) in a particular cell-type versus a reference sample of all cells at the same stage. WBPaper00037950:all-neurons\_embryo\_enriched | 31 | 2.87 | 6.28e-05 |
| Genes significantly enriched (> 2x, FDR < 5%) in a particular cell-type versus a reference sample of all cells at both embryonic and larval stages. WBPaper00037950:all-neurons\_CoreEnriched | 14 | 5.72 | 6.33e-05 |
| Genes that show selective expression in a subset of cell types vs broadly expressed in many cell types. Correspond to 20% - 57% of enriched\_genes for a given cell type. WBPaper00037950:dopaminergic-neurons\_embryo\_SelectivelyEnriched | 17 | 4.12 | 3.32e-04 |
| Genes significantly enriched (> 2x, FDR < 5%) in a particular cell-type versus a reference sample of all cells at both embryonic and larval stages. WBPaper00037950:A-class-motor-neurons\_CoreEnriched | 13 | 5.12 | 5.62e-04 |
| Genes that show selective expression in a subset of cell types vs broadly expressed in many cell types. Correspond to 20% - 57% of enriched\_genes for a given cell type. WBPaper00037950:A-class-motor-neurons\_larva\_SelectivelyEnriched | 21 | 3.12 | 1.49e-03 |
| Genes significantly enriched (> 2x, FDR < 5%) in a particular cell-type versus a reference sample of all cells at both embryonic and larval stages. WBPaper00037950:dopaminergic-neurons\_CoreEnriched | 14 | 3.89 | 4.52e-03 |
| Larval A-class motor neuron enriched genes. | 25 | 2.58 | 4.56e-03 |
| Genes with increased expression after 24 hours of infection by P.lumniescens Fold changes shown are pathogen vs OP50. WBPaper00038438:P.lumniescens\_24hr\_upregulated\_TilingArray | 125 | 1.38 | 8.73e-03 |
| Genome-wide analysis of developmental and sex-regulated gene expression profile. cgc4489\_group\_17 | 25 | 2.46 | 9.42e-03 |
| Genes that showed expression levels higher than the corresponding reference sample (embryonic 0hr reference). WBPaper00037950:BAG-neuron\_expressed | 146 | 1.32 | 1.05e-02 |
| Genes that show selective expression in a subset of cell types vs broadly expressed in many cell types. Correspond to 20% - 57% of enriched\_genes for a given cell type. WBPaper00037950:dopaminergic-neurons\_larva\_SelectivelyEnriched | 26 | 2.36 | 1.28e-02 |
| Genes with expression level down regulated in mir-35 mutants comparing with N2. | 44 | 1.81 | 2.32e-02 |
| Candidate daf-19 down regulated genes with a statistically significant signal variation of 1.5-fold or greater. These were identified using a Significance Analysis of Microarrays (SAM). | 14 | 3.16 | 3.48e-02 |
| Genes with decreased expression after 12 hours of infection by D.coniospora Fold changes shown are pathogen vs OP50. | 81 | 1.48 | 3.51e-02 |
| Genes predicted to be upregulated more than 2.0 fold in AFD dataset as compared to AWB dataset. | 10 | 4.05 | 4.04e-02 |

### Motifs enriched

|  |  |  |  |  |  |
| --- | --- | --- | --- | --- | --- |
| **Motif** | **Logo** | **Possible orthologs** | **Number of motifs in cluster** | **Enrichment** | **FDR corrected p** |
| pTH10722 |  | ref-2 eor-1 egrh-3 | 212 | 1.76 | 1.3e-18 |
| NR2F6\_f1 |  | nhr-2 nhr-239 nhr-62 | 227 | 1.69 | 2.4e-18 |
| MA0079.3 |  | ZC328.2 klf-1 klf-2 | 180 | 1.93 | 3.5e-18 |
| CG5669\_SOLEXA\_5\_FBgn0039169 |  | klf-1 klf-2 | 137 | 2.20 | 6.2e-17 |
| Sp4\_1011 |  | klf-1 klf-2 sptf-3 | 217 | 1.68 | 9.8e-17 |
| PLAG1\_si |  | Y53H1A.2 klf-2 plp-2 | 192 | 1.76 | 9.3e-16 |
| MA0537.1 |  | blmp-1 | 208 | 1.69 | 9.3e-16 |
| EGR2\_si |  | ZC328.2 klf-1 klf-2 | 230 | 1.58 | 3.4e-15 |
| pTH9314 |  | fos-1 atf-6 crh-1 atf-7 C27D6.4 | 126 | 2.16 | 1.4e-14 |
| MA0543.1 |  | daf-8 eor-1 | 225 | 1.58 | 1.4e-14 |
| CG31670\_SANGER\_5\_FBgn0031375 |  | F21A9.2 CELE\_Y38H8A.5 | 253 | 1.47 | 1.1e-13 |
| Egr1\_2580 |  | ZC328.2 | 195 | 1.67 | 1.1e-13 |
| MA0474.1 |  | lin-1 C24A1.2 | 193 | 1.68 | 1.4e-13 |
| Otx1\_2325 |  | ceh-36 (0.74) ceh-53 dve-1 pha-2 alr-1 ceh-45 | 208 | 1.61 | 1.9e-13 |
| MA0186.1 |  | lim-7 lin-39 php-3 unc-86 cfi-1 | 185 | 1.70 | 2.8e-13 |
| Antp\_SOLEXA\_FBgn0000095 |  | ceh-23 (0.82) ceh-36 (0.74) ceh-31 (0.73) ceh-18 (0.69) ceh-30 (0.59) pal-1 (-0.59) lim-4 lim-7 lin-39 ceh-16 lim-6 ceh-53 ceh-10 ceh-43 pha-2 ceh-14 alr-1 egl-5 cog-1 mls-2 and 9 others  [full list] | 193 | 1.66 | 3.1e-13 |
| MA0472.1 |  | ZC328.2 daf-16 | 214 | 1.58 | 4.0e-13 |
| NR2E3\_f1 |  | nhr-100 (0.78) lin-39 lin-1 | 199 | 1.63 | 6.0e-13 |
| MA0547.1 |  | skn-1 ceh-2 | 200 | 1.62 | 6.1e-13 |
| RFX5\_2 |  | daf-19 (0.74) F52B5.7 | 100 | 2.33 | 9.5e-13 |
| MA0058.2 |  | irx-1 (-0.63) mxl-1 hlh-30 | 192 | 1.65 | 1.0e-12 |
| Hoxc5\_2630 |  | ceh-18 (0.69) lin-39 ceh-53 npax-3 ceh-43 alr-1 ceh-1 ceh-12 ceh-45 | 179 | 1.70 | 1.5e-12 |
| MA0467.1 |  | ceh-45 tbx-39 | 209 | 1.57 | 2.6e-12 |
| pTH3120 |  | K11D2.4 che-1 tbp-1 | 262 | 1.41 | 2.8e-12 |
| ETS2\_f1 |  | lin-1 C24A1.2 | 243 | 1.46 | 4.3e-12 |
| PROX1\_1 |  | crh-1 ceh-26 | 164 | 1.75 | 4.6e-12 |
| pTH10041 |  | ztf-29 | 187 | 1.64 | 4.7e-12 |
| ZN384\_f1 |  | K11D2.4 lin-29 | 257 | 1.42 | 5.1e-12 |
| pTH9385 |  | daf-19 (0.74) | 305 | 1.30 | 5.6e-12 |
| pTH6436 |  | ceh-53 | 187 | 1.64 | 6.2e-12 |
| HeLa-S3\_ZNF274\_UCD |  | C28G1.4 | 211 | 1.54 | 9.6e-12 |
| V$HOX13\_01 |  | lin-39 | 253 | 1.42 | 1.0e-11 |
| CG2052\_SOLEXA\_2.5\_FBgn0039905 |  | mel-28 fkh-7 lin-29 | 223 | 1.50 | 1.1e-11 |
| Plagl1\_0972 |  | Y53H1A.2 | 146 | 1.83 | 1.2e-11 |
| TBX2\_2 |  | tbx-38 mab-9 tbx-39 tbx-43 tbx-42 | 199 | 1.58 | 1.3e-11 |
| I$E74A\_01 |  | lin-1 nhr-19 C24A1.2 | 223 | 1.50 | 1.5e-11 |
| pTH9242 |  | mel-28 | 139 | 1.87 | 1.6e-11 |
| pTH9199 |  | daf-19 (0.74) mab-3 | 304 | 1.29 | 1.7e-11 |
| pTH9237 |  | mel-28 | 187 | 1.62 | 1.8e-11 |
| MA0143.3 |  | sox-4 (0.96) nhr-100 (0.78) pop-1 (-0.68) | 246 | 1.43 | 1.8e-11 |
| rn\_SOLEXA\_5\_FBgn0259172 |  | lin-29 | 209 | 1.54 | 1.9e-11 |
| pTH10015 |  | ces-1 (-0.57) ztf-28 che-1 | 213 | 1.53 | 2.0e-11 |
| MA0076.2 |  | lin-1 C24A1.2 | 270 | 1.37 | 2.0e-11 |
| pTH3751 |  | tbx-39 | 213 | 1.53 | 2.1e-11 |
| Elf4 |  | C24A1.2 | 260 | 1.39 | 2.4e-11 |
| exd\_FlyReg\_FBgn0000611 |  | ceh-20 let-381 cfi-1 | 251 | 1.41 | 3.0e-11 |
| pTH3819 |  | ceh-18 (0.69) | 179 | 1.64 | 3.2e-11 |
| Hoxb5\_3122 |  | lin-39 | 190 | 1.60 | 3.5e-11 |
| V$YY1\_01 |  | lsy-2 | 154 | 1.76 | 3.9e-11 |
| Hoxa2\_3079 |  | lin-39 | 221 | 1.49 | 4.4e-11 |
| pTH9108 |  | daf-12 nhr-5 | 146 | 1.79 | 6.1e-11 |
| PDX1\_do |  | ceh-18 (0.69) pal-1 (-0.59) lin-39 ceh-43 alr-1 ceh-12 | 208 | 1.53 | 6.4e-11 |
| Bsh\_Cell\_FBgn0000529 |  | ceh-9 (0.81) ceh-31 (0.73) ceh-8 (0.71) ceh-30 (0.59) ceh-24 (0.59) lim-7 lin-39 ceh-19 ceh-43 alr-1 cog-1 ceh-1 | 201 | 1.55 | 6.7e-11 |
| pTH5914 |  | attf-1 | 257 | 1.39 | 6.8e-11 |
| Foxg1\_1 |  | fkh-8 (0.64) fkh-10 fkh-7 pha-4 let-381 lin-31 daf-16 | 244 | 1.42 | 7.0e-11 |
| ESRRA\_3 |  | nhr-71 (0.57) nhr-2 nhr-213 nhr-10 nhr-6 nhr-68 Y67D8A.3 | 208 | 1.52 | 7.3e-11 |
| pTH9915 |  | crh-1 zip-3 | 187 | 1.59 | 1.4e-10 |
| Hoxc4\_3491 |  | lin-39 | 177 | 1.62 | 1.4e-10 |
| pTH10630 |  | lsy-27 | 180 | 1.61 | 1.5e-10 |
| BSX\_1 |  | ceh-9 (0.81) ceh-31 (0.73) lim-7 lin-39 ceh-16 ceh-43 alr-1 ceh-1 | 191 | 1.57 | 1.7e-10 |
| Sox1\_2631 |  | sox-4 (0.96) | 168 | 1.66 | 1.7e-10 |
| I$UBX\_01 |  | lin-39 | 175 | 1.63 | 1.8e-10 |
| MA0476.1 |  | jun-1 (0.83) fos-1 crh-1 sknr-1 F45H11.6 | 196 | 1.55 | 2.0e-10 |
| POU3F2\_1 |  | ceh-18 (0.69) unc-86 | 227 | 1.45 | 2.1e-10 |
| pTH3064 |  | crh-1 | 198 | 1.54 | 2.2e-10 |
| Evx1\_3952 |  | ceh-53 | 199 | 1.54 | 2.2e-10 |
| pTH10031 |  | mbr-1 (0.86) | 243 | 1.41 | 2.2e-10 |
| pTH9365 |  | ceh-18 (0.69) lin-39 ceh-6 | 166 | 1.66 | 2.3e-10 |
| pTH5159 |  | ref-1 mxl-1 hlh-26 hlh-30 mxl-2 lin-22 aha-1 C27D6.4 | 134 | 1.83 | 2.4e-10 |
| pTH10038 |  | sox-4 (0.96) gei-3 (0.91) F56D1.1 | 219 | 1.47 | 2.7e-10 |
| disco-r-Cl1\_SANGER\_5\_FBgn0042650 |  | nhr-68 lin-31 F55C5.11 | 232 | 1.44 | 2.8e-10 |
| Pou2f2\_3748 |  | ceh-18 (0.69) lim-7 alr-1 | 212 | 1.49 | 3.1e-10 |
| Prop1\_3949 |  | ceh-16 ceh-53 | 124 | 1.89 | 3.2e-10 |
| pTH5778 |  | egl-5 | 209 | 1.50 | 3.3e-10 |
| MA0264.1 |  | ceh-24 (0.59) ceh-22 dsc-1 | 215 | 1.48 | 3.7e-10 |
| SRP000217\_Sox2 |  | sox-4 (0.96) ceh-18 (0.69) ceh-6 tbp-1 | 170 | 1.63 | 4.5e-10 |
| MA0227.1 |  | lin-32 lin-39 ceh-32 | 159 | 1.68 | 4.6e-10 |
| pTH2283 |  | odd-2 | 245 | 1.40 | 5.2e-10 |
| Mv90 |  | mef-2 | 198 | 1.53 | 5.3e-10 |
| V$OCT1\_03 |  | ceh-18 (0.69) | 201 | 1.51 | 6.2e-10 |
| Hoxa5\_3415 |  | lin-39 | 190 | 1.55 | 6.6e-10 |
| PO3F2\_si |  | ceh-18 (0.69) dmd-3 | 165 | 1.64 | 6.7e-10 |
| MA0468.1 |  | ceh-14 alr-1 cfi-1 ZC204.2 | 163 | 1.65 | 6.8e-10 |
| MCR\_f1 |  | nhr-255 (0.55) | 192 | 1.54 | 7.0e-10 |
| Hoxa7\_3750 |  | lin-39 | 130 | 1.82 | 7.3e-10 |
| T-47D\_GATA3\_HudsonAlpha |  | elt-1 | 227 | 1.44 | 7.4e-10 |
| FOXG1\_2 |  | nhr-213 lin-31 | 212 | 1.48 | 7.6e-10 |
| pTH9250 |  | dmd-3 C34D1.1 | 263 | 1.35 | 7.9e-10 |
| pTH5072 |  | mdl-1 (0.63) hlh-30 pax-1 aha-1 | 162 | 1.65 | 8.5e-10 |
| PAX5\_si |  | D1081.8 pax-2 F45H11.6 | 180 | 1.58 | 8.5e-10 |
| MA0163.1 |  | Y53H1A.2 C09F5.3 D1081.8 | 172 | 1.61 | 8.8e-10 |
| pTH6486 |  | nhr-145 (0.67) | 215 | 1.47 | 9.2e-10 |
| cad\_FlyReg\_FBgn0000251 |  | ceh-24 (0.59) pal-1 (-0.59) ceh-13 lin-39 php-3 T27F2.4 D1005.3 | 262 | 1.35 | 9.4e-10 |
| MA0482.1 |  | elt-1 ztf-29 | 250 | 1.38 | 9.5e-10 |
| Hoxa4\_3426 |  | lin-39 | 170 | 1.61 | 1.0e-09 |
| EPAS1\_si |  | ceh-9 (0.81) hif-1 (0.59) daf-12 ztf-3 Y5F2A.4 | 190 | 1.54 | 1.1e-09 |
| IRX5\_1 |  | irx-1 (-0.63) | 229 | 1.42 | 1.2e-09 |
| Hoxb4\_2627 |  | lin-39 | 174 | 1.59 | 1.4e-09 |
| ONEC2\_si |  | ceh-48 dsc-1 | 251 | 1.37 | 1.4e-09 |
| K562\_ZBTB7A\_HudsonAlpha |  | ZC328.2 | 165 | 1.63 | 1.5e-09 |
| MA0452.2 |  | B0310.2 ZK177.3 | 240 | 1.39 | 1.5e-09 |
| Tbp\_pr781 |  | tbp-1 | 293 | 1.28 | 1.7e-09 |
| Lhx1\_2240 |  | lim-7 | 131 | 1.79 | 1.7e-09 |
| MA0033.1 |  | hlh-15 hlh-32 hlh-8 ngn-1 lin-31 | 254 | 1.36 | 1.8e-09 |
| H1-hESC\_SRF\_HudsonAlpha |  | hlh-10 (0.73) unc-120 | 320 | 1.23 | 1.8e-09 |
| Meox1\_2310 |  | ceh-31 (0.73) | 173 | 1.59 | 1.9e-09 |
| Hoxb3\_1720 |  | lin-39 | 191 | 1.53 | 1.9e-09 |
| MA0049.1 |  | lin-39 php-3 hbl-1 | 275 | 1.31 | 2.0e-09 |
| Hoxa3\_2783 |  | lin-39 | 186 | 1.54 | 2.0e-09 |
| pTH10714 |  | nhr-84 (0.71) nhr-216 nhr-142 | 193 | 1.52 | 2.3e-09 |
| pTH3998 |  | tbx-39 | 205 | 1.48 | 2.3e-09 |
| pTH10640 |  | dmd-4 dmd-5 ceh-32 | 181 | 1.56 | 2.4e-09 |
| pTH5877 |  | nhr-100 (0.78) elt-1 nhr-10 nhr-7 | 208 | 1.47 | 2.4e-09 |
| V$CREB\_Q4 |  | crh-1 W08E12.1 attf-1 | 180 | 1.56 | 2.5e-09 |
| pTH9326 |  | nhr-122 (-0.68) | 216 | 1.45 | 2.6e-09 |
| pTH9135 |  | pop-1 (-0.68) | 158 | 1.64 | 2.7e-09 |
| Pou3f4\_3773 |  | ceh-6 | 130 | 1.79 | 2.9e-09 |
| MSX2\_1 |  | ceh-31 (0.73) lin-39 ceh-43 ceh-14 ceh-1 | 256 | 1.35 | 3.2e-09 |
| pTH10026 |  | ces-1 (-0.57) ceh-20 ceh-32 F55C5.11 | 192 | 1.52 | 3.2e-09 |
| pTH9925 |  | nhr-100 (0.78) ztf-11 (-0.54) | 187 | 1.53 | 3.4e-09 |
| pTH5539 |  | unc-120 | 164 | 1.61 | 3.4e-09 |
| pTH9198 |  | dmd-3 | 200 | 1.49 | 3.4e-09 |
| pTH9125 |  | sox-4 (0.96) egl-13 (0.56) K11D2.4 | 255 | 1.35 | 3.5e-09 |
| pTH6445 |  | ceh-5 | 225 | 1.42 | 3.5e-09 |
| TCF4\_2 |  | hlh-2 (-0.66) ztf-6 | 193 | 1.51 | 3.6e-09 |
| pTH9182 |  | tbx-39 | 195 | 1.50 | 3.9e-09 |
| PTF1A\_f1 |  | hlh-2 (-0.66) lin-32 | 196 | 1.50 | 4.0e-09 |
| pTH8991 |  | cey-3 | 194 | 1.50 | 4.0e-09 |
| pTH9164 |  | ceh-26 | 191 | 1.51 | 4.3e-09 |
| pTH10030 |  | xbp-1 (0.76) C01B12.2 | 153 | 1.66 | 4.3e-09 |
| pTH9911 |  | fos-1 atf-5 crh-1 | 174 | 1.57 | 4.4e-09 |
| MAFA\_f1 |  | daf-8 pax-2 F45H11.6 | 209 | 1.46 | 4.8e-09 |
| Hoxa6\_1040 |  | lin-39 | 172 | 1.58 | 4.9e-09 |
| Vax1\_3499 |  | C02F12.10 | 162 | 1.61 | 5.0e-09 |
| SMAD3\_1 |  | sma-4 daf-8 hlh-8 | 268 | 1.32 | 5.5e-09 |
| Hoxc8\_3429 |  | lin-39 | 170 | 1.58 | 6.0e-09 |
| ELF3\_f1 |  | K02D7.2 unc-120 C24A1.2 | 236 | 1.39 | 6.5e-09 |
| pTH9137 |  | nhr-65 | 172 | 1.57 | 6.9e-09 |
| pTH2684 |  | fos-1 | 196 | 1.49 | 7.0e-09 |
| pTH6449 |  | ceh-43 | 183 | 1.53 | 7.2e-09 |
| Nkx1-2\_3214 |  | ceh-30 (0.59) | 174 | 1.56 | 7.5e-09 |
| Elf3 |  | C24A1.2 | 272 | 1.31 | 7.9e-09 |
| I$CROC\_01 |  | mel-28 mef-2 let-381 Y116A8C.22 Y61A9LA.9 | 232 | 1.39 | 8.8e-09 |
| HLH25 |  | lin-22 aha-1 hlh-27 hlh-28 | 152 | 1.64 | 9.4e-09 |
| pTH6425 |  | pop-1 (-0.68) ceh-20 | 213 | 1.44 | 9.4e-09 |
| Fli1 |  | F19F10.1 lin-1 C24A1.2 | 241 | 1.37 | 9.4e-09 |
| pTH5887 |  | lin-39 | 171 | 1.57 | 9.8e-09 |
| SOX2\_4 |  | sox-4 (0.96) dmd-4 grh-1 | 151 | 1.65 | 9.9e-09 |
| ZNF75A\_1 |  | F26F4.8 lag-1 ztf-3 | 245 | 1.36 | 1.0e-08 |
| Vax2\_3500 |  | C02F12.10 | 160 | 1.61 | 1.0e-08 |
| Nkx3-1\_2923 |  | ceh-24 (0.59) dsc-1 | 184 | 1.52 | 1.2e-08 |
| CG14962\_SANGER\_5\_FBgn0035407 |  | ceh-24 (0.59) ces-1 (-0.57) C34H4.5 T22H9.4 | 229 | 1.39 | 1.2e-08 |
| V$LYF1\_01 |  | mbr-1 (0.86) F26F4.8 nhr-177 bed-3 | 209 | 1.44 | 1.2e-08 |
| Gsh2\_3990 |  | ceh-31 (0.73) | 155 | 1.62 | 1.3e-08 |
| Ubx\_FlyReg\_FBgn0003944 |  | lin-39 ceh-10 alr-1 eyg-1 ceh-1 ceh-45 | 116 | 1.83 | 1.3e-08 |
| pTH6562 |  | ceh-5 | 121 | 1.80 | 1.4e-08 |
| pTH10816 |  | dmd-6 | 232 | 1.39 | 1.4e-08 |
| pTH10623 |  | scrt-1 | 198 | 1.47 | 1.4e-08 |
| Irx3\_0920 |  | irx-1 (-0.63) | 175 | 1.54 | 1.5e-08 |
| Elf5 |  | C24A1.2 | 275 | 1.29 | 1.5e-08 |
| V$OCT1\_06 |  | ceh-18 (0.69) ztf-9 (0.54) | 175 | 1.54 | 1.7e-08 |
| pTH9951 |  | pal-1 (-0.59) mex-6 | 251 | 1.34 | 1.7e-08 |
| pTH5812 |  | ceh-14 | 172 | 1.55 | 1.7e-08 |
| Emx2\_3420 |  | ceh-2 | 114 | 1.84 | 1.9e-08 |
| Jundm2\_0911 |  | fos-1 skn-1 | 122 | 1.78 | 1.9e-08 |
| V$FAC1\_01 |  | gei-8 | 183 | 1.51 | 2.0e-08 |
| V$ZIC1\_01 |  | ref-2 pax-3 ZC328.2 lin-1 ztf-14 | 160 | 1.59 | 2.0e-08 |
| pTH10796 |  | F10B5.3 (0.79) hsf-1 Y53C10A.3 | 239 | 1.36 | 2.2e-08 |
| MA0481.1 |  | fkh-8 (0.64) fkh-10 fkh-7 let-381 lin-31 daf-16 | 203 | 1.45 | 2.3e-08 |
| Ara\_Cell\_FBgn0015904 |  | irx-1 (-0.63) fkh-9 daf-16 | 301 | 1.24 | 2.3e-08 |
| Hoxd1\_3448 |  | ceh-12 | 174 | 1.54 | 2.3e-08 |
| RORA\_2 |  | nhr-213 nhr-118 | 175 | 1.53 | 2.5e-08 |
| Elf3\_3876 |  | C24A1.2 | 245 | 1.35 | 2.5e-08 |
| Etv3 |  | lin-1 | 241 | 1.36 | 2.6e-08 |
| Spdef |  | lin-1 | 190 | 1.49 | 2.6e-08 |
| pTH6508 |  | nhr-36 (0.93) | 221 | 1.40 | 2.6e-08 |
| Nkx1-1\_3856 |  | ceh-30 (0.59) | 170 | 1.55 | 2.7e-08 |
| pTH9245 |  | ceh-18 (0.69) | 193 | 1.48 | 2.7e-08 |
| Mw160 |  | nhr-68 | 237 | 1.37 | 2.9e-08 |
| pTH9323 |  | nhr-79 slr-2 odr-7 lin-1 nhr-273 nhr-28 tbx-39 | 218 | 1.41 | 3.4e-08 |
| pTH9247 |  | dmd-3 C34D1.1 | 229 | 1.38 | 3.4e-08 |
| MA0124.1 |  | ceh-24 (0.59) ceh-48 | 185 | 1.50 | 3.5e-08 |
| MA0095.2 |  | lsy-2 | 156 | 1.60 | 3.5e-08 |
| NR2F6\_2 |  | nhr-2 nhr-62 nhr-19 | 195 | 1.47 | 3.6e-08 |
| V$ZID\_01 |  | skn-1 ztf-28 ceh-32 | 206 | 1.44 | 3.7e-08 |
| ELK3\_f1 |  | lin-1 | 115 | 1.81 | 3.9e-08 |
| Irx2\_0900 |  | irx-1 (-0.63) | 177 | 1.52 | 3.9e-08 |
| pTH9884 |  | tbx-39 | 189 | 1.48 | 4.3e-08 |
| pTH6003 |  | nhr-134 nhr-182 | 200 | 1.45 | 4.4e-08 |
| Hlxb9\_3422 |  | ceh-12 | 179 | 1.51 | 5.3e-08 |
| pTH10768 |  | med-2 | 150 | 1.61 | 5.3e-08 |
| SH-SY5Y\_GATA2\_UCD |  | elt-1 | 211 | 1.42 | 5.6e-08 |
| pTH9256 |  | ceh-18 (0.69) | 137 | 1.67 | 5.8e-08 |
| pTH10805 |  | ztf-16 | 184 | 1.49 | 5.8e-08 |
| pTH9261 |  | dmd-3 pax-3 lin-48 | 192 | 1.46 | 7.0e-08 |
| pTH9282 |  | attf-1 C01B12.2 | 260 | 1.31 | 7.0e-08 |
| pTH5119 |  | cfi-1 | 221 | 1.39 | 7.2e-08 |
| Barx2\_3447 |  | ceh-43 | 163 | 1.55 | 7.7e-08 |
| HOXC10\_2 |  | ceh-24 (0.59) pal-1 (-0.59) lin-39 php-3 | 165 | 1.55 | 7.8e-08 |
| HXA1\_f1 |  | lin-39 ceh-20 ceh-12 | 208 | 1.42 | 8.0e-08 |
| En2\_0952 |  | ceh-24 (0.59) lim-7 ceh-16 lim-6 | 198 | 1.44 | 8.1e-08 |
| pTH9297 |  | ceh-18 (0.69) | 138 | 1.65 | 8.1e-08 |
| Tcf3\_3787 |  | pop-1 (-0.68) | 158 | 1.57 | 8.3e-08 |
| MA0038.1 |  | lim-6 odd-1 | 157 | 1.57 | 8.4e-08 |
| MA0014.2 |  | pax-3 pax-2 | 175 | 1.51 | 8.7e-08 |
| Nr2f2\_2192 |  | nhr-2 nhr-213 nhr-10 nhr-69 nhr-19 | 287 | 1.26 | 8.9e-08 |
| pTH5118 |  | cfi-1 | 219 | 1.39 | 9.0e-08 |
| tgo\_ss\_SANGER\_5\_FBgn0015014 |  | ahr-1 aha-1 | 175 | 1.51 | 9.1e-08 |
| Vsx1\_1728 |  | alr-1 | 121 | 1.74 | 9.1e-08 |
| V$CDXA\_01 |  | ceh-13 php-3 | 189 | 1.47 | 9.1e-08 |
| Pbx1\_3203 |  | ceh-20 | 217 | 1.39 | 9.8e-08 |
| MA0244.1 |  | C48E7.11 | 184 | 1.48 | 9.8e-08 |
| V$TCF11\_01 |  | skn-1 | 205 | 1.42 | 1.0e-07 |
| Lbx2\_3869 |  | mls-2 | 164 | 1.54 | 1.0e-07 |
| pTH10028 |  | nhr-204 | 198 | 1.44 | 1.1e-07 |
| Srf\_3509 |  | unc-120 | 148 | 1.60 | 1.1e-07 |
| pTH10650 |  | nhr-153 | 183 | 1.48 | 1.1e-07 |
| MA0066.1 |  | nhr-71 (0.57) nhr-6 nhr-43 | 166 | 1.53 | 1.1e-07 |
| pTH5437 |  | ceh-34 | 287 | 1.25 | 1.2e-07 |
| FOXB1\_3 |  | let-381 lin-31 | 219 | 1.39 | 1.2e-07 |
| Bsx\_3483 |  | ceh-31 (0.73) | 151 | 1.59 | 1.3e-07 |
| Six6\_2267 |  | elt-1 ceh-34 elt-3 ceh-32 elt-6 elt-7 egl-27 | 189 | 1.46 | 1.3e-07 |
| EN1\_2 |  | ceh-16 ceh-2 | 276 | 1.27 | 1.4e-07 |
| Hoxb8\_3780 |  | lin-39 | 187 | 1.46 | 1.4e-07 |
| pTH9969 |  | pag-3 | 151 | 1.58 | 1.5e-07 |
| pTH9254 |  | mel-28 | 189 | 1.46 | 1.6e-07 |
| pTH10027 |  | M03D4.4 | 186 | 1.46 | 1.6e-07 |
| V$CETS1P54\_02 |  | C52B9.2 | 226 | 1.37 | 1.7e-07 |
| HXD10\_f1 |  | php-3 nhr-2 | 234 | 1.35 | 1.8e-07 |
| pTH6478 |  | lim-7 | 152 | 1.58 | 1.8e-07 |
| Tcf1\_2666 |  | hmbx-1 | 298 | 1.23 | 1.9e-07 |
| Sox17\_2837 |  | sox-4 (0.96) | 226 | 1.36 | 2.0e-07 |
| pTH2936 |  | nhr-239 | 185 | 1.46 | 2.0e-07 |
| MA0173.1 |  | irx-1 (-0.63) hlh-32 D1081.8 | 260 | 1.30 | 2.0e-07 |
| Irx5\_2385 |  | irx-1 (-0.63) | 179 | 1.48 | 2.0e-07 |
| pTH9097 |  | Y116A8C.22 | 280 | 1.26 | 2.0e-07 |
| V$IK2\_01 |  | F26F4.8 | 189 | 1.45 | 2.4e-07 |
| pTH1292 |  | ceh-24 (0.59) pzf-1 | 212 | 1.39 | 2.4e-07 |
| pTH1001 |  | dnj-17 | 183 | 1.46 | 2.6e-07 |
| Nkx6-3\_3446 |  | cog-1 | 169 | 1.51 | 2.8e-07 |
| pTH10647 |  | nhr-232 | 195 | 1.43 | 2.8e-07 |
| Tcf7\_0950 |  | pop-1 (-0.68) | 235 | 1.34 | 2.9e-07 |
| CG4854\_SANGER\_10\_FBgn0038766 |  | mxl-1 K11D2.4 | 201 | 1.41 | 2.9e-07 |
| pTH9215 |  | C34D1.1 | 194 | 1.43 | 3.0e-07 |
| MA0046.1 |  | lin-39 hmbx-1 ceh-53 ceh-43 hmg-12 let-381 lin-31 Y116A8C.22 | 221 | 1.37 | 3.0e-07 |
| GRHL1\_2 |  | grh-1 | 286 | 1.25 | 3.0e-07 |
| pTH8330 |  | ztf-6 C34D1.1 gei-11 | 239 | 1.33 | 3.1e-07 |
| pTH7875 |  | mel-28 | 305 | 1.21 | 3.1e-07 |
| Hoxa7\_2668 |  | lin-39 | 155 | 1.55 | 3.2e-07 |
| FLI1\_f1 |  | lin-1 | 187 | 1.45 | 3.5e-07 |
| pTH5922 |  | ceh-24 (0.59) | 159 | 1.53 | 3.7e-07 |
| pTH9260 |  | mel-28 | 219 | 1.37 | 4.0e-07 |
| PBDE\_GATA1\_UCD |  | elt-1 alr-1 | 203 | 1.40 | 4.1e-07 |
| Hoxc11\_3718 |  | ceh-24 (0.59) pal-1 (-0.59) | 129 | 1.65 | 4.1e-07 |
| TLX1\_f1 |  | ceh-19 | 302 | 1.21 | 4.9e-07 |
| pTH6569 |  | ceh-43 | 184 | 1.45 | 5.0e-07 |
| pTH5919 |  | irx-1 (-0.63) | 156 | 1.54 | 5.2e-07 |
| pTH9708 |  | ceh-34 | 290 | 1.23 | 5.3e-07 |
| pTH9082 |  | mab-23 | 257 | 1.29 | 5.4e-07 |
| pTH6327 |  | dsc-1 | 200 | 1.41 | 5.9e-07 |
| KLF8\_f1 |  | ZC328.2 klf-1 | 164 | 1.51 | 6.0e-07 |
| Caup\_SOLEXA\_FBgn0015919 |  | irx-1 (-0.63) | 191 | 1.43 | 6.1e-07 |
| scrt\_SANGER\_2.5\_FBgn0004880 |  | hlh-2 (-0.66) ces-1 (-0.57) hlh-15 K02D7.2 hlh-1 hlh-8 | 220 | 1.36 | 6.1e-07 |
| pTH10718 |  | egl-43 | 315 | 1.19 | 6.4e-07 |
| V$GATA3\_01 |  | elt-1 | 305 | 1.21 | 6.9e-07 |
| Zbtb12\_2932 |  | lsy-27 ceh-90 | 211 | 1.38 | 7.2e-07 |
| MA0531.1 |  | F58G1.2 Y5F2A.4 | 183 | 1.44 | 7.9e-07 |
| HXA7\_f1 |  | lin-39 elt-1 | 195 | 1.41 | 8.0e-07 |
| pTH9246 |  | fkh-10 let-381 lin-31 C34D1.1 | 229 | 1.34 | 8.3e-07 |
| pTH9222 |  | mel-28 | 264 | 1.27 | 8.9e-07 |
| pTH9384 |  | cfi-1 | 223 | 1.35 | 9.0e-07 |
| Atf1\_3026 |  | crh-1 | 177 | 1.46 | 9.6e-07 |
| pTH10037 |  | T22C8.4 ref-2 | 271 | 1.26 | 1.0e-06 |
| Osr1\_3033 |  | odd-2 odd-1 | 284 | 1.24 | 1.1e-06 |
| CG8765\_SANGER\_5\_FBgn0036900 |  | H20J04.3 | 202 | 1.39 | 1.1e-06 |
| Spdef\_0905 |  | lin-1 | 180 | 1.44 | 1.1e-06 |
| V$BRN2\_01 |  | ceh-18 (0.69) | 171 | 1.47 | 1.1e-06 |
| pTH9335 |  | mel-28 | 164 | 1.49 | 1.2e-06 |
| Tcf1\_2666 |  | hmbx-1 | 293 | 1.22 | 1.3e-06 |
| pTH9244 |  | tbx-39 | 169 | 1.47 | 1.3e-06 |
| pTH5169 |  | cfi-1 | 152 | 1.53 | 1.3e-06 |
| pTH6268 |  | ceh-2 | 84 | 1.92 | 1.4e-06 |
| pTH10823 |  | B0310.2 | 185 | 1.43 | 1.4e-06 |
| pTH8399 |  | lin-54 | 172 | 1.46 | 1.4e-06 |
| pTH5423 |  | klf-2 | 177 | 1.45 | 1.5e-06 |
| pTH4325 |  | ceh-18 (0.69) | 141 | 1.57 | 1.5e-06 |
| pTH9974 |  | hlh-32 ngn-1 hlh-16 | 257 | 1.28 | 1.5e-06 |
| Hoxd10\_2368 |  | lin-39 php-3 | 136 | 1.58 | 1.6e-06 |
| pTH9381 |  | ceh-18 (0.69) | 131 | 1.60 | 1.9e-06 |
| Barhl1\_1 |  | ceh-31 (0.73) | 175 | 1.45 | 1.9e-06 |
| NR2F1\_3 |  | nhr-2 nhr-213 nhr-15 nhr-239 | 282 | 1.23 | 2.0e-06 |
| HES1\_f1 |  | lin-22 | 176 | 1.44 | 2.1e-06 |
| pTH9163 |  | nhr-3 (0.57) | 214 | 1.35 | 2.2e-06 |
| LHX6\_3 |  | gei-3 (0.91) lim-6 cfi-1 nhr-208 | 200 | 1.38 | 2.2e-06 |
| Hmx2\_3424 |  | ceh-9 (0.81) | 232 | 1.32 | 2.2e-06 |
| V$TBP\_01 |  | tbp-1 | 299 | 1.21 | 2.2e-06 |
| pTH9709 |  | die-1 | 136 | 1.58 | 2.2e-06 |
| pTH9216 |  | ceh-18 (0.69) | 180 | 1.43 | 2.3e-06 |
| MA0131.1 |  | F39B2.1 ZC416.1 | 296 | 1.21 | 2.7e-06 |
| V$AREB6\_02 |  | ztf-6 | 183 | 1.42 | 2.7e-06 |
| MA0118.1 |  | ref-2 | 149 | 1.52 | 2.9e-06 |
| pTH6447 |  | ceh-19 | 112 | 1.68 | 3.0e-06 |
| Nkx6-1\_2825 |  | cog-1 | 166 | 1.46 | 3.1e-06 |
| MA0027.1 |  | ceh-16 | 205 | 1.37 | 3.2e-06 |
| MYB\_f1 |  | D1081.8 B0310.2 | 225 | 1.33 | 3.3e-06 |
| pTH10777 |  | dmd-3 | 175 | 1.43 | 3.8e-06 |
| Mw140 |  | efl-1 F49E12.6 | 179 | 1.42 | 4.1e-06 |
| TBX2\_f1 |  | ztf-6 tbx-39 | 236 | 1.30 | 4.1e-06 |
| Pou3f1\_3819 |  | ceh-6 | 166 | 1.46 | 4.3e-06 |
| pTH9220 |  | mbr-1 (0.86) | 229 | 1.31 | 4.6e-06 |
| Poxm\_SOLEXA\_5\_FBgn0003129 |  | pax-2 | 283 | 1.22 | 4.7e-06 |
| Dlx3\_1030 |  | ceh-43 | 157 | 1.48 | 4.8e-06 |
| pTH6423 |  | pha-2 | 156 | 1.48 | 5.0e-06 |
| MA0032.1 |  | let-381 | 127 | 1.59 | 5.0e-06 |
| pTH6482 |  | ceh-19 | 144 | 1.52 | 5.6e-06 |
| Hlf\_1 |  | ces-2 (-0.64) atf-2 C01B12.2 C48E7.11 Y51H4A.4 F23F12.9 | 295 | 1.20 | 5.7e-06 |
| pTH5561 |  | nhr-239 | 205 | 1.36 | 5.7e-06 |
| MA0007.2 |  | npax-1 (0.6) nhr-255 (0.55) lin-14 | 200 | 1.37 | 6.3e-06 |
| Barx1\_2877 |  | ceh-43 | 171 | 1.43 | 6.4e-06 |
| pTH8649 |  | mbr-1 (0.86) | 196 | 1.37 | 7.0e-06 |
| pTH5924 |  | nhr-255 (0.55) | 326 | 1.16 | 7.0e-06 |
| pTH6071 |  | C46E10.8 C33G8.2 | 180 | 1.41 | 7.1e-06 |
| Pou2f1\_3081 |  | ceh-18 (0.69) | 163 | 1.45 | 7.1e-06 |
| pTH10788 |  | tbx-33 | 229 | 1.31 | 7.3e-06 |
| V$CEBP\_01 |  | C48E7.11 | 221 | 1.32 | 7.4e-06 |
| MA0262.1 |  | mab-3 hsf-1 | 200 | 1.36 | 7.6e-06 |
| V$FREAC7\_01 |  | lin-31 | 178 | 1.41 | 7.7e-06 |
| V$EN1\_01 |  | ceh-16 atf-2 | 192 | 1.38 | 8.1e-06 |
| Pou2f3\_3986 |  | ceh-18 (0.69) | 130 | 1.56 | 8.7e-06 |
| pTH5714 |  | nhr-239 | 187 | 1.39 | 8.8e-06 |
| GATA6\_f2 |  | end-3 elt-1 | 208 | 1.34 | 8.9e-06 |
| Smad3\_3805 |  | daf-8 | 191 | 1.38 | 9.1e-06 |
| Cutl1\_3494 |  | ceh-44 | 287 | 1.21 | 9.7e-06 |
| CUX1\_2 |  | ceh-48 | 226 | 1.31 | 1.0e-05 |
| pTH6641 |  | lin-31 | 290 | 1.20 | 1.1e-05 |
| V$FOXJ2\_02 |  | elt-1 lin-31 | 316 | 1.17 | 1.1e-05 |
| MA0502.1 |  | nfya-2 dro-1 ceh-20 cey-3 lin-31 | 202 | 1.35 | 1.1e-05 |
| twi\_da\_SANGER\_5\_FBgn0003900 |  | hlh-15 hlh-32 hlh-12 hlh-8 ngn-1 | 145 | 1.50 | 1.1e-05 |
| pTH10034 |  | nhr-66 | 218 | 1.32 | 1.2e-05 |
| V$S8\_01 |  | ceh-45 | 205 | 1.34 | 1.3e-05 |
| V$PAX2\_02 |  | pax-1 | 194 | 1.36 | 1.3e-05 |
| Mf28 |  | elt-1 | 204 | 1.34 | 1.3e-05 |
| MA0078.1 |  | sox-4 (0.96) gei-3 (0.91) pop-1 (-0.68) C05C9.3 | 193 | 1.37 | 1.4e-05 |
| Hoxd13\_2356 |  | pal-1 (-0.59) | 279 | 1.22 | 1.4e-05 |
| pTH9958 |  | ztf-6 ztf-2 | 215 | 1.32 | 1.6e-05 |
| pTH9353 |  | ceh-51 | 241 | 1.27 | 1.6e-05 |
| pTH5781 |  | ceh-32 | 172 | 1.41 | 1.6e-05 |
| Pou3f3\_3235 |  | ceh-6 | 207 | 1.33 | 1.7e-05 |
| pTH10633 |  | R07H5.10 C48E7.11 | 322 | 1.15 | 2.0e-05 |
| Hoxa11\_2218 |  | php-3 | 193 | 1.36 | 2.0e-05 |
| Dlx1\_1741 |  | ceh-43 | 157 | 1.45 | 2.0e-05 |
| Fer2\_da\_SANGER\_5\_FBgn0038402 |  | lin-32 hlh-15 hlh-14 hlh-1 hlh-11 | 193 | 1.36 | 2.2e-05 |
| pTH9149 |  | ztf-30 | 245 | 1.26 | 2.2e-05 |
| pTH6497 |  | lin-31 | 219 | 1.31 | 2.3e-05 |
| Hnf4a\_2640 |  | nhr-62 | 284 | 1.21 | 2.4e-05 |
| I$MTTFA\_01 |  | hmg-5 (-0.54) nsy-7 | 163 | 1.43 | 2.4e-05 |
| V$NCX\_01 |  | ceh-19 | 165 | 1.42 | 2.4e-05 |
| pTH9080 |  | mnm-2 | 178 | 1.39 | 2.5e-05 |
| Dlx2\_2273 |  | ceh-43 | 155 | 1.45 | 2.6e-05 |
| pTH8318 |  | attf-1 | 247 | 1.26 | 2.7e-05 |
| Hoxa10\_2318 |  | ceh-24 (0.59) | 174 | 1.39 | 2.9e-05 |
| pTH10013 |  | nhr-168 | 212 | 1.32 | 2.9e-05 |
| pTH8745 |  | attf-1 | 241 | 1.26 | 3.5e-05 |
| Mafk\_3106 |  | F45H11.6 | 207 | 1.32 | 3.7e-05 |
| Gata3\_1024 |  | elt-1 | 191 | 1.35 | 3.7e-05 |
| V$GR\_Q6 |  | nhr-255 (0.55) | 202 | 1.33 | 3.7e-05 |
| pTH8985 |  | athp-1 | 179 | 1.37 | 4.3e-05 |
| MA0161.1 |  | nfi-1 F49E12.6 | 239 | 1.26 | 4.4e-05 |
| V$HEN1\_01 |  | hlh-15 hlh-1 | 270 | 1.22 | 4.5e-05 |
| V$GATA1\_02 |  | elt-1 | 297 | 1.18 | 4.5e-05 |
| pTH9924 |  | nhr-46 | 205 | 1.32 | 4.6e-05 |
| ZBTB49\_1 |  | C46E10.9 aha-1 | 104 | 1.63 | 4.9e-05 |
| Gata5\_3768 |  | elt-1 | 193 | 1.34 | 5.2e-05 |
| pTH9043 |  | sem-2 | 218 | 1.30 | 5.3e-05 |
| pTH6106 |  | nhr-182 | 182 | 1.36 | 5.3e-05 |
| Tcfap2a\_2337 |  | F58G1.2 aptf-1 | 188 | 1.35 | 5.9e-05 |
| pTH8556 |  | pax-2 | 257 | 1.23 | 6.1e-05 |
| pTH3477 |  | daf-16 | 215 | 1.30 | 6.1e-05 |
| Hey\_SANGER\_5\_FBgn0027788 |  | lin-22 gei-11 | 169 | 1.39 | 6.3e-05 |
| pTH10808 |  | ztf-19 | 248 | 1.24 | 6.7e-05 |
| Cdx1\_2245 |  | ceh-13 | 245 | 1.25 | 7.1e-05 |
| pTH9907 |  | nhr-34 | 197 | 1.32 | 8.2e-05 |
| Sox15\_3457 |  | sox-4 (0.96) | 215 | 1.29 | 8.8e-05 |
| pTH4269 |  | F13H6.1 nhr-2 nhr-86 nhr-177 | 302 | 1.17 | 1.0e-04 |
| Six2\_2307 |  | ceh-34 ceh-32 | 258 | 1.22 | 1.0e-04 |
| pTH2846 |  | lin-31 | 148 | 1.43 | 1.0e-04 |
| pTH2280 |  | mnm-2 | 256 | 1.23 | 1.1e-04 |
| pTH6636 |  | egl-5 | 184 | 1.34 | 1.1e-04 |
| SMAD3\_f1 |  | daf-8 | 290 | 1.18 | 1.2e-04 |
| YY1\_1 |  | lsy-2 | 90 | 1.67 | 1.3e-04 |
| pTH5928 |  | ceh-34 | 141 | 1.44 | 1.4e-04 |
| pTH1014 |  | atf-5 | 281 | 1.19 | 1.5e-04 |
| pTH9026 |  | attf-1 | 235 | 1.25 | 1.7e-04 |
| pTH9085 |  | nhr-42 | 136 | 1.45 | 1.9e-04 |
| pTH9165 |  | ztf-27 | 175 | 1.35 | 1.9e-04 |
| pTH3041 |  | atf-2 | 292 | 1.17 | 2.0e-04 |
| pTH9387 |  | C34D1.1 | 273 | 1.20 | 2.0e-04 |
| Cdx2\_4272 |  | ceh-13 | 203 | 1.30 | 2.3e-04 |
| pTH8863 |  | hmg-12 | 198 | 1.30 | 2.4e-04 |
| Pknox2\_3077 |  | ceh-32 | 171 | 1.35 | 2.6e-04 |
| pTH6591 |  | lin-31 | 224 | 1.26 | 2.8e-04 |
| I$DFD\_01 |  | lin-39 | 309 | 1.15 | 3.2e-04 |
| pTH10772 |  | ceh-52 | 308 | 1.15 | 3.9e-04 |
| MA0540.1 |  | dpy-27 | 281 | 1.18 | 4.0e-04 |
| HIF1A\_si |  | hif-1 (0.59) | 264 | 1.20 | 4.2e-04 |
| Six1\_0935 |  | ceh-32 | 262 | 1.20 | 4.6e-04 |
| Gmeb1\_1745 |  | attf-1 | 245 | 1.22 | 4.8e-04 |
| pTH3997 |  | C04F5.9 | 264 | 1.20 | 4.8e-04 |
| pTH1049 |  | elt-1 | 318 | 1.13 | 5.1e-04 |
| CXXC1\_si |  | F52B11.1 F21D5.4 | 85 | 1.64 | 5.1e-04 |
| V$GATA1\_01 |  | elt-1 | 255 | 1.21 | 5.2e-04 |
| HepG2\_HSF1\_Stanford |  | Y53C10A.3 | 159 | 1.36 | 5.4e-04 |
| Hmbox1\_2674 |  | hmbx-1 | 156 | 1.37 | 6.1e-04 |
| pTH5690 |  | ceh-32 | 278 | 1.17 | 7.8e-04 |
| pTH9934 |  | Y53H1A.2 | 260 | 1.19 | 1.4e-03 |
| PAX8\_f1 |  | pax-2 | 96 | 1.53 | 1.5e-03 |
| pTH5257 |  | C48E7.11 | 154 | 1.34 | 1.6e-03 |
| pTH8983 |  | tag-347 | 226 | 1.22 | 1.8e-03 |
| pTH10769 |  | Y48G1C.6 | 186 | 1.28 | 1.9e-03 |
| V$GATA6\_01 |  | elt-1 | 186 | 1.28 | 2.0e-03 |
| pTH8216 |  | Y116A8C.22 | 165 | 1.31 | 2.6e-03 |
| Six4\_2860 |  | ceh-32 | 248 | 1.19 | 3.5e-03 |
| pTH3046 |  | Y116A8C.22 | 101 | 1.45 | 5.1e-03 |
| pTH7032 |  | F52B11.1 | 239 | 1.19 | 5.3e-03 |
| pTH5250 |  | C48E7.11 | 169 | 1.28 | 6.0e-03 |
| pTH5891 |  | nhr-49 | 152 | 1.30 | 7.5e-03 |
| pTH9096 |  | T07C12.11 | 242 | 1.18 | 8.2e-03 |
| Six3\_1732 |  | ceh-34 | 259 | 1.16 | 8.5e-03 |
| pTH9076 |  | C01G12.1 | 201 | 1.22 | 1.1e-02 |
| pTH8982 |  | ceh-48 | 332 | 1.09 | 1.4e-02 |
| pTH1294 |  | mel-28 | 73 | 1.52 | 1.6e-02 |
| V$MYB\_Q6 |  | D1081.8 | 284 | 1.12 | 2.7e-02 |
| pTH5078 |  | ces-2 (-0.64) | 151 | 1.26 | 2.9e-02 |
| pTH9880 |  | end-1 | 150 | 1.25 | 3.9e-02 |

### Correlated (and anti-correlated) transcription factors

|  |  |
| --- | --- |
| **Transcription factor** | **Correlation** |
| sox-4 | 0.96 |
| nhr-36 | 0.93 |
| dmd-10 | 0.92 |
| gei-3 | 0.91 |
| nhr-105 | 0.90 |
| nhr-198 | 0.90 |
| C06E2.1 | 0.90 |
| nhr-30 | 0.89 |
| nhr-197 | 0.89 |
| nhr-167 | 0.89 |
| nhr-26 | 0.89 |
| ceh-62 | 0.89 |
| saeg-1 | 0.88 |
| saeg-2 | 0.87 |
| nhr-158 | 0.87 |
| ceh-54 | 0.87 |
| mbr-1 | 0.86 |
| nhr-124 | 0.86 |
| nhr-196 | 0.85 |
| K12H6.12 | 0.83 |
| jun-1 | 0.83 |
| nhr-95 | 0.83 |
| ceh-23 | 0.82 |
| madf-4 | 0.82 |
| daf-3 | 0.82 |
| cnd-1 | -0.53 |
| flh-3 | -0.53 |
| attf-3 | -0.53 |
| ztf-11 | -0.54 |
| sup-35 | -0.54 |
| hmg-5 | -0.54 |
| vab-15 | -0.55 |
| lpd-2 | -0.55 |
| zip-7 | -0.56 |
| C16A3.4 | -0.56 |
| Y53F4B.3 | -0.57 |
| efl-3 | -0.57 |
| ces-1 | -0.57 |
| dnj-11 | -0.58 |
| ztf-13 | -0.59 |
| pal-1 | -0.59 |
| sex-1 | -0.60 |
| irx-1 | -0.63 |
| ces-2 | -0.64 |
| hlh-2 | -0.66 |
| F27D4.4 | -0.67 |
| pop-1 | -0.68 |
| nhr-13 | -0.68 |
| nhr-122 | -0.68 |
| duxl-1 | -0.74 |

### ChIP peaks enriched

|  |  |  |  |  |
| --- | --- | --- | --- | --- |
| **Gene** | **Experiment** | **Number of upstream peaks** | **Enrichment** | **FDR corrected p** |
| alr-1 | ALR-1\_Larvae-L2-stage | 72 | 2.29 | 2.8e-09 |
| ces-1 | CES-1\_Embryos | 80 | 2.09 | 1.4e-08 |
| ces-1 | CES-1\_Larvae-L4-stage | 39 | 2.57 | 5.7e-06 |
| zag-1 | ZAG-1\_Larvae-L2-stage | 56 | 2.12 | 5.8e-06 |
| ces-1 | CES-1\_Larvae-L3-stage | 43 | 2.39 | 9.3e-06 |
| ces-1 | CES-1\_Fed-L1-stage-larvae | 44 | 2.32 | 1.4e-05 |
| nhr-6 | NHR-6\_Larvae-L2-stage | 54 | 1.63 | 9.2e-03 |
| mml-1 | MML-1\_Larvae-L3-stage | 13 | 2.99 | 1.7e-02 |
| zag-1 | ZAG-1\_Fed-L1-stage-larvae | 22 | 2.11 | 3.0e-02 |
| F45C12.2 | F45C12.2\_Fed-L1-stage-larvae | 44 | 1.63 | 3.3e-02 |
