# Supplementary material for: Overlapping cell population expression profiling and regulatory inference in C. elegans
Source: BMC Genomics. 2016 Feb 29;17:159. doi: 10.1186/s12864-016-2482-z (PMC4772325; doi:10.1186/s12864-016-2482-z)
Supplement: Additional file 13: — Web supplement. (DOC 21 kb) [file 12864_2016_2482_MOESM13_ESM.zip › sortWeb/clusters/hier.300.clusters/246.html]

Cluster 246 

## Cluster 246

### Expression

| cnd-1 rep. 1 | cnd-1 rep. 2 | cnd-1 rep. 3 | pha-4 rep. 1 | pha-4 rep. 2 | pha-4 rep. 3 | ceh-27 | ceh-36 | ceh-6 | F21D5.9 | mir-57 | mls-2 | pal-1 | pros-1 | ttx-3 | unc-130 | hlh-16 | irx-1 | ceh-6 (+) hlh-16 (+) | ceh-6 (+) hlh-16 (-) | ceh-6 (-) hlh-16 (+) | cnd-1 singlets | pha-4 singlets | 0 | 60 | 120 | 150 | 180 | 240 | 330 | 390 | 420 | 480 | 540 | 570 | 600 | 630 | 660 | NAME | Functional description |
| --- | --- | --- | --- | --- | --- | --- | --- | --- | --- | --- | --- | --- | --- | --- | --- | --- | --- | --- | --- | --- | --- | --- | --- | --- | --- | --- | --- | --- | --- | --- | --- | --- | --- | --- | --- | --- | --- | --- | --- |
|  |  |  |  |  |  |  |  |  |  |  |  |  |  |  |  |  |  |  |  |  |  |  |  |  |  |  |  |  |  |  |  |  |  |  |  |  |  | F35C12.5 |  |
|  |  |  |  |  |  |  |  |  |  |  |  |  |  |  |  |  |  |  |  |  |  |  |  |  |  |  |  |  |  |  |  |  |  |  |  |  |  | *cyp-33C9* | CYtochrome P450 family |
|  |  |  |  |  |  |  |  |  |  |  |  |  |  |  |  |  |  |  |  |  |  |  |  |  |  |  |  |  |  |  |  |  |  |  |  |  |  | Y119D3B.t1 |  |
|  |  |  |  |  |  |  |  |  |  |  |  |  |  |  |  |  |  |  |  |  |  |  |  |  |  |  |  |  |  |  |  |  |  |  |  |  |  | F10E9.15 |  |
|  |  |  |  |  |  |  |  |  |  |  |  |  |  |  |  |  |  |  |  |  |  |  |  |  |  |  |  |  |  |  |  |  |  |  |  |  |  | *srg-62* | Serpentine Receptor, class G (gamma) |
|  |  |  |  |  |  |  |  |  |  |  |  |  |  |  |  |  |  |  |  |  |  |  |  |  |  |  |  |  |  |  |  |  |  |  |  |  |  | C15C8.6 |  |
|  |  |  |  |  |  |  |  |  |  |  |  |  |  |  |  |  |  |  |  |  |  |  |  |  |  |  |  |  |  |  |  |  |  |  |  |  |  | F40H6.5 |  |
|  |  |  |  |  |  |  |  |  |  |  |  |  |  |  |  |  |  |  |  |  |  |  |  |  |  |  |  |  |  |  |  |  |  |  |  |  |  | Y106G6D.4 |  |
|  |  |  |  |  |  |  |  |  |  |  |  |  |  |  |  |  |  |  |  |  |  |  |  |  |  |  |  |  |  |  |  |  |  |  |  |  |  | *sru-9* | Serpentine Receptor, class U |
|  |  |  |  |  |  |  |  |  |  |  |  |  |  |  |  |  |  |  |  |  |  |  |  |  |  |  |  |  |  |  |  |  |  |  |  |  |  | *col-80* | COLlagen |
|  |  |  |  |  |  |  |  |  |  |  |  |  |  |  |  |  |  |  |  |  |  |  |  |  |  |  |  |  |  |  |  |  |  |  |  |  |  | F46F11.12 |  |
|  |  |  |  |  |  |  |  |  |  |  |  |  |  |  |  |  |  |  |  |  |  |  |  |  |  |  |  |  |  |  |  |  |  |  |  |  |  | F16G10.1 |  |
|  |  |  |  |  |  |  |  |  |  |  |  |  |  |  |  |  |  |  |  |  |  |  |  |  |  |  |  |  |  |  |  |  |  |  |  |  |  | *srbc-2* | Serpentine Receptor, class BC (class B-like) |
|  |  |  |  |  |  |  |  |  |  |  |  |  |  |  |  |  |  |  |  |  |  |  |  |  |  |  |  |  |  |  |  |  |  |  |  |  |  | *srd-34* | Serpentine Receptor, class D (delta) |
|  |  |  |  |  |  |  |  |  |  |  |  |  |  |  |  |  |  |  |  |  |  |  |  |  |  |  |  |  |  |  |  |  |  |  |  |  |  | T03G11.16 |  |
|  |  |  |  |  |  |  |  |  |  |  |  |  |  |  |  |  |  |  |  |  |  |  |  |  |  |  |  |  |  |  |  |  |  |  |  |  |  | F32A6.10 |  |
|  |  |  |  |  |  |  |  |  |  |  |  |  |  |  |  |  |  |  |  |  |  |  |  |  |  |  |  |  |  |  |  |  |  |  |  |  |  | Y51H7C.3 |  |
|  |  |  |  |  |  |  |  |  |  |  |  |  |  |  |  |  |  |  |  |  |  |  |  |  |  |  |  |  |  |  |  |  |  |  |  |  |  | C25H3.1 |  |
|  |  |  |  |  |  |  |  |  |  |  |  |  |  |  |  |  |  |  |  |  |  |  |  |  |  |  |  |  |  |  |  |  |  |  |  |  |  | *nhr-48* | Nuclear Hormone Receptor family |
|  |  |  |  |  |  |  |  |  |  |  |  |  |  |  |  |  |  |  |  |  |  |  |  |  |  |  |  |  |  |  |  |  |  |  |  |  |  | *pgp-2* | P-GlycoProtein related |
|  |  |  |  |  |  |  |  |  |  |  |  |  |  |  |  |  |  |  |  |  |  |  |  |  |  |  |  |  |  |  |  |  |  |  |  |  |  | *hpo-38* | Hypersensitive to POre-forming toxin |
|  |  |  |  |  |  |  |  |  |  |  |  |  |  |  |  |  |  |  |  |  |  |  |  |  |  |  |  |  |  |  |  |  |  |  |  |  |  | Y110A2AM.4 |  |
|  |  |  |  |  |  |  |  |  |  |  |  |  |  |  |  |  |  |  |  |  |  |  |  |  |  |  |  |  |  |  |  |  |  |  |  |  |  | *klp-7* | Kinesin-Like Protein |
|  |  |  |  |  |  |  |  |  |  |  |  |  |  |  |  |  |  |  |  |  |  |  |  |  |  |  |  |  |  |  |  |  |  |  |  |  |  | *kcc-2* | K+/Cl- Cotransporter |
|  |  |  |  |  |  |  |  |  |  |  |  |  |  |  |  |  |  |  |  |  |  |  |  |  |  |  |  |  |  |  |  |  |  |  |  |  |  | *nkb-1* | Na+/K+ ATPase, Beta subunit |
|  |  |  |  |  |  |  |  |  |  |  |  |  |  |  |  |  |  |  |  |  |  |  |  |  |  |  |  |  |  |  |  |  |  |  |  |  |  | *gpb-1* | G Protein, Beta subunit |
|  |  |  |  |  |  |  |  |  |  |  |  |  |  |  |  |  |  |  |  |  |  |  |  |  |  |  |  |  |  |  |  |  |  |  |  |  |  | *mpk-1* | MAP Kinase |
|  |  |  |  |  |  |  |  |  |  |  |  |  |  |  |  |  |  |  |  |  |  |  |  |  |  |  |  |  |  |  |  |  |  |  |  |  |  | *tax-6* | abnormal CHEmotaxis |
|  |  |  |  |  |  |  |  |  |  |  |  |  |  |  |  |  |  |  |  |  |  |  |  |  |  |  |  |  |  |  |  |  |  |  |  |  |  | T28D6.5 |  |
|  |  |  |  |  |  |  |  |  |  |  |  |  |  |  |  |  |  |  |  |  |  |  |  |  |  |  |  |  |  |  |  |  |  |  |  |  |  | Y92H12A.2 |  |
|  |  |  |  |  |  |  |  |  |  |  |  |  |  |  |  |  |  |  |  |  |  |  |  |  |  |  |  |  |  |  |  |  |  |  |  |  |  | E04D5.1 |  |
|  |  |  |  |  |  |  |  |  |  |  |  |  |  |  |  |  |  |  |  |  |  |  |  |  |  |  |  |  |  |  |  |  |  |  |  |  |  | *egl-4* | EGg Laying defective |
|  |  |  |  |  |  |  |  |  |  |  |  |  |  |  |  |  |  |  |  |  |  |  |  |  |  |  |  |  |  |  |  |  |  |  |  |  |  | *hif-1* | HIF (Hypoxia Inducible Factor) homologa |
|  |  |  |  |  |  |  |  |  |  |  |  |  |  |  |  |  |  |  |  |  |  |  |  |  |  |  |  |  |  |  |  |  |  |  |  |  |  | F53F10.2 |  |
|  |  |  |  |  |  |  |  |  |  |  |  |  |  |  |  |  |  |  |  |  |  |  |  |  |  |  |  |  |  |  |  |  |  |  |  |  |  | *uba-1* | UBA (human ubiquitin) related |
|  |  |  |  |  |  |  |  |  |  |  |  |  |  |  |  |  |  |  |  |  |  |  |  |  |  |  |  |  |  |  |  |  |  |  |  |  |  | *kvs-4* | K (potassium) Voltage-Sensitive channel subunit |
|  |  |  |  |  |  |  |  |  |  |  |  |  |  |  |  |  |  |  |  |  |  |  |  |  |  |  |  |  |  |  |  |  |  |  |  |  |  | F53B7.4 |  |
|  |  |  |  |  |  |  |  |  |  |  |  |  |  |  |  |  |  |  |  |  |  |  |  |  |  |  |  |  |  |  |  |  |  |  |  |  |  | *nlp-5* | Neuropeptide-Like Protein |
|  |  |  |  |  |  |  |  |  |  |  |  |  |  |  |  |  |  |  |  |  |  |  |  |  |  |  |  |  |  |  |  |  |  |  |  |  |  | T14E8.2 |  |
|  |  |  |  |  |  |  |  |  |  |  |  |  |  |  |  |  |  |  |  |  |  |  |  |  |  |  |  |  |  |  |  |  |  |  |  |  |  | *lev-10* | LEVamisole resistant |
|  |  |  |  |  |  |  |  |  |  |  |  |  |  |  |  |  |  |  |  |  |  |  |  |  |  |  |  |  |  |  |  |  |  |  |  |  |  | *ptp-1* | Protein Tyrosine Phosphatase |
|  |  |  |  |  |  |  |  |  |  |  |  |  |  |  |  |  |  |  |  |  |  |  |  |  |  |  |  |  |  |  |  |  |  |  |  |  |  | *mvk-1* | MeValonate Kinase |
|  |  |  |  |  |  |  |  |  |  |  |  |  |  |  |  |  |  |  |  |  |  |  |  |  |  |  |  |  |  |  |  |  |  |  |  |  |  | T22D1.3 |  |
|  |  |  |  |  |  |  |  |  |  |  |  |  |  |  |  |  |  |  |  |  |  |  |  |  |  |  |  |  |  |  |  |  |  |  |  |  |  | *ugt-25* | UDP-GlucuronosylTransferase |
|  |  |  |  |  |  |  |  |  |  |  |  |  |  |  |  |  |  |  |  |  |  |  |  |  |  |  |  |  |  |  |  |  |  |  |  |  |  | Y34F4.5 |  |
|  |  |  |  |  |  |  |  |  |  |  |  |  |  |  |  |  |  |  |  |  |  |  |  |  |  |  |  |  |  |  |  |  |  |  |  |  |  | *fat-1* | FATty acid desaturase |
|  |  |  |  |  |  |  |  |  |  |  |  |  |  |  |  |  |  |  |  |  |  |  |  |  |  |  |  |  |  |  |  |  |  |  |  |  |  | *chp-1* | CHORD Protein |
|  |  |  |  |  |  |  |  |  |  |  |  |  |  |  |  |  |  |  |  |  |  |  |  |  |  |  |  |  |  |  |  |  |  |  |  |  |  | Y48G10A.1 |  |
|  |  |  |  |  |  |  |  |  |  |  |  |  |  |  |  |  |  |  |  |  |  |  |  |  |  |  |  |  |  |  |  |  |  |  |  |  |  | H03G16.1 |  |
|  |  |  |  |  |  |  |  |  |  |  |  |  |  |  |  |  |  |  |  |  |  |  |  |  |  |  |  |  |  |  |  |  |  |  |  |  |  | K02F6.5 |  |
|  |  |  |  |  |  |  |  |  |  |  |  |  |  |  |  |  |  |  |  |  |  |  |  |  |  |  |  |  |  |  |  |  |  |  |  |  |  | *cyp-34A7* | CYtochrome P450 family |
|  |  |  |  |  |  |  |  |  |  |  |  |  |  |  |  |  |  |  |  |  |  |  |  |  |  |  |  |  |  |  |  |  |  |  |  |  |  | *cct-2* | Chaperonin Containing TCP-1 |
|  |  |  |  |  |  |  |  |  |  |  |  |  |  |  |  |  |  |  |  |  |  |  |  |  |  |  |  |  |  |  |  |  |  |  |  |  |  | *hsp-1* | Heat Shock Protein |
|  |  |  |  |  |  |  |  |  |  |  |  |  |  |  |  |  |  |  |  |  |  |  |  |  |  |  |  |  |  |  |  |  |  |  |  |  |  | *cct-1* | Chaperonin Containing TCP-1 |
|  |  |  |  |  |  |  |  |  |  |  |  |  |  |  |  |  |  |  |  |  |  |  |  |  |  |  |  |  |  |  |  |  |  |  |  |  |  | *cct-6* | Chaperonin Containing TCP-1 |
|  |  |  |  |  |  |  |  |  |  |  |  |  |  |  |  |  |  |  |  |  |  |  |  |  |  |  |  |  |  |  |  |  |  |  |  |  |  | *mmcm-1* | MethylMalonylCoA Mutase homolog |
|  |  |  |  |  |  |  |  |  |  |  |  |  |  |  |  |  |  |  |  |  |  |  |  |  |  |  |  |  |  |  |  |  |  |  |  |  |  | *atfs-1* | Activating Transcription Factor associated with Stress |

### Phenotypes enriched

none found

### Anatomy terms enriched

none found

### GO terms enriched

|  |  |  |
| --- | --- | --- |
| **GO term** | **Number of genes** | **FDR-corrected p-value** |
| adenyl nucleotide binding | 10 | 0.015 |
| unfolded protein binding | 3 | 0.019 |

### Expression clusters enriched

|  |  |  |  |
| --- | --- | --- | --- |
| **Group name** | **Number in cluster** | **Enrichment** | **FDR corrected p** |
| Genes with expression level up in zfp-1 mutant background. | 8 | 6.65 | 0.00686 |

### Motifs enriched

|  |  |  |  |  |  |
| --- | --- | --- | --- | --- | --- |
| **Motif** | **Logo** | **Possible orthologs** | **Number of motifs in cluster** | **Enrichment** | **FDR corrected p** |
| pTH6556 |  | lim-6 | 9 | 6.16 | 0.0022 |
| SPDEF\_6 |  | nhr-100 (0.6) lin-1 | 40 | 1.64 | 0.0036 |
| sqz\_SANGER\_5\_FBgn0010768 |  | fkh-7 lin-29 | 45 | 1.51 | 0.0037 |
| MA0049.1 |  | hbl-1 (0.52) lin-39 | 50 | 1.39 | 0.0045 |
| pTH10779 |  | nhr-182 | 24 | 2.26 | 0.0048 |
| pTH9242 |  | mel-28 | 37 | 1.70 | 0.0052 |
| eve\_FlyReg\_FBgn0000606 |  | ceh-53 lin-31 | 41 | 1.57 | 0.0065 |
| pTH9884 |  | tbx-39 | 39 | 1.60 | 0.0086 |
| pTH5916 |  | efl-2 | 39 | 1.60 | 0.0088 |
| pTH9393 |  | ZC416.1 | 44 | 1.47 | 0.0100 |
| pTH9900 |  | C46E10.8 | 37 | 1.64 | 0.0110 |
| MA0493.1 |  | klf-1 klf-2 | 33 | 1.73 | 0.0130 |
| EN1\_2 |  | ceh-16 | 14 | 3.01 | 0.0140 |
| MA0456.1 |  | ref-2 | 13 | 3.19 | 0.0140 |
| pTH9260 |  | mel-28 | 48 | 1.37 | 0.0150 |
| pTH9137 |  | nhr-65 | 46 | 1.41 | 0.0160 |
| pTH9220 |  | mbr-1 (0.73) | 31 | 1.76 | 0.0170 |
| FOXJ3\_1 |  | daf-16 lin-31 fkh-7 | 48 | 1.36 | 0.0180 |
| pTH3043 |  | lin-31 | 48 | 1.36 | 0.0180 |
| pTH9214 |  | cfi-1 | 17 | 2.54 | 0.0180 |
| ZN384\_f1 |  | lin-29 | 45 | 1.42 | 0.0180 |
| MA0079.3 |  | klf-2 | 14 | 2.92 | 0.0180 |
| FOXJ3\_2 |  | lin-31 let-381 | 44 | 1.44 | 0.0190 |
| pTH0977 |  | sptf-3 klf-1 | 26 | 1.93 | 0.0190 |
| V$GATA3\_03 |  | elt-1 | 23 | 2.07 | 0.0200 |
| pnr\_SANGER\_5\_FBgn0003117 |  | elt-1 | 40 | 1.51 | 0.0210 |
| pTH10798 |  | Y75B8A.6 | 31 | 1.73 | 0.0220 |
| pTH9180 |  | mel-28 Y61A9LA.9 | 47 | 1.37 | 0.0230 |
| pTH6143 |  | pal-1 php-3 | 35 | 1.61 | 0.0240 |
| MA0135.1 |  | lim-7 | 8 | 4.57 | 0.0260 |
| pTH9189 |  | dmd-3 | 12 | 3.12 | 0.0270 |
| HXA7\_f1 |  | lin-39 | 29 | 1.76 | 0.0290 |
| V$XFD3\_01 |  | let-381 | 44 | 1.41 | 0.0300 |
| pTH8985 |  | athp-1 | 37 | 1.54 | 0.0320 |
| pTH10040 |  | slr-2 | 16 | 2.47 | 0.0320 |
| pTH9097 |  | Y116A8C.22 | 46 | 1.37 | 0.0330 |
| I$DFD\_01 |  | lin-39 | 29 | 1.75 | 0.0330 |
| pTH9149 |  | ztf-30 | 40 | 1.46 | 0.0380 |
| Gata6\_3769 |  | elt-1 | 25 | 1.85 | 0.0430 |
| ARI3A\_f1 |  | cfi-1 | 26 | 1.80 | 0.0450 |
| Abd-A\_FlyReg\_FBgn0000014 |  | lin-39 | 36 | 1.53 | 0.0450 |
| GATA1\_si |  | elt-1 | 22 | 1.96 | 0.0470 |
| MA0543.1 |  | eor-1 | 30 | 1.67 | 0.0480 |
| ZNF75A\_1 |  | ztf-3 | 36 | 1.52 | 0.0490 |
| MA0094.2 |  | lin-39 | 34 | 1.56 | 0.0500 |

### Correlated (and anti-correlated) transcription factors

|  |  |
| --- | --- |
| **Transcription factor** | **Correlation** |
| hif-1 | 0.90 |
| tag-68 | 0.79 |
| nhr-48 | 0.77 |
| ctbp-1 | 0.75 |
| nhr-36 | 0.75 |
| nhr-158 | 0.74 |
| egrh-1 | 0.73 |
| mbr-1 | 0.73 |
| nhr-4 | 0.73 |
| F10B5.3 | 0.73 |
| miz-1 | 0.72 |
| nhr-1 | 0.72 |
| madf-4 | 0.72 |
| dhhc-14 | 0.71 |
| ceh-36 | 0.71 |
| C17E4.6 | 0.71 |
| nhr-26 | 0.70 |
| egl-13 | 0.70 |
| ceh-62 | 0.70 |
| sem-4 | 0.70 |
| sox-4 | 0.70 |
| skn-1 | 0.70 |
| sta-1 | 0.70 |
| C33H5.17 | 0.69 |
| nhr-47 | 0.69 |
| nhr-210 | -0.34 |
| ceh-13 | -0.34 |
| ccch-3 | -0.34 |
| nhr-13 | -0.35 |
| ces-1 | -0.35 |
| nhr-168 | -0.35 |
| nhr-104 | -0.36 |
| nhr-207 | -0.36 |
| snu-23 | -0.40 |
| ceh-53 | -0.41 |
| atf-2 | -0.42 |
| C16A3.4 | -0.42 |
| lst-5 | -0.45 |
| ceh-7 | -0.46 |
| zip-6 | -0.46 |
| cebp-2 | -0.46 |
| grh-1 | -0.46 |
| mxl-2 | -0.46 |
| hlh-12 | -0.50 |
| hmg-11 | -0.51 |
| hlh-15 | -0.52 |
| C01F6.9 | -0.53 |
| Y56A3A.18 | -0.59 |
| mxl-1 | -0.62 |
| nhr-122 | -0.69 |

### ChIP peaks enriched

|  |  |  |  |  |
| --- | --- | --- | --- | --- |
| **Gene** | **Experiment** | **Number of upstream peaks** | **Enrichment** | **FDR corrected p** |
| ztf-4 | ZTF-4\_Larvae-L2-stage | 14 | 3.83 | 0.00055 |
| lin-13 | LIN-13\_Larvae-L4-stage | 17 | 2.82 | 0.00250 |
| nhr-76 | NHR-76\_Larvae-L4-stage | 15 | 2.90 | 0.00520 |
| nhr-10 | NHR-10\_Larvae-L4-stage | 10 | 4.14 | 0.00530 |
| C34F6.9 | C34F6.9\_Larvae-L2-stage | 23 | 2.06 | 0.00970 |
| gei-11 | GEI-11\_Larvae-L2-stage | 20 | 2.14 | 0.01700 |
| alr-1 | ALR-1\_Larvae-L2-stage | 23 | 1.97 | 0.01700 |
| ces-1 | CES-1\_Fed-L1-stage-larvae | 14 | 2.67 | 0.01800 |
| sax-3 | SAX-3\_Fed-L1-stage-larvae | 12 | 2.93 | 0.02200 |
| F23B12.7 | F23B12.7\_Young-adult | 16 | 2.37 | 0.02400 |
| fos-1 | FOS-1\_Larvae-L3-stage | 15 | 2.45 | 0.02700 |
| W03F9.2 | W03F9.2\_L4-Young-Adult-stage-larvae | 23 | 1.90 | 0.02800 |
| nhr-77 | NHR-77\_Larvae-L4-stage | 31 | 1.63 | 0.02900 |
| fos-1 | FOS-1\_Larvae-L4-stage | 10 | 3.21 | 0.03200 |
| lsy-2 | LSY-2\_Larvae-L4-stage | 12 | 2.77 | 0.03400 |
| F45C12.2 | F45C12.2\_Fed-L1-stage-larvae | 21 | 1.93 | 0.04100 |
| ceh-38 | CEH-38\_Larvae-L3-stage | 19 | 2.01 | 0.04600 |
| ceh-38 | CEH-38\_Larvae-L4-stage | 11 | 2.80 | 0.04900 |
