# Supplementary material for: Overlapping cell population expression profiling and regulatory inference in C. elegans
Source: BMC Genomics. 2016 Feb 29;17:159. doi: 10.1186/s12864-016-2482-z (PMC4772325; doi:10.1186/s12864-016-2482-z)
Supplement: Additional file 13: — Web supplement. (DOC 21 kb) [file 12864_2016_2482_MOESM13_ESM.zip › sortWeb/clusters/hier.300.clusters/250.html]

Cluster 250 

## Cluster 250

### Expression

| cnd-1 rep. 1 | cnd-1 rep. 2 | cnd-1 rep. 3 | pha-4 rep. 1 | pha-4 rep. 2 | pha-4 rep. 3 | ceh-27 | ceh-36 | ceh-6 | F21D5.9 | mir-57 | mls-2 | pal-1 | pros-1 | ttx-3 | unc-130 | hlh-16 | irx-1 | ceh-6 (+) hlh-16 (+) | ceh-6 (+) hlh-16 (-) | ceh-6 (-) hlh-16 (+) | cnd-1 singlets | pha-4 singlets | 0 | 60 | 120 | 150 | 180 | 240 | 330 | 390 | 420 | 480 | 540 | 570 | 600 | 630 | 660 | NAME | Functional description |
| --- | --- | --- | --- | --- | --- | --- | --- | --- | --- | --- | --- | --- | --- | --- | --- | --- | --- | --- | --- | --- | --- | --- | --- | --- | --- | --- | --- | --- | --- | --- | --- | --- | --- | --- | --- | --- | --- | --- | --- |
|  |  |  |  |  |  |  |  |  |  |  |  |  |  |  |  |  |  |  |  |  |  |  |  |  |  |  |  |  |  |  |  |  |  |  |  |  |  | F16C3.4 |  |
|  |  |  |  |  |  |  |  |  |  |  |  |  |  |  |  |  |  |  |  |  |  |  |  |  |  |  |  |  |  |  |  |  |  |  |  |  |  | T05H10.8 |  |
|  |  |  |  |  |  |  |  |  |  |  |  |  |  |  |  |  |  |  |  |  |  |  |  |  |  |  |  |  |  |  |  |  |  |  |  |  |  | *nape-2* | N-Acyl Phosphatidyl Ethanolamine specific phospholipase D (NAPE-PLD) homolog |
|  |  |  |  |  |  |  |  |  |  |  |  |  |  |  |  |  |  |  |  |  |  |  |  |  |  |  |  |  |  |  |  |  |  |  |  |  |  | F58A6.2 |  |
|  |  |  |  |  |  |  |  |  |  |  |  |  |  |  |  |  |  |  |  |  |  |  |  |  |  |  |  |  |  |  |  |  |  |  |  |  |  | *snet-1* | snet (Suppressor of NEp Two) |
|  |  |  |  |  |  |  |  |  |  |  |  |  |  |  |  |  |  |  |  |  |  |  |  |  |  |  |  |  |  |  |  |  |  |  |  |  |  | *ceh-8* | C. Elegans Homeobox |
|  |  |  |  |  |  |  |  |  |  |  |  |  |  |  |  |  |  |  |  |  |  |  |  |  |  |  |  |  |  |  |  |  |  |  |  |  |  | T01B7.9 |  |
|  |  |  |  |  |  |  |  |  |  |  |  |  |  |  |  |  |  |  |  |  |  |  |  |  |  |  |  |  |  |  |  |  |  |  |  |  |  | *daf-28* | abnormal DAuer Formation |
|  |  |  |  |  |  |  |  |  |  |  |  |  |  |  |  |  |  |  |  |  |  |  |  |  |  |  |  |  |  |  |  |  |  |  |  |  |  | *flp-34* | FMRF-Like Peptide |
|  |  |  |  |  |  |  |  |  |  |  |  |  |  |  |  |  |  |  |  |  |  |  |  |  |  |  |  |  |  |  |  |  |  |  |  |  |  | M03F8.6 |  |
|  |  |  |  |  |  |  |  |  |  |  |  |  |  |  |  |  |  |  |  |  |  |  |  |  |  |  |  |  |  |  |  |  |  |  |  |  |  | *twk-14* | TWiK family of potassium channels |
|  |  |  |  |  |  |  |  |  |  |  |  |  |  |  |  |  |  |  |  |  |  |  |  |  |  |  |  |  |  |  |  |  |  |  |  |  |  | *ram-5* | abnormal RAy Morphology |
|  |  |  |  |  |  |  |  |  |  |  |  |  |  |  |  |  |  |  |  |  |  |  |  |  |  |  |  |  |  |  |  |  |  |  |  |  |  | T05C3.2 |  |
|  |  |  |  |  |  |  |  |  |  |  |  |  |  |  |  |  |  |  |  |  |  |  |  |  |  |  |  |  |  |  |  |  |  |  |  |  |  | F07A11.1 |  |
|  |  |  |  |  |  |  |  |  |  |  |  |  |  |  |  |  |  |  |  |  |  |  |  |  |  |  |  |  |  |  |  |  |  |  |  |  |  | *ocr-4* | Osm-9 and Capsaicin receptor-Related |
|  |  |  |  |  |  |  |  |  |  |  |  |  |  |  |  |  |  |  |  |  |  |  |  |  |  |  |  |  |  |  |  |  |  |  |  |  |  | *acr-20* | AcetylCholine Receptor |
|  |  |  |  |  |  |  |  |  |  |  |  |  |  |  |  |  |  |  |  |  |  |  |  |  |  |  |  |  |  |  |  |  |  |  |  |  |  | *acr-23* | AcetylCholine Receptor |
|  |  |  |  |  |  |  |  |  |  |  |  |  |  |  |  |  |  |  |  |  |  |  |  |  |  |  |  |  |  |  |  |  |  |  |  |  |  | R05G6.5 |  |
|  |  |  |  |  |  |  |  |  |  |  |  |  |  |  |  |  |  |  |  |  |  |  |  |  |  |  |  |  |  |  |  |  |  |  |  |  |  | *dyla-1* | DYnein Light chain, Axonemal p28 type |
|  |  |  |  |  |  |  |  |  |  |  |  |  |  |  |  |  |  |  |  |  |  |  |  |  |  |  |  |  |  |  |  |  |  |  |  |  |  | *arrd-16* | ARRestin Domain protein |
|  |  |  |  |  |  |  |  |  |  |  |  |  |  |  |  |  |  |  |  |  |  |  |  |  |  |  |  |  |  |  |  |  |  |  |  |  |  | *pnc-2* | Pyrazinamidase and NiCotinamidase |
|  |  |  |  |  |  |  |  |  |  |  |  |  |  |  |  |  |  |  |  |  |  |  |  |  |  |  |  |  |  |  |  |  |  |  |  |  |  | E04F6.2 |  |
|  |  |  |  |  |  |  |  |  |  |  |  |  |  |  |  |  |  |  |  |  |  |  |  |  |  |  |  |  |  |  |  |  |  |  |  |  |  | T07C12.15 |  |
|  |  |  |  |  |  |  |  |  |  |  |  |  |  |  |  |  |  |  |  |  |  |  |  |  |  |  |  |  |  |  |  |  |  |  |  |  |  | *flp-3* | FMRF-Like Peptide |
|  |  |  |  |  |  |  |  |  |  |  |  |  |  |  |  |  |  |  |  |  |  |  |  |  |  |  |  |  |  |  |  |  |  |  |  |  |  | W05B2.7 |  |
|  |  |  |  |  |  |  |  |  |  |  |  |  |  |  |  |  |  |  |  |  |  |  |  |  |  |  |  |  |  |  |  |  |  |  |  |  |  | R02E12.4 |  |
|  |  |  |  |  |  |  |  |  |  |  |  |  |  |  |  |  |  |  |  |  |  |  |  |  |  |  |  |  |  |  |  |  |  |  |  |  |  | *ets-5* | ETS class transcription factor |
|  |  |  |  |  |  |  |  |  |  |  |  |  |  |  |  |  |  |  |  |  |  |  |  |  |  |  |  |  |  |  |  |  |  |  |  |  |  | F49E10.4 |  |
|  |  |  |  |  |  |  |  |  |  |  |  |  |  |  |  |  |  |  |  |  |  |  |  |  |  |  |  |  |  |  |  |  |  |  |  |  |  | *srz-24* | Serpentine Receptor, class Z |
|  |  |  |  |  |  |  |  |  |  |  |  |  |  |  |  |  |  |  |  |  |  |  |  |  |  |  |  |  |  |  |  |  |  |  |  |  |  | *sre-41* | Serpentine Receptor, class E (epsilon) |
|  |  |  |  |  |  |  |  |  |  |  |  |  |  |  |  |  |  |  |  |  |  |  |  |  |  |  |  |  |  |  |  |  |  |  |  |  |  | *srt-70* | Serpentine Receptor, class T |
|  |  |  |  |  |  |  |  |  |  |  |  |  |  |  |  |  |  |  |  |  |  |  |  |  |  |  |  |  |  |  |  |  |  |  |  |  |  | Y55H10A.2 |  |
|  |  |  |  |  |  |  |  |  |  |  |  |  |  |  |  |  |  |  |  |  |  |  |  |  |  |  |  |  |  |  |  |  |  |  |  |  |  | *ncs-5* | Neuronal Calcium Sensor family |
|  |  |  |  |  |  |  |  |  |  |  |  |  |  |  |  |  |  |  |  |  |  |  |  |  |  |  |  |  |  |  |  |  |  |  |  |  |  | *srh-119* | Serpentine Receptor, class H |
|  |  |  |  |  |  |  |  |  |  |  |  |  |  |  |  |  |  |  |  |  |  |  |  |  |  |  |  |  |  |  |  |  |  |  |  |  |  | *xbx-9* | X-BoX promoter element regulated |
|  |  |  |  |  |  |  |  |  |  |  |  |  |  |  |  |  |  |  |  |  |  |  |  |  |  |  |  |  |  |  |  |  |  |  |  |  |  | K07C5.9 |  |
|  |  |  |  |  |  |  |  |  |  |  |  |  |  |  |  |  |  |  |  |  |  |  |  |  |  |  |  |  |  |  |  |  |  |  |  |  |  | F15A4.5 |  |
|  |  |  |  |  |  |  |  |  |  |  |  |  |  |  |  |  |  |  |  |  |  |  |  |  |  |  |  |  |  |  |  |  |  |  |  |  |  | K04D7.6 |  |
|  |  |  |  |  |  |  |  |  |  |  |  |  |  |  |  |  |  |  |  |  |  |  |  |  |  |  |  |  |  |  |  |  |  |  |  |  |  | *nhr-188* | Nuclear Hormone Receptor family |
|  |  |  |  |  |  |  |  |  |  |  |  |  |  |  |  |  |  |  |  |  |  |  |  |  |  |  |  |  |  |  |  |  |  |  |  |  |  | C16D9.9 |  |
|  |  |  |  |  |  |  |  |  |  |  |  |  |  |  |  |  |  |  |  |  |  |  |  |  |  |  |  |  |  |  |  |  |  |  |  |  |  | *hlh-4* | Helix Loop Helix |
|  |  |  |  |  |  |  |  |  |  |  |  |  |  |  |  |  |  |  |  |  |  |  |  |  |  |  |  |  |  |  |  |  |  |  |  |  |  | *snt-6* | SyNapTotagmin |
|  |  |  |  |  |  |  |  |  |  |  |  |  |  |  |  |  |  |  |  |  |  |  |  |  |  |  |  |  |  |  |  |  |  |  |  |  |  | *deg-1* | DEGeneration of certain neurons |
|  |  |  |  |  |  |  |  |  |  |  |  |  |  |  |  |  |  |  |  |  |  |  |  |  |  |  |  |  |  |  |  |  |  |  |  |  |  | *srd-23* | Serpentine Receptor, class D (delta) |
|  |  |  |  |  |  |  |  |  |  |  |  |  |  |  |  |  |  |  |  |  |  |  |  |  |  |  |  |  |  |  |  |  |  |  |  |  |  | C56G2.9 |  |
|  |  |  |  |  |  |  |  |  |  |  |  |  |  |  |  |  |  |  |  |  |  |  |  |  |  |  |  |  |  |  |  |  |  |  |  |  |  | *ocr-2* | Osm-9 and Capsaicin receptor-Related |
|  |  |  |  |  |  |  |  |  |  |  |  |  |  |  |  |  |  |  |  |  |  |  |  |  |  |  |  |  |  |  |  |  |  |  |  |  |  | *sri-30* | Serpentine Receptor, class I |
|  |  |  |  |  |  |  |  |  |  |  |  |  |  |  |  |  |  |  |  |  |  |  |  |  |  |  |  |  |  |  |  |  |  |  |  |  |  | Y49A10A.1 |  |
|  |  |  |  |  |  |  |  |  |  |  |  |  |  |  |  |  |  |  |  |  |  |  |  |  |  |  |  |  |  |  |  |  |  |  |  |  |  | K01D12.8 |  |
|  |  |  |  |  |  |  |  |  |  |  |  |  |  |  |  |  |  |  |  |  |  |  |  |  |  |  |  |  |  |  |  |  |  |  |  |  |  | *arrd-28* | ARRestin Domain protein |
|  |  |  |  |  |  |  |  |  |  |  |  |  |  |  |  |  |  |  |  |  |  |  |  |  |  |  |  |  |  |  |  |  |  |  |  |  |  | Y54E10BL.1 |  |
|  |  |  |  |  |  |  |  |  |  |  |  |  |  |  |  |  |  |  |  |  |  |  |  |  |  |  |  |  |  |  |  |  |  |  |  |  |  | K11D12.9 |  |
|  |  |  |  |  |  |  |  |  |  |  |  |  |  |  |  |  |  |  |  |  |  |  |  |  |  |  |  |  |  |  |  |  |  |  |  |  |  | *gcy-34* | Guanylyl CYclase |
|  |  |  |  |  |  |  |  |  |  |  |  |  |  |  |  |  |  |  |  |  |  |  |  |  |  |  |  |  |  |  |  |  |  |  |  |  |  | C10F3.7 |  |
|  |  |  |  |  |  |  |  |  |  |  |  |  |  |  |  |  |  |  |  |  |  |  |  |  |  |  |  |  |  |  |  |  |  |  |  |  |  | T14B4.9 |  |
|  |  |  |  |  |  |  |  |  |  |  |  |  |  |  |  |  |  |  |  |  |  |  |  |  |  |  |  |  |  |  |  |  |  |  |  |  |  | K07D4.5 |  |
|  |  |  |  |  |  |  |  |  |  |  |  |  |  |  |  |  |  |  |  |  |  |  |  |  |  |  |  |  |  |  |  |  |  |  |  |  |  | *frpr-17* | FMRFamide Peptide Receptor family |
|  |  |  |  |  |  |  |  |  |  |  |  |  |  |  |  |  |  |  |  |  |  |  |  |  |  |  |  |  |  |  |  |  |  |  |  |  |  | ZK994.6 |  |
|  |  |  |  |  |  |  |  |  |  |  |  |  |  |  |  |  |  |  |  |  |  |  |  |  |  |  |  |  |  |  |  |  |  |  |  |  |  | *srt-69* | Serpentine Receptor, class T |
|  |  |  |  |  |  |  |  |  |  |  |  |  |  |  |  |  |  |  |  |  |  |  |  |  |  |  |  |  |  |  |  |  |  |  |  |  |  | ZK616.1 |  |
|  |  |  |  |  |  |  |  |  |  |  |  |  |  |  |  |  |  |  |  |  |  |  |  |  |  |  |  |  |  |  |  |  |  |  |  |  |  | C40H1.8 |  |
|  |  |  |  |  |  |  |  |  |  |  |  |  |  |  |  |  |  |  |  |  |  |  |  |  |  |  |  |  |  |  |  |  |  |  |  |  |  | F08B4.4 |  |
|  |  |  |  |  |  |  |  |  |  |  |  |  |  |  |  |  |  |  |  |  |  |  |  |  |  |  |  |  |  |  |  |  |  |  |  |  |  | EGAP5.1 |  |
|  |  |  |  |  |  |  |  |  |  |  |  |  |  |  |  |  |  |  |  |  |  |  |  |  |  |  |  |  |  |  |  |  |  |  |  |  |  | *gcy-36* | Guanylyl CYclase |
|  |  |  |  |  |  |  |  |  |  |  |  |  |  |  |  |  |  |  |  |  |  |  |  |  |  |  |  |  |  |  |  |  |  |  |  |  |  | *gcy-35* | Guanylyl CYclase |
|  |  |  |  |  |  |  |  |  |  |  |  |  |  |  |  |  |  |  |  |  |  |  |  |  |  |  |  |  |  |  |  |  |  |  |  |  |  | *gcy-37* | Guanylyl CYclase |
|  |  |  |  |  |  |  |  |  |  |  |  |  |  |  |  |  |  |  |  |  |  |  |  |  |  |  |  |  |  |  |  |  |  |  |  |  |  | *gcy-32* | Guanylyl CYclase |
|  |  |  |  |  |  |  |  |  |  |  |  |  |  |  |  |  |  |  |  |  |  |  |  |  |  |  |  |  |  |  |  |  |  |  |  |  |  | T13F3.7 |  |
|  |  |  |  |  |  |  |  |  |  |  |  |  |  |  |  |  |  |  |  |  |  |  |  |  |  |  |  |  |  |  |  |  |  |  |  |  |  | C16D9.8 |  |
|  |  |  |  |  |  |  |  |  |  |  |  |  |  |  |  |  |  |  |  |  |  |  |  |  |  |  |  |  |  |  |  |  |  |  |  |  |  | *sri-31* | Serpentine Receptor, class I |
|  |  |  |  |  |  |  |  |  |  |  |  |  |  |  |  |  |  |  |  |  |  |  |  |  |  |  |  |  |  |  |  |  |  |  |  |  |  | *eak-3* | Enhancer of AKt-1 null |
|  |  |  |  |  |  |  |  |  |  |  |  |  |  |  |  |  |  |  |  |  |  |  |  |  |  |  |  |  |  |  |  |  |  |  |  |  |  | *oac-41* | O-ACyltransferase homolog |
|  |  |  |  |  |  |  |  |  |  |  |  |  |  |  |  |  |  |  |  |  |  |  |  |  |  |  |  |  |  |  |  |  |  |  |  |  |  | T06D8.2 |  |
|  |  |  |  |  |  |  |  |  |  |  |  |  |  |  |  |  |  |  |  |  |  |  |  |  |  |  |  |  |  |  |  |  |  |  |  |  |  | F31F7.2 |  |
|  |  |  |  |  |  |  |  |  |  |  |  |  |  |  |  |  |  |  |  |  |  |  |  |  |  |  |  |  |  |  |  |  |  |  |  |  |  | F28H7.2 |  |
|  |  |  |  |  |  |  |  |  |  |  |  |  |  |  |  |  |  |  |  |  |  |  |  |  |  |  |  |  |  |  |  |  |  |  |  |  |  | C25G6.1 |  |
|  |  |  |  |  |  |  |  |  |  |  |  |  |  |  |  |  |  |  |  |  |  |  |  |  |  |  |  |  |  |  |  |  |  |  |  |  |  | F35C5.11 |  |
|  |  |  |  |  |  |  |  |  |  |  |  |  |  |  |  |  |  |  |  |  |  |  |  |  |  |  |  |  |  |  |  |  |  |  |  |  |  | F28A12.3 |  |
|  |  |  |  |  |  |  |  |  |  |  |  |  |  |  |  |  |  |  |  |  |  |  |  |  |  |  |  |  |  |  |  |  |  |  |  |  |  | R09A1.3 |  |
|  |  |  |  |  |  |  |  |  |  |  |  |  |  |  |  |  |  |  |  |  |  |  |  |  |  |  |  |  |  |  |  |  |  |  |  |  |  | F28C6.9 |  |
|  |  |  |  |  |  |  |  |  |  |  |  |  |  |  |  |  |  |  |  |  |  |  |  |  |  |  |  |  |  |  |  |  |  |  |  |  |  | F18G5.1 |  |
|  |  |  |  |  |  |  |  |  |  |  |  |  |  |  |  |  |  |  |  |  |  |  |  |  |  |  |  |  |  |  |  |  |  |  |  |  |  | *srg-51* | Serpentine Receptor, class G (gamma) |
|  |  |  |  |  |  |  |  |  |  |  |  |  |  |  |  |  |  |  |  |  |  |  |  |  |  |  |  |  |  |  |  |  |  |  |  |  |  | *flp-8* | FMRF-Like Peptide |
|  |  |  |  |  |  |  |  |  |  |  |  |  |  |  |  |  |  |  |  |  |  |  |  |  |  |  |  |  |  |  |  |  |  |  |  |  |  | C47C12.2 |  |
|  |  |  |  |  |  |  |  |  |  |  |  |  |  |  |  |  |  |  |  |  |  |  |  |  |  |  |  |  |  |  |  |  |  |  |  |  |  | *sdf-9* | Synthetic Dauer Formation |
|  |  |  |  |  |  |  |  |  |  |  |  |  |  |  |  |  |  |  |  |  |  |  |  |  |  |  |  |  |  |  |  |  |  |  |  |  |  | C14E2.6 |  |
|  |  |  |  |  |  |  |  |  |  |  |  |  |  |  |  |  |  |  |  |  |  |  |  |  |  |  |  |  |  |  |  |  |  |  |  |  |  | K12B6.4 |  |
|  |  |  |  |  |  |  |  |  |  |  |  |  |  |  |  |  |  |  |  |  |  |  |  |  |  |  |  |  |  |  |  |  |  |  |  |  |  | F09B12.5 |  |
|  |  |  |  |  |  |  |  |  |  |  |  |  |  |  |  |  |  |  |  |  |  |  |  |  |  |  |  |  |  |  |  |  |  |  |  |  |  | *tba-6* | TuBulin, Alpha |
|  |  |  |  |  |  |  |  |  |  |  |  |  |  |  |  |  |  |  |  |  |  |  |  |  |  |  |  |  |  |  |  |  |  |  |  |  |  | D1069.1 |  |
|  |  |  |  |  |  |  |  |  |  |  |  |  |  |  |  |  |  |  |  |  |  |  |  |  |  |  |  |  |  |  |  |  |  |  |  |  |  | F14D7.11 |  |
|  |  |  |  |  |  |  |  |  |  |  |  |  |  |  |  |  |  |  |  |  |  |  |  |  |  |  |  |  |  |  |  |  |  |  |  |  |  | T23F4.1 |  |
|  |  |  |  |  |  |  |  |  |  |  |  |  |  |  |  |  |  |  |  |  |  |  |  |  |  |  |  |  |  |  |  |  |  |  |  |  |  | *twk-25* | TWiK family of potassium channels |
|  |  |  |  |  |  |  |  |  |  |  |  |  |  |  |  |  |  |  |  |  |  |  |  |  |  |  |  |  |  |  |  |  |  |  |  |  |  | Y116A8C.465 |  |
|  |  |  |  |  |  |  |  |  |  |  |  |  |  |  |  |  |  |  |  |  |  |  |  |  |  |  |  |  |  |  |  |  |  |  |  |  |  | R186.10 |  |
|  |  |  |  |  |  |  |  |  |  |  |  |  |  |  |  |  |  |  |  |  |  |  |  |  |  |  |  |  |  |  |  |  |  |  |  |  |  | *lurp-3* | LU (Ly6 Urokinase plasminogen) domain Receptor-related Protein |
|  |  |  |  |  |  |  |  |  |  |  |  |  |  |  |  |  |  |  |  |  |  |  |  |  |  |  |  |  |  |  |  |  |  |  |  |  |  | *srz-11* | Serpentine Receptor, class Z |
|  |  |  |  |  |  |  |  |  |  |  |  |  |  |  |  |  |  |  |  |  |  |  |  |  |  |  |  |  |  |  |  |  |  |  |  |  |  | F22A3.10 |  |
|  |  |  |  |  |  |  |  |  |  |  |  |  |  |  |  |  |  |  |  |  |  |  |  |  |  |  |  |  |  |  |  |  |  |  |  |  |  | T24A6.7 |  |
|  |  |  |  |  |  |  |  |  |  |  |  |  |  |  |  |  |  |  |  |  |  |  |  |  |  |  |  |  |  |  |  |  |  |  |  |  |  | *srz-103* | Serpentine Receptor, class Z |
|  |  |  |  |  |  |  |  |  |  |  |  |  |  |  |  |  |  |  |  |  |  |  |  |  |  |  |  |  |  |  |  |  |  |  |  |  |  | *vap-1* | Venom-Allergen-like Protein |
|  |  |  |  |  |  |  |  |  |  |  |  |  |  |  |  |  |  |  |  |  |  |  |  |  |  |  |  |  |  |  |  |  |  |  |  |  |  | *ckb-3* | Choline Kinase B |
|  |  |  |  |  |  |  |  |  |  |  |  |  |  |  |  |  |  |  |  |  |  |  |  |  |  |  |  |  |  |  |  |  |  |  |  |  |  | *linc-15* | Long Intervening Non-Coding RNA |
|  |  |  |  |  |  |  |  |  |  |  |  |  |  |  |  |  |  |  |  |  |  |  |  |  |  |  |  |  |  |  |  |  |  |  |  |  |  | C13A10.2 |  |
|  |  |  |  |  |  |  |  |  |  |  |  |  |  |  |  |  |  |  |  |  |  |  |  |  |  |  |  |  |  |  |  |  |  |  |  |  |  | C11E4.8 |  |
|  |  |  |  |  |  |  |  |  |  |  |  |  |  |  |  |  |  |  |  |  |  |  |  |  |  |  |  |  |  |  |  |  |  |  |  |  |  | *tkr-2* | TachyKinin Receptor family |
|  |  |  |  |  |  |  |  |  |  |  |  |  |  |  |  |  |  |  |  |  |  |  |  |  |  |  |  |  |  |  |  |  |  |  |  |  |  | *flp-7* | FMRF-Like Peptide |
|  |  |  |  |  |  |  |  |  |  |  |  |  |  |  |  |  |  |  |  |  |  |  |  |  |  |  |  |  |  |  |  |  |  |  |  |  |  | C39D10.7 |  |
|  |  |  |  |  |  |  |  |  |  |  |  |  |  |  |  |  |  |  |  |  |  |  |  |  |  |  |  |  |  |  |  |  |  |  |  |  |  | *osm-10* | OSMotic avoidance abnormal |
|  |  |  |  |  |  |  |  |  |  |  |  |  |  |  |  |  |  |  |  |  |  |  |  |  |  |  |  |  |  |  |  |  |  |  |  |  |  | *srh-21* | Serpentine Receptor, class H |
|  |  |  |  |  |  |  |  |  |  |  |  |  |  |  |  |  |  |  |  |  |  |  |  |  |  |  |  |  |  |  |  |  |  |  |  |  |  | C02B4.4 |  |
|  |  |  |  |  |  |  |  |  |  |  |  |  |  |  |  |  |  |  |  |  |  |  |  |  |  |  |  |  |  |  |  |  |  |  |  |  |  | F58E6.6 |  |
|  |  |  |  |  |  |  |  |  |  |  |  |  |  |  |  |  |  |  |  |  |  |  |  |  |  |  |  |  |  |  |  |  |  |  |  |  |  | Y119C1B.3 |  |
|  |  |  |  |  |  |  |  |  |  |  |  |  |  |  |  |  |  |  |  |  |  |  |  |  |  |  |  |  |  |  |  |  |  |  |  |  |  | T23F4.2 |  |
|  |  |  |  |  |  |  |  |  |  |  |  |  |  |  |  |  |  |  |  |  |  |  |  |  |  |  |  |  |  |  |  |  |  |  |  |  |  | *cutl-12* | CUTiclin-Like |
|  |  |  |  |  |  |  |  |  |  |  |  |  |  |  |  |  |  |  |  |  |  |  |  |  |  |  |  |  |  |  |  |  |  |  |  |  |  | *grd-15* | GRounDhog (hedgehog-like family) |
|  |  |  |  |  |  |  |  |  |  |  |  |  |  |  |  |  |  |  |  |  |  |  |  |  |  |  |  |  |  |  |  |  |  |  |  |  |  | *col-75* | COLlagen |
|  |  |  |  |  |  |  |  |  |  |  |  |  |  |  |  |  |  |  |  |  |  |  |  |  |  |  |  |  |  |  |  |  |  |  |  |  |  | F15A8.1 |  |
|  |  |  |  |  |  |  |  |  |  |  |  |  |  |  |  |  |  |  |  |  |  |  |  |  |  |  |  |  |  |  |  |  |  |  |  |  |  | R102.11 |  |
|  |  |  |  |  |  |  |  |  |  |  |  |  |  |  |  |  |  |  |  |  |  |  |  |  |  |  |  |  |  |  |  |  |  |  |  |  |  | C43F9.11 |  |
|  |  |  |  |  |  |  |  |  |  |  |  |  |  |  |  |  |  |  |  |  |  |  |  |  |  |  |  |  |  |  |  |  |  |  |  |  |  | *col-187* | COLlagen |
|  |  |  |  |  |  |  |  |  |  |  |  |  |  |  |  |  |  |  |  |  |  |  |  |  |  |  |  |  |  |  |  |  |  |  |  |  |  | *sri-73* | Serpentine Receptor, class I |
|  |  |  |  |  |  |  |  |  |  |  |  |  |  |  |  |  |  |  |  |  |  |  |  |  |  |  |  |  |  |  |  |  |  |  |  |  |  | C34B4.5 |  |
|  |  |  |  |  |  |  |  |  |  |  |  |  |  |  |  |  |  |  |  |  |  |  |  |  |  |  |  |  |  |  |  |  |  |  |  |  |  | ZK105.8 |  |
|  |  |  |  |  |  |  |  |  |  |  |  |  |  |  |  |  |  |  |  |  |  |  |  |  |  |  |  |  |  |  |  |  |  |  |  |  |  | *srsx-34* | Serpentine Receptor, class SX |
|  |  |  |  |  |  |  |  |  |  |  |  |  |  |  |  |  |  |  |  |  |  |  |  |  |  |  |  |  |  |  |  |  |  |  |  |  |  | T01C8.3 |  |
|  |  |  |  |  |  |  |  |  |  |  |  |  |  |  |  |  |  |  |  |  |  |  |  |  |  |  |  |  |  |  |  |  |  |  |  |  |  | F52G2.3 |  |
|  |  |  |  |  |  |  |  |  |  |  |  |  |  |  |  |  |  |  |  |  |  |  |  |  |  |  |  |  |  |  |  |  |  |  |  |  |  | T09B9.3 |  |
|  |  |  |  |  |  |  |  |  |  |  |  |  |  |  |  |  |  |  |  |  |  |  |  |  |  |  |  |  |  |  |  |  |  |  |  |  |  | F55F8.7 |  |
|  |  |  |  |  |  |  |  |  |  |  |  |  |  |  |  |  |  |  |  |  |  |  |  |  |  |  |  |  |  |  |  |  |  |  |  |  |  | C46H11.3 |  |
|  |  |  |  |  |  |  |  |  |  |  |  |  |  |  |  |  |  |  |  |  |  |  |  |  |  |  |  |  |  |  |  |  |  |  |  |  |  | H41C03.2 |  |

### Phenotypes enriched

none found

### Anatomy terms enriched

|  |  |  |  |
| --- | --- | --- | --- |
| **Group name** | **Number in cluster** | **Enrichment** | **FDR corrected p** |
| PQR | 7 | 26.19 | 2.34e-05 |
| AQR | 5 | 33.25 | 7.79e-04 |
| URXR | 6 | 17.10 | 2.91e-03 |
| URXL | 6 | 17.10 | 2.91e-03 |
| URX | 6 | 17.10 | 2.91e-03 |
| lumbar lateral left ganglion neuron | 11 | 6.01 | 5.02e-03 |
| lumbar lateral right ganglion neuron | 11 | 5.85 | 6.40e-03 |
| lumbar neuron | 11 | 5.80 | 6.83e-03 |
| lumbar lateral left ganglion | 11 | 5.68 | 8.28e-03 |
| lumbar lateral ganglion | 11 | 5.56 | 9.89e-03 |
| lumbar lateral right ganglion | 11 | 5.56 | 9.89e-03 |
| lumbar ganglion | 11 | 5.18 | 1.82e-02 |
| dorsal pharyngeal ganglion | 7 | 9.31 | 1.98e-02 |

### GO terms enriched

|  |  |  |
| --- | --- | --- |
| **GO term** | **Number of genes** | **FDR-corrected p-value** |
| guanylate cyclase activity | 5 | 0.00019 |
| cGMP biosynthetic process | 5 | 0.00020 |
| cyclic nucleotide biosynthetic process | 5 | 0.00087 |
| purine nucleotide biosynthetic process | 5 | 0.00560 |
| ribonucleotide biosynthetic process | 5 | 0.00750 |
| heme binding | 5 | 0.01200 |
| GTP binding | 6 | 0.02400 |
| guanyl nucleotide binding | 6 | 0.02700 |
| lyase activity | 5 | 0.02900 |
| nucleoside phosphate biosynthetic process | 5 | 0.03000 |
| organonitrogen compound biosynthetic process | 6 | 0.04800 |

### Expression clusters enriched

|  |  |  |  |
| --- | --- | --- | --- |
| **Group name** | **Number in cluster** | **Enrichment** | **FDR corrected p** |
| Larval Pan-neural Enriched Genes. | 46 | 3.56 | 3.67e-12 |
| Genes that show selective expression in a subset of cell types vs broadly expressed in many cell types. Correspond to 20% - 57% of enriched\_genes for a given cell type. WBPaper00037950:all-neurons\_larva\_SelectivelyEnriched | 27 | 5.92 | 5.48e-11 |
| Genes significantly enriched (> 2x, FDR < 5%) in a particular cell-type versus a reference sample of all cells at the same stage. WBPaper00037950:all-neurons\_larva\_enriched | 35 | 4.14 | 2.35e-10 |
| WT-Pico Pan-neural Enriched Genes, with genes found multiple times in a single dataset removed (without dups). | 33 | 2.46 | 2.84e-04 |
| Genes that show selective expression in a subset of cell types vs broadly expressed in many cell types. Correspond to 20% - 57% of enriched\_genes for a given cell type. WBPaper00037950:BAG-neuron\_embryo\_SelectivelyEnriched | 11 | 6.30 | 5.36e-04 |
| Genes that show selective expression in a subset of cell types vs broadly expressed in many cell types. Correspond to 20% - 57% of enriched\_genes for a given cell type. WBPaper00037950:glr-1(+)-neurons\_larva\_SelectivelyEnriched | 11 | 5.12 | 3.18e-03 |
| Candidate daf-19 down regulated genes with a statistically significant signal variation of 1.5-fold or greater. These were identified using a class comparisons tool from BRB Array Tools. | 14 | 3.10 | 3.86e-02 |

### Motifs enriched

|  |  |  |  |  |  |
| --- | --- | --- | --- | --- | --- |
| **Motif** | **Logo** | **Possible orthologs** | **Number of motifs in cluster** | **Enrichment** | **FDR corrected p** |
| pTH9226 |  | daf-19 | 76 | 1.82 | 4.0e-07 |
| nau\_da\_SANGER\_5\_FBgn0002922 |  | hlh-15 hlh-1 K02D7.2 ces-1 | 65 | 1.81 | 2.2e-05 |
| pTH10638 |  | dmd-3 C34D1.1 | 115 | 1.28 | 9.7e-05 |
| pTH9199 |  | daf-19 | 38 | 2.32 | 1.2e-04 |
| V$AREB6\_02 |  | ztf-6 | 86 | 1.49 | 1.4e-04 |
| pTH9246 |  | lin-31 (0.54) let-381 C34D1.1 | 116 | 1.24 | 3.9e-04 |
| MEIS2\_2 |  | ceh-32 | 86 | 1.44 | 6.4e-04 |
| MA0500.1 |  | lin-32 hlh-11 hlh-1 hlh-14 | 76 | 1.52 | 7.3e-04 |
| HXC8\_f1 |  | lin-39 | 103 | 1.31 | 9.7e-04 |
| K562\_ELF1\_HudsonAlpha |  | lin-1 C24A1.2 | 123 | 1.18 | 1.0e-03 |
| Foxg1\_1 |  | fkh-8 (0.55) lin-31 (0.54) let-381 pha-4 fkh-7 daf-16 | 89 | 1.40 | 1.1e-03 |
| CG4328\_SOLEXA\_FBgn0036274 |  | alr-1 (0.63) ceh-18 (0.52) ceh-30 lim-7 ceh-31 cog-1 ceh-10 ceh-45 lin-39 ceh-43 lim-4 egl-5 pal-1 ceh-14 lim-6 pha-2 ceh-16 T13C5.4 ZC123.3 | 13 | 4.50 | 1.3e-03 |
| NR4A2\_3 |  | nhr-6 nhr-2 | 97 | 1.34 | 1.4e-03 |
| V$NCX\_01 |  | ceh-19 (0.55) | 112 | 1.24 | 1.5e-03 |
| pTH9142 |  | C34D1.1 | 71 | 1.53 | 1.5e-03 |
| CG34031\_SOLEXA\_FBgn0054031 |  | ceh-8 (0.86) alr-1 (0.63) ceh-19 (0.55) ceh-30 ceh-31 lin-39 ceh-9 | 99 | 1.32 | 1.7e-03 |
| SOX2\_f1 |  | ceh-18 (0.52) sox-4 | 75 | 1.48 | 1.9e-03 |
| MA0509.1 |  | daf-19 F52B5.7 | 36 | 2.07 | 2.0e-03 |
| dsx-F\_FlyReg\_FBgn0000504 |  | sox-4 dmd-4 | 65 | 1.57 | 2.1e-03 |
| pTH6498 |  | alr-1 (0.63) ceh-1 lin-39 | 84 | 1.41 | 2.2e-03 |
| SPDEF\_1 |  | nhr-19 lin-1 C24A1.2 | 17 | 3.30 | 2.4e-03 |
| V$XFD1\_01 |  | lin-31 (0.54) let-381 | 105 | 1.27 | 2.6e-03 |
| MA0058.2 |  | mxl-1 | 113 | 1.22 | 2.6e-03 |
| MEIS3\_1 |  | ceh-32 ces-1 | 85 | 1.39 | 2.9e-03 |
| NR5A2\_f1 |  | nhr-68 | 65 | 1.55 | 3.2e-03 |
| TBX1\_1 |  | tbx-39 mab-9 | 99 | 1.30 | 3.2e-03 |
| pTH5781 |  | ceh-32 | 78 | 1.43 | 3.6e-03 |
| SP4\_1 |  | klf-2 | 75 | 1.45 | 3.9e-03 |
| MYF6\_f1 |  | hlh-1 | 49 | 1.73 | 4.0e-03 |
| HMGA1\_f1 |  | Y116A8C.22 | 95 | 1.32 | 4.1e-03 |
| MA0483.1 |  | odd-1 lim-6 | 40 | 1.87 | 5.1e-03 |
| pTH6641 |  | lin-31 (0.54) | 105 | 1.25 | 5.4e-03 |
| MA0161.1 |  | nfi-1 | 84 | 1.37 | 5.5e-03 |
| TFE3\_1 |  | hlh-30 mxl-1 | 61 | 1.55 | 5.9e-03 |
| Oc\_SOLEXA\_FBgn0004102 |  | alr-1 (0.63) dve-1 ceh-45 ceh-53 ceh-36 | 106 | 1.24 | 6.2e-03 |
| PAX8\_f1 |  | pax-2 | 102 | 1.26 | 6.2e-03 |
| pTH5919 |  | irx-1 | 101 | 1.27 | 6.4e-03 |
| vnd\_FlyReg\_FBgn0003986 |  | ceh-24 (0.51) ceh-22 dsc-1 | 125 | 1.13 | 7.1e-03 |
| PHOX2B\_2 |  | alr-1 (0.63) | 78 | 1.40 | 8.0e-03 |
| FOXO3\_2 |  | fkh-8 (0.55) lin-31 (0.54) fkh-7 daf-16 | 98 | 1.28 | 8.9e-03 |
| HXC6\_f1 |  | lin-39 | 89 | 1.32 | 9.0e-03 |
| Elf3\_3876 |  | C24A1.2 | 95 | 1.29 | 9.3e-03 |
| MA0069.1 |  | pax-3 (-0.69) | 45 | 1.71 | 9.7e-03 |
| MA0598.1 |  | lin-1 C24A1.2 | 64 | 1.49 | 9.9e-03 |
| pTH10647 |  | nhr-232 | 123 | 1.14 | 1.1e-02 |
| pTH6071 |  | C33G8.2 | 34 | 1.91 | 1.2e-02 |
| Hmx3\_3490 |  | ceh-9 | 114 | 1.19 | 1.2e-02 |
| Spdef |  | lin-1 | 28 | 2.09 | 1.2e-02 |
| Hlx1\_2350 |  | ceh-24 (0.51) lim-7 ceh-16 | 24 | 2.26 | 1.3e-02 |
| pTH10816 |  | dmd-6 | 86 | 1.33 | 1.3e-02 |
| MA0017.1 |  | nhr-239 nhr-62 nhr-2 | 71 | 1.42 | 1.3e-02 |
| pTH3477 |  | daf-16 | 53 | 1.58 | 1.3e-02 |
| MSX2\_2 |  | ceh-1 lim-7 ceh-43 ceh-16 | 86 | 1.33 | 1.3e-02 |
| pTH5337 |  | ZC328.2 (0.54) | 26 | 2.16 | 1.3e-02 |
| pTH9916 |  | W08E12.1 | 39 | 1.78 | 1.4e-02 |
| pTH3041 |  | atf-2 | 106 | 1.22 | 1.4e-02 |
| pTH9708 |  | ceh-34 | 22 | 2.35 | 1.4e-02 |
| TFEB\_1 |  | hlh-30 aha-1 | 60 | 1.50 | 1.4e-02 |
| pTH6562 |  | ceh-5 | 112 | 1.19 | 1.5e-02 |
| PO3F2\_si |  | ceh-18 (0.52) | 111 | 1.20 | 1.5e-02 |
| Pou3f4\_3773 |  | ceh-6 | 57 | 1.53 | 1.6e-02 |
| HNF4A\_6 |  | nhr-62 nhr-2 | 86 | 1.32 | 1.6e-02 |
| PDX1\_do |  | alr-1 (0.63) ceh-18 (0.52) lin-39 ceh-12 | 68 | 1.43 | 1.6e-02 |
| pTH10015 |  | che-1 ces-1 | 116 | 1.17 | 1.7e-02 |
| gl\_FlyReg\_FBgn0004618 |  | ces-1 | 74 | 1.39 | 1.7e-02 |
| V$HMX1\_01 |  | ceh-9 | 70 | 1.41 | 1.9e-02 |
| pTH9300 |  | dmd-3 | 91 | 1.29 | 1.9e-02 |
| pTH2936 |  | nhr-239 | 76 | 1.37 | 1.9e-02 |
| Elf5 |  | C24A1.2 | 120 | 1.15 | 1.9e-02 |
| En1\_3123 |  | ceh-53 ceh-16 | 120 | 1.15 | 1.9e-02 |
| V$DELTAEF1\_01 |  | ztf-6 | 96 | 1.26 | 2.0e-02 |
| Lhx1\_2240 |  | lim-7 | 22 | 2.28 | 2.0e-02 |
| pTH9132 |  | ceh-24 (0.51) T27F2.4 | 89 | 1.29 | 2.0e-02 |
| MA0060.2 |  | lin-31 (0.54) dro-1 ceh-20 nfya-2 | 36 | 1.79 | 2.1e-02 |
| I$KR\_01 |  | B0310.2 | 71 | 1.40 | 2.1e-02 |
| MA0476.1 |  | fos-1 | 109 | 1.20 | 2.2e-02 |
| V$HOX13\_01 |  | lin-39 | 53 | 1.54 | 2.2e-02 |
| pTH5082 |  | atf-2 | 72 | 1.39 | 2.3e-02 |
| Oli\_da\_SANGER\_5\_2\_FBgn0032651 |  | hlh-32 | 105 | 1.21 | 2.3e-02 |
| MA0244.1 |  | C48E7.11 | 115 | 1.17 | 2.4e-02 |
| pTH8411 |  | tbx-39 | 84 | 1.31 | 2.4e-02 |
| V$GR\_Q6 |  | nhr-255 (0.55) | 78 | 1.35 | 2.4e-02 |
| V$TATA\_C |  | tbp-1 | 82 | 1.32 | 2.4e-02 |
| pTH6486 |  | nhr-145 | 32 | 1.87 | 2.4e-02 |
| pTH9198 |  | dmd-3 | 98 | 1.24 | 2.5e-02 |
| Msx3\_1 |  | ceh-1 | 72 | 1.38 | 2.5e-02 |
| Evx2\_2645 |  | alr-1 (0.63) ceh-45 ceh-53 | 92 | 1.27 | 2.6e-02 |
| pTH5812 |  | ceh-14 | 56 | 1.50 | 2.7e-02 |
| pTH3091 |  | ZC328.2 (0.54) | 58 | 1.48 | 2.7e-02 |
| N$SKN1\_01 |  | skn-1 | 81 | 1.32 | 2.8e-02 |
| RORA\_2 |  | nhr-213 (0.53) | 59 | 1.47 | 2.9e-02 |
| SP1\_f1 |  | klf-2 | 73 | 1.37 | 2.9e-02 |
| Nkx1-2\_3214 |  | ceh-30 | 105 | 1.20 | 3.1e-02 |
| MA0174.1 |  | ceh-24 (0.51) | 102 | 1.22 | 3.1e-02 |
| V$AREB6\_01 |  | ztf-6 | 10 | 3.68 | 3.1e-02 |
| Irx3\_2226 |  | irx-1 | 105 | 1.20 | 3.1e-02 |
| Nkx2-3\_3435 |  | ceh-24 (0.51) | 107 | 1.19 | 3.4e-02 |
| Mw138 |  | dsc-1 | 106 | 1.20 | 3.5e-02 |
| LHX6\_3 |  | lim-6 | 64 | 1.41 | 3.5e-02 |
| Pou2f2\_1 |  | ceh-18 (0.52) unc-86 | 84 | 1.29 | 3.8e-02 |
| I$SN\_02 |  | K02D7.2 | 120 | 1.13 | 3.8e-02 |
| MA0467.1 |  | ceh-45 | 12 | 3.05 | 4.0e-02 |
| Mv90 |  | mef-2 | 116 | 1.15 | 4.0e-02 |
| pTH9137 |  | nhr-65 | 83 | 1.29 | 4.1e-02 |
| Tcf7\_0950 |  | pop-1 | 111 | 1.17 | 4.2e-02 |
| Aef1\_FlyReg\_FBgn0005694 |  | K11D2.4 | 79 | 1.31 | 4.3e-02 |
| DLX2\_f1 |  | ceh-43 | 39 | 1.65 | 4.4e-02 |
| Pknox2\_3077 |  | ceh-32 | 62 | 1.41 | 4.4e-02 |
| pTH9216 |  | ceh-18 (0.52) | 69 | 1.37 | 4.4e-02 |
| pTH9380 |  | mel-28 | 99 | 1.22 | 4.5e-02 |
| PO4F2\_si |  | unc-86 | 90 | 1.26 | 4.6e-02 |
| ZN384\_f1 |  | lin-29 | 105 | 1.19 | 4.8e-02 |
| pTH10772 |  | ceh-52 | 108 | 1.18 | 4.8e-02 |
| pTH2820 |  | ZC328.2 (0.54) | 64 | 1.39 | 4.8e-02 |
| Hoxa2\_3079 |  | lin-39 | 70 | 1.36 | 4.9e-02 |
| pTH9163 |  | nhr-3 | 88 | 1.26 | 5.0e-02 |
| SPDEF\_2 |  | lin-1 | 106 | 1.19 | 5.0e-02 |

### Correlated (and anti-correlated) transcription factors

|  |  |
| --- | --- |
| **Transcription factor** | **Correlation** |
| hlh-4 | 0.86 |
| nhr-188 | 0.86 |
| ceh-8 | 0.86 |
| nhr-216 | 0.85 |
| nhr-187 | 0.81 |
| ets-5 | 0.81 |
| npax-2 | 0.73 |
| tab-1 | 0.72 |
| F13H6.1 | 0.70 |
| ceh-90 | 0.67 |
| nhr-181 | 0.66 |
| Y22D7AL.16 | 0.65 |
| lfi-1 | 0.65 |
| bed-3 | 0.63 |
| alr-1 | 0.63 |
| elt-6 | 0.63 |
| nhr-253 | 0.63 |
| nhr-40 | 0.62 |
| odr-7 | 0.61 |
| nhr-183 | 0.60 |
| nhr-277 | 0.58 |
| nhr-153 | 0.58 |
| nhr-1 | 0.58 |
| dhhc-2 | 0.58 |
| nhr-96 | 0.58 |
| sup-35 | -0.49 |
| zip-11 | -0.50 |
| ceh-51 | -0.51 |
| Y57A10A.31 | -0.51 |
| nhr-210 | -0.52 |
| mex-5 | -0.52 |
| hmbx-1 | -0.53 |
| F49E8.2 | -0.54 |
| zip-8 | -0.54 |
| Y48G9A.11 | -0.54 |
| nhr-150 | -0.55 |
| spe-44 | -0.56 |
| ubxn-1 | -0.57 |
| pqn-75 | -0.58 |
| nhr-80 | -0.59 |
| nhr-246 | -0.60 |
| hmg-12 | -0.60 |
| pzf-1 | -0.60 |
| ccch-3 | -0.60 |
| cep-1 | -0.65 |
| ztf-28 | -0.66 |
| pos-1 | -0.68 |
| pax-3 | -0.69 |
| nhr-171 | -0.75 |
| cey-2 | -0.80 |

### ChIP peaks enriched

none found
