# Supplementary material for: Overlapping cell population expression profiling and regulatory inference in C. elegans
Source: BMC Genomics. 2016 Feb 29;17:159. doi: 10.1186/s12864-016-2482-z (PMC4772325; doi:10.1186/s12864-016-2482-z)
Supplement: Additional file 13: — Web supplement. (DOC 21 kb) [file 12864_2016_2482_MOESM13_ESM.zip › sortWeb/clusters/hier.300.clusters/251.html]

Cluster 251 

## Cluster 251

### Expression

| cnd-1 rep. 1 | cnd-1 rep. 2 | cnd-1 rep. 3 | pha-4 rep. 1 | pha-4 rep. 2 | pha-4 rep. 3 | ceh-27 | ceh-36 | ceh-6 | F21D5.9 | mir-57 | mls-2 | pal-1 | pros-1 | ttx-3 | unc-130 | hlh-16 | irx-1 | ceh-6 (+) hlh-16 (+) | ceh-6 (+) hlh-16 (-) | ceh-6 (-) hlh-16 (+) | cnd-1 singlets | pha-4 singlets | 0 | 60 | 120 | 150 | 180 | 240 | 330 | 390 | 420 | 480 | 540 | 570 | 600 | 630 | 660 | NAME | Functional description |
| --- | --- | --- | --- | --- | --- | --- | --- | --- | --- | --- | --- | --- | --- | --- | --- | --- | --- | --- | --- | --- | --- | --- | --- | --- | --- | --- | --- | --- | --- | --- | --- | --- | --- | --- | --- | --- | --- | --- | --- |
|  |  |  |  |  |  |  |  |  |  |  |  |  |  |  |  |  |  |  |  |  |  |  |  |  |  |  |  |  |  |  |  |  |  |  |  |  |  | *lips-11* | LIPaSe related |
|  |  |  |  |  |  |  |  |  |  |  |  |  |  |  |  |  |  |  |  |  |  |  |  |  |  |  |  |  |  |  |  |  |  |  |  |  |  | *pud-3* |  |
|  |  |  |  |  |  |  |  |  |  |  |  |  |  |  |  |  |  |  |  |  |  |  |  |  |  |  |  |  |  |  |  |  |  |  |  |  |  | *math-35* | MATH (meprin-associated Traf homology) domain containing |
|  |  |  |  |  |  |  |  |  |  |  |  |  |  |  |  |  |  |  |  |  |  |  |  |  |  |  |  |  |  |  |  |  |  |  |  |  |  | *dgat-2* | acyl-CoA:DiacylGlycerol AcylTransferase |
|  |  |  |  |  |  |  |  |  |  |  |  |  |  |  |  |  |  |  |  |  |  |  |  |  |  |  |  |  |  |  |  |  |  |  |  |  |  | *math-3* | MATH (meprin-associated Traf homology) domain containing |
|  |  |  |  |  |  |  |  |  |  |  |  |  |  |  |  |  |  |  |  |  |  |  |  |  |  |  |  |  |  |  |  |  |  |  |  |  |  | *math-26* | MATH (meprin-associated Traf homology) domain containing |
|  |  |  |  |  |  |  |  |  |  |  |  |  |  |  |  |  |  |  |  |  |  |  |  |  |  |  |  |  |  |  |  |  |  |  |  |  |  | *trpl-1* | TRP-channel-Like |
|  |  |  |  |  |  |  |  |  |  |  |  |  |  |  |  |  |  |  |  |  |  |  |  |  |  |  |  |  |  |  |  |  |  |  |  |  |  | C23H4.7 |  |
|  |  |  |  |  |  |  |  |  |  |  |  |  |  |  |  |  |  |  |  |  |  |  |  |  |  |  |  |  |  |  |  |  |  |  |  |  |  | F49E12.10 |  |
|  |  |  |  |  |  |  |  |  |  |  |  |  |  |  |  |  |  |  |  |  |  |  |  |  |  |  |  |  |  |  |  |  |  |  |  |  |  | *lmp-2* | LAMP (lysosome-associated membrane protein) homolog |
|  |  |  |  |  |  |  |  |  |  |  |  |  |  |  |  |  |  |  |  |  |  |  |  |  |  |  |  |  |  |  |  |  |  |  |  |  |  | *nhr-79* | Nuclear Hormone Receptor family |
|  |  |  |  |  |  |  |  |  |  |  |  |  |  |  |  |  |  |  |  |  |  |  |  |  |  |  |  |  |  |  |  |  |  |  |  |  |  | C06C3.11 |  |
|  |  |  |  |  |  |  |  |  |  |  |  |  |  |  |  |  |  |  |  |  |  |  |  |  |  |  |  |  |  |  |  |  |  |  |  |  |  | *cyp-13A6* | CYtochrome P450 family |
|  |  |  |  |  |  |  |  |  |  |  |  |  |  |  |  |  |  |  |  |  |  |  |  |  |  |  |  |  |  |  |  |  |  |  |  |  |  | *zip-10* | bZIP transcription factor family |
|  |  |  |  |  |  |  |  |  |  |  |  |  |  |  |  |  |  |  |  |  |  |  |  |  |  |  |  |  |  |  |  |  |  |  |  |  |  | *fbxa-54* | F-box A protein |
|  |  |  |  |  |  |  |  |  |  |  |  |  |  |  |  |  |  |  |  |  |  |  |  |  |  |  |  |  |  |  |  |  |  |  |  |  |  | *dct-11* | DAF-16/FOXO Controlled, germline Tumor affecting |
|  |  |  |  |  |  |  |  |  |  |  |  |  |  |  |  |  |  |  |  |  |  |  |  |  |  |  |  |  |  |  |  |  |  |  |  |  |  | *drr-1* | Dietary Restriction Response (WT but not eat-2 lifespan increased) |
|  |  |  |  |  |  |  |  |  |  |  |  |  |  |  |  |  |  |  |  |  |  |  |  |  |  |  |  |  |  |  |  |  |  |  |  |  |  | *swt-6* | SWEET sugar transporter family |
|  |  |  |  |  |  |  |  |  |  |  |  |  |  |  |  |  |  |  |  |  |  |  |  |  |  |  |  |  |  |  |  |  |  |  |  |  |  | K09H11.1 |  |
|  |  |  |  |  |  |  |  |  |  |  |  |  |  |  |  |  |  |  |  |  |  |  |  |  |  |  |  |  |  |  |  |  |  |  |  |  |  | *ugt-49* | UDP-GlucuronosylTransferase |
|  |  |  |  |  |  |  |  |  |  |  |  |  |  |  |  |  |  |  |  |  |  |  |  |  |  |  |  |  |  |  |  |  |  |  |  |  |  | *nhr-176* | Nuclear Hormone Receptor family |
|  |  |  |  |  |  |  |  |  |  |  |  |  |  |  |  |  |  |  |  |  |  |  |  |  |  |  |  |  |  |  |  |  |  |  |  |  |  | C29F9.4 |  |
|  |  |  |  |  |  |  |  |  |  |  |  |  |  |  |  |  |  |  |  |  |  |  |  |  |  |  |  |  |  |  |  |  |  |  |  |  |  | Y105C5A.24 |  |
|  |  |  |  |  |  |  |  |  |  |  |  |  |  |  |  |  |  |  |  |  |  |  |  |  |  |  |  |  |  |  |  |  |  |  |  |  |  | C14A4.7 |  |
|  |  |  |  |  |  |  |  |  |  |  |  |  |  |  |  |  |  |  |  |  |  |  |  |  |  |  |  |  |  |  |  |  |  |  |  |  |  | F39G3.5 |  |
|  |  |  |  |  |  |  |  |  |  |  |  |  |  |  |  |  |  |  |  |  |  |  |  |  |  |  |  |  |  |  |  |  |  |  |  |  |  | Y32F6B.1 |  |
|  |  |  |  |  |  |  |  |  |  |  |  |  |  |  |  |  |  |  |  |  |  |  |  |  |  |  |  |  |  |  |  |  |  |  |  |  |  | T04F3.4 |  |
|  |  |  |  |  |  |  |  |  |  |  |  |  |  |  |  |  |  |  |  |  |  |  |  |  |  |  |  |  |  |  |  |  |  |  |  |  |  | C25F9.10 |  |
|  |  |  |  |  |  |  |  |  |  |  |  |  |  |  |  |  |  |  |  |  |  |  |  |  |  |  |  |  |  |  |  |  |  |  |  |  |  | F58A6.1 |  |
|  |  |  |  |  |  |  |  |  |  |  |  |  |  |  |  |  |  |  |  |  |  |  |  |  |  |  |  |  |  |  |  |  |  |  |  |  |  | C49A9.5 |  |
|  |  |  |  |  |  |  |  |  |  |  |  |  |  |  |  |  |  |  |  |  |  |  |  |  |  |  |  |  |  |  |  |  |  |  |  |  |  | *cdd-1* | CytiDine Deaminase |
|  |  |  |  |  |  |  |  |  |  |  |  |  |  |  |  |  |  |  |  |  |  |  |  |  |  |  |  |  |  |  |  |  |  |  |  |  |  | C49C3.4 |  |
|  |  |  |  |  |  |  |  |  |  |  |  |  |  |  |  |  |  |  |  |  |  |  |  |  |  |  |  |  |  |  |  |  |  |  |  |  |  | ZK185.5 |  |
|  |  |  |  |  |  |  |  |  |  |  |  |  |  |  |  |  |  |  |  |  |  |  |  |  |  |  |  |  |  |  |  |  |  |  |  |  |  | Y45G12C.1 |  |
|  |  |  |  |  |  |  |  |  |  |  |  |  |  |  |  |  |  |  |  |  |  |  |  |  |  |  |  |  |  |  |  |  |  |  |  |  |  | *nhr-81* | Nuclear Hormone Receptor family |
|  |  |  |  |  |  |  |  |  |  |  |  |  |  |  |  |  |  |  |  |  |  |  |  |  |  |  |  |  |  |  |  |  |  |  |  |  |  | R10E8.8 |  |
|  |  |  |  |  |  |  |  |  |  |  |  |  |  |  |  |  |  |  |  |  |  |  |  |  |  |  |  |  |  |  |  |  |  |  |  |  |  | F12B6.2 |  |
|  |  |  |  |  |  |  |  |  |  |  |  |  |  |  |  |  |  |  |  |  |  |  |  |  |  |  |  |  |  |  |  |  |  |  |  |  |  | *pcp-3* | Prolyl Carboxy Peptidase like |
|  |  |  |  |  |  |  |  |  |  |  |  |  |  |  |  |  |  |  |  |  |  |  |  |  |  |  |  |  |  |  |  |  |  |  |  |  |  | *amt-4* | AMmonium Transporter homolog |
|  |  |  |  |  |  |  |  |  |  |  |  |  |  |  |  |  |  |  |  |  |  |  |  |  |  |  |  |  |  |  |  |  |  |  |  |  |  | R09F10.1 |  |
|  |  |  |  |  |  |  |  |  |  |  |  |  |  |  |  |  |  |  |  |  |  |  |  |  |  |  |  |  |  |  |  |  |  |  |  |  |  | C18E9.5 |  |
|  |  |  |  |  |  |  |  |  |  |  |  |  |  |  |  |  |  |  |  |  |  |  |  |  |  |  |  |  |  |  |  |  |  |  |  |  |  | K11E4.2 |  |
|  |  |  |  |  |  |  |  |  |  |  |  |  |  |  |  |  |  |  |  |  |  |  |  |  |  |  |  |  |  |  |  |  |  |  |  |  |  | F59C6.14 |  |
|  |  |  |  |  |  |  |  |  |  |  |  |  |  |  |  |  |  |  |  |  |  |  |  |  |  |  |  |  |  |  |  |  |  |  |  |  |  | Y54G2A.52 |  |
|  |  |  |  |  |  |  |  |  |  |  |  |  |  |  |  |  |  |  |  |  |  |  |  |  |  |  |  |  |  |  |  |  |  |  |  |  |  | C06B8.2 |  |
|  |  |  |  |  |  |  |  |  |  |  |  |  |  |  |  |  |  |  |  |  |  |  |  |  |  |  |  |  |  |  |  |  |  |  |  |  |  | *aqp-1* | AQuaPorin or aquaglyceroporin related |
|  |  |  |  |  |  |  |  |  |  |  |  |  |  |  |  |  |  |  |  |  |  |  |  |  |  |  |  |  |  |  |  |  |  |  |  |  |  | LLC1.2 |  |
|  |  |  |  |  |  |  |  |  |  |  |  |  |  |  |  |  |  |  |  |  |  |  |  |  |  |  |  |  |  |  |  |  |  |  |  |  |  | *zig-9* | 2 (Zwei) IG domain protein |
|  |  |  |  |  |  |  |  |  |  |  |  |  |  |  |  |  |  |  |  |  |  |  |  |  |  |  |  |  |  |  |  |  |  |  |  |  |  | *cdr-2* | CaDmium Responsive |
|  |  |  |  |  |  |  |  |  |  |  |  |  |  |  |  |  |  |  |  |  |  |  |  |  |  |  |  |  |  |  |  |  |  |  |  |  |  | C17F4.7 |  |
|  |  |  |  |  |  |  |  |  |  |  |  |  |  |  |  |  |  |  |  |  |  |  |  |  |  |  |  |  |  |  |  |  |  |  |  |  |  | *clec-83* | C-type LECtin |
|  |  |  |  |  |  |  |  |  |  |  |  |  |  |  |  |  |  |  |  |  |  |  |  |  |  |  |  |  |  |  |  |  |  |  |  |  |  | F56D5.6 |  |
|  |  |  |  |  |  |  |  |  |  |  |  |  |  |  |  |  |  |  |  |  |  |  |  |  |  |  |  |  |  |  |  |  |  |  |  |  |  | *acs-7* | fatty Acid CoA Synthetase family |
|  |  |  |  |  |  |  |  |  |  |  |  |  |  |  |  |  |  |  |  |  |  |  |  |  |  |  |  |  |  |  |  |  |  |  |  |  |  | K06G5.1 |  |
|  |  |  |  |  |  |  |  |  |  |  |  |  |  |  |  |  |  |  |  |  |  |  |  |  |  |  |  |  |  |  |  |  |  |  |  |  |  | C39H7.4 |  |
|  |  |  |  |  |  |  |  |  |  |  |  |  |  |  |  |  |  |  |  |  |  |  |  |  |  |  |  |  |  |  |  |  |  |  |  |  |  | T25C12.3 |  |
|  |  |  |  |  |  |  |  |  |  |  |  |  |  |  |  |  |  |  |  |  |  |  |  |  |  |  |  |  |  |  |  |  |  |  |  |  |  | *pho-1* | intestinal acid PHOsphatase |
|  |  |  |  |  |  |  |  |  |  |  |  |  |  |  |  |  |  |  |  |  |  |  |  |  |  |  |  |  |  |  |  |  |  |  |  |  |  | K10C2.1 |  |
|  |  |  |  |  |  |  |  |  |  |  |  |  |  |  |  |  |  |  |  |  |  |  |  |  |  |  |  |  |  |  |  |  |  |  |  |  |  | *lbp-6* | Lipid Binding Protein |
|  |  |  |  |  |  |  |  |  |  |  |  |  |  |  |  |  |  |  |  |  |  |  |  |  |  |  |  |  |  |  |  |  |  |  |  |  |  | Y119D3B.21 |  |
|  |  |  |  |  |  |  |  |  |  |  |  |  |  |  |  |  |  |  |  |  |  |  |  |  |  |  |  |  |  |  |  |  |  |  |  |  |  | *mul-1* | Mucin Like Gene |
|  |  |  |  |  |  |  |  |  |  |  |  |  |  |  |  |  |  |  |  |  |  |  |  |  |  |  |  |  |  |  |  |  |  |  |  |  |  | C29F9.2 |  |
|  |  |  |  |  |  |  |  |  |  |  |  |  |  |  |  |  |  |  |  |  |  |  |  |  |  |  |  |  |  |  |  |  |  |  |  |  |  | C26B9.5 |  |
|  |  |  |  |  |  |  |  |  |  |  |  |  |  |  |  |  |  |  |  |  |  |  |  |  |  |  |  |  |  |  |  |  |  |  |  |  |  | *pbo-1* | PBOc defective (defecation) |
|  |  |  |  |  |  |  |  |  |  |  |  |  |  |  |  |  |  |  |  |  |  |  |  |  |  |  |  |  |  |  |  |  |  |  |  |  |  | Y42G9A.1 |  |
|  |  |  |  |  |  |  |  |  |  |  |  |  |  |  |  |  |  |  |  |  |  |  |  |  |  |  |  |  |  |  |  |  |  |  |  |  |  | T04F8.7 |  |
|  |  |  |  |  |  |  |  |  |  |  |  |  |  |  |  |  |  |  |  |  |  |  |  |  |  |  |  |  |  |  |  |  |  |  |  |  |  | Y51H4A.25 |  |
|  |  |  |  |  |  |  |  |  |  |  |  |  |  |  |  |  |  |  |  |  |  |  |  |  |  |  |  |  |  |  |  |  |  |  |  |  |  | B0272.4 |  |
|  |  |  |  |  |  |  |  |  |  |  |  |  |  |  |  |  |  |  |  |  |  |  |  |  |  |  |  |  |  |  |  |  |  |  |  |  |  | F18E2.1 |  |
|  |  |  |  |  |  |  |  |  |  |  |  |  |  |  |  |  |  |  |  |  |  |  |  |  |  |  |  |  |  |  |  |  |  |  |  |  |  | *pmp-1* | Peroxisomal Membrane Protein related |
|  |  |  |  |  |  |  |  |  |  |  |  |  |  |  |  |  |  |  |  |  |  |  |  |  |  |  |  |  |  |  |  |  |  |  |  |  |  | *oac-59* | O-ACyltransferase homolog |
|  |  |  |  |  |  |  |  |  |  |  |  |  |  |  |  |  |  |  |  |  |  |  |  |  |  |  |  |  |  |  |  |  |  |  |  |  |  | R07B5.2 |  |
|  |  |  |  |  |  |  |  |  |  |  |  |  |  |  |  |  |  |  |  |  |  |  |  |  |  |  |  |  |  |  |  |  |  |  |  |  |  | H34I24.2 |  |
|  |  |  |  |  |  |  |  |  |  |  |  |  |  |  |  |  |  |  |  |  |  |  |  |  |  |  |  |  |  |  |  |  |  |  |  |  |  | *ugt-33* | UDP-GlucuronosylTransferase |
|  |  |  |  |  |  |  |  |  |  |  |  |  |  |  |  |  |  |  |  |  |  |  |  |  |  |  |  |  |  |  |  |  |  |  |  |  |  | *ifd-2* | Intermediate Filament, D |
|  |  |  |  |  |  |  |  |  |  |  |  |  |  |  |  |  |  |  |  |  |  |  |  |  |  |  |  |  |  |  |  |  |  |  |  |  |  | C55A6.7 |  |
|  |  |  |  |  |  |  |  |  |  |  |  |  |  |  |  |  |  |  |  |  |  |  |  |  |  |  |  |  |  |  |  |  |  |  |  |  |  | *clec-227* | C-type LECtin |
|  |  |  |  |  |  |  |  |  |  |  |  |  |  |  |  |  |  |  |  |  |  |  |  |  |  |  |  |  |  |  |  |  |  |  |  |  |  | F57C12.2 |  |
|  |  |  |  |  |  |  |  |  |  |  |  |  |  |  |  |  |  |  |  |  |  |  |  |  |  |  |  |  |  |  |  |  |  |  |  |  |  | *haf-9* | HAlF transporter (PGP related) |
|  |  |  |  |  |  |  |  |  |  |  |  |  |  |  |  |  |  |  |  |  |  |  |  |  |  |  |  |  |  |  |  |  |  |  |  |  |  | *pho-13* | intestinal acid PHOsphatase |
|  |  |  |  |  |  |  |  |  |  |  |  |  |  |  |  |  |  |  |  |  |  |  |  |  |  |  |  |  |  |  |  |  |  |  |  |  |  | K12H4.7 |  |
|  |  |  |  |  |  |  |  |  |  |  |  |  |  |  |  |  |  |  |  |  |  |  |  |  |  |  |  |  |  |  |  |  |  |  |  |  |  | F32A5.3 |  |
|  |  |  |  |  |  |  |  |  |  |  |  |  |  |  |  |  |  |  |  |  |  |  |  |  |  |  |  |  |  |  |  |  |  |  |  |  |  | F28B4.3 |  |
|  |  |  |  |  |  |  |  |  |  |  |  |  |  |  |  |  |  |  |  |  |  |  |  |  |  |  |  |  |  |  |  |  |  |  |  |  |  | C47F8.7 |  |
|  |  |  |  |  |  |  |  |  |  |  |  |  |  |  |  |  |  |  |  |  |  |  |  |  |  |  |  |  |  |  |  |  |  |  |  |  |  | Y51F10.7 |  |
|  |  |  |  |  |  |  |  |  |  |  |  |  |  |  |  |  |  |  |  |  |  |  |  |  |  |  |  |  |  |  |  |  |  |  |  |  |  | *lys-2* | LYSozyme |
|  |  |  |  |  |  |  |  |  |  |  |  |  |  |  |  |  |  |  |  |  |  |  |  |  |  |  |  |  |  |  |  |  |  |  |  |  |  | Y82E9BR.21 |  |
|  |  |  |  |  |  |  |  |  |  |  |  |  |  |  |  |  |  |  |  |  |  |  |  |  |  |  |  |  |  |  |  |  |  |  |  |  |  | F38B6.4 |  |
|  |  |  |  |  |  |  |  |  |  |  |  |  |  |  |  |  |  |  |  |  |  |  |  |  |  |  |  |  |  |  |  |  |  |  |  |  |  | H34I24.3 |  |
|  |  |  |  |  |  |  |  |  |  |  |  |  |  |  |  |  |  |  |  |  |  |  |  |  |  |  |  |  |  |  |  |  |  |  |  |  |  | C08B6.11 |  |
|  |  |  |  |  |  |  |  |  |  |  |  |  |  |  |  |  |  |  |  |  |  |  |  |  |  |  |  |  |  |  |  |  |  |  |  |  |  | *math-21* | MATH (meprin-associated Traf homology) domain containing |
|  |  |  |  |  |  |  |  |  |  |  |  |  |  |  |  |  |  |  |  |  |  |  |  |  |  |  |  |  |  |  |  |  |  |  |  |  |  | *acs-5* | fatty Acid CoA Synthetase family |
|  |  |  |  |  |  |  |  |  |  |  |  |  |  |  |  |  |  |  |  |  |  |  |  |  |  |  |  |  |  |  |  |  |  |  |  |  |  | K12B6.9 |  |
|  |  |  |  |  |  |  |  |  |  |  |  |  |  |  |  |  |  |  |  |  |  |  |  |  |  |  |  |  |  |  |  |  |  |  |  |  |  | R08E3.1 |  |
|  |  |  |  |  |  |  |  |  |  |  |  |  |  |  |  |  |  |  |  |  |  |  |  |  |  |  |  |  |  |  |  |  |  |  |  |  |  | *haf-4* | HAlF transporter (PGP related) |
|  |  |  |  |  |  |  |  |  |  |  |  |  |  |  |  |  |  |  |  |  |  |  |  |  |  |  |  |  |  |  |  |  |  |  |  |  |  | *scav-6* | SCAVenger receptor (CD36 family) related |
|  |  |  |  |  |  |  |  |  |  |  |  |  |  |  |  |  |  |  |  |  |  |  |  |  |  |  |  |  |  |  |  |  |  |  |  |  |  | *gst-13* | Glutathione S-Transferase |
|  |  |  |  |  |  |  |  |  |  |  |  |  |  |  |  |  |  |  |  |  |  |  |  |  |  |  |  |  |  |  |  |  |  |  |  |  |  | *hpo-34* | Hypersensitive to POre-forming toxin |
|  |  |  |  |  |  |  |  |  |  |  |  |  |  |  |  |  |  |  |  |  |  |  |  |  |  |  |  |  |  |  |  |  |  |  |  |  |  | R12C12.1 |  |
|  |  |  |  |  |  |  |  |  |  |  |  |  |  |  |  |  |  |  |  |  |  |  |  |  |  |  |  |  |  |  |  |  |  |  |  |  |  | C29F9.3 |  |
|  |  |  |  |  |  |  |  |  |  |  |  |  |  |  |  |  |  |  |  |  |  |  |  |  |  |  |  |  |  |  |  |  |  |  |  |  |  | F01D5.5 |  |
|  |  |  |  |  |  |  |  |  |  |  |  |  |  |  |  |  |  |  |  |  |  |  |  |  |  |  |  |  |  |  |  |  |  |  |  |  |  | Y16B4A.2 |  |
|  |  |  |  |  |  |  |  |  |  |  |  |  |  |  |  |  |  |  |  |  |  |  |  |  |  |  |  |  |  |  |  |  |  |  |  |  |  | *ugt-46* | UDP-GlucuronosylTransferase |
|  |  |  |  |  |  |  |  |  |  |  |  |  |  |  |  |  |  |  |  |  |  |  |  |  |  |  |  |  |  |  |  |  |  |  |  |  |  | T01B11.2 |  |
|  |  |  |  |  |  |  |  |  |  |  |  |  |  |  |  |  |  |  |  |  |  |  |  |  |  |  |  |  |  |  |  |  |  |  |  |  |  | T24C2.5 |  |
|  |  |  |  |  |  |  |  |  |  |  |  |  |  |  |  |  |  |  |  |  |  |  |  |  |  |  |  |  |  |  |  |  |  |  |  |  |  | C36B1.6 |  |
|  |  |  |  |  |  |  |  |  |  |  |  |  |  |  |  |  |  |  |  |  |  |  |  |  |  |  |  |  |  |  |  |  |  |  |  |  |  | F20B6.1 |  |
|  |  |  |  |  |  |  |  |  |  |  |  |  |  |  |  |  |  |  |  |  |  |  |  |  |  |  |  |  |  |  |  |  |  |  |  |  |  | *plc-2* | PhosphoLipase C |
|  |  |  |  |  |  |  |  |  |  |  |  |  |  |  |  |  |  |  |  |  |  |  |  |  |  |  |  |  |  |  |  |  |  |  |  |  |  | F21D5.3 |  |
|  |  |  |  |  |  |  |  |  |  |  |  |  |  |  |  |  |  |  |  |  |  |  |  |  |  |  |  |  |  |  |  |  |  |  |  |  |  | *nhr-8* | Nuclear Hormone Receptor family |
|  |  |  |  |  |  |  |  |  |  |  |  |  |  |  |  |  |  |  |  |  |  |  |  |  |  |  |  |  |  |  |  |  |  |  |  |  |  | *klo-1* | KLOtho (mammalian aging-associated protein) homolog |
|  |  |  |  |  |  |  |  |  |  |  |  |  |  |  |  |  |  |  |  |  |  |  |  |  |  |  |  |  |  |  |  |  |  |  |  |  |  | *fbxa-19* | F-box A protein |
|  |  |  |  |  |  |  |  |  |  |  |  |  |  |  |  |  |  |  |  |  |  |  |  |  |  |  |  |  |  |  |  |  |  |  |  |  |  | *gly-19* | GLYcosylation related |
|  |  |  |  |  |  |  |  |  |  |  |  |  |  |  |  |  |  |  |  |  |  |  |  |  |  |  |  |  |  |  |  |  |  |  |  |  |  | *ads-1* | Alkyl-Dihydroxyacetonephosphate Synthase |
|  |  |  |  |  |  |  |  |  |  |  |  |  |  |  |  |  |  |  |  |  |  |  |  |  |  |  |  |  |  |  |  |  |  |  |  |  |  | *ifd-1* | Intermediate Filament, D |
|  |  |  |  |  |  |  |  |  |  |  |  |  |  |  |  |  |  |  |  |  |  |  |  |  |  |  |  |  |  |  |  |  |  |  |  |  |  | T16G12.1 |  |
|  |  |  |  |  |  |  |  |  |  |  |  |  |  |  |  |  |  |  |  |  |  |  |  |  |  |  |  |  |  |  |  |  |  |  |  |  |  | K08D8.3 |  |
|  |  |  |  |  |  |  |  |  |  |  |  |  |  |  |  |  |  |  |  |  |  |  |  |  |  |  |  |  |  |  |  |  |  |  |  |  |  | F10C1.8 |  |
|  |  |  |  |  |  |  |  |  |  |  |  |  |  |  |  |  |  |  |  |  |  |  |  |  |  |  |  |  |  |  |  |  |  |  |  |  |  | *ugt-22* | UDP-GlucuronosylTransferase |
|  |  |  |  |  |  |  |  |  |  |  |  |  |  |  |  |  |  |  |  |  |  |  |  |  |  |  |  |  |  |  |  |  |  |  |  |  |  | *ifp-1* | Intermediate Filament Protein, class E |
|  |  |  |  |  |  |  |  |  |  |  |  |  |  |  |  |  |  |  |  |  |  |  |  |  |  |  |  |  |  |  |  |  |  |  |  |  |  | T19D12.4 |  |
|  |  |  |  |  |  |  |  |  |  |  |  |  |  |  |  |  |  |  |  |  |  |  |  |  |  |  |  |  |  |  |  |  |  |  |  |  |  | *pept-1* | PEPTide transporter family |
|  |  |  |  |  |  |  |  |  |  |  |  |  |  |  |  |  |  |  |  |  |  |  |  |  |  |  |  |  |  |  |  |  |  |  |  |  |  | *npa-1* | Nematode Polyprotein Allergen related |
|  |  |  |  |  |  |  |  |  |  |  |  |  |  |  |  |  |  |  |  |  |  |  |  |  |  |  |  |  |  |  |  |  |  |  |  |  |  | *nep-22* | NEPrilysin metallopeptidase family |
|  |  |  |  |  |  |  |  |  |  |  |  |  |  |  |  |  |  |  |  |  |  |  |  |  |  |  |  |  |  |  |  |  |  |  |  |  |  | K11H12.11 |  |
|  |  |  |  |  |  |  |  |  |  |  |  |  |  |  |  |  |  |  |  |  |  |  |  |  |  |  |  |  |  |  |  |  |  |  |  |  |  | *lys-1* | LYSozyme |
|  |  |  |  |  |  |  |  |  |  |  |  |  |  |  |  |  |  |  |  |  |  |  |  |  |  |  |  |  |  |  |  |  |  |  |  |  |  | *inx-15* | INneXin |
|  |  |  |  |  |  |  |  |  |  |  |  |  |  |  |  |  |  |  |  |  |  |  |  |  |  |  |  |  |  |  |  |  |  |  |  |  |  | *inx-17* | INneXin |
|  |  |  |  |  |  |  |  |  |  |  |  |  |  |  |  |  |  |  |  |  |  |  |  |  |  |  |  |  |  |  |  |  |  |  |  |  |  | K10B2.2 |  |
|  |  |  |  |  |  |  |  |  |  |  |  |  |  |  |  |  |  |  |  |  |  |  |  |  |  |  |  |  |  |  |  |  |  |  |  |  |  | *cav-2* | CAVeolin |
|  |  |  |  |  |  |  |  |  |  |  |  |  |  |  |  |  |  |  |  |  |  |  |  |  |  |  |  |  |  |  |  |  |  |  |  |  |  | *dhs-18* | DeHydrogenases, Short chain |
|  |  |  |  |  |  |  |  |  |  |  |  |  |  |  |  |  |  |  |  |  |  |  |  |  |  |  |  |  |  |  |  |  |  |  |  |  |  | R07E3.1 |  |
|  |  |  |  |  |  |  |  |  |  |  |  |  |  |  |  |  |  |  |  |  |  |  |  |  |  |  |  |  |  |  |  |  |  |  |  |  |  | ZC204.12 |  |
|  |  |  |  |  |  |  |  |  |  |  |  |  |  |  |  |  |  |  |  |  |  |  |  |  |  |  |  |  |  |  |  |  |  |  |  |  |  | *cpz-2* | CathePsin Z |
|  |  |  |  |  |  |  |  |  |  |  |  |  |  |  |  |  |  |  |  |  |  |  |  |  |  |  |  |  |  |  |  |  |  |  |  |  |  | *clec-42* | C-type LECtin |
|  |  |  |  |  |  |  |  |  |  |  |  |  |  |  |  |  |  |  |  |  |  |  |  |  |  |  |  |  |  |  |  |  |  |  |  |  |  | *ugt-48* | UDP-GlucuronosylTransferase |
|  |  |  |  |  |  |  |  |  |  |  |  |  |  |  |  |  |  |  |  |  |  |  |  |  |  |  |  |  |  |  |  |  |  |  |  |  |  | C01B10.10 |  |
|  |  |  |  |  |  |  |  |  |  |  |  |  |  |  |  |  |  |  |  |  |  |  |  |  |  |  |  |  |  |  |  |  |  |  |  |  |  | *kqt-2* | potassium channel, KvQLT family |
|  |  |  |  |  |  |  |  |  |  |  |  |  |  |  |  |  |  |  |  |  |  |  |  |  |  |  |  |  |  |  |  |  |  |  |  |  |  | Y46D2A.5 |  |
|  |  |  |  |  |  |  |  |  |  |  |  |  |  |  |  |  |  |  |  |  |  |  |  |  |  |  |  |  |  |  |  |  |  |  |  |  |  | Y46D2A.2 |  |
|  |  |  |  |  |  |  |  |  |  |  |  |  |  |  |  |  |  |  |  |  |  |  |  |  |  |  |  |  |  |  |  |  |  |  |  |  |  | *tsp-1* | TetraSPanin family |
|  |  |  |  |  |  |  |  |  |  |  |  |  |  |  |  |  |  |  |  |  |  |  |  |  |  |  |  |  |  |  |  |  |  |  |  |  |  | *ugt-12* | UDP-GlucuronosylTransferase |
|  |  |  |  |  |  |  |  |  |  |  |  |  |  |  |  |  |  |  |  |  |  |  |  |  |  |  |  |  |  |  |  |  |  |  |  |  |  | T25D10.1 |  |
|  |  |  |  |  |  |  |  |  |  |  |  |  |  |  |  |  |  |  |  |  |  |  |  |  |  |  |  |  |  |  |  |  |  |  |  |  |  | C16D9.4 |  |
|  |  |  |  |  |  |  |  |  |  |  |  |  |  |  |  |  |  |  |  |  |  |  |  |  |  |  |  |  |  |  |  |  |  |  |  |  |  | *clec-165* | C-type LECtin |
|  |  |  |  |  |  |  |  |  |  |  |  |  |  |  |  |  |  |  |  |  |  |  |  |  |  |  |  |  |  |  |  |  |  |  |  |  |  | Y41D4B.17 |  |
|  |  |  |  |  |  |  |  |  |  |  |  |  |  |  |  |  |  |  |  |  |  |  |  |  |  |  |  |  |  |  |  |  |  |  |  |  |  | *fbxa-6* | F-box A protein |
|  |  |  |  |  |  |  |  |  |  |  |  |  |  |  |  |  |  |  |  |  |  |  |  |  |  |  |  |  |  |  |  |  |  |  |  |  |  | *catp-5* | Cation transporting ATPase |
|  |  |  |  |  |  |  |  |  |  |  |  |  |  |  |  |  |  |  |  |  |  |  |  |  |  |  |  |  |  |  |  |  |  |  |  |  |  | F08G2.5 |  |
|  |  |  |  |  |  |  |  |  |  |  |  |  |  |  |  |  |  |  |  |  |  |  |  |  |  |  |  |  |  |  |  |  |  |  |  |  |  | C44F1.1 |  |
|  |  |  |  |  |  |  |  |  |  |  |  |  |  |  |  |  |  |  |  |  |  |  |  |  |  |  |  |  |  |  |  |  |  |  |  |  |  | F14B6.6 |  |
|  |  |  |  |  |  |  |  |  |  |  |  |  |  |  |  |  |  |  |  |  |  |  |  |  |  |  |  |  |  |  |  |  |  |  |  |  |  | *glc-1* | Glutamate-gated ChLoride channel |
|  |  |  |  |  |  |  |  |  |  |  |  |  |  |  |  |  |  |  |  |  |  |  |  |  |  |  |  |  |  |  |  |  |  |  |  |  |  | F58F9.3 |  |
|  |  |  |  |  |  |  |  |  |  |  |  |  |  |  |  |  |  |  |  |  |  |  |  |  |  |  |  |  |  |  |  |  |  |  |  |  |  | C52D10.1 |  |
|  |  |  |  |  |  |  |  |  |  |  |  |  |  |  |  |  |  |  |  |  |  |  |  |  |  |  |  |  |  |  |  |  |  |  |  |  |  | *ges-1* | abnormal Gut ESterase |
|  |  |  |  |  |  |  |  |  |  |  |  |  |  |  |  |  |  |  |  |  |  |  |  |  |  |  |  |  |  |  |  |  |  |  |  |  |  | *math-24* | MATH (meprin-associated Traf homology) domain containing |
|  |  |  |  |  |  |  |  |  |  |  |  |  |  |  |  |  |  |  |  |  |  |  |  |  |  |  |  |  |  |  |  |  |  |  |  |  |  | *ugt-4* | UDP-GlucuronosylTransferase |
|  |  |  |  |  |  |  |  |  |  |  |  |  |  |  |  |  |  |  |  |  |  |  |  |  |  |  |  |  |  |  |  |  |  |  |  |  |  | *prg-1* | Piwi (fruitfly) Related Gene |
|  |  |  |  |  |  |  |  |  |  |  |  |  |  |  |  |  |  |  |  |  |  |  |  |  |  |  |  |  |  |  |  |  |  |  |  |  |  | *nhr-80* | Nuclear Hormone Receptor family |
|  |  |  |  |  |  |  |  |  |  |  |  |  |  |  |  |  |  |  |  |  |  |  |  |  |  |  |  |  |  |  |  |  |  |  |  |  |  | H43E16.1 |  |
|  |  |  |  |  |  |  |  |  |  |  |  |  |  |  |  |  |  |  |  |  |  |  |  |  |  |  |  |  |  |  |  |  |  |  |  |  |  | *clec-56* | C-type LECtin |
|  |  |  |  |  |  |  |  |  |  |  |  |  |  |  |  |  |  |  |  |  |  |  |  |  |  |  |  |  |  |  |  |  |  |  |  |  |  | *fut-2* | FUcosyl Transferase |
|  |  |  |  |  |  |  |  |  |  |  |  |  |  |  |  |  |  |  |  |  |  |  |  |  |  |  |  |  |  |  |  |  |  |  |  |  |  | *ppt-1* | Palmitoyl Protein Thioesterase |
|  |  |  |  |  |  |  |  |  |  |  |  |  |  |  |  |  |  |  |  |  |  |  |  |  |  |  |  |  |  |  |  |  |  |  |  |  |  | T07D3.4 |  |
|  |  |  |  |  |  |  |  |  |  |  |  |  |  |  |  |  |  |  |  |  |  |  |  |  |  |  |  |  |  |  |  |  |  |  |  |  |  | C17E7.13 |  |
|  |  |  |  |  |  |  |  |  |  |  |  |  |  |  |  |  |  |  |  |  |  |  |  |  |  |  |  |  |  |  |  |  |  |  |  |  |  | *alh-5* | ALdehyde deHydrogenase |
|  |  |  |  |  |  |  |  |  |  |  |  |  |  |  |  |  |  |  |  |  |  |  |  |  |  |  |  |  |  |  |  |  |  |  |  |  |  | *cht-4* | CHiTinase |
|  |  |  |  |  |  |  |  |  |  |  |  |  |  |  |  |  |  |  |  |  |  |  |  |  |  |  |  |  |  |  |  |  |  |  |  |  |  | Y43F8C.13 |  |
|  |  |  |  |  |  |  |  |  |  |  |  |  |  |  |  |  |  |  |  |  |  |  |  |  |  |  |  |  |  |  |  |  |  |  |  |  |  | *cpz-1* | CathePsin Z |
|  |  |  |  |  |  |  |  |  |  |  |  |  |  |  |  |  |  |  |  |  |  |  |  |  |  |  |  |  |  |  |  |  |  |  |  |  |  | K02D7.1 |  |
|  |  |  |  |  |  |  |  |  |  |  |  |  |  |  |  |  |  |  |  |  |  |  |  |  |  |  |  |  |  |  |  |  |  |  |  |  |  | T01D3.6 |  |
|  |  |  |  |  |  |  |  |  |  |  |  |  |  |  |  |  |  |  |  |  |  |  |  |  |  |  |  |  |  |  |  |  |  |  |  |  |  | T28H10.3 |  |
|  |  |  |  |  |  |  |  |  |  |  |  |  |  |  |  |  |  |  |  |  |  |  |  |  |  |  |  |  |  |  |  |  |  |  |  |  |  | *cpr-6* | Cysteine PRotease related |
|  |  |  |  |  |  |  |  |  |  |  |  |  |  |  |  |  |  |  |  |  |  |  |  |  |  |  |  |  |  |  |  |  |  |  |  |  |  | *pgrn-1* | ProGRaNulin homolog |
|  |  |  |  |  |  |  |  |  |  |  |  |  |  |  |  |  |  |  |  |  |  |  |  |  |  |  |  |  |  |  |  |  |  |  |  |  |  | *elt-2* | Erythroid-Like Transcription factor family |
|  |  |  |  |  |  |  |  |  |  |  |  |  |  |  |  |  |  |  |  |  |  |  |  |  |  |  |  |  |  |  |  |  |  |  |  |  |  | *cdf-2* | Cation Diffusion Facilitator family |
|  |  |  |  |  |  |  |  |  |  |  |  |  |  |  |  |  |  |  |  |  |  |  |  |  |  |  |  |  |  |  |  |  |  |  |  |  |  | *smd-1* | SAM Decarboxylase |
|  |  |  |  |  |  |  |  |  |  |  |  |  |  |  |  |  |  |  |  |  |  |  |  |  |  |  |  |  |  |  |  |  |  |  |  |  |  | C44H9.7 |  |
|  |  |  |  |  |  |  |  |  |  |  |  |  |  |  |  |  |  |  |  |  |  |  |  |  |  |  |  |  |  |  |  |  |  |  |  |  |  | *ttr-50* | TransThyretin-Related family domain |
|  |  |  |  |  |  |  |  |  |  |  |  |  |  |  |  |  |  |  |  |  |  |  |  |  |  |  |  |  |  |  |  |  |  |  |  |  |  | *pes-9* | Patterned Expression Site |
|  |  |  |  |  |  |  |  |  |  |  |  |  |  |  |  |  |  |  |  |  |  |  |  |  |  |  |  |  |  |  |  |  |  |  |  |  |  | T07D3.5 |  |
|  |  |  |  |  |  |  |  |  |  |  |  |  |  |  |  |  |  |  |  |  |  |  |  |  |  |  |  |  |  |  |  |  |  |  |  |  |  | F39E9.22 |  |
|  |  |  |  |  |  |  |  |  |  |  |  |  |  |  |  |  |  |  |  |  |  |  |  |  |  |  |  |  |  |  |  |  |  |  |  |  |  | C29F7.2 |  |
|  |  |  |  |  |  |  |  |  |  |  |  |  |  |  |  |  |  |  |  |  |  |  |  |  |  |  |  |  |  |  |  |  |  |  |  |  |  | *ugt-51* | UDP-GlucuronosylTransferase |
|  |  |  |  |  |  |  |  |  |  |  |  |  |  |  |  |  |  |  |  |  |  |  |  |  |  |  |  |  |  |  |  |  |  |  |  |  |  | W05H9.1 |  |
|  |  |  |  |  |  |  |  |  |  |  |  |  |  |  |  |  |  |  |  |  |  |  |  |  |  |  |  |  |  |  |  |  |  |  |  |  |  | B0286.3 |  |
|  |  |  |  |  |  |  |  |  |  |  |  |  |  |  |  |  |  |  |  |  |  |  |  |  |  |  |  |  |  |  |  |  |  |  |  |  |  | *scav-1* | SCAVenger receptor (CD36 family) related |
|  |  |  |  |  |  |  |  |  |  |  |  |  |  |  |  |  |  |  |  |  |  |  |  |  |  |  |  |  |  |  |  |  |  |  |  |  |  | *sgk-1* | Serum- and Glucocorticoid- inducible Kinase homolog |
|  |  |  |  |  |  |  |  |  |  |  |  |  |  |  |  |  |  |  |  |  |  |  |  |  |  |  |  |  |  |  |  |  |  |  |  |  |  | D1044.1 |  |
|  |  |  |  |  |  |  |  |  |  |  |  |  |  |  |  |  |  |  |  |  |  |  |  |  |  |  |  |  |  |  |  |  |  |  |  |  |  | B0462.1 |  |
|  |  |  |  |  |  |  |  |  |  |  |  |  |  |  |  |  |  |  |  |  |  |  |  |  |  |  |  |  |  |  |  |  |  |  |  |  |  | *zip-12* | bZIP transcription factor family |
|  |  |  |  |  |  |  |  |  |  |  |  |  |  |  |  |  |  |  |  |  |  |  |  |  |  |  |  |  |  |  |  |  |  |  |  |  |  | M153.2 |  |
|  |  |  |  |  |  |  |  |  |  |  |  |  |  |  |  |  |  |  |  |  |  |  |  |  |  |  |  |  |  |  |  |  |  |  |  |  |  | T03A1.5 |  |
|  |  |  |  |  |  |  |  |  |  |  |  |  |  |  |  |  |  |  |  |  |  |  |  |  |  |  |  |  |  |  |  |  |  |  |  |  |  | *spp-20* | SaPosin-like Protein family |
|  |  |  |  |  |  |  |  |  |  |  |  |  |  |  |  |  |  |  |  |  |  |  |  |  |  |  |  |  |  |  |  |  |  |  |  |  |  | C18A3.10 |  |
|  |  |  |  |  |  |  |  |  |  |  |  |  |  |  |  |  |  |  |  |  |  |  |  |  |  |  |  |  |  |  |  |  |  |  |  |  |  | *clec-49* | C-type LECtin |
|  |  |  |  |  |  |  |  |  |  |  |  |  |  |  |  |  |  |  |  |  |  |  |  |  |  |  |  |  |  |  |  |  |  |  |  |  |  | *ugt-47* | UDP-GlucuronosylTransferase |
|  |  |  |  |  |  |  |  |  |  |  |  |  |  |  |  |  |  |  |  |  |  |  |  |  |  |  |  |  |  |  |  |  |  |  |  |  |  | H10E21.1 |  |
|  |  |  |  |  |  |  |  |  |  |  |  |  |  |  |  |  |  |  |  |  |  |  |  |  |  |  |  |  |  |  |  |  |  |  |  |  |  | *ent-6* | Equilibrative Nucleoside Transporter |
|  |  |  |  |  |  |  |  |  |  |  |  |  |  |  |  |  |  |  |  |  |  |  |  |  |  |  |  |  |  |  |  |  |  |  |  |  |  | *asp-13* | ASpartyl Protease |
|  |  |  |  |  |  |  |  |  |  |  |  |  |  |  |  |  |  |  |  |  |  |  |  |  |  |  |  |  |  |  |  |  |  |  |  |  |  | *srr-6* | Serpentine Receptor, class R |
|  |  |  |  |  |  |  |  |  |  |  |  |  |  |  |  |  |  |  |  |  |  |  |  |  |  |  |  |  |  |  |  |  |  |  |  |  |  | K08D8.6 |  |
|  |  |  |  |  |  |  |  |  |  |  |  |  |  |  |  |  |  |  |  |  |  |  |  |  |  |  |  |  |  |  |  |  |  |  |  |  |  | *clc-1* | CLaudin-like in Caenorhabditis |
|  |  |  |  |  |  |  |  |  |  |  |  |  |  |  |  |  |  |  |  |  |  |  |  |  |  |  |  |  |  |  |  |  |  |  |  |  |  | R01B10.3 |  |
|  |  |  |  |  |  |  |  |  |  |  |  |  |  |  |  |  |  |  |  |  |  |  |  |  |  |  |  |  |  |  |  |  |  |  |  |  |  | *srv-1* | Serpentine Receptor, class V |
|  |  |  |  |  |  |  |  |  |  |  |  |  |  |  |  |  |  |  |  |  |  |  |  |  |  |  |  |  |  |  |  |  |  |  |  |  |  | *fbxa-105* | F-box A protein |
|  |  |  |  |  |  |  |  |  |  |  |  |  |  |  |  |  |  |  |  |  |  |  |  |  |  |  |  |  |  |  |  |  |  |  |  |  |  | K08D12.6 |  |
|  |  |  |  |  |  |  |  |  |  |  |  |  |  |  |  |  |  |  |  |  |  |  |  |  |  |  |  |  |  |  |  |  |  |  |  |  |  | *spp-1* | SaPosin-like Protein family |
|  |  |  |  |  |  |  |  |  |  |  |  |  |  |  |  |  |  |  |  |  |  |  |  |  |  |  |  |  |  |  |  |  |  |  |  |  |  | Y49E10.18 |  |
|  |  |  |  |  |  |  |  |  |  |  |  |  |  |  |  |  |  |  |  |  |  |  |  |  |  |  |  |  |  |  |  |  |  |  |  |  |  | *flp-23* | FMRF-Like Peptide |
|  |  |  |  |  |  |  |  |  |  |  |  |  |  |  |  |  |  |  |  |  |  |  |  |  |  |  |  |  |  |  |  |  |  |  |  |  |  | *cpr-3* | Cysteine PRotease related |
|  |  |  |  |  |  |  |  |  |  |  |  |  |  |  |  |  |  |  |  |  |  |  |  |  |  |  |  |  |  |  |  |  |  |  |  |  |  | *sqst-2* | SeQueSTosome related |
|  |  |  |  |  |  |  |  |  |  |  |  |  |  |  |  |  |  |  |  |  |  |  |  |  |  |  |  |  |  |  |  |  |  |  |  |  |  | *nhr-177* | Nuclear Hormone Receptor family |
|  |  |  |  |  |  |  |  |  |  |  |  |  |  |  |  |  |  |  |  |  |  |  |  |  |  |  |  |  |  |  |  |  |  |  |  |  |  | *fbxa-219* | F-box A protein |
|  |  |  |  |  |  |  |  |  |  |  |  |  |  |  |  |  |  |  |  |  |  |  |  |  |  |  |  |  |  |  |  |  |  |  |  |  |  | ZK6.11 |  |
|  |  |  |  |  |  |  |  |  |  |  |  |  |  |  |  |  |  |  |  |  |  |  |  |  |  |  |  |  |  |  |  |  |  |  |  |  |  | *math-20* | MATH (meprin-associated Traf homology) domain containing |
|  |  |  |  |  |  |  |  |  |  |  |  |  |  |  |  |  |  |  |  |  |  |  |  |  |  |  |  |  |  |  |  |  |  |  |  |  |  | F35E12.10 |  |
|  |  |  |  |  |  |  |  |  |  |  |  |  |  |  |  |  |  |  |  |  |  |  |  |  |  |  |  |  |  |  |  |  |  |  |  |  |  | F40F12.7 |  |
|  |  |  |  |  |  |  |  |  |  |  |  |  |  |  |  |  |  |  |  |  |  |  |  |  |  |  |  |  |  |  |  |  |  |  |  |  |  | K12C11.6 |  |
|  |  |  |  |  |  |  |  |  |  |  |  |  |  |  |  |  |  |  |  |  |  |  |  |  |  |  |  |  |  |  |  |  |  |  |  |  |  | Y54G2A.45 |  |
|  |  |  |  |  |  |  |  |  |  |  |  |  |  |  |  |  |  |  |  |  |  |  |  |  |  |  |  |  |  |  |  |  |  |  |  |  |  | R10E8.1 |  |
|  |  |  |  |  |  |  |  |  |  |  |  |  |  |  |  |  |  |  |  |  |  |  |  |  |  |  |  |  |  |  |  |  |  |  |  |  |  | *sid-2* | Systemic RNA Interference Defective |
|  |  |  |  |  |  |  |  |  |  |  |  |  |  |  |  |  |  |  |  |  |  |  |  |  |  |  |  |  |  |  |  |  |  |  |  |  |  | R10E8.7 |  |
|  |  |  |  |  |  |  |  |  |  |  |  |  |  |  |  |  |  |  |  |  |  |  |  |  |  |  |  |  |  |  |  |  |  |  |  |  |  | T19D12.1 |  |
|  |  |  |  |  |  |  |  |  |  |  |  |  |  |  |  |  |  |  |  |  |  |  |  |  |  |  |  |  |  |  |  |  |  |  |  |  |  | *ttr-37* | TransThyretin-Related family domain |
|  |  |  |  |  |  |  |  |  |  |  |  |  |  |  |  |  |  |  |  |  |  |  |  |  |  |  |  |  |  |  |  |  |  |  |  |  |  | *ets-9* | ETS class transcription factor |
|  |  |  |  |  |  |  |  |  |  |  |  |  |  |  |  |  |  |  |  |  |  |  |  |  |  |  |  |  |  |  |  |  |  |  |  |  |  | Y43C5A.2 |  |
|  |  |  |  |  |  |  |  |  |  |  |  |  |  |  |  |  |  |  |  |  |  |  |  |  |  |  |  |  |  |  |  |  |  |  |  |  |  | C23H4.2 |  |
|  |  |  |  |  |  |  |  |  |  |  |  |  |  |  |  |  |  |  |  |  |  |  |  |  |  |  |  |  |  |  |  |  |  |  |  |  |  | *pmp-4* | Peroxisomal Membrane Protein related |
|  |  |  |  |  |  |  |  |  |  |  |  |  |  |  |  |  |  |  |  |  |  |  |  |  |  |  |  |  |  |  |  |  |  |  |  |  |  | C29F3.7 |  |
|  |  |  |  |  |  |  |  |  |  |  |  |  |  |  |  |  |  |  |  |  |  |  |  |  |  |  |  |  |  |  |  |  |  |  |  |  |  | F56F10.1 |  |
|  |  |  |  |  |  |  |  |  |  |  |  |  |  |  |  |  |  |  |  |  |  |  |  |  |  |  |  |  |  |  |  |  |  |  |  |  |  | K12C11.3 |  |
|  |  |  |  |  |  |  |  |  |  |  |  |  |  |  |  |  |  |  |  |  |  |  |  |  |  |  |  |  |  |  |  |  |  |  |  |  |  | F01D5.1 |  |
|  |  |  |  |  |  |  |  |  |  |  |  |  |  |  |  |  |  |  |  |  |  |  |  |  |  |  |  |  |  |  |  |  |  |  |  |  |  | *lec-11* | gaLECtin |
|  |  |  |  |  |  |  |  |  |  |  |  |  |  |  |  |  |  |  |  |  |  |  |  |  |  |  |  |  |  |  |  |  |  |  |  |  |  | Y14H12A.2 |  |
|  |  |  |  |  |  |  |  |  |  |  |  |  |  |  |  |  |  |  |  |  |  |  |  |  |  |  |  |  |  |  |  |  |  |  |  |  |  | *lipl-2* | LIPase Like |
|  |  |  |  |  |  |  |  |  |  |  |  |  |  |  |  |  |  |  |  |  |  |  |  |  |  |  |  |  |  |  |  |  |  |  |  |  |  | D1054.5 |  |
|  |  |  |  |  |  |  |  |  |  |  |  |  |  |  |  |  |  |  |  |  |  |  |  |  |  |  |  |  |  |  |  |  |  |  |  |  |  | *ugt-6* | UDP-GlucuronosylTransferase |
|  |  |  |  |  |  |  |  |  |  |  |  |  |  |  |  |  |  |  |  |  |  |  |  |  |  |  |  |  |  |  |  |  |  |  |  |  |  | C25F9.5 |  |
|  |  |  |  |  |  |  |  |  |  |  |  |  |  |  |  |  |  |  |  |  |  |  |  |  |  |  |  |  |  |  |  |  |  |  |  |  |  | K05B2.4 |  |
|  |  |  |  |  |  |  |  |  |  |  |  |  |  |  |  |  |  |  |  |  |  |  |  |  |  |  |  |  |  |  |  |  |  |  |  |  |  | Y40D12A.2 |  |
|  |  |  |  |  |  |  |  |  |  |  |  |  |  |  |  |  |  |  |  |  |  |  |  |  |  |  |  |  |  |  |  |  |  |  |  |  |  | K11H12.4 |  |
|  |  |  |  |  |  |  |  |  |  |  |  |  |  |  |  |  |  |  |  |  |  |  |  |  |  |  |  |  |  |  |  |  |  |  |  |  |  | F20G2.5 |  |
|  |  |  |  |  |  |  |  |  |  |  |  |  |  |  |  |  |  |  |  |  |  |  |  |  |  |  |  |  |  |  |  |  |  |  |  |  |  | K10G6.5 |  |
|  |  |  |  |  |  |  |  |  |  |  |  |  |  |  |  |  |  |  |  |  |  |  |  |  |  |  |  |  |  |  |  |  |  |  |  |  |  | *tag-234* | Temporarily Assigned Gene name |
|  |  |  |  |  |  |  |  |  |  |  |  |  |  |  |  |  |  |  |  |  |  |  |  |  |  |  |  |  |  |  |  |  |  |  |  |  |  | Y66H1A.5 |  |
|  |  |  |  |  |  |  |  |  |  |  |  |  |  |  |  |  |  |  |  |  |  |  |  |  |  |  |  |  |  |  |  |  |  |  |  |  |  | *cyp-37A1* | CYtochrome P450 family |
|  |  |  |  |  |  |  |  |  |  |  |  |  |  |  |  |  |  |  |  |  |  |  |  |  |  |  |  |  |  |  |  |  |  |  |  |  |  | B0035.13 |  |
|  |  |  |  |  |  |  |  |  |  |  |  |  |  |  |  |  |  |  |  |  |  |  |  |  |  |  |  |  |  |  |  |  |  |  |  |  |  | *nep-17* | NEPrilysin metallopeptidase family |
|  |  |  |  |  |  |  |  |  |  |  |  |  |  |  |  |  |  |  |  |  |  |  |  |  |  |  |  |  |  |  |  |  |  |  |  |  |  | *ifb-2* | Intermediate Filament, B |
|  |  |  |  |  |  |  |  |  |  |  |  |  |  |  |  |  |  |  |  |  |  |  |  |  |  |  |  |  |  |  |  |  |  |  |  |  |  | *ctl-2* | CaTaLase |
|  |  |  |  |  |  |  |  |  |  |  |  |  |  |  |  |  |  |  |  |  |  |  |  |  |  |  |  |  |  |  |  |  |  |  |  |  |  | *dct-18* | DAF-16/FOXO Controlled, germline Tumor affecting |
|  |  |  |  |  |  |  |  |  |  |  |  |  |  |  |  |  |  |  |  |  |  |  |  |  |  |  |  |  |  |  |  |  |  |  |  |  |  | *act-5* | ACTin |
|  |  |  |  |  |  |  |  |  |  |  |  |  |  |  |  |  |  |  |  |  |  |  |  |  |  |  |  |  |  |  |  |  |  |  |  |  |  | F57F4.4 |  |
|  |  |  |  |  |  |  |  |  |  |  |  |  |  |  |  |  |  |  |  |  |  |  |  |  |  |  |  |  |  |  |  |  |  |  |  |  |  | F40F4.6 |  |
|  |  |  |  |  |  |  |  |  |  |  |  |  |  |  |  |  |  |  |  |  |  |  |  |  |  |  |  |  |  |  |  |  |  |  |  |  |  | F59C6.11 |  |
|  |  |  |  |  |  |  |  |  |  |  |  |  |  |  |  |  |  |  |  |  |  |  |  |  |  |  |  |  |  |  |  |  |  |  |  |  |  | *fmo-1* | Flavin-containing MonoOxygenase family |
|  |  |  |  |  |  |  |  |  |  |  |  |  |  |  |  |  |  |  |  |  |  |  |  |  |  |  |  |  |  |  |  |  |  |  |  |  |  | F56C9.7 |  |
|  |  |  |  |  |  |  |  |  |  |  |  |  |  |  |  |  |  |  |  |  |  |  |  |  |  |  |  |  |  |  |  |  |  |  |  |  |  | *lec-10* | gaLECtin |
|  |  |  |  |  |  |  |  |  |  |  |  |  |  |  |  |  |  |  |  |  |  |  |  |  |  |  |  |  |  |  |  |  |  |  |  |  |  | *lec-9* | gaLECtin |
|  |  |  |  |  |  |  |  |  |  |  |  |  |  |  |  |  |  |  |  |  |  |  |  |  |  |  |  |  |  |  |  |  |  |  |  |  |  | *oac-14* | O-ACyltransferase homolog |
|  |  |  |  |  |  |  |  |  |  |  |  |  |  |  |  |  |  |  |  |  |  |  |  |  |  |  |  |  |  |  |  |  |  |  |  |  |  | F35C8.5 |  |
|  |  |  |  |  |  |  |  |  |  |  |  |  |  |  |  |  |  |  |  |  |  |  |  |  |  |  |  |  |  |  |  |  |  |  |  |  |  | *trx-3* | ThioRedoXin [see also xtr] |
|  |  |  |  |  |  |  |  |  |  |  |  |  |  |  |  |  |  |  |  |  |  |  |  |  |  |  |  |  |  |  |  |  |  |  |  |  |  | *gst-6* | Glutathione S-Transferase |
|  |  |  |  |  |  |  |  |  |  |  |  |  |  |  |  |  |  |  |  |  |  |  |  |  |  |  |  |  |  |  |  |  |  |  |  |  |  | F58G6.9 |  |
|  |  |  |  |  |  |  |  |  |  |  |  |  |  |  |  |  |  |  |  |  |  |  |  |  |  |  |  |  |  |  |  |  |  |  |  |  |  | *vha-6* | Vacuolar H ATPase |
|  |  |  |  |  |  |  |  |  |  |  |  |  |  |  |  |  |  |  |  |  |  |  |  |  |  |  |  |  |  |  |  |  |  |  |  |  |  | *ugt-62* | UDP-GlucuronosylTransferase |
|  |  |  |  |  |  |  |  |  |  |  |  |  |  |  |  |  |  |  |  |  |  |  |  |  |  |  |  |  |  |  |  |  |  |  |  |  |  | K06A9.1 |  |
|  |  |  |  |  |  |  |  |  |  |  |  |  |  |  |  |  |  |  |  |  |  |  |  |  |  |  |  |  |  |  |  |  |  |  |  |  |  | M04C3.1 |  |
|  |  |  |  |  |  |  |  |  |  |  |  |  |  |  |  |  |  |  |  |  |  |  |  |  |  |  |  |  |  |  |  |  |  |  |  |  |  | *dsc-4* | Defecation Suppressor of Clk-1 |
|  |  |  |  |  |  |  |  |  |  |  |  |  |  |  |  |  |  |  |  |  |  |  |  |  |  |  |  |  |  |  |  |  |  |  |  |  |  | R09H10.5 |  |
|  |  |  |  |  |  |  |  |  |  |  |  |  |  |  |  |  |  |  |  |  |  |  |  |  |  |  |  |  |  |  |  |  |  |  |  |  |  | F55G11.2 |  |
|  |  |  |  |  |  |  |  |  |  |  |  |  |  |  |  |  |  |  |  |  |  |  |  |  |  |  |  |  |  |  |  |  |  |  |  |  |  | C32H11.4 |  |
|  |  |  |  |  |  |  |  |  |  |  |  |  |  |  |  |  |  |  |  |  |  |  |  |  |  |  |  |  |  |  |  |  |  |  |  |  |  | F55G11.8 |  |
|  |  |  |  |  |  |  |  |  |  |  |  |  |  |  |  |  |  |  |  |  |  |  |  |  |  |  |  |  |  |  |  |  |  |  |  |  |  | *asp-5* | ASpartyl Protease |
|  |  |  |  |  |  |  |  |  |  |  |  |  |  |  |  |  |  |  |  |  |  |  |  |  |  |  |  |  |  |  |  |  |  |  |  |  |  | *ttr-46* | TransThyretin-Related family domain |
|  |  |  |  |  |  |  |  |  |  |  |  |  |  |  |  |  |  |  |  |  |  |  |  |  |  |  |  |  |  |  |  |  |  |  |  |  |  | T20D3.2 |  |
|  |  |  |  |  |  |  |  |  |  |  |  |  |  |  |  |  |  |  |  |  |  |  |  |  |  |  |  |  |  |  |  |  |  |  |  |  |  | F59B1.2 |  |
|  |  |  |  |  |  |  |  |  |  |  |  |  |  |  |  |  |  |  |  |  |  |  |  |  |  |  |  |  |  |  |  |  |  |  |  |  |  | *asp-3* | ASpartyl Protease |
|  |  |  |  |  |  |  |  |  |  |  |  |  |  |  |  |  |  |  |  |  |  |  |  |  |  |  |  |  |  |  |  |  |  |  |  |  |  | *asp-6* | ASpartyl Protease |
|  |  |  |  |  |  |  |  |  |  |  |  |  |  |  |  |  |  |  |  |  |  |  |  |  |  |  |  |  |  |  |  |  |  |  |  |  |  | *asp-1* | ASpartyl Protease |
|  |  |  |  |  |  |  |  |  |  |  |  |  |  |  |  |  |  |  |  |  |  |  |  |  |  |  |  |  |  |  |  |  |  |  |  |  |  | F54C9.3 |  |
|  |  |  |  |  |  |  |  |  |  |  |  |  |  |  |  |  |  |  |  |  |  |  |  |  |  |  |  |  |  |  |  |  |  |  |  |  |  | T04C12.1 |  |
|  |  |  |  |  |  |  |  |  |  |  |  |  |  |  |  |  |  |  |  |  |  |  |  |  |  |  |  |  |  |  |  |  |  |  |  |  |  | C46G7.1 |  |
|  |  |  |  |  |  |  |  |  |  |  |  |  |  |  |  |  |  |  |  |  |  |  |  |  |  |  |  |  |  |  |  |  |  |  |  |  |  | F46G10.1 |  |
|  |  |  |  |  |  |  |  |  |  |  |  |  |  |  |  |  |  |  |  |  |  |  |  |  |  |  |  |  |  |  |  |  |  |  |  |  |  | *clec-17* | C-type LECtin |
|  |  |  |  |  |  |  |  |  |  |  |  |  |  |  |  |  |  |  |  |  |  |  |  |  |  |  |  |  |  |  |  |  |  |  |  |  |  | *spp-5* | SaPosin-like Protein family |
|  |  |  |  |  |  |  |  |  |  |  |  |  |  |  |  |  |  |  |  |  |  |  |  |  |  |  |  |  |  |  |  |  |  |  |  |  |  | F19C7.1 |  |
|  |  |  |  |  |  |  |  |  |  |  |  |  |  |  |  |  |  |  |  |  |  |  |  |  |  |  |  |  |  |  |  |  |  |  |  |  |  | Y34B4A.6 |  |
|  |  |  |  |  |  |  |  |  |  |  |  |  |  |  |  |  |  |  |  |  |  |  |  |  |  |  |  |  |  |  |  |  |  |  |  |  |  | *mth-1* | MeTHuselah (Drosophila aging-associated GPCR) homolog |
|  |  |  |  |  |  |  |  |  |  |  |  |  |  |  |  |  |  |  |  |  |  |  |  |  |  |  |  |  |  |  |  |  |  |  |  |  |  | *clec-84* | C-type LECtin |
|  |  |  |  |  |  |  |  |  |  |  |  |  |  |  |  |  |  |  |  |  |  |  |  |  |  |  |  |  |  |  |  |  |  |  |  |  |  | C18H9.6 |  |
|  |  |  |  |  |  |  |  |  |  |  |  |  |  |  |  |  |  |  |  |  |  |  |  |  |  |  |  |  |  |  |  |  |  |  |  |  |  | C05C12.4 |  |
|  |  |  |  |  |  |  |  |  |  |  |  |  |  |  |  |  |  |  |  |  |  |  |  |  |  |  |  |  |  |  |  |  |  |  |  |  |  | C05D12.3 |  |
|  |  |  |  |  |  |  |  |  |  |  |  |  |  |  |  |  |  |  |  |  |  |  |  |  |  |  |  |  |  |  |  |  |  |  |  |  |  | *scav-5* | SCAVenger receptor (CD36 family) related |
|  |  |  |  |  |  |  |  |  |  |  |  |  |  |  |  |  |  |  |  |  |  |  |  |  |  |  |  |  |  |  |  |  |  |  |  |  |  | C35A11.4 |  |
|  |  |  |  |  |  |  |  |  |  |  |  |  |  |  |  |  |  |  |  |  |  |  |  |  |  |  |  |  |  |  |  |  |  |  |  |  |  | M60.2 |  |
|  |  |  |  |  |  |  |  |  |  |  |  |  |  |  |  |  |  |  |  |  |  |  |  |  |  |  |  |  |  |  |  |  |  |  |  |  |  | *cyp-37B1* | CYtochrome P450 family |
|  |  |  |  |  |  |  |  |  |  |  |  |  |  |  |  |  |  |  |  |  |  |  |  |  |  |  |  |  |  |  |  |  |  |  |  |  |  | T08B1.1 |  |
|  |  |  |  |  |  |  |  |  |  |  |  |  |  |  |  |  |  |  |  |  |  |  |  |  |  |  |  |  |  |  |  |  |  |  |  |  |  | *spp-3* | SaPosin-like Protein family |
|  |  |  |  |  |  |  |  |  |  |  |  |  |  |  |  |  |  |  |  |  |  |  |  |  |  |  |  |  |  |  |  |  |  |  |  |  |  | *ttr-44* | TransThyretin-Related family domain |
|  |  |  |  |  |  |  |  |  |  |  |  |  |  |  |  |  |  |  |  |  |  |  |  |  |  |  |  |  |  |  |  |  |  |  |  |  |  | *asp-2* | ASpartyl Protease |
|  |  |  |  |  |  |  |  |  |  |  |  |  |  |  |  |  |  |  |  |  |  |  |  |  |  |  |  |  |  |  |  |  |  |  |  |  |  | F54D5.3 |  |
|  |  |  |  |  |  |  |  |  |  |  |  |  |  |  |  |  |  |  |  |  |  |  |  |  |  |  |  |  |  |  |  |  |  |  |  |  |  | *clec-63* | C-type LECtin |
|  |  |  |  |  |  |  |  |  |  |  |  |  |  |  |  |  |  |  |  |  |  |  |  |  |  |  |  |  |  |  |  |  |  |  |  |  |  | *dod-19* | Downstream Of DAF-16 (regulated by DAF-16) |
|  |  |  |  |  |  |  |  |  |  |  |  |  |  |  |  |  |  |  |  |  |  |  |  |  |  |  |  |  |  |  |  |  |  |  |  |  |  | C49C8.5 |  |
|  |  |  |  |  |  |  |  |  |  |  |  |  |  |  |  |  |  |  |  |  |  |  |  |  |  |  |  |  |  |  |  |  |  |  |  |  |  | *clec-66* | C-type LECtin |
|  |  |  |  |  |  |  |  |  |  |  |  |  |  |  |  |  |  |  |  |  |  |  |  |  |  |  |  |  |  |  |  |  |  |  |  |  |  | Y34B4A.9 |  |
|  |  |  |  |  |  |  |  |  |  |  |  |  |  |  |  |  |  |  |  |  |  |  |  |  |  |  |  |  |  |  |  |  |  |  |  |  |  | D2045.8 |  |
|  |  |  |  |  |  |  |  |  |  |  |  |  |  |  |  |  |  |  |  |  |  |  |  |  |  |  |  |  |  |  |  |  |  |  |  |  |  | *hmit-1.1* | H(+) MyoInositol coTransporter |
|  |  |  |  |  |  |  |  |  |  |  |  |  |  |  |  |  |  |  |  |  |  |  |  |  |  |  |  |  |  |  |  |  |  |  |  |  |  | F54D5.4 |  |
|  |  |  |  |  |  |  |  |  |  |  |  |  |  |  |  |  |  |  |  |  |  |  |  |  |  |  |  |  |  |  |  |  |  |  |  |  |  | Y71H2AM.14 |  |
|  |  |  |  |  |  |  |  |  |  |  |  |  |  |  |  |  |  |  |  |  |  |  |  |  |  |  |  |  |  |  |  |  |  |  |  |  |  | Y70C5A.3 |  |
|  |  |  |  |  |  |  |  |  |  |  |  |  |  |  |  |  |  |  |  |  |  |  |  |  |  |  |  |  |  |  |  |  |  |  |  |  |  | *gcsh-1* | Glycine Cleavage System H protein |
|  |  |  |  |  |  |  |  |  |  |  |  |  |  |  |  |  |  |  |  |  |  |  |  |  |  |  |  |  |  |  |  |  |  |  |  |  |  | F10A3.4 |  |
|  |  |  |  |  |  |  |  |  |  |  |  |  |  |  |  |  |  |  |  |  |  |  |  |  |  |  |  |  |  |  |  |  |  |  |  |  |  | *skr-5* | SKp1 Related (ubiquitin ligase complex component) |
|  |  |  |  |  |  |  |  |  |  |  |  |  |  |  |  |  |  |  |  |  |  |  |  |  |  |  |  |  |  |  |  |  |  |  |  |  |  | Y82E9BR.13 |  |
|  |  |  |  |  |  |  |  |  |  |  |  |  |  |  |  |  |  |  |  |  |  |  |  |  |  |  |  |  |  |  |  |  |  |  |  |  |  | *scav-4* | SCAVenger receptor (CD36 family) related |
|  |  |  |  |  |  |  |  |  |  |  |  |  |  |  |  |  |  |  |  |  |  |  |  |  |  |  |  |  |  |  |  |  |  |  |  |  |  | F26G1.11 |  |
|  |  |  |  |  |  |  |  |  |  |  |  |  |  |  |  |  |  |  |  |  |  |  |  |  |  |  |  |  |  |  |  |  |  |  |  |  |  | *pud-1.2* |  |

### Phenotypes enriched

none found

### Anatomy terms enriched

none found

### GO terms enriched

|  |  |  |
| --- | --- | --- |
| **GO term** | **Number of genes** | **FDR-corrected p-value** |
| aspartic-type endopeptidase activity | 5 | 0.0012 |
| proteolysis | 18 | 0.0033 |
| protein maturation | 18 | 0.0040 |
| intermediate filament | 4 | 0.0160 |
| peroxisome | 4 | 0.0270 |
| transferase activity, transferring hexosyl groups | 9 | 0.0420 |
| apical plasma membrane | 5 | 0.0470 |

### Expression clusters enriched

|  |  |  |  |
| --- | --- | --- | --- |
| **Group name** | **Number in cluster** | **Enrichment** | **FDR corrected p** |
| Genes significantly enriched (> 2x, FDR < 5%) in a particular cell-type versus a reference sample of all cells at the same stage. WBPaper00037950:intestine\_embryo\_enriched | 219 | 12.10 | 4.18e-197 |
| Genes that show selective expression in a subset of cell types vs broadly expressed in many cell types. Correspond to 20% - 57% of enriched\_genes for a given cell type. WBPaper00037950:intestine\_embryo\_SelectivelyEnriched | 173 | 18.43 | 4.03e-183 |
| Genes significantly enriched (> 2x, FDR < 5%) in a particular cell-type versus a reference sample of all cells at both embryonic and larval stages. WBPaper00037950:intestine\_CoreEnriched | 106 | 17.43 | 7.04e-102 |
| Genes significantly enriched (> 2x, FDR < 5%) in a particular cell-type versus a reference sample of all cells at the same stage. WBPaper00037950:intestine\_larva\_enriched | 152 | 7.87 | 8.00e-96 |
| Genes that show selective expression in a subset of cell types vs broadly expressed in many cell types. Correspond to 20% - 57% of enriched\_genes for a given cell type. WBPaper00037950:pharyngeal-muscle\_embryo\_SelectivelyEnriched | 83 | 15.35 | 1.03e-72 |
| Genes significantly enriched (> 2x, FDR < 5%) in a particular cell-type versus a reference sample of all cells at the same stage. WBPaper00037950:pharyngeal-muscle\_embryo\_enriched | 105 | 8.59 | 6.66e-66 |
| Genes that show selective expression in a subset of cell types vs broadly expressed in many cell types. Correspond to 20% - 57% of enriched\_genes for a given cell type. WBPaper00037950:intestine\_larva\_SelectivelyEnriched | 92 | 10.29 | 4.23e-64 |
| Genes predicted to be downregulated more than 2.0 fold in (AFD+AWB) datasets as compared to unsorted whole embryonic cells dataset. | 98 | 9.13 | 1.34e-63 |
| Genes with increased expression after 24 hours of infection by S.marcescens Fold changes shown are pathogen vs OP50. WBPaper00038438:S.marcescens\_24hr\_upregulated\_RNAseq | 170 | 3.67 | 1.39e-56 |
| Genes with increased expression after 24 hours of infection by S.marcescens Fold changes shown are pathogen vs OP50. WBPaper00038438:S.marcescens\_24hr\_upregulated\_TilingArray | 170 | 3.14 | 9.21e-47 |
| Gene significantly up-regulated by treatment with 2.0mM of HuminFeed Hydroquinone until young adult stage (3 days), with a minimum fold change in gene expression of 1.25. | 107 | 4.88 | 1.97e-42 |
| WT-Pico Pan-neural Depleted Genes, with genes found multiple times in a single dataset removed (without dups). | 113 | 4.41 | 6.97e-41 |
| Genes with expression altered >= 3-fold in dpy-10(e128) mutants. | 174 | 2.56 | 1.19e-35 |
| Expression Pattern Group C, enriched for genes involved in metabolic processes. | 94 | 4.64 | 1.14e-34 |
| Developmentally modulated gene cluster. cgc4386\_cluster\_1\_3 | 41 | 14.35 | 8.81e-33 |
| Genes upregulated in worms grown on P. aeruginosa PA14 as compared to worms grown on OP50 for 8 hours by at least 2 fold and P < 0.01, as determined by a t-test. | 48 | 8.98 | 1.30e-28 |
| Gene significantly up-regulated by treatment with 0.2mM of HuminFeed until young adult stage (3 days), with a minimum fold change in gene expression of 1.25. | 98 | 3.61 | 2.08e-27 |
| TGF- Dauer pathway adult transcriptional targets. Results obtained by comparing the microarray results of the dauer-constitutive mutants daf-7(e1372), daf-7(m62), and daf-1(m40) with dauer-defective mutants daf-3(mgDf90), daf-5(e1386), and daf-7(e1372);daf-3(mgDf90) double mutants at the permissive temperature, 20C, on the first day of adulthood. WBPaper00031040:TGF-beta\_adult\_upregulated | 126 | 2.84 | 6.94e-27 |
| Genes with expression altered >= 3-fold in dpy-9(e12) mutants. | 145 | 2.53 | 8.51e-27 |
| Genes up-regulated by RPW-24. | 46 | 8.57 | 2.05e-26 |
| hermaphrodite soma-enriched | 55 | 6.64 | 2.74e-26 |
| Genes up regulated by P. aeruginosa Infection. | 41 | 10.05 | 4.25e-26 |
| Germline-enriched and sex-biased expression profile cluster C. | 78 | 4.35 | 5.18e-26 |
| Genes upregulated in worms grown on P. aeruginosa PA14 as compared to worms grown on OP50 for 4 hours by at least 2 fold and P < 0.01, as determined by a t-test. | 48 | 7.64 | 2.32e-25 |
| Genes with a >= 10-fold decrease in expression in Day 15 adults relative to expression levels in Day 6 adults are listed. | 52 | 6.78 | 3.78e-25 |
| Genes downregulated by CYD-1/CDK-4 for more than two fold. | 61 | 5.54 | 4.05e-25 |
| Genes up-regulated after 300 um Tannic acid treatment. Fold change > 1.25. | 88 | 3.73 | 4.19e-25 |
| Genes differentially expressed in control vs under EtBr treatment without UVC exposure, at the -25h timepoint. | 63 | 5.22 | 1.34e-24 |
| Genes up regulated in alg-1(gk214) comparing to in N2. | 80 | 4.03 | 1.49e-24 |
| Genes that showed increased expression in nhr-8(hd117) comparing to N2. | 47 | 7.02 | 3.63e-23 |
| Genes upregulated in rde-4(-/-) adult animals by at least 1.5 fold and P < 0.05, as determined by a multisample t-test. | 50 | 6.45 | 4.35e-23 |
| Transcripts that cycle in warm/cold (WC) condition but not in constant cold (CC) condition (pF24<0.02). | 103 | 3.01 | 1.12e-22 |
| Differentially expressed genes in the following exposure comparison:live C. albicans versus heat-killed E. coli. | 44 | 7.41 | 1.66e-22 |
| Genes changed expression level after Y. pestis treatment. P-value < 0.05. | 41 | 7.97 | 4.56e-22 |
| Genes with expression altered >= 3-fold in osm-8(n1518) mutants. | 78 | 3.68 | 2.41e-21 |
| Genes down-regulated after 100 um Quercetin treatment. Fold change < 0.8. | 83 | 3.35 | 2.67e-20 |
| Genes with expression level induced by bacteria strain PA14. | 39 | 7.44 | 9.24e-20 |
| Differentially expressed genes in the following exposure comparison :heat-killed C. albicans versus heat-killed E. coli. | 39 | 7.16 | 3.73e-19 |
| Genes up-regulated after 200 um Tannic acid treatment. Fold change > 1.25. | 53 | 4.68 | 4.41e-18 |
| Genes in the top 10% of expression level across the triplicate L3 samples. To generate the top10 and bottom10 gene sets, authors ranked all genes by mean expression array signal intensity across the three replicates, then took the top and bottom deciles (1,841 genes each) to represent genes with high and low expression. | 100 | 2.66 | 6.79e-18 |
| Genes enriched in intestine. | 105 | 2.57 | 7.46e-18 |
| Genes that showed significantly changed expression during aging (ANOVA, p < 0.0001) | 79 | 3.18 | 1.03e-17 |
| Genes downregulated in hcf-1(-), downregulated in sir-2.1(O/E) and downregulated in daf-2(-). | 37 | 6.77 | 3.15e-17 |
| Genes regulated by octr-1(ok371) after infected with P. aeruginosa PA14 for 4 hours at 25 centigrade. | 69 | 3.46 | 4.76e-17 |
| Gene significantly up-regulated by treatment with 2.0mM of HuminFeed until young adult stage (3 days), with a minimum fold change in gene expression of 1.25. | 88 | 2.81 | 1.06e-16 |
| Genes differentially expressed in control vs after UVC exposure and EtBr treatment at the -25h timepoint (just prior to the second UVC dose (24h)). | 72 | 3.24 | 2.71e-16 |
| Genes with expression altered >= 3-fold in osm-11(n1604) mutants. | 42 | 5.29 | 8.40e-16 |
| Genes significantly enriched (> 2x, FDR < 5%) in a particular cell-type versus a reference sample of all cells at the same stage. WBPaper00037950:PVD-OLL-neurons\_larva\_enriched | 63 | 3.52 | 1.24e-15 |
| Genome-wide analysis of developmental and sex-regulated gene expression profile. cgc4489\_group\_3 | 59 | 3.72 | 1.30e-15 |
| Genes down-regulated after 200 um Quercetin treatment. Fold change < 0.8. | 78 | 2.93 | 1.98e-15 |
| Genes with expression level down regulated in mir-35 mutants comparing with N2. | 66 | 3.19 | 2.24e-14 |
| Developmentally modulated gene cluster. cgc4386\_cluster\_1\_2 | 22 | 11.12 | 3.41e-14 |
| Genes that show selective expression in a subset of cell types vs broadly expressed in many cell types. Correspond to 20% - 57% of enriched\_genes for a given cell type. WBPaper00037950:PVD-OLL-neurons\_larva\_SelectivelyEnriched | 38 | 5.22 | 6.34e-14 |
| Genes that showed decreased expression in nhr-8(hd117) comparing to N2. | 31 | 6.63 | 6.36e-14 |
| Differentially expressed genes during worm lifespan. Medoid 4 Fig.4. | 26 | 7.96 | 2.38e-13 |
| Genes enriched in intestinal AIN-2 miRISCs. Pges-1-ain-2-gfp IP was performed in mixed stage worms. | 42 | 4.30 | 1.30e-12 |
| Genes upregulated in worms grown on wild-type P. aeruginosa PA14 as compared to worms grown on an isogenic PA14 mutant gacA for 4 hours by at least 2 fold and P < 0.01, as determined by a t-test. | 27 | 6.72 | 4.58e-12 |
| Significantly differentially expressed genes after different bacteria treatment. Transcriptional responses of wild-type C. elegans adults were assayed after growth on each of the four bacteria - E. coli, M. luteus, Pseudomonas sp., or B. megaterium. These genes were differentially expressed and statistically significant with multiple testing correction (q < 0.01) across all pair-wise comparisons. | 27 | 6.62 | 6.60e-12 |
| Genes differentially expressed under EtBr treatment without UVC exposure vs after UVC exposure but without EtBr treatment at the -25h timepoint (just prior to the second UVC dose (24h)). | 37 | 4.51 | 1.50e-11 |
| Genes upregulated in worms grown on wild-type P. aeruginosa PA14 as compared to worms grown on an isogenic PA14 mutant gacA for 8 hours by at least 2 fold and P < 0.01, as determined by a t-test. | 24 | 7.22 | 2.91e-11 |
| Genes up-regulated after 100 um Tannic acid treatment. Fold change > 1.25. | 40 | 4.09 | 2.96e-11 |
| Genes up-regulated during spg-7(RNAi) treatment. | 47 | 3.54 | 3.29e-11 |
| Significantly upregulated genes from cyc-1(RNAi) microarrays using SAM algorithm with an FDR < 0.1 from adult-only chips. | 138 | 1.79 | 3.68e-11 |
| Genes that were downregulated in lin-15B(n744). | 50 | 3.27 | 9.60e-11 |
| Differentially expressed genes between C.elegans fed with L.rhamnosus strain CNCM I-3690 and control strain E.coli OP50 after 3 days of feeding. | 66 | 2.66 | 1.09e-10 |
| Caenorhabditis elegans Genes with expression levels changed significantly after treatment of Staphylococcus aureus. | 24 | 6.57 | 2.25e-10 |
| Genes that showed upregulated expression level after virus infection in JU1580. | 23 | 6.87 | 2.69e-10 |
| Genes up regulated in the absence of TDP-1, when the threshold was set at a fold change (FC) of 1.2. | 78 | 2.31 | 6.21e-10 |
| Gene significantly up-regulated by treatment with 0.2mM of HuminFeed until older adult stage (11 days), with a minimum fold change in gene expression of 1.25. | 35 | 4.07 | 1.33e-09 |
| Genes that showed increased expression after treated with 2-deoxy-D-glucose. | 27 | 5.23 | 1.68e-09 |
| Genes up-regulated during spg-7(RNAi) treatment that are dependent on atfs-1(tm4525). | 32 | 4.36 | 2.07e-09 |
| Genes upregulated by oxidative stress. | 52 | 2.89 | 3.06e-09 |
| Genes down-regulated in long-lived daf-12(rh273) are listed including the log2 fold-induction in daf-12(rh273) compared to daf-12(rh61rh411). | 20 | 7.05 | 5.22e-09 |
| Genes upregulated in dcr-1(-/-) adult animals by at least 1.5 fold and P < 0.01, as determined by a multisample t-test and the Benjamini and Hochberg false discovery rate correction. | 58 | 2.64 | 5.29e-09 |
| Genes up-regulated in acs-3(ft5) mutants. | 27 | 4.96 | 5.53e-09 |
| Genes differentially expressed under EtBr treatment and UVC exposure vs under UVC exposure but without EtBr treatment at the -25h timepoint (just prior to the second UVC dose (24h)). | 44 | 3.16 | 8.32e-09 |
| Genes up-regulated greater than 2-fold, demonstrating a robust inducible response to Y. pestis. | 17 | 8.33 | 1.28e-08 |
| Expression Pattern Group I, enriched for genes involved in transport. | 64 | 2.42 | 1.50e-08 |
| Candidate daf-19 down regulated genes with a statistically significant signal variation of 1.5-fold or greater. These were identified using a class comparisons tool from BRB Array Tools. | 37 | 3.35 | 7.70e-08 |
| Genes that showed decreased expression in adult animals after 12 hour exposure to B. pseudomallei R15 vs. exposure to OP50 | 35 | 3.45 | 1.14e-07 |
| The cluster contains genes that are upregulated with daf-16 RNAi treatment and downregulated with daf-2 RNAi treatment and in daf-2 pathway mutants. | 24 | 4.82 | 1.35e-07 |
| Genes that showed increased expression in adult animals after 8 hour exposure to B. pseudomallei R15 vs. exposure to OP50 | 39 | 3.15 | 1.42e-07 |
| Genes showing < 0.5-fold down-regulated expression (p < 0.001) both in aex-3::His-SUMO-1 or myo-4::His-SUMO-1 C. elegans. | 30 | 3.87 | 1.63e-07 |
| Genes with differential expression under 0.5mg/l CPF and 0.5mg/l DZN treatment at 24 centigrade. | 73 | 2.11 | 2.60e-07 |
| Genes that showed decreased expression after 24h of DATS(garlic constituent diallyl trisulfide) treatment in spe-9(hc88); fer-15(b26) worms. | 10 | 15.32 | 3.03e-07 |
| Genes with expression level up in alg-1 mutant background. | 18 | 6.21 | 4.21e-07 |
| Genes up-regulated in animals infected with Photorhabdus luminescens compared to the E. coli OP50 control. | 38 | 3.07 | 4.83e-07 |
| Genes downregulated in mdt-15(RNAi) animals. | 20 | 5.45 | 4.97e-07 |
| Gene significantly down-regulated by treatment with 2.0mM of HuminFeed until older adult stage (11 days), with a minimum fold change in gene expression of 0.8. | 20 | 5.45 | 4.97e-07 |
| Genes upregulated by > 2-fold in CY262(sqt-1(sc13) age-1(mg44); bvIs1) adults, which intestinally express age-1, relative to wildtype. | 44 | 2.73 | 7.99e-07 |
| Genes upregulated by > 2-fold in CY251(sqt-1(sc13) age-1(mg44); bvIs2) adults, which neuronally express age-1, relative to wildtype. | 44 | 2.73 | 7.99e-07 |
| Genes upregulated by > 2-fold in SP75 (sqt-1(sc13) age-1(mg44)/mnC1) adults relative to wildtype. | 44 | 2.73 | 7.99e-07 |
| Genes that are up or down regulated by more than 2.1 fold with the t-test p-value less than 0.01 are included in this cluster. | 58 | 2.31 | 8.22e-07 |
| Genes with altered expression after 8 h S. aureus infection. | 29 | 3.70 | 8.39e-07 |
| Genes that showed expression levels higher than the corresponding reference sample (L3/L4 all cell reference). | 174 | 1.42 | 1.61e-06 |
| Genes that showed expression levels higher than the corresponding reference sample (embryonic 24hr reference). WBPaper00037950:pharyngeal-muscle\_expressed | 182 | 1.40 | 1.81e-06 |
| Genes downregulated in daf-2(-) compared to daf-16(-); daf-2(-) | 24 | 4.13 | 2.60e-06 |
| Genes that showed increased expression after exposure to 7.5uM HgCl2 for 24 hours. | 12 | 8.65 | 5.91e-06 |
| Genes upregulated in daf-2(-) compared to daf-16(-); daf-2(-) | 17 | 5.55 | 5.95e-06 |
| TGF-beta adult-specific up/dauer down | 13 | 7.41 | 9.84e-06 |
| Genes with decreased expression after 12 hours of infection by D.coniospora Fold changes shown are pathogen vs OP50. | 84 | 1.80 | 1.36e-05 |
| Genes down regulated by nasp-1. This experiment compares nasp-1 mutant versus N2 strain in C. elegans, after both have been exposed to the pathogenic bacteria B. thuringiensis DB27. | 21 | 4.24 | 1.39e-05 |
| Genes that showed increased expression in adult animals after 12 hour exposure to B. pseudomallei R15 vs. exposure to OP50 | 30 | 3.16 | 1.41e-05 |
| Genes that change expression level at day 8 adult stage after ash-2 RNAi. | 33 | 2.94 | 1.50e-05 |
| Genes that were upregulated in lin-15B(n744). | 57 | 2.13 | 1.58e-05 |
| Genes up-regulated in animals infected with Enterococcus faecalis compared to the E. coli OP50 control. | 34 | 2.82 | 2.46e-05 |
| Genes with increased expression after 24 hours of infection by Harposporium Fold changes shown are pathogen vs OP50. | 77 | 1.83 | 2.72e-05 |
| Genes differentially expressed in control vs after UVC exposure and EtBr treatment at the 3h timepoint (3 h after the third UVC dose (51h), which is also 3 h after being placed on food). | 121 | 1.54 | 2.83e-05 |
| Genes with expression altered >= 3-fold in osm-7(n1515) mutants. | 21 | 4.04 | 3.00e-05 |
| Genes downregulated in worms grown on wild-type P. aeruginosa PA14 as compared to worms grown on an isogenic PA14 mutant gacA for 4 hours by at least 2 fold and P < 0.01, as determined by a t-test. | 12 | 7.35 | 3.43e-05 |
| C-lineage related expression profile. WBPaper00025032:cluster\_16 | 11 | 8.17 | 3.92e-05 |
| Up-regulated genes under 1 mg/l DZN treatment at 16 centigrade | 14 | 5.77 | 6.23e-05 |
| Genes that showed differential expression in adult animals after 12 hour exposure to B. pseudomallei R15 vs. exposure to OP50, confirmed by microarray and qPCR. | 8 | 12.65 | 6.38e-05 |
| Genes upregulated in rde-1(-/-) adult animals by at least 1.5 fold and P < 0.05, as determined by a multisample t-test. | 10 | 8.75 | 7.34e-05 |
| Caenorhabditis elegans Genes with expression levels changed significantly after treatment of Xenorhabdus nematophila. | 187 | 1.31 | 1.19e-04 |
| Genes down-regulated by RPW-24. | 10 | 8.17 | 1.39e-04 |
| Genes down regulated in hcf-1(-), downregulated in sir-2.1(O/E) and no change in daf-2(-). | 12 | 6.32 | 1.67e-04 |
| Genes downregulated in worms grown on P. aeruginosa PA14 as compared to worms grown on OP50 for 8 hours by at least 2 fold and P < 0.01, as determined by a t-test. | 18 | 4.08 | 1.95e-04 |
| Genes upregulated by fasting anytime between 9 hour to 12 hour time course in N2 worms. | 35 | 2.52 | 2.07e-04 |
| Developmentally modulated gene cluster. cgc4386\_cluster\_2\_2 | 11 | 6.74 | 2.56e-04 |
| Genes enriched in L4 larva intestinal AIN-2 miRISCs, potentially involved in non-development processes. Pges-1-ain-2-gfp IP was performed in synchronized L4 larva. | 26 | 2.96 | 3.24e-04 |
| Transcripts that cycle both in LD (light/dark) and DD (constant darkness) (pF24<0.02). | 20 | 3.58 | 3.58e-04 |
| Developmentally modulated gene cluster. cgc4386\_cluster\_1\_1 | 14 | 4.94 | 3.75e-04 |
| Up-regulated genes under 0.5mg/l CPF treatment at 16 centigrade. | 15 | 4.57 | 4.14e-04 |
| Genes upregulated on Comamonas DA1877 relative to E. coli OP50, Gravid adult stage | 49 | 2.05 | 4.21e-04 |
| Candidate daf-19 down regulated genes with a statistically significant signal variation of 1.5-fold or greater. These were identified using a Significance Analysis of Microarrays (SAM). | 16 | 4.24 | 4.86e-04 |
| A large cluster of genes up-regulated during early larval development.. | 67 | 1.79 | 4.95e-04 |
| The cluster contains genes that are upregulated with daf-2 RNAi treatment and in daf-2 pathway mutants, and downregulated with daf-16 RNAi treatment. | 19 | 3.64 | 5.10e-04 |
| Genes up regulated in the absence of TDP-1, when the threshold was set at a fold change (FC) of 1.5. | 17 | 3.89 | 7.11e-04 |
| Genes downregulated in worms grown on wild-type P. aeruginosa PA14 as compared to worms grown on an isogenic PA14 mutant gacA for 8 hours by at least 2 fold and P < 0.01, as determined by a t-test. | 15 | 4.32 | 7.77e-04 |
| Gene significantly up-regulated by treatment with 0.2mM of HuminFeed Hydroquinone until young adult stage (3 days), with a minimum fold change in gene expression of 1.25. | 24 | 2.95 | 8.69e-04 |
| Genes that showed decreased expression in adult animals after 4 hour exposure to B. pseudomallei R15 vs. exposure to OP50 | 10 | 6.62 | 8.80e-04 |
| Caenorhabditis elegans Genes with expression levels changed significantly after treatment of Serratia marcescens. | 108 | 1.49 | 9.43e-04 |
| Strictly embryonic (SE) subclasses are based on the earliest significant increase(abbreviated pi for primary increase). [cgc5767]:expression\_class\_SE\_pi(122\_min) | 11 | 5.86 | 9.46e-04 |
| Genes with expression altered >= 3-fold at one or more timepoint by the osmotic changes | 20 | 3.33 | 9.70e-04 |
| Genes downregulated in worms grown on P. aeruginosa PA14 as compared to worms grown on OP50 for 4 hours by at least 2 fold and P < 0.01, as determined by a t-test. | 12 | 5.11 | 1.37e-03 |
| C-lineage related expression profile. WBPaper00025032:cluster\_37 | 7 | 10.09 | 1.55e-03 |
| Up-Regulated genes under 0.5mg/l CPF+ 1 mg/l DZN treatment at 16 centigrade. | 14 | 4.32 | 1.60e-03 |
| Genes downregulated by oxidative stress. | 22 | 2.96 | 1.95e-03 |
| Genes that showed decreased expression in adult animals after 8 hour exposure to B. pseudomallei R15 vs. exposure to OP50 | 17 | 3.58 | 2.00e-03 |
| Developmentally modulated gene cluster. cgc4386\_cluster\_1\_4 | 11 | 5.29 | 2.33e-03 |
| Developmentally modulated gene cluster. cgc4386\_cluster\_2\_3 | 10 | 5.83 | 2.48e-03 |
| Genes upregulated by wild-type pmk-1 (downregulated in daf-2(e1368);pmk-1(km25) strain as compared to daf-2(e1368) strain) by at least 2 fold and P < 0.01, as determined by a t-test. | 10 | 5.77 | 2.73e-03 |
| The third of four clusters of genes up-regulated during L1 arrest. | 20 | 3.03 | 3.55e-03 |
| Significantly up-regulated genes (FDR< 10%) following exposure of C. elegans to albendazole. | 7 | 8.37 | 5.01e-03 |
| Genes up regulated by nasp-1. This experiment compares nasp-1 mutant versus N2 strain in C. elegans, after both have been exposed to the pathogenic bacteria B. thuringiensis DB27. | 6 | 10.50 | 5.19e-03 |
| Genes involved in oxidative stress responses and lifespan regulation that were differentially regulated in ung-1(7600). These are only part of the 1680 genes differentially regulated by ung-1(7600). | 11 | 4.77 | 5.69e-03 |
| Genes up or down regulated by 10e-05M of progesterone. The normalized values used were G/R ratio > 2.6 for up-regulation and G/R ratio < 0.38 for down-regulation, which corresponds to 1.39 and -1.39 log(base2) G/R ratio, respectively. | 77 | 1.57 | 6.40e-03 |
| Genes upregulated in mdt-15(RNAi) animals. | 11 | 4.65 | 7.08e-03 |
| Class A gene expression showed down regulation in lin-14(lf) in L1, no change in lin-4(lf) in L2. | 12 | 4.20 | 8.29e-03 |
| Genes up or down regulated by 10e-09M of cholesterol . The normalized values used were G/R ratio > 2.6 for up-regulation and G/R ratio < 0.38 for down-regulation, which corresponds to 1.39 and -1.39 log(base2) G/R ratio, respectively. | 61 | 1.67 | 1.01e-02 |
| Expression Pattern Group D, enriched for genes involved in catabolic processes. | 46 | 1.81 | 1.47e-02 |
| Genes upregulated by fasting anytime during the 48 hour time course in N2 worms. | 56 | 1.68 | 1.66e-02 |
| Genes down-regulated in daf-10(m79) mutants versus wild-type animals. | 7 | 6.73 | 1.83e-02 |
| Genes with no change in hcf-1(-), no change in sir-2.1(O/E) and upregulated in daf-2(-). | 36 | 1.95 | 2.43e-02 |
| Genome-wide analysis of developmental and sex-regulated gene expression profile. cgc4489\_group\_8 | 26 | 2.25 | 2.52e-02 |
| Genes predicted to be upregulated more than 2.0 fold in rde-3(r459) mutant worms as compared to wild-type animals (t-test P-value < 0.05). | 16 | 2.96 | 2.68e-02 |
| Genes that showed expression levels higher than the corresponding reference sample (embryonic 24hr reference). WBPaper00037950:intestine\_expressed | 227 | 1.16 | 2.97e-02 |
| Genes differentially expressed in control vs after UVC exposure and EtBr treatment at the -45h timepoint (3 hours after the first UVC dose). | 23 | 2.36 | 2.97e-02 |
| Genes up-regulated following nhr-25(RNAi). | 24 | 2.30 | 3.15e-02 |
| Differentially-expressed genes in the NI lines. | 10 | 4.19 | 3.25e-02 |
| Differentially expressed genes in the following exposure comparison:live C. albicans versus heat-killed C. albicans. | 7 | 5.81 | 4.19e-02 |
| Genes that showed decreased expression in adult animals after 2 hour exposure to B. pseudomallei R15 vs. exposure to OP50 | 5 | 9.08 | 4.22e-02 |
| Genes with changed expression in lin-54(n2290) embryo. | 37 | 1.86 | 4.46e-02 |
| Genes downregulated in hcf-1(-), no change in sir-2.1(O/E) and no change in daf-2(-). | 6 | 6.84 | 4.78e-02 |
| heme-responsive genes identified using Affymetrix C.elegans whole genome microarrays. Category 5 genes were upregulated by 4um heme treatment, also upregulated by 500 um heme treatment. Gene expression profiles were categorized based on the fold changes relative to 20 uM heme samples. Summary of genes that showed change in their expression profiles in response to heme was determined by MAS 5.0 and RMA methods. | 5 | 8.75 | 4.90e-02 |
| Genes that showed increased expression in adult animals after 2 hour exposure to B. pseudomallei R15 vs. exposure to OP50. | 11 | 3.64 | 4.91e-02 |
| Genes up or down regulated by 10e-05M of estrogen. The normalized values used were G/R ratio > 2.6 for up-regulation and G/R ratio < 0.38 for down-regulation, which corresponds to 1.39 and -1.39 log(base2) G/R ratio, respectively. | 46 | 1.71 | 4.97e-02 |
| Genes differentially expressed under EtBr treatment and UVC exposure vs under UVC exposure but without EtBr treatment at the -3h timepoint (3 h after the third UVC dose (51h), which is also 3 h after being placed on food). | 72 | 1.49 | 4.97e-02 |

### Motifs enriched

|  |  |  |  |  |  |
| --- | --- | --- | --- | --- | --- |
| **Motif** | **Logo** | **Possible orthologs** | **Number of motifs in cluster** | **Enrichment** | **FDR corrected p** |
| srp\_SANGER\_5\_FBgn0003507 |  | elt-7 (0.61) elt-3 ceh-34 egl-27 elt-6 elt-1 ceh-32 | 271 | 2.49 | 1.2e-71 |
| pTH1049 |  | elt-1 | 261 | 2.59 | 2.5e-70 |
| GATA3\_2 |  | elt-1 end-3 | 269 | 2.47 | 2.5e-70 |
| Gata5\_3768 |  | elt-1 | 263 | 2.52 | 6.0e-69 |
| GATA1\_si |  | ztf-29 elt-1 | 269 | 2.05 | 1.9e-51 |
| pTH9880 |  | end-1 | 209 | 2.72 | 4.0e-50 |
| HUVEC\_GATA2\_UCD |  | elt-1 | 269 | 1.95 | 8.3e-47 |
| V$GATA6\_01 |  | elt-1 | 209 | 2.34 | 2.3e-39 |
| I$MTTFA\_01 |  | hmg-5 | 221 | 2.18 | 4.7e-38 |
| Gata3\_1024 |  | elt-1 | 200 | 1.97 | 2.1e-25 |
| Mv73 |  | elt-1 | 152 | 2.20 | 1.3e-20 |
| V$GATA1\_02 |  | elt-1 | 257 | 1.43 | 2.7e-16 |
| So\_Cell\_FBgn0003460 |  | skn-1 ceh-32 | 294 | 1.23 | 1.2e-11 |
| Mf28 |  | elt-1 | 88 | 2.23 | 3.2e-10 |
| T-47D\_GATA3\_HudsonAlpha |  | elt-1 | 280 | 1.15 | 8.0e-05 |
| pTH9384 |  | cfi-1 | 255 | 1.19 | 8.4e-05 |
| exd\_SOLEXA\_2\_FBgn0000611 |  | ceh-20 | 294 | 1.12 | 1.3e-04 |
| pTH9913 |  | skn-1 | 73 | 1.76 | 2.1e-04 |
| GATA5\_f1 |  | elt-1 | 303 | 1.10 | 2.7e-04 |
| Foxa2\_2830 |  | let-381 | 307 | 1.09 | 3.5e-04 |
| V$GATA3\_01 |  | elt-1 | 222 | 1.23 | 3.6e-04 |
| pTH5119 |  | cfi-1 | 250 | 1.17 | 6.7e-04 |
| Arid3a\_3875 |  | cfi-1 | 277 | 1.13 | 7.3e-04 |
| V$PAX2\_02 |  | pax-1 | 267 | 1.15 | 7.7e-04 |
| pTH6556 |  | lim-6 | 156 | 1.34 | 7.7e-04 |
| pTH10028 |  | nhr-204 | 255 | 1.16 | 9.8e-04 |
| dsx-F\_FlyReg\_FBgn0000504 |  | dmd-4 sox-4 | 158 | 1.31 | 1.8e-03 |
| pTH9174 |  | nhr-273 (0.63) nhr-28 odr-7 | 85 | 1.55 | 2.4e-03 |
| ALX4\_1 |  | alr-1 cfi-1 | 296 | 1.09 | 2.5e-03 |
| pTH2808 |  | let-381 lin-31 | 208 | 1.21 | 3.0e-03 |
| pTH8983 |  | tag-347 | 285 | 1.11 | 3.0e-03 |
| Lim3\_SOLEXA\_FBgn0002023 |  | lim-6 cog-1 lim-7 lim-4 ceh-18 alr-1 | 280 | 1.11 | 3.4e-03 |
| pTH9300 |  | dmd-3 C34D1.1 | 209 | 1.21 | 3.4e-03 |
| pTH6425 |  | ceh-20 | 292 | 1.09 | 7.0e-03 |
| I$SN\_02 |  | K02D7.2 | 269 | 1.12 | 8.0e-03 |
| pTH3998 |  | tbx-39 | 123 | 1.35 | 8.2e-03 |
| Hlx1\_2350 |  | ceh-24 | 88 | 1.47 | 8.6e-03 |
| V$XFD3\_01 |  | let-381 | 317 | 1.04 | 9.1e-03 |
| MEF2C\_f1 |  | mef-2 | 304 | 1.07 | 9.6e-03 |
| MA0495.1 |  | fos-1 jun-1 F45H11.6 | 283 | 1.10 | 1.1e-02 |
| pTH10927 |  | tbx-38 tbx-39 mab-9 tbx-43 | 268 | 1.12 | 1.1e-02 |
| Tcf1\_2666 |  | hmbx-1 | 204 | 1.19 | 1.1e-02 |
| MA0493.1 |  | klf-1 | 115 | 1.36 | 1.2e-02 |
| pTH10034 |  | nhr-66 | 129 | 1.31 | 1.4e-02 |
| MA0126.1 |  | pax-3 lin-48 | 122 | 1.33 | 1.4e-02 |
| pTH5118 |  | cfi-1 | 158 | 1.26 | 1.4e-02 |
| MA0488.1 |  | crh-1 | 176 | 1.23 | 1.4e-02 |
| MA0594.1 |  | lin-39 | 211 | 1.18 | 1.5e-02 |
| MA0102.3 |  | C48E7.11 | 242 | 1.14 | 1.5e-02 |
| LHX6\_3 |  | lim-6 | 226 | 1.16 | 1.6e-02 |
| MA0235.1 |  | ceh-48 | 254 | 1.13 | 1.8e-02 |
| Pou3f1\_3819 |  | ceh-6 | 184 | 1.21 | 2.0e-02 |
| pTH3751 |  | tbx-39 | 127 | 1.31 | 2.0e-02 |
| FOXB1\_2 |  | let-381 lin-31 | 250 | 1.13 | 2.3e-02 |
| N$SKN1\_01 |  | skn-1 | 75 | 1.47 | 2.5e-02 |
| PO4F2\_si |  | unc-86 | 291 | 1.08 | 2.6e-02 |
| pTH3819 |  | ceh-18 | 274 | 1.10 | 2.8e-02 |
| pTH10837 |  | T22H9.4 | 311 | 1.05 | 2.8e-02 |
| Tcf1\_2666 |  | hmbx-1 | 199 | 1.18 | 2.9e-02 |
| pTH10808 |  | ztf-19 | 293 | 1.07 | 3.1e-02 |
| pTH10777 |  | dmd-3 | 215 | 1.16 | 3.1e-02 |
| MA0070.1 |  | ceh-20 | 110 | 1.33 | 3.1e-02 |
| Irx6\_2623 |  | irx-1 | 86 | 1.40 | 3.2e-02 |
| V$NKX22\_01 |  | dsc-1 | 149 | 1.25 | 3.2e-02 |
| pTH5164 |  | irx-1 hlh-32 | 114 | 1.32 | 3.3e-02 |
| MA0040.1 |  | let-381 daf-16 | 311 | 1.05 | 3.5e-02 |
| POU4F2\_2 |  | unc-86 | 253 | 1.12 | 3.5e-02 |
| MYB\_f1 |  | D1081.8 | 130 | 1.28 | 3.5e-02 |
| HMX2\_1 |  | ceh-19 ceh-9 lin-39 | 290 | 1.08 | 3.8e-02 |
| pTH9246 |  | C34D1.1 | 229 | 1.14 | 3.8e-02 |
| pTH10038 |  | gei-3 | 292 | 1.07 | 4.7e-02 |
| pTH9260 |  | mel-28 | 320 | 1.02 | 4.8e-02 |

### Correlated (and anti-correlated) transcription factors

|  |  |
| --- | --- |
| **Transcription factor** | **Correlation** |
| ets-9 | 0.95 |
| nhr-68 | 0.89 |
| nhr-8 | 0.88 |
| elt-2 | 0.88 |
| nhr-121 | 0.84 |
| nhr-176 | 0.83 |
| zip-5 | 0.83 |
| zip-10 | 0.82 |
| nhr-81 | 0.79 |
| pqm-1 | 0.78 |
| nhr-79 | 0.75 |
| nhr-16 | 0.73 |
| tbx-8 | 0.72 |
| F55B11.4 | 0.72 |
| nhr-226 | 0.69 |
| nhr-80 | 0.68 |
| nhr-170 | 0.67 |
| nhr-210 | 0.67 |
| zip-12 | 0.65 |
| tbx-9 | 0.65 |
| nhr-108 | 0.65 |
| nhr-109 | 0.64 |
| xnd-1 | 0.64 |
| nhr-177 | 0.64 |
| klf-3 | 0.63 |
| nhr-89 | -0.36 |
| grl-25 | -0.37 |
| madf-10 | -0.37 |
| ceh-43 | -0.38 |
| nhr-78 | -0.38 |
| lim-6 | -0.38 |
| ets-7 | -0.39 |
| Y54G2A.20 | -0.39 |
| T06G6.5 | -0.39 |
| nhr-157 | -0.40 |
| mxl-3 | -0.40 |
| ceh-49 | -0.40 |
| F21G4.5 | -0.41 |
| F38C2.7 | -0.42 |
| vab-3 | -0.42 |
| nhr-271 | -0.45 |
| ceh-84 | -0.45 |
| tbx-35 | -0.46 |
| tbx-33 | -0.46 |
| nhr-33 | -0.46 |
| tbx-31 | -0.47 |
| ceh-87 | -0.47 |
| sdz-38 | -0.48 |
| tbx-37 | -0.58 |
| ccch-2 | -0.58 |

### ChIP peaks enriched

|  |  |  |  |  |
| --- | --- | --- | --- | --- |
| **Gene** | **Experiment** | **Number of upstream peaks** | **Enrichment** | **FDR corrected p** |
| fos-1 | FOS-1\_Larvae-L2-stage | 268 | 3.43 | 1.8e-106 |
| nhr-28 | NHR-28\_Larvae-L4-stage | 252 | 3.18 | 7.7e-88 |
| nhr-77 | NHR-77\_Larvae-L4-stage | 246 | 2.70 | 9.6e-69 |
| C01B12.2 | C01B12.2\_Larvae-L2-stage | 226 | 2.69 | 1.3e-58 |
| dve-1 | DVE-1\_Larvae-L4-stage | 119 | 3.02 | 1.9e-27 |
| W03F9.2 | W03F9.2\_L4-Young-Adult-stage-larvae | 192 | 1.93 | 4.6e-24 |
| fos-1 | FOS-1\_Larvae-L3-stage | 110 | 2.62 | 4.3e-20 |
| unc-62 | UNC-62\_Day-Four-Young-Adult | 101 | 2.80 | 4.9e-20 |
| unc-62 | UNC-62\_Young-adult-Day-4 | 101 | 2.80 | 4.9e-20 |
| jun-1 | JUN-1\_Larvae-L3-stage | 89 | 2.54 | 1.1e-14 |
| tlp-1 | TLP-1\_Fed-L1-stage-larvae | 25 | 8.06 | 2.5e-13 |
| pha-4 | PHA-4\_Larvae-L2-stage | 129 | 1.85 | 8.3e-12 |
| lin-35 | LIN-35\_Starved-L1-stage-larvae | 61 | 2.50 | 3.0e-09 |
| sax-3 | SAX-3\_Larvae-L4-stage | 114 | 1.63 | 9.2e-07 |
| hpl-2 | HPL-2\_Fed-L1-stage-larvae | 118 | 1.57 | 3.7e-06 |
| fos-1 | FOS-1\_Larvae-L4-stage | 54 | 2.11 | 9.3e-06 |
| pha-4 | PHA-4\_Young-adult | 55 | 1.92 | 1.2e-04 |
| ztf-7 | ZTF-7\_Larvae-L4-stage | 70 | 1.71 | 2.1e-04 |
| jun-1 | JUN-1\_Larvae-L1-stage | 70 | 1.55 | 4.1e-03 |
| ham-1 | HAM-1\_Larvae-L4-stage | 88 | 1.35 | 4.1e-02 |
