# Supplementary material for: Overlapping cell population expression profiling and regulatory inference in C. elegans
Source: BMC Genomics. 2016 Feb 29;17:159. doi: 10.1186/s12864-016-2482-z (PMC4772325; doi:10.1186/s12864-016-2482-z)
Supplement: Additional file 13: — Web supplement. (DOC 21 kb) [file 12864_2016_2482_MOESM13_ESM.zip › sortWeb/clusters/hier.300.clusters/256.html]

Cluster 256 

## Cluster 256

### Expression

| cnd-1 rep. 1 | cnd-1 rep. 2 | cnd-1 rep. 3 | pha-4 rep. 1 | pha-4 rep. 2 | pha-4 rep. 3 | ceh-27 | ceh-36 | ceh-6 | F21D5.9 | mir-57 | mls-2 | pal-1 | pros-1 | ttx-3 | unc-130 | hlh-16 | irx-1 | ceh-6 (+) hlh-16 (+) | ceh-6 (+) hlh-16 (-) | ceh-6 (-) hlh-16 (+) | cnd-1 singlets | pha-4 singlets | 0 | 60 | 120 | 150 | 180 | 240 | 330 | 390 | 420 | 480 | 540 | 570 | 600 | 630 | 660 | NAME | Functional description |
| --- | --- | --- | --- | --- | --- | --- | --- | --- | --- | --- | --- | --- | --- | --- | --- | --- | --- | --- | --- | --- | --- | --- | --- | --- | --- | --- | --- | --- | --- | --- | --- | --- | --- | --- | --- | --- | --- | --- | --- |
|  |  |  |  |  |  |  |  |  |  |  |  |  |  |  |  |  |  |  |  |  |  |  |  |  |  |  |  |  |  |  |  |  |  |  |  |  |  | Y57G11A.225 |  |
|  |  |  |  |  |  |  |  |  |  |  |  |  |  |  |  |  |  |  |  |  |  |  |  |  |  |  |  |  |  |  |  |  |  |  |  |  |  | F11D5.30 |  |
|  |  |  |  |  |  |  |  |  |  |  |  |  |  |  |  |  |  |  |  |  |  |  |  |  |  |  |  |  |  |  |  |  |  |  |  |  |  | F16G10.8 |  |
|  |  |  |  |  |  |  |  |  |  |  |  |  |  |  |  |  |  |  |  |  |  |  |  |  |  |  |  |  |  |  |  |  |  |  |  |  |  | F10A3.7 |  |
|  |  |  |  |  |  |  |  |  |  |  |  |  |  |  |  |  |  |  |  |  |  |  |  |  |  |  |  |  |  |  |  |  |  |  |  |  |  | Y76G2A.2 |  |
|  |  |  |  |  |  |  |  |  |  |  |  |  |  |  |  |  |  |  |  |  |  |  |  |  |  |  |  |  |  |  |  |  |  |  |  |  |  | F01F1.16 |  |
|  |  |  |  |  |  |  |  |  |  |  |  |  |  |  |  |  |  |  |  |  |  |  |  |  |  |  |  |  |  |  |  |  |  |  |  |  |  | *srj-20* | Serpentine Receptor, class J |
|  |  |  |  |  |  |  |  |  |  |  |  |  |  |  |  |  |  |  |  |  |  |  |  |  |  |  |  |  |  |  |  |  |  |  |  |  |  | *srh-149* | Serpentine Receptor, class H |
|  |  |  |  |  |  |  |  |  |  |  |  |  |  |  |  |  |  |  |  |  |  |  |  |  |  |  |  |  |  |  |  |  |  |  |  |  |  | T22E6.1 |  |
|  |  |  |  |  |  |  |  |  |  |  |  |  |  |  |  |  |  |  |  |  |  |  |  |  |  |  |  |  |  |  |  |  |  |  |  |  |  | K11E4.9 |  |
|  |  |  |  |  |  |  |  |  |  |  |  |  |  |  |  |  |  |  |  |  |  |  |  |  |  |  |  |  |  |  |  |  |  |  |  |  |  | C40C9.16 |  |
|  |  |  |  |  |  |  |  |  |  |  |  |  |  |  |  |  |  |  |  |  |  |  |  |  |  |  |  |  |  |  |  |  |  |  |  |  |  | C40C9.13 |  |
|  |  |  |  |  |  |  |  |  |  |  |  |  |  |  |  |  |  |  |  |  |  |  |  |  |  |  |  |  |  |  |  |  |  |  |  |  |  | R01H5.t1 |  |
|  |  |  |  |  |  |  |  |  |  |  |  |  |  |  |  |  |  |  |  |  |  |  |  |  |  |  |  |  |  |  |  |  |  |  |  |  |  | C40H5.14 |  |
|  |  |  |  |  |  |  |  |  |  |  |  |  |  |  |  |  |  |  |  |  |  |  |  |  |  |  |  |  |  |  |  |  |  |  |  |  |  | ZC506.7 |  |
|  |  |  |  |  |  |  |  |  |  |  |  |  |  |  |  |  |  |  |  |  |  |  |  |  |  |  |  |  |  |  |  |  |  |  |  |  |  | H22K11.11 |  |
|  |  |  |  |  |  |  |  |  |  |  |  |  |  |  |  |  |  |  |  |  |  |  |  |  |  |  |  |  |  |  |  |  |  |  |  |  |  | C14F11.28 |  |
|  |  |  |  |  |  |  |  |  |  |  |  |  |  |  |  |  |  |  |  |  |  |  |  |  |  |  |  |  |  |  |  |  |  |  |  |  |  | C38C5.3 |  |
|  |  |  |  |  |  |  |  |  |  |  |  |  |  |  |  |  |  |  |  |  |  |  |  |  |  |  |  |  |  |  |  |  |  |  |  |  |  | C41A3.3 |  |
|  |  |  |  |  |  |  |  |  |  |  |  |  |  |  |  |  |  |  |  |  |  |  |  |  |  |  |  |  |  |  |  |  |  |  |  |  |  | C24A8.8 |  |
|  |  |  |  |  |  |  |  |  |  |  |  |  |  |  |  |  |  |  |  |  |  |  |  |  |  |  |  |  |  |  |  |  |  |  |  |  |  | F11D5.25 |  |
|  |  |  |  |  |  |  |  |  |  |  |  |  |  |  |  |  |  |  |  |  |  |  |  |  |  |  |  |  |  |  |  |  |  |  |  |  |  | C09B7.10 |  |
|  |  |  |  |  |  |  |  |  |  |  |  |  |  |  |  |  |  |  |  |  |  |  |  |  |  |  |  |  |  |  |  |  |  |  |  |  |  | T14F9.16 |  |
|  |  |  |  |  |  |  |  |  |  |  |  |  |  |  |  |  |  |  |  |  |  |  |  |  |  |  |  |  |  |  |  |  |  |  |  |  |  | Y75D11A.6 |  |
|  |  |  |  |  |  |  |  |  |  |  |  |  |  |  |  |  |  |  |  |  |  |  |  |  |  |  |  |  |  |  |  |  |  |  |  |  |  | ZK1193.9 |  |
|  |  |  |  |  |  |  |  |  |  |  |  |  |  |  |  |  |  |  |  |  |  |  |  |  |  |  |  |  |  |  |  |  |  |  |  |  |  | Y73B3A.t1 |  |
|  |  |  |  |  |  |  |  |  |  |  |  |  |  |  |  |  |  |  |  |  |  |  |  |  |  |  |  |  |  |  |  |  |  |  |  |  |  | Y39B6A.t10 |  |
|  |  |  |  |  |  |  |  |  |  |  |  |  |  |  |  |  |  |  |  |  |  |  |  |  |  |  |  |  |  |  |  |  |  |  |  |  |  | ZK228.10 |  |
|  |  |  |  |  |  |  |  |  |  |  |  |  |  |  |  |  |  |  |  |  |  |  |  |  |  |  |  |  |  |  |  |  |  |  |  |  |  | Y51A2B.8 |  |
|  |  |  |  |  |  |  |  |  |  |  |  |  |  |  |  |  |  |  |  |  |  |  |  |  |  |  |  |  |  |  |  |  |  |  |  |  |  | Y51A2A.15 |  |
|  |  |  |  |  |  |  |  |  |  |  |  |  |  |  |  |  |  |  |  |  |  |  |  |  |  |  |  |  |  |  |  |  |  |  |  |  |  | Y59A8B.33 |  |
|  |  |  |  |  |  |  |  |  |  |  |  |  |  |  |  |  |  |  |  |  |  |  |  |  |  |  |  |  |  |  |  |  |  |  |  |  |  | W06D12.t2 |  |
|  |  |  |  |  |  |  |  |  |  |  |  |  |  |  |  |  |  |  |  |  |  |  |  |  |  |  |  |  |  |  |  |  |  |  |  |  |  | F40G12.13 |  |
|  |  |  |  |  |  |  |  |  |  |  |  |  |  |  |  |  |  |  |  |  |  |  |  |  |  |  |  |  |  |  |  |  |  |  |  |  |  | W04D2.12 |  |
|  |  |  |  |  |  |  |  |  |  |  |  |  |  |  |  |  |  |  |  |  |  |  |  |  |  |  |  |  |  |  |  |  |  |  |  |  |  | R04F11.26 |  |
|  |  |  |  |  |  |  |  |  |  |  |  |  |  |  |  |  |  |  |  |  |  |  |  |  |  |  |  |  |  |  |  |  |  |  |  |  |  | ZK836.6 |  |
|  |  |  |  |  |  |  |  |  |  |  |  |  |  |  |  |  |  |  |  |  |  |  |  |  |  |  |  |  |  |  |  |  |  |  |  |  |  | F29F11.13 |  |
|  |  |  |  |  |  |  |  |  |  |  |  |  |  |  |  |  |  |  |  |  |  |  |  |  |  |  |  |  |  |  |  |  |  |  |  |  |  | F13H6.25 |  |
|  |  |  |  |  |  |  |  |  |  |  |  |  |  |  |  |  |  |  |  |  |  |  |  |  |  |  |  |  |  |  |  |  |  |  |  |  |  | F13H6.14 |  |
|  |  |  |  |  |  |  |  |  |  |  |  |  |  |  |  |  |  |  |  |  |  |  |  |  |  |  |  |  |  |  |  |  |  |  |  |  |  | F31F7.8 |  |
|  |  |  |  |  |  |  |  |  |  |  |  |  |  |  |  |  |  |  |  |  |  |  |  |  |  |  |  |  |  |  |  |  |  |  |  |  |  | C10F3.16 |  |
|  |  |  |  |  |  |  |  |  |  |  |  |  |  |  |  |  |  |  |  |  |  |  |  |  |  |  |  |  |  |  |  |  |  |  |  |  |  | Y32G9A.12 |  |
|  |  |  |  |  |  |  |  |  |  |  |  |  |  |  |  |  |  |  |  |  |  |  |  |  |  |  |  |  |  |  |  |  |  |  |  |  |  | Y39D8B.4 |  |
|  |  |  |  |  |  |  |  |  |  |  |  |  |  |  |  |  |  |  |  |  |  |  |  |  |  |  |  |  |  |  |  |  |  |  |  |  |  | Y45F10B.60 |  |
|  |  |  |  |  |  |  |  |  |  |  |  |  |  |  |  |  |  |  |  |  |  |  |  |  |  |  |  |  |  |  |  |  |  |  |  |  |  | T04A11.14 |  |
|  |  |  |  |  |  |  |  |  |  |  |  |  |  |  |  |  |  |  |  |  |  |  |  |  |  |  |  |  |  |  |  |  |  |  |  |  |  | F35G2.8 |  |
|  |  |  |  |  |  |  |  |  |  |  |  |  |  |  |  |  |  |  |  |  |  |  |  |  |  |  |  |  |  |  |  |  |  |  |  |  |  | F25H8.13 |  |
|  |  |  |  |  |  |  |  |  |  |  |  |  |  |  |  |  |  |  |  |  |  |  |  |  |  |  |  |  |  |  |  |  |  |  |  |  |  | K07F5.7 |  |
|  |  |  |  |  |  |  |  |  |  |  |  |  |  |  |  |  |  |  |  |  |  |  |  |  |  |  |  |  |  |  |  |  |  |  |  |  |  | T05A1.17 |  |
|  |  |  |  |  |  |  |  |  |  |  |  |  |  |  |  |  |  |  |  |  |  |  |  |  |  |  |  |  |  |  |  |  |  |  |  |  |  | C53D6.21 |  |
|  |  |  |  |  |  |  |  |  |  |  |  |  |  |  |  |  |  |  |  |  |  |  |  |  |  |  |  |  |  |  |  |  |  |  |  |  |  | M03D4.76 |  |
|  |  |  |  |  |  |  |  |  |  |  |  |  |  |  |  |  |  |  |  |  |  |  |  |  |  |  |  |  |  |  |  |  |  |  |  |  |  | B0547.2 |  |
|  |  |  |  |  |  |  |  |  |  |  |  |  |  |  |  |  |  |  |  |  |  |  |  |  |  |  |  |  |  |  |  |  |  |  |  |  |  | F28E10.10 |  |
|  |  |  |  |  |  |  |  |  |  |  |  |  |  |  |  |  |  |  |  |  |  |  |  |  |  |  |  |  |  |  |  |  |  |  |  |  |  | Y37E11C.3 |  |
|  |  |  |  |  |  |  |  |  |  |  |  |  |  |  |  |  |  |  |  |  |  |  |  |  |  |  |  |  |  |  |  |  |  |  |  |  |  | F56B3.13 |  |
|  |  |  |  |  |  |  |  |  |  |  |  |  |  |  |  |  |  |  |  |  |  |  |  |  |  |  |  |  |  |  |  |  |  |  |  |  |  | F45G2.12 |  |
|  |  |  |  |  |  |  |  |  |  |  |  |  |  |  |  |  |  |  |  |  |  |  |  |  |  |  |  |  |  |  |  |  |  |  |  |  |  | Y39A1B.8 |  |
|  |  |  |  |  |  |  |  |  |  |  |  |  |  |  |  |  |  |  |  |  |  |  |  |  |  |  |  |  |  |  |  |  |  |  |  |  |  | B0393.15 |  |
|  |  |  |  |  |  |  |  |  |  |  |  |  |  |  |  |  |  |  |  |  |  |  |  |  |  |  |  |  |  |  |  |  |  |  |  |  |  | F45H7.t1 |  |
|  |  |  |  |  |  |  |  |  |  |  |  |  |  |  |  |  |  |  |  |  |  |  |  |  |  |  |  |  |  |  |  |  |  |  |  |  |  | Y39A3CL.15 |  |
|  |  |  |  |  |  |  |  |  |  |  |  |  |  |  |  |  |  |  |  |  |  |  |  |  |  |  |  |  |  |  |  |  |  |  |  |  |  | H14E04.4 |  |
|  |  |  |  |  |  |  |  |  |  |  |  |  |  |  |  |  |  |  |  |  |  |  |  |  |  |  |  |  |  |  |  |  |  |  |  |  |  | F18A11.11 |  |
|  |  |  |  |  |  |  |  |  |  |  |  |  |  |  |  |  |  |  |  |  |  |  |  |  |  |  |  |  |  |  |  |  |  |  |  |  |  | M176.14 |  |
|  |  |  |  |  |  |  |  |  |  |  |  |  |  |  |  |  |  |  |  |  |  |  |  |  |  |  |  |  |  |  |  |  |  |  |  |  |  | T22C8.t1 |  |
|  |  |  |  |  |  |  |  |  |  |  |  |  |  |  |  |  |  |  |  |  |  |  |  |  |  |  |  |  |  |  |  |  |  |  |  |  |  | K01C8.11 |  |
|  |  |  |  |  |  |  |  |  |  |  |  |  |  |  |  |  |  |  |  |  |  |  |  |  |  |  |  |  |  |  |  |  |  |  |  |  |  | D2062.6 |  |
|  |  |  |  |  |  |  |  |  |  |  |  |  |  |  |  |  |  |  |  |  |  |  |  |  |  |  |  |  |  |  |  |  |  |  |  |  |  | W09G3.15 |  |
|  |  |  |  |  |  |  |  |  |  |  |  |  |  |  |  |  |  |  |  |  |  |  |  |  |  |  |  |  |  |  |  |  |  |  |  |  |  | R06C1.10 |  |
|  |  |  |  |  |  |  |  |  |  |  |  |  |  |  |  |  |  |  |  |  |  |  |  |  |  |  |  |  |  |  |  |  |  |  |  |  |  | W04G5.12 |  |
|  |  |  |  |  |  |  |  |  |  |  |  |  |  |  |  |  |  |  |  |  |  |  |  |  |  |  |  |  |  |  |  |  |  |  |  |  |  | T24D1.6 |  |
|  |  |  |  |  |  |  |  |  |  |  |  |  |  |  |  |  |  |  |  |  |  |  |  |  |  |  |  |  |  |  |  |  |  |  |  |  |  | Y69H2.t1 |  |
|  |  |  |  |  |  |  |  |  |  |  |  |  |  |  |  |  |  |  |  |  |  |  |  |  |  |  |  |  |  |  |  |  |  |  |  |  |  | F32B5.10 |  |
|  |  |  |  |  |  |  |  |  |  |  |  |  |  |  |  |  |  |  |  |  |  |  |  |  |  |  |  |  |  |  |  |  |  |  |  |  |  | F39E9.15 |  |
|  |  |  |  |  |  |  |  |  |  |  |  |  |  |  |  |  |  |  |  |  |  |  |  |  |  |  |  |  |  |  |  |  |  |  |  |  |  | F10D11.7 |  |
|  |  |  |  |  |  |  |  |  |  |  |  |  |  |  |  |  |  |  |  |  |  |  |  |  |  |  |  |  |  |  |  |  |  |  |  |  |  | Y105C5B.17 |  |
|  |  |  |  |  |  |  |  |  |  |  |  |  |  |  |  |  |  |  |  |  |  |  |  |  |  |  |  |  |  |  |  |  |  |  |  |  |  | ZK721.6 |  |
|  |  |  |  |  |  |  |  |  |  |  |  |  |  |  |  |  |  |  |  |  |  |  |  |  |  |  |  |  |  |  |  |  |  |  |  |  |  | K09C6.9 |  |
|  |  |  |  |  |  |  |  |  |  |  |  |  |  |  |  |  |  |  |  |  |  |  |  |  |  |  |  |  |  |  |  |  |  |  |  |  |  | B0344.3 |  |
|  |  |  |  |  |  |  |  |  |  |  |  |  |  |  |  |  |  |  |  |  |  |  |  |  |  |  |  |  |  |  |  |  |  |  |  |  |  | *str-226* | Seven TM Receptor |
|  |  |  |  |  |  |  |  |  |  |  |  |  |  |  |  |  |  |  |  |  |  |  |  |  |  |  |  |  |  |  |  |  |  |  |  |  |  | *srh-154* | Serpentine Receptor, class H |
|  |  |  |  |  |  |  |  |  |  |  |  |  |  |  |  |  |  |  |  |  |  |  |  |  |  |  |  |  |  |  |  |  |  |  |  |  |  | Y51H7BR.5 |  |
|  |  |  |  |  |  |  |  |  |  |  |  |  |  |  |  |  |  |  |  |  |  |  |  |  |  |  |  |  |  |  |  |  |  |  |  |  |  | *srw-98* | Serpentine Receptor, class W |
|  |  |  |  |  |  |  |  |  |  |  |  |  |  |  |  |  |  |  |  |  |  |  |  |  |  |  |  |  |  |  |  |  |  |  |  |  |  | F45E10.2 |  |
|  |  |  |  |  |  |  |  |  |  |  |  |  |  |  |  |  |  |  |  |  |  |  |  |  |  |  |  |  |  |  |  |  |  |  |  |  |  | C46F11.8 |  |
|  |  |  |  |  |  |  |  |  |  |  |  |  |  |  |  |  |  |  |  |  |  |  |  |  |  |  |  |  |  |  |  |  |  |  |  |  |  | *plst-1* | PLaSTin (actin bundling protein) homolog |
|  |  |  |  |  |  |  |  |  |  |  |  |  |  |  |  |  |  |  |  |  |  |  |  |  |  |  |  |  |  |  |  |  |  |  |  |  |  | C17H11.1 |  |
|  |  |  |  |  |  |  |  |  |  |  |  |  |  |  |  |  |  |  |  |  |  |  |  |  |  |  |  |  |  |  |  |  |  |  |  |  |  | *cah-2* | Carbonic AnHydrase |
|  |  |  |  |  |  |  |  |  |  |  |  |  |  |  |  |  |  |  |  |  |  |  |  |  |  |  |  |  |  |  |  |  |  |  |  |  |  | H35B03.1 |  |
|  |  |  |  |  |  |  |  |  |  |  |  |  |  |  |  |  |  |  |  |  |  |  |  |  |  |  |  |  |  |  |  |  |  |  |  |  |  | *clp-1* | CaLPain family |
|  |  |  |  |  |  |  |  |  |  |  |  |  |  |  |  |  |  |  |  |  |  |  |  |  |  |  |  |  |  |  |  |  |  |  |  |  |  | *lgc-53* | Ligand-Gated ion Channel |
|  |  |  |  |  |  |  |  |  |  |  |  |  |  |  |  |  |  |  |  |  |  |  |  |  |  |  |  |  |  |  |  |  |  |  |  |  |  | *glna-3* | GLutamiNAse |
|  |  |  |  |  |  |  |  |  |  |  |  |  |  |  |  |  |  |  |  |  |  |  |  |  |  |  |  |  |  |  |  |  |  |  |  |  |  | *npr-17* | NeuroPeptide Receptor family |

### Phenotypes enriched

none found

### Anatomy terms enriched

none found

### GO terms enriched

none found

### Expression clusters enriched

none found

### Motifs enriched

|  |  |  |  |  |  |
| --- | --- | --- | --- | --- | --- |
| **Motif** | **Logo** | **Possible orthologs** | **Number of motifs in cluster** | **Enrichment** | **FDR corrected p** |
| MA0262.1 |  | mab-3 | 44 | 1.89 | 0.00058 |
| Six6\_2267 |  | ceh-34 | 36 | 2.11 | 0.00064 |
| Irx3\_2226 |  | irx-1 | 10 | 6.02 | 0.00120 |
| IRX2\_1 |  | irx-1 | 32 | 2.17 | 0.00140 |
| NKX22\_si |  | ceh-24 (0.68) ceh-22 dsc-1 | 33 | 2.03 | 0.00330 |
| Pbx1\_3203 |  | ceh-20 | 42 | 1.78 | 0.00350 |
| Six2\_2307 |  | ceh-32 ceh-34 | 32 | 2.06 | 0.00350 |
| pTH8330 |  | C34D1.1 | 31 | 2.08 | 0.00410 |
| Six1\_0935 |  | ceh-32 | 28 | 2.19 | 0.00460 |
| MEF2B\_1 |  | mef-2 | 14 | 3.53 | 0.00500 |
| pTH9247 |  | dmd-3 C34D1.1 | 31 | 2.04 | 0.00560 |
| ETS2\_f1 |  | lin-1 | 42 | 1.74 | 0.00600 |
| MA0481.1 |  | daf-16 fkh-7 lin-31 | 42 | 1.72 | 0.00750 |
| HeLa-S3\_ZNF274\_UCD |  | C28G1.4 | 29 | 2.03 | 0.01000 |
| HXC8\_f1 |  | lin-39 | 31 | 1.95 | 0.01000 |
| V$HOX13\_01 |  | lin-39 | 8 | 5.53 | 0.01100 |
| Six3\_1732 |  | ceh-34 | 38 | 1.76 | 0.01100 |
| V$DELTAEF1\_01 |  | ztf-6 hlh-2 | 34 | 1.86 | 0.01100 |
| pTH9261 |  | dmd-3 | 20 | 2.48 | 0.01200 |
| FOXJ2\_f1 |  | lin-31 | 41 | 1.70 | 0.01200 |
| MA0543.1 |  | eor-1 | 31 | 1.94 | 0.01200 |
| pTH5437 |  | ceh-34 | 11 | 3.89 | 0.01200 |
| Irx5\_2385 |  | irx-1 | 19 | 2.55 | 0.01200 |
| V$FAC1\_01 |  | gei-8 | 45 | 1.62 | 0.01300 |
| TBX20\_3 |  | mab-9 tbx-39 tbx-43 | 35 | 1.81 | 0.01300 |
| pTH8981 |  | pax-3 | 24 | 2.19 | 0.01400 |
| CG7386\_F10-12\_SANGER\_5\_FBgn0035691 |  | F56D1.1 | 17 | 2.71 | 0.01400 |
| pTH9879 |  | C27D6.4 | 34 | 1.83 | 0.01400 |
| pTH3046 |  | Y116A8C.22 | 32 | 1.88 | 0.01500 |
| pTH9116 |  | lin-31 | 37 | 1.74 | 0.01700 |
| pTH6423 |  | pha-2 | 14 | 3.05 | 0.01700 |
| MA0244.1 |  | C48E7.11 | 42 | 1.64 | 0.01700 |
| ISL2\_1 |  | lim-7 | 32 | 1.86 | 0.01700 |
| ELK1\_f1 |  | lin-1 | 41 | 1.66 | 0.01700 |
| PROX1\_1 |  | crh-1 ceh-26 | 31 | 1.87 | 0.02000 |
| FLI1\_f1 |  | lin-1 | 8 | 4.90 | 0.02000 |
| EGR1\_1 |  | ZC328.2 | 30 | 1.89 | 0.02100 |
| pTH9708 |  | ceh-34 | 17 | 2.58 | 0.02100 |
| ZNF75A\_1 |  | ztf-3 | 38 | 1.69 | 0.02200 |
| Irx2\_0900 |  | irx-1 | 6 | 6.78 | 0.02200 |
| Sox4 |  | pop-1 | 38 | 1.69 | 0.02200 |
| pTH5808 |  | pal-1 | 34 | 1.77 | 0.02300 |
| pTH9925 |  | ztf-11 | 26 | 2.01 | 0.02300 |
| pTH10810 |  | lsy-2 | 13 | 3.07 | 0.02400 |
| pTH9137 |  | nhr-65 | 44 | 1.58 | 0.02500 |
| MAFA\_f1 |  | F45H11.6 | 14 | 2.89 | 0.02500 |
| Hoxa7\_3750 |  | lin-39 | 29 | 1.88 | 0.02700 |
| MA0014.2 |  | pax-3 pax-2 | 42 | 1.60 | 0.02700 |
| Nkx3-1\_2923 |  | ceh-24 (0.68) | 29 | 1.88 | 0.02800 |
| tgo\_trh\_SANGER\_5\_FBgn0015014 |  | aha-1 | 35 | 1.73 | 0.02800 |
| pTH5922 |  | ceh-24 (0.68) | 21 | 2.22 | 0.02800 |
| pTH9957 |  | fkh-9 | 20 | 2.27 | 0.02800 |
| V$MYOD\_01 |  | hlh-2 ces-1 hlh-1 | 33 | 1.77 | 0.02900 |
| HXD10\_f1 |  | php-3 | 24 | 2.04 | 0.03100 |
| pTH10718 |  | egl-43 | 25 | 2.00 | 0.03100 |
| ELF3\_2 |  | C24A1.2 | 50 | 1.48 | 0.03100 |
| pTH5690 |  | ceh-32 | 26 | 1.96 | 0.03200 |
| pTH10650 |  | nhr-153 | 37 | 1.67 | 0.03200 |
| CG34031\_Cell\_FBgn0054031 |  | ceh-24 (0.68) ceh-19 | 34 | 1.73 | 0.03200 |
| rn\_SOLEXA\_5\_FBgn0259172 |  | lin-29 | 38 | 1.65 | 0.03400 |
| ETV4\_f1 |  | lin-1 | 16 | 2.53 | 0.03600 |
| pTH6508 |  | nhr-36 | 35 | 1.70 | 0.03600 |
| ARNT2\_si |  | aha-1 | 24 | 2.00 | 0.03800 |
| pTH9326 |  | nhr-122 | 32 | 1.75 | 0.03900 |
| V$AREB6\_02 |  | ztf-6 | 30 | 1.80 | 0.04000 |
| MA0163.1 |  | Y53H1A.2 | 39 | 1.61 | 0.04000 |
| pTH9198 |  | dmd-3 | 21 | 2.13 | 0.04000 |
| pTH9300 |  | dmd-3 | 34 | 1.70 | 0.04000 |
| FOXO6\_3 |  | daf-16 | 13 | 2.84 | 0.04100 |
| Hey\_SANGER\_5\_FBgn0027788 |  | lin-22 | 11 | 3.20 | 0.04200 |
| sqz\_SOLEXA\_5\_FBgn0010768 |  | lin-29 | 51 | 1.45 | 0.04200 |
| pTH9072 |  | klf-1 | 39 | 1.60 | 0.04500 |
| pTH9222 |  | mel-28 | 26 | 1.89 | 0.04700 |
| I$KR\_01 |  | B0310.2 | 40 | 1.58 | 0.04800 |
| pTH10024 |  | F52B5.7 | 25 | 1.92 | 0.04800 |
| UNCX\_1 |  | alr-1 | 34 | 1.68 | 0.04800 |
| MYF6\_1 |  | hlh-1 | 16 | 2.43 | 0.04900 |
| pTH9915 |  | zip-3 | 34 | 1.68 | 0.05000 |
| ECC-1\_ERALPHA\_HudsonAlpha |  | nhr-71 | 38 | 1.60 | 0.05000 |

### Correlated (and anti-correlated) transcription factors

|  |  |
| --- | --- |
| **Transcription factor** | **Correlation** |
| aptf-1 | 0.77 |
| ctbp-1 | 0.75 |
| ZK337.2 | 0.74 |
| egl-13 | 0.73 |
| nhr-1 | 0.73 |
| med-2 | 0.73 |
| F26H9.2 | 0.72 |
| sta-1 | 0.71 |
| sem-4 | 0.71 |
| hbl-1 | 0.70 |
| ceh-24 | 0.68 |
| zip-1 | 0.67 |
| ceh-17 | 0.66 |
| lag-1 | 0.64 |
| F26A10.2 | 0.62 |
| hlh-13 | 0.62 |
| miz-1 | 0.62 |
| daf-3 | 0.62 |
| nhr-20 | 0.61 |
| nhr-25 | 0.61 |
| T10D4.6 | 0.60 |
| Y17G7B.22 | 0.60 |
| atf-6 | 0.59 |
| unc-130 | 0.59 |
| tag-68 | 0.59 |
| nhr-90 | -0.36 |
| hlh-29 | -0.36 |
| zip-8 | -0.36 |
| sma-4 | -0.37 |
| Y53F4B.3 | -0.37 |
| sptf-1 | -0.37 |
| dhhc-13 | -0.38 |
| lin-32 | -0.38 |
| Y48G9A.11 | -0.38 |
| klf-1 | -0.39 |
| ceh-81 | -0.39 |
| hmg-11 | -0.39 |
| spe-44 | -0.40 |
| zip-7 | -0.41 |
| nhr-271 | -0.43 |
| mxl-2 | -0.43 |
| C01F6.9 | -0.44 |
| mxl-1 | -0.45 |
| sup-35 | -0.46 |
| nhr-210 | -0.47 |
| cebp-2 | -0.47 |
| D2030.7 | -0.47 |
| Y56A3A.18 | -0.48 |
| hmg-6 | -0.58 |
| T26A5.8 | -0.60 |

### ChIP peaks enriched

|  |  |  |  |  |
| --- | --- | --- | --- | --- |
| **Gene** | **Experiment** | **Number of upstream peaks** | **Enrichment** | **FDR corrected p** |
| nhr-77 | NHR-77\_Larvae-L4-stage | 5 | 10.44 | 0.0051 |
| sax-3 | SAX-3\_Larvae-L4-stage | 5 | 9.87 | 0.0064 |
| pha-4 | PHA-4\_Larvae-L4-stage | 4 | 11.27 | 0.0160 |
