# Supplementary material for: Overlapping cell population expression profiling and regulatory inference in C. elegans
Source: BMC Genomics. 2016 Feb 29;17:159. doi: 10.1186/s12864-016-2482-z (PMC4772325; doi:10.1186/s12864-016-2482-z)
Supplement: Additional file 13: — Web supplement. (DOC 21 kb) [file 12864_2016_2482_MOESM13_ESM.zip › sortWeb/clusters/hier.300.clusters/257.html]

Cluster 257 

## Cluster 257

### Expression

| cnd-1 rep. 1 | cnd-1 rep. 2 | cnd-1 rep. 3 | pha-4 rep. 1 | pha-4 rep. 2 | pha-4 rep. 3 | ceh-27 | ceh-36 | ceh-6 | F21D5.9 | mir-57 | mls-2 | pal-1 | pros-1 | ttx-3 | unc-130 | hlh-16 | irx-1 | ceh-6 (+) hlh-16 (+) | ceh-6 (+) hlh-16 (-) | ceh-6 (-) hlh-16 (+) | cnd-1 singlets | pha-4 singlets | 0 | 60 | 120 | 150 | 180 | 240 | 330 | 390 | 420 | 480 | 540 | 570 | 600 | 630 | 660 | NAME | Functional description |
| --- | --- | --- | --- | --- | --- | --- | --- | --- | --- | --- | --- | --- | --- | --- | --- | --- | --- | --- | --- | --- | --- | --- | --- | --- | --- | --- | --- | --- | --- | --- | --- | --- | --- | --- | --- | --- | --- | --- | --- |
|  |  |  |  |  |  |  |  |  |  |  |  |  |  |  |  |  |  |  |  |  |  |  |  |  |  |  |  |  |  |  |  |  |  |  |  |  |  | Y45G5AM.3 |  |
|  |  |  |  |  |  |  |  |  |  |  |  |  |  |  |  |  |  |  |  |  |  |  |  |  |  |  |  |  |  |  |  |  |  |  |  |  |  | F40E3.5 |  |
|  |  |  |  |  |  |  |  |  |  |  |  |  |  |  |  |  |  |  |  |  |  |  |  |  |  |  |  |  |  |  |  |  |  |  |  |  |  | *abu-8* | Activated in Blocked Unfolded protein response |
|  |  |  |  |  |  |  |  |  |  |  |  |  |  |  |  |  |  |  |  |  |  |  |  |  |  |  |  |  |  |  |  |  |  |  |  |  |  | *hmit-1.3* | H(+) MyoInositol coTransporter |
|  |  |  |  |  |  |  |  |  |  |  |  |  |  |  |  |  |  |  |  |  |  |  |  |  |  |  |  |  |  |  |  |  |  |  |  |  |  | R02D5.3 |  |
|  |  |  |  |  |  |  |  |  |  |  |  |  |  |  |  |  |  |  |  |  |  |  |  |  |  |  |  |  |  |  |  |  |  |  |  |  |  | F54H5.5 |  |
|  |  |  |  |  |  |  |  |  |  |  |  |  |  |  |  |  |  |  |  |  |  |  |  |  |  |  |  |  |  |  |  |  |  |  |  |  |  | C12D12.1 |  |
|  |  |  |  |  |  |  |  |  |  |  |  |  |  |  |  |  |  |  |  |  |  |  |  |  |  |  |  |  |  |  |  |  |  |  |  |  |  | C18B2.5 |  |
|  |  |  |  |  |  |  |  |  |  |  |  |  |  |  |  |  |  |  |  |  |  |  |  |  |  |  |  |  |  |  |  |  |  |  |  |  |  | *srd-5* | Serpentine Receptor, class D (delta) |
|  |  |  |  |  |  |  |  |  |  |  |  |  |  |  |  |  |  |  |  |  |  |  |  |  |  |  |  |  |  |  |  |  |  |  |  |  |  | B0563.5 |  |
|  |  |  |  |  |  |  |  |  |  |  |  |  |  |  |  |  |  |  |  |  |  |  |  |  |  |  |  |  |  |  |  |  |  |  |  |  |  | *sra-35* | Serpentine Receptor, class A (alpha) |
|  |  |  |  |  |  |  |  |  |  |  |  |  |  |  |  |  |  |  |  |  |  |  |  |  |  |  |  |  |  |  |  |  |  |  |  |  |  | Y42A5A.3 |  |
|  |  |  |  |  |  |  |  |  |  |  |  |  |  |  |  |  |  |  |  |  |  |  |  |  |  |  |  |  |  |  |  |  |  |  |  |  |  | H37A05.2 |  |
|  |  |  |  |  |  |  |  |  |  |  |  |  |  |  |  |  |  |  |  |  |  |  |  |  |  |  |  |  |  |  |  |  |  |  |  |  |  | T08H10.3 |  |
|  |  |  |  |  |  |  |  |  |  |  |  |  |  |  |  |  |  |  |  |  |  |  |  |  |  |  |  |  |  |  |  |  |  |  |  |  |  | C29G2.3 |  |
|  |  |  |  |  |  |  |  |  |  |  |  |  |  |  |  |  |  |  |  |  |  |  |  |  |  |  |  |  |  |  |  |  |  |  |  |  |  | T05B11.4 |  |
|  |  |  |  |  |  |  |  |  |  |  |  |  |  |  |  |  |  |  |  |  |  |  |  |  |  |  |  |  |  |  |  |  |  |  |  |  |  | T21D12.12 |  |
|  |  |  |  |  |  |  |  |  |  |  |  |  |  |  |  |  |  |  |  |  |  |  |  |  |  |  |  |  |  |  |  |  |  |  |  |  |  | *klf-2* | Kruppel-Like Factor (zinc finger protein) |
|  |  |  |  |  |  |  |  |  |  |  |  |  |  |  |  |  |  |  |  |  |  |  |  |  |  |  |  |  |  |  |  |  |  |  |  |  |  | ZK675.4 |  |
|  |  |  |  |  |  |  |  |  |  |  |  |  |  |  |  |  |  |  |  |  |  |  |  |  |  |  |  |  |  |  |  |  |  |  |  |  |  | *pes-8* | Patterned Expression Site |
|  |  |  |  |  |  |  |  |  |  |  |  |  |  |  |  |  |  |  |  |  |  |  |  |  |  |  |  |  |  |  |  |  |  |  |  |  |  | *ptd-2* | disPaTcheD family |
|  |  |  |  |  |  |  |  |  |  |  |  |  |  |  |  |  |  |  |  |  |  |  |  |  |  |  |  |  |  |  |  |  |  |  |  |  |  | H01M10.2 |  |
|  |  |  |  |  |  |  |  |  |  |  |  |  |  |  |  |  |  |  |  |  |  |  |  |  |  |  |  |  |  |  |  |  |  |  |  |  |  | K04G2.9 |  |
|  |  |  |  |  |  |  |  |  |  |  |  |  |  |  |  |  |  |  |  |  |  |  |  |  |  |  |  |  |  |  |  |  |  |  |  |  |  | *osm-11* | OSMotic avoidance abnormal |
|  |  |  |  |  |  |  |  |  |  |  |  |  |  |  |  |  |  |  |  |  |  |  |  |  |  |  |  |  |  |  |  |  |  |  |  |  |  | F13H10.1 |  |
|  |  |  |  |  |  |  |  |  |  |  |  |  |  |  |  |  |  |  |  |  |  |  |  |  |  |  |  |  |  |  |  |  |  |  |  |  |  | C02F5.14 |  |
|  |  |  |  |  |  |  |  |  |  |  |  |  |  |  |  |  |  |  |  |  |  |  |  |  |  |  |  |  |  |  |  |  |  |  |  |  |  | C25D7.15 |  |
|  |  |  |  |  |  |  |  |  |  |  |  |  |  |  |  |  |  |  |  |  |  |  |  |  |  |  |  |  |  |  |  |  |  |  |  |  |  | *twk-45* | TWiK family of potassium channels |
|  |  |  |  |  |  |  |  |  |  |  |  |  |  |  |  |  |  |  |  |  |  |  |  |  |  |  |  |  |  |  |  |  |  |  |  |  |  | *osm-7* | OSMotic avoidance abnormal |
|  |  |  |  |  |  |  |  |  |  |  |  |  |  |  |  |  |  |  |  |  |  |  |  |  |  |  |  |  |  |  |  |  |  |  |  |  |  | *gln-3* | GLutamiNe synthetase (glutamate-ammonia ligase) |
|  |  |  |  |  |  |  |  |  |  |  |  |  |  |  |  |  |  |  |  |  |  |  |  |  |  |  |  |  |  |  |  |  |  |  |  |  |  | *nas-7* | Nematode AStacin protease |
|  |  |  |  |  |  |  |  |  |  |  |  |  |  |  |  |  |  |  |  |  |  |  |  |  |  |  |  |  |  |  |  |  |  |  |  |  |  | *pkg-2* | Protein Kinase, cGMP-dependent |
|  |  |  |  |  |  |  |  |  |  |  |  |  |  |  |  |  |  |  |  |  |  |  |  |  |  |  |  |  |  |  |  |  |  |  |  |  |  | K06A9.2 |  |
|  |  |  |  |  |  |  |  |  |  |  |  |  |  |  |  |  |  |  |  |  |  |  |  |  |  |  |  |  |  |  |  |  |  |  |  |  |  | *sft-4* | SurFeiT homolog |
|  |  |  |  |  |  |  |  |  |  |  |  |  |  |  |  |  |  |  |  |  |  |  |  |  |  |  |  |  |  |  |  |  |  |  |  |  |  | F36H2.3 |  |
|  |  |  |  |  |  |  |  |  |  |  |  |  |  |  |  |  |  |  |  |  |  |  |  |  |  |  |  |  |  |  |  |  |  |  |  |  |  | *lgc-21* | Ligand-Gated ion Channel |
|  |  |  |  |  |  |  |  |  |  |  |  |  |  |  |  |  |  |  |  |  |  |  |  |  |  |  |  |  |  |  |  |  |  |  |  |  |  | *esyt-2* | Extended SYnapTotagmin homolog |
|  |  |  |  |  |  |  |  |  |  |  |  |  |  |  |  |  |  |  |  |  |  |  |  |  |  |  |  |  |  |  |  |  |  |  |  |  |  | *aakb-1* | AMP-Activated Kinase Beta subunit |
|  |  |  |  |  |  |  |  |  |  |  |  |  |  |  |  |  |  |  |  |  |  |  |  |  |  |  |  |  |  |  |  |  |  |  |  |  |  | *str-262* | Seven TM Receptor |
|  |  |  |  |  |  |  |  |  |  |  |  |  |  |  |  |  |  |  |  |  |  |  |  |  |  |  |  |  |  |  |  |  |  |  |  |  |  | *dhcr-7* | DeHydroCholesterol Reductase |
|  |  |  |  |  |  |  |  |  |  |  |  |  |  |  |  |  |  |  |  |  |  |  |  |  |  |  |  |  |  |  |  |  |  |  |  |  |  | C06A6.4 |  |
|  |  |  |  |  |  |  |  |  |  |  |  |  |  |  |  |  |  |  |  |  |  |  |  |  |  |  |  |  |  |  |  |  |  |  |  |  |  | *atf-5* | ATF (cAMP-dependent transcription factor) family |
|  |  |  |  |  |  |  |  |  |  |  |  |  |  |  |  |  |  |  |  |  |  |  |  |  |  |  |  |  |  |  |  |  |  |  |  |  |  | F40F9.10 |  |
|  |  |  |  |  |  |  |  |  |  |  |  |  |  |  |  |  |  |  |  |  |  |  |  |  |  |  |  |  |  |  |  |  |  |  |  |  |  | F18G5.6 |  |
|  |  |  |  |  |  |  |  |  |  |  |  |  |  |  |  |  |  |  |  |  |  |  |  |  |  |  |  |  |  |  |  |  |  |  |  |  |  | *shc-2* | SHC (Src Homology domain C-terminal) adaptor homolog |
|  |  |  |  |  |  |  |  |  |  |  |  |  |  |  |  |  |  |  |  |  |  |  |  |  |  |  |  |  |  |  |  |  |  |  |  |  |  | *hsp-12.2* | Heat Shock Protein |
|  |  |  |  |  |  |  |  |  |  |  |  |  |  |  |  |  |  |  |  |  |  |  |  |  |  |  |  |  |  |  |  |  |  |  |  |  |  | *kqt-1* | potassium channel, KvQLT family |
|  |  |  |  |  |  |  |  |  |  |  |  |  |  |  |  |  |  |  |  |  |  |  |  |  |  |  |  |  |  |  |  |  |  |  |  |  |  | *set-30* | SET (trithorax/polycomb) domain containing |
|  |  |  |  |  |  |  |  |  |  |  |  |  |  |  |  |  |  |  |  |  |  |  |  |  |  |  |  |  |  |  |  |  |  |  |  |  |  | *unc-96* | UNCoordinated |
|  |  |  |  |  |  |  |  |  |  |  |  |  |  |  |  |  |  |  |  |  |  |  |  |  |  |  |  |  |  |  |  |  |  |  |  |  |  | Y71H2AM.15 |  |
|  |  |  |  |  |  |  |  |  |  |  |  |  |  |  |  |  |  |  |  |  |  |  |  |  |  |  |  |  |  |  |  |  |  |  |  |  |  | *unc-45* | UNCoordinated |
|  |  |  |  |  |  |  |  |  |  |  |  |  |  |  |  |  |  |  |  |  |  |  |  |  |  |  |  |  |  |  |  |  |  |  |  |  |  | F28H1.4 |  |
|  |  |  |  |  |  |  |  |  |  |  |  |  |  |  |  |  |  |  |  |  |  |  |  |  |  |  |  |  |  |  |  |  |  |  |  |  |  | *tag-163* | Temporarily Assigned Gene name |
|  |  |  |  |  |  |  |  |  |  |  |  |  |  |  |  |  |  |  |  |  |  |  |  |  |  |  |  |  |  |  |  |  |  |  |  |  |  | *ikb-1* | I Kappa B homolog |
|  |  |  |  |  |  |  |  |  |  |  |  |  |  |  |  |  |  |  |  |  |  |  |  |  |  |  |  |  |  |  |  |  |  |  |  |  |  | *ketn-1* | KETtiN (Drosophila actin-binding) homolog |
|  |  |  |  |  |  |  |  |  |  |  |  |  |  |  |  |  |  |  |  |  |  |  |  |  |  |  |  |  |  |  |  |  |  |  |  |  |  | C06G1.5 |  |
|  |  |  |  |  |  |  |  |  |  |  |  |  |  |  |  |  |  |  |  |  |  |  |  |  |  |  |  |  |  |  |  |  |  |  |  |  |  | *gar-3* | G-protein-linked Acetylcholine Receptor |
|  |  |  |  |  |  |  |  |  |  |  |  |  |  |  |  |  |  |  |  |  |  |  |  |  |  |  |  |  |  |  |  |  |  |  |  |  |  | F44A2.5 |  |
|  |  |  |  |  |  |  |  |  |  |  |  |  |  |  |  |  |  |  |  |  |  |  |  |  |  |  |  |  |  |  |  |  |  |  |  |  |  | Y18D10A.8 |  |
|  |  |  |  |  |  |  |  |  |  |  |  |  |  |  |  |  |  |  |  |  |  |  |  |  |  |  |  |  |  |  |  |  |  |  |  |  |  | *linc-52* | Long Intervening Non-Coding RNA |
|  |  |  |  |  |  |  |  |  |  |  |  |  |  |  |  |  |  |  |  |  |  |  |  |  |  |  |  |  |  |  |  |  |  |  |  |  |  | *egal-1* | EGALitarian (Drosophila exonuclease-like) homolog |
|  |  |  |  |  |  |  |  |  |  |  |  |  |  |  |  |  |  |  |  |  |  |  |  |  |  |  |  |  |  |  |  |  |  |  |  |  |  | F47G9.4 |  |
|  |  |  |  |  |  |  |  |  |  |  |  |  |  |  |  |  |  |  |  |  |  |  |  |  |  |  |  |  |  |  |  |  |  |  |  |  |  | *lag-2* | Lin-12 And Glp-1 phenotype |
|  |  |  |  |  |  |  |  |  |  |  |  |  |  |  |  |  |  |  |  |  |  |  |  |  |  |  |  |  |  |  |  |  |  |  |  |  |  | C24A1.3 |  |
|  |  |  |  |  |  |  |  |  |  |  |  |  |  |  |  |  |  |  |  |  |  |  |  |  |  |  |  |  |  |  |  |  |  |  |  |  |  | Y7A5A.1 |  |
|  |  |  |  |  |  |  |  |  |  |  |  |  |  |  |  |  |  |  |  |  |  |  |  |  |  |  |  |  |  |  |  |  |  |  |  |  |  | *sre-23* | Serpentine Receptor, class E (epsilon) |
|  |  |  |  |  |  |  |  |  |  |  |  |  |  |  |  |  |  |  |  |  |  |  |  |  |  |  |  |  |  |  |  |  |  |  |  |  |  | *nhr-12* | Nuclear Hormone Receptor family |
|  |  |  |  |  |  |  |  |  |  |  |  |  |  |  |  |  |  |  |  |  |  |  |  |  |  |  |  |  |  |  |  |  |  |  |  |  |  | Y55B1AL.1 |  |
|  |  |  |  |  |  |  |  |  |  |  |  |  |  |  |  |  |  |  |  |  |  |  |  |  |  |  |  |  |  |  |  |  |  |  |  |  |  | B0353.1 |  |
|  |  |  |  |  |  |  |  |  |  |  |  |  |  |  |  |  |  |  |  |  |  |  |  |  |  |  |  |  |  |  |  |  |  |  |  |  |  | *dep-1* | EP (Density-Enhanced Phosphatase) homolog |
|  |  |  |  |  |  |  |  |  |  |  |  |  |  |  |  |  |  |  |  |  |  |  |  |  |  |  |  |  |  |  |  |  |  |  |  |  |  | H24G06.1 |  |
|  |  |  |  |  |  |  |  |  |  |  |  |  |  |  |  |  |  |  |  |  |  |  |  |  |  |  |  |  |  |  |  |  |  |  |  |  |  | *zak-1* | mammalian ZAK kinase homolog |
|  |  |  |  |  |  |  |  |  |  |  |  |  |  |  |  |  |  |  |  |  |  |  |  |  |  |  |  |  |  |  |  |  |  |  |  |  |  | *sma-2* | SMAll |
|  |  |  |  |  |  |  |  |  |  |  |  |  |  |  |  |  |  |  |  |  |  |  |  |  |  |  |  |  |  |  |  |  |  |  |  |  |  | *fbxa-89* | F-box A protein |
|  |  |  |  |  |  |  |  |  |  |  |  |  |  |  |  |  |  |  |  |  |  |  |  |  |  |  |  |  |  |  |  |  |  |  |  |  |  | K04G11.3 |  |
|  |  |  |  |  |  |  |  |  |  |  |  |  |  |  |  |  |  |  |  |  |  |  |  |  |  |  |  |  |  |  |  |  |  |  |  |  |  | C49A9.6 |  |
|  |  |  |  |  |  |  |  |  |  |  |  |  |  |  |  |  |  |  |  |  |  |  |  |  |  |  |  |  |  |  |  |  |  |  |  |  |  | C08B6.3 |  |
|  |  |  |  |  |  |  |  |  |  |  |  |  |  |  |  |  |  |  |  |  |  |  |  |  |  |  |  |  |  |  |  |  |  |  |  |  |  | Y75B8A.3 |  |
|  |  |  |  |  |  |  |  |  |  |  |  |  |  |  |  |  |  |  |  |  |  |  |  |  |  |  |  |  |  |  |  |  |  |  |  |  |  | F35E12.8 |  |
|  |  |  |  |  |  |  |  |  |  |  |  |  |  |  |  |  |  |  |  |  |  |  |  |  |  |  |  |  |  |  |  |  |  |  |  |  |  | C15C8.4 |  |
|  |  |  |  |  |  |  |  |  |  |  |  |  |  |  |  |  |  |  |  |  |  |  |  |  |  |  |  |  |  |  |  |  |  |  |  |  |  | *irg-1* | Infection Response Gene |
|  |  |  |  |  |  |  |  |  |  |  |  |  |  |  |  |  |  |  |  |  |  |  |  |  |  |  |  |  |  |  |  |  |  |  |  |  |  | *nhr-131* | Nuclear Hormone Receptor family |
|  |  |  |  |  |  |  |  |  |  |  |  |  |  |  |  |  |  |  |  |  |  |  |  |  |  |  |  |  |  |  |  |  |  |  |  |  |  | F22H10.3 |  |
|  |  |  |  |  |  |  |  |  |  |  |  |  |  |  |  |  |  |  |  |  |  |  |  |  |  |  |  |  |  |  |  |  |  |  |  |  |  | *best-9* | BESTrophin (chloride channel) homolog |
|  |  |  |  |  |  |  |  |  |  |  |  |  |  |  |  |  |  |  |  |  |  |  |  |  |  |  |  |  |  |  |  |  |  |  |  |  |  | *gpa-17* | G Protein, Alpha subunit |
|  |  |  |  |  |  |  |  |  |  |  |  |  |  |  |  |  |  |  |  |  |  |  |  |  |  |  |  |  |  |  |  |  |  |  |  |  |  | *tag-343* | Temporarily Assigned Gene name |
|  |  |  |  |  |  |  |  |  |  |  |  |  |  |  |  |  |  |  |  |  |  |  |  |  |  |  |  |  |  |  |  |  |  |  |  |  |  | ZK353.2 |  |
|  |  |  |  |  |  |  |  |  |  |  |  |  |  |  |  |  |  |  |  |  |  |  |  |  |  |  |  |  |  |  |  |  |  |  |  |  |  | R10E11.9 |  |
|  |  |  |  |  |  |  |  |  |  |  |  |  |  |  |  |  |  |  |  |  |  |  |  |  |  |  |  |  |  |  |  |  |  |  |  |  |  | C04G6.5 |  |
|  |  |  |  |  |  |  |  |  |  |  |  |  |  |  |  |  |  |  |  |  |  |  |  |  |  |  |  |  |  |  |  |  |  |  |  |  |  | C49A9.9 |  |
|  |  |  |  |  |  |  |  |  |  |  |  |  |  |  |  |  |  |  |  |  |  |  |  |  |  |  |  |  |  |  |  |  |  |  |  |  |  | C47D12.5 |  |
|  |  |  |  |  |  |  |  |  |  |  |  |  |  |  |  |  |  |  |  |  |  |  |  |  |  |  |  |  |  |  |  |  |  |  |  |  |  | T04F3.2 |  |
|  |  |  |  |  |  |  |  |  |  |  |  |  |  |  |  |  |  |  |  |  |  |  |  |  |  |  |  |  |  |  |  |  |  |  |  |  |  | *kgb-1* | Kinase, GLH-Binding |
|  |  |  |  |  |  |  |  |  |  |  |  |  |  |  |  |  |  |  |  |  |  |  |  |  |  |  |  |  |  |  |  |  |  |  |  |  |  | Y105C5B.19 |  |
|  |  |  |  |  |  |  |  |  |  |  |  |  |  |  |  |  |  |  |  |  |  |  |  |  |  |  |  |  |  |  |  |  |  |  |  |  |  | EGAP9.3 |  |
|  |  |  |  |  |  |  |  |  |  |  |  |  |  |  |  |  |  |  |  |  |  |  |  |  |  |  |  |  |  |  |  |  |  |  |  |  |  | *nhr-170* | Nuclear Hormone Receptor family |
|  |  |  |  |  |  |  |  |  |  |  |  |  |  |  |  |  |  |  |  |  |  |  |  |  |  |  |  |  |  |  |  |  |  |  |  |  |  | Y38H6C.17 |  |
|  |  |  |  |  |  |  |  |  |  |  |  |  |  |  |  |  |  |  |  |  |  |  |  |  |  |  |  |  |  |  |  |  |  |  |  |  |  | M195.2 |  |
|  |  |  |  |  |  |  |  |  |  |  |  |  |  |  |  |  |  |  |  |  |  |  |  |  |  |  |  |  |  |  |  |  |  |  |  |  |  | K03A11.5 |  |
|  |  |  |  |  |  |  |  |  |  |  |  |  |  |  |  |  |  |  |  |  |  |  |  |  |  |  |  |  |  |  |  |  |  |  |  |  |  | *uba-5* | UBA (human ubiquitin) related |
|  |  |  |  |  |  |  |  |  |  |  |  |  |  |  |  |  |  |  |  |  |  |  |  |  |  |  |  |  |  |  |  |  |  |  |  |  |  | F27D4.1 |  |
|  |  |  |  |  |  |  |  |  |  |  |  |  |  |  |  |  |  |  |  |  |  |  |  |  |  |  |  |  |  |  |  |  |  |  |  |  |  | ZK669.4 |  |
|  |  |  |  |  |  |  |  |  |  |  |  |  |  |  |  |  |  |  |  |  |  |  |  |  |  |  |  |  |  |  |  |  |  |  |  |  |  | *aco-2* | ACOnitase |
|  |  |  |  |  |  |  |  |  |  |  |  |  |  |  |  |  |  |  |  |  |  |  |  |  |  |  |  |  |  |  |  |  |  |  |  |  |  | *gdh-1* | Glutamate DeHydrogenase |
|  |  |  |  |  |  |  |  |  |  |  |  |  |  |  |  |  |  |  |  |  |  |  |  |  |  |  |  |  |  |  |  |  |  |  |  |  |  | C34B4.4 |  |
|  |  |  |  |  |  |  |  |  |  |  |  |  |  |  |  |  |  |  |  |  |  |  |  |  |  |  |  |  |  |  |  |  |  |  |  |  |  | C34B4.2 |  |
|  |  |  |  |  |  |  |  |  |  |  |  |  |  |  |  |  |  |  |  |  |  |  |  |  |  |  |  |  |  |  |  |  |  |  |  |  |  | *nucb-1* | NUCleoBindin homolog |
|  |  |  |  |  |  |  |  |  |  |  |  |  |  |  |  |  |  |  |  |  |  |  |  |  |  |  |  |  |  |  |  |  |  |  |  |  |  | C37H5.2 |  |
|  |  |  |  |  |  |  |  |  |  |  |  |  |  |  |  |  |  |  |  |  |  |  |  |  |  |  |  |  |  |  |  |  |  |  |  |  |  | C50F4.1 |  |
|  |  |  |  |  |  |  |  |  |  |  |  |  |  |  |  |  |  |  |  |  |  |  |  |  |  |  |  |  |  |  |  |  |  |  |  |  |  | *odd-2* | Drosophila ODD-skipped-like |
|  |  |  |  |  |  |  |  |  |  |  |  |  |  |  |  |  |  |  |  |  |  |  |  |  |  |  |  |  |  |  |  |  |  |  |  |  |  | T25B2.1 |  |
|  |  |  |  |  |  |  |  |  |  |  |  |  |  |  |  |  |  |  |  |  |  |  |  |  |  |  |  |  |  |  |  |  |  |  |  |  |  | *acr-7* | AcetylCholine Receptor |
|  |  |  |  |  |  |  |  |  |  |  |  |  |  |  |  |  |  |  |  |  |  |  |  |  |  |  |  |  |  |  |  |  |  |  |  |  |  | *nhr-142* | Nuclear Hormone Receptor family |
|  |  |  |  |  |  |  |  |  |  |  |  |  |  |  |  |  |  |  |  |  |  |  |  |  |  |  |  |  |  |  |  |  |  |  |  |  |  | F44E7.12 |  |

### Phenotypes enriched

|  |  |  |  |
| --- | --- | --- | --- |
| **Group name** | **Number in cluster** | **Enrichment** | **FDR corrected p** |
| quiescence variant | 3 | 45.86 | 0.0335 |

### Anatomy terms enriched

none found

### GO terms enriched

none found

### Expression clusters enriched

|  |  |  |  |
| --- | --- | --- | --- |
| **Group name** | **Number in cluster** | **Enrichment** | **FDR corrected p** |
| Genes enriched in muscle cells (0hr muscle dataset). Dissociated myo-3::GFP embryos were cultured for 0 hours before FACS sorting. | 23 | 4.15 | 2.72e-06 |
| Total muscle enriched genes (complete list of non-overlapping genes from the 0hr and 24hr muscle enriched datasets). | 29 | 3.10 | 1.28e-05 |
| TGF- Dauer pathway adult transcriptional targets. Results obtained by comparing the microarray results of the dauer-constitutive mutants daf-7(e1372), daf-7(m62), and daf-1(m40) with dauer-defective mutants daf-3(mgDf90), daf-5(e1386), and daf-7(e1372);daf-3(mgDf90) double mutants at the permissive temperature, 20C, on the first day of adulthood. WBPaper00031040:TGF-beta\_adult\_upregulated | 36 | 2.28 | 3.53e-04 |
| Genes with no change in hcf-1(-), no change in sir-2.1(O/E) and upregulated in daf-2(-). | 19 | 2.88 | 7.64e-03 |
| Significantly upregulated genes from cyc-1(RNAi) microarrays using SAM algorithm with an FDR < 0.1 from adult-only chips. | 47 | 1.71 | 1.05e-02 |

### Motifs enriched

|  |  |  |  |  |  |
| --- | --- | --- | --- | --- | --- |
| **Motif** | **Logo** | **Possible orthologs** | **Number of motifs in cluster** | **Enrichment** | **FDR corrected p** |
| FOXJ2\_f1 |  | lin-31 (0.6) let-381 | 67 | 1.85 | 1.5e-06 |
| pTH5334 |  | fkh-7 (0.63) lin-31 (0.6) daf-16 fkh-10 fkh-8 let-381 | 40 | 2.62 | 2.0e-06 |
| FOXB1\_4 |  | pha-4 (0.64) fkh-7 (0.63) lin-31 (0.6) daf-16 fkh-10 let-381 fkh-8 | 56 | 2.05 | 2.5e-06 |
| V$GATA6\_01 |  | elt-1 | 45 | 2.36 | 3.1e-06 |
| Sox11\_2266 |  | pop-1 gei-3 sox-4 C05C9.3 | 23 | 3.63 | 2.5e-05 |
| pTH10707 |  | elt-3 (0.59) ceh-34 (0.54) elt-6 ceh-32 egl-27 elt-1 elt-7 | 54 | 1.94 | 2.9e-05 |
| Hr51\_SANGER\_5\_FBgn0034012 |  | pop-1 nhr-100 sox-4 | 78 | 1.56 | 3.7e-05 |
| I$MTTFA\_01 |  | hmg-5 | 41 | 2.27 | 4.3e-05 |
| NR2F6\_f1 |  | nhr-62 (0.68) nhr-239 nhr-2 | 79 | 1.54 | 4.8e-05 |
| pTH9925 |  | nhr-100 ztf-11 | 78 | 1.54 | 6.7e-05 |
| pTH3043 |  | lin-31 (0.6) fkh-10 let-381 C34D1.1 | 62 | 1.75 | 6.7e-05 |
| pTH9323 |  | nhr-28 (0.73) nhr-273 odr-7 nhr-79 slr-2 | 54 | 1.89 | 6.9e-05 |
| pTH9296 |  | ztf-6 C34D1.1 gei-11 | 55 | 1.87 | 6.9e-05 |
| pTH10808 |  | ztf-19 | 62 | 1.74 | 7.7e-05 |
| SRP000217\_Sox2 |  | ceh-6 ceh-18 sox-4 tbp-1 | 68 | 1.65 | 8.3e-05 |
| HUVEC\_GATA2\_UCD |  | elt-1 | 53 | 1.89 | 8.6e-05 |
| pTH10779 |  | nhr-134 (0.56) nhr-182 (0.55) | 66 | 1.67 | 9.2e-05 |
| GATA4\_1 |  | elt-1 end-3 | 47 | 2.01 | 1.2e-04 |
| pTH10638 |  | dmd-3 C34D1.1 | 57 | 1.79 | 1.4e-04 |
| pTH9957 |  | fkh-9 (0.79) irx-1 daf-16 | 89 | 1.40 | 1.4e-04 |
| MA0543.1 |  | daf-8 eor-1 | 84 | 1.45 | 1.6e-04 |
| MA0599.1 |  | klf-2 (0.94) klf-1 | 67 | 1.64 | 1.6e-04 |
| V$HOX13\_01 |  | lin-39 | 67 | 1.63 | 1.7e-04 |
| pTH6497 |  | lin-31 (0.6) | 60 | 1.73 | 1.9e-04 |
| So\_Cell\_FBgn0003460 |  | ztf-28 ceh-32 skn-1 | 33 | 2.44 | 2.0e-04 |
| pTH9137 |  | nhr-65 | 58 | 1.74 | 2.3e-04 |
| exd\_FlyReg\_FBgn0000611 |  | cfi-1 let-381 ceh-20 | 58 | 1.74 | 2.3e-04 |
| pTH9462 |  | mab-9 tbx-39 tbx-38 tbx-43 | 70 | 1.58 | 2.5e-04 |
| pTH6591 |  | lin-31 (0.6) | 62 | 1.68 | 2.6e-04 |
| pTH2353 |  | lin-48 (0.73) B0310.2 D1081.8 | 11 | 6.44 | 3.0e-04 |
| K562b\_GATA1\_UCD |  | elt-1 ztf-29 | 45 | 1.97 | 3.3e-04 |
| Sox15\_3457 |  | sox-4 | 15 | 4.45 | 3.3e-04 |
| pTH10629 |  | daf-12 (0.69) hif-1 ceh-9 Y5F2A.4 ztf-3 | 71 | 1.55 | 3.9e-04 |
| MA0476.1 |  | fos-1 (0.64) jun-1 sknr-1 crh-1 | 46 | 1.94 | 3.9e-04 |
| CG5669\_SOLEXA\_5\_FBgn0039169 |  | klf-2 (0.94) klf-1 | 64 | 1.63 | 4.0e-04 |
| TBX2\_f1 |  | ztf-6 tbx-39 | 72 | 1.53 | 4.2e-04 |
| pTH6106 |  | nhr-182 (0.55) | 59 | 1.69 | 4.2e-04 |
| V$FREAC7\_01 |  | lin-31 (0.6) | 52 | 1.80 | 4.6e-04 |
| V$FAC1\_01 |  | gei-8 | 87 | 1.38 | 4.8e-04 |
| FOXD2\_1 |  | let-381 hmbx-1 Y116A8C.22 | 47 | 1.90 | 4.9e-04 |
| pTH6641 |  | lin-31 (0.6) | 16 | 4.01 | 5.1e-04 |
| MA0537.1 |  | blmp-1 | 95 | 1.31 | 5.1e-04 |
| pTH9199 |  | mab-3 daf-19 | 94 | 1.32 | 6.0e-04 |
| Tcf7l2\_3461 |  | pop-1 | 73 | 1.50 | 6.4e-04 |
| pTH5828 |  | nhr-142 (0.71) nhr-84 nhr-216 | 68 | 1.56 | 6.6e-04 |
| MA0261.1 |  | nhr-255 lin-14 | 35 | 2.20 | 6.6e-04 |
| pTH1049 |  | elt-1 | 66 | 1.58 | 6.7e-04 |
| pTH9901 |  | pal-1 lin-39 D1005.3 T27F2.4 | 80 | 1.43 | 7.1e-04 |
| pTH6508 |  | nhr-36 | 40 | 2.03 | 7.2e-04 |
| NR1D1\_f1 |  | nhr-213 nhr-118 | 62 | 1.62 | 7.8e-04 |
| MA0126.1 |  | lin-48 (0.73) dmd-3 pax-3 | 57 | 1.69 | 8.0e-04 |
| CG14962\_SANGER\_5\_FBgn0035407 |  | C34H4.5 T22H9.4 | 75 | 1.47 | 8.5e-04 |
| Mf28 |  | elt-1 | 60 | 1.64 | 8.6e-04 |
| pTH9135 |  | pop-1 | 83 | 1.40 | 8.8e-04 |
| V$MEF2\_02 |  | mef-2 (0.52) | 78 | 1.44 | 9.2e-04 |
| FOXO3\_3 |  | ZC328.2 daf-16 | 62 | 1.61 | 9.3e-04 |
| NR5A2\_f1 |  | nhr-68 | 61 | 1.62 | 9.5e-04 |
| Rfx4\_3761 |  | daf-19 | 49 | 1.80 | 9.6e-04 |
| pTH5561 |  | nhr-239 | 62 | 1.60 | 1.2e-03 |
| pTH9214 |  | cfi-1 | 110 | 1.16 | 1.3e-03 |
| pTH6556 |  | odd-1 lim-6 | 56 | 1.67 | 1.3e-03 |
| ESRRA\_3 |  | nhr-10 (0.56) nhr-213 nhr-68 nhr-71 nhr-6 | 62 | 1.59 | 1.4e-03 |
| Sp4\_1011 |  | klf-2 (0.94) sptf-3 klf-1 | 35 | 2.12 | 1.4e-03 |
| pTH9880 |  | end-1 | 59 | 1.62 | 1.4e-03 |
| pTH9244 |  | tbx-39 | 47 | 1.81 | 1.5e-03 |
| V$GATA1\_02 |  | elt-1 | 59 | 1.62 | 1.6e-03 |
| Sox17\_2837 |  | sox-4 | 65 | 1.55 | 1.6e-03 |
| V$AREB6\_02 |  | ztf-6 | 59 | 1.62 | 1.6e-03 |
| Ets97D\_SANGER\_10\_FBgn0004510 |  | lin-1 | 68 | 1.51 | 1.9e-03 |
| Gata5\_3768 |  | elt-1 | 42 | 1.89 | 1.9e-03 |
| EGR3\_f1 |  | klf-2 (0.94) ZC328.2 klf-1 | 47 | 1.78 | 2.0e-03 |
| NR2E3\_f1 |  | nhr-100 lin-39 | 84 | 1.36 | 2.0e-03 |
| Meis3\_1 |  | ceh-32 ces-1 ceh-20 F55C5.11 | 69 | 1.49 | 2.1e-03 |
| MA0027.1 |  | ceh-16 | 44 | 1.84 | 2.1e-03 |
| Foxj3\_1 |  | lin-31 (0.6) | 72 | 1.46 | 2.4e-03 |
| Aef1\_FlyReg\_FBgn0005694 |  | K11D2.4 egl-13 sox-4 | 72 | 1.45 | 2.7e-03 |
| POU3F3\_3 |  | unc-86 ceh-18 | 77 | 1.41 | 2.8e-03 |
| GATA5\_f1 |  | elt-1 | 75 | 1.42 | 2.9e-03 |
| pTH1292 |  | pzf-1 | 52 | 1.67 | 3.0e-03 |
| CG2052\_SANGER\_2.5\_FBgn0039905 |  | fkh-7 (0.63) lin-29 (0.58) | 89 | 1.31 | 3.0e-03 |
| FOXI1\_f1 |  | lin-31 (0.6) | 72 | 1.45 | 3.0e-03 |
| TBX3\_f1 |  | tbx-39 | 69 | 1.47 | 3.2e-03 |
| pTH10777 |  | dmd-3 | 65 | 1.50 | 3.7e-03 |
| MA0173.1 |  | irx-1 D1081.8 | 49 | 1.70 | 3.7e-03 |
| Sox1\_3 |  | dmd-4 sox-4 | 73 | 1.43 | 4.1e-03 |
| pTH9384 |  | cfi-1 | 80 | 1.37 | 4.2e-03 |
| PBX1\_do |  | ceh-12 lin-39 ceh-20 | 60 | 1.55 | 4.2e-03 |
| TCF4\_2 |  | hlh-2 ztf-6 | 33 | 2.05 | 4.2e-03 |
| pTH2283 |  | odd-2 (0.85) | 67 | 1.48 | 4.4e-03 |
| V$CDXA\_01 |  | php-3 ceh-13 | 63 | 1.51 | 4.4e-03 |
| ELF2\_f1 |  | C24A1.2 | 68 | 1.46 | 4.6e-03 |
| pTH9080 |  | mnm-2 | 60 | 1.54 | 4.7e-03 |
| pTH8996 |  | sma-4 | 65 | 1.49 | 4.7e-03 |
| N$SKN1\_01 |  | ceh-2 (0.57) skn-1 | 66 | 1.48 | 4.7e-03 |
| PTF1A\_f1 |  | hlh-2 lin-32 | 58 | 1.57 | 4.7e-03 |
| pTH9254 |  | mel-28 | 107 | 1.17 | 4.9e-03 |
| pTH5882 |  | nhr-19 (0.59) nhr-2 | 57 | 1.57 | 4.9e-03 |
| pTH5922 |  | ceh-24 | 90 | 1.29 | 5.2e-03 |
| COT2\_f2 |  | nhr-62 (0.68) nhr-2 | 38 | 1.89 | 5.2e-03 |
| MA0253.1 |  | ceh-22 (0.66) dsc-1 (0.58) | 13 | 3.78 | 5.3e-03 |
| PBDE\_GATA1\_UCD |  | elt-1 | 35 | 1.96 | 5.3e-03 |
| HOXC10\_1 |  | php-3 hbl-1 lin-39 | 83 | 1.34 | 5.5e-03 |
| MA0151.1 |  | cfi-1 | 66 | 1.47 | 5.5e-03 |
| TFE3\_f1 |  | hlh-30 (0.67) | 51 | 1.64 | 5.5e-03 |
| Gata3\_1024 |  | elt-1 | 56 | 1.58 | 5.6e-03 |
| pTH5083 |  | atf-5 (0.76) fos-1 (0.64) | 40 | 1.83 | 5.7e-03 |
| Mv75 |  | lin-31 (0.6) elt-1 | 53 | 1.61 | 5.9e-03 |
| HLH4C\_da\_SANGER\_5\_3\_FBgn0011277 |  | hlh-1 ces-1 hlh-15 | 60 | 1.53 | 6.0e-03 |
| pTH9108 |  | daf-12 (0.69) nhr-5 | 103 | 1.20 | 6.1e-03 |
| I$CROC\_01 |  | let-381 mel-28 Y116A8C.22 | 48 | 1.68 | 6.1e-03 |
| pTH10769 |  | Y48G1C.6 | 33 | 2.00 | 6.4e-03 |
| CG4854\_SANGER\_10\_FBgn0038766 |  | mxl-1 (-0.64) K11D2.4 | 38 | 1.86 | 6.4e-03 |
| pTH5257 |  | C48E7.11 | 41 | 1.80 | 6.5e-03 |
| PDX1\_1 |  | lin-39 | 66 | 1.46 | 6.8e-03 |
| V$PAX6\_01 |  | pax-2 pax-3 | 42 | 1.77 | 7.0e-03 |
| pTH3064 |  | crh-1 | 54 | 1.59 | 7.2e-03 |
| pTH5119 |  | cfi-1 | 71 | 1.42 | 7.2e-03 |
| pTH10633 |  | R07H5.10 C48E7.11 | 83 | 1.33 | 7.2e-03 |
| MEIS1\_f2 |  | ceh-32 lin-39 | 72 | 1.41 | 7.4e-03 |
| Zfp691\_0895 |  | F21A9.2 CELE\_Y38H8A.5 | 42 | 1.76 | 7.6e-03 |
| pTH5714 |  | nhr-239 | 55 | 1.57 | 7.7e-03 |
| pTH6071 |  | C46E10.8 C33G8.2 | 59 | 1.52 | 7.8e-03 |
| pTH9915 |  | zip-3 | 61 | 1.50 | 7.9e-03 |
| pTH4337 |  | crh-1 attf-1 | 38 | 1.84 | 8.2e-03 |
| pTH10788 |  | tbx-33 | 81 | 1.34 | 8.3e-03 |
| ONECUT2\_1 |  | ceh-48 | 75 | 1.38 | 8.4e-03 |
| pTH2846 |  | lin-31 (0.6) | 30 | 2.05 | 8.8e-03 |
| ZNF75A\_1 |  | ztf-3 | 75 | 1.37 | 9.3e-03 |
| V$TCF11\_01 |  | skn-1 | 64 | 1.46 | 9.3e-03 |
| pTH8411 |  | tbx-39 | 76 | 1.37 | 9.6e-03 |
| pTH9043 |  | sem-2 | 75 | 1.37 | 1.0e-02 |
| IRX5\_1 |  | irx-1 | 19 | 2.63 | 1.0e-02 |
| pTH3998 |  | tbx-39 | 62 | 1.48 | 1.0e-02 |
| pTH5808 |  | pal-1 ceh-24 | 60 | 1.50 | 1.0e-02 |
| HeLa-S3\_ZNF274\_UCD |  | C28G1.4 | 71 | 1.40 | 1.1e-02 |
| pTH9163 |  | nhr-3 (0.58) | 77 | 1.35 | 1.1e-02 |
| ARI3A\_do |  | cfi-1 | 79 | 1.34 | 1.1e-02 |
| pTH9924 |  | nhr-46 (0.56) | 47 | 1.65 | 1.1e-02 |
| pTH9237 |  | mel-28 | 76 | 1.36 | 1.2e-02 |
| pTH10034 |  | nhr-66 | 58 | 1.51 | 1.2e-02 |
| Tcf7\_0950 |  | pop-1 | 58 | 1.51 | 1.2e-02 |
| V$TATA\_C |  | tbp-1 | 76 | 1.35 | 1.2e-02 |
| pTH5778 |  | egl-5 | 62 | 1.47 | 1.2e-02 |
| pTH9969 |  | pag-3 | 72 | 1.38 | 1.3e-02 |
| pTH3477 |  | daf-16 | 55 | 1.53 | 1.3e-02 |
| pTH10647 |  | nhr-232 | 66 | 1.42 | 1.4e-02 |
| pTH10816 |  | dmd-6 | 52 | 1.56 | 1.4e-02 |
| PURA\_f1 |  | plp-2 | 32 | 1.92 | 1.4e-02 |
| pTH9480 |  | ces-2 atf-2 | 64 | 1.44 | 1.4e-02 |
| pTH3997 |  | C04F5.9 | 31 | 1.95 | 1.4e-02 |
| MA0509.1 |  | daf-19 | 57 | 1.50 | 1.5e-02 |
| CG8765\_SANGER\_5\_FBgn0036900 |  | H20J04.3 | 74 | 1.36 | 1.5e-02 |
| Nkx3-1\_2923 |  | ceh-24 | 55 | 1.52 | 1.5e-02 |
| Sox1\_2631 |  | sox-4 | 62 | 1.45 | 1.5e-02 |
| pTH10028 |  | nhr-204 (0.58) | 61 | 1.46 | 1.5e-02 |
| MA0058.2 |  | mxl-1 (-0.64) | 60 | 1.47 | 1.6e-02 |
| pTH5250 |  | C48E7.11 | 45 | 1.64 | 1.6e-02 |
| Caup\_SOLEXA\_FBgn0015919 |  | irx-1 | 38 | 1.76 | 1.7e-02 |
| V$CEBPA\_01 |  | C48E7.11 | 92 | 1.24 | 1.8e-02 |
| pTH6486 |  | nhr-145 | 84 | 1.29 | 1.8e-02 |
| Hoxa11\_2218 |  | php-3 | 54 | 1.52 | 1.8e-02 |
| V$NKX61\_01 |  | cog-1 (0.63) lin-39 | 42 | 1.68 | 1.8e-02 |
| Tcf3\_3787 |  | pop-1 | 42 | 1.68 | 1.8e-02 |
| V$MYB\_Q6 |  | D1081.8 | 31 | 1.91 | 1.8e-02 |
| I$ABDB\_01 |  | ceh-24 | 25 | 2.13 | 1.8e-02 |
| pTH6281 |  | nhr-213 nhr-239 nhr-2 | 43 | 1.66 | 1.9e-02 |
| Spt15 |  | tbp-1 | 43 | 1.66 | 1.9e-02 |
| pTH10823 |  | B0310.2 | 58 | 1.48 | 1.9e-02 |
| GM12878\_SRF\_HudsonAlpha |  | unc-120 (0.66) | 99 | 1.19 | 2.0e-02 |
| pTH9242 |  | mel-28 | 90 | 1.25 | 2.0e-02 |
| pTH9182 |  | tbx-39 | 58 | 1.47 | 2.0e-02 |
| pTH9096 |  | T07C12.11 | 81 | 1.30 | 2.0e-02 |
| pTH10650 |  | nhr-153 (0.61) | 60 | 1.45 | 2.0e-02 |
| MA0488.1 |  | crh-1 | 34 | 1.82 | 2.1e-02 |
| pTH9907 |  | nhr-34 (0.85) | 34 | 1.82 | 2.2e-02 |
| pTH3751 |  | tbx-39 | 61 | 1.44 | 2.2e-02 |
| pTH3120 |  | K11D2.4 tbp-1 | 50 | 1.55 | 2.2e-02 |
| HXD13\_f1 |  | pal-1 mex-6 | 32 | 1.86 | 2.2e-02 |
| FOXB1\_1 |  | lin-31 (0.6) | 62 | 1.43 | 2.3e-02 |
| CG7386\_F10-12\_SANGER\_5\_FBgn0035691 |  | F56D1.1 | 58 | 1.46 | 2.3e-02 |
| Pou3f3\_3235 |  | ceh-6 | 60 | 1.45 | 2.4e-02 |
| pTH9245 |  | ceh-18 | 58 | 1.46 | 2.4e-02 |
| pTH5118 |  | cfi-1 | 64 | 1.41 | 2.4e-02 |
| NFIA\_1 |  | nfi-1 F49E12.6 | 89 | 1.25 | 2.4e-02 |
| pTH9250 |  | C34D1.1 | 57 | 1.47 | 2.5e-02 |
| EHF\_si |  | C24A1.2 | 59 | 1.45 | 2.6e-02 |
| pTH10640 |  | ceh-32 dmd-4 | 50 | 1.54 | 2.6e-02 |
| Pbx1\_3203 |  | ceh-20 | 31 | 1.86 | 2.7e-02 |
| Otx1\_1 |  | ceh-45 | 49 | 1.55 | 2.7e-02 |
| V$CEBP\_01 |  | C48E7.11 | 107 | 1.14 | 2.8e-02 |
| pTH2820 |  | ZC328.2 | 44 | 1.61 | 2.8e-02 |
| pTH9262 |  | lin-54 | 82 | 1.28 | 2.9e-02 |
| pTH9158 |  | K02D7.2 | 8 | 4.57 | 2.9e-02 |
| pTH9085 |  | nhr-42 (0.68) | 35 | 1.76 | 2.9e-02 |
| V$CREB\_02 |  | crh-1 | 85 | 1.26 | 3.0e-02 |
| ISL1\_f1 |  | lin-39 lim-7 | 49 | 1.53 | 3.2e-02 |
| MA0262.1 |  | mab-3 | 70 | 1.35 | 3.2e-02 |
| pTH9215 |  | C34D1.1 | 82 | 1.28 | 3.2e-02 |
| pTH10796 |  | hsf-1 | 111 | 1.10 | 3.2e-02 |
| rn\_SOLEXA\_5\_FBgn0259172 |  | lin-29 (0.58) | 54 | 1.48 | 3.2e-02 |
| MYF6\_1 |  | hlh-1 hlh-15 | 98 | 1.19 | 3.3e-02 |
| HXD10\_f1 |  | php-3 | 70 | 1.35 | 3.4e-02 |
| pTH5065 |  | hlh-30 (0.67) hlh-26 | 22 | 2.14 | 3.4e-02 |
| pTH9198 |  | dmd-3 | 56 | 1.46 | 3.4e-02 |
| pTH3510 |  | F13H6.1 (0.62) nhr-86 | 28 | 1.91 | 3.5e-02 |
| Hoxa7\_2668 |  | lin-39 | 99 | 1.18 | 3.5e-02 |
| pTH4325 |  | ceh-18 | 73 | 1.33 | 3.6e-02 |
| ZN384\_f1 |  | lin-29 (0.58) | 64 | 1.39 | 3.7e-02 |
| K562\_SP2\_HudsonAlpha |  | klf-2 (0.94) | 31 | 1.81 | 3.7e-02 |
| pTH9260 |  | mel-28 | 87 | 1.24 | 3.8e-02 |
| pTH9913 |  | fos-1 (0.64) skn-1 | 62 | 1.40 | 3.8e-02 |
| Mw144 |  | elt-1 | 36 | 1.70 | 4.0e-02 |
| SMAD3\_f1 |  | daf-8 | 47 | 1.53 | 4.2e-02 |
| Zfp161\_2858 |  | pzf-1 | 38 | 1.66 | 4.2e-02 |
| Mafk\_3106 |  | F45H11.6 | 48 | 1.52 | 4.3e-02 |
| MA0238.1 |  | ceh-53 lin-39 ceh-18 | 56 | 1.44 | 4.3e-02 |
| pTH10623 |  | scrt-1 | 43 | 1.58 | 4.3e-02 |
| Eip93F\_SANGER\_10\_FBgn0013948 |  | mbr-1 | 97 | 1.18 | 4.3e-02 |
| pTH5539 |  | unc-120 (0.66) | 44 | 1.57 | 4.3e-02 |
| pTH7875 |  | mel-28 | 67 | 1.35 | 4.5e-02 |
| MA0033.1 |  | lin-31 (0.6) | 60 | 1.40 | 4.5e-02 |
| pTH9222 |  | mel-28 | 80 | 1.27 | 4.7e-02 |
| pTH9164 |  | ceh-26 | 64 | 1.37 | 4.7e-02 |
| pTH10013 |  | nhr-168 | 58 | 1.41 | 4.8e-02 |
| pTH9026 |  | attf-1 | 28 | 1.85 | 4.9e-02 |
| pTH2280 |  | mnm-2 | 12 | 2.95 | 4.9e-02 |
| V$POU3F2\_01 |  | ceh-18 | 70 | 1.33 | 4.9e-02 |

### Correlated (and anti-correlated) transcription factors

|  |  |
| --- | --- |
| **Transcription factor** | **Correlation** |
| klf-2 | 0.94 |
| nhr-98 | 0.91 |
| nhr-97 | 0.86 |
| nhr-34 | 0.85 |
| odd-2 | 0.85 |
| ets-4 | 0.82 |
| nhr-243 | 0.81 |
| C34B4.2 | 0.81 |
| ref-1 | 0.80 |
| nhr-131 | 0.80 |
| sma-2 | 0.80 |
| peb-1 | 0.80 |
| nhr-203 | 0.80 |
| fkh-9 | 0.79 |
| nhr-44 | 0.79 |
| nhr-212 | 0.79 |
| sbp-1 | 0.78 |
| nhr-170 | 0.78 |
| nhr-123 | 0.78 |
| sma-3 | 0.76 |
| atf-5 | 0.76 |
| nhr-130 | 0.74 |
| nhr-103 | 0.73 |
| nhr-143 | 0.73 |
| nhr-28 | 0.73 |
| ceh-5 | -0.46 |
| F37B4.10 | -0.46 |
| mxl-2 | -0.47 |
| F10E7.11 | -0.48 |
| zip-8 | -0.48 |
| F52B5.7 | -0.49 |
| nfyc-1 | -0.49 |
| Y53H1A.2 | -0.50 |
| hlh-16 | -0.50 |
| pie-1 | -0.50 |
| vab-3 | -0.53 |
| spe-44 | -0.53 |
| T06G6.5 | -0.53 |
| D2030.7 | -0.55 |
| zip-4 | -0.55 |
| hmg-11 | -0.55 |
| F21D5.9 | -0.56 |
| C01F6.9 | -0.58 |
| him-8 | -0.59 |
| madf-10 | -0.60 |
| hmg-12 | -0.62 |
| ztf-4 | -0.62 |
| mxl-1 | -0.64 |
| repo-1 | -0.65 |
| ccch-3 | -0.65 |

### ChIP peaks enriched

|  |  |  |  |  |
| --- | --- | --- | --- | --- |
| **Gene** | **Experiment** | **Number of upstream peaks** | **Enrichment** | **FDR corrected p** |
| pha-4 | PHA-4\_Larvae-L2-stage | 57 | 1.94 | 2.0e-06 |
| pha-4 | PHA-4\_Larvae-L4-stage | 38 | 2.34 | 1.5e-05 |
| nhr-28 | NHR-28\_Larvae-L4-stage | 48 | 1.85 | 1.7e-04 |
| C01B12.2 | C01B12.2\_Larvae-L2-stage | 57 | 1.65 | 4.2e-04 |
| nhr-77 | NHR-77\_Larvae-L4-stage | 27 | 2.28 | 1.5e-03 |
| fos-1 | FOS-1\_Larvae-L2-stage | 53 | 1.62 | 2.0e-03 |
| dve-1 | DVE-1\_Larvae-L4-stage | 16 | 3.15 | 2.1e-03 |
| pha-4 | PHA-4\_Young-adult | 12 | 3.54 | 6.2e-03 |
| nhr-129 | NHR-129\_Larvae-L2-stage | 45 | 1.62 | 9.3e-03 |
| nhr-6 | NHR-6\_Larvae-L2-stage | 33 | 1.81 | 1.3e-02 |
| W03F9.2 | W03F9.2\_L4-Young-Adult-stage-larvae | 58 | 1.41 | 3.0e-02 |
