# Supplementary material for: Overlapping cell population expression profiling and regulatory inference in C. elegans
Source: BMC Genomics. 2016 Feb 29;17:159. doi: 10.1186/s12864-016-2482-z (PMC4772325; doi:10.1186/s12864-016-2482-z)
Supplement: Additional file 13: — Web supplement. (DOC 21 kb) [file 12864_2016_2482_MOESM13_ESM.zip › sortWeb/clusters/hier.300.clusters/258.html]

Cluster 258 

## Cluster 258

### Expression

| cnd-1 rep. 1 | cnd-1 rep. 2 | cnd-1 rep. 3 | pha-4 rep. 1 | pha-4 rep. 2 | pha-4 rep. 3 | ceh-27 | ceh-36 | ceh-6 | F21D5.9 | mir-57 | mls-2 | pal-1 | pros-1 | ttx-3 | unc-130 | hlh-16 | irx-1 | ceh-6 (+) hlh-16 (+) | ceh-6 (+) hlh-16 (-) | ceh-6 (-) hlh-16 (+) | cnd-1 singlets | pha-4 singlets | 0 | 60 | 120 | 150 | 180 | 240 | 330 | 390 | 420 | 480 | 540 | 570 | 600 | 630 | 660 | NAME | Functional description |
| --- | --- | --- | --- | --- | --- | --- | --- | --- | --- | --- | --- | --- | --- | --- | --- | --- | --- | --- | --- | --- | --- | --- | --- | --- | --- | --- | --- | --- | --- | --- | --- | --- | --- | --- | --- | --- | --- | --- | --- |
|  |  |  |  |  |  |  |  |  |  |  |  |  |  |  |  |  |  |  |  |  |  |  |  |  |  |  |  |  |  |  |  |  |  |  |  |  |  | C18H7.6 |  |
|  |  |  |  |  |  |  |  |  |  |  |  |  |  |  |  |  |  |  |  |  |  |  |  |  |  |  |  |  |  |  |  |  |  |  |  |  |  | F43C9.1 |  |
|  |  |  |  |  |  |  |  |  |  |  |  |  |  |  |  |  |  |  |  |  |  |  |  |  |  |  |  |  |  |  |  |  |  |  |  |  |  | *irld-40* | Insulin/EGF-Receptor L Domain protein |
|  |  |  |  |  |  |  |  |  |  |  |  |  |  |  |  |  |  |  |  |  |  |  |  |  |  |  |  |  |  |  |  |  |  |  |  |  |  | K04F1.9 |  |
|  |  |  |  |  |  |  |  |  |  |  |  |  |  |  |  |  |  |  |  |  |  |  |  |  |  |  |  |  |  |  |  |  |  |  |  |  |  | F23F12.13 |  |
|  |  |  |  |  |  |  |  |  |  |  |  |  |  |  |  |  |  |  |  |  |  |  |  |  |  |  |  |  |  |  |  |  |  |  |  |  |  | Y75B8A.47 |  |
|  |  |  |  |  |  |  |  |  |  |  |  |  |  |  |  |  |  |  |  |  |  |  |  |  |  |  |  |  |  |  |  |  |  |  |  |  |  | *asp-12* | ASpartyl Protease |
|  |  |  |  |  |  |  |  |  |  |  |  |  |  |  |  |  |  |  |  |  |  |  |  |  |  |  |  |  |  |  |  |  |  |  |  |  |  | R02D5.7 |  |
|  |  |  |  |  |  |  |  |  |  |  |  |  |  |  |  |  |  |  |  |  |  |  |  |  |  |  |  |  |  |  |  |  |  |  |  |  |  | H13N06.10 |  |
|  |  |  |  |  |  |  |  |  |  |  |  |  |  |  |  |  |  |  |  |  |  |  |  |  |  |  |  |  |  |  |  |  |  |  |  |  |  | *srab-11* | Serpentine Receptor, class AB (class A-like) |
|  |  |  |  |  |  |  |  |  |  |  |  |  |  |  |  |  |  |  |  |  |  |  |  |  |  |  |  |  |  |  |  |  |  |  |  |  |  | *grd-2* | GRounDhog (hedgehog-like family) |
|  |  |  |  |  |  |  |  |  |  |  |  |  |  |  |  |  |  |  |  |  |  |  |  |  |  |  |  |  |  |  |  |  |  |  |  |  |  | T01B6.8 |  |
|  |  |  |  |  |  |  |  |  |  |  |  |  |  |  |  |  |  |  |  |  |  |  |  |  |  |  |  |  |  |  |  |  |  |  |  |  |  | T04F3.3 |  |
|  |  |  |  |  |  |  |  |  |  |  |  |  |  |  |  |  |  |  |  |  |  |  |  |  |  |  |  |  |  |  |  |  |  |  |  |  |  | *smp-2* | SeMaPhorin related |
|  |  |  |  |  |  |  |  |  |  |  |  |  |  |  |  |  |  |  |  |  |  |  |  |  |  |  |  |  |  |  |  |  |  |  |  |  |  | R17.3 |  |
|  |  |  |  |  |  |  |  |  |  |  |  |  |  |  |  |  |  |  |  |  |  |  |  |  |  |  |  |  |  |  |  |  |  |  |  |  |  | T03F7.14 |  |
|  |  |  |  |  |  |  |  |  |  |  |  |  |  |  |  |  |  |  |  |  |  |  |  |  |  |  |  |  |  |  |  |  |  |  |  |  |  | *aakg-5* | AMP-Activated protein Kinase Gamma subunit |
|  |  |  |  |  |  |  |  |  |  |  |  |  |  |  |  |  |  |  |  |  |  |  |  |  |  |  |  |  |  |  |  |  |  |  |  |  |  | *emb-8* | abnormal EMBroygenesis |
|  |  |  |  |  |  |  |  |  |  |  |  |  |  |  |  |  |  |  |  |  |  |  |  |  |  |  |  |  |  |  |  |  |  |  |  |  |  | *asd-2* | Alternative Splicing Defective |
|  |  |  |  |  |  |  |  |  |  |  |  |  |  |  |  |  |  |  |  |  |  |  |  |  |  |  |  |  |  |  |  |  |  |  |  |  |  | *nipi-4* | No Induction of Peptide after Drechmeria Infection |
|  |  |  |  |  |  |  |  |  |  |  |  |  |  |  |  |  |  |  |  |  |  |  |  |  |  |  |  |  |  |  |  |  |  |  |  |  |  | *npr-24* | NeuroPeptide Receptor family |
|  |  |  |  |  |  |  |  |  |  |  |  |  |  |  |  |  |  |  |  |  |  |  |  |  |  |  |  |  |  |  |  |  |  |  |  |  |  | B0391.8 |  |
|  |  |  |  |  |  |  |  |  |  |  |  |  |  |  |  |  |  |  |  |  |  |  |  |  |  |  |  |  |  |  |  |  |  |  |  |  |  | *srg-55* | Serpentine Receptor, class G (gamma) |
|  |  |  |  |  |  |  |  |  |  |  |  |  |  |  |  |  |  |  |  |  |  |  |  |  |  |  |  |  |  |  |  |  |  |  |  |  |  | *fbxa-16* | F-box A protein |
|  |  |  |  |  |  |  |  |  |  |  |  |  |  |  |  |  |  |  |  |  |  |  |  |  |  |  |  |  |  |  |  |  |  |  |  |  |  | *tpa-1* | TPA (tetradecanoyl phorbol acetate) resistant |
|  |  |  |  |  |  |  |  |  |  |  |  |  |  |  |  |  |  |  |  |  |  |  |  |  |  |  |  |  |  |  |  |  |  |  |  |  |  | *sms-3* | SphingoMyelin Synthase |
|  |  |  |  |  |  |  |  |  |  |  |  |  |  |  |  |  |  |  |  |  |  |  |  |  |  |  |  |  |  |  |  |  |  |  |  |  |  | *srd-33* | Serpentine Receptor, class D (delta) |
|  |  |  |  |  |  |  |  |  |  |  |  |  |  |  |  |  |  |  |  |  |  |  |  |  |  |  |  |  |  |  |  |  |  |  |  |  |  | *mec-8* | MEChanosensory abnormality |
|  |  |  |  |  |  |  |  |  |  |  |  |  |  |  |  |  |  |  |  |  |  |  |  |  |  |  |  |  |  |  |  |  |  |  |  |  |  | F53A10.2 |  |
|  |  |  |  |  |  |  |  |  |  |  |  |  |  |  |  |  |  |  |  |  |  |  |  |  |  |  |  |  |  |  |  |  |  |  |  |  |  | *dre-1* | Daf-12, REdundant with |
|  |  |  |  |  |  |  |  |  |  |  |  |  |  |  |  |  |  |  |  |  |  |  |  |  |  |  |  |  |  |  |  |  |  |  |  |  |  | *faah-1* | Fatty Acid Amide Hydrolase homolog |
|  |  |  |  |  |  |  |  |  |  |  |  |  |  |  |  |  |  |  |  |  |  |  |  |  |  |  |  |  |  |  |  |  |  |  |  |  |  | W09D6.5 |  |
|  |  |  |  |  |  |  |  |  |  |  |  |  |  |  |  |  |  |  |  |  |  |  |  |  |  |  |  |  |  |  |  |  |  |  |  |  |  | Y45F10A.7 |  |
|  |  |  |  |  |  |  |  |  |  |  |  |  |  |  |  |  |  |  |  |  |  |  |  |  |  |  |  |  |  |  |  |  |  |  |  |  |  | Y45F10A.3 |  |
|  |  |  |  |  |  |  |  |  |  |  |  |  |  |  |  |  |  |  |  |  |  |  |  |  |  |  |  |  |  |  |  |  |  |  |  |  |  | F36G3.1 |  |
|  |  |  |  |  |  |  |  |  |  |  |  |  |  |  |  |  |  |  |  |  |  |  |  |  |  |  |  |  |  |  |  |  |  |  |  |  |  | W06A11.1 |  |
|  |  |  |  |  |  |  |  |  |  |  |  |  |  |  |  |  |  |  |  |  |  |  |  |  |  |  |  |  |  |  |  |  |  |  |  |  |  | F22E12.1 |  |
|  |  |  |  |  |  |  |  |  |  |  |  |  |  |  |  |  |  |  |  |  |  |  |  |  |  |  |  |  |  |  |  |  |  |  |  |  |  | F41C3.11 |  |
|  |  |  |  |  |  |  |  |  |  |  |  |  |  |  |  |  |  |  |  |  |  |  |  |  |  |  |  |  |  |  |  |  |  |  |  |  |  | F49H12.5 |  |
|  |  |  |  |  |  |  |  |  |  |  |  |  |  |  |  |  |  |  |  |  |  |  |  |  |  |  |  |  |  |  |  |  |  |  |  |  |  | T19B10.5 |  |
|  |  |  |  |  |  |  |  |  |  |  |  |  |  |  |  |  |  |  |  |  |  |  |  |  |  |  |  |  |  |  |  |  |  |  |  |  |  | C05C8.7 |  |
|  |  |  |  |  |  |  |  |  |  |  |  |  |  |  |  |  |  |  |  |  |  |  |  |  |  |  |  |  |  |  |  |  |  |  |  |  |  | *cyp-42A1* | CYtochrome P450 family |
|  |  |  |  |  |  |  |  |  |  |  |  |  |  |  |  |  |  |  |  |  |  |  |  |  |  |  |  |  |  |  |  |  |  |  |  |  |  | *scav-2* | SCAVenger receptor (CD36 family) related |
|  |  |  |  |  |  |  |  |  |  |  |  |  |  |  |  |  |  |  |  |  |  |  |  |  |  |  |  |  |  |  |  |  |  |  |  |  |  | *col-118* | COLlagen |
|  |  |  |  |  |  |  |  |  |  |  |  |  |  |  |  |  |  |  |  |  |  |  |  |  |  |  |  |  |  |  |  |  |  |  |  |  |  | *gfi-1* | GEI-4 (Four) Interacting protein |
|  |  |  |  |  |  |  |  |  |  |  |  |  |  |  |  |  |  |  |  |  |  |  |  |  |  |  |  |  |  |  |  |  |  |  |  |  |  | F10C2.3 |  |
|  |  |  |  |  |  |  |  |  |  |  |  |  |  |  |  |  |  |  |  |  |  |  |  |  |  |  |  |  |  |  |  |  |  |  |  |  |  | T25B9.9 |  |
|  |  |  |  |  |  |  |  |  |  |  |  |  |  |  |  |  |  |  |  |  |  |  |  |  |  |  |  |  |  |  |  |  |  |  |  |  |  | Y37A1B.5 |  |
|  |  |  |  |  |  |  |  |  |  |  |  |  |  |  |  |  |  |  |  |  |  |  |  |  |  |  |  |  |  |  |  |  |  |  |  |  |  | *fbxa-169* | F-box A protein |
|  |  |  |  |  |  |  |  |  |  |  |  |  |  |  |  |  |  |  |  |  |  |  |  |  |  |  |  |  |  |  |  |  |  |  |  |  |  | F35F10.4 |  |
|  |  |  |  |  |  |  |  |  |  |  |  |  |  |  |  |  |  |  |  |  |  |  |  |  |  |  |  |  |  |  |  |  |  |  |  |  |  | *nhr-110* | Nuclear Hormone Receptor family |
|  |  |  |  |  |  |  |  |  |  |  |  |  |  |  |  |  |  |  |  |  |  |  |  |  |  |  |  |  |  |  |  |  |  |  |  |  |  | Y82E9BL.9 |  |
|  |  |  |  |  |  |  |  |  |  |  |  |  |  |  |  |  |  |  |  |  |  |  |  |  |  |  |  |  |  |  |  |  |  |  |  |  |  | *sma-6* | SMAll |
|  |  |  |  |  |  |  |  |  |  |  |  |  |  |  |  |  |  |  |  |  |  |  |  |  |  |  |  |  |  |  |  |  |  |  |  |  |  | *cutl-19* | CUTiclin-Like |
|  |  |  |  |  |  |  |  |  |  |  |  |  |  |  |  |  |  |  |  |  |  |  |  |  |  |  |  |  |  |  |  |  |  |  |  |  |  | *cog-1* | Connection Of Gonad defective |
|  |  |  |  |  |  |  |  |  |  |  |  |  |  |  |  |  |  |  |  |  |  |  |  |  |  |  |  |  |  |  |  |  |  |  |  |  |  | *egl-5* | EGg Laying defective |
|  |  |  |  |  |  |  |  |  |  |  |  |  |  |  |  |  |  |  |  |  |  |  |  |  |  |  |  |  |  |  |  |  |  |  |  |  |  | *egl-20* | EGg Laying defective |
|  |  |  |  |  |  |  |  |  |  |  |  |  |  |  |  |  |  |  |  |  |  |  |  |  |  |  |  |  |  |  |  |  |  |  |  |  |  | F21A3.3 |  |
|  |  |  |  |  |  |  |  |  |  |  |  |  |  |  |  |  |  |  |  |  |  |  |  |  |  |  |  |  |  |  |  |  |  |  |  |  |  | *col-34* | COLlagen |
|  |  |  |  |  |  |  |  |  |  |  |  |  |  |  |  |  |  |  |  |  |  |  |  |  |  |  |  |  |  |  |  |  |  |  |  |  |  | F11E6.3 |  |
|  |  |  |  |  |  |  |  |  |  |  |  |  |  |  |  |  |  |  |  |  |  |  |  |  |  |  |  |  |  |  |  |  |  |  |  |  |  | Y75B12B.3 |  |
|  |  |  |  |  |  |  |  |  |  |  |  |  |  |  |  |  |  |  |  |  |  |  |  |  |  |  |  |  |  |  |  |  |  |  |  |  |  | *col-146* | COLlagen |
|  |  |  |  |  |  |  |  |  |  |  |  |  |  |  |  |  |  |  |  |  |  |  |  |  |  |  |  |  |  |  |  |  |  |  |  |  |  | F22F4.4 |  |
|  |  |  |  |  |  |  |  |  |  |  |  |  |  |  |  |  |  |  |  |  |  |  |  |  |  |  |  |  |  |  |  |  |  |  |  |  |  | *cutl-18* | CUTiclin-Like |
|  |  |  |  |  |  |  |  |  |  |  |  |  |  |  |  |  |  |  |  |  |  |  |  |  |  |  |  |  |  |  |  |  |  |  |  |  |  | C30F8.3 |  |
|  |  |  |  |  |  |  |  |  |  |  |  |  |  |  |  |  |  |  |  |  |  |  |  |  |  |  |  |  |  |  |  |  |  |  |  |  |  | C45G9.6 |  |
|  |  |  |  |  |  |  |  |  |  |  |  |  |  |  |  |  |  |  |  |  |  |  |  |  |  |  |  |  |  |  |  |  |  |  |  |  |  | *sma-3* | SMAll |
|  |  |  |  |  |  |  |  |  |  |  |  |  |  |  |  |  |  |  |  |  |  |  |  |  |  |  |  |  |  |  |  |  |  |  |  |  |  | ZC513.2 |  |
|  |  |  |  |  |  |  |  |  |  |  |  |  |  |  |  |  |  |  |  |  |  |  |  |  |  |  |  |  |  |  |  |  |  |  |  |  |  | ZC513.1 |  |
|  |  |  |  |  |  |  |  |  |  |  |  |  |  |  |  |  |  |  |  |  |  |  |  |  |  |  |  |  |  |  |  |  |  |  |  |  |  | W09C2.8 |  |
|  |  |  |  |  |  |  |  |  |  |  |  |  |  |  |  |  |  |  |  |  |  |  |  |  |  |  |  |  |  |  |  |  |  |  |  |  |  | F07G6.8 |  |
|  |  |  |  |  |  |  |  |  |  |  |  |  |  |  |  |  |  |  |  |  |  |  |  |  |  |  |  |  |  |  |  |  |  |  |  |  |  | T12A2.1 |  |
|  |  |  |  |  |  |  |  |  |  |  |  |  |  |  |  |  |  |  |  |  |  |  |  |  |  |  |  |  |  |  |  |  |  |  |  |  |  | Y51H4A.7 |  |

### Phenotypes enriched

none found

### Anatomy terms enriched

none found

### GO terms enriched

none found

### Expression clusters enriched

|  |  |  |  |
| --- | --- | --- | --- |
| **Group name** | **Number in cluster** | **Enrichment** | **FDR corrected p** |
| Expression Pattern Group C, enriched for genes involved in metabolic processes. | 17 | 3.68 | 0.000796 |
| Germline-enriched and sex-biased expression profile cluster B. | 14 | 4.00 | 0.002510 |
| Genes with increased expression after 24 hours of infection by S.marcescens Fold changes shown are pathogen vs OP50. WBPaper00038438:S.marcescens\_24hr\_upregulated\_RNAseq | 25 | 2.36 | 0.005350 |
| Genes upregulated in sma-2 L4 (3 arrays) or sma-4 L4 (1 array) vs. N2 L4. | 10 | 4.15 | 0.031500 |

### Motifs enriched

|  |  |  |  |  |  |
| --- | --- | --- | --- | --- | --- |
| **Motif** | **Logo** | **Possible orthologs** | **Number of motifs in cluster** | **Enrichment** | **FDR corrected p** |
| MA0481.1 |  | lin-31 (0.51) fkh-7 fkh-8 fkh-10 daf-16 let-381 | 33 | 2.26 | 0.00026 |
| Hoxa13\_3126 |  | pal-1 (0.6) ceh-24 | 24 | 2.80 | 0.00038 |
| pTH6549 |  | lin-31 (0.51) pha-4 fkh-7 fkh-8 daf-16 let-381 | 19 | 3.11 | 0.00120 |
| Hoxd10\_2368 |  | php-3 (0.61) | 58 | 1.46 | 0.00140 |
| HOXC10\_2 |  | php-3 (0.61) pal-1 (0.6) lin-39 ceh-24 | 29 | 2.22 | 0.00170 |
| HOXA10\_1 |  | php-3 (0.61) lin-39 ceh-24 | 31 | 2.11 | 0.00200 |
| V$HFH3\_01 |  | lin-31 (0.51) let-381 | 36 | 1.89 | 0.00260 |
| pTH3043 |  | lin-31 (0.51) fkh-10 let-381 | 19 | 2.89 | 0.00270 |
| MA0600.1 |  | daf-19 | 64 | 1.33 | 0.00280 |
| Hoxd13\_2356 |  | pal-1 (0.6) | 22 | 2.56 | 0.00310 |
| Optix\_Cell\_FBgn0025360 |  | elt-6 elt-1 ceh-32 ceh-34 | 54 | 1.48 | 0.00360 |
| pTH5561 |  | nhr-239 | 47 | 1.60 | 0.00360 |
| MA0173.1 |  | irx-1 | 14 | 3.53 | 0.00440 |
| SP4\_1 |  | klf-2 (0.71) klf-1 | 46 | 1.60 | 0.00510 |
| Hoxa11\_2218 |  | php-3 (0.61) | 31 | 1.97 | 0.00580 |
| pTH3467 |  | nhr-68 nhr-6 nhr-71 Y67D8A.3 | 15 | 3.13 | 0.00770 |
| Hoxd9\_1 |  | php-3 (0.61) lin-39 | 40 | 1.69 | 0.00780 |
| Nsy-7 |  | nsy-7 | 9 | 5.03 | 0.00820 |
| pTH9880 |  | end-1 | 63 | 1.31 | 0.00860 |
| pTH3477 |  | daf-16 | 14 | 3.22 | 0.00970 |
| V$POU3F2\_02 |  | ceh-18 tbp-1 | 39 | 1.69 | 0.01000 |
| Irx2\_0900 |  | irx-1 | 63 | 1.30 | 0.01000 |
| pTH2846 |  | lin-31 (0.51) | 13 | 3.38 | 0.01100 |
| SOX2\_4 |  | sox-4 | 63 | 1.30 | 0.01100 |
| Hoxa10\_2318 |  | ceh-24 | 32 | 1.86 | 0.01100 |
| Oc\_Cell\_FBgn0004102 |  | ceh-53 ceh-45 alr-1 | 41 | 1.63 | 0.01200 |
| Msx3\_1 |  | ceh-1 (0.57) | 43 | 1.59 | 0.01200 |
| pTH9353 |  | ceh-51 | 33 | 1.79 | 0.01600 |
| Hoxd11\_3873 |  | php-3 (0.61) | 25 | 2.06 | 0.01700 |
| Gata6\_3769 |  | elt-1 | 46 | 1.51 | 0.01700 |
| pTH6423 |  | pha-2 | 8 | 4.95 | 0.01800 |
| V$TATA\_01 |  | tbp-1 | 37 | 1.67 | 0.01900 |
| EGR2\_2 |  | ZC328.2 | 60 | 1.31 | 0.02100 |
| Cdx1\_2245 |  | ceh-13 | 31 | 1.81 | 0.02100 |
| V$TST1\_01 |  | pal-1 (0.6) ceh-18 | 55 | 1.37 | 0.02100 |
| V$FREAC7\_01 |  | lin-31 (0.51) | 32 | 1.78 | 0.02100 |
| POU4F2\_2 |  | unc-86 | 31 | 1.81 | 0.02200 |
| pTH8216 |  | Y116A8C.22 | 24 | 2.05 | 0.02400 |
| HepG2\_SRF\_HudsonAlpha |  | unc-120 (0.63) | 24 | 2.04 | 0.02500 |
| Cdx2\_4272 |  | ceh-13 | 34 | 1.71 | 0.02600 |
| Zbtb12\_2932 |  | lsy-27 | 58 | 1.32 | 0.02700 |
| GRHL1\_2 |  | grh-1 | 29 | 1.83 | 0.02800 |
| V$SP1\_Q6 |  | klf-2 (0.71) | 22 | 2.10 | 0.03100 |
| pTH10797 |  | K11D2.4 | 31 | 1.75 | 0.03300 |
| YMR043W\_831 |  | unc-120 (0.63) | 68 | 1.19 | 0.03400 |
| F$MCM1\_01 |  | unc-120 (0.63) | 21 | 2.13 | 0.03400 |
| So\_Cell\_FBgn0003460 |  | ceh-32 | 40 | 1.55 | 0.03500 |
| HXC6\_f1 |  | lin-39 | 24 | 1.98 | 0.03500 |
| pTH9387 |  | C34D1.1 | 58 | 1.31 | 0.03600 |
| pTH3464 |  | nhr-213 | 55 | 1.34 | 0.03700 |
| pTH2340 |  | ceh-32 ces-1 | 32 | 1.71 | 0.03800 |
| pTH6641 |  | lin-31 (0.51) | 12 | 3.01 | 0.03800 |
| EN1\_2 |  | ceh-16 | 25 | 1.91 | 0.04100 |
| pTH9189 |  | dmd-3 (0.55) | 26 | 1.88 | 0.04100 |
| pTH9080 |  | mnm-2 | 22 | 2.03 | 0.04200 |
| TBX20\_5 |  | mab-9 | 67 | 1.20 | 0.04300 |
| MA0261.1 |  | lin-14 | 21 | 2.06 | 0.04700 |
| Mv108 |  | pax-2 | 18 | 2.25 | 0.04700 |
| inv\_SOLEXA\_5\_FBgn0001269 |  | ceh-16 | 52 | 1.36 | 0.04900 |

### Correlated (and anti-correlated) transcription factors

|  |  |
| --- | --- |
| **Transcription factor** | **Correlation** |
| nhr-97 | 0.83 |
| sma-3 | 0.82 |
| elt-3 | 0.82 |
| cog-1 | 0.79 |
| egl-5 | 0.78 |
| lin-48 | 0.78 |
| ztf-30 | 0.77 |
| nhr-120 | 0.73 |
| B0310.2 | 0.72 |
| klf-2 | 0.71 |
| dsc-1 | 0.70 |
| klf-3 | 0.70 |
| nhr-147 | 0.69 |
| ceh-99 | 0.69 |
| mls-1 | 0.69 |
| blmp-1 | 0.69 |
| nhr-34 | 0.68 |
| nhr-109 | 0.67 |
| nhr-69 | 0.67 |
| sbp-1 | 0.67 |
| nhr-260 | 0.67 |
| nhr-148 | 0.66 |
| nhr-43 | 0.65 |
| ets-8 | 0.65 |
| ref-1 | 0.65 |
| C01F6.9 | -0.44 |
| R05D3.3 | -0.44 |
| mxl-1 | -0.45 |
| tbx-36 | -0.45 |
| Y54G2A.20 | -0.46 |
| dhhc-13 | -0.46 |
| zip-4 | -0.46 |
| nfyc-1 | -0.47 |
| sptf-1 | -0.48 |
| nhr-234 | -0.49 |
| tbx-34 | -0.49 |
| pzf-1 | -0.49 |
| lir-3 | -0.50 |
| vab-3 | -0.50 |
| madf-10 | -0.52 |
| K11D12.12 | -0.52 |
| T06G6.5 | -0.54 |
| nhr-276 | -0.55 |
| fkh-2 | -0.56 |
| repo-1 | -0.57 |
| nhr-215 | -0.57 |
| D2030.7 | -0.57 |
| zip-8 | -0.57 |
| snu-23 | -0.59 |
| ztf-4 | -0.63 |

### ChIP peaks enriched

|  |  |  |  |  |
| --- | --- | --- | --- | --- |
| **Gene** | **Experiment** | **Number of upstream peaks** | **Enrichment** | **FDR corrected p** |
| nhr-28 | NHR-28\_Larvae-L4-stage | 17 | 2.66 | 0.006 |
