# Supplementary material for: Overlapping cell population expression profiling and regulatory inference in C. elegans
Source: BMC Genomics. 2016 Feb 29;17:159. doi: 10.1186/s12864-016-2482-z (PMC4772325; doi:10.1186/s12864-016-2482-z)
Supplement: Additional file 13: — Web supplement. (DOC 21 kb) [file 12864_2016_2482_MOESM13_ESM.zip › sortWeb/clusters/hier.300.clusters/259.html]

Cluster 259 

## Cluster 259

### Expression

| cnd-1 rep. 1 | cnd-1 rep. 2 | cnd-1 rep. 3 | pha-4 rep. 1 | pha-4 rep. 2 | pha-4 rep. 3 | ceh-27 | ceh-36 | ceh-6 | F21D5.9 | mir-57 | mls-2 | pal-1 | pros-1 | ttx-3 | unc-130 | hlh-16 | irx-1 | ceh-6 (+) hlh-16 (+) | ceh-6 (+) hlh-16 (-) | ceh-6 (-) hlh-16 (+) | cnd-1 singlets | pha-4 singlets | 0 | 60 | 120 | 150 | 180 | 240 | 330 | 390 | 420 | 480 | 540 | 570 | 600 | 630 | 660 | NAME | Functional description |
| --- | --- | --- | --- | --- | --- | --- | --- | --- | --- | --- | --- | --- | --- | --- | --- | --- | --- | --- | --- | --- | --- | --- | --- | --- | --- | --- | --- | --- | --- | --- | --- | --- | --- | --- | --- | --- | --- | --- | --- |
|  |  |  |  |  |  |  |  |  |  |  |  |  |  |  |  |  |  |  |  |  |  |  |  |  |  |  |  |  |  |  |  |  |  |  |  |  |  | *gly-1* | GLYcosylation related |
|  |  |  |  |  |  |  |  |  |  |  |  |  |  |  |  |  |  |  |  |  |  |  |  |  |  |  |  |  |  |  |  |  |  |  |  |  |  | Y46G5A.41 |  |
|  |  |  |  |  |  |  |  |  |  |  |  |  |  |  |  |  |  |  |  |  |  |  |  |  |  |  |  |  |  |  |  |  |  |  |  |  |  | C07H4.4 |  |
|  |  |  |  |  |  |  |  |  |  |  |  |  |  |  |  |  |  |  |  |  |  |  |  |  |  |  |  |  |  |  |  |  |  |  |  |  |  | W06B11.10 |  |
|  |  |  |  |  |  |  |  |  |  |  |  |  |  |  |  |  |  |  |  |  |  |  |  |  |  |  |  |  |  |  |  |  |  |  |  |  |  | *fbxb-93* | F-box B protein |
|  |  |  |  |  |  |  |  |  |  |  |  |  |  |  |  |  |  |  |  |  |  |  |  |  |  |  |  |  |  |  |  |  |  |  |  |  |  | F13C5.8 |  |
|  |  |  |  |  |  |  |  |  |  |  |  |  |  |  |  |  |  |  |  |  |  |  |  |  |  |  |  |  |  |  |  |  |  |  |  |  |  | *unc-25* | UNCoordinated |
|  |  |  |  |  |  |  |  |  |  |  |  |  |  |  |  |  |  |  |  |  |  |  |  |  |  |  |  |  |  |  |  |  |  |  |  |  |  | *tba-9* | TuBulin, Alpha |
|  |  |  |  |  |  |  |  |  |  |  |  |  |  |  |  |  |  |  |  |  |  |  |  |  |  |  |  |  |  |  |  |  |  |  |  |  |  | *unc-46* | UNCoordinated |
|  |  |  |  |  |  |  |  |  |  |  |  |  |  |  |  |  |  |  |  |  |  |  |  |  |  |  |  |  |  |  |  |  |  |  |  |  |  | F35B3.4 |  |
|  |  |  |  |  |  |  |  |  |  |  |  |  |  |  |  |  |  |  |  |  |  |  |  |  |  |  |  |  |  |  |  |  |  |  |  |  |  | C15C7.7 |  |
|  |  |  |  |  |  |  |  |  |  |  |  |  |  |  |  |  |  |  |  |  |  |  |  |  |  |  |  |  |  |  |  |  |  |  |  |  |  | *unc-86* | UNCoordinated |
|  |  |  |  |  |  |  |  |  |  |  |  |  |  |  |  |  |  |  |  |  |  |  |  |  |  |  |  |  |  |  |  |  |  |  |  |  |  | C30A5.4 |  |
|  |  |  |  |  |  |  |  |  |  |  |  |  |  |  |  |  |  |  |  |  |  |  |  |  |  |  |  |  |  |  |  |  |  |  |  |  |  | *tol-1* | TOLl (Drosophila) family |
|  |  |  |  |  |  |  |  |  |  |  |  |  |  |  |  |  |  |  |  |  |  |  |  |  |  |  |  |  |  |  |  |  |  |  |  |  |  | *lron-15* | eLRR (extracellular Leucine-Rich Repeat) ONly |
|  |  |  |  |  |  |  |  |  |  |  |  |  |  |  |  |  |  |  |  |  |  |  |  |  |  |  |  |  |  |  |  |  |  |  |  |  |  | *rin-1* | RIN (Ras/Rab INteractor) homolog |
|  |  |  |  |  |  |  |  |  |  |  |  |  |  |  |  |  |  |  |  |  |  |  |  |  |  |  |  |  |  |  |  |  |  |  |  |  |  | *unc-83* | UNCoordinated |
|  |  |  |  |  |  |  |  |  |  |  |  |  |  |  |  |  |  |  |  |  |  |  |  |  |  |  |  |  |  |  |  |  |  |  |  |  |  | *mnr-1* | MeNoRin (dendritic branching protein) |
|  |  |  |  |  |  |  |  |  |  |  |  |  |  |  |  |  |  |  |  |  |  |  |  |  |  |  |  |  |  |  |  |  |  |  |  |  |  | W01F3.2 |  |
|  |  |  |  |  |  |  |  |  |  |  |  |  |  |  |  |  |  |  |  |  |  |  |  |  |  |  |  |  |  |  |  |  |  |  |  |  |  | *srap-1* | Serine Rich Adhesion Protein-like |
|  |  |  |  |  |  |  |  |  |  |  |  |  |  |  |  |  |  |  |  |  |  |  |  |  |  |  |  |  |  |  |  |  |  |  |  |  |  | *nas-38* | Nematode AStacin protease |
|  |  |  |  |  |  |  |  |  |  |  |  |  |  |  |  |  |  |  |  |  |  |  |  |  |  |  |  |  |  |  |  |  |  |  |  |  |  | *grd-16* | GRounDhog (hedgehog-like family) |
|  |  |  |  |  |  |  |  |  |  |  |  |  |  |  |  |  |  |  |  |  |  |  |  |  |  |  |  |  |  |  |  |  |  |  |  |  |  | *clec-229* | C-type LECtin |
|  |  |  |  |  |  |  |  |  |  |  |  |  |  |  |  |  |  |  |  |  |  |  |  |  |  |  |  |  |  |  |  |  |  |  |  |  |  | *oac-1* | O-ACyltransferase homolog |
|  |  |  |  |  |  |  |  |  |  |  |  |  |  |  |  |  |  |  |  |  |  |  |  |  |  |  |  |  |  |  |  |  |  |  |  |  |  | *aex-2* | ABoc, EXpulsion (defecation) defective |
|  |  |  |  |  |  |  |  |  |  |  |  |  |  |  |  |  |  |  |  |  |  |  |  |  |  |  |  |  |  |  |  |  |  |  |  |  |  | CD4.11 |  |
|  |  |  |  |  |  |  |  |  |  |  |  |  |  |  |  |  |  |  |  |  |  |  |  |  |  |  |  |  |  |  |  |  |  |  |  |  |  | CD4.5 |  |
|  |  |  |  |  |  |  |  |  |  |  |  |  |  |  |  |  |  |  |  |  |  |  |  |  |  |  |  |  |  |  |  |  |  |  |  |  |  | *wrt-8* | WaRThog (hedgehog-like family) |
|  |  |  |  |  |  |  |  |  |  |  |  |  |  |  |  |  |  |  |  |  |  |  |  |  |  |  |  |  |  |  |  |  |  |  |  |  |  | F29G6.1 |  |
|  |  |  |  |  |  |  |  |  |  |  |  |  |  |  |  |  |  |  |  |  |  |  |  |  |  |  |  |  |  |  |  |  |  |  |  |  |  | *oac-30* | O-ACyltransferase homolog |
|  |  |  |  |  |  |  |  |  |  |  |  |  |  |  |  |  |  |  |  |  |  |  |  |  |  |  |  |  |  |  |  |  |  |  |  |  |  | *clec-231* | C-type LECtin |
|  |  |  |  |  |  |  |  |  |  |  |  |  |  |  |  |  |  |  |  |  |  |  |  |  |  |  |  |  |  |  |  |  |  |  |  |  |  | C04F6.2 |  |
|  |  |  |  |  |  |  |  |  |  |  |  |  |  |  |  |  |  |  |  |  |  |  |  |  |  |  |  |  |  |  |  |  |  |  |  |  |  | *nmur-3* | NMUR (NeuroMedin U Receptor) homolog |
|  |  |  |  |  |  |  |  |  |  |  |  |  |  |  |  |  |  |  |  |  |  |  |  |  |  |  |  |  |  |  |  |  |  |  |  |  |  | *slcf-2* | SoLute Carrier Family |
|  |  |  |  |  |  |  |  |  |  |  |  |  |  |  |  |  |  |  |  |  |  |  |  |  |  |  |  |  |  |  |  |  |  |  |  |  |  | *wrt-6* | WaRThog (hedgehog-like family) |
|  |  |  |  |  |  |  |  |  |  |  |  |  |  |  |  |  |  |  |  |  |  |  |  |  |  |  |  |  |  |  |  |  |  |  |  |  |  | *clh-2* | CLC-type chloride cHannel |
|  |  |  |  |  |  |  |  |  |  |  |  |  |  |  |  |  |  |  |  |  |  |  |  |  |  |  |  |  |  |  |  |  |  |  |  |  |  | *pqn-22* | Prion-like-(Q/N-rich)-domain-bearing protein |
|  |  |  |  |  |  |  |  |  |  |  |  |  |  |  |  |  |  |  |  |  |  |  |  |  |  |  |  |  |  |  |  |  |  |  |  |  |  | *ttm-2* | Toxin-regulated Targets of MAPK |
|  |  |  |  |  |  |  |  |  |  |  |  |  |  |  |  |  |  |  |  |  |  |  |  |  |  |  |  |  |  |  |  |  |  |  |  |  |  | *unc-47* | UNCoordinated |
|  |  |  |  |  |  |  |  |  |  |  |  |  |  |  |  |  |  |  |  |  |  |  |  |  |  |  |  |  |  |  |  |  |  |  |  |  |  | *grl-13* | GRound-Like (grd related) |
|  |  |  |  |  |  |  |  |  |  |  |  |  |  |  |  |  |  |  |  |  |  |  |  |  |  |  |  |  |  |  |  |  |  |  |  |  |  | Y39B6A.8 |  |
|  |  |  |  |  |  |  |  |  |  |  |  |  |  |  |  |  |  |  |  |  |  |  |  |  |  |  |  |  |  |  |  |  |  |  |  |  |  | F27D9.7 |  |
|  |  |  |  |  |  |  |  |  |  |  |  |  |  |  |  |  |  |  |  |  |  |  |  |  |  |  |  |  |  |  |  |  |  |  |  |  |  | F16C3.2 |  |
|  |  |  |  |  |  |  |  |  |  |  |  |  |  |  |  |  |  |  |  |  |  |  |  |  |  |  |  |  |  |  |  |  |  |  |  |  |  | F57H12.5 |  |
|  |  |  |  |  |  |  |  |  |  |  |  |  |  |  |  |  |  |  |  |  |  |  |  |  |  |  |  |  |  |  |  |  |  |  |  |  |  | C50D2.6 |  |
|  |  |  |  |  |  |  |  |  |  |  |  |  |  |  |  |  |  |  |  |  |  |  |  |  |  |  |  |  |  |  |  |  |  |  |  |  |  | F41G3.1 |  |
|  |  |  |  |  |  |  |  |  |  |  |  |  |  |  |  |  |  |  |  |  |  |  |  |  |  |  |  |  |  |  |  |  |  |  |  |  |  | R07B1.5 |  |
|  |  |  |  |  |  |  |  |  |  |  |  |  |  |  |  |  |  |  |  |  |  |  |  |  |  |  |  |  |  |  |  |  |  |  |  |  |  | T21B4.3 |  |
|  |  |  |  |  |  |  |  |  |  |  |  |  |  |  |  |  |  |  |  |  |  |  |  |  |  |  |  |  |  |  |  |  |  |  |  |  |  | F19H8.2 |  |
|  |  |  |  |  |  |  |  |  |  |  |  |  |  |  |  |  |  |  |  |  |  |  |  |  |  |  |  |  |  |  |  |  |  |  |  |  |  | K01A6.4 |  |
|  |  |  |  |  |  |  |  |  |  |  |  |  |  |  |  |  |  |  |  |  |  |  |  |  |  |  |  |  |  |  |  |  |  |  |  |  |  | *flp-28* | FMRF-Like Peptide |
|  |  |  |  |  |  |  |  |  |  |  |  |  |  |  |  |  |  |  |  |  |  |  |  |  |  |  |  |  |  |  |  |  |  |  |  |  |  | *clec-223* | C-type LECtin |
|  |  |  |  |  |  |  |  |  |  |  |  |  |  |  |  |  |  |  |  |  |  |  |  |  |  |  |  |  |  |  |  |  |  |  |  |  |  | *fbxa-196* | F-box A protein |
|  |  |  |  |  |  |  |  |  |  |  |  |  |  |  |  |  |  |  |  |  |  |  |  |  |  |  |  |  |  |  |  |  |  |  |  |  |  | K04A8.3 |  |
|  |  |  |  |  |  |  |  |  |  |  |  |  |  |  |  |  |  |  |  |  |  |  |  |  |  |  |  |  |  |  |  |  |  |  |  |  |  | F52C9.5 |  |
|  |  |  |  |  |  |  |  |  |  |  |  |  |  |  |  |  |  |  |  |  |  |  |  |  |  |  |  |  |  |  |  |  |  |  |  |  |  | *slt-1* | SLiT (Drosophila) homolog |
|  |  |  |  |  |  |  |  |  |  |  |  |  |  |  |  |  |  |  |  |  |  |  |  |  |  |  |  |  |  |  |  |  |  |  |  |  |  | *best-13* | BESTrophin (chloride channel) homolog |
|  |  |  |  |  |  |  |  |  |  |  |  |  |  |  |  |  |  |  |  |  |  |  |  |  |  |  |  |  |  |  |  |  |  |  |  |  |  | F59E11.2 |  |
|  |  |  |  |  |  |  |  |  |  |  |  |  |  |  |  |  |  |  |  |  |  |  |  |  |  |  |  |  |  |  |  |  |  |  |  |  |  | C03A3.3 |  |
|  |  |  |  |  |  |  |  |  |  |  |  |  |  |  |  |  |  |  |  |  |  |  |  |  |  |  |  |  |  |  |  |  |  |  |  |  |  | *sre-1* | Serpentine Receptor, class E (epsilon) |
|  |  |  |  |  |  |  |  |  |  |  |  |  |  |  |  |  |  |  |  |  |  |  |  |  |  |  |  |  |  |  |  |  |  |  |  |  |  | *daf-7* | abnormal DAuer Formation |
|  |  |  |  |  |  |  |  |  |  |  |  |  |  |  |  |  |  |  |  |  |  |  |  |  |  |  |  |  |  |  |  |  |  |  |  |  |  | *alr-1* | AristaLess (Drosophila homeodomain) Related |
|  |  |  |  |  |  |  |  |  |  |  |  |  |  |  |  |  |  |  |  |  |  |  |  |  |  |  |  |  |  |  |  |  |  |  |  |  |  | *lgc-43* | Ligand-Gated ion Channel |
|  |  |  |  |  |  |  |  |  |  |  |  |  |  |  |  |  |  |  |  |  |  |  |  |  |  |  |  |  |  |  |  |  |  |  |  |  |  | *acp-1* | ACid Phosphatase family |
|  |  |  |  |  |  |  |  |  |  |  |  |  |  |  |  |  |  |  |  |  |  |  |  |  |  |  |  |  |  |  |  |  |  |  |  |  |  | F56F3.4 |  |
|  |  |  |  |  |  |  |  |  |  |  |  |  |  |  |  |  |  |  |  |  |  |  |  |  |  |  |  |  |  |  |  |  |  |  |  |  |  | ZK337.2 |  |
|  |  |  |  |  |  |  |  |  |  |  |  |  |  |  |  |  |  |  |  |  |  |  |  |  |  |  |  |  |  |  |  |  |  |  |  |  |  | *cah-1* | Carbonic AnHydrase |
|  |  |  |  |  |  |  |  |  |  |  |  |  |  |  |  |  |  |  |  |  |  |  |  |  |  |  |  |  |  |  |  |  |  |  |  |  |  | K01A12.3 |  |

### Phenotypes enriched

|  |  |  |  |
| --- | --- | --- | --- |
| **Group name** | **Number in cluster** | **Enrichment** | **FDR corrected p** |
| shrinker | 3 | 69.19 | 0.0121 |

### Anatomy terms enriched

|  |  |  |  |
| --- | --- | --- | --- |
| **Group name** | **Number in cluster** | **Enrichment** | **FDR corrected p** |
| anterior ganglion | 9 | 9.88 | 0.000769 |
| anterior pharyngeal ganglion (post) | 8 | 10.98 | 0.001560 |
| head ganglion | 12 | 5.13 | 0.006720 |
| RME | 4 | 25.63 | 0.030800 |
| socket cell | 5 | 13.41 | 0.048800 |

### GO terms enriched

none found

### Expression clusters enriched

|  |  |  |  |
| --- | --- | --- | --- |
| **Group name** | **Number in cluster** | **Enrichment** | **FDR corrected p** |
| Genes that show selective expression in a subset of cell types vs broadly expressed in many cell types. Correspond to 20% - 57% of enriched\_genes for a given cell type. WBPaper00037950:GABAergic-motor-neurons\_embryo\_SelectivelyEnriched | 7 | 14.68 | 0.000192 |
| Genes up regulated by mir-243(n4759). | 20 | 2.56 | 0.013000 |
| Genes significantly enriched (> 2x, FDR < 5%) in a particular cell-type versus a reference sample of all cells at both embryonic and larval stages. WBPaper00037950:GABAergic-motor-neurons\_CoreEnriched | 4 | 19.22 | 0.014000 |

### Motifs enriched

|  |  |  |  |  |  |
| --- | --- | --- | --- | --- | --- |
| **Motif** | **Logo** | **Possible orthologs** | **Number of motifs in cluster** | **Enrichment** | **FDR corrected p** |
| Nkx2-9\_3082 |  | ceh-24 dsc-1 | 23 | 3.75 | 6.0e-06 |
| pTH10927 |  | tbx-39 tbx-38 mab-9 tbx-43 | 35 | 2.31 | 4.8e-05 |
| EN1\_1 |  | alr-1 (0.74) ceh-43 ceh-1 lim-7 ceh-31 ceh-9 ceh-16 lin-39 | 30 | 2.60 | 5.2e-05 |
| MSX2\_1 |  | ceh-1 ceh-14 | 34 | 2.35 | 5.4e-05 |
| Nkx1-1\_3856 |  | ceh-30 | 27 | 2.78 | 7.5e-05 |
| Pou3f2\_2824 |  | ceh-18 ceh-6 lin-39 | 29 | 2.57 | 1.1e-04 |
| Prrx1\_3442 |  | alr-1 (0.74) ceh-10 ceh-1 eyg-1 ceh-45 lin-39 | 31 | 2.43 | 1.1e-04 |
| pTH9923 |  | alr-1 (0.74) lim-6 (0.52) ceh-10 ceh-30 ceh-2 ceh-43 ceh-1 ceh-14 cog-1 egl-5 ceh-12 lim-7 ceh-18 dsc-1 ceh-36 ceh-16 ceh-23 pha-2 ceh-53 ceh-45 and 6 others  [full list] | 25 | 2.89 | 1.1e-04 |
| pTH6478 |  | lim-7 | 30 | 2.49 | 1.2e-04 |
| Ipf1\_3815 |  | alr-1 (0.74) npax-3 ceh-43 ceh-1 ceh-12 ceh-53 ceh-45 lin-39 | 28 | 2.61 | 1.4e-04 |
| Barx1\_2877 |  | ceh-43 | 28 | 2.57 | 1.8e-04 |
| pTH3751 |  | tbx-39 | 23 | 2.98 | 2.2e-04 |
| Ceh-22 |  | ceh-24 dsc-1 ceh-22 | 24 | 2.83 | 2.7e-04 |
| pTH0977 |  | sptf-3 klf-1 klf-2 | 40 | 1.93 | 3.1e-04 |
| pTH3998 |  | tbx-39 | 22 | 3.01 | 3.2e-04 |
| Pou2f3\_3986 |  | ceh-18 | 31 | 2.29 | 3.5e-04 |
| pTH6215 |  | ceh-43 ceh-12 lin-39 | 26 | 2.60 | 3.7e-04 |
| ISL1\_f1 |  | lim-7 lin-39 | 8 | 9.76 | 3.8e-04 |
| NKX28\_f1 |  | ceh-24 C34H4.5 | 33 | 2.17 | 4.4e-04 |
| Barx2\_3447 |  | ceh-43 | 28 | 2.43 | 4.8e-04 |
| pTH5812 |  | ceh-14 | 34 | 2.11 | 4.8e-04 |
| PRRX1\_3 |  | alr-1 (0.74) ceh-14 | 31 | 2.25 | 5.1e-04 |
| Egr1\_1 |  | ZC328.2 klf-1 klf-2 | 56 | 1.49 | 5.3e-04 |
| Nkx1-2\_3214 |  | ceh-30 | 26 | 2.55 | 5.3e-04 |
| POU3F1\_2 |  | unc-86 (0.7) ceh-18 | 24 | 2.67 | 6.7e-04 |
| pTH5887 |  | lin-39 | 38 | 1.92 | 7.0e-04 |
| Pou3f1\_3819 |  | ceh-6 | 28 | 2.37 | 7.1e-04 |
| pTH9226 |  | daf-19 | 60 | 1.40 | 7.1e-04 |
| Bsx\_3483 |  | ceh-31 | 26 | 2.48 | 8.1e-04 |
| pTH6562 |  | ceh-5 | 26 | 2.44 | 1.1e-03 |
| EN1\_2 |  | ceh-2 ceh-16 | 23 | 2.67 | 1.1e-03 |
| MA0599.1 |  | klf-1 klf-2 | 32 | 2.11 | 1.1e-03 |
| Hoxc4\_3491 |  | lin-39 | 27 | 2.34 | 1.3e-03 |
| pTH9297 |  | ceh-18 | 17 | 3.34 | 1.4e-03 |
| Hoxa7\_2668 |  | lin-39 | 28 | 2.25 | 1.6e-03 |
| pTH5828 |  | nhr-142 nhr-84 | 47 | 1.62 | 1.6e-03 |
| MA0066.1 |  | nhr-43 | 35 | 1.94 | 1.8e-03 |
| pTH3819 |  | ceh-18 | 26 | 2.34 | 2.0e-03 |
| pTH9480 |  | ces-2 C01B12.2 | 29 | 2.17 | 2.0e-03 |
| pTH9256 |  | ceh-18 | 17 | 3.22 | 2.1e-03 |
| Hlxb9\_3422 |  | ceh-12 | 25 | 2.39 | 2.1e-03 |
| Hoxb5\_3122 |  | lin-39 | 25 | 2.39 | 2.1e-03 |
| pTH9244 |  | tbx-39 | 27 | 2.26 | 2.2e-03 |
| Hoxd1\_3448 |  | ceh-12 | 25 | 2.38 | 2.2e-03 |
| En2\_0952 |  | lim-6 (0.52) lim-7 ceh-16 | 30 | 2.11 | 2.3e-03 |
| Vax2\_3500 |  | C02F12.10 | 14 | 3.78 | 2.3e-03 |
| pTH6569 |  | ceh-43 | 25 | 2.37 | 2.4e-03 |
| Hoxa3\_2783 |  | lin-39 | 34 | 1.94 | 2.5e-03 |
| CG7386\_F10-12\_SANGER\_5\_FBgn0035691 |  | F56D1.1 gei-3 | 31 | 2.05 | 2.5e-03 |
| SRP000217\_Klf4 |  | klf-1 klf-2 | 58 | 1.39 | 2.5e-03 |
| Vax1\_3499 |  | C02F12.10 | 25 | 2.36 | 2.6e-03 |
| Hoxa5\_3415 |  | lin-39 | 25 | 2.35 | 2.7e-03 |
| V$RORA2\_01 |  | nhr-213 nhr-118 | 29 | 2.13 | 2.7e-03 |
| pTH8997 |  | hmg-12 ceh-43 let-381 lin-39 | 32 | 2.00 | 2.8e-03 |
| pTH6327 |  | dsc-1 | 13 | 3.97 | 2.8e-03 |
| pTH6516 |  | F19F10.1 | 33 | 1.96 | 2.9e-03 |
| pTH6449 |  | ceh-43 | 25 | 2.33 | 3.0e-03 |
| pTH9182 |  | tbx-39 | 20 | 2.72 | 3.1e-03 |
| Hoxb3\_1720 |  | lin-39 | 25 | 2.32 | 3.2e-03 |
| En1\_3123 |  | ceh-16 | 24 | 2.39 | 3.2e-03 |
| pTH9930 |  | lin-29 fkh-7 | 41 | 1.71 | 3.3e-03 |
| Dlx3\_1030 |  | ceh-43 | 25 | 2.31 | 3.3e-03 |
| ONEC2\_si |  | ceh-48 | 32 | 1.97 | 3.5e-03 |
| pTH9219 |  | C01B12.2 | 6 | 10.38 | 3.5e-03 |
| Mw137 |  | blmp-1 (0.53) | 33 | 1.93 | 3.7e-03 |
| MA0069.1 |  | pax-3 pax-2 | 17 | 3.03 | 3.8e-03 |
| EPAS1\_si |  | hif-1 ztf-3 Y5F2A.4 | 39 | 1.75 | 3.8e-03 |
| Egr1\_2580 |  | ZC328.2 | 31 | 2.00 | 4.0e-03 |
| pTH10797 |  | lin-29 K11D2.4 | 41 | 1.68 | 4.4e-03 |
| pTH9381 |  | ceh-18 | 15 | 3.25 | 5.2e-03 |
| TFEB\_f1 |  | hlh-30 pax-1 aha-1 | 29 | 2.04 | 5.3e-03 |
| pTH9322 |  | nhr-10 | 41 | 1.67 | 5.3e-03 |
| MA0262.1 |  | mab-3 | 37 | 1.77 | 5.6e-03 |
| Evx1\_3952 |  | ceh-53 | 32 | 1.91 | 6.1e-03 |
| ARI3A\_do |  | cfi-1 | 31 | 1.94 | 6.1e-03 |
| exd\_SOLEXA\_2\_FBgn0000611 |  | ceh-32 ceh-20 | 22 | 2.40 | 6.4e-03 |
| pTH5922 |  | ceh-24 | 30 | 1.97 | 6.6e-03 |
| MA0049.1 |  | hbl-1 lin-39 | 39 | 1.70 | 6.8e-03 |
| pTH10013 |  | nhr-168 | 30 | 1.96 | 7.0e-03 |
| HOXA10\_1 |  | lin-39 php-3 pal-1 D1005.3 | 66 | 1.21 | 7.0e-03 |
| Spt15 |  | tbp-1 | 19 | 2.63 | 7.1e-03 |
| I$UBX\_01 |  | lin-39 | 57 | 1.36 | 7.2e-03 |
| MA0179.1 |  | alr-1 (0.74) ceh-8 ceh-30 ceh-43 ceh-1 lim-7 ceh-19 ceh-9 lin-39 | 21 | 2.44 | 7.4e-03 |
| OTX2\_1 |  | pha-2 ceh-53 ceh-45 | 25 | 2.18 | 7.5e-03 |
| V$CEBP\_01 |  | C48E7.11 | 45 | 1.56 | 7.6e-03 |
| Hoxb4\_2627 |  | lin-39 | 25 | 2.17 | 7.8e-03 |
| pTH5111 |  | aha-1 | 33 | 1.85 | 7.8e-03 |
| pTH6268 |  | ceh-2 | 6 | 8.63 | 7.9e-03 |
| Dlx1\_1741 |  | ceh-43 | 26 | 2.12 | 8.0e-03 |
| MA0476.1 |  | fos-1 jun-1 | 27 | 2.07 | 8.2e-03 |
| HXD10\_f1 |  | nhr-2 php-3 | 21 | 2.42 | 8.2e-03 |
| pTH9118 |  | eor-1 egrh-3 | 43 | 1.59 | 8.3e-03 |
| pTH10647 |  | nhr-232 | 45 | 1.55 | 8.3e-03 |
| Hoxa6\_1040 |  | lin-39 | 27 | 2.06 | 8.6e-03 |
| pTH10795 |  | ztf-14 | 33 | 1.83 | 8.8e-03 |
| pTH9260 |  | mel-28 | 36 | 1.74 | 9.3e-03 |
| Hoxa4\_3426 |  | lin-39 | 24 | 2.20 | 9.3e-03 |
| Dlx2\_2273 |  | ceh-43 | 23 | 2.25 | 9.4e-03 |
| pTH6447 |  | ceh-19 | 40 | 1.64 | 9.6e-03 |
| Pou3f4\_3773 |  | ceh-6 | 25 | 2.13 | 1.0e-02 |
| pTH6003 |  | nhr-134 | 22 | 2.30 | 1.0e-02 |
| HXA5\_si |  | lin-39 | 28 | 1.99 | 1.0e-02 |
| Nkx6-3\_3446 |  | cog-1 | 25 | 2.13 | 1.0e-02 |
| Emx2\_3420 |  | ceh-2 | 35 | 1.76 | 1.1e-02 |
| Hoxb8\_3780 |  | lin-39 | 24 | 2.17 | 1.1e-02 |
| ems\_FlyReg\_FBgn0000576 |  | ceh-2 skn-1 | 24 | 2.16 | 1.1e-02 |
| MEIS1\_f2 |  | ceh-32 | 26 | 2.05 | 1.2e-02 |
| FOXJ3\_2 |  | lin-31 | 17 | 2.67 | 1.3e-02 |
| SRF\_do |  | unc-120 | 30 | 1.88 | 1.3e-02 |
| pTH6436 |  | ceh-53 | 19 | 2.47 | 1.3e-02 |
| Irx4\_2242 |  | irx-1 | 59 | 1.30 | 1.3e-02 |
| MA0029.1 |  | ztf-29 (0.51) | 24 | 2.13 | 1.4e-02 |
| Gsh2\_3990 |  | ceh-31 | 22 | 2.24 | 1.4e-02 |
| pTH9237 |  | mel-28 | 34 | 1.75 | 1.4e-02 |
| V$TCF11\_01 |  | skn-1 | 28 | 1.94 | 1.5e-02 |
| MA0502.1 |  | lin-31 nfya-2 ceh-20 dro-1 | 35 | 1.72 | 1.5e-02 |
| pTH10654 |  | ceh-90 | 21 | 2.29 | 1.5e-02 |
| Hoxa7\_3750 |  | lin-39 | 26 | 2.02 | 1.5e-02 |
| V$OCT1\_03 |  | ceh-18 | 32 | 1.80 | 1.5e-02 |
| Poxm\_SOLEXA\_5\_FBgn0003129 |  | pax-2 | 22 | 2.22 | 1.5e-02 |
| ELK3\_f1 |  | lin-1 | 35 | 1.72 | 1.6e-02 |
| Hoxa2\_3079 |  | lin-39 | 23 | 2.16 | 1.6e-02 |
| MA0244.1 |  | C48E7.11 | 29 | 1.88 | 1.7e-02 |
| V$BRN2\_01 |  | ceh-18 | 61 | 1.26 | 1.7e-02 |
| PURA\_f1 |  | plp-2 | 28 | 1.91 | 1.8e-02 |
| Tcfap2a\_2337 |  | aptf-1 | 13 | 3.14 | 1.8e-02 |
| HLH1 |  | hlh-1 hlh-11 | 39 | 1.61 | 1.8e-02 |
| GM12878\_ETS1\_HudsonAlpha |  | nhr-28 nhr-273 lin-1 | 40 | 1.59 | 1.8e-02 |
| MA0118.1 |  | ref-2 | 12 | 3.34 | 1.8e-02 |
| MA0331.1 |  | unc-120 | 62 | 1.25 | 1.9e-02 |
| Zfp691\_0895 |  | F21A9.2 | 28 | 1.91 | 1.9e-02 |
| MA0027.1 |  | ceh-16 | 11 | 3.59 | 1.9e-02 |
| pTH9082 |  | mab-23 | 18 | 2.47 | 1.9e-02 |
| Cart1\_1275 |  | alr-1 (0.74) ceh-18 | 38 | 1.63 | 1.9e-02 |
| MA0007.2 |  | nhr-255 npax-1 | 26 | 1.98 | 1.9e-02 |
| V$S8\_01 |  | ceh-45 | 36 | 1.67 | 2.0e-02 |
| GM12878\_MEF2A\_HudsonAlpha |  | mef-2 | 65 | 1.20 | 2.0e-02 |
| pTH9290 |  | ceh-18 tbp-1 | 30 | 1.83 | 2.0e-02 |
| V$CDC5\_01 |  | irx-1 D1081.8 | 25 | 2.01 | 2.1e-02 |
| Lhx1\_2240 |  | lim-7 | 26 | 1.97 | 2.1e-02 |
| I$DFD\_01 |  | lin-39 | 28 | 1.89 | 2.1e-02 |
| ELK1\_2 |  | lin-1 | 28 | 1.89 | 2.1e-02 |
| KLF8\_f1 |  | klf-1 | 34 | 1.71 | 2.1e-02 |
| Six4\_2860 |  | ceh-32 | 20 | 2.29 | 2.1e-02 |
| PO3F2\_si |  | ceh-18 | 32 | 1.76 | 2.1e-02 |
| Tcf2\_0913 |  | hmbx-1 | 15 | 2.75 | 2.2e-02 |
| Hoxc8\_3429 |  | lin-39 | 26 | 1.96 | 2.2e-02 |
| pTH9282 |  | attf-1 | 9 | 4.22 | 2.2e-02 |
| Sox1\_2631 |  | sox-4 | 36 | 1.66 | 2.2e-02 |
| Etv6 |  | lin-1 C24A1.2 | 31 | 1.78 | 2.2e-02 |
| pTH9245 |  | ceh-18 | 42 | 1.53 | 2.3e-02 |
| Vsx1\_1728 |  | alr-1 (0.74) | 25 | 1.99 | 2.4e-02 |
| SPDEF\_3 |  | lin-1 | 33 | 1.72 | 2.4e-02 |
| FOXO1\_3 |  | ZC328.2 daf-16 | 12 | 3.21 | 2.4e-02 |
| pTH9335 |  | mel-28 | 32 | 1.74 | 2.5e-02 |
| pTH9199 |  | daf-19 | 56 | 1.32 | 2.5e-02 |
| Pou2f1\_3081 |  | ceh-18 | 23 | 2.07 | 2.5e-02 |
| pTH9900 |  | C46E10.8 | 26 | 1.93 | 2.6e-02 |
| Hoxa13\_3126 |  | pal-1 | 55 | 1.33 | 2.7e-02 |
| Foxk1\_1 |  | lin-31 | 36 | 1.64 | 2.7e-02 |
| Hmx3\_3490 |  | ceh-9 | 25 | 1.96 | 2.8e-02 |
| pTH9216 |  | ceh-18 | 22 | 2.10 | 2.8e-02 |
| RORA\_1 |  | nhr-213 | 31 | 1.76 | 2.8e-02 |
| Tcf7\_0950 |  | pop-1 | 37 | 1.61 | 2.8e-02 |
| Irx2\_0900 |  | irx-1 | 29 | 1.81 | 2.8e-02 |
| pTH9173 |  | efl-2 | 31 | 1.75 | 2.8e-02 |
| Sox1\_1 |  | sox-4 | 12 | 3.14 | 2.8e-02 |
| CG4854\_SANGER\_10\_FBgn0038766 |  | K11D2.4 | 36 | 1.63 | 2.8e-02 |
| Sox4 |  | pop-1 | 45 | 1.47 | 2.9e-02 |
| Eip74EF\_FlyReg\_FBgn0000567 |  | C24A1.2 | 35 | 1.65 | 2.9e-02 |
| pTH10633 |  | R07H5.10 | 62 | 1.23 | 3.0e-02 |
| pTH6556 |  | lim-6 (0.52) | 39 | 1.56 | 3.1e-02 |
| pTH9254 |  | mel-28 | 21 | 2.14 | 3.1e-02 |
| Spdef |  | lin-1 | 37 | 1.60 | 3.2e-02 |
| MA0033.1 |  | lin-31 | 24 | 1.98 | 3.2e-02 |
| pTH9242 |  | mel-28 | 37 | 1.59 | 3.4e-02 |
| MA0139.1 |  | F58G1.2 | 54 | 1.33 | 3.4e-02 |
| pTH4325 |  | ceh-18 | 21 | 2.12 | 3.4e-02 |
| FLI1\_f1 |  | lin-1 | 23 | 2.01 | 3.4e-02 |
| MA0543.1 |  | eor-1 | 36 | 1.61 | 3.5e-02 |
| Nsy-7 |  | nsy-7 | 19 | 2.22 | 3.7e-02 |
| pTH5924 |  | nhr-255 | 25 | 1.91 | 3.7e-02 |
| Irx3\_0920 |  | irx-1 | 32 | 1.69 | 3.8e-02 |
| Meox1\_2310 |  | ceh-31 | 10 | 3.47 | 3.8e-02 |
| Nkx6-1\_2825 |  | cog-1 | 23 | 1.99 | 3.9e-02 |
| Sox15\_3457 |  | sox-4 | 20 | 2.14 | 4.0e-02 |
| pTH3510 |  | nhr-2 nhr-86 | 30 | 1.73 | 4.1e-02 |
| pTH10810 |  | lsy-2 | 13 | 2.80 | 4.1e-02 |
| HNF4G\_f1 |  | nhr-62 | 56 | 1.29 | 4.1e-02 |
| MA0098.2 |  | lin-1 | 31 | 1.70 | 4.3e-02 |
| pTH5561 |  | nhr-239 | 35 | 1.61 | 4.3e-02 |
| pTH5919 |  | irx-1 | 21 | 2.07 | 4.4e-02 |
| FOXB1\_1 |  | lin-31 | 27 | 1.82 | 4.4e-02 |
| pTH6425 |  | ceh-20 | 20 | 2.12 | 4.4e-02 |
| pTH8745 |  | attf-1 | 8 | 4.12 | 4.5e-02 |
| V$MEF2\_01 |  | mef-2 | 20 | 2.11 | 4.5e-02 |
| Optix\_SOLEXA\_FBgn0025360 |  | ceh-34 | 28 | 1.78 | 4.5e-02 |
| MA0503.1 |  | ceh-24 | 39 | 1.53 | 4.5e-02 |
| pTH6445 |  | ceh-5 | 30 | 1.72 | 4.6e-02 |
| V$HOX13\_01 |  | lin-39 | 19 | 2.17 | 4.6e-02 |
| Pbx1\_3203 |  | ceh-20 | 38 | 1.54 | 4.7e-02 |
| Barhl1\_1 |  | ceh-31 | 60 | 1.24 | 4.7e-02 |
| MA0041.1 |  | let-381 | 31 | 1.69 | 4.8e-02 |
| pTH5118 |  | cfi-1 | 25 | 1.87 | 4.8e-02 |
| Tcf1\_2666 |  | hmbx-1 | 16 | 2.39 | 4.8e-02 |
| ovo\_FlyReg\_FBgn0003028 |  | lin-48 (0.56) | 28 | 1.77 | 4.8e-02 |
| pTH10640 |  | dmd-4 | 34 | 1.62 | 4.9e-02 |
| Atf6\_SANGER\_5\_FBgn0033010 |  | atf-6 | 61 | 1.22 | 4.9e-02 |
| MCR\_f1 |  | nhr-255 | 63 | 1.20 | 5.0e-02 |
| MITF\_f1 |  | hlh-30 | 29 | 1.74 | 5.0e-02 |

### Correlated (and anti-correlated) transcription factors

|  |  |
| --- | --- |
| **Transcription factor** | **Correlation** |
| ZK337.2 | 0.89 |
| hlh-19 | 0.83 |
| alr-1 | 0.74 |
| odr-7 | 0.73 |
| elt-6 | 0.70 |
| unc-86 | 0.70 |
| nhr-67 | 0.68 |
| ets-5 | 0.65 |
| F13H6.1 | 0.65 |
| nhr-187 | 0.65 |
| fkh-5 | 0.64 |
| lag-1 | 0.63 |
| egl-13 | 0.61 |
| nhr-25 | 0.61 |
| moe-3 | 0.61 |
| nhr-181 | 0.59 |
| bed-3 | 0.59 |
| nhr-216 | 0.58 |
| nhr-40 | 0.58 |
| nhr-175 | 0.58 |
| lin-48 | 0.56 |
| daf-12 | 0.55 |
| nhr-14 | 0.55 |
| lin-11 | 0.54 |
| blmp-1 | 0.53 |
| ztf-28 | -0.42 |
| hmg-3 | -0.42 |
| nhr-176 | -0.43 |
| nhr-271 | -0.43 |
| aptf-2 | -0.44 |
| D2030.7 | -0.44 |
| F17C11.1 | -0.44 |
| sup-35 | -0.44 |
| nhr-2 | -0.44 |
| ceh-51 | -0.45 |
| Y48G9A.11 | -0.46 |
| F19F10.1 | -0.46 |
| nhr-269 | -0.46 |
| ccch-3 | -0.47 |
| pzf-1 | -0.51 |
| pqn-75 | -0.53 |
| nhr-171 | -0.53 |
| nhr-108 | -0.53 |
| zip-8 | -0.54 |
| cep-1 | -0.55 |
| F49E8.2 | -0.57 |
| nhr-248 | -0.59 |
| nhr-210 | -0.62 |
| cey-2 | -0.63 |
| T26A5.8 | -0.70 |

### ChIP peaks enriched

none found
