# Supplementary material for: Overlapping cell population expression profiling and regulatory inference in C. elegans
Source: BMC Genomics. 2016 Feb 29;17:159. doi: 10.1186/s12864-016-2482-z (PMC4772325; doi:10.1186/s12864-016-2482-z)
Supplement: Additional file 13: — Web supplement. (DOC 21 kb) [file 12864_2016_2482_MOESM13_ESM.zip › sortWeb/clusters/hier.300.clusters/260.html]

Cluster 260 

## Cluster 260

### Expression

| cnd-1 rep. 1 | cnd-1 rep. 2 | cnd-1 rep. 3 | pha-4 rep. 1 | pha-4 rep. 2 | pha-4 rep. 3 | ceh-27 | ceh-36 | ceh-6 | F21D5.9 | mir-57 | mls-2 | pal-1 | pros-1 | ttx-3 | unc-130 | hlh-16 | irx-1 | ceh-6 (+) hlh-16 (+) | ceh-6 (+) hlh-16 (-) | ceh-6 (-) hlh-16 (+) | cnd-1 singlets | pha-4 singlets | 0 | 60 | 120 | 150 | 180 | 240 | 330 | 390 | 420 | 480 | 540 | 570 | 600 | 630 | 660 | NAME | Functional description |
| --- | --- | --- | --- | --- | --- | --- | --- | --- | --- | --- | --- | --- | --- | --- | --- | --- | --- | --- | --- | --- | --- | --- | --- | --- | --- | --- | --- | --- | --- | --- | --- | --- | --- | --- | --- | --- | --- | --- | --- |
|  |  |  |  |  |  |  |  |  |  |  |  |  |  |  |  |  |  |  |  |  |  |  |  |  |  |  |  |  |  |  |  |  |  |  |  |  |  | *tag-138* | Temporarily Assigned Gene name |
|  |  |  |  |  |  |  |  |  |  |  |  |  |  |  |  |  |  |  |  |  |  |  |  |  |  |  |  |  |  |  |  |  |  |  |  |  |  | *linc-145* | Long Intervening Non-Coding RNA |
|  |  |  |  |  |  |  |  |  |  |  |  |  |  |  |  |  |  |  |  |  |  |  |  |  |  |  |  |  |  |  |  |  |  |  |  |  |  | F58H1.14 |  |
|  |  |  |  |  |  |  |  |  |  |  |  |  |  |  |  |  |  |  |  |  |  |  |  |  |  |  |  |  |  |  |  |  |  |  |  |  |  | F37H8.3 |  |
|  |  |  |  |  |  |  |  |  |  |  |  |  |  |  |  |  |  |  |  |  |  |  |  |  |  |  |  |  |  |  |  |  |  |  |  |  |  | F58G6.8 |  |
|  |  |  |  |  |  |  |  |  |  |  |  |  |  |  |  |  |  |  |  |  |  |  |  |  |  |  |  |  |  |  |  |  |  |  |  |  |  | F57F5.1 |  |
|  |  |  |  |  |  |  |  |  |  |  |  |  |  |  |  |  |  |  |  |  |  |  |  |  |  |  |  |  |  |  |  |  |  |  |  |  |  | K01D12.2 |  |
|  |  |  |  |  |  |  |  |  |  |  |  |  |  |  |  |  |  |  |  |  |  |  |  |  |  |  |  |  |  |  |  |  |  |  |  |  |  | *npr-26* | NeuroPeptide Receptor family |
|  |  |  |  |  |  |  |  |  |  |  |  |  |  |  |  |  |  |  |  |  |  |  |  |  |  |  |  |  |  |  |  |  |  |  |  |  |  | *lgc-49* | Ligand-Gated ion Channel |
|  |  |  |  |  |  |  |  |  |  |  |  |  |  |  |  |  |  |  |  |  |  |  |  |  |  |  |  |  |  |  |  |  |  |  |  |  |  | *ser-1* | SERotonin/octopamine receptor family |
|  |  |  |  |  |  |  |  |  |  |  |  |  |  |  |  |  |  |  |  |  |  |  |  |  |  |  |  |  |  |  |  |  |  |  |  |  |  | *ins-1* | INSulin related |
|  |  |  |  |  |  |  |  |  |  |  |  |  |  |  |  |  |  |  |  |  |  |  |  |  |  |  |  |  |  |  |  |  |  |  |  |  |  | K07D4.4 |  |
|  |  |  |  |  |  |  |  |  |  |  |  |  |  |  |  |  |  |  |  |  |  |  |  |  |  |  |  |  |  |  |  |  |  |  |  |  |  | C07A12.2 |  |
|  |  |  |  |  |  |  |  |  |  |  |  |  |  |  |  |  |  |  |  |  |  |  |  |  |  |  |  |  |  |  |  |  |  |  |  |  |  | C50E3.6 |  |
|  |  |  |  |  |  |  |  |  |  |  |  |  |  |  |  |  |  |  |  |  |  |  |  |  |  |  |  |  |  |  |  |  |  |  |  |  |  | C50D2.2 |  |
|  |  |  |  |  |  |  |  |  |  |  |  |  |  |  |  |  |  |  |  |  |  |  |  |  |  |  |  |  |  |  |  |  |  |  |  |  |  | *lgc-41* | Ligand-Gated ion Channel |
|  |  |  |  |  |  |  |  |  |  |  |  |  |  |  |  |  |  |  |  |  |  |  |  |  |  |  |  |  |  |  |  |  |  |  |  |  |  | *iglr-3* | IG(immunoglobulin) and LRR(Leucine Rich Repeat) domains |
|  |  |  |  |  |  |  |  |  |  |  |  |  |  |  |  |  |  |  |  |  |  |  |  |  |  |  |  |  |  |  |  |  |  |  |  |  |  | T05H4.3 |  |
|  |  |  |  |  |  |  |  |  |  |  |  |  |  |  |  |  |  |  |  |  |  |  |  |  |  |  |  |  |  |  |  |  |  |  |  |  |  | T25D10.7 |  |
|  |  |  |  |  |  |  |  |  |  |  |  |  |  |  |  |  |  |  |  |  |  |  |  |  |  |  |  |  |  |  |  |  |  |  |  |  |  | W01D2.3 |  |
|  |  |  |  |  |  |  |  |  |  |  |  |  |  |  |  |  |  |  |  |  |  |  |  |  |  |  |  |  |  |  |  |  |  |  |  |  |  | F16F9.18 |  |
|  |  |  |  |  |  |  |  |  |  |  |  |  |  |  |  |  |  |  |  |  |  |  |  |  |  |  |  |  |  |  |  |  |  |  |  |  |  | F55C12.15 |  |
|  |  |  |  |  |  |  |  |  |  |  |  |  |  |  |  |  |  |  |  |  |  |  |  |  |  |  |  |  |  |  |  |  |  |  |  |  |  | F22A3.12 |  |
|  |  |  |  |  |  |  |  |  |  |  |  |  |  |  |  |  |  |  |  |  |  |  |  |  |  |  |  |  |  |  |  |  |  |  |  |  |  | T25B9.3 |  |
|  |  |  |  |  |  |  |  |  |  |  |  |  |  |  |  |  |  |  |  |  |  |  |  |  |  |  |  |  |  |  |  |  |  |  |  |  |  | *npr-25* | NeuroPeptide Receptor family |
|  |  |  |  |  |  |  |  |  |  |  |  |  |  |  |  |  |  |  |  |  |  |  |  |  |  |  |  |  |  |  |  |  |  |  |  |  |  | *ubc-17* | UBiquitin Conjugating enzyme |
|  |  |  |  |  |  |  |  |  |  |  |  |  |  |  |  |  |  |  |  |  |  |  |  |  |  |  |  |  |  |  |  |  |  |  |  |  |  | Y67D8C.12 |  |
|  |  |  |  |  |  |  |  |  |  |  |  |  |  |  |  |  |  |  |  |  |  |  |  |  |  |  |  |  |  |  |  |  |  |  |  |  |  | K10D11.6 |  |
|  |  |  |  |  |  |  |  |  |  |  |  |  |  |  |  |  |  |  |  |  |  |  |  |  |  |  |  |  |  |  |  |  |  |  |  |  |  | T02E9.24 |  |
|  |  |  |  |  |  |  |  |  |  |  |  |  |  |  |  |  |  |  |  |  |  |  |  |  |  |  |  |  |  |  |  |  |  |  |  |  |  | *cat-1* | abnormal CATecholamine distribution |
|  |  |  |  |  |  |  |  |  |  |  |  |  |  |  |  |  |  |  |  |  |  |  |  |  |  |  |  |  |  |  |  |  |  |  |  |  |  | *glr-8* | GLutamate Receptor family (AMPA) |
|  |  |  |  |  |  |  |  |  |  |  |  |  |  |  |  |  |  |  |  |  |  |  |  |  |  |  |  |  |  |  |  |  |  |  |  |  |  | C09B9.1 |  |
|  |  |  |  |  |  |  |  |  |  |  |  |  |  |  |  |  |  |  |  |  |  |  |  |  |  |  |  |  |  |  |  |  |  |  |  |  |  | *srd-46* | Serpentine Receptor, class D (delta) |
|  |  |  |  |  |  |  |  |  |  |  |  |  |  |  |  |  |  |  |  |  |  |  |  |  |  |  |  |  |  |  |  |  |  |  |  |  |  | *gcy-19* | Guanylyl CYclase |
|  |  |  |  |  |  |  |  |  |  |  |  |  |  |  |  |  |  |  |  |  |  |  |  |  |  |  |  |  |  |  |  |  |  |  |  |  |  | *srh-15* | Serpentine Receptor, class H |
|  |  |  |  |  |  |  |  |  |  |  |  |  |  |  |  |  |  |  |  |  |  |  |  |  |  |  |  |  |  |  |  |  |  |  |  |  |  | *mnm-2* | M Neuron Morphology abnormal |
|  |  |  |  |  |  |  |  |  |  |  |  |  |  |  |  |  |  |  |  |  |  |  |  |  |  |  |  |  |  |  |  |  |  |  |  |  |  | *fbxa-154* | F-box A protein |
|  |  |  |  |  |  |  |  |  |  |  |  |  |  |  |  |  |  |  |  |  |  |  |  |  |  |  |  |  |  |  |  |  |  |  |  |  |  | *nhr-67* | Nuclear Hormone Receptor family |
|  |  |  |  |  |  |  |  |  |  |  |  |  |  |  |  |  |  |  |  |  |  |  |  |  |  |  |  |  |  |  |  |  |  |  |  |  |  | *lag-1* | Lin-12 And Glp-1 phenotype |
|  |  |  |  |  |  |  |  |  |  |  |  |  |  |  |  |  |  |  |  |  |  |  |  |  |  |  |  |  |  |  |  |  |  |  |  |  |  | *pqn-74* | Prion-like-(Q/N-rich)-domain-bearing protein |
|  |  |  |  |  |  |  |  |  |  |  |  |  |  |  |  |  |  |  |  |  |  |  |  |  |  |  |  |  |  |  |  |  |  |  |  |  |  | C10B5.3 |  |
|  |  |  |  |  |  |  |  |  |  |  |  |  |  |  |  |  |  |  |  |  |  |  |  |  |  |  |  |  |  |  |  |  |  |  |  |  |  | R11.2 |  |
|  |  |  |  |  |  |  |  |  |  |  |  |  |  |  |  |  |  |  |  |  |  |  |  |  |  |  |  |  |  |  |  |  |  |  |  |  |  | *ggr-2* | GABA/Glycine Receptor family (see gbr) |
|  |  |  |  |  |  |  |  |  |  |  |  |  |  |  |  |  |  |  |  |  |  |  |  |  |  |  |  |  |  |  |  |  |  |  |  |  |  | C09G1.5 |  |
|  |  |  |  |  |  |  |  |  |  |  |  |  |  |  |  |  |  |  |  |  |  |  |  |  |  |  |  |  |  |  |  |  |  |  |  |  |  | C10E2.4 |  |
|  |  |  |  |  |  |  |  |  |  |  |  |  |  |  |  |  |  |  |  |  |  |  |  |  |  |  |  |  |  |  |  |  |  |  |  |  |  | C10E2.5 |  |
|  |  |  |  |  |  |  |  |  |  |  |  |  |  |  |  |  |  |  |  |  |  |  |  |  |  |  |  |  |  |  |  |  |  |  |  |  |  | F16B4.2 |  |
|  |  |  |  |  |  |  |  |  |  |  |  |  |  |  |  |  |  |  |  |  |  |  |  |  |  |  |  |  |  |  |  |  |  |  |  |  |  | *frpr-6* | FMRFamide Peptide Receptor family |
|  |  |  |  |  |  |  |  |  |  |  |  |  |  |  |  |  |  |  |  |  |  |  |  |  |  |  |  |  |  |  |  |  |  |  |  |  |  | *acr-19* | AcetylCholine Receptor |
|  |  |  |  |  |  |  |  |  |  |  |  |  |  |  |  |  |  |  |  |  |  |  |  |  |  |  |  |  |  |  |  |  |  |  |  |  |  | *npr-29* | NeuroPeptide Receptor family |
|  |  |  |  |  |  |  |  |  |  |  |  |  |  |  |  |  |  |  |  |  |  |  |  |  |  |  |  |  |  |  |  |  |  |  |  |  |  | *hlh-19* | Helix Loop Helix |
|  |  |  |  |  |  |  |  |  |  |  |  |  |  |  |  |  |  |  |  |  |  |  |  |  |  |  |  |  |  |  |  |  |  |  |  |  |  | *nhr-236* | Nuclear Hormone Receptor family |
|  |  |  |  |  |  |  |  |  |  |  |  |  |  |  |  |  |  |  |  |  |  |  |  |  |  |  |  |  |  |  |  |  |  |  |  |  |  | C50F4.9 |  |
|  |  |  |  |  |  |  |  |  |  |  |  |  |  |  |  |  |  |  |  |  |  |  |  |  |  |  |  |  |  |  |  |  |  |  |  |  |  | *srx-2* | Serpentine Receptor, class X |
|  |  |  |  |  |  |  |  |  |  |  |  |  |  |  |  |  |  |  |  |  |  |  |  |  |  |  |  |  |  |  |  |  |  |  |  |  |  | H35N09.1 |  |

### Phenotypes enriched

none found

### Anatomy terms enriched

none found

### GO terms enriched

|  |  |  |
| --- | --- | --- |
| **GO term** | **Number of genes** | **FDR-corrected p-value** |
| ligand-gated channel activity | 5 | 0.0042 |
| extracellular ligand-gated ion channel activity | 4 | 0.0170 |
| synapse part | 4 | 0.0420 |
| ion transport | 6 | 0.0450 |

### Expression clusters enriched

|  |  |  |  |
| --- | --- | --- | --- |
| **Group name** | **Number in cluster** | **Enrichment** | **FDR corrected p** |
| Genes that show selective expression in a subset of cell types vs broadly expressed in many cell types. Correspond to 20% - 57% of enriched\_genes for a given cell type. WBPaper00037950:all-neurons\_larva\_SelectivelyEnriched | 14 | 7.31 | 2.18e-06 |
| Larval Pan-neural Enriched Genes. | 21 | 3.88 | 1.03e-05 |
| Genes significantly enriched (> 2x, FDR < 5%) in a particular cell-type versus a reference sample of all cells at the same stage. WBPaper00037950:all-neurons\_larva\_enriched | 16 | 4.50 | 9.55e-05 |
| Genes that show selective expression in a subset of cell types vs broadly expressed in many cell types. Correspond to 20% - 57% of enriched\_genes for a given cell type. WBPaper00037950:A-class-motor-neurons\_larva\_SelectivelyEnriched | 7 | 7.10 | 1.37e-02 |

### Motifs enriched

|  |  |  |  |  |  |
| --- | --- | --- | --- | --- | --- |
| **Motif** | **Logo** | **Possible orthologs** | **Number of motifs in cluster** | **Enrichment** | **FDR corrected p** |
| MA0165.1 |  | ceh-24 (0.55) ceh-13 php-3 lin-39 pal-1 T27F2.4 D1005.3 | 26 | 3.67 | 3.9e-07 |
| Nkx3-1\_2923 |  | ceh-24 (0.55) dsc-1 | 40 | 2.29 | 4.0e-07 |
| klu\_SANGER\_10\_FBgn0013469 |  | ZC328.2 | 34 | 2.66 | 7.1e-07 |
| NKX28\_f1 |  | ceh-24 (0.55) ces-1 T22H9.4 C34H4.5 | 27 | 3.37 | 8.8e-07 |
| ETV4\_f1 |  | lin-1 C24A1.2 | 31 | 2.87 | 1.2e-06 |
| MA0079.3 |  | klf-1 klf-2 | 20 | 4.71 | 1.2e-06 |
| ELF3\_f1 |  | K02D7.2 unc-120 C24A1.2 | 30 | 2.96 | 1.3e-06 |
| I$E74A\_01 |  | nhr-19 lin-1 C24A1.2 | 31 | 2.74 | 3.3e-06 |
| NK7.1\_SOLEXA\_FBgn0024321 |  | alr-1 (0.72) ceh-2 (0.54) lim-6 ceh-10 ceh-30 lim-7 ceh-45 ceh-31 ceh-18 lin-39 ceh-12 lim-4 ceh-23 ceh-14 ceh-1 ceh-53 dsc-1 cog-1 pha-2 ceh-36 and 9 others  [full list] | 30 | 2.78 | 4.6e-06 |
| Elf5 |  | C24A1.2 | 35 | 2.38 | 5.3e-06 |
| MA0459.1 |  | nhr-239 nhr-62 nhr-2 | 30 | 2.76 | 5.4e-06 |
| pTH3477 |  | daf-16 | 34 | 2.42 | 7.0e-06 |
| OLIG2\_1 |  | hlh-32 hlh-8 hlh-15 ngn-1 lin-31 | 33 | 2.49 | 7.2e-06 |
| ISL2\_1 |  | alr-1 (0.72) lim-7 ceh-9 ceh-31 lin-39 ceh-1 ceh-16 ceh-43 | 37 | 2.23 | 7.4e-06 |
| pTH5099 |  | lin-32 hlh-15 hlh-14 hlh-1 hlh-11 | 39 | 2.12 | 7.5e-06 |
| ERG\_1 |  | lin-1 C24A1.2 | 33 | 2.47 | 8.2e-06 |
| Evx2\_2645 |  | alr-1 (0.72) npax-3 ceh-45 ceh-18 ceh-12 lin-39 ceh-1 ceh-53 ceh-43 | 36 | 2.26 | 9.5e-06 |
| gl\_SANGER\_5\_FBgn0004618 |  | ztf-28 che-1 ces-1 | 44 | 1.86 | 1.0e-05 |
| pTH6562 |  | ceh-5 | 38 | 2.14 | 1.0e-05 |
| UNCX\_1 |  | alr-1 (0.72) cfi-1 ceh-14 ZC204.2 | 29 | 2.76 | 1.1e-05 |
| pTH5922 |  | ceh-24 (0.55) | 35 | 2.31 | 1.1e-05 |
| pTH10633 |  | R07H5.10 | 24 | 3.30 | 1.3e-05 |
| pTH3846 |  | daf-16 pha-4 fkh-7 fkh-8 lin-31 fkh-10 let-381 | 32 | 2.49 | 1.3e-05 |
| Hmx\_SOLEXA\_FBgn0085448 |  | alr-1 (0.72) ceh-24 (0.55) ceh-8 ceh-30 ceh-19 lim-7 ceh-9 ceh-31 lin-39 ceh-1 cog-1 ceh-43 | 29 | 2.73 | 1.4e-05 |
| pTH10650 |  | nhr-153 | 38 | 2.10 | 1.7e-05 |
| Nkx1-2\_3214 |  | ceh-30 | 36 | 2.21 | 1.8e-05 |
| eve\_FlyReg\_FBgn0000606 |  | hmg-12 lin-39 hmbx-1 ceh-53 lin-31 ceh-43 let-381 Y116A8C.22 | 43 | 1.87 | 1.8e-05 |
| tgo\_tai\_SANGER\_5\_FBgn0015014 |  | pax-1 aha-1 hlh-30 mdl-1 | 29 | 2.69 | 1.8e-05 |
| pTH9355 |  | klf-1 ZC328.2 klf-2 | 39 | 2.04 | 1.9e-05 |
| pTH10787 |  | alr-1 (0.72) ceh-10 eyg-1 ceh-45 lin-39 ceh-1 | 28 | 2.77 | 1.9e-05 |
| Hoxd11\_3873 |  | ceh-13 php-3 | 48 | 1.66 | 2.0e-05 |
| Ceh-22 |  | ceh-24 (0.55) ceh-22 dsc-1 | 33 | 2.37 | 2.1e-05 |
| I$DFD\_01 |  | lin-39 | 37 | 2.13 | 2.2e-05 |
| HLH4C\_da\_SANGER\_5\_3\_FBgn0011277 |  | K02D7.2 hlh-8 hlh-15 ces-1 hlh-2 hlh-1 | 36 | 2.18 | 2.3e-05 |
| Sox1\_2631 |  | sox-4 | 34 | 2.29 | 2.5e-05 |
| pTH2820 |  | ZC328.2 | 32 | 2.42 | 2.5e-05 |
| Elf3\_3876 |  | C24A1.2 | 35 | 2.22 | 2.7e-05 |
| pTH4269 |  | F13H6.1 (0.58) nhr-2 nhr-177 nhr-86 | 35 | 2.22 | 2.9e-05 |
| Vsx1\_1728 |  | alr-1 (0.72) | 26 | 2.90 | 3.0e-05 |
| MA0124.1 |  | ceh-24 (0.55) ceh-48 | 10 | 9.52 | 3.0e-05 |
| BARHL2\_6 |  | ceh-31 lin-39 ceh-1 ceh-14 ceh-43 | 40 | 1.95 | 3.4e-05 |
| MEF2D\_1 |  | mef-2 | 27 | 2.78 | 3.4e-05 |
| Meox1\_2310 |  | ceh-31 | 35 | 2.20 | 3.5e-05 |
| MA0225.1 |  | unc-86 (0.63) lim-7 php-3 cfi-1 lin-39 | 40 | 1.94 | 3.9e-05 |
| V$XFD2\_01 |  | lin-31 let-381 | 33 | 2.30 | 4.1e-05 |
| pTH10013 |  | nhr-168 | 39 | 1.98 | 4.1e-05 |
| Nkx1-1\_3856 |  | ceh-30 | 35 | 2.18 | 4.3e-05 |
| pTH9080 |  | mnm-2 (0.69) | 38 | 2.03 | 4.4e-05 |
| Pax7\_3783 |  | alr-1 (0.72) lim-7 ceh-18 | 35 | 2.18 | 4.5e-05 |
| Sp4\_1011 |  | klf-1 sptf-3 klf-2 | 38 | 2.01 | 5.2e-05 |
| pTH5656 |  | daf-16 fkh-7 fkh-8 lin-31 fkh-10 let-381 | 33 | 2.27 | 5.5e-05 |
| MA0537.1 |  | blmp-1 | 37 | 2.05 | 5.6e-05 |
| pTH6569 |  | ceh-43 | 36 | 2.10 | 5.7e-05 |
| pTH10823 |  | B0310.2 | 38 | 2.00 | 5.8e-05 |
| PAX5\_1 |  | pax-3 pax-2 | 31 | 2.39 | 6.0e-05 |
| Meis3\_2 |  | ceh-32 lin-39 | 24 | 3.01 | 6.0e-05 |
| Irx6\_2623 |  | irx-1 | 35 | 2.14 | 6.3e-05 |
| pTH5462 |  | fos-1 jun-1 crh-1 sknr-1 | 30 | 2.44 | 7.0e-05 |
| CG9895\_SOLEXA\_5\_FBgn0034810 |  | klf-1 klf-2 | 26 | 2.76 | 7.1e-05 |
| PAX8\_f1 |  | pax-2 | 23 | 3.08 | 7.8e-05 |
| MA0174.1 |  | ceh-24 (0.55) php-3 lin-39 pal-1 | 31 | 2.35 | 7.9e-05 |
| V$HMX1\_01 |  | daf-12 hif-1 ceh-9 Y5F2A.4 ztf-3 | 29 | 2.49 | 8.1e-05 |
| Hlx1\_2350 |  | ceh-24 (0.55) lim-6 lim-7 ceh-16 | 33 | 2.23 | 8.3e-05 |
| pTH10722 |  | ref-2 eor-1 egrh-3 | 28 | 2.56 | 8.7e-05 |
| Hoxc11\_3718 |  | ceh-24 (0.55) pal-1 | 31 | 2.33 | 1.0e-04 |
| Zbtb12\_2932 |  | lsy-27 | 36 | 2.05 | 1.0e-04 |
| pTH9237 |  | mel-28 | 37 | 2.00 | 1.1e-04 |
| CG31670\_SOLEXA\_5\_FBgn0031375 |  | F21A9.2 CELE\_Y38H8A.5 | 43 | 1.76 | 1.1e-04 |
| V$OCT1\_03 |  | ceh-18 | 38 | 1.96 | 1.1e-04 |
| POU4F2\_2 |  | unc-86 (0.63) ceh-18 | 41 | 1.83 | 1.1e-04 |
| pTH9879 |  | mxl-1 aha-1 lin-22 ref-1 C27D6.4 | 28 | 2.52 | 1.1e-04 |
| pTH4337 |  | crh-1 attf-1 W08E12.1 | 36 | 2.04 | 1.2e-04 |
| Hoxa11\_2218 |  | php-3 | 45 | 1.67 | 1.3e-04 |
| RORG\_f1 |  | nhr-213 nhr-118 | 39 | 1.90 | 1.3e-04 |
| Evx1\_3952 |  | ceh-53 | 31 | 2.29 | 1.4e-04 |
| Hoxa7\_3750 |  | lin-39 | 26 | 2.64 | 1.5e-04 |
| Mafk\_3106 |  | F45H11.6 | 33 | 2.16 | 1.5e-04 |
| EN1\_2 |  | ceh-2 (0.54) ceh-16 | 28 | 2.48 | 1.5e-04 |
| NR2E3\_f1 |  | nhr-100 lin-39 lin-1 | 36 | 2.01 | 1.6e-04 |
| Atf6\_SANGER\_5\_FBgn0033010 |  | atf-6 (0.52) fos-1 crh-1 atf-7 C27D6.4 | 36 | 2.01 | 1.6e-04 |
| Fkh2 |  | lin-31 fkh-10 let-381 C34D1.1 | 48 | 1.56 | 1.6e-04 |
| Tcf3\_3787 |  | pop-1 | 29 | 2.40 | 1.6e-04 |
| TBX20\_4 |  | mab-9 tbx-39 tbx-38 tbx-42 tbx-43 | 30 | 2.33 | 1.7e-04 |
| pTH9165 |  | ztf-27 | 37 | 1.95 | 1.9e-04 |
| Hoxa4\_3426 |  | lin-39 | 33 | 2.14 | 1.9e-04 |
| V$TAXCREB\_02 |  | zip-3 crh-1 | 35 | 2.04 | 2.0e-04 |
| pTH9957 |  | daf-16 irx-1 fkh-9 | 36 | 1.99 | 2.0e-04 |
| HXA10\_f1 |  | hbl-1 php-3 lin-39 | 39 | 1.86 | 2.2e-04 |
| pTH9054 |  | nhr-255 npax-1 lin-14 | 26 | 2.59 | 2.2e-04 |
| Elk4 |  | F19F10.1 lin-1 C24A1.2 | 30 | 2.30 | 2.2e-04 |
| MA0543.1 |  | daf-8 eor-1 | 37 | 1.94 | 2.3e-04 |
| PDX1\_do |  | alr-1 (0.72) lin-39 ceh-12 pal-1 ceh-43 | 32 | 2.17 | 2.4e-04 |
| pTH9180 |  | mef-2 let-381 mel-28 Y116A8C.22 Y61A9LA.9 | 39 | 1.85 | 2.4e-04 |
| Mv102 |  | nhr-71 nhr-213 nhr-2 nhr-6 nhr-10 nhr-68 Y67D8A.3 | 31 | 2.22 | 2.5e-04 |
| Sox4 |  | sox-4 nhr-100 pop-1 | 37 | 1.93 | 2.6e-04 |
| MA0015.1 |  | che-1 K11D2.4 | 27 | 2.48 | 2.7e-04 |
| CUX1\_2 |  | ceh-48 | 38 | 1.88 | 2.7e-04 |
| Six6\_2267 |  | egl-27 ceh-32 elt-1 elt-3 ceh-34 elt-7 | 26 | 2.55 | 2.7e-04 |
| Hoxd13\_2356 |  | pal-1 | 31 | 2.21 | 2.9e-04 |
| V$EN1\_01 |  | atf-2 ceh-16 | 35 | 2.00 | 3.0e-04 |
| pTH9216 |  | ceh-18 | 36 | 1.96 | 3.0e-04 |
| MA0222.1 |  | ceh-32 ceh-20 ces-1 F55C5.11 | 34 | 2.05 | 3.0e-04 |
| Mw160 |  | nhr-68 | 40 | 1.80 | 3.0e-04 |
| MA0503.1 |  | ceh-24 (0.55) pzf-1 | 27 | 2.45 | 3.2e-04 |
| V$CDP\_01 |  | ceh-48 dsc-1 | 37 | 1.91 | 3.2e-04 |
| I$UBX\_01 |  | lin-39 | 11 | 6.15 | 3.2e-04 |
| pTH6327 |  | dsc-1 | 33 | 2.08 | 3.5e-04 |
| pTH9907 |  | nhr-34 | 36 | 1.94 | 3.5e-04 |
| pTH10822 |  | hlh-10 unc-120 | 33 | 2.08 | 3.5e-04 |
| Dlx1\_1741 |  | ceh-43 | 33 | 2.08 | 3.5e-04 |
| V$XFD3\_01 |  | cfi-1 ceh-20 let-381 | 35 | 1.99 | 3.5e-04 |
| pTH2936 |  | nhr-239 | 33 | 2.08 | 3.6e-04 |
| Hoxa3\_2783 |  | lin-39 | 35 | 1.98 | 3.6e-04 |
| Hlxb9\_3422 |  | ceh-12 | 31 | 2.18 | 3.6e-04 |
| Hoxb6\_3428 |  | ceh-18 lin-39 ceh-6 | 30 | 2.24 | 3.6e-04 |
| Hoxc4\_3491 |  | lin-39 | 37 | 1.90 | 3.7e-04 |
| PLAG1\_si |  | klf-2 Y53H1A.2 plp-2 | 33 | 2.07 | 3.8e-04 |
| Hoxa9\_2622 |  | php-3 lin-39 | 46 | 1.58 | 3.8e-04 |
| pTH5914 |  | attf-1 | 20 | 3.13 | 3.9e-04 |
| Hoxb4\_2627 |  | lin-39 | 37 | 1.89 | 3.9e-04 |
| pTH5887 |  | lin-39 | 30 | 2.22 | 4.1e-04 |
| V$CEBP\_01 |  | C48E7.11 | 47 | 1.55 | 4.1e-04 |
| Hmbox1\_2674 |  | hmbx-1 | 51 | 1.42 | 4.1e-04 |
| Elf4 |  | C24A1.2 | 31 | 2.16 | 4.2e-04 |
| V$AREB6\_01 |  | ztf-6 tbx-39 | 21 | 2.98 | 4.2e-04 |
| PROX1\_1 |  | crh-1 ceh-26 | 51 | 1.42 | 4.3e-04 |
| pTH9097 |  | Y116A8C.22 | 23 | 2.74 | 4.4e-04 |
| I$MTTFA\_01 |  | hmg-5 nsy-7 | 37 | 1.88 | 4.5e-04 |
| Elf3 |  | C24A1.2 | 32 | 2.10 | 4.6e-04 |
| pTH6447 |  | ceh-19 | 55 | 1.27 | 4.6e-04 |
| VENTX\_1 |  | alr-1 (0.72) dve-1 ceh-45 ceh-53 pha-2 ceh-36 | 33 | 2.05 | 4.9e-04 |
| Hoxc8\_3429 |  | lin-39 | 24 | 2.63 | 5.0e-04 |
| Sox17\_2837 |  | sox-4 | 34 | 2.00 | 5.0e-04 |
| MA0486.1 |  | F10B5.3 hsf-1 Y53C10A.3 | 40 | 1.76 | 5.1e-04 |
| V$TATA\_01 |  | tbp-1 | 38 | 1.83 | 5.2e-04 |
| Hoxa5\_3415 |  | lin-39 | 35 | 1.95 | 5.2e-04 |
| pTH6556 |  | lim-6 odd-1 | 28 | 2.31 | 5.2e-04 |
| HXD13\_f1 |  | mex-6 pal-1 | 29 | 2.25 | 5.3e-04 |
| FLI1\_f1 |  | lin-1 | 36 | 1.91 | 5.4e-04 |
| pTH5812 |  | ceh-14 | 33 | 2.03 | 5.5e-04 |
| pTH10718 |  | egl-43 | 36 | 1.90 | 5.7e-04 |
| pTH5164 |  | hlh-32 irx-1 D1081.8 | 10 | 6.47 | 5.8e-04 |
| pTH8983 |  | tag-347 | 21 | 2.91 | 5.9e-04 |
| pTH9254 |  | mel-28 | 36 | 1.90 | 6.0e-04 |
| V$VMYB\_01 |  | lin-48 D1081.8 B0310.2 | 39 | 1.78 | 6.1e-04 |
| V$AREB6\_02 |  | ztf-6 | 25 | 2.51 | 6.1e-04 |
| pTH6425 |  | ceh-20 pop-1 | 45 | 1.59 | 6.2e-04 |
| pTH2283 |  | odd-2 | 45 | 1.59 | 6.3e-04 |
| En1\_3123 |  | ceh-53 ceh-16 | 32 | 2.06 | 6.6e-04 |
| MA0246.1 |  | ceh-32 dmd-4 dmd-5 | 28 | 2.28 | 6.9e-04 |
| Osr1\_3033 |  | odd-1 | 28 | 2.27 | 7.1e-04 |
| pTH9163 |  | nhr-3 | 37 | 1.84 | 7.3e-04 |
| Hoxa2\_3079 |  | lin-39 | 35 | 1.92 | 7.4e-04 |
| GMEB2\_1 |  | ces-2 C01B12.2 F23F12.9 Y51H4A.4 | 25 | 2.48 | 7.4e-04 |
| Mv75 |  | elt-1 lin-31 | 30 | 2.15 | 7.6e-04 |
| MA0163.1 |  | C09F5.3 D1081.8 Y53H1A.2 | 47 | 1.52 | 7.8e-04 |
| MA0467.1 |  | ceh-45 tbx-39 | 32 | 2.04 | 7.9e-04 |
| Mv129 |  | sox-4 ceh-18 ceh-6 tbp-1 | 27 | 2.32 | 7.9e-04 |
| pTH10041 |  | ztf-29 | 37 | 1.83 | 8.0e-04 |
| Dlx3\_1030 |  | ceh-43 | 23 | 2.63 | 8.1e-04 |
| ETS2\_f1 |  | lin-1 C24A1.2 | 18 | 3.25 | 8.1e-04 |
| Pou3f4\_3773 |  | ceh-6 | 39 | 1.76 | 8.1e-04 |
| pTH6445 |  | ceh-5 | 40 | 1.73 | 8.2e-04 |
| Barx1\_2877 |  | ceh-43 | 34 | 1.95 | 8.3e-04 |
| pTH5080 |  | fos-1 crh-1 atf-5 | 22 | 2.72 | 8.5e-04 |
| MA0540.1 |  | dpy-27 | 28 | 2.25 | 8.7e-04 |
| pTH5267 |  | hlh-32 hlh-16 ngn-1 | 28 | 2.24 | 8.9e-04 |
| NFIA\_1 |  | nfi-1 | 27 | 2.30 | 9.1e-04 |
| Etv3 |  | lin-1 | 39 | 1.75 | 9.1e-04 |
| HXD10\_f1 |  | nhr-2 php-3 | 32 | 2.03 | 9.2e-04 |
| Hoxb5\_3122 |  | lin-39 | 30 | 2.13 | 9.3e-04 |
| Hoxd1\_3448 |  | ceh-12 | 19 | 3.04 | 1.0e-03 |
| pTH5778 |  | egl-5 | 37 | 1.81 | 1.0e-03 |
| Barhl1\_1 |  | ceh-31 | 53 | 1.33 | 1.0e-03 |
| Vax2\_3500 |  | C02F12.10 | 20 | 2.90 | 1.1e-03 |
| pTH9164 |  | ceh-26 | 38 | 1.77 | 1.1e-03 |
| K562\_SP2\_HudsonAlpha |  | klf-2 | 48 | 1.47 | 1.1e-03 |
| pTH10714 |  | nhr-216 (0.57) nhr-84 nhr-142 | 35 | 1.88 | 1.1e-03 |
| RFX3\_2 |  | daf-19 | 35 | 1.88 | 1.1e-03 |
| Mw154 |  | lin-39 ceh-12 ceh-20 | 18 | 3.15 | 1.1e-03 |
| Vax1\_3499 |  | C02F12.10 | 21 | 2.76 | 1.2e-03 |
| Nkx6-3\_3446 |  | cog-1 | 37 | 1.80 | 1.2e-03 |
| Gata5\_3768 |  | elt-1 | 34 | 1.92 | 1.2e-03 |
| NR2F1\_4 |  | nhr-62 nhr-2 nhr-19 | 31 | 2.04 | 1.2e-03 |
| MITF\_f1 |  | mxl-1 hlh-30 irx-1 | 29 | 2.15 | 1.2e-03 |
| Tcf7\_0950 |  | pop-1 | 31 | 2.04 | 1.2e-03 |
| Hmx1\_3423 |  | ceh-9 | 27 | 2.26 | 1.2e-03 |
| Nkx6-1\_2825 |  | cog-1 | 25 | 2.39 | 1.2e-03 |
| pTH6508 |  | nhr-36 | 29 | 2.14 | 1.3e-03 |
| pTH10816 |  | dmd-6 | 32 | 1.99 | 1.3e-03 |
| pTH9262 |  | lin-54 | 22 | 2.64 | 1.3e-03 |
| pTH1014 |  | atf-5 | 31 | 2.04 | 1.3e-03 |
| V$NCX\_01 |  | ceh-19 | 33 | 1.94 | 1.4e-03 |
| pTH3751 |  | tbx-39 | 27 | 2.25 | 1.4e-03 |
| pTH10768 |  | med-2 (0.51) | 29 | 2.13 | 1.4e-03 |
| pTH3064 |  | crh-1 | 35 | 1.86 | 1.4e-03 |
| V$CETS1P54\_02 |  | C52B9.2 | 18 | 3.09 | 1.4e-03 |
| Hoxb3\_1720 |  | lin-39 | 33 | 1.94 | 1.4e-03 |
| ovo\_FlyReg\_FBgn0003028 |  | lin-48 dmd-3 pax-3 | 32 | 1.98 | 1.5e-03 |
| pTH9924 |  | nhr-46 (0.51) | 39 | 1.72 | 1.5e-03 |
| V$HOX13\_01 |  | lin-39 | 36 | 1.82 | 1.5e-03 |
| MA0035.3 |  | ztf-29 elt-1 | 37 | 1.78 | 1.5e-03 |
| V$AREB6\_04 |  | ztf-6 gei-11 C34D1.1 | 28 | 2.17 | 1.6e-03 |
| Hoxa6\_1040 |  | lin-39 | 36 | 1.81 | 1.6e-03 |
| pTH9326 |  | nhr-122 | 38 | 1.74 | 1.6e-03 |
| K562\_ZBTB7A\_HudsonAlpha |  | ZC328.2 | 20 | 2.79 | 1.6e-03 |
| Gsh2\_3990 |  | ceh-31 | 10 | 5.59 | 1.7e-03 |
| I$DRI\_01 |  | cfi-1 | 16 | 3.38 | 1.7e-03 |
| PAX5\_si |  | pax-2 D1081.8 | 8 | 7.53 | 1.7e-03 |
| Lhx1\_2240 |  | lim-7 | 25 | 2.34 | 1.7e-03 |
| pTH6641 |  | lin-31 | 28 | 2.15 | 1.7e-03 |
| LHX6\_3 |  | lim-6 cfi-1 gei-3 nhr-208 | 29 | 2.10 | 1.8e-03 |
| pTH7875 |  | mel-28 | 40 | 1.67 | 1.8e-03 |
| pTH5561 |  | nhr-239 | 36 | 1.80 | 1.8e-03 |
| pTH9049 |  | ztf-2 ztf-6 | 39 | 1.70 | 1.8e-03 |
| MA0027.1 |  | ceh-16 | 35 | 1.84 | 1.8e-03 |
| MCR\_f1 |  | nhr-255 | 37 | 1.77 | 1.8e-03 |
| pTH7876 |  | lin-29 fkh-7 mel-28 | 32 | 1.95 | 1.9e-03 |
| Pou2f3\_3986 |  | ceh-18 | 33 | 1.91 | 1.9e-03 |
| pTH10721 |  | ceh-18 ztf-9 | 32 | 1.95 | 1.9e-03 |
| MA0131.1 |  | F39B2.1 ZC416.1 | 31 | 1.99 | 1.9e-03 |
| V$AHR\_01 |  | aha-1 ahr-1 | 17 | 3.16 | 1.9e-03 |
| CG7386\_F10-12\_SANGER\_5\_FBgn0035691 |  | sox-4 F56D1.1 gei-3 | 35 | 1.83 | 1.9e-03 |
| MA0085.1 |  | lag-1 (0.8) ztf-3 F26F4.8 | 27 | 2.19 | 2.0e-03 |
| Bsx\_3483 |  | ceh-31 | 29 | 2.08 | 2.0e-03 |
| pTH9219 |  | xbp-1 C01B12.2 | 25 | 2.31 | 2.2e-03 |
| Dlx2\_2273 |  | ceh-43 | 31 | 1.98 | 2.2e-03 |
| pTH10623 |  | scrt-1 | 11 | 4.80 | 2.2e-03 |
| SOX2\_1 |  | sox-4 dmd-4 | 41 | 1.63 | 2.2e-03 |
| V$S8\_01 |  | ceh-45 | 33 | 1.89 | 2.3e-03 |
| pTH8985 |  | athp-1 | 23 | 2.43 | 2.4e-03 |
| pTH10034 |  | nhr-66 | 21 | 2.60 | 2.5e-03 |
| TCF4\_2 |  | ztf-6 hlh-2 | 10 | 5.28 | 2.5e-03 |
| Jundm2\_0911 |  | fos-1 | 28 | 2.11 | 2.5e-03 |
| V$FREAC7\_01 |  | lin-31 | 34 | 1.84 | 2.5e-03 |
| Pbx1\_3203 |  | ceh-20 | 25 | 2.28 | 2.5e-03 |
| V$GATA1\_04 |  | elt-1 | 37 | 1.74 | 2.6e-03 |
| pTH8411 |  | tbx-39 | 33 | 1.88 | 2.6e-03 |
| V$RFX1\_02 |  | daf-19 F52B5.7 | 52 | 1.32 | 2.6e-03 |
| pTH6436 |  | ceh-53 | 32 | 1.92 | 2.6e-03 |
| pTH10769 |  | Y48G1C.6 | 33 | 1.88 | 2.6e-03 |
| Mw140 |  | efl-1 F49E12.6 | 41 | 1.62 | 2.6e-03 |
| pTH9244 |  | tbx-39 | 34 | 1.84 | 2.6e-03 |
| Lbx2\_3869 |  | mls-2 | 27 | 2.15 | 2.7e-03 |
| V$BRN2\_01 |  | ceh-18 | 27 | 2.15 | 2.7e-03 |
| Spdef\_0905 |  | lin-1 | 32 | 1.91 | 2.7e-03 |
| Oli\_da\_SANGER\_5\_3\_FBgn0032651 |  | hlh-32 hlh-12 hlh-8 hlh-15 ngn-1 | 27 | 2.15 | 2.8e-03 |
| V$HEN1\_02 |  | hlh-15 hlh-1 | 13 | 3.91 | 2.8e-03 |
| pTH9245 |  | ceh-18 | 39 | 1.67 | 2.8e-03 |
| pTH3084 |  | C01B12.2 attf-1 | 25 | 2.27 | 2.8e-03 |
| T-47D\_GATA3\_HudsonAlpha |  | elt-1 | 26 | 2.20 | 2.8e-03 |
| ELK3\_f1 |  | lin-1 | 35 | 1.80 | 2.9e-03 |
| pTH8598 |  | odr-7 (0.74) nhr-28 nhr-273 tbx-39 slr-2 lin-1 | 33 | 1.87 | 2.9e-03 |
| V$FAC1\_01 |  | gei-8 | 33 | 1.86 | 3.0e-03 |
| MA0102.3 |  | C48E7.11 | 31 | 1.94 | 3.1e-03 |
| pTH6497 |  | lin-31 | 40 | 1.63 | 3.2e-03 |
| Srf\_3509 |  | unc-120 | 28 | 2.07 | 3.2e-03 |
| FOXB1\_1 |  | lin-31 F55C5.11 | 22 | 2.46 | 3.3e-03 |
| Hr46\_FlyReg\_FBgn0000448 |  | nhr-213 lin-31 | 36 | 1.75 | 3.3e-03 |
| pTH9709 |  | die-1 | 40 | 1.63 | 3.3e-03 |
| HES1\_f1 |  | lin-22 | 33 | 1.85 | 3.4e-03 |
| Zfp652\_1 |  | B0310.2 ZK177.3 | 34 | 1.81 | 3.4e-03 |
| KLF8\_f1 |  | klf-1 | 31 | 1.93 | 3.4e-03 |
| PRRX1\_f1 |  | alr-1 (0.72) elt-1 | 49 | 1.39 | 3.5e-03 |
| Pou3f1\_3819 |  | ceh-6 | 36 | 1.74 | 3.6e-03 |
| pTH5437 |  | ceh-34 | 21 | 2.52 | 3.6e-03 |
| pTH6478 |  | lim-7 | 23 | 2.36 | 3.7e-03 |
| pTH1284 |  | nhr-213 nhr-2 nhr-10 nhr-19 nhr-69 | 24 | 2.29 | 3.7e-03 |
| V$YY1\_01 |  | lsy-2 | 27 | 2.10 | 3.8e-03 |
| pTH9220 |  | mbr-1 | 25 | 2.22 | 3.8e-03 |
| V$T3R\_01 |  | nhr-239 nhr-15 nhr-213 nhr-2 | 32 | 1.88 | 3.8e-03 |
| Hoxb8\_3780 |  | lin-39 | 38 | 1.68 | 3.8e-03 |
| HepG2\_HSF1\_Stanford |  | Y53C10A.3 | 32 | 1.87 | 3.9e-03 |
| V$TCF11\_01 |  | skn-1 | 27 | 2.10 | 3.9e-03 |
| Barx2\_3447 |  | ceh-43 | 24 | 2.27 | 4.0e-03 |
| Pou3f3\_3235 |  | ceh-6 | 31 | 1.91 | 4.1e-03 |
| pTH2846 |  | lin-31 | 40 | 1.61 | 4.1e-03 |
| pTH9082 |  | mab-23 | 32 | 1.86 | 4.3e-03 |
| Sox11\_2266 |  | sox-4 gei-3 pop-1 C05C9.3 | 33 | 1.83 | 4.4e-03 |
| pTH5059 |  | lin-22 hlh-28 | 19 | 2.67 | 4.4e-03 |
| PTF1A\_f1 |  | lin-32 hlh-2 | 22 | 2.40 | 4.5e-03 |
| pTH10779 |  | nhr-134 nhr-182 | 34 | 1.79 | 4.5e-03 |
| pTH5078 |  | ces-2 | 50 | 1.36 | 4.6e-03 |
| pTH10630 |  | lsy-27 | 31 | 1.90 | 4.6e-03 |
| Emx2\_3420 |  | ceh-2 (0.54) | 14 | 3.43 | 4.7e-03 |
| Tbp\_pr781 |  | tbp-1 | 25 | 2.18 | 4.7e-03 |
| pTH9250 |  | C34D1.1 | 19 | 2.66 | 4.7e-03 |
| pTH8556 |  | pax-2 | 45 | 1.48 | 4.7e-03 |
| Cdx2\_4272 |  | ceh-13 | 38 | 1.66 | 4.8e-03 |
| Pou2f1\_3081 |  | ceh-18 | 31 | 1.89 | 4.8e-03 |
| Six4\_2860 |  | ceh-32 | 30 | 1.93 | 4.8e-03 |
| pTH6636 |  | egl-5 | 30 | 1.93 | 4.8e-03 |
| ems\_FlyReg\_FBgn0000576 |  | ceh-2 (0.54) skn-1 | 34 | 1.78 | 4.9e-03 |
| V$GATA3\_01 |  | elt-1 | 50 | 1.35 | 4.9e-03 |
| pTH8996 |  | sma-4 | 36 | 1.71 | 5.0e-03 |
| pTH9096 |  | T07C12.11 | 27 | 2.06 | 5.2e-03 |
| Mcm1 |  | unc-120 | 34 | 1.77 | 5.2e-03 |
| pTH8649 |  | mbr-1 | 30 | 1.92 | 5.3e-03 |
| pTH9247 |  | C34D1.1 | 32 | 1.84 | 5.3e-03 |
| ARNT2\_si |  | aha-1 C46E10.9 | 29 | 1.96 | 5.4e-03 |
| pTH9900 |  | C46E10.8 C33G8.2 | 38 | 1.65 | 5.4e-03 |
| pTH9189 |  | dmd-3 ceh-18 | 40 | 1.59 | 5.7e-03 |
| V$LYF1\_01 |  | bed-3 nhr-177 F26F4.8 | 36 | 1.70 | 5.8e-03 |
| pTH10810 |  | lsy-2 lsl-1 | 19 | 2.61 | 5.8e-03 |
| Irx3\_1 |  | irx-1 | 36 | 1.70 | 5.9e-03 |
| pTH5877 |  | nhr-7 nhr-100 elt-1 nhr-10 | 40 | 1.59 | 5.9e-03 |
| pTH9335 |  | mel-28 | 43 | 1.51 | 6.0e-03 |
| pTH9026 |  | attf-1 | 15 | 3.13 | 6.1e-03 |
| CG8765\_SANGER\_5\_FBgn0036900 |  | H20J04.3 | 36 | 1.69 | 6.2e-03 |
| V$YY1\_02 |  | lsy-2 | 34 | 1.75 | 6.4e-03 |
| Hoxa7\_2668 |  | lin-39 | 17 | 2.81 | 6.5e-03 |
| MA0066.1 |  | nhr-43 | 30 | 1.89 | 6.6e-03 |
| TLX1\_f1 |  | ceh-19 | 8 | 5.92 | 6.7e-03 |
| pTH9135 |  | pop-1 | 43 | 1.50 | 6.7e-03 |
| pTH2684 |  | fos-1 | 27 | 2.02 | 6.8e-03 |
| Hoxa10\_2318 |  | ceh-24 (0.55) | 37 | 1.66 | 7.0e-03 |
| pTH9380 |  | mel-28 | 37 | 1.65 | 7.0e-03 |
| MA0456.1 |  | ref-2 | 44 | 1.48 | 7.1e-03 |
| pTH6449 |  | ceh-43 | 28 | 1.97 | 7.1e-03 |
| pTH6268 |  | ceh-2 (0.54) | 27 | 2.01 | 7.3e-03 |
| pTH9108 |  | daf-12 nhr-5 | 32 | 1.81 | 7.4e-03 |
| pTH9925 |  | ztf-11 nhr-100 | 39 | 1.59 | 7.6e-03 |
| GATA5\_1 |  | elt-1 | 36 | 1.67 | 8.2e-03 |
| pTH6591 |  | lin-31 | 27 | 1.99 | 8.3e-03 |
| Zfp161\_2858 |  | pzf-1 | 43 | 1.49 | 8.4e-03 |
| pTH8216 |  | Y116A8C.22 | 51 | 1.30 | 8.8e-03 |
| pTH8998 |  | mab-3 | 37 | 1.63 | 8.9e-03 |
| pTH9149 |  | ztf-30 | 24 | 2.13 | 9.3e-03 |
| pTH6423 |  | pha-2 | 31 | 1.82 | 9.4e-03 |
| pTH8982 |  | ceh-48 | 36 | 1.66 | 9.5e-03 |
| CG4854\_SANGER\_10\_FBgn0038766 |  | mxl-1 K11D2.4 | 35 | 1.68 | 9.7e-03 |
| Gata3\_1024 |  | elt-1 | 31 | 1.81 | 9.9e-03 |
| Cdx1\_2245 |  | ceh-13 | 25 | 2.07 | 9.9e-03 |
| Tcf1\_2666 |  | hmbx-1 | 48 | 1.37 | 1.0e-02 |
| MAFA\_f1 |  | daf-8 F45H11.6 | 31 | 1.81 | 1.0e-02 |
| pTH8318 |  | attf-1 | 26 | 2.01 | 1.0e-02 |
| pTH10805 |  | ztf-16 | 32 | 1.77 | 1.0e-02 |
| pTH6482 |  | ceh-19 | 31 | 1.80 | 1.0e-02 |
| pTH8745 |  | attf-1 | 23 | 2.17 | 1.0e-02 |
| ZBTB7A\_1 |  | ZC328.2 ztf-14 | 28 | 1.92 | 1.1e-02 |
| pTH9381 |  | ceh-18 | 18 | 2.56 | 1.1e-02 |
| pTH9969 |  | pag-3 | 35 | 1.67 | 1.1e-02 |
| pTH5690 |  | ceh-32 | 29 | 1.87 | 1.1e-02 |
| GRHL1\_2 |  | grh-1 | 28 | 1.91 | 1.1e-02 |
| Aef1\_FlyReg\_FBgn0005694 |  | egl-13 (0.66) sox-4 K11D2.4 | 38 | 1.59 | 1.1e-02 |
| pTH10788 |  | tbx-33 | 43 | 1.47 | 1.1e-02 |
| Smad3\_3805 |  | daf-8 | 32 | 1.76 | 1.2e-02 |
| Sox15\_3457 |  | sox-4 | 27 | 1.94 | 1.2e-02 |
| Atf1\_3026 |  | crh-1 | 50 | 1.31 | 1.2e-02 |
| N$SKN1\_02 |  | ceh-32 skn-1 | 36 | 1.63 | 1.3e-02 |
| Tcf2\_0913 |  | hmbx-1 | 23 | 2.14 | 1.3e-02 |
| pTH1049 |  | elt-1 | 34 | 1.69 | 1.3e-02 |
| pTH4325 |  | ceh-18 | 26 | 1.97 | 1.3e-02 |
| pTH9242 |  | mel-28 | 36 | 1.63 | 1.3e-02 |
| Hnf4a\_2640 |  | nhr-62 | 29 | 1.85 | 1.3e-02 |
| Cutl1\_3494 |  | ceh-44 | 29 | 1.84 | 1.4e-02 |
| pTH3998 |  | tbx-39 | 34 | 1.68 | 1.4e-02 |
| Six3\_1732 |  | ceh-34 | 27 | 1.92 | 1.4e-02 |
| MA0032.1 |  | let-381 | 24 | 2.06 | 1.5e-02 |
| pTH10777 |  | dmd-3 | 24 | 2.05 | 1.5e-02 |
| ZN384\_f1 |  | lin-29 K11D2.4 | 36 | 1.61 | 1.5e-02 |
| pTH9297 |  | ceh-18 | 38 | 1.56 | 1.5e-02 |
| V$MYB\_Q6 |  | D1081.8 | 17 | 2.56 | 1.6e-02 |
| pTH6486 |  | nhr-145 | 35 | 1.63 | 1.7e-02 |
| pTH9137 |  | nhr-65 (0.51) | 34 | 1.66 | 1.7e-02 |
| pTH10647 |  | nhr-232 | 37 | 1.58 | 1.7e-02 |
| Plagl1\_0972 |  | Y53H1A.2 | 11 | 3.58 | 1.7e-02 |
| pTH10028 |  | nhr-204 | 24 | 2.03 | 1.7e-02 |
| pTH9260 |  | mel-28 | 17 | 2.52 | 1.8e-02 |
| pTH9182 |  | tbx-39 | 31 | 1.74 | 1.9e-02 |
| pTH5714 |  | nhr-239 | 33 | 1.67 | 1.9e-02 |
| pTH9215 |  | C34D1.1 | 25 | 1.96 | 2.0e-02 |
| pTH1001 |  | dnj-17 | 27 | 1.87 | 2.0e-02 |
| srp\_FlyReg\_FBgn0003507 |  | elt-1 | 18 | 2.38 | 2.2e-02 |
| pTH3819 |  | ceh-18 | 21 | 2.15 | 2.2e-02 |
| pTH9353 |  | ceh-51 | 13 | 3.00 | 2.3e-02 |
| Gmeb1\_1745 |  | attf-1 | 23 | 2.03 | 2.3e-02 |
| MA0139.1 |  | F58G1.2 | 30 | 1.74 | 2.4e-02 |
| pTH5781 |  | ceh-32 | 25 | 1.93 | 2.4e-02 |
| MA0118.1 |  | ref-2 | 24 | 1.97 | 2.4e-02 |
| pTH10027 |  | M03D4.4 | 46 | 1.36 | 2.5e-02 |
| pTH9198 |  | dmd-3 | 25 | 1.92 | 2.5e-02 |
| pTH9708 |  | ceh-34 | 27 | 1.83 | 2.6e-02 |
| pTH9173 |  | efl-2 | 17 | 2.42 | 2.6e-02 |
| MA0262.1 |  | mab-3 | 26 | 1.87 | 2.7e-02 |
| Irx5\_2385 |  | irx-1 | 13 | 2.92 | 2.8e-02 |
| rn\_SOLEXA\_5\_FBgn0259172 |  | lin-29 | 42 | 1.43 | 2.9e-02 |
| pTH5118 |  | cfi-1 | 27 | 1.82 | 2.9e-02 |
| pTH9076 |  | C01G12.1 | 37 | 1.53 | 2.9e-02 |
| pTH9043 |  | sem-2 | 41 | 1.45 | 3.0e-02 |
| pTH9256 |  | ceh-18 | 37 | 1.53 | 3.0e-02 |
| Tcfap2a\_2337 |  | aptf-1 (0.6) F58G1.2 | 11 | 3.29 | 3.0e-02 |
| Spdef |  | lin-1 | 49 | 1.29 | 3.1e-02 |
| Irx3\_0920 |  | irx-1 | 48 | 1.31 | 3.3e-02 |
| pTH5924 |  | nhr-255 | 29 | 1.73 | 3.3e-02 |
| pTH5257 |  | C48E7.11 | 37 | 1.52 | 3.3e-02 |
| Mf28 |  | elt-1 | 30 | 1.70 | 3.4e-02 |
| pTH5119 |  | cfi-1 | 30 | 1.69 | 3.7e-02 |
| Poxm\_SOLEXA\_5\_FBgn0003129 |  | pax-2 | 18 | 2.25 | 3.7e-02 |
| HIF1A\_si |  | hif-1 | 13 | 2.77 | 4.0e-02 |
| Irx2\_0900 |  | irx-1 | 29 | 1.70 | 4.2e-02 |
| pTH9384 |  | cfi-1 | 27 | 1.74 | 4.9e-02 |
| HeLa-S3\_ZNF274\_UCD |  | C28G1.4 | 32 | 1.60 | 4.9e-02 |
| pTH10798 |  | Y75B8A.6 | 29 | 1.68 | 4.9e-02 |

### Correlated (and anti-correlated) transcription factors

|  |  |
| --- | --- |
| **Transcription factor** | **Correlation** |
| ZK337.2 | 0.86 |
| hlh-19 | 0.85 |
| lag-1 | 0.80 |
| nhr-67 | 0.78 |
| odr-7 | 0.74 |
| alr-1 | 0.72 |
| mnm-2 | 0.69 |
| fkh-5 | 0.68 |
| ceh-27 | 0.67 |
| egl-13 | 0.66 |
| nhr-175 | 0.64 |
| unc-86 | 0.63 |
| aptf-1 | 0.60 |
| ctbp-1 | 0.59 |
| F47E1.3 | 0.58 |
| F13H6.1 | 0.58 |
| sta-1 | 0.58 |
| nhr-1 | 0.58 |
| elt-6 | 0.57 |
| nhr-216 | 0.57 |
| lin-28 | 0.57 |
| ets-5 | 0.57 |
| nhr-12 | 0.57 |
| unc-130 | 0.56 |
| nhr-37 | 0.55 |
| nhr-171 | -0.35 |
| nhr-172 | -0.35 |
| ceh-13 | -0.35 |
| sup-35 | -0.36 |
| ccch-3 | -0.36 |
| tbx-32 | -0.36 |
| pie-1 | -0.37 |
| tbx-34 | -0.37 |
| Y48G9A.11 | -0.38 |
| ceh-81 | -0.39 |
| sma-4 | -0.40 |
| zip-8 | -0.40 |
| mxl-1 | -0.41 |
| spe-44 | -0.41 |
| nhr-108 | -0.43 |
| mab-5 | -0.44 |
| tbx-41 | -0.44 |
| F19F10.1 | -0.46 |
| nhr-248 | -0.47 |
| Y56A3A.18 | -0.47 |
| nhr-271 | -0.52 |
| mxl-2 | -0.52 |
| cey-2 | -0.53 |
| nhr-210 | -0.53 |
| T26A5.8 | -0.72 |

### ChIP peaks enriched

|  |  |  |  |  |
| --- | --- | --- | --- | --- |
| **Gene** | **Experiment** | **Number of upstream peaks** | **Enrichment** | **FDR corrected p** |
| alr-1 | ALR-1\_Larvae-L2-stage | 13 | 2.83 | 0.018 |
