# Supplementary material for: Overlapping cell population expression profiling and regulatory inference in C. elegans
Source: BMC Genomics. 2016 Feb 29;17:159. doi: 10.1186/s12864-016-2482-z (PMC4772325; doi:10.1186/s12864-016-2482-z)
Supplement: Additional file 13: — Web supplement. (DOC 21 kb) [file 12864_2016_2482_MOESM13_ESM.zip › sortWeb/clusters/hier.300.clusters/263.html]

Cluster 263 

## Cluster 263

### Expression

| cnd-1 rep. 1 | cnd-1 rep. 2 | cnd-1 rep. 3 | pha-4 rep. 1 | pha-4 rep. 2 | pha-4 rep. 3 | ceh-27 | ceh-36 | ceh-6 | F21D5.9 | mir-57 | mls-2 | pal-1 | pros-1 | ttx-3 | unc-130 | hlh-16 | irx-1 | ceh-6 (+) hlh-16 (+) | ceh-6 (+) hlh-16 (-) | ceh-6 (-) hlh-16 (+) | cnd-1 singlets | pha-4 singlets | 0 | 60 | 120 | 150 | 180 | 240 | 330 | 390 | 420 | 480 | 540 | 570 | 600 | 630 | 660 | NAME | Functional description |
| --- | --- | --- | --- | --- | --- | --- | --- | --- | --- | --- | --- | --- | --- | --- | --- | --- | --- | --- | --- | --- | --- | --- | --- | --- | --- | --- | --- | --- | --- | --- | --- | --- | --- | --- | --- | --- | --- | --- | --- |
|  |  |  |  |  |  |  |  |  |  |  |  |  |  |  |  |  |  |  |  |  |  |  |  |  |  |  |  |  |  |  |  |  |  |  |  |  |  | *str-112* | Seven TM Receptor |
|  |  |  |  |  |  |  |  |  |  |  |  |  |  |  |  |  |  |  |  |  |  |  |  |  |  |  |  |  |  |  |  |  |  |  |  |  |  | F42G4.6 |  |
|  |  |  |  |  |  |  |  |  |  |  |  |  |  |  |  |  |  |  |  |  |  |  |  |  |  |  |  |  |  |  |  |  |  |  |  |  |  | R13F6.11 |  |
|  |  |  |  |  |  |  |  |  |  |  |  |  |  |  |  |  |  |  |  |  |  |  |  |  |  |  |  |  |  |  |  |  |  |  |  |  |  | C52E4.15 |  |
|  |  |  |  |  |  |  |  |  |  |  |  |  |  |  |  |  |  |  |  |  |  |  |  |  |  |  |  |  |  |  |  |  |  |  |  |  |  | T10G3.4 |  |
|  |  |  |  |  |  |  |  |  |  |  |  |  |  |  |  |  |  |  |  |  |  |  |  |  |  |  |  |  |  |  |  |  |  |  |  |  |  | F02C12.2 |  |
|  |  |  |  |  |  |  |  |  |  |  |  |  |  |  |  |  |  |  |  |  |  |  |  |  |  |  |  |  |  |  |  |  |  |  |  |  |  | F08B6.6 |  |
|  |  |  |  |  |  |  |  |  |  |  |  |  |  |  |  |  |  |  |  |  |  |  |  |  |  |  |  |  |  |  |  |  |  |  |  |  |  | Y69A2AR.36 |  |
|  |  |  |  |  |  |  |  |  |  |  |  |  |  |  |  |  |  |  |  |  |  |  |  |  |  |  |  |  |  |  |  |  |  |  |  |  |  | Y7A9D.64 |  |
|  |  |  |  |  |  |  |  |  |  |  |  |  |  |  |  |  |  |  |  |  |  |  |  |  |  |  |  |  |  |  |  |  |  |  |  |  |  | T02G6.t1 |  |
|  |  |  |  |  |  |  |  |  |  |  |  |  |  |  |  |  |  |  |  |  |  |  |  |  |  |  |  |  |  |  |  |  |  |  |  |  |  | W04E12.12 |  |
|  |  |  |  |  |  |  |  |  |  |  |  |  |  |  |  |  |  |  |  |  |  |  |  |  |  |  |  |  |  |  |  |  |  |  |  |  |  | C45E1.6 |  |
|  |  |  |  |  |  |  |  |  |  |  |  |  |  |  |  |  |  |  |  |  |  |  |  |  |  |  |  |  |  |  |  |  |  |  |  |  |  | Y51H4A.22 |  |

### Phenotypes enriched

none found

### Anatomy terms enriched

none found

### GO terms enriched

none found

### Expression clusters enriched

none found

### Motifs enriched

|  |  |  |  |  |  |
| --- | --- | --- | --- | --- | --- |
| **Motif** | **Logo** | **Possible orthologs** | **Number of motifs in cluster** | **Enrichment** | **FDR corrected p** |
| SMAD3\_1 |  | daf-8 | 12 | 2.24 | 0.017 |
| V$FREAC3\_01 |  | let-381 | 8 | 3.34 | 0.045 |

### Correlated (and anti-correlated) transcription factors

|  |  |
| --- | --- |
| **Transcription factor** | **Correlation** |
| C46E10.9 | 0.57 |
| ZC328.2 | 0.55 |
| nhr-153 | 0.54 |
| nhr-154 | 0.53 |
| tbx-7 | 0.51 |
| let-607 | 0.51 |
| tbx-2 | 0.51 |
| sma-2 | 0.51 |
| nhr-189 | 0.51 |
| nhr-238 | 0.50 |
| nhr-184 | 0.50 |
| fkh-9 | 0.49 |
| nhr-49 | 0.49 |
| dve-1 | 0.49 |
| hlh-25 | 0.49 |
| nhr-164 | 0.48 |
| med-2 | 0.47 |
| ceh-1 | 0.47 |
| F19F10.1 | 0.46 |
| nhr-99 | 0.46 |
| dpr-1 | 0.46 |
| nhr-219 | 0.46 |
| nhr-39 | 0.46 |
| eea-1 | 0.45 |
| nhr-212 | 0.45 |
| nhr-227 | -0.30 |
| hlh-31 | -0.30 |
| nhr-114 | -0.31 |
| nhr-16 | -0.31 |
| Y41D4B.26 | -0.31 |
| hmg-12 | -0.32 |
| nhr-152 | -0.33 |
| nhr-165 | -0.34 |
| dhhc-13 | -0.34 |
| hlh-26 | -0.35 |
| hmg-11 | -0.35 |
| lin-32 | -0.36 |
| mxl-1 | -0.36 |
| nhr-210 | -0.36 |
| T20H4.2 | -0.37 |
| nhr-218 | -0.38 |
| R144.3 | -0.39 |
| nhr-270 | -0.40 |
| tbx-35 | -0.43 |
| elk-2 | -0.44 |
| F10E7.11 | -0.46 |
| tbx-31 | -0.47 |
| Y73F8A.33 | -0.49 |
| ztf-28 | -0.53 |
| spe-44 | -0.56 |

### ChIP peaks enriched

none found
