# Supplementary material for: Overlapping cell population expression profiling and regulatory inference in C. elegans
Source: BMC Genomics. 2016 Feb 29;17:159. doi: 10.1186/s12864-016-2482-z (PMC4772325; doi:10.1186/s12864-016-2482-z)
Supplement: Additional file 13: — Web supplement. (DOC 21 kb) [file 12864_2016_2482_MOESM13_ESM.zip › sortWeb/clusters/hier.300.clusters/268.html]

Cluster 268 

## Cluster 268

### Expression

| cnd-1 rep. 1 | cnd-1 rep. 2 | cnd-1 rep. 3 | pha-4 rep. 1 | pha-4 rep. 2 | pha-4 rep. 3 | ceh-27 | ceh-36 | ceh-6 | F21D5.9 | mir-57 | mls-2 | pal-1 | pros-1 | ttx-3 | unc-130 | hlh-16 | irx-1 | ceh-6 (+) hlh-16 (+) | ceh-6 (+) hlh-16 (-) | ceh-6 (-) hlh-16 (+) | cnd-1 singlets | pha-4 singlets | 0 | 60 | 120 | 150 | 180 | 240 | 330 | 390 | 420 | 480 | 540 | 570 | 600 | 630 | 660 | NAME | Functional description |
| --- | --- | --- | --- | --- | --- | --- | --- | --- | --- | --- | --- | --- | --- | --- | --- | --- | --- | --- | --- | --- | --- | --- | --- | --- | --- | --- | --- | --- | --- | --- | --- | --- | --- | --- | --- | --- | --- | --- | --- |
|  |  |  |  |  |  |  |  |  |  |  |  |  |  |  |  |  |  |  |  |  |  |  |  |  |  |  |  |  |  |  |  |  |  |  |  |  |  | *col-81* | COLlagen |
|  |  |  |  |  |  |  |  |  |  |  |  |  |  |  |  |  |  |  |  |  |  |  |  |  |  |  |  |  |  |  |  |  |  |  |  |  |  | T24F1.8 |  |
|  |  |  |  |  |  |  |  |  |  |  |  |  |  |  |  |  |  |  |  |  |  |  |  |  |  |  |  |  |  |  |  |  |  |  |  |  |  | *srx-44* | Serpentine Receptor, class X |
|  |  |  |  |  |  |  |  |  |  |  |  |  |  |  |  |  |  |  |  |  |  |  |  |  |  |  |  |  |  |  |  |  |  |  |  |  |  | *tsp-19* | TetraSPanin family |
|  |  |  |  |  |  |  |  |  |  |  |  |  |  |  |  |  |  |  |  |  |  |  |  |  |  |  |  |  |  |  |  |  |  |  |  |  |  | F53G12.17 |  |
|  |  |  |  |  |  |  |  |  |  |  |  |  |  |  |  |  |  |  |  |  |  |  |  |  |  |  |  |  |  |  |  |  |  |  |  |  |  | *pud-1.1* |  |
|  |  |  |  |  |  |  |  |  |  |  |  |  |  |  |  |  |  |  |  |  |  |  |  |  |  |  |  |  |  |  |  |  |  |  |  |  |  | F25F2.4 |  |
|  |  |  |  |  |  |  |  |  |  |  |  |  |  |  |  |  |  |  |  |  |  |  |  |  |  |  |  |  |  |  |  |  |  |  |  |  |  | F46C8.18 |  |
|  |  |  |  |  |  |  |  |  |  |  |  |  |  |  |  |  |  |  |  |  |  |  |  |  |  |  |  |  |  |  |  |  |  |  |  |  |  | Y6G8.5 |  |
|  |  |  |  |  |  |  |  |  |  |  |  |  |  |  |  |  |  |  |  |  |  |  |  |  |  |  |  |  |  |  |  |  |  |  |  |  |  | Y67A6A.3 |  |
|  |  |  |  |  |  |  |  |  |  |  |  |  |  |  |  |  |  |  |  |  |  |  |  |  |  |  |  |  |  |  |  |  |  |  |  |  |  | *srw-116* | Serpentine Receptor, class W |
|  |  |  |  |  |  |  |  |  |  |  |  |  |  |  |  |  |  |  |  |  |  |  |  |  |  |  |  |  |  |  |  |  |  |  |  |  |  | *str-67* | Seven TM Receptor |
|  |  |  |  |  |  |  |  |  |  |  |  |  |  |  |  |  |  |  |  |  |  |  |  |  |  |  |  |  |  |  |  |  |  |  |  |  |  | C54D2.17 |  |
|  |  |  |  |  |  |  |  |  |  |  |  |  |  |  |  |  |  |  |  |  |  |  |  |  |  |  |  |  |  |  |  |  |  |  |  |  |  | F47E1.14 |  |
|  |  |  |  |  |  |  |  |  |  |  |  |  |  |  |  |  |  |  |  |  |  |  |  |  |  |  |  |  |  |  |  |  |  |  |  |  |  | W02A2.5 |  |
|  |  |  |  |  |  |  |  |  |  |  |  |  |  |  |  |  |  |  |  |  |  |  |  |  |  |  |  |  |  |  |  |  |  |  |  |  |  | *srh-60* | Serpentine Receptor, class H |
|  |  |  |  |  |  |  |  |  |  |  |  |  |  |  |  |  |  |  |  |  |  |  |  |  |  |  |  |  |  |  |  |  |  |  |  |  |  | Y53C12A.7 |  |
|  |  |  |  |  |  |  |  |  |  |  |  |  |  |  |  |  |  |  |  |  |  |  |  |  |  |  |  |  |  |  |  |  |  |  |  |  |  | Y48G8AR.7 |  |
|  |  |  |  |  |  |  |  |  |  |  |  |  |  |  |  |  |  |  |  |  |  |  |  |  |  |  |  |  |  |  |  |  |  |  |  |  |  | Y48G8AR.3 |  |
|  |  |  |  |  |  |  |  |  |  |  |  |  |  |  |  |  |  |  |  |  |  |  |  |  |  |  |  |  |  |  |  |  |  |  |  |  |  | R07E5.15 |  |
|  |  |  |  |  |  |  |  |  |  |  |  |  |  |  |  |  |  |  |  |  |  |  |  |  |  |  |  |  |  |  |  |  |  |  |  |  |  | *best-7* | BESTrophin (chloride channel) homolog |
|  |  |  |  |  |  |  |  |  |  |  |  |  |  |  |  |  |  |  |  |  |  |  |  |  |  |  |  |  |  |  |  |  |  |  |  |  |  | F40H3.1 |  |
|  |  |  |  |  |  |  |  |  |  |  |  |  |  |  |  |  |  |  |  |  |  |  |  |  |  |  |  |  |  |  |  |  |  |  |  |  |  | *lgc-33* | Ligand-Gated ion Channel |
|  |  |  |  |  |  |  |  |  |  |  |  |  |  |  |  |  |  |  |  |  |  |  |  |  |  |  |  |  |  |  |  |  |  |  |  |  |  | *kcnl-4* | KCNN (potassium K ChaNNel, calcium activated)-Like |
|  |  |  |  |  |  |  |  |  |  |  |  |  |  |  |  |  |  |  |  |  |  |  |  |  |  |  |  |  |  |  |  |  |  |  |  |  |  | *pqn-83* | Prion-like-(Q/N-rich)-domain-bearing protein |
|  |  |  |  |  |  |  |  |  |  |  |  |  |  |  |  |  |  |  |  |  |  |  |  |  |  |  |  |  |  |  |  |  |  |  |  |  |  | Y67A10A.9 |  |
|  |  |  |  |  |  |  |  |  |  |  |  |  |  |  |  |  |  |  |  |  |  |  |  |  |  |  |  |  |  |  |  |  |  |  |  |  |  | T10B5.10 |  |
|  |  |  |  |  |  |  |  |  |  |  |  |  |  |  |  |  |  |  |  |  |  |  |  |  |  |  |  |  |  |  |  |  |  |  |  |  |  | *cal-7* | CALmodulin related genes |
|  |  |  |  |  |  |  |  |  |  |  |  |  |  |  |  |  |  |  |  |  |  |  |  |  |  |  |  |  |  |  |  |  |  |  |  |  |  | B0198.2 |  |
|  |  |  |  |  |  |  |  |  |  |  |  |  |  |  |  |  |  |  |  |  |  |  |  |  |  |  |  |  |  |  |  |  |  |  |  |  |  | *lgc-46* | Ligand-Gated ion Channel |
|  |  |  |  |  |  |  |  |  |  |  |  |  |  |  |  |  |  |  |  |  |  |  |  |  |  |  |  |  |  |  |  |  |  |  |  |  |  | C30G7.2 |  |
|  |  |  |  |  |  |  |  |  |  |  |  |  |  |  |  |  |  |  |  |  |  |  |  |  |  |  |  |  |  |  |  |  |  |  |  |  |  | Y45F3A.1 |  |
|  |  |  |  |  |  |  |  |  |  |  |  |  |  |  |  |  |  |  |  |  |  |  |  |  |  |  |  |  |  |  |  |  |  |  |  |  |  | *cah-4* | Carbonic AnHydrase |
|  |  |  |  |  |  |  |  |  |  |  |  |  |  |  |  |  |  |  |  |  |  |  |  |  |  |  |  |  |  |  |  |  |  |  |  |  |  | *ipp-5* | Inositol Polyphosphate 5-Phosphatase |
|  |  |  |  |  |  |  |  |  |  |  |  |  |  |  |  |  |  |  |  |  |  |  |  |  |  |  |  |  |  |  |  |  |  |  |  |  |  | *mdl-1* | MAD-Like |
|  |  |  |  |  |  |  |  |  |  |  |  |  |  |  |  |  |  |  |  |  |  |  |  |  |  |  |  |  |  |  |  |  |  |  |  |  |  | *seb-2* | SEcretin/class B GPCR |
|  |  |  |  |  |  |  |  |  |  |  |  |  |  |  |  |  |  |  |  |  |  |  |  |  |  |  |  |  |  |  |  |  |  |  |  |  |  | Y37A1A.3 |  |
|  |  |  |  |  |  |  |  |  |  |  |  |  |  |  |  |  |  |  |  |  |  |  |  |  |  |  |  |  |  |  |  |  |  |  |  |  |  | *mam-4* | MAM (Meprin, A5-protein, PTPmu) domain protein |
|  |  |  |  |  |  |  |  |  |  |  |  |  |  |  |  |  |  |  |  |  |  |  |  |  |  |  |  |  |  |  |  |  |  |  |  |  |  | *igeg-2* | IG (immunoglobulin), EGF and transmmembrane domain |
|  |  |  |  |  |  |  |  |  |  |  |  |  |  |  |  |  |  |  |  |  |  |  |  |  |  |  |  |  |  |  |  |  |  |  |  |  |  | *arrd-19* | ARRestin Domain protein |
|  |  |  |  |  |  |  |  |  |  |  |  |  |  |  |  |  |  |  |  |  |  |  |  |  |  |  |  |  |  |  |  |  |  |  |  |  |  | T23F6.5 |  |
|  |  |  |  |  |  |  |  |  |  |  |  |  |  |  |  |  |  |  |  |  |  |  |  |  |  |  |  |  |  |  |  |  |  |  |  |  |  | *acs-3* | fatty Acid CoA Synthetase family |
|  |  |  |  |  |  |  |  |  |  |  |  |  |  |  |  |  |  |  |  |  |  |  |  |  |  |  |  |  |  |  |  |  |  |  |  |  |  | Y58A7A.2 |  |
|  |  |  |  |  |  |  |  |  |  |  |  |  |  |  |  |  |  |  |  |  |  |  |  |  |  |  |  |  |  |  |  |  |  |  |  |  |  | F59B10.6 |  |
|  |  |  |  |  |  |  |  |  |  |  |  |  |  |  |  |  |  |  |  |  |  |  |  |  |  |  |  |  |  |  |  |  |  |  |  |  |  | F59B10.5 |  |
|  |  |  |  |  |  |  |  |  |  |  |  |  |  |  |  |  |  |  |  |  |  |  |  |  |  |  |  |  |  |  |  |  |  |  |  |  |  | *valv-1* | VALVe cells defective |
|  |  |  |  |  |  |  |  |  |  |  |  |  |  |  |  |  |  |  |  |  |  |  |  |  |  |  |  |  |  |  |  |  |  |  |  |  |  | *lips-9* | LIPaSe related |
|  |  |  |  |  |  |  |  |  |  |  |  |  |  |  |  |  |  |  |  |  |  |  |  |  |  |  |  |  |  |  |  |  |  |  |  |  |  | *acdh-6* | Acyl CoA DeHydrogenase |
|  |  |  |  |  |  |  |  |  |  |  |  |  |  |  |  |  |  |  |  |  |  |  |  |  |  |  |  |  |  |  |  |  |  |  |  |  |  | R12E2.7 |  |
|  |  |  |  |  |  |  |  |  |  |  |  |  |  |  |  |  |  |  |  |  |  |  |  |  |  |  |  |  |  |  |  |  |  |  |  |  |  | ZK287.3 |  |
|  |  |  |  |  |  |  |  |  |  |  |  |  |  |  |  |  |  |  |  |  |  |  |  |  |  |  |  |  |  |  |  |  |  |  |  |  |  | C11E4.6 |  |
|  |  |  |  |  |  |  |  |  |  |  |  |  |  |  |  |  |  |  |  |  |  |  |  |  |  |  |  |  |  |  |  |  |  |  |  |  |  | T17H7.7 |  |
|  |  |  |  |  |  |  |  |  |  |  |  |  |  |  |  |  |  |  |  |  |  |  |  |  |  |  |  |  |  |  |  |  |  |  |  |  |  | F13H6.1 |  |
|  |  |  |  |  |  |  |  |  |  |  |  |  |  |  |  |  |  |  |  |  |  |  |  |  |  |  |  |  |  |  |  |  |  |  |  |  |  | *npax-2* | N-terminal PAX (PAI domain only) protein |
|  |  |  |  |  |  |  |  |  |  |  |  |  |  |  |  |  |  |  |  |  |  |  |  |  |  |  |  |  |  |  |  |  |  |  |  |  |  | F58E6.13 |  |
|  |  |  |  |  |  |  |  |  |  |  |  |  |  |  |  |  |  |  |  |  |  |  |  |  |  |  |  |  |  |  |  |  |  |  |  |  |  | *col-113* | COLlagen |
|  |  |  |  |  |  |  |  |  |  |  |  |  |  |  |  |  |  |  |  |  |  |  |  |  |  |  |  |  |  |  |  |  |  |  |  |  |  | *col-97* | COLlagen |
|  |  |  |  |  |  |  |  |  |  |  |  |  |  |  |  |  |  |  |  |  |  |  |  |  |  |  |  |  |  |  |  |  |  |  |  |  |  | *col-109* | COLlagen |
|  |  |  |  |  |  |  |  |  |  |  |  |  |  |  |  |  |  |  |  |  |  |  |  |  |  |  |  |  |  |  |  |  |  |  |  |  |  | *col-48* | COLlagen |
|  |  |  |  |  |  |  |  |  |  |  |  |  |  |  |  |  |  |  |  |  |  |  |  |  |  |  |  |  |  |  |  |  |  |  |  |  |  | *col-14* | COLlagen |
|  |  |  |  |  |  |  |  |  |  |  |  |  |  |  |  |  |  |  |  |  |  |  |  |  |  |  |  |  |  |  |  |  |  |  |  |  |  | C34C6.3 |  |
|  |  |  |  |  |  |  |  |  |  |  |  |  |  |  |  |  |  |  |  |  |  |  |  |  |  |  |  |  |  |  |  |  |  |  |  |  |  | *pph-1* | Protein PHosphatase |
|  |  |  |  |  |  |  |  |  |  |  |  |  |  |  |  |  |  |  |  |  |  |  |  |  |  |  |  |  |  |  |  |  |  |  |  |  |  | *tes-1* | TEStin (human testis-derived transcript) homolog |
|  |  |  |  |  |  |  |  |  |  |  |  |  |  |  |  |  |  |  |  |  |  |  |  |  |  |  |  |  |  |  |  |  |  |  |  |  |  | *arrd-18* | ARRestin Domain protein |
|  |  |  |  |  |  |  |  |  |  |  |  |  |  |  |  |  |  |  |  |  |  |  |  |  |  |  |  |  |  |  |  |  |  |  |  |  |  | C27D6.1 |  |
|  |  |  |  |  |  |  |  |  |  |  |  |  |  |  |  |  |  |  |  |  |  |  |  |  |  |  |  |  |  |  |  |  |  |  |  |  |  | *glb-14* | GLoBin related |
|  |  |  |  |  |  |  |  |  |  |  |  |  |  |  |  |  |  |  |  |  |  |  |  |  |  |  |  |  |  |  |  |  |  |  |  |  |  | C04F12.5 |  |
|  |  |  |  |  |  |  |  |  |  |  |  |  |  |  |  |  |  |  |  |  |  |  |  |  |  |  |  |  |  |  |  |  |  |  |  |  |  | *pho-8* | intestinal acid PHOsphatase |
|  |  |  |  |  |  |  |  |  |  |  |  |  |  |  |  |  |  |  |  |  |  |  |  |  |  |  |  |  |  |  |  |  |  |  |  |  |  | *abch-1* | ABC transporter, class H |
|  |  |  |  |  |  |  |  |  |  |  |  |  |  |  |  |  |  |  |  |  |  |  |  |  |  |  |  |  |  |  |  |  |  |  |  |  |  | *abcx-1* | ABC transporter, eXtended |
|  |  |  |  |  |  |  |  |  |  |  |  |  |  |  |  |  |  |  |  |  |  |  |  |  |  |  |  |  |  |  |  |  |  |  |  |  |  | F32B4.8 |  |
|  |  |  |  |  |  |  |  |  |  |  |  |  |  |  |  |  |  |  |  |  |  |  |  |  |  |  |  |  |  |  |  |  |  |  |  |  |  | R04B3.1 |  |
|  |  |  |  |  |  |  |  |  |  |  |  |  |  |  |  |  |  |  |  |  |  |  |  |  |  |  |  |  |  |  |  |  |  |  |  |  |  | C09G1.4 |  |
|  |  |  |  |  |  |  |  |  |  |  |  |  |  |  |  |  |  |  |  |  |  |  |  |  |  |  |  |  |  |  |  |  |  |  |  |  |  | F31B12.4 |  |

### Phenotypes enriched

none found

### Anatomy terms enriched

none found

### GO terms enriched

|  |  |  |
| --- | --- | --- |
| **GO term** | **Number of genes** | **FDR-corrected p-value** |
| structural constituent of cuticle | 6 | 0.0042 |

### Expression clusters enriched

|  |  |  |  |
| --- | --- | --- | --- |
| **Group name** | **Number in cluster** | **Enrichment** | **FDR corrected p** |
| Expression Pattern Group G, enriched for genes involved in locomotion. | 21 | 2.97 | 0.00120 |
| Genes with expression level down regulated in mir-35 mutants comparing with N2. | 17 | 3.55 | 0.00126 |
| Genes with expression level up regulated after treatment with Methylmercury (MeHg) by RNAseq analysis. | 12 | 4.84 | 0.00190 |
| Genes downregulated on Comamonas DA1877 relative to E. coli OP50, Young adult | 9 | 6.06 | 0.00490 |
| Genes down regulated in crh-1(nn3315) comparing to in N2. | 11 | 4.31 | 0.01150 |
| Developmentally modulated gene cluster. cgc4386\_cluster\_2\_4 | 6 | 9.21 | 0.01230 |
| Genes up regulated by mir-243(n4759). | 21 | 2.47 | 0.01500 |
| Genes showing < 0.5-fold down-regulated expression (p < 0.001) both in aex-3::His-SUMO-1 or myo-4::His-SUMO-1 C. elegans. | 9 | 5.02 | 0.01790 |
| Genes regulated by octr-1(ok371) after infected with P. aeruginosa PA14 for 4 hours at 25 centigrade. | 14 | 3.04 | 0.03680 |

### Motifs enriched

|  |  |  |  |  |  |
| --- | --- | --- | --- | --- | --- |
| **Motif** | **Logo** | **Possible orthologs** | **Number of motifs in cluster** | **Enrichment** | **FDR corrected p** |
| EN2\_1 |  | ceh-1 alr-1 ceh-31 ceh-16 ceh-9 lim-7 ceh-43 | 34 | 2.65 | 5.7e-06 |
| pTH9250 |  | dmd-3 C34D1.1 | 22 | 3.98 | 5.9e-06 |
| MA0537.1 |  | blmp-1 (0.61) | 46 | 2.04 | 8.2e-06 |
| I$E74A\_01 |  | lin-1 C24A1.2 | 53 | 1.81 | 1.0e-05 |
| MA0192.1 |  | ceh-8 (0.63) ceh-24 (0.51) ceh-19 ceh-1 alr-1 ceh-30 ceh-31 ceh-9 lim-7 lin-39 ceh-43 | 33 | 2.61 | 1.3e-05 |
| pTH10816 |  | dmd-6 | 38 | 2.32 | 1.5e-05 |
| pTH9296 |  | ztf-6 C34D1.1 gei-11 | 28 | 2.94 | 2.1e-05 |
| pTH10787 |  | ceh-1 alr-1 eyg-1 ceh-45 ceh-10 lin-39 | 36 | 2.36 | 2.6e-05 |
| pTH9198 |  | dmd-3 | 32 | 2.56 | 3.4e-05 |
| Nkx1-2\_3214 |  | ceh-30 | 30 | 2.70 | 3.4e-05 |
| Gbx1\_2883 |  | ceh-12 alr-1 ceh-1 ceh-45 lin-39 ceh-53 ceh-43 | 34 | 2.40 | 4.9e-05 |
| V$TAXCREB\_02 |  | crh-1 zip-3 | 45 | 1.95 | 4.9e-05 |
| CG31670\_SOLEXA\_5\_FBgn0031375 |  | CELE\_Y38H8A.5 | 44 | 1.98 | 5.3e-05 |
| V$S8\_01 |  | ceh-45 | 30 | 2.61 | 6.5e-05 |
| pTH4325 |  | ceh-18 (0.53) | 36 | 2.25 | 8.0e-05 |
| SP1\_f2 |  | klf-2 klf-1 | 34 | 2.32 | 9.8e-05 |
| pTH9923 |  | alr-1 ceh-12 ceh-1 ceh-2 egl-5 ceh-30 ceh-31 ceh-16 ceh-14 cog-1 ceh-23 lim-6 lim-7 mls-2 ceh-45 pha-2 ceh-10 lim-4 ceh-36 lin-39 and 5 others  [full list] | 21 | 3.43 | 1.1e-04 |
| Mv129 |  | ceh-18 (0.53) ceh-6 tbp-1 | 37 | 2.17 | 1.1e-04 |
| pTH9220 |  | mbr-1 | 48 | 1.80 | 1.2e-04 |
| VENTX\_1 |  | alr-1 ceh-45 pha-2 ceh-36 ceh-53 | 31 | 2.45 | 1.3e-04 |
| luna\_SOLEXA\_5\_FBgn0040765 |  | klf-2 klf-1 | 45 | 1.87 | 1.4e-04 |
| PHOX2B\_2 |  | alr-1 | 35 | 2.23 | 1.4e-04 |
| N$SKN1\_01 |  | ceh-2 skn-1 | 36 | 2.18 | 1.5e-04 |
| MA0071.1 |  | nhr-71 (0.51) nhr-6 nhr-213 nhr-68 nhr-10 | 42 | 1.96 | 1.5e-04 |
| CENPB\_1 |  | F21D5.4 | 43 | 1.92 | 1.6e-04 |
| pTH9245 |  | ceh-18 (0.53) | 34 | 2.26 | 1.7e-04 |
| pTH9177 |  | F10B5.3 Y53C10A.3 | 52 | 1.68 | 1.9e-04 |
| pTH9336 |  | mab-9 tbx-38 tbx-39 tbx-43 | 35 | 2.20 | 2.0e-04 |
| pTH6281 |  | nhr-2 (-0.52) nhr-213 nhr-239 | 36 | 2.14 | 2.3e-04 |
| PROX1\_1 |  | crh-1 ceh-26 | 23 | 3.00 | 2.3e-04 |
| Pax7\_3783 |  | alr-1 lim-7 | 12 | 5.67 | 3.0e-04 |
| MA0162.2 |  | ZC328.2 klf-2 klf-1 | 32 | 2.29 | 3.1e-04 |
| pTH9155 |  | lin-48 (0.66) B0310.2 D1081.8 | 34 | 2.19 | 3.2e-04 |
| pTH9222 |  | mel-28 | 33 | 2.23 | 3.4e-04 |
| MA0204.1 |  | ceh-32 dmd-5 dmd-4 | 38 | 2.02 | 3.6e-04 |
| Lhx1\_2240 |  | lim-7 | 22 | 3.00 | 3.9e-04 |
| Foxk1\_1 |  | lin-31 | 45 | 1.80 | 4.0e-04 |
| V$TCF11\_01 |  | skn-1 | 36 | 2.08 | 4.3e-04 |
| ETS2\_f1 |  | lin-1 C24A1.2 | 53 | 1.61 | 4.3e-04 |
| pTH8996 |  | sma-4 daf-8 | 36 | 2.08 | 4.4e-04 |
| Vsx1\_1728 |  | alr-1 | 29 | 2.41 | 4.4e-04 |
| Nsy-7 |  | hmg-5 (-0.57) nsy-7 | 34 | 2.15 | 4.5e-04 |
| pTH9237 |  | mel-28 | 17 | 3.70 | 4.8e-04 |
| pTH5690 |  | ceh-32 | 38 | 1.99 | 5.0e-04 |
| En2\_0952 |  | ceh-16 lim-6 lim-7 | 29 | 2.39 | 5.2e-04 |
| Six2\_2307 |  | ceh-32 ceh-34 | 34 | 2.14 | 5.2e-04 |
| pTH5267 |  | hlh-16 ngn-1 hlh-32 | 33 | 2.18 | 5.3e-04 |
| pTH9215 |  | C34D1.1 | 29 | 2.38 | 5.5e-04 |
| HXC8\_f1 |  | ceh-12 ceh-20 lin-39 | 36 | 2.03 | 6.7e-04 |
| MA0264.1 |  | ceh-24 (0.51) dsc-1 ceh-22 | 35 | 2.07 | 7.0e-04 |
| NR2F6\_f1 |  | nhr-2 (-0.52) nhr-239 | 46 | 1.74 | 7.1e-04 |
| MA0594.1 |  | lin-39 | 37 | 1.99 | 7.6e-04 |
| pTH10777 |  | dmd-3 | 27 | 2.45 | 7.7e-04 |
| Sp4\_1011 |  | klf-2 sptf-3 klf-1 | 30 | 2.27 | 8.1e-04 |
| En1\_3123 |  | ceh-16 ceh-53 | 27 | 2.44 | 8.1e-04 |
| MA0260.1 |  | ztf-28 che-1 | 43 | 1.80 | 8.6e-04 |
| Titf1\_1722 |  | ceh-24 (0.51) dsc-1 | 32 | 2.17 | 8.7e-04 |
| pTH5714 |  | nhr-239 | 29 | 2.31 | 9.2e-04 |
| Six3\_1732 |  | ceh-34 | 37 | 1.95 | 1.1e-03 |
| Nkx1-1\_3856 |  | ceh-30 | 26 | 2.46 | 1.1e-03 |
| RORA\_f1 |  | nhr-213 nhr-118 | 33 | 2.09 | 1.1e-03 |
| MEF2D\_1 |  | mef-2 | 30 | 2.22 | 1.2e-03 |
| HLH4C\_da\_SANGER\_5\_4\_FBgn0011277 |  | ces-1 hlh-15 hlh-1 | 39 | 1.88 | 1.3e-03 |
| MA0543.1 |  | eor-1 | 43 | 1.77 | 1.3e-03 |
| Ets65A\_SANGER\_10\_FBgn0005658 |  | lin-1 C24A1.2 | 29 | 2.26 | 1.3e-03 |
| MA0254.1 |  | ceh-18 (0.53) unc-86 | 31 | 2.16 | 1.4e-03 |
| pTH5508 |  | nhr-2 (-0.52) nhr-19 nhr-213 nhr-10 nhr-69 | 36 | 1.95 | 1.5e-03 |
| MCR\_f1 |  | nhr-255 (0.56) | 43 | 1.75 | 1.6e-03 |
| ovo\_FlyReg\_FBgn0003028 |  | lin-48 (0.66) pax-3 | 26 | 2.38 | 1.7e-03 |
| PLAG1\_si |  | klf-2 Y53H1A.2 | 35 | 1.97 | 1.8e-03 |
| V$NCX\_01 |  | ceh-19 | 36 | 1.93 | 1.9e-03 |
| MA0033.1 |  | lin-31 hlh-8 hlh-15 hlh-32 | 27 | 2.31 | 1.9e-03 |
| Spdef |  | lin-1 | 30 | 2.15 | 2.0e-03 |
| Jundm2\_0911 |  | fos-1 (0.58) | 35 | 1.96 | 2.0e-03 |
| disco-r-Cl1\_SANGER\_5\_FBgn0042650 |  | lin-31 nhr-68 F55C5.11 | 34 | 1.99 | 2.0e-03 |
| MA0451.1 |  | nhr-2 (-0.52) | 41 | 1.78 | 2.1e-03 |
| pTH10823 |  | B0310.2 | 18 | 3.07 | 2.2e-03 |
| pTH6447 |  | ceh-19 | 30 | 2.13 | 2.3e-03 |
| Mv75 |  | elt-1 | 41 | 1.77 | 2.3e-03 |
| V$OCT1\_03 |  | ceh-18 (0.53) | 28 | 2.23 | 2.3e-03 |
| pTH8745 |  | attf-1 | 25 | 2.40 | 2.3e-03 |
| Gmeb1\_1745 |  | attf-1 | 26 | 2.33 | 2.4e-03 |
| GRHL1\_2 |  | grh-1 | 37 | 1.87 | 2.4e-03 |
| pTH9381 |  | ceh-18 (0.53) | 18 | 3.03 | 2.5e-03 |
| klu\_SOLEXA\_5\_FBgn0013469 |  | ZC328.2 daf-16 | 45 | 1.67 | 2.6e-03 |
| pTH9928 |  | fos-1 (0.58) jun-1 (0.54) crh-1 | 36 | 1.90 | 2.7e-03 |
| MEIS2\_do |  | ceh-32 | 24 | 2.44 | 2.7e-03 |
| Barx2\_3447 |  | ceh-43 | 30 | 2.11 | 2.7e-03 |
| Dlx2\_2273 |  | ceh-43 | 28 | 2.20 | 2.7e-03 |
| pTH10768 |  | med-2 | 33 | 1.99 | 2.8e-03 |
| MA0043.1 |  | ces-2 C01B12.2 Y51H4A.4 | 31 | 2.06 | 2.9e-03 |
| pTH9054 |  | nhr-255 (0.56) npax-1 | 19 | 2.85 | 3.2e-03 |
| I$KR\_01 |  | B0310.2 | 28 | 2.18 | 3.3e-03 |
| Six1\_0935 |  | ceh-32 | 31 | 2.04 | 3.5e-03 |
| Bbx\_3753 |  | gei-3 | 39 | 1.78 | 3.6e-03 |
| pTH8649 |  | mbr-1 | 44 | 1.67 | 3.6e-03 |
| Dlx3\_1030 |  | ceh-43 | 18 | 2.93 | 3.6e-03 |
| pTH9247 |  | dmd-3 C34D1.1 | 49 | 1.57 | 3.7e-03 |
| V$HEN1\_02 |  | hlh-15 | 26 | 2.26 | 3.8e-03 |
| pTH10805 |  | ztf-16 | 29 | 2.11 | 3.8e-03 |
| V$CREB\_01 |  | crh-1 W08E12.1 | 31 | 2.03 | 3.9e-03 |
| V$ROAZ\_01 |  | D1081.8 Y53H1A.2 C09F5.3 | 31 | 2.03 | 3.9e-03 |
| pTH9244 |  | tbx-39 | 33 | 1.95 | 3.9e-03 |
| V$TST1\_01 |  | ceh-18 (0.53) lin-39 | 30 | 2.06 | 4.0e-03 |
| pTH3751 |  | tbx-39 | 32 | 1.98 | 4.2e-03 |
| V$DELTAEF1\_01 |  | ztf-6 | 41 | 1.72 | 4.3e-03 |
| pTH6478 |  | lim-7 | 28 | 2.13 | 4.5e-03 |
| Spdef\_0905 |  | lin-1 | 27 | 2.18 | 4.5e-03 |
| ARNT\_f1 |  | lin-22 aha-1 hlh-27 | 21 | 2.57 | 4.5e-03 |
| Atf1\_3026 |  | crh-1 | 34 | 1.90 | 4.6e-03 |
| Elf3\_3876 |  | C24A1.2 | 36 | 1.84 | 4.6e-03 |
| pTH5080 |  | fos-1 (0.58) atf-5 crh-1 | 30 | 2.04 | 4.7e-03 |
| pTH10633 |  | R07H5.10 | 51 | 1.52 | 4.8e-03 |
| Pou3f3\_3235 |  | ceh-6 | 28 | 2.12 | 4.9e-03 |
| pTH10788 |  | tbx-33 | 35 | 1.86 | 5.1e-03 |
| pTH3510 |  | F13H6.1 (0.81) nhr-86 | 35 | 1.86 | 5.1e-03 |
| CG7386\_F10-12\_SANGER\_5\_FBgn0035691 |  | F56D1.1 | 25 | 2.26 | 5.3e-03 |
| Elk4 |  | F19F10.1 lin-1 C24A1.2 | 41 | 1.70 | 5.4e-03 |
| CG14962\_SANGER\_5\_FBgn0035407 |  | ceh-24 (0.51) ces-1 C34H4.5 | 44 | 1.64 | 5.5e-03 |
| FOXF2\_f1 |  | lin-31 let-381 | 36 | 1.82 | 5.5e-03 |
| Hmx3\_3490 |  | ceh-9 | 17 | 2.94 | 5.5e-03 |
| cato\_da\_SANGER\_10\_FBgn0024249 |  | lin-32 hlh-15 hlh-1 | 44 | 1.63 | 5.6e-03 |
| V$AREB6\_02 |  | ztf-6 | 38 | 1.76 | 6.0e-03 |
| pTH6436 |  | ceh-53 | 25 | 2.24 | 6.1e-03 |
| pTH9149 |  | ztf-30 (0.61) | 26 | 2.18 | 6.2e-03 |
| Hoxc10\_1 |  | ceh-13 php-3 D1005.3 | 30 | 2.00 | 6.2e-03 |
| pTH5928 |  | ceh-34 | 29 | 2.04 | 6.4e-03 |
| FLI1\_f1 |  | lin-1 | 23 | 2.35 | 6.5e-03 |
| pTH1292 |  | pzf-1 | 47 | 1.57 | 6.5e-03 |
| Lbx2\_3869 |  | mls-2 | 35 | 1.83 | 6.6e-03 |
| NR2F1\_2 |  | nhr-2 (-0.52) | 42 | 1.66 | 7.0e-03 |
| pTH6445 |  | ceh-5 | 43 | 1.64 | 7.0e-03 |
| pTH3998 |  | tbx-39 | 31 | 1.95 | 7.4e-03 |
| pTH10822 |  | hlh-10 unc-120 | 26 | 2.15 | 7.5e-03 |
| Nkx6-3\_3446 |  | cog-1 | 29 | 2.02 | 7.7e-03 |
| pTH9930 |  | lin-29 fkh-7 | 49 | 1.52 | 7.7e-03 |
| EN1\_2 |  | ceh-16 | 16 | 2.97 | 8.0e-03 |
| pTH1001 |  | dnj-17 | 41 | 1.67 | 8.0e-03 |
| NR2E3\_f1 |  | nhr-100 lin-1 lin-39 | 24 | 2.24 | 8.1e-03 |
| pTH9189 |  | ceh-18 (0.53) dmd-3 | 33 | 1.87 | 8.2e-03 |
| ERG\_4 |  | lin-1 | 20 | 2.52 | 8.2e-03 |
| Elf4 |  | C24A1.2 | 28 | 2.05 | 8.2e-03 |
| pTH3046 |  | Y116A8C.22 | 39 | 1.71 | 8.3e-03 |
| Six6\_2267 |  | elt-6 (0.56) ceh-32 elt-3 elt-1 ceh-34 | 40 | 1.68 | 8.4e-03 |
| DLX2\_f1 |  | lin-39 ceh-43 | 36 | 1.78 | 8.6e-03 |
| FOXQ1\_f1 |  | lin-31 let-381 daf-16 | 37 | 1.75 | 8.8e-03 |
| pTH10810 |  | lsl-1 lsy-2 | 23 | 2.29 | 8.8e-03 |
| pTH5065 |  | hlh-30 | 19 | 2.59 | 9.0e-03 |
| pTH9246 |  | let-381 C34D1.1 | 53 | 1.45 | 9.1e-03 |
| hth\_SOLEXA\_2\_FBgn0001235 |  | ceh-32 ceh-20 | 42 | 1.63 | 9.3e-03 |
| Sox1\_2631 |  | sox-4 | 46 | 1.56 | 9.3e-03 |
| V$OCT1\_06 |  | ceh-18 (0.53) | 32 | 1.88 | 9.5e-03 |
| pTH5778 |  | egl-5 | 17 | 2.78 | 9.6e-03 |
| Dlx1\_1741 |  | ceh-43 | 22 | 2.33 | 9.7e-03 |
| pTH6569 |  | ceh-43 | 27 | 2.06 | 9.9e-03 |
| Elf3 |  | C24A1.2 | 30 | 1.94 | 9.9e-03 |
| pTH8671 |  | attf-1 C01B12.2 | 25 | 2.15 | 1.0e-02 |
| pTH2936 |  | nhr-239 | 41 | 1.65 | 1.0e-02 |
| HeLa-S3\_ZNF274\_UCD |  | C28G1.4 (-0.68) | 32 | 1.87 | 1.0e-02 |
| pTH5916 |  | efl-2 | 14 | 3.20 | 1.0e-02 |
| pTH6327 |  | dsc-1 | 37 | 1.73 | 1.0e-02 |
| SOX2\_5 |  | sox-4 | 32 | 1.87 | 1.0e-02 |
| BARHL2\_4 |  | ceh-31 | 27 | 2.05 | 1.0e-02 |
| Hoxb6\_3428 |  | lin-39 | 25 | 2.15 | 1.0e-02 |
| pTH9934 |  | Y53H1A.2 | 38 | 1.71 | 1.1e-02 |
| PAX6\_f1 |  | pax-3 pax-2 | 44 | 1.59 | 1.1e-02 |
| pnr\_SANGER\_5\_FBgn0003117 |  | elt-1 | 54 | 1.43 | 1.1e-02 |
| Hoxa7\_2668 |  | lin-39 | 35 | 1.78 | 1.1e-02 |
| pTH2820 |  | ZC328.2 | 42 | 1.62 | 1.1e-02 |
| pTH9135 |  | pop-1 | 32 | 1.86 | 1.1e-02 |
| pTH6449 |  | ceh-43 | 27 | 2.04 | 1.1e-02 |
| pTH6562 |  | ceh-5 | 27 | 2.04 | 1.2e-02 |
| pTH9279 |  | Y116A8C.22 | 40 | 1.66 | 1.2e-02 |
| ELF3\_f1 |  | C24A1.2 | 14 | 3.13 | 1.2e-02 |
| pTH9708 |  | ceh-34 | 36 | 1.74 | 1.2e-02 |
| pTH6423 |  | pha-2 | 16 | 2.83 | 1.2e-02 |
| Eip93F\_SANGER\_10\_FBgn0013948 |  | mbr-1 | 53 | 1.43 | 1.3e-02 |
| Elf5 |  | C24A1.2 | 33 | 1.82 | 1.3e-02 |
| V$AREB6\_01 |  | ztf-6 | 33 | 1.81 | 1.3e-02 |
| Hlxb9\_3422 |  | ceh-12 | 24 | 2.16 | 1.3e-02 |
| pTH6071 |  | C33G8.2 | 12 | 3.52 | 1.3e-02 |
| ARNT2\_si |  | aha-1 | 35 | 1.76 | 1.3e-02 |
| Hoxa4\_3426 |  | lin-39 | 21 | 2.33 | 1.3e-02 |
| MSX2\_1 |  | ceh-1 ceh-31 | 30 | 1.90 | 1.4e-02 |
| Hoxa3\_2783 |  | lin-39 | 26 | 2.06 | 1.4e-02 |
| TFEC\_1 |  | hlh-30 pax-1 aha-1 | 30 | 1.90 | 1.4e-02 |
| pTH10030 |  | xbp-1 C01B12.2 | 37 | 1.70 | 1.4e-02 |
| MA0473.1 |  | lin-1 C24A1.2 | 34 | 1.78 | 1.4e-02 |
| V$GATA1\_03 |  | elt-1 | 39 | 1.66 | 1.5e-02 |
| pTH10722 |  | egrh-3 | 35 | 1.75 | 1.5e-02 |
| Hoxd1\_3448 |  | ceh-12 | 23 | 2.19 | 1.5e-02 |
| pTH10769 |  | Y48G1C.6 | 30 | 1.89 | 1.5e-02 |
| Zbtb12\_2932 |  | lsy-27 | 13 | 3.23 | 1.5e-02 |
| Hoxc8\_3429 |  | lin-39 | 40 | 1.63 | 1.5e-02 |
| Oli\_da\_SANGER\_5\_3\_FBgn0032651 |  | hlh-12 hlh-32 | 42 | 1.59 | 1.5e-02 |
| MA0027.1 |  | ceh-16 | 36 | 1.72 | 1.6e-02 |
| MA0454.1 |  | odd-1 odd-2 | 36 | 1.71 | 1.6e-02 |
| MA0467.1 |  | ceh-45 tbx-39 | 27 | 1.99 | 1.6e-02 |
| V$GATA1\_01 |  | elt-1 | 31 | 1.85 | 1.6e-02 |
| pTH10623 |  | scrt-1 | 42 | 1.59 | 1.7e-02 |
| V$HMX1\_01 |  | ceh-9 | 37 | 1.69 | 1.7e-02 |
| pTH10650 |  | nhr-153 | 26 | 2.02 | 1.7e-02 |
| ONEC2\_si |  | ceh-48 | 32 | 1.81 | 1.7e-02 |
| exd\_FlyReg\_FBgn0000611 |  | ceh-20 | 22 | 2.22 | 1.7e-02 |
| MA0085.1 |  | lag-1 | 32 | 1.81 | 1.7e-02 |
| pTH6482 |  | ceh-19 | 25 | 2.06 | 1.7e-02 |
| Hoxa6\_1040 |  | lin-39 | 36 | 1.70 | 1.8e-02 |
| Mf28 |  | elt-1 | 26 | 2.01 | 1.8e-02 |
| Etv3 |  | lin-1 | 44 | 1.54 | 1.8e-02 |
| pTH9082 |  | mab-23 | 38 | 1.65 | 1.9e-02 |
| GM12878\_PBX3\_HudsonAlpha |  | ceh-20 nfya-2 | 40 | 1.61 | 1.9e-02 |
| SOX10\_1 |  | sox-4 K11D2.4 | 19 | 2.40 | 1.9e-02 |
| pTH10041 |  | ztf-29 | 26 | 2.00 | 2.0e-02 |
| pTH2283 |  | odd-2 | 51 | 1.43 | 2.1e-02 |
| V$HOX13\_01 |  | lin-39 | 23 | 2.12 | 2.1e-02 |
| Barx1\_2877 |  | ceh-43 | 14 | 2.91 | 2.2e-02 |
| pTH6408 |  | irx-1 | 30 | 1.84 | 2.2e-02 |
| Tcf3\_3787 |  | pop-1 | 50 | 1.44 | 2.3e-02 |
| K562\_ETS1\_HudsonAlpha |  | odr-7 nhr-79 lin-1 | 22 | 2.16 | 2.3e-02 |
| Six4\_2860 |  | ceh-32 | 30 | 1.83 | 2.4e-02 |
| Irx3\_0920 |  | irx-1 | 25 | 2.00 | 2.4e-02 |
| MA0331.1 |  | unc-120 | 26 | 1.96 | 2.4e-02 |
| Zfp161\_2858 |  | pzf-1 | 33 | 1.74 | 2.5e-02 |
| Vax2\_3500 |  | C02F12.10 | 20 | 2.27 | 2.5e-02 |
| I$UBX\_01 |  | lin-39 | 22 | 2.14 | 2.5e-02 |
| PAX5\_si |  | pax-2 | 66 | 1.24 | 2.5e-02 |
| pTH5924 |  | nhr-255 (0.56) | 42 | 1.55 | 2.5e-02 |
| pTH9108 |  | daf-12 (0.63) | 32 | 1.76 | 2.5e-02 |
| MA0032.1 |  | let-381 | 27 | 1.92 | 2.6e-02 |
| V$CEBP\_01 |  | C48E7.11 | 48 | 1.46 | 2.6e-02 |
| pTH6508 |  | nhr-36 | 41 | 1.57 | 2.6e-02 |
| pTH5922 |  | ceh-24 (0.51) | 30 | 1.81 | 2.7e-02 |
| pTH8998 |  | mab-3 | 34 | 1.71 | 2.7e-02 |
| Hoxb8\_3780 |  | lin-39 | 38 | 1.62 | 2.7e-02 |
| E2F4\_1 |  | F49E12.6 | 28 | 1.87 | 2.8e-02 |
| pTH10808 |  | ztf-19 | 22 | 2.12 | 2.9e-02 |
| Dfd\_Cell\_FBgn0000439 |  | lin-39 | 20 | 2.23 | 2.9e-02 |
| GATA4\_1 |  | elt-1 | 43 | 1.52 | 2.9e-02 |
| pTH3120 |  | che-1 K11D2.4 | 19 | 2.29 | 3.0e-02 |
| pTH10811 |  | nhr-216 (0.52) | 26 | 1.93 | 3.0e-02 |
| pTH3064 |  | crh-1 | 38 | 1.61 | 3.0e-02 |
| pTH5018 |  | fos-1 (0.58) atf-7 | 19 | 2.29 | 3.0e-02 |
| pTH5877 |  | nhr-100 | 45 | 1.49 | 3.0e-02 |
| MA0146.2 |  | F58G1.2 | 23 | 2.05 | 3.2e-02 |
| N$SKN1\_02 |  | ceh-32 skn-1 | 34 | 1.68 | 3.3e-02 |
| Emx2\_3420 |  | ceh-2 | 22 | 2.08 | 3.4e-02 |
| MA0124.1 |  | ceh-24 (0.51) | 21 | 2.13 | 3.5e-02 |
| V$BRN2\_01 |  | ceh-18 (0.53) | 32 | 1.72 | 3.7e-02 |
| V$FOXO1\_02 |  | lin-31 daf-16 fkh-7 | 48 | 1.43 | 3.8e-02 |
| V$PAX2\_02 |  | pax-1 | 35 | 1.64 | 3.8e-02 |
| V$RFX1\_02 |  | daf-19 | 40 | 1.55 | 3.8e-02 |
| V$YY1\_01 |  | lsy-2 | 41 | 1.53 | 3.9e-02 |
| Evx1\_3952 |  | ceh-53 | 14 | 2.69 | 3.9e-02 |
| Srf\_3509 |  | unc-120 | 17 | 2.37 | 3.9e-02 |
| MA0262.1 |  | mab-3 | 48 | 1.43 | 4.0e-02 |
| pTH10034 |  | nhr-66 (0.57) | 39 | 1.56 | 4.0e-02 |
| Spt15 |  | tbp-1 | 18 | 2.29 | 4.0e-02 |
| ZBTB7A\_1 |  | ZC328.2 | 33 | 1.68 | 4.1e-02 |
| pTH6556 |  | odd-1 lim-6 | 29 | 1.78 | 4.1e-02 |
| MA0037.2 |  | elt-1 | 45 | 1.46 | 4.3e-02 |
| Nkx6-1\_2825 |  | cog-1 | 16 | 2.44 | 4.3e-02 |
| K562\_SP2\_HudsonAlpha |  | klf-2 | 36 | 1.61 | 4.4e-02 |
| pTH2684 |  | fos-1 (0.58) | 19 | 2.20 | 4.4e-02 |
| pTH9182 |  | tbx-39 | 22 | 2.03 | 4.4e-02 |
| Hnf4a\_2640 |  | nhr-62 | 6 | 5.64 | 4.5e-02 |
| pTH9080 |  | mnm-2 | 33 | 1.67 | 4.5e-02 |
| KLF8\_f1 |  | klf-1 | 17 | 2.33 | 4.6e-02 |
| Vax1\_3499 |  | C02F12.10 | 19 | 2.18 | 4.6e-02 |
| TLX1\_f1 |  | ceh-19 | 36 | 1.60 | 4.7e-02 |
| Hoxa7\_3750 |  | lin-39 | 26 | 1.85 | 4.7e-02 |
| Hoxa5\_3415 |  | lin-39 | 25 | 1.89 | 4.7e-02 |
| RFX1\_4537 |  | daf-19 | 30 | 1.73 | 4.8e-02 |
| Cdx2\_4272 |  | ceh-13 | 29 | 1.76 | 4.8e-02 |
| pTH9335 |  | mel-28 | 49 | 1.40 | 4.9e-02 |
| pTH10028 |  | nhr-204 | 34 | 1.63 | 5.0e-02 |

### Correlated (and anti-correlated) transcription factors

|  |  |
| --- | --- |
| **Transcription factor** | **Correlation** |
| npax-2 | 0.85 |
| F13H6.1 | 0.81 |
| moe-3 | 0.75 |
| hlh-33 | 0.73 |
| bed-3 | 0.72 |
| nhr-1 | 0.70 |
| nhr-3 | 0.70 |
| ets-5 | 0.68 |
| mdl-1 | 0.66 |
| lin-48 | 0.66 |
| nhr-181 | 0.65 |
| tag-97 | 0.65 |
| nhr-120 | 0.64 |
| nhr-31 | 0.64 |
| nhr-91 | 0.64 |
| atf-8 | 0.64 |
| ceh-8 | 0.63 |
| daf-12 | 0.63 |
| zfh-2 | 0.63 |
| nhr-14 | 0.63 |
| hlh-13 | 0.62 |
| nhr-40 | 0.62 |
| zip-1 | 0.61 |
| blmp-1 | 0.61 |
| madf-1 | 0.61 |
| ceh-91 | -0.50 |
| cid-1 | -0.51 |
| F27D4.4 | -0.51 |
| nhr-210 | -0.51 |
| lpd-2 | -0.51 |
| nhr-2 | -0.52 |
| Y82E9BR.1 | -0.53 |
| spe-44 | -0.54 |
| Y53F4B.3 | -0.54 |
| mex-6 | -0.55 |
| zip-7 | -0.55 |
| sup-35 | -0.55 |
| hmg-3 | -0.56 |
| hmg-5 | -0.57 |
| F49E8.2 | -0.57 |
| C16A3.4 | -0.59 |
| D2030.7 | -0.59 |
| cey-2 | -0.61 |
| madf-8 | -0.62 |
| Y48G9A.11 | -0.62 |
| ceh-40 | -0.63 |
| cep-1 | -0.64 |
| F23A7.6 | -0.65 |
| C28G1.4 | -0.68 |
| zip-8 | -0.70 |

### ChIP peaks enriched

none found
