# Supplementary material for: Overlapping cell population expression profiling and regulatory inference in C. elegans
Source: BMC Genomics. 2016 Feb 29;17:159. doi: 10.1186/s12864-016-2482-z (PMC4772325; doi:10.1186/s12864-016-2482-z)
Supplement: Additional file 13: — Web supplement. (DOC 21 kb) [file 12864_2016_2482_MOESM13_ESM.zip › sortWeb/clusters/hier.300.clusters/269.html]

Cluster 269 

## Cluster 269

### Expression

| cnd-1 rep. 1 | cnd-1 rep. 2 | cnd-1 rep. 3 | pha-4 rep. 1 | pha-4 rep. 2 | pha-4 rep. 3 | ceh-27 | ceh-36 | ceh-6 | F21D5.9 | mir-57 | mls-2 | pal-1 | pros-1 | ttx-3 | unc-130 | hlh-16 | irx-1 | ceh-6 (+) hlh-16 (+) | ceh-6 (+) hlh-16 (-) | ceh-6 (-) hlh-16 (+) | cnd-1 singlets | pha-4 singlets | 0 | 60 | 120 | 150 | 180 | 240 | 330 | 390 | 420 | 480 | 540 | 570 | 600 | 630 | 660 | NAME | Functional description |
| --- | --- | --- | --- | --- | --- | --- | --- | --- | --- | --- | --- | --- | --- | --- | --- | --- | --- | --- | --- | --- | --- | --- | --- | --- | --- | --- | --- | --- | --- | --- | --- | --- | --- | --- | --- | --- | --- | --- | --- |
|  |  |  |  |  |  |  |  |  |  |  |  |  |  |  |  |  |  |  |  |  |  |  |  |  |  |  |  |  |  |  |  |  |  |  |  |  |  | *dct-6* | DAF-16/FOXO Controlled, germline Tumor affecting |
|  |  |  |  |  |  |  |  |  |  |  |  |  |  |  |  |  |  |  |  |  |  |  |  |  |  |  |  |  |  |  |  |  |  |  |  |  |  | *tag-312* | Temporarily Assigned Gene name |
|  |  |  |  |  |  |  |  |  |  |  |  |  |  |  |  |  |  |  |  |  |  |  |  |  |  |  |  |  |  |  |  |  |  |  |  |  |  | ZK1053.3 |  |
|  |  |  |  |  |  |  |  |  |  |  |  |  |  |  |  |  |  |  |  |  |  |  |  |  |  |  |  |  |  |  |  |  |  |  |  |  |  | *pqn-25* | Prion-like-(Q/N-rich)-domain-bearing protein |
|  |  |  |  |  |  |  |  |  |  |  |  |  |  |  |  |  |  |  |  |  |  |  |  |  |  |  |  |  |  |  |  |  |  |  |  |  |  | D2085.5 |  |
|  |  |  |  |  |  |  |  |  |  |  |  |  |  |  |  |  |  |  |  |  |  |  |  |  |  |  |  |  |  |  |  |  |  |  |  |  |  | C05G5.1 |  |
|  |  |  |  |  |  |  |  |  |  |  |  |  |  |  |  |  |  |  |  |  |  |  |  |  |  |  |  |  |  |  |  |  |  |  |  |  |  | H01G02.4 |  |
|  |  |  |  |  |  |  |  |  |  |  |  |  |  |  |  |  |  |  |  |  |  |  |  |  |  |  |  |  |  |  |  |  |  |  |  |  |  | C29G2.1 |  |
|  |  |  |  |  |  |  |  |  |  |  |  |  |  |  |  |  |  |  |  |  |  |  |  |  |  |  |  |  |  |  |  |  |  |  |  |  |  | F23B12.1 |  |
|  |  |  |  |  |  |  |  |  |  |  |  |  |  |  |  |  |  |  |  |  |  |  |  |  |  |  |  |  |  |  |  |  |  |  |  |  |  | *trpa-2* | TRPA cation channel homolog |
|  |  |  |  |  |  |  |  |  |  |  |  |  |  |  |  |  |  |  |  |  |  |  |  |  |  |  |  |  |  |  |  |  |  |  |  |  |  | M05B5.3 |  |
|  |  |  |  |  |  |  |  |  |  |  |  |  |  |  |  |  |  |  |  |  |  |  |  |  |  |  |  |  |  |  |  |  |  |  |  |  |  | W03G11.4 |  |
|  |  |  |  |  |  |  |  |  |  |  |  |  |  |  |  |  |  |  |  |  |  |  |  |  |  |  |  |  |  |  |  |  |  |  |  |  |  | W04B5.1 |  |
|  |  |  |  |  |  |  |  |  |  |  |  |  |  |  |  |  |  |  |  |  |  |  |  |  |  |  |  |  |  |  |  |  |  |  |  |  |  | F58E6.8 |  |
|  |  |  |  |  |  |  |  |  |  |  |  |  |  |  |  |  |  |  |  |  |  |  |  |  |  |  |  |  |  |  |  |  |  |  |  |  |  | *srt-39* | Serpentine Receptor, class T |
|  |  |  |  |  |  |  |  |  |  |  |  |  |  |  |  |  |  |  |  |  |  |  |  |  |  |  |  |  |  |  |  |  |  |  |  |  |  | *ltd-1* | Lim and Transglutaminase Domain |
|  |  |  |  |  |  |  |  |  |  |  |  |  |  |  |  |  |  |  |  |  |  |  |  |  |  |  |  |  |  |  |  |  |  |  |  |  |  | *exc-7* | EXCretory canal abnormal |
|  |  |  |  |  |  |  |  |  |  |  |  |  |  |  |  |  |  |  |  |  |  |  |  |  |  |  |  |  |  |  |  |  |  |  |  |  |  | C08G9.2 |  |
|  |  |  |  |  |  |  |  |  |  |  |  |  |  |  |  |  |  |  |  |  |  |  |  |  |  |  |  |  |  |  |  |  |  |  |  |  |  | *sel-7* | Suppressor/Enhancer of Lin-12 |
|  |  |  |  |  |  |  |  |  |  |  |  |  |  |  |  |  |  |  |  |  |  |  |  |  |  |  |  |  |  |  |  |  |  |  |  |  |  | *nhr-183* | Nuclear Hormone Receptor family |
|  |  |  |  |  |  |  |  |  |  |  |  |  |  |  |  |  |  |  |  |  |  |  |  |  |  |  |  |  |  |  |  |  |  |  |  |  |  | T13C2.2 |  |
|  |  |  |  |  |  |  |  |  |  |  |  |  |  |  |  |  |  |  |  |  |  |  |  |  |  |  |  |  |  |  |  |  |  |  |  |  |  | *ifa-1* | Intermediate Filament, A |
|  |  |  |  |  |  |  |  |  |  |  |  |  |  |  |  |  |  |  |  |  |  |  |  |  |  |  |  |  |  |  |  |  |  |  |  |  |  | *hum-2* | Heavy chain, Unconventional Myosin |
|  |  |  |  |  |  |  |  |  |  |  |  |  |  |  |  |  |  |  |  |  |  |  |  |  |  |  |  |  |  |  |  |  |  |  |  |  |  | *cyp-29A2* | CYtochrome P450 family |
|  |  |  |  |  |  |  |  |  |  |  |  |  |  |  |  |  |  |  |  |  |  |  |  |  |  |  |  |  |  |  |  |  |  |  |  |  |  | *srg-5* | Serpentine Receptor, class G (gamma) |
|  |  |  |  |  |  |  |  |  |  |  |  |  |  |  |  |  |  |  |  |  |  |  |  |  |  |  |  |  |  |  |  |  |  |  |  |  |  | *nhr-194* | Nuclear Hormone Receptor family |
|  |  |  |  |  |  |  |  |  |  |  |  |  |  |  |  |  |  |  |  |  |  |  |  |  |  |  |  |  |  |  |  |  |  |  |  |  |  | *nhr-182* | Nuclear Hormone Receptor family |
|  |  |  |  |  |  |  |  |  |  |  |  |  |  |  |  |  |  |  |  |  |  |  |  |  |  |  |  |  |  |  |  |  |  |  |  |  |  | *ttm-5* | Toxin-regulated Targets of MAPK |
|  |  |  |  |  |  |  |  |  |  |  |  |  |  |  |  |  |  |  |  |  |  |  |  |  |  |  |  |  |  |  |  |  |  |  |  |  |  | *nhr-213* | Nuclear Hormone Receptor family |
|  |  |  |  |  |  |  |  |  |  |  |  |  |  |  |  |  |  |  |  |  |  |  |  |  |  |  |  |  |  |  |  |  |  |  |  |  |  | F13E9.14 |  |
|  |  |  |  |  |  |  |  |  |  |  |  |  |  |  |  |  |  |  |  |  |  |  |  |  |  |  |  |  |  |  |  |  |  |  |  |  |  | Y113G7B.11 |  |
|  |  |  |  |  |  |  |  |  |  |  |  |  |  |  |  |  |  |  |  |  |  |  |  |  |  |  |  |  |  |  |  |  |  |  |  |  |  | F57F5.3 |  |
|  |  |  |  |  |  |  |  |  |  |  |  |  |  |  |  |  |  |  |  |  |  |  |  |  |  |  |  |  |  |  |  |  |  |  |  |  |  | F16F9.1 |  |
|  |  |  |  |  |  |  |  |  |  |  |  |  |  |  |  |  |  |  |  |  |  |  |  |  |  |  |  |  |  |  |  |  |  |  |  |  |  | *uig-1* | UNC-112-Interacting Guanine nucleotide exchange factor |
|  |  |  |  |  |  |  |  |  |  |  |  |  |  |  |  |  |  |  |  |  |  |  |  |  |  |  |  |  |  |  |  |  |  |  |  |  |  | *magu-2* | MAGUK family |
|  |  |  |  |  |  |  |  |  |  |  |  |  |  |  |  |  |  |  |  |  |  |  |  |  |  |  |  |  |  |  |  |  |  |  |  |  |  | F59B10.4 |  |
|  |  |  |  |  |  |  |  |  |  |  |  |  |  |  |  |  |  |  |  |  |  |  |  |  |  |  |  |  |  |  |  |  |  |  |  |  |  | *nhr-151* | Nuclear Hormone Receptor family |
|  |  |  |  |  |  |  |  |  |  |  |  |  |  |  |  |  |  |  |  |  |  |  |  |  |  |  |  |  |  |  |  |  |  |  |  |  |  | *lec-4* | gaLECtin |
|  |  |  |  |  |  |  |  |  |  |  |  |  |  |  |  |  |  |  |  |  |  |  |  |  |  |  |  |  |  |  |  |  |  |  |  |  |  | *cutl-28* | CUTiclin-Like |
|  |  |  |  |  |  |  |  |  |  |  |  |  |  |  |  |  |  |  |  |  |  |  |  |  |  |  |  |  |  |  |  |  |  |  |  |  |  | *obr-2* | Oxysterol Binding protein (OSBP) Related |
|  |  |  |  |  |  |  |  |  |  |  |  |  |  |  |  |  |  |  |  |  |  |  |  |  |  |  |  |  |  |  |  |  |  |  |  |  |  | *lact-2* | beta-LACTamase domain containing |
|  |  |  |  |  |  |  |  |  |  |  |  |  |  |  |  |  |  |  |  |  |  |  |  |  |  |  |  |  |  |  |  |  |  |  |  |  |  | T05C1.1 |  |
|  |  |  |  |  |  |  |  |  |  |  |  |  |  |  |  |  |  |  |  |  |  |  |  |  |  |  |  |  |  |  |  |  |  |  |  |  |  | *nhr-55* | Nuclear Hormone Receptor family |
|  |  |  |  |  |  |  |  |  |  |  |  |  |  |  |  |  |  |  |  |  |  |  |  |  |  |  |  |  |  |  |  |  |  |  |  |  |  | F58E6.11 |  |
|  |  |  |  |  |  |  |  |  |  |  |  |  |  |  |  |  |  |  |  |  |  |  |  |  |  |  |  |  |  |  |  |  |  |  |  |  |  | C06A8.8 |  |
|  |  |  |  |  |  |  |  |  |  |  |  |  |  |  |  |  |  |  |  |  |  |  |  |  |  |  |  |  |  |  |  |  |  |  |  |  |  | K02E10.6 |  |
|  |  |  |  |  |  |  |  |  |  |  |  |  |  |  |  |  |  |  |  |  |  |  |  |  |  |  |  |  |  |  |  |  |  |  |  |  |  | *syx-2* | SYntaXin |
|  |  |  |  |  |  |  |  |  |  |  |  |  |  |  |  |  |  |  |  |  |  |  |  |  |  |  |  |  |  |  |  |  |  |  |  |  |  | R07B7.9 |  |
|  |  |  |  |  |  |  |  |  |  |  |  |  |  |  |  |  |  |  |  |  |  |  |  |  |  |  |  |  |  |  |  |  |  |  |  |  |  | *nhr-129* | Nuclear Hormone Receptor family |
|  |  |  |  |  |  |  |  |  |  |  |  |  |  |  |  |  |  |  |  |  |  |  |  |  |  |  |  |  |  |  |  |  |  |  |  |  |  | K02E11.4 |  |
|  |  |  |  |  |  |  |  |  |  |  |  |  |  |  |  |  |  |  |  |  |  |  |  |  |  |  |  |  |  |  |  |  |  |  |  |  |  | F12D9.16 |  |
|  |  |  |  |  |  |  |  |  |  |  |  |  |  |  |  |  |  |  |  |  |  |  |  |  |  |  |  |  |  |  |  |  |  |  |  |  |  | C06A6.12 |  |
|  |  |  |  |  |  |  |  |  |  |  |  |  |  |  |  |  |  |  |  |  |  |  |  |  |  |  |  |  |  |  |  |  |  |  |  |  |  | *srz-16* | Serpentine Receptor, class Z |
|  |  |  |  |  |  |  |  |  |  |  |  |  |  |  |  |  |  |  |  |  |  |  |  |  |  |  |  |  |  |  |  |  |  |  |  |  |  | H03E18.4 |  |
|  |  |  |  |  |  |  |  |  |  |  |  |  |  |  |  |  |  |  |  |  |  |  |  |  |  |  |  |  |  |  |  |  |  |  |  |  |  | *dmd-11* | DM (Doublesex/MAB-3) Domain family |
|  |  |  |  |  |  |  |  |  |  |  |  |  |  |  |  |  |  |  |  |  |  |  |  |  |  |  |  |  |  |  |  |  |  |  |  |  |  | T08H4.2 |  |
|  |  |  |  |  |  |  |  |  |  |  |  |  |  |  |  |  |  |  |  |  |  |  |  |  |  |  |  |  |  |  |  |  |  |  |  |  |  | F11D5.32 |  |
|  |  |  |  |  |  |  |  |  |  |  |  |  |  |  |  |  |  |  |  |  |  |  |  |  |  |  |  |  |  |  |  |  |  |  |  |  |  | F21F8.5 |  |
|  |  |  |  |  |  |  |  |  |  |  |  |  |  |  |  |  |  |  |  |  |  |  |  |  |  |  |  |  |  |  |  |  |  |  |  |  |  | F28A12.9 |  |
|  |  |  |  |  |  |  |  |  |  |  |  |  |  |  |  |  |  |  |  |  |  |  |  |  |  |  |  |  |  |  |  |  |  |  |  |  |  | Y75B8A.39 |  |
|  |  |  |  |  |  |  |  |  |  |  |  |  |  |  |  |  |  |  |  |  |  |  |  |  |  |  |  |  |  |  |  |  |  |  |  |  |  | C07G2.4 |  |
|  |  |  |  |  |  |  |  |  |  |  |  |  |  |  |  |  |  |  |  |  |  |  |  |  |  |  |  |  |  |  |  |  |  |  |  |  |  | F31E8.18 |  |
|  |  |  |  |  |  |  |  |  |  |  |  |  |  |  |  |  |  |  |  |  |  |  |  |  |  |  |  |  |  |  |  |  |  |  |  |  |  | *srbc-55* | Serpentine Receptor, class BC (class B-like) |
|  |  |  |  |  |  |  |  |  |  |  |  |  |  |  |  |  |  |  |  |  |  |  |  |  |  |  |  |  |  |  |  |  |  |  |  |  |  | *dct-5* | DAF-16/FOXO Controlled, germline Tumor affecting |
|  |  |  |  |  |  |  |  |  |  |  |  |  |  |  |  |  |  |  |  |  |  |  |  |  |  |  |  |  |  |  |  |  |  |  |  |  |  | *srxa-2* | Serpentine Receptor, class XA |

### Phenotypes enriched

none found

### Anatomy terms enriched

none found

### GO terms enriched

|  |  |  |
| --- | --- | --- |
| **GO term** | **Number of genes** | **FDR-corrected p-value** |
| steroid hormone mediated signaling pathway | 6 | 0.024 |
| response to steroid hormone | 6 | 0.024 |
| cellular response to lipid | 6 | 0.024 |
| steroid hormone receptor activity | 6 | 0.024 |
| cellular response to organic cyclic compound | 6 | 0.027 |
| cellular response to hormone stimulus | 6 | 0.030 |
| response to endogenous stimulus | 6 | 0.045 |

### Expression clusters enriched

|  |  |  |  |
| --- | --- | --- | --- |
| **Group name** | **Number in cluster** | **Enrichment** | **FDR corrected p** |
| Genes depleted in muscle cells (0hr muscle dataset). Dissociated myo-3::GFP embryos were cultured for 0 hours before FACS sorting. | 21 | 2.97 | 0.0010 |
| Total muscle depleted genes (complete list of non-overlapping genes from the 0hr and 24hr muscle depleted datasets). | 23 | 2.23 | 0.0223 |
| C-lineage related expression profile. WBPaper00025032:cluster\_16 | 4 | 14.62 | 0.0362 |

### Motifs enriched

|  |  |  |  |  |  |
| --- | --- | --- | --- | --- | --- |
| **Motif** | **Logo** | **Possible orthologs** | **Number of motifs in cluster** | **Enrichment** | **FDR corrected p** |
| MA0546.1 |  | let-381 lin-31 | 33 | 3.01 | 2.7e-07 |
| Mv129 |  | ceh-18 ceh-6 sox-4 tbp-1 | 37 | 2.25 | 2.4e-05 |
| Foxk1\_2 |  | pha-4 (0.62) fkh-7 let-381 daf-16 lin-31 fkh-10 fkh-8 | 29 | 2.69 | 3.8e-05 |
| Tcf7\_0950 |  | pop-1 | 38 | 2.12 | 5.8e-05 |
| pTH2673 |  | let-381 lin-31 fkh-10 C34D1.1 | 21 | 3.51 | 6.1e-05 |
| pTH9250 |  | dmd-3 C34D1.1 | 34 | 2.31 | 6.1e-05 |
| pTH10823 |  | B0310.2 | 41 | 1.96 | 9.4e-05 |
| MA0028.1 |  | lin-1 nhr-19 C24A1.2 | 28 | 2.60 | 1.2e-04 |
| pTH10042 |  | daf-12 (0.68) nhr-5 | 31 | 2.39 | 1.3e-04 |
| V$FREAC2\_01 |  | fkh-7 let-381 daf-16 lin-31 fkh-10 fkh-8 | 18 | 3.87 | 1.3e-04 |
| Tcf1\_2666 |  | hmbx-1 | 35 | 2.16 | 1.5e-04 |
| V$CDPCR3\_01 |  | ceh-48 | 14 | 4.98 | 1.6e-04 |
| Barhl1\_2 |  | lin-39 (-0.59) alr-1 lim-7 ceh-1 ceh-9 ceh-43 ceh-31 ceh-16 | 33 | 2.23 | 2.1e-04 |
| pTH9261 |  | pax-3 (-0.6) lin-48 dmd-3 | 33 | 2.22 | 2.3e-04 |
| Pou2f2\_1 |  | ceh-18 unc-86 | 30 | 2.38 | 2.3e-04 |
| DLX6\_1 |  | ceh-2 (0.74) ceh-53 (0.66) lin-39 (-0.59) ceh-45 alr-1 lim-7 ceh-1 pal-1 lim-6 ceh-12 ceh-23 egl-5 ceh-36 ceh-10 ceh-43 cog-1 ceh-14 ceh-16 C07E3.6 | 30 | 2.37 | 2.6e-04 |
| pTH5661 |  | ceh-19 (0.8) lin-39 (-0.59) ceh-8 alr-1 lim-7 ceh-1 ceh-9 ceh-43 ceh-30 cog-1 ceh-31 ceh-24 | 28 | 2.49 | 2.6e-04 |
| NEUROG2\_2 |  | hlh-32 hlh-15 ngn-1 | 38 | 1.98 | 2.9e-04 |
| Foxk1\_1 |  | nhr-213 (0.85) lin-31 | 41 | 1.87 | 3.1e-04 |
| ISL1\_f1 |  | lin-39 (-0.59) lim-7 cfi-1 | 17 | 3.75 | 3.6e-04 |
| Prrx2\_3072 |  | ceh-53 (0.66) lin-39 (-0.59) ceh-45 alr-1 ceh-1 ceh-12 ceh-43 | 32 | 2.21 | 3.7e-04 |
| Spdef |  | lin-1 | 41 | 1.85 | 3.7e-04 |
| pTH6591 |  | lin-31 | 27 | 2.50 | 4.0e-04 |
| TCF7L1\_1 |  | nhr-100 sox-4 pop-1 | 50 | 1.60 | 4.3e-04 |
| FEV\_1 |  | lin-1 C24A1.2 | 28 | 2.42 | 4.4e-04 |
| BARHL2\_4 |  | ceh-31 | 21 | 3.03 | 4.6e-04 |
| V$FOXJ2\_02 |  | elt-1 (-0.72) lin-31 | 19 | 3.27 | 5.4e-04 |
| V$XFD3\_01 |  | ceh-20 (-0.68) let-381 | 23 | 2.78 | 5.4e-04 |
| ETS2\_f1 |  | lin-1 C24A1.2 | 42 | 1.79 | 5.6e-04 |
| PURA\_f1 |  | plp-2 | 41 | 1.82 | 5.8e-04 |
| pTH9174 |  | nhr-28 (0.6) odr-7 | 34 | 2.06 | 6.6e-04 |
| pTH3467 |  | nhr-213 (0.85) nhr-71 (0.62) nhr-10 nhr-2 nhr-6 nhr-68 Y67D8A.3 | 39 | 1.87 | 7.2e-04 |
| MA0150.2 |  | jun-1 sknr-1 | 26 | 2.48 | 7.3e-04 |
| pTH3477 |  | daf-16 | 24 | 2.62 | 7.9e-04 |
| pTH9080 |  | mnm-2 | 22 | 2.80 | 8.0e-04 |
| Nkx1-2\_3214 |  | ceh-30 | 22 | 2.80 | 8.2e-04 |
| pTH10717 |  | lsy-2 syd-9 lsl-1 | 24 | 2.61 | 8.2e-04 |
| YMR043W\_831 |  | unc-120 | 10 | 6.23 | 8.4e-04 |
| Fli1 |  | lin-1 F19F10.1 C24A1.2 | 27 | 2.38 | 8.9e-04 |
| pTH9137 |  | nhr-65 (0.65) | 39 | 1.85 | 9.1e-04 |
| Barx1\_2877 |  | ceh-43 | 19 | 3.13 | 9.2e-04 |
| pTH6449 |  | ceh-43 | 15 | 3.86 | 9.7e-04 |
| Hnf4a\_2640 |  | nhr-62 (0.75) | 36 | 1.94 | 9.9e-04 |
| Barx2\_3447 |  | ceh-43 | 15 | 3.84 | 1.0e-03 |
| pTH6747 |  | nhr-213 (0.85) nhr-10 nhr-2 nhr-19 | 11 | 5.37 | 1.0e-03 |
| V$CDXA\_01 |  | ceh-13 (-0.57) php-3 | 14 | 4.10 | 1.1e-03 |
| pTH9242 |  | mel-28 | 40 | 1.80 | 1.1e-03 |
| Elf3\_3876 |  | C24A1.2 | 30 | 2.18 | 1.1e-03 |
| Tcf1\_2666 |  | hmbx-1 | 33 | 2.04 | 1.2e-03 |
| MCR\_f1 |  | nhr-255 | 44 | 1.69 | 1.2e-03 |
| EGR2\_si |  | ZC328.2 (0.63) klf-1 | 28 | 2.27 | 1.2e-03 |
| NR2F6\_f1 |  | nhr-62 (0.75) nhr-239 nhr-2 | 41 | 1.76 | 1.2e-03 |
| TBX20\_1 |  | mab-9 tbx-39 tbx-38 tbx-42 tbx-43 | 15 | 3.76 | 1.3e-03 |
| pTH8587 |  | daf-19 | 37 | 1.88 | 1.3e-03 |
| pTH9901 |  | lin-39 (-0.59) pal-1 php-3 ceh-24 D1005.3 T27F2.4 | 31 | 2.11 | 1.4e-03 |
| Ubx\_FlyReg\_FBgn0003944 |  | lin-39 (-0.59) ceh-45 eyg-1 alr-1 ceh-1 | 26 | 2.38 | 1.4e-03 |
| pTH3220 |  | daf-12 (0.68) Y5F2A.4 ztf-3 ceh-9 | 25 | 2.44 | 1.4e-03 |
| MA0027.1 |  | ceh-16 | 40 | 1.78 | 1.5e-03 |
| Mw137 |  | blmp-1 | 29 | 2.19 | 1.6e-03 |
| V$RFX1\_02 |  | F52B5.7 daf-19 | 24 | 2.50 | 1.6e-03 |
| CG9895\_SOLEXA\_5\_FBgn0034810 |  | klf-2 klf-1 | 26 | 2.35 | 1.6e-03 |
| pTH9159 |  | atf-6 | 13 | 4.19 | 1.7e-03 |
| pTH9188 |  | dmd-5 ceh-32 | 15 | 3.64 | 1.8e-03 |
| ETV4\_f1 |  | lin-1 C24A1.2 | 25 | 2.40 | 1.8e-03 |
| pTH6497 |  | lin-31 | 26 | 2.33 | 1.9e-03 |
| MA0386.1 |  | K11D2.4 tbp-1 | 22 | 2.61 | 2.0e-03 |
| Cdx2\_4272 |  | ceh-13 (-0.57) | 19 | 2.92 | 2.1e-03 |
| pTH9282 |  | attf-1 | 31 | 2.05 | 2.2e-03 |
| SOX2\_4 |  | grh-1 (0.69) dmd-4 sox-4 | 29 | 2.14 | 2.2e-03 |
| Exex\_Cell\_FBgn0041156 |  | lin-39 (-0.59) ceh-18 pal-1 ceh-12 ceh-43 | 24 | 2.43 | 2.3e-03 |
| pTH6589 |  | nhr-213 (0.85) nhr-15 nhr-2 | 18 | 3.01 | 2.4e-03 |
| pTH9155 |  | lin-48 D1081.8 | 29 | 2.13 | 2.4e-03 |
| Bsx\_3483 |  | ceh-31 | 26 | 2.29 | 2.5e-03 |
| pTH9076 |  | C01G12.1 | 25 | 2.35 | 2.5e-03 |
| pTH10638 |  | dmd-3 C34D1.1 | 36 | 1.85 | 2.5e-03 |
| V$CEBP\_01 |  | C48E7.11 | 46 | 1.59 | 2.5e-03 |
| Spdef\_0905 |  | lin-1 | 33 | 1.95 | 2.7e-03 |
| Hoxa11\_2218 |  | php-3 | 27 | 2.22 | 2.7e-03 |
| pTH5778 |  | egl-5 | 25 | 2.33 | 2.8e-03 |
| pTH10779 |  | nhr-182 (0.89) | 21 | 2.63 | 2.8e-03 |
| I$KR\_01 |  | B0310.2 | 23 | 2.46 | 2.9e-03 |
| Elf3 |  | C24A1.2 | 43 | 1.65 | 3.0e-03 |
| pTH6641 |  | lin-31 | 24 | 2.38 | 3.0e-03 |
| NR2F6\_1 |  | nhr-62 (0.75) nhr-2 nhr-19 | 60 | 1.30 | 3.0e-03 |
| CG7386\_F10-12\_SANGER\_5\_FBgn0035691 |  | F56D1.1 sox-4 | 29 | 2.09 | 3.2e-03 |
| Etv3 |  | lin-1 | 28 | 2.14 | 3.2e-03 |
| pTH6447 |  | ceh-19 (0.8) | 27 | 2.19 | 3.3e-03 |
| TBX2\_f1 |  | tbx-39 ztf-6 | 19 | 2.80 | 3.3e-03 |
| amos\_da\_SANGER\_10\_FBgn0003270 |  | hlh-32 hlh-12 hlh-15 hlh-8 | 40 | 1.71 | 3.3e-03 |
| Elf5 |  | C24A1.2 | 42 | 1.66 | 3.3e-03 |
| pTH6562 |  | ceh-5 | 26 | 2.23 | 3.5e-03 |
| V$AREB6\_02 |  | ztf-6 | 24 | 2.36 | 3.5e-03 |
| HXA9\_f1 |  | lin-39 (-0.59) lin-32 (-0.51) ceh-32 | 17 | 3.04 | 3.5e-03 |
| MA0078.1 |  | sox-4 pop-1 gei-3 C05C9.3 | 33 | 1.92 | 3.5e-03 |
| ZNF75A\_1 |  | ztf-3 F26F4.8 | 30 | 2.03 | 3.6e-03 |
| Hmbox1\_2674 |  | hmbx-1 | 18 | 2.89 | 3.7e-03 |
| pTH10028 |  | nhr-204 (0.7) | 29 | 2.07 | 3.7e-03 |
| Isl2\_3430 |  | alr-1 lim-7 | 26 | 2.22 | 3.9e-03 |
| pTH2846 |  | lin-31 | 37 | 1.78 | 3.9e-03 |
| pTH10647 |  | nhr-232 | 26 | 2.21 | 4.0e-03 |
| pTH9298 |  | crh-1 attf-1 | 30 | 2.02 | 4.0e-03 |
| Sox15\_3457 |  | sox-4 | 44 | 1.60 | 4.1e-03 |
| V$OCT1\_06 |  | ceh-18 ztf-9 | 31 | 1.98 | 4.1e-03 |
| VENTX\_1 |  | pha-2 | 27 | 2.15 | 4.2e-03 |
| Clk\_cyc\_SANGER\_5\_FBgn0023094 |  | hlh-26 aha-1 C27D6.4 | 35 | 1.83 | 4.2e-03 |
| CG2052\_SANGER\_2.5\_FBgn0039905 |  | lin-29 (0.58) fkh-7 | 18 | 2.85 | 4.2e-03 |
| pTH2283 |  | odd-2 | 27 | 2.15 | 4.3e-03 |
| MA0500.1 |  | hlh-11 hlh-1 hlh-15 | 37 | 1.77 | 4.4e-03 |
| Pou3f3\_3235 |  | ceh-6 | 35 | 1.82 | 4.6e-03 |
| pTH6143 |  | lin-39 (-0.59) pal-1 php-3 ceh-24 | 23 | 2.37 | 4.8e-03 |
| Meis2\_1 |  | ceh-20 (-0.68) ceh-32 | 33 | 1.88 | 4.9e-03 |
| pTH3041 |  | atf-2 | 22 | 2.43 | 4.9e-03 |
| pTH9900 |  | C46E10.8 C33G8.2 | 29 | 2.03 | 5.0e-03 |
| pTH9245 |  | ceh-18 | 25 | 2.23 | 5.1e-03 |
| NR1D1\_f1 |  | nhr-213 (0.85) nhr-118 | 28 | 2.08 | 5.1e-03 |
| Osr2\_1727 |  | odd-1 | 35 | 1.81 | 5.1e-03 |
| MA0079.3 |  | klf-2 klf-1 | 39 | 1.70 | 5.2e-03 |
| HXC6\_f1 |  | lin-39 (-0.59) | 34 | 1.84 | 5.4e-03 |
| pTH9125 |  | sox-4 egl-13 | 36 | 1.78 | 5.6e-03 |
| V$DELTAEF1\_01 |  | ztf-6 hlh-2 | 35 | 1.80 | 5.8e-03 |
| tgo\_sima\_SANGER\_5\_FBgn0015014 |  | hlh-30 (0.51) aha-1 | 34 | 1.83 | 5.8e-03 |
| Lmx1a\_2238 |  | lim-6 ceh-16 | 26 | 2.15 | 5.9e-03 |
| pTH9072 |  | klf-1 sptf-3 | 37 | 1.74 | 6.0e-03 |
| MA0547.1 |  | ceh-2 (0.74) skn-1 | 27 | 2.10 | 6.0e-03 |
| pTH10777 |  | dmd-3 | 29 | 2.01 | 6.1e-03 |
| pTH3037 |  | hlh-15 | 34 | 1.83 | 6.1e-03 |
| MSX1\_1 |  | lin-39 (-0.59) ceh-1 ceh-43 ceh-31 | 28 | 2.05 | 6.2e-03 |
| pTH9219 |  | xbp-1 C01B12.2 | 30 | 1.96 | 6.3e-03 |
| Irx3\_1 |  | irx-1 | 29 | 2.00 | 6.3e-03 |
| pTH10714 |  | nhr-84 (0.59) nhr-142 | 23 | 2.32 | 6.3e-03 |
| HXD10\_f1 |  | php-3 | 34 | 1.82 | 6.4e-03 |
| NFIA\_1 |  | nfi-1 | 20 | 2.54 | 6.6e-03 |
| MA0264.1 |  | dsc-1 | 41 | 1.63 | 6.9e-03 |
| pTH9085 |  | nhr-42 (0.74) | 31 | 1.91 | 6.9e-03 |
| pTH9974 |  | hlh-16 ngn-1 | 63 | 1.22 | 7.2e-03 |
| V$PAX6\_01 |  | pax-3 (-0.6) pax-2 | 39 | 1.67 | 7.2e-03 |
| MA0046.1 |  | ceh-53 (0.66) lin-39 (-0.59) hmbx-1 let-381 lin-31 | 16 | 2.96 | 7.3e-03 |
| Six6\_2267 |  | ceh-34 (0.52) ceh-32 | 38 | 1.70 | 7.3e-03 |
| pTH9254 |  | mel-28 | 38 | 1.69 | 7.7e-03 |
| pTH9951 |  | mex-6 pal-1 | 32 | 1.87 | 7.7e-03 |
| Cdx1\_2245 |  | ceh-13 (-0.57) | 17 | 2.80 | 7.9e-03 |
| pTH7875 |  | mel-28 | 22 | 2.34 | 8.0e-03 |
| pTH10808 |  | ztf-19 | 26 | 2.10 | 8.1e-03 |
| En1\_3123 |  | ceh-16 | 28 | 2.01 | 8.3e-03 |
| pTH9709 |  | die-1 | 40 | 1.64 | 8.5e-03 |
| pTH9353 |  | ceh-51 | 23 | 2.26 | 8.6e-03 |
| ELF3\_f1 |  | K02D7.2 unc-120 C24A1.2 | 43 | 1.57 | 8.8e-03 |
| pTH5561 |  | nhr-239 | 39 | 1.65 | 9.2e-03 |
| Mv90 |  | mef-2 (0.63) | 21 | 2.38 | 9.3e-03 |
| V$CETS1P54\_02 |  | C52B9.2 | 19 | 2.53 | 1.0e-02 |
| pTH10623 |  | scrt-1 | 37 | 1.69 | 1.0e-02 |
| Hoxd13\_2356 |  | pal-1 | 31 | 1.87 | 1.0e-02 |
| pTH10645 |  | nhr-7 (0.78) nhr-100 | 32 | 1.83 | 1.1e-02 |
| V$MYB\_Q6 |  | D1081.8 | 35 | 1.74 | 1.1e-02 |
| GRHL1\_1 |  | grh-1 (0.69) | 19 | 2.50 | 1.1e-02 |
| pTH9198 |  | dmd-3 | 18 | 2.59 | 1.2e-02 |
| pTH6429 |  | F13H6.1 (0.6) nhr-2 nhr-86 nhr-177 | 26 | 2.05 | 1.2e-02 |
| Dlx3\_1030 |  | ceh-43 | 25 | 2.09 | 1.2e-02 |
| Hmx3\_3490 |  | ceh-9 | 15 | 2.95 | 1.2e-02 |
| MA0007.2 |  | nhr-255 npax-1 | 57 | 1.31 | 1.2e-02 |
| Elf4 |  | C24A1.2 | 24 | 2.14 | 1.2e-02 |
| Nkx2-6\_3437 |  | dsc-1 | 37 | 1.68 | 1.2e-02 |
| Dlx2\_2273 |  | ceh-43 | 25 | 2.08 | 1.2e-02 |
| Vsx1\_1728 |  | alr-1 | 25 | 2.08 | 1.3e-02 |
| pTH5914 |  | attf-1 | 13 | 3.27 | 1.3e-02 |
| V$CDP\_01 |  | ceh-48 | 16 | 2.78 | 1.3e-02 |
| FOXO6\_3 |  | ZC328.2 (0.63) daf-16 | 37 | 1.67 | 1.3e-02 |
| Caup\_SOLEXA\_FBgn0015919 |  | irx-1 | 38 | 1.64 | 1.4e-02 |
| CG4854\_SANGER\_10\_FBgn0038766 |  | K11D2.4 | 29 | 1.90 | 1.4e-02 |
| pTH9237 |  | mel-28 | 35 | 1.71 | 1.4e-02 |
| MA0173.1 |  | hlh-32 irx-1 | 28 | 1.93 | 1.4e-02 |
| pTH9884 |  | tbx-39 | 35 | 1.71 | 1.5e-02 |
| V$LYF1\_01 |  | F26F4.8 mbr-1 | 26 | 2.01 | 1.5e-02 |
| Nkx1-1\_3856 |  | ceh-30 | 23 | 2.15 | 1.5e-02 |
| V$AREB6\_04 |  | ztf-6 C34D1.1 | 40 | 1.59 | 1.5e-02 |
| V$FREAC7\_01 |  | lin-31 | 32 | 1.78 | 1.6e-02 |
| pTH8998 |  | mab-3 | 37 | 1.65 | 1.6e-02 |
| pTH10034 |  | nhr-66 (0.89) | 31 | 1.81 | 1.6e-02 |
| Emx2\_3420 |  | ceh-2 (0.74) | 23 | 2.14 | 1.6e-02 |
| I$CROC\_01 |  | let-381 mel-28 | 30 | 1.84 | 1.7e-02 |
| V$EN1\_01 |  | ceh-16 | 35 | 1.69 | 1.7e-02 |
| CEBPE\_f1 |  | C48E7.11 | 40 | 1.58 | 1.7e-02 |
| Hoxa7\_3750 |  | lin-39 (-0.59) | 25 | 2.03 | 1.7e-02 |
| pTH6569 |  | ceh-43 | 22 | 2.18 | 1.8e-02 |
| Hlf\_1 |  | ces-2 C01B12.2 | 7 | 5.81 | 1.8e-02 |
| Caup\_Cell\_FBgn0015919 |  | daf-16 irx-1 | 42 | 1.54 | 1.8e-02 |
| Dlx1\_1741 |  | ceh-43 | 25 | 2.02 | 1.8e-02 |
| Hoxb9\_3413 |  | pal-1 ceh-24 | 11 | 3.60 | 1.8e-02 |
| pTH9924 |  | nhr-46 (0.67) | 28 | 1.90 | 1.8e-02 |
| scrt\_SOLEXA\_2.5\_2\_FBgn0004880 |  | hlh-1 ces-1 hlh-15 | 34 | 1.71 | 1.9e-02 |
| Hoxa10\_2318 |  | ceh-24 | 17 | 2.55 | 1.9e-02 |
| CENPB\_1 |  | F21D5.4 | 29 | 1.85 | 2.0e-02 |
| MA0543.1 |  | eor-1 daf-8 | 39 | 1.59 | 2.0e-02 |
| pTH9381 |  | ceh-18 | 36 | 1.65 | 2.0e-02 |
| TLX1\_f1 |  | ceh-19 (0.8) | 30 | 1.82 | 2.0e-02 |
| Gmeb1\_1745 |  | attf-1 | 29 | 1.84 | 2.1e-02 |
| V$GATA1\_01 |  | elt-1 (-0.72) | 31 | 1.78 | 2.1e-02 |
| pTH6482 |  | ceh-19 (0.8) | 24 | 2.05 | 2.1e-02 |
| PBX1\_do |  | ceh-20 (-0.68) | 37 | 1.62 | 2.2e-02 |
| HeLa-S3\_ZNF274\_UCD |  | C28G1.4 | 42 | 1.52 | 2.2e-02 |
| pTH5919 |  | irx-1 | 53 | 1.34 | 2.2e-02 |
| V$NCX\_01 |  | ceh-19 (0.8) | 23 | 2.08 | 2.3e-02 |
| Lhx1\_2240 |  | lim-7 | 25 | 1.98 | 2.3e-02 |
| NR4A2\_1 |  | nhr-6 | 27 | 1.90 | 2.3e-02 |
| pTH8566 |  | lin-54 | 30 | 1.80 | 2.4e-02 |
| pTH10816 |  | dmd-6 | 42 | 1.51 | 2.4e-02 |
| SMAD3\_1 |  | daf-8 | 5 | 8.49 | 2.5e-02 |
| MA0488.1 |  | crh-1 | 16 | 2.58 | 2.5e-02 |
| pTH6268 |  | ceh-2 (0.74) | 18 | 2.38 | 2.5e-02 |
| V$OCT1\_03 |  | ceh-18 | 32 | 1.73 | 2.5e-02 |
| ELK3\_f1 |  | lin-1 | 21 | 2.16 | 2.6e-02 |
| Hoxa9\_2622 |  | lin-39 (-0.59) | 27 | 1.88 | 2.6e-02 |
| Mw160 |  | nhr-68 | 33 | 1.70 | 2.6e-02 |
| CG8765\_SANGER\_5\_FBgn0036900 |  | H20J04.3 | 28 | 1.84 | 2.7e-02 |
| HepG2\_SRF\_HudsonAlpha |  | unc-120 | 22 | 2.10 | 2.7e-02 |
| pTH6106 |  | nhr-182 (0.89) | 8 | 4.56 | 2.7e-02 |
| N$SKN1\_02 |  | skn-1 | 31 | 1.75 | 2.7e-02 |
| pTH9135 |  | pop-1 | 32 | 1.72 | 2.8e-02 |
| V$CMYB\_01 |  | D1081.8 | 52 | 1.34 | 2.8e-02 |
| Hoxb6\_3428 |  | lin-39 (-0.59) | 11 | 3.36 | 2.8e-02 |
| I$DRI\_01 |  | cfi-1 | 14 | 2.77 | 3.0e-02 |
| V$BRN2\_01 |  | ceh-18 | 31 | 1.74 | 3.0e-02 |
| V$CEBPA\_01 |  | C48E7.11 | 23 | 2.03 | 3.0e-02 |
| MA0468.1 |  | ZC204.2 | 15 | 2.63 | 3.0e-02 |
| pTH9220 |  | mbr-1 | 19 | 2.26 | 3.0e-02 |
| pTH9165 |  | ztf-27 (0.77) | 36 | 1.61 | 3.1e-02 |
| pTH10041 |  | ztf-29 | 36 | 1.61 | 3.1e-02 |
| disco-r-Cl1\_SANGER\_5\_FBgn0042650 |  | F55C5.11 | 21 | 2.12 | 3.2e-02 |
| pTH5812 |  | ceh-14 | 28 | 1.82 | 3.2e-02 |
| Hoxb5\_3122 |  | lin-39 (-0.59) | 23 | 2.02 | 3.2e-02 |
| pTH9215 |  | C34D1.1 | 16 | 2.50 | 3.2e-02 |
| pTH2936 |  | nhr-239 | 22 | 2.07 | 3.2e-02 |
| pTH9244 |  | tbx-39 | 17 | 2.40 | 3.3e-02 |
| V$TATA\_C |  | tbp-1 | 27 | 1.84 | 3.5e-02 |
| V$IK2\_01 |  | F26F4.8 | 36 | 1.60 | 3.5e-02 |
| V$YY1\_01 |  | lsy-2 | 41 | 1.50 | 3.5e-02 |
| V$TAXCREB\_02 |  | crh-1 | 7 | 4.97 | 3.6e-02 |
| I$DFD\_01 |  | lin-39 (-0.59) | 28 | 1.80 | 3.6e-02 |
| MA0540.1 |  | dpy-27 | 14 | 2.69 | 3.7e-02 |
| PAX5\_si |  | pax-2 | 31 | 1.71 | 3.7e-02 |
| pTH6486 |  | nhr-145 | 31 | 1.71 | 3.8e-02 |
| pTH1001 |  | dnj-17 | 21 | 2.08 | 3.9e-02 |
| pTH9059 |  | ztf-28 (-0.55) | 12 | 2.98 | 3.9e-02 |
| Hoxa5\_3415 |  | lin-39 (-0.59) | 23 | 1.98 | 3.9e-02 |
| pTH10805 |  | ztf-16 (0.73) | 9 | 3.78 | 3.9e-02 |
| PAX8\_f1 |  | pax-2 | 22 | 2.02 | 4.0e-02 |
| FLI1\_f1 |  | lin-1 | 12 | 2.96 | 4.1e-02 |
| Hoxd9\_1 |  | lin-39 (-0.59) | 27 | 1.81 | 4.1e-02 |
| pTH6423 |  | pha-2 | 20 | 2.12 | 4.2e-02 |
| pTH9907 |  | nhr-34 (0.57) | 25 | 1.88 | 4.2e-02 |
| Hoxb3\_1720 |  | lin-39 (-0.59) | 23 | 1.96 | 4.3e-02 |
| Sox17\_2837 |  | sox-4 | 33 | 1.64 | 4.3e-02 |
| pTH9163 |  | nhr-3 (0.52) | 15 | 2.51 | 4.3e-02 |
| Vax2\_3500 |  | C02F12.10 | 23 | 1.96 | 4.4e-02 |
| pTH5078 |  | ces-2 | 22 | 2.00 | 4.5e-02 |
| Sox1\_2631 |  | sox-4 | 30 | 1.71 | 4.5e-02 |
| TBX3\_f1 |  | tbx-39 | 40 | 1.50 | 4.5e-02 |
| K562\_GATA2\_UChicago |  | elt-1 (-0.72) | 26 | 1.83 | 4.5e-02 |
| pTH6478 |  | lim-7 | 29 | 1.74 | 4.5e-02 |
| Pou3f4\_3773 |  | ceh-6 | 24 | 1.91 | 4.6e-02 |
| pTH9256 |  | ceh-18 | 36 | 1.57 | 4.6e-02 |
| pTH10654 |  | ceh-90 | 11 | 3.09 | 4.8e-02 |
| SPDEF\_2 |  | lin-1 | 14 | 2.59 | 4.8e-02 |
| V$TCF11\_01 |  | skn-1 | 20 | 2.09 | 4.8e-02 |
| PO3F2\_si |  | ceh-18 | 28 | 1.76 | 4.8e-02 |
| gl\_FlyReg\_FBgn0004618 |  | ces-1 | 47 | 1.38 | 4.9e-02 |
| VDR\_1 |  | nhr-208 | 20 | 2.09 | 4.9e-02 |
| pTH5257 |  | C48E7.11 | 15 | 2.47 | 4.9e-02 |

### Correlated (and anti-correlated) transcription factors

|  |  |
| --- | --- |
| **Transcription factor** | **Correlation** |
| nhr-183 | 0.94 |
| nhr-55 | 0.92 |
| nhr-66 | 0.89 |
| nhr-182 | 0.89 |
| nhr-237 | 0.89 |
| nhr-129 | 0.88 |
| C08G9.2 | 0.88 |
| nhr-162 | 0.88 |
| nhr-112 | 0.86 |
| nhr-50 | 0.86 |
| nhr-56 | 0.85 |
| nhr-213 | 0.85 |
| nhr-18 | 0.85 |
| nhr-103 | 0.84 |
| nhr-126 | 0.83 |
| nhr-133 | 0.82 |
| nhr-96 | 0.82 |
| nhr-219 | 0.81 |
| nhr-117 | 0.81 |
| nhr-63 | 0.80 |
| nhr-128 | 0.80 |
| nhr-134 | 0.80 |
| nhr-184 | 0.80 |
| ceh-19 | 0.80 |
| nhr-70 | 0.79 |
| T22C8.3 | -0.54 |
| mab-5 | -0.54 |
| ztf-28 | -0.55 |
| lir-3 | -0.56 |
| R144.3 | -0.57 |
| rcor-1 | -0.57 |
| ceh-13 | -0.57 |
| lin-39 | -0.59 |
| dhhc-10 | -0.60 |
| pax-3 | -0.60 |
| bar-1 | -0.61 |
| ceh-41 | -0.61 |
| him-8 | -0.61 |
| cey-2 | -0.62 |
| nhr-172 | -0.63 |
| dhhc-7 | -0.63 |
| zag-1 | -0.64 |
| nhr-171 | -0.66 |
| C09F5.3 | -0.67 |
| ceh-20 | -0.68 |
| unc-3 | -0.71 |
| F21D5.9 | -0.71 |
| elt-1 | -0.72 |
| hmg-12 | -0.72 |
| zip-11 | -0.77 |

### ChIP peaks enriched

none found
