# Supplementary material for: Overlapping cell population expression profiling and regulatory inference in C. elegans
Source: BMC Genomics. 2016 Feb 29;17:159. doi: 10.1186/s12864-016-2482-z (PMC4772325; doi:10.1186/s12864-016-2482-z)
Supplement: Additional file 13: — Web supplement. (DOC 21 kb) [file 12864_2016_2482_MOESM13_ESM.zip › sortWeb/clusters/hier.300.clusters/271.html]

Cluster 271 

## Cluster 271

### Expression

| cnd-1 rep. 1 | cnd-1 rep. 2 | cnd-1 rep. 3 | pha-4 rep. 1 | pha-4 rep. 2 | pha-4 rep. 3 | ceh-27 | ceh-36 | ceh-6 | F21D5.9 | mir-57 | mls-2 | pal-1 | pros-1 | ttx-3 | unc-130 | hlh-16 | irx-1 | ceh-6 (+) hlh-16 (+) | ceh-6 (+) hlh-16 (-) | ceh-6 (-) hlh-16 (+) | cnd-1 singlets | pha-4 singlets | 0 | 60 | 120 | 150 | 180 | 240 | 330 | 390 | 420 | 480 | 540 | 570 | 600 | 630 | 660 | NAME | Functional description |
| --- | --- | --- | --- | --- | --- | --- | --- | --- | --- | --- | --- | --- | --- | --- | --- | --- | --- | --- | --- | --- | --- | --- | --- | --- | --- | --- | --- | --- | --- | --- | --- | --- | --- | --- | --- | --- | --- | --- | --- |
|  |  |  |  |  |  |  |  |  |  |  |  |  |  |  |  |  |  |  |  |  |  |  |  |  |  |  |  |  |  |  |  |  |  |  |  |  |  | *srh-70* | Serpentine Receptor, class H |
|  |  |  |  |  |  |  |  |  |  |  |  |  |  |  |  |  |  |  |  |  |  |  |  |  |  |  |  |  |  |  |  |  |  |  |  |  |  | *spat-2* | Suppressor of PAr-Two defect |
|  |  |  |  |  |  |  |  |  |  |  |  |  |  |  |  |  |  |  |  |  |  |  |  |  |  |  |  |  |  |  |  |  |  |  |  |  |  | F09C12.8 |  |
|  |  |  |  |  |  |  |  |  |  |  |  |  |  |  |  |  |  |  |  |  |  |  |  |  |  |  |  |  |  |  |  |  |  |  |  |  |  | Y69E1A.12 |  |
|  |  |  |  |  |  |  |  |  |  |  |  |  |  |  |  |  |  |  |  |  |  |  |  |  |  |  |  |  |  |  |  |  |  |  |  |  |  | *lin-17* | abnormal cell LINeage |
|  |  |  |  |  |  |  |  |  |  |  |  |  |  |  |  |  |  |  |  |  |  |  |  |  |  |  |  |  |  |  |  |  |  |  |  |  |  | *riok-1* | RIO Kinase homolog |
|  |  |  |  |  |  |  |  |  |  |  |  |  |  |  |  |  |  |  |  |  |  |  |  |  |  |  |  |  |  |  |  |  |  |  |  |  |  | Y51A2D.20 |  |
|  |  |  |  |  |  |  |  |  |  |  |  |  |  |  |  |  |  |  |  |  |  |  |  |  |  |  |  |  |  |  |  |  |  |  |  |  |  | T28F3.7 |  |
|  |  |  |  |  |  |  |  |  |  |  |  |  |  |  |  |  |  |  |  |  |  |  |  |  |  |  |  |  |  |  |  |  |  |  |  |  |  | *snf-9* | Sodium: Neurotransmitter symporter Family |
|  |  |  |  |  |  |  |  |  |  |  |  |  |  |  |  |  |  |  |  |  |  |  |  |  |  |  |  |  |  |  |  |  |  |  |  |  |  | Y26D4A.22 |  |
|  |  |  |  |  |  |  |  |  |  |  |  |  |  |  |  |  |  |  |  |  |  |  |  |  |  |  |  |  |  |  |  |  |  |  |  |  |  | *clec-153* | C-type LECtin |
|  |  |  |  |  |  |  |  |  |  |  |  |  |  |  |  |  |  |  |  |  |  |  |  |  |  |  |  |  |  |  |  |  |  |  |  |  |  | H38K22.6 |  |
|  |  |  |  |  |  |  |  |  |  |  |  |  |  |  |  |  |  |  |  |  |  |  |  |  |  |  |  |  |  |  |  |  |  |  |  |  |  | F01E11.14 |  |
|  |  |  |  |  |  |  |  |  |  |  |  |  |  |  |  |  |  |  |  |  |  |  |  |  |  |  |  |  |  |  |  |  |  |  |  |  |  | F07B10.4 |  |
|  |  |  |  |  |  |  |  |  |  |  |  |  |  |  |  |  |  |  |  |  |  |  |  |  |  |  |  |  |  |  |  |  |  |  |  |  |  | C29F5.3 |  |
|  |  |  |  |  |  |  |  |  |  |  |  |  |  |  |  |  |  |  |  |  |  |  |  |  |  |  |  |  |  |  |  |  |  |  |  |  |  | *linc-106* | Long Intervening Non-Coding RNA |
|  |  |  |  |  |  |  |  |  |  |  |  |  |  |  |  |  |  |  |  |  |  |  |  |  |  |  |  |  |  |  |  |  |  |  |  |  |  | *sri-49* | Serpentine Receptor, class I |
|  |  |  |  |  |  |  |  |  |  |  |  |  |  |  |  |  |  |  |  |  |  |  |  |  |  |  |  |  |  |  |  |  |  |  |  |  |  | T05A6.8 |  |
|  |  |  |  |  |  |  |  |  |  |  |  |  |  |  |  |  |  |  |  |  |  |  |  |  |  |  |  |  |  |  |  |  |  |  |  |  |  | F25F2.1 |  |
|  |  |  |  |  |  |  |  |  |  |  |  |  |  |  |  |  |  |  |  |  |  |  |  |  |  |  |  |  |  |  |  |  |  |  |  |  |  | M01H9.5 |  |
|  |  |  |  |  |  |  |  |  |  |  |  |  |  |  |  |  |  |  |  |  |  |  |  |  |  |  |  |  |  |  |  |  |  |  |  |  |  | T25B2.3 |  |
|  |  |  |  |  |  |  |  |  |  |  |  |  |  |  |  |  |  |  |  |  |  |  |  |  |  |  |  |  |  |  |  |  |  |  |  |  |  | C29H12.12 |  |
|  |  |  |  |  |  |  |  |  |  |  |  |  |  |  |  |  |  |  |  |  |  |  |  |  |  |  |  |  |  |  |  |  |  |  |  |  |  | C33H5.1 |  |
|  |  |  |  |  |  |  |  |  |  |  |  |  |  |  |  |  |  |  |  |  |  |  |  |  |  |  |  |  |  |  |  |  |  |  |  |  |  | T02E9.13 |  |
|  |  |  |  |  |  |  |  |  |  |  |  |  |  |  |  |  |  |  |  |  |  |  |  |  |  |  |  |  |  |  |  |  |  |  |  |  |  | C10A4.6 |  |
|  |  |  |  |  |  |  |  |  |  |  |  |  |  |  |  |  |  |  |  |  |  |  |  |  |  |  |  |  |  |  |  |  |  |  |  |  |  | *rap-1* | RAP homolog (vertebrate Rap GTPase family) |
|  |  |  |  |  |  |  |  |  |  |  |  |  |  |  |  |  |  |  |  |  |  |  |  |  |  |  |  |  |  |  |  |  |  |  |  |  |  | F34H10.3 |  |
|  |  |  |  |  |  |  |  |  |  |  |  |  |  |  |  |  |  |  |  |  |  |  |  |  |  |  |  |  |  |  |  |  |  |  |  |  |  | *ral-1* | RAL (Ras-related GTPase) homolog |
|  |  |  |  |  |  |  |  |  |  |  |  |  |  |  |  |  |  |  |  |  |  |  |  |  |  |  |  |  |  |  |  |  |  |  |  |  |  | C54G4.7 |  |
|  |  |  |  |  |  |  |  |  |  |  |  |  |  |  |  |  |  |  |  |  |  |  |  |  |  |  |  |  |  |  |  |  |  |  |  |  |  | *acy-3* | Adenylyl CYclase |
|  |  |  |  |  |  |  |  |  |  |  |  |  |  |  |  |  |  |  |  |  |  |  |  |  |  |  |  |  |  |  |  |  |  |  |  |  |  | *deb-1* | DEnse Body |
|  |  |  |  |  |  |  |  |  |  |  |  |  |  |  |  |  |  |  |  |  |  |  |  |  |  |  |  |  |  |  |  |  |  |  |  |  |  | C13E3.1 |  |
|  |  |  |  |  |  |  |  |  |  |  |  |  |  |  |  |  |  |  |  |  |  |  |  |  |  |  |  |  |  |  |  |  |  |  |  |  |  | *egl-18* | EGg Laying defective |
|  |  |  |  |  |  |  |  |  |  |  |  |  |  |  |  |  |  |  |  |  |  |  |  |  |  |  |  |  |  |  |  |  |  |  |  |  |  | *elt-6* | Erythroid-Like Transcription factor family |
|  |  |  |  |  |  |  |  |  |  |  |  |  |  |  |  |  |  |  |  |  |  |  |  |  |  |  |  |  |  |  |  |  |  |  |  |  |  | *unc-116* | UNCoordinated |
|  |  |  |  |  |  |  |  |  |  |  |  |  |  |  |  |  |  |  |  |  |  |  |  |  |  |  |  |  |  |  |  |  |  |  |  |  |  | R10H10.7 |  |
|  |  |  |  |  |  |  |  |  |  |  |  |  |  |  |  |  |  |  |  |  |  |  |  |  |  |  |  |  |  |  |  |  |  |  |  |  |  | *gfi-2* | GEI-4 (Four) Interacting protein |
|  |  |  |  |  |  |  |  |  |  |  |  |  |  |  |  |  |  |  |  |  |  |  |  |  |  |  |  |  |  |  |  |  |  |  |  |  |  | *nrfl-1* | NHERF (mammalian Na/H Exchange Regulatory Factor) Like |
|  |  |  |  |  |  |  |  |  |  |  |  |  |  |  |  |  |  |  |  |  |  |  |  |  |  |  |  |  |  |  |  |  |  |  |  |  |  | C27H2.2 |  |
|  |  |  |  |  |  |  |  |  |  |  |  |  |  |  |  |  |  |  |  |  |  |  |  |  |  |  |  |  |  |  |  |  |  |  |  |  |  | *let-413* | LEThal |
|  |  |  |  |  |  |  |  |  |  |  |  |  |  |  |  |  |  |  |  |  |  |  |  |  |  |  |  |  |  |  |  |  |  |  |  |  |  | *ham-2* | HSN Abnormal Migration |
|  |  |  |  |  |  |  |  |  |  |  |  |  |  |  |  |  |  |  |  |  |  |  |  |  |  |  |  |  |  |  |  |  |  |  |  |  |  | *nhr-35* | Nuclear Hormone Receptor family |
|  |  |  |  |  |  |  |  |  |  |  |  |  |  |  |  |  |  |  |  |  |  |  |  |  |  |  |  |  |  |  |  |  |  |  |  |  |  | *rgl-1* | Ral GDS-Like |
|  |  |  |  |  |  |  |  |  |  |  |  |  |  |  |  |  |  |  |  |  |  |  |  |  |  |  |  |  |  |  |  |  |  |  |  |  |  | *dlg-1* | Drosophila Discs LarGe homolog |
|  |  |  |  |  |  |  |  |  |  |  |  |  |  |  |  |  |  |  |  |  |  |  |  |  |  |  |  |  |  |  |  |  |  |  |  |  |  | *dex-1* |  |
|  |  |  |  |  |  |  |  |  |  |  |  |  |  |  |  |  |  |  |  |  |  |  |  |  |  |  |  |  |  |  |  |  |  |  |  |  |  | *atn-1* | AcTiniN |
|  |  |  |  |  |  |  |  |  |  |  |  |  |  |  |  |  |  |  |  |  |  |  |  |  |  |  |  |  |  |  |  |  |  |  |  |  |  | *ras-1* | R-RAS related |
|  |  |  |  |  |  |  |  |  |  |  |  |  |  |  |  |  |  |  |  |  |  |  |  |  |  |  |  |  |  |  |  |  |  |  |  |  |  | *magu-4* | MAGUK family |
|  |  |  |  |  |  |  |  |  |  |  |  |  |  |  |  |  |  |  |  |  |  |  |  |  |  |  |  |  |  |  |  |  |  |  |  |  |  | *cdh-3* | CaDHerin family |
|  |  |  |  |  |  |  |  |  |  |  |  |  |  |  |  |  |  |  |  |  |  |  |  |  |  |  |  |  |  |  |  |  |  |  |  |  |  | *ncx-3* | Na/Ca eXchangers |
|  |  |  |  |  |  |  |  |  |  |  |  |  |  |  |  |  |  |  |  |  |  |  |  |  |  |  |  |  |  |  |  |  |  |  |  |  |  | C25F6.13 |  |
|  |  |  |  |  |  |  |  |  |  |  |  |  |  |  |  |  |  |  |  |  |  |  |  |  |  |  |  |  |  |  |  |  |  |  |  |  |  | *moc-1* | MOlybdenum Cofactor biosynthesis |
|  |  |  |  |  |  |  |  |  |  |  |  |  |  |  |  |  |  |  |  |  |  |  |  |  |  |  |  |  |  |  |  |  |  |  |  |  |  | T01B6.1 |  |
|  |  |  |  |  |  |  |  |  |  |  |  |  |  |  |  |  |  |  |  |  |  |  |  |  |  |  |  |  |  |  |  |  |  |  |  |  |  | *crb-1* | Drosophila CRumBs homolog |

### Phenotypes enriched

none found

### Anatomy terms enriched

none found

### GO terms enriched

|  |  |  |
| --- | --- | --- |
| **GO term** | **Number of genes** | **FDR-corrected p-value** |
| adherens junction | 3 | 0.014 |

### Expression clusters enriched

none found

### Motifs enriched

|  |  |  |  |  |  |
| --- | --- | --- | --- | --- | --- |
| **Motif** | **Logo** | **Possible orthologs** | **Number of motifs in cluster** | **Enrichment** | **FDR corrected p** |
| Dfd\_Cell\_FBgn0000439 |  | cfi-1 (0.54) unc-86 php-3 lim-7 lin-39 | 36 | 2.34 | 3.4e-06 |
| pTH9480 |  | ces-2 (0.56) atf-2 Y51H4A.4 C48E7.11 F23F12.9 | 23 | 3.70 | 3.8e-06 |
| Pou3f3\_3235 |  | ceh-6 | 17 | 5.00 | 8.3e-06 |
| pTH9342 |  | ceh-45 alr-1 ceh-12 ceh-1 ceh-53 npax-3 ceh-43 lin-39 ceh-18 | 36 | 2.25 | 8.8e-06 |
| pTH8991 |  | cey-3 | 24 | 3.35 | 9.1e-06 |
| pTH5812 |  | ceh-14 | 32 | 2.50 | 1.1e-05 |
| pTH9885 |  | unc-86 ceh-18 | 30 | 2.65 | 1.1e-05 |
| pTH6520 |  | ceh-2 (0.62) cog-1 (0.51) lim-4 ceh-45 pha-2 alr-1 ceh-31 ceh-12 ceh-1 egl-5 ceh-36 pal-1 ceh-16 dsc-1 ceh-53 mls-2 lim-6 lim-7 ceh-43 lin-39 and 9 others  [full list] | 29 | 2.73 | 1.2e-05 |
| ems\_FlyReg\_FBgn0000576 |  | ceh-2 (0.62) skn-1 | 28 | 2.82 | 1.3e-05 |
| Pou3f4\_3773 |  | ceh-6 | 37 | 2.16 | 1.4e-05 |
| pTH6268 |  | ceh-2 (0.62) | 28 | 2.80 | 1.5e-05 |
| Emx2\_3420 |  | ceh-2 (0.62) | 35 | 2.21 | 2.6e-05 |
| pTH9216 |  | ceh-18 | 33 | 2.31 | 3.3e-05 |
| Pou2f2\_3748 |  | alr-1 lim-7 ceh-18 | 35 | 2.14 | 6.0e-05 |
| pTH6436 |  | ceh-53 | 35 | 2.14 | 6.0e-05 |
| pTH9188 |  | dmd-5 dmd-4 | 34 | 2.17 | 7.1e-05 |
| pTH5437 |  | ceh-34 (0.53) | 24 | 2.93 | 8.3e-05 |
| MA0015.1 |  | che-1 K11D2.4 tbp-1 | 14 | 5.19 | 8.7e-05 |
| MA0543.1 |  | daf-8 (0.61) eor-1 | 39 | 1.91 | 8.9e-05 |
| OLIG2\_1 |  | hlh-8 ngn-1 hlh-32 hlh-15 | 24 | 2.90 | 1.0e-04 |
| HLH4C\_da\_SANGER\_5\_4\_FBgn0011277 |  | hlh-2 (0.52) hlh-8 hlh-1 ces-1 K02D7.2 hlh-15 | 36 | 2.01 | 1.4e-04 |
| Arx\_1738 |  | eyg-1 (0.55) ceh-45 alr-1 ceh-1 lin-39 ceh-10 | 29 | 2.39 | 1.6e-04 |
| MA0078.1 |  | pop-1 gei-3 sox-4 C05C9.3 | 42 | 1.75 | 1.7e-04 |
| Hoxa2\_3079 |  | lin-39 | 32 | 2.19 | 1.8e-04 |
| Pou3f2\_2824 |  | lin-39 ceh-6 ceh-18 | 33 | 2.14 | 1.8e-04 |
| MEIS2\_do |  | lin-39 lin-32 ceh-32 | 40 | 1.82 | 1.9e-04 |
| NR4A1\_f1 |  | nhr-213 (0.54) nhr-10 nhr-6 nhr-71 nhr-2 nhr-68 Y67D8A.3 | 36 | 1.98 | 1.9e-04 |
| Sox15\_3457 |  | sox-4 | 38 | 1.90 | 1.9e-04 |
| V$AREB6\_02 |  | ztf-6 | 35 | 2.03 | 2.0e-04 |
| Vnd\_SOLEXA\_FBgn0003986 |  | ceh-22 (0.58) dsc-1 ceh-24 | 22 | 2.95 | 2.6e-04 |
| MA0058.2 |  | hlh-30 (0.8) mxl-1 (-0.57) irx-1 | 37 | 1.91 | 2.6e-04 |
| Vax2\_3500 |  | C02F12.10 | 35 | 1.99 | 2.8e-04 |
| TCF4\_2 |  | hlh-2 (0.52) | 33 | 2.08 | 3.1e-04 |
| MA0446.1 |  | lin-31 let-381 | 29 | 2.31 | 3.1e-04 |
| Oli\_da\_SANGER\_5\_2\_FBgn0032651 |  | hlh-8 ngn-1 hlh-32 hlh-12 hlh-15 | 35 | 1.98 | 3.4e-04 |
| pTH6562 |  | ceh-5 | 31 | 2.17 | 3.6e-04 |
| pTH5169 |  | cfi-1 (0.54) | 20 | 3.14 | 3.6e-04 |
| I$UBX\_01 |  | lin-39 | 28 | 2.34 | 4.1e-04 |
| IRX2\_1 |  | irx-1 | 38 | 1.83 | 4.3e-04 |
| Hoxb3\_1720 |  | lin-39 | 35 | 1.95 | 4.7e-04 |
| Atf1\_3026 |  | crh-1 | 37 | 1.86 | 4.9e-04 |
| En2\_0952 |  | ceh-16 lim-6 lim-7 ceh-24 | 33 | 2.03 | 5.2e-04 |
| Evx1\_3952 |  | ceh-53 | 33 | 2.03 | 5.2e-04 |
| pTH9215 |  | C34D1.1 | 41 | 1.71 | 5.4e-04 |
| Foxj3\_2 |  | fkh-7 (0.73) lin-31 daf-16 let-381 fkh-8 | 33 | 2.02 | 5.5e-04 |
| pTH10717 |  | syd-9 lsl-1 | 32 | 2.06 | 5.9e-04 |
| Pou3f1\_3819 |  | ceh-6 | 33 | 2.02 | 5.9e-04 |
| RAX\_1 |  | alr-1 ceh-31 ceh-1 ceh-16 lim-7 ceh-43 | 34 | 1.97 | 6.0e-04 |
| V$LYF1\_01 |  | nhr-177 (0.54) F26F4.8 | 26 | 2.42 | 6.2e-04 |
| pTH5018 |  | fos-1 atf-7 | 34 | 1.96 | 6.3e-04 |
| pTH3819 |  | ceh-18 | 32 | 2.04 | 7.1e-04 |
| pTH8399 |  | lin-54 | 28 | 2.26 | 7.1e-04 |
| Bsh\_Cell\_FBgn0000529 |  | cog-1 (0.51) ceh-19 alr-1 ceh-9 ceh-31 ceh-1 ceh-43 lim-7 ceh-8 ceh-24 ceh-30 | 31 | 2.09 | 7.2e-04 |
| Titf1\_1722 |  | dsc-1 ceh-24 | 17 | 3.45 | 7.2e-04 |
| pTH8983 |  | tag-347 | 30 | 2.13 | 7.9e-04 |
| Fer2\_da\_SANGER\_5\_FBgn0038402 |  | hlh-11 hlh-14 hlh-1 lin-32 hlh-15 | 23 | 2.63 | 8.0e-04 |
| Mv129 |  | sox-4 ceh-6 ceh-18 tbp-1 | 21 | 2.82 | 8.3e-04 |
| SOX2\_1 |  | sox-4 | 29 | 2.18 | 8.7e-04 |
| PBX1\_do |  | ceh-12 lin-39 ceh-20 | 38 | 1.78 | 8.8e-04 |
| DLX2\_f1 |  | hmg-12 (-0.52) lin-31 let-381 ceh-53 ceh-43 Y116A8C.22 | 35 | 1.89 | 8.9e-04 |
| ss\_tgo\_SANGER\_10\_FBgn0015014 |  | aha-1 ahr-1 | 34 | 1.93 | 9.0e-04 |
| FOXO4\_3 |  | ZC328.2 daf-16 | 23 | 2.58 | 1.0e-03 |
| Vsx1\_1728 |  | alr-1 | 31 | 2.05 | 1.1e-03 |
| PURA\_f1 |  | klf-2 (0.66) Y53H1A.2 (-0.56) plp-2 | 26 | 2.34 | 1.1e-03 |
| V$ZID\_01 |  | ztf-28 skn-1 ceh-32 | 16 | 3.51 | 1.1e-03 |
| pTH2684 |  | fos-1 | 33 | 1.95 | 1.1e-03 |
| pTH5078 |  | ces-2 (0.56) | 32 | 2.00 | 1.1e-03 |
| MA0150.2 |  | fos-1 crh-1 sknr-1 jun-1 F45H11.6 | 35 | 1.87 | 1.1e-03 |
| pTH6636 |  | egl-5 | 36 | 1.83 | 1.1e-03 |
| pTH9901 |  | php-3 lin-39 ceh-24 D1005.3 T27F2.4 | 26 | 2.33 | 1.1e-03 |
| TBX1\_1 |  | tbx-39 tbx-38 mab-9 tbx-43 | 20 | 2.87 | 1.1e-03 |
| Vax1\_3499 |  | C02F12.10 | 30 | 2.08 | 1.3e-03 |
| TFE3\_f1 |  | hlh-30 (0.8) pax-1 (0.63) aha-1 mdl-1 | 38 | 1.75 | 1.3e-03 |
| MA0599.1 |  | klf-2 (0.66) ZC328.2 klf-1 | 34 | 1.90 | 1.3e-03 |
| PDX1\_do |  | alr-1 ceh-12 pal-1 lin-39 ceh-18 | 30 | 2.07 | 1.3e-03 |
| STF1\_f1 |  | nhr-68 | 24 | 2.44 | 1.4e-03 |
| Gsh2\_3990 |  | ceh-31 | 29 | 2.11 | 1.5e-03 |
| pTH9381 |  | ceh-18 | 34 | 1.88 | 1.5e-03 |
| EGR2\_2 |  | klf-2 (0.66) ZC328.2 klf-1 | 34 | 1.88 | 1.6e-03 |
| HXD13\_f1 |  | pal-1 | 23 | 2.50 | 1.6e-03 |
| V$E47\_02 |  | hlh-2 (0.52) lin-32 | 22 | 2.58 | 1.6e-03 |
| Msx3\_1 |  | ceh-31 ceh-1 ceh-43 lin-39 ceh-14 | 36 | 1.80 | 1.6e-03 |
| pTH9118 |  | ref-2 (0.67) eor-1 egrh-3 | 23 | 2.49 | 1.7e-03 |
| PO3F2\_si |  | ceh-18 | 34 | 1.87 | 1.7e-03 |
| Pou2f1\_3081 |  | ceh-18 | 24 | 2.40 | 1.8e-03 |
| Hoxc8\_3429 |  | lin-39 | 35 | 1.83 | 1.8e-03 |
| Hoxa3\_2783 |  | lin-39 | 33 | 1.91 | 1.8e-03 |
| pTH6486 |  | nhr-145 | 37 | 1.76 | 1.8e-03 |
| Ehf |  | lin-1 C24A1.2 | 20 | 2.76 | 1.8e-03 |
| Nkx1-2\_3214 |  | ceh-30 | 28 | 2.14 | 1.9e-03 |
| EPAS1\_si |  | daf-12 (0.72) hif-1 ztf-3 Y5F2A.4 | 23 | 2.47 | 1.9e-03 |
| Mw151 |  | ztf-6 gei-11 C34D1.1 | 33 | 1.90 | 1.9e-03 |
| Meox1\_2310 |  | ceh-31 | 31 | 1.98 | 1.9e-03 |
| pTH9913 |  | fos-1 skn-1 | 24 | 2.38 | 2.0e-03 |
| Ara\_Cell\_FBgn0015904 |  | irx-1 | 15 | 3.53 | 2.0e-03 |
| MA0007.2 |  | npax-1 nhr-255 lin-14 | 33 | 1.90 | 2.0e-03 |
| Prop1\_3949 |  | ceh-16 ceh-53 | 29 | 2.07 | 2.0e-03 |
| RFX1\_4537 |  | daf-19 | 12 | 4.40 | 2.0e-03 |
| pTH5887 |  | lin-39 | 31 | 1.98 | 2.1e-03 |
| GRHL1\_1 |  | grh-1 (0.52) | 11 | 4.81 | 2.2e-03 |
| Zfp652\_1 |  | B0310.2 ZK177.3 | 34 | 1.85 | 2.2e-03 |
| pTH9155 |  | lin-48 (0.61) D1081.8 | 27 | 2.17 | 2.2e-03 |
| FEV\_f1 |  | lin-1 C24A1.2 | 19 | 2.83 | 2.3e-03 |
| V$GATA6\_01 |  | elt-1 | 33 | 1.88 | 2.3e-03 |
| V$FOXJ2\_02 |  | lin-31 | 31 | 1.96 | 2.4e-03 |
| Pbx1\_3203 |  | ceh-20 | 38 | 1.70 | 2.4e-03 |
| pTH8333 |  | ZC416.1 | 17 | 3.08 | 2.5e-03 |
| MA0535.1 |  | daf-8 (0.61) | 21 | 2.59 | 2.5e-03 |
| K562b\_GATA2\_UCD |  | ztf-29 elt-1 | 9 | 6.00 | 2.5e-03 |
| V$CEBPA\_01 |  | C48E7.11 | 11 | 4.71 | 2.5e-03 |
| MEF2D\_1 |  | mef-2 | 41 | 1.61 | 2.6e-03 |
| MA0508.1 |  | blmp-1 (0.56) | 35 | 1.80 | 2.6e-03 |
| Caup\_SOLEXA\_FBgn0015919 |  | irx-1 | 29 | 2.04 | 2.6e-03 |
| pTH9125 |  | sox-4 egl-13 K11D2.4 | 20 | 2.68 | 2.6e-03 |
| pTH5270 |  | ngn-1 hlh-32 hlh-16 | 31 | 1.95 | 2.7e-03 |
| HepG2\_SRF\_HudsonAlpha |  | unc-120 hlh-10 | 11 | 4.67 | 2.7e-03 |
| pTH6478 |  | lim-7 | 23 | 2.40 | 2.8e-03 |
| pTH2340 |  | ces-1 ceh-20 ceh-32 | 35 | 1.79 | 2.8e-03 |
| V$FAC1\_01 |  | gei-8 | 24 | 2.32 | 2.8e-03 |
| Hoxa4\_3426 |  | lin-39 | 30 | 1.98 | 2.9e-03 |
| CG14962\_SANGER\_5\_FBgn0035407 |  | C34H4.5 T22H9.4 | 39 | 1.65 | 3.0e-03 |
| SP1\_1 |  | klf-2 (0.66) klf-1 | 32 | 1.89 | 3.1e-03 |
| Dlx3\_1030 |  | ceh-43 | 30 | 1.97 | 3.2e-03 |
| Pou2f3\_3986 |  | ceh-18 | 32 | 1.89 | 3.2e-03 |
| V$EN1\_01 |  | ceh-16 atf-2 | 17 | 3.00 | 3.3e-03 |
| pTH9256 |  | ceh-18 | 39 | 1.64 | 3.4e-03 |
| pTH1001 |  | dnj-17 | 33 | 1.84 | 3.5e-03 |
| ZBTB49\_1 |  | aha-1 C46E10.9 | 35 | 1.76 | 3.6e-03 |
| pTH9300 |  | dmd-3 C34D1.1 | 27 | 2.10 | 3.7e-03 |
| V$GATA1\_03 |  | elt-1 | 30 | 1.95 | 3.7e-03 |
| pTH5083 |  | fos-1 crh-1 | 30 | 1.95 | 3.8e-03 |
| SMAD3\_f1 |  | daf-8 (0.61) | 29 | 2.00 | 3.8e-03 |
| Mw145 |  | ceh-34 (0.53) elt-3 elt-1 ceh-32 | 36 | 1.73 | 3.8e-03 |
| MCR\_f1 |  | nhr-255 | 25 | 2.21 | 3.8e-03 |
| V$OCT1\_03 |  | ceh-18 | 30 | 1.95 | 3.8e-03 |
| CG31670\_SANGER\_5\_FBgn0031375 |  | F21A9.2 CELE\_Y38H8A.5 | 33 | 1.83 | 3.9e-03 |
| ELF1\_2 |  | lin-1 C24A1.2 | 32 | 1.86 | 3.9e-03 |
| MA0503.1 |  | pzf-1 ceh-24 | 29 | 1.99 | 4.0e-03 |
| K562\_ZBTB7A\_HudsonAlpha |  | ZC328.2 | 29 | 1.99 | 4.0e-03 |
| pTH9026 |  | attf-1 | 26 | 2.14 | 4.2e-03 |
| Hoxa7\_3750 |  | lin-39 | 34 | 1.78 | 4.2e-03 |
| EN1\_4 |  | ceh-2 (0.62) ceh-16 | 34 | 1.78 | 4.4e-03 |
| pTH5778 |  | egl-5 | 35 | 1.75 | 4.4e-03 |
| V$CREB\_Q2 |  | crh-1 | 34 | 1.78 | 4.5e-03 |
| MA0488.1 |  | crh-1 | 36 | 1.71 | 4.5e-03 |
| MA0066.1 |  | nhr-6 nhr-71 nhr-43 | 30 | 1.93 | 4.5e-03 |
| HeLa-S3\_ZNF274\_UCD |  | C28G1.4 | 22 | 2.39 | 4.6e-03 |
| V$GATA3\_01 |  | elt-1 | 12 | 3.96 | 4.6e-03 |
| pTH10034 |  | nhr-66 (0.61) | 33 | 1.81 | 4.6e-03 |
| pTH9326 |  | nhr-122 (0.53) | 24 | 2.24 | 4.7e-03 |
| V$CMYB\_01 |  | Y53H1A.2 (-0.56) D1081.8 | 31 | 1.88 | 4.8e-03 |
| pTH2882 |  | hlh-30 (0.8) mxl-1 (-0.57) mxl-2 (-0.53) aha-1 hlh-26 | 31 | 1.88 | 4.8e-03 |
| I$CROC\_01 |  | let-381 | 26 | 2.11 | 5.0e-03 |
| pTH3086 |  | klf-2 (0.66) klf-1 | 28 | 2.00 | 5.2e-03 |
| HUVEC\_GATA2\_UCD |  | elt-1 | 34 | 1.77 | 5.2e-03 |
| pTH8216 |  | Y116A8C.22 | 29 | 1.95 | 5.7e-03 |
| pTH9323 |  | odr-7 nhr-273 | 37 | 1.66 | 5.7e-03 |
| pTH6327 |  | dsc-1 | 12 | 3.83 | 6.0e-03 |
| NR2F6\_f1 |  | nhr-239 nhr-2 | 40 | 1.57 | 6.1e-03 |
| pTH9246 |  | let-381 fkh-10 C34D1.1 | 38 | 1.63 | 6.3e-03 |
| V$PAX2\_02 |  | pax-1 (0.63) | 35 | 1.71 | 6.3e-03 |
| FLI1\_f1 |  | lin-1 | 33 | 1.78 | 6.4e-03 |
| disco-r-Cl1\_SANGER\_5\_FBgn0042650 |  | F55C5.11 | 32 | 1.81 | 6.6e-03 |
| MA0486.1 |  | Y53C10A.3 | 24 | 2.18 | 6.8e-03 |
| V$FOXO1\_02 |  | fkh-7 (0.73) daf-16 let-381 fkh-10 fkh-8 | 30 | 1.88 | 6.9e-03 |
| Elf3\_3876 |  | C24A1.2 | 19 | 2.56 | 7.0e-03 |
| SPDEF\_2 |  | ztf-14 lin-1 | 31 | 1.84 | 7.0e-03 |
| Hoxa7\_2668 |  | lin-39 | 32 | 1.80 | 7.0e-03 |
| Gata3\_1024 |  | elt-1 | 15 | 3.08 | 7.0e-03 |
| HES1\_f1 |  | lin-22 | 27 | 2.01 | 7.1e-03 |
| MA0139.1 |  | F58G1.2 | 31 | 1.83 | 7.5e-03 |
| Mw140 |  | efl-1 F49E12.6 | 23 | 2.22 | 7.5e-03 |
| V$IK2\_01 |  | F26F4.8 | 33 | 1.76 | 7.5e-03 |
| CG2052\_SANGER\_2.5\_FBgn0039905 |  | fkh-7 (0.73) lin-29 (0.63) mel-28 | 35 | 1.70 | 7.7e-03 |
| pTH3041 |  | atf-2 | 29 | 1.91 | 7.7e-03 |
| pTH9915 |  | zip-3 | 31 | 1.83 | 7.7e-03 |
| Mw144 |  | elt-1 | 35 | 1.70 | 7.8e-03 |
| SPDEF\_3 |  | lin-39 nhr-100 lin-1 | 29 | 1.91 | 7.9e-03 |
| V$TCF11\_01 |  | skn-1 | 35 | 1.69 | 8.2e-03 |
| Irx5\_2385 |  | irx-1 | 30 | 1.86 | 8.2e-03 |
| Lbx2\_3869 |  | mls-2 | 29 | 1.90 | 8.3e-03 |
| FOXI1\_f1 |  | lin-31 | 17 | 2.73 | 8.4e-03 |
| V$CETS1P54\_02 |  | C52B9.2 | 14 | 3.19 | 8.4e-03 |
| V$YY1\_01 |  | lsy-2 | 37 | 1.63 | 8.5e-03 |
| pTH2820 |  | ZC328.2 | 31 | 1.82 | 8.7e-03 |
| pTH9245 |  | ceh-18 | 36 | 1.66 | 8.8e-03 |
| MA0544.1 |  | gei-11 | 27 | 1.98 | 8.8e-03 |
| pTH4325 |  | ceh-18 | 21 | 2.33 | 9.2e-03 |
| HOXC10\_1 |  | php-3 lin-39 | 17 | 2.71 | 9.2e-03 |
| TBP\_f1 |  | tbp-1 | 26 | 2.02 | 9.3e-03 |
| SMAD3\_1 |  | daf-8 (0.61) hlh-8 sma-4 | 25 | 2.07 | 9.3e-03 |
| Dlx2\_2273 |  | ceh-43 | 29 | 1.89 | 9.3e-03 |
| pTH10808 |  | ztf-19 | 23 | 2.18 | 9.4e-03 |
| V$S8\_01 |  | ceh-45 | 31 | 1.81 | 9.5e-03 |
| pTH9222 |  | mel-28 | 21 | 2.31 | 9.7e-03 |
| MA0174.1 |  | ceh-24 | 26 | 2.01 | 9.8e-03 |
| pTH5919 |  | irx-1 | 28 | 1.92 | 9.9e-03 |
| ZBT7A\_f1 |  | ZC328.2 klf-1 | 17 | 2.68 | 1.0e-02 |
| pTH10797 |  | lin-29 (0.63) K11D2.4 | 45 | 1.42 | 1.1e-02 |
| pTH6569 |  | ceh-43 | 27 | 1.95 | 1.1e-02 |
| GATA5\_1 |  | elt-1 end-3 | 26 | 2.00 | 1.1e-02 |
| pTH9043 |  | sem-2 (0.68) | 28 | 1.91 | 1.1e-02 |
| pTH9247 |  | dmd-3 C34D1.1 | 30 | 1.83 | 1.1e-02 |
| MA0027.1 |  | ceh-16 | 13 | 3.30 | 1.1e-02 |
| pTH10650 |  | nhr-153 (0.67) | 26 | 2.00 | 1.1e-02 |
| Elf2 |  | lin-1 C24A1.2 | 17 | 2.66 | 1.1e-02 |
| Hoxc13\_3127 |  | pal-1 ceh-24 | 16 | 2.79 | 1.1e-02 |
| Hoxc4\_3491 |  | lin-39 | 33 | 1.72 | 1.1e-02 |
| GATA5\_f1 |  | elt-1 nhr-100 | 25 | 2.04 | 1.1e-02 |
| Mf28 |  | elt-1 | 31 | 1.79 | 1.1e-02 |
| pTH9137 |  | nhr-65 | 39 | 1.56 | 1.1e-02 |
| Dlx1\_1741 |  | ceh-43 | 29 | 1.86 | 1.1e-02 |
| pTH2283 |  | odd-2 (0.64) | 24 | 2.09 | 1.1e-02 |
| pTH5257 |  | C48E7.11 | 30 | 1.82 | 1.1e-02 |
| Elf5 |  | C24A1.2 | 18 | 2.54 | 1.1e-02 |
| Sox4 |  | pop-1 sox-4 nhr-100 | 42 | 1.48 | 1.2e-02 |
| Gsc\_Cell\_FBgn0010323 |  | dve-1 (0.66) ceh-45 ceh-53 | 31 | 1.78 | 1.2e-02 |
| Elf4 |  | C24A1.2 | 17 | 2.62 | 1.2e-02 |
| pTH8745 |  | attf-1 | 21 | 2.26 | 1.3e-02 |
| Barx1\_2877 |  | ceh-43 | 27 | 1.93 | 1.3e-02 |
| pTH10623 |  | scrt-1 | 33 | 1.71 | 1.3e-02 |
| Srf\_3509 |  | unc-120 | 19 | 2.41 | 1.3e-02 |
| pTH5423 |  | klf-2 (0.66) | 23 | 2.12 | 1.4e-02 |
| ZNF75A\_1 |  | lag-1 F26F4.8 ztf-3 | 11 | 3.72 | 1.4e-02 |
| MA0161.1 |  | nfi-1 F49E12.6 | 33 | 1.70 | 1.4e-02 |
| Mcm1 |  | unc-120 | 29 | 1.83 | 1.4e-02 |
| V$GATA1\_02 |  | elt-1 | 27 | 1.91 | 1.4e-02 |
| MA0126.1 |  | lin-48 (0.61) pax-3 | 33 | 1.70 | 1.4e-02 |
| V$ARP1\_01 |  | nhr-2 | 35 | 1.64 | 1.5e-02 |
| Hoxa6\_1040 |  | lin-39 | 33 | 1.69 | 1.5e-02 |
| Hr46\_FlyReg\_FBgn0000448 |  | nhr-213 (0.54) lin-31 | 35 | 1.63 | 1.5e-02 |
| pTH9080 |  | mnm-2 | 19 | 2.38 | 1.5e-02 |
| pTH10788 |  | tbx-33 | 42 | 1.46 | 1.6e-02 |
| Bbx\_3753 |  | gei-3 | 29 | 1.82 | 1.6e-02 |
| Nkx6-1\_2825 |  | cog-1 (0.51) | 10 | 3.96 | 1.6e-02 |
| TLX1\_f1 |  | ceh-19 | 15 | 2.79 | 1.7e-02 |
| MA0124.1 |  | ceh-48 ceh-24 | 31 | 1.74 | 1.7e-02 |
| Pknox2\_3077 |  | ceh-32 | 30 | 1.78 | 1.7e-02 |
| MAX\_1 |  | mxl-1 (-0.57) K11D2.4 | 18 | 2.44 | 1.7e-02 |
| PRRX1\_3 |  | cfi-1 (0.54) alr-1 ceh-14 | 33 | 1.68 | 1.7e-02 |
| Hoxa5\_3415 |  | lin-39 | 27 | 1.89 | 1.7e-02 |
| ERG\_4 |  | lin-1 | 11 | 3.58 | 1.7e-02 |
| pTH9085 |  | nhr-42 (0.7) | 29 | 1.80 | 1.8e-02 |
| Nkx1-1\_3856 |  | ceh-30 | 20 | 2.26 | 1.8e-02 |
| Nkx6-3\_3446 |  | cog-1 (0.51) | 29 | 1.80 | 1.9e-02 |
| Hoxa10\_2318 |  | ceh-24 | 31 | 1.73 | 1.9e-02 |
| MA0173.1 |  | irx-1 hlh-32 D1081.8 | 35 | 1.61 | 1.9e-02 |
| pTH6071 |  | C33G8.2 | 9 | 4.28 | 1.9e-02 |
| pTH5250 |  | C48E7.11 | 15 | 2.74 | 1.9e-02 |
| Lhx1\_2240 |  | lim-7 | 29 | 1.79 | 1.9e-02 |
| Hlxb9\_3422 |  | ceh-12 | 30 | 1.76 | 2.0e-02 |
| MA0095.2 |  | lsy-2 | 25 | 1.95 | 2.0e-02 |
| Hoxb5\_3122 |  | lin-39 | 30 | 1.76 | 2.0e-02 |
| pTH1049 |  | elt-1 | 24 | 2.00 | 2.0e-02 |
| pTH10811 |  | nhr-216 (0.56) | 37 | 1.56 | 2.1e-02 |
| Hoxd1\_3448 |  | ceh-12 | 29 | 1.79 | 2.1e-02 |
| pTH10779 |  | nhr-182 (0.61) | 35 | 1.60 | 2.1e-02 |
| Elf3 |  | C24A1.2 | 17 | 2.48 | 2.1e-02 |
| pTH5882 |  | nhr-19 nhr-2 | 30 | 1.75 | 2.1e-02 |
| Zic1\_0991 |  | ref-2 (0.67) T22C8.4 | 25 | 1.94 | 2.1e-02 |
| Plagl1\_0972 |  | Y53H1A.2 (-0.56) | 14 | 2.85 | 2.1e-02 |
| Hoxd13\_2356 |  | pal-1 | 13 | 3.01 | 2.2e-02 |
| Etv3 |  | lin-1 | 24 | 1.98 | 2.2e-02 |
| CXXC1\_si |  | F52B11.1 | 10 | 3.76 | 2.2e-02 |
| pTH9880 |  | end-1 | 22 | 2.09 | 2.2e-02 |
| pTH8985 |  | athp-1 | 37 | 1.55 | 2.2e-02 |
| Mv109 |  | pax-3 pax-2 | 27 | 1.85 | 2.3e-02 |
| Irx2\_0900 |  | irx-1 | 28 | 1.81 | 2.3e-02 |
| pTH5781 |  | ceh-32 | 32 | 1.68 | 2.3e-02 |
| CG8765\_SANGER\_5\_FBgn0036900 |  | H20J04.3 | 25 | 1.93 | 2.3e-02 |
| pTH10769 |  | Y48G1C.6 | 10 | 3.73 | 2.3e-02 |
| V$CDPCR1\_01 |  | ceh-48 | 30 | 1.74 | 2.4e-02 |
| pTH10823 |  | B0310.2 | 27 | 1.84 | 2.4e-02 |
| V$XFD3\_01 |  | let-381 ceh-20 | 27 | 1.84 | 2.5e-02 |
| F$MCM1\_01 |  | unc-120 C24A1.2 | 26 | 1.87 | 2.5e-02 |
| MA0118.1 |  | ref-2 (0.67) | 19 | 2.26 | 2.5e-02 |
| pTH9297 |  | ceh-18 | 32 | 1.67 | 2.5e-02 |
| Hoxb4\_2627 |  | lin-39 | 32 | 1.67 | 2.5e-02 |
| V$GATA1\_01 |  | elt-1 | 24 | 1.96 | 2.6e-02 |
| RORA\_2 |  | nhr-213 (0.54) | 18 | 2.33 | 2.6e-02 |
| pTH9282 |  | attf-1 C01B12.2 | 22 | 2.06 | 2.6e-02 |
| pTH10768 |  | med-2 | 20 | 2.18 | 2.6e-02 |
| pTH9108 |  | daf-12 (0.72) | 24 | 1.95 | 2.7e-02 |
| pTH9244 |  | tbx-39 | 24 | 1.95 | 2.7e-02 |
| pTH6449 |  | ceh-43 | 17 | 2.41 | 2.8e-02 |
| pTH9387 |  | C34D1.1 | 25 | 1.90 | 2.8e-02 |
| pTH10013 |  | nhr-168 | 20 | 2.17 | 2.8e-02 |
| HLH29 |  | hlh-28 | 27 | 1.81 | 2.9e-02 |
| pTH9150 |  | odd-1 | 27 | 1.81 | 3.0e-02 |
| pTH9709 |  | die-1 | 23 | 1.97 | 3.1e-02 |
| Six4\_2860 |  | ceh-32 | 18 | 2.28 | 3.2e-02 |
| Hoxd11\_3873 |  | php-3 | 31 | 1.67 | 3.2e-02 |
| MA0164.1 |  | nhr-100 | 22 | 2.01 | 3.3e-02 |
| BARHL2\_1 |  | ceh-31 | 8 | 4.33 | 3.3e-02 |
| Spt15 |  | tbp-1 | 14 | 2.68 | 3.4e-02 |
| V$RFX1\_02 |  | F52B5.7 daf-19 | 30 | 1.69 | 3.4e-02 |
| pTH6447 |  | ceh-19 | 26 | 1.83 | 3.4e-02 |
| pTH8649 |  | mbr-1 | 33 | 1.61 | 3.4e-02 |
| pTH8318 |  | attf-1 | 22 | 2.01 | 3.4e-02 |
| V$CDP\_01 |  | ceh-48 | 36 | 1.53 | 3.5e-02 |
| pTH9049 |  | ztf-2 | 35 | 1.56 | 3.5e-02 |
| Etv6 |  | C24A1.2 | 16 | 2.43 | 3.5e-02 |
| pTH2193 |  | nhr-2 | 28 | 1.74 | 3.7e-02 |
| pTH10721 |  | ztf-9 | 31 | 1.65 | 3.8e-02 |
| pTH6556 |  | lim-6 | 23 | 1.94 | 3.8e-02 |
| pTH10798 |  | Y75B8A.6 | 21 | 2.04 | 3.8e-02 |
| pTH6508 |  | nhr-36 | 35 | 1.55 | 3.8e-02 |
| pTH3998 |  | tbx-39 | 18 | 2.23 | 3.9e-02 |
| HXD10\_f1 |  | php-3 | 18 | 2.23 | 3.9e-02 |
| pTH3064 |  | crh-1 | 28 | 1.74 | 4.0e-02 |
| pTH6425 |  | ceh-20 | 40 | 1.44 | 4.0e-02 |
| pTH9220 |  | mbr-1 | 33 | 1.59 | 4.0e-02 |
| PAX8\_f1 |  | pax-2 | 28 | 1.73 | 4.0e-02 |
| Nsy-7 |  | hmg-5 nsy-7 | 28 | 1.73 | 4.0e-02 |
| pTH9969 |  | pag-3 (0.57) | 37 | 1.50 | 4.1e-02 |
| pTH8863 |  | hmg-12 (-0.52) | 29 | 1.70 | 4.1e-02 |
| pTH10038 |  | gei-3 F56D1.1 | 37 | 1.49 | 4.2e-02 |
| pTH3997 |  | C04F5.9 | 25 | 1.83 | 4.3e-02 |
| Bsx\_3483 |  | ceh-31 | 25 | 1.83 | 4.4e-02 |
| pTH9182 |  | tbx-39 | 23 | 1.91 | 4.5e-02 |
| pTH3751 |  | tbx-39 | 24 | 1.86 | 4.5e-02 |
| V$FREAC7\_01 |  | lin-31 | 30 | 1.66 | 4.5e-02 |
| pTH9884 |  | tbx-39 | 50 | 1.24 | 4.7e-02 |
| Hoxa9\_2622 |  | lin-39 | 29 | 1.68 | 4.9e-02 |
| pTH9279 |  | Y116A8C.22 | 28 | 1.71 | 4.9e-02 |
| pTH9242 |  | mel-28 | 38 | 1.46 | 4.9e-02 |

### Correlated (and anti-correlated) transcription factors

|  |  |
| --- | --- |
| **Transcription factor** | **Correlation** |
| nhr-35 | 0.89 |
| ham-2 | 0.86 |
| egl-18 | 0.83 |
| nhr-143 | 0.81 |
| egl-43 | 0.81 |
| elt-6 | 0.80 |
| hlh-30 | 0.80 |
| nhr-136 | 0.77 |
| peb-1 | 0.77 |
| ztf-16 | 0.75 |
| nhr-201 | 0.75 |
| nhr-34 | 0.75 |
| nhr-98 | 0.74 |
| fkh-7 | 0.73 |
| daf-12 | 0.72 |
| nhr-243 | 0.72 |
| nhr-123 | 0.72 |
| ztf-27 | 0.72 |
| nhr-44 | 0.72 |
| sma-2 | 0.71 |
| nhr-12 | 0.71 |
| fkh-9 | 0.71 |
| nhr-154 | 0.71 |
| nhr-186 | 0.71 |
| bed-2 | 0.70 |
| nhr-197 | -0.46 |
| spe-44 | -0.47 |
| nhr-268 | -0.48 |
| unc-3 | -0.48 |
| lim-4 | -0.49 |
| C01F6.9 | -0.49 |
| F21D5.9 | -0.49 |
| fkh-2 | -0.49 |
| madf-10 | -0.51 |
| Y56A3A.18 | -0.51 |
| hmg-12 | -0.52 |
| mxl-2 | -0.53 |
| T26A5.8 | -0.53 |
| mbf-1 | -0.55 |
| pie-1 | -0.56 |
| him-8 | -0.56 |
| Y53H1A.2 | -0.56 |
| mxl-1 | -0.57 |
| cey-2 | -0.58 |
| lir-3 | -0.59 |
| ztf-4 | -0.59 |
| dhhc-7 | -0.59 |
| zip-4 | -0.63 |
| C09F5.3 | -0.68 |
| ccch-3 | -0.74 |

### ChIP peaks enriched

|  |  |  |  |  |
| --- | --- | --- | --- | --- |
| **Gene** | **Experiment** | **Number of upstream peaks** | **Enrichment** | **FDR corrected p** |
| ham-1 | HAM-1\_Fed-L1-stage-larvae | 17 | 3.89 | 4.3e-05 |
| fos-1 | FOS-1\_Larvae-L2-stage | 18 | 3.17 | 3.0e-04 |
| pha-4 | PHA-4\_Larvae-L4-stage | 12 | 4.38 | 6.7e-04 |
| lsy-2 | LSY-2\_Larvae-L1-stage | 18 | 2.90 | 9.3e-04 |
| nhr-28 | NHR-28\_Larvae-L4-stage | 18 | 2.90 | 9.3e-04 |
| hpl-2 | HPL-2\_Fed-L1-stage-larvae | 16 | 3.13 | 1.2e-03 |
| lsy-2 | LSY-2\_Fed-L1-stage-larvae | 5 | 12.30 | 2.4e-03 |
| hlh-30 | HLH-30\_Larvae-L4-stage | 10 | 4.47 | 2.9e-03 |
| dve-1 | DVE-1\_Larvae-L4-stage | 10 | 4.15 | 5.0e-03 |
| C34F6.9 | C34F6.9\_Larvae-L2-stage | 14 | 2.86 | 9.5e-03 |
| pha-4 | PHA-4\_Larvae-L2-stage | 15 | 2.68 | 1.1e-02 |
| ceh-38 | CEH-38\_Larvae-L3-stage | 3 | 22.83 | 1.2e-02 |
| jun-1 | JUN-1\_Larvae-L1-stage | 4 | 11.99 | 1.3e-02 |
| nhr-2 | NHR-2\_Embryos | 8 | 4.25 | 1.9e-02 |
| C01B12.2 | C01B12.2\_Larvae-L2-stage | 14 | 2.63 | 2.0e-02 |
| sax-3 | SAX-3\_Larvae-L3-stage | 11 | 2.92 | 3.6e-02 |
| nhr-6 | NHR-6\_Larvae-L4-stage | 8 | 3.77 | 3.8e-02 |
| ceh-38 | CEH-38\_Larvae-L4-stage | 8 | 3.76 | 3.8e-02 |
| nfya-1 | NFYA-1\_Late-Embryos | 12 | 2.67 | 4.3e-02 |
| pes-1 | PES-1\_Larvae-L4-stage | 12 | 2.67 | 4.3e-02 |
