# Supplementary material for: Overlapping cell population expression profiling and regulatory inference in C. elegans
Source: BMC Genomics. 2016 Feb 29;17:159. doi: 10.1186/s12864-016-2482-z (PMC4772325; doi:10.1186/s12864-016-2482-z)
Supplement: Additional file 13: — Web supplement. (DOC 21 kb) [file 12864_2016_2482_MOESM13_ESM.zip › sortWeb/clusters/hier.300.clusters/272.html]

Cluster 272 

## Cluster 272

### Expression

| cnd-1 rep. 1 | cnd-1 rep. 2 | cnd-1 rep. 3 | pha-4 rep. 1 | pha-4 rep. 2 | pha-4 rep. 3 | ceh-27 | ceh-36 | ceh-6 | F21D5.9 | mir-57 | mls-2 | pal-1 | pros-1 | ttx-3 | unc-130 | hlh-16 | irx-1 | ceh-6 (+) hlh-16 (+) | ceh-6 (+) hlh-16 (-) | ceh-6 (-) hlh-16 (+) | cnd-1 singlets | pha-4 singlets | 0 | 60 | 120 | 150 | 180 | 240 | 330 | 390 | 420 | 480 | 540 | 570 | 600 | 630 | 660 | NAME | Functional description |
| --- | --- | --- | --- | --- | --- | --- | --- | --- | --- | --- | --- | --- | --- | --- | --- | --- | --- | --- | --- | --- | --- | --- | --- | --- | --- | --- | --- | --- | --- | --- | --- | --- | --- | --- | --- | --- | --- | --- | --- |
|  |  |  |  |  |  |  |  |  |  |  |  |  |  |  |  |  |  |  |  |  |  |  |  |  |  |  |  |  |  |  |  |  |  |  |  |  |  | K03H9.14 |  |
|  |  |  |  |  |  |  |  |  |  |  |  |  |  |  |  |  |  |  |  |  |  |  |  |  |  |  |  |  |  |  |  |  |  |  |  |  |  | F53B7.12 |  |
|  |  |  |  |  |  |  |  |  |  |  |  |  |  |  |  |  |  |  |  |  |  |  |  |  |  |  |  |  |  |  |  |  |  |  |  |  |  | *nhr-168* | Nuclear Hormone Receptor family |
|  |  |  |  |  |  |  |  |  |  |  |  |  |  |  |  |  |  |  |  |  |  |  |  |  |  |  |  |  |  |  |  |  |  |  |  |  |  | ZK1128.3 |  |
|  |  |  |  |  |  |  |  |  |  |  |  |  |  |  |  |  |  |  |  |  |  |  |  |  |  |  |  |  |  |  |  |  |  |  |  |  |  | C39H7.2 |  |
|  |  |  |  |  |  |  |  |  |  |  |  |  |  |  |  |  |  |  |  |  |  |  |  |  |  |  |  |  |  |  |  |  |  |  |  |  |  | Y22D7AL.15 |  |
|  |  |  |  |  |  |  |  |  |  |  |  |  |  |  |  |  |  |  |  |  |  |  |  |  |  |  |  |  |  |  |  |  |  |  |  |  |  | *hex-5* | HEXosaminidase |
|  |  |  |  |  |  |  |  |  |  |  |  |  |  |  |  |  |  |  |  |  |  |  |  |  |  |  |  |  |  |  |  |  |  |  |  |  |  | *fhod-1* | Formin HOmology Domain |
|  |  |  |  |  |  |  |  |  |  |  |  |  |  |  |  |  |  |  |  |  |  |  |  |  |  |  |  |  |  |  |  |  |  |  |  |  |  | *nhr-44* | Nuclear Hormone Receptor family |
|  |  |  |  |  |  |  |  |  |  |  |  |  |  |  |  |  |  |  |  |  |  |  |  |  |  |  |  |  |  |  |  |  |  |  |  |  |  | *nhr-143* | Nuclear Hormone Receptor family |
|  |  |  |  |  |  |  |  |  |  |  |  |  |  |  |  |  |  |  |  |  |  |  |  |  |  |  |  |  |  |  |  |  |  |  |  |  |  | C04G6.4 |  |
|  |  |  |  |  |  |  |  |  |  |  |  |  |  |  |  |  |  |  |  |  |  |  |  |  |  |  |  |  |  |  |  |  |  |  |  |  |  | *daf-14* | abnormal DAuer Formation |
|  |  |  |  |  |  |  |  |  |  |  |  |  |  |  |  |  |  |  |  |  |  |  |  |  |  |  |  |  |  |  |  |  |  |  |  |  |  | *cdr-4* | CaDmium Responsive |
|  |  |  |  |  |  |  |  |  |  |  |  |  |  |  |  |  |  |  |  |  |  |  |  |  |  |  |  |  |  |  |  |  |  |  |  |  |  | Y59H11AR.9 |  |
|  |  |  |  |  |  |  |  |  |  |  |  |  |  |  |  |  |  |  |  |  |  |  |  |  |  |  |  |  |  |  |  |  |  |  |  |  |  | *linc-151* | Long Intervening Non-Coding RNA |
|  |  |  |  |  |  |  |  |  |  |  |  |  |  |  |  |  |  |  |  |  |  |  |  |  |  |  |  |  |  |  |  |  |  |  |  |  |  | Y4C6B.5 |  |
|  |  |  |  |  |  |  |  |  |  |  |  |  |  |  |  |  |  |  |  |  |  |  |  |  |  |  |  |  |  |  |  |  |  |  |  |  |  | F54C8.6 |  |
|  |  |  |  |  |  |  |  |  |  |  |  |  |  |  |  |  |  |  |  |  |  |  |  |  |  |  |  |  |  |  |  |  |  |  |  |  |  | *oac-39* | O-ACyltransferase homolog |
|  |  |  |  |  |  |  |  |  |  |  |  |  |  |  |  |  |  |  |  |  |  |  |  |  |  |  |  |  |  |  |  |  |  |  |  |  |  | F13H8.6 |  |
|  |  |  |  |  |  |  |  |  |  |  |  |  |  |  |  |  |  |  |  |  |  |  |  |  |  |  |  |  |  |  |  |  |  |  |  |  |  | Y7A5A.6 |  |
|  |  |  |  |  |  |  |  |  |  |  |  |  |  |  |  |  |  |  |  |  |  |  |  |  |  |  |  |  |  |  |  |  |  |  |  |  |  | M151.7 |  |
|  |  |  |  |  |  |  |  |  |  |  |  |  |  |  |  |  |  |  |  |  |  |  |  |  |  |  |  |  |  |  |  |  |  |  |  |  |  | D2092.10 |  |
|  |  |  |  |  |  |  |  |  |  |  |  |  |  |  |  |  |  |  |  |  |  |  |  |  |  |  |  |  |  |  |  |  |  |  |  |  |  | M04D8.7 |  |
|  |  |  |  |  |  |  |  |  |  |  |  |  |  |  |  |  |  |  |  |  |  |  |  |  |  |  |  |  |  |  |  |  |  |  |  |  |  | *srh-98* | Serpentine Receptor, class H |
|  |  |  |  |  |  |  |  |  |  |  |  |  |  |  |  |  |  |  |  |  |  |  |  |  |  |  |  |  |  |  |  |  |  |  |  |  |  | *linc-104* | Long Intervening Non-Coding RNA |
|  |  |  |  |  |  |  |  |  |  |  |  |  |  |  |  |  |  |  |  |  |  |  |  |  |  |  |  |  |  |  |  |  |  |  |  |  |  | C34D4.2 |  |

### Phenotypes enriched

none found

### Anatomy terms enriched

none found

### GO terms enriched

none found

### Expression clusters enriched

none found

### Motifs enriched

|  |  |  |  |  |  |
| --- | --- | --- | --- | --- | --- |
| **Motif** | **Logo** | **Possible orthologs** | **Number of motifs in cluster** | **Enrichment** | **FDR corrected p** |
| NHLH1\_2 |  | hlh-15 | 14 | 2.99 | 0.0052 |
| pTH9194 |  | daf-19 | 6 | 8.92 | 0.0055 |
| Ascl2\_1 |  | hlh-14 | 10 | 4.29 | 0.0057 |
| V$FREAC7\_01 |  | lin-31 | 22 | 1.83 | 0.0070 |
| Rfxdc2\_3516 |  | daf-19 | 6 | 7.68 | 0.0100 |
| FKH2\_4517 |  | let-381 fkh-10 | 25 | 1.47 | 0.0210 |
| pTH6251 |  | ceh-12 ceh-18 | 25 | 1.46 | 0.0220 |
| V$FOXO4\_01 |  | lin-31 daf-16 | 25 | 1.44 | 0.0280 |
| pTH2673 |  | let-381 | 24 | 1.52 | 0.0280 |
| HLH10 |  | hlh-10 | 7 | 4.81 | 0.0320 |
| FOXF1\_f1 |  | lin-31 let-381 | 26 | 1.34 | 0.0330 |
| MEF2B\_1 |  | mef-2 | 4 | 10.48 | 0.0370 |
| MA0484.1 |  | nhr-62 (0.65) | 10 | 3.08 | 0.0460 |
| HXB6\_f1 |  | lin-39 | 8 | 3.81 | 0.0470 |
| RFX5\_1 |  | daf-19 | 7 | 4.38 | 0.0480 |
| srp\_SANGER\_5\_FBgn0003507 |  | elt-1 (-0.56) | 15 | 2.17 | 0.0480 |

### Correlated (and anti-correlated) transcription factors

|  |  |
| --- | --- |
| **Transcription factor** | **Correlation** |
| nhr-143 | 0.88 |
| nhr-44 | 0.88 |
| egl-43 | 0.82 |
| nhr-129 | 0.79 |
| nhr-182 | 0.78 |
| nhr-10 | 0.76 |
| nhr-132 | 0.75 |
| nhr-96 | 0.75 |
| nhr-70 | 0.73 |
| nhr-134 | 0.72 |
| nhr-49 | 0.72 |
| ztf-27 | 0.72 |
| Y22D7AL.16 | 0.71 |
| nhr-168 | 0.71 |
| nhr-46 | 0.70 |
| nhr-183 | 0.69 |
| nhr-230 | 0.69 |
| nhr-98 | 0.68 |
| nhr-34 | 0.67 |
| fkh-9 | 0.67 |
| atf-5 | 0.66 |
| nhr-184 | 0.66 |
| nhr-153 | 0.66 |
| nhr-102 | 0.65 |
| nhr-62 | 0.65 |
| Y53H1A.2 | -0.45 |
| dhhc-10 | -0.46 |
| ztf-28 | -0.46 |
| zip-8 | -0.46 |
| ceh-13 | -0.47 |
| unc-62 | -0.47 |
| mig-5 | -0.49 |
| lin-39 | -0.49 |
| lim-4 | -0.50 |
| hlh-32 | -0.51 |
| hlh-17 | -0.51 |
| mbl-1 | -0.52 |
| cey-2 | -0.54 |
| cep-1 | -0.54 |
| zag-1 | -0.56 |
| elt-1 | -0.56 |
| cnd-1 | -0.58 |
| bar-1 | -0.58 |
| ztf-4 | -0.59 |
| hmg-12 | -0.60 |
| ccch-3 | -0.61 |
| C09F5.3 | -0.61 |
| ceh-5 | -0.62 |
| F21D5.9 | -0.64 |
| unc-3 | -0.66 |

### ChIP peaks enriched

none found
