# Supplementary material for: Overlapping cell population expression profiling and regulatory inference in C. elegans
Source: BMC Genomics. 2016 Feb 29;17:159. doi: 10.1186/s12864-016-2482-z (PMC4772325; doi:10.1186/s12864-016-2482-z)
Supplement: Additional file 13: — Web supplement. (DOC 21 kb) [file 12864_2016_2482_MOESM13_ESM.zip › sortWeb/clusters/hier.300.clusters/273.html]

Cluster 273 

## Cluster 273

### Expression

| cnd-1 rep. 1 | cnd-1 rep. 2 | cnd-1 rep. 3 | pha-4 rep. 1 | pha-4 rep. 2 | pha-4 rep. 3 | ceh-27 | ceh-36 | ceh-6 | F21D5.9 | mir-57 | mls-2 | pal-1 | pros-1 | ttx-3 | unc-130 | hlh-16 | irx-1 | ceh-6 (+) hlh-16 (+) | ceh-6 (+) hlh-16 (-) | ceh-6 (-) hlh-16 (+) | cnd-1 singlets | pha-4 singlets | 0 | 60 | 120 | 150 | 180 | 240 | 330 | 390 | 420 | 480 | 540 | 570 | 600 | 630 | 660 | NAME | Functional description |
| --- | --- | --- | --- | --- | --- | --- | --- | --- | --- | --- | --- | --- | --- | --- | --- | --- | --- | --- | --- | --- | --- | --- | --- | --- | --- | --- | --- | --- | --- | --- | --- | --- | --- | --- | --- | --- | --- | --- | --- |
|  |  |  |  |  |  |  |  |  |  |  |  |  |  |  |  |  |  |  |  |  |  |  |  |  |  |  |  |  |  |  |  |  |  |  |  |  |  | *vglu-2* | Vesicular GLUtamate transporter |
|  |  |  |  |  |  |  |  |  |  |  |  |  |  |  |  |  |  |  |  |  |  |  |  |  |  |  |  |  |  |  |  |  |  |  |  |  |  | *pbo-5* | PBOc defective (defecation) |
|  |  |  |  |  |  |  |  |  |  |  |  |  |  |  |  |  |  |  |  |  |  |  |  |  |  |  |  |  |  |  |  |  |  |  |  |  |  | R11.3 |  |
|  |  |  |  |  |  |  |  |  |  |  |  |  |  |  |  |  |  |  |  |  |  |  |  |  |  |  |  |  |  |  |  |  |  |  |  |  |  | F35D2.3 |  |
|  |  |  |  |  |  |  |  |  |  |  |  |  |  |  |  |  |  |  |  |  |  |  |  |  |  |  |  |  |  |  |  |  |  |  |  |  |  | F35D2.2 |  |
|  |  |  |  |  |  |  |  |  |  |  |  |  |  |  |  |  |  |  |  |  |  |  |  |  |  |  |  |  |  |  |  |  |  |  |  |  |  | K10D6.2 |  |
|  |  |  |  |  |  |  |  |  |  |  |  |  |  |  |  |  |  |  |  |  |  |  |  |  |  |  |  |  |  |  |  |  |  |  |  |  |  | *ccch-1* | CCCH-type zinc finger putative transcription factor |
|  |  |  |  |  |  |  |  |  |  |  |  |  |  |  |  |  |  |  |  |  |  |  |  |  |  |  |  |  |  |  |  |  |  |  |  |  |  | *nhr-91* | Nuclear Hormone Receptor family |
|  |  |  |  |  |  |  |  |  |  |  |  |  |  |  |  |  |  |  |  |  |  |  |  |  |  |  |  |  |  |  |  |  |  |  |  |  |  | *gmeb-1* | GMEB (Glucocorticoid Modulatory Element Binding protein) transcriptional regulator homolog |
|  |  |  |  |  |  |  |  |  |  |  |  |  |  |  |  |  |  |  |  |  |  |  |  |  |  |  |  |  |  |  |  |  |  |  |  |  |  | F14F9.3 |  |
|  |  |  |  |  |  |  |  |  |  |  |  |  |  |  |  |  |  |  |  |  |  |  |  |  |  |  |  |  |  |  |  |  |  |  |  |  |  | K12G11.6 |  |
|  |  |  |  |  |  |  |  |  |  |  |  |  |  |  |  |  |  |  |  |  |  |  |  |  |  |  |  |  |  |  |  |  |  |  |  |  |  | *mltn-13* | MLt-TeN (mlt-10) related |
|  |  |  |  |  |  |  |  |  |  |  |  |  |  |  |  |  |  |  |  |  |  |  |  |  |  |  |  |  |  |  |  |  |  |  |  |  |  | T01D3.7 |  |
|  |  |  |  |  |  |  |  |  |  |  |  |  |  |  |  |  |  |  |  |  |  |  |  |  |  |  |  |  |  |  |  |  |  |  |  |  |  | C26G2.2 |  |
|  |  |  |  |  |  |  |  |  |  |  |  |  |  |  |  |  |  |  |  |  |  |  |  |  |  |  |  |  |  |  |  |  |  |  |  |  |  | *mfb-1* | MAFBx (F-box) protein homolog |
|  |  |  |  |  |  |  |  |  |  |  |  |  |  |  |  |  |  |  |  |  |  |  |  |  |  |  |  |  |  |  |  |  |  |  |  |  |  | Y59H11AR.6 |  |
|  |  |  |  |  |  |  |  |  |  |  |  |  |  |  |  |  |  |  |  |  |  |  |  |  |  |  |  |  |  |  |  |  |  |  |  |  |  | C47A4.3 |  |
|  |  |  |  |  |  |  |  |  |  |  |  |  |  |  |  |  |  |  |  |  |  |  |  |  |  |  |  |  |  |  |  |  |  |  |  |  |  | E01G6.2 |  |
|  |  |  |  |  |  |  |  |  |  |  |  |  |  |  |  |  |  |  |  |  |  |  |  |  |  |  |  |  |  |  |  |  |  |  |  |  |  | F45E12.6 |  |
|  |  |  |  |  |  |  |  |  |  |  |  |  |  |  |  |  |  |  |  |  |  |  |  |  |  |  |  |  |  |  |  |  |  |  |  |  |  | *col-61* | COLlagen |
|  |  |  |  |  |  |  |  |  |  |  |  |  |  |  |  |  |  |  |  |  |  |  |  |  |  |  |  |  |  |  |  |  |  |  |  |  |  | R10E11.5 |  |
|  |  |  |  |  |  |  |  |  |  |  |  |  |  |  |  |  |  |  |  |  |  |  |  |  |  |  |  |  |  |  |  |  |  |  |  |  |  | C30H6.5 |  |
|  |  |  |  |  |  |  |  |  |  |  |  |  |  |  |  |  |  |  |  |  |  |  |  |  |  |  |  |  |  |  |  |  |  |  |  |  |  | C08G5.6 |  |
|  |  |  |  |  |  |  |  |  |  |  |  |  |  |  |  |  |  |  |  |  |  |  |  |  |  |  |  |  |  |  |  |  |  |  |  |  |  | *swt-3* | SWEET sugar transporter family |
|  |  |  |  |  |  |  |  |  |  |  |  |  |  |  |  |  |  |  |  |  |  |  |  |  |  |  |  |  |  |  |  |  |  |  |  |  |  | *hacd-1* | Hydroxy-Acyl-CoA Dehydrogenase |
|  |  |  |  |  |  |  |  |  |  |  |  |  |  |  |  |  |  |  |  |  |  |  |  |  |  |  |  |  |  |  |  |  |  |  |  |  |  | *col-105* | COLlagen |
|  |  |  |  |  |  |  |  |  |  |  |  |  |  |  |  |  |  |  |  |  |  |  |  |  |  |  |  |  |  |  |  |  |  |  |  |  |  | C24A3.4 |  |
|  |  |  |  |  |  |  |  |  |  |  |  |  |  |  |  |  |  |  |  |  |  |  |  |  |  |  |  |  |  |  |  |  |  |  |  |  |  | *pept-3* | PEPTide transporter family |
|  |  |  |  |  |  |  |  |  |  |  |  |  |  |  |  |  |  |  |  |  |  |  |  |  |  |  |  |  |  |  |  |  |  |  |  |  |  | M03A1.3 |  |
|  |  |  |  |  |  |  |  |  |  |  |  |  |  |  |  |  |  |  |  |  |  |  |  |  |  |  |  |  |  |  |  |  |  |  |  |  |  | C23H5.11 |  |
|  |  |  |  |  |  |  |  |  |  |  |  |  |  |  |  |  |  |  |  |  |  |  |  |  |  |  |  |  |  |  |  |  |  |  |  |  |  | *nhr-221* | Nuclear Hormone Receptor family |
|  |  |  |  |  |  |  |  |  |  |  |  |  |  |  |  |  |  |  |  |  |  |  |  |  |  |  |  |  |  |  |  |  |  |  |  |  |  | R12E2.6 |  |
|  |  |  |  |  |  |  |  |  |  |  |  |  |  |  |  |  |  |  |  |  |  |  |  |  |  |  |  |  |  |  |  |  |  |  |  |  |  | F26A1.8 |  |
|  |  |  |  |  |  |  |  |  |  |  |  |  |  |  |  |  |  |  |  |  |  |  |  |  |  |  |  |  |  |  |  |  |  |  |  |  |  | *jud-4* | JUDang (Korean for resistant to alcohol) |
|  |  |  |  |  |  |  |  |  |  |  |  |  |  |  |  |  |  |  |  |  |  |  |  |  |  |  |  |  |  |  |  |  |  |  |  |  |  | *arrd-5* | ARRestin Domain protein |
|  |  |  |  |  |  |  |  |  |  |  |  |  |  |  |  |  |  |  |  |  |  |  |  |  |  |  |  |  |  |  |  |  |  |  |  |  |  | F35B12.11 |  |
|  |  |  |  |  |  |  |  |  |  |  |  |  |  |  |  |  |  |  |  |  |  |  |  |  |  |  |  |  |  |  |  |  |  |  |  |  |  | *hpo-39* | Hypersensitive to POre-forming toxin |
|  |  |  |  |  |  |  |  |  |  |  |  |  |  |  |  |  |  |  |  |  |  |  |  |  |  |  |  |  |  |  |  |  |  |  |  |  |  | C41A3.1 |  |
|  |  |  |  |  |  |  |  |  |  |  |  |  |  |  |  |  |  |  |  |  |  |  |  |  |  |  |  |  |  |  |  |  |  |  |  |  |  | C43C3.11 |  |
|  |  |  |  |  |  |  |  |  |  |  |  |  |  |  |  |  |  |  |  |  |  |  |  |  |  |  |  |  |  |  |  |  |  |  |  |  |  | F48C5.2 |  |
|  |  |  |  |  |  |  |  |  |  |  |  |  |  |  |  |  |  |  |  |  |  |  |  |  |  |  |  |  |  |  |  |  |  |  |  |  |  | F26A1.9 |  |
|  |  |  |  |  |  |  |  |  |  |  |  |  |  |  |  |  |  |  |  |  |  |  |  |  |  |  |  |  |  |  |  |  |  |  |  |  |  | F33D4.6 |  |
|  |  |  |  |  |  |  |  |  |  |  |  |  |  |  |  |  |  |  |  |  |  |  |  |  |  |  |  |  |  |  |  |  |  |  |  |  |  | F26A1.3 |  |
|  |  |  |  |  |  |  |  |  |  |  |  |  |  |  |  |  |  |  |  |  |  |  |  |  |  |  |  |  |  |  |  |  |  |  |  |  |  | *spe-29* | defective SPErmatogenesis |
|  |  |  |  |  |  |  |  |  |  |  |  |  |  |  |  |  |  |  |  |  |  |  |  |  |  |  |  |  |  |  |  |  |  |  |  |  |  | C27A2.7 |  |
|  |  |  |  |  |  |  |  |  |  |  |  |  |  |  |  |  |  |  |  |  |  |  |  |  |  |  |  |  |  |  |  |  |  |  |  |  |  | *amt-2* | AMmonium Transporter homolog |
|  |  |  |  |  |  |  |  |  |  |  |  |  |  |  |  |  |  |  |  |  |  |  |  |  |  |  |  |  |  |  |  |  |  |  |  |  |  | Y45F10D.15 |  |
|  |  |  |  |  |  |  |  |  |  |  |  |  |  |  |  |  |  |  |  |  |  |  |  |  |  |  |  |  |  |  |  |  |  |  |  |  |  | *dnj-25* | DNaJ domain (prokaryotic heat shock protein) |
|  |  |  |  |  |  |  |  |  |  |  |  |  |  |  |  |  |  |  |  |  |  |  |  |  |  |  |  |  |  |  |  |  |  |  |  |  |  | *nhr-230* | Nuclear Hormone Receptor family |
|  |  |  |  |  |  |  |  |  |  |  |  |  |  |  |  |  |  |  |  |  |  |  |  |  |  |  |  |  |  |  |  |  |  |  |  |  |  | R11F4.2 |  |
|  |  |  |  |  |  |  |  |  |  |  |  |  |  |  |  |  |  |  |  |  |  |  |  |  |  |  |  |  |  |  |  |  |  |  |  |  |  | *acy-2* | Adenylyl CYclase |
|  |  |  |  |  |  |  |  |  |  |  |  |  |  |  |  |  |  |  |  |  |  |  |  |  |  |  |  |  |  |  |  |  |  |  |  |  |  | *ptr-22* | PaTched Related family |
|  |  |  |  |  |  |  |  |  |  |  |  |  |  |  |  |  |  |  |  |  |  |  |  |  |  |  |  |  |  |  |  |  |  |  |  |  |  | F10G8.2 |  |
|  |  |  |  |  |  |  |  |  |  |  |  |  |  |  |  |  |  |  |  |  |  |  |  |  |  |  |  |  |  |  |  |  |  |  |  |  |  | *grl-25* | GRound-Like (grd related) |

### Phenotypes enriched

none found

### Anatomy terms enriched

none found

### GO terms enriched

none found

### Expression clusters enriched

none found

### Motifs enriched

|  |  |  |  |  |  |
| --- | --- | --- | --- | --- | --- |
| **Motif** | **Logo** | **Possible orthologs** | **Number of motifs in cluster** | **Enrichment** | **FDR corrected p** |
| Otp\_3496 |  | ceh-43 (0.57) alr-1 ceh-1 ceh-18 npax-3 ceh-53 lin-39 ceh-12 ceh-45 | 47 | 1.78 | 2.4e-06 |
| Hbn\_SOLEXA\_FBgn0008636 |  | mls-2 (0.59) ceh-43 (0.57) ceh-23 (0.51) alr-1 ceh-10 ceh-14 lim-7 dsc-1 ceh-30 ceh-1 pal-1 ceh-31 ceh-18 ceh-53 cog-1 pha-2 egl-5 lin-39 ceh-12 ceh-2 and 9 others  [full list] | 46 | 1.80 | 3.8e-06 |
| Rfx2\_2 |  | daf-19 (0.52) | 50 | 1.62 | 4.4e-06 |
| twi\_da\_SANGER\_5\_FBgn0003900 |  | hlh-8 hlh-32 | 52 | 1.50 | 9.7e-06 |
| LHX2\_f1 |  | alr-1 ceh-14 cfi-1 | 51 | 1.54 | 1.1e-05 |
| Prop1\_3949 |  | ceh-53 ceh-16 | 45 | 1.71 | 5.1e-05 |
| Mw137 |  | blmp-1 (0.54) | 34 | 2.16 | 8.6e-05 |
| Lhx1\_2240 |  | lim-7 | 49 | 1.53 | 9.6e-05 |
| Lmx1b\_3433 |  | lim-7 ceh-24 ceh-16 lim-6 | 40 | 1.84 | 1.3e-04 |
| Six6\_2267 |  | ceh-34 | 53 | 1.36 | 1.6e-04 |
| pTH6503 |  | ceh-43 (0.57) alr-1 lim-7 ceh-1 ceh-31 ceh-16 | 44 | 1.68 | 1.7e-04 |
| pTH3120 |  | che-1 K11D2.4 | 52 | 1.39 | 1.8e-04 |
| YY1\_1 |  | lsy-2 | 37 | 1.90 | 3.2e-04 |
| pTH9137 |  | nhr-65 | 25 | 2.59 | 3.4e-04 |
| PAX6\_1 |  | pax-3 (-0.53) pax-2 (0.52) | 50 | 1.44 | 3.9e-04 |
| pTH5644 |  | alr-1 ceh-53 ceh-45 dve-1 ceh-36 | 36 | 1.90 | 5.2e-04 |
| pTH10721 |  | ztf-9 ceh-18 | 14 | 4.29 | 5.8e-04 |
| Irx5\_2385 |  | irx-1 | 51 | 1.38 | 6.6e-04 |
| pTH6268 |  | ceh-2 | 49 | 1.44 | 8.5e-04 |
| pTH6408 |  | irx-1 | 49 | 1.44 | 8.7e-04 |
| YMR043W\_831 |  | unc-120 | 43 | 1.61 | 9.1e-04 |
| V$TCF11\_01 |  | skn-1 | 13 | 4.37 | 1.0e-03 |
| COT2\_f2 |  | nhr-2 (-0.57) | 39 | 1.73 | 1.1e-03 |
| CG33980\_SOLEXA\_2\_10\_FBgn0053980 |  | ceh-10 alr-1 ceh-1 lin-39 eyg-1 ceh-45 | 42 | 1.63 | 1.1e-03 |
| MA0246.1 |  | dmd-4 ceh-32 dmd-5 | 50 | 1.40 | 1.1e-03 |
| Irx2\_0900 |  | irx-1 | 50 | 1.39 | 1.1e-03 |
| pTH9215 |  | C34D1.1 | 47 | 1.48 | 1.3e-03 |
| MA0599.1 |  | ZC328.2 klf-1 klf-2 | 27 | 2.24 | 1.3e-03 |
| Pou3f1\_3819 |  | ceh-6 | 54 | 1.24 | 1.3e-03 |
| pTH9900 |  | C46E10.8 | 25 | 2.37 | 1.4e-03 |
| pTH9365 |  | ceh-18 lin-39 ceh-6 | 52 | 1.32 | 1.5e-03 |
| MA0188.1 |  | ceh-8 (0.71) ceh-43 (0.57) alr-1 ceh-30 ceh-1 ceh-31 ceh-19 cog-1 lin-39 ceh-9 | 39 | 1.71 | 1.5e-03 |
| RORA\_2 |  | nhr-118 nhr-213 | 49 | 1.41 | 1.5e-03 |
| pTH10647 |  | nhr-232 | 45 | 1.52 | 1.5e-03 |
| pTH9026 |  | attf-1 | 41 | 1.64 | 1.6e-03 |
| MA0060.2 |  | dro-1 lin-31 cey-3 nfya-2 ceh-20 | 37 | 1.77 | 1.6e-03 |
| I$DFD\_01 |  | lin-39 | 33 | 1.91 | 1.6e-03 |
| MA0224.1 |  | ceh-43 (0.57) lin-39 ceh-12 | 40 | 1.66 | 1.8e-03 |
| pTH10638 |  | dmd-3 C34D1.1 | 51 | 1.34 | 1.9e-03 |
| K562\_GATA2\_HudsonAlpha |  | alr-1 elt-1 | 37 | 1.75 | 1.9e-03 |
| YLR176C\_1478 |  | daf-19 (0.52) F52B5.7 | 44 | 1.54 | 1.9e-03 |
| pTH2353 |  | B0310.2 | 44 | 1.54 | 1.9e-03 |
| pTH9219 |  | xbp-1 C01B12.2 | 25 | 2.31 | 2.0e-03 |
| pTH3467 |  | nhr-2 (-0.57) nhr-6 nhr-68 nhr-213 nhr-71 | 41 | 1.62 | 2.1e-03 |
| pTH9165 |  | ztf-27 | 50 | 1.37 | 2.3e-03 |
| tgo\_sim\_SANGER\_5\_FBgn0015014 |  | aha-1 | 48 | 1.42 | 2.4e-03 |
| PDX1\_1 |  | ceh-43 (0.57) ceh-14 ceh-1 ceh-31 lin-39 | 45 | 1.50 | 2.5e-03 |
| OLIG3\_1 |  | hlh-8 ngn-1 hlh-32 | 51 | 1.33 | 2.7e-03 |
| Sry\_2833 |  | sox-4 gei-3 pop-1 C05C9.3 | 48 | 1.41 | 2.8e-03 |
| pTH10822 |  | hlh-10 unc-120 | 49 | 1.39 | 2.8e-03 |
| Nkx6-3\_3446 |  | cog-1 | 42 | 1.57 | 2.8e-03 |
| MA0118.1 |  | ref-2 | 44 | 1.51 | 2.9e-03 |
| pTH9387 |  | C34D1.1 | 47 | 1.43 | 3.1e-03 |
| IRX5\_1 |  | irx-1 | 33 | 1.85 | 3.1e-03 |
| MEIS2\_1 |  | ceh-32 | 52 | 1.29 | 3.3e-03 |
| Emx2\_3420 |  | ceh-2 | 50 | 1.35 | 3.3e-03 |
| sqz\_SANGER\_5\_FBgn0010768 |  | lin-29 | 45 | 1.48 | 3.4e-03 |
| pTH3997 |  | C04F5.9 | 46 | 1.45 | 3.6e-03 |
| pTH5270 |  | ngn-1 hlh-16 | 49 | 1.37 | 3.8e-03 |
| pTH5812 |  | ceh-14 | 50 | 1.34 | 3.8e-03 |
| pTH10722 |  | egrh-3 | 29 | 1.98 | 4.2e-03 |
| V$ZID\_01 |  | ztf-28 skn-1 | 31 | 1.90 | 4.2e-03 |
| Pou2f3\_3986 |  | ceh-18 | 39 | 1.63 | 4.3e-03 |
| ems\_FlyReg\_FBgn0000576 |  | ceh-2 skn-1 | 43 | 1.52 | 4.4e-03 |
| Zbtb12\_2932 |  | lsy-27 | 37 | 1.68 | 4.5e-03 |
| pTH9340 |  | tbx-39 mab-9 | 34 | 1.78 | 4.6e-03 |
| pTH9297 |  | ceh-18 | 45 | 1.46 | 4.6e-03 |
| Hoxa11\_2218 |  | php-3 | 53 | 1.25 | 4.7e-03 |
| ELK1\_2 |  | lin-1 | 17 | 2.88 | 4.9e-03 |
| Hoxc11\_3718 |  | ceh-24 | 44 | 1.48 | 5.0e-03 |
| pTH9244 |  | tbx-39 | 26 | 2.11 | 5.0e-03 |
| pTH10623 |  | scrt-1 | 49 | 1.36 | 5.1e-03 |
| Mw154 |  | lin-39 ceh-12 ceh-20 | 41 | 1.56 | 5.2e-03 |
| pTH3819 |  | ceh-18 | 53 | 1.24 | 5.4e-03 |
| Tcf3\_3787 |  | pop-1 | 47 | 1.40 | 5.6e-03 |
| pTH9216 |  | ceh-18 | 52 | 1.27 | 5.9e-03 |
| HLH4C\_da\_SANGER\_5\_FBgn0011277 |  | hlh-8 hlh-15 | 50 | 1.33 | 6.2e-03 |
| V$EN1\_01 |  | ceh-16 atf-2 | 49 | 1.35 | 6.5e-03 |
| ERG\_2 |  | lin-1 | 46 | 1.42 | 6.6e-03 |
| pTH9256 |  | ceh-18 | 45 | 1.44 | 6.9e-03 |
| V$YY1\_02 |  | lsy-2 | 20 | 2.46 | 7.2e-03 |
| pTH9198 |  | dmd-3 | 46 | 1.42 | 7.3e-03 |
| CG5669\_SOLEXA\_5\_FBgn0039169 |  | klf-1 klf-2 | 19 | 2.55 | 7.3e-03 |
| pTH8598 |  | nhr-79 nhr-273 | 28 | 1.96 | 7.4e-03 |
| PURA\_f1 |  | Y53H1A.2 plp-2 | 31 | 1.83 | 7.6e-03 |
| FOXB1\_4 |  | lin-31 let-381 daf-16 | 45 | 1.44 | 7.7e-03 |
| pTH2283 |  | odd-2 | 18 | 2.63 | 7.9e-03 |
| pTH3998 |  | tbx-39 | 26 | 2.04 | 7.9e-03 |
| PTF1A\_f1 |  | lin-32 | 33 | 1.76 | 7.9e-03 |
| V$NCX\_01 |  | ceh-19 | 26 | 2.04 | 7.9e-03 |
| pTH6445 |  | ceh-5 | 54 | 1.19 | 8.1e-03 |
| pTH3510 |  | nhr-86 | 44 | 1.46 | 8.1e-03 |
| pTH9381 |  | ceh-18 | 54 | 1.19 | 8.4e-03 |
| pTH5508 |  | nhr-2 (-0.57) nhr-19 nhr-213 | 44 | 1.45 | 8.5e-03 |
| pTH2280 |  | mnm-2 | 18 | 2.61 | 8.7e-03 |
| V$GATA3\_03 |  | nhr-100 (0.63) elt-1 | 45 | 1.43 | 9.3e-03 |
| Hr51\_SANGER\_5\_FBgn0034012 |  | nhr-100 (0.63) pop-1 | 49 | 1.33 | 9.5e-03 |
| pTH10630 |  | lsy-27 | 22 | 2.25 | 9.6e-03 |
| Cart1\_1275 |  | alr-1 lim-7 ceh-18 | 39 | 1.57 | 9.7e-03 |
| Pou2f1\_3081 |  | ceh-18 | 53 | 1.23 | 9.7e-03 |
| MA0131.1 |  | F39B2.1 | 14 | 3.13 | 1.0e-02 |
| pTH9298 |  | crh-1 attf-1 | 42 | 1.49 | 1.0e-02 |
| Nkx6-1\_2825 |  | cog-1 | 42 | 1.49 | 1.0e-02 |
| pTH5778 |  | egl-5 | 39 | 1.56 | 1.0e-02 |
| pTH10823 |  | B0310.2 | 16 | 2.81 | 1.0e-02 |
| tgo\_ss\_SANGER\_5\_FBgn0015014 |  | aha-1 | 31 | 1.80 | 1.0e-02 |
| Poxm\_SOLEXA\_5\_FBgn0003129 |  | pax-2 (0.52) | 24 | 2.10 | 1.1e-02 |
| SMAD1\_si |  | daf-8 | 19 | 2.45 | 1.1e-02 |
| MA0222.1 |  | ceh-32 ceh-20 F55C5.11 | 49 | 1.33 | 1.1e-02 |
| HMGA1\_f1 |  | Y116A8C.22 | 53 | 1.22 | 1.1e-02 |
| pTH10013 |  | nhr-168 | 43 | 1.46 | 1.1e-02 |
| V$CDXA\_01 |  | ceh-13 php-3 | 25 | 2.04 | 1.1e-02 |
| SOX2\_2 |  | sox-4 | 44 | 1.44 | 1.2e-02 |
| Hoxa2\_3079 |  | lin-39 | 51 | 1.27 | 1.2e-02 |
| SMAD3\_1 |  | daf-8 | 7 | 6.25 | 1.2e-02 |
| I$ABDB\_01 |  | ceh-24 | 18 | 2.52 | 1.3e-02 |
| pTH5561 |  | nhr-239 | 50 | 1.30 | 1.3e-02 |
| Vsx1\_1728 |  | alr-1 | 38 | 1.57 | 1.3e-02 |
| Hoxb3\_1720 |  | lin-39 | 51 | 1.27 | 1.3e-02 |
| POU3F3\_2 |  | unc-86 ceh-18 | 52 | 1.24 | 1.3e-02 |
| pTH6436 |  | ceh-53 | 49 | 1.32 | 1.3e-02 |
| FOXC2\_f1 |  | let-381 | 28 | 1.88 | 1.4e-02 |
| Hoxd1\_3448 |  | ceh-12 | 35 | 1.64 | 1.4e-02 |
| pTH9159 |  | atf-7 atf-6 | 21 | 2.24 | 1.4e-02 |
| K562b\_TR4\_UCD |  | nhr-19 lin-1 C24A1.2 | 8 | 5.16 | 1.4e-02 |
| pTH9237 |  | mel-28 | 39 | 1.54 | 1.4e-02 |
| Hlxb9\_3422 |  | ceh-12 | 50 | 1.29 | 1.4e-02 |
| pTH6508 |  | nhr-36 | 37 | 1.58 | 1.5e-02 |
| pTH8318 |  | attf-1 | 22 | 2.17 | 1.5e-02 |
| V$OCT1\_03 |  | ceh-18 | 51 | 1.27 | 1.5e-02 |
| Pbx1\_3203 |  | ceh-20 | 36 | 1.61 | 1.5e-02 |
| pTH3041 |  | atf-2 | 23 | 2.10 | 1.5e-02 |
| CREM\_f1 |  | crh-1 ceh-26 | 23 | 2.10 | 1.5e-02 |
| K562\_SP2\_HudsonAlpha |  | klf-2 | 46 | 1.38 | 1.5e-02 |
| pTH9282 |  | attf-1 C01B12.2 | 25 | 1.99 | 1.5e-02 |
| MA0162.2 |  | ZC328.2 | 23 | 2.10 | 1.6e-02 |
| pTH10038 |  | gei-3 | 54 | 1.17 | 1.6e-02 |
| MA0451.1 |  | nhr-2 (-0.57) | 40 | 1.50 | 1.6e-02 |
| HeLa-S3\_ZNF274\_UCD |  | C28G1.4 | 42 | 1.46 | 1.6e-02 |
| pTH9182 |  | tbx-39 | 29 | 1.81 | 1.7e-02 |
| MA0027.1 |  | ceh-16 | 35 | 1.62 | 1.7e-02 |
| Dlx2\_2273 |  | ceh-43 (0.57) | 39 | 1.52 | 1.7e-02 |
| FOXJ3\_1 |  | lin-31 let-381 daf-16 | 37 | 1.57 | 1.7e-02 |
| ZN384\_f1 |  | K11D2.4 lin-29 | 43 | 1.43 | 1.8e-02 |
| Meox1\_2310 |  | ceh-31 | 51 | 1.26 | 1.8e-02 |
| Rfxdc2\_3516 |  | daf-19 (0.52) | 16 | 2.63 | 1.8e-02 |
| pTH8679 |  | pax-2 (0.52) | 17 | 2.52 | 1.8e-02 |
| Ftz\_Cell\_FBgn0001077 |  | lim-7 unc-86 lin-39 | 42 | 1.45 | 1.8e-02 |
| TFAP4\_1 |  | hlh-11 | 46 | 1.37 | 1.8e-02 |
| Gsh2\_3990 |  | ceh-31 | 32 | 1.69 | 2.0e-02 |
| pTH8566 |  | lin-54 | 43 | 1.42 | 2.0e-02 |
| pTH5078 |  | ces-2 | 47 | 1.34 | 2.0e-02 |
| Dlx3\_1030 |  | ceh-43 (0.57) | 52 | 1.23 | 2.1e-02 |
| Hoxa3\_2783 |  | lin-39 | 50 | 1.28 | 2.1e-02 |
| V$S8\_01 |  | ceh-45 | 44 | 1.40 | 2.1e-02 |
| Six2\_2307 |  | ceh-32 | 43 | 1.42 | 2.1e-02 |
| CXXC1\_si |  | F52B11.1 | 46 | 1.36 | 2.1e-02 |
| pTH9052 |  | ces-2 Y51H4A.4 C01B12.2 | 16 | 2.58 | 2.1e-02 |
| Nkx2-9\_3082 |  | dsc-1 | 47 | 1.34 | 2.1e-02 |
| Mw151 |  | gei-11 | 17 | 2.47 | 2.2e-02 |
| MA0484.1 |  | nhr-62 nhr-239 | 52 | 1.23 | 2.2e-02 |
| Zfp691\_0895 |  | F21A9.2 | 20 | 2.22 | 2.2e-02 |
| GRHL1\_1 |  | grh-1 | 8 | 4.71 | 2.2e-02 |
| pTH9080 |  | mnm-2 | 23 | 2.03 | 2.3e-02 |
| pTH10816 |  | dmd-6 | 26 | 1.88 | 2.3e-02 |
| pTH10041 |  | ztf-29 | 22 | 2.08 | 2.4e-02 |
| pTH9059 |  | ztf-28 | 37 | 1.54 | 2.4e-02 |
| TCF4\_2 |  | hlh-2 | 48 | 1.31 | 2.4e-02 |
| Hmbox1\_2674 |  | hmbx-1 (-0.63) | 29 | 1.77 | 2.4e-02 |
| E2F4\_1 |  | F49E12.6 | 29 | 1.77 | 2.4e-02 |
| pTH6562 |  | ceh-5 | 50 | 1.27 | 2.4e-02 |
| FOXO1\_3 |  | daf-16 | 37 | 1.54 | 2.5e-02 |
| Nkx1-1\_3856 |  | ceh-30 | 15 | 2.66 | 2.5e-02 |
| pTH9353 |  | ceh-51 (-0.51) | 40 | 1.47 | 2.5e-02 |
| Cdx1\_2245 |  | ceh-13 | 52 | 1.22 | 2.6e-02 |
| Vax2\_3500 |  | C02F12.10 | 50 | 1.26 | 2.6e-02 |
| TBX3\_f1 |  | tbx-39 | 40 | 1.47 | 2.7e-02 |
| pTH9958 |  | ztf-6 | 19 | 2.24 | 2.7e-02 |
| HSFY2\_1 |  | hsf-1 | 26 | 1.86 | 2.7e-02 |
| pTH6425 |  | ceh-20 | 19 | 2.24 | 2.7e-02 |
| pTH10769 |  | Y48G1C.6 | 34 | 1.60 | 2.7e-02 |
| MA0037.2 |  | elt-1 | 31 | 1.69 | 2.7e-02 |
| MA0594.1 |  | lin-39 | 44 | 1.38 | 2.8e-02 |
| SOX2\_f1 |  | sox-4 ceh-6 | 49 | 1.28 | 2.8e-02 |
| GATA3\_1 |  | elt-1 | 51 | 1.24 | 2.9e-02 |
| pTH10718 |  | egl-43 | 33 | 1.62 | 3.0e-02 |
| HepG2\_HSF1\_Stanford |  | Y53C10A.3 | 47 | 1.32 | 3.0e-02 |
| pTH9300 |  | dmd-3 C34D1.1 | 43 | 1.40 | 3.0e-02 |
| V$BRN2\_01 |  | ceh-18 | 10 | 3.56 | 3.1e-02 |
| Barx1\_2877 |  | ceh-43 (0.57) | 8 | 4.40 | 3.1e-02 |
| HNF6\_f1 |  | ceh-48 | 47 | 1.32 | 3.1e-02 |
| V$IK1\_01 |  | F26F4.8 | 39 | 1.48 | 3.2e-02 |
| pTH1014 |  | atf-5 | 22 | 2.02 | 3.2e-02 |
| Hoxa5\_3415 |  | lin-39 | 50 | 1.26 | 3.3e-02 |
| pTH9096 |  | T07C12.11 | 24 | 1.92 | 3.3e-02 |
| Irx3\_0920 |  | irx-1 | 29 | 1.73 | 3.3e-02 |
| pTH9246 |  | C34D1.1 | 40 | 1.45 | 3.3e-02 |
| pTH8745 |  | attf-1 | 38 | 1.49 | 3.3e-02 |
| V$TBP\_01 |  | tbp-1 | 40 | 1.45 | 3.4e-02 |
| V$YY1\_01 |  | lsy-2 | 41 | 1.43 | 3.4e-02 |
| ECC-1\_ERALPHA\_HudsonAlpha |  | nhr-71 | 27 | 1.79 | 3.4e-02 |
| I$UBX\_01 |  | lin-39 | 6 | 5.97 | 3.5e-02 |
| Lbx2\_3869 |  | mls-2 (0.59) | 47 | 1.31 | 3.5e-02 |
| Hoxa4\_3426 |  | lin-39 | 51 | 1.23 | 3.6e-02 |
| Hoxc4\_3491 |  | lin-39 | 51 | 1.23 | 3.7e-02 |
| pTH9384 |  | cfi-1 | 34 | 1.57 | 3.7e-02 |
| V$ARNT\_02 |  | aha-1 | 9 | 3.77 | 3.8e-02 |
| pTH3751 |  | tbx-39 | 49 | 1.27 | 3.8e-02 |
| HXC6\_f1 |  | nhr-100 (0.63) lin-39 lin-1 | 34 | 1.57 | 3.8e-02 |
| MAFA\_f1 |  | F45H11.6 | 21 | 2.04 | 3.8e-02 |
| MA0173.1 |  | irx-1 | 53 | 1.18 | 3.9e-02 |
| MCR\_f1 |  | nhr-255 (0.52) | 30 | 1.68 | 3.9e-02 |
| V$IK2\_01 |  | F26F4.8 | 36 | 1.52 | 3.9e-02 |
| V$MEF2\_03 |  | mef-2 | 50 | 1.25 | 3.9e-02 |
| HXD13\_f1 |  | pal-1 | 27 | 1.77 | 3.9e-02 |
| Tcf2\_0913 |  | hmbx-1 (-0.63) | 25 | 1.84 | 3.9e-02 |
| V$FAC1\_01 |  | gei-8 | 35 | 1.54 | 4.0e-02 |
| MA0531.1 |  | Y5F2A.4 | 29 | 1.70 | 4.0e-02 |
| Vax1\_3499 |  | C02F12.10 | 50 | 1.25 | 4.0e-02 |
| pTH9957 |  | fkh-9 | 42 | 1.40 | 4.0e-02 |
| Erg |  | lin-1 | 15 | 2.49 | 4.1e-02 |
| pTH9326 |  | nhr-122 | 52 | 1.20 | 4.1e-02 |
| pTH4325 |  | ceh-18 | 44 | 1.36 | 4.1e-02 |
| Hr46\_FlyReg\_FBgn0000448 |  | nhr-213 | 41 | 1.41 | 4.2e-02 |
| Gmeb1\_1745 |  | attf-1 | 18 | 2.21 | 4.2e-02 |
| Pou3f4\_3773 |  | ceh-6 | 53 | 1.18 | 4.3e-02 |
| Evx1\_3952 |  | ceh-53 | 15 | 2.47 | 4.4e-02 |
| pTH8649 |  | mbr-1 (0.53) | 37 | 1.49 | 4.4e-02 |
| MA0261.1 |  | nhr-255 (0.52) lin-14 | 30 | 1.66 | 4.4e-02 |
| pTH9044 |  | nhr-177 | 20 | 2.07 | 4.4e-02 |
| Mw140 |  | efl-1 | 30 | 1.66 | 4.4e-02 |
| pTH9884 |  | tbx-39 | 21 | 2.01 | 4.5e-02 |
| EMX2\_2 |  | ceh-2 | 45 | 1.34 | 4.5e-02 |
| Hoxb4\_2627 |  | lin-39 | 51 | 1.22 | 4.5e-02 |
| Six1\_0935 |  | ceh-32 | 43 | 1.37 | 4.6e-02 |
| pTH5976 |  | irx-1 | 49 | 1.26 | 4.6e-02 |
| V$FOXJ2\_02 |  | lin-31 | 49 | 1.26 | 4.6e-02 |
| NKX28\_f1 |  | ceh-24 | 36 | 1.51 | 4.7e-02 |
| FOXB1\_3 |  | lin-31 | 49 | 1.26 | 4.7e-02 |
| FOXC1\_2 |  | let-381 | 47 | 1.30 | 4.7e-02 |
| NKX22\_si |  | dsc-1 | 38 | 1.46 | 4.7e-02 |
| PAX5\_si |  | pax-2 (0.52) | 28 | 1.71 | 4.8e-02 |
| Hoxd9\_3 |  | lin-39 | 42 | 1.39 | 4.8e-02 |
| Hoxc8\_3429 |  | lin-39 | 21 | 1.99 | 4.8e-02 |
| NR2F1\_3 |  | nhr-2 (-0.57) | 43 | 1.37 | 4.9e-02 |
| pTH9135 |  | pop-1 | 35 | 1.52 | 5.0e-02 |

### Correlated (and anti-correlated) transcription factors

|  |  |
| --- | --- |
| **Transcription factor** | **Correlation** |
| bed-3 | 0.84 |
| elt-6 | 0.79 |
| Y105C5A.15 | 0.78 |
| ets-5 | 0.77 |
| Y22D7AL.16 | 0.76 |
| nhr-91 | 0.75 |
| ccch-1 | 0.74 |
| grl-25 | 0.73 |
| dac-1 | 0.73 |
| nhr-214 | 0.72 |
| nhr-277 | 0.71 |
| nhr-281 | 0.71 |
| ceh-8 | 0.71 |
| nhr-230 | 0.68 |
| hlh-4 | 0.67 |
| madf-1 | 0.66 |
| fkh-8 | 0.66 |
| nhr-138 | 0.66 |
| nhr-181 | 0.65 |
| F13H6.1 | 0.65 |
| nhr-253 | 0.64 |
| tag-97 | 0.64 |
| fozi-1 | 0.64 |
| nhr-100 | 0.63 |
| egl-18 | 0.62 |
| snu-23 | -0.48 |
| Y48G9A.11 | -0.49 |
| zip-8 | -0.49 |
| Y57A10A.31 | -0.49 |
| ccch-3 | -0.50 |
| ceh-51 | -0.51 |
| nhr-171 | -0.52 |
| ccch-5 | -0.53 |
| ceh-40 | -0.53 |
| pax-3 | -0.53 |
| hmg-3 | -0.54 |
| pos-1 | -0.54 |
| elt-7 | -0.54 |
| nhr-80 | -0.55 |
| zip-12 | -0.56 |
| nhr-269 | -0.56 |
| nhr-210 | -0.57 |
| nhr-2 | -0.57 |
| ztf-20 | -0.58 |
| pqn-75 | -0.58 |
| cey-2 | -0.60 |
| F49E8.2 | -0.60 |
| hmbx-1 | -0.63 |
| nhr-246 | -0.67 |
| cep-1 | -0.71 |

### ChIP peaks enriched

none found
